# Theoretical Investigation of Ethane‐to‐Aromatics Conversion Over [GaH_2_ ]‐ZSM‐5

**DOI:** 10.1002/jcc.70537

**Published:** 2026-11-03

**Authors:** João V. F. Costa, Thiago M. Mizumoto, Lenin J. Díaz Soto, Leonardo J. Duarte, Leandro Martins, Pedro Vidinha, Liane Rossi, Ataualpa A. C. Braga

**Affiliations:** ^1^ Instituto de Química, Universidade de São Paulo São Paulo Brazil; ^2^ Centro de Ciências Naturais e Humanas (CCNH), Universidade Federal Do ABC (UFABC) São Paulo Brazil; ^3^ São Paulo State University (UNESP), Institute of Chemistry Araraquara São Paulo Brazil

**Keywords:** aromatization, dehydrogenation, DFT, Ga species

## Abstract

Gallium‐modified ZSM‐5 (Ga‐ZSM‐5) zeolites are highly effective catalysts for the ethane‐to‐aromatics (ETA) process. In this work, the catalytic role of the Ga^+^, [GaO]^+^ and [GaH_2_]^+^ species in Ga–ZSM‐5 is investigated using a cluster model and density functional theory (DFT) calculations, focusing on ethane dehydrogenation (EDH) and subsequent ethene aromatization steps. Anharmonic vibrational analysis reveals that the H–Ga–H asymmetric stretching vibration corresponds to the experimentally observed band. Moreover, the Interaction Region Indicator (IRI) analysis shows that the ethane‐zeolite interaction is dominated by van der Waals forces. Although ethane activation over [GaH_2_]^+^ remains energetically demanding during the EDH stage, with an activation free energy of 68.7 kcal mol^−1^, the subsequent steps are comparatively more accessible, and the active site can be regenerated without the severe energetic penalty observed for the Ga^+^ and [GaO]^+^ pathways. Finally, C_6_ ring closure is identified as the turnover frequency‐determining transition state species for the ETA process. These results provide detailed mechanistic insight into the role of [GaH_2_]^+^ in the ETA process and support the use of cluster models as reliable and efficient alternatives for investigating metal‐modified zeolite catalysis.

## Graphical abstract

DFT calculations with two ZSM‐5 cluster models describe the complete ethane‐to‐aromatics cycle over [GaH_2_]^+^, which shows a lower maximum barrier than Ga^+^ and avoids the severe regeneration penalty of [GaO]^+^. Irrespective of cluster size, C_6_ ring closure, rather than the final dehydrogenation to benzene, is the turnover‐frequency‐determining transition state.

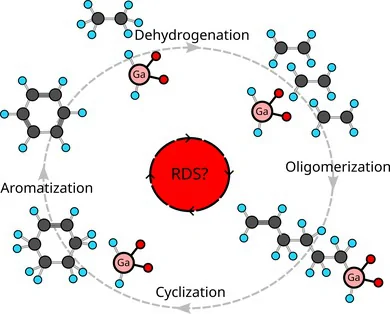

## 1 Introduction

Aromatic compounds are essential chemical commodities with widespread applications in the chemical industry, including the production of solvents, dyes, polymers, surfactants, pharmaceuticals and optoelectronics [1, 2]. Benzene, toluene, and xylene (BTX) are primarily obtained through naphtha reforming and thermal cracking processes. However, these conventional routes are increasingly challenged by the growing global demand for BTX and the need for more sustainable feedstocks [3]. In this context, the direct aromatization of light alkanes over zeolitic materials has emerged as a promising alternative strategy [4]. The conversion of ethane to aromatics (ETA) over zinc‐ and gallium‐modified zeolites represents an attractive pathway for BTX production [5, 6, 7]. Although zinc‐modified zeolites exhibit high catalytic activity and selectivity toward BTX formation [8, 9], their practical application is limited by poor thermal stability and rapid deactivation under high‐temperature reaction conditions [10]. In contrast, gallium‐modified zeolites generally offer superior thermal stability and improved resistance to deactivation, making them promising candidates for sustained BTX production under industrial conditions [11].

The isomorphous substitution of framework Si‐atoms with trivalent atoms such as Al^3+^ or Ga^3+^ generates a negative charge that can be compensated either by a metal cation that leads to the formation of a Lewis acid site, or by a proton which gives rise to a Brønsted acid site [12]. Gallium can be present as intra‐ and extra‐framework species [13, 14]. The nature of the extra‐framework species depends on experimental preparation conditions and pretreatments, and it has been reported the presence of Ga_2_O_3_, [Ga]^+^, [GaO]^+^, [Ga_2_O_2_]^2+^, [Ga(OH)_2_]^+^, and Ga hydrides, GaH_X_, ([GaH]^2+^ and [GaH_2_]^+^) [15, 16].

Although both experimental and theoretical investigations have examined light‐alkane dehydrogenation in several Ga sites supported in zeolites [16, 17, 18, 19, 20, 21], the exact speciation of gallium and the structure of the true active site under reaction conditions remain unresolved [22]. However, Mengwen et al. [15], investigating ethane dehydrogenation (EDH) reported that the Ga‐ZSM‐5 catalyst containing the [GaH_2_]^+^ species shows more selectivity and less coke formation than other Ga‐ZSM‐5 based catalysts with lower amounts of Ga.

In the aluminosilicate zeolites, monovalent cationic species are stabilized by one Al site, while doubly charged cations interact with two Al sites [23, 24]. Experimentally, Huang et al. [25], obtained the [GaH]^2+^ species supported in ZSM‐5 zeolite with a Ga/Al of 0.3, whereas the [GaH_2_]^+^ ions are obtained with higher Ga loading (Ga/Al = 1.0). The GaH_X_ in ZSM‐5 were characterized using IR spectroscopy where bands corresponding to the stretching Ga‐H vibration at ~2040 and ~2060 cm^−1^ are associated with [GaH_2_]^+^ and [GaH]^2+^ species, respectively [17, 26, 27]. Moreover, Bell et al. [28], using a hybrid QM/MM theoretical approach, studied ethane dehydrogenation over Ga–ZSM‐5 and reported that hydrogenated Ga species such as [GaH]^2+^ and [GaH_2_]^+^ exhibit lower activation barriers than Brønsted acid sites (H‐ZSM‐5) and isolated [Ga]^+^ sites, with ethane activation over [Ga]^+^ proceeding via a higher‐energy alkyl pathway.

Experimental studies have demonstrated that Ga‐modified zeolites exhibit excellent catalytic performance in the ethane‐to‐aromatics (ETA) process [29, 30, 31]. From a mechanistic perspective, the reaction is generally initiated by the dehydrogenation of ethane to ethene, EDH, over the gallium center, which is recognized as a key elementary step controlling overall catalytic performance [32, 33]. Ethene then undergoes subsequent oligomerization, cyclization, and dehydrogenation steps, ultimately leading to the formation of aromatic compounds within the zeolitic framework [30, 34, 35, 36].

The EDH process over the [GaH_2_]‐ZSM‐5 catalyst has been theoretically investigated [19, 28, 37, 38]. Frash and van Santen [37] proposed a three‐step mechanism involving C–H bond activation of ethane, followed by H_2_ formation and subsequent ethene production. Mansoor et al. [28], examined both a stepwise alkyl pathway and a concerted mechanism, while Fang et al. [38], extended the analysis to Ga‐FAU zeolites. In all cases, the stepwise alkyl pathway was found to be energetically more favorable than the concerted mechanism.

Using QM/MM approaches based on the 2‐ONIOM methodology, several studies have reported the aromatization of alkenes in zeolite catalysts [38, 39, 40]. Wongnongwa et al. [39], investigated benzene formation from *n*‐pentane over Ga–ZSM‐5 and suggested that cyclization and aromatization proceed predominantly via the [Ga]^+^ species. Fang et al. [38], studied the ETA mechanism over gallium‐modified FAU zeolite (Ga‐FAU) and proposed that [GaH_2_]^+^ may act as the active species for ethene oligomerization and aromatization, given the facile interconversion between [GaH]^2+^ and [GaH_2_]^+^ species. In contrast, Mo et al. [40], evaluated multiple Ga species in Ga–ZSM‐5 for cyclohexane dehydrogenation and identified [GaH]^2+^, [Ga(OH)]^2+^ and [Ga_2_O_2_]^2+^ as the most favorable sites for cycloalkane dehydrogenation. These contrasting conclusions further emphasize that the nature of the active Ga species strongly depends on the reaction environment, zeolite topology, and mechanistic considerations.

Zeolite frameworks are commonly modeled using two approaches: periodic boundary conditions (PBC) and cluster models [41]. In the PBC methodology, the unit cell is repeated periodically in three dimensions. In the cluster approach, by contrast, a finite fragment of the zeolite structure representing the catalytic center and its local environment is extracted, keeping the computational cost manageable. Although cluster models offer a lower‐computational‐cost alternative to PBC approaches, careful construction is required to properly account for local structural and electronic effects and to avoid neglecting relevant framework interactions [42]. For example, a 46 T cluster model of H‐ZSM‐5, where T denotes a tetrahedral framework unit (Si or Al), was employed to investigate the benzene methylation process [43].

Herein, a cluster approach and the density functional theory (DFT) methodology were employed to investigate the complete catalytic cycle of the ETA process in the [GaH_2_]^+^–ZSM‐5 catalyst. The [GaH_2_]^+^ species was selected as the active site based on previous experimental results and on preliminary assessments of the ethane dehydrogenation step, in which different Ga motifs such as [GaO]^+^, [Ga]^+^, and [GaH_2_]^+^ were evaluated for the conversion of ethane to ethene. To the best of our knowledge, this work represents the first comprehensive computational investigation of the complete ETA reaction mechanism over the Ga–ZSM‐5 framework, explicitly considering [GaH_2_]^+^ as the active site.

## 2 Methodology

### 2.1 Computational Methods

All geometry optimizations were performed using the r^2^SCAN‐3c composite method, which can offer an efficient and reliable description of structures and interaction energies for large systems [44]. Single‐point energy calculations were performed using the *ω*B97X‐D4rev functional combined with the def2‐mTZVPP basis set to refine the electronic energies. To account for basis set superposition error (BSSE), the counterpoise (CP) correction in the Boys‐Bernardi formulation [45], was applied to the interaction energies at the *ω*B97X‐D4rev/def2‐mTZVPP level of theory.

Vibrational frequency calculations were carried out to confirm the nature of the stationary points and to obtain zero‐point energy (ZPE) and thermal corrections at 673.15 K and 1 atm (1.0132 bar), unless otherwise specified. All calculations were performed using the ORCA software package (version 6.0.0) [46]. Additionally, to characterize the nature of the noncovalent interactions between the zeolite framework and the adsorbed species, an Interaction Region Indicator (IRI) analysis was performed using the Multiwfn software (version 3.8) [47]. The Cartesian coordinates, electronic energies and free‐energy corrections of all optimized structures are provided in Data S2.

Using the energetic span model [48], we analyze the ETA catalytic cycle to find the Turnover Determining Intermediate (TDI) and Turnover frequency Determining Transition State (TDTS).

### 2.2 ZSM‐5 Cluster

The ZSM‐5 zeolite framework was modeled using a cluster approach with two sizes: a small cluster containing 239 atoms and 70 T (Figure 1a), and a large cluster with 379 atoms and 112 T (Figure 1b). The cluster was derived from the crystallographic structure of ZSM‐5 (MFI topology) sourced from the International Zeolite Association (IZA) database [49].

**FIGURE 1:**
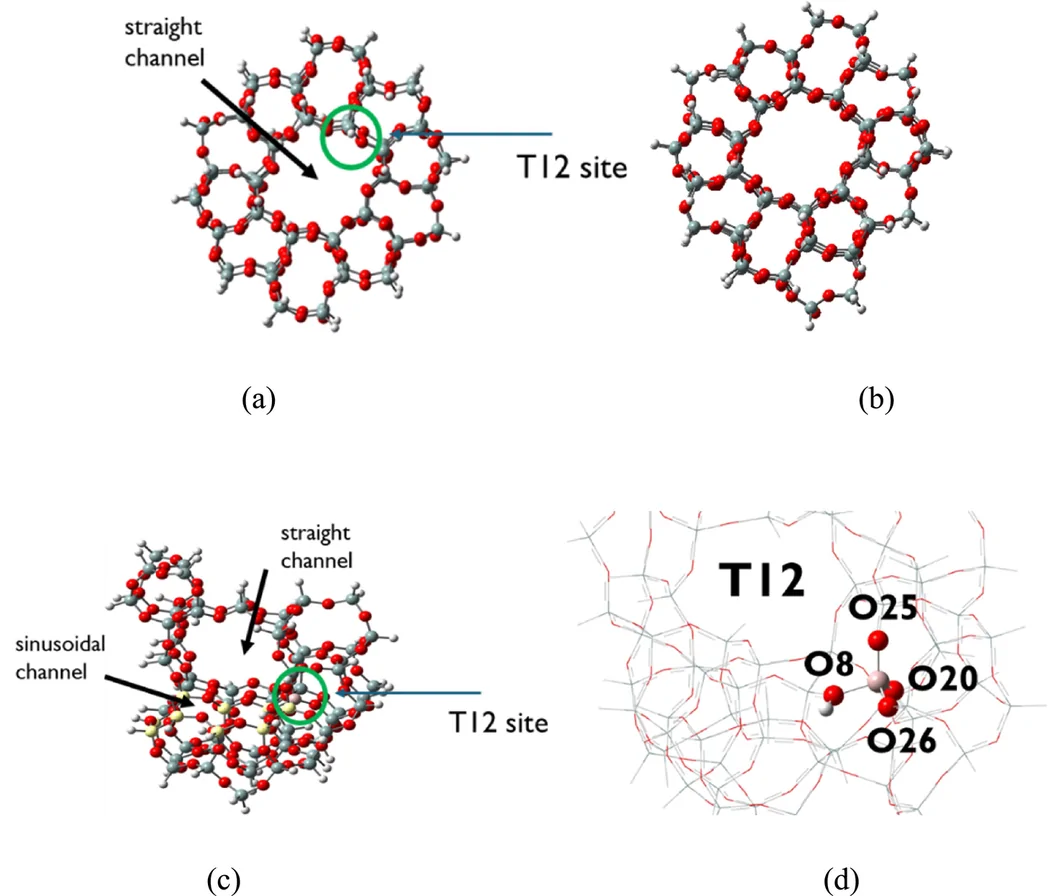
Cluster models of the ZSM‐5 zeolite. (a) Small cluster with 239 atoms; (b) large cluster with 379 atoms; (c) display of the straight and sinusoidal channels; and (d) labels of the O atoms in the T12 crystallographic position. Si atoms are depicted in gray, O atoms in red, Al atom in pink, and H atoms in white.

From the 12 different crystallographic positions in ZSM‐5, eight sites (T1, T2, T3, T5, T6, T7, T9, and T12) are positioned at the intersection of the straight and sinusoidal channels [50]. Using PBC calculations, Jones and Iglesia determined that the most stable site in H‐ZSM‐5 is the Al at the T3 position, with a difference of 4.2 kcal mol^−1^ from the T12 site [51]. On the other hand, Piccini et al. [52] reproduced the experimental adsorption enthalpies of methanol and ethanol by modeling the T12 site. Moreover, previous studies have shown that aluminum atoms located at channel intersections are favorable for catalytic activity in aromatization reactions [53], particularly at the T12 site [54, 55, 56]. Accordingly, the construction of the cluster was centered on the intersection cavity and its surroundings, ensuring a chemically meaningful representation of the catalytic environment around the T12 site (Figure 1c,d).

At the boundaries of the cluster, terminal oxygen atoms were replaced by hydrogen atoms, such that the newly formed Si–H bonds replace the original Si–O bonds with a fixed bond length of 1.47 Å. During the geometry optimization procedure, these boundary hydrogen atoms were kept fixed to avoid structural distortions and collapse of the zeolite framework, while the remaining atoms were allowed to relax. In this way, the catalytic center is positioned closer to the center of the cluster and proper relaxation around the active site can be achieved.

To evaluate the ability of the small cluster to capture adsorbate–framework interactions, the electronic adsorption energy, *E*_ads_, of ammonia and pyridine was compared against values obtained with 2‐ONIOM and PBC approaches, computational details are provided in the Supporting Information, and the results are shown in Table S1 and Figure S1. Using the cluster approach *E*_ads_(ammonia) is similar to that obtained with the PBC calculations. With pyridine as probe molecule the cluster model outcome differs by only 4.6 and 3.8 kcal mol^−1^ from ONIOM and PBC results, respectively. Thus, when the size of the adsorbate increases, a divergence of the outcomes from the small cluster is expected relative to models that consider a larger portion of the zeolite framework.

Consequently, the influence of cluster size on the ETA process with the [GaH_2_]^+^–ZSM‐5 catalyst was examined using a larger cluster, Figure 1b. Given the increased computational cost associated with this cluster size, geometry optimization was performed at the r^2^SCAN‐D4/def2‐SVP level including geometrical CP correction, followed by single‐point energy corrections at the *ω*B97X‐D4rev/def2‐mTZVPP level with Boys‐Bernardi CP correction.

## 3 Results

Figure 2 depicts the [GaH_2_]‐ZSM‐5 structure, where the Ga atom is coordinated symmetrically between the H and O atoms and the Ga–H and Ga–O bond lengths are similar to previously reported values [57]. From the charge analysis (Table S2 and Figure S2), the Ga atom carries a positive charge of +0.2 a.u., while the H atoms attached to it are nearly neutral, with a charge of +0.02 a.u.

**FIGURE 2:**
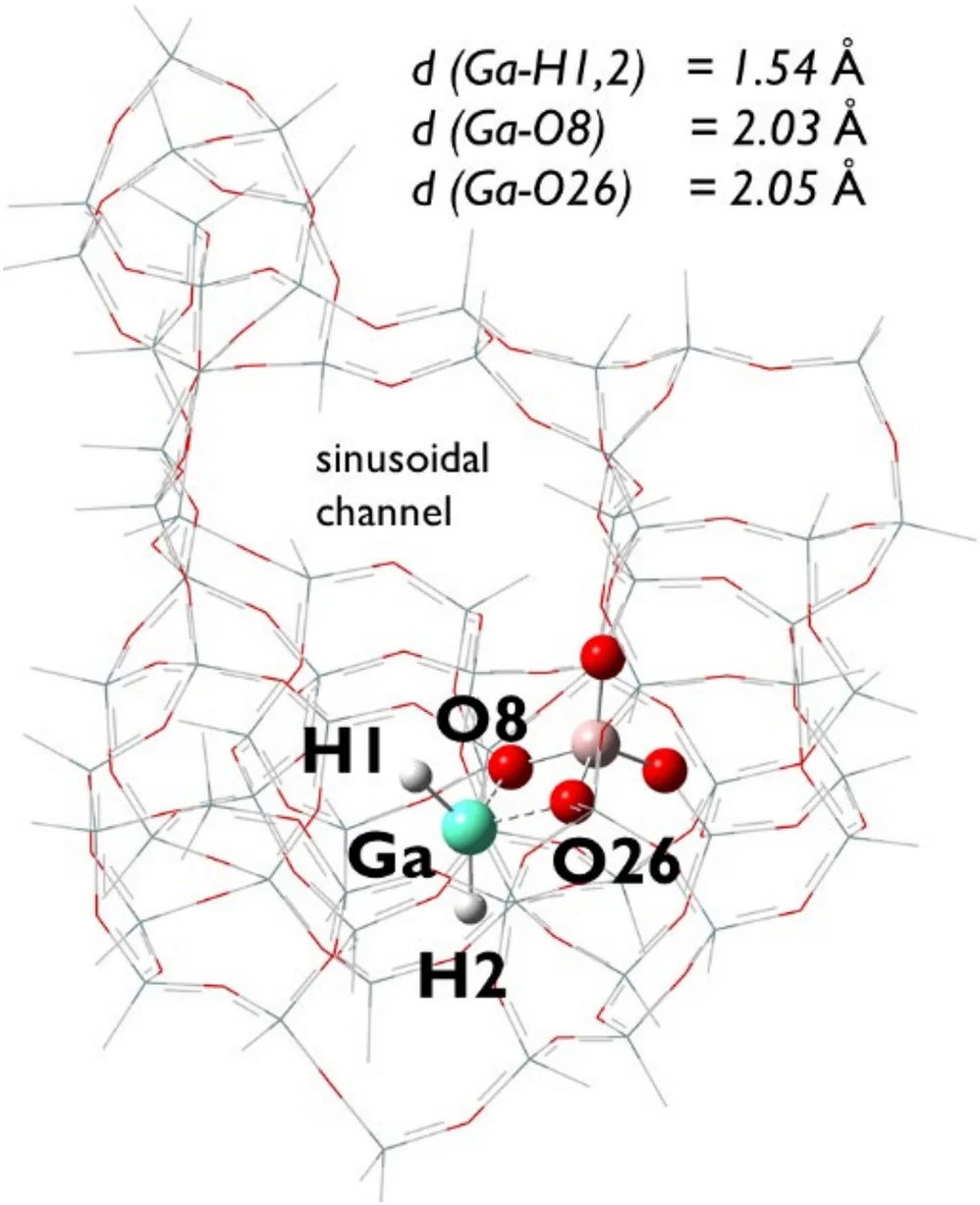
2‐ONIOM model of the [GaH_2_]‐ZSM‐5(T12) structure with [GaH_2_AlO_4_] motif represented in high‐level (balls and sticks) and 244 atoms in low‐level (wireframes). Si atoms are depicted in gray, O atoms in red, Al atom in pink, Ga atom in blue and H atoms in white.

A 2‐ONIOM model with a minimal high‐level region, [GaH_2_AlO_4_] (Figure 2), was used to obtain the harmonic (*ω*), anharmonic (*ν*) frequencies, and their associated IR intensities, *I*_*ω*_ and *I*_*ν*_, for the Ga–H vibrational stretching modes (see Supporting Information for methodological details). For the symmetric mode, the values obtained were *ω* = 2040.4 cm^−1^ (*I*_*ω*_ = 93.9 km mol^−1^) and *ν* = 1983.9 cm^−1^ (*I*_*ν*_ = 91.4 km mol^−1^). While for the asymmetric mode, *ω* = 2076.0 cm^−1^; (*I*_*ω*_ = 322.5 km mol^−1^) and *ν* = 2010.0 cm^−1^ (*I*_*ν*_ = 236.9 km mol^−1^). Thus, the anharmonic corrections reduced both the frequency and the IR intensity. The asymmetric intensity is larger than the symmetric one; therefore, this vibration mode should be experimentally observable, as previously noted [58]. However, *ν*_*asym*_ differs by 30 cm^−1^ from the Ga–H experimental frequency associated with the [GaH_2_] species at ~2040 cm^−1^ [17, 26, 27]. This discrepancy could be reduced by increasing the high‐level region of the 2‐ONIOM model to better represent the active site.

The complete catalytic cycle of the ETA process is illustrated in Figures 3, 4, 5, 6, 7, 8. For clarity, the reaction pathway is divided into three stages: (i) the conversion of ethane to ethene, (ii) the oligomerization and cyclization of ethene leading to the formation of a six‐membered cyclic intermediate, and (iii) subsequent dehydrogenation steps that yield benzene within the Ga‐ZSM‐5 zeolite framework.

**FIGURE 3:**
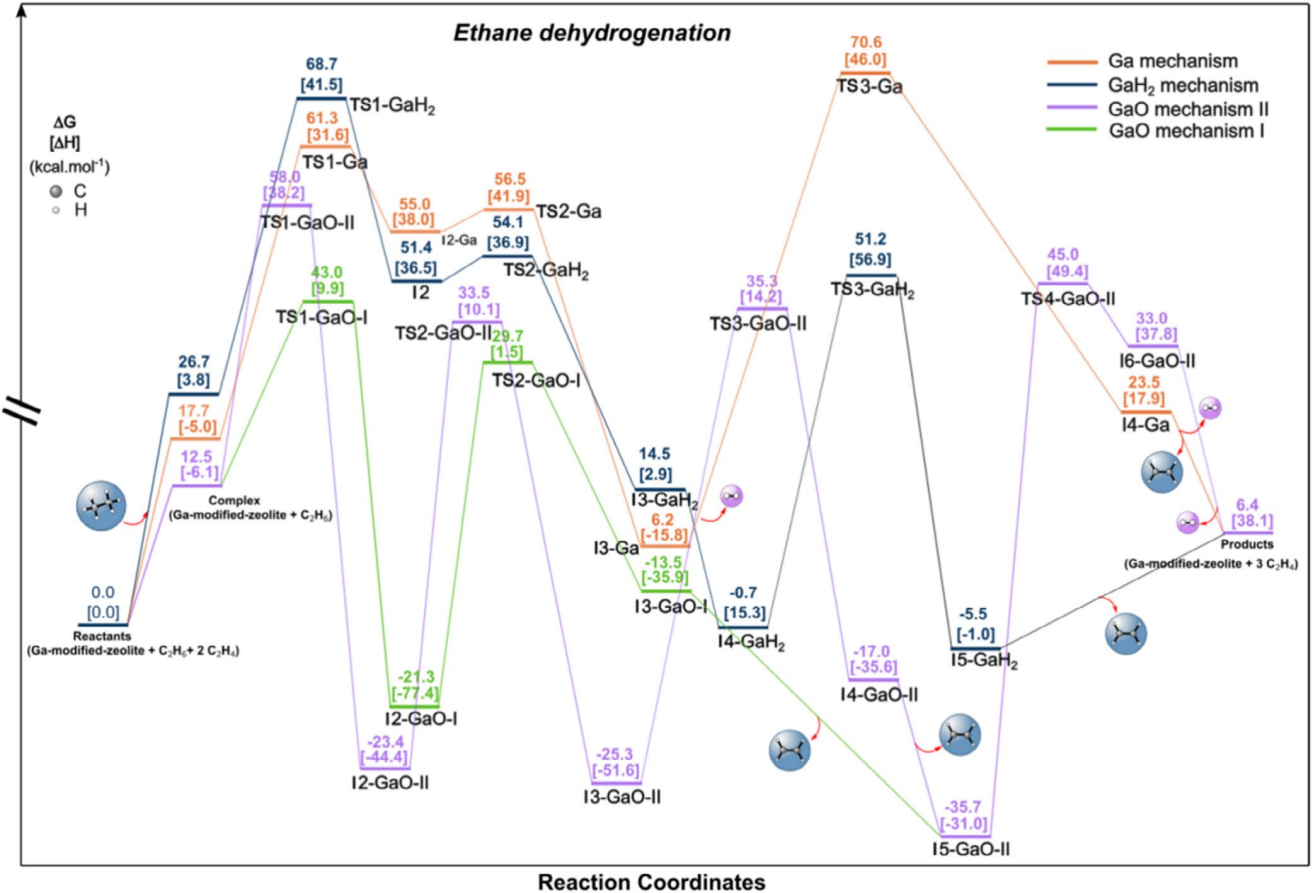
Enthalpy and Gibbs free‐energy profiles for ethane dehydrogenation over Ga^+^, [GaH_2_]^+^, and [GaO]^+^ sites of Ga–ZSM‐5 catalyst according to the stepwise mechanism. For the [GaO]^+^ site, the alkyl (GaO‐I) and carbenium (GaO‐II) pathways were considered.

**FIGURE 4:**
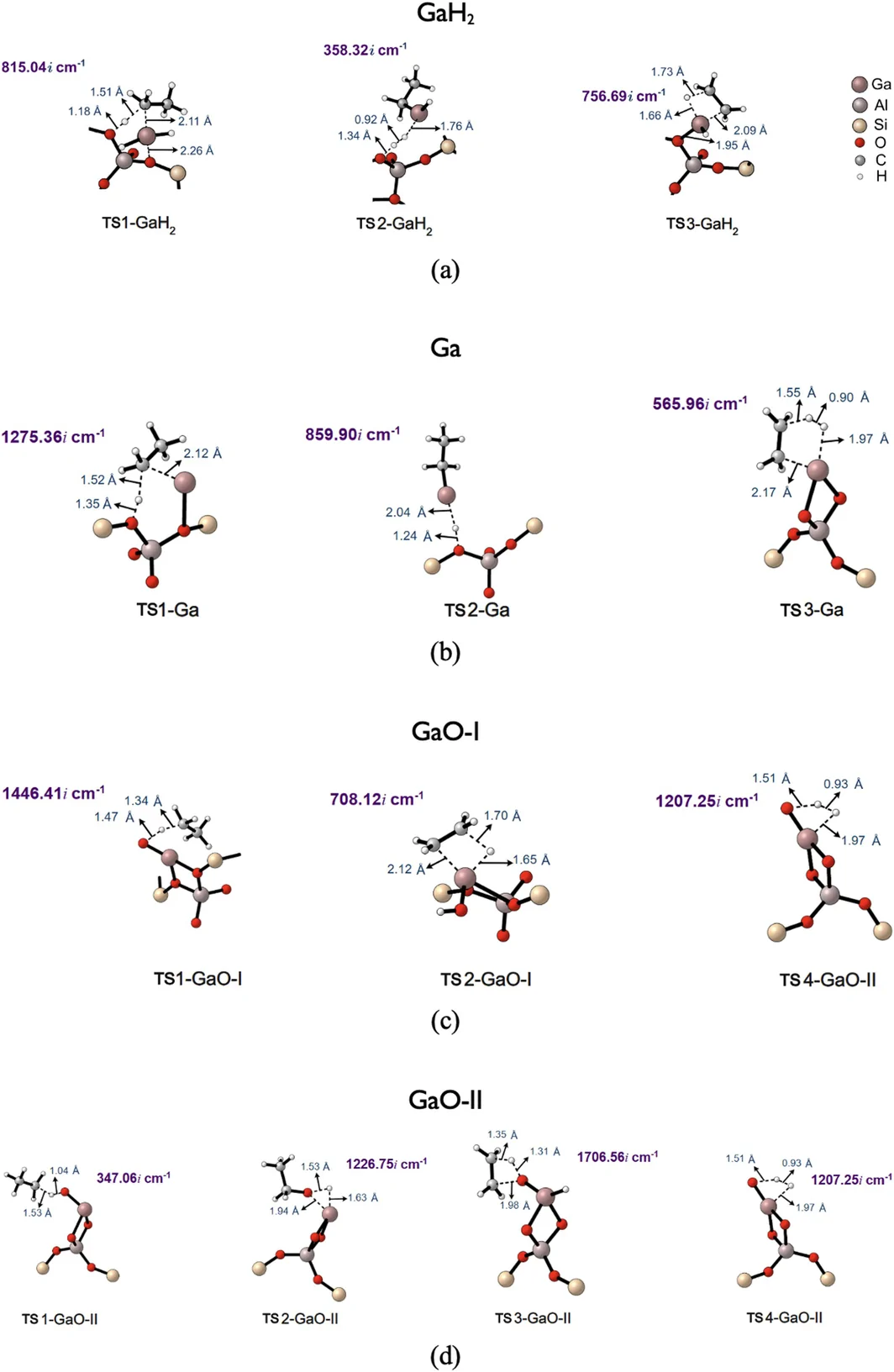
Optimized transition‐state structures involved in the ethane dehydrogenation pathways over different Ga active‐site motifs in ZSM‐5. (a) [GaH_2_]^+^, (b) Ga^+^, (c) [GaO]^+^ alkyl pathway (GaO‐I), and (d) [GaO]^+^ carbenium pathway (GaO‐II). Imaginary frequencies and selected interatomic distances associated with the corresponding bond‐breaking and bond‐forming events are indicated for each transition state. For clarity, only the active‐site region of the Ga–ZSM‐5 cluster is shown.

**FIGURE 5:**
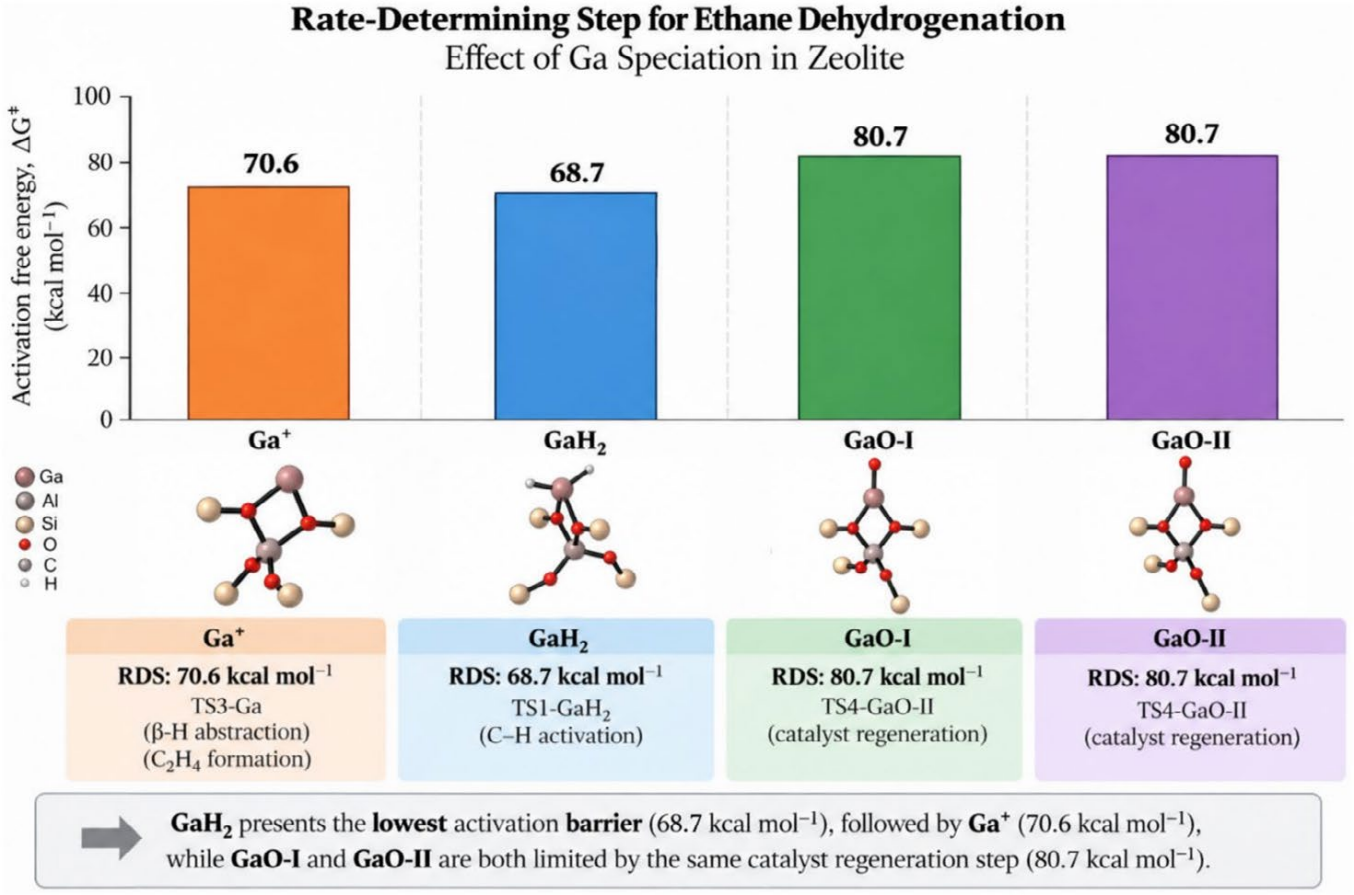
Comparison of the rate‐determining steps identified for ethane dehydrogenation over the Ga^+^, [GaH_2_]^+^, and [GaO]^+^ active‐sites. For the [GaO]^+^ site, two distinct reaction pathways were investigated, denoted GaO‐I and GaO‐II.

### 3.1 First Stage: Ethane Dehydrogenation (EDH)

Three different Ga species, Ga^+^, [GaH_2_]^+^, and [GaO]^+^, were evaluated as active‐site models for the ethane dehydrogenation (EDH) step over ZSM‐5, with the aim of identifying the most suitable motif for the subsequent investigation of the complete ETA catalytic cycle. The corresponding energetic profiles are presented in Figure 3. Previous studies have identified [GaH_2_]^+^ as a highly active Ga species for alkane dehydrogenation and have shown that it can be formed under hydrogen‐containing conditions [28, 59, 60]. For the [GaO]^+^ species, both the alkyl and carbenium mechanisms were considered, denoted as GaO‐I and GaO‐II, respectively. On the other hand, the [GaH]^2+^ species was omitted because its stability and catalytic activity have been reported to depend on the local Al distribution within the zeolite framework [18]. A systematic investigation of this effect requires considering different Si/Al ratios and Al distributions, which is beyond the scope of the present work.

The stepwise energetic profiles obtained for the different Ga motifs reveal distinct energetic requirements for the EDH step. Throughout the reaction profile, the Ga^+^ pathway involves activation free energies of 61.3, 56.5, and 70.6 kcal mol^−1^ for the successive elementary steps. The highest barrier corresponds to TS3‐Ga, associated with the final dehydrogenation step and regeneration of the Ga^+^ active site. For the [GaH_2_]^+^ pathway, the corresponding activation free energies are 68.7, 54.1, and 51.2 kcal mol^−1^, with the highest barrier associated with the initial C–H activation through TS1‐GaH_2_. Thus, although both Ga^+^ and [GaH_2_]^+^ pathways can proceed through a complete stepwise EDH cycle, the highest activation requirement is slightly lower for the [GaH_2_]^+^ model (68.7 kcal mol^−1^) than for Ga^+^ (70.6 kcal mol^−1^).

In contrast, the initial stages of the [GaO]^+^ pathways are comparatively more accessible. The alkyl pathway (GaO‐I) proceeds through lower‐energy elementary steps, whereas the carbenium pathway (GaO‐II) involves higher barriers during the initial stages. Although GaO‐I and GaO‐II follow distinct reaction sequences during the early stages, the two pathways converge at a common intermediate (labeled I5‐GaO‐II), before active‐site regeneration, and from there proceed through the same transition state, TS4‐GaO‐II, leading to regeneration of the [GaO]^+^ active site. Consequently, the final regeneration step represents a common energetic barrier bottleneck for both GaO‐I and GaO‐II: GaO‐II lies at −35.7 kcal mol^−1^ relative to the reactants, whereas TS4‐GaO‐II lies at +45.0 kcal mol^−1^, given an activation free energy of 80.7 kcal mol^−1^ for the regeneration step. This energetic penalty indicates that regeneration of the [GaO]^+^ active site constitutes a kinetic bottleneck, despite the comparatively accessible initial stages of ethane dehydrogenation. Therefore, the [GaO]^+^ model was not considered further for the construction of the complete ETA catalytic cycle.

The optimized transition‐state structures for each profile are shown in Figure 4. The selected interatomic distances and imaginary frequencies are consistent with the bond‐breaking and bond‐forming events associated with each elementary step. For the [GaH_2_]^+^ pathway (Figure 4a), TS1 exhibits the characteristic elongation of the activated C–H bond, consistent with heterolytic cleavage of this bond and formation of the ethyl intermediate, with an imaginary frequency of 815.0i cm^−1^ corresponding to this reaction coordinate. TS2 is characterized by the simultaneous rearrangement of the hydride and proton positions, with a short H–H distance of 0.92 Å, consistent with H_2_ formation and a corresponding imaginary frequency of 358.3i cm^−1^. Finally, TS3 displays the structural changes associated with *β*‐hydride transfer, including elongation of the C–H bond and reorganization of the Ga–C and Ga–H interactions, leading to ethene formation and regeneration of the [GaH_2_]^+^ site. This transition state has an imaginary frequency of 756.7i cm^−1^.

Similar structural features are observed for the Ga^+^ pathway (Figure 4b). TS1‐Ga is characterized by the activation of a C–H bond, with an imaginary frequency of 1275.4i cm^−1^ corresponding to this reaction coordinate. TS2‐Ga involves the rearrangement associated with H_2_ formation, with a corresponding imaginary frequency of 859.9i cm^−1^. Finally, TS3‐Ga presents the structural changes required for the second dehydrogenation and regeneration of the Ga^+^ site, with an imaginary frequency of 566.0i cm^−1^. For the [GaO]^+^ pathways (Figure 4c,d), the transition‐state structures and their associated imaginary frequencies depend on the alkyl and carbenium mechanisms.

Concerted pathways were also investigated for the three Ga‐based active‐site models, and the corresponding energetic profiles are provided in Figures S3–S8. For the [GaH_2_]^+^ and Ga^+^ sites, the concerted pathways exhibit higher energy barriers than the corresponding stepwise mechanisms, with a maximum energy difference of 11 and 10 kcal mol^−1^, respectively, considering the highest transition states for each site. For the [GaO]^+^ motif, the outcome of this comparison depends on the pathway considered. The concerted GaO‐I pathway involves a barrier of 57.1 kcal mol^−1^, which is slightly lower than the 58.0 kcal mol^−1^ barrier associated with the initial step of the corresponding stepwise pathway. In contrast, the concerted GaO‐II pathway exhibits a higher barrier of 70.0 kcal mol^−1^ compared with 58.0 kcal mol^−1^ for the stepwise counterpart. Therefore, the concerted mechanism is clearly disfavored for the [GaH_2_]^+^, Ga^+^, and GaO‐II pathways, whereas the two alternatives for GaO‐I have comparable energetic requirements. These results support the stepwise mechanism as the primary mechanistic description for the EDH cycle, except for GaO‐I, where the two mechanisms are energetically analogous.

A comparison of the rate‐determining steps identified for the different Ga species is summarized in Figure 5. Among the Ga‐based active‐site models considered, Ga^+^ and [GaH_2_]^+^ are both able to undergo the complete stepwise EDH sequence without the severe regeneration barrier identified for the [GaO]^+^ motif. However, comparison of their energetic profiles shows a slightly higher energy requirement for the Ga^+^ pathway (Figure 3). The highest transition state along the Ga^+^ pathway is TS3‐Ga, which lies at 70.6 kcal mol^−1^ relative to the reactants, whereas the highest transition state for the [GaH_2_]^+^ pathway is TS1‐GaH_2_ at 68.7 kcal mol^−1^. Thus, the [GaH_2_]^+^ model presents the lower maximum transition‐state energy between the two sites considered. On this basis, [GaH_2_]^+^ was selected as the active‐site motif for the subsequent investigation of the ETA catalytic cycle.

A detailed analysis of the EDH stepwise mechanism on [GaH_2_]‐ZSM‐5 reveals that the reaction proceeds through three elementary steps following ethane adsorption, with the *α*‐carbon maintaining an alkyl character throughout the catalytic cycle as shown in Figure 6. The initial adsorption leads to the formation of a weakly bound complex, followed by heterolytic C–H bond activation via TS1. In this step, a C–H bond of ethane is cleaved, forming an ethyl intermediate (I2) bound to the Ga center, and a Brønsted acid site on the zeolite. This process represents the most energetically demanding step of the [GaH_2_]^+^ pathway, with a barrier of 68.7 kcal mol^−1^. The structural features of TS1, particularly the elongation of the activated C–H bond, are consistent with this assignment. In the subsequent step, the system evolves from the ethyl intermediate toward H_2_ formation through TS2. This process involves hydride transfer from the ethyl fragment to the proton, accompanied by H–H bond formation and cleavage of the Ga–H and O–H interactions. The corresponding barrier is 53.4 kcal mol^−1^, and the short H–H distance observed in TS2 supports the proposed H_2_‐forming event. Finally, *β*‐hydride transfer through TS3 leads to ethene formation and regeneration of the [GaH_2_]^+^ active site, with a barrier of 54.8 kcal mol^−1^. The corresponding TS structure displays the expected changes in the C–H and Ga–C interactions associated with this process. Thus, the initial C–H activation represents the highest‐energy transition state along the [GaH_2_]^+^ pathway.

**FIGURE 6:**
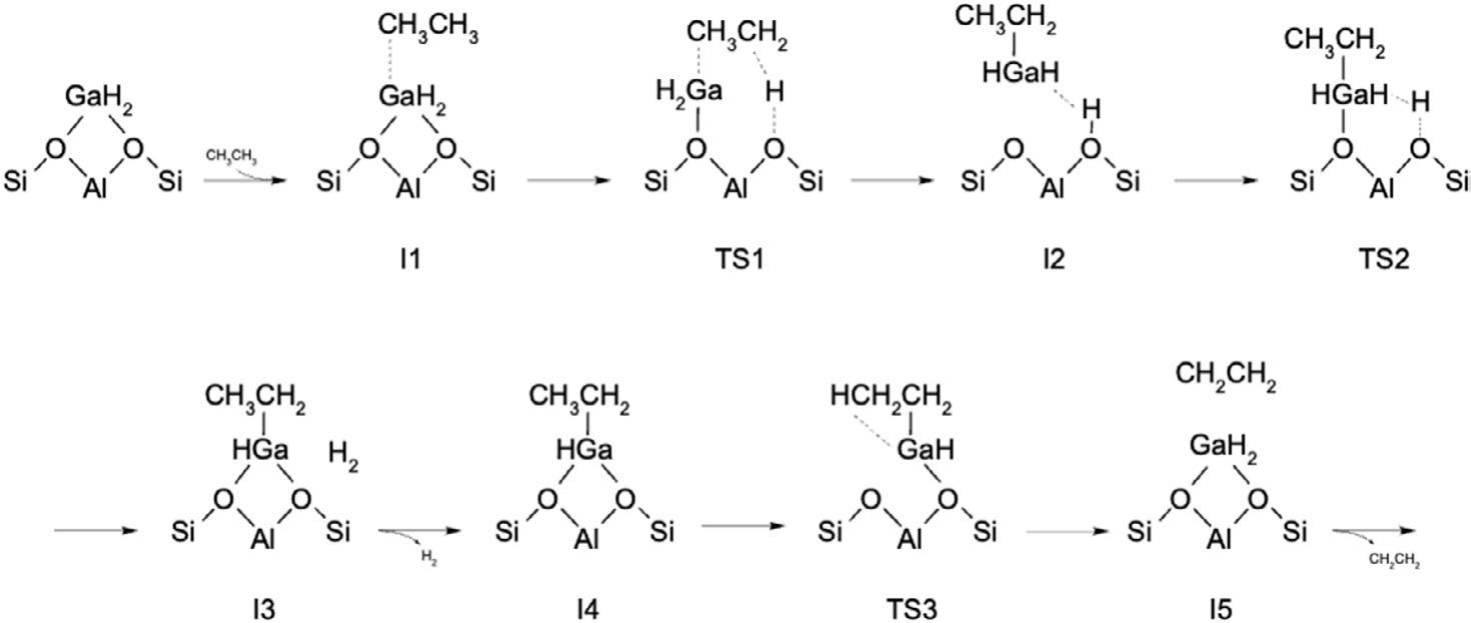
EDH according to the stepwise alkyl mechanism on [GaH_2_]‐ZSM‐5.

The IRI analysis shows that ethane adsorption is dominated by van der Waals forces between the alkane and the zeolite framework, with a weak interaction also present between the alkane and the GaH_2_ motif, Figures S9 and S10.

The energetic span analysis for the [GaH_2_]^+^ motif in the stepwise mechanism identifies TS1 as the TDTS, while I4 is identified as the TDI. This result is consistent with the theoretical work of Mansoor et al. [28], who investigated ethane dehydrogenation over [GaH_2_]‐ZSM‐5 at 823 K and identified the initial C–H activation as the rate‐determining transition state in the stepwise alkyl mechanism. In contrast, Fang et al. [38] investigated computationally the [GaH_2_]^+^ species in Ga‐FAU; their free‐energy profile identifies the formation of ethylene through TS3 as the rate‐determining step, with a free‐energy barrier of 57.2 kcal mol^−1^ at 673.15 K. Thus, although both studies consider the same [GaH_2_]^+^ active‐site motif, the TOF‐determining transition state differs between the MFI and FAU frameworks. Beyond the methodological differences between the two studies, this discrepancy may reflect the influence of the zeolite framework and the local environment surrounding the Ga center on the relative kinetic importance of the initial C–H activation step compared with the subsequent ethylene‐forming step.

Based on the comparative analysis of the three Ga‐based active‐site models, the [GaH_2_]^+^ species was selected as the active‐site model for the subsequent investigation of ethene aromatization. The [GaO]^+^ model was excluded because, despite the comparatively accessible barriers associated with the initial stages of ethane dehydrogenation, its regeneration requires a high activation free energy of 80.7 kcal mol^−1^, which represents a severe kinetic bottleneck for closing the catalytic cycle. The Ga^+^ model, although capable of completing the stepwise EDH sequence, exhibits a higher maximum transition‐state energy than [GaH_2_]^+^, with TS3‐Ga located at 70.6 kcal mol^−1^ compared with 68.7 kcal mol^−1^ for TS1‐GaH_2_. Additionally, the use of the [GaH_2_]‐ZSM‐5 system has been reported to suppress coke formation in the EDH process [15], which is relevant for the durability of the catalyst.

Finally, our results show that the predicted activation enthalpy barriers for ethane dehydrogenation on Ga‐ZSM‐5 are: 9.9 kcal mol^−1^ ([GaO]^+^‐I), 38.2 kcal mol^−1^ ([GaO]^+^‐II), 31.6 kcal mol^−1^ (Ga^+^), and 41.5 kcal mol^−1^ ([GaH_2_]^+^). It was experimentally reported that the apparent activation energy for the EDH stage over Ga_2_O_3_/H‐ZSM‐5 is 30.5 kcal mol^−1^ [61]. Thus, although the Ga^+^ pathway is numerically closer to the experimental value, the [GaO]^+^‐II carbenium pathway is considered the more appropriate point of comparison, since it reflects the oxidized Ga speciation reported for the experimental catalyst. Comparisons with the other Ga motifs should be made with caution, as they do not correspond to this oxidation state.

It should be noted that the high activation free energies computed at 673.15 K arise largely from the entropic penalty associated with bringing gas‐phase ethane to the transition state, rather than from an overestimation of the electronic barrier. For the [GaH_2_]^+^ pathway, the activation free energy of TS1 (68.7 kcal mol^−1^) exceeds the corresponding activation enthalpy (41.5 kcal mol^−1^) by approximately 27 kcal mol^−1^, which corresponds to the −TΔS^‡^ term at this temperature. Because the experimental apparent activation energy is an enthalpic quantity, the activation enthalpies, rather than the free energy barriers, provide the appropriate basis for this comparison.

### 3.2 Second Stage: Oligomerization and Cyclization

As illustrated in Figure 7, the oligomerization and cyclization mechanism proceeds through successive ethene adsorption and C–C coupling steps, followed by intramolecular cyclization to generate the C_6_ cyclic intermediate. The calculated energy profile reveals that chain growth and ring closure are dependent on the interaction between the Ga active site and the progressively larger hydrocarbon fragment.

**FIGURE 7:**
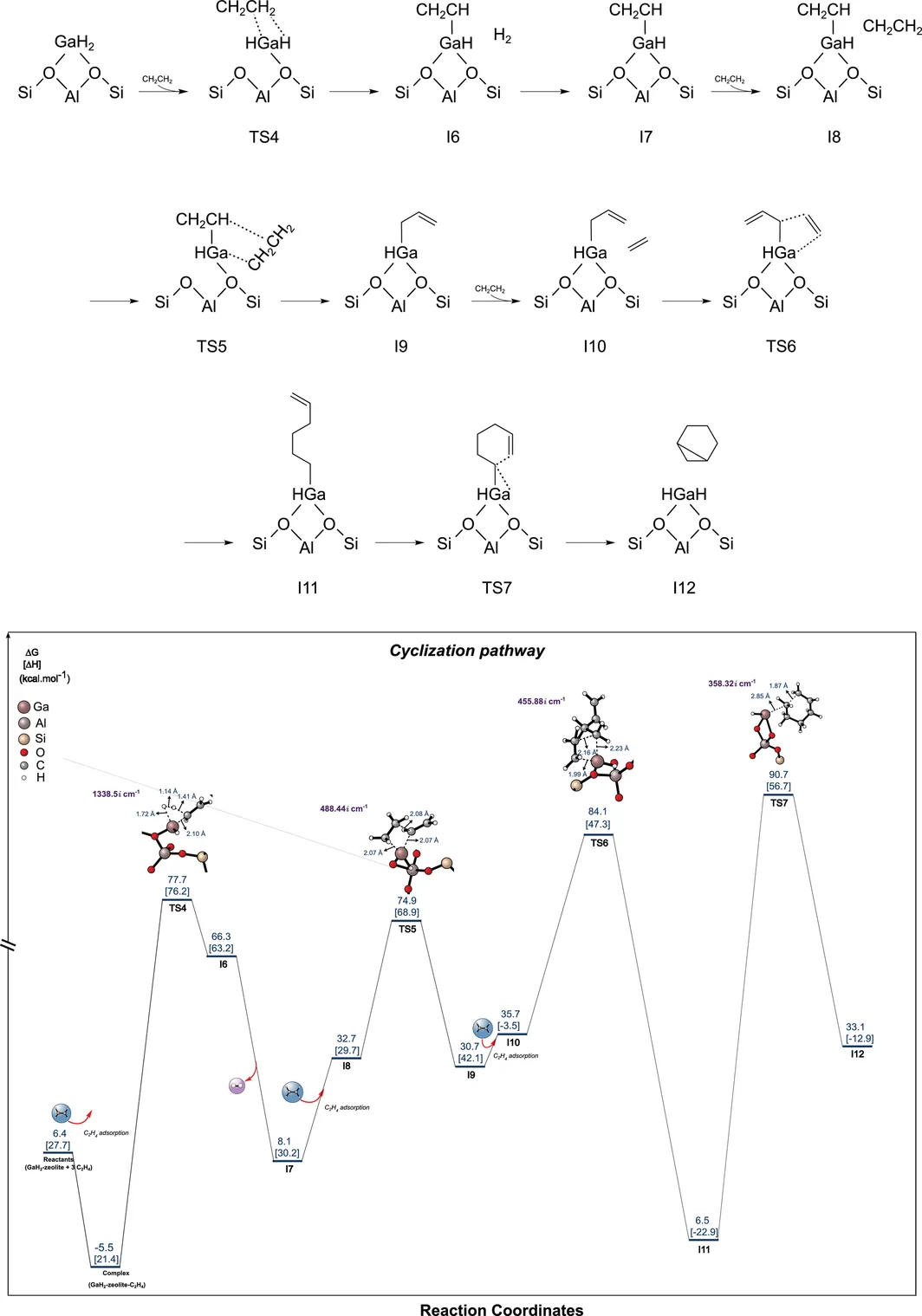
Ethene cyclization mechanism on [GaH_2_]‐ZSM‐5 and its energy profile. For clarity, only the active site region of the transition states is displayed.

The process begins with the adsorption of ethene on the [GaH_2_]‐ZSM‐5 active site, forming the C_2_H_4_‐[GaH_2_]‐ZSM‐5 complex. The adsorption process is associated with a Δ*G*_ads_ of −11.9 kcal mol^−1^. The adsorbed ethene subsequently undergoes dehydrogenation via TS4, which lies at 77.7 kcal mol^−1^ above the reference state, leading to intermediate I6 at 66.3 kcal mol^−1^. Thus, the local activation barrier for this transformation is 83.2 kcal mol^−1^ relative to the preceding adsorbed complex. At TS4, simultaneous cleavage of a C–H bond in ethene and a Ga–H bond occurs, allowing the two hydrogen atoms to combine and form H_2_. Subsequent removal of H_2_ yields the Ga‐bound hydrocarbon species that can undergo further chain‐growth reactions.

A second ethene molecule then enters the catalytic cycle, leading to intermediate I8 at 32.7 kcal mol^−1^. The first C–C coupling occurs via TS5, at 74.9 kcal mol^−1^, resulting in the formation of the C_4_ intermediate I9 at 30.7 kcal mol^−1^. The corresponding local activation barrier is 42.2 kcal mol^−1^ relative to I8. Structurally, TS5 is characterized by the approach of the Ga‐bound carbon fragment toward the C=C bond of the adsorbed ethene molecule, resulting in the formation of a new C–C bond and propagation of the hydrocarbon chain.

The resulting C_4_ species subsequently interacts with another ethene molecule, producing intermediate I10 at 35.7 kcal mol^−1^. The second C–C coupling step proceeds via TS6 at 84.1 kcal mol^−1^, with a local activation barrier of 48.4 kcal mol^−1^, and leads to intermediate I11 at 5.8 kcal mol^−1^. Although TS6 exhibits a structure analogous to that of TS5, the larger hydrocarbon fragment introduces greater steric constraints within the zeolite cavity. These constraints may hinder the proper orientation of the reacting carbon atoms and consequently make the second chain‐growth event less favorable than the first. Nevertheless, the relatively low energy of I11 indicates that the formation of the C_6_ hydrocarbon intermediate becomes energetically accessible once the transition state has been overcome.

The subsequent cyclization step represents the most energetically demanding transformation of the entire oligomerization and cyclization sequence. From I11, the hydrocarbon chain undergoes intramolecular ring closure through TS7, which lies at 90.7 kcal mol^−1^, to form I12, at 33.1 kcal mol^−1^. This corresponds to an activation barrier of 84.2 kcal mol^−1^ relative to I11, making TS7 the highest‐energy transition state in the entire reaction profile. At TS7, the Ga‐bound carbon approaches the terminal carbon of the hydrocarbon chain, accompanied by hydrogen transfer involving the Ga–H/alkyl moiety. This reorganization promotes the formation of the new C–C bond and closure of the hydrocarbon framework, while contributing to the regeneration of the Ga hydride active site. The resulting I12 contains the characteristic fused‐ring structure generated by the cyclization process.

The high activation energy associated with TS7 can be rationalized not only in terms of the electronic reorganization required for C–C bond formation, but also by the conformational and entropic constraints imposed on the growing hydrocarbon chain. In intermediate I11, the C_6_ fragment adopts a relatively extended and flexible structure. Cyclization requires the two reacting termini to approach each other with a specific orientation inside the confined zeolite environment, resulting in a substantial loss of conformational freedom and an unfavorable entropic contribution. Consequently, the ring‐closure event constitutes the principal kinetic bottleneck of this stage.

Accordingly, TS7 and I11 can be identified as the TDTS and TDI, respectively, for the cyclization. The high energy of TS7 suggests that the formation of the cyclic precursor is considerably more demanding than the preceding C–C coupling reactions. This result indicates that, within the present [GaH_2_]‐ZSM‐5 model, cyclization rather than chain growth controls the kinetics of the transformation toward the cyclic intermediate. The occurrence of a high‐energy ring‐closure transition state is consistent with the findings of Fang et al. for Ga‐FAU systems [38].

As shown in Figure S10, the IRI analysis shows that a repulsive region can be identified during the cyclization stage. This is associated with the interaction of the ring structure with the zeolite framework.

### 3.3 Third Stage: Aromatization

The aromatization pathway of the C_6_ cyclic intermediate is presented in Figure 8. In contrast to the cyclization stage, the aromatization sequence proceeds through a series of hydrogen‐transfer, C–H activation, H_2_ formation, and dehydrogenation events.

**FIGURE 8:**
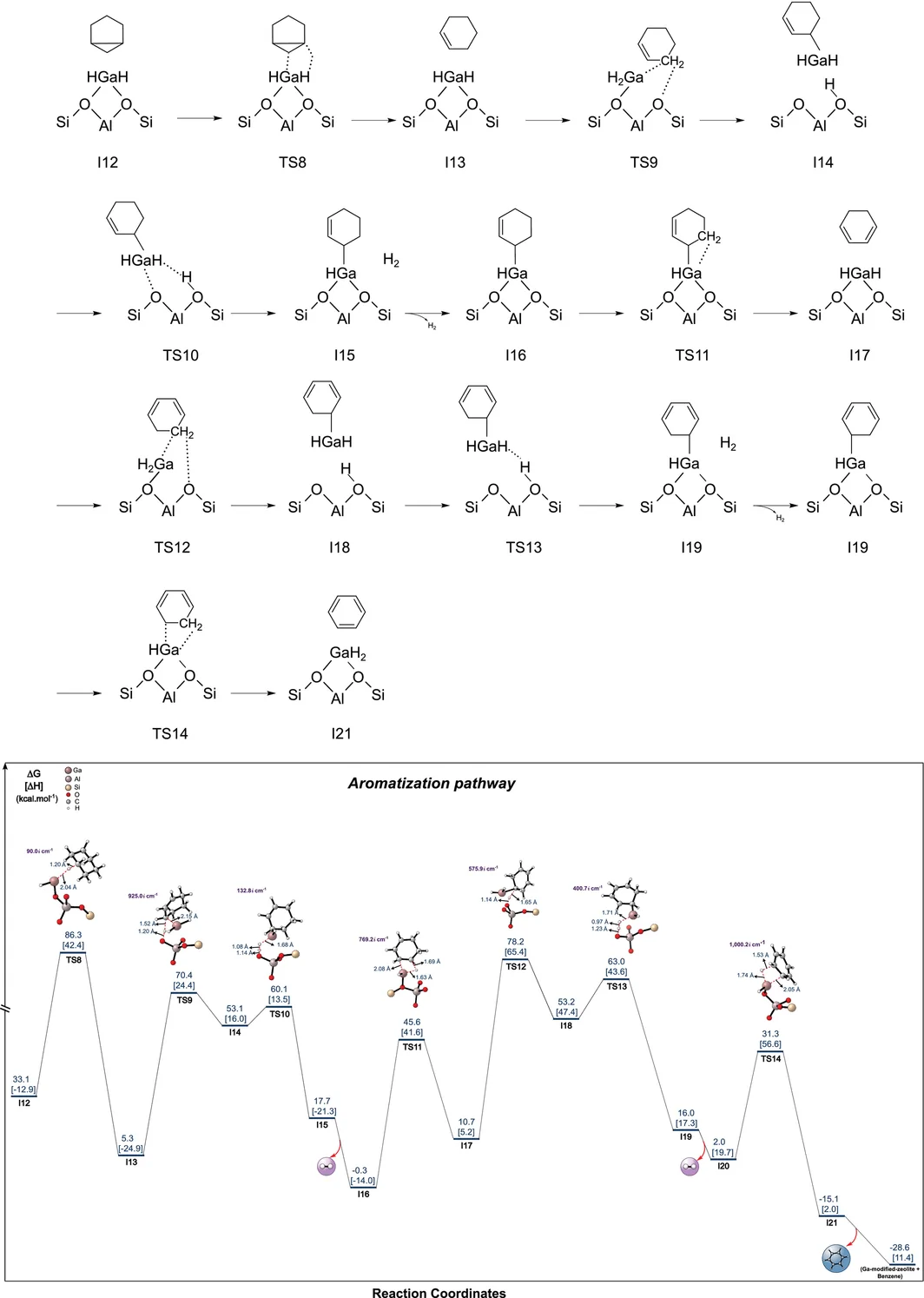
Aromatization mechanism on [GaH_2_]‐ZSM‐5 and its energy profile. For clarity, only the active site region of the transition states is displayed, omitting the extended zeolite framework.

The aromatization sequence starts from intermediate I12, which lies at 33.1 kcal mol^−1^ and undergoes ring rearrangement through TS8, which lies at 86.3 kcal mol^−1^. This transformation has an activation barrier of 53.2 kcal mol^−1^ and produces I13, at 5.3 kcal mol^−1^. The structural changes associated with TS8 involve the opening and rearrangement of the strained fused‐ring system formed during the preceding cyclization step, providing the structural arrangement required for subsequent dehydrogenation.

From I13, hydrogen transfer between the hydrocarbon fragment and the Ga center occurs through TS9, which lies at 70.4 kcal mol^−1^, leading to I14, at 53.1 kcal mol^−1^. This step has an activation barrier of 65.1 kcal mol^−1^. The transition state involves C–H activation within the hydrocarbon fragment and concomitant interaction with the Ga center, highlighting the important role of the gallium site in initiating the dehydrogenation sequence.

The system subsequently proceeds through TS10, which lies at 60.1 kcal mol^−1^, with a relatively small additional barrier of 7.0 kcal mol^−1^ relative to I14, leading I15 at 17.7 kcal mol^−1^. This step is followed by H_2_ formation and desorption, yielding I16 at −0.3 kcal mol^−1^. Thus, the hydrogen‐transfer and H_2_‐elimination events are energetically distinguishable rather than occurring as a single concerted process. Further dehydrogenation occurs through TS11 at 45.6 kcal mol^−1^, corresponding to an activation barrier of 45.9 kcal mol^−1^ relative to I16 and producing I17 at 10.7 kcal mol^−1^. In this step, hydrogen is abstracted from the cyclic hydrocarbon and transferred to the Ga center, increasing the degree of unsaturation of the carbon structure.

The resulting intermediate I17 subsequently undergoes another hydrogen‐transfer and structural‐reorganization step via TS12, which lies at 78.2 kcal mol^−1^, leading to I18 at 53.2 kcal mol^−1^. The corresponding activation barrier is 67.5 kcal mol^−1^, making TS12 the transition state with the highest local activation barrier within the aromatization sequence. This step represents another significant kinetic bottleneck in the progressive dehydrogenation of the cyclic hydrocarbon. The following transformation proceeds through TS13, which lies at 63.0 kcal mol^−1^, leading to I19 at 16.0 kcal mol^−1^. In contrast to TS9 and TS12, this step presents a relatively small activation barrier of only 9.8 kcal mol^−1^ relative to I18. Therefore, TS13 can be regarded as a relatively facile hydrogen rearrangement within the increasingly unsaturated hydrocarbon structure. Subsequent H_2_ formation and desorption lead to I20, at 2.0 kcal mol^−1^.

Finally, the remaining dehydrogenation occurs through TS14, which lies at 31.3 kcal mol^−1^, with an activation barrier of 29.3 kcal mol^−1^ relative to I20. This transformation produces I21 at −15.1 kcal mol^−1^, corresponding to the fully dehydrogenated aromatic product. In this final transition state, hydrogen is abstracted from the carbon skeleton and transferred to the Ga center, completing the formation of the conjugated aromatic structure. The subsequent stabilization toward the final benzene‐containing product further demonstrates the thermodynamic driving force of the overall aromatization process.

Overall, the aromatization mechanism is characterized by successive and mechanistically distinct hydrogen‐transfer and H_2_‐elimination events, accompanied by the progressive formation of C=C bonds and increasing conjugation within the hydrocarbon structure. Among the elementary steps of this stage, TS12 exhibits the largest local activation barrier, at 67.5 kcal mol^−1^, closely followed by TS9, with a barrier of 65.1 kcal mol^−1^. Therefore, TS12 represents the highest local barrier within the aromatization pathway, although both barriers (TS9 and T12) contribute significantly to the kinetic difficulty of the dehydrogenation sequence.

### 3.4 Impact of the Cluster Size on the Catalytic Cycle

The influence of the size and boundary effects of the zeolite cluster on the ETA process with the [GaH_2_]^+^–ZSM‐5 catalyst was examined by comparing two cluster models, Figure 1a,b, under the stepwise mechanism. The comparative profile is shown in Figure S11.

During the dehydrogenation of ethane, the larger cluster follows the same mechanisms as the smaller one but yields different energetics. With the larger cluster, the free energy barriers for TS1, TS2, and TS3 increased by up to almost 12 kcal mol^−1^ relative to the smaller cluster. For the C–H activation, TS1 lies at 70.2 kcal mol^−1^, while the H_2_ formation and *β*‐hydride transfer lie at 61.2 and 62.9 kcal mol^−1^, respectively. In both clusters, the rate‐determining transition state remains unchanged.

For the cyclization stage, an increase in barrier energies is also observed with the larger cluster. All the transition states in this stage, from TS4 to TS7, increased by up to almost 12 kcal mol^−1^ relative to the smaller cluster. Particularly, the TS7, which is the highest‐energy transition state in the entire reaction profile, is shifted from 90.7 kcal mol^−1^ (small cluster) to 99.3 kcal mol^−1^ (large cluster). A difference is noted between models; for the larger cluster, the formation of the fused three‐membered‐five‐membered (corresponding to I12 in the Figure 7) is not observed; in this model, the intermediate is a cyclohexyl substituent bound to the Ga center, Figure S12.

With the larger cluster model, the aromatization stage starts with a *β*‐hydride transfer with a barrier of 60.8 kcal mol^−1^ (TS8). This process forms cyclohexene (I13), which then proceeds to a C–H activation, resulting in TS9, which lies at 73.1 kcal mol^−1^, approximately 6 kcal mol^−1^ higher in energy than the smaller‐cluster counterpart. This transition‐state structure is composed of a Brønsted site (H‐ZSM‐5) and a cyclohexen‐2‐yl group at the gallium center. The mechanism proceeds by the transformation of TS9 directly into I15, in which neither the I14 intermediate nor the TS10 structure is obtained within the larger model. Similarly, TS12 occurs barrier‐free, producing intermediate I19.

Overall, except for TS8, all the transition states are higher in energy by up to 30 kcal mol^−1^ relative to the smaller cluster, because the confined space of the channel intersection, which is more accurately represented with the larger cluster, restricts the formation of voluminous species. Consequently, extending the cluster model provides a more complete representation of repulsive wall effects, minimizing over‐relaxation and altering the energetics, in agreement with the IRI analysis (Figure S10) and with previous results [62] regarding truncated and extended cluster models.

Regardless of the cluster size, the overall kinetics of the process are controlled by the cyclization step rather than by the final dehydrogenation leading to the aromatic product. This result differs from the mechanism proposed by Fang et al. [38] for Ga‐FAU zeolites, in which the final *β*‐hydride elimination associated with benzene formation was identified as the turnover‐determining transition state. Our results show that an energetic bottleneck occurs earlier, at TS7, during the formation of the cyclic C_6_ intermediate. This difference highlights the potential influence of zeolite topology and active‐site environment on the relative stability of the hydrocarbon intermediates and transition states. In particular, the confined ZSM‐5 environment may impose additional geometric and conformational constraints on the long‐chain intermediate, increasing the energetic cost of bringing the reacting carbon atoms into proximity for intramolecular C–C bond formation.

Furthermore, the present results support a mechanistic picture in which oligomerization, cyclization, and aromatization are kinetically differentiated processes. Chain growth through successive C–C coupling events generates the C_6_ precursor, but the subsequent conformational reorganization required for cyclization constitutes the dominant kinetic barrier. Once the cyclic intermediate is formed, aromatization proceeds through a sequence of hydrogen‐transfer and dehydrogenation reactions, ultimately yielding the thermodynamically stabilized aromatic product. The stepwise character of this latter process is consistent with the mechanism reported by Mo et al. [40] for cyclohexane dehydrogenation over [GaH_2_]‐ZSM‐5, further supporting the role of gallium hydride sites in mediating sequential C–H activation and hydrogen elimination.

## 4 Conclusions

The Ga^+^, [GaO]^+^, and [GaH_2_]^+^ species in the ZSM‐5 zeolite were theoretically investigated as an active site for the ethane aromatization process using two cluster models and a combination of r^2^SCAN and *ω*B97X‐D4rev functionals.

The ETA cycle comprises three stages: ethane dehydrogenation (EDH), cyclization, and aromatization. For the initial EDH stage, the three different Ga species were evaluated for both concerted and stepwise mechanisms. The results indicate that the stepwise mechanism is preferred for the initial ethane dehydrogenation step, except over GaO‐I, for which the two mechanisms exhibit similar energetics. Within the EDH stepwise mechanism, the [GaO]^+^ pathways presented comparatively lower barriers for the initial steps, but the regeneration of the active site incurs a substantial energetic penalty, particularly for the carbenium pathway. The Ga^+^ pathway also exhibited high activation barriers, including a 70.6 kcal mol^−1^ barrier for ethene formation and active‐site regeneration. In comparison, the [GaH_2_]^+^ pathway exhibits a maximum barrier of 68.7 kcal mol^−1^ for the initial C–H bond activation, followed by lower barriers of 54.1 and 51.2 kcal mol^−1^ for H_2_ formation and ethene generation, respectively. Thus, although ethane activation over [GaH_2_]^+^ remains energetically demanding, the subsequent steps are more accessible, allowing the active site to be regenerated without the severe energetic penalty observed for the [GaO]^+^ and Ga^+^ pathways. These findings support the selection of [GaH_2_]^+^ as the representative Ga species for the subsequent investigation of the complete ETA mechanism.

The initial stage, corresponding to ethane dehydrogenation, is governed by heterolytic C–H bond activation (TS1), which represents one of the highest energy barriers in the EDH stage. Once this barrier is overcome, the formation of ethene and H_2_ proceeds through comparatively accessible steps, regenerating the [GaH_2_]^+^ active site and enabling further reactions. In the second stage, the oligomerization and cyclization of ethene involve successive C–C coupling reactions that lead to chain growth and the formation of C_6_ intermediates. The increase in the effective barrier for the second oligomerization step reflects the growing steric constraints associated with larger hydrocarbon fragments within the ZSM‐5 channels. These confinement effects play a dual role: they stabilize reactive intermediates while simultaneously imposing geometric restrictions that influence the energetics of C–C bond formation. The subsequent cyclization yields a cyclic C_6_ intermediate that serves as the key precursor for aromatization. The final aromatization stage proceeds through successive hydrogen transfer and H_2_ elimination steps, progressively increasing the degree of unsaturation of the cyclic intermediate. This sequence demonstrates that each elementary transformation, C–H activation, hydrogen transfer, H_2_ formation, and catalyst regeneration occurs in a stepwise manner, highlighting the critical role of the ZSM‐5 framework in promoting a sequential reaction pathway.

The overall ETA mechanism on the [GaH_2_]^+^site in the Ga‐ZSM‐5 zeolite reveals a complex interplay between dehydrogenation, C–C coupling, and hydrogen transfer steps, ultimately leading to benzene formation. The reaction pathway proceeds through a sequence of kinetically demanding elementary steps (TS6 and TS7), where the nature of the active site and the confinement imposed by the zeolite framework play a crucial role in stabilizing intermediates and transition states. The modeled EDH and cyclization mechanisms remain unchanged when employing a larger cluster model. Conversely, an extended representation of the zeolite cavity does impact the energetics of the aromatization stage. Ultimately, however, the overall kinetics of the ETA process are controlled by the cyclization step rather than the final dehydrogenation leading to the aromatic product, irrespective of the cluster size utilized.

## Funding

This work was supported by the São Paulo Research Foundation (FAPESP), 2023/13760‐5, 2024/15049‐0, 2015/01491‐3, 2019/17874‐0, and 2013/08293‐7; Brazilian National Agency of Petroleum, Natural Gas and Biofuels (ANP). Research project 4179, n° ANP 24333‐7; CNPq, 313720/2023‐1; Coordenação de Aperfeiçoamento de Pessoal de Nível Superior (CAPES), Finance Code 001, Program 33002010191P0; National Laboratory for Scientific Computing (LNCC/MCTI, Brazil).

## Conflicts of Interest

The authors declare no conflicts of interest.

## Supporting information


**Table S1:** Electronic adsorption energy, *E*_ads_, in kcal mol^−1^, for ammonia and pyridine in H‐ZSM‐5 obtained using the small cluster, hybrid (2‐ONIOM), and periodic boundary conditions (PBC) approaches.
**Table S2:** Charge distribution of selected atoms, in a.u., for the [GaH_2_]‐ZSM‐5 structure. The atom labels correspond to Figure S2.
**Figure S1:** 2‐ONIOM model with 239 atoms represented in high‐level (balls and sticks) and 722 atoms in low‐level (wireframes). Si atoms are depicted in gray, O atoms in red, and H atoms in white.
**Figure S2:** [GaH_2_]‐ZSM‐5 structure.
**Figure S3:** Enthalpy and Gibbs free‐energy profile for ethane dehydrogenation over the [GaH_2_]^+^ site of Ga–ZSM‐5. The blue pathway corresponds to the stepwise mechanism discussed in the main text, whereas the gray pathway represents the alternative concerted mechanism.
**Figure S4:** Optimized transition‐state structures for the ethane dehydrogenation pathways over the [GaH_2_]^+^ site of Ga–ZSM‐5. (a) Stepwise mechanism; (b) alternative concerted mechanism. Imaginary frequencies and selected interatomic distances associated with the corresponding bond‐breaking and bond‐forming events are indicated. For clarity, only the active‐site region of the Ga–ZSM‐5 cluster is shown.
**Figure S5:** Enthalpy and Gibbs free‐energy profile for ethane dehydrogenation over the Ga^+^ site of Ga–ZSM‐5. The orange pathway corresponds to the stepwise mechanism discussed in the main text, whereas the gray pathway represents an alternative concerted mechanism for ethane dehydrogenation.
**Figure S6:** Optimized transition‐state structures for the ethane dehydrogenation pathways over the Ga^+^ site of Ga–ZSM‐5. (a) Stepwise mechanism; (b) alternative concerted mechanism. Imaginary frequencies and selected interatomic distances associated with the corresponding bond‐breaking and bond‐forming events are indicated. For clarity, only the active‐site region of the Ga–ZSM‐5 cluster is shown.
**Figure S7:** Enthalpy and Gibbs free‐energy profiles for ethane dehydrogenation over the [GaO]^+^ site of Ga–ZSM‐5. The green pathway corresponds to the alkyl mechanism (GaO mechanism I), the purple pathway to the carbenium mechanism (GaO mechanism II), and the gray pathway to the alternative concerted mechanism.
**Figure S8:** Optimized transition‐state structures for the ethane dehydrogenation pathways over the [GaO]^+^ site of Ga–ZSM‐5. (a) GaO mechanism I (alkyl pathway) and the alternative concerted mechanism; (b) GaO mechanism II (carbenium pathway) and the alternative concerted mechanism. Imaginary frequencies and selected interatomic distances associated with the corresponding bond‐breaking and bond‐forming events are indicated. For clarity, only the active‐site region of the Ga–ZSM‐5 cluster is shown.
**Figure S9:** (a) Structure of the ethane adsorbed near the catalytic center. The zeolite cavity is represented by the dotted blue line, (b) removal of the first hydrogen, and (c) intermediate before the removal of the second hydrogen atom. In all steps, the zeolite framework stabilizes the Ga‐alkane via van der Waals forces.
**Figure S10:** Intermolecular interactions between the alkane, the gallium ion, and the zeolite framework. The colored region indicates the type of interaction. In (a), the alkane is stabilized by van der Waals forces with the zeolite structure. Interestingly, the interaction between the adsorbed alkane and the gallium atom is slightly repulsive. Therefore, explicit modeling of the cluster structure is needed to accurately predict reaction barriers in these systems. After the first hydrogen is removed from the alkane, an attractive interaction develops between the carbon and the metal, as seen in (b). After the second hydrogen atom is removed, the alkene is stabilized by van der Waals forces, and the catalyst is regenerated. The process repeats until the cyclization of hexane, where a new bond is formed and a repulsive region associated with the ring structure is formed (d).
**Figure S11:** Comparison of the Gibbs free‐energy profiles for ETA conversion over the [GaH_2_]‐ZSM‐5 catalyst, following the stepwise mechanism, for the small (70 T) and large (112 T) zeolite clusters. (a) Ethane dehydrogenation stage, (b) ethene cyclization stage, and (c) aromatization stage. Calculations were performed at the *ω*B97X‐D4rev/def2‐mTZVPP//r^2^SCAN‐3c level for the small cluster and at the *ω*B97X‐D4rev/def2‐mTZVPP//r^2^SCAN‐D4/def2‐SVP level for the large one. The BSSE correction was applied.
**Figure S12:** Structure of the I12 intermediate in ethane‐to‐aromatic conversion over the [GaH_2_]–ZSM‐5 catalyst, according to the stepwise mechanism. (a) Small cluster with 239 atoms and (b) large cluster with 379 atoms. Si atoms are depicted in light gray, O atoms in red, Al atom in pink, Ga atom in blue, C atoms in dark gray, and H atoms in white.

**Data S2:** Cartesian coordinates (XYZ format), electronic energies and free‐energy corrections of all optimized intermediates and transition states.

## Data Availability

The data that supports the findings of this study are available in the Supporting Information of this article.
