# Supplementary material for: Theoretical Investigation of Ethane‐to‐Aromatics Conversion Over [GaH2 ]‐ZSM‐5
Source: J Comput Chem. 2026 Nov 3;47(29):e70537. doi: 10.1002/jcc.70537 (PMC13730986; doi:10.1002/jcc.70537)
Supplement: Supplementary file 1 [file JCC-47-0-s001.pdf]

Supporting Information

Theoretical Investigation of Ethane-to-Aromatics Conversion over $[GaH_2]$-ZSM-5

João V. F. da Costa,[1] Thiago M. Mizumoto,[1] Lenin J. Diaz Soto,[1] Leonardo J. Duarte,[2] Leandro Martins,[3] Pedro Vidinha,[1] Liane Rossi,[1] and Ataualpa A. C. Braga.[,1]*

[1] Instituto de Química, Universidade de São Paulo, Av. Prof. Lineu Prestes, 748, São Paulo, 05508-000, Brazil.[2] Centro de Ciências Naturais e Humanas (CCNH), Universidade Federal do ABC (UFABC), Avenida dos Estados 5001, Santo André, São Paulo 09210-580, Brazil.[3] São Paulo State University (UNESP), Institute of Chemistry, Araraquara, SP, 14800-060, Brazil

SI.1 Ammonia and pyridine adsorption.

SI.1.1 Hybrid cluster (2-ONIOM).

Hybrid calculations were performed to assess the influence of the zeolite framework on the ammonia and pyridine adsorption energies using the ONIOM methodology with mechanical embedding, as implemented in the Gaussian 16 package (Revision C.01) [1]. The model consisted of 239 HL atoms and 722 LL atoms, as shown in **Figure S1**. For the HL region, the *ω*B97X-D with the D95 full double zeta basis set for Si and Al atoms; the rest of the atoms were treated with the def2-TZVPP basis set. The LL region was represented by the Universal Force Field (UFF) and the wave-function approach, Hartree-Fock (HF), with the minimal STO-3G basis set. This level of theory was already tested in previous work [2].

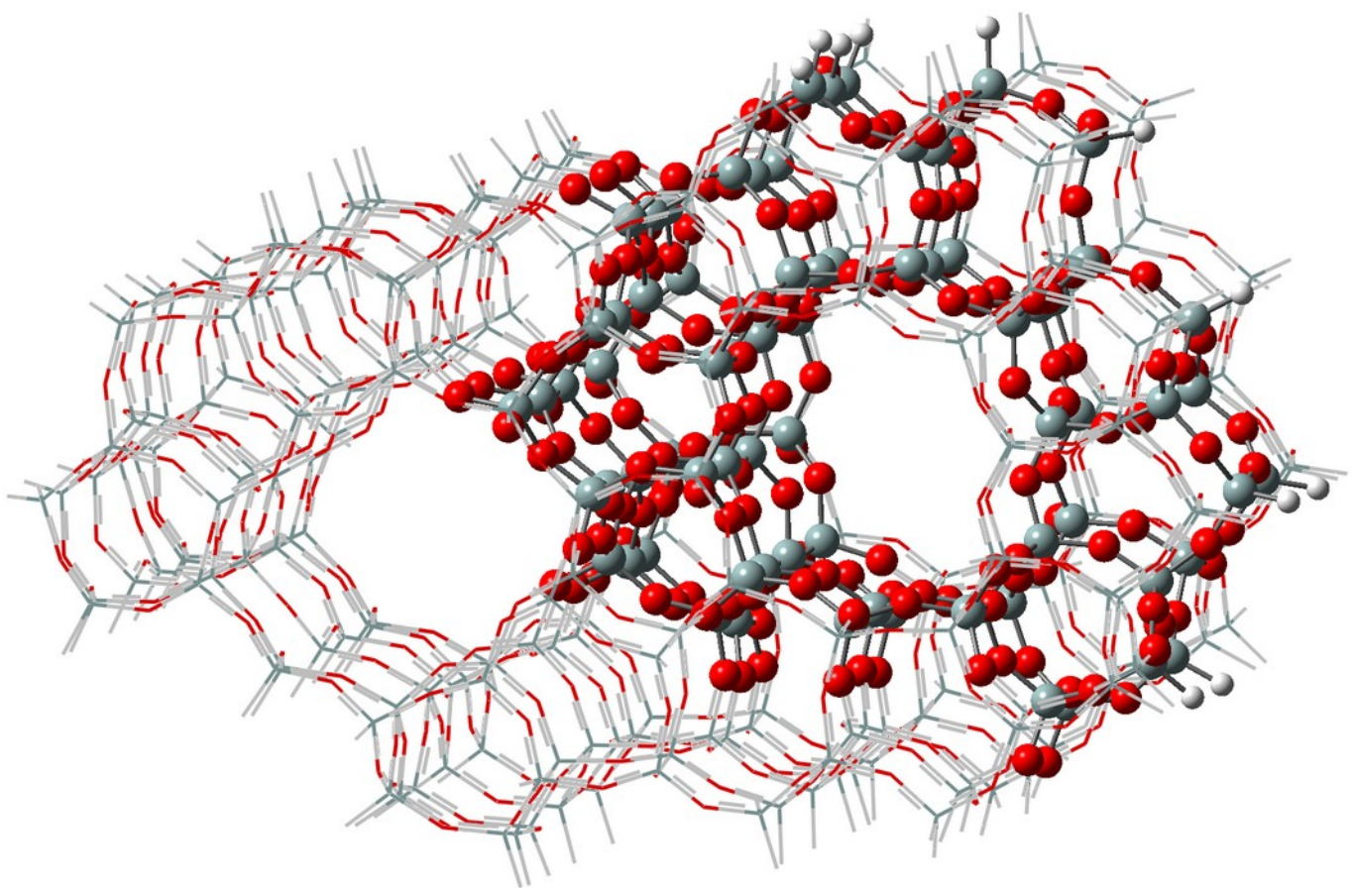

Figure S1. 2-ONIOM model with 239 atoms represented in high-level (balls and sticks) and 722 atoms in low-level (wireframes). Si atoms are depicted in gray, O atoms in red, and H atoms in white.

SI.1.2 Periodic boundary condition (PBC).

For periodic calculations, the unit cell from the work by van Koningsveld *et al.* [3] with parameters a = 20.022 Å, b = 19.899 Å, c = 13.383 Å, and $\alpha = \beta = \gamma = 90.0°$ was employed.

The PBE functional was employed with the Tkatchenko–Scheffler dispersion correction and Hirshfeld iterative partitioning. A Monkhorst–Pack k-point mesh of 1 × 1 × 1 was used. In the Vienna *ab initio* simulation package (VASP) version 5.4.4 [4], electron–ion interactions were treated with the projected augmented waves (PAW), and the plane wave energy cutoff was set to 520 eV. Atoms were allowed to relax until the change in electronic energy varied less than 0.01 meV and the forces were below the 0.01 eV/Å threshold.

Table S1. Electronic adsorption energy, E_{ads}, in kcal mol^{-1}, for ammonia and pyridine in H-ZSM-5 obtained using the small cluster, hybrid (2-ONIOM), and periodic boundary conditions (PBC) approaches.

Probe molecule	small cluster	2-ONIOM	PBC
ammonia	-35.2	-32.2	-34.6
pyridine	-42.8	-47.4	-46.6

SI.2 Charge analysis.

From the final optimized geometry of the [GaH_2]-ZSM-5 structure, the charge distributions were obtained using the CM5 model [5] with the *ω*B97X-D functional and the D95 full double-zeta basis set for Si and Al atoms. The Ga atom was treated with the SDD (ECP), and O and H atoms were treated with the def2-TZVPP basis set. These calculations were performed using the Gaussian 16 package (Revision C.01) [1].

Table S2. Charge distribution of selected atoms, in a.u., for the [GaH_2]-ZSM-5 structure. The atom labels correspond to **Figure S2**.

Atom	Ga	H1	H2	Al	O8	O20	O25	O26
charge	0.1982	0.0222	0.0234	0.6169	-0.3355	-0.4129	-0.4120	-0.3342

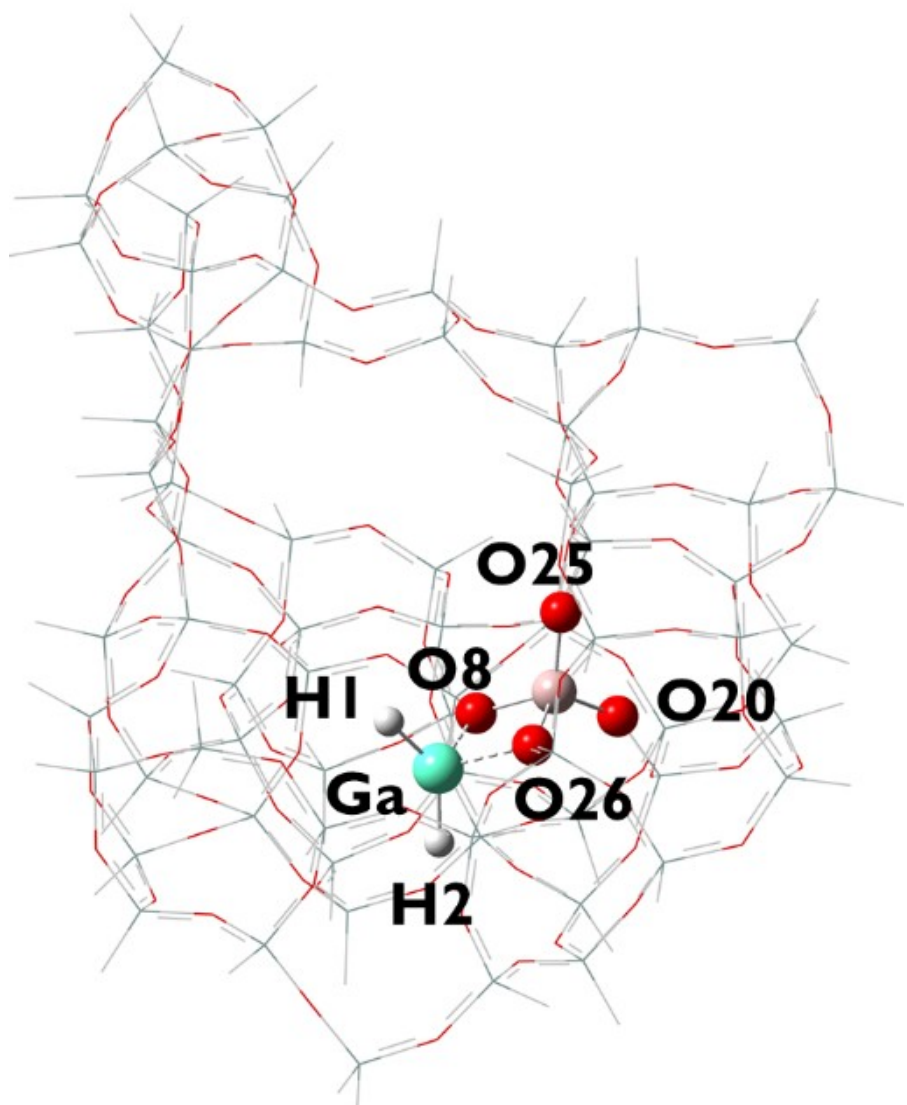

Figure S2. [GaH_2]-ZSM-5 structure.

SI.3 Anharmonic frequencies.

The anharmonic correction was obtained using second-order vibrational perturbation theory (VPT2), specifically the deperturbed VPT2 (DVPT2) approach [6], with the double-hybrid B2PLYP-D3 functional within a 2-layer ONIOM framework. Only the atoms in the [GaH_2]–AlO_4 region (Figure S2) were treated at the

B2PLYP-D3/def2-TZVPP level, while the rest of the structure was treated at the HF/STO-3G level. These calculations were performed using Gaussian 16 (Revision C.01) [1].

SI.4 Alternative mechanisms

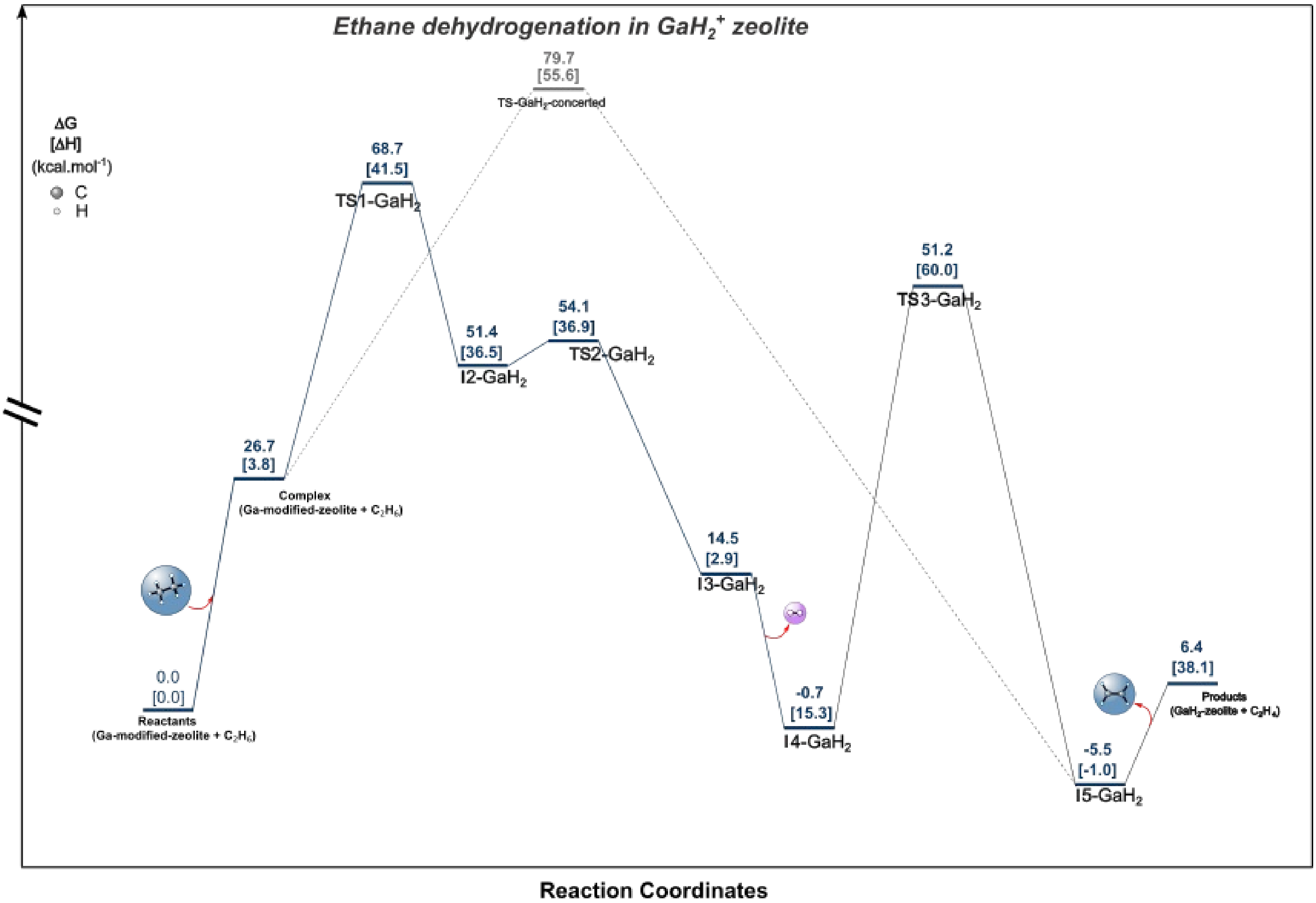

Figure S3. Enthalpy and Gibbs free-energy profile for ethane dehydrogenation over the $[GaH_2]^+$ site of Ga–ZSM-5. The blue pathway corresponds to the stepwise mechanism discussed in the main text, whereas the gray pathway represents the alternative concerted mechanism.

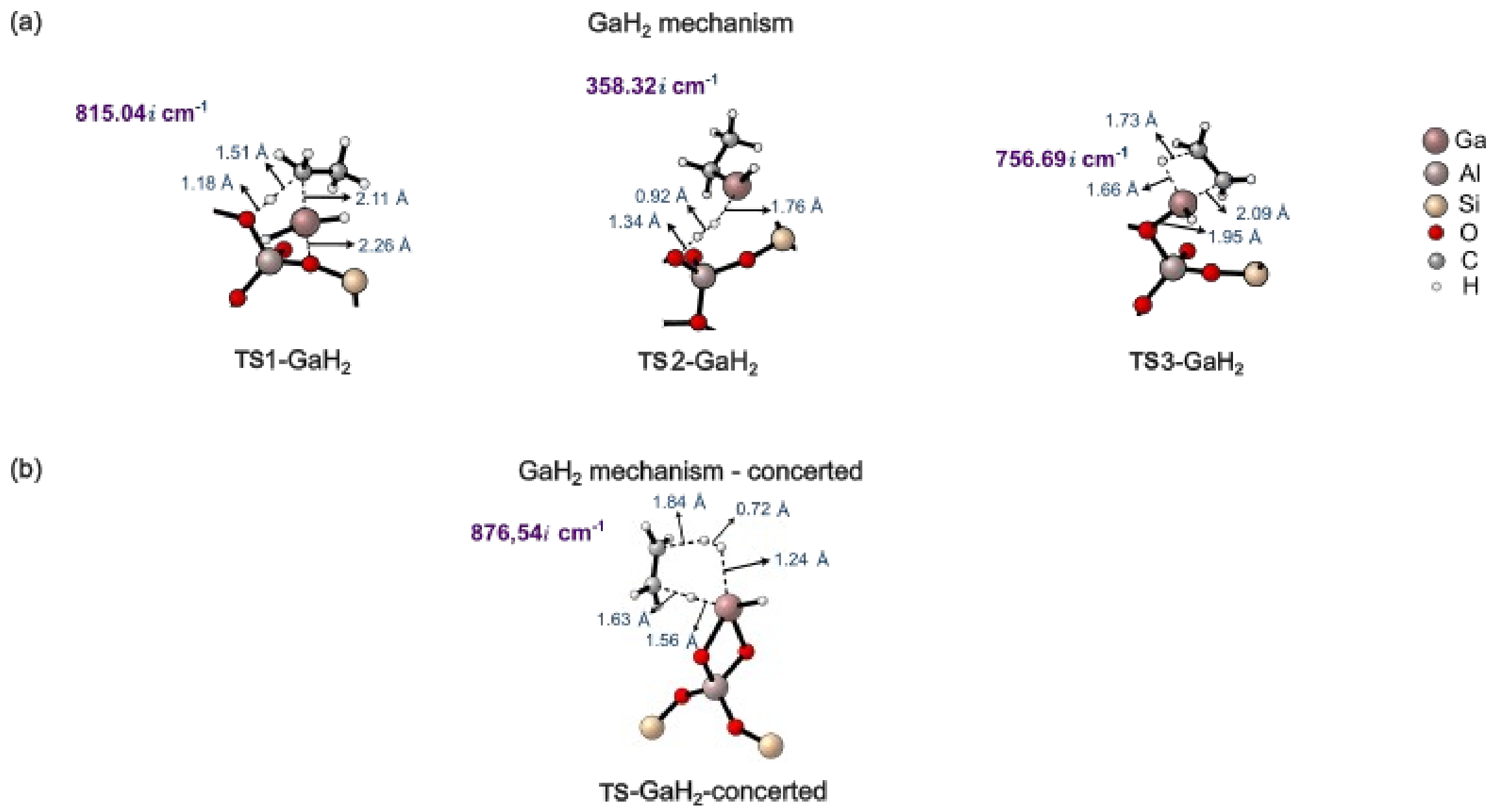

Figure S4. Optimized transition-state structures for the ethane dehydrogenation pathways over the $[GaH_2]^+$ site of Ga–ZSM-5. (a) Stepwise mechanism; (b) alternative concerted mechanism. Imaginary frequencies and selected interatomic distances associated with the corresponding bond-breaking and bond-forming events are indicated. For clarity, only the active-site region of the Ga–ZSM-5 cluster is shown.

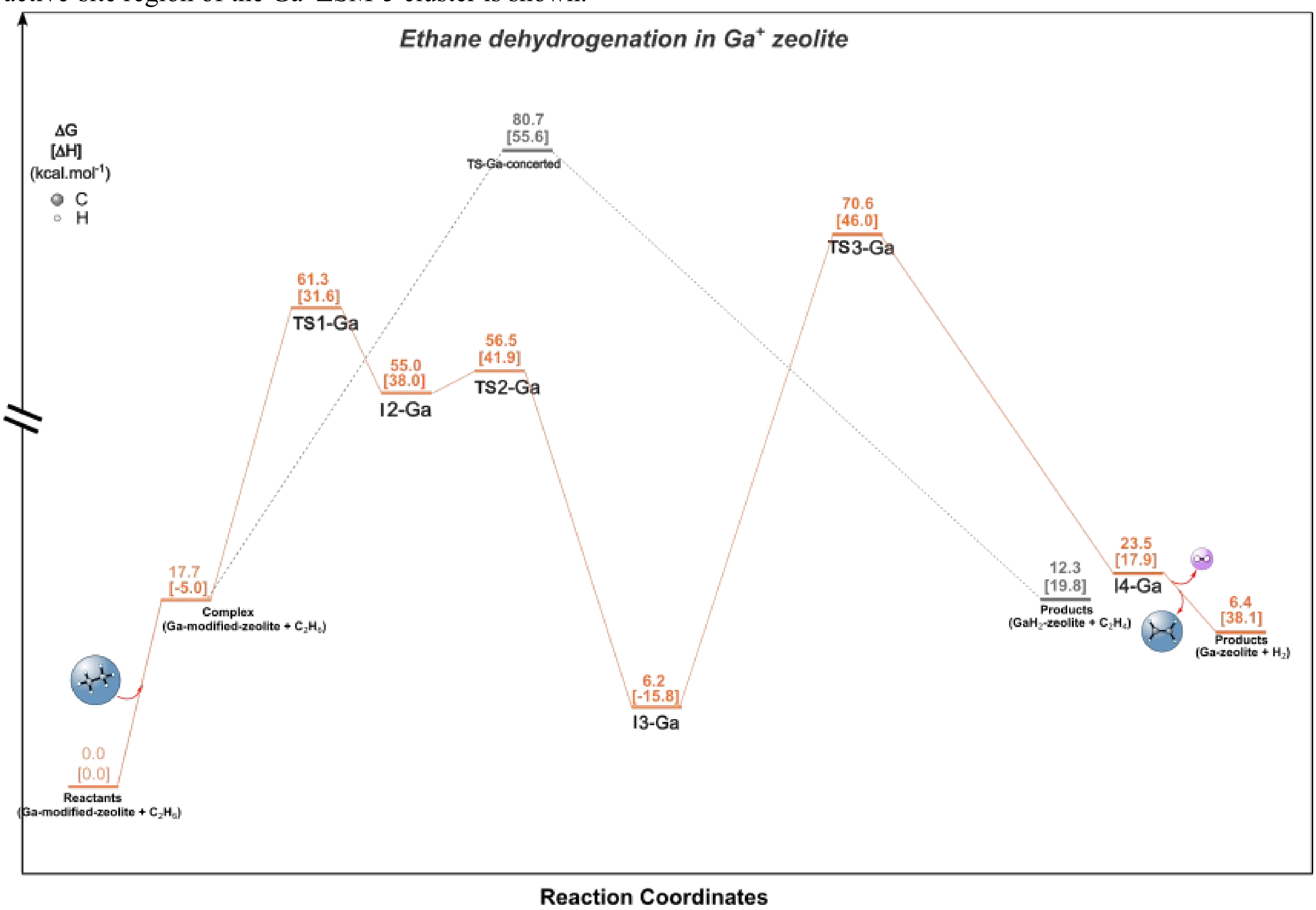

Figure S5. Enthalpy and Gibbs free-energy profile for ethane dehydrogenation over the Ga^+ site of Ga–ZSM-5. The orange pathway corresponds to the stepwise mechanism discussed in the main text, whereas the gray pathway represents an alternative concerted mechanism for ethane dehydrogenation.

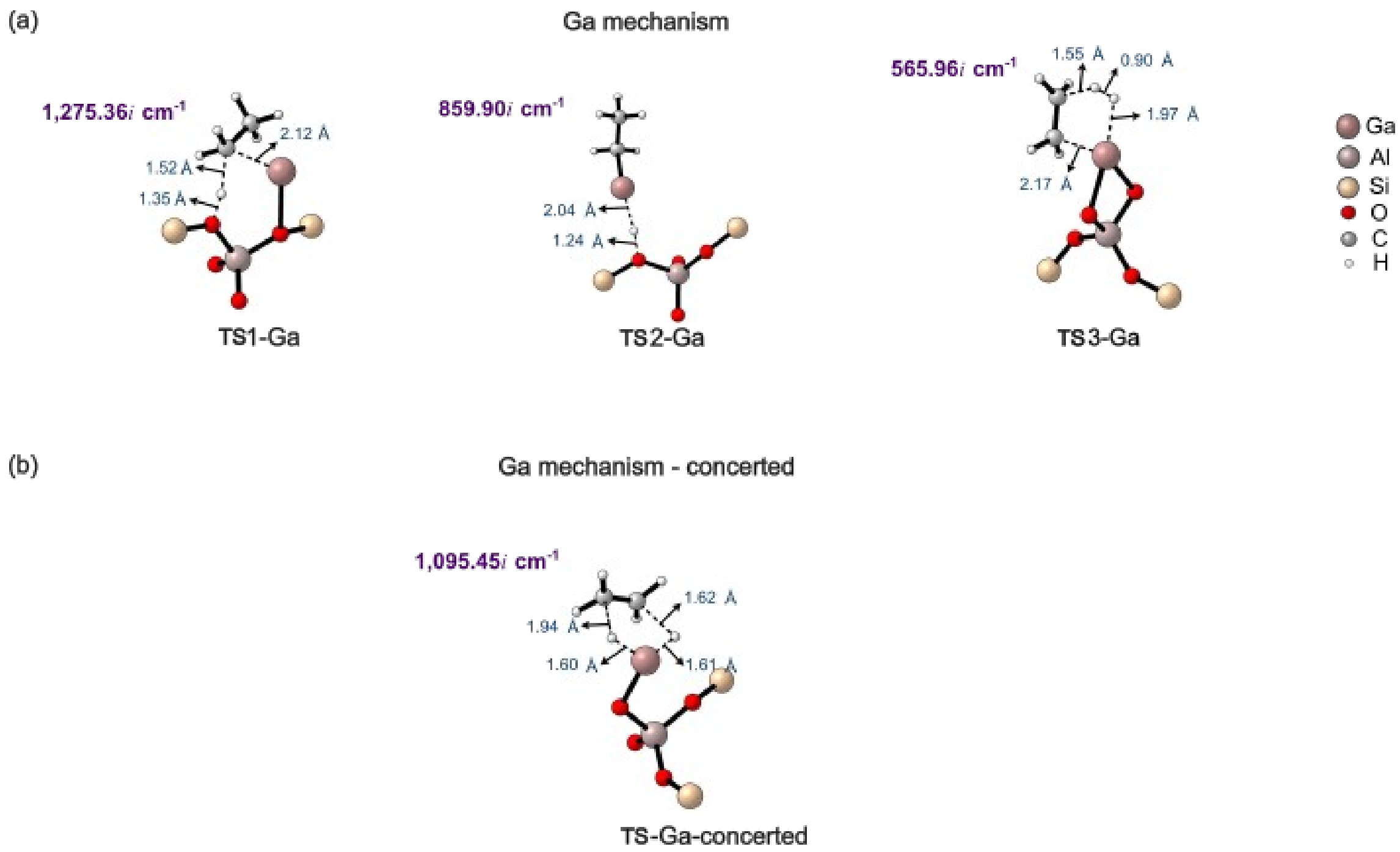

Figure S6. Optimized transition-state structures for the ethane dehydrogenation pathways over the Ga^{+} site of Ga–ZSM-5. (a) Stepwise mechanism; (b) alternative concerted mechanism. Imaginary frequencies and selected interatomic distances associated with the corresponding bond-breaking and bond-forming events are indicated. For clarity, only the active-site region of the Ga–ZSM-5 cluster is shown.

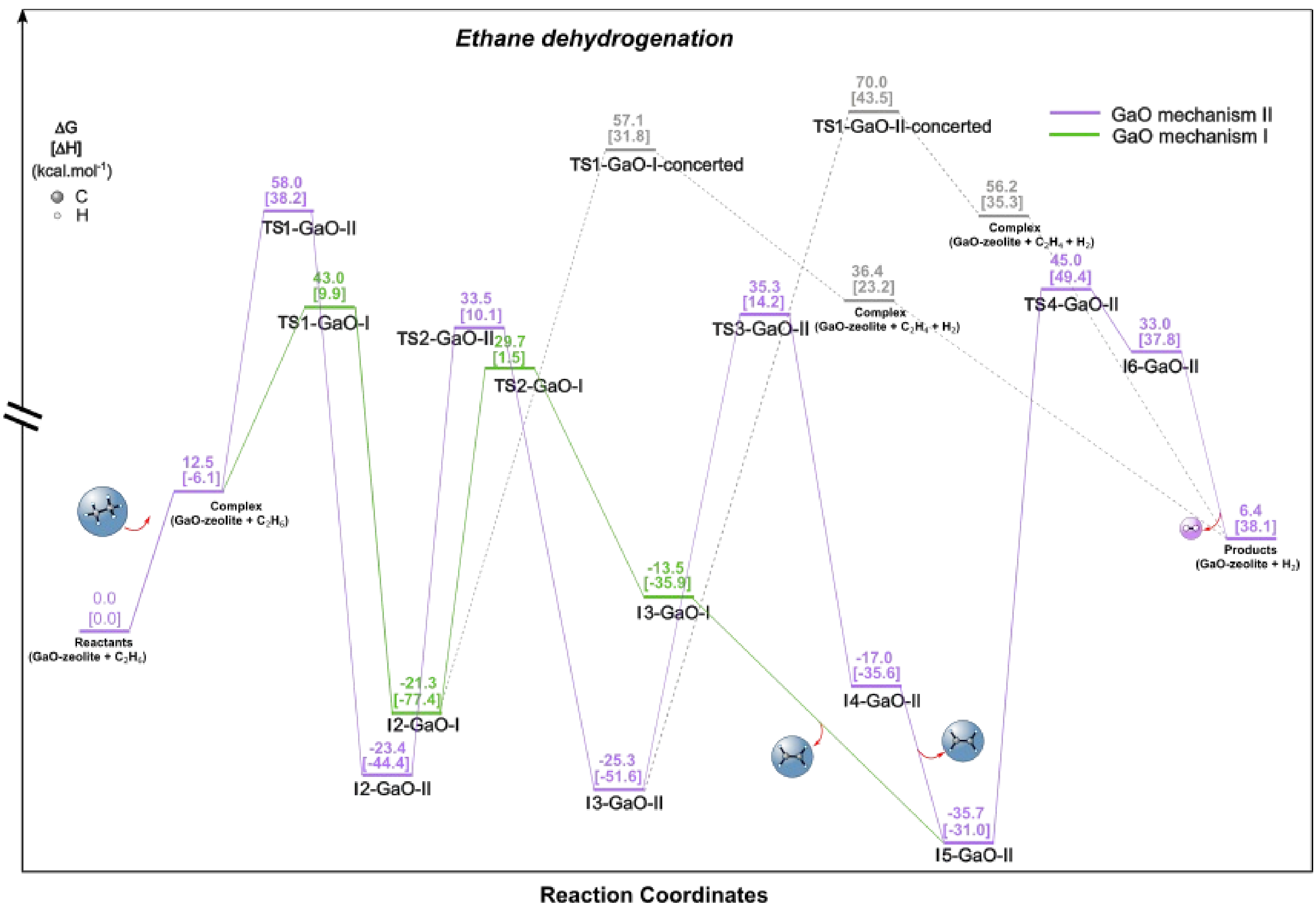

Figure S7. Enthalpy and Gibbs free-energy profiles for ethane dehydrogenation over the $[GaO]^+$ site of Ga–ZSM-5. The green pathway corresponds to the alkyl mechanism (GaO mechanism I), the purple pathway to the carbenium mechanism (GaO mechanism II), and the gray pathway to the alternative concerted mechanism.

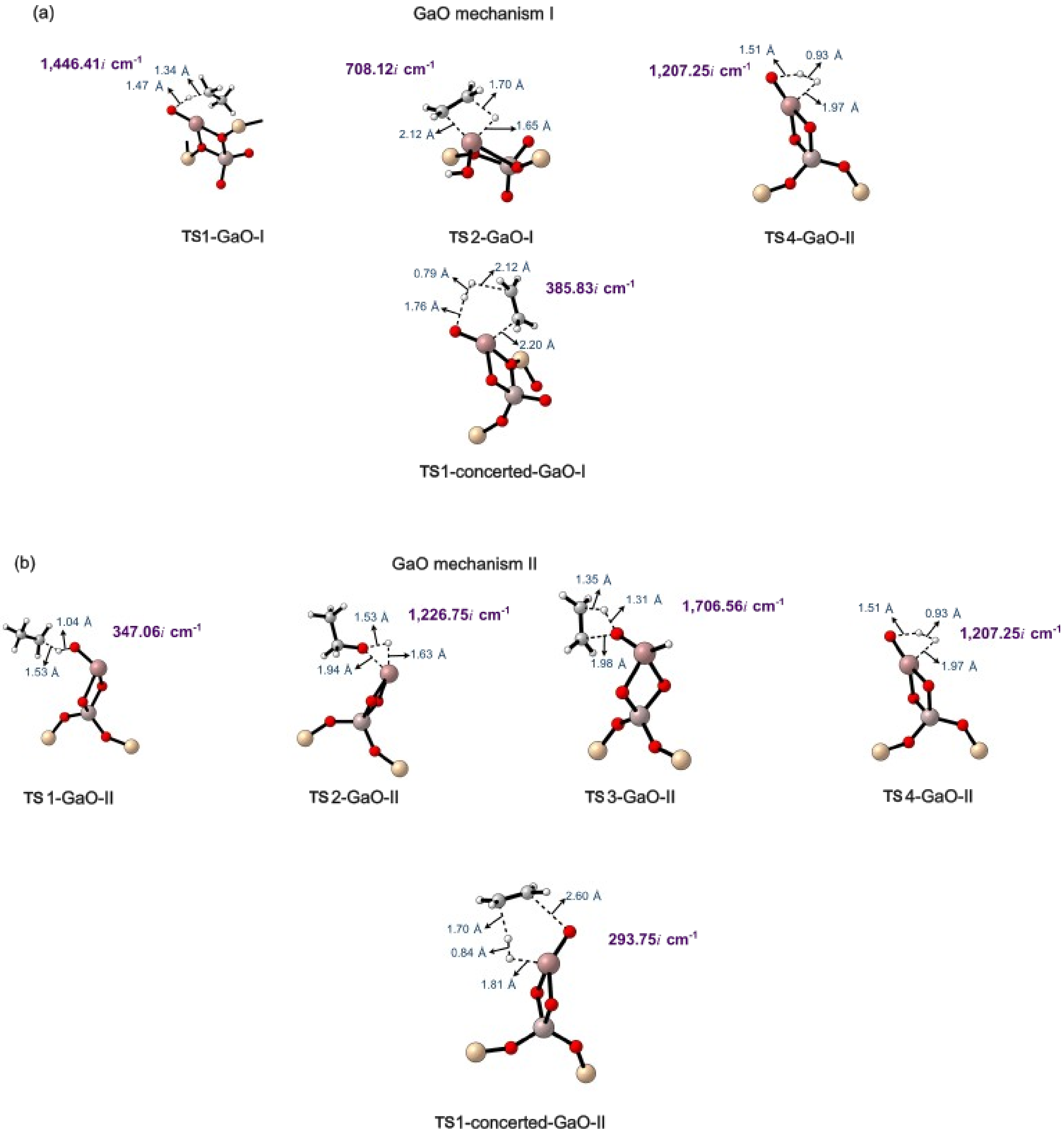

Figure S8. Optimized transition-state structures for the ethane dehydrogenation pathways over the $[GaO]^+$ site of Ga–ZSM-5. (a) GaO mechanism I (alkyl pathway) and the alternative concerted mechanism; (b) GaO mechanism II (carbenium pathway) and the alternative concerted mechanism. Imaginary frequencies and selected interatomic distances associated with the corresponding bond-breaking and bond-forming events are indicated. For clarity, only the active-site region of the Ga–ZSM-5 cluster is shown.

SI.5 IRI analysis.

An IRI analysis is performed on each intermediate and transition state of the catalytic cycle. A recurring pattern of intermolecular interactions, which appears and disappears at each step, is observed throughout the entire reaction. The principal steps are summarized in **Figure S9**.

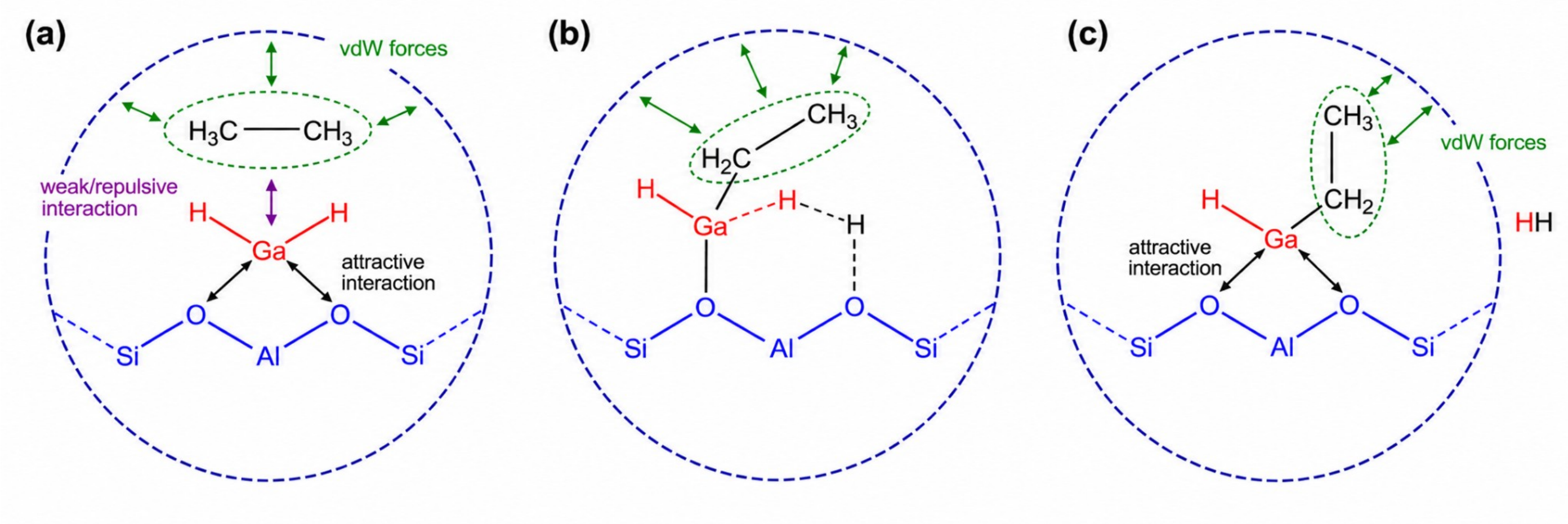

Figure S9. (a) Structure of the ethane adsorbed near the catalytic center. The zeolite cavity is represented by the dotted blue line, (b) removal of the first hydrogen, and (c) intermediate before the removal of the second hydrogen atom. In all steps, the zeolite framework stabilizes the Ga-alkane via van der Waals forces.

The analysis shows that alkane adsorption is strongly stabilized by van der Waals forces between the alkane and the zeolite framework, as indicated by the dotted blue line in **Figure S9**. These forces also contribute to the effective approximation of the alkane to the catalytic center, since there is a weak repulsive interaction between the Ga atom and the alkane.

After the removal of the first hydrogen atom, the Ga ion maintains its tetrahedral geometry and four attractive regions can be seen in the IRI analysis, as shown in **Figure S10**. At this step (I2), illustrated in **Figure S9**b, one of the attractive interactions between the Ga ion and the oxygen of the framework disappears; the metal interacts effectively with only one oxygen atom in the vicinity of the aluminum atom, and the vacant interactive site is taken by the alkane. At this point, the alkane keeps interacting with the framework via van der Waals forces. These interactions become more significant as the aliphatic chain length increases.

The third and final step is the formation and release of the hydrogen molecule (I3), as shown in **Figure S9**c, restoring the interaction of the metal with the two oxygens of the framework. The process repeats until cyclization.

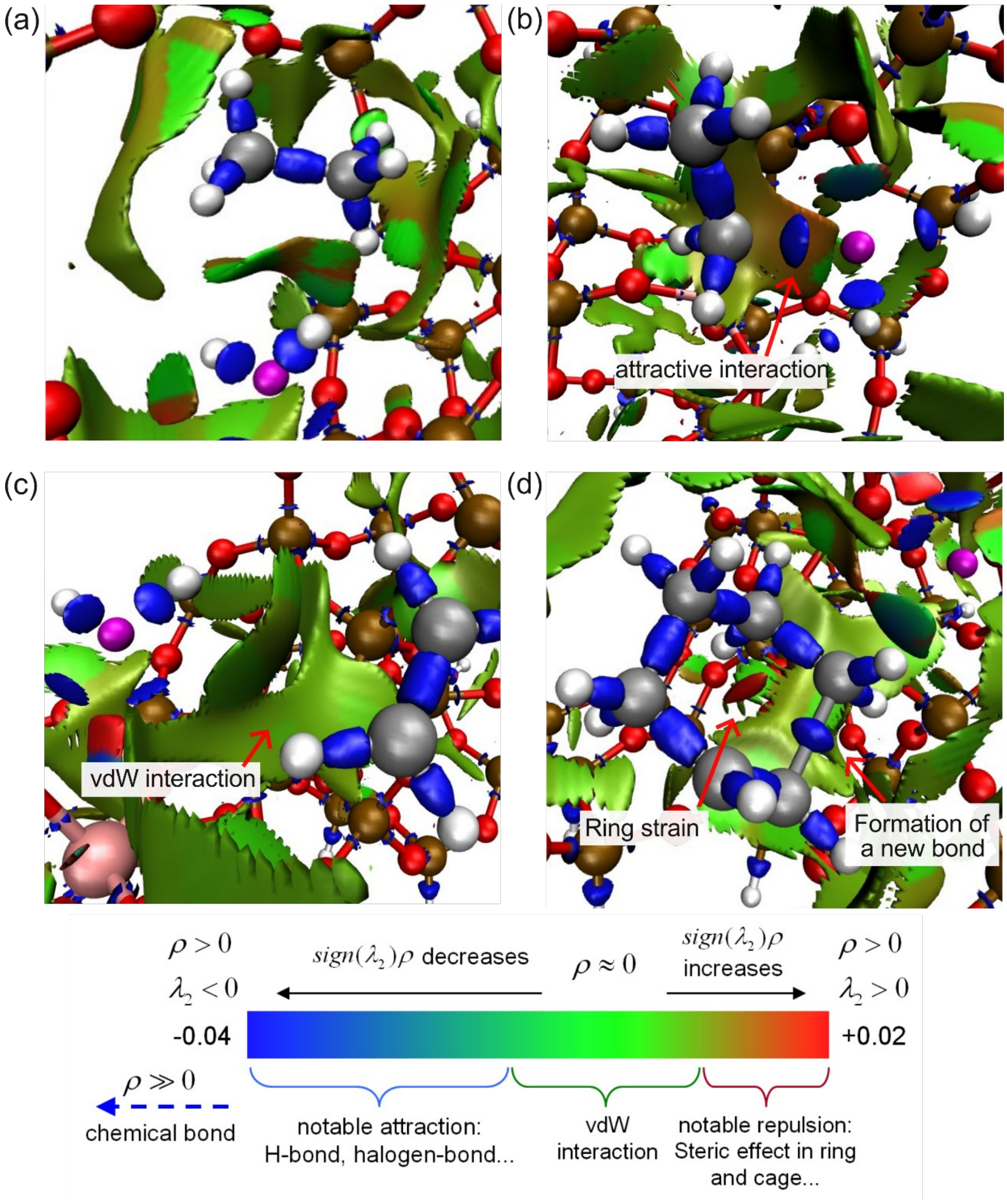

Figure S10. Intermolecular interactions between the alkane, the gallium ion, and the zeolite framework. The colored region indicates the type of interaction. In (a), the alkane is stabilized by van der Waals forces with the zeolite structure. Interestingly, the interaction between the adsorbed alkane and the gallium atom is slightly repulsive. Therefore, explicit modeling of the cluster structure is needed to accurately predict reaction barriers in these systems. After the first hydrogen is removed from the alkane, an attractive interaction develops between the carbon and the metal, as seen in (b). After the second hydrogen atom is removed, the alkene is stabilized by van der Waals forces, and the catalyst is regenerated. The process repeats until the cyclization of hexane, where a new bond is formed and a repulsive region associated with the ring structure is formed (d).

SI.6 Impact of the zeolite cluster size on the ETA conversion.

The influence of cluster size on the ETA process with the $[GaH_2]^+$–ZSM-5 catalyst within the stepwise mechanism was examined using a small cluster of 239 atoms (70T sites) and a large cluster of 379 atoms (112T) (**Figure S11**).

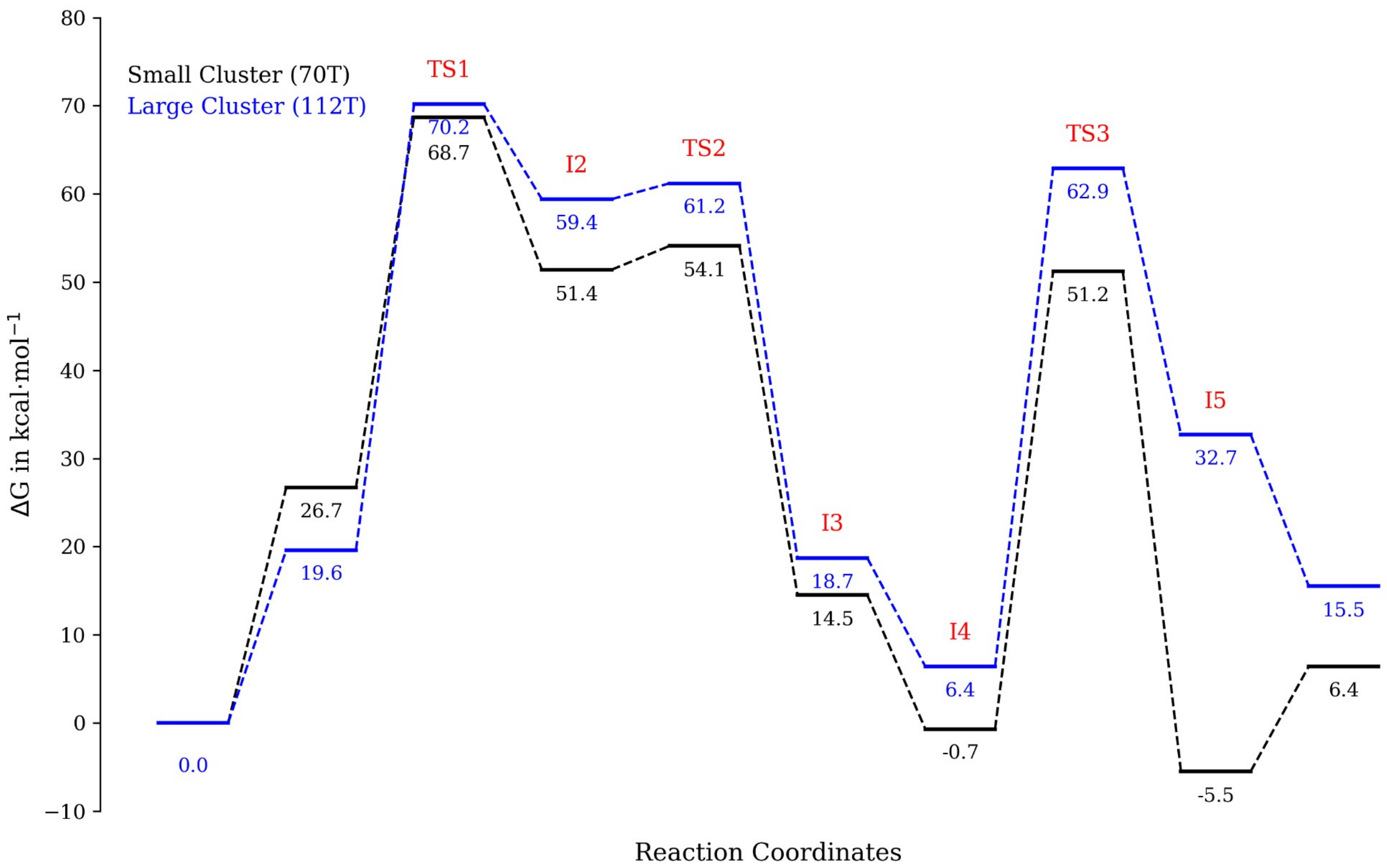
80
70
60
50
40
30
20
10
0
−10
ΔG in kcal·mol⁻¹
Small Cluster (70T)
Large Cluster (112T)
TS1
70.2
68.7
I2
59.4
51.4
TS2
61.2
54.1
26.7
19.6
0.0
I3
18.7
14.5
I4
6.4
-0.7
TS3
62.9
51.2
I5
32.7
-5.5
15.5
6.4
Reaction Coordinates

(a)

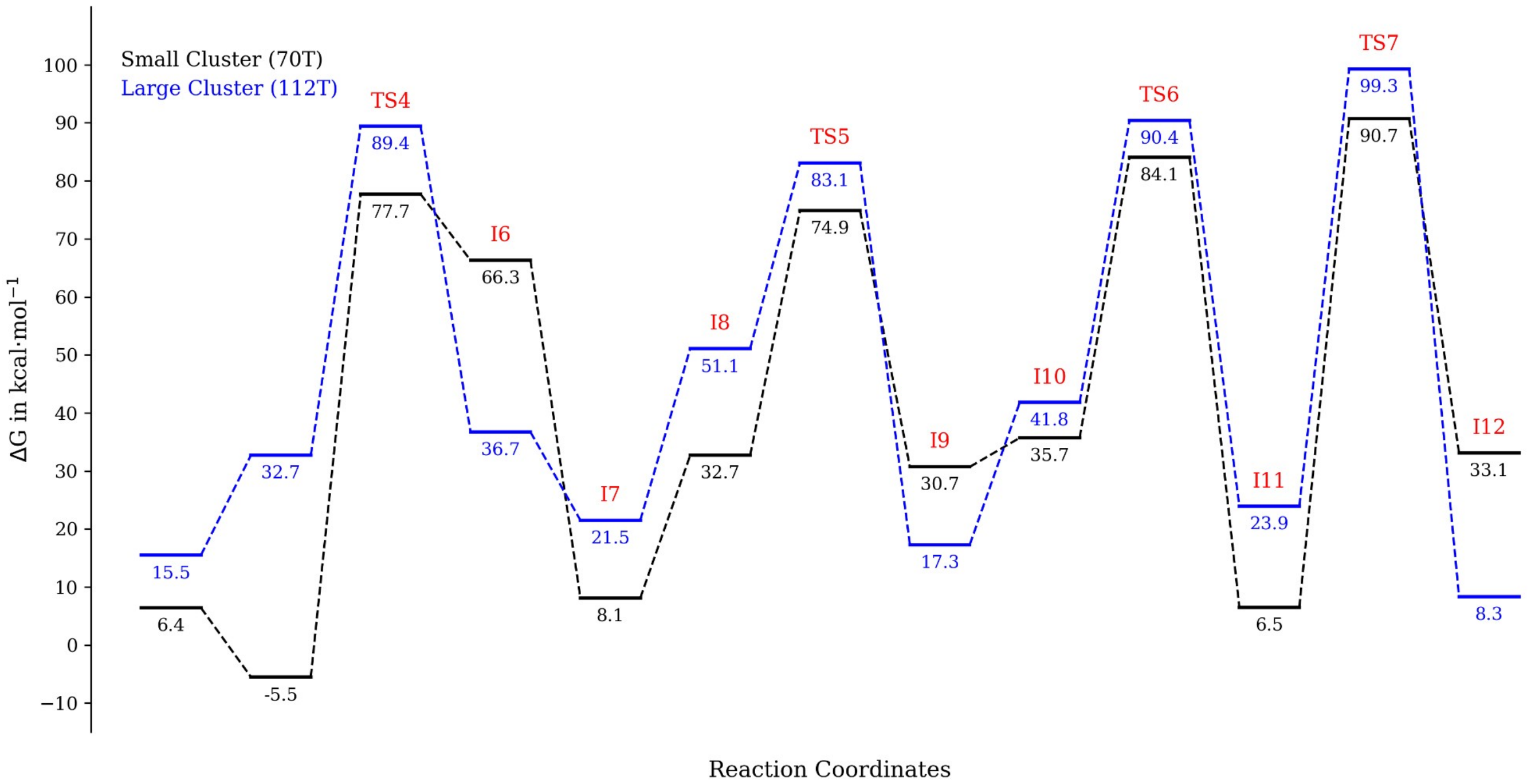

(b)

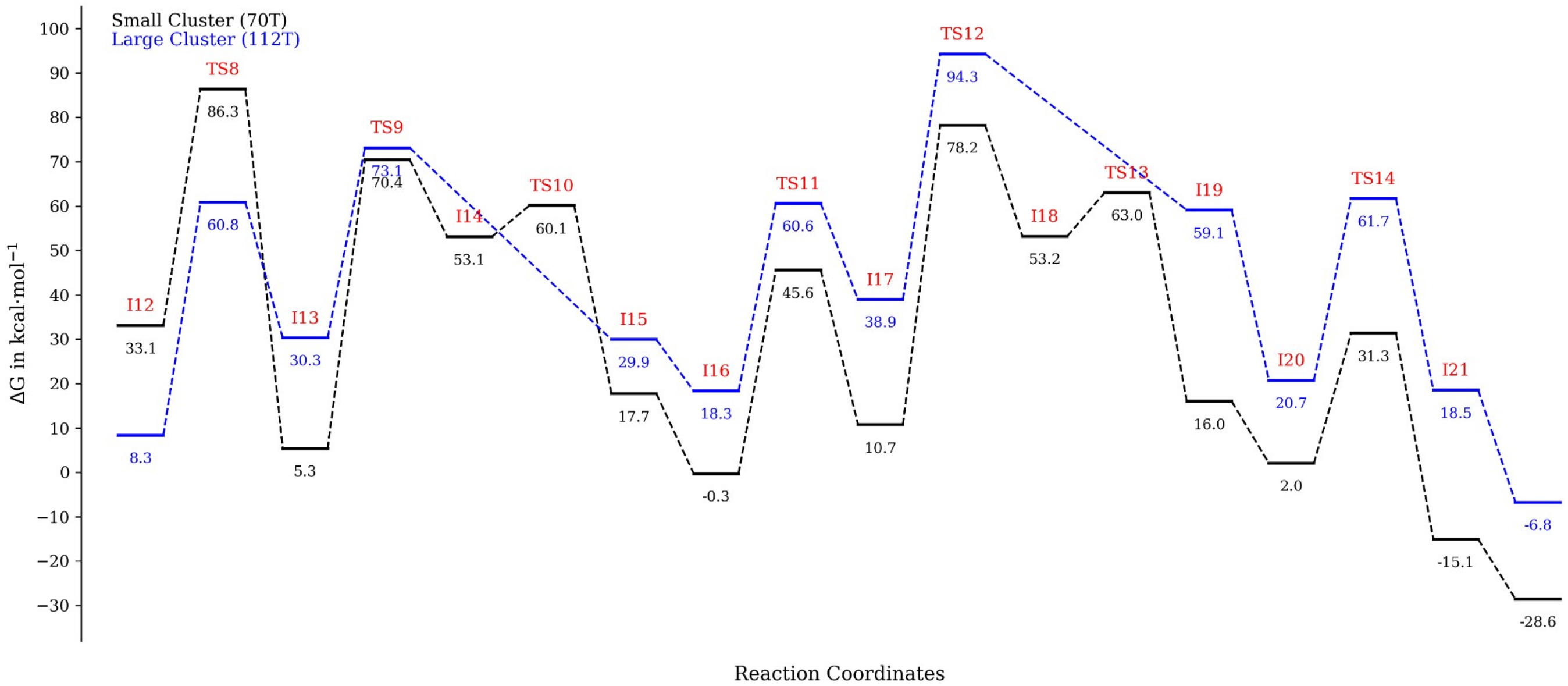

(c)

Figure S11. Comparison of the Gibbs free-energy profiles for ETA conversion over the [GaH_2]-ZSM-5 catalyst, following the stepwise mechanism, for the small (70T) and large (112T) zeolite clusters. (a) Ethane dehydrogenation stage, (b) ethene cyclization stage, and (c) aromatization stage. Calculations were performed at the *ω*B97X-D4rev/def2-mTZVPP//r^{2}SCAN-3c level for the small cluster and at the *ω*B97X-D4rev/def2-mTZVPP//r^{2}SCAN-D4/def2-SVP level for the large one. The BSSE correction was applied.

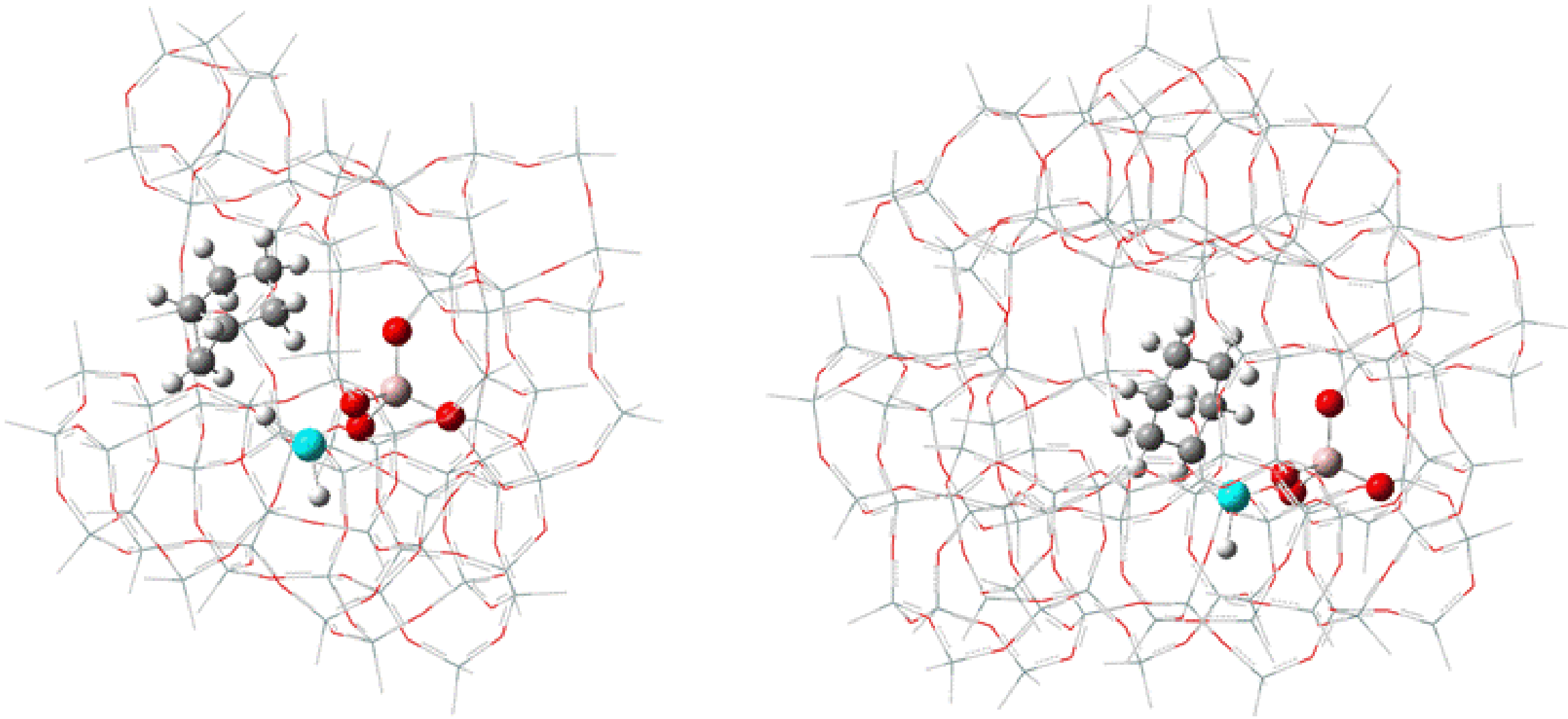

(a) (b)

Figure S12. Structure of the I12 intermediate in ethane-to-aromatic conversion over the $[GaH_2]$–ZSM-5 catalyst, according to the stepwise mechanism. (a) Small cluster with 239 atoms and (b) large cluster with 379 atoms. Si atoms are depicted in light gray, O atoms in red, Al atom in pink, Ga atom in blue, C atoms in dark gray, and H atoms in white.

References

[1] Frisch, M. J.; Trucks, G. W.; Schlegel, H. B.; Scuseria, G. E.; Robb, M. A. Gaussian 16, Revision C.01, *Gaussian, Inc*, *Wallingford*, *CT* **2016**.

[2] L. Díaz Soto, A. Sierraalta, R. Añez, M. A. C. Nascimento, *The Journal of Physical Chemistry C*, **2015**, DOI:10.1021/jp5122189.

[3] H. Van Koningsveld, *Acta Crystallographica Section B*, **1990**, DOI:10.1107/S0108768190007522.

[4] G. Kresse, J. Furthmüller, *Physical Review B*, **1996**, DOI:10.1103/PhysRevB.54.11169.

[5] A. V. Marenich, S. V. Jerome, C. J. Cramer, D. G. Truhlar, *Journal of Chemical Theory and Computation*, **2012**, DOI:10.1021/ct200866d.

[6] V. Barone, *Journal of Chemical Physics*, **2005**, DOI:10.1063/1.1824881.
