# Supplementary material for: Theoretical Investigation of Ethane‐to‐Aromatics Conversion Over [GaH2 ]‐ZSM‐5
Source: J Comput Chem. 2026 Nov 3;47(29):e70537. doi: 10.1002/jcc.70537 (PMC13730986; doi:10.1002/jcc.70537)
Supplement: Supplementary file 2 [file JCC-47-0-s002.pdf]

Data S2

Theoretical Investigation of Ethane-to-Aromatics Conversion over $[GaH_2]$-ZSM-5

Cartesian coordinates (Å), electronic energies and Gibbs free-energy corrections of all optimized structures. Energies are given in hartree (E_h). G_{corr} was obtained at 673.15 K and 1 atm. Section S2.1 lists the structures computed with the small cluster (70T) and Section S2.2 those computed with the large cluster (112T).

Contents

S2.1 Small cluster (70T) 2

S2.1.1 Gas-phase molecules 2

Ethane 2

Ethene 2

H_2 2

Benzene 3

S2.1.2 $[GaH_2]^+$ site 3

GaH_2-zeolite 3

Complex (GaH_2-zeolite + C_2H_6) 8

I2-GaH 2 12

I3-GaH 2 17

I4-GaH 2 21

I5-GaH 2 26

I6-GaH 2 30

I7-GaH 2 35

I8-GaH 2 39

I9-GaH 2 44

I10-GaH 2 48

I11-GaH 2 53

I12-GaH 2 58

I13-GaH 2 62

I14-GaH 2 67

I15-GaH 2 72

I16-GaH 2 76

I17-GaH 2 81

I18-GaH 2 86

I19-GaH 2 90

I20-GaH 2 95

I21-GaH 2 99

TS1-GaH 2 104

TS1-concerted-GaH 2 109

TS2-GaH 2 113

TS3-GaH 2 118

TS4-GaH 2 122

TS5-GaH 2 127

TS6-GaH 2 131

TS7-GaH 2 136

TS8-GaH 2 141

TS9-GaH 2 145

TS10-GaH 2 150

TS11-GaH 2 155

TS12-GaH 2 159

TS13-GaH 2 164

TS14-GaH 2 169

S2.1.3 Ga^+ site 173

Ga$^+$-zeolite 173
Complex (Ga-modified-zeolite + C_2H_6) 178
TS1-Ga 182
I2-Ga 186
TS2-Ga 191
I3-Ga 195
TS3-Ga 199
I4-Ga 204
TS-Ga-concerted 208
S2.1.4 [GaO]$^+$ site 212
GaO-zeolite 212
Complex (GaO-zeolite + C_2H_6) 217
TS1-GaO-I 221
I2-GaO-I 226
TS2-GaO-I 230
I3-GaO-I 235
I5-GaO-II 239
TS1-GaO-I-concerted 244
I2-GaO-II 248
TS2-GaO-II 253
I3-GaO-II 257
TS3-GaO-II 262
I4-GaO-II 266
TS1-GaO-II-concerted 271

S2.2 Large cluster (112T) 275

S2.2.1 Gas-phase molecules 275
Ethane 275
Ethene 276
H_2 276
Benzene 276
S2.2.2 [GaH_2]$^+$ site 276
GaH_2-zeolite 277
Complex (GaH_2-zeolite + C_2H_6) 283
TS1-GaH_2 290
I2-GaH_2 297
TS2-GaH_2 304
I3-GaH_2 311
I4-GaH_2 318
TS3-GaH_2 325
I5-GaH_2 332
TS4-GaH_2 339
I6-GaH_2 346
I7-GaH_2 353
I8-GaH_2 360
TS5-GaH_2 367
I9-GaH_2 374
I10-GaH_2 381
TS6-GaH_2 388
I11-GaH_2 395
TS7-GaH_2 402
I12-GaH_2 409
TS8-GaH_2 416
I13-GaH_2 423
TS9-GaH_2 430
I15-GaH_2 437
I16-GaH_2 444
TS11-GaH_2 451

I17-GaH_2 458
TS12-GaH_2 465
I19-GaH_2 472
I20-GaH_2 479
TS14-GaH_2 486
I21-GaH_2 493

S2.1 Small cluster (70T)

Geometries optimized at the r^{2}SCAN-3c level.
Single-point energies at the ωB97X-D4rev/def2-mTZVPP level.

S2.1.1 Gas-phase molecules

Ethane

Electronic energy (r^{2}SCAN-3c): -79.78889 E_h
Electronic energy (ωB97X-D4rev/def2-mTZVPP): -79.902354414266 E_h
G_{corr} (673.15 K, 1 atm): 0.08934 E_h
Number of atoms: 8

Cartesian coordinates (Å)

```
C        -1.94568694044660       0.86319300219625       0.00527125103060
H        -1.59827043550557      -0.17455030690554      -0.02459359945152
H        -1.59668743907891       1.35509563343217      -0.90840466339387
H        -3.03991071911424       0.84651741246476      -0.02558729966967
C        -1.43664218969975       1.58307030207519       1.25213563840777
H        -1.78401533337713       2.62082951635206       1.28196795937417
H        -1.78568558943717       1.09120399517196       2.16581383804219
H        -0.34241848334062       1.59970783521314       1.28301637566032
```

Ethene

Electronic energy (r^{2}SCAN-3c): -78.557039654294 E_h
Electronic energy (ωB97X-D4rev/def2-mTZVPP): -78.653702325267 E_h
G_{corr} (673.15 K, 1 atm): -0.00474 E_h
Number of atoms: 6

Cartesian coordinates (Å)

```
C         2.86297591653516      -1.29071384615554       1.20115434886997
C         2.86297591707643      -1.29071384558865       2.52797758734766
H         2.86297591715716      -0.36530771494255       0.63158556221361
H         2.86297591718264      -2.21611997761045       0.63158556232468
H         2.86297591703704      -0.36530771447425       3.09754637444803
H         2.86297591701155      -2.21611997722856       3.09754637279605
```

H_2

Electronic energy (r^{2}SCAN-3c): -1.169381617821 E_h
Electronic energy (ωB97X-D4rev/def2-mTZVPP): -1.179493792555 E_h
G_{corr} (673.15 K, 1 atm): -0.02818 E_h
Number of atoms: 2

Cartesian coordinates (Å)

```
H         0.38594645091737       0.01054852000000       0.00000000000000
H        -0.35556671091737       0.01054852000000       0.00000000000000
```

Benzene

Electronic energy (r^{2}SCAN-3c): -232.183141061357 E_h
Electronic energy (ωB97X-D4rev/def2-mTZVPP): -232.428138775959 E_h
G_{corr} (673.15 K, 1 atm): 0.02557 E_h
Number of atoms: 12

Cartesian coordinates (Å)

C	5.51104605200072	1.20858712450487	3.05064921785708
C	4.35834495427040	0.85485415100814	3.74679804717909
C	6.66374714861216	1.56232009905333	3.74679804993428
H	3.45887600622326	0.57900892647530	3.20363762777363
H	7.56321609940649	1.83816531282947	3.20363762989789
C	4.35834495464530	0.85485415728768	5.13911156619719
C	6.66374715125151	1.56232009474768	5.13911156909718
H	3.45887601050759	0.57900894134622	5.68227198509081
H	7.56321610396015	1.83816530389860	5.68227198760935
C	5.51104605402279	1.20858712307596	5.83526039864910
H	5.51104605558637	1.20858711951904	6.92161435267366
H	5.51104605551325	1.20858712025372	1.96429526404071

S2.1.2 $[GaH_2]^+$ site

GaH_2-zeolite

Electronic energy (r^{2}SCAN-3c): -30538.756617153907 E_h
Electronic energy (ωB97X-D4rev/def2-mTZVPP): -30546.841205221226 E_h
G_{corr} (673.15 K, 1 atm): 0.38033836 E_h
Number of atoms: 242

Cartesian coordinates (Å)

O	1.48847600000000	2.89786700000000	1.02859400000000
O	-1.94476400000000	6.77180200000000	1.42640800000000
O	5.26858900000000	2.07912500000000	0.80309200000000
O	3.60743700000000	3.88261600000000	-0.15451500000000
O	6.09386400000000	4.57702700000000	0.41974400000000
O	1.45287400000000	3.28859400000000	-1.61257000000000
O	1.37115700000000	5.32809200000000	0.03056300000000
O	-1.71131400000000	6.88911300000000	-1.19167800000000
O	0.32405900000000	7.70197600000000	0.40664800000000
O	2.93351100000000	7.47946300000000	-0.04657400000000
Si	4.84693600000000	3.63993400000000	0.87013100000000
Si	1.98901300000000	3.84633500000000	-0.18693500000000
Si	-1.29432500000000	7.62914600000000	0.20236700000000
Si	1.64936800000000	6.79443700000000	0.66076000000000
O	8.95225100000000	-3.83480500000000	2.22544800000000
O	1.99811300000000	6.65348400000000	2.24415500000000
O	7.14526400000000	-2.28026300000000	-0.92348800000000
O	2.50530400000000	4.75023100000000	4.08410000000000
O	6.74870800000000	-4.90473300000000	-0.77221700000000
O	0.04657900000000	5.11719300000000	3.14235100000000
O	5.85497600000000	-3.26741100000000	1.13664400000000
O	4.44160100000000	4.07284500000000	2.38774600000000
O	9.40133000000000	-5.01130300000000	-1.12818300000000
O	5.15383600000000	-0.24718200000000	2.01912100000000
O	7.73381500000000	4.37790000000000	-1.68849300000000
O	10.70336700000000	-3.18509400000000	0.34587200000000

O	5.43003800000000	5.63179300000000	-1.91088000000000
O	4.09173500000000	5.67311800000000	-4.17473800000000
O	8.76180100000000	-1.46092600000000	1.04438200000000
O	3.92197900000000	7.73622200000000	-2.52986000000000
O	1.17165200000000	9.52025300000000	-4.04603800000000
O	8.98102100000000	2.07174500000000	-1.88684300000000
O	6.94727400000000	0.18962600000000	0.10316800000000
O	2.27284400000000	9.55388700000000	-1.56413300000000
O	8.99970800000000	-0.54768500000000	-1.44277900000000
Si	1.43287500000000	5.86176200000000	3.55308700000000
Si	6.11614800000000	-3.44043600000000	-0.44905100000000
Si	3.63026800000000	3.62377000000000	3.72738400000000
Si	8.07923400000000	-5.83640700000000	-0.65664500000000
Si	10.87644100000000	-4.45890300000000	-0.67196300000000
Si	6.67130100000000	5.32652900000000	-0.90803800000000
Si	9.79486900000000	-2.60859000000000	1.56639400000000
Si	4.92399000000000	6.59432400000000	-3.11872000000000
Si	9.20519800000000	3.67313400000000	-1.66750200000000
Si	6.17150900000000	0.85325800000000	1.37340600000000
Si	1.72972500000000	10.45280500000000	-2.82526600000000
Si	9.80041400000000	0.68290000000000	-2.15349900000000
Si	7.95505100000000	-1.01651000000000	-0.30249100000000
Si	3.46910600000000	8.51798400000000	-1.17831400000000
O	2.97784600000000	-0.47030900000000	3.61669400000000
O	0.96116500000000	0.76758900000000	2.47632100000000
O	1.24108300000000	0.88681600000000	5.08258300000000
O	2.96804400000000	2.16187600000000	3.48372300000000
O	-0.40946200000000	1.19090900000000	0.28188300000000
O	-0.82315800000000	2.73230300000000	2.35798100000000
O	-3.75453600000000	4.98307300000000	0.57216100000000
O	-2.53438600000000	4.66048600000000	2.92999000000000
O	-1.17616300000000	3.43228300000000	4.84019100000000
Si	2.04462500000000	0.84181500000000	3.67984000000000
Si	0.30816200000000	1.91008100000000	1.53682500000000
Si	-3.13355900000000	5.74588800000000	1.86715300000000
Si	-1.10867400000000	3.99715000000000	3.32995100000000
O	8.28941600000000	-6.04346000000000	3.49638000000000
O	-3.19963500000000	-3.06746000000000	6.58299000000000
O	6.68168200000000	-7.84704900000000	2.44442200000000
O	-1.31185900000000	-1.56701500000000	7.69316400000000
O	8.25716100000000	-6.34256200000000	0.87199900000000
O	-5.42808100000000	-3.48495400000000	5.24754000000000
O	6.65217700000000	-3.92538700000000	3.57202600000000
O	-5.13563500000000	-1.21964800000000	6.59000000000000
O	-8.67415000000000	0.34309700000000	5.57746300000000
O	4.47648300000000	-2.51251800000000	3.25138000000000
O	-6.44693900000000	1.04781700000000	6.95196200000000
O	4.49237900000000	-5.06553500000000	2.60494800000000
O	-3.88655100000000	0.85865400000000	7.58039300000000
O	-2.25415100000000	2.85033500000000	7.14694100000000
O	0.39928300000000	2.65945900000000	6.86527100000000
O	4.25483800000000	-7.39973900000000	1.37498300000000
O	-1.50903500000000	0.86676600000000	8.78632300000000
O	0.72039100000000	0.08454600000000	7.53291100000000
Si	8.12653200000000	-7.12399500000000	2.28705600000000
Si	-2.12779800000000	-2.97404200000000	7.81769500000000
Si	8.20026400000000	-4.41713100000000	3.55234600000000
Si	-4.80606800000000	-2.81414700000000	6.58675900000000

Si	4.52738100000000	-0.89517700000000	3.37812100000000
Si	-8.08251600000000	1.12887500000000	6.87963100000000
Si	5.36075000000000	-3.70499700000000	2.61452900000000
Si	-5.30022700000000	0.06963300000000	7.56787500000000
Si	-0.93671600000000	3.48802900000000	6.44383800000000
Si	3.44313100000000	-6.09961400000000	1.92560700000000
Si	-2.74962800000000	1.78655900000000	8.27082500000000
Si	1.29614700000000	1.31852200000000	6.64906200000000
Si	5.41663200000000	-8.50874400000000	1.64881500000000
Si	-0.51659300000000	-0.38634300000000	8.47556600000000
O	1.80061400000000	4.26937200000000	-4.05903600000000
O	-0.06775000000000	5.26979700000000	-2.46354300000000
O	-0.61770700000000	7.68258100000000	-3.46442500000000
Si	0.70238200000000	3.91745600000000	-2.91481000000000
Si	-1.20387000000000	6.40595300000000	-2.65457100000000
O	1.24756900000000	-1.73182000000000	-4.49807400000000
O	-3.98449600000000	3.94370600000000	-4.54880500000000
O	3.34282300000000	-2.95946700000000	-3.48316200000000
O	-2.44830500000000	5.81656400000000	-3.50492900000000
O	-3.82331400000000	4.16745500000000	-1.91794600000000
O	1.33507800000000	-4.41198500000000	-4.41613900000000
O	-4.66788200000000	1.77326800000000	-5.91807600000000
O	4.72073700000000	-3.30734500000000	-1.25766400000000
O	-2.55797100000000	1.70712800000000	-4.37872100000000
O	-3.17027400000000	-0.36761200000000	-6.53547200000000
O	-1.11005300000000	-1.79615300000000	-5.66030800000000
O	-0.33068400000000	0.32759400000000	-4.21071300000000
O	-0.36992500000000	2.85369700000000	-3.48536700000000
O	1.78061000000000	6.92595000000000	-4.27161100000000
Si	-3.84090100000000	4.99987700000000	-3.31570100000000
Si	2.19336500000000	-3.04485100000000	-4.63927600000000
Si	-4.05193900000000	2.31883000000000	-4.52838900000000
Si	4.00902600000000	-4.07682900000000	-2.50717400000000
Si	-4.32481700000000	0.66641900000000	-7.07058300000000
Si	-0.30442300000000	-1.29335800000000	-4.34578200000000
Si	-1.22278200000000	1.47667500000000	-3.50252300000000
Si	2.55434900000000	5.56496100000000	-4.71058000000000
Si	0.62550600000000	8.05122100000000	-4.45445800000000
O	-2.79740200000000	-5.59308200000000	-7.11437500000000
O	-4.52782700000000	-4.33459500000000	-5.54204500000000
O	-2.91440300000000	-2.92991600000000	-7.21987200000000
O	-2.83269200000000	0.12603100000000	0.17486800000000
O	-2.48910800000000	2.76366200000000	-0.14645900000000
O	-1.66455200000000	1.00694100000000	-2.00827000000000
O	-5.13117600000000	2.80018600000000	-0.00860800000000
Si	-3.76695600000000	-4.29563100000000	-6.97838500000000
Si	-1.85558400000000	1.29596400000000	-0.43234600000000
Si	-3.81793700000000	3.67790600000000	-0.38022000000000
O	-5.01445500000000	1.90405200000000	-3.29043900000000
O	2.81596700000000	-5.05214400000000	-1.98283600000000
O	-5.60863700000000	-2.50275700000000	2.82577500000000
O	-7.38695300000000	-1.79062400000000	4.67515400000000
O	-0.25157800000000	-6.30671800000000	-3.40412400000000
O	-8.00996400000000	0.18746400000000	3.00719100000000
O	-0.39103100000000	-5.57326600000000	-5.96327900000000
O	-0.96585500000000	-1.92352900000000	-3.01400700000000
O	-3.48628600000000	-6.50734200000000	0.73139200000000
O	-0.97086400000000	-4.27050200000000	-1.86850500000000

O	-6.60092500000000	1.03683900000000	-1.38690300000000
O	0.62667000000000	-6.08365700000000	-0.87379100000000
O	-8.35434600000000	-0.62812900000000	0.48600000000000
O	-7.36274300000000	1.78037600000000	1.02252100000000
O	-1.87371800000000	-6.74893700000000	-1.40576800000000
O	-6.54022200000000	-0.21170100000000	-3.70745400000000
O	2.70817600000000	-5.41952200000000	0.64025400000000
O	-9.07351300000000	-2.65892700000000	-1.03840600000000
O	-4.46985100000000	-0.39295800000000	-2.08184600000000
O	-7.01697000000000	-2.91748800000000	0.60985600000000
O	-8.03897600000000	-2.38350900000000	-3.48093500000000
O	-5.38349100000000	-2.61092000000000	-3.74223500000000
O	-7.26520900000000	-4.36783000000000	-1.88516500000000
O	-5.00782300000000	-5.15248500000000	-3.07564700000000
O	-2.89847000000000	-3.62960600000000	-3.61249800000000
O	-5.69547300000000	-6.15512500000000	-0.71864400000000
O	-2.78387300000000	-2.52332900000000	-1.18081600000000
O	-4.76715400000000	-4.21496300000000	0.91457300000000
O	-4.67825000000000	-1.54222500000000	0.67081700000000
Si	-6.47858800000000	-3.01377100000000	4.10879400000000
Si	0.60023400000000	-5.83277100000000	-4.70722500000000
Si	-8.49708900000000	-0.65338800000000	4.31623700000000
Si	-1.24382900000000	-6.06967900000000	-7.25972400000000
Si	-6.64418900000000	2.28818200000000	-0.35359600000000
Si	-2.43766000000000	-7.48863900000000	-0.05859200000000
Si	-0.61948200000000	-5.84198100000000	-1.89189800000000
Si	-8.42968400000000	0.64643500000000	1.49788900000000
Si	-5.63183900000000	0.56204300000000	-2.60622900000000
Si	2.24988800000000	-6.02046900000000	-0.81035100000000
Si	-8.49003900000000	-2.24220400000000	0.41685800000000
Si	-6.70639900000000	-1.75590900000000	-4.17562500000000
Si	-8.52051300000000	-3.44614700000000	-2.35538600000000
Si	-4.44867100000000	-3.92051200000000	-3.97900700000000
Si	-6.26058300000000	-5.61911600000000	-2.14624600000000
Si	-1.93118500000000	-3.08483000000000	-2.40807000000000
Si	-4.95856400000000	-5.82927800000000	0.69835700000000
Al	-3.69136900000000	-1.14527300000000	-0.77473100000000
Si	-5.52817300000000	-2.82487500000000	1.23406100000000
Si	-2.11224800000000	-1.55013000000000	-6.91107200000000
H	11.67374700000000	-5.53703600000000	-0.03807400000000
H	9.19348300000000	-8.10585800000000	2.36346300000000
H	-3.88155000000000	1.39981100000000	-8.25725800000000
H	-5.54661900000000	-0.07985100000000	-7.37196300000000
H	-6.93206200000000	-1.73335100000000	-5.60666700000000
H	-9.61083500000000	-4.23371600000000	-2.91527900000000
H	-4.80806200000000	-4.38616400000000	-7.98466800000000
H	-1.25558100000000	-7.52827100000000	-7.35483800000000
H	-8.46603500000000	2.53218800000000	6.77163500000000
H	-4.20950400000000	6.50518800000000	2.49497300000000
H	-1.81143200000000	9.00591900000000	0.20164700000000
H	0.67612800000000	11.32783200000000	-2.29765500000000
H	-4.95337000000000	5.93157200000000	-3.34598500000000
H	-7.43212800000000	3.36467300000000	-0.94325500000000
H	-9.81433500000000	1.14100200000000	1.45030700000000
H	-3.26082300000000	2.50672900000000	9.43326900000000
H	-0.74504100000000	4.85495100000000	6.90563300000000
H	1.17176500000000	6.84606700000000	4.60638300000000
H	0.01383700000000	-0.85268900000000	9.75320600000000

H	2.65630400000000	1.61378700000000	7.09828800000000
H	4.53076900000000	3.47379800000000	4.86924400000000
H	1.53720800000000	-6.93798300000000	-4.97365700000000
H	4.63541500000000	9.21535600000000	-0.64625200000000
H	-8.67488800000000	0.53574600000000	8.08886500000000
H	-5.67875100000000	-0.35844200000000	8.92691800000000
H	-5.40516100000000	-3.45826600000000	7.74597000000000
H	-2.86007300000000	-3.03986700000000	9.10083200000000
H	-1.22701000000000	-4.11162700000000	7.70400900000000
H	2.57413300000000	5.42807900000000	-6.16363200000000
H	0.22482900000000	8.10275900000000	-5.85603200000000
H	2.79507600000000	11.25689300000000	-3.42192600000000
H	6.06067100000000	7.26917500000000	-3.76691100000000
H	10.01065000000000	4.23779400000000	-2.76113100000000
H	9.87926500000000	0.42371400000000	-3.59460900000000
H	11.16639500000000	0.78744100000000	-1.60472500000000
H	9.89847200000000	3.90341200000000	-0.38688400000000
H	-1.32399900000000	-1.19594400000000	-8.10330100000000
H	-0.61662900000000	-5.49814400000000	-8.46011600000000
H	2.84818300000000	-3.04332200000000	-5.95038400000000
H	-7.31517900000000	-4.15812800000000	3.75060700000000
H	-9.78250000000000	-1.30915800000000	4.07997000000000
H	-9.40319700000000	-2.75383200000000	1.45013700000000
H	-5.76898900000000	-6.41021300000000	1.77986100000000
H	-3.08084500000000	-8.75083000000000	-0.46797100000000
H	-7.02180600000000	-6.69287800000000	-2.78419800000000
H	2.78359000000000	-7.36886300000000	-0.99247300000000
H	4.89691600000000	-9.57738000000000	2.49409400000000
H	5.82084500000000	-9.04713700000000	0.34374500000000
H	-1.34437700000000	-7.77605500000000	0.87467400000000
H	10.66950900000000	-2.02029700000000	2.56380700000000
H	8.82314100000000	-3.95574100000000	4.78387700000000
H	11.59080000000000	-3.97453400000000	-1.85888000000000
H	5.33815600000000	-0.46451100000000	4.51995500000000
H	7.15219500000000	1.34004700000000	2.37234700000000
H	7.32251200000000	6.57841100000000	-0.51330700000000
H	5.00684300000000	-4.86484800000000	-3.24385800000000
H	7.90214800000000	-7.00114300000000	-1.50659200000000
H	2.44258300000000	-6.49974700000000	2.91143200000000
Ga	-3.70196200000000	-0.28608200000000	1.96152400000000
H	-2.67544200000000	-1.13700800000000	2.73796100000000
H	-4.66894900000000	0.86655400000000	2.29221400000000

Complex (GaH_2-zeolite + C_2H_6)

Electronic energy (r^2SCAN-3c): -30618.555027392587 E_h
Electronic energy (ωB97X-D4rev/def2-mTZVPP): -30626.742898404194 E_h
G_{corr} (673.15 K, 1 atm): 0.44308 E_h
Number of atoms: 250

Cartesian coordinates (Å)

O	1.50623433044980	2.87100217243284	0.99297638218292
O	-1.95034459825225	6.77860109038583	1.42681150078488
O	5.25860294551271	2.07497791513364	0.81660007604677
O	3.61742298176794	3.88793293339005	-0.16349238837969
O	6.10799149189505	4.56363866431964	0.41081700051025
O	1.46061178358041	3.29274286905946	-1.63148461195023
O	1.37377330787915	5.31993144073131	0.03608029842311
O	-1.70350674793245	6.88924775414176	-1.18843314560350
O	0.32762191203454	7.69694992436002	0.41478462512644
O	2.93459384059992	7.47202275242524	-0.05820580634863
Si	4.85250317728656	3.64165520251624	0.86653338452116
Si	1.99864532193938	3.84360268485557	-0.20402306470333
Si	-1.29067356439701	7.63056777224391	0.20516562516581
Si	1.65513987531273	6.78961726121075	0.65934710572880
O	8.95501732600233	-3.83547985597809	2.22527914684491
O	2.01815668555168	6.65758852924264	2.24144070319656
O	7.13821618773086	-2.27166474879639	-0.92211922152987
O	2.49549656141312	4.73585416348191	4.07027073405428
O	6.75197459509801	-4.90027391620250	-0.78202135704317
O	0.03888939650528	5.16291160281398	3.13629929253369
O	5.85884440678890	-3.27777101251371	1.13613166737337
O	4.45473304589138	4.09767067868242	2.38100929128412
O	9.40301116045598	-5.01264161361021	-1.12778477529926
O	5.18644928198009	-0.27190727716259	2.00871619068323
O	7.73778066144869	4.38315608856030	-1.70020651359281
O	10.70324287707284	-3.18353697208178	0.34469922528694
O	5.42951239743669	5.63536767970074	-1.90911257724866
O	4.09036725732096	5.67032249801490	-4.17165011751123
O	8.75964875934362	-1.46130440316420	1.04364937218195
O	3.92536100776467	7.74196906146466	-2.53591950076067
O	1.17377031052565	9.52336726658934	-4.04952125267277
O	8.98091238101856	2.07080156508570	-1.88554244275476
O	6.96161980051536	0.20690436033048	0.09416553262482
O	2.27233235299995	9.55358065723025	-1.56524025904438
O	9.00513167478925	-0.55218802067010	-1.44539300512106
Si	1.44149207966287	5.87115008611073	3.54818431674667
Si	6.11592548067768	-3.43923383197003	-0.45123850733244
Si	3.64448883140053	3.63161498067960	3.71542769798105
Si	8.07901820678039	-5.83541343835626	-0.65823175032425
Si	10.87717226549245	-4.45845747522032	-0.67174394544556
Si	6.67535276196007	5.32621292731487	-0.91340288427971
Si	9.79474706591637	-2.60765767010242	1.56559931329987
Si	4.92405813452584	6.59489387919436	-3.11976671350146
Si	9.20626501490620	3.67264048600284	-1.66881321805806
Si	6.17928047407322	0.85356328790094	1.36918141307834
Si	1.72972441781353	10.45350222150486	-2.82609394566371
Si	9.80136159411357	0.68260665456237	-2.15400847631740
Si	7.95716824617169	-1.01234878234138	-0.30473943019253
Si	3.47008525758606	8.51831577464036	-1.18238458539002
O	2.99292882745966	-0.47669135390720	3.57037404135543

O	0.97029037621035	0.79238865098070	2.48863917622727
O	1.32697588154327	0.89016531707628	5.08428246811092
O	3.01655806625408	2.15922903352194	3.44561794777146
O	-0.43225918578665	1.15503555526480	0.32096518843128
O	-0.79345862555886	2.79013396657329	2.32770768902018
O	-3.73242869154706	4.96894423592472	0.56436754748754
O	-2.53806851188262	4.67260903291265	2.93577641325684
O	-1.16101929280307	3.45881097768408	4.82910279769539
Si	2.08562173877444	0.84988392422307	3.65966091367005
Si	0.31383087706313	1.91684024020346	1.53102466461125
Si	-3.13560285551959	5.74698821243526	1.86153273624758
Si	-1.10084855587225	4.02913523391920	3.31943433864683
O	8.30004130470832	-6.04754246820807	3.49694898445160
O	-3.20460358618002	-3.06249924688181	6.58822310720301
O	6.68022053693391	-7.84471169468641	2.44513503262033
O	-1.30402978797832	-1.57734212792550	7.69935975097651
O	8.25049797591910	-6.33921904531135	0.87226254375104
O	-5.40586264096054	-3.45431394773214	5.21579538974304
O	6.65612994589099	-3.93917797288209	3.57147833600032
O	-5.15865367612403	-1.22097017350252	6.61344296514268
O	-8.67814231236486	0.33093850269032	5.58385930020628
O	4.48886475915388	-2.52233005333116	3.25649515457301
O	-6.45317707897754	1.05459168498034	6.94869518896276
O	4.49345462900565	-5.07283217737082	2.60467922352011
O	-3.91703908266028	0.87706244814146	7.63113001497893
O	-2.25632280327528	2.83693026298832	7.12650004464406
O	0.39422405570072	2.64843194468723	6.84517692238688
O	4.25197136774411	-7.40357909004578	1.37245690080916
O	-1.50783617473115	0.86472299798192	8.77332538664700
O	0.71495218293926	0.07758163283645	7.51323317843709
Si	8.12658621448209	-7.12453982295353	2.28603506402739
Si	-2.13053967072122	-2.97916510870461	7.82030357100282
Si	8.20614400234198	-4.42171286201313	3.55194765860374
Si	-4.81338863723489	-2.81046799979099	6.58224370542730
Si	4.54525802608131	-0.90496697171783	3.36710718950549
Si	-8.08857688202046	1.12832031245842	6.87831825400406
Si	5.36642885045522	-3.71600383537247	2.61384060744227
Si	-5.31787822558443	0.07533336523491	7.57870389784485
Si	-0.93748050177426	3.48965480631540	6.43522028811180
Si	3.44187401412150	-6.10288650215044	1.92360481214293
Si	-2.74923715710376	1.79222952828034	8.27265254904556
Si	1.30802224111355	1.31665161909616	6.64685836797919
Si	5.41652764975950	-8.50903371533372	1.64871143543594
Si	-0.51247263410932	-0.38763161297915	8.47185299282474
O	1.79366574846266	4.27069194068149	-4.07387846266583
O	-0.07019467132416	5.26694379537504	-2.46904117185846
O	-0.61147337254153	7.68662524029753	-3.46014258079390
Si	0.69766528416833	3.91608741557416	-2.92919333155124
Si	-1.20087066156557	6.40968878039588	-2.65341335124327
O	1.24347770579926	-1.73059524601968	-4.49248989113539
O	-3.96957508864582	3.96119675697072	-4.56142409830788
O	3.34233140565417	-2.95804825141724	-3.48362819352338
O	-2.45094289592026	5.83145233614229	-3.50122925328976
O	-3.80786147859638	4.15366470948467	-1.92446615442428
O	1.33504168030701	-4.41086782273487	-4.41510340976017
O	-4.65867914659825	1.78858007250296	-5.93423652780703
O	4.71915482057682	-3.30691537362270	-1.25742770405657
O	-2.59094918637566	1.70181669051304	-4.33704794479910

O	-3.16625123765095	-0.36364661362150	-6.53250138146812
O	-1.10520919961935	-1.80922877780602	-5.67172438989169
O	-0.34970931622745	0.32816280660356	-4.24007340415073
O	-0.37450489344080	2.85383913668238	-3.50406708957532
O	1.78413712132534	6.92921032448857	-4.27230479124457
Si	-3.83654236853685	5.00318147860676	-3.31459987411735
Si	2.19130286195986	-3.04243034443076	-4.63879497594957
Si	-4.06805341364493	2.33857890548997	-4.53610814519908
Si	4.00871308634489	-4.07589788501226	-2.50831897522767
Si	-4.32235497583027	0.66690032419354	-7.07272297183864
Si	-0.31151697989954	-1.29433869798347	-4.35504460121363
Si	-1.22678911769170	1.47443386547308	-3.50592236250944
Si	2.55448014995165	5.56692487883405	-4.71297151917805
Si	0.62803599344853	8.05344771723532	-4.45504462685678
O	-2.79659372771527	-5.58879794991192	-7.10193655348960
O	-4.55863146643796	-4.33361517019425	-5.55271214414204
O	-2.92644665630605	-2.92611724577399	-7.21845109138978
O	-2.83309057813528	0.05579622667034	0.12079354747545
O	-2.50317866631809	2.71743700178833	-0.15081976849567
O	-1.60950474872618	0.99162984284118	-2.00409111988395
O	-5.13457794591135	2.80254791387876	-0.00695294758771
Si	-3.77327415876777	-4.29517281538133	-6.97682336217790
Si	-1.85942887591968	1.25200612715331	-0.43317809417029
Si	-3.81045025264996	3.66041040367001	-0.38493837342764
O	-5.09094564054723	1.95609347703784	-3.33491140678048
O	2.81691338862926	-5.05267560710711	-1.98433367177136
O	-5.61882252703645	-2.50644968360858	2.76924402226464
O	-7.37321945460239	-1.78221653158923	4.64716619614138
O	-0.25157941411321	-6.30637400270222	-3.40519237135755
O	-8.01867091619675	0.20859639932648	3.01374097067468
O	-0.38585503613069	-5.57303534336648	-5.96591804136118
O	-0.98307974438472	-1.92296340927775	-3.02638418893303
O	-3.48863966678385	-6.51445656675924	0.73159911270691
O	-0.98252901721941	-4.26800907995179	-1.87827578213267
O	-6.59332289274117	1.03275934286922	-1.38883018992728
O	0.62472691454006	-6.07408753589244	-0.87588542178387
O	-8.33205638468753	-0.64239757407126	0.49874879274757
O	-7.36201837659632	1.77613124495740	1.01767402632932
O	-1.87333034717308	-6.74861387727603	-1.40586633959428
O	-6.56662220529325	-0.20849625718755	-3.71277497312301
O	2.70748948896700	-5.42051028838114	0.63941889789923
O	-9.08889228907687	-2.64407028994840	-1.05412074063690
O	-4.43422866164353	-0.32321304764777	-2.16963137307124
O	-7.06917077359695	-2.99280457290122	0.60345839355967
O	-8.03486567218859	-2.39470364947345	-3.48877360857202
O	-5.37430769998145	-2.60040110383745	-3.73933309487499
O	-7.27110341836792	-4.37052063552156	-1.88127266130510
O	-5.01201844681018	-5.14453744428708	-3.07436698638874
O	-2.89012494932949	-3.65327721063768	-3.66215033507361
O	-5.69846853113332	-6.17201478532282	-0.72540931500871
O	-2.85152140000563	-2.55088398578299	-1.24586054900813
O	-4.77179460806889	-4.22319316178094	0.88300635808711
O	-4.79860116097298	-1.55289819813650	0.53193960399265
Si	-6.46824111380496	-3.00551227614342	4.07453083683601
Si	0.60146779884069	-5.83211726777686	-4.70744309591255
Si	-8.49648117786398	-0.65140151925917	4.31186636840525
Si	-1.24452404153469	-6.06850656719153	-7.25834203124381
Si	-6.64371837868393	2.28595172568660	-0.35689085658307

Si	-2.43675858358687	-7.49001748730407	-0.05954649243165
Si	-0.62253763129275	-5.83838707572192	-1.89468300095062
Si	-8.42828127790466	0.64375496383579	1.49624107721924
Si	-5.64921350952290	0.59381323409364	-2.64083059074011
Si	2.24839203609955	-6.01950314809844	-0.81209975843666
Si	-8.50698063675631	-2.25227449716708	0.40865500153369
Si	-6.70554773537485	-1.75615663792973	-4.17746115642946
Si	-8.52304732671639	-3.44669637899618	-2.35757923774928
Si	-4.45439332106949	-3.92146105092649	-3.99044139260423
Si	-6.26360168066154	-5.61940537373836	-2.14675933421605
Si	-1.94785440790356	-3.09678504078448	-2.44189535552159
Si	-4.96142196339044	-5.83883891826222	0.69003674825430
Al	-3.74208449732128	-1.15505531200750	-0.86209658122340
Si	-5.56641812362831	-2.84686360875102	1.17371223554224
Si	-2.11476080323615	-1.55139930943952	-6.91342528292661
H	11.67374700946798	-5.53703598704364	-0.03807398845150
H	9.19348301009095	-8.10585800793837	2.36346302192766
H	-3.88154999133603	1.39981102636156	-8.25725798751155
H	-5.54661900345942	-0.07985102010424	-7.37196298660016
H	-6.93206202560946	-1.73335099136815	-5.60666714102906
H	-9.61083497560983	-4.23371597678915	-2.91527898478764
H	-4.80806209749057	-4.38616403439681	-7.98466804951661
H	-1.25558100811387	-7.52827101540989	-7.35483797744987
H	-8.46603498416586	2.53218826607896	6.77163489641355
H	-4.20950408025876	6.50518804067986	2.49497300739367
H	-1.81143200866292	9.00591901824625	0.20164699301875
H	0.67612773429065	11.32783163869859	-2.29765475317075
H	-4.95336998212630	5.93157202390638	-3.34598499504045
H	-7.43212788150884	3.36467285291842	-0.94325492603363
H	-9.81433498537998	1.14100200125775	1.45030699488084
H	-3.26082302884572	2.50672904663722	9.43326910496180
H	-0.74504099227742	4.85495103012859	6.90563299225573
H	1.17176500461831	6.84606703728337	4.60638304736981
H	0.01383700826380	-0.85268901819299	9.75320601822807
H	2.65630399303734	1.61378698396621	7.09828800121707
H	4.53076901772899	3.47379798684088	4.86924400758991
H	1.53720802515347	-6.93798301679724	-4.97365699375432
H	4.63541501113655	9.21535601255763	-0.64625199907995
H	-8.67488798082118	0.53574592638795	8.08886504172344
H	-5.67875094318131	-0.35844196224251	8.92691788629056
H	-5.40516103558291	-3.45826608616980	7.74597011921561
H	-2.86007297173671	-3.03986700335542	9.10083193577769
H	-1.22700999611837	-4.11162698554438	7.70400901366844
H	2.57413300369818	5.42807899123940	-6.16363205252594
H	0.22482901054305	8.10275900724166	-5.85603198292216
H	2.79507611398940	11.25689300033608	-3.42192607808859
H	6.06067099498488	7.26917499876560	-3.76691099070104
H	10.01064999412712	4.23779399949649	-2.76113101272151
H	9.87926499831127	0.42371398803849	-3.59460902818271
H	11.16639501362296	0.78744101238380	-1.60472497882134
H	9.89847199787595	3.90341199979117	-0.38688398290773
H	-1.32399895310139	-1.19594397753688	-8.10330101526000
H	-0.61662896173663	-5.49814396022917	-8.46011604323616
H	2.84818302350205	-3.04332201547457	-5.95038404192954
H	-7.31517896184326	-4.15812794103234	3.75060700797062
H	-9.78250002696951	-1.30915800122704	4.07996997742820
H	-9.40319699950572	-2.75383197742688	1.45013701711842
H	-5.76898900260996	-6.41021298881221	1.77986099781742

H	-3.08084499679184	-8.75082999071695	-0.46797097783235
H	-7.02180599420136	-6.69287800858163	-2.78419799513893
H	2.78359000566918	-7.36886302340894	-0.99247300870376
H	4.89691599522502	-9.57738000551007	2.49409402046606
H	5.82084501160223	-9.04713700173841	0.34374497868436
H	-1.34437701976477	-7.77605498595585	0.87467399019825
H	10.66950900054702	-2.02029699252363	2.56380699010336
H	8.82314100235325	-3.95574100233782	4.78387697490943
H	11.59080000886169	-3.97453399481426	-1.85888001049673
H	5.33815598422419	-0.46451098517395	4.51995501009683
H	7.15219498068510	1.34004699125181	2.37234697971112
H	7.32251200948755	6.57841101808623	-0.51330699815619
H	5.00684293988375	-4.86484796114347	-3.24385796486778
H	7.90214800677442	-7.00114298745339	-1.50659200799800
H	2.44258298923779	-6.49974699501510	2.91143199437568
Ga	-3.61751703188812	-0.54610159331620	1.89137257920481
H	-2.71080810448179	-1.71065772422513	2.32899226010893
H	-4.42654005970250	0.64677415719867	2.42704227016298
C	-2.86367399826780	-0.10329378801472	4.91155392862541
H	-3.47122831699773	-0.90723512097142	4.48185542756937
H	-3.48926006345936	0.79061481316469	4.98955498754196
H	-2.60649361546234	-0.42701414660180	5.91870248255267
C	-1.60548539812086	0.16033586932848	4.11540958458682
H	-0.99303743223290	-0.73791510322525	4.00036413091731
H	-1.00058062772986	0.92907250196184	4.59290576253681
H	-1.81336599999537	0.55025692441299	3.10919422207644

I2-GaH 2

Electronic energy (r^2SCAN-3c): -30618.498769064528 E_h
Electronic energy (ωB97X-D4rev/def2-mTZVPP): -30626.690933766589 E_h
G_{corr} (673.15 K, 1 atm): 0.43041 E_h
Number of atoms: 248

Cartesian coordinates (Å)

O	5.26703300000000	2.07807200000000	0.80215600000000
O	3.59974700000000	3.87158200000000	-0.16353100000000
O	6.08423400000000	4.57826100000000	0.41764200000000
O	1.43075200000000	3.28010900000000	-1.61497000000000
O	1.36818400000000	5.32296200000000	0.02817000000000
O	-1.70363500000000	6.88547100000000	-1.18629600000000
O	0.32751500000000	7.70105000000000	0.41135900000000
O	2.93414200000000	7.47377200000000	-0.04997500000000
Si	4.83921200000000	3.63728100000000	0.86497700000000
Si	1.98075100000000	3.83927200000000	-0.19354400000000
Si	-1.29082600000000	7.63054700000000	0.20591100000000
Si	1.65092300000000	6.78920700000000	0.65975600000000
O	8.95290600000000	-3.83524500000000	2.22538900000000
O	2.00207900000000	6.64508100000000	2.24244000000000
O	7.14470900000000	-2.28003000000000	-0.92363700000000
O	2.50145300000000	4.74703900000000	4.08761300000000
O	6.74849300000000	-4.90460400000000	-0.77298500000000
O	0.04263000000000	5.12201700000000	3.14618200000000
O	5.85380400000000	-3.26772800000000	1.13572800000000
O	4.42973700000000	4.07049700000000	2.38199300000000
O	9.40116000000000	-5.01121300000000	-1.12769600000000
O	5.15291100000000	-0.24648000000000	2.01941700000000

O	7.73254800000000	4.37632400000000	-1.68514800000000
O	10.70351600000000	-3.18481100000000	0.34562900000000
O	5.42954300000000	5.63204300000000	-1.91231400000000
O	4.09580200000000	5.67617500000000	-4.17907100000000
O	8.76135600000000	-1.46135800000000	1.04464300000000
O	3.92237800000000	7.73706300000000	-2.53114300000000
O	1.17814000000000	9.52244600000000	-4.05029600000000
O	8.98090600000000	2.07169800000000	-1.88690300000000
O	6.94716600000000	0.18940000000000	0.10337700000000
O	2.27196700000000	9.55230000000000	-1.56410800000000
O	8.99971000000000	-0.54760800000000	-1.44249300000000
Si	1.43233300000000	5.86103800000000	3.55416500000000
Si	6.11539800000000	-3.44062200000000	-0.45018100000000
Si	3.62772200000000	3.62307800000000	3.72774500000000
Si	8.07895100000000	-5.83640300000000	-0.65667800000000
Si	10.87645500000000	-4.45893400000000	-0.67201000000000
Si	6.66933400000000	5.32662600000000	-0.90780200000000
Si	9.79510100000000	-2.60879800000000	1.56632600000000
Si	4.92488200000000	6.59560600000000	-3.11964600000000
Si	9.20495400000000	3.67300200000000	-1.66718700000000
Si	6.17139200000000	0.85329500000000	1.37309400000000
Si	1.73053700000000	10.45306400000000	-2.82527300000000
Si	9.80047200000000	0.68283200000000	-2.15351500000000
Si	7.95508600000000	-1.01694200000000	-0.30246800000000
Si	3.46895000000000	8.51692200000000	-1.17869900000000
O	2.97775400000000	-0.47291000000000	3.61706700000000
O	0.95455800000000	0.76359200000000	2.48852700000000
O	1.24053000000000	0.87963700000000	5.08823900000000
O	2.96436400000000	2.16044700000000	3.48733200000000
O	-0.41930200000000	1.17141800000000	0.30770100000000
O	-0.82126200000000	2.73942200000000	2.36056900000000
O	-3.73761100000000	4.98783700000000	0.56004000000000
O	-2.53710200000000	4.66221300000000	2.92860700000000
O	-1.17940700000000	3.43423900000000	4.84236200000000
Si	2.04545800000000	0.83890800000000	3.68702100000000
Si	0.30662400000000	1.90907600000000	1.54941200000000
Si	-3.13197800000000	5.74872200000000	1.86492000000000
Si	-1.11220800000000	4.00151300000000	3.33384600000000
O	8.28930800000000	-6.04368600000000	3.49652800000000
O	-3.21008200000000	-3.04845000000000	6.59282100000000
O	6.68160000000000	-7.84731300000000	2.44469800000000
O	-1.30632400000000	-1.56838600000000	7.69168700000000
O	8.25586500000000	-6.34191400000000	0.87232400000000
O	-5.41248200000000	-3.47153800000000	5.22560700000000
O	6.65272700000000	-3.92497100000000	3.57120300000000
O	-5.21276800000000	-1.22983700000000	6.62391200000000
O	-8.64068100000000	0.28866000000000	5.61063400000000
O	4.47663300000000	-2.51341500000000	3.25099200000000
O	-6.43660100000000	1.08486300000000	6.97699700000000
O	4.49232600000000	-5.06617700000000	2.60574200000000
O	-3.87367500000000	0.83316500000000	7.57988300000000
O	-2.25735200000000	2.84628700000000	7.14738300000000
O	0.39670300000000	2.65805300000000	6.86486100000000
O	4.25493200000000	-7.39998000000000	1.37489400000000
O	-1.50448700000000	0.86872500000000	8.78823100000000
O	0.72364100000000	0.08349100000000	7.53721000000000
Si	8.12629600000000	-7.12419900000000	2.28704500000000
Si	-2.12980300000000	-2.97138500000000	7.81970900000000

Si	8.20076900000000	-4.41745700000000	3.55218500000000
Si	-4.82036700000000	-2.80636800000000	6.58452100000000
Si	4.52847100000000	-0.89654400000000	3.37799600000000
Si	-8.07350700000000	1.12912800000000	6.88792000000000
Si	5.36121500000000	-3.70616200000000	2.61372100000000
Si	-5.30743800000000	0.07807200000000	7.57819800000000
Si	-0.94060900000000	3.48703900000000	6.44638800000000
Si	3.44322400000000	-6.10001800000000	1.92515100000000
Si	-2.75121800000000	1.77998100000000	8.27079700000000
Si	1.29537100000000	1.31821600000000	6.65350700000000
Si	5.41667800000000	-8.50903900000000	1.64884200000000
Si	-0.51592400000000	-0.38656500000000	8.47719900000000
O	1.81118700000000	4.26974800000000	-4.05435700000000
O	-0.07400900000000	5.26485100000000	-2.47413100000000
O	-0.60821300000000	7.69061000000000	-3.45515100000000
Si	0.69818700000000	3.91466100000000	-2.92683700000000
Si	-1.20094000000000	6.41216200000000	-2.65370800000000
O	1.25294500000000	-1.72051900000000	-4.51571100000000
O	-3.96524200000000	3.96858500000000	-4.57206900000000
O	3.34035000000000	-2.95572700000000	-3.48167400000000
O	-2.45048600000000	5.83958400000000	-3.50624500000000
O	-3.81266400000000	4.16310100000000	-1.93341300000000
O	1.33439800000000	-4.40526900000000	-4.41731100000000
O	-4.65940900000000	1.79493600000000	-5.94360500000000
O	4.72020500000000	-3.30676900000000	-1.25870500000000
O	-2.59134400000000	1.71030200000000	-4.34932800000000
O	-3.16595700000000	-0.36256700000000	-6.53218600000000
O	-1.11297900000000	-1.81743200000000	-5.66899500000000
O	-0.35147900000000	0.32847500000000	-4.25847900000000
O	-0.36741700000000	2.85647800000000	-3.52354000000000
O	1.78163000000000	6.92554000000000	-4.27257100000000
Si	-3.83541900000000	5.00956700000000	-3.32525800000000
Si	2.19379100000000	-3.03952300000000	-4.64158400000000
Si	-4.07098600000000	2.34579200000000	-4.54586100000000
Si	4.00779100000000	-4.07560600000000	-2.50877700000000
Si	-4.32237300000000	0.66659700000000	-7.07490100000000
Si	-0.30387400000000	-1.29607400000000	-4.36427300000000
Si	-1.22307500000000	1.47983800000000	-3.53145200000000
Si	2.55794100000000	5.56634300000000	-4.71141500000000
Si	0.62917600000000	8.05367900000000	-4.45474800000000
O	-2.79512100000000	-5.58785600000000	-7.11061600000000
O	-4.52295900000000	-4.32787500000000	-5.53653900000000
O	-2.92718900000000	-2.92330500000000	-7.23189200000000
O	-2.83822700000000	0.12616200000000	0.11446900000000
O	-2.47798200000000	2.76725800000000	-0.16753000000000
O	-1.59849500000000	1.00606000000000	-2.01914000000000
O	-5.12103100000000	2.80566800000000	-0.00884400000000
Si	-3.76900600000000	-4.29318900000000	-6.97681100000000
Si	-1.82478300000000	1.31793000000000	-0.46538900000000
Si	-3.81311800000000	3.68363200000000	-0.39218200000000
O	-5.09422800000000	1.96544100000000	-3.34746100000000
O	2.81638700000000	-5.05237600000000	-1.98381800000000
O	-5.45696700000000	-2.53688000000000	2.77997200000000
O	-7.33276400000000	-1.76894100000000	4.55113800000000
O	-0.25620700000000	-6.28370300000000	-3.39784200000000
O	-8.04030500000000	0.26950400000000	3.03463000000000
O	-0.38732700000000	-5.57467200000000	-5.96393600000000
O	-0.94284800000000	-1.92551800000000	-3.02370500000000

O	-3.48832200000000	-6.52897400000000	0.74412700000000
O	-0.98296200000000	-4.25438800000000	-1.83978400000000
O	-6.57596100000000	1.03697700000000	-1.39155700000000
O	0.62532700000000	-6.07447300000000	-0.87053100000000
O	-8.28119800000000	-0.66080200000000	0.53667000000000
O	-7.35895000000000	1.78437900000000	1.01236800000000
O	-1.87525800000000	-6.74013500000000	-1.40028200000000
O	-6.54203700000000	-0.21958700000000	-3.69661800000000
O	2.70933300000000	-5.41874400000000	0.64010400000000
O	-9.02644400000000	-2.63490100000000	-1.04066500000000
O	-4.37416800000000	-0.27440300000000	-2.15960000000000
O	-7.12193500000000	-3.08789100000000	0.79579900000000
O	-8.02389900000000	-2.41364500000000	-3.49554400000000
O	-5.36496200000000	-2.60010500000000	-3.73288900000000
O	-7.25475800000000	-4.37717600000000	-1.86394100000000
O	-5.00310900000000	-5.14829300000000	-3.07020400000000
O	-2.88736800000000	-3.62906000000000	-3.60746000000000
O	-5.68854500000000	-6.20822200000000	-0.73291200000000
O	-2.78327400000000	-2.46817200000000	-1.19900000000000
O	-4.76840900000000	-4.24281400000000	0.85010400000000
O	-4.94871600000000	-1.59422900000000	0.39583200000000
Si	-6.42696200000000	-3.01544800000000	4.04600800000000
Si	0.60047400000000	-5.82799000000000	-4.70410900000000
Si	-8.48763700000000	-0.64766700000000	4.30121400000000
Si	-1.24318500000000	-6.06894200000000	-7.25884900000000
Si	-6.63521400000000	2.28753000000000	-0.36035400000000
Si	-2.43615200000000	-7.49184500000000	-0.05820900000000
Si	-0.62543400000000	-5.82484100000000	-1.88309800000000
Si	-8.42175200000000	0.65004200000000	1.49707200000000
Si	-5.62109800000000	0.59619000000000	-2.63574300000000
Si	2.24855500000000	-6.01928600000000	-0.81070400000000
Si	-8.48493900000000	-2.26964800000000	0.44544600000000
Si	-6.69668400000000	-1.76046800000000	-4.17500000000000
Si	-8.50642700000000	-3.45473700000000	-2.35021100000000
Si	-4.43850600000000	-3.91704900000000	-3.97182600000000
Si	-6.25355300000000	-5.63002700000000	-2.14385600000000
Si	-1.92840000000000	-3.06345400000000	-2.40346700000000
Si	-4.96187700000000	-5.85450300000000	0.68413500000000
Al	-3.79786800000000	-1.15193300000000	-0.80996000000000
Si	-5.55622300000000	-2.85700400000000	1.14141200000000
Si	-2.11598900000000	-1.55154800000000	-6.91503600000000
H	11.67374700000000	-5.53703600000000	-0.03807400000000
H	9.19348300000000	-8.10585800000000	2.36346300000000
H	-3.88155000000000	1.39981100000000	-8.25725800000000
H	-5.54661900000000	-0.07985100000000	-7.37196300000000
H	-6.93206200000000	-1.73335100000000	-5.60666700000000
H	-9.61083500000000	-4.23371600000000	-2.91527900000000
H	-4.80806200000000	-4.38616400000000	-7.98466800000000
H	-1.25558100000000	-7.52827100000000	-7.35483800000000
H	-8.46603500000000	2.53218800000000	6.77163500000000
H	-4.20950400000000	6.50518800000000	2.49497300000000
H	-1.81143200000000	9.00591900000000	0.20164700000000
H	0.67612800000000	11.32783200000000	-2.29765500000000
H	-4.95337000000000	5.93157200000000	-3.34598500000000
H	-7.43212800000000	3.36467300000000	-0.94325500000000
H	-9.81433500000000	1.14100200000000	1.45030700000000
H	-3.26082300000000	2.50672900000000	9.43326900000000
H	-0.74504100000000	4.85495100000000	6.90563300000000

H	1.17176500000000	6.84606700000000	4.60638300000000
H	0.01383700000000	-0.85268900000000	9.75320600000000
H	2.65630400000000	1.61378700000000	7.09828800000000
H	4.53076900000000	3.47379800000000	4.86924400000000
H	1.53720800000000	-6.93798300000000	-4.97365700000000
H	4.63541500000000	9.21535600000000	-0.64625200000000
H	-8.67488800000000	0.53574600000000	8.08886500000000
H	-5.67875100000000	-0.35844200000000	8.92691800000000
H	-5.40516100000000	-3.45826600000000	7.74597000000000
H	-2.86007300000000	-3.03986700000000	9.10083200000000
H	-1.22701000000000	-4.11162700000000	7.70400900000000
H	2.57413300000000	5.42807900000000	-6.16363200000000
H	0.22482900000000	8.10275900000000	-5.85603200000000
H	2.79507600000000	11.25689300000000	-3.42192600000000
H	6.06067100000000	7.26917500000000	-3.76691100000000
H	10.01065000000000	4.23779400000000	-2.76113100000000
H	9.87926500000000	0.42371400000000	-3.59460900000000
H	11.16639500000000	0.78744100000000	-1.60472500000000
H	9.89847200000000	3.90341200000000	-0.38688400000000
H	-1.32399900000000	-1.19594400000000	-8.10330100000000
H	-0.61662900000000	-5.49814400000000	-8.46011600000000
H	2.84818300000000	-3.04332200000000	-5.95038400000000
H	-7.31517900000000	-4.15812800000000	3.75060700000000
H	-9.78250000000000	-1.30915800000000	4.07997000000000
H	-9.40319700000000	-2.75383200000000	1.45013700000000
H	-5.76898900000000	-6.41021300000000	1.77986100000000
H	-3.08084500000000	-8.75083000000000	-0.46797100000000
H	-7.02180600000000	-6.69287800000000	-2.78419800000000
H	2.78359000000000	-7.36886300000000	-0.99247300000000
H	4.89691600000000	-9.57738000000000	2.49409400000000
H	5.82084500000000	-9.04713700000000	0.34374500000000
H	-1.34437700000000	-7.77605500000000	0.87467400000000
H	10.66950900000000	-2.02029700000000	2.56380700000000
H	8.82314100000000	-3.95574100000000	4.78387700000000
H	11.59080000000000	-3.97453400000000	-1.85888000000000
H	5.33815600000000	-0.46451100000000	4.51995500000000
H	7.15219500000000	1.34004700000000	2.37234700000000
H	7.32251200000000	6.57841100000000	-0.51330700000000
H	5.00684300000000	-4.86484800000000	-3.24385800000000
H	7.90214800000000	-7.00114300000000	-1.50659200000000
H	2.44258300000000	-6.49974700000000	2.91143200000000
Ga	-3.40271500000000	-1.35157500000000	3.16434600000000
H	-3.74971900000000	-1.02031200000000	4.63257400000000
H	-3.55156400000000	0.02808700000000	2.29848400000000
C	-2.15318600000000	-4.02713900000000	3.20878300000000
H	-3.13377100000000	-4.51498500000000	3.20180000000000
H	-1.87760200000000	-3.87300400000000	4.25690500000000
H	-1.43803600000000	-4.73370700000000	2.77027500000000
C	-2.17608400000000	-2.70843400000000	2.42795100000000
H	-2.39078900000000	-2.89283000000000	1.36916900000000
H	-1.18161100000000	-2.23638700000000	2.44682200000000
H	-3.09939600000000	0.10417100000000	1.14930100000000

I3-GaH 2

Electronic energy (r^{2}SCAN-3c): -30618.547497224154 E_h
Electronic energy (ωB97X-D4rev/def2-mTZVPP): -30626.742840495248 E_h
G_{corr} (673.15 K, 1 atm): 0.42362 E_h
Number of atoms: 250

Cartesian coordinates (Å)

O	1.48841823479817	2.89617455304681	1.02886625975598
O	-1.94354872017817	6.77314902072941	1.42706174323468
O	5.26784480692196	2.07862569610894	0.80390875892002
O	3.60715973689611	3.88279878982927	-0.15282558587258
O	6.09517283438982	4.57574239689970	0.41837881006253
O	1.45310261439881	3.28855321025357	-1.61188780992541
O	1.37062146438015	5.32727298400821	0.03299934808824
O	-1.71108431944155	6.88859999880154	-1.19215420891908
O	0.32451825096167	7.70227223758341	0.40524238254275
O	2.93380465380922	7.47848123902177	-0.04767019118774
Si	4.84784546549333	3.64012383403836	0.87003042840115
Si	1.98866837150966	3.84590448337816	-0.18621030457971
Si	-1.29415157802344	7.62924463303008	0.20160163044004
Si	1.64939268072881	6.79471184989189	0.66034413590362
O	8.95222714842544	-3.83472174463197	2.22541343748764
O	1.99879090681746	6.65556783031230	2.24402430028795
O	7.14475445163479	-2.27972126276674	-0.92313772070152
O	2.50623141365360	4.75061740140150	4.08257360705907
O	6.74896497091341	-4.90439830948903	-0.77245700621431
O	0.04806871943849	5.11754073733874	3.14020781102556
O	5.85435991748277	-3.26797695817546	1.13651422948050
O	4.44551108808763	4.07477473226644	2.38809338495952
O	9.40145724693856	-5.01141211484268	-1.12815568295821
O	5.15720227515971	-0.24927088423954	2.01799615278682
O	7.73427256611083	4.37850377504289	-1.68983839742607
O	10.70337620759980	-3.18513904070694	0.34588523662596
O	5.42979559666250	5.63186266708495	-1.91114354074916
O	4.09439708973146	5.67577478853422	-4.17759920275703
O	8.76170270242662	-1.46091773520013	1.04431663152980
O	3.92240664169918	7.73674852215822	-2.53014785095218
O	1.17037743457479	9.52040813969241	-4.04548214132879
O	8.98092644147781	2.07158895925028	-1.88650704554358
O	6.94888111142195	0.19113394398515	0.10212446705061
O	2.27292290541069	9.55401381480378	-1.56414683913849
O	9.00034536560409	-0.54822211659008	-1.44313896165605
Si	1.43375771947366	5.86274387980049	3.55213251575000
Si	6.11596625375641	-3.44036543639863	-0.44930231432709
Si	3.63172608944904	3.62478788981178	3.72567499058264
Si	8.07927421560186	-5.83636334244478	-0.65673525476994
Si	10.87652764020398	-4.45893518698764	-0.67199997213555
Si	6.67158671213330	5.32660194718108	-0.90904003700604
Si	9.79490765071369	-2.60851593433858	1.56630813623292
Si	4.92456524361548	6.59508636574999	-3.11894347812932
Si	9.20528483236803	3.67308575353355	-1.66765709337084
Si	6.17265533036265	0.85348618596926	1.37256934919629
Si	1.72955792998726	10.45285817587039	-2.82532558866140
Si	9.80054576584922	0.68293593606879	-2.15359280468631
Si	7.95551231869531	-1.01627444381478	-0.30284202333703
Si	3.46916236811526	8.51807696689820	-1.17858204598430
O	2.97815932657195	-0.46969928191485	3.60934875627306

O	0.95615829036867	0.76683399257949	2.47663891188872
O	1.25016681404673	0.88914969407284	5.08095731311984
O	2.96941739032995	2.16332915661420	3.47572827279400
O	-0.41123665858868	1.19206801888715	0.27882083607836
O	-0.82431358642792	2.73305882836422	2.35687080032857
O	-3.75456252068372	4.98854063881258	0.57065945844613
O	-2.53410606710258	4.66232016958975	2.92937348258829
O	-1.17399428964182	3.43362414846145	4.83798546881917
Si	2.04674444249817	0.84365127803595	3.67467441791041
Si	0.30539661924047	1.91011769118082	1.53374696515707
Si	-3.13360852305526	5.74793572845146	1.86659722665604
Si	-1.10865499100065	3.99938476773929	3.32750021340306
O	8.28955061946819	-6.04358484918219	3.49644651394715
O	-3.19855619171247	-3.06557683593339	6.58086392602722
O	6.68166856580407	-7.84701488944498	2.44450909050585
O	-1.31037934321287	-1.56951709216622	7.69646789486787
O	8.25678127136544	-6.34225853368378	0.87210005767704
O	-5.42595166480772	-3.48372356319013	5.24659543648555
O	6.65224929990866	-3.92583385437309	3.57193556261608
O	-5.13443005302598	-1.21886970233002	6.59064173595828
O	-8.67357586779252	0.34575602829720	5.57471136345717
O	4.47681629269668	-2.51290452461585	3.25187060176194
O	-6.44767908167430	1.04856351321574	6.95451653162142
O	4.49172834473939	-5.06555544227892	2.60538450933404
O	-3.88729833474777	0.85864045380092	7.58039750513728
O	-2.25598210159179	2.84923672417537	7.14284026593609
O	0.39705589034606	2.65729715617990	6.86389197485354
O	4.25489896595486	-7.39981934225752	1.37506233380938
O	-1.50874781095275	0.86714399711816	8.78334914823493
O	0.71843811789778	0.08177625194198	7.52682180564456
Si	8.12655518122004	-7.12400497440679	2.28700378960546
Si	-2.12835268429605	-2.97495417728788	7.81815156757722
Si	8.20053276209807	-4.41729332404327	3.55227707920648
Si	-4.80632432873469	-2.81374776817833	6.58645476395901
Si	4.52874758872703	-0.89548625558653	3.37653014425853
Si	-8.08355857605794	1.12940784890162	6.87813490166825
Si	5.36089283812727	-3.70555918058597	2.61446790896171
Si	-5.30153069257434	0.07057697896006	7.56880744622751
Si	-0.93766534836866	3.48792606768168	6.44186912780820
Si	3.44304024827235	-6.09984235186653	1.92536364796404
Si	-2.74971904255117	1.78730055641988	8.26897616660928
Si	1.29664159405982	1.31862827593510	6.64756466472813
Si	5.41675217244106	-8.50883126249956	1.64880003396058
Si	-0.51590314850891	-0.38572380367494	8.47487288730766
O	1.80678867618242	4.27132943560447	-4.05613374984495
O	-0.06530559539597	5.27066822852265	-2.46358073334689
O	-0.61843945235723	7.68127844587687	-3.46547804843128
Si	0.70554057311536	3.91888285274903	-2.91542337102934
Si	-1.20387466548026	6.40448579839383	-2.65501339346170
O	1.24854459206155	-1.73139364417238	-4.50074723306106
O	-3.98869830799098	3.94140326642445	-4.54754669110629
O	3.34243179577125	-2.95948143355347	-3.48306898780048
O	-2.44736410482310	5.81263878108202	-3.50502593651235
O	-3.82803485061544	4.16926987399800	-1.91775485672450
O	1.33525620153965	-4.41220045521322	-4.41639402916624
O	-4.66949489852850	1.77067164468346	-5.91586149342599
O	4.72051505984640	-3.30737856896861	-1.25771679872051
O	-2.54946882404421	1.71279262447240	-4.39047844779806

O	-3.17094521067986	-0.36863448534788	-6.53609475439058
O	-1.11125655050820	-1.79611026323782	-5.65917930797161
O	-0.32710607526488	0.32843821769190	-4.21274117554170
O	-0.36321833563028	2.85641323520485	-3.49291694675352
O	1.78179436577125	6.92732918156911	-4.27198970498962
Si	-3.84222356495585	4.99895631358990	-3.31617258165438
Si	2.19325001596129	-3.04493540924208	-4.63979167878399
Si	-4.04812376824513	2.31593623288737	-4.52825615581835
Si	4.00873855462468	-4.07681638884360	-2.50744068486023
Si	-4.32516773378056	0.66616669426541	-7.07027981680324
Si	-0.30329804622617	-1.29253155823762	-4.34621110996912
Si	-1.21827687924816	1.48075840349875	-3.50849856515234
Si	2.55638785998759	5.56681586854620	-4.71100313870257
Si	0.62514312392221	8.05111447640647	-4.45449695170801
O	-2.79766872288943	-5.59376195964737	-7.11657262015693
O	-4.52109042110523	-4.33346756197963	-5.53970349061187
O	-2.91241206540995	-2.93064464821991	-7.22242714818505
O	-2.83888711765262	0.14172834142576	0.16886229599357
O	-2.48396626059018	2.77423087780596	-0.14935534015927
O	-1.66309250068562	1.01800783794419	-2.01411300947229
O	-5.13135053666645	2.80645603185066	-0.00489789453847
Si	-3.76586522427154	-4.29535304550110	-6.97864113830526
Si	-1.85839469533241	1.30243397702589	-0.43723752325781
Si	-3.81774147672287	3.68177656770146	-0.37964378503871
O	-4.99801280946575	1.89311750762091	-3.28388036614424
O	2.81589322200690	-5.05212492643854	-1.98257323005902
O	-5.61353624826625	-2.50117890155995	2.82968515791731
O	-7.38953976545999	-1.79196777421873	4.68209436339006
O	-0.25140933168247	-6.30640991488848	-3.40367327022866
O	-8.00831405369346	0.18036701281116	3.00304722820228
O	-0.39192996035985	-5.57346929961092	-5.96290738335537
O	-0.96085985763627	-1.92249591324329	-3.01288165130836
O	-3.48629674494373	-6.50694861952570	0.73198704027625
O	-0.96902525477548	-4.26939069791281	-1.86755891588602
O	-6.59433238646237	1.03720945857741	-1.38455000250206
O	0.62641646919142	-6.08394499743204	-0.87327862620820
O	-8.35642803327439	-0.62756696479439	0.48179876607564
O	-7.36419699415753	1.78206734968088	1.02275553556207
O	-1.87386405041958	-6.74798718699131	-1.40533722105428
O	-6.53955778561781	-0.21068926980806	-3.70619563049347
O	2.70768638321837	-5.41966546175620	0.64043275031567
O	-9.07330018331823	-2.66169644411249	-1.03705868236885
O	-4.47486158553593	-0.41245884681190	-2.07775423718090
O	-7.01371491239778	-2.91433707751136	0.61015006315942
O	-8.04241720878906	-2.38061843525929	-3.48103312664667
O	-5.38835502577121	-2.61332992081217	-3.74319335648384
O	-7.26418257533433	-4.36640962374629	-1.88831363717364
O	-5.00639975446454	-5.15349419956588	-3.07606720123374
O	-2.90094864268894	-3.62201415523404	-3.60257496917388
O	-5.69511319791792	-6.15088420850049	-0.71777662869150
O	-2.76875100701147	-2.51506582209534	-1.16786701968264
O	-4.76644296729781	-4.21552280493143	0.92486663902996
O	-4.66711751525879	-1.54610587611488	0.68184451807918
Si	-6.48176589550420	-3.01310637675923	4.11064603474703
Si	0.59988921120351	-5.83277147893133	-4.70727736278351
Si	-8.49689822950103	-0.65347155201177	4.31550849279780
Si	-1.24399479203553	-6.06980554122422	-7.26005202518605
Si	-6.64178758472254	2.28921143682914	-0.35280218409125

Si	-2.43801039255120	-7.48817799452477	-0.05857112339763
Si	-0.61972372894206	-5.84091468922668	-1.89176046268377
Si	-8.42973382674766	0.64521846030500	1.49556170087668
Si	-5.62616216936559	0.55443569309601	-2.60205317252099
Si	2.24946468817238	-6.02062669644737	-0.81057166927144
Si	-8.48825001574088	-2.24252448072322	0.41687763338784
Si	-6.70855132357938	-1.75449143778735	-4.17520493683360
Si	-8.52078839663807	-3.44537425286820	-2.35605116728612
Si	-4.44803760069575	-3.91864473199591	-3.97641843575889
Si	-6.26000365029777	-5.61819956781842	-2.14693417534526
Si	-1.92833277627515	-3.07932698035030	-2.40136415242124
Si	-4.95864703643247	-5.82778233856330	0.69993034973779
Al	-3.68763611298792	-1.14224678023977	-0.76088736861805
Si	-5.52505727524929	-2.82106367700280	1.23732123147847
Si	-2.11204806602565	-1.55030387437684	-6.91116662387144
H	11.67374699811842	-5.53703598746025	-0.03807399952156
H	9.19348299738350	-8.10585797943379	2.36346300080957
H	-3.88155000243274	1.39981099784974	-8.25725803942013
H	-5.54661901416375	-0.07985100308516	-7.37196301134286
H	-6.93206199210727	-1.73335100627201	-5.60666704842214
H	-9.61083499240370	-4.23371601337825	-2.91527902073194
H	-4.80806199875740	-4.38616399251153	-7.98466802514359
H	-1.25558101124963	-7.52827095073345	-7.35483801831407
H	-8.46603564240908	2.53218801841887	6.77163616969133
H	-4.20950398167282	6.50518798103033	2.49497289068626
H	-1.81143199777071	9.00591900218731	0.20164699907449
H	0.67612793515710	11.32783143937238	-2.29765487497109
H	-4.95336996862676	5.93157195496841	-3.34598497036165
H	-7.43212819850687	3.36467329983644	-0.94325520112429
H	-9.81433440767548	1.14100181706002	1.45030697808984
H	-3.26082299456998	2.50672899271902	9.43326898393752
H	-0.74504099336285	4.85495099846393	6.90563300201929
H	1.17176500924002	6.84606700874594	4.60638300240615
H	0.01383700135127	-0.85268900175900	9.75320600666759
H	2.65630399463037	1.61378700044833	7.09828799870882
H	4.53076901815537	3.47379800214803	4.86924400873840
H	1.53720802508275	-6.93798302428492	-4.97365700012038
H	4.63541501994961	9.21535602511021	-0.64625199363424
H	-8.67488780700341	0.53574627904857	8.08886451980492
H	-5.67875097142885	-0.35844194498225	8.92691782074025
H	-5.40516100721277	-3.45826601993801	7.74597000667503
H	-2.86007300763002	-3.03986699275531	9.10083199976902
H	-1.22700997934834	-4.11162701060458	7.70400899141313
H	2.57413299458764	5.42807900413681	-6.16363199771765
H	0.22482899582979	8.10275901125802	-5.85603202083328
H	2.79507606301503	11.25689304127840	-3.42192604096316
H	6.06067099835755	7.26917500664801	-3.76691100066968
H	10.01064999918303	4.23779399900359	-2.76113100043752
H	9.87926500342773	0.42371400079096	-3.59460900119316
H	11.16639499359438	0.78744100194489	-1.60472500028126
H	9.89847199786269	3.90341200113844	-0.38688400244985
H	-1.32399897936590	-1.19594398237421	-8.10330102748613
H	-0.61662896334247	-5.49814398620689	-8.46011603460227
H	2.84818300544895	-3.04332200348385	-5.95038402716404
H	-7.31517901044160	-4.15812798648407	3.75060699286165
H	-9.78250011637775	-1.30915801646013	4.07996996450464
H	-9.40319713293080	-2.75383207285731	1.45013708193131
H	-5.76898900377490	-6.41021300121848	1.77986100863456

H	-3.08084500949684	-8.75083000195207	-0.46797100638003
H	-7.02180600014185	-6.69287801231726	-2.78419801360135
H	2.78359000214907	-7.36886300855811	-0.99247300291360
H	4.89691600543415	-9.57737999359801	2.49409399846435
H	5.82084500113264	-9.04713700228822	0.34374500031005
H	-1.34437697773664	-7.77605499963470	0.87467400668992
H	10.66950901724980	-2.02029696831345	2.56380700003633
H	8.82314100215564	-3.95574098652900	4.78387699392745
H	11.59079999951154	-3.97453400125537	-1.85888001310792
H	5.33815600187785	-0.46451099697534	4.51995500075218
H	7.15219500353251	1.34004700733440	2.37234699781345
H	7.32251200492391	6.57841101323250	-0.51330699893962
H	5.00684300767096	-4.86484799844779	-3.24385800596101
H	7.90214800068105	-7.00114299306152	-1.50659200288976
H	2.44258300663802	-6.49974699959465	2.91143200110394
Ga	-3.71038303811085	-0.25115488293286	1.98995245811600
H	-4.72197195969856	0.88873543514528	2.24901694026288
H	-6.09412126049163	2.84515314971219	4.48455559750716
C	-1.99318684644153	-0.43169528704892	4.34329940920281
H	-2.85746799654850	-0.32638877021664	5.00840273189025
H	-1.61663880207963	0.57502997653535	4.13037100836100
H	-1.21245630707301	-0.95681343775604	4.90479711626553
C	-2.35477293072955	-1.19069615471015	3.05748000540969
H	-2.72848007796965	-2.19383726569067	3.29678599020732
H	-1.46837595701478	-1.32851640339570	2.42589923270417
H	-6.21751740119735	2.62527435629313	3.78460435754347

I4-GaH 2

Electronic energy (r^2SCAN-3c): -30617.375584191112 E_h
Electronic energy (ωB97X-D4rev/def2-mTZVPP): -30625.559492307049 E_h
G_{corr} (673.15 K, 1 atm): 0.42537 E_h
Number of atoms: 248

Cartesian coordinates (Å)

O	1.48831139168674	2.89587227139117	1.02877287247357
O	-1.94347110366801	6.77373331474832	1.42691484586209
O	5.26755080585491	2.07849995090157	0.80433398828344
O	3.60697638046624	3.88247586350687	-0.15328624750474
O	6.09492346884047	4.57548497776301	0.41823096676000
O	1.45247454194777	3.28851028857247	-1.61192414778156
O	1.37053154463019	5.32713621761192	0.03305836056331
O	-1.71074697977474	6.88858428055542	-1.19227662950687
O	0.32475016671584	7.70236120422059	0.40540661081872
O	2.93386625450923	7.47817103429334	-0.04803283204299
Si	4.84751420913952	3.64000750236911	0.86989384317744
Si	1.98840684489773	3.84568332071864	-0.18630731359750
Si	-1.29384739815614	7.62943864966956	0.20130330781203
Si	1.64957883327013	6.79458434095487	0.66031343794242
O	8.95227043280128	-3.83470391670287	2.22545485434965
O	1.99922804212912	6.65561187531541	2.24402181528903
O	7.14446879186121	-2.27952515301430	-0.92322875111450
O	2.50551593169125	4.74996225801993	4.08258088427131
O	6.74895118380153	-4.90434833112804	-0.77240204390223
O	0.04774805523749	5.11844586005851	3.13969589803402
O	5.85455430979657	-3.26792532485572	1.13662664493308
O	4.44474251606912	4.07499716823450	2.38782142635304

O	9.40141803928266	-5.01132246071622	-1.12811564275132
O	5.15745925719800	-0.24974051951024	2.01797362658953
O	7.73420899730684	4.37848053663308	-1.68979239290798
O	10.70339925070529	-3.18502869754385	0.34585020475009
O	5.42968518195889	5.63206815674334	-1.91098620303963
O	4.09448986568925	5.67562187275587	-4.17759628370095
O	8.76159549135709	-1.46100994658176	1.04423502472271
O	3.92245423303765	7.73687870874737	-2.53036892213403
O	1.17106821219683	9.52069099292164	-4.04609233994736
O	8.98096602625219	2.07160798760959	-1.88654450392554
O	6.94896640022515	0.19132802392371	0.10213181089036
O	2.27285857245243	9.55391697333662	-1.56417205310261
O	9.00036297914168	-0.54827847476632	-1.44318143311305
Si	1.43380895589543	5.86282275193798	3.55200584654347
Si	6.11584076690931	-3.44031500925476	-0.44927018942338
Si	3.63161875392238	3.62466141024677	3.72566449125069
Si	8.07923321205154	-5.83631030022516	-0.65674997844480
Si	10.87651773115229	-4.45888796192053	-0.67197603585606
Si	6.67153170129750	5.32660150829984	-0.90900636738114
Si	9.79487687266045	-2.60848723242756	1.56629444421213
Si	4.92456916502573	6.59511280528672	-3.11898156113087
Si	9.20528244941782	3.67311762173399	-1.66765383632336
Si	6.17263180468283	0.85340142739666	1.37265358791272
Si	1.72960079795399	10.45285497634573	-2.82536384238613
Si	9.80053860886652	0.68292575429676	-2.15360158405604
Si	7.95542227186622	-1.01624034930100	-0.30291806742725
Si	3.46918330633023	8.51805474635214	-1.17872080434612
O	2.97823437998428	-0.47030665930608	3.60916256738204
O	0.95668957031127	0.76643892117842	2.47657260431519
O	1.25023328911001	0.88866250955359	5.08090710210587
O	2.97005695248339	2.16271269664432	3.47627773439177
O	-0.41199344819122	1.19143176070284	0.27989265795629
O	-0.82359621203958	2.73285018985287	2.35785424910736
O	-3.75510084325079	4.98969405464639	0.56929410949190
O	-2.53414668343753	4.66204090082072	2.92768677359217
O	-1.17485366258453	3.43488470288270	4.83786953060718
Si	2.04706055558874	0.84319471435998	3.67476508460112
Si	0.30546602953431	1.90979155675222	1.53413389182897
Si	-3.13364006760866	5.74839336032737	1.86545470653010
Si	-1.10857427643510	3.99998013716154	3.32718470455836
O	8.28999798482654	-6.04369490776056	3.49643283942273
O	-3.19845727340747	-3.06600709478507	6.58075515272635
O	6.68156256516122	-7.84680791401657	2.44448757849588
O	-1.31053762782841	-1.56931901726853	7.69617746876103
O	8.25682647255449	-6.34234394870056	0.87203672166943
O	-5.42636682793988	-3.48398169674286	5.24674715472766
O	6.65229227725376	-3.92632133265725	3.57196059393959
O	-5.13388853336686	-1.21917926063677	6.59050289841081
O	-8.67509384652396	0.34446036270919	5.57626262303395
O	4.47700848444093	-2.51325602691048	3.25219642935869
O	-6.44779211257014	1.04708890261366	6.95126013573866
O	4.49181269634263	-5.06578268657266	2.60525747456031
O	-3.88723557498756	0.85807525500843	7.58085725002014
O	-2.25543773449844	2.84855994594388	7.14281479097244
O	0.39742990193385	2.65758011978487	6.86278877244035
O	4.25473077119605	-7.39994840139679	1.37490535030934
O	-1.50880136487935	0.86706279917293	8.78391824217457
O	0.71836156688753	0.08207724585960	7.52704915070760

Si	8.12656845938139	-7.12403368935145	2.28695505446112
Si	-2.12828282040400	-2.97492937059359	7.81811423855359
Si	8.20062578324379	-4.41741838017562	3.55228903660515
Si	-4.80620050463848	-2.81412533241048	6.58641322604529
Si	4.52890811002710	-0.89581859217368	3.37657004883919
Si	-8.08385086854724	1.12886690011630	6.87877740513320
Si	5.36099305143110	-3.70580538805070	2.61448453489220
Si	-5.30166888911537	0.07041087009389	7.56840209576726
Si	-0.93763575798356	3.48806400051184	6.44170880288480
Si	3.44297915604767	-6.09992718152963	1.92524803827386
Si	-2.74974280618165	1.78715335626224	8.26912956476642
Si	1.29672844085596	1.31858061589606	6.64735894536614
Si	5.41669973995306	-8.50881086735488	1.64879446239531
Si	-0.51598685306840	-0.38570222266868	8.47493626606719
O	1.80673472863744	4.27136637912695	-4.05604567495468
O	-0.06640168313367	5.27008169275423	-2.46428115530423
O	-0.61750709482688	7.68197823081042	-3.46487485590071
Si	0.70518071914796	3.91858923572496	-2.91572513187604
Si	-1.20389074535663	6.40498938739533	-2.65547258373438
O	1.24860380619973	-1.73092046402677	-4.50119772617971
O	-3.98799074851358	3.94243256208969	-4.54876641191312
O	3.34239824385638	-2.95929413610073	-3.48300422021902
O	-2.44770814354911	5.81518365711411	-3.50644248328216
O	-3.82494936706056	4.16933910289208	-1.91927013584670
O	1.33524909561614	-4.41183939836704	-4.41615931113317
O	-4.66924157927368	1.77139578220483	-5.91658076168290
O	4.72038774674753	-3.30752083497240	-1.25763353074732
O	-2.55067644692762	1.71281954682415	-4.38896174507872
O	-3.17070341532407	-0.36834842241301	-6.53588198586546
O	-1.11171711277900	-1.79696207583701	-5.65889578021498
O	-0.32857076166187	0.32788208122155	-4.21266118925364
O	-0.36309528845440	2.85575546531601	-3.49350333315075
O	1.78198718515146	6.92733719701095	-4.27214895509316
Si	-3.84157404821591	4.99976419914920	-3.31717355914592
Si	2.19324711367828	-3.04462476862774	-4.63982726056899
Si	-4.04868813406419	2.31696106539687	-4.52872518956370
Si	4.00865159152458	-4.07679755063052	-2.50751875110498
Si	-4.32506532873251	0.66620236306519	-7.07035119170526
Si	-0.30353387218747	-1.29301235100732	-4.34612713892105
Si	-1.21893386286441	1.48054847987918	-3.50801601496247
Si	2.55645062236879	5.56676010270011	-4.71102383166570
Si	0.62556400048197	8.05137130699310	-4.45459779821864
O	-2.79751133206948	-5.59344577629848	-7.11620312589274
O	-4.52094154623317	-4.33297151786601	-5.53948648713348
O	-2.91294043300148	-2.93022611312586	-7.22284324095454
O	-2.84154128705480	0.14468494910162	0.16948083801953
O	-2.48260369119228	2.77625549549335	-0.14893271895837
O	-1.66281529610965	1.01817644967573	-2.01316492414180
O	-5.13037007075910	2.80623552645644	-0.00791580525408
Si	-3.76591313817653	-4.29511758100030	-6.97855219569193
Si	-1.85880963821074	1.30369683720204	-0.43658011116305
Si	-3.81714316609167	3.68250569890996	-0.38089059405181
O	-5.00037037851933	1.89511910862557	-3.28538215035918
O	2.81589434503686	-5.05223195209637	-1.98269938519823
O	-5.61230754067641	-2.50200029831580	2.82983365345186
O	-7.38914307933300	-1.79127857348475	4.68053012104451
O	-0.25178187714152	-6.30523605096717	-3.40364932680446
O	-8.00923663530172	0.18529343909850	3.00603143864696

O	-0.39144806800913	-5.57331753481576	-5.96316280796609
O	-0.95991093366131	-1.92351669434261	-3.01245277058237
O	-3.48623040304580	-6.50688625076991	0.73200694333047
O	-0.97049863408541	-4.26946161788631	-1.86537300002157
O	-6.59481363945635	1.03731458430418	-1.38533399829894
O	0.62640243755856	-6.08396354007869	-0.87342367497780
O	-8.35487903867571	-0.62768863192893	0.48489667640466
O	-7.36459987962046	1.78249232607531	1.02113577528046
O	-1.87378972584156	-6.74864197790795	-1.40549476929695
O	-6.53928186108644	-0.21063005036109	-3.70673182244956
O	2.70768818346490	-5.41961935573184	0.64035219851485
O	-9.07249361053533	-2.66046843653942	-1.03688952206601
O	-4.47381042570891	-0.40969971393943	-2.07868772392994
O	-7.01370124306910	-2.91491076217764	0.61110853782861
O	-8.04214729107187	-2.38083014417800	-3.48101883585003
O	-5.38804699031911	-2.61279129109234	-3.74272828341964
O	-7.26448753598013	-4.36651904580173	-1.88784247382253
O	-5.00673878972725	-5.15328370322429	-3.07606786283294
O	-2.90110364726737	-3.62180325304685	-3.60159579551370
O	-5.69504185678931	-6.15117481868706	-0.71782543561311
O	-2.76892800272756	-2.51279985775044	-1.16745786832835
O	-4.76605360353500	-4.21514359049043	0.92379302019317
O	-4.66782473397410	-1.54470172725701	0.68188481185925
Si	-6.48155112940759	-3.01315724186198	4.11022513984036
Si	0.59999303676679	-5.83247196690973	-4.70721691376678
Si	-8.49740909885973	-0.65310200292783	4.31557927229122
Si	-1.24391524796882	-6.06972493831109	-7.25998687191122
Si	-6.64174025590920	2.28935425581602	-0.35335460807498
Si	-2.43804584420100	-7.48826120475032	-0.05852409934247
Si	-0.62007635381820	-5.84069863407471	-1.89144981986922
Si	-8.42902282680622	0.64691510870100	1.49670102253417
Si	-5.62655697460589	0.55573414723879	-2.60293714912869
Si	2.24940154611859	-6.02065093414695	-0.81060736750698
Si	-8.48783688468714	-2.24220957290331	0.41765551010121
Si	-6.70847618929356	-1.75440054638655	-4.17524208379680
Si	-8.52085470917340	-3.44510325093235	-2.35571596386939
Si	-4.44807708753494	-3.91831601480924	-3.97609759191807
Si	-6.26018700007907	-5.61814819213555	-2.14683280610622
Si	-1.92869946202086	-3.07894274820592	-2.40028915140713
Si	-4.95857015058730	-5.82764165325701	0.69980638581791
Al	-3.68842522882192	-1.14010576873911	-0.76095334599373
Si	-5.52462234211565	-2.82078867825757	1.23713445662381
Si	-2.11208123073949	-1.55027895131691	-6.91109547954080
H	11.67374700736457	-5.53703600574499	-0.03807400122139
H	9.19348300181550	-8.10585801070516	2.36346300099199
H	-3.88155000239440	1.39981099980683	-8.25725800178754
H	-5.54661900233510	-0.07985100083153	-7.37196300076358
H	-6.93206201639106	-1.73335100676009	-5.60666702179739
H	-9.61083495112214	-4.23371596079559	-2.91527895177550
H	-4.80806199888054	-4.38616401137096	-7.98466799826152
H	-1.25558098707460	-7.52827102450666	-7.35483800186048
H	-8.46603500794365	2.53218797560416	6.77163500481693
H	-4.20950401423599	6.50518800890346	2.49497301527413
H	-1.81143199247978	9.00591898255069	0.20164700229812
H	0.67612803008759	11.32783136396046	-2.29765508887778
H	-4.95336998553866	5.93157198384508	-3.34598500025035
H	-7.43212797504836	3.36467296499701	-0.94325499683893
H	-9.81433500636275	1.14100200448009	1.45030700292651

H	-3.26082300003911	2.50672900006712	9.43326900249087
H	-0.74504100031092	4.85495100110428	6.90563299971989
H	1.17176500366014	6.84606697968666	4.60638298456637
H	0.01383700189276	-0.85268900366629	9.75320600778061
H	2.65630399808867	1.61378699971651	7.09828800102761
H	4.53076899635758	3.47379799834256	4.86924399707741
H	1.53720799838392	-6.93798300052785	-4.97365699876309
H	4.63541501060605	9.21535599604549	-0.64625199552613
H	-8.67488802636900	0.53574596894355	8.08886504541573
H	-5.67875099480822	-0.35844200227501	8.92691799370111
H	-5.40516100424307	-3.45826601992426	7.74597004386206
H	-2.86007300455147	-3.03986699212697	9.10083198891208
H	-1.22700999024272	-4.11162699874653	7.70400899406273
H	2.57413300093786	5.42807899288664	-6.16363200038122
H	0.22482900219437	8.10275899360349	-5.85603201456177
H	2.79507598397522	11.25689298191634	-3.42192598607231
H	6.06067100126741	7.26917499547832	-3.76691099806312
H	10.01064999912630	4.23779399753024	-2.76113099830848
H	9.87926499450308	0.42371399911677	-3.59460899730456
H	11.16639500037843	0.78744100084918	-1.60472499937726
H	9.89847200065044	3.90341200060374	-0.38688400106646
H	-1.32399900460042	-1.19594400938857	-8.10330098905502
H	-0.61662901376075	-5.49814400417565	-8.46011599017770
H	2.84818299411664	-3.04332200025513	-5.95038399192968
H	-7.31517900766512	-4.15812801379483	3.75060699472844
H	-9.78250001934697	-1.30915801307599	4.07996999241528
H	-9.40319699804102	-2.75383199921932	1.45013695950608
H	-5.76898900928426	-6.41021300594978	1.77986101823817
H	-3.08084500796662	-8.75083000922098	-0.46797100682960
H	-7.02180601566151	-6.69287802361156	-2.78419801008846
H	2.78358999478357	-7.36886298982214	-0.99247299101198
H	4.89691599383190	-9.57738001295851	2.49409400519147
H	5.82084499016341	-9.04713699243932	0.34374501100840
H	-1.34437699453306	-7.77605499208815	0.87467400361508
H	10.66950898756767	-2.02029702356749	2.56380699672836
H	8.82314100293067	-3.95574101882261	4.78387700091267
H	11.59079999553416	-3.97453400184850	-1.85887999548269
H	5.33815599594023	-0.46451100343816	4.51995500081401
H	7.15219500476648	1.34004699941762	2.37234700105261
H	7.32251199418995	6.57841099055752	-0.51330699892170
H	5.00684301073926	-4.86484800534189	-3.24385800298283
H	7.90214800019802	-7.00114299863502	-1.50659199990325
H	2.44258303478007	-6.49974701682441	2.91143196979395
Ga	-3.71064989615259	-0.24991041086250	1.99071594091831
H	-4.72309477153252	0.88849057042848	2.25896489677808
C	-1.99071975366959	-0.43460278969359	4.34193478527470
H	-2.85414710947289	-0.33281252634480	5.00871890851592
H	-1.61633491200059	0.57345709917101	4.13142099814429
H	-1.20830462255065	-0.95976255874114	4.90109046417636
C	-2.35269310934580	-1.19067885476947	3.05454470765827
H	-2.72543907947518	-2.19477531233382	3.29141009643900
H	-1.46671674609915	-1.32605338834050	2.42186035859839

I5-GaH 2

Electronic energy (r^2SCAN-3c): -30617.323628861450 E_h
Electronic energy (ωB97X-D4rev/def2-mTZVPP): -30625.507223181208 E_h
G_{corr} (673.15 K, 1 atm): 0.41604 E_h
Number of atoms: 248

Cartesian coordinates (Å)

O	1.49066238375580	2.89919694948138	1.03044121039956
O	-1.94461086662868	6.77185477432337	1.42704674000059
O	5.26891096902994	2.08010569139917	0.80263013726635
O	3.60808847022728	3.88455004491228	-0.15431228702705
O	6.09463070746436	4.57756961920442	0.42001762733617
O	1.45425141636259	3.28909646226239	-1.61198874656135
O	1.37097574390078	5.32871007892073	0.03048009856260
O	-1.71086261863495	6.88903131592417	-1.19117815133421
O	0.32411213850453	7.70259894624887	0.40732217817871
O	2.93351209956384	7.47982221042351	-0.04640840000021
Si	4.84725741199145	3.64103139128703	0.87015344923806
Si	1.98948490592807	3.84695861698163	-0.18609327560009
Si	-1.29426453116856	7.62915503913624	0.20290153580266
Si	1.64934633906245	6.79480624275596	0.66092506314227
O	8.95037419984760	-3.83349478327947	2.22563580034743
O	1.99849385963182	6.65384217256876	2.24426354959135
O	7.14516689276315	-2.28006358427939	-0.92325871092033
O	2.50630468696815	4.75135888868424	4.08460680771254
O	6.74870178122964	-4.90446475479685	-0.77122490101255
O	0.04773399513795	5.11625234169292	3.14241114798868
O	5.85361044668999	-3.26578522617023	1.13653132442954
O	4.44247825114333	4.07323755288331	2.38808628123654
O	9.40123764076674	-5.01133208652325	-1.12869123355225
O	5.15349612300733	-0.24582942438344	2.01859824503966
O	7.73385380022863	4.37789590228302	-1.68862907687111
O	10.70258860318331	-3.18547344057142	0.34613258340790
O	5.43019757037567	5.63177332857043	-1.91100638660450
O	4.09156991286033	5.67296519346016	-4.17465948399548
O	8.76194680929905	-1.45999894728712	1.04430069684437
O	3.92191738789927	7.73617216320103	-2.52982538078037
O	1.17166308945112	9.52009815496481	-4.04597375281840
O	8.98102601979751	2.07175711631327	-1.88678086272287
O	6.94715447234474	0.18997543944715	0.10273310615064
O	2.27294249006076	9.55409185008068	-1.56412921094169
O	8.99956534779581	-0.54766341502902	-1.44317782783536
Si	1.43320035571318	5.86200047411070	3.55305025007971
Si	6.11602216083421	-3.44016754639018	-0.44882442278325
Si	3.63110757019700	3.62503432538530	3.72786122248777
Si	8.07931580143956	-5.83643419105419	-0.65653639526096
Si	10.87624519307689	-4.45898906596981	-0.67202241173305
Si	6.67144655962498	5.32671095649448	-0.90821544837559
Si	9.79435063206461	-2.60825823516195	1.56652161030862
Si	4.92386597563868	6.59429896883231	-3.11878223843954
Si	9.20520343818201	3.67318988823696	-1.66750951847442
Si	6.17174160805081	0.85437262191948	1.37267150218583
Si	1.72969335999390	10.45279919363746	-2.82531898177283
Si	9.80045834260746	0.68301435004157	-2.15358611378953
Si	7.95511478122725	-1.01617364183205	-0.30267475376644
Si	3.46908293644395	8.51807518051860	-1.17831244352868
O	2.97826284001490	-0.46847757964738	3.61798737302828

O	0.96129515278913	0.77213617859903	2.47998193358399
O	1.24133392722966	0.88445041831389	5.08469289614056
O	2.97006955620129	2.16232078161342	3.48714564953730
O	-0.40320788974289	1.19294213522735	0.27821268575406
O	-0.82428392525377	2.73344137132203	2.35431196601795
O	-3.74975589355473	4.97893515919360	0.57343998212334
O	-2.53351136773287	4.66203862293299	2.93357785136592
O	-1.17306880969800	3.42979878621669	4.83949237003737
Si	2.04428038482701	0.84419268380272	3.68148601284518
Si	0.30936701178461	1.91013051295891	1.53596694995474
Si	-3.13265571441150	5.74508963042090	1.86828517633863
Si	-1.10774885477142	3.99620754786277	3.32935922144106
O	8.28575584947507	-6.04191663214168	3.49577949459156
O	-3.19931988925729	-3.06718593802624	6.58245418360376
O	6.68166872223603	-7.84755724818933	2.44361374901489
O	-1.31134443985178	-1.56734123249813	7.69208046658560
O	8.25823277707352	-6.34248824029511	0.87192121600299
O	-5.42793246570392	-3.48474653524567	5.24720502306277
O	6.65038904912277	-3.92061957192816	3.57256005747694
O	-5.13520811837368	-1.21971431778245	6.59012832014872
O	-8.67517472845915	0.34343173764951	5.57755624454425
O	4.47073898997038	-2.51071233434305	3.24739346068927
O	-6.44728258366565	1.04732086410082	6.95149152023906
O	4.49072893118134	-5.06357496389096	2.60525885086265
O	-3.88699586981333	0.85921010923514	7.57990978239573
O	-2.25383506013420	2.85017403764363	7.14645666717315
O	0.39945972053736	2.65919409554183	6.86659287107823
O	4.25504342113811	-7.39734458103169	1.37525553212557
O	-1.50983591224826	0.86611279037585	8.78604931202731
O	0.72060735838881	0.08410253775464	7.53402995955819
Si	8.12599297454834	-7.12385950191330	2.28699133757458
Si	-2.12774184338634	-2.97427293686611	7.81755517616255
Si	8.19806818009804	-4.41554726599484	3.55251664678004
Si	-4.80595827793812	-2.81422988996713	6.58658205695498
Si	4.52769311429023	-0.89324168370023	3.37763144437183
Si	-8.08280875627728	1.12887331986648	6.87958225621292
Si	5.35881773702695	-3.70371508781371	2.61412805651052
Si	-5.30037716338564	0.06964802320498	7.56777151050555
Si	-0.93613624284924	3.48777264111705	6.44334622833659
Si	3.44067654151095	-6.09756398241088	1.92567638650512
Si	-2.74983850148232	1.78678442425219	8.27047174594759
Si	1.29611488550568	1.31828789871533	6.65044993015617
Si	5.41538259562044	-8.50788362842123	1.64849509434325
Si	-0.51698032230349	-0.38666793268197	8.47577807451671
O	1.80019334850944	4.26915513593936	-4.05895943032433
O	-0.06806435128370	5.26917213007003	-2.46337588669155
O	-0.61753915334503	7.68225989279411	-3.46413243962301
Si	0.70269660115548	3.91698072941202	-2.91394599766875
Si	-1.20382173321660	6.40571357922369	-2.65413969837217
O	1.24660421925356	-1.73320313705202	-4.49516036633568
O	-3.98475115282344	3.94374899192414	-4.54787321258477
O	3.34309188329783	-2.95945872961120	-3.48359906691304
O	-2.44857540667683	5.81688768312146	-3.50440269261401
O	-3.82052777134889	4.16635763356575	-1.91692678740801
O	1.33466193436280	-4.41276538367089	-4.41507417037929
O	-4.66737095761900	1.77319492612000	-5.91747785931010
O	4.72053580790216	-3.30708354175340	-1.25766534685292
O	-2.55774662608934	1.70749865894696	-4.37740029971158

O	-3.16974915626127	-0.36720103959095	-6.53511020605768
O	-1.10873567117145	-1.79692859236045	-5.66159325157360
O	-0.33093247111441	0.32714439476943	-4.21224733476129
O	-0.36935804250386	2.85228261007012	-3.48335656413736
O	1.78051528241826	6.92573077163258	-4.27165814309115
Si	-3.84071240814020	4.99957104434556	-3.31438413750545
Si	2.19292708327533	-3.04551736115889	-4.63904519794346
Si	-4.05169686054217	2.31893411182819	-4.52757733068233
Si	4.00918827004283	-4.07659956032081	-2.50735450885814
Si	-4.32468287731537	0.66657415112674	-7.07026614562321
Si	-0.30532220016815	-1.29352516451876	-4.34600974555216
Si	-1.22246789046576	1.47550621156434	-3.50168098874696
Si	2.55412337727131	5.56463542074402	-4.71047637131947
Si	0.62544465942007	8.05105038989717	-4.45435474670833
O	-2.79728062595875	-5.59277910077964	-7.11273199171385
O	-4.53496715183042	-4.33690053946050	-5.54536471883203
O	-2.91596949817478	-2.92972424897433	-7.21785329029332
O	-2.82151288378554	0.11430104508700	0.17679801789498
O	-2.49126033343281	2.75442956871396	-0.14670201363538
O	-1.66334588712542	1.00166292762745	-2.00880241266704
O	-5.13203999427281	2.80069183930143	-0.00811707214141
Si	-3.76804050544206	-4.29606217142332	-6.97853978502366
Si	-1.85073486205868	1.28914625393006	-0.43195869279609
Si	-3.81597876383717	3.67416909249379	-0.37966648965668
O	-5.01455523677489	1.90402987557947	-3.28994143165786
O	2.81439947343829	-5.05004786627013	-1.98338271184302
O	-5.61085521344221	-2.50046954526522	2.82624639278671
O	-7.38793922690157	-1.79086018826639	4.67692554675636
O	-0.25199496606194	-6.31169947732767	-3.40694134853282
O	-8.00969838187844	0.18702311365300	3.00750980257066
O	-0.38995619626711	-5.57246209271890	-5.96453902967696
O	-0.97056907558538	-1.92336919033297	-3.01525557988908
O	-3.48461240900653	-6.50468282946800	0.73057850152477
O	-0.97415441505705	-4.27367309784623	-1.87930985653399
O	-6.60094275784399	1.03642805475203	-1.38631647763260
O	0.62665685881316	-6.07994481500230	-0.87741456116214
O	-8.35372805977365	-0.62858362059869	0.48665677312668
O	-7.36312646388848	1.78046888910132	1.02280764140001
O	-1.87387605262283	-6.75071527937268	-1.40662896399575
O	-6.53798009039403	-0.21278722898186	-3.70581803309258
O	2.71162902141754	-5.41704531312438	0.63685135427583
O	-9.07282971057637	-2.65902395976027	-1.03829560496679
O	-4.46700187610910	-0.39055269654218	-2.07864304419694
O	-7.01569118024859	-2.91783643105262	0.60904013507323
O	-8.03942799341350	-2.38322358382060	-3.48156699011357
O	-5.38372109545510	-2.61206024503867	-3.74245731173278
O	-7.26472290896606	-4.36722985036086	-1.88653977565582
O	-5.00736218006794	-5.15339914013689	-3.07635304571803
O	-2.89853934695603	-3.63267967331137	-3.62513451304400
O	-5.69492882928826	-6.15208956077901	-0.71791926700076
O	-2.79693429261742	-2.53551822325683	-1.19146673505855
O	-4.76462660080801	-4.21446886896784	0.91922584086699
O	-4.67468238842480	-1.54437300623380	0.67128952135417
Si	-6.47941562990680	-3.01329317890850	4.10939221428800
Si	0.60032932256570	-5.83350637070345	-4.70832962292984
Si	-8.49742111026695	-0.65329201707146	4.31665458398593
Si	-1.24396859835367	-6.06958640164368	-7.25988298982473
Si	-6.64443951725064	2.28816022420178	-0.35340268421155

Si	-2.43721622138803	-7.48867675112318	-0.05792457051770
Si	-0.62056701781833	-5.84533480997883	-1.89576148034172
Si	-8.42961662751581	0.64630126000301	1.49821821274290
Si	-5.63032883592108	0.56194079425574	-2.60455996300888
Si	2.25016306166332	-6.02010700192271	-0.81097123090514
Si	-8.48926586498836	-2.24260441500606	0.41702063306271
Si	-6.70621605073906	-1.75603897130436	-4.17554806978406
Si	-8.52053796725079	-3.44582860629101	-2.35579918945539
Si	-4.45124525773402	-3.92252027418818	-3.98286018517169
Si	-6.26035093761746	-5.61877297776433	-2.14643141919294
Si	-1.93622150953249	-3.08826897262932	-2.41740070156451
Si	-4.95765685177019	-5.82848050802497	0.69933745500474
Al	-3.68813322693122	-1.14887359983663	-0.77490991933257
Si	-5.52774117127226	-2.82420192135402	1.23522362822891
Si	-2.11242004563360	-1.55023091827288	-6.91102740342092
H	11.67374700397130	-5.53703598684250	-0.03807401648238
H	9.19348299323473	-8.10585798854036	2.36346299588806
H	-3.88154996363261	1.39981103324447	-8.25725803018510
H	-5.54661904396718	-0.07985102540862	-7.37196298193669
H	-6.93206200488458	-1.73335099460998	-5.60666702432276
H	-9.61083499794406	-4.23371599828064	-2.91527898133046
H	-4.80806199883990	-4.38616400133195	-7.98466800099943
H	-1.25558100289063	-7.52827101545087	-7.35483799423904
H	-8.46603500395355	2.53218798868142	6.77163500632974
H	-4.20950399816433	6.50518800210657	2.49497299483891
H	-1.81143200058251	9.00591899390701	0.20164700211665
H	0.67612801213449	11.32783100324395	-2.29765500549014
H	-4.95336998537865	5.93157198063225	-3.34598500701515
H	-7.43212798776896	3.36467297041521	-0.94325498919006
H	-9.81433499990789	1.14100199799804	1.45030700070207
H	-3.26082300533368	2.50672900194482	9.43326900412758
H	-0.74504100086856	4.85495100181140	6.90563299893563
H	1.17176499843534	6.84606700196343	4.60638300161586
H	0.01383699253282	-0.85268899573431	9.75320598369921
H	2.65630400901447	1.61378699936519	7.09828799799860
H	4.53076899523347	3.47379799355804	4.86924399232859
H	1.53720799755380	-6.93798299789799	-4.97365700005618
H	4.63541500535111	9.21535600291208	-0.64625200185299
H	-8.67488798842539	0.53574601146575	8.08886496606338
H	-5.67875100430285	-0.35844200209975	8.92691800735431
H	-5.40516097790784	-3.45826597575220	7.74596993474120
H	-2.86007289557452	-3.03986706844098	9.10083186383314
H	-1.22701008918875	-4.11162702575795	7.70400928287398
H	2.57413300929340	5.42807900675593	-6.16363198050333
H	0.22482900128630	8.10275900412900	-5.85603200174290
H	2.79507596750901	11.25689298132940	-3.42192598025861
H	6.06067098373219	7.26917498895133	-3.76691099753701
H	10.01065000337008	4.23779400401812	-2.76113098978749
H	9.87926500104576	0.42371400468408	-3.59460897990890
H	11.16639498964800	0.78744099671481	-1.60472500912924
H	9.89847199328641	3.90341199586425	-0.38688401672746
H	-1.32399899506919	-1.19594399346718	-8.10330100691237
H	-0.61662900007987	-5.49814398420884	-8.46011600219966
H	2.84818300047525	-3.04332200608170	-5.95038397129079
H	-7.31517898414418	-4.15812797687162	3.75060702265820
H	-9.78249999660285	-1.30915799312070	4.07997000085698
H	-9.40319700030782	-2.75383200670437	1.45013698280360
H	-5.76898902882651	-6.41021302756775	1.77986103575198

H	-3.08084502971444	-8.75083011852453	-0.46797102488068
H	-7.02180600683719	-6.69287800696467	-2.78419800095477
H	2.78359002329799	-7.36886307195159	-0.99247299382854
H	4.89691600758452	-9.57738004064598	2.49409398478330
H	5.82084496460907	-9.04713697414494	0.34374506399401
H	-1.34437703534652	-7.77605471181606	0.87467398300610
H	10.66950898795337	-2.02029700802829	2.56380698915965
H	8.82314098447684	-3.95574100513445	4.78387699215733
H	11.59079999290744	-3.97453401339084	-1.85887997094316
H	5.33815602442044	-0.46451097673380	4.51995504666593
H	7.15219498391311	1.34004698985119	2.37234699103117
H	7.32251199895621	6.57841100188602	-0.51330699796615
H	5.00684299761004	-4.86484799636797	-3.24385799627885
H	7.90214800286127	-7.00114300491371	-1.50659200541984
H	2.44258310563921	-6.49974696238455	2.91143186920076
Ga	-3.69798906247813	-0.28556682022052	1.96126609921193
H	-4.65801278267828	0.87709394744046	2.27817967169785
C	0.35864275269418	-2.61298575848051	2.32334636393356
H	-0.20932182843656	-2.37673100580456	3.22012440418513
H	-2.69028163314126	-1.14337420337517	2.75354191572764
H	1.43181016404556	-2.44685695028154	2.36808786946925
C	-0.22859187709180	-3.07222052673411	1.22538788927112
H	-1.30170163882362	-3.23901812168300	1.16815006558993
H	0.34228947416477	-3.30278488774856	0.33036527307298

I6-GaH 2

Electronic energy (r^{2}SCAN-3c): -30617.312313364739 E_h
Electronic energy (ωB97X-D4rev/def2-mTZVPP): -30625.440937199899 E_h
G_corr (673.15 K, 1 atm): 0.41191 E_h
Number of atoms: 248

Cartesian coordinates (Å)

O	1.56362467758425	2.83695070343640	1.04364367871345
O	-1.92570318302555	6.77865089453258	1.47530946934619
O	5.21724971344114	2.06211438957638	0.82651694356314
O	3.61947189429759	3.89755588160112	-0.22795246646042
O	6.12603198984953	4.56090855602549	0.45812071488009
O	1.37987953460882	3.23540332373244	-1.61751672828474
O	1.33828988476751	5.29675168668805	0.04873156517594
O	-1.73628702867070	6.87186497892693	-1.17224253486030
O	0.34844424153842	7.72261178965603	0.41685024373939
O	2.97447067512236	7.43120441668370	-0.06302399620087
Si	4.82650798195516	3.64537755396023	0.86133003086969
Si	1.98489296415762	3.81292764864975	-0.20840131059356
Si	-1.28847337767802	7.63774611701249	0.22331485136971
Si	1.66924645418166	6.77946079190277	0.66921323562713
O	8.97173254485491	-3.87686209712949	2.21692678096005
O	2.04689025468020	6.64210794089196	2.26280447566331
O	7.12013450196974	-2.31063811878003	-0.96599342980889
O	2.45005611923277	4.70693847396540	4.15035672329261
O	6.66839992345310	-4.93767001419882	-0.79440499562000
O	0.02552771865372	5.10253981001808	3.09225427256974
O	5.87968937344325	-3.27241054306995	1.17583132939550
O	4.36373701093573	4.08872526407340	2.37605566962836
O	9.36936533434477	-4.97910990039910	-1.10436921184484
O	5.12287913038388	-0.29026858644237	2.02984828423015

O	7.71181704327953	4.39378002377090	-1.72994489761860
O	10.73680859001664	-3.14462216695816	0.33738484518944
O	5.37931437349228	5.67095751698414	-1.86396332862051
O	4.13115133042505	5.64797159162385	-4.21310274977187
O	8.71717622547872	-1.46545178277127	1.06148685503165
O	3.90312061938388	7.78092723815339	-2.59082296296572
O	1.24525919150163	9.55768777260297	-4.11717214811946
O	8.95350258705215	2.05421401169760	-1.87342811979952
O	6.91201408829862	0.18486652462929	0.06999557790298
O	2.23824163968332	9.55504228103531	-1.53600060205158
O	9.01506305611799	-0.59638160739320	-1.45605578804910
Si	1.41432490413218	5.85080405000379	3.56375248524895
Si	6.06768250268679	-3.44822733045845	-0.43641472544977
Si	3.59959325391553	3.59680640189317	3.74915804315460
Si	8.04080785171554	-5.83661481054182	-0.65249606264704
Si	10.87897692474003	-4.44915254458351	-0.67312114299185
Si	6.65831365021351	5.33401913442378	-0.89626759937748
Si	9.79464437241599	-2.60481104585345	1.56888312467266
Si	4.91946910702529	6.60333024179221	-3.13435040621282
Si	9.19148714871463	3.67140052193910	-1.67272539943869
Si	6.14916714351020	0.83132863918483	1.37904633017972
Si	1.73187078712260	10.46608716302370	-2.82710734145018
Si	9.80105934016118	0.66749809946101	-2.15696759386015
Si	7.92986546692684	-1.03665419717298	-0.32486678229738
Si	3.46921260314984	8.52157951498320	-1.18801423525510
O	2.93826434819701	-0.55070915216854	3.71209266565874
O	0.96500607449817	0.64425777246686	2.44041793866263
O	1.14762376851693	0.88904235190859	5.08508204648799
O	2.95171070262309	2.10244155715880	3.52916082970507
O	-0.32966730505360	1.13800380240471	0.19451645704805
O	-0.80224530558887	2.65527440881906	2.32719338291070
O	-3.76609093944313	5.03519273633251	0.50845697141420
O	-2.57847238834543	4.58462981466101	2.89397429207725
O	-1.13777406620434	3.41524878893263	4.82504063114374
Si	2.00229010859713	0.77480802865925	3.70037803712169
Si	0.35089307100347	1.83144563629905	1.50186253498134
Si	-3.14770768663270	5.74110334366287	1.86028848456916
Si	-1.12170718776702	3.94491534526784	3.28698678692019
O	8.31757825295452	-6.08177300376357	3.56814933120230
O	-3.17932200804728	-3.12666974440025	6.54279822001275
O	6.64940894246959	-7.85680543168882	2.49675171523065
O	-1.33307555428477	-1.54231780440398	7.67520310047862
O	8.18393754013420	-6.33998845053379	0.89915588434152
O	-5.42231549029521	-3.58408928849087	5.23661411725354
O	6.66383300677126	-3.92558129930422	3.63311430424745
O	-5.12617999981304	-1.24663747847731	6.54500066933456
O	-8.72774988004903	0.35448013362230	5.58708840045567
O	4.43087143587897	-2.57252012833084	3.30863269670872
O	-6.43283168923134	1.05605243960981	6.92327692420529
O	4.53325593333093	-5.13036809408629	2.62169262511807
O	-3.85554438895576	0.80858031307097	7.60185409775306
O	-2.32162291850922	2.89944163966853	7.12373581537304
O	0.36420153740008	2.63310282746151	6.93979734825590
O	4.21499005238125	-7.42148458004916	1.31957624750194
O	-1.44560567966517	0.92477876512584	8.76590244415138
O	0.80632552635017	0.02614681066850	7.56740003606926
Si	8.11307833938941	-7.14148882529194	2.32703700295799
Si	-2.12505906829521	-2.97797216189307	7.80693699514128

Si	8.21925705040992	-4.44107609789464	3.57449359832719
Si	-4.79721343079644	-2.85879774981210	6.56387757973661
Si	4.50244274944642	-0.93973613516936	3.41415515252561
Si	-8.08629546244484	1.13832080171643	6.88327093739006
Si	5.36571106536799	-3.73549187191183	2.65977926307418
Si	-5.29517581318098	0.04619040568412	7.54771487413945
Si	-0.94977122665470	3.49239803278192	6.44818402534989
Si	3.42668881855124	-6.10657956635041	1.91478671172932
Si	-2.74423363371827	1.80328877069360	8.26868031845779
Si	1.29444049651705	1.30534471898148	6.66895704358857
Si	5.39939597781047	-8.51746394694543	1.64349205889943
Si	-0.49317367326113	-0.38429007086042	8.47677263300753
O	1.83666100967831	4.20951269214495	-4.06713226644939
O	-0.05170086358025	5.31769352458304	-2.51829441191588
O	-0.58312451340795	7.81242243626174	-3.38715033263253
Si	0.67848200609690	3.91600661495398	-2.94507718381365
Si	-1.19884810337269	6.47478171201798	-2.66730767275145
O	1.30550862684610	-1.63379730198608	-4.51582013373768
O	-3.98583943960696	4.03650454793668	-4.67413788217541
O	3.38308352131410	-2.90227122350698	-3.48590626518600
O	-2.42834757191976	5.94275906883166	-3.61108654582659
O	-3.67632987704178	4.18362346066491	-2.00734793489810
O	1.32766256332652	-4.36229230258532	-4.40635017225902
O	-4.77884102847734	1.81655936044804	-5.95955585870103
O	4.63233277140134	-3.30764241571685	-1.19671121439069
O	-2.65653364975906	1.72724105441305	-4.42592780047394
O	-3.18011883989714	-0.36672202051464	-6.50621774379908
O	-1.08343133887894	-1.82578214410557	-5.65591487537481
O	-0.35097307215931	0.39639534615565	-4.31521382755723
O	-0.45731840322373	2.92011935525618	-3.55010102938212
O	1.75832883847777	6.89034154229497	-4.26609194967669
Si	-3.80017273056080	5.06102492480499	-3.39721751316004
Si	2.21296375404869	-2.99339248259293	-4.64042867960137
Si	-4.14037721490033	2.40419714113980	-4.58365635190392
Si	3.98918939135152	-4.07238718196354	-2.50971371128259
Si	-4.34105079852954	0.67524395441173	-7.07091932221260
Si	-0.27187412454296	-1.24237589689112	-4.35911166057111
Si	-1.28589434409756	1.51185708057644	-3.57705303661789
Si	2.56948625850356	5.53579069428771	-4.72035059326832
Si	0.64491208441341	8.08008697792895	-4.46059283357285
O	-2.83077941491747	-5.63402666866055	-7.09050021491699
O	-4.57379346637366	-4.29318863093229	-5.53255220593432
O	-2.90535893925267	-2.94542429171382	-7.23843835348278
O	-2.82313411336556	0.17323488993142	0.19335713399582
O	-2.38844896320730	2.81936679085415	-0.15928845394764
O	-1.69072040145124	1.02072943821644	-2.06169780424161
O	-5.06768583009145	2.79292976728979	-0.10379443841555
Si	-3.78650294362322	-4.30600212206336	-6.97321496467050
Si	-1.81667030078514	1.30656413406699	-0.46283937894740
Si	-3.74777906069110	3.70808757470888	-0.44612820374112
O	-5.12020774844629	2.05885900308597	-3.30794944288537
O	2.75747338229349	-5.05840071500112	-2.02874798631152
O	-5.59125563310540	-2.44964905807793	2.84464872561940
O	-7.39307022447767	-1.81444430717332	4.74544123256003
O	-0.25220709195532	-6.31035155904037	-3.38876558074291
O	-7.97386664980945	0.17203284069197	3.01896075962541
O	-0.37984686171311	-5.55300934608347	-5.96891626432542
O	-0.90893875636926	-1.85105565865503	-2.98316541409121

O	-3.47018143931930	-6.51954840065208	0.81613992601623
O	-0.94181810222815	-4.22903318193557	-1.83957283818118
O	-6.65935636568081	1.03194896373175	-1.38665640028662
O	0.58221968002769	-6.12888791046606	-0.82278843066224
O	-8.34419139368403	-0.59998514792924	0.47368389235683
O	-7.31853617864311	1.83118030702038	1.07162818854688
O	-1.94699751665941	-6.71488607028085	-1.41600098947107
O	-6.54150991301761	-0.13162624609269	-3.78445151515320
O	2.70230764420438	-5.36877834775651	0.63384952930245
O	-9.14034568493097	-2.66483546200037	-1.02744178744683
O	-4.44892414548212	-0.27145931026498	-2.12415727502817
O	-7.01952971818607	-2.91596701524187	0.61605110398573
O	-8.07683347378810	-2.31056400168531	-3.46605524515120
O	-5.40292073955354	-2.58783923591350	-3.68450705880475
O	-7.25470183604031	-4.34557893434963	-1.91086488367936
O	-4.95309580252999	-5.15574193724934	-3.04470118395603
O	-2.85845621331007	-3.57218207996770	-3.63218801769018
O	-5.68534834620800	-6.17075226511870	-0.69651918431078
O	-2.81048981822634	-2.46883769752550	-1.18393873073703
O	-4.73875384313139	-4.18813836795332	0.93321687288066
O	-4.69211102716288	-1.46462801925509	0.66744159476152
Si	-6.47548085084436	-3.02380005599472	4.11638589186757
Si	0.61269700329263	-5.81448262324301	-4.69595711609064
Si	-8.49906720678228	-0.66246013328310	4.33663814262086
Si	-1.25169623464647	-6.07193665384470	-7.26082863451020
Si	-6.62695746784565	2.30641229670141	-0.35033719134517
Si	-2.44706229841198	-7.49160019435002	-0.04310736204038
Si	-0.64914663835912	-5.83018981679807	-1.87293816372907
Si	-8.40891294314181	0.67799478924247	1.51095229923839
Si	-5.66590956589823	0.64930956414748	-2.63999298992162
Si	2.22228934476046	-6.02326003590379	-0.81071839623768
Si	-8.50630042711498	-2.22811516045946	0.41593443072714
Si	-6.71830831121272	-1.71297605897732	-4.17696757114416
Si	-8.54204436740537	-3.42455622536680	-2.35847616031096
Si	-4.43191357783753	-3.89097870050244	-3.95443013437594
Si	-6.24617866384835	-5.62634469671975	-2.13869586117492
Si	-1.90069598984315	-3.02378803771088	-2.39038931399959
Si	-4.94353996807340	-5.81803235807893	0.73138721053602
Al	-3.71160229337770	-1.06393972336701	-0.79452454366843
Si	-5.51054425694001	-2.78081175299978	1.22931019308978
Si	-2.10463695637326	-1.55381594911345	-6.90788164603855
H	11.67374700156125	-5.53703599811865	-0.03807399897792
H	9.19348300140191	-8.10585799987823	2.36346300095603
H	-3.88155000220125	1.39981100042694	-8.25725800110281
H	-5.54661900060419	-0.07985099943365	-7.37196300017806
H	-6.93206199863346	-1.73335099840679	-5.60666699836320
H	-9.61083500077409	-4.23371600052467	-2.91527899809350
H	-4.80806199799890	-4.38616400068252	-7.98466800005957
H	-1.25558100037836	-7.52827099906893	-7.35483800037911
H	-8.46603499978124	2.53218800027249	6.77163500010405
H	-4.20950400006696	6.50518800001894	2.49497299932110
H	-1.81143199994957	9.00591899820800	0.20164700205709
H	0.67612799920709	11.32783099939043	-2.29765499972456
H	-4.95337000003996	5.93157199849492	-3.34598500069577
H	-7.43212799900666	3.36467300006328	-0.94325499798531
H	-9.81433500133745	1.14100200069751	1.45030699993585
H	-3.26082299939645	2.50672899908262	9.43327000105429
H	-0.74504100043292	4.85494999871427	6.90563299960759

H	1.17176499852311	6.84606699784126	4.60638300111364
H	0.01383699972033	-0.85268900111857	9.75320600079303
H	2.65630400061152	1.61378700061266	7.09828800010897
H	4.53076900174599	3.47379799779909	4.86924400024063
H	1.53720799968329	-6.93798299926533	-4.97365699812134
H	4.63541500108284	9.21535600148930	-0.64625199843039
H	-8.67488799901540	0.53574600095864	8.08886499851877
H	-5.67875099881969	-0.35844199775141	8.92691800020012
H	-5.40516099990700	-3.45826600073319	7.74596999949446
H	-2.86007299857772	-3.03986699857172	9.10083199984798
H	-1.22700999829174	-4.11162699790373	7.70400899852214
H	2.57413299912541	5.42807899857677	-6.16363199963773
H	0.22482900130801	8.10275900033271	-5.85603199931072
H	2.79507599850348	11.25689300071400	-3.42192599975051
H	6.06067100085349	7.26917500018454	-3.76691099952953
H	10.01064999858110	4.23779399922867	-2.76113100049078
H	9.87926499787917	0.42371400052618	-3.59460900063271
H	11.16639499824700	0.78744100168685	-1.60472500007588
H	9.89847199806964	3.90341200100530	-0.38688400170478
H	-1.32399899926668	-1.19594399933179	-8.10330099962482
H	-0.61662900169638	-5.49814399758787	-8.46011599945512
H	2.84818300033076	-3.04332200116734	-5.95038399938853
H	-7.31517899981081	-4.15812800025972	3.75060700175227
H	-9.78250000183035	-1.30915800160646	4.07996999808557
H	-9.40319699857854	-2.75383200056818	1.45013700168782
H	-5.76898900008831	-6.41021299988506	1.77986100165426
H	-3.08084499945269	-8.75082999801487	-0.46797100041667
H	-7.02180600198740	-6.69287800087789	-2.78419799853986
H	2.78359000042523	-7.36886300061269	-0.99247300205160
H	4.89691599971166	-9.57738000002223	2.49409399909440
H	5.82084500096839	-9.04713700184680	0.34374500000385
H	-1.34437699982678	-7.77605499880284	0.87467400155537
H	10.66950899856089	-2.02029700018207	2.56380700009817
H	8.82314099950494	-3.95574100149971	4.78387700077464
H	11.59079999858017	-3.97453399994012	-1.85888000173060
H	5.33815599996824	-0.46451100180974	4.51995500115605
H	7.15219500175280	1.34004700159733	2.37234699783674
H	7.32251199882621	6.57841100156155	-0.51330699883452
H	5.00684300190412	-4.86484799841453	-3.24385800107844
H	7.90214800022762	-7.00114299801780	-1.50659200212765
H	2.44258300048599	-6.49974700017019	2.91143200074893
Ga	-3.49431419888718	-0.42918275208478	2.03668125377190
H	-4.36637571306747	0.72107122690936	2.63603040064460
C	-2.05497134547902	-1.65578004578626	2.53459190351738
H	-1.91318401092942	0.06707254187966	4.95508295643273
H	-2.33116819973042	0.68735180983783	5.09205066701400
H	-1.05164817383180	-1.38658175097854	2.15689269867776
C	-2.15855369381475	-2.79400356666344	3.24201808883017
H	-3.10410528628533	-3.15903816777007	3.66473422827115
H	-1.28004156786782	-3.43041109088887	3.43419817663311

I7-GaH 2

Electronic energy (r²SCAN-3c): -30616.142447952523 E_h
Electronic energy (ωB97X-D4rev/def2-mTZVPP): -30624.312919846496 E_h
G_{corr} (673.15 K, 1 atm): 0.39870 E_h
Number of atoms: 246

Cartesian coordinates (Å)

O	1.48307287784168	2.89752436510693	1.02690779612281
O	-1.94408702619575	6.77296109006133	1.42763679314957
O	5.26789481753332	2.07918363829151	0.80365571476228
O	3.60502341120715	3.88122698885407	-0.15352529875523
O	6.09238177032789	4.57678574408313	0.41910844593396
O	1.45209350964279	3.28851283146934	-1.61380157312435
O	1.37029525349753	5.32841181806164	0.02927640243355
O	-1.71070473520748	6.88921238115602	-1.19101332080500
O	0.32451313327950	7.70263034560415	0.40743782939112
O	2.93361239196631	7.47939425042555	-0.04593564289349
Si	4.84535495480221	3.64005782054591	0.87005568974027
Si	1.98624091082112	3.84557006921077	-0.18714420947131
Si	-1.29388520703221	7.62949480016028	0.20291544230671
Si	1.64918698825211	6.79393002825945	0.66083517940358
O	8.95261017804776	-3.83502787965320	2.22539014663016
O	1.99778274925700	6.65122077578574	2.24422388114228
O	7.14508892493438	-2.28015880227830	-0.92363843608659
O	2.50281431985172	4.74701605646069	4.08380077424632
O	6.74860367005239	-4.90479363500577	-0.77301290200684
O	0.04461561710623	5.11647274770876	3.14306009340089
O	5.85478316539625	-3.26830994993975	1.13616724579569
O	4.44123036623643	4.07348793316745	2.38788415012621
O	9.40135517848091	-5.01131397365174	-1.12783516567554
O	5.15271138375595	-0.24715579102022	2.01952226577133
O	7.73365533633733	4.37785235711115	-1.68810334799437
O	10.70354126701541	-3.18495070197440	0.34584073335222
O	5.43000622674920	5.63166123710489	-1.91133263029068
O	4.09100505705519	5.67279477195535	-4.17437151381000
O	8.76139615311499	-1.46129485573438	1.04444029272360
O	3.92195598860970	7.73623168457362	-2.52955578611167
O	1.17231261111051	9.52048110278554	-4.04649974008656
O	8.98096206311984	2.07189363350145	-1.88688963420898
O	6.94631133136178	0.18907534987767	0.10378088133518
O	2.27279228904207	9.55380033877924	-1.56400928757853
O	8.99940596575285	-0.54723482468378	-1.44239265163589
Si	1.43136782293613	5.86026137669011	3.55319044045397
Si	6.11616268758628	-3.44053336742139	-0.44974610826633
Si	3.63060043699045	3.62327222946400	3.72746744038777
Si	8.07918704892298	-5.83637636465429	-0.65677798845857
Si	10.87656087103735	-4.45883889620808	-0.67192903366291
Si	6.67079210641048	5.32659400089874	-0.90807289092279
Si	9.79495921972002	-2.60852044980162	1.56629511165436
Si	4.92356865613602	6.59433784035393	-3.11894250732014
Si	9.20506225849057	3.67336452717650	-1.66744499394335
Si	6.17077621455817	0.85353973396814	1.37377814528887
Si	1.72980113001111	10.45275131668262	-2.82525272337498
Si	9.80035596974404	0.68314047553805	-2.15348564213801
Si	7.95473208138933	-1.01632359136444	-0.30235630421721
Si	3.46894364661096	8.51785150620006	-1.17792579109733
O	2.97750696135844	-0.47474865425501	3.61996281388303

O	0.97294685941096	0.76448271455586	2.46882241854871
O	1.24156123715382	0.88703407070104	5.08019282232785
O	2.97421801611370	2.15843630513485	3.48503328906606
O	-0.41206761685751	1.18612289853096	0.28288263511125
O	-0.82117141098470	2.72732951998237	2.36283378556616
O	-3.75449161907763	4.98846035967642	0.56824987419345
O	-2.53494135167210	4.65731450084427	2.92508622144275
O	-1.18015574454453	3.43426990800443	4.84083255178984
Si	2.04731960860536	0.84064174860967	3.67851043603537
Si	0.30622672032273	1.90556878181356	1.53767621406720
Si	-3.13351143272671	5.74660383024229	1.86588631571403
Si	-1.10989161706748	3.99491574681234	3.32917386862799
O	8.28992387096448	-6.04377578981292	3.49653561770531
O	-3.19857978233974	-3.06411536816567	6.58063499006948
O	6.68145265999845	-7.84682635440467	2.44469551661440
O	-1.31012755135756	-1.56758068160256	7.69042763185947
O	8.25615139794140	-6.34228628158404	0.87211023438926
O	-5.42622217488836	-3.48235812117207	5.24490184332689
O	6.65247558050689	-3.92629009563140	3.57171874209602
O	-5.13210036821687	-1.21853584958328	6.58919965077381
O	-8.67532831844905	0.34221358391992	5.57840803408506
O	4.47668986376724	-2.51362630749695	3.25116153417672
O	-6.44723925929901	1.04726308205476	6.95136374214302
O	4.49175573501954	-5.06573150563271	2.60526206251476
O	-3.88623830229366	0.85867285726409	7.58022184510927
O	-2.25384490459864	2.85067694610282	7.14811069144266
O	0.39880567963632	2.65917830747564	6.86354131468313
O	4.25466584425882	-7.40010214174031	1.37476433252981
O	-1.50966000629796	0.86533474238306	8.78610691273331
O	0.72014650605739	0.08424086503145	7.53040695601195
Si	8.12650194432154	-7.12404705513143	2.28703334237782
Si	-2.12770929611718	-2.97348190332987	7.81769125861155
Si	8.20086648658870	-4.41747438936980	3.55223511908684
Si	-4.80740227375538	-2.81388647784629	6.58553424003670
Si	4.52774869511204	-0.89609264931285	3.37855232088848
Si	-8.08327371648613	1.12868517152245	6.87929841698333
Si	5.36141975292896	-3.70582709007155	2.61411347386879
Si	-5.30035914387940	0.07046268269467	7.56778684833129
Si	-0.93737067016256	3.48855937107792	6.44316211192996
Si	3.44329915857119	-6.10012217348468	1.92524759026251
Si	-2.74952589788168	1.78682746037418	8.27093159353923
Si	1.29618099446695	1.31899877624542	6.64663350370453
Si	5.41683566037137	-8.50893979108178	1.64883082810156
Si	-0.51570841300679	-0.38592888904762	8.47456413349334
O	1.79899007144488	4.26912617642691	-4.06008031424483
O	-0.06842257670931	5.27016314315474	-2.46442523118406
O	-0.61723511088478	7.68377027586892	-3.46356742071808
Si	0.70080027442230	3.91732958821416	-2.91555625791654
Si	-1.20409190840148	6.40686984852267	-2.65446478008473
O	1.24901435403874	-1.72907145205442	-4.50201282909165
O	-3.98317978887587	3.94463692003575	-4.55000211200768
O	3.34196543875901	-2.95879814246488	-3.48269696106624
O	-2.44883961244037	5.81920417966314	-3.50546582141314
O	-3.82002304029269	4.16606875269686	-1.91973190417040
O	1.33476816572200	-4.41054483895016	-4.41644689178321
O	-4.66759423830992	1.77430510424137	-5.91891219104392
O	4.72039327267250	-3.30736361746780	-1.25773313167230
O	-2.56056232889101	1.70570451133154	-4.37587071120966

O	-3.17064601297639	-0.36757902713357	-6.53569606686857
O	-1.11179943642933	-1.79557496016672	-5.65857848137981
O	-0.33265844026009	0.32754591050330	-4.20750256239286
O	-0.37221650787168	2.85380078976099	-3.48517413956250
O	1.78047278601613	6.92599348085690	-4.27161901789439
Si	-3.84027171135218	5.00036889073091	-3.31641234062644
Si	2.19316955903207	-3.04354639176026	-4.63995197572399
Si	-4.05331799567068	2.31977321751195	-4.52835092472455
Si	4.00864815281499	-4.07653889184389	-2.50764821895286
Si	-4.32501831436065	0.66675598812690	-7.07063529175765
Si	-0.30360985025768	-1.29290464409775	-4.34553741403719
Si	-1.22535360695554	1.47665485926171	-3.49941838152578
Si	2.55361668917308	5.56467041623047	-4.71058390505624
Si	0.62555084319121	8.05148550556682	-4.45437573203596
O	-2.79746173389311	-5.59287797009060	-7.11469133464375
O	-4.52315665364927	-4.33154251696051	-5.53990101755336
O	-2.91301242134469	-2.92985521463303	-7.22198194007946
O	-2.84044692948536	0.13112607369828	0.17998078304025
O	-2.48867491977660	2.76696966350291	-0.14270572404655
O	-1.66587311312668	1.00871147299355	-2.00445954931775
O	-5.13186749581242	2.80530010866794	-0.00808796843843
Si	-3.76630199529042	-4.29471203543511	-6.97820101803073
Si	-1.86012894860214	1.29691511966626	-0.42830024162357
Si	-3.81763596258253	3.68060971697996	-0.38052959754782
O	-5.01911944220825	1.90698004058230	-3.29250829435511
O	2.81573464899354	-5.05177859784215	-1.98247841025775
O	-5.61211511174845	-2.49861103912050	2.82403098091697
O	-7.38721726691221	-1.79051152232421	4.67686978601662
O	-0.25168131053302	-6.30276948900015	-3.40289082786298
O	-8.01014473073175	0.18979907886008	3.00878028718656
O	-0.39107154861317	-5.57347825017503	-5.96311240444993
O	-0.95701633832509	-1.92780634282418	-3.01242774190012
O	-3.48658067829105	-6.50669729511249	0.73143845490697
O	-0.97383307024397	-4.26992881900084	-1.86070517203182
O	-6.59539957413468	1.03514238617763	-1.38363528745532
O	0.62647862223272	-6.08292671052538	-0.87259754129862
O	-8.34926017068395	-0.63056225576714	0.48905383409395
O	-7.36533010186230	1.78262041014137	1.02179072233908
O	-1.87324654301911	-6.75058014940050	-1.40568582699863
O	-6.54118881020314	-0.21167755472251	-3.70532370859351
O	2.70748915290676	-5.41932914509065	0.64069502141170
O	-9.07239555732346	-2.65741101900377	-1.03848706807948
O	-4.46223140057121	-0.38990461263625	-2.08841481015385
O	-7.02109936160973	-2.92890383757461	0.61525775404267
O	-8.04180101071649	-2.38285038438734	-3.48263599652849
O	-5.38620682325341	-2.61109607374843	-3.74055572303151
O	-7.26484231162144	-4.36666970446226	-1.88771476924537
O	-5.00746526292502	-5.15297930961401	-3.07592003558480
O	-2.90064825847010	-3.62413200350340	-3.60357841291302
O	-5.69536118934757	-6.15290431967112	-0.71794241044650
O	-2.77221707785731	-2.50972675605135	-1.17258452025062
O	-4.76529027836891	-4.21277435529596	0.91545081579002
O	-4.69927663127304	-1.53469407855993	0.65794994283518
Si	-6.47907923985596	-3.01233618024060	4.10581058522436
Si	0.60025606804810	-5.83177990311829	-4.70694314883582
Si	-8.49715016874245	-0.65322369474786	4.31610251921908
Si	-1.24399302573884	-6.06961226695010	-7.25975789618734
Si	-6.64347174623451	2.28860186106508	-0.35318980627185

Si	-2.43866949610539	-7.48839126022827	-0.05786230559141
Si	-0.62076315929775	-5.84091145465789	-1.89006411995605
Si	-8.42901254746570	0.64597663711196	1.49792829850144
Si	-5.62946902186555	0.56223703684400	-2.60648568557416
Si	2.24946671089535	-6.02052055824260	-0.81052835059070
Si	-8.48836565147723	-2.24482284005470	0.41825602521901
Si	-6.70818476154890	-1.75521938784866	-4.17506483907310
Si	-8.52107840973612	-3.44520577849971	-2.35553487669882
Si	-4.44882261550393	-3.91866133203258	-3.97615994389481
Si	-6.26063527467594	-5.61864732633311	-2.14594956617201
Si	-1.92966653063551	-3.08057693259988	-2.40199017877523
Si	-4.95839205553133	-5.82603863082725	0.69943083858009
Al	-3.69748943327349	-1.14081798585035	-0.77009647173067
Si	-5.52819333552248	-2.82208874263960	1.23188350486318
Si	-2.11228331553998	-1.54968947837376	-6.91094182086258
H	11.67374700174548	-5.53703599655583	-0.03807399958472
H	9.19348301331734	-8.10585800847765	2.36346300490893
H	-3.88155000262408	1.39981099848830	-8.25725799507086
H	-5.54661900082106	-0.07985099807717	-7.37196300066531
H	-6.93206199579255	-1.73335099462338	-5.60666698495488
H	-9.61083499295927	-4.23371599589454	-2.91527899653116
H	-4.80806200015883	-4.38616399944418	-7.98466800430987
H	-1.25558100054093	-7.52827099935461	-7.35483799938795
H	-8.46603499878260	2.53218799427736	6.77163500066992
H	-4.20950399396624	6.50518799557309	2.49497299552810
H	-1.81143200109631	9.00591900023668	0.20164700153234
H	0.67612802102143	11.32783097782496	-2.29765501091790
H	-4.95337000810518	5.93157200678898	-3.34598500334689
H	-7.43212798919808	3.36467298439412	-0.94325499371189
H	-9.81433501323107	1.14100200527841	1.45030699738372
H	-3.26082299830902	2.50672899915633	9.43327000165914
H	-0.74504100053489	4.85494999609173	6.90563299934648
H	1.17176499718239	6.84606699822092	4.60638300039490
H	0.01383699839320	-0.85268899946011	9.75320599813184
H	2.65630400040134	1.61378700142544	7.09828799969637
H	4.53076900602123	3.47379799678473	4.86924400547187
H	1.53720800330863	-6.93798300023292	-4.97365699720351
H	4.63541500040262	9.21535600353157	-0.64625199801623
H	-8.67488799767080	0.53574600577375	8.08886499242276
H	-5.67875099940397	-0.35844199600812	8.92691799996614
H	-5.40516099835397	-3.45826599677637	7.74596999144650
H	-2.86007300305483	-3.03986699529100	9.10083200638248
H	-1.22700999103487	-4.11162700348956	7.70400899605030
H	2.57413299915970	5.42807899996174	-6.16363200335348
H	0.22482900336191	8.10275900205467	-5.85603198948636
H	2.79507597144444	11.25689298215873	-3.42192598502070
H	6.06067099865308	7.26917499917210	-3.76691099882107
H	10.01064999943551	4.23779399951022	-2.76113100101286
H	9.87926500001650	0.42371400116251	-3.59460900139716
H	11.16639499720707	0.78744100208632	-1.60472500095880
H	9.89847199672043	3.90341200134375	-0.38688400269818
H	-1.32399899442145	-1.19594399628636	-8.10330100718711
H	-0.61662900059610	-5.49814399499317	-8.46011600068953
H	2.84818300149793	-3.04332199966495	-5.95038400091960
H	-7.31517900304674	-4.15812801235465	3.75060699872589
H	-9.78249998082842	-1.30915798898553	4.07997000065243
H	-9.40319700030866	-2.75383199755602	1.45013700351380
H	-5.76898900476241	-6.41021300268086	1.77986100707105

H	-3.08084500140141	-8.75082999889927	-0.46797100380522
H	-7.02180600746795	-6.69287800880091	-2.78419800536130
H	2.78359001631228	-7.36886303260701	-0.99247301626546
H	4.89691601058471	-9.57737998559233	2.49409398521348
H	5.82084502032651	-9.04713702163993	0.34374498216975
H	-1.34437699588981	-7.77605499652660	0.87467400548702
H	10.66950900299051	-2.02029699540296	2.56380700193491
H	8.82314100453392	-3.95574099711320	4.78387700198103
H	11.59079999892575	-3.97453400146108	-1.85888000183166
H	5.33815600322995	-0.46451100434889	4.51995500141574
H	7.15219499870013	1.34004700112001	2.37234699461615
H	7.32251200089549	6.57841100612915	-0.51330699743526
H	5.00684299222455	-4.86484799002355	-3.24385799482797
H	7.90214799968307	-7.00114299703587	-1.50659200251840
H	2.44258291626503	-6.49974694547806	2.91143203335009
Ga	-3.73407277744203	-0.24879897911432	1.97014660595251
H	-4.74133403059100	0.88001329257239	2.26371783913975
C	-2.42513353985036	-1.25851258735363	2.99030643521903
H	-2.60662220967702	-1.44258397499205	4.05054509295569
C	-1.29724267333528	-1.72806251249612	2.45322053675280
H	-1.04225670591954	-1.58172783259324	1.40488141028964
H	-0.56598638015859	-2.28184203302397	3.04179749382552

I8-GaH 2

Electronic energy (r^{2}SCAN-3c): -30694.703470338805 E_h
Electronic energy (ωB97X-D4rev/def2-mTZVPP): -30702.972522008131 E_h
G_corr (673.15 K, 1 atm): 0.43882 E_h
Number of atoms: 252

Cartesian coordinates (Å)

O	1.47983214180747	2.86949680826319	1.01330691627817
O	-1.94420061349411	6.78138886609309	1.43146025657858
O	5.26730098910875	2.07722322336778	0.80306385782625
O	3.59998886770205	3.87554674508185	-0.15245529396856
O	6.08978505914398	4.57512202183949	0.41501295088267
O	1.44597058025865	3.28900761750299	-1.61648681098197
O	1.36573387451218	5.31706508196508	0.05212470769036
O	-1.70570346933941	6.88919194752156	-1.18696173874899
O	0.32937700829319	7.70020770483678	0.41109337078744
O	2.93433661100126	7.46454063687780	-0.06740047276340
Si	4.84412726649600	3.63787748538554	0.86737767151677
Si	1.98102872820747	3.83693169235141	-0.18694903455660
Si	-1.28927456925896	7.63157514839240	0.20519791696443
Si	1.65540105591140	6.79161358462714	0.65991738646584
O	8.95162397296275	-3.83420817469460	2.22546643699113
O	2.02395809495953	6.67328985592190	2.24234164559820
O	7.14487722676988	-2.27986911991327	-0.92312145062584
O	2.51014894964509	4.75811235692443	4.08066044330334
O	6.74882501392910	-4.90452170503186	-0.77192285584333
O	0.06701982451873	5.13928912199496	3.09660345153075
O	5.85384064974263	-3.26777776467369	1.13630311992166
O	4.44446259953745	4.07302199777870	2.38628850463119
O	9.40133534373586	-5.01117890216489	-1.12825553916194
O	5.15865565895666	-0.25010256247482	2.01745282094733
O	7.73293976914949	4.37903558575307	-1.68997058919091
O	10.70341195123820	-3.18528558601712	0.34602524583446

O	5.42723623398945	5.63614898907365	-1.91046330672419
O	4.09104919467584	5.67282174842659	-4.17489975478532
O	8.76203533807958	-1.46072496379583	1.04401220563536
O	3.92682730815676	7.74612655767744	-2.54039470169544
O	1.17305668774247	9.52123106489975	-4.04742530130063
O	8.98064105013813	2.07193732939683	-1.88641400143395
O	6.94981458240120	0.19136256613935	0.10146986984366
O	2.27305512605323	9.55462080758149	-1.56418613642261
O	9.00071838824643	-0.54838186089882	-1.44367116559216
Si	1.44723032028006	5.87605353021652	3.54305619654806
Si	6.11560935626987	-3.44035967152206	-0.44970333392345
Si	3.63191567202476	3.62799972448186	3.72567128677525
Si	8.07926134875933	-5.83629381309476	-0.65680966396546
Si	10.87650129360440	-4.45897520075808	-0.67204962485754
Si	6.66960266568949	5.32761751704534	-0.91003806807759
Si	9.79492274056419	-2.60844495290554	1.56626268034153
Si	4.92364034530412	6.59619284684787	-3.12169470968928
Si	9.20441620504977	3.67369076076229	-1.66767185298401
Si	6.17348305873246	0.85332051779816	1.37174310907725
Si	1.72969184405729	10.45321785354243	-2.82574506615551
Si	9.80051314374030	0.68335240871704	-2.15371805691537
Si	7.95601170086707	-1.01650760382854	-0.30340137453049
Si	3.47058197527365	8.51798171603756	-1.18467703016121
O	2.98128193336328	-0.46632243897794	3.61080424718954
O	0.94852989395278	0.76284668438793	2.49205777103454
O	1.25036992567427	0.88521873532761	5.08664858559769
O	2.96218665763480	2.16917019555557	3.47784215366755
O	-0.44268270133771	1.15447156134745	0.31757569462213
O	-0.81728279040094	2.74238443717755	2.36833948570168
O	-3.73888546521727	4.97990532765590	0.57546094665572
O	-2.52100132462224	4.67407403049414	2.94010406675737
O	-1.11825021685724	3.48443996434657	4.84152165918614
Si	2.04844545467175	0.84501715891970	3.68076010960210
Si	0.29463076461718	1.89955715844560	1.54178811957583
Si	-3.12847564842225	5.75048752668103	1.86933496284811
Si	-1.08336622742608	4.02395668059262	3.32337304916904
O	8.28928189127025	-6.04333737339421	3.49629365757742
O	-3.19861937026374	-3.06547511098649	6.58175831032521
O	6.68157594900259	-7.84669009143488	2.44432260045699
O	-1.31449976732379	-1.56595549844429	7.69480577000716
O	8.25735743747512	-6.34234382357235	0.87197028429184
O	-5.41321066140650	-3.46426715025139	5.22688958542860
O	6.65164918993425	-3.92571915640920	3.57187585593394
O	-5.12453734900519	-1.21177273817582	6.59271385675789
O	-8.67133487193121	0.33100518311773	5.58592136769861
O	4.47588050840505	-2.51289111542464	3.25148978470341
O	-6.44381748041022	1.04940140913290	6.95024841127931
O	4.49072940658809	-5.06532607194475	2.60543193136797
O	-3.89174945146023	0.85941424765506	7.62417419861016
O	-2.26083059048057	2.88285033150265	7.15889950158318
O	0.38783356137193	2.64254332683073	6.87842159332505
O	4.25474649701287	-7.39993534307288	1.37511081544233
O	-1.49310995923416	0.88054456815155	8.76861245583419
O	0.73023077951470	0.06756144659863	7.52911685688338
Si	8.12660606473651	-7.12392312209876	2.28685754989301
Si	-2.12781011723542	-2.97413134398310	7.81911600377535
Si	8.20013618944704	-4.41706752816748	3.55224954935303
Si	-4.80653329292350	-2.80923764093315	6.58194463053814

Si	4.53067732361662	-0.89566672045037	3.37608312645478
Si	-8.08154929901190	1.12815701339541	6.88056799739049
Si	5.36047886030379	-3.70570607647879	2.61404717407648
Si	-5.30032035408615	0.07328816643275	7.57757138649005
Si	-0.93293635958500	3.49310813282156	6.45117206227509
Si	3.44268414298346	-6.09996839540305	1.92462010713509
Si	-2.74287438411042	1.79949106261059	8.27032548399682
Si	1.29651050719343	1.31285680674864	6.65487509926077
Si	5.41674992044889	-8.50886289476766	1.64876470359521
Si	-0.51329009254575	-0.38424827857306	8.47314582080356
O	1.80011128893578	4.26950413241137	-4.06083607182470
O	-0.06596479740723	5.27219814233488	-2.46441586960418
O	-0.61593681271995	7.68601831959838	-3.46143248093191
Si	0.69772192404605	3.91762255769218	-2.92054102450114
Si	-1.20204425716744	6.40893317503952	-2.65194714886070
O	1.25183579365377	-1.72491083185573	-4.50359239891588
O	-3.97429148275355	3.95177839917384	-4.55279170228096
O	3.34189009506350	-2.95848781869755	-3.48302634528744
O	-2.44910299399326	5.82309319265141	-3.49923973194839
O	-3.82513192580025	4.16671196261663	-1.91684829373626
O	1.33331657327410	-4.40885659433926	-4.41573433114513
O	-4.66244188876068	1.78250186244570	-5.92714156295784
O	4.72047375554242	-3.30722285846282	-1.25820013375095
O	-2.57601747858748	1.70132076065150	-4.35540860705736
O	-3.16848704536981	-0.36528910117408	-6.53427015654393
O	-1.10442408410241	-1.79834393371488	-5.66822308366982
O	-0.33740216981008	0.33177338647131	-4.22517050672179
O	-0.37517336702020	2.85888885971464	-3.49904135936738
O	1.78059636928486	6.92641101792287	-4.27157487030474
Si	-3.83953661743016	5.00189753191044	-3.31371967400081
Si	2.19250797328055	-3.04210288718553	-4.64018206403937
Si	-4.06052305344710	2.32781951874486	-4.53145697473917
Si	4.00834688845466	-4.07631808125149	-2.50831235575972
Si	-4.32366870526579	0.66701927821882	-7.07166915705185
Si	-0.30227108906500	-1.28973139522067	-4.35410105359916
Si	-1.22439902923957	1.47745170061681	-3.50288293441010
Si	2.55363766165788	5.56515278818614	-4.71111960797128
Si	0.62582037450905	8.05210149886945	-4.45431090411276
O	-2.79712882779359	-5.59114074482640	-7.11004799730155
O	-4.53930946668070	-4.33584328866362	-5.54458197877151
O	-2.91958389860796	-2.92806493854537	-7.21740542100216
O	-2.87408142060862	0.12176106775426	0.14322508977556
O	-2.48536185565131	2.75802035854012	-0.15241670431630
O	-1.63044852984532	1.00535535085730	-2.00279749735006
O	-5.13028973769986	2.80821578738018	0.00234283092490
Si	-3.76988587160364	-4.29535206424216	-6.97659135992659
Si	-1.87318675461101	1.27717107757806	-0.43091895078615
Si	-3.81245708787689	3.67514373920154	-0.37870240070085
O	-5.05704205927148	1.93054457924955	-3.31452439740060
O	2.81656721301006	-5.05249147084383	-1.98302465254696
O	-5.60139962705990	-2.51583154118053	2.78789495767061
O	-7.37175001687933	-1.78086605633340	4.64892337873404
O	-0.24986805856160	-6.30677423031679	-3.40345194122716
O	-8.01847456582509	0.21153798287571	3.01556930263918
O	-0.38903517702152	-5.57364320214632	-5.96397554417435
O	-0.95940217477379	-1.92657660292486	-3.02471847463750
O	-3.48865924601630	-6.52239388126770	0.73643373721709
O	-0.97455310101611	-4.26821499723467	-1.87341584995092

O	-6.58601726969946	1.03366844043808	-1.38135761914929
O	0.62524407396496	-6.07957291359303	-0.87369488048235
O	-8.33108437469949	-0.63984101161865	0.50089808210609
O	-7.36366427584711	1.78289502851852	1.02021312243748
O	-1.87398509494958	-6.74719244886128	-1.40505884228881
O	-6.56175406871358	-0.21000334239891	-3.70612055211793
O	2.70703816609743	-5.41979943976356	0.64021159617267
O	-9.07794985249780	-2.64698967914602	-1.04804992084788
O	-4.44803688664847	-0.36565231776275	-2.14173396198999
O	-7.05592897776058	-2.98013051994583	0.62001836201913
O	-8.03596070115262	-2.39567338452464	-3.48782263221999
O	-5.37875097972993	-2.60451786505360	-3.74295657660512
O	-7.26814425841719	-4.37258903918720	-1.87836245661798
O	-5.00791105779487	-5.14524017188648	-3.07132990692084
O	-2.89214555212110	-3.63615362548806	-3.63409489162845
O	-5.69591824525338	-6.17953794369109	-0.72699015826866
O	-2.80021839195125	-2.51968919806462	-1.21381874439627
O	-4.77309573960716	-4.23701064843248	0.89184399015277
O	-4.74946491673440	-1.57812052340164	0.57340058131888
Si	-6.46976808579859	-3.00829938313187	4.08216864461064
Si	0.60087445075078	-5.83147873929433	-4.70685282046709
Si	-8.49614522421944	-0.65004881219681	4.31189399275939
Si	-1.24426210420859	-6.06906658384328	-7.25904445749297
Si	-6.63880890917574	2.28900908702205	-0.35339479873402
Si	-2.43569442770011	-7.49141691537064	-0.06001949817806
Si	-0.62131669070453	-5.83845480972874	-1.89290333329552
Si	-8.42686007752417	0.64711551263595	1.49648949236144
Si	-5.63839143018881	0.57482159384456	-2.62443657047755
Si	2.24824476937664	-6.02018796206923	-0.81136505549329
Si	-8.49817106463569	-2.25099036595794	0.41451353632105
Si	-6.70663825645562	-1.75575162862961	-4.17647712246821
Si	-8.52013683034648	-3.44850812884048	-2.35557250812429
Si	-4.44864626376594	-3.91826143216814	-3.98241805039167
Si	-6.26080630397122	-5.62132548541718	-2.14674715608053
Si	-1.93264515401438	-3.08442871576681	-2.42443022594848
Si	-4.96148725784735	-5.84807710899124	0.68965115183397
Al	-3.71971092937030	-1.14540018464024	-0.81758226118279
Si	-5.54942204092355	-2.85320890151175	1.18879056142921
Si	-2.11324729496865	-1.54994379128403	-6.91230582200565
H	11.67374698604971	-5.53703600328092	-0.03807396717355
H	9.19348298648974	-8.10585797936303	2.36346299623163
H	-3.88154999810603	1.39981100011986	-8.25725803737360
H	-5.54661900508826	-0.07985099869763	-7.37196299573394
H	-6.93206200229343	-1.73335100703551	-5.60666699508683
H	-9.61083499796287	-4.23371599307411	-2.91527898564559
H	-4.80806197562378	-4.38616400095269	-7.98466797912257
H	-1.25558100606697	-7.52827098277180	-7.35483798954376
H	-8.46603498960435	2.53218799019154	6.77163499989274
H	-4.20950399329586	6.50518798705566	2.49497299818382
H	-1.81143198930609	9.00591898029160	0.20164700743521
H	0.67612800530623	11.32783096722423	-2.29765500552706
H	-4.95337005702034	5.93157204879207	-3.34598502763635
H	-7.43212795579918	3.36467295522103	-0.94325496113222
H	-9.81433504248577	1.14100201043594	1.45030700017233
H	-3.26082299976676	2.50672899931816	9.43327002489028
H	-0.74504100411777	4.85495001776081	6.90563301867273
H	1.17176499688029	6.84606698305967	4.60638300299592
H	0.01383701228995	-0.85268901935960	9.75320604081761

H	2.65630398378381	1.61378699471789	7.09828799343759
H	4.53076899826483	3.47379800317188	4.86924400598190
H	1.53720799999276	-6.93798300486260	-4.97365699830789
H	4.63541497452779	9.21535598559247	-0.64625200363112
H	-8.67488797811004	0.53574601781061	8.08886496079366
H	-5.67875097217064	-0.35844195931615	8.92691788560080
H	-5.40516098987687	-3.45826599517700	7.74596996733807
H	-2.86007297275192	-3.03986703916988	9.10083199019546
H	-1.22701003544641	-4.11162696110119	7.70400902803381
H	2.57413299890775	5.42807899867195	-6.16363197232642
H	0.22482900672444	8.10275900075983	-5.85603196816759
H	2.79507598705899	11.25689299037109	-3.42192599431788
H	6.06067101130426	7.26917500506186	-3.76691101038635
H	10.01064999875077	4.23779399887012	-2.76113100014905
H	9.87926499251316	0.42371400049985	-3.59460899020688
H	11.16639500332474	0.78744100692496	-1.60472499997403
H	9.89847199211426	3.90341200106450	-0.38688400760036
H	-1.32399901005525	-1.19594400476721	-8.10330099467922
H	-0.61662900159491	-5.49814399732907	-8.46011600005545
H	2.84818300522584	-3.04332200141178	-5.95038398811006
H	-7.31517898651008	-4.15812798787302	3.75060702836334
H	-9.78249998812381	-1.30915799842100	4.07996999687559
H	-9.40319698723830	-2.75383199048161	1.45013698454276
H	-5.76898900644180	-6.41021301179234	1.77986100797557
H	-3.08084501040744	-8.75083000377936	-0.46797101234234
H	-7.02180600329355	-6.69287799744999	-2.78419799993924
H	2.78359000035110	-7.36886300101666	-0.99247300247734
H	4.89691599438317	-9.57738001075913	2.49409400412803
H	5.82084499573003	-9.04713700183645	0.34374500028835
H	-1.34437698282667	-7.77605499845215	0.87467400753746
H	10.66950896702040	-2.02029702658331	2.56380696856943
H	8.82314100999015	-3.95574098615999	4.78387703650275
H	11.59080001938566	-3.97453397387233	-1.85888004436861
H	5.33815598699877	-0.46451101774492	4.51995497516301
H	7.15219500798286	1.34004700750087	2.37234700337183
H	7.32251199902409	6.57841100788444	-0.51330699863746
H	5.00684298695237	-4.86484798820323	-3.24385798619988
H	7.90214800531463	-7.00114300288905	-1.50659200278992
H	2.44258300717615	-6.49974698481218	2.91143198919095
Ga	-3.66685502221786	-0.45497299086408	1.96290008734518
H	-4.71589707709937	0.61062545225754	2.31013968454460
C	-2.20455186568636	-1.61443507127518	2.52796713172387
H	-1.20945628225493	-1.23468170553599	2.28121880458683
C	-2.28180878632320	-2.80884821631658	3.11367447814931
H	-3.22387845084381	-3.26928073609819	3.39864208050662
H	-1.38583411928774	-3.38517639743734	3.34205071946231
C	-2.30280258686715	0.46758824105291	4.80396976611932
C	-3.35542672543607	1.24002816482233	4.56011534235066
H	-1.30679237394155	0.73420620144843	4.46117561219932
H	-2.39797684870580	-0.47099636307354	5.34203724609263
H	-3.27721627982167	2.17612311399142	4.01556703561481
H	-4.35370730740083	0.96832019262089	4.88992783693921

I9-GaH 2

Electronic energy (r^2SCAN-3c): -30694.749376280542 E_h
Electronic energy (ωB97X-D4rev/def2-mTZVPP): -30703.018227109613 E_h
G_{corr} (673.15 K, 1 atm): 0.48122 E_h
Number of atoms: 252

Cartesian coordinates (Å)

O	1.49085038364940	2.90216336436172	1.03130786374941
O	-1.94265757473841	6.77308236802025	1.42694764779016
O	5.26847639874849	2.07874352577054	0.80279185663605
O	3.60853607091709	3.88325125132829	-0.15541698667036
O	6.09452066788339	4.57690004452014	0.41998251726093
O	1.45219626050681	3.28909490113746	-1.61120331481453
O	1.37228033549107	5.32993345040235	0.02933569895314
O	-1.71190116925403	6.88860104723471	-1.19284355755151
O	0.32420661842009	7.70329909513721	0.40427108384310
O	2.93442407441281	7.48101457142927	-0.04515123658201
Si	4.84755818584613	3.63975063130421	0.86953356676164
Si	1.99012885563383	3.84831852035134	-0.18719202252567
Si	-1.29438784354462	7.62936493747384	0.20076096884664
Si	1.64920330765460	6.79615916335892	0.66001421265229
O	8.95237560022923	-3.83489951318613	2.22549802533862
O	1.99629595243486	6.65428767263922	2.24382732185659
O	7.14457510570112	-2.28039351242277	-0.92323658550057
O	2.50560950236112	4.75115662985569	4.08353719256118
O	6.74874217403762	-4.90483862371060	-0.77123850727425
O	0.04588500088893	5.11727683734449	3.14478729392003
O	5.85391642894248	-3.26694682651111	1.13687227835042
O	4.44127855088633	4.07138049209020	2.38743807221018
O	9.40123117372008	-5.01122318093568	-1.12834765918488
O	5.15375428462148	-0.24699267127129	2.01911067609925
O	7.73383373452149	4.37762194703555	-1.68849142955992
O	10.70346686973556	-3.18521316636582	0.34591763173533
O	5.43011187227568	5.63156239694159	-1.91092660153641
O	4.09444128850374	5.67579320821119	-4.17744234502912
O	8.76178220758401	-1.46113222697394	1.04436157851526
O	3.92177399752370	7.73567088737583	-2.52922327224067
O	1.17138552002340	9.52070773657172	-4.04624666005353
O	8.98096827401092	2.07153013469694	-1.88662303918626
O	6.94753775629693	0.18946781588177	0.10309868507300
O	2.27286583609441	9.55399604967910	-1.56432727606090
O	8.99966859393779	-0.54812102602443	-1.44297472900956
Si	1.43261011167194	5.86246634825306	3.55293197518635
Si	6.11547267749387	-3.44078470845211	-0.44877963716182
Si	3.63028455088540	3.62439975608432	3.72760182049910
Si	8.07930172162771	-5.83650751093766	-0.65646533015878
Si	10.87636391666542	-4.45900439870028	-0.67205629933615
Si	6.67151570335123	5.32650235702817	-0.90822306849666
Si	9.79497070466360	-2.60880459493900	1.56639560353069
Si	4.92467629100547	6.59487692011828	-3.11854887458072
Si	9.20530099865634	3.67291268430945	-1.66752293361191
Si	6.17187010610369	0.85308534250210	1.37309776656170
Si	1.72960733111937	10.45297134257103	-2.82548057710450
Si	9.80039980460048	0.68266395343924	-2.15347123976474
Si	7.95509919370837	-1.01706176852224	-0.30264232868130
Si	3.46920070005902	8.51850028006881	-1.17830628246888
O	2.97661197179508	-0.47131681963473	3.61359153773355

O	0.96073762051135	0.77005255551372	2.47488320235070
O	1.23852782843080	0.87998623199101	5.08131461054343
O	2.96642823095268	2.16225991105517	3.48770862943737
O	-0.40548324433373	1.19678603716746	0.27743379350085
O	-0.82258961036879	2.73239455083243	2.35917255395500
O	-3.75466951284230	4.99081675119847	0.56837622277665
O	-2.53526204482866	4.66022867737221	2.92671528989549
O	-1.18067683267167	3.43115580494536	4.83917229877300
Si	2.04455972192245	0.84133321802472	3.67883253544017
Si	0.30903454755420	1.91440998559609	1.53467214722613
Si	-3.13347679679265	5.74837567461659	1.86539573625897
Si	-1.11025792309077	3.99826099708238	3.32933592851946
O	8.28866336277640	-6.04332007080782	3.49629504891213
O	-3.19780945861121	-3.06851088957250	6.58088685608634
O	6.68192041009995	-7.84746276604944	2.44416997119781
O	-1.31054505648272	-1.56757216783946	7.69005581883687
O	8.25803016351742	-6.34255278811143	0.87208187821486
O	-5.43257438329370	-3.49095663953921	5.25451215676457
O	6.65242993199565	-3.92404711802484	3.57213433624188
O	-5.12972195031006	-1.22025175116993	6.58927178865993
O	-8.67271319388894	0.34189236738648	5.57597718992254
O	4.47600642349119	-2.51286327256691	3.25194492355030
O	-6.44700471966294	1.04580909098284	6.95093876307545
O	4.49287755764460	-5.06607471554246	2.60639959321671
O	-3.88654688450157	0.85891687257689	7.57931530718324
O	-2.25310186656399	2.84949286920330	7.14733366407364
O	0.39969571682068	2.65888788085356	6.85996458397856
O	4.25500179358941	-7.39932896627820	1.37562210507683
O	-1.51000790813393	0.86530395878362	8.78604054323011
O	0.71997592421553	0.08439422918772	7.53232729133880
Si	8.12644494473647	-7.12411865150418	2.28700337156748
Si	-2.12796659932483	-2.97388408591973	7.81729714825185
Si	8.20014443991841	-4.41706212657710	3.55233441262666
Si	-4.80586935543832	-2.81626687525178	6.58687574386364
Si	4.52753824985083	-0.89582708943186	3.37760149204488
Si	-8.08309924404430	1.12802597384641	6.87826085246652
Si	5.36055117807635	-3.70553569772587	2.61471958999317
Si	-5.30009180751002	0.06881183334871	7.56705757610497
Si	-0.93738984356359	3.48813306531709	6.44243557454770
Si	3.44268974234051	-6.09911899369358	1.92595995433525
Si	-2.75021096763515	1.78622496660999	8.27082405770823
Si	1.29605831405708	1.31732443183149	6.64717215323598
Si	5.41630520846989	-8.50861015349859	1.64875223515713
Si	-0.51681226086702	-0.38668423161113	8.47515786709288
O	1.80672206293937	4.27155062856076	-4.05553841835621
O	-0.06725139501567	5.27053372122344	-2.46458680991333
O	-0.61758769761800	7.68278440572291	-3.46468587950175
Si	0.70516035960672	3.91933894649896	-2.91515849165537
Si	-1.20459751024392	6.40552163911611	-2.65623997905605
O	1.25069884395473	-1.72972413952281	-4.50214837290339
O	-3.98968631218083	3.94052374644506	-4.54749906583656
O	3.34247485651896	-2.95995062283318	-3.48290076686880
O	-2.44781257011236	5.81613876534489	-3.50876281163114
O	-3.81875981245492	4.16934745015562	-1.91998392742001
O	1.33371350461518	-4.41080884937957	-4.41703328638089
O	-4.66981174804194	1.76981838803535	-5.91456604819439
O	4.72024157919186	-3.30800083696304	-1.25755533532644
O	-2.54931488251671	1.71243660555698	-4.38898363770085

O	-3.17022769438927	-0.36771062582422	-6.53577378051835
O	-1.10988461294596	-1.79490629965305	-5.65995134697996
O	-0.32669649210649	0.32876609237849	-4.21105706963914
O	-0.36319970866824	2.85599395562215	-3.49172879400650
O	1.78155482493803	6.92713874408410	-4.27210072512359
Si	-3.84104102975357	4.99978724888448	-3.31795425026486
Si	2.19388358561237	-3.04488342639432	-4.63987941606818
Si	-4.04831923310511	2.31488470674798	-4.52687644777132
Si	4.00827760285160	-4.07767730520043	-2.50702432959376
Si	-4.32520705077544	0.66632138094862	-7.06997698543397
Si	-0.30103734168901	-1.29176832626300	-4.34753202439665
Si	-1.21837338931231	1.48067035217111	-3.50702998549352
Si	2.55648231337751	5.56684846478932	-4.71092504622555
Si	0.62558240954925	8.05157963007449	-4.45462855268809
O	-2.79728800867096	-5.59384300012347	-7.11920734593443
O	-4.51860372293736	-4.33933640567387	-5.53782306536959
O	-2.91400163529479	-2.93009011166177	-7.21944471283946
O	-2.83090283736025	0.14183217947725	0.17603494393220
O	-2.48472730054425	2.77421176202813	-0.14479173024791
O	-1.66351355684548	1.01831435412203	-2.01183692020827
O	-5.13177321915263	2.80972776684781	-0.01088254923052
Si	-3.76585831684967	-4.29641333698046	-6.97818152611447
Si	-1.85467389560047	1.30518599891364	-0.43494462857876
Si	-3.81698215853742	3.68358093858130	-0.38153732837266
O	-4.99585746461683	1.89036046916824	-3.28134000491272
O	2.81482612653426	-5.05257738726702	-1.98315461418566
O	-5.59845187391758	-2.51425268593703	2.83438420177642
O	-7.38403235845237	-1.79082707124845	4.67333507175112
O	-0.25164242372740	-6.30417437206108	-3.40195335684789
O	-8.00896728290605	0.18670722075775	3.00494966204613
O	-0.39304283910382	-5.57438764112309	-5.96176974100155
O	-0.95767178555465	-1.92346921847639	-3.01442456762211
O	-3.48872491466499	-6.50973689214096	0.73239955849998
O	-0.96530773669227	-4.26820034612922	-1.86275880252603
O	-6.59550379246425	1.03695308892841	-1.38323116139014
O	0.62758877641615	-6.08726496108802	-0.87181942159005
O	-8.36110804118241	-0.62284931416994	0.48223095532578
O	-7.36938254913063	1.78803931264691	1.02073840407007
O	-1.87491046173302	-6.74475231880832	-1.40405609180634
O	-6.53557609806878	-0.21413538169884	-3.70310438219860
O	2.71113788977795	-5.41801297421632	0.63963658900854
O	-9.06791403845113	-2.66158353758225	-1.03462745020560
O	-4.47456003499119	-0.41211478026090	-2.06705736391286
O	-7.00672216519999	-2.89688970855180	0.61663078640028
O	-8.03529438640052	-2.38825535302575	-3.47948151793881
O	-5.38130305958841	-2.61252283343386	-3.74588706561251
O	-7.26345739184322	-4.37159911548070	-1.87905108894678
O	-5.00525952689915	-5.15238050296315	-3.07131229116771
O	-2.89715547992525	-3.62637600479857	-3.60168708198861
O	-5.69630546206831	-6.17379322669875	-0.72319147140417
O	-2.76328871604630	-2.51391917308583	-1.16986946313726
O	-4.77997730207558	-4.22222786686597	0.89373999023110
O	-4.64150804139469	-1.55534518042176	0.69555084397347
Si	-6.47680369308072	-3.01766095779734	4.11082282698995
Si	0.59957266527759	-5.83254876759968	-4.70626813078939
Si	-8.49529692784648	-0.65454739837597	4.31381199996126
Si	-1.24352523216008	-6.07006733974211	-7.26009937841267
Si	-6.64316405594527	2.29038849252670	-0.35294451326340

Si	-2.43722396062113	-7.48841579563730	-0.05891003105974
Si	-0.61778400273093	-5.84053701627105	-1.88861020943502
Si	-8.43035740815930	0.64931958533405	1.49674729897636
Si	-5.62515076735999	0.55273172248744	-2.59776275694728
Si	2.25033628566726	-6.02060228087423	-0.80911934867357
Si	-8.48564736061026	-2.23814181251228	0.41997106877230
Si	-6.70486386322864	-1.75706839375483	-4.17500299250252
Si	-8.51801722869188	-3.44898299083874	-2.35209560250170
Si	-4.44508395494394	-3.92122484467794	-3.97541152217225
Si	-6.25979504817531	-5.62239545871756	-2.14590573049069
Si	-1.92333858565467	-3.08108393128215	-2.40334615573385
Si	-4.96455520177621	-5.83580032394867	0.69291696487011
Al	-3.68003590637799	-1.14191122906987	-0.75515846996674
Si	-5.51540915098047	-2.82451878538292	1.24224423913312
Si	-2.11213946608445	-1.55000359066563	-6.91100165990089
H	11.67374701229332	-5.53703601122023	-0.03807399941702
H	9.19348300761745	-8.10585800172211	2.36346299381767
H	-3.88155002266702	1.39981099559285	-8.25725794165238
H	-5.54661898816362	-0.07985100141701	-7.37196300625711
H	-6.93206200515211	-1.73335101990878	-5.60666714024770
H	-9.61083496757048	-4.23371598319133	-2.91527896608251
H	-4.80806205696297	-4.38616400881197	-7.98466804548395
H	-1.25558097849543	-7.52827105250332	-7.35483802017495
H	-8.46603500280006	2.53218808508277	6.77163494332435
H	-4.20950398940379	6.50518798373775	2.49497298209718
H	-1.81143199527289	9.00591898873196	0.20164699865587
H	0.67612811109515	11.32783134676527	-2.29765511542279
H	-4.95336994928274	5.93157195711766	-3.34598497107235
H	-7.43212801951050	3.36467302569331	-0.94325499548868
H	-9.81433500645767	1.14100199196614	1.45030699187772
H	-3.26082302443963	2.50672901793810	9.43327004362540
H	-0.74504100606900	4.85495000326091	6.90563298497944
H	1.17176498696804	6.84606699427653	4.60638298748429
H	0.01383700127926	-0.85268899971441	9.75320601106204
H	2.65630400757697	1.61378699366243	7.09828799604203
H	4.53076903893836	3.47379798742113	4.86924404500558
H	1.53720799553987	-6.93798299395909	-4.97365699343986
H	4.63541502513886	9.21535600040059	-0.64625199505009
H	-8.67488807109585	0.53574590385769	8.08886515188532
H	-5.67875097745882	-0.35844198318350	8.92691792824317
H	-5.40516103813798	-3.45826604341069	7.74597004732469
H	-2.86007302917210	-3.03986694980384	9.10083200710583
H	-1.22700997651360	-4.11162705513154	7.70400893342035
H	2.57413299914965	5.42807899137043	-6.16363197941460
H	0.22482900195889	8.10275900962685	-5.85603203630316
H	2.79507584251800	11.25689289250074	-3.42192590915479
H	6.06067101505342	7.26917500637724	-3.76691100455975
H	10.01065000338062	4.23779399802304	-2.76113099423610
H	9.87926498878875	0.42371399976392	-3.59460899110714
H	11.16639500703132	0.78744100195245	-1.60472500242972
H	9.89847200161767	3.90341200009155	-0.38688400963363
H	-1.32399899777063	-1.19594400363582	-8.10330098674534
H	-0.61662900031533	-5.49814399787544	-8.46011600459514
H	2.84818298970111	-3.04332200021132	-5.95038398535718
H	-7.31517895228167	-4.15812796525099	3.75060703127124
H	-9.78249998599001	-1.30915800355856	4.07996999901086
H	-9.40319705054270	-2.75383202526145	1.45013706731166
H	-5.76898895900010	-6.41021298755947	1.77986095991345

H	-3.08084500884363	-8.75083001586481	-0.46797100768572
H	-7.02180600822488	-6.69287802595757	-2.78419800452113
H	2.78358999553406	-7.36886300082704	-0.99247300316393
H	4.89691599763096	-9.57738000288024	2.49409399102086
H	5.82084500319483	-9.04713700687666	0.34374500227544
H	-1.34437701283738	-7.77605498156109	0.87467402515454
H	10.66950900031396	-2.02029700668872	2.56380701050610
H	8.82314101531152	-3.95574100686682	4.78387702081226
H	11.59080000058520	-3.97453399955741	-1.85888000048822
H	5.33815601235004	-0.46451099554156	4.51995502080875
H	7.15219498780786	1.34004699788221	2.37234698833346
H	7.32251198464154	6.57841097803729	-0.51330700869481
H	5.00684303122049	-4.86484801313714	-3.24385801939332
H	7.90214799762228	-7.00114300728244	-1.50659200675601
H	2.44258299700670	-6.49974699864129	2.91143200736670
Ga	-3.75195623270592	-0.19419798222338	1.98124230287273
H	-4.85353389637105	0.87658311464054	2.15498454015431
C	-1.87848154357525	-3.16231316184672	2.29173598480886
H	-2.32954662143920	-3.23200733096843	1.30138719040601
C	-1.89200438046580	-4.23163845779944	3.08265496766621
H	-1.44278415754819	-4.20674024571634	4.07387246622731
H	-2.34952770664670	-5.16375953756696	2.76477197256282
C	-1.29217781492113	-1.83567039095863	2.67489224039250
C	-2.35209067473050	-0.84709344289189	3.19729203961250
H	-0.77060156166245	-1.40034067548842	1.81310644512813
H	-0.53583755868906	-1.98344762664197	3.45800975639705
H	-1.85584120964157	0.07413635956533	3.54277954037074
H	-2.85059152530055	-1.27104479523274	4.07883671947149

I10-GaH 2

Electronic energy (r^{2}SCAN-3c): -30773.315302235962 E_h
Electronic energy (ωB97X-D4rev/def2-mTZVPP): -30781.682620769789 E_h
G_corr (673.15 K, 1 atm): 0.49480 E_h
Number of atoms: 258

Cartesian coordinates (Å)

O	1.48961606258991	2.90433276131560	1.03227343878458
O	-1.94414898411607	6.77363133478222	1.43007481245569
O	5.26893160888992	2.07914065138566	0.80195860148290
O	3.60786710547714	3.88305952121498	-0.15540100795579
O	6.09405512287617	4.57737690071710	0.41975018869999
O	1.45131728747305	3.28855306652177	-1.61095490499709
O	1.37230753861327	5.33096248693277	0.02825782242327
O	-1.71095778096487	6.88810688481469	-1.19095966138556
O	0.32326431924941	7.70331705869499	0.40726330545090
O	2.93366899797329	7.48203529787929	-0.04446434435714
Si	4.84723044265366	3.63992482547850	0.86909882755730
Si	1.98936486750356	3.84899932434463	-0.18729797655360
Si	-1.29569881315870	7.62860779936754	0.20372524851977
Si	1.64866635861045	6.79660973323934	0.66073183576732
O	8.95222188104727	-3.83477179787225	2.22548881768303
O	1.99732243783454	6.65317664311543	2.24408569838082
O	7.14511177992371	-2.28093527671134	-0.92328192133142
O	2.50969207142366	4.75392592870620	4.08624648663788
O	6.74849853179060	-4.90515430618999	-0.77057744236284
O	0.05060217665729	5.11198416389120	3.14709739489025

O	5.85367778873998	-3.26638118324784	1.13686636938311
O	4.44172426667933	4.07127067959048	2.38722961527171
O	9.40112753425343	-5.01110827746462	-1.12831608514572
O	5.15226431620787	-0.24549150449429	2.01956021027819
O	7.73390643813943	4.37772963829724	-1.68839934395351
O	10.70342037206344	-3.18517476206272	0.34588840209993
O	5.43018446515352	5.63130531224129	-1.91137892726795
O	4.09464024528193	5.67626579290710	-4.17792449054845
O	8.76191214142890	-1.46098892894223	1.04436625759654
O	3.92156707257599	7.73536993067046	-2.52870088422757
O	1.17070354250566	9.52051595769526	-4.04601283458231
O	8.98101355849501	2.07163033409981	-1.88662379071366
O	6.94676743688311	0.18865913487082	0.10344656096746
O	2.27288874138977	9.55422696859446	-1.56439241643547
O	8.99928796761140	-0.54775059686008	-1.44288020245297
Si	1.43447330801059	5.86212005670659	3.55386538388065
Si	6.11553210302823	-3.44082081169145	-0.44873592103010
Si	3.63115546174375	3.62522812476750	3.72781680852427
Si	8.07931136992789	-5.83653983522719	-0.65640040508444
Si	10.87633765825824	-4.45899305220828	-0.67205807151909
Si	6.67141251704141	5.32661988310525	-0.90838831545716
Si	9.79497154817047	-2.60871637393620	1.56636680683110
Si	4.92459070888882	6.59503563157991	-3.11859762854348
Si	9.20530135450152	3.67304735129742	-1.66751326683882
Si	6.17150237095280	0.85340140858304	1.37309454791640
Si	1.72944766966484	10.45290457233404	-2.82558692785796
Si	9.80034763358738	0.68276268549877	-2.15347115102549
Si	7.95496871293263	-1.01719039631015	-0.30257172556822
Si	3.46897506016281	8.51862166180981	-1.17796828669815
O	2.97632993989027	-0.46984835764289	3.61599440361245
O	0.96105648425682	0.77131096911748	2.47497236205183
O	1.23693850771295	0.88260272346425	5.08170149967613
O	2.96527256487775	2.16391006144688	3.48820947193137
O	-0.40368258954462	1.19632136276501	0.27741885485669
O	-0.82482559995212	2.73141739320901	2.35926384034056
O	-3.74759020234641	4.99081581786553	0.56830274934714
O	-2.53158060757096	4.66474443015819	2.93342301501687
O	-1.18095126811449	3.42761454329167	4.84032641993405
Si	2.04402427649601	0.84273635409577	3.67958071815579
Si	0.30833120150652	1.91550683757036	1.53479877849039
Si	-3.13440797211592	5.74617383509029	1.86947472941407
Si	-1.10978271902864	3.99719023612247	3.33065075739910
O	8.28799988471551	-6.04304119937269	3.49627185580882
O	-3.19744341532268	-3.07105730138575	6.58062779068611
O	6.68200390449029	-7.84755229828090	2.44417681124297
O	-1.31109312343675	-1.56764822392659	7.68887054335204
O	8.25843943037609	-6.34289805567195	0.87200350110470
O	-5.43327586211307	-3.49045554754681	5.25381399165292
O	6.65216397289941	-3.92330248284502	3.57206637942565
O	-5.12556514496000	-1.22048425901775	6.58849467579419
O	-8.67416388851640	0.34212369124171	5.57618495103028
O	4.47535177616003	-2.51203270159659	3.25156248367047
O	-6.44792067777030	1.04156607565621	6.95033408347282
O	4.49253989449730	-5.06542139275647	2.60656345983926
O	-3.88883958046015	0.86228997706531	7.57590870068233
O	-2.25745127223216	2.85175088668434	7.14569023108634
O	0.39666357786743	2.65693880222659	6.86478129242221
O	4.25525797602424	-7.39904438853372	1.37578506065299

O	-1.51062347516478	0.86549257414794	8.78261608713625
O	0.72075154354068	0.08287388543283	7.53220409465310
Si	8.12646925763856	-7.12407041830172	2.28706804407951
Si	-2.12799579088874	-2.97428098397705	7.81725685129626
Si	8.19981849738929	-4.41673253568113	3.55230330199145
Si	-4.80536856795733	-2.81731793645627	6.58625001916483
Si	4.52683816612326	-0.89501021989575	3.37800000986035
Si	-8.08398051264854	1.13080128200039	6.87751618286626
Si	5.36015275948595	-3.70479031544363	2.61476484289559
Si	-5.30076245411474	0.06765334866853	7.56697205157552
Si	-0.93802614035255	3.48776612541800	6.44303073516054
Si	3.44280266276107	-6.09894481043519	1.92612042820087
Si	-2.75141174047628	1.78672865895882	8.26988954998410
Si	1.29556610550647	1.31734637265124	6.64807292813361
Si	5.41638763880246	-8.50860435053246	1.64878738046477
Si	-0.51682737075318	-0.38713281043998	8.47437462457653
O	1.80759103734657	4.27159445418567	-4.05498035076056
O	-0.06688989608596	5.27044346382941	-2.46424603071715
O	-0.61818958654407	7.68204580256946	-3.46442581827327
Si	0.70524984024521	3.91943947528009	-2.91533132544609
Si	-1.20477185127993	6.40526613171281	-2.65454985994635
O	1.25020599201049	-1.73064937902998	-4.50102940207667
O	-3.99021406323224	3.93938192644184	-4.54477882716305
O	3.34237807841896	-2.96034034943348	-3.48306787370107
O	-2.44823788335483	5.81512415580932	-3.50626125136441
O	-3.81909194178208	4.16773126864296	-1.91734520998862
O	1.33345467737909	-4.41164639735404	-4.41687222714001
O	-4.67007951694408	1.76900954291176	-5.91332275860356
O	4.72041990252866	-3.30799521845016	-1.25769477025187
O	-2.54988112219114	1.71108114347986	-4.38850745580492
O	-3.17055419670816	-0.36830932105890	-6.53604493205072
O	-1.10898537995603	-1.79516079841465	-5.66129282003400
O	-0.32559526742733	0.32944796897388	-4.21392080931336
O	-0.36306796492177	2.85697181508520	-3.49400528687516
O	1.78135646492032	6.92717858766371	-4.27197081014920
Si	-3.84140939019233	4.99871347822224	-3.31488830326457
Si	2.19350560726504	-3.04552031173226	-4.63980042483028
Si	-4.04893514027798	2.31388350989879	-4.52516421791929
Si	4.00830356336389	-4.07770393796133	-2.50701316782924
Si	-4.32527265074177	0.66643486797858	-7.06961343157838
Si	-0.30132040678688	-1.29122709746139	-4.34859626024490
Si	-1.21707661701735	1.48099847038218	-3.50878026228341
Si	2.55650731289065	5.56706100688459	-4.71090379066683
Si	0.62484802650617	8.05124663411174	-4.45429215038464
O	-2.79766760587136	-5.59446291978334	-7.11921859686532
O	-4.52090047720171	-4.34042134979502	-5.53911407339378
O	-2.91377600559838	-2.93083686729196	-7.21900778706872
O	-2.82118655056619	0.12982922173240	0.17148873111448
O	-2.48863588356407	2.76638289966420	-0.14355147276906
O	-1.65934289455205	1.01754256400776	-2.01348011341060
O	-5.13478725293472	2.81469375278981	-0.00770659210774
Si	-3.76614509116834	-4.29689183927429	-6.97841179285099
Si	-1.85235160910534	1.30095481033245	-0.43648248242824
Si	-3.81690635754867	3.68250442700877	-0.37780934325252
O	-4.99685083730402	1.88881876253898	-3.27980755035656
O	2.81472244677073	-5.05241887750047	-1.98290882804940
O	-5.60151011944424	-2.51096526613734	2.83279402802248
O	-7.38311271551229	-1.78905924681830	4.67332603689817

O	-0.24998683167763	-6.30909030550361	-3.40247467586652
O	-8.00606010525532	0.18904165167654	3.00671190004981
O	-0.39382907523308	-5.57437031002720	-5.96132244699569
O	-0.96113850239423	-1.92135354368605	-3.01611153134656
O	-3.48880277728428	-6.51195450659781	0.73323706246126
O	-0.96007539346544	-4.26917532461104	-1.87037256679899
O	-6.59239026712658	1.03544720889778	-1.37708769199012
O	0.62763409966106	-6.08783764271151	-0.87150748465296
O	-8.35752667648293	-0.62368643467562	0.48507468801504
O	-7.37269189135983	1.79264403816670	1.02470082949486
O	-1.87502550774892	-6.74352380033671	-1.40385519287141
O	-6.53713809489054	-0.21563252692356	-3.69813403181427
O	2.71111648079884	-5.41807599702813	0.63973283698221
O	-9.06641071390919	-2.65989063449410	-1.03514744882673
O	-4.47216472547395	-0.41362364348236	-2.06734421667870
O	-7.00618502963036	-2.90034363369665	0.61605261456229
O	-8.03518711616080	-2.39030071093348	-3.48112370161615
O	-5.38041696841923	-2.61285254281339	-3.74646691170768
O	-7.26355618752032	-4.37213533478064	-1.87886657897963
O	-5.00509459037137	-5.15256754386483	-3.07156676421292
O	-2.89596607518877	-3.62918591625337	-3.60647693142432
O	-5.69561013361655	-6.17375180932356	-0.72338649265397
O	-2.76388836619450	-2.52388984345314	-1.17253767971155
O	-4.77812538131923	-4.22481342505145	0.89701240088408
O	-4.63876322448504	-1.56247205451799	0.69072321383887
Si	-6.47723411635544	-3.01683956948961	4.10988504562102
Si	0.59951193892824	-5.83342630894876	-4.70661103191930
Si	-8.49594842629524	-0.65366074676136	4.31546786276413
Si	-1.24374046220718	-6.07010696828771	-7.26008402608869
Si	-6.64252042051771	2.29188326210353	-0.35016975299729
Si	-2.43681122119145	-7.48876440909396	-0.05929778831360
Si	-0.61635907367471	-5.84227702229585	-1.89029276092197
Si	-8.43070753267305	0.64918130706830	1.49754031671464
Si	-5.62441040558704	0.55137755715855	-2.59444452832923
Si	2.25048545536465	-6.02070340641936	-0.80904945781021
Si	-8.48407623841759	-2.23938552778393	0.42023998007319
Si	-6.70447217080734	-1.75739400569595	-4.17465589441363
Si	-8.51774780031977	-3.44928068222014	-2.35228494230576
Si	-4.44512367351586	-3.92221971068243	-3.97689355411215
Si	-6.25947711175221	-5.62257502748488	-2.14604896786930
Si	-1.92233738871325	-3.08424813259897	-2.40777728044607
Si	-4.96419886029667	-5.83739340017019	0.69313583076303
Al	-3.67684384702090	-1.14944414965322	-0.75908220718123
Si	-5.51447482282125	-2.82721652130205	1.24175273387935
Si	-2.11227824195694	-1.55033380645354	-6.91140602480211
H	11.67374700198191	-5.53703599901040	-0.03807399823529
H	9.19348300038822	-8.10585799377981	2.36346300146924
H	-3.88154999297200	1.39981100628587	-8.25725800442401
H	-5.54661900926272	-0.07985100340641	-7.37196298734889
H	-6.93206200841403	-1.73335100281443	-5.60666704120743
H	-9.61083500241879	-4.23371600408759	-2.91527899655546
H	-4.80806200308925	-4.38616399593825	-7.98466800596498
H	-1.25558099910454	-7.52827099077458	-7.35483800754076
H	-8.46603498771763	2.53218806267083	6.77163494659215
H	-4.20950399961432	6.50518792460151	2.49497301039588
H	-1.81143199729560	9.00591902094010	0.20164699515333
H	0.67612798185757	11.32783092622202	-2.29765498373796
H	-4.95337000105153	5.93157200382847	-3.34598499809198

H	-7.43212802090059	3.36467303636927	-0.94325501118665
H	-9.81433494073859	1.14100197989242	1.45030699923288
H	-3.26082301622466	2.50672900125518	9.43326995915070
H	-0.74504099905327	4.85495001446410	6.90563299977625
H	1.17176500429730	6.84606700232413	4.60638300430467
H	0.01383700268364	-0.85268900415097	9.75320600087094
H	2.65630397907732	1.61378699517496	7.09828799363533
H	4.53076900130901	3.47379799737136	4.86924399502248
H	1.53720800456152	-6.93798300426386	-4.97365699828077
H	4.63541500616621	9.21535600672182	-0.64625199792118
H	-8.67488799343118	0.53574595290691	8.08886504675334
H	-5.67875104001081	-0.35844205269601	8.92691816472073
H	-5.40516096693458	-3.45826592087473	7.74596987535052
H	-2.86007297896137	-3.03986700018572	9.10083200193254
H	-1.22701003668749	-4.11162699041310	7.70400901073496
H	2.57413299915323	5.42807899573613	-6.16363200061582
H	0.22482900217903	8.10275900237930	-5.85603199829353
H	2.79507599862872	11.25689300589201	-3.42192599779616
H	6.06067100156336	7.26917499940355	-3.76691100040352
H	10.01064999864728	4.23779399884044	-2.76113099987912
H	9.87926499836560	0.42371400048805	-3.59460899893094
H	11.16639499715055	0.78744100146552	-1.60472500104641
H	9.89847199843424	3.90341200119996	-0.38688400204438
H	-1.32399899629349	-1.19594399506924	-8.10330099631738
H	-0.61662899893882	-5.49814399592737	-8.46011600064633
H	2.84818300463817	-3.04332200158895	-5.95038400708645
H	-7.31517900084509	-4.15812799974868	3.75060701194162
H	-9.78249996801226	-1.30915797156742	4.07997000776204
H	-9.40319701141415	-2.75383200832514	1.45013701274881
H	-5.76898899581103	-6.41021300095335	1.77986100215506
H	-3.08084499218219	-8.75082999134667	-0.46797099384915
H	-7.02180600549572	-6.69287800358074	-2.78419799659327
H	2.78359000068231	-7.36886300478836	-0.99247300267655
H	4.89691599966001	-9.57738000041444	2.49409399787318
H	5.82084500126680	-9.04713700287879	0.34374500019238
H	-1.34437700802267	-7.77605500412194	0.87467399483448
H	10.66950899842273	-2.02029699476125	2.56380700234540
H	8.82314100024047	-3.95574100291842	4.78387700486020
H	11.59079999773554	-3.97453399949129	-1.85888000629761
H	5.33815599837844	-0.46451100275439	4.51995499495300
H	7.15219500247814	1.34004700196174	2.37234699857153
H	7.32251199783033	6.57841100005610	-0.51330699900510
H	5.00684299398274	-4.86484799418841	-3.24385799707993
H	7.90214800025548	-7.00114299845299	-1.50659200272908
H	2.44258299848312	-6.49974700106088	2.91143200299250
Ga	-3.76577381235269	-0.18353139050737	1.96687438230575
H	-4.87165240899303	0.88817412301432	2.06183671089923
C	-1.91390002106197	-3.12621945628371	2.38449016083088
H	-2.31555376126308	-3.16180473125672	1.37041646551215
C	-1.96514521474897	-4.22247937668681	3.13593575395549
H	-1.56582317864659	-4.23095910240355	4.14833938440884
H	-2.40384947439778	-5.14322880318426	2.76339421228338
C	-1.34297141363948	-1.81573345978897	2.83729582333028
C	-2.42502586629443	-0.80558270144167	3.26435065123170
H	-0.73486664588203	-1.38304858521091	2.03291128325504
H	-0.66923654236068	-1.98787455288184	3.68946853516138
H	-1.94289339118986	0.12271410880416	3.61074051562589
H	-2.97699717676652	-1.19976875764881	4.12795360166094

C	-6.22700436425502	4.07270460179360	3.72243506614467
C	-5.70508341588950	2.95744668009565	4.21837671957454
H	-5.91481265704274	5.05322363820150	4.07222442890690
H	-6.98858945318000	4.04442984886808	2.94709890029869
H	-4.93988395638870	2.97710870523638	4.99073093201463
H	-6.02265249187595	1.98108275211059	3.86358172393947

I11-GaH 2

Electronic energy (r^{2}SCAN-3c): -30773.352436778827 E_h
Electronic energy (ωB97X-D4rev/def2-mTZVPP): -30781.719292920643 E_h
G_corr (673.15 K, 1 atm): 0.48495 E_h
Number of atoms: 258

Cartesian coordinates (Å)

O	1.49416242660713	2.90199763301627	1.02827476241318
O	-1.94056677413803	6.77540272978916	1.42825757997327
O	5.26874236335023	2.07954807458447	0.80315290297469
O	3.61201665832346	3.88816869712253	-0.15392767779995
O	6.09832506490627	4.57661533911270	0.42051770716524
O	1.45953638359933	3.29226242442487	-1.61246587362323
O	1.37288671456019	5.33081386658408	0.03071018462361
O	-1.71236876368040	6.88869278707356	-1.19310442710691
O	0.32500656741561	7.70439695381379	0.40245575132818
O	2.93531315085377	7.48133371958520	-0.04539254318541
Si	4.85037320532565	3.64121586103782	0.87061671888651
Si	1.99390830177696	3.85078320361168	-0.18734053302489
Si	-1.29368427504914	7.62998914524511	0.20008080550763
Si	1.64980662277379	6.79749877443481	0.65997122626106
O	8.95276635590320	-3.83521165001135	2.22551437011088
O	1.99681607381401	6.65689373883949	2.24394655251082
O	7.14367586317192	-2.28002579473550	-0.92384885479175
O	2.50488980217185	4.75047070119882	4.08111108996542
O	6.74878536709237	-4.90458272137695	-0.77066097549991
O	0.04461986051312	5.12209407336288	3.14473810795323
O	5.85376877600963	-3.26535359996001	1.13695340205432
O	4.44486957472617	4.07325548509220	2.38856559753994
O	9.40113369362188	-5.01126889224587	-1.12802940401891
O	5.15293934577820	-0.24621936705917	2.01932736391094
O	7.73436655092649	4.37823525901481	-1.68998039969547
O	10.70356890620667	-3.18501470746900	0.34575525235839
O	5.43027948735661	5.63172892028104	-1.91038176017764
O	4.09392200527143	5.67531004176211	-4.17663954459023
O	8.76131297773810	-1.46162424436094	1.04413680740997
O	3.92213870596704	7.73593846961828	-2.52951826398941
O	1.16976441523058	9.52065162459123	-4.04551260484423
O	8.98095840029867	2.07152878491523	-1.88660449001097
O	6.94709675360937	0.18937247203975	0.10331734550734
O	2.27298223946947	9.55407630473986	-1.56451425688395
O	8.99934816610540	-0.54794363656309	-1.44284986081702
Si	1.43315296664781	5.86392390760143	3.55231895611170
Si	6.11511770852850	-3.44067671319988	-0.44858497058389
Si	3.63167727260162	3.62511628269611	3.72694403299184
Si	8.07916863509556	-5.83666446974436	-0.65611605516571
Si	10.87631710270421	-4.45901154644916	-0.67203013451072
Si	6.67248040258115	5.32657578848809	-0.90861150683394
Si	9.79494594355001	-2.60886997610957	1.56632977268754

Si	4.92479691571457	6.59466587304603	-3.11834585705723
Si	9.20549778064756	3.67290600259395	-1.66769359165462
Si	6.17162887971724	0.85337379508548	1.37323514260334
Si	1.72941386148860	10.45297664490641	-2.82547276473846
Si	9.80031039936059	0.68261943745573	-2.15341770777184
Si	7.95459409886665	-1.01715478317991	-0.30272431209990
Si	3.46953222366824	8.51875618242526	-1.17866528569874
O	2.97543664989271	-0.47119190779764	3.61363275347617
O	0.96418297042642	0.77349747206233	2.47028789835834
O	1.23882671012871	0.88379758789045	5.07852133395600
O	2.97060580960194	2.16204677071391	3.48575008870586
O	-0.40818667024567	1.20244675065081	0.27287029577460
O	-0.81814455658573	2.73728577441060	2.35631039326132
O	-3.75642238881777	5.00097707977746	0.56589213627248
O	-2.53549631170742	4.65965670855668	2.92328670243124
O	-1.18136033585291	3.43317282778900	4.83741219328158
Si	2.04532341989135	0.84349075988781	3.67602278592120
Si	0.31027880860397	1.91605402960783	1.53040024044026
Si	-3.13252618096150	5.75159955539140	1.86511439296938
Si	-1.10923879500461	4.00087500719259	3.32757521994535
O	8.28713372251742	-6.04297913796185	3.49629977051134
O	-3.19886164310711	-3.06191821868374	6.58139401687772
O	6.68227824101419	-7.84891462575499	2.44467200304085
O	-1.30993670260547	-1.56714094546883	7.68937742524804
O	8.25780376482075	-6.34301928152114	0.87225271441734
O	-5.43240856177203	-3.49066247642267	5.25610027982965
O	6.65260413045293	-3.92175299000888	3.57207421209271
O	-5.13442396745483	-1.21900262372191	6.58859156977478
O	-8.67348704746692	0.34361844694976	5.57614963138780
O	4.47461805021853	-2.51238344078357	3.25176206872468
O	-6.44701441972120	1.04784892736311	6.95167748031024
O	4.49298856139469	-5.06494292694390	2.60679532351851
O	-3.88572960742670	0.85793146374878	7.57839421221724
O	-2.25356282277422	2.84926931635288	7.14578259351530
O	0.39932060503057	2.65949021087656	6.85848923344669
O	4.25834948785428	-7.39614358343031	1.37228437835428
O	-1.50918560189207	0.86586061705884	8.78532600568859
O	0.71974927306826	0.08476925725530	7.52994715159187
Si	8.12600364806397	-7.12450672254667	2.28730727539778
Si	-2.12662167959638	-2.97421476308609	7.81722689749541
Si	8.19982582773050	-4.41666697520064	3.55230198691131
Si	-4.80682763154528	-2.81420226642675	6.58900180302236
Si	4.52674000483973	-0.89526301506215	3.37765645219352
Si	-8.08313391510579	1.12874789561234	6.87881444131676
Si	5.36031800524083	-3.70475253201180	2.61464675723591
Si	-5.30050068249760	0.07008985635700	7.56744831877203
Si	-0.93781837152273	3.48855333264631	6.44095815808082
Si	3.44221387575961	-6.09969717936423	1.92768494997559
Si	-2.74965325639394	1.78628893602198	8.27018066715114
Si	1.29606314539263	1.31816708085149	6.64539817424086
Si	5.41602495947654	-8.50829514816541	1.64838173041989
Si	-0.51610717136113	-0.38655987823458	8.47425457319925
O	1.80483510220204	4.27204693589650	-4.05806035213580
O	-0.06527657232119	5.27180591804469	-2.46264030878186
O	-0.61935214328838	7.68131424521321	-3.46624321447713
Si	0.70730336123845	3.92077747471918	-2.91363710916540
Si	-1.20486229034206	6.40429545174464	-2.65619457170962
O	1.25210911357292	-1.72923690962593	-4.50518017369471

O	-3.99439046830192	3.93499972688605	-4.54377221245204
O	3.34158403203246	-2.96111018136363	-3.48218890689697
O	-2.44686009279811	5.81143581955542	-3.50871750360572
O	-3.82309760075350	4.17107171697031	-1.91928152165744
O	1.33289639006594	-4.41059352105753	-4.41821887500435
O	-4.67094990834192	1.76476083719739	-5.90943470946294
O	4.71941221127191	-3.30911464624399	-1.25692959405681
O	-2.53843709574388	1.71536173937621	-4.39982890348092
O	-3.17193398384642	-0.36990090945186	-6.53695590284201
O	-1.11215579572179	-1.79063110001107	-5.65541703953198
O	-0.32222879031844	0.32804535346578	-4.19933949117033
O	-0.36203420682106	2.85564177206299	-3.48367568328316
O	1.78170976165426	6.92781817919483	-4.27192513683851
Si	-3.84228790808793	4.99838889054692	-3.31828855916923
Si	2.19419523273528	-3.04513273354936	-4.64026357862609
Si	-4.04236298753749	2.30866696104560	-4.52419961319954
Si	4.00733889857240	-4.07893344775428	-2.50621332529976
Si	-4.32569858827851	0.66592917332682	-7.06938884875520
Si	-0.29903031717898	-1.29166970889031	-4.34377441727752
Si	-1.21773503669459	1.48116839029828	-3.50267496183860
Si	2.55620487175212	5.56736811031842	-4.71124370993901
Si	0.62479806829030	8.05133193925825	-4.45457106566353
O	-2.79810221927953	-5.59566437605069	-7.12386893541560
O	-4.50621263452763	-4.33755156665186	-5.53367892134422
O	-2.90863864861291	-2.93190547445905	-7.22140960817312
O	-2.84359475593985	0.15959834042387	0.18731920945052
O	-2.48560395101272	2.78519335613832	-0.14027759780818
O	-1.67958353452368	1.02378792124236	-2.01000464940121
O	-5.13482439370624	2.82035222571381	-0.00570257878521
Si	-3.76335956296843	-4.29618584307227	-6.97887851604443
Si	-1.86139591550482	1.31375432218603	-0.43119033219519
Si	-3.81946435180110	3.69127372054101	-0.37978197420459
O	-4.97157247566776	1.87306282522036	-3.26909120054623
O	2.81289772421118	-5.05287511705995	-1.98175026815308
O	-5.60008540465126	-2.51287852002981	2.84083688844928
O	-7.38731582227251	-1.79084596572870	4.67735990535427
O	-0.24739232970829	-6.30739319530225	-3.40107785090486
O	-8.00789370266924	0.18692247668119	3.00559140326669
O	-0.39636906866445	-5.57498395227438	-5.95984233043272
O	-0.94963498686530	-1.92657379600344	-3.00985898951618
O	-3.48855049895734	-6.51146609613243	0.72692496972603
O	-0.95814931157722	-4.27138270891269	-1.86014398620369
O	-6.58567720657589	1.03603068031028	-1.37752946191040
O	0.62707178162716	-6.08879734328632	-0.86661025417507
O	-8.35588653534432	-0.62394433220534	0.48472446336256
O	-7.37590610348867	1.79338425371240	1.01941777103749
O	-1.87380768152457	-6.74677407580938	-1.40676215284418
O	-6.52943213481323	-0.21510073314094	-3.69840634883494
O	2.71038513762541	-5.41576602987876	0.64139679224950
O	-9.06532622872095	-2.66176340002826	-1.03312595715273
O	-4.47958701747480	-0.43899579912421	-2.05159699156078
O	-7.00201153485000	-2.89857366333888	0.61715978253302
O	-8.04017437463421	-2.38265380653000	-3.48043215494505
O	-5.38703729518377	-2.61718779744946	-3.74612134530545
O	-7.26247066701822	-4.36778279334874	-1.88543973130449
O	-5.00400264519504	-5.15549800097871	-3.07265070464885
O	-2.90069382695311	-3.61774103168120	-3.58142079959471
O	-5.69884519882718	-6.16135350562705	-0.71968376696025

O	-2.73368309555997	-2.50830455444324	-1.14300734149859
O	-4.76951510120052	-4.22185505183956	0.91429157634971
O	-4.62951930688373	-1.55974139978587	0.71177449260707
Si	-6.48026013027024	-3.01718753393839	4.11572189938100
Si	0.59990106708227	-5.83278661434870	-4.70698249917942
Si	-8.49648855760721	-0.65305144483920	4.31450538926132
Si	-1.24369950312566	-6.07052318527253	-7.26056228322451
Si	-6.64214783973571	2.29252125101127	-0.35175509028056
Si	-2.43483197037298	-7.49164992451794	-0.06195706953355
Si	-0.61471227693979	-5.84411293959614	-1.88870574379917
Si	-8.43021811942665	0.65002584309709	1.49714219192345
Si	-5.61498858241686	0.54081421290466	-2.58791340504764
Si	2.25059656283826	-6.02122700346547	-0.80624294800857
Si	-8.48237315126852	-2.23901085523520	0.42117622827480
Si	-6.70626020022721	-1.75595194037335	-4.17444247583871
Si	-8.51898297505220	-3.44614711412157	-2.35408090714052
Si	-4.44280694195807	-3.91997576867271	-3.97069342706461
Si	-6.26014311561878	-5.62016528905269	-2.14735886145967
Si	-1.91591443291227	-3.07839208197175	-2.38918492444441
Si	-4.96269590675979	-5.83384604858176	0.69567222585379
Al	-3.66648560488377	-1.14538411854413	-0.73643270668056
Si	-5.51347311649772	-2.82374577170905	1.24859777119692
Si	-2.11095073927519	-1.54981284640880	-6.91021548123575
H	11.67374700176376	-5.53703602111312	-0.03807397570410
H	9.19348299310057	-8.10585799226128	2.36346298350600
H	-3.88155001570940	1.39981098955906	-8.25725797200906
H	-5.54661898995386	-0.07985100165079	-7.37196301227770
H	-6.93206199123887	-1.73335099967820	-5.60666702293286
H	-9.61083502003468	-4.23371601964297	-2.91527899778347
H	-4.80806202735196	-4.38616400834871	-7.98466801882415
H	-1.25558099110790	-7.52827102948673	-7.35483799929981
H	-8.46603499713805	2.53218801673448	6.77163498367227
H	-4.20950399715622	6.50518799130438	2.49497299269561
H	-1.81143199282675	9.00591898610947	0.20164700330588
H	0.67612795411187	11.32783118588534	-2.29765499281284
H	-4.95336997906162	5.93157198694976	-3.34598498018196
H	-7.43212801512805	3.36467303241586	-0.94325500225298
H	-9.81433501000045	1.14100200363006	1.45030700289754
H	-3.26082301933914	2.50672901323781	9.43327002992507
H	-0.74504100594004	4.85495000080619	6.90563298854424
H	1.17176499949021	6.84606699634825	4.60638300047216
H	0.01383700389211	-0.85268901271915	9.75320602319250
H	2.65630401901360	1.61378699913968	7.09828800030137
H	4.53076899424943	3.47379799557813	4.86924399473124
H	1.53720800169927	-6.93798300152487	-4.97365699728747
H	4.63541500710775	9.21535599578814	-0.64625199363617
H	-8.67488800324967	0.53574598346002	8.08886501866476
H	-5.67875099132025	-0.35844199407340	8.92691798616423
H	-5.40516100936143	-3.45826600803403	7.74597000440238
H	-2.86007297442879	-3.03986700241497	9.10083197919258
H	-1.22701001637846	-4.11162698689202	7.70400901651881
H	2.57413299069315	5.42807898752271	-6.16363199432723
H	0.22482900038329	8.10275900187722	-5.85603203392807
H	2.79507601221430	11.25689299060401	-3.42192600571454
H	6.06067102209573	7.26917500928800	-3.76691100258948
H	10.01064999580877	4.23779399895281	-2.76113099367533
H	9.87926498552229	0.42371399715175	-3.59460899700232
H	11.16639501289248	0.78744100514402	-1.60472499485290

H	9.89847200019120	3.90341199959043	-0.38688400573999
H	-1.32399899132792	-1.19594400099203	-8.10330100584360
H	-0.61662900330525	-5.49814399352463	-8.46011600227270
H	2.84818299760404	-3.04332200008451	-5.95038400093642
H	-7.31517900604304	-4.15812801696599	3.75060700048264
H	-9.78249998197123	-1.30915799625076	4.07996999803346
H	-9.40319699027114	-2.75383199917442	1.45013700164928
H	-5.76898900505751	-6.41021300889312	1.77986101364938
H	-3.08084500171633	-8.75082999215426	-0.46797100903771
H	-7.02180600694422	-6.69287800589561	-2.78419800131676
H	2.78359000824362	-7.36886303341708	-0.99247299341382
H	4.89691599081323	-9.57738002024842	2.49409399360908
H	5.82084498985822	-9.04713699549779	0.34374501601747
H	-1.34437698797286	-7.77605500446762	0.87467400646107
H	10.66950898337037	-2.02029701808786	2.56380699481626
H	8.82314102556350	-3.95574099386328	4.78387705294196
H	11.59080001364343	-3.97453398006719	-1.85888003530841
H	5.33815599182056	-0.46451101664802	4.51995498547593
H	7.15219501475524	1.34004700724254	2.37234700865331
H	7.32251199044388	6.57841098668737	-0.51330700267863
H	5.00684299610143	-4.86484799063836	-3.24385799416733
H	7.90214800084442	-7.00114301379568	-1.50659200298090
H	2.44258303951934	-6.49974698979995	2.91143196535227
Ga	-3.81537673080657	-0.13425527025338	1.98076337564622
H	-4.95843712802868	0.90191498835224	2.06511976496651
C	-1.05430655045620	-5.48947196353659	3.09943631058200
H	-1.92058918419921	-5.89326685855481	2.57540529703472
C	-0.45472099209019	-6.22911973512471	4.02844819472853
H	0.40509784376143	-5.85482292004710	4.58041831579529
H	-0.79858037254603	-7.23139630369267	4.26741273252431
C	-0.66312619465025	-4.09455336658576	2.72879331451547
C	-1.80236330430417	-3.09643394469423	2.98623223768555
H	-0.38538249382948	-4.05387039553952	1.66443399101376
H	0.22491690679885	-3.79577990206716	3.30276725027311
H	-2.62572141787270	-3.29606895109980	2.28581690775664
H	-2.20181725305710	-3.26424598139852	3.99588313313885
C	-2.42274695748784	-0.63773775996590	3.26885334847721
C	-1.34141506845654	-1.64764621005443	2.86864804489278
H	-1.96123055039604	0.33528031815892	3.50220751896366
H	-2.91373157320907	-0.95526623491233	4.19948708724896
H	-0.97553120253266	-1.45143506815922	1.85102999946980
H	-0.47302455613243	-1.51088097718297	3.52896046938673

I12-GaH 2

Electronic energy (r^2SCAN-3c): -30773.328911092169 E_h
Electronic energy (ωB97X-D4rev/def2-mTZVPP): -30781.698696285410 E_h
G_{corr} (673.15 K, 1 atm): 0.50674 E_h
Number of atoms: 258

Cartesian coordinates (Å)

O	1.48036368750078	2.90028303136627	1.02228544874246
O	-1.94068426968440	6.77650510645594	1.42976461908215
O	5.26615226104049	2.07927075901689	0.80639183230948
O	3.60628801877832	3.88343272532547	-0.15165490232725
O	6.09548956769235	4.57500462273846	0.41776773080416
O	1.45552257082820	3.29160989989978	-1.61628865659522
O	1.37091638673850	5.33100155408279	0.02707124604026
O	-1.71152580704619	6.88865149484765	-1.19124480963827
O	0.32547070495851	7.70486712445533	0.40408738211047
O	2.93497744085669	7.48071744097057	-0.04574378856663
Si	4.84742391976462	3.64114287881360	0.87099821018530
Si	1.98791333307600	3.84881627657999	-0.18946967985294
Si	-1.29300755090714	7.63046523995462	0.20164171917423
Si	1.64962479534994	6.79618805019517	0.65953787876376
O	8.95054092086786	-3.83350431271748	2.22553905199633
O	1.99644201842645	6.65372190213698	2.24318148851382
O	7.14368193184718	-2.27964943577968	-0.92117484986971
O	2.50625489296461	4.75180003776203	4.08319228120506
O	6.74841818027004	-4.90424250304985	-0.77241013287327
O	0.04640396175543	5.11579286374986	3.14224705921321
O	5.85128609348077	-3.26946164318976	1.13727696922180
O	4.44634648657185	4.07919421401282	2.38840804140028
O	9.40123838625036	-5.01154650463905	-1.12822429365736
O	5.15462528765790	-0.24928019242884	2.01868055341500
O	7.73456879465388	4.37883672151812	-1.69032145755623
O	10.70242103306352	-3.18514694596866	0.34597277195121
O	5.42997473319299	5.63172290038396	-1.91113733504264
O	4.09058092572374	5.67221099395910	-4.17378631493778
O	8.76174969357630	-1.45947244344867	1.04469491439107
O	3.92215323961361	7.73638896633614	-2.53005334601813
O	1.17365429585495	9.52203635292761	-4.04831432640139
O	8.98099486371851	2.07173593618722	-1.88672075445072
O	6.94694243980133	0.19131740721393	0.10293657070628
O	2.27256071824103	9.55366340900109	-1.56486034635630
O	8.99863550103674	-0.54756310529425	-1.44303500664163
Si	1.43214633662268	5.86196192375743	3.55245097450940
Si	6.11478039178868	-3.44060286161690	-0.44814143363404
Si	3.63290870946922	3.62731777330225	3.72578574917251
Si	8.07883489951962	-5.83622686007744	-0.65667540363706
Si	10.87620454387308	-4.45887268513627	-0.67191258599998
Si	6.67182441056214	5.32649440142263	-0.90913216725731
Si	9.79417720178117	-2.60799242054817	1.56650942272621
Si	4.92366867592698	6.59416393727392	-3.11895932333240
Si	9.20543659864534	3.67315430388634	-1.66770553190159
Si	6.17085435835155	0.85336316107419	1.37392745479357
Si	1.72982714696699	10.45318196822873	-2.82568376696896
Si	9.80019784221245	0.68277753777964	-2.15341701316058
Si	7.95433853123888	-1.01556544802327	-0.30197090486124
Si	3.46930969864925	8.51847137339334	-1.17869604752082
O	2.98235104320639	-0.46522514128584	3.62264679927092

O	0.97589758195608	0.76573527535453	2.46400760563363
O	1.24153284183590	0.90328796378586	5.07562599047479
O	2.97238566520367	2.16618936738657	3.47395873265893
O	-0.42051847519653	1.18511750728816	0.28706925058358
O	-0.81891762426996	2.72470978530282	2.36774289010861
O	-3.75200761834014	5.00055769560780	0.56236312859952
O	-2.53354558256861	4.65632415599574	2.91972017942149
O	-1.18279011467913	3.43756060355356	4.84180037226665
Si	2.04993277758838	0.84697418234822	3.67700624946728
Si	0.30629198215852	1.90791028711303	1.53434242534865
Si	-3.13135100597323	5.75043660957273	1.86473733030920
Si	-1.10900676146547	3.99579357558681	3.32965725425083
O	8.28877165738950	-6.04279997026717	3.49625699031848
O	-3.19896952375132	-3.06902457478618	6.58415859302103
O	6.68261841088738	-7.84816320244492	2.44485180288044
O	-1.31546725875150	-1.56664909339384	7.69886840429000
O	8.25647542174355	-6.34213132809221	0.87212328456671
O	-5.42539933771227	-3.48455548026581	5.24536344392197
O	6.65075488197817	-3.92475789900488	3.57236803918838
O	-5.13375800602438	-1.21953165614002	6.59028372439470
O	-8.67403252493005	0.34451096280128	5.57623792735397
O	4.47399913233297	-2.51271424234034	3.25188098545067
O	-6.44673820655420	1.04709088244975	6.95142832027302
O	4.49125628167390	-5.06744277666441	2.60842074896748
O	-3.88542593978143	0.85734941246708	7.58133132373399
O	-2.25575586839459	2.85116611642547	7.14776570623257
O	0.39718161595653	2.66010651810759	6.86628170136706
O	4.25584821778165	-7.40094380094570	1.37641329258488
O	-1.50758238353381	0.86814285873209	8.78662514808637
O	0.71654986329815	0.08373123167429	7.52447224271786
Si	8.12667021972358	-7.12381638558865	2.28715436643086
Si	-2.12809046280064	-2.97545973638212	7.81979715296273
Si	8.19898482764502	-4.41644235403025	3.55253411478461
Si	-4.80622893931544	-2.81366841362810	6.58576822833871
Si	4.52907624924456	-0.89583171448779	3.37897601735837
Si	-8.08249024581087	1.12883856304769	6.87927722982556
Si	5.35807906496353	-3.70583282657302	2.61587233615202
Si	-5.29977188965768	0.06972295895865	7.56799232602536
Si	-0.93845981397423	3.48890829088889	6.44439249178731
Si	3.44338774006105	-6.10068784058404	1.92486763267786
Si	-2.74938159214552	1.78680218626294	8.27123765283955
Si	1.29543199633944	1.32067943056688	6.64564588685730
Si	5.41754347177532	-8.50952122012168	1.64922087040817
Si	-0.51507435121021	-0.38443816525829	8.47507069665079
O	1.79675517036509	4.27011218944076	-4.06269692633739
O	-0.07062857715295	5.27045715686535	-2.46595891611494
O	-0.61479670524935	7.68684894163472	-3.46108142067553
Si	0.70048030988788	3.91857813170885	-2.91659233050741
Si	-1.20456273441649	6.40897882156013	-2.65580017352844
O	1.24741438189837	-1.73069029429676	-4.49875500863559
O	-3.98394866437614	3.94388841513893	-4.55085203330156
O	3.34296573346234	-2.95828233514545	-3.48386995014193
O	-2.44866140124873	5.82598547981815	-3.51184310998316
O	-3.80490726861579	4.16801326036494	-1.92439984072202
O	1.33481951658973	-4.41134761384441	-4.41602089993872
O	-4.66855114699739	1.77257413603622	-5.91661723944669
O	4.71998868296124	-3.30711839009668	-1.25792379496348
O	-2.55742822580927	1.70667737123501	-4.37859979323540

O	-3.17062870891718	-0.36713710615263	-6.53587745694953
O	-1.11175203140513	-1.79294645750789	-5.65709533884981
O	-0.33267173078234	0.32598510180924	-4.19839210897307
O	-0.37221755080578	2.85310551344889	-3.48237329366263
O	1.78104268207234	6.92695507096697	-4.27181878798769
Si	-3.83747730344321	5.00282817359837	-3.32038915604893
Si	2.19296060745956	-3.04410205890075	-4.63961979991053
Si	-4.05142600610442	2.31858674637734	-4.52733715008074
Si	4.00875680538984	-4.07596896429483	-2.50798809907237
Si	-4.32528590674355	0.66695824839016	-7.07021232342423
Si	-0.30485786510583	-1.29387047855650	-4.34183502419106
Si	-1.22664435542723	1.47699520886005	-3.49548810558560
Si	2.55350233333163	5.56536200082492	-4.71107219854471
Si	0.62642936456787	8.05277910539352	-4.45449266320650
O	-2.79704827624545	-5.59337601383032	-7.11590358627645
O	-4.52472372744629	-4.33692758070582	-5.54108109640214
O	-2.91338657564398	-2.92991870508905	-7.21862230784992
O	-2.85587923737128	0.14120366078207	0.18542483191166
O	-2.49344541659126	2.77177373493871	-0.13252013298454
O	-1.67460415895570	1.01441458733922	-2.00055421262938
O	-5.13854001242245	2.82755395840083	-0.01502963270942
Si	-3.76620670150142	-4.29575407478075	-6.97851116023602
Si	-1.86870431722797	1.30090034963360	-0.42368931545094
Si	-3.81655426267709	3.69084187372044	-0.38318073196730
O	-5.01103891203775	1.90222849333371	-3.28767629369189
O	2.81337618135186	-5.04931572842193	-1.98476641628872
O	-5.61498206258221	-2.50050229259372	2.82438786155504
O	-7.38741559239482	-1.79041497881154	4.67756899764165
O	-0.25858289945436	-6.30250393431372	-3.40837738583000
O	-8.00703651521795	0.18775763193028	3.00620147439804
O	-0.38878525992017	-5.57175386216828	-5.96648490801039
O	-0.96156188090095	-1.92996993103766	-3.01052995911327
O	-3.48322507155266	-6.50149464018520	0.73251570545186
O	-0.96922723726215	-4.27233439889433	-1.85926002655821
O	-6.58670799419634	1.03342758880846	-1.37741004256499
O	0.63032477125212	-6.09711717667515	-0.88293890580976
O	-8.34815549009100	-0.62916073233085	0.48760813493213
O	-7.37334101523895	1.79002400035474	1.01853405565864
O	-1.87446853053886	-6.74976578614925	-1.40567246567629
O	-6.53608676700584	-0.21419579100366	-3.70024135958451
O	2.71683784407989	-5.42212367354352	0.63453235508853
O	-9.07308029474189	-2.65604083107113	-1.04013356758698
O	-4.45945766310918	-0.39792142193228	-2.08083725784164
O	-7.02334492864112	-2.92841371599369	0.61360190696796
O	-8.03623647963142	-2.38649576133891	-3.48279547662933
O	-5.37985420485121	-2.61183770076729	-3.74388268313462
O	-7.26578586197657	-4.36885847435780	-1.88352681438605
O	-5.00784964479049	-5.15315425811840	-3.07490466359757
O	-2.89519045544798	-3.63578745694865	-3.60924841554869
O	-5.69403775186390	-6.15618048514674	-0.71868297248731
O	-2.78438217273977	-2.52502574546935	-1.18010197377575
O	-4.76904646804477	-4.21380595711005	0.91428407277137
O	-4.69888650866764	-1.53735832210351	0.66331995187373
Si	-6.47949247950402	-3.01310068826575	4.10869997933235
Si	0.59958005275152	-5.83184482042950	-4.70851551146691
Si	-8.49664509367861	-0.65264923494466	4.31532906100769
Si	-1.24356913513244	-6.06981697417159	-7.26072485310493
Si	-6.64392431052135	2.29227243740974	-0.35435361125693

Si	-2.43783764848922	-7.48754486383648	-0.05780164934109
Si	-0.61956927427297	-5.84430756536598	-1.89257278016687
Si	-8.42945926937096	0.64763027615117	1.49667642328209
Si	-5.62235554814435	0.55776195777758	-2.60106731515757
Si	2.25230995909202	-6.02201652682698	-0.81361664161412
Si	-8.49056958645051	-2.24314459201085	0.41683447329047
Si	-6.70332423204274	-1.75631193803332	-4.17499882486177
Si	-8.51999750488315	-3.44614596884560	-2.35556140810487
Si	-4.44603101565559	-3.92231487196208	-3.97816565165763
Si	-6.26073972600572	-5.61934612082727	-2.14592274279889
Si	-1.92986200926416	-3.08841695784186	-2.40434885237140
Si	-4.95890063570212	-5.82850307530822	0.69831984295373
Al	-3.68702467603826	-1.14713925000515	-0.76431109363827
Si	-5.53327223221972	-2.82452914869419	1.23398169819314
Si	-2.11203712204393	-1.54930072836238	-6.91003262759707
H	11.67374699513043	-5.53703596779133	-0.03807401533953
H	9.19348297816635	-8.10585795251339	2.36346301884405
H	-3.88155000785305	1.39981100249160	-8.25725798448071
H	-5.54661899719499	-0.07985100461888	-7.37196300905250
H	-6.93206200265672	-1.73335099949469	-5.60666708284165
H	-9.61083503229384	-4.23371602984608	-2.91527900069244
H	-4.80806202791318	-4.38616399362848	-7.98466803601921
H	-1.25558101710060	-7.52827098955006	-7.35483798895354
H	-8.46603499698468	2.53218805460810	6.77163501379887
H	-4.20950400947888	6.50518800606008	2.49497300000896
H	-1.81143200738373	9.00591900219559	0.20164699955605
H	0.67612790954291	11.32783048785747	-2.29765488584276
H	-4.95337001455653	5.93157203418305	-3.34598497580168
H	-7.43212805471538	3.36467308136757	-0.94325501107820
H	-9.81433498341085	1.14100200717124	1.45030699552726
H	-3.26082301142770	2.50672901033050	9.43327001980603
H	-0.74504101576536	4.85494999885681	6.90563299288372
H	1.17176499746065	6.84606702277463	4.60638302251926
H	0.01383700211473	-0.85268901570262	9.75320599990823
H	2.65630406276247	1.61378701211316	7.09828801834219
H	4.53076899098979	3.47379800256686	4.86924398917652
H	1.53720800896003	-6.93798300883095	-4.97365699732068
H	4.63541500437786	9.21535601931812	-0.64625199548176
H	-8.67488798721928	0.53574602110893	8.08886496085740
H	-5.67875099904159	-0.35844200024239	8.92691800794341
H	-5.40516097735813	-3.45826597883658	7.74596992776950
H	-2.86007296820657	-3.03986707588906	9.10083198271071
H	-1.22701004630200	-4.11162690849369	7.70400908430467
H	2.57413299531092	5.42807900185249	-6.16363200483075
H	0.22482900720727	8.10275902404384	-5.85603199274395
H	2.79507606250633	11.25689303904931	-3.42192603780947
H	6.06067098698005	7.26917500568184	-3.76691099261514
H	10.01065000157447	4.23779399981222	-2.76113100444170
H	9.87926499976511	0.42371400142656	-3.59460899210618
H	11.16639499224493	0.78744100090082	-1.60472500366857
H	9.89847199845776	3.90341200151521	-0.38688400070919
H	-1.32399898058287	-1.19594398501541	-8.10330100277007
H	-0.61662897044411	-5.49814396508699	-8.46011603787180
H	2.84818302430812	-3.04332199944803	-5.95038404212543
H	-7.31517900204261	-4.15812799827889	3.75060700195434
H	-9.78249997011875	-1.30915796366338	4.07997000629474
H	-9.40319700233344	-2.75383200266800	1.45013701170085
H	-5.76898902479446	-6.41021302262967	1.77986104112089

H	-3.08084495246904	-8.75082992796682	-0.46797096460061
H	-7.02180599010905	-6.69287798976843	-2.78419800259754
H	2.78358999713595	-7.36886302569418	-0.99247302713090
H	4.89691599262734	-9.57737998344586	2.49409400192218
H	5.82084498032918	-9.04713698527311	0.34374501077320
H	-1.34437702644869	-7.77605500530805	0.87467396281017
H	10.66950899305367	-2.02029698133504	2.56380698202873
H	8.82314097220577	-3.95574100281857	4.78387696896441
H	11.59079998764552	-3.97453401424362	-1.85887997734990
H	5.33815597787131	-0.46451100811217	4.51995499163772
H	7.15219500607683	1.34004701368940	2.37234700496785
H	7.32251200101649	6.57841100868646	-0.51330699907253
H	5.00684298442432	-4.86484799426609	-3.24385798488521
H	7.90214799889238	-7.00114297826723	-1.50659199978060
H	2.44258314550072	-6.49974710410867	2.91143204700587
Ga	-3.88627789290792	-0.14076897434168	1.92974857856884
H	-4.91994502133334	0.99399289055720	2.02857379399748
C	-0.07083486425038	-3.18496217230884	4.06250732684564
H	0.09461822683103	-3.73057888737566	4.98812501051239
C	-0.80716400116082	-1.87676309311550	4.12740947634085
H	-0.46775582710634	-1.07833240959206	3.47197604080427
H	-1.18658690281963	-1.54529398496805	5.08929467826039
C	0.90771160674884	-3.37436581166704	2.90352452568230
C	0.03206970134551	-3.84065894732406	1.72353373375147
H	1.68851654659066	-4.09828534265809	3.15723937679112
H	1.41569500841501	-2.43253682959249	2.66412554377516
H	0.46135837333090	-3.60036091114337	0.74545089213467
H	-0.08851235749094	-4.93099314798216	1.77075426383612
C	-1.46309093726518	-3.06278311038034	3.47499122796170
C	-1.33540852445911	-3.17111835264346	1.95683617098596
H	-2.93027059282214	-0.84480505826991	2.90237991225490
H	-2.31705811325522	-3.51217781344814	3.97441393754485
H	-2.15386774251778	-3.74257839174000	1.50900482646568
H	-1.34763931551455	-2.17438275396756	1.49262896367143

I13-GaH 2

Electronic energy (r^2SCAN-3c): -30773.348768024298 E_h
Electronic energy (ωB97X-D4rev/def2-mTZVPP): -30781.717562384190 E_h
G_{corr} (673.15 K, 1 atm): 0.48130 E_h
Number of atoms: 258

Cartesian coordinates (Å)

O	1.47420332243317	2.93217600815589	1.02906018309156
O	-1.94534058914412	6.76572157316214	1.41368900101527
O	5.26671600420645	2.07548315974681	0.80247903359740
O	3.60899997794886	3.87899226346066	-0.15593164059520
O	6.09821006075635	4.57027823537458	0.41048337965995
O	1.44713050323596	3.30368256663930	-1.61354297351624
O	1.39504042488413	5.35836199607193	0.00564074935122
O	-1.71605737355296	6.90395742842878	-1.20650802598905
O	0.31380200715951	7.71656329934871	0.39745157592485
O	2.90407722716059	7.54959018991335	-0.07095639692268
Si	4.85047295795189	3.63800563260145	0.86635415882544
Si	1.99041681084116	3.86512619749407	-0.19193996110511
Si	-1.30188646598527	7.63217079271613	0.19384371712751
Si	1.64999854730725	6.82485444022934	0.64486399689122

O	8.95019152539640	-3.83363257027488	2.22527934103752
O	1.99739688134369	6.67180835730787	2.22890310047956
O	7.14383823257533	-2.27914699762198	-0.92223595497436
O	2.51703900317145	4.76936396759105	4.06945922958062
O	6.74853917695236	-4.90381467413001	-0.77051835925928
O	0.05441710279587	5.12022903965839	3.12999595199256
O	5.85186568486556	-3.26631990504518	1.13774136738849
O	4.45439823781817	4.07622428707745	2.38481158985386
O	9.40089801082289	-5.01164336174333	-1.12804889415115
O	5.16406197477472	-0.25401315402594	2.01382082347034
O	7.73128657821457	4.38400703882273	-1.69890345034433
O	10.70328941095407	-3.18606113045210	0.34636556665740
O	5.41913216683117	5.65402343443808	-1.90210705133257
O	4.09215894547772	5.67423114792803	-4.17284583077956
O	8.76276458098171	-1.46039309253739	1.04312353163894
O	3.89540972433315	7.75086055761188	-2.54884762211131
O	1.23993069434327	9.54802443096382	-4.03241798012038
O	8.97969845284358	2.07274652706736	-1.88638697646295
O	6.95305083599371	0.19400690206949	0.09913038620012
O	2.35928468762937	9.69323121519993	-1.58085883350163
O	9.00134093990632	-0.54906262773847	-1.44535073868887
Si	1.43732828344950	5.87404632422068	3.53854082640420
Si	6.11529844567703	-3.43972182850240	-0.44758924224683
Si	3.63647989997520	3.63474418249585	3.72289459506382
Si	8.07868399569199	-5.83646512191609	-0.65629732739195
Si	10.87619578490858	-4.45944027290665	-0.67198036173370
Si	6.66833518710407	5.33102831026044	-0.91407597724485
Si	9.79463857099181	-2.60858228566026	1.56626702519371
Si	4.91168851765073	6.60850318993811	-3.11792201648345
Si	9.20233785289699	3.67542778962721	-1.66863949084977
Si	6.17540948174945	0.85319827802871	1.37008552440844
Si	1.52858025948536	10.51953721774525	-2.73559587233399
Si	9.80022470796165	0.68426998504958	-2.15409945831995
Si	7.95698737351237	-1.01592075664933	-0.30431264154831
Si	3.47626928050549	8.56909612724010	-1.20539617072756
O	2.98691685099527	-0.45512653492229	3.60714597633931
O	0.97184541021319	0.78459624078444	2.46672236655392
O	1.24854859448751	0.89260425196905	5.07840969791499
O	2.96818816344174	2.17637377779197	3.47882661015770
O	-0.40109718553080	1.19808714928319	0.27869348501938
O	-0.82884649079825	2.73567256693334	2.35839587290892
O	-3.76210324667536	4.97865831937979	0.57451606189161
O	-2.53147554211275	4.66774613647007	2.93158535980566
O	-1.16820881046306	3.44345728407294	4.83921869049865
Si	2.04916093806516	0.85398476656359	3.67457066927576
Si	0.30801421521824	1.92543901172419	1.53407873647047
Si	-3.13468055290447	5.74536710135796	1.86389960828470
Si	-1.10452471207520	4.00462348445778	3.32780941222034
O	8.29074134828415	-6.04367456574013	3.49641111202190
O	-3.20428734839532	-3.07237610345721	6.58971091028776
O	6.68291650495190	-7.84849244890609	2.44554559306354
O	-1.31498947284056	-1.56696379192519	7.69291642395718
O	8.25657531785608	-6.34286543036438	0.87224133650924
O	-5.42728233506003	-3.48344959791732	5.24617423844601
O	6.65073550124375	-3.92866391240435	3.57187394954960
O	-5.13536489422775	-1.21844316517230	6.58962124267599
O	-8.67277409955736	0.34870447660229	5.57231970453726
O	4.47824349902138	-2.50947187439888	3.25563861229783

O	-6.44716523376527	1.04800433890107	6.95174957503648
O	4.48430097176857	-5.05972699817455	2.60584230277827
O	-3.88666459819129	0.85988023989278	7.58102045891415
O	-2.25382222509279	2.85005884840833	7.14306944483675
O	0.39943960409384	2.65813478614858	6.86506508402797
O	4.25796087103127	-7.39826952206921	1.37751788032081
O	-1.50689020710758	0.86790830012182	8.78347404797483
O	0.71943835006937	0.08097298748565	7.52569392338968
Si	8.12691742375222	-7.12436042157062	2.28739361288862
Si	-2.12786342026363	-2.97557239982134	7.82079139347420
Si	8.19987172705365	-4.41755117241079	3.55241170706717
Si	-4.80877021683974	-2.81303678681172	6.58740355045172
Si	4.53303189558312	-0.89145872974719	3.37471320844751
Si	-8.08266324881372	1.12865911317523	6.87912360118574
Si	5.35923120107524	-3.70394514565544	2.61614539036128
Si	-5.29980895075001	0.07066079294231	7.56805188121147
Si	-0.93488691317276	3.48941523422773	6.44363591065764
Si	3.44193436329670	-6.09945535259707	1.92441010419731
Si	-2.74826921039863	1.78774888580833	8.26923196138579
Si	1.29700980560332	1.31804327427929	6.64646217616267
Si	5.41728779235344	-8.50936605803000	1.65029247077495
Si	-0.51578573321176	-0.38672542070425	8.47292300000567
O	1.79704836833182	4.27957731983245	-4.06085048222639
O	-0.07223846983094	5.28547830308308	-2.47664774776852
O	-0.57614292937879	7.72125676651290	-3.44767357894043
Si	0.69848951555826	3.93022321378068	-2.91790889642904
Si	-1.19310570829724	6.43632048563162	-2.66942409234114
O	1.25064227670984	-1.73037645565211	-4.50009124618044
O	-3.95765342339651	3.95473666735022	-4.55276028530947
O	3.34426797234723	-2.95845936467272	-3.48404084776305
O	-2.43938318913206	5.86471123339214	-3.53310116647067
O	-3.77113919603981	4.19046786612397	-1.92720501326534
O	1.33443771153876	-4.41155712107515	-4.41313831062269
O	-4.65412659340979	1.79068942818359	-5.92652300361745
O	4.71995717561418	-3.30743680200963	-1.25701299985984
O	-2.55288313003247	1.70423498322538	-4.37867599464675
O	-3.15203480195125	-0.34507696734860	-6.54868281987752
O	-1.10777824359386	-1.78784730474414	-5.66210813147166
O	-0.32224895231778	0.33132283514437	-4.20673880838126
O	-0.37154942149289	2.86189480631349	-3.48550142858415
O	1.79376031368327	6.93777356950032	-4.31142083957382
Si	-3.81652067281830	5.02046036592102	-3.32707794169205
Si	2.19408858563752	-3.04512346422212	-4.63940434601677
Si	-4.04027224392743	2.32980415594458	-4.53267784213001
Si	4.00957669030669	-4.07594388659272	-2.50766967685843
Si	-4.34504445948179	0.65556964847316	-7.06194388266986
Si	-0.30036516694622	-1.28907704472659	-4.34761405781191
Si	-1.21740003053979	1.48059255807153	-3.50186502826110
Si	2.56018237590236	5.56400583932003	-4.72257102841348
Si	0.65797605441749	8.08717609869067	-4.45642676408636
O	-2.79406260756649	-5.58346070046601	-7.11487678309872
O	-4.53844585426862	-4.34273996668222	-5.54473275375122
O	-2.92862969936041	-2.91720315822276	-7.20804449720270
O	-2.82343731213802	0.12780487451243	0.17726104624925
O	-2.48571015466835	2.76456099822417	-0.14390708370820
O	-1.65791632909962	1.00879631022293	-2.00805312698980
O	-5.12768282232396	2.80686792377037	-0.05029137717753
Si	-3.77033588340032	-4.29144968913646	-6.97709894407482

Si	-1.84907902971287	1.29885148982794	-0.43262022385006
Si	-3.80753576134027	3.68462755696663	-0.39426793932061
O	-5.00798954973482	1.91757385551928	-3.29636014783269
O	2.81345287522439	-5.04945702555032	-1.98663875950463
O	-5.61225001945552	-2.49594665244565	2.82835613002009
O	-7.38942268853836	-1.78981867928395	4.67755793622562
O	-0.26507619821135	-6.30493946958830	-3.41494913391891
O	-8.00641889359909	0.18490987912217	3.00138376348726
O	-0.38374758512069	-5.56856820311434	-5.97116306541954
O	-0.96274227058446	-1.92650530781386	-3.01924405130566
O	-3.48809514606062	-6.50632049083534	0.73385834315910
O	-0.97588647282048	-4.27167455242849	-1.87270472162610
O	-6.61094213964723	1.04195380357149	-1.40612472917244
O	0.62842423556074	-6.09700528617887	-0.89263318336175
O	-8.35957416963976	-0.62590865278878	0.47995859936125
O	-7.35585186994280	1.77784793872338	1.01288269790894
O	-1.87824512287131	-6.74579106228284	-1.40443710439409
O	-6.51873258930399	-0.20678321098846	-3.72400657230403
O	2.71707862683195	-5.42393539499987	0.63099789501032
O	-9.07650862808724	-2.66591112827797	-1.03352460149452
O	-4.46556167870537	-0.37550051087872	-2.07214570898847
O	-7.01153773422383	-2.90653251614305	0.60834519188292
O	-8.03084829115827	-2.37193349652495	-3.46775745712228
O	-5.37660098944937	-2.61093058247768	-3.74373868756933
O	-7.26464383595611	-4.36795476874100	-1.88636856498199
O	-5.00488187032518	-5.15236259965365	-3.07300373698647
O	-2.89165608025393	-3.64012614484576	-3.63345147121611
O	-5.69530282580393	-6.15349274312538	-0.71762192141410
O	-2.80485303663619	-2.52519221974897	-1.21227069373524
O	-4.77087980582720	-4.21281238902829	0.91825645097539
O	-4.66641063360848	-1.53771235862744	0.67914944744379
Si	-6.48058805443981	-3.01197898613240	4.10997222922524
Si	0.59950606404974	-5.83144358809832	-4.71029278223795
Si	-8.49676829178342	-0.65101024205558	4.31334647136932
Si	-1.24412127457751	-6.07045523703861	-7.25996327120691
Si	-6.64421830598983	2.28832968256254	-0.36686237444771
Si	-2.43912635029100	-7.48745356983963	-0.05726656978940
Si	-0.62384447696597	-5.84423596527845	-1.89971969145206
Si	-8.42682479836657	0.64839349929058	1.49263382445229
Si	-5.62466951368559	0.57222742350086	-2.61404706039803
Si	2.25084565827814	-6.02255971280819	-0.81693625475810
Si	-8.48948713276917	-2.24078489519484	0.41789553822735
Si	-6.69965524710371	-1.75427649434739	-4.17558601905938
Si	-8.51953821882176	-3.44425212539553	-2.35382462762004
Si	-4.44781068691607	-3.92461345774399	-3.98349694503572
Si	-6.25984141092167	-5.61877286946855	-2.14627559303273
Si	-1.93434165697939	-3.08787982596036	-2.42452901791235
Si	-4.96082975777272	-5.82688239569793	0.70000095628552
Al	-3.68551819812087	-1.14118782353345	-0.77156803112941
Si	-5.52376296936824	-2.81743735008757	1.23746577966006
Si	-2.10957042744289	-1.54381203775744	-6.91341474908209
H	11.67353999825855	-5.53749700104076	-0.03813000157091
H	9.19325800076411	-8.10610399820809	2.36365699748086
H	-3.96156900041180	1.36419899800481	-8.28506099781335
H	-5.56398599732779	-0.12270100103949	-7.29597600038565
H	-6.93632899656159	-1.73777199874153	-5.60525599619854
H	-9.61070199474458	-4.23261599947592	-2.91602399846048
H	-4.80937899688301	-4.38478700203919	-7.98416800087740

H	-1.26201400209182	-7.53013899865133	-7.34713300028326
H	-8.46716099935862	2.53175400438412	6.77435899770159
H	-4.20618400107771	6.50892100186644	2.49273000233767
H	-1.81213400120674	9.00646799820816	0.20177800000662
H	0.23145499167991	10.95011998393610	-2.10809599117895
H	-4.94421500117075	5.93630900121550	-3.34769400014095
H	-7.43440299905789	3.36541399893472	-0.94274899866265
H	-9.81225000101160	1.14342200163618	1.44886400200081
H	-3.26051300279369	2.50761900163341	9.43266799942116
H	-0.74472599802000	4.85510199907936	6.90777200204246
H	1.17261700003421	6.84873799863236	4.60176299833184
H	0.01371299825369	-0.85239400120871	9.75320300198030
H	2.65644500505054	1.61373199966444	7.09816900023847
H	4.53016699932601	3.47572500176462	4.86954700192586
H	1.53638199997112	-6.93834199783966	-4.97336599790931
H	4.68077899865986	9.17388299768496	-0.65420600238962
H	-8.67443700028626	0.53296800235221	8.08822299717992
H	-5.67876700111044	-0.35798099906836	8.92693200318173
H	-5.40538799852625	-3.45793999867753	7.74582399611633
H	-2.86030599860023	-3.03941600365566	9.10082099990147
H	-1.22727300175654	-4.11134499665850	7.70409200111544
H	2.59107099812195	5.38865099919983	-6.17172399809597
H	0.22684599754471	8.11439299833384	-5.85590800266787
H	2.35648400382998	11.66568599979657	-3.24752300005561
H	6.05893599825062	7.26486299966634	-3.76938199769572
H	10.01162899765711	4.23690500246962	-2.76123799990081
H	9.87912999949457	0.42295200122935	-3.59486199998463
H	11.16666299979049	0.78647299907198	-1.60521600256939
H	9.89955900187947	3.90245199958563	-0.38701600014022
H	-1.31531299973238	-1.20647000051741	-8.10616500169468
H	-0.61432200060661	-5.50729800108113	-8.46106599900144
H	2.84834100146714	-3.04402000288020	-5.95043800047600
H	-7.31528200382927	-4.15742500722756	3.75037499884412
H	-9.78237299684380	-1.30797899925577	4.07942699877380
H	-9.40254699618458	-2.75213299766480	1.45267099626914
H	-5.76896400245163	-6.40939899988310	1.78048400265838
H	-3.08137191467138	-8.75069596265781	-0.46757193383764
H	-7.02203599453188	-6.69280399912604	-2.78388199799620
H	2.78306900283381	-7.36900600744242	-0.99232700076650
H	4.89569300221190	-9.57645900048875	2.49579400259504
H	5.81992199869922	-9.04892400075293	0.34487199996268
H	-1.34472310415970	-7.77598302695203	0.87488992175148
H	10.66941400036396	-2.02071799777097	2.56381700122798
H	8.82291799904876	-3.95596100131059	4.78390500018233
H	11.59057499760487	-3.97495100240332	-1.85889599899603
H	5.33835300114186	-0.46465100089969	4.51989000254053
H	7.15208500123856	1.34023100166939	2.37194499700579
H	7.32230800111477	6.57802299882796	-0.51391500208949
H	5.00675699719340	-4.86530399895801	-3.24361799534164
H	7.90182200069598	-7.00149500115640	-1.50647500149476
H	2.44258800477074	-6.50026699497077	2.91141099256962
Ga	-3.77735729793826	-0.18762259417431	1.94664576257293
H	-4.78815510624567	0.95882490721590	2.13941223967562
C	0.34719767235427	-2.58491648854151	3.68335370572467
H	0.98187153136682	-2.07634911895930	4.40757041375313
C	-0.86156381205900	-3.00350957173937	4.06099003191316
H	-2.77034912210944	-0.91536717491887	2.85654314236478
H	-1.18647034804498	-2.82901815051729	5.08274529615356

C	0.89525807016592	-2.78390010699461	2.30051651894260
C	0.06085530336360	-3.77750305688623	1.49390161006733
H	1.94044954264036	-3.11636507062985	2.36163754742676
H	0.92915924006664	-1.81310857789554	1.78246544893789
H	0.33486858826467	-3.72623666524611	0.43570086042647
H	0.28117069452365	-4.79979632370149	1.83097147897948
C	-1.82938587918433	-3.69165102083600	3.14012254045592
C	-1.43062979402424	-3.51078563299882	1.67632870316944
H	-2.84326812411100	-3.30750851042933	3.32111336209155
H	-1.87761526095038	-4.76452548526037	3.38757455148710
H	-2.02815673624303	-4.15995947658669	1.02881881046852
H	-1.64048035417502	-2.47741403958533	1.36537897950548

I14-GaH 2

Electronic energy (r^2SCAN-3c): -30773.286035279329 E_h
Electronic energy (ωB97X-D4rev/def2-mTZVPP): -30781.647950459377 E_h
G_{corr} (673.15 K, 1 atm): 0.48779 E_h
Number of atoms: 258

Cartesian coordinates (Å)

O	1.48022876334006	2.93810857808915	1.03048145935580
O	-1.94399087084666	6.76926611507662	1.41705642959443
O	5.26460127063651	2.07477910487053	0.80365083713142
O	3.60774523461309	3.87262719971097	-0.16804511034901
O	6.09339237063096	4.57083284237318	0.40934046081851
O	1.42669283278992	3.29594860592222	-1.61238513439449
O	1.39774735483953	5.36048111580199	-0.00438623239644
O	-1.70627782587085	6.89906221230598	-1.20236549297628
O	0.31671919674493	7.71724996563580	0.40475529333208
O	2.90614024355332	7.55138159888330	-0.06717229714897
Si	4.84622930558660	3.63646079839744	0.86163987413604
Si	1.98927926721949	3.86568336818813	-0.19973133373174
Si	-1.29837138557723	7.63328732804962	0.19664963798624
Si	1.65257966570713	6.82352431201836	0.64544629315492
O	8.95094086477650	-3.83409157372746	2.22525235176164
O	2.00033010484904	6.66008958979226	2.22821548183147
O	7.14501095769622	-2.28058123886851	-0.92195867916008
O	2.50815057552829	4.75863977431247	4.07109414351363
O	6.74855239330521	-4.90501295156188	-0.77116661047998
O	0.04616134097070	5.12594165161174	3.13501232448677
O	5.85251988581282	-3.26839491454090	1.13727707916926
O	4.44289848052330	4.07765840259754	2.37820492005169
O	9.40101947660088	-5.01141936089937	-1.12856351600373
O	5.15726031186785	-0.25127027243358	2.01853448185659
O	7.73094315887426	4.38324452435605	-1.69729155998134
O	10.70298198933251	-3.18563864037132	0.34585287244940
O	5.41905436217450	5.65326361913848	-1.90320599574255
O	4.10206756930534	5.68269404299905	-4.18232872350092
O	8.76267337707597	-1.46010168441019	1.04406138883375
O	3.89398891376886	7.74868639551428	-2.54679719265646
O	1.24336729504348	9.55029049831441	-4.03468897196976
O	8.97960086205077	2.07301937099936	-1.88677597182478
O	6.94853991710773	0.19041317282013	0.10215464298534
O	2.35805517477933	9.69190161166250	-1.58079828256813
O	8.99931437858524	-0.54714056549932	-1.44379330141072
Si	1.43503219463202	5.87065760128196	3.54053795102198

Si	6.11552926071990	-3.44094404245663	-0.44833751025076
Si	3.63540965028945	3.63231402860126	3.72135173709505
Si	8.07921751368902	-5.83673272563396	-0.65661596592042
Si	10.87623285407659	-4.45943538360008	-0.67211561461148
Si	6.66786753724030	5.33086670563995	-0.91416044041690
Si	9.79481151067334	-2.60872233246691	1.56629177329256
Si	4.91423519099706	6.61070839024175	-3.11743250703887
Si	9.20262606646220	3.67529169269965	-1.66857291520554
Si	6.17236120243707	0.85152031701772	1.37284615578597
Si	1.52891376539523	10.52010183413350	-2.73573639490168
Si	9.79993996367698	0.68415618169001	-2.15388356441728
Si	7.95583737753784	-1.01667037646004	-0.30299762925404
Si	3.47619810900859	8.56937604120270	-1.20448275734919
O	2.98127193507282	-0.46588043144565	3.61459016532305
O	0.97328269850186	0.78237558647223	2.46698118274226
O	1.24421351125842	0.89640991970596	5.07501216493171
O	2.97549413278483	2.16889105244015	3.47718239071407
O	-0.39699388265701	1.20596181663567	0.28481190628646
O	-0.82087426491543	2.73767407591508	2.36428191766077
O	-3.76171623684623	4.99391729483968	0.56199617763815
O	-2.53593030559494	4.66226099327043	2.92021446398621
O	-1.17762528158210	3.44458920054282	4.84029343873524
Si	2.05537327735138	0.84900818154799	3.67561273050236
Si	0.31760073982075	1.93410935847662	1.54134018767710
Si	-3.13563295001314	5.74908064552340	1.86002511198223
Si	-1.10993332103648	4.00721068638376	3.33025006497437
O	8.28888894445871	-6.04333359523255	3.49607622886693
O	-3.21274917697152	-3.05609508031646	6.58939747159633
O	6.68114607044142	-7.84649957719949	2.44378817302876
O	-1.31588681214242	-1.56283414300307	7.69884067808962
O	8.25814441788258	-6.34275554023288	0.87193643934108
O	-5.42829920292380	-3.48636106358658	5.24471675246188
O	6.65176719096397	-3.92503996852049	3.57256131773421
O	-5.20477548334702	-1.23029008522770	6.62358055217268
O	-8.63476442844062	0.29489203758067	5.60118993400616
O	4.47516892980137	-2.51400197402320	3.25311929648487
O	-6.43516152029577	1.08360403727210	6.97738323028629
O	4.49212810011510	-5.06701003584428	2.60746522794955
O	-3.87150188197269	0.83312878703768	7.58159661475979
O	-2.25869973791393	2.84642450484784	7.14232681288785
O	0.39626297323261	2.65805566561898	6.86123832443558
O	4.25414231187204	-7.39948587892997	1.37489509232521
O	-1.49660229315715	0.87766393227594	8.78629894923215
O	0.71837704945004	0.07882847591960	7.52098688658128
Si	8.12638284243385	-7.12419182431140	2.28688862902247
Si	-2.13207809366884	-2.97049973400905	7.81407072962990
Si	8.19984610463937	-4.41711318828408	3.55236586385555
Si	-4.82148165903370	-2.80999087027249	6.58741560585117
Si	4.52967626740941	-0.89749062582956	3.37767407830074
Si	-8.07249834174572	1.12767322472414	6.88572049946645
Si	5.35975404910606	-3.70625591422039	2.61558013657970
Si	-5.30548867641621	0.07651068615904	7.57790445307926
Si	-0.94095072119035	3.48888903039283	6.44546104521807
Si	3.44285672753182	-6.09924626009920	1.92581859586696
Si	-2.74951956185024	1.78147987040717	8.26992888120290
Si	1.29533622094103	1.31883275182535	6.64703843544922
Si	5.41571968073416	-8.50887442445062	1.64930916732855
Si	-0.51619070497928	-0.38470436674109	8.47520802930316

O	1.82118362357196	4.28306380077451	-4.05019342637628
O	-0.07324673448925	5.28178035726865	-2.48818028153790
O	-0.56938404825173	7.72561901022760	-3.44277196991480
Si	0.70365271469982	3.93092887106926	-2.92961474148360
Si	-1.18906996250319	6.43949110403872	-2.66968185365625
O	1.26001426287540	-1.71894149880941	-4.52506567392177
O	-3.94552057022189	3.97397539613285	-4.57420075690527
O	3.34085533181907	-2.95650732369421	-3.48071638034584
O	-2.43915244310482	5.87665999038257	-3.53235033046103
O	-3.78470248002173	4.19234047027868	-1.94220605689544
O	1.33433504119175	-4.40500490301115	-4.41748480731936
O	-4.65033432869436	1.80629870120661	-5.94796847159598
O	4.72080912079386	-3.30724647398521	-1.25789104821787
O	-2.56471616674197	1.71716906877203	-4.38137372974649
O	-3.14938691599681	-0.34222308961704	-6.54648924323484
O	-1.11434626094762	-1.80809977837465	-5.66668998443321
O	-0.32976955169069	0.33453279626346	-4.25884196581608
O	-0.35471803873836	2.86964910425335	-3.53341528412026
O	1.79631138232736	6.93917247889943	-4.31294473319746
Si	-3.81515672439366	5.02886530302537	-3.33905946994188
Si	2.19650432374526	-3.04114079418574	-4.64236022651617
Si	-4.04865964632940	2.35000310554651	-4.55258226065240
Si	4.00827595622642	-4.07620676834634	-2.50753975032108
Si	-4.34351127264463	0.65466821661191	-7.06626897742196
Si	-0.29420880095498	-1.29071430176607	-4.36751186072427
Si	-1.20560290895492	1.49159482184997	-3.54883524528260
Si	2.56886139459481	5.56923013340165	-4.72426538281368
Si	0.66114552953445	8.08945221655377	-4.45716715457741
O	-2.79278866213888	-5.57959676842413	-7.11934819757148
O	-4.50573249717917	-4.33187375018459	-5.52500774610337
O	-2.93710636305817	-2.91159657581002	-7.22576867127990
O	-2.82989924137340	0.18819626917242	0.11024315497888
O	-2.45424642004305	2.80964612259296	-0.17808414048290
O	-1.60037261767297	1.02980212294757	-2.03600746673116
O	-5.10669197401639	2.80039787001535	-0.04301027277635
Si	-3.76922946393738	-4.28833388146800	-6.97407244362455
Si	-1.80279253187444	1.36316457945265	-0.48552140845685
Si	-3.80773564242340	3.70090332557614	-0.40601541876739
O	-5.04339060460543	1.95350939399784	-3.33528934026917
O	2.81619567025065	-5.05228177731908	-1.98253998023591
O	-5.46122727877495	-2.59223969683466	2.79194020792190
O	-7.32937262780938	-1.77112601736429	4.55278069777521
O	-0.25536005860354	-6.28330768067592	-3.39642491556498
O	-8.03500952158387	0.25856340144862	3.02325871080950
O	-0.38949051362885	-5.57410920296087	-5.96242835103619
O	-0.92658886675006	-1.91929640561086	-3.02442519396680
O	-3.49552230874959	-6.53127296557817	0.73687357392769
O	-0.97317828038817	-4.25030154772577	-1.84040478866586
O	-6.57962190859088	1.04346333455744	-1.41152726155588
O	0.62549391508023	-6.07521176103230	-0.86824138123983
O	-8.30449729352748	-0.64819908779490	0.51555338490012
O	-7.34451456029301	1.77831902598863	1.00469019898374
O	-1.87515920834774	-6.73482104582606	-1.39890671938552
O	-6.51597232986974	-0.21007511510172	-3.71814022942467
O	2.70793465547513	-5.41801852382935	0.64149414565857
O	-9.04641542552637	-2.64682332832376	-1.03531508845463
O	-4.38771185359872	-0.30510058115737	-2.13119577254518
O	-7.09496871697383	-3.03670532460586	0.74903954290848

O	-8.02490966058013	-2.38936289029328	-3.47578872873949
O	-5.37241317765250	-2.60411240910127	-3.73350403242193
O	-7.25622871180900	-4.37461231851369	-1.87030204044418
O	-4.99856830950031	-5.14758501441818	-3.06030014863532
O	-2.89308592830751	-3.60754268668755	-3.57804290870044
O	-5.70231025313761	-6.22285226917716	-0.73430596931987
O	-2.72990901152642	-2.43484554933997	-1.16718510676077
O	-4.78075120639726	-4.24519406578000	0.81492772665596
O	-4.85336759439064	-1.58167904719952	0.49060075522586
Si	-6.42734511953004	-3.02668798093345	4.05372216935788
Si	0.60004886048717	-5.82781673269153	-4.70363883118089
Si	-8.48234933779878	-0.65160615369653	4.29715304169087
Si	-1.24359738597590	-6.07030841069228	-7.25814907868195
Si	-6.63025761022646	2.28701365279715	-0.37142278337479
Si	-2.43716163195688	-7.49101204844733	-0.06000846599341
Si	-0.62356811879799	-5.82222097853903	-1.88201531093156
Si	-8.41993534626864	0.65306602193808	1.49063787845368
Si	-5.60542524289878	0.59009415626575	-2.63610540731940
Si	2.24864191560484	-6.01930658307538	-0.80936685333966
Si	-8.48799041444299	-2.26021109468129	0.43989189829912
Si	-6.69771199347902	-1.75596826545571	-4.17407816459712
Si	-8.51018583899877	-3.45025610836009	-2.34762622838907
Si	-4.43597472168357	-3.91431774431449	-3.96067559889079
Si	-6.25683157513569	-5.63020525932464	-2.14342061880387
Si	-1.91293044269124	-3.04808460985957	-2.38895623170718
Si	-4.97109769492137	-5.85759793417492	0.67731046527290
Al	-3.76887514971234	-1.14073054629606	-0.77428430736079
Si	-5.53838814110303	-2.85251657757280	1.16287805896981
Si	-2.11275210176325	-1.54563080420183	-6.91748964361114
H	11.67353999810775	-5.53749700147967	-0.03813000147160
H	9.19325800358002	-8.10610400280403	2.36365699723483
H	-3.96156899972460	1.36419899873061	-8.28506099869983
H	-5.56398599840276	-0.12270100181695	-7.29597600038031
H	-6.93632899750452	-1.73777199818019	-5.60525599894299
H	-9.61070200746851	-4.23261601014578	-2.91602401013438
H	-4.80937899802987	-4.38478700247749	-7.98416800209089
H	-1.26201400142072	-7.53013900000742	-7.34713300078904
H	-8.46716099481583	2.53175399339756	6.77435899888942
H	-4.20618400069117	6.50892100143352	2.49273000213510
H	-1.81213400100820	9.00646799783765	0.20177800005824
H	0.23145499914927	10.95012002811110	-2.10809600394671
H	-4.94421500116017	5.93630900170232	-3.34769400014389
H	-7.43440300522252	3.36541400606646	-0.94274899881133
H	-9.81225000649977	1.14342200259003	1.44886400266440
H	-3.26051300188642	2.50761900127839	9.43266799936948
H	-0.74472599723875	4.85510199830107	6.90777200216982
H	1.17261699991806	6.84873799836118	4.60176299826599
H	0.01371299822844	-0.85239400090336	9.75320300168483
H	2.65644499999305	1.61373199742213	7.09816899843723
H	4.53016699927638	3.47572500159231	4.86954700194003
H	1.53638200120386	-6.93834199838875	-4.97336599849880
H	4.68077899822589	9.17388299745826	-0.65420600254935
H	-8.67443700258693	0.53296800007187	8.08822300591684
H	-5.67876700057631	-0.35798099973831	8.92693200340820
H	-5.40538799566315	-3.45793999996381	7.74582399788034
H	-2.86030599852417	-3.03941600263526	9.10082100178314
H	-1.22727299600497	-4.11134499687663	7.70409200064534
H	2.59107099821740	5.38865099929417	-6.17172399793943

H	0.22684599761922	8.11439299835733	-5.85590800257897
H	2.35648400031432	11.66568599744226	-3.24752299790841
H	6.05893599847355	7.26486299970083	-3.76938199780740
H	10.01162899760712	4.23690500220038	-2.76123799955343
H	9.87912999881378	0.42295200080883	-3.59486199981512
H	11.16666299957539	0.78647299876443	-1.60521600215520
H	9.89955900178661	3.90245199952479	-0.38701600028216
H	-1.31531299973773	-1.20647000067075	-8.10616500224061
H	-0.61432200111026	-5.50729800128894	-8.46106599985540
H	2.84834100214902	-3.04402000250953	-5.95043800036422
H	-7.31528200901502	-4.15742501783862	3.75037498761904
H	-9.78237299219207	-1.30797899686997	4.07942699712343
H	-9.40254698729710	-2.75213299224165	1.45267099268992
H	-5.76896401131223	-6.40939900379409	1.78048401305476
H	-3.08137200539820	-8.75069600224102	-0.46757200571612
H	-7.02203599246017	-6.69280399513730	-2.78388199449974
H	2.78306900068388	-7.36900600353806	-0.99232700726409
H	4.89569299973479	-9.57645899364088	2.49579400073531
H	5.81992200304530	-9.04892400663881	0.34487199453275
H	-1.34472300627035	-7.77598298303606	0.87489001531218
H	10.66941399967774	-2.02071799879211	2.56381700143103
H	8.82291800274841	-3.95596100293158	4.78390499941761
H	11.59057499680958	-3.97495100232972	-1.85889599852663
H	5.33835300149837	-0.46465100074543	4.51989000341658
H	7.15208500117837	1.34023100139393	2.37194499744305
H	7.32230800084244	6.57802299833589	-0.51391500203776
H	5.00675699931055	-4.86530400142901	-3.24361800154210
H	7.90182200022592	-7.00149500060137	-1.50647500064948
H	2.44258799787953	-6.50026700934126	2.91141100860422
Ga	-3.25459938233955	-1.09128480559748	3.17606598863697
H	-3.53546158153170	0.28241419083792	2.33822356453476
C	-0.74749355715178	-1.98934949838614	3.80320354022765
H	-0.35209504688162	-1.01083715207221	4.07318023519419
C	-0.43748374563471	-3.04144174505370	4.57087813220598
H	-3.85537484251635	-0.98714099836286	4.59207475130783
H	0.22521056323547	-2.89274558807747	5.42282257677521
C	-1.60910526366470	-2.08111370192209	2.58262750891896
C	-1.73539308882994	-3.52809924347746	2.10207705047134
H	-1.18345610665287	-1.45171693381767	1.78784766516868
H	-3.09953833284663	0.24755520094544	1.10959942586649
H	-2.47002586148526	-3.62212830053637	1.29939382284990
H	-0.76593077467577	-3.83172408508425	1.67556040740788
C	-0.94024980562273	-4.43257566597972	4.30941428080861
C	-2.06789707412762	-4.45218193313057	3.27273550007250
H	-1.28136036511527	-4.88671954587777	5.24975844752969
H	-0.10161394994560	-5.05957874399193	3.96598279615707
H	-2.23990131377564	-5.47449978866321	2.91850431621294
H	-3.00142132070505	-4.11625357139724	3.74752539272794

I15-GaH 2

Electronic energy (r^2SCAN-3c): -30773.330022668622 E_h
Electronic energy (ωB97X-D4rev/def2-mTZVPP): -30781.695243901675 E_h
G_{corr} (673.15 K, 1 atm): 0.48330 E_h
Number of atoms: 258

Cartesian coordinates (Å)

O	1.50276811165561	2.92898576846529	1.03573956555626
O	-1.94987890359108	6.76762737449262	1.42802853383603
O	5.26272093484413	2.08045961171264	0.80918982146820
O	3.61447210480445	3.88931004090588	-0.16523610545442
O	6.10002368318050	4.57362827283298	0.41806883837840
O	1.44925568578865	3.28769908442574	-1.61395435939695
O	1.37931577646841	5.34385467692549	0.00871261902885
O	-1.69881748352843	6.88609137706135	-1.18544356279968
O	0.32354006527595	7.70698158119681	0.42478967009257
O	2.93554707000425	7.49283460339297	-0.03727096811026
Si	4.84744925427135	3.64407355152216	0.86733953796102
Si	1.99539387085515	3.86007163064755	-0.19729344505236
Si	-1.29376210435015	7.62850322965157	0.20993493808061
Si	1.65217013677730	6.80076137892874	0.66300248702552
O	8.94670177274811	-3.83052765714667	2.22487451747704
O	2.00677590521088	6.64346699570877	2.24324860883365
O	7.14266833382135	-2.27838461124479	-0.91740017337723
O	2.50423544302950	4.74819576077858	4.08820783752646
O	6.74873484961957	-4.90348572924716	-0.77039869846409
O	0.04489868612361	5.12691488078322	3.14932808030204
O	5.84676994575325	-3.27067471874296	1.13872965475947
O	4.44038837945067	4.08484328269364	2.38287366105581
O	9.40115563769885	-5.01147866685591	-1.12886246856398
O	5.15872809978798	-0.25113368817259	2.01700673655754
O	7.73551425605651	4.37917852426460	-1.69180567039309
O	10.70079541097710	-3.18521374305627	0.34607215172401
O	5.43122401411706	5.63135844475137	-1.91115165830977
O	4.09451404467815	5.67450575247525	-4.17662295336584
O	8.76258631960776	-1.45613302474728	1.04495675467574
O	3.91981154502619	7.73302029360846	-2.52671512384374
O	1.17318951798046	9.52076689275745	-4.04836601197251
O	8.98106726074037	2.07157372263936	-1.88629668974542
O	6.94787717995219	0.19512698000749	0.10150039357302
O	2.27227648976125	9.55481344169945	-1.56573708889145
O	8.99824011569056	-0.54791950637246	-1.44409951184248
Si	1.43524067708665	5.86251688841117	3.55512282030986
Si	6.11421323170522	-3.44026432897194	-0.44647833529619
Si	3.63727265355582	3.63281234247938	3.72620159568093
Si	8.07906907714209	-5.83607167931198	-0.65675888497481
Si	10.87603762920359	-4.45877775102258	-0.67194233769455
Si	6.67330865796991	5.32647626800529	-0.90972021029692
Si	9.79331456478517	-2.60648408843513	1.56632516045020
Si	4.92427464356680	6.59461015259612	-3.11812140475204
Si	9.20592452447605	3.67308517213288	-1.66786790797394
Si	6.17129857921943	0.85572693695397	1.37291517269970
Si	1.72917538689949	10.45336276826235	-2.82682763036959
Si	9.80031504216424	0.68288336331628	-2.15365466691687
Si	7.95444906561624	-1.01329192273056	-0.30172451519558
Si	3.46919231719344	8.52099600570074	-1.17797863052443
O	2.97590850383947	-0.46384938045069	3.60391975497623

O	0.96366012323655	0.81072603881363	2.49171758351735
O	1.25214703718570	0.88941883891080	5.08854942404349
O	2.99195451459763	2.16323962308359	3.48748563478006
O	-0.37426322984665	1.20267429642508	0.28535374379400
O	-0.82774576647879	2.76334999345145	2.33848102557448
O	-3.72411154092339	4.93804082360466	0.58741614108117
O	-2.53668200007523	4.67830047957611	2.96631018843988
O	-1.15610084335315	3.41937648420698	4.84100122501889
Si	2.05482124118598	0.85740075498356	3.68700872945186
Si	0.31761743391385	1.94581966891643	1.53729076564552
Si	-3.13326199999965	5.73664022973179	1.87511173181424
Si	-1.10932637718921	4.00611837409299	3.33853423512201
O	8.28572334816237	-6.04059527713647	3.49517333833253
O	-3.20421568800676	-3.07025688599259	6.59058117365398
O	6.68390057840804	-7.84918340159664	2.44526493082505
O	-1.31219816441525	-1.56659737926406	7.69383068640121
O	8.25753644461026	-6.34257472824179	0.87174620646460
O	-5.42192113606811	-3.48565618167376	5.24744819959529
O	6.64907399428335	-3.92040261254493	3.57474388903965
O	-5.13984789512096	-1.21876289273460	6.59100180892955
O	-8.66060582387321	0.33002679998360	5.58075309831594
O	4.46792827759306	-2.51086123952006	3.25172484395749
O	-6.44372709933574	1.05504036852334	6.95920181883240
O	4.48661000356155	-5.06396404408881	2.61582894918906
O	-3.88268298763601	0.85495210064635	7.58182618675618
O	-2.25569832910132	2.85204345656382	7.14580162903984
O	0.39867278646256	2.65615171592332	6.87774824657043
O	4.25868067197894	-7.39865031394247	1.37878252281605
O	-1.50471485339063	0.86993865886043	8.78411847214303
O	0.72363683967170	0.07918103741367	7.53373762511209
Si	8.12710166089822	-7.12330766218943	2.28713500776815
Si	-2.12730120312339	-2.97273091455381	7.81902334351711
Si	8.19725996583677	-4.41427206155299	3.55254444448270
Si	-4.80983553696990	-2.81202201756745	6.58940358460227
Si	4.52713445573213	-0.89305210345116	3.37587260817489
Si	-8.07923684774410	1.12844367153692	6.88000411849798
Si	5.35584709687842	-3.70400849087955	2.61870966708432
Si	-5.29925103119728	0.07132706585643	7.56889009727749
Si	-0.93413519391135	3.48567643817525	6.44692818768359
Si	3.44504947832419	-6.09997390462237	1.92783913483537
Si	-2.74860620720300	1.78687420630155	8.27027616484921
Si	1.29686093481070	1.31769127074242	6.65615980976114
Si	5.41868535559774	-8.50985279507416	1.64978810764647
Si	-0.51618890263818	-0.38667599601856	8.47553024438100
O	1.80766913776819	4.26804034298769	-4.05839608487306
O	-0.07254673266503	5.26097478475962	-2.47292757268850
O	-0.61264294648090	7.68272096508850	-3.46037474339624
Si	0.70228387558514	3.91145565220548	-2.92269864283818
Si	-1.20111071031533	6.40611382083395	-2.65195895915044
O	1.24270217156554	-1.73423153870087	-4.49166371809337
O	-3.97276120346277	3.95462147287742	-4.55022118132062
O	3.34386433136213	-2.95706101353797	-3.48343153637535
O	-2.45349461639635	5.82831308394799	-3.49646392823492
O	-3.81244149160382	4.16018939593764	-1.91138628167619
O	1.33911131245124	-4.41231367238353	-4.41622329378455
O	-4.65913477533305	1.78349987737543	-5.92653623786598
O	4.72012981756789	-3.30633892473804	-1.25752723647269
O	-2.58444312475198	1.70090139380525	-4.33913642132331

O	-3.16508813088822	-0.36407881669896	-6.53258356911027
O	-1.10760766573801	-1.81786317046971	-5.67201112108691
O	-0.34672196644990	0.32360392695216	-4.25277832556897
O	-0.36166364628596	2.84684525599463	-3.50882057612886
O	1.78226224007195	6.92560136581509	-4.27237327914484
Si	-3.83912350102027	5.00062979121399	-3.30657126069002
Si	2.19319461852794	-3.04317987014986	-4.63892657649102
Si	-4.06543787349665	2.33184465824498	-4.52834799430370
Si	4.00970338867940	-4.07520882556698	-2.50830456259766
Si	-4.32231981714313	0.66708157920996	-7.07066966468372
Si	-0.31250644301301	-1.29889455547571	-4.35784814753968
Si	-1.21806138529629	1.47054107077163	-3.51304544955344
Si	2.55674374689231	5.56508697829289	-4.71105997437770
Si	0.62702868950805	8.05095159050353	-4.45456058232526
O	-2.79562841204837	-5.58977588973664	-7.10636964519479
O	-4.55477062587138	-4.34256034294305	-5.55244966144139
O	-2.92965571335558	-2.92623626477460	-7.22004652631836
O	-2.72812183951102	0.01743663908559	0.14756620789676
O	-2.50213766741069	2.69390155909032	-0.16490495573016
O	-1.59654389117795	0.97944153867847	-2.01367467172350
O	-5.13273173051244	2.78084866905804	-0.01157395610010
Si	-3.77292007759017	-4.29738567296505	-6.97810510235858
Si	-1.81959129933395	1.24546787732229	-0.43948920276213
Si	-3.80733725464713	3.64238932261505	-0.37933376796014
O	-5.07625925207352	1.94327067920215	-3.31957845534621
O	2.81675061450829	-5.05074293037050	-1.98403462106364
O	-5.56275260918113	-2.55419556633602	2.81548053098288
O	-7.36766725249827	-1.78940123753468	4.64240122816652
O	-0.25029183749012	-6.29390638456485	-3.39735514690976
O	-8.01408444164950	0.19385226436197	3.00534152565942
O	-0.38813648015040	-5.57504656550531	-5.96248894194863
O	-0.98450568458585	-1.92379514754061	-3.02628158360533
O	-3.48529413929120	-6.51099057572977	0.73583256687338
O	-0.98599218809794	-4.25868107809605	-1.85794535064761
O	-6.63202601536055	1.04278321526112	-1.39934100464257
O	0.62684449475093	-6.06584752204601	-0.86761492913006
O	-8.36528013244373	-0.62439867092102	0.48097022682661
O	-7.35986007836598	1.77771953216406	1.02533280918231
O	-1.87118863952771	-6.74343093020762	-1.40086248293745
O	-6.54564431440098	-0.21660416557230	-3.70944775032691
O	2.71994942203166	-5.42042588024410	0.63751764369996
O	-9.07303299288944	-2.65728541251618	-1.04220689748620
O	-4.45573692029464	-0.31216155028358	-2.09217575372463
O	-7.02560224403969	-2.90976433857582	0.61925462070611
O	-8.01755437809784	-2.40554305571906	-3.48066401038673
O	-5.35684597137286	-2.59961728304904	-3.74510574366453
O	-7.26726530286409	-4.38030074358791	-1.86389441663244
O	-5.01134048892509	-5.14662676526194	-3.07305064826433
O	-2.88080706649266	-3.66887908348448	-3.66401271978675
O	-5.69026534690431	-6.18843401925120	-0.72754624962240
O	-2.87038478198056	-2.54737086958272	-1.25801029657710
O	-4.78063291891932	-4.22664408244913	0.87099992453639
O	-4.66999540915719	-1.55718030069529	0.65110410637273
Si	-6.46180716554223	-3.02240611282108	4.09394889383259
Si	0.60127411582696	-5.83154787021021	-4.70503678881885
Si	-8.49055505587244	-0.65726801224156	4.31086316225905
Si	-1.24346972340388	-6.06902732630292	-7.25815866571917
Si	-6.65024223346797	2.28587783414258	-0.35488992003148

Si	-2.43700981207519	-7.48891071115550	-0.05717457296578
Si	-0.62186537335952	-5.82946876670061	-1.88574007003545
Si	-8.43253435864905	0.64711849850052	1.49608614095687
Si	-5.65538738195654	0.59227431885344	-2.62040225909206
Si	2.25143795155785	-6.01802904495903	-0.81080925639207
Si	-8.49649953834455	-2.23826359427469	0.41536506582311
Si	-6.69411546050689	-1.76185675466548	-4.17683530885790
Si	-8.51516415804211	-3.45530729509138	-2.35041307787024
Si	-4.44745208360165	-3.92843321127976	-3.99103188812257
Si	-6.25929163239594	-5.62598046080667	-2.14318460499256
Si	-1.95293182231369	-3.09525223287171	-2.44170621452432
Si	-4.96186106586648	-5.84245886082658	0.68961445488238
Al	-3.72201176642037	-1.14079860306364	-0.80891654906934
Si	-5.52508710288119	-2.83012509584295	1.21145275663283
Si	-2.11554930986194	-1.55323796490202	-6.91422678390906
H	11.67374700311426	-5.53703600161094	-0.03807400344056
H	9.19348298964011	-8.10585798627139	2.36346299789104
H	-3.88155000544783	1.39981099756106	-8.25725798813924
H	-5.54661899506108	-0.07985099413357	-7.37196299848122
H	-6.93206199396053	-1.73335099315026	-5.60666699183952
H	-9.61083499878739	-4.23371599452055	-2.91527900547433
H	-4.80806201594046	-4.38616400451688	-7.98466800625495
H	-1.25558099205886	-7.52827102213784	-7.35483799501596
H	-8.46603468467859	2.53218812173632	6.77163474757210
H	-4.20950414090803	6.50518809343944	2.49497307363691
H	-1.81143197728226	9.00591892728894	0.20164700665517
H	0.67612807431253	11.32783113776928	-2.29765503744343
H	-4.95337002177163	5.93157201335490	-3.34598500046624
H	-7.43212793526600	3.36467291969506	-0.94325489616976
H	-9.81433500577158	1.14100199441181	1.45030700234759
H	-3.26082300292529	2.50672900527715	9.43327002132235
H	-0.74504101034079	4.85494997646298	6.90563300271163
H	1.17176499075592	6.84606699164701	4.60638300812210
H	0.01383699759962	-0.85268899615527	9.75320600018668
H	2.65630400169864	1.61378699990802	7.09828800542953
H	4.53076898531681	3.47379800133455	4.86924397872225
H	1.53720799816737	-6.93798299693291	-4.97365699681916
H	4.63541500055972	9.21535600066599	-0.64625199987545
H	-8.67488802837374	0.53574593339421	8.08886500249040
H	-5.67875099259513	-0.35844198506574	8.92691793194945
H	-5.40516100610556	-3.45826601270867	7.74597000720250
H	-2.86007300269512	-3.03986699953760	9.10083200860470
H	-1.22700998919559	-4.11162699604958	7.70400899494182
H	2.57413299934595	5.42807899425019	-6.16363198705393
H	0.22482900096536	8.10275899384373	-5.85603198118089
H	2.79507593487917	11.25689298204874	-3.42192596554150
H	6.06067100386399	7.26917500187526	-3.76691099992415
H	10.01064999767368	4.23779399639145	-2.76113099846117
H	9.87926499409375	0.42371400127820	-3.59460900497413
H	11.16639499987934	0.78744100014433	-1.60472500109980
H	9.89847199906906	3.90341200229981	-0.38688400188687
H	-1.32399900181668	-1.19594401087165	-8.10330098882937
H	-0.61662901337487	-5.49814399560440	-8.46011599568093
H	2.84818300398600	-3.04332200182189	-5.95038398783412
H	-7.31517901623650	-4.15812802799424	3.75060699894039
H	-9.78250007921801	-1.30915805203246	4.07996999134169
H	-9.40319703736377	-2.75383201703428	1.45013703163228
H	-5.76898901212610	-6.41021300022119	1.77986101355152

H	-3.08084504553069	-8.75083001672700	-0.46797103804940
H	-7.02180600036512	-6.69287799595412	-2.78419799669371
H	2.78358999134110	-7.36886299976774	-0.99247300525710
H	4.89691600523570	-9.57738000630009	2.49409399153788
H	5.82084499700677	-9.04713700034797	0.34374501046603
H	-1.34437694973696	-7.77605498037290	0.87467404041822
H	10.66950898589964	-2.02029700830846	2.56380699077993
H	8.82314099989826	-3.95574100018629	4.78387700548540
H	11.59079999371475	-3.97453400257599	-1.85887999267949
H	5.33815599291712	-0.46451101158651	4.51995499768142
H	7.15219500755589	1.34004700458691	2.37234700500459
H	7.32251199877980	6.57841100188686	-0.51330699869405
H	5.00684300481818	-4.86484800466688	-3.24385800359632
H	7.90214800220576	-7.00114299756319	-1.50659200007325
H	2.44258299494880	-6.49974698299837	2.91143200484448
Ga	-3.16044630805423	-0.84246932015831	1.95602251093407
H	-5.32578836905165	2.82246007387810	3.97652676112649
C	-0.75572755086464	-3.30518107899936	4.06305837602858
H	-0.79313901166388	-3.67353135905120	5.08706366477267
C	-1.89723386037371	-2.97178297568681	3.45371695050949
H	-3.75206702143996	0.28971003074958	2.82519271366059
H	-2.83439332541631	-3.11076121514403	3.99038141967780
C	0.58615773007278	-3.25805960659555	3.39063474454968
C	0.44461481381729	-3.17843016426466	1.86970959463154
H	1.16599093277657	-4.14770284907968	3.67431931245303
H	1.16886689390608	-2.40184020152244	3.76230189347489
H	1.41427259138558	-2.95959872650149	1.40662663735862
H	0.11386700223258	-4.15080151658952	1.47927283488247
C	-1.96450788571371	-2.42926294778463	2.05920431382977
C	-0.57407568501147	-2.10349524164060	1.48792283851666
H	-5.81602788595885	2.60302368220921	3.46153481972466
H	-2.45583960991701	-3.17131774943321	1.40985082969752
H	-0.63060560011543	-1.99851275219479	0.39854045750345
H	-0.21224918308662	-1.14698623373332	1.88037149632958

I16-GaH 2

Electronic energy (r^{2}SCAN-3c): -30772.157768752229 E_h
Electronic energy (ωB97X-D4rev/def2-mTZVPP): -30780.511225200360 E_h
G_{corr} (673.15 K, 1 atm): 0.47833 E_h
Number of atoms: 256

Cartesian coordinates (Å)

O	1.47841956682908	2.95189586717759	1.03250186741422
O	-1.95107474460760	6.75930185483934	1.40983996594323
O	5.26557026583616	2.07576291252041	0.80413041481578
O	3.61204270793400	3.87823892308481	-0.16406076899858
O	6.09803166787743	4.57047619533272	0.41124765546554
O	1.44176944391763	3.30078939312357	-1.61460812135833
O	1.40264697528260	5.36768040933093	-0.01078477712006
O	-1.71015307711092	6.90269123500117	-1.20591911157910
O	0.31177561168581	7.71657609657992	0.40864797241996
O	2.90388584660954	7.56072207268684	-0.06086534238292
Si	4.84913896393585	3.63828315361100	0.86453227478437
Si	1.99342401083422	3.87195852136365	-0.19971846901913
Si	-1.30296448228013	7.63063531382214	0.19617872425647
Si	1.65086518328248	6.82706688509206	0.64699940309912

O	8.95054857553103	-3.83391391342525	2.22526084284895
O	1.99931190348520	6.65972199496623	2.22925633011569
O	7.14616251813983	-2.28075523253585	-0.92216620105870
O	2.50840047963177	4.75675674293738	4.07106663422545
O	6.74878019477446	-4.90510794953717	-0.77132651295615
O	0.04802178209322	5.12100482535293	3.13309537368824
O	5.85427543030097	-3.26851585862732	1.13767408381651
O	4.44773034667289	4.07821463041343	2.38155757189773
O	9.40133696234596	-5.01179308014382	-1.12871884834843
O	5.15656344499656	-0.25133472542657	2.01893824695638
O	7.73146888321084	4.38390304442586	-1.69820685936619
O	10.70291137757899	-3.18590841554228	0.34614957463489
O	5.41985293558639	5.65273451034150	-1.90269885835927
O	4.09727431412015	5.67920546103233	-4.17775989222427
O	8.76282624601953	-1.45988918951361	1.04409106540767
O	3.89266212763296	7.74678768668344	-2.54398645848317
O	1.23875483588392	9.54795178099084	-4.03230618542094
O	8.97969198250182	2.07326593307124	-1.88688203924396
O	6.94755849791412	0.18982629765944	0.10276246538916
O	2.35940592453453	9.69427308815744	-1.58162697753304
O	8.99932078184434	-0.54667013841851	-1.44349617240232
Si	1.43462703517007	5.86845431725209	3.54063877716719
Si	6.11687123948265	-3.44055671874909	-0.44788777863264
Si	3.63625714416745	3.63055411536642	3.72177062229148
Si	8.07945816270109	-5.83671129497609	-0.65672685674617
Si	10.87634221704781	-4.45932185062912	-0.67201576293933
Si	6.66841426589095	5.33080620710863	-0.91362128756804
Si	9.79460152009581	-2.60843438744889	1.56633399247655
Si	4.91259273502546	6.60941931433566	-3.11675018844719
Si	9.20250734578164	3.67570963794401	-1.66858820891841
Si	6.17144065806679	0.85197290777117	1.37357796414857
Si	1.52813535550227	10.51987719400010	-2.73614365949624
Si	9.79986510870324	0.68460797712063	-2.15386787683405
Si	7.95568999926695	-1.01582377607480	-0.30272074453347
Si	3.47572144791852	8.57078612727713	-1.20329901147421
O	2.97926011678208	-0.46821092860366	3.61615775647299
O	0.98557470302714	0.78859270814114	2.45324728625876
O	1.24278579473855	0.90353640580442	5.07118799089317
O	2.98294750140133	2.16467851878296	3.47880944365123
O	-0.38890886597964	1.20920852713681	0.27337062879832
O	-0.82189199721886	2.72928288874141	2.36553007672681
O	-3.77030924665209	4.96539086876896	0.57830433949273
O	-2.53392942469718	4.66168457173878	2.93019188762447
O	-1.16966387089316	3.43934739719801	4.84068113063088
Si	2.05385720114429	0.84859039289881	3.67136825351320
Si	0.31820150102066	1.93676096677628	1.52921933900429
Si	-3.13952262997592	5.73844774034910	1.86237230075315
Si	-1.10744340295455	4.00120979717054	3.32929358225798
O	8.28961398573386	-6.04346132625001	3.49616368584911
O	-3.20514163961658	-3.07700850865562	6.58976431555957
O	6.68103649760241	-7.84612664377806	2.44389554514519
O	-1.32807623004001	-1.55812689629714	7.71074391639086
O	8.25807658110232	-6.34301452344636	0.87180635869419
O	-5.43258216671801	-3.49445915037097	5.25795385238468
O	6.65131639424408	-3.92671347154434	3.57306529944446
O	-5.13584320523599	-1.21943726747961	6.58957054644818
O	-8.66046741839579	0.33830029359437	5.57461896496894
O	4.47534822524549	-2.51363397191634	3.25289655467602

O	-6.44270295340248	1.05250779987513	6.95741230922515
O	4.49124968237598	-5.06588286112209	2.60591280947619
O	-3.87939367761399	0.85187311708595	7.58526536930546
O	-2.25687808881050	2.85173118248965	7.14292652554168
O	0.39724064977548	2.65745542297114	6.86805048181561
O	4.25403218216984	-7.39955262063597	1.37414449900793
O	-1.49814189025482	0.88126015876975	8.78816297299969
O	0.71257526116501	0.07701883938939	7.51471550074502
Si	8.12661327850863	-7.12397071560609	2.28693439792269
Si	-2.13102522545554	-2.97284456786243	7.81922961083498
Si	8.19984974985024	-4.41711360446341	3.55242503709496
Si	-4.80867809001836	-2.81376897168507	6.59019012210324
Si	4.52779110235263	-0.89621126083634	3.37841877555422
Si	-8.07875126217427	1.12779632885537	6.87956102531828
Si	5.36006183705890	-3.70527019026676	2.61575363880478
Si	-5.29722987550905	0.06975541677504	7.56856387275506
Si	-0.93684023132545	3.48940795249983	6.44480864520102
Si	3.44298614126567	-6.09952571588903	1.92576901681334
Si	-2.74752712186503	1.78911479148160	8.27036131134792
Si	1.29543963786497	1.31908175072929	6.64362989274474
Si	5.41597348863442	-8.50872309387603	1.64933071125840
Si	-0.51715343843305	-0.37985366535526	8.47723816076053
O	1.80960570997483	4.28098690089683	-4.05692610796952
O	-0.07014853801214	5.28365685460906	-2.48245622628208
O	-0.57719538305292	7.71916292639614	-3.45000745752673
Si	0.70207621853172	3.92979593470393	-2.92416043559384
Si	-1.19183296145562	6.43416066473744	-2.66981283348330
O	1.25645451619285	-1.72427449185879	-4.50717603590256
O	-3.95865559374521	3.95807543734024	-4.55509535751348
O	3.34385729465793	-2.95803256055564	-3.48179645286500
O	-2.44064946129192	5.86047139114032	-3.52732579206668
O	-3.78879884203664	4.18794954660479	-1.92593453530431
O	1.33565625504928	-4.40758369626013	-4.41532986692737
O	-4.65504321827501	1.79397704174898	-5.93189651184552
O	4.72143827853547	-3.30746217217149	-1.25674339680994
O	-2.55745716920518	1.70831254352415	-4.38051891854122
O	-3.15062262240517	-0.34394281774320	-6.54763031576293
O	-1.10378422377827	-1.79449916666897	-5.66983976511464
O	-0.32419797602773	0.33448694774017	-4.23101393592030
O	-0.36210606528833	2.86614440440091	-3.51053774462367
O	1.79508432389787	6.93875068547553	-4.31208946684932
Si	-3.81992756771787	5.01899410623584	-3.32491571667459
Si	2.19630503122966	-3.04196214593930	-4.64002679671050
Si	-4.04464398844559	2.33365991246419	-4.53704771547112
Si	4.01011662963413	-4.07612398793501	-2.50681592077631
Si	-4.34464451419675	0.65530300552624	-7.06282550135282
Si	-0.29621078087670	-1.28662732473054	-4.35923127729541
Si	-1.21132857345252	1.48657865374202	-3.51983832874579
Si	2.56442342540918	5.56673346284969	-4.72343787677608
Si	0.65784135336541	8.08677778262640	-4.45683423371322
O	-2.79336116812872	-5.58204818709384	-7.11403853965814
O	-4.54165226428722	-4.34577812397834	-5.54417305952906
O	-2.93405138527877	-2.91561738071651	-7.20823098334160
O	-2.79947702597699	0.12549236573210	0.14848674321645
O	-2.47164255955404	2.76948284653855	-0.16339228707725
O	-1.63240883292928	1.01780665155602	-2.02265431299251
O	-5.11726555696112	2.77809531271155	-0.04830568104187
Si	-3.77210653482504	-4.29183743105115	-6.97600661956994

Si	-1.83592774556996	1.30165663697425	-0.44777369113420
Si	-3.80714574172173	3.67301036827927	-0.39487025624962
O	-5.02167065337866	1.92400314414210	-3.30734114182090
O	2.81733647754420	-5.05193995369771	-1.98206229296361
O	-5.56452773517403	-2.55137678455516	2.83361292176101
O	-7.37349199031582	-1.78922683172461	4.65177040889190
O	-0.25345849444738	-6.29213657519407	-3.40078754881996
O	-8.00964552994564	0.18789421930568	2.99878503103622
O	-0.38717007733163	-5.57292486845263	-5.96446933716973
O	-0.95365626155712	-1.92608435195712	-3.03001016742097
O	-3.48752149970824	-6.51517209382202	0.73346624184602
O	-0.98974205420871	-4.25999052992693	-1.85647227710756
O	-6.63834820276956	1.05352974678636	-1.42263148794205
O	0.62394484046054	-6.06878429649427	-0.87117897613938
O	-8.37670804401940	-0.61834870472598	0.47273991729011
O	-7.34421155264452	1.77080361677527	1.01564043673010
O	-1.87276386738847	-6.74478718232171	-1.40334191477337
O	-6.53359337729875	-0.20324171766320	-3.73534919034889
O	2.70509438084838	-5.41911392331193	0.64213506513521
O	-9.07024220483439	-2.67198258440308	-1.02840420660415
O	-4.49421526625226	-0.36354975847656	-2.07384763454229
O	-6.99519688094937	-2.86874319517573	0.61389742854722
O	-8.02212809028004	-2.38338738807317	-3.46349350875894
O	-5.36937231664778	-2.60350268553021	-3.74744659213528
O	-7.26358400167947	-4.37724331081334	-1.87069953780009
O	-5.00373904176377	-5.14541964420295	-3.06746989070622
O	-2.88680595007475	-3.64103262047748	-3.64300975931346
O	-5.69353091155455	-6.18687422413945	-0.72651828093277
O	-2.81598105323309	-2.49821627267349	-1.23820560135749
O	-4.78100644872307	-4.23142065429490	0.87964967346189
O	-4.59088988740913	-1.57758213955964	0.70604875901420
Si	-6.46803436049044	-3.02305483325401	4.10586868674388
Si	0.60150657268612	-5.82891746706551	-4.70609767233572
Si	-8.49103666318659	-0.65464743618902	4.30853926159712
Si	-1.24375896357444	-6.07028954208301	-7.25804165836859
Si	-6.64363777041387	2.28681832236416	-0.36717374668886
Si	-2.43650048058245	-7.49001400555119	-0.06019738420377
Si	-0.62408703138045	-5.82901002327377	-1.88875345266070
Si	-8.42768474249227	0.65126039166359	1.49114793710878
Si	-5.64778641454248	0.58362126198101	-2.62511313993511
Si	2.24827309056083	-6.01858854625357	-0.81000320813724
Si	-8.48636622462989	-2.23395092470583	0.42061122020829
Si	-6.69916969535343	-1.75545797520662	-4.17687102269125
Si	-8.51501222907749	-3.45188350013263	-2.34778958288712
Si	-4.44607387108176	-3.92198489993544	-3.98427770644726
Si	-6.25737366169318	-5.62468855155418	-2.14514442296781
Si	-1.93608248782612	-3.07712351899387	-2.43376916824339
Si	-4.96394947118392	-5.84456775657782	0.68981183502549
Al	-3.70062737041578	-1.11734154856391	-0.78033447760469
Si	-5.50190811924722	-2.82742120894525	1.23333959958183
Si	-2.11088962879449	-1.54451161786674	-6.91541578298239
H	11.67354000353074	-5.53749700665284	-0.03813000113902
H	9.19325798466774	-8.10610399279567	2.36365699775449
H	-3.96156899439296	1.36419899872810	-8.28506100354010
H	-5.56398600368815	-0.12270100717160	-7.29597600024989
H	-6.93632899725800	-1.73777200339321	-5.60525601073859
H	-9.61070202474070	-4.23261601656931	-2.91602401645196
H	-4.80937899800599	-4.38478700746720	-7.98416800173056

H	-1.26201399632968	-7.53013901556135	-7.34713300572950
H	-8.46716100414752	2.53175400632232	6.77435899828799
H	-4.20618400037179	6.50892100137479	2.49273000207400
H	-1.81213399583825	9.00646799297461	0.20177800004662
H	0.23145498851512	10.95011916905336	-2.10809602884289
H	-4.94421500654068	5.93630901250906	-3.34769399991814
H	-7.43440302192810	3.36541403348701	-0.94274900371005
H	-9.81224999125742	1.14342200290240	1.44886400795950
H	-3.26051299193272	2.50761898529303	9.43266798335090
H	-0.74472599769918	4.85510199970924	6.90777200228135
H	1.17261699996724	6.84873799852620	4.60176299831649
H	0.01371299298822	-0.85239400139524	9.75320299731738
H	2.65644499751446	1.61373199906541	7.09816899966181
H	4.53016699392071	3.47572500163854	4.86954700198512
H	1.53638199624069	-6.93834199275998	-4.97336599798404
H	4.68077900329928	9.17388299229762	-0.65420600267486
H	-8.67443701270228	0.53296798536383	8.08822302092892
H	-5.67876700635545	-0.35798100456057	8.92693201262544
H	-5.40538800088749	-3.45794000068684	7.74582399722719
H	-2.86030599486940	-3.03941600747675	9.10082099239361
H	-1.22727300497474	-4.11134499293354	7.70409200548979
H	2.59107099803103	5.38865099408815	-6.17172400321268
H	0.22684599754208	8.11439299319795	-5.85590800234392
H	2.35648400054839	11.66568598711780	-3.24752299282660
H	6.05893599831766	7.26486299994039	-3.76938199771180
H	10.01162899767686	4.23690500249943	-2.76123799974831
H	9.87912999931029	0.42295200124969	-3.59486199489319
H	11.16666299999362	0.78647299908677	-1.60521600233840
H	9.89955900172802	3.90245199954186	-0.38701600037681
H	-1.31531300502823	-1.20647000577352	-8.10616499690388
H	-0.61432200638749	-5.50729800642808	-8.46106599953600
H	2.84834100748827	-3.04402000194982	-5.95043801243608
H	-7.31528200031365	-4.15742498974223	3.75037500511394
H	-9.78237301549917	-1.30797901093002	4.07942699274621
H	-9.40254699993905	-2.75213299772878	1.45267099934321
H	-5.76896399123069	-6.40939899333722	1.78048399187586
H	-3.08137199617831	-8.75069599397927	-0.46757199005409
H	-7.02203599357424	-6.69280399080244	-2.78388199002044
H	2.78306898283621	-7.36900596807450	-0.99232701245458
H	4.89569298619159	-9.57645900414911	2.49579400743020
H	5.81992197756617	-9.04892398536630	0.34487202120251
H	-1.34472301872453	-7.77598299867243	0.87488999022713
H	10.66941399509711	-2.02071801369820	2.56381700135435
H	8.82291798795949	-3.95596101774060	4.78390498879926
H	11.59057499755422	-3.97495100209207	-1.85889599404197
H	5.33835299684535	-0.46465100066399	4.51989000301374
H	7.15208500704023	1.34023100198932	2.37194500276466
H	7.32230800100338	6.57802299323469	-0.51391500204157
H	5.00675701626317	-4.86530401162092	-3.24361801427485
H	7.90182200042547	-7.00149500093285	-1.50647500129417
H	2.44258815267633	-6.50026711379485	2.91141105960686
Ga	-3.27560334665626	-0.61568689739813	2.02291326359643
C	-1.06461524629892	-1.52529066923549	3.71106204767554
H	-0.84972591049811	-0.51128206033491	4.04635189017281
C	-0.75102606936696	-2.54657560524417	4.51320647561457
H	-4.13783664694350	0.46425008197743	2.72217536725496
H	-0.27030384247094	-2.33250365497137	5.46573751654085
C	-1.61738037505257	-1.68720361776367	2.33143532520203

C	-1.63683287936520	-3.16036306789347	1.88497184084665
H	-0.95402045026813	-1.12779142837988	1.65098330620800
H	-2.27550431148545	-3.31264157073793	1.01113668420348
H	-0.61852846655115	-3.43475926228294	1.56922171279757
C	-1.03802026898310	-3.98016997406638	4.17361140387522
C	-2.04894781703557	-4.07937950583949	3.03281366638213
H	-1.41208823785243	-4.50519048554524	5.06222030824708
H	-0.10237881488110	-4.49069755481371	3.89356931778838
H	-2.13461952130997	-5.11259686749604	2.67849119999308
H	-3.03914074497219	-3.78362909011646	3.40982110872986

I17-GaH 2

Electronic energy (r^{2}SCAN-3c): -30772.127406662563 E_h
Electronic energy (ωB97X-D4rev/def2-mTZVPP): -30780.479165551365 E_h
G_{corr} (673.15 K, 1 atm): 0.46377 E_h
Number of atoms: 256

Cartesian coordinates (Å)

O	1.48052963647815	2.92810730094610	1.03173740166638
O	-1.94641119089288	6.76573614322426	1.41464186587547
O	5.26827586986739	2.07519989529469	0.79969322817021
O	3.60977478552175	3.87881139556766	-0.15842759993662
O	6.09638915240764	4.57207185555716	0.41261713784480
O	1.44527083763098	3.30220560774695	-1.61158692924548
O	1.39455094086980	5.35567705476089	0.00908935104996
O	-1.71526238410953	6.90407273819152	-1.20534446302410
O	0.31393804493551	7.71498510688373	0.39986957038132
O	2.90344104387584	7.54805156165090	-0.07122905049247
Si	4.84961485414870	3.63681952427430	0.86573328669637
Si	1.99113710953127	3.86305756707921	-0.19056076275095
Si	-1.30171990546875	7.63194204480042	0.19530267548857
Si	1.65030888649108	6.82305669474485	0.64626782489793
O	8.95043779910694	-3.83404404823246	2.22529306986166
O	1.99899002777476	6.67038740891121	2.23018586710254
O	7.14556870499571	-2.28230881197289	-0.92171859843196
O	2.51451580328728	4.76512315593666	4.07016025995501
O	6.74751343273744	-4.90538104515340	-0.76574733486298
O	0.05400306726780	5.11990771124005	3.12952370405917
O	5.84745943807798	-3.26226117499076	1.13782665825237
O	4.44939570209942	4.06984025406863	2.38453642257583
O	9.40013340711690	-5.01069496639552	-1.12886997654491
O	5.15568823851789	-0.24929179478673	2.01780280552527
O	7.73062139353705	4.38323242519170	-1.69688537751165
O	10.70364760520630	-3.18632147530169	0.34643657459571
O	5.41920043092297	5.65375045231043	-1.90192588356611
O	4.09330383800267	5.67530169306310	-4.17395348839721
O	8.76363283117346	-1.46034172172199	1.04354009370998
O	3.89546498536548	7.75050945611858	-2.54845921267410
O	1.23926473364758	9.54756096766513	-4.03184343256097
O	8.97969280445523	2.07297578351758	-1.88667746567169
O	6.94865802841549	0.18850502125304	0.10213036167597
O	2.35915357831436	9.69274252030950	-1.58054457667768
O	8.99920072056925	-0.54776203619938	-1.44448945567272
Si	1.43743771245211	5.87245198816848	3.53922758786571
Si	6.11417667724254	-3.44059650840092	-0.44650396928007
Si	3.63339389627512	3.62926830308025	3.72421507040528

Si	8.07889061970612	-5.83685446902423	-0.65558332045706
Si	10.87569457149518	-4.45968582681767	-0.67223726987092
Si	6.66770235667004	5.33093788296395	-0.91282235173057
Si	9.79494534352351	-2.60917390398055	1.56641786861351
Si	4.91212619764431	6.60851195579677	-3.11765044846076
Si	9.20216325623458	3.67546314359740	-1.66843636926622
Si	6.17301881007074	0.85201931779551	1.37181901800781
Si	1.52860908842987	10.51927171910599	-2.73534199434939
Si	9.79975291725122	0.68406393472441	-2.15380058796732
Si	7.95615340449372	-1.01789459501581	-0.30338408207584
Si	3.47604540008074	8.56842001779203	-1.20492253566469
O	2.98242668449925	-0.46317957668719	3.61952660270484
O	0.97417514133418	0.77468422318871	2.46421877184576
O	1.23830855851054	0.88966028341742	5.07955852827105
O	2.96524779467191	2.16934600060392	3.48687306124845
O	-0.39539056467591	1.20024278014571	0.27496529965599
O	-0.82240038110508	2.73061334147503	2.36102070475869
O	-3.75946443106950	4.97522247899803	0.57451475041232
O	-2.52978335870794	4.66397154192170	2.92955725321482
O	-1.16778076523784	3.44229703484114	4.83918404817780
Si	2.04451898043611	0.84623258688181	3.67857218485956
Si	0.31362991207292	1.92032586897023	1.53405595777425
Si	-3.13436778092389	5.74340784955701	1.86420234525017
Si	-1.10255550124695	4.00172996400068	3.32682409405491
O	8.28370333830605	-6.04168407747128	3.49557576855435
O	-3.19759570528474	-3.07291043541978	6.58013710236555
O	6.68135335519683	-7.84815004335741	2.44259284640758
O	-1.31147826095012	-1.56719264354772	7.69178445986545
O	8.26185031273570	-6.34425818075866	0.87176862739095
O	-5.42694185572688	-3.48629898903547	5.24725194340333
O	6.65062801121370	-3.91758151961510	3.57197578449795
O	-5.13090825228672	-1.22049041706713	6.59065907885009
O	-8.67183798561326	0.34399912162417	5.57441080477393
O	4.46997291350528	-2.51033500677244	3.25382068402065
O	-6.44688668708387	1.04584365034751	6.95077863123080
O	4.49239337185551	-5.06447590045878	2.60946481047522
O	-3.88682663715030	0.86013873930129	7.58050367633679
O	-2.25297816642959	2.84911676207381	7.14286859927109
O	0.40026833057297	2.65944712055623	6.86629392777823
O	4.25372233462581	-7.39362165448559	1.37881201580296
O	-1.50890485937232	0.86662617900862	8.78492168633053
O	0.72103270967515	0.08395993550802	7.53114413240841
Si	8.12535392035945	-7.12454025494238	2.28705510609938
Si	-2.12743569475556	-2.97318825661628	7.81680913218900
Si	8.19726536491213	-4.41528062278258	3.55222472047269
Si	-4.80447868293252	-2.81533259331160	6.58611688464293
Si	4.52968090299693	-0.89286173611160	3.37848160733859
Si	-8.08245994494529	1.12786649659432	6.87903424042954
Si	5.35611545073247	-3.70309385994443	2.61615054496719
Si	-5.29920875295692	0.06950044738798	7.56775525561306
Si	-0.93427533244005	3.48955185999834	6.44347824460243
Si	3.43711802391753	-6.09125800291353	1.92642748840051
Si	-2.74861789219840	1.78785885670330	8.26927676441674
Si	1.29626516367029	1.31832405711383	6.64677828388647
Si	5.41222859694121	-8.50595691204770	1.64885595729638
Si	-0.51567803664990	-0.38596647297400	8.47419356924423
O	1.79997381378225	4.28000825742713	-4.05817535286807
O	-0.06984309170773	5.28668270148963	-2.47496021269849

O	-0.57732072387580	7.72013796712674	-3.44851199617327
Si	0.69979868983899	3.93097196446691	-2.91676988030939
Si	-1.19252186303618	6.43565834124230	-2.66797874380678
O	1.24886773549039	-1.73241689145977	-4.49381249818870
O	-3.95856210511394	3.95495397387164	-4.55187808800627
O	3.34362691148936	-2.96018775464191	-3.48383124112283
O	-2.43913126408399	5.86123972315220	-3.52913062949576
O	-3.77523837151125	4.18635471117919	-1.92549275778967
O	1.33290063009363	-4.41233365938912	-4.41514867034324
O	-4.65405657784152	1.79113245293038	-5.92715719305880
O	4.72040224511612	-3.30717769190518	-1.25816627056374
O	-2.55163723059081	1.70572650325673	-4.38076337842605
O	-3.15182580383683	-0.34484236584560	-6.54839786863925
O	-1.10511646961518	-1.78678509186947	-5.66420772897639
O	-0.31887745909317	0.33555160181319	-4.21259350759533
O	-0.37079904449628	2.86517093147903	-3.48798669526506
O	1.79366779795954	6.93768825725750	-4.31141442377886
Si	-3.81749127859552	5.01878154424971	-3.32439164574235
Si	2.19334211392121	-3.04660865332427	-4.63873566298284
Si	-4.03957464952164	2.33008696289677	-4.53354485019886
Si	4.00883034509351	-4.07798283682477	-2.50705500569133
Si	-4.34490241081558	0.65553075973969	-7.06206959898495
Si	-0.30085095727401	-1.28541837660028	-4.34889351346893
Si	-1.21499684299902	1.48298072715961	-3.50544385868481
Si	2.56100907999512	5.56432921190685	-4.72239042491570
Si	0.65749596333625	8.08672553702124	-4.45630872747098
O	-2.79433674912248	-5.58340501074504	-7.11588687698252
O	-4.54255557502958	-4.34492069443858	-5.54648121469771
O	-2.92957992350604	-2.91706957363765	-7.20546240027928
O	-2.81019167765832	0.11462456039281	0.17035124511508
O	-2.48599712515199	2.75730807534656	-0.14474317149812
O	-1.65345694587313	1.00933031290243	-2.01192278752674
O	-5.12591935320467	2.80168574341613	-0.04355495622872
Si	-3.77084622221329	-4.29210128590842	-6.97726635288885
Si	-1.84366478968452	1.29420243898598	-0.43553923911151
Si	-3.80656089821151	3.67950753153167	-0.39225817593274
O	-5.00687564152367	1.91634078779664	-3.29743015454847
O	2.81398462739768	-5.05157825630784	-1.98597869226274
O	-5.59828108295135	-2.50843639768999	2.82626375062293
O	-7.38299576752813	-1.78852136502741	4.66885307192336
O	-0.25713067217948	-6.30941918673613	-3.40535867525468
O	-8.00878267743797	0.18864363796483	3.00281120931963
O	-0.38942067684323	-5.57283138277324	-5.96416728534270
O	-0.97503281788934	-1.91275648343248	-3.02066471136188
O	-3.49066949466606	-6.51223537858986	0.73865738967895
O	-0.96800747964927	-4.26405727183629	-1.89006384882865
O	-6.60701636780488	1.04089340166910	-1.40569173655296
O	0.62730226798706	-6.08481404074324	-0.88122509375646
O	-8.35729004920758	-0.62741236230555	0.48182932376202
O	-7.35268410614355	1.77527215436240	1.01369202342148
O	-1.88037431434706	-6.73401107089119	-1.40002443587818
O	-6.51938520429733	-0.20750467832072	-3.72302255715940
O	2.71429004780390	-5.41562959116821	0.63344315663339
O	-9.07476608277811	-2.66658687551615	-1.03277778284459
O	-4.46140968006921	-0.37750741426936	-2.07750153348232
O	-7.01191689731807	-2.91067811344522	0.61206564484865
O	-8.03290684780941	-2.37179661538254	-3.46810555581863
O	-5.37989019396913	-2.61282661296633	-3.74360024269752

O	-7.26338367687536	-4.36781415207215	-1.88680677257482
O	-5.00341702144839	-5.15274477451635	-3.07238727760221
O	-2.89446329007108	-3.63600590439272	-3.64052671248595
O	-5.69647532847433	-6.15795467040912	-0.71927349002450
O	-2.80538316193768	-2.53755346359890	-1.21051139558631
O	-4.76621301574656	-4.21697967331206	0.91048967243245
O	-4.66423342398405	-1.54527379213405	0.67055826523629
Si	-6.47537161681104	-3.01414351986325	4.10651214734662
Si	0.59857162861488	-5.83409961647981	-4.70555045575822
Si	-8.49542477717428	-0.65205231453098	4.31244587861348
Si	-1.24389001484019	-6.07070475228012	-7.25826376740797
Si	-6.64250247455265	2.28732714103915	-0.36620956309708
Si	-2.43728241170099	-7.48584208532555	-0.05706291798495
Si	-0.61996208439132	-5.83789602327905	-1.89260387417983
Si	-8.42641260288787	0.64840596664687	1.49276293273656
Si	-5.62253389945497	0.57105101429059	-2.61503117572911
Si	2.24953907951142	-6.01968436583193	-0.81111778330015
Si	-8.48857168760737	-2.24196715916657	0.41896825527968
Si	-6.70146335471026	-1.75440721713926	-4.17563542330298
Si	-8.51920592691670	-3.44472252167477	-2.35377528620335
Si	-4.45048308905016	-3.92534301517085	-3.98602119030129
Si	-6.25940610122046	-5.61942016534439	-2.14727425193570
Si	-1.93605267241487	-3.08650597929858	-2.43242563505337
Si	-4.96145320635093	-5.83184470107648	0.69748654522619
Al	-3.68234842964461	-1.14617672934901	-0.77866984252570
Si	-5.52121766850160	-2.82252520135905	1.23324840007636
Si	-2.10954238310886	-1.54375305752235	-6.91351099986206
H	11.67354000357192	-5.53749701166224	-0.03813000169679
H	9.19325801091469	-8.10610401364427	2.36365699751417
H	-3.96156899927171	1.36419899394932	-8.28506099336883
H	-5.56398599878685	-0.12270100231273	-7.29597599999392
H	-6.93632899758291	-1.73777199867017	-5.60525601587680
H	-9.61070200819216	-4.23261600510759	-2.91602400512273
H	-4.80937900363218	-4.38478701289431	-7.98416800186205
H	-1.26201398552095	-7.53013903148288	-7.34713300556374
H	-8.46716099930106	2.53175394751700	6.77435900895615
H	-4.20618399516312	6.50892099603648	2.49272999683029
H	-1.81213399566234	9.00646798709333	0.20177800003425
H	0.23145508862326	10.95011941020538	-2.10809613532923
H	-4.94421499612842	5.93630899671864	-3.34769399985124
H	-7.43440299512664	3.36541399640514	-0.94274899829518
H	-9.81225000172062	1.14342200278143	1.44886400243321
H	-3.26051299167899	2.50761899012056	9.43266799320002
H	-0.74472599720947	4.85510199356706	6.90777200249316
H	1.17261700006851	6.84873799296664	4.60176299287679
H	0.01371298714413	-0.85239401645100	9.75320298971000
H	2.65644498477208	1.61373198766787	7.09816899903031
H	4.53016699945185	3.47572500165609	4.86954700197503
H	1.53638200169736	-6.93834199831418	-4.97336599793099
H	4.68077900333475	9.17388299221293	-0.65420600266146
H	-8.67443703388199	0.53296796954830	8.08822304764797
H	-5.67876700122391	-0.35798099966045	8.92693200247152
H	-5.40538809069084	-3.45794000652262	7.74582398910328
H	-2.86030616465352	-3.03941603244354	9.10082109947382
H	-1.22727265518571	-4.11134493653476	7.70409199246347
H	2.59107099816204	5.38865099406027	-6.17172400277132
H	0.22684599766960	8.11439299317007	-5.85590800223314
H	2.35648394775126	11.66568595542108	-3.24752296647456

H	6.05893600381341	7.26486299988169	-3.76938199789827
H	10.01162899771249	4.23690500246652	-2.76123799946368
H	9.87912999919405	0.42295200117362	-3.59486200021999
H	11.16666299999881	0.78647299911904	-1.60521600227744
H	9.89955900186159	3.90245199962533	-0.38701600053415
H	-1.31531299978128	-1.20647001128465	-8.10616499634946
H	-0.61432201693846	-5.50729800644306	-8.46106598348631
H	2.84834100220205	-3.04402000196930	-5.95043800162140
H	-7.31528200446210	-4.15742497286362	3.75037499461649
H	-9.78237303118842	-1.30797901111224	4.07942698781245
H	-9.40254700464326	-2.75213299788457	1.45267099824154
H	-5.76896401761336	-6.40939900885282	1.78048399121997
H	-3.08137201221846	-8.75069603024017	-0.46757201150381
H	-7.02203600892819	-6.69280401109874	-2.78388200541336
H	2.78306899424514	-7.36900599554375	-0.99232700235234
H	4.89569300195254	-9.57645900368668	2.49579400209191
H	5.81992199483520	-9.04892399679433	0.34487200394916
H	-1.34472301214449	-7.77598299394135	0.87488999665832
H	10.66941399506266	-2.02071801899546	2.56381700119193
H	8.82291800928011	-3.95596101250507	4.78390500988894
H	11.59057499740249	-3.97495100252710	-1.85889599349883
H	5.33835300186535	-0.46465101127068	4.51989000257146
H	7.15208500176937	1.34023100192847	2.37194499758994
H	7.32230800065180	6.57802299281754	-0.51391500217067
H	5.00675700050018	-4.86530400085305	-3.24361799792387
H	7.90182200061077	-7.00149500103840	-1.50647500124080
H	2.44258800023056	-6.50026702804663	2.91141099314968
Ga	-3.69434723839900	-0.26996856005468	1.95560533086665
C	0.88516661914793	-2.99714372447201	2.32732097240003
H	1.71654340766616	-2.33619459392611	2.09937672691221
C	0.82294952824664	-3.63093280432745	3.50814053495437
H	-4.66800879813530	0.88968948417363	2.24505099511742
H	1.61248634375380	-3.50230787320117	4.24552836429545
C	-0.14714683579685	-3.20374889674445	1.31856581449989
C	-1.31738660569465	-3.77333350740347	1.64473819744226
H	0.03561399713731	-2.86477855467414	0.30179606348018
H	-2.68203495549497	-1.09851816328433	2.76996559547566
H	-2.10440823336618	-3.87684080751940	0.90327232131305
C	-0.29936767948072	-4.59757537528076	3.78414019565680
C	-1.58840771742331	-4.18386461100833	3.06666488664508
H	-0.47920904901719	-4.69529987067633	4.86081440539323
H	0.01051380117378	-5.59704087487043	3.42946681838702
H	-2.32100695511980	-4.99863352424208	3.10129474114129
H	-2.05143752551573	-3.33280964660767	3.59726411706068

I18-GaH 2

Electronic energy (r^{2}SCAN-3c): -30772.067162103907 E_h
Electronic energy (ωB97X-D4rev/def2-mTZVPP): -30780.410625836412 E_h
G_{corr} (673.15 K, 1 atm): 0.46290 E_h
Number of atoms: 256

Cartesian coordinates (Å)

O	1.50565892854943	2.97029678927906	1.05334540690321
O	-1.94585385911923	6.76944096916242	1.42306234917420
O	5.26174048638552	2.07738020341865	0.80455211458256
O	3.61679850889399	3.88472224782924	-0.17493960066948
O	6.10181727238568	4.56933910203685	0.40946415172306
O	1.42516937163409	3.29808774124995	-1.60255490865735
O	1.40468153729768	5.37581489129394	-0.01551507272753
O	-1.69431838679725	6.89613018142062	-1.19496791331920
O	0.31700017501744	7.72457497685461	0.42052486867329
O	2.90791580661187	7.56387759330805	-0.06027926482993
Si	4.84933666884572	3.64193539401477	0.85982571227110
Si	1.99743645750282	3.88019039732686	-0.19789646559861
Si	-1.29716925295519	7.63363836421533	0.20467686289993
Si	1.65550915428860	6.83286192729375	0.64923367369742
O	8.94352906539921	-3.82865451094243	2.22482965670671
O	2.01036791280805	6.66080635315680	2.22909689670670
O	7.14353070539899	-2.27872793717105	-0.91780811638386
O	2.51895238088820	4.77094061165065	4.07803534744528
O	6.74819760566445	-4.90341494359955	-0.76595158193002
O	0.05809133145064	5.12520959130150	3.13273351085397
O	5.84350017413379	-3.26424445733277	1.13834143313995
O	4.44594404761285	4.08321675705935	2.37631083409483
O	9.40033610009179	-5.01122366391752	-1.12932179748727
O	5.16177515720927	-0.25118011833661	2.01280497037791
O	7.73233123671564	4.38398616892906	-1.70044402662380
O	10.70007784753953	-3.18636009837933	0.34645868776704
O	5.42008030799697	5.65225591813520	-1.90366495374570
O	4.11168639214651	5.69037196303313	-4.19114458331696
O	8.76371675955365	-1.45508271806794	1.04413447977048
O	3.89234369730859	7.74560770150015	-2.54380083050442
O	1.24351310322731	9.55201483082552	-4.03684617436124
O	8.97966249885837	2.07265345800249	-1.88578186988923
O	6.95089823902688	0.19580947077859	0.09840579978206
O	2.35872280338290	9.69386708987840	-1.58327307395071
O	8.99955124978190	-0.54818634794031	-1.44607029192321
Si	1.44133740950603	5.87508860372380	3.54120706615326
Si	6.11383584311436	-3.43948543898001	-0.44621211993081
Si	3.64143693935414	3.64427134103754	3.72290788015288
Si	8.07896437082566	-5.83648486275816	-0.65623118199510
Si	10.87564339814929	-4.45954340638126	-0.67224241652230
Si	6.67004907156124	5.33117072164614	-0.91596895397833
Si	9.79311290711813	-2.60668262454337	1.56630204086531
Si	4.91634759968230	6.61237220019576	-3.11659690375969
Si	9.20327067321558	3.67528162131869	-1.66890912772432
Si	6.17481524368420	0.85653575319691	1.36864815830060
Si	1.52818133138598	10.52117218499514	-2.73738501630840
Si	9.80036395997896	0.68446784378299	-2.15441076160676
Si	7.95687890579409	-1.01413408803572	-0.30368292925174
Si	3.47659799010627	8.57272140155616	-1.20478182284492
O	2.98631302695481	-0.44856798242410	3.60623559673782

O	0.97799314821148	0.80897945017038	2.47479087456660
O	1.24996597694455	0.88343987084563	5.08422229495730
O	2.98081549240790	2.18019528703715	3.49110441353856
O	-0.32758742620307	1.24290135570171	0.25295721523939
O	-0.82585845262394	2.76066178878070	2.33581881954805
O	-3.71279790258070	4.94208319717112	0.58111554490591
O	-2.52838583413445	4.68188986026440	2.96103413782929
O	-1.14373336322685	3.42907202971164	4.83752936754228
Si	2.05551878550093	0.86744557210063	3.68327239738297
Si	0.33537009114414	1.96128876792168	1.54124773823129
Si	-3.12896441175356	5.74025116247823	1.87204911424170
Si	-1.10090863763156	4.01014349645675	3.33359063663026
O	8.27963126765855	-6.03827868301439	3.49427461837320
O	-3.20333443654741	-3.08825423900836	6.58478305309726
O	6.68386552220890	-7.85020588498025	2.44448574788490
O	-1.31056602918326	-1.57346852786080	7.68615736180733
O	8.25987095747766	-6.34350295794754	0.87167901813109
O	-5.43245711159797	-3.47763641391588	5.23178498497490
O	6.64619517379289	-3.91291094105681	3.57457559294512
O	-5.11960556822536	-1.22190764860983	6.59446703876796
O	-8.68539161009931	0.34765221693905	5.57732429306445
O	4.46109922792468	-2.50694267773527	3.24886572995944
O	-6.45038534408354	1.04179271010601	6.94878002951828
O	4.48619850387566	-5.06187548976211	2.61392279432240
O	-3.89244982933041	0.86536645928108	7.58023520509610
O	-2.25120212750399	2.84888241276703	7.14178754197829
O	0.40309734871497	2.65736417915827	6.87229931645485
O	4.25907253245522	-7.39629394584952	1.37975362936465
O	-1.51031930613702	0.86461244549908	8.77885335826176
O	0.71878025920690	0.07640441926565	7.52749099780350
Si	8.12635591401713	-7.12345073467981	2.28724699804173
Si	-2.13512458032141	-2.97799032365404	7.81576522424576
Si	8.19380284295497	-4.41199027521138	3.55255202279263
Si	-4.81263397859064	-2.82015480739909	6.57375968841891
Si	4.53403076833719	-0.88966626116256	3.37330511978254
Si	-8.08669519284054	1.12638198608339	6.87714717345076
Si	5.35260583199053	-3.70144477956095	2.61714132125110
Si	-5.30295182408420	0.06616204181865	7.56536322502072
Si	-0.93191367636466	3.48713652108500	6.44594798259695
Si	3.44260093143830	-6.09789990979564	1.92724989179415
Si	-2.75333511602162	1.78689937624521	8.26742684511062
Si	1.29606224850346	1.31618422461782	6.65383522520413
Si	5.41709903002510	-8.50957474552043	1.65014002070141
Si	-0.52102429285430	-0.39089263071542	8.47288545291275
O	1.84331644657359	4.28791279158191	-4.03651734157870
O	-0.07387377938818	5.27630700324014	-2.49260728465811
O	-0.56345646415071	7.72483930764486	-3.43851323319684
Si	0.71517415036712	3.93168295536753	-2.92907729896667
Si	-1.18553316855034	6.43943611576068	-2.66561767263010
O	1.24917606880501	-1.73513775607359	-4.50105418010004
O	-3.93612612512940	3.97991190870464	-4.56843706093301
O	3.34155089097807	-2.95962532572816	-3.48240195035952
O	-2.44187271415858	5.88590186969384	-3.52370782284081
O	-3.76096166823878	4.19035461580161	-1.92852167843047
O	1.33513606187157	-4.41553866653992	-4.41297307750687
O	-4.64459858129450	1.80842134797690	-5.94407492677091
O	4.72047716931205	-3.30518723437418	-1.25837269051125
O	-2.55253299893764	1.72561401470993	-4.38829254659127

O	-3.14783275812352	-0.34272916121687	-6.54499578224443
O	-1.10676950239648	-1.81950814154025	-5.67989963130295
O	-0.32037791299144	0.33546318919805	-4.29661929018152
O	-0.32724671986485	2.86942285325402	-3.55787742122478
O	1.80045587084399	6.94178916164963	-4.31529377767171
Si	-3.81195305050304	5.02841065879678	-3.32618683556858
Si	2.19319352519190	-3.04945742488053	-4.64008882896823
Si	-4.03903363412234	2.35627571368191	-4.55103707068688
Si	4.00913572799223	-4.07702797497682	-2.50733045862974
Si	-4.34277492551030	0.65598843734040	-7.06363366622141
Si	-0.30306354644919	-1.29077830439783	-4.37664586192367
Si	-1.18375553311237	1.49591469116233	-3.57455651413314
Si	2.57639252643909	5.57361188120141	-4.72483704472700
Si	0.66269715994641	8.08995243276352	-4.45735266356532
O	-2.79306729919512	-5.57943933565831	-7.11088633259327
O	-4.55064506885304	-4.33966802101311	-5.55091465907064
O	-2.94364887812563	-2.91293605725145	-7.22186734967569
O	-2.61149157409471	0.01576418355589	0.12094647643752
O	-2.50032796018709	2.69890121501987	-0.18763848519991
O	-1.54817101464465	1.02047364596209	-2.06239463586122
O	-5.12958356692128	2.80515715863254	-0.03965968712148
Si	-3.77512116930133	-4.29124893879632	-6.97834660853515
Si	-1.74596554041287	1.31061714256752	-0.50545764780432
Si	-3.80471687787463	3.66081732172528	-0.40224619282043
O	-5.02659008002892	1.96594055381932	-3.32711603797285
O	2.81700074645602	-5.05142288772332	-1.98204402261088
O	-5.67562039597437	-2.48214349550840	2.79214289553480
O	-7.39649007502371	-1.79146391326336	4.69504679303225
O	-0.24578474764215	-6.31940121501058	-3.40020584754362
O	-8.01666728235393	0.19750254288962	3.01427893786956
O	-0.39038883472683	-5.57417836672102	-5.96125716585262
O	-0.97627422485133	-1.89394070137308	-3.03634015418977
O	-3.48687470717333	-6.50457985739328	0.74377848293057
O	-0.96899672174570	-4.25350464489636	-1.92241992354041
O	-6.59401063319285	1.04935769656512	-1.42170562375687
O	0.62072965473628	-6.05531419348151	-0.87093684789685
O	-8.32658600670526	-0.65493608344936	0.49685926323311
O	-7.35361790721301	1.76194630237456	1.00884820510361
O	-1.87908779115539	-6.72161011161640	-1.39263964946785
O	-6.44510790572523	-0.21639620726041	-3.71099343376783
O	2.71318080406635	-5.42292180243333	0.63902225325735
O	-9.08747134065765	-2.65843842109886	-1.04418418421722
O	-4.33465201468051	-0.22477307259084	-2.04894041173958
O	-7.07057808449086	-3.02975137358681	0.62013000986737
O	-8.02811332541528	-2.34942848623588	-3.46618607137736
O	-5.36990588732397	-2.61499532861798	-3.72981028989340
O	-7.25985562194654	-4.35945923763734	-1.91731846114182
O	-4.99581696913807	-5.16234710471633	-3.07851662211740
O	-2.89117321611658	-3.63138944722727	-3.66071192784559
O	-5.68734018469748	-6.13438548071681	-0.71577753396207
O	-2.80692439605301	-2.52271388276722	-1.22896832074850
O	-4.75763451324327	-4.21600201433349	0.96446817143400
O	-4.80957202258460	-1.58550939791430	0.52555850908641
Si	-6.48855070335998	-3.00359997076825	4.08943161541690
Si	0.59924251193894	-5.83652572346610	-4.70541535977996
Si	-8.49769640034599	-0.65443865264735	4.31866873119542
Si	-1.24478067733365	-6.07005479041038	-7.25732273152963
Si	-6.64743723742516	2.28060741917064	-0.36597698536469

Si	-2.44372176531279	-7.48163106790271	-0.05390833260160
Si	-0.62355796216118	-5.82861293997817	-1.89830611104412
Si	-8.42418337279135	0.62916397455622	1.49485949034250
Si	-5.57638246403940	0.60572822571025	-2.61186750382682
Si	2.24601945821966	-6.01738971131973	-0.80983155677560
Si	-8.49127033935572	-2.27049798531668	0.41875725317127
Si	-6.68703205217911	-1.75155686744727	-4.17048920343418
Si	-8.52175496104365	-3.43352731116166	-2.35979795127125
Si	-4.45042137534565	-3.93107938889380	-3.98744868547785
Si	-6.25731738077172	-5.61555568621280	-2.14707502025565
Si	-1.94407337287777	-3.06914414974880	-2.45185574072306
Si	-4.96190326225099	-5.81302352620382	0.71178390349341
Al	-3.75005961696447	-1.17434690046118	-0.75267208003438
Si	-5.56548616346027	-2.81357251576276	1.20338510557098
Si	-2.11544529117052	-1.54841005603896	-6.92093081618891
H	11.67354006142478	-5.53749702770613	-0.03812997374345
H	9.19325800485087	-8.10610401943575	2.36365700905761
H	-3.96156925318146	1.36419899890007	-8.28506147344373
H	-5.56398595103368	-0.12270098708021	-7.29597598378760
H	-6.93632899439825	-1.73777199555350	-5.60525598719536
H	-9.61070199905843	-4.23261600271244	-2.91602399487365
H	-4.80937900394122	-4.38478700709412	-7.98416800695591
H	-1.26201399586596	-7.53013900731688	-7.34713300182770
H	-8.46716099634525	2.53175399308430	6.77435904603286
H	-4.20618400112883	6.50892100196160	2.49273000998588
H	-1.81213400098090	9.00646799729914	0.20177800000948
H	0.23145501112030	10.95011905292963	-2.10809601983097
H	-4.94421500118577	5.93630900187114	-3.34769399302444
H	-7.43440299672399	3.36541399447457	-0.94274898558488
H	-9.81225001501629	1.14342200863163	1.44886400819933
H	-3.26051300231245	2.50761900104105	9.43266799986301
H	-0.74472599784555	4.85510199813803	6.90777200259048
H	1.17261700063077	6.84873799676656	4.60176300694305
H	0.01371299591198	-0.85239400195268	9.75320299545976
H	2.65644500988616	1.61373199842721	7.09816899854404
H	4.53016699830427	3.47572500171555	4.86954700623754
H	1.53638200221171	-6.93834199911426	-4.97336599920952
H	4.68077905096195	9.17388303502362	-0.65420595478797
H	-8.67443703758061	0.53296797178675	8.08822310236022
H	-5.67876699588074	-0.35798100357531	8.92693201815796
H	-5.40538799693862	-3.45793999734772	7.74582399511576
H	-2.86030599654532	-3.03941600226472	9.10082098787568
H	-1.22727299495041	-4.11134500983176	7.70409202577313
H	2.59107100837536	5.38865101058681	-6.17172396513851
H	0.22684599738572	8.11439299922749	-5.85590799566792
H	2.35648399710489	11.66568599112521	-3.24752299551679
H	6.05893603012746	7.26486301061685	-3.76938198685832
H	10.01162899712638	4.23690500199661	-2.76123799927728
H	9.87912999856223	0.42295200070718	-3.59486199519345
H	11.16666300493620	0.78647299880249	-1.60521600160461
H	9.89955900150840	3.90245199949111	-0.38701600030220
H	-1.31531301472792	-1.20647000554286	-8.10616497023077
H	-0.61432200146518	-5.50729800040975	-8.46106599956793
H	2.84834101361635	-3.04402000207512	-5.95043800806624
H	-7.31528199693925	-4.15742499559608	3.75037499693124
H	-9.78237298851770	-1.30797898872263	4.07942700965641
H	-9.40254698754513	-2.75213299797392	1.45267098859954
H	-5.76896400480911	-6.40939900395843	1.78048400691615

H	-3.08137202492058	-8.75069601047363	-0.46757201775155
H	-7.02203599809937	-6.69280400211788	-2.78388199962465
H	2.78306900318411	-7.36900600520001	-0.99232700318598
H	4.89569299466595	-9.57645899869611	2.49579400848474
H	5.81992200235938	-9.04892400017023	0.3448719956242б
H	-1.34472298292619	-7.77598297940598	0.87489001796716
H	10.66941400438597	-2.02071799765171	2.56381701191263
H	8.82291800695900	-3.95596100215940	4.78390500999026
H	11.59057498095196	-3.97495101743827	-1.85889596701131
H	5.33835300312912	-0.46465100556682	4.51989000362013
H	7.15208499194687	1.34023099708836	2.37194499463396
H	7.32230800588342	6.57802299298776	-0.51391500153172
H	5.00675700543693	-4.86530400659612	-3.24361799861539
H	7.90182202153122	-7.00149497990543	-1.50647497936274
H	2.44258802361953	-6.50026701032810	2.91141101477337
Ga	-2.02498397262254	-2.35880950181720	2.67116022647316
C	0.76394668447857	-2.82975291366304	3.15346386649487
H	1.30370510182517	-2.23482749988428	3.88522104685873
C	-0.13441051470331	-3.77308934702041	3.57288814951928
H	-1.76610658204796	-0.82124308884080	2.24335053719091
H	-0.26578353246878	-3.97172814041051	4.63438604803028
C	0.98374851707793	-2.58670156551175	1.75216617864636
C	0.00695705958336	-2.98988946827020	0.89534480013811
H	1.80608708625270	-1.95855221685521	1.42926599991390
H	-3.12160847153583	-2.59534055734981	3.74546757506144
H	0.01795424028818	-2.66430489621268	-0.14156207166466
C	-0.73327636580033	-4.69087904952742	2.52539736230358
C	-1.14475647052222	-3.73258403052896	1.41158536997777
H	-1.59758223568623	-5.23563775976257	2.91809994284176
H	0.01258879860956	-5.43420236435547	2.18603569932631
H	-1.82056916977782	-4.12745242374791	0.65408182310529
H	-2.27892121209191	-0.32542344254710	1.01799715058281

I19-GaH 2

Electronic energy (r^2SCAN-3c): -30772.105860605177 E_h
Electronic energy (ωB97X-D4rev/def2-mTZVPP): -30780.455360547894 E_h
G_{corr} (673.15 K, 1 atm): 0.45148 E_h
Number of atoms: 256

Cartesian coordinates (Å)

O	1.49499759369138	2.99153335209868	1.04776967952725
O	-1.95396523039313	6.75981020478169	1.41975312305240
O	5.26374452551463	2.08206913245358	0.80703183253876
O	3.62254546890957	3.89624658239918	-0.16619678379648
O	6.11033109047175	4.56994302402342	0.41107475282698
O	1.45194602354121	3.30647282684823	-1.61068563886912
O	1.41018161780455	5.38860616972747	-0.02919596740294
O	-1.69385698294557	6.89803071088185	-1.19325488710847
O	0.31249886451273	7.72601073315872	0.43310089655116
O	2.90584544269734	7.57747871555828	-0.05152279997427
Si	4.85520712740979	3.64731494455144	0.86498789434161
Si	2.00354723000451	3.89152510297818	-0.20129654190238
Si	-1.30046002996440	7.63112349613783	0.20906978569988
Si	1.65521083871793	6.83755910723822	0.65186785632843
O	8.94299900912151	-3.82834992554218	2.22522086172305
O	2.01337049112210	6.65531435164667	2.22992430524733

O	7.14725847052890	-2.27918742247170	-0.91990357153627
O	2.51787429265884	4.76395612781066	4.07823767918912
O	6.74935956122709	-4.90355904520845	-0.76593051013728
O	0.05791145240838	5.12663246774973	3.13877194589081
O	5.85089443116785	-3.26177266170219	1.13877740388254
O	4.45749681787306	4.09372136382552	2.38124328401709
O	9.40131968285186	-5.01214726044404	-1.13098045098715
O	5.15429546399567	-0.24590297704328	2.01702911392362
O	7.73371980663287	4.38623784897447	-1.70385234442067
O	10.69925919367809	-3.18674770015367	0.34680110124707
O	5.42147505790320	5.65294073803828	-1.90238363680172
O	4.09412706374436	5.67508325822538	-4.17363551197071
O	8.76383834028271	-1.45411259469255	1.04466201939419
O	3.89011074780102	7.74395605152024	-2.54153921847255
O	1.23909929721838	9.54669940230350	-4.03318992675810
O	8.97972986140502	2.07370124415915	-1.88690411436389
O	6.94567085605446	0.19318951726933	0.10171552128265
O	2.36065170731491	9.69736962661693	-1.58387724063938
O	8.99823758339866	-0.54521687385684	-1.44392913488035
Si	1.44376435416418	5.87216189611980	3.54302902355431
Si	6.11736713689179	-3.43831571821471	-0.44575584953622
Si	3.64759797295109	3.64373134288441	3.72096600019682
Si	8.07989433656747	-5.83648251663735	-0.65656380881714
Si	10.87556286280065	-4.45932857318022	-0.67211698067511
Si	6.67195431543378	5.33160898243344	-0.91615675129243
Si	9.79222658445724	-2.60612774354053	1.56657383187945
Si	4.91130110151673	6.60832549565512	-3.11621515056841
Si	9.20329353553932	3.67605963553143	-1.66921395094400
Si	6.17039547745304	0.85716390251721	1.37228184992373
Si	1.52743743519261	10.52030189540057	-2.73799916222416
Si	9.79990348383790	0.68527073442902	-2.15412009050350
Si	7.95513671746346	-1.01239838962422	-0.30207070222783
Si	3.47645903156469	8.57469461266320	-1.20386549315383
O	2.97953214036280	-0.44870436861201	3.61847467665237
O	0.98240750930861	0.84131435892954	2.47542720965861
O	1.25132549314644	0.92222931906808	5.08006961661850
O	3.00685567747079	2.17397000428300	3.48088830373348
O	-0.33175043559819	1.23276576793015	0.26266013008692
O	-0.83648278377269	2.77133495066311	2.32845505655502
O	-3.70494948753019	4.89741896112608	0.60727870617922
O	-2.52762131405661	4.69514080626890	2.99418177175581
O	-1.13004803907951	3.41629626724502	4.84065077895163
Si	2.05918815703733	0.87553728327778	3.67809418638574
Si	0.32937418874452	1.97418938506364	1.53093108806507
Si	-3.13069412593630	5.72791111346065	1.88168769770161
Si	-1.09928857716496	4.01016969796255	3.33922221603415
O	8.28110117678409	-6.03870240413173	3.49405294188083
O	-3.20461101305309	-3.07451301288880	6.58838587425838
O	6.68172173961145	-7.84693037856282	2.44223035791117
O	-1.31376202448079	-1.56768815293065	7.68690159628500
O	8.26287105713864	-6.34407628482650	0.87088909954862
O	-5.41625491567001	-3.47338881142357	5.23223017884660
O	6.64525835756982	-3.91609800744993	3.57573537330263
O	-5.12976188037002	-1.21806205692089	6.59099097393522
O	-8.67400186885438	0.35312995467299	5.56975484126848
O	4.46300153030236	-2.50467808486379	3.24625054718772
O	-6.44789239573007	1.04669258887078	6.95175572160792
O	4.48675028180823	-5.05883199621965	2.60463839938499

O	-3.88622233597403	0.85995195930912	7.58082867901556
O	-2.25520879691157	2.85049433958309	7.13920532378419
O	0.39890162404264	2.65850035997058	6.89741024595110
O	4.25612637958279	-7.39433573976161	1.37423071569451
O	-1.50630748988165	0.86750138723894	8.77920733950517
O	0.71756299025215	0.07476755978184	7.51601686688654
Si	8.12623774624802	-7.12296205516507	2.28667615838962
Si	-2.12878608702865	-2.97536713476484	7.81881556924976
Si	8.19330236782402	-4.41217614577693	3.55281210814870
Si	-4.81014094896822	-2.81313703675572	6.58471143352832
Si	4.52589784518648	-0.88684358645682	3.37743071042421
Si	-8.08341372378717	1.12913553647586	6.87841652457872
Si	5.35404350792024	-3.69809031367854	2.61627932405814
Si	-5.30006858944144	0.07156126137204	7.56812162406287
Si	-0.92909460453633	3.48654655281497	6.44867386764926
Si	3.43991871594662	-6.09623903292438	1.92612547063728
Si	-2.74846388042273	1.78941019195709	8.26737761727603
Si	1.29728003235077	1.32457825195474	6.65387772676745
Si	5.41467556144548	-8.50731854807997	1.64883865307776
Si	-0.51532425076159	-0.38709109206275	8.47052641078743
O	1.80382277845360	4.27481474845197	-4.06126721676740
O	-0.07638499422014	5.27158906773617	-2.48726911920447
O	-0.57238982654189	7.71674286700696	-3.44353705196018
Si	0.70240831067783	3.91979233085022	-2.92237329251569
Si	-1.18833583998909	6.43282542872521	-2.66198198375785
O	1.24087251506135	-1.74214489501491	-4.48214356675754
O	-3.94366321360519	3.96204216292290	-4.54239211192059
O	3.34737704249265	-2.95830368159234	-3.48414717552133
O	-2.44792529748756	5.87351932879584	-3.51143933321743
O	-3.75465746711488	4.17546860877785	-1.90498065371800
O	1.34252570029757	-4.41743870511233	-4.41113726169180
O	-4.64115356059099	1.79898096266191	-5.92964586103229
O	4.72308184421509	-3.30524042923914	-1.25691260800701
O	-2.57509611155888	1.69501772521920	-4.33434781976257
O	-3.14600379554011	-0.34291692462209	-6.54538633970660
O	-1.09965759357664	-1.81465382625565	-5.68149810759004
O	-0.33575024828070	0.32196166838448	-4.25672297712727
O	-0.35774957977852	2.84454446244191	-3.49721032835703
O	1.79423377529421	6.93537078803826	-4.31202038959202
Si	-3.81582293602424	5.01537369611823	-3.30331811232715
Si	2.19459623568141	-3.04706117413766	-4.63702692070560
Si	-4.04946983907889	2.33940358243847	-4.52599648816630
Si	4.01268257183153	-4.07463407798519	-2.50700000234635
Si	-4.34150539498781	0.65642977458718	-7.05995468897813
Si	-0.31299485827778	-1.30040021877103	-4.36122822031855
Si	-1.20765473925796	1.46459070211502	-3.51061516483334
Si	2.56191378766014	5.56215998676058	-4.72255085702253
Si	0.65804323153039	8.08462324894638	-4.45577641040419
O	-2.79202082510383	-5.58121496744662	-7.10566604349422
O	-4.58379193098368	-4.36114112162818	-5.56624958019298
O	-2.94791335818677	-2.91474050662073	-7.20397362019511
O	-2.61124670577772	-0.07977303814571	0.16227670446195
O	-2.52665981984242	2.62469174729942	-0.15804676577205
O	-1.58223362442216	0.95364534128042	-2.01720971270785
O	-5.13984880307137	2.77985779742300	-0.04327671106665
Si	-3.77858930582611	-4.29629019332812	-6.97836656252753
Si	-1.78519792059641	1.20703623991256	-0.43938706364618
Si	-3.79412529153499	3.61926980750156	-0.38370129249883

O	-5.06217460674821	1.95452676395100	-3.31650429505841
O	2.82002327762474	-5.04976188776202	-1.98192425342070
O	-5.64598871737587	-2.49103140148979	2.81354859213290
O	-7.39623148135266	-1.79436267959920	4.69280299076117
O	-0.25884985399244	-6.30086490788987	-3.40795550278511
O	-8.00597879262632	0.17185489840280	2.99876929344518
O	-0.38092754629588	-5.57011894872489	-5.96942332971511
O	-1.00731800344515	-1.92638785907582	-3.04005209560528
O	-3.48474797639523	-6.49900389192935	0.73189841510120
O	-1.00086731798660	-4.26429793099049	-1.87609126118973
O	-6.65630227660008	1.04970874924071	-1.42242188824651
O	0.62427552574039	-6.06158494395059	-0.88221254391477
O	-8.38561855938418	-0.62060126732372	0.47002332126119
O	-7.35208196767573	1.76666146209677	1.02158109144865
O	-1.87384288143262	-6.74765705637429	-1.40360568158481
O	-6.49035642271624	-0.22104584928429	-3.71738413624313
O	2.70457379103442	-5.41742384526836	0.63888760382797
O	-9.08777009723194	-2.66836964874383	-1.04075239245688
O	-4.43578418803519	-0.26254428245437	-2.02672304940169
O	-7.00477339529198	-2.87420298010527	0.56832827879584
O	-8.00428054813429	-2.38464368189986	-3.46455745754980
O	-5.34070072007194	-2.60820306648210	-3.75081844794939
O	-7.26817546490672	-4.37732174321173	-1.87608066093325
O	-5.00677631606337	-5.15425228925918	-3.07613418610857
O	-2.86511009272674	-3.70709807335940	-3.72533129659018
O	-5.69022382741871	-6.16085328281498	-0.72074863423272
O	-2.95395871676965	-2.59572020738668	-1.34316406009515
O	-4.79016621330407	-4.21977152413786	0.93105432143006
O	-4.61001770398323	-1.56671585112331	0.68504054439904
Si	-6.48816278384900	-3.00834768356201	4.10682626658328
Si	0.60116057332063	-5.83281281893989	-4.70836488817601
Si	-8.49770253138803	-0.65335845489066	4.31625029802874
Si	-1.24337217123769	-6.06974202284864	-7.25779530998463
Si	-6.65856175686027	2.28209348895479	-0.36543684847954
Si	-2.43872142601711	-7.48633562128263	-0.05596207584766
Si	-0.62813474859701	-5.83413386397944	-1.89725261038674
Si	-8.43202892646074	0.64325423529025	1.49463629486852
Si	-5.63717445450364	0.60351541110238	-2.61061129433876
Si	2.24887422753400	-6.01807238617104	-0.81282212592312
Si	-8.49790693108516	-2.23547583076756	0.40728163051196
Si	-6.67630646811882	-1.76370138445677	-4.17646917228897
Si	-8.51589473827740	-3.44930629499620	-2.35377787666671
Si	-4.44548158971836	-3.94565341197107	-4.00805001573213
Si	-6.25858606715301	-5.62176210674509	-2.14586230596436
Si	-1.97088019864175	-3.11533599407236	-2.48525730443306
Si	-4.96295047306696	-5.83135700792077	0.69940250609597
Al	-3.69034000070933	-1.16175036784306	-0.79672378144240
Si	-5.52859887259508	-2.80707086482331	1.22222209753245
Si	-2.11357275494462	-1.54921379980228	-6.91796865849774
H	11.67354001912897	-5.53749701222656	-0.03813000606196
H	9.19325800607732	-8.10610399847682	2.36365699777415
H	-3.96156900008478	1.36419899839719	-8.28506099327802
H	-5.56398599771070	-0.12270100118464	-7.29597600009383
H	-6.93632900255024	-1.73777199837254	-5.60525603061742
H	-9.61070198696528	-4.23261599470579	-2.91602399973139
H	-4.80937898150238	-4.38478700744913	-7.98416800643908
H	-1.26201399053344	-7.53013901026508	-7.34713302123791
H	-8.46716123712598	2.53175410641498	6.77435920538652

H	-4.20618397908397	6.50892098128571	2.49272999119465
H	-1.81213400107566	9.00646800278290	0.20177800028877
H	0.23145501959239	10.95011898234081	-2.10809601906636
H	-4.94421499617757	5.93630900151781	-3.34769399985123
H	-7.43440300568219	3.36541401165577	-0.94274899334839
H	-9.81224999668557	1.14342200216623	1.44886400218500
H	-3.26051300191178	2.50761899607410	9.43266799392727
H	-0.74472599752406	4.85510200362113	6.90777200738017
H	1.17261700017499	6.84873799328110	4.60176299314847
H	0.01371299751075	-0.85239400059929	9.75320300013560
H	2.65644499488277	1.61373199808060	7.09816899886683
H	4.53016699956298	3.47572500686596	4.86954700715912
H	1.53638200666714	-6.93834199859916	-4.97336599808476
H	4.68077899266135	9.17388299243649	-0.65420600277268
H	-8.67443698653017	0.53296801179954	8.08822297824549
H	-5.67876699573586	-0.35798100464388	8.92693200191182
H	-5.40538800155866	-3.45794000670799	7.74582400480374
H	-2.86030600516739	-3.03941599656985	9.10082099789557
H	-1.22727298356750	-4.11134500941831	7.70409199999753
H	2.59107099856607	5.38865099444428	-6.17172399241594
H	0.22684600280244	8.11439299302561	-5.85590801306655
H	2.35648401109979	11.66568600779327	-3.24752300303953
H	6.05893600360682	7.26486299965966	-3.76938199811804
H	10.01162899759325	4.23690500234034	-2.76123799949580
H	9.87912999943977	0.42295200123964	-3.59486200048759
H	11.16666299991890	0.78647299904965	-1.60521600231326
H	9.89955900184536	3.90245199962535	-0.38701600039794
H	-1.31531298954334	-1.20646999520852	-8.10616501264572
H	-0.61432198012232	-5.50729800110643	-8.46106602561849
H	2.84834100176971	-3.04402000190842	-5.95043800111105
H	-7.31528198325285	-4.15742499419745	3.75037499446304
H	-9.78237299418659	-1.30797900554937	4.07942699833601
H	-9.40254701486762	-2.75213300310620	1.45267101946575
H	-5.76896400153581	-6.40939899832395	1.78048400171621
H	-3.08137199678749	-8.75069600428976	-0.46757199628669
H	-7.02203600391555	-6.69280401152016	-2.78388200591467
H	2.78306899862978	-7.36900599462092	-0.99232700232649
H	4.89569300748905	-9.57645899342834	2.49579400157060
H	5.81992199955044	-9.04892400181935	0.34487199451771
H	-1.34472300698152	-7.77598299895566	0.87488899668103
H	10.66941400018719	-2.02071799826802	2.56381699588186
H	8.82291799855143	-3.95596100219447	4.78390499370310
H	11.59057502943416	-3.97495100188813	-1.85889601050400
H	5.33835299701284	-0.46465100120381	4.51988899727141
H	7.15208500214610	1.34023100219011	2.37194499783853
H	7.32230800070209	6.57802299831847	-0.51391500205043
H	5.00675701077867	-4.86530400645365	-3.24361800875858
H	7.90182201131434	-7.00149501112829	-1.50647501165766
H	2.44258800161269	-6.50026700126762	2.91141099758863
Ga	-3.14355344671532	-0.78256462470613	2.00303279169026
C	0.56451563624755	-2.45016682316105	2.38383838376849
H	1.61456973740231	-2.26169936444939	2.17804411379563
C	0.02091846329144	-2.05572796745133	3.54442061679268
H	-5.14218918791077	3.21912650013518	3.57762539462841
H	0.62405201694947	-1.58073541809355	4.31293508821462
C	-0.23242925431579	-3.15663025627653	1.39349529578341
C	-1.57213296635265	-3.19675282201425	1.49604061953605
H	0.26628501062316	-3.63435902404544	0.55596056891332

H	-3.72308361854089	0.46704334864896	2.72054536871100
H	-2.16870049604638	-3.68911025518364	0.73537214185605
C	-1.40919949293540	-2.38191169522174	3.86869181547254
C	-2.28009659950566	-2.49898757214813	2.60650404820898
H	-1.82236473638734	-1.65878976470759	4.58150969676987
H	-1.42822094656532	-3.35419828528774	4.39443398711007
H	-3.18501037833807	-3.06199898779080	2.87257815593650
H	-5.60753515860849	2.86740538336878	3.11568261372968

I20-GaH 2

Electronic energy (r^2SCAN-3c): -30770.933850742549 E_h
Electronic energy (ωB97X-D4rev/def2-mTZVPP): -30779.271911496260 E_h
G_{corr} (673.15 K, 1 atm): 0.45330 E_h
Number of atoms: 254

Cartesian coordinates (Å)

O	1.49506023410015	2.99124339304912	1.04762031583911
O	-1.95243693683177	6.76118028884001	1.42078103260850
O	5.26379534103659	2.08222047785128	0.80683632511556
O	3.62243237923326	3.89653975287160	-0.16607665234186
O	6.11021012556909	4.57014731556689	0.41114127862705
O	1.45181444260900	3.30633902547040	-1.61064011776796
O	1.40976592639362	5.38847145864928	-0.02901163326173
O	-1.69347141927045	6.89762226999623	-1.19284691465718
O	0.31333111379609	7.72671135882532	0.43229944787042
O	2.90642407657746	7.57680918120381	-0.05180411050900
Si	4.85514675165307	3.64744112547818	0.86506176572678
Si	2.00344066648207	3.89149206693296	-0.20130215510778
Si	-1.29960571099766	7.63160138236775	0.20891253651490
Si	1.65549893197263	6.83750150083104	0.65169448549976
O	8.94297304516879	-3.82831765124275	2.22515413654082
O	2.01306311727567	6.65536433448496	2.22987518736167
O	7.14753182572298	-2.27942950576195	-0.91984930050036
O	2.51676624849717	4.76323438245959	4.07755386287470
O	6.74923610514867	-4.90374742246452	-0.76571581244925
O	0.05642145156945	5.12783364661821	3.13840585478226
O	5.85074557320216	-3.26162947418207	1.13873274364850
O	4.45749881521474	4.09372745343690	2.38137676727887
O	9.40126381097978	-5.01202460504209	-1.13100957239572
O	5.15383576272257	-0.24551965465180	2.01719815127339
O	7.73368817333871	4.38624563399928	-1.70377948016108
O	10.69932671944407	-3.18673181763051	0.34679262719572
O	5.42141198839891	5.65290624942320	-1.90241446128745
O	4.09440484032857	5.67525012760872	-4.17391610357910
O	8.76395977601369	-1.45400372975130	1.04466343628612
O	3.89025762299525	7.74409246920655	-2.54174567553770
O	1.23923794595607	9.54678067759221	-4.03324629366120
O	8.97972373062666	2.07373536684842	-1.88690039181365
O	6.94537806893728	0.19289138567220	0.10188565841591
O	2.36056588974128	9.69716181894896	-1.58379902598282
O	8.99807408924202	-0.54510259908873	-1.44393687045073
Si	1.44303177506589	5.87210651585501	3.54281707712503
Si	6.11741458779376	-3.43837180334067	-0.44575583500833
Si	3.64734822718933	3.64358058990779	3.72093544936436
Si	8.07993318493918	-5.83649465234374	-0.65654512791644
Si	10.87556142054530	-4.45932699557458	-0.67213248541277

Si	6.67190222793672	5.33164375193058	-0.91614254628053
Si	9.79226024516026	-2.60610987200026	1.56654854636229
Si	4.91140316628537	6.60835415041083	-3.11626576637810
Si	9.20327747137489	3.67608785348532	-1.66920457180581
Si	6.17022803524607	0.85722944766027	1.37234265669262
Si	1.52748699390172	10.52027945039820	-2.73794434247450
Si	9.79988109062948	0.68529643356997	-2.15411658995758
Si	7.95510870871044	-1.01243030963779	-0.30202139553899
Si	3.47656562701971	8.57459166448376	-1.20394786454580
O	2.97945013653197	-0.44890124085810	3.61925276703597
O	0.98276745085645	0.84095476778233	2.47520444269843
O	1.25109840366275	0.92281160448946	5.07999221537545
O	3.00704114970809	2.17369581101627	3.48054637819884
O	-0.33251465784074	1.23264368617926	0.26322615167481
O	-0.83605830505604	2.77119350829297	2.32878371290140
O	-3.70318066710692	4.90024607165261	0.60523326196501
O	-2.52909534196029	4.69303593557967	2.99186299200426
O	-1.12926658427313	3.41693168426065	4.84053470823757
Si	2.05922085671585	0.87543061755172	3.67819927194865
Si	0.32954414017054	1.97397025023149	1.53107819710742
Si	-3.12938172600834	5.72924348639102	1.88108091217055
Si	-1.09991399594740	4.01033952010886	3.33900277659104
O	8.28089105473365	-6.03859950978559	3.49399333236067
O	-3.20459689529844	-3.07494344334528	6.58845267564521
O	6.68173607878302	-7.84689029354257	2.44210816194878
O	-1.31379740405428	-1.56773260819784	7.68683712129589
O	8.26322666205525	-6.34413858158009	0.87084705007255
O	-5.41582961841301	-3.47309214704927	5.23167030381367
O	6.64528392061754	-3.91574348163595	3.57571884177526
O	-5.12920885728014	-1.21813364351179	6.59110211122323
O	-8.67410278855023	0.35365299361968	5.56900839213905
O	4.46281272298517	-2.50467072320197	3.24611517871717
O	-6.44812799694549	1.04618250559203	6.95140832610954
O	4.48690154281744	-5.05888391579269	2.60469364340597
O	-3.88637958410370	0.86007373111828	7.58097760544374
O	-2.25476230843054	2.85014983486115	7.13923468544179
O	0.39912091605654	2.65882865547319	6.89753414482566
O	4.25609613924600	-7.39436191151347	1.37425120005578
O	-1.50648794238722	0.86727030310222	8.77946338652783
O	0.71735970633162	0.07489115548721	7.51585047625677
Si	8.12626330752526	-7.12293873672980	2.28665186425097
Si	-2.12879133917075	-2.97542968236445	7.81883904548709
Si	8.19326195791233	-4.41206980729092	3.55278055255342
Si	-4.81007806181478	-2.81331822103096	6.58454956988497
Si	4.52578054606987	-0.88684824468141	3.37756015973307
Si	-8.08377801941816	1.12883623652371	6.87821461933726
Si	5.35403025817870	-3.69801526512221	2.61624569064354
Si	-5.30007447776994	0.07144262503522	7.56819004478672
Si	-0.92900662049424	3.48668511894778	6.44865934874912
Si	3.43998842880022	-6.09622345869796	1.92617246451702
Si	-2.74841598314235	1.78940965553005	8.26743379033739
Si	1.29721368290800	1.32480564507706	6.65391758788623
Si	5.41466178956214	-8.50733379296723	1.64881541625821
Si	-0.51538936739666	-0.38711890857440	8.47051614546643
O	1.80441436147855	4.27494634819717	-4.06096246485922
O	-0.07660271166210	5.27117764712980	-2.48750521376219
O	-0.57198028639089	7.71685033302934	-3.44309342956792
Si	0.70264628468499	3.91961439858887	-2.92257040593738

Si	-1.18814063867514	6.43282996371303	-2.66188059767780
O	1.24090058966871	-1.74199436885267	-4.48240775250108
O	-3.94339321104172	3.96271399140514	-4.54366556262707
O	3.34735669588235	-2.95823274854793	-3.48412571500191
O	-2.44781261057581	5.87416885783975	-3.51168050630190
O	-3.75515983163828	4.17569321315130	-1.90637206395783
O	1.34255204652931	-4.41731798370508	-4.41113845339943
O	-4.64081913163533	1.79918327216957	-5.93011090205193
O	4.72320092509809	-3.30517009069017	-1.25700713779590
O	-2.57505058483359	1.69588699099452	-4.33404177378215
O	-3.14583704588489	-0.34290881508834	-6.54523106610646
O	-1.09988295043899	-1.81561354785581	-5.68152781193990
O	-0.33647201771885	0.32159120958767	-4.25751164478762
O	-0.35685610670085	2.84418350418119	-3.49816138840858
O	1.79434433380833	6.93542447990543	-4.31202614031708
Si	-3.81570606153874	5.01585250638249	-3.30449567632669
Si	2.19461999166264	-3.04696423012185	-4.63705580356535
Si	-4.04936615947931	2.34001560046728	-4.52661174996200
Si	4.01270427133687	-4.07458872828289	-2.50702424267969
Si	-4.34138019635247	0.65629592674087	-7.06007440777192
Si	-0.31314029030902	-1.30077785147700	-4.36148522948013
Si	-1.20744127705612	1.46461142477858	-3.51101439877824
Si	2.56213735297469	5.56228268834819	-4.72258931672321
Si	0.65816910835174	8.08468756772370	-4.45575048111294
O	-2.79194356231665	-5.58103305917835	-7.10526001102211
O	-4.58365728120038	-4.36024464332051	-5.56621416622405
O	-2.94811460116594	-2.91455186607410	-7.20483721347013
O	-2.61335316310250	-0.07721349522180	0.16250278262173
O	-2.52641273311156	2.62643012750598	-0.15936449846526
O	-1.58102678936762	0.95401838066217	-2.01720008807206
O	-5.13985321558962	2.78371853810780	-0.04018946464871
Si	-3.77857871136128	-4.29609572003775	-6.97839475100796
Si	-1.78547456925965	1.20833705531228	-0.43982999214804
Si	-3.79398985602662	3.62112703537110	-0.38481524838925
O	-5.06216689603724	1.95541163333623	-3.31723282951232
O	2.82008130915053	-5.04972278827550	-1.98187680735881
O	-5.64781386500420	-2.48967542942541	2.81330310849679
O	-7.39680212875105	-1.79489579415204	4.69456425771480
O	-0.25881226751280	-6.30041950997152	-3.40780647063434
O	-8.00601273070690	0.16893393416161	2.99763609782778
O	-0.38075179001418	-5.57018237375762	-5.96945442503350
O	-1.00683808119420	-1.92670731570636	-3.03999317871931
O	-3.48472496308552	-6.49838132455481	0.73152687224879
O	-1.00122700123360	-4.26424942470693	-1.87526651919724
O	-6.65267290151833	1.05048872862263	-1.42076307163123
O	0.62430407366381	-6.06140331973628	-0.88200956238736
O	-8.38646838815669	-0.62042493083351	0.46888158403774
O	-7.35341233852617	1.76676176440904	1.02101675657865
O	-1.87367638825047	-6.74796167495200	-1.40369290607922
O	-6.49024666031157	-0.22057053565655	-3.71649754222620
O	2.70465926808847	-5.41731649212641	0.63895369972944
O	-9.08856621387696	-2.66888123340336	-1.04075211412382
O	-4.43344463707599	-0.26183084914640	-2.02852874653416
O	-7.00516325966341	-2.87413050546057	0.56738964960550
O	-8.00473901087600	-2.38375642128605	-3.46439193748548
O	-5.34113231676783	-2.60799865475258	-3.75036571652536
O	-7.26812059895730	-4.37682276868349	-1.87690764901347
O	-5.00679712082965	-5.15427842449746	-3.07641158168055

O	-2.86535656614845	-3.70664236079361	-3.72467259780346
O	-5.69037451113738	-6.15975557432169	-0.72060055263739
O	-2.95328413926317	-2.59477838476839	-1.34241041331274
O	-4.79043242126763	-4.21917720517134	0.93224520917084
O	-4.61102184036468	-1.56595690935182	0.68463070182970
Si	-6.48867692138651	-3.00808925649858	4.10703025177958
Si	0.60123052674278	-5.83272760288090	-4.70829383540159
Si	-8.49784127853763	-0.65387261145723	4.31604858913229
Si	-1.24335872668126	-6.06970939068950	-7.25774105487161
Si	-6.65804186363544	2.28303127275167	-0.36365108476103
Si	-2.43882256703884	-7.48622193476381	-0.05593994049497
Si	-0.62818138709602	-5.83400621592088	-1.89702360588277
Si	-8.43198943936913	0.64327369600101	1.49425989562652
Si	-5.63593709618724	0.60422744078993	-2.61056606517855
Si	2.24891590280644	-6.01800924974554	-0.81275544144762
Si	-8.49843902005228	-2.23515648761518	0.40699514086521
Si	-6.67652035071384	-1.76309605005610	-4.17615256028654
Si	-8.51618234571936	-3.44894218372626	-2.35396008318122
Si	-4.44566994290684	-3.94523183557737	-4.00784476415450
Si	-6.25871371556790	-5.62144684665375	-2.14603090907289
Si	-1.97085029289016	-3.11507285741670	-2.48473897123336
Si	-4.96301746412940	-5.83074211041146	0.69962829793114
Al	-3.69091492476899	-1.16120469393083	-0.79669428143985
Si	-5.52951018280803	-2.80644391018299	1.22216445425539
Si	-2.11358361696695	-1.54933693755542	-6.91805204259914
H	11.67353995006027	-5.53749694871332	-0.03813002778672
H	9.19325799004535	-8.10610399958537	2.36365699767815
H	-3.96156899422962	1.36419900455137	-8.28506100548702
H	-5.56398600411881	-0.12270100777071	-7.29597600022568
H	-6.93632899327768	-1.73777199906743	-5.60525598820989
H	-9.61070201633419	-4.23261600724605	-2.91602400713290
H	-4.80937901329507	-4.38478699196659	-7.98416797451275
H	-1.26201402795273	-7.53013897854558	-7.34713296325872
H	-8.46716052357666	2.53175378506338	6.77435861238838
H	-4.20618401215208	6.50892100722223	2.49273000772669
H	-1.81213400069374	9.00646799768804	0.20177799995816
H	0.23145495379310	10.95011902979720	-2.10809595634461
H	-4.94421500944109	5.93630900553076	-3.34769399968154
H	-7.43440301072539	3.36541401492032	-0.94274899871305
H	-9.81225000749429	1.14342200847202	1.44886400286110
H	-3.26051300212221	2.50761900616055	9.43266800411862
H	-0.74472599749957	4.85510199918195	6.90777200246571
H	1.17261700036015	6.84873800345368	4.60176300325226
H	0.01371299294086	-0.85239399612982	9.75320299709630
H	2.65644500018084	1.61373199835850	7.09816899901371
H	4.53016699900577	3.47572499677929	4.86954700125355
H	1.53638199507820	-6.93834199075837	-4.97336599637893
H	4.68077900373207	9.17388299731193	-0.65420599715635
H	-8.67443702948537	0.53296798981211	8.08822303295547
H	-5.67876701141750	-0.35798098372262	8.92693199707382
H	-5.40538800621264	-3.45793999638881	7.74582400426174
H	-2.86030599984238	-3.03941600213534	9.10082099201651
H	-1.22727301548959	-4.11134498201960	7.70409200023578
H	2.59107099321839	5.38865100451202	-6.17172399266438
H	0.22684598711038	8.11439300871542	-5.85590798145985
H	2.35648399058001	11.66568598707728	-3.24752299288960
H	6.05893599884035	7.26486299970675	-3.76938199783590
H	10.01162899772035	4.23690500243210	-2.76123799970131

H	9.87912999902293	0.42295200122802	-3.59486199488289
H	11.16666300064122	0.78647299920577	-1.60521600236440
H	9.89955900213359	3.90245199960594	-0.38701600022915
H	-1.31531301126209	-1.20647000624937	-8.10616498477197
H	-0.61432203814426	-5.50729800148871	-8.46106595147137
H	2.84834100533850	-3.04402000100843	-5.95043800857235
H	-7.31528201534575	-4.15742499292231	3.75037501102795
H	-9.78237300604421	-1.30797899647247	4.07942699776849
H	-9.40254699590863	-2.75213299341823	1.45267098440210
H	-5.76896400706009	-6.40939899774724	1.78048400674094
H	-3.08137199903285	-8.75069598958543	-0.46757200178604
H	-7.02203599758285	-6.69280398878673	-2.78388199437816
H	2.78306900810476	-7.36900602682450	-0.99232700835741
H	4.89569299335375	-9.57645901489763	2.49579400704228
H	5.81992199781431	-9.04892399437517	0.34487200763689
H	-1.34472300222411	-7.77598299946800	0.87489000179572
H	10.66941400047750	-2.02071799869691	2.56381701199426
H	8.82291800008289	-3.95596100141447	4.78390501185276
H	11.59057491276377	-3.97495102887707	-1.85889593056517
H	5.33835300673766	-0.46465099565814	4.51989001238257
H	7.15208499150301	1.34023099710031	2.37194499256627
H	7.32230800100514	6.57802299835624	-0.51391500214543
H	5.00675698246183	-4.86530398547290	-3.24361797664489
H	7.90182197831573	-7.00149495234652	-1.50647495879868
H	2.44258800748603	-6.50026700348482	2.91141100209226
Ga	-3.14743909067843	-0.77571178261676	2.00273601406759
C	0.56205727207493	-2.44966819642900	2.38869629321263
H	1.61288925795174	-2.26505493227771	2.18332446116094
C	0.01924571880110	-2.05241384175856	3.54864719171036
H	0.62371296731584	-1.57913939930390	4.31714716481120
C	-0.23692607418625	-3.15399160504296	1.39846447219485
C	-1.57676300067042	-3.19030500799709	1.50075068266369
H	0.26050167929890	-3.63392432127814	0.56139135832158
H	-3.73250904116975	0.47405286490745	2.71771105285057
H	-2.17431903297837	-3.68173079648913	0.74024997341801
C	-1.41207342899113	-2.37325831610713	3.87271011845088
C	-2.28336681752291	-2.49030098290540	2.61077314179116
H	-1.82310513088650	-1.64767146066734	4.58426523165591
H	-1.43434711658863	-3.34450451539540	4.40015519916166
H	-3.18880534078362	-3.05199361455298	2.87792004320624

I21-GaH 2

Electronic energy (r^2SCAN-3c): -30770.961650408797 E_h
Electronic energy (ωB97X-D4rev/def2-mTZVPP): -30779.295535316949 E_h
G_{corr} (673.15 K, 1 atm): 0.44978 E_h
Number of atoms: 254

Cartesian coordinates (Å)

O	1.48081029568475	2.93264790137931	1.03298613410008
O	-1.94670731713469	6.76507981711031	1.41457581795949
O	5.26816005497486	2.07618572482488	0.79968962514677
O	3.61022899864315	3.88022986673590	-0.15853701251572
O	6.09726784108205	4.57229912465047	0.41244425393487
O	1.44595619202082	3.30279063345521	-1.61156326394299
O	1.39534267398523	5.35817015432490	0.00652564573820
O	-1.71410166158783	6.90385352674439	-1.20497490315327

O	0.31374282266338	7.71608401591990	0.40162250360292
O	2.90364683688266	7.55005164839791	-0.06993147538430
Si	4.84986159740100	3.63809772620865	0.86554655474881
Si	1.99145013757374	3.86517301543897	-0.19104926672338
Si	-1.30180071930634	7.63189605090001	0.19584239432956
Si	1.65040298502309	6.82437657310020	0.64649949397056
O	8.94889882517272	-3.83289218345065	2.22534408268752
O	1.99960913815015	6.66975538575877	2.23010932242078
O	7.14581141454616	-2.28170952940349	-0.92176455858122
O	2.51462693747177	4.76532208316957	4.07041794566276
O	6.74775861671831	-4.90507234822680	-0.76651785214366
O	0.05421083406905	5.12005028063102	3.12978611539322
O	5.84839446150027	-3.26225143433402	1.13774452693831
O	4.45030760562309	4.07157306839736	2.38441749796149
O	9.40028831203737	-5.01101518716291	-1.12896862610220
O	5.15609939852234	-0.24845303902781	2.01669877379410
O	7.73073616360778	4.38330535532106	-1.69732426875655
O	10.70287090923477	-3.18647915274634	0.34652930634981
O	5.41932465938395	5.65369804916775	-1.90210747636762
O	4.09345220059228	5.67547771212786	-4.17411079024925
O	8.76370955999516	-1.45939609079315	1.04355425519942
O	3.89510468111616	7.75017773600422	-2.54802172885487
O	1.23934879789062	9.54756132933697	-4.03194164449566
O	8.97967203910753	2.07301261165352	-1.88658536720577
O	6.94896471541955	0.18943328411251	0.10143205972834
O	2.35924226553369	9.69314107206863	-1.58073374276962
O	8.99951827022694	-0.54770834783521	-1.44476104696097
Si	1.43739707366293	5.87260661034123	3.53923311257950
Si	6.11489963182260	-3.44032360694526	-0.44666850213276
Si	3.63468649729402	3.63112337407716	3.72421446441472
Si	8.07896348366161	-5.83689045852668	-0.65572561100661
Si	10.87570844210475	-4.45968248228528	-0.67221356211654
Si	6.66796419469487	5.33114583872029	-0.91323553371298
Si	9.79452739240672	-2.60874089967791	1.56647696864331
Si	4.91201420480278	6.60864922988528	-3.11764796296902
Si	9.20221906817088	3.67556083412642	-1.66848120447549
Si	6.17347651622530	0.85327198921078	1.37093890327486
Si	1.52851499870384	10.51940427100633	-2.73552729484488
Si	9.79991597014777	0.68431685096081	-2.15395599793960
Si	7.95648277713896	-1.01722236173143	-0.30358486989806
Si	3.47603971318116	8.56894882433028	-1.20485623411813
O	2.98181120828027	-0.46202152303395	3.61599340427886
O	0.97331430340165	0.78248679932690	2.46684194370343
O	1.24178646926305	0.88651314824879	5.08080239076588
O	2.96952400686380	2.16967412197199	3.48813206059369
O	-0.39101589981908	1.20249839939257	0.27275363748790
O	-0.82568696238090	2.73426244845781	2.35688632699841
O	-3.75739756144423	4.97083224076614	0.57687365225576
O	-2.53034841556979	4.66687195611018	2.93433687022702
O	-1.16494654123580	3.44035600683409	4.83862947945064
Si	2.04536904890742	0.84966347266019	3.67810140992177
Si	0.31299340503252	1.92443279199472	1.53307495934648
Si	-3.13446930803018	5.74276903661198	1.86515529305738
Si	-1.10312822996942	4.00254569859463	3.32704437658980
O	8.28285568672963	-6.04094498292840	3.49531038103646
O	-3.19884208780919	-3.07475948703756	6.58260143944586
O	6.68210719389409	-7.84891935001066	2.44317032794133
O	-1.31028461857699	-1.56836861736450	7.69131195160148

O	8.26105672672151	-6.34391102138594	0.87185633829055
O	-5.42817720848842	-3.48779717538493	5.24819012958677
O	6.64945493142244	-3.91674648930389	3.57265658461523
O	-5.13084250952887	-1.22064003276057	6.59055504377087
O	-8.67248939001787	0.34415734230534	5.57459582754204
O	4.46763928760204	-2.50930133697531	3.25037148190292
O	-6.44710120138123	1.04568770398041	6.95076226193201
O	4.49028225737177	-5.06308554470081	2.60807755678952
O	-3.88712109307920	0.86019595147205	7.58052043563734
O	-2.25295198996315	2.84943213140569	7.14274694363402
O	0.40014291132333	2.65840139121582	6.86637255884742
O	4.25615166655625	-7.39471824294428	1.37647335806268
O	-1.50881191700523	0.86640205668691	8.78368291073039
O	0.72165346169932	0.08252735737796	7.53098728225758
Si	8.12555811377778	-7.12432373730893	2.28719768666978
Si	-2.12810794604094	-2.97327740788218	7.81770990141587
Si	8.19637872409529	-4.41459571789474	3.55241874034743
Si	-4.80524511827616	-2.81553283566977	6.58606464291143
Si	4.53010555406043	-0.89184674245589	3.37700850231211
Si	-8.08267758817088	1.12784167787019	6.87899036642135
Si	5.35577034341362	-3.70290417676895	2.61575752296613
Si	-5.29948516970580	0.06925810675770	7.56751535060309
Si	-0.93411789436169	3.48925493340378	6.44322305089768
Si	3.43866146503671	-6.09537050278354	1.92675433296016
Si	-2.74896332360362	1.78791189676188	8.26885814301450
Si	1.29652833123728	1.31780310976171	6.64747787023652
Si	5.41381650602225	-8.50731575669780	1.64903207291575
Si	-0.51581047567511	-0.38636584214176	8.47403616916566
O	1.80053743488567	4.27978765035452	-4.05833768419153
O	-0.06998403395499	5.28619152016537	-2.47591395867945
O	-0.57711409826736	7.72015116949636	-3.44837315050255
Si	0.70017089439157	3.93069292657094	-2.91708976352837
Si	-1.19239794244826	6.43558520976843	-2.66796143964977
O	1.24988620534554	-1.73212189412406	-4.49418820841505
O	-3.95762777471369	3.95499921771283	-4.55128417775861
O	3.34368497035108	-2.96020767162922	-3.48337586574561
O	-2.43944815075795	5.86183064876395	-3.52870161974089
O	-3.77521345580398	4.18741162601365	-1.92427345945314
O	1.33304299960108	-4.41216102002341	-4.41420839127073
O	-4.65347568345668	1.79133790950441	-5.92698111613297
O	4.72073133760168	-3.30658992842466	-1.25793994994864
O	-2.55142852038484	1.70545329470196	-4.38032845373093
O	-3.15167724689762	-0.34492870639773	-6.54832154117791
O	-1.10368196597030	-1.78753169782506	-5.66604555250214
O	-0.31862024652472	0.33508342950106	-4.21498324648744
O	-0.36955603284247	2.86421103412285	-3.48854538884141
O	1.79371899491070	6.93765529433698	-4.31148718184554
Si	-3.81750448651022	5.01879916513812	-3.32362093963552
Si	2.19375353813313	-3.04649085589845	-4.63871693187970
Si	-4.03930602130702	2.33019377726876	-4.53307027922327
Si	4.00929513076635	-4.07779429734648	-2.50671325279286
Si	-4.34488046002001	0.65567255299299	-7.06186403300367
Si	-0.30016753547786	-1.28556360253200	-4.35079109375600
Si	-1.21413100027987	1.48226172827833	-3.50615749974777
Si	2.56110925521625	5.56436488055513	-4.72245629340086
Si	0.65751609101074	8.08670857841107	-4.45631434643910
O	-2.79439420465138	-5.58344039713493	-7.11444202894633
O	-4.54717144424280	-4.34631030082548	-5.54842304117014

O	-2.93019066282645	-2.91743696422393	-7.20412357874420
O	-2.80150369067076	0.10858929493939	0.17049465896037
O	-2.48580756668213	2.75307416721406	-0.14792431578586
O	-1.64994628922372	1.00600270293764	-2.01321620386117
O	-5.12575530467997	2.79947071103943	-0.04440164661680
Si	-3.77171866184034	-4.29245929595058	-6.97714135617525
Si	-1.84040597684067	1.29069253250334	-0.43660621288277
Si	-3.80577605617625	3.67657216666928	-0.39214456379849
O	-5.00708931966812	1.91714914388250	-3.29728924733962
O	2.81458373853090	-5.05099002681724	-1.98444591801224
O	-5.59881116090366	-2.50849306003208	2.82792236100725
O	-7.38365386759633	-1.78875684251793	4.67037827084167
O	-0.25517317879966	-6.31115412019705	-3.40614137284755
O	-8.00900674253519	0.18859296373980	3.00334324439259
O	-0.38889268430738	-5.57228043158393	-5.96459150393376
O	-0.97374189823531	-1.91553543742405	-3.02323982662501
O	-3.48982949573446	-6.51012291730069	0.73595524233522
O	-0.97468192820681	-4.26599786223436	-1.89218667249749
O	-6.61045550014420	1.04199668829488	-1.40752468627309
O	0.62576659836120	-6.07561244608464	-0.87929115811282
O	-8.35668403068199	-0.62799269913163	0.48247927191610
O	-7.35260196724863	1.77490076225362	1.01397628448715
O	-1.87901540496595	-6.73637831722714	-1.40034395678195
O	-6.51791589489592	-0.20710903891307	-3.72397189008124
O	2.71497902322530	-5.41580773678102	0.63542256289072
O	-9.07332989647873	-2.66738234821133	-1.03167881467630
O	-4.46229534185594	-0.37449846975872	-2.07422131186366
O	-7.00868098974200	-2.91016122054175	0.61213748391934
O	-8.03344736178420	-2.37033204721601	-3.46778188912270
O	-5.38038359264213	-2.61316584619563	-3.74390904675315
O	-7.26270665147863	-4.36705499035943	-1.88895685892490
O	-5.00248210137429	-5.15315540755000	-3.07250649907936
O	-2.89473097122810	-3.63692812906941	-3.64882616486849
O	-5.69733383581059	-6.15623883679342	-0.71894998886242
O	-2.81171571688672	-2.53833126494654	-1.21885976902732
O	-4.76350552606671	-4.21788310567597	0.91464584753167
O	-4.65953047004357	-1.54636781820148	0.67374857546484
Si	-6.47603637837283	-3.01406854522673	4.10718452825018
Si	0.59941246916407	-5.83382680539001	-4.70667405165347
Si	-8.49557986576853	-0.65214587010469	4.31283544229310
Si	-1.24415617485075	-6.07061059965789	-7.25831920211520
Si	-6.64315454554433	2.28696552131608	-0.36614285190988
Si	-2.43711724896673	-7.48718653176522	-0.05609759482255
Si	-0.62096597204364	-5.83937555123460	-1.89513225827973
Si	-8.42629207681321	0.64797749730976	1.49311235979580
Si	-5.62335862867614	0.57216995936336	-2.61450315628649
Si	2.24922166868796	-6.01865185294425	-0.80954404371532
Si	-8.48619721105048	-2.24278235946708	0.41996832075505
Si	-6.70173697861914	-1.75391377492425	-4.17561204951014
Si	-8.51926927544255	-3.44411127742690	-2.35385782132432
Si	-4.45255545831849	-3.92614766094625	-3.98830736714137
Si	-6.25932021698357	-5.61911161582867	-2.14761632430512
Si	-1.94031907338384	-3.08639485160528	-2.43882389180597
Si	-4.96144526320425	-5.83143896494574	0.69769870931411
Al	-3.68302308473636	-1.14557034477161	-0.77666711335527
Si	-5.51779763013937	-2.82205682069129	1.23515334952610
Si	-2.10985482807055	-1.54395545130099	-6.91389463469483
H	11.67354000262543	-5.53749699640661	-0.03813000440327

H	9.19325799737771	-8.10610398960237	2.36365699805997
H	-3.96156899539099	1.36419900326256	-8.28506100644429
H	-5.56398600334909	-0.12270100639329	-7.29597599472967
H	-6.93632900644700	-1.73777200261214	-5.60525604803834
H	-9.61070199522731	-4.23261600001119	-2.91602399499931
H	-4.80937900865035	-4.38478699680672	-7.98416801745144
H	-1.26201400117746	-7.53013898882888	-7.34713300424139
H	-8.46716099842831	2.53175402475599	6.77435899465137
H	-4.20618400474482	6.50892100633285	2.49273000083786
H	-1.81213400220643	9.00646800114511	0.20177800011743
H	0.23145496793273	10.95011887109719	-2.10809596497209
H	-4.94421501700272	5.93630902558772	-3.34769400066760
H	-7.43440301632222	3.36541403160525	-0.94274899862551
H	-9.81225000478889	1.14342200462374	1.44886400227744
H	-3.26051299201280	2.50761899480363	9.43266798954723
H	-0.74472599830664	4.85510199684969	6.90777200141424
H	1.17261699912510	6.84873800080137	4.60176300062261
H	0.01371300553636	-0.85239400850238	9.75320299143587
H	2.65644502090510	1.61373200088096	7.09816899422546
H	4.53016699272656	3.47572499622662	4.86954699843829
H	1.53638201241483	-6.93834201062571	-4.97336600668448
H	4.68077899759732	9.17388299716479	-0.65420600266397
H	-8.67443699938250	0.53296800385524	8.08822300072637
H	-5.67876700029815	-0.35798099501818	8.92693199749431
H	-5.40538801721611	-3.45794000535867	7.74582398439799
H	-2.86030616926888	-3.03941591825876	9.10082113438004
H	-1.22727278861846	-4.11134506790047	7.70409193935866
H	2.59107099785611	5.38865100043671	-6.17172399371285
H	0.22684599710434	8.11439299945485	-5.85590800306999
H	2.35648401652326	11.66568600953371	-3.24752300822540
H	6.05893599949249	7.26486299996705	-3.76938199803193
H	10.01162899724599	4.23690500176463	-2.76123799987006
H	9.87913001033449	0.42295200629361	-3.59486199908509
H	11.16666300200578	0.78647300443656	-1.60521601117257
H	9.89955900619108	3.90245199968169	-0.38701600015240
H	-1.31531299554473	-1.20646999687642	-8.10616500648824
H	-0.61432199387820	-5.50729799902061	-8.46106600889771
H	2.84834103427869	-3.04402000082725	-5.95043807026241
H	-7.31528200197894	-4.15742499837942	3.75037499237124
H	-9.78237298838450	-1.30797898871093	4.07942700397759
H	-9.40254699358340	-2.75213299637833	1.45267099654916
H	-5.76896401086971	-6.40939900509789	1.78048401654767
H	-3.08137200217306	-8.75069603739959	-0.46757201026810
H	-7.02203600017488	-6.69280401026221	-2.78388200590624
H	2.78306902065822	-7.36900603964162	-0.99232703625828
H	4.89569299654700	-9.57645897725737	2.49579400800694
H	5.81992201792807	-9.04892402183037	0.34487196303822
H	-1.34472302314151	-7.77598297741971	0.87488999098224
H	10.66941399826187	-2.02071799783521	2.56381699581941
H	8.82291798752133	-3.95596098374820	4.78390500283036
H	11.59057500256544	-3.97495100561958	-1.85889599538369
H	5.33835314289195	-0.46465091568771	4.51989017817313
H	7.15208491774447	1.34023094603196	2.37194493228397
H	7.32230800217441	6.57802300680584	-0.51391500657175
H	5.00675693519872	-4.86530395971564	-3.24361797849527
H	7.90182201020156	-7.00149500826662	-1.50647501349578
H	2.44258794212849	-6.50026702751720	2.91141106678828
Ga	-3.67468657453601	-0.27966827327447	1.96562255088960

C	0.82317294396767	-2.72872695823603	2.15866173468269
H	1.74377179424528	-2.22768647548191	1.87486813966918
C	0.56360923306484	-2.99515619772590	3.50076868561753
H	1.28462640865429	-2.70091857375715	4.25871140412778
C	-0.09803756185837	-3.10133393645336	1.18420322210455
C	-1.27813423777771	-3.74432822915155	1.54935217418854
H	0.10425252880864	-2.89723825615307	0.13670369758990
H	-4.64695441450168	0.88316498016458	2.25106847444823
H	-1.99870353267810	-4.02617601041940	0.78838026362566
C	-0.61939123874955	-3.63383422190581	3.86683208439832
C	-1.53970390259687	-4.00930128270849	2.89134134431241
H	-2.66170553375760	-1.11787474565843	2.76724067401059
H	-0.82617401486061	-3.83916594516956	4.91337858741766
H	-2.46562409881967	-4.50277697840000	3.17651626133499

TS1-GaH 2

Electronic energy (r^{2}SCAN-3c): -30618.482003404341 E_h
Electronic energy (ωB97X-D4rev/def2-mTZVPP): -30626.673415987967 E_h
G_{corr} (673.15 K, 1 atm): 0.44058 E_h
Number of atoms: 250

Cartesian coordinates (Å)

O	1.53247537538723	3.00008006024254	1.07487225939545
O	-1.94487306422257	6.77184308801775	1.43014508371140
O	5.26440390709820	2.08271710960487	0.80764677540162
O	3.63197580577789	3.90449860304935	-0.17408573415854
O	6.11157748310339	4.57489899782057	0.42294816905613
O	1.44807116855164	3.29211249683737	-1.59544079907293
O	1.39898555622125	5.37697923884211	-0.02512471352660
O	-1.68718631406890	6.87745418934010	-1.18784838856952
O	0.32365175871611	7.71952329057718	0.43179314450749
O	2.94251428834357	7.52453486380348	-0.00878242293656
Si	4.85595080410557	3.64777270770234	0.86758297147952
Si	2.01369571566801	3.88944831979465	-0.19626335440326
Si	-1.29182518741566	7.62920238541721	0.20682850068767
Si	1.65547762659921	6.81881326540588	0.66697265647154
O	8.94984496000016	-3.83261058728788	2.22502985767150
O	2.00275461769561	6.63355904012235	2.24478219658314
O	7.14595773490948	-2.27925587084263	-0.92044977498759
O	2.50014385954057	4.73924704970254	4.08854608656747
O	6.74920130718423	-4.90406686406544	-0.77072561998017
O	0.04058385190216	5.12203046220963	3.15557564090040
O	5.85309915049619	-3.26741742542547	1.13760436733834
O	4.44110316549261	4.08542643967319	2.38211206947409
O	9.40148018387329	-5.01159195432545	-1.12884938189747
O	5.15201650751506	-0.24547455371731	2.01914981201559
O	7.73795270013077	4.37871515852673	-1.69322338373695
O	10.70124087481803	-3.18445445743865	0.34525595259422
O	5.43471150334837	5.62434487319851	-1.91320704318136
O	4.12604110882820	5.70157223893203	-4.20611010620105
O	8.76186446615033	-1.45737521267375	1.04607054329702
O	3.91462486203058	7.71845832208219	-2.51272950948272
O	1.17203735045698	9.52913678877679	-4.05568110929743
O	8.98143363412034	2.07115556266467	-1.88637040466702
O	6.94517134633979	0.19177882183462	0.10342692632687
O	2.27048459411673	9.55277213757559	-1.57170294182233

O	8.99884430273046	-0.54677849786403	-1.44191812002943
Si	1.43292038649589	5.85652598778358	3.55838546939489
Si	6.11657769571277	-3.43986586748988	-0.44813429891453
Si	3.63824999952335	3.62876572007173	3.72429417017572
Si	8.07952130676369	-5.83640158435613	-0.65676120552497
Si	10.87621921652642	-4.45874384244804	-0.67203511521481
Si	6.67731465267714	5.32524368239278	-0.90982240154117
Si	9.79400805030697	-2.60715499678607	1.56630146938547
Si	4.93311833666840	6.59790733747608	-3.11374581658945
Si	9.20767396971293	3.67210579740385	-1.66813159010139
Si	6.16984778554409	0.85569148567589	1.37370368169388
Si	1.72814805366430	10.45534843926051	-2.82976696598405
Si	9.80068525722573	0.68249905803465	-2.15354321019912
Si	7.95456944765815	-1.01432068372156	-0.30100773760130
Si	3.46945117681894	8.52593401340467	-1.17428304820171
O	2.97085975911740	-0.47420599041129	3.60709927227317
O	0.99200867642489	0.81191607331707	2.44277796485784
O	1.23934030283575	0.89675073083675	5.06659863741244
O	3.00104591512696	2.15482666108372	3.48755677360183
O	-0.29229814984191	1.30996382874746	0.20238299192998
O	-0.81469829268053	2.74948366205496	2.34102662157755
O	-3.71118206767578	4.95086256870930	0.57587482867810
O	-2.53650899964307	4.66724678237250	2.95175812014283
O	-1.17103186823679	3.41242872349504	4.83786401362238
Si	2.05635042016058	0.85156419814245	3.66955578339566
Si	0.35471557883753	1.98333885111945	1.52122122976230
Si	-3.12984270743154	5.74005120487261	1.87115260723019
Si	-1.11107182836475	3.99842774528288	3.33476954029606
O	8.28873788072032	-6.04251327586566	3.49576094268063
O	-3.19772764874454	-3.05703494021703	6.57974006466039
O	6.68207696132074	-7.84693280216682	2.44433479200275
O	-1.30763373810667	-1.56809190114199	7.69161365220180
O	8.25810730343889	-6.34241758861782	0.87189184334653
O	-5.43859898593953	-3.50413145915972	5.27552497243820
O	6.65162213340205	-3.92395583003168	3.57352184161330
O	-5.14512738237818	-1.22217239431743	6.60254660767338
O	-8.65203822537909	0.33845339167821	5.56774969180769
O	4.47501884454691	-2.51245749109379	3.25187932915846
O	-6.44298394323492	1.05806508719865	6.96038329329174
O	4.49168219754501	-5.06552892577052	2.60797445187448
O	-3.88101163094603	0.85342521404963	7.58006090952358
O	-2.25298353262754	2.84941071115113	7.14677443140274
O	0.40176681585011	2.65896062406739	6.86408827035735
O	4.25579108454072	-7.39947417676084	1.37560230701931
O	-1.50325324795807	0.86886569796010	8.78372386783004
O	0.71830967127806	0.08127662739252	7.51811623918902
Si	8.12683091194086	-7.12381550420644	2.28683472663084
Si	-2.12824168174795	-2.97311272324692	7.81701501087076
Si	8.19984382011389	-4.41634373142021	3.55234158986746
Si	-4.80798237138423	-2.81234253995183	6.59446629375196
Si	4.52560767283844	-0.89525529530824	3.37700205940328
Si	-8.07926937722143	1.12778905279717	6.87660645977772
Si	5.36045902539686	-3.70538757208905	2.61559839998048
Si	-5.30089787155969	0.07219441583657	7.57043510617172
Si	-0.93586376190019	3.48583818643799	6.44318217496513
Si	3.44392550348761	-6.10010147575898	1.92640936119396
Si	-2.74874086793116	1.78512732104098	8.27123837332585
Si	1.29705700326349	1.31866476923772	6.64005677071070

Si	5.41719463445944	-8.50912832663413	1.64893515587530
Si	-0.51335833070268	-0.38595178651993	8.47250645166934
O	1.87555618030285	4.28999152057168	-4.02154364549927
O	-0.07670349754226	5.24893587933011	-2.49026736404696
O	-0.59839969398917	7.68326498462762	-3.45560558139403
Si	0.74107887112895	3.92086607106832	-2.92539252430650
Si	-1.19763140247164	6.40332111208650	-2.66100080447305
O	1.21951974685434	-1.76884747821639	-4.46616751252356
O	-3.99703221758420	3.94778140151380	-4.54653597681179
O	3.34609663280697	-2.96088053263497	-3.48535612268735
O	-2.45114390887825	5.84357033794746	-3.52001778241600
O	-3.75777671705574	4.17749519423140	-1.92145127741350
O	1.35361535071010	-4.43393901588306	-4.41711080205843
O	-4.67015414318900	1.76646105499344	-5.90876898601381
O	4.72250948545196	-3.30488798754852	-1.25812353856222
O	-2.49327997986095	1.76823593556413	-4.46921151693314
O	-3.16789193809328	-0.36984533936697	-6.53474745264286
O	-1.12655713295356	-1.83472381897924	-5.65779367551725
O	-0.31661580938731	0.31192343327754	-4.27868235816276
O	-0.27034594791885	2.84905086689923	-3.57868947745947
O	1.79726669613754	6.93867680436317	-4.27918457784458
Si	-3.83264654429674	5.00712259764977	-3.31897896212240
Si	2.19178169203024	-3.05685673952673	-4.63649315661977
Si	-4.01782200845482	2.32029254538536	-4.53812512113252
Si	4.01095903448244	-4.07630565685663	-2.50695199720688
Si	-4.32415171386093	0.66527355271795	-7.06796325783481
Si	-0.32985672668420	-1.31364680691926	-4.34419682824427
Si	-1.16549506052294	1.50354204006356	-3.59518483590494
Si	2.58295266108074	5.58425574949107	-4.71486369158561
Si	0.63346882214326	8.05559932281642	-4.45727133507704
O	-2.79606800505725	-5.59535980167070	-7.12481979258451
O	-4.51917095859425	-4.35468997505453	-5.54143872991211
O	-2.92769004277960	-2.92991067579209	-7.23387433528022
O	-2.51454790025113	-0.01670670826034	0.09488313459443
O	-2.51503955085093	2.68446515750166	-0.15538826614563
O	-1.60249057621773	1.07387670890701	-2.09195918749675
O	-5.14925851345687	2.83631583723168	-0.04999294354450
Si	-3.76782518399578	-4.30125060259841	-6.98134822825013
Si	-1.74234698597743	1.30862852063826	-0.51413525727446
Si	-3.80124565795507	3.65795919977714	-0.39326706334079
O	-4.89888248247737	1.86139842796793	-3.25857807190468
O	2.81927310255272	-5.05055305835032	-1.97913259340287
O	-5.58809623316935	-2.68121983452597	2.82686214894482
O	-7.36762766197607	-1.79284563606678	4.64891685554598
O	-0.25614906756943	-6.30362973218312	-3.39808691679391
O	-8.00252318894232	0.17448744628198	2.98869229815697
O	-0.39539355241990	-5.57463711078459	-5.95959981504662
O	-1.03054266139260	-1.88300743919912	-2.99944947204302
O	-3.49224192533573	-6.51843734663280	0.74193825407343
O	-0.94636652594712	-4.24610539039450	-1.87414035991100
O	-6.61015859539567	1.05706960623505	-1.42263097601319
O	0.62715494654129	-6.07622614796298	-0.87086551346855
O	-8.38354764338189	-0.62198714148033	0.46360548026260
O	-7.35601136057371	1.76922633991815	1.00957468418090
O	-1.87881228182212	-6.72174818070328	-1.39406233760948
O	-6.44584736594806	-0.22700238701574	-3.70286530778850
O	2.70922888788064	-5.41879892012153	0.64208218945987
O	-9.07679129337883	-2.67640462026468	-1.03502264468785

O	-4.44562442129635	-0.38118896515269	-1.94005140231450
O	-6.99781037795308	-2.87375058076341	0.59329001788549
O	-8.01171573980410	-2.37247960491749	-3.46157859998139
O	-5.36079477052866	-2.62730227845700	-3.74569353669619
O	-7.25771667265282	-4.37552594107192	-1.88174564246506
O	-5.00195097327820	-5.17110513360478	-3.07246376480918
O	-2.88623759330446	-3.66003474554060	-3.61173636165229
O	-5.70111880251799	-6.19891525727783	-0.72547314036142
O	-2.84196463558484	-2.58836731124398	-1.16099158407049
O	-4.76232138547307	-4.21387929502414	0.81086045270739
O	-4.64319977020328	-1.50782256523310	0.80414808406161
Si	-6.47799987278294	-3.04660042453301	4.12191868132253
Si	0.59592636889496	-5.84085821599720	-4.70496384763908
Si	-8.48555529148246	-0.66065053725732	4.30444181409451
Si	-1.24257519638295	-6.07055961958947	-7.26043822676696
Si	-6.65046042641498	2.28853471502882	-0.36868477813917
Si	-2.43925178304552	-7.48553908918109	-0.05701712940542
Si	-0.61934640665245	-5.82537485127425	-1.88787717277974
Si	-8.43250978806523	0.64411556380820	1.48480227145333
Si	-5.57501851996730	0.54700548477293	-2.57030112197362
Si	2.25025942724472	-6.01943088447512	-0.80926719240279
Si	-8.48789339951851	-2.23965494083722	0.41311128522215
Si	-6.67782401081786	-1.76168473768849	-4.17272860599404
Si	-8.51116730412480	-3.44988189560641	-2.35363586430318
Si	-4.43745799767397	-3.94430684498836	-3.97731886151033
Si	-6.25794872691336	-5.63105236953213	-2.14387145521120
Si	-1.95542239618669	-3.08600827003461	-2.39253673325831
Si	-4.96301223126891	-5.83494045821099	0.68282026894416
Al	-3.67206263882664	-1.18230420741051	-0.65341503082991
Si	-5.50589586180228	-2.83440068426752	1.22539337369427
Si	-2.11669757025616	-1.55864783825316	-6.91367317539425
H	11.67374699848072	-5.53703600421345	-0.03807401109165
H	9.19348302752889	-8.10585804530100	2.36346303413582
H	-3.88155000886412	1.39981098935661	-8.25725798674703
H	-5.54661899577891	-0.07985099655893	-7.37196300530664
H	-6.93206200098221	-1.73335100211541	-5.60666701042037
H	-9.61083500447362	-4.23371600286174	-2.91527899895116
H	-4.80806200212655	-4.38616401572956	-7.98466799660679
H	-1.25558098189833	-7.52827104263359	-7.35483800313728
H	-8.46603500888364	2.53218793084371	6.77163500520534
H	-4.20950399722384	6.50518799920472	2.49497300197010
H	-1.81143199203489	9.00591898271602	0.20164700171966
H	0.67612816615006	11.32783157175827	-2.29765517488943
H	-4.95336997518468	5.93157197727617	-3.34598499271249
H	-7.43212799123108	3.36467298506736	-0.94325498917064
H	-9.81433499665104	1.14100199235583	1.45030700981576
H	-3.26082300389822	2.50672899949587	9.43326900645721
H	-0.74504100316737	4.85495099423476	6.90563299607767
H	1.17176499788758	6.84606698631826	4.60638299339890
H	0.01383699998133	-0.85268900514336	9.75320601245574
H	2.65630400963068	1.61378700007953	7.09828800478868
H	4.53076899790854	3.47379799229000	4.86924399874981
H	1.53720799987047	-6.93798300072629	-4.97365700027739
H	4.63541500626601	9.21535599510155	-0.64625199564761
H	-8.67488801837249	0.53574598023646	8.08886504590073
H	-5.67875100051140	-0.35844199742910	8.92691799577926
H	-5.40516099340802	-3.45826599831243	7.74596998331379
H	-2.86007299727298	-3.03986700643991	9.10083199218577

H	-1.22701000319548	-4.11162699266584	7.70400900563950
H	2.57413299964021	5.42807899183427	-6.16363200628222
H	0.22482900090303	8.10275899246729	-5.85603201329477
H	2.79507591563549	11.25689296598081	-3.42192595037514
H	6.06067100109707	7.26917499470075	-3.76691099705097
H	10.01064999776603	4.23779399748937	-2.76113100044728
H	9.87926499637054	0.42371399888358	-3.59460899868251
H	11.16639499865955	0.78744100019332	-1.60472499961490
H	9.89847200089591	3.90341200058608	-0.38688399733121
H	-1.32399900342390	-1.19594401149104	-8.10330098761577
H	-0.61662902210662	-5.49814400616753	-8.46011598242764
H	2.84818299871667	-3.04332200245741	-5.95038399214709
H	-7.31517900284680	-4.15812801335491	3.75060701407409
H	-9.78250007293294	-1.30915806777801	4.07996999403521
H	-9.40319700326395	-2.75383201135046	1.45013701594184
H	-5.76898901185709	-6.41021300985312	1.77986100841001
H	-3.08084500228362	-8.75082999556951	-0.46797099797299
H	-7.02180600709034	-6.69287800527294	-2.78419799895541
H	2.78359000257792	-7.36886301170454	-0.99247300311793
H	4.89691600147639	-9.57737998552939	2.49409399706958
H	5.82084500004225	-9.04713700770969	0.34374498672108
H	-1.34437700266970	-7.77605499575940	0.87467399911186
H	10.66950899069830	-2.02029702338261	2.56380700273306
H	8.82314099548312	-3.95574102301011	4.78387698335579
H	11.59079999571440	-3.97453400155052	-1.85887999159213
H	5.33815600831126	-0.46451100955719	4.51995500846693
H	7.15219500216481	1.34004699702197	2.37234699952994
H	7.32251199028045	6.57841098647547	-0.51330699629123
H	5.00684299905667	-4.86484799862931	-3.24385799413075
H	7.90214799997216	-7.00114299629936	-1.50659200683248
H	2.44258299877619	-6.49974700256474	2.91143200234819
Ga	-4.09811767620946	-0.10757208181780	2.49701610720295
H	-4.70940286527160	-0.53088908399972	3.85165062235521
H	-4.41674719338198	1.21573122000927	1.76107517760489
C	-2.07098384617372	-2.27713206985403	2.79868395966508
H	-2.12298975965752	-2.78849342000977	1.83047547999350
H	-2.91364807917838	-2.62785668798072	3.40285392701612
H	-1.15119093101627	-2.60531301974985	3.29472545448952
C	-2.09033044355932	-0.75130945111246	2.62502677977789
H	-1.14162658828914	-0.44494699269132	2.16156851950175
H	-2.02238270205110	-0.24047121391636	3.59971808854442
H	-2.40554052049382	-0.24347260815337	1.24289278193033

TS1-concerted-GaH 2

Electronic energy (r^2SCAN-3c): -30618.456282470866 E_h
Electronic energy (ωB97X-D4rev/def2-mTZVPP): -30626.642227356097 E_h
G_{corr} (673.15 K, 1 atm): 0.42676765 E_h
Number of atoms: 250

Cartesian coordinates (Å)

O	1.59309487272722	2.84184355077565	0.99460391403130
O	-1.95407786973960	6.64436017281950	1.31110374551797
O	5.21835565032095	2.03391801061180	0.80396676544908
O	3.64771429582955	3.97140617478960	-0.12779470890144
O	6.17989974727830	4.47332493838177	0.37254234400734
O	1.54225311383530	3.34610604023491	-1.64842881696251
O	1.32672505678382	5.28804140969313	0.09900058932458
O	-1.78304409093528	6.96751387855605	-1.27109053989275
O	0.29602490684240	7.68906235176141	0.30365934646410
O	2.90860031428548	7.42120978127306	-0.11765620271775
Si	4.89046157512912	3.61942130707141	0.86300918357837
Si	2.03523089927205	3.85795693089853	-0.19344760874933
Si	-1.33340909413474	7.61080656745865	0.15921354744439
Si	1.61606010532599	6.79160637763931	0.63094751374944
O	8.93697757570266	-3.81514331947321	2.20602284409100
O	1.93042523594387	6.74970983032049	2.22874835941323
O	7.22278790674124	-2.34271196355330	-0.84144256634802
O	2.54069832524055	4.78111292544534	3.99123867829982
O	6.69767266010913	-4.93907655631757	-0.75405688762555
O	0.05124340439500	5.14836455569808	3.18307500839107
O	5.76911112035294	-3.29723652480802	1.13142585697354
O	4.53042474696984	4.04785428973991	2.39369818782380
O	9.36148512182068	-4.95683045174337	-1.07340845510177
O	5.17815829102634	-0.27070695236396	2.03364442599377
O	7.76715620783763	4.44818267073735	-1.76462766564143
O	10.74079451805193	-3.19950384408077	0.37585552252481
O	5.41314207741387	5.66924109287085	-1.86524912476300
O	4.07482539382521	5.63250878595323	-4.11202125997959
O	8.84459593493206	-1.39265154124975	1.03554370895282
O	3.97363348167029	7.79408868198654	-2.55194069508618
O	1.16363846133284	9.52799972724389	-4.05030226351755
O	8.97341618926823	2.07601035349866	-1.85513866497149
O	6.93866578290368	0.16194092678938	0.10429659485129
O	2.26548701645738	9.54703105645681	-1.57126419606863
O	8.96309247106072	-0.54345123698990	-1.48842732465109
Si	1.44286252090628	5.89791126595510	3.53614363103719
Si	6.11118132384765	-3.45430955043430	-0.43665163393103
Si	3.66106120344295	3.63114681571060	3.70406531866419
Si	8.05863336572953	-5.83500955842669	-0.64865484158068
Si	10.87409161159234	-4.45584092333354	-0.66552818612753
Si	6.68795590816253	5.32533570280873	-0.92232571550291
Si	9.80801331921601	-2.59559887334010	1.56364192752278
Si	4.92559100427492	6.59998337191317	-3.10818690785850
Si	9.20588479637234	3.68164884283313	-1.67327262865998
Si	6.17277120107023	0.83603807949306	1.37204749736603
Si	1.72604800108055	10.45429215511973	-2.82836329050510
Si	9.79332653490460	0.69454350177855	-2.15456829798884
Si	7.97960974529376	-1.01886774750563	-0.29721555556694
Si	3.46345161592986	8.51824123624675	-1.18429383540602
O	2.97719332271715	-0.43053169948694	3.60395345719672

O	0.90340233844102	0.81750364768119	2.51503640557501
O	1.24209797886179	0.87695882403498	5.09485362695435
O	2.96251024264622	2.17999685050437	3.50232550302001
O	-0.34468116385330	1.23685383270175	0.22472198191370
O	-0.79114903504658	2.82784258012957	2.26322795894403
O	-3.84312515203134	4.84632343314492	0.68012712702192
O	-2.52592740175261	4.68979651840029	3.00393846813340
O	-1.13070935102998	3.38029300476809	4.81822994419529
Si	2.02595177687887	0.86770245626187	3.68017720100961
Si	0.33984289661402	1.95453084543214	1.50252143048628
Si	-3.15324846407573	5.69487814120747	1.88351103031127
Si	-1.09083902325170	4.01407373330471	3.32654288792504
O	8.27976064684957	-6.03503219402150	3.49042748261006
O	-3.19752366295757	-3.08861105529557	6.57627358697866
O	6.70087047799462	-7.90592605209320	2.49520390206479
O	-1.34603579156410	-1.54719668733955	7.69063294972337
O	8.18265451194003	-6.35516747294706	0.88000847230142
O	-5.47060754745467	-3.50955088865768	5.28213685037388
O	6.65360648622560	-3.90462824419133	3.54780987182875
O	-5.10605683768638	-1.22558077057788	6.59643206365080
O	-8.73648873498505	0.28703312767474	5.64370035478662
O	4.45963512393571	-2.50370936737564	3.29478220090385
O	-6.45716690952583	1.03005402456691	6.92945862861118
O	4.41501172644911	-5.02387879189641	2.66880630705084
O	-3.89535970534236	0.87325934615356	7.58352377799613
O	-2.27543343131351	2.87728678200751	7.13316645430271
O	0.38070225659947	2.63476116201019	6.88782719907501
O	4.32622637400380	-7.33937040889807	1.37026734522856
O	-1.48008484592120	0.90250961648622	8.73853294655077
O	0.74749826527362	0.05856562946227	7.54924367827383
Si	8.11684920905540	-7.13567845852286	2.29678837765950
Si	-2.12986577660668	-2.97311548312993	7.81577912668424
Si	8.20275424994175	-4.40942626098802	3.53772322501173
Si	-4.80312616824169	-2.82415739003285	6.59025286135909
Si	4.51823938333438	-0.89038034563512	3.38755119939283
Si	-8.09363155892703	1.12241343989465	6.88349112100888
Si	5.33051581852626	-3.69505395993972	2.63429654441901
Si	-5.30253134008208	0.06814373697961	7.56154281500964
Si	-0.93635579089558	3.48084143743456	6.43394132173929
Si	3.44029058776306	-6.09220926248235	1.93848460486592
Si	-2.75254648396740	1.80246224868936	8.25715313804537
Si	1.29715198835251	1.30424084626977	6.65997037136392
Si	5.42790559242085	-8.50708194722323	1.65516365532567
Si	-0.51777608789409	-0.38237022316824	8.47253990478153
O	1.74478352023257	4.29456834736153	-4.10924625468561
O	-0.09246842583319	5.26231545958508	-2.42002157900296
O	-0.60599892496421	7.65567973658881	-3.49601771066604
Si	0.70469417903810	3.93593330963393	-2.91826944028310
Si	-1.21949629100692	6.39990411435680	-2.68067257682813
O	1.21208833923148	-1.77086931084403	-4.49323099752431
O	-4.00026009139129	3.89425372497157	-4.49842593020375
O	3.34837621046968	-2.93339067908465	-3.49161971886623
O	-2.44465266146165	5.77553691922494	-3.53212823436085
O	-3.84452694246302	4.25663427899795	-1.85750355792702
O	1.37385252083242	-4.45042849882042	-4.36165055485607
O	-4.67234531233913	1.71652122375266	-5.88444264758320
O	4.79114612536406	-3.20934865691241	-1.33766791232809
O	-2.53406985149677	1.68916858289830	-4.40349418445380

O	-3.18397090540913	-0.40346216844041	-6.55736079346099
O	-1.14976290568575	-1.84208333400130	-5.65527451874349
O	-0.29318587110591	0.32508573173356	-4.23600060119042
O	-0.34589562555232	2.84503554771181	-3.47511044824048
O	1.81648121136060	6.95293187133241	-4.26836613776367
Si	-3.85096597076529	4.99014691976461	-3.30620925359609
Si	2.19044757124249	-3.06275863183411	-4.63384555353709
Si	-4.05086848313321	2.26932460959085	-4.50494168920159
Si	4.03458199611325	-4.04475966141055	-2.51583122974579
Si	-4.32453194857449	0.65175846592263	-7.07201314827912
Si	-0.33290167913860	-1.29702128158142	-4.36312272585680
Si	-1.19567379132648	1.46921078341435	-3.52845521533998
Si	2.56013553337422	5.57558731008524	-4.71340152624703
Si	0.63778289578268	8.05316008583717	-4.46465542813555
O	-2.79047473138166	-5.59675492769857	-7.01344169786754
O	-4.53984992227323	-4.19188944724397	-5.58092564978883
O	-2.88263147183763	-2.94777741991747	-7.32070527031510
O	-2.69581074930043	0.06550367187442	0.15245383775231
O	-2.48147492850512	2.73738873193829	-0.20603673459672
O	-1.64893841461661	0.98802941636305	-2.04444122862782
O	-5.10758086805378	2.63366946942409	-0.12831277339092
Si	-3.75216011865432	-4.28436104814042	-6.99645881363803
Si	-1.80965270863111	1.28493328655276	-0.46566483287321
Si	-3.84174558704251	3.61976580143571	-0.37880621465280
O	-4.93654867678109	1.81510058801736	-3.22634053715450
O	2.86190360048656	-4.99995998196360	-1.92071271741496
O	-5.63824242103336	-2.51626145081826	2.86686040342109
O	-7.39013173285394	-1.76262155483490	4.68154029555578
O	-0.33826383181668	-6.30701128840047	-3.45541976422278
O	-8.04021429454963	0.29853796941698	3.08486236393353
O	-0.30376253602023	-5.54036580410474	-6.02462751170072
O	-1.03497325591946	-1.87036468086080	-3.02636711504031
O	-3.49507895029390	-6.55860065566613	0.80016198130268
O	-0.88924684804936	-4.22623357740878	-1.92892290184212
O	-6.79602871159083	1.07263321048780	-1.49034241697734
O	0.64177818372499	-6.13266697696473	-0.95925699305183
O	-8.41045524686676	-0.62328820279883	0.57569929622233
O	-7.32041951462121	1.75501727831422	1.03563261072425
O	-1.91374292437015	-6.63656539250290	-1.35819787140228
O	-6.49915712121098	-0.23811439876723	-3.72013960562258
O	2.66353728956188	-5.43934904960663	0.66292569260431
O	-8.98312817861993	-2.59003087072698	-1.06096802805065
O	-4.60527210085717	-0.40507192551597	-1.85321351827823
O	-6.89811281652334	-2.75783613936758	0.56076115273575
O	-8.00630620640660	-2.48180619343915	-3.54168834985303
O	-5.34286998969200	-2.58868781896571	-3.69173284376410
O	-7.24668078699072	-4.38019133679448	-1.85552390598103
O	-5.05981045369393	-5.18140055979754	-3.17270895600669
O	-2.88877998185111	-3.68830800885513	-3.62426063266512
O	-5.62626608239621	-6.13678556319719	-0.74195566316662
O	-2.83209451901918	-2.58498569519823	-1.22147753048454
O	-4.76813167928468	-4.23822843724832	0.94157453654950
O	-4.41704852953387	-1.62232185926261	0.86205507706711
Si	-6.50652644305001	-3.01254923970921	4.13887118248359
Si	0.59655311174959	-5.83832485365300	-4.71174625993133
Si	-8.51374958008060	-0.63376167815703	4.33503188279323
Si	-1.23826356079159	-6.06296798816228	-7.25116926809360
Si	-6.67935836601699	2.25753189544587	-0.38298353955567

Si	-2.44033457542068	-7.47812173195126	-0.05243140723348
Si	-0.62933881502830	-5.81763614141554	-1.92408833884331
Si	-8.43092752562496	0.67589794275239	1.54241239617578
Si	-5.68123171929270	0.52973919710098	-2.54555352615058
Si	2.25743611437070	-6.01623740702185	-0.81258759139190
Si	-8.43998016017188	-2.23298495216372	0.43166413871827
Si	-6.68383308851238	-1.77530328669419	-4.17806892602681
Si	-8.49866538368381	-3.46355480548599	-2.34873611692222
Si	-4.45139318887590	-3.91711418048258	-3.99260871809885
Si	-6.24922725367870	-5.63141973925319	-2.15687868569165
Si	-1.93630915735190	-3.08865180194081	-2.43763524246787
Si	-4.94787941922094	-5.84100513622622	0.70907947058485
Al	-3.65134180561029	-1.18699052247278	-0.69170129097879
Si	-5.44820112599061	-2.81410302302569	1.28970540664068
Si	-2.11331417180406	-1.57004814683798	-6.93392276756235
H	11.67374717030420	-5.53703622109374	-0.03807377114726
H	9.19348299925762	-8.10585819838494	2.36346311870165
H	-3.88154976766942	1.39981077973606	-8.25725791513313
H	-5.54661909120599	-0.07985125359363	-7.37196296249479
H	-6.93206218048882	-1.73335117576822	-5.60666694603599
H	-9.61083501112981	-4.23371614317095	-2.91527905986429
H	-4.80806180408779	-4.38616415997394	-7.98466815025278
H	-1.25558088501839	-7.52827132705132	-7.35483825877276
H	-8.46603525850511	2.53218809733480	6.77163522969983
H	-4.20950416906329	6.50518815388364	2.49497318080084
H	-1.81143180241417	9.00591878273910	0.20164720963228
H	0.67612813483913	11.32783143601786	-2.29765516993096
H	-4.95337016196127	5.93157222834064	-3.34598485865361
H	-7.43212829466562	3.36467275185665	-0.94325520337133
H	-9.81433487017745	1.14100184778204	1.45030698590927
H	-3.26082278257136	2.50672885254105	9.43326906883409
H	-0.74504076630618	4.85495113839148	6.90563295386748
H	1.17176503576068	6.84606717947660	4.60638284382286
H	0.01383692571069	-0.85268916961134	9.75320590630557
H	2.65630396626605	1.61378676002795	7.09828785515488
H	4.53076924133414	3.47379808875744	4.86924421485797
H	1.53720793830655	-6.93798314430161	-4.97365722869566
H	4.63541509314463	9.21535601009308	-0.64625184786597
H	-8.67488827421826	0.53574588762194	8.08886525978842
H	-5.67875093652799	-0.35844188804562	8.92691791692340
H	-5.40516102671903	-3.45826620844022	7.74597006902192
H	-2.86007317651404	-3.03986695486766	9.10083200728667
H	-1.22701007822453	-4.11162702295746	7.70400896208336
H	2.57413279977982	5.42807916254603	-6.16363178146072
H	0.22482887571030	8.10275880746068	-5.85603218148424
H	2.79507592723046	11.25689311418747	-3.42192602183588
H	6.06067082988975	7.26917505187178	-3.76691093340824
H	10.01065009023267	4.23779398275817	-2.76113079668267
H	9.87926491395525	0.42371378030166	-3.59460924600659
H	11.16639481511047	0.78744109032979	-1.60472512946845
H	9.89847193000219	3.90341219496067	-0.38688410477724
H	-1.32399926497046	-1.19594420088126	-8.10330121410186
H	-0.61662903677683	-5.49814381280122	-8.46011588222395
H	2.84818309376226	-3.04332195287764	-5.95038394901103
H	-7.31517907270164	-4.15812794118835	3.75060698354032
H	-9.78250009834674	-1.30915796091348	4.07997005466961
H	-9.40319698629290	-2.75383185541268	1.45013712002457
H	-5.76898888041722	-6.41021301560240	1.77986109001170

H	-3.08084485054835	-8.75082996657773	-0.46797098715833
H	-7.02180587286307	-6.69287778540114	-2.78419816048314
H	2.78359013586781	-7.36886304239332	-0.99247291740300
H	4.89691594957051	-9.57737989867931	2.49409421745353
H	5.82084507202245	-9.04713692487355	0.34374504936721
H	-1.34437682100792	-7.77605485181618	0.87467383683830
H	10.66950905597119	-2.02029698920316	2.56380696486347
H	8.82314097409321	-3.95574094181524	4.78387681801789
H	11.59080023002678	-3.97453414128324	-1.85887994785257
H	5.33815607909728	-0.46451122655344	4.51995497585212
H	7.15219513762957	1.34004709306831	2.37234694571829
H	7.32251188131312	6.57841093373350	-0.51330718634802
H	5.00684298994581	-4.86484781058979	-3.24385789036702
H	7.90214825598456	-7.00114309030894	-1.50659186159254
H	2.44258304938981	-6.49974715380114	2.91143194154102
Ga	-2.97493585273544	-0.70846456666578	2.05283292083832
H	-2.99651887445933	-2.43205242289284	3.04454752729809
H	-3.70296886348320	0.40443797121731	2.85521321300911
C	-0.92609874901665	-3.65749349068704	2.89414213452919
H	-1.25104530714374	-4.54821568480040	2.36061826201583
H	-2.40704192037393	-3.01650113512024	3.13368531176586
H	-0.77146840539589	-3.76586686677113	3.96940429740075
C	-0.34783876427076	-2.61853288448342	2.20117875590239
H	-0.19831392238080	-2.71781817114747	1.12478891639440
H	0.31360470296581	-1.91283485627624	2.70338750860614
H	-1.52624126978779	-1.51588580094763	2.00551283628495

TS2-GaH 2

Electronic energy (r^2SCAN-3c): -30618.495085122886 E_h
Electronic energy (ωB97X-D4rev/def2-mTZVPP): -30626.685537021465 E_h
G_{corr} (673.15 K, 1 atm): 0.42939 E_h
Number of atoms: 250

Cartesian coordinates (Å)

O	1.48879762102705	2.89786587626645	1.03152764307376
O	-1.94287272260561	6.77808560990800	1.43271939788495
O	5.26668998123461	2.07926666405626	0.80286331440954
O	3.60113174534038	3.87650541010013	-0.16172988031362
O	6.08660540105304	4.57801267951845	0.41813189933497
O	1.43598637983835	3.28151490773900	-1.61332590236334
O	1.36935085198692	5.32709953673626	0.02592966307356
O	-1.70115579687618	6.88519006331212	-1.18641382476266
O	0.32753264154436	7.70369905370815	0.41436870231824
O	2.93470539826033	7.47763782140018	-0.04664298390124
Si	4.83960090638085	3.63923408861282	0.86541448407596
Si	1.98117767014506	3.84202519328540	-0.19177840991906
Si	-1.29059947006929	7.63027470542608	0.20589083964715
Si	1.65084228843743	6.79126325163948	0.66031973545303
O	8.95262081912214	-3.83486670727803	2.22552815358988
O	2.00260998352370	6.64507242120617	2.24318591703945
O	7.14487396257006	-2.27991057640017	-0.92324196754785
O	2.50171087869850	4.74672774114196	4.08845828360262
O	6.74854195917148	-4.90437412706850	-0.77306278304746
O	0.04286250265704	5.12122207510466	3.14802887180638
O	5.85368577137464	-3.26836824426799	1.13568776197814
O	4.43285496454239	4.07182507158664	2.38358826425391

O	9.40136348594150	-5.01152473317994	-1.12762441966047
O	5.15311451016958	-0.24619689058119	2.01941351429292
O	7.73292486032429	4.37698451623107	-1.68572292930997
O	10.70345278156018	-3.18497531968179	0.34588341132123
O	5.42975847937036	5.63157676464407	-1.91281455066844
O	4.09932669062185	5.67979833017689	-4.18317346227882
O	8.76122257155061	-1.46109307415367	1.04459875928706
O	3.92230208653511	7.73629911194708	-2.52962243162941
O	1.17493683467888	9.52226623818481	-4.04918043840626
O	8.98107323255029	2.07189539968623	-1.88673068368272
O	6.94678780125963	0.18982484860725	0.10359055457753
O	2.27234501398052	9.55305953266783	-1.56433149723309
O	8.99974178123128	-0.54732714602642	-1.44220983373401
Si	1.43262915506595	5.86074475082253	3.55391623199552
Si	6.11516562739579	-3.44022651571781	-0.45059074888431
Si	3.62869106172604	3.62389171544925	3.72737572767846
Si	8.07876184842683	-5.83613917782943	-0.65690740939580
Si	10.87648283555053	-4.45884812941498	-0.67194332910133
Si	6.66914806952265	5.32683602231978	-0.90807388522708
Si	9.79483879947870	-2.60831196579198	1.56617813934488
Si	4.92522199623201	6.59613828584148	-3.11976792209236
Si	9.20471586079191	3.67345180033180	-1.66721569367865
Si	6.17079200230751	0.85465089488080	1.37293176768499
Si	1.72991719084933	10.45270356391817	-2.82568976548362
Si	9.80036171718420	0.68319415255318	-2.15352994775697
Si	7.95466448362609	-1.01611871012374	-0.30245849859676
Si	3.46885641282361	8.51749123626987	-1.17811812122645
O	2.97635246597633	-0.47284013519634	3.61614636256621
O	0.95649065273153	0.76634985193373	2.48124437734260
O	1.23957535090797	0.87966532999103	5.08378534901570
O	2.96843286174064	2.15950207324049	3.48937041760646
O	-0.40735648517097	1.18644368740216	0.28814418736606
O	-0.81972181135920	2.73437855504064	2.36310104279286
O	-3.73990926283120	4.98827873620728	0.56155831954809
O	-2.53677463053680	4.66189578850619	2.92863556647200
O	-1.18273251482866	3.43343284782946	4.84052898682506
Si	2.04455121019306	0.84079350736141	3.67988309321687
Si	0.30559580071649	1.91051603651519	1.53679396795791
Si	-3.13224331604853	5.74828764496416	1.86335453593044
Si	-1.11148530383209	3.99954823013858	3.32920494022070
O	8.28948354474899	-6.04361095106329	3.49662320533669
O	-3.20539011437402	-3.02977778174337	6.58649015065580
O	6.68179399724206	-7.84753923989071	2.44514577030181
O	-1.30215194701508	-1.56657786193561	7.69220516651337
O	8.25522253180706	-6.34192756795646	0.87228697281255
O	-5.39610002950607	-3.45285888710881	5.21537733928557
O	6.65209256913901	-3.92585148944713	3.57137843503412
O	-5.20612318113910	-1.22522975848882	6.62549643689572
O	-8.64229644726789	0.29310596527654	5.60877161532450
O	4.47657157218527	-2.51272027819057	3.25142529976864
O	-6.43724693461365	1.08408627998425	6.97507672105083
O	4.49075891431693	-5.06508912901636	2.60617506218821
O	-3.87227072356374	0.83587470183753	7.57996906616661
O	-2.25593715387179	2.84681293921777	7.14619722012971
O	0.39604671368412	2.65753295729796	6.86317444925262
O	4.25500669895377	-7.39985294878081	1.37561987810628
O	-1.50485360339955	0.86812648318857	8.78922734559737
O	0.72470845262229	0.08536877421766	7.53422805321419

Si	8.12646087792412	-7.12394997630031	2.28700927026275
Si	-2.12561034485658	-2.96699240005476	7.82068401257397
Si	8.20051596642834	-4.41721567792948	3.55207287924713
Si	-4.81761141705237	-2.80057082078425	6.58940910868180
Si	4.52752376477100	-0.89521924864272	3.37759502412456
Si	-8.07466511781157	1.13057644149579	6.88825030960918
Si	5.36081938242730	-3.70531189456019	2.61372388284751
Si	-5.30605285939214	0.08413917430003	7.58124658780074
Si	-0.93924065924572	3.48868362544710	6.44192187771416
Si	3.44319132625720	-6.09963972870627	1.92482666191434
Si	-2.74769756108077	1.78372334548500	8.27081987366356
Si	1.29708180743150	1.31995275870549	6.64742917319013
Si	5.41701687700344	-8.50887989764811	1.64893100920797
Si	-0.51103156265626	-0.38242282172049	8.47715731395334
O	1.81917802192965	4.27205306567532	-4.04998793487604
O	-0.06931584697436	5.26537786757785	-2.47396305429748
O	-0.61047840294145	7.68666913435565	-3.45794343584509
Si	0.70271073478334	3.91405976148091	-2.92582873818928
Si	-1.20046848700439	6.40769542426989	-2.65370221656187
O	1.24841190042637	-1.72455765794988	-4.51209213749776
O	-3.97299045838492	3.96264111093113	-4.56596132240814
O	3.34044522483122	-2.95565798856049	-3.48233566371600
O	-2.45048729178527	5.83200391542432	-3.50312733502920
O	-3.82255437314577	4.16480031983379	-1.92821120047860
O	1.33822674144695	-4.40846758988099	-4.41784612594637
O	-4.66131557284712	1.78928781117002	-5.93685221526170
O	4.72009419133239	-3.30690551025086	-1.25881652772522
O	-2.57637055632192	1.71535189494477	-4.36440990302689
O	-3.16861726017281	-0.36610446494220	-6.53408094102994
O	-1.11704094649616	-1.81644018896309	-5.66242919000488
O	-0.34674464425712	0.32890967000945	-4.25245199562752
O	-0.35685430995714	2.85859477552398	-3.53230521453061
O	1.78414373836078	6.92796965175286	-4.27360156343938
Si	-3.83843920966906	5.00558084142033	-3.32129617726465
Si	2.19238883522576	-3.03907823119117	-4.64165652465623
Si	-4.06226825186512	2.33832661784283	-4.54123711689091
Si	4.00754003613344	-4.07485688004468	-2.50964453614652
Si	-4.32302992278441	0.66691729669717	-7.07317880463408
Si	-0.30750149850213	-1.29389919978505	-4.35675506390771
Si	-1.21810433921887	1.48388524864190	-3.52554572690583
Si	2.55986819585692	5.56779624031915	-4.71148836030822
Si	0.62799052847850	8.05234611670669	-4.45458458971200
O	-2.79623836400478	-5.59061792698381	-7.11505913882043
O	-4.51244470356904	-4.32884495949884	-5.53351159274592
O	-2.92143955595063	-2.92600138275155	-7.23428432918448
O	-2.83258526973834	0.13845153810504	0.14233144502919
O	-2.46962778762883	2.77274344325786	-0.16690461282797
O	-1.60252736220426	1.02318951418941	-2.01960032181896
O	-5.11965760307806	2.80802341065600	-0.00070784332567
Si	-3.76639182344603	-4.29298952708735	-6.97769745410977
Si	-1.84429894288611	1.29660034169553	-0.45076224690105
Si	-3.80444536498475	3.67883104759538	-0.38832314790958
O	-5.06847457999361	1.94510596330513	-3.33265884410779
O	2.81673066569327	-5.05211639078125	-1.98315048168607
O	-5.46894128487035	-2.52678988402164	2.77588486295919
O	-7.33913381250178	-1.76818513128153	4.55903703428015
O	-0.25753486732791	-6.28154098238049	-3.39791649644636
O	-8.03505864266424	0.26407105637739	3.03044311549155

O	-0.38891653047718	-5.57485737912611	-5.96368346041062
O	-0.94600102040637	-1.92296569432609	-3.01525090960538
O	-3.48834996032474	-6.53211799505703	0.74500006338686
O	-0.98485586437029	-4.25246496889040	-1.83395633091955
O	-6.57173862571719	1.03531492533013	-1.38791957308600
O	0.62462889243790	-6.07504541764022	-0.87094932500234
O	-8.28214762435991	-0.66138573137928	0.53559390358787
O	-7.35579025219776	1.78401850190508	1.01218290192608
O	-1.87523988408514	-6.73913651023834	-1.40063887715733
O	-6.54338217422220	-0.21772395904224	-3.69482192759963
O	2.70719218173464	-5.41963657797045	0.64068717997424
O	-9.03258411686900	-2.63717481569986	-1.04227850820503
O	-4.39124543527206	-0.31937589080272	-2.14600943452870
O	-7.12121675526417	-3.08587906020328	0.78426991903623
O	-8.03045558448010	-2.40733653621261	-3.49620575457525
O	-5.37634502879168	-2.60740550219471	-3.73629431897381
O	-7.25591918072254	-4.37410328500814	-1.86606317036263
O	-5.00299026151818	-5.15034392884581	-3.07165670274155
O	-2.89265864538033	-3.62077628826017	-3.59044455737618
O	-5.69190153486110	-6.20108559273704	-0.73229891160058
O	-2.77850745605817	-2.46788288759222	-1.17203841232814
O	-4.76562072027911	-4.24827337171624	0.86800200859049
O	-4.91849663061864	-1.58916124240400	0.43287398587362
Si	-6.43669106092642	-3.01154236299301	4.05245697075447
Si	0.60012805046185	-5.82792282732278	-4.70486398485453
Si	-8.49190621045267	-0.64202407301440	4.29952634828294
Si	-1.24354824234625	-6.06914329729380	-7.25957849198328
Si	-6.62827717211704	2.28975590003935	-0.36244267484750
Si	-2.43377958327723	-7.49265306264528	-0.06078866552371
Si	-0.62572006724981	-5.82078662630248	-1.88390862758670
Si	-8.41992606822455	0.65291363595720	1.49228417721145
Si	-5.61183865989166	0.57980556982635	-2.62582045708188
Si	2.24800888439424	-6.01925639332631	-0.81150108075898
Si	-8.48990435522738	-2.26815177316381	0.43978002889141
Si	-6.69949222449446	-1.75660475050398	-4.17542692861371
Si	-8.50766508880082	-3.45224565483077	-2.35495312107989
Si	-4.43765180034519	-3.91400615996765	-3.96928211783858
Si	-6.25446967543845	-5.62683499722210	-2.14790306362717
Si	-1.93277751085652	-3.05480803141082	-2.38603864903498
Si	-4.96155803023668	-5.86008212375447	0.68295515526992
Al	-3.74833102689214	-1.11168646014058	-0.76753748861349
Si	-5.55734521856722	-2.86183947217612	1.12349422155370
Si	-2.11444005630354	-1.55160137786826	-6.91308141338678
H	11.67374700396706	-5.53703599156417	-0.03807400235143
H	9.19348302046459	-8.10585800267391	2.36346301020452
H	-3.88155000533477	1.39981099896019	-8.25725801360493
H	-5.54661899494258	-0.07985098954052	-7.37196299753247
H	-6.93206199377262	-1.73335099967228	-5.60666697563577
H	-9.61083497549883	-4.23371598556926	-2.91527897409408
H	-4.80806200377572	-4.38616399967296	-7.98466800885419
H	-1.25558099928340	-7.52827101910738	-7.35483800937573
H	-8.46603498923006	2.53218798989431	6.77163498064403
H	-4.20950401568534	6.50518801931775	2.49497300894582
H	-1.81143199495992	9.00591899414697	0.20164700271852
H	0.67612804254366	11.32783197301225	-2.29765501705591
H	-4.95337000590943	5.93157196177033	-3.34598500826682
H	-7.43212796335306	3.36467295092372	-0.94325497412467
H	-9.81433504128089	1.14100200784616	1.45030700574379

H	-3.26082300415656	2.50672899118916	9.43326898593559
H	-0.74504100502784	4.85495100486240	6.90563299699629
H	1.17176499698137	6.84606700052581	4.60638299767046
H	0.01383699681238	-0.85268901193804	9.75320599710755
H	2.65630401035965	1.61378699860502	7.09828799941775
H	4.53076900871627	3.47379799443082	4.86924400426471
H	1.53720798561142	-6.93798297987364	-4.97365699069052
H	4.63541500075602	9.21535600278554	-0.64625199668273
H	-8.67488801556765	0.53574599062608	8.08886503023087
H	-5.67875100234053	-0.35844200051693	8.92691799358076
H	-5.40516101707633	-3.45826601351185	7.74596998797715
H	-2.86007300704085	-3.03986699950147	9.10083200242124
H	-1.22701000371437	-4.11162699876683	7.70400899155021
H	2.57413300487605	5.42807900566919	-6.16363199816131
H	0.22482900503685	8.10275900070362	-5.85603199447787
H	2.79507594117351	11.25689295678126	-3.42192596750501
H	6.06067099506771	7.26917499314455	-3.76691100495820
H	10.01064999907639	4.23779399872879	-2.76113100012476
H	9.87926499721731	0.42371400047472	-3.59460900135106
H	11.16639500310567	0.78744100341931	-1.60472499947488
H	9.89847199372179	3.90341200025984	-0.38688400543099
H	-1.32399900514301	-1.19594399625399	-8.10330099081816
H	-0.61662899846348	-5.49814399436578	-8.46011599881447
H	2.84818298749975	-3.04332199907939	-5.95038397625305
H	-7.31517891130024	-4.15812789934618	3.75060704182389
H	-9.78250001285190	-1.30915799547514	4.07997000100137
H	-9.40319695732180	-2.75383198892363	1.45013694858435
H	-5.76898898189542	-6.41021299086897	1.77986097041844
H	-3.08084499112365	-8.75083000025263	-0.46797099750352
H	-7.02180602142450	-6.69287802512608	-2.78419800913753
H	2.78359000516824	-7.36886298866347	-0.99247300415951
H	4.89691600700500	-9.57737998962381	2.49409399694573
H	5.82084502614916	-9.04713701588292	0.34374497921177
H	-1.34437701415575	-7.77605499958597	0.87467400039959
H	10.66950899653436	-2.02029699515977	2.56380699225358
H	8.82314099665566	-3.95574100354121	4.78387698413749
H	11.59079999423780	-3.97453400446005	-1.85887999348769
H	5.33815599946200	-0.46451100068151	4.51995499280085
H	7.15219500839433	1.34004700834145	2.37234700241667
H	7.32251200644523	6.57841101406695	-0.51330699619800
H	5.00684301034137	-4.86484800118699	-3.24385800759329
H	7.90214800104287	-7.00114299567666	-1.50659200617886
H	2.44258288680766	-6.49974697672399	2.91143202418138
Ga	-3.61355113156743	-1.31799875443328	3.13014577665004
H	-4.08388369073065	-0.72414698604674	4.47284079571939
H	-3.71630258253599	0.18113517878050	2.22302867827207
C	-2.12352146391291	-3.84467737194854	3.19908001782437
H	-3.05644428344844	-4.41235598236125	3.12289324199732
H	-1.91089024089505	-3.70260021549555	4.26294534111395
H	-1.32914254841878	-4.46997264169106	2.77532365435692
C	-2.21344148362285	-2.51076884682995	2.44774724893479
H	-2.35909517161194	-2.68248908278672	1.37581427128159
H	-1.27304664753494	-1.94882878354612	2.53834246274037
H	-3.30637300666743	0.15369017532929	1.39317250935935

TS3-GaH 2

Electronic energy (r^{2}SCAN-3c): -30617.291221346921 E_h
Electronic energy (ωB97X-D4rev/def2-mTZVPP): -30625.465958238801 E_h
G_{corr} (673.15 K, 1 atm): 0.41278 E_h
Number of atoms: 248

Cartesian coordinates (Å)

O	1.53531530937986	2.89184897489491	1.01515494383425
O	-1.94664844543605	6.77513599189931	1.43347264931378
O	5.26527461862942	2.08156960956917	0.80553503195917
O	3.62668699666163	3.90206948924917	-0.16987741495823
O	6.10905583334929	4.57443628859488	0.42086458916338
O	1.46443032281718	3.29409631153439	-1.62098119377036
O	1.37522904050323	5.32813389656351	0.03287320328615
O	-1.69965609453389	6.88665705524504	-1.18234076393052
O	0.32712237860527	7.70250151941491	0.42185799389112
O	2.93547268223231	7.47857733788781	-0.05163589502606
Si	4.85469258107033	3.64618777063860	0.86543340872339
Si	2.00843741814995	3.85530496544259	-0.20089182123081
Si	-1.29120739796919	7.63014038290883	0.21074550257331
Si	1.65475963396325	6.79576630804701	0.66209961450966
O	8.95048615344597	-3.83310204606366	2.22525549710855
O	2.01644719680903	6.65382389721469	2.24330353002233
O	7.14475901804554	-2.27942964899221	-0.92235649283579
O	2.49897681365237	4.74410800053770	4.07889495345441
O	6.74867098594589	-4.90437931870449	-0.77157587060176
O	0.03680042331241	5.16095932080068	3.15585111386225
O	5.85206146780567	-3.26710011082642	1.13568724506685
O	4.44304908259316	4.08094765882482	2.38220045232235
O	9.40114355142418	-5.01101283309599	-1.12827627395846
O	5.15177106184119	-0.24465217499202	2.01878580208957
O	7.73576557917219	4.37985451193031	-1.69449174821672
O	10.70205496662665	-3.18467528974082	0.34547183695317
O	5.43112177395068	5.63405758640450	-1.90841346769668
O	4.08736685689999	5.66606114535641	-4.16771832195880
O	8.76105529210828	-1.45926466386590	1.04514710242892
O	3.92376474132825	7.73821190088228	-2.53291923236317
O	1.17533969040122	9.52114563975827	-4.04941003738443
O	8.98076037297223	2.07169678689233	-1.88600548442094
O	6.94553089209302	0.19044339810746	0.10308734936649
O	2.27243948135972	9.55383332239895	-1.56548239916146
O	8.99908767831350	-0.54679539469556	-1.44232430658619
Si	1.44069569961991	5.87267761480614	3.55253759751451
Si	6.11539258386780	-3.44032122312756	-0.45049981315840
Si	3.63827645091958	3.63291664719208	3.72464616093975
Si	8.07932492126559	-5.83634455299886	-0.65689924446589
Si	10.87641427159516	-4.45887385328021	-0.67213043875151
Si	6.67545285582900	5.32742201267373	-0.91059439629565
Si	9.79444849586445	-2.60763503850102	1.56609703674006
Si	4.92331123078621	6.59290398650551	-3.11917768987184
Si	9.20611488511088	3.67318984377376	-1.66824879590053
Si	6.17094489240031	0.85581230280299	1.37237404554449
Si	1.72947450632308	10.45328916207545	-2.82674508023396
Si	9.80054340260932	0.68326738104324	-2.15365976633979
Si	7.95475109698033	-1.01558812763131	-0.30231586388116
Si	3.47007772851761	8.51940139500038	-1.18164280789392
O	2.97504617116991	-0.47346785641235	3.61275569483728

O	0.97645968798428	0.79447890846311	2.48133557110418
O	1.24409388443891	0.88161657324243	5.08262282478745
O	2.99230279883053	2.16079815400833	3.49362265818465
O	-0.38119425693911	1.19223893497597	0.28966303959818
O	-0.78296634651716	2.79917822220027	2.32333129763454
O	-3.72281168784593	4.95888365705456	0.57496158291180
O	-2.53585741566074	4.67123419169170	2.95104145750352
O	-1.15097155496994	3.42340279560618	4.83133457274765
Si	2.06110718094583	0.85255560996339	3.68565112475471
Si	0.33221693370698	1.93916918539656	1.53033350151432
Si	-3.13187261457483	5.74309683478095	1.87047606358639
Si	-1.09980243862057	4.02308163173103	3.33211252029818
O	8.28816499230749	-6.04273037920015	3.49598903706922
O	-3.20018174862974	-3.06164592524195	6.57653190075002
O	6.68149445533516	-7.84649317164352	2.44434427376973
O	-1.30986138632482	-1.56922137730225	7.68591521926260
O	8.25709050946744	-6.34180100073622	0.87212954673206
O	-5.40598592473582	-3.46182410366497	5.21414116111272
O	6.65136920425976	-3.92395651025860	3.57197000199178
O	-5.13191053507444	-1.21684527036080	6.59262861511262
O	-8.67246909429069	0.32666218316571	5.58884998913560
O	4.47432956653839	-2.51293578897967	3.24979781128082
O	-6.44644009043980	1.05342584925432	6.95629440212715
O	4.48975528728829	-5.06493647718393	2.60649335111254
O	-3.88543700421343	0.85813624091976	7.57923396051082
O	-2.25132285431556	2.84626038173341	7.13834566878713
O	0.40126712990798	2.65815975026950	6.86735817625903
O	4.25545810231408	-7.39969181032621	1.37485556627498
O	-1.50856575981051	0.86690220011268	8.78201906849179
O	0.71732703750806	0.07919645205203	7.52603516738568
Si	8.12654229382474	-7.12395142265217	2.28672762085635
Si	-2.13244523196172	-2.97513505335758	7.81558671031158
Si	8.20003748120396	-4.41665676147232	3.55215388801224
Si	-4.81446904933079	-2.81318773126660	6.57420680380709
Si	4.52915991317590	-0.89619435018154	3.37648459250770
Si	-8.08418531144580	1.12714029701036	6.87954343170036
Si	5.36075274636585	-3.70638425598860	2.61336255378648
Si	-5.30303531771614	0.07181418130020	7.56827974789697
Si	-0.93358038635908	3.48775636601178	6.43913396409138
Si	3.44292631007770	-6.10070667374322	1.92433722901454
Si	-2.75093493615809	1.78799340597896	8.26760403024290
Si	1.29634070725122	1.31819633007100	6.65131482137194
Si	5.41703430470612	-8.50935601502963	1.64873542746306
Si	-0.51863632258333	-0.38753553357671	8.47473026141425
O	1.78896876141003	4.26582963483470	-4.07034234397686
O	-0.07187368729434	5.26280210505469	-2.46543320060636
O	-0.60898483090380	7.68521576343909	-3.45535627723133
Si	0.69678667945109	3.91112086292557	-2.92072159216522
Si	-1.20015795444038	6.40855687227677	-2.64900664035366
O	1.25134983225625	-1.72270163832132	-4.51415940368867
O	-3.96072140284221	3.96173903258099	-4.55885264113283
O	3.34070105179541	-2.95496199406822	-3.48172247072666
O	-2.45185147901181	5.83485524813918	-3.49589273979653
O	-3.80584354542881	4.15798639457135	-1.91763310336157
O	1.33764970508960	-4.40737548874976	-4.41664617509351
O	-4.65146485231516	1.79061087185562	-5.93286483304441
O	4.72048877849221	-3.30608350657687	-1.25897909275585
O	-2.59028950705125	1.69959127024411	-4.32586312377915

O	-3.16664032418824	-0.36492658711152	-6.53324399910221
O	-1.11577517502262	-1.81452274575194	-5.66312047300078
O	-0.35184486421372	0.32258753148316	-4.23523231076818
O	-0.37548860512472	2.84035277054672	-3.47801937852295
O	1.78216966961401	6.92519994887127	-4.27170679652871
Si	-3.83623751628168	5.00303628117577	-3.31162998857929
Si	2.19331026329564	-3.03966383401485	-4.64187898713415
Si	-4.06678433818801	2.33871491427256	-4.53134586388575
Si	4.00782620543975	-4.07491156222654	-2.50965679590010
Si	-4.32211665739800	0.66741411314141	-7.07173638826232
Si	-0.30602146738193	-1.29990236968312	-4.35589874646626
Si	-1.23052927063299	1.46172718365878	-3.49434380704252
Si	2.55149176492337	5.56202036103508	-4.71110285583032
Si	0.62766644144484	8.05146569009749	-4.45413530901678
O	-2.79582417917907	-5.58911597207266	-7.11077870977385
O	-4.51774101490876	-4.32232982695808	-5.53573844356554
O	-2.92076707311876	-2.92581369511244	-7.23452594143616
O	-2.76787013368714	0.05640195028256	0.21634469193143
O	-2.50218545703546	2.71061428578261	-0.15128980483860
O	-1.61281797873923	0.96535300117990	-1.99225633895564
O	-5.13375108488355	2.79831767748804	0.00114042418936
Si	-3.76766237686545	-4.29223815436638	-6.97710032109384
Si	-1.83098945833750	1.25989015577358	-0.42715585578689
Si	-3.81252289271142	3.65620348735139	-0.38160885218600
O	-5.08969907962587	1.96444477608220	-3.33138378295276
O	2.81703546060479	-5.05096957726915	-1.98218271991789
O	-5.62017742492293	-2.51952545524134	2.77446617800107
O	-7.37365873010805	-1.78746186546519	4.65634048388516
O	-0.25653878797671	-6.28264865719483	-3.39932239107704
O	-8.02034246682684	0.21399035257058	3.02183877135333
O	-0.38649474066636	-5.57333252010881	-5.96519915199440
O	-0.93856758662763	-1.93884346829953	-3.01644171378919
O	-3.48762985898064	-6.50748711235267	0.73414310379984
O	-0.98850642948334	-4.26143816179651	-1.82917614753739
O	-6.59141047186812	1.03550147052050	-1.38970254555850
O	0.62590381548601	-6.07644317844802	-0.87139226190225
O	-8.31222950495323	-0.65870231294380	0.51109295941721
O	-7.36466253326545	1.77358242332986	1.02038088389377
O	-1.87204560696848	-6.75187860701864	-1.40364993701619
O	-6.51278904953448	-0.22035171029225	-3.69379755363685
O	2.70798772988342	-5.41864824265787	0.64036242717428
O	-9.07744948743901	-2.64077235817281	-1.05324176599042
O	-4.36452851406493	-0.25497000191073	-2.10795509027551
O	-7.10014795400237	-3.06259834721305	0.65814812076186
O	-8.03529710953932	-2.38514995825696	-3.49109236583751
O	-5.37195967052515	-2.60512757284542	-3.72816700856860
O	-7.26443642152578	-4.36504627962358	-1.89597684887620
O	-5.00625122397933	-5.15509773295610	-3.07619163798752
O	-2.89441167492082	-3.62610474342530	-3.59373179747754
O	-5.69561955884414	-6.16148048225360	-0.72080404454303
O	-2.77306294343383	-2.47012413194661	-1.17671698091646
O	-4.75974902024961	-4.21505469115253	0.90326852191335
O	-4.90089849429813	-1.56357586470059	0.46057354518447
Si	-6.46998824341177	-3.00828299229071	4.06603644054535
Si	0.60050031722836	-5.82796281543326	-4.70544700156985
Si	-8.49352060661064	-0.65647570004109	4.31435513651487
Si	-1.24387241061988	-6.06885354993601	-7.25929291101641
Si	-6.64738559513544	2.28186030051128	-0.35346674926668

Si	-2.44201539471419	-7.48788718485496	-0.05571625984242
Si	-0.62725530647306	-5.83066562175943	-1.88342770612242
Si	-8.42630429365773	0.63235255504729	1.49789368825469
Si	-5.61385492335644	0.59589091314489	-2.61561656824414
Si	2.24813297658206	-6.01996576711309	-0.81176253437599
Si	-8.49697609932210	-2.27130108116786	0.41921918634199
Si	-6.69747108048109	-1.75692928266803	-4.17245103857460
Si	-8.52103940724182	-3.44152208276459	-2.35906835214348
Si	-4.44277924951415	-3.91870578449680	-3.96847378118500
Si	-6.26147471271029	-5.61917798620454	-2.14484224082426
Si	-1.93224196271379	-3.06705097490077	-2.39155950566984
Si	-4.96218595180365	-5.82193467872087	0.69788301404580
Al	-3.76525491979362	-1.13569074615939	-0.76996152444038
Si	-5.57558825824733	-2.82730571042581	1.16979770337052
Si	-2.11464627757784	-1.55182931920819	-6.91383545970955
H	11.67374700296396	-5.53703600883703	-0.03807399092974
H	9.19348298992750	-8.10585799573766	2.36346298978418
H	-3.88155001709024	1.39981099448551	-8.25725798482032
H	-5.54661898900568	-0.07985099151493	-7.37196300738052
H	-6.93206199366283	-1.73335099146369	-5.60666697117412
H	-9.61083499557691	-4.23371600072838	-2.91527899357856
H	-4.80806202079147	-4.38616400128324	-7.98466802315713
H	-1.25558100160233	-7.52827100313682	-7.35483800157792
H	-8.46603499758511	2.53218798957717	6.77163499923869
H	-4.20950399319972	6.50518799186420	2.49497299453025
H	-1.81143200237290	9.00591900123207	0.20164700155677
H	0.67612798331517	11.32783100975926	-2.29765499831403
H	-4.95336995394869	5.93157195056814	-3.34598498541169
H	-7.43212804616177	3.36467305779531	-0.94325502247868
H	-9.81433496786530	1.14100198788065	1.45030699981094
H	-3.26082303616469	2.50672903864351	9.43326904907019
H	-0.74504100541681	4.85495099487898	6.90563298894110
H	1.17176499766781	6.84606700164162	4.60638300751199
H	0.01383702454744	-0.85268902949946	9.75320604147840
H	2.65630402740674	1.61378700151377	7.09828799999083
H	4.53076898459984	3.47379799763591	4.86924397842729
H	1.53720800560616	-6.93798300466483	-4.97365699690248
H	4.63541500296201	9.21535600339900	-0.64625199665281
H	-8.67488799092893	0.53574600077316	8.08886499565591
H	-5.67875098535897	-0.35844199814828	8.92691798521377
H	-5.40516100813468	-3.45826600855593	7.74596999994044
H	-2.86007299143392	-3.03986700375022	9.10083198464574
H	-1.22701001105502	-4.11162698446262	7.70400899845322
H	2.57413299516519	5.42807899528495	-6.16363201097478
H	0.22482899905484	8.10275900200809	-5.85603199766557
H	2.79507602366888	11.25689302056537	-3.42192601443500
H	6.06067099373600	7.26917499927015	-3.76691099376637
H	10.01064999810442	4.23779399869259	-2.76113100056124
H	9.87926500340162	0.42371400120780	-3.59460899910850
H	11.16639498954769	0.78744100117823	-1.60472500456360
H	9.89847200249498	3.90341200290431	-0.38688399757144
H	-1.32399899597581	-1.19594400047873	-8.10330099822424
H	-0.61662900011873	-5.49814399575456	-8.46011600275584
H	2.84818300816565	-3.04332200005517	-5.95038402445114
H	-7.31517900939419	-4.15812801088561	3.75060699663462
H	-9.78250000523803	-1.30915800610299	4.07997000222113
H	-9.40319699853634	-2.75383200716526	1.45013699388994
H	-5.76898899729550	-6.41021300049770	1.77986099960405

H	-3.08084500217628	-8.75083000384981	-0.46797099794235
H	-7.02180601083103	-6.69287801238472	-2.78419800246102
H	2.78359000171426	-7.36886300861110	-0.99247300444482
H	4.89691600876869	-9.57738000490593	2.49409397691794
H	5.82084499003002	-9.04713699846727	0.34374502960294
H	-1.34437700109370	-7.77605499769533	0.87467400430471
H	10.66950900065345	-2.02029700131932	2.56380700433704
H	8.82314101142889	-3.95574099852665	4.78387702427156
H	11.59080000681067	-3.97453399204274	-1.85888001883113
H	5.33815599770760	-0.46451100515115	4.51995499621668
H	7.15219500191070	1.34004700106076	2.37234699545985
H	7.32251199101174	6.57841099033648	-0.51330699859385
H	5.00684298067238	-4.86484798867618	-3.24385799074046
H	7.90214800295137	-7.00114299652239	-1.50659200004342
H	2.44258300691234	-6.49974699689452	2.91143199619770
Ga	-3.08711753362207	-0.27853224893580	2.11082541903372
H	-4.30016957119029	0.32343995715847	2.81519945733195
C	-1.26983706294861	-1.39539167097737	2.83018424134929
H	-1.04145945883511	-1.21500008236282	3.87861373967557
H	-1.64906822661881	0.28785204453469	2.72296149343554
H	-0.43695037227442	-1.24468106966233	2.14886962509369
C	-2.36746325593159	-2.20931589234335	2.45701924568072
H	-2.95958258530717	-2.67554197255843	3.23904576795642
H	-2.31510303445632	-2.73018655021746	1.50297618289781

TS4-GaH 2

Electronic energy (r^{2}SCAN-3c): -30617.245333109760 E_h
Electronic energy (ωB97X-D4rev/def2-mTZVPP): -30625.417854755538 E_h
G_corr (673.15 K, 1 atm): 0.40697 E_h
Number of atoms: 248

Cartesian coordinates (Å)

O	1.53723686649930	2.85860052077154	1.00385874885409
O	-1.94874846639346	6.77333610685401	1.42651815021505
O	5.26875484598508	2.07964761796287	0.80088606943350
O	3.62384313778010	3.89599923403824	-0.17042541249587
O	6.10338385252156	4.57624814756807	0.42264771250810
O	1.46262618949358	3.29166081401671	-1.62416396498862
O	1.37073655915196	5.31111814764595	0.04818353299981
O	-1.71006546947154	6.88987670769269	-1.18996765135218
O	0.32567315078608	7.69265234652345	0.40841456058769
O	2.93180013653337	7.46652754589425	-0.06070267513496
Si	4.85165753571460	3.64211113460380	0.86567931810887
Si	2.00546258888458	3.83977921815479	-0.19746298323944
Si	-1.29345051989288	7.62850233955515	0.20432403262592
Si	1.65228638503267	6.78597767638590	0.65966441602594
O	8.95288083964608	-3.83521035347336	2.22552223330548
O	2.01247834768118	6.65899700032341	2.24247403092743
O	7.14504383568998	-2.28009363488574	-0.92382941734521
O	2.49580986515114	4.74276807225716	4.07493240388295
O	6.74850360467792	-4.90466445279513	-0.77374391082017
O	0.03092925228949	5.16928261258999	3.15573611084613
O	5.85454815726151	-3.26872144727081	1.13561777884662
O	4.43803532420854	4.07038031637610	2.38311424417439
O	9.40131888332298	-5.01134852435571	-1.12747520409200
O	5.15341714494715	-0.24601456563162	2.01881829085027

O	7.73428305414437	4.37881996841160	-1.69129729249183
O	10.70366480430335	-3.18498885075912	0.34584335844113
O	5.43050118054708	5.63460064221943	-1.90826341826687
O	4.08897493512431	5.66844557854295	-4.16957516625256
O	8.76120694780632	-1.46170784668004	1.04447885877757
O	3.92601037955830	7.74132366028009	-2.53534836977638
O	1.17272669049397	9.52152666347292	-4.04730547549318
O	8.98083421257522	2.07167613629294	-1.88638196204271
O	6.94735723246577	0.18933380891025	0.10318427268460
O	2.27289176663182	9.55311022275023	-1.56425487978802
O	9.00015489249478	-0.54775974014747	-1.44249400344243
Si	1.43896694544014	5.87352625605050	3.55132207392263
Si	6.11588549609353	-3.44050002110248	-0.45054453422873
Si	3.63011751142011	3.62327083246148	3.72536757249901
Si	8.07897359532545	-5.83630842406760	-0.65687419538480
Si	10.87665165803506	-4.45888648075320	-0.67195327960168
Si	6.67328753055414	5.32706749967510	-0.90821844163641
Si	9.79512295869437	-2.60865497272626	1.56626317383743
Si	4.92423495200538	6.59372191856883	-3.11905979466255
Si	9.20525131620575	3.67328930126016	-1.66781013671525
Si	6.17151334231120	0.85434213392480	1.37244220032849
Si	1.72992916249193	10.45287610994224	-2.82524822740957
Si	9.80048547639013	0.68312797585396	-2.15353810657096
Si	7.95523784110629	-1.01661252815923	-0.30271192645212
Si	3.46953455434495	8.51637094014570	-1.18137584466761
O	2.97581088819347	-0.47487702365073	3.61315472514518
O	0.95913937943471	0.77243510765338	2.48147448319625
O	1.24116229498881	0.87507937117546	5.08480320758395
O	2.97340785843847	2.15673876170474	3.49351259233309
O	-0.40142832584650	1.18924214321855	0.28143470306485
O	-0.77185227318876	2.80362016850680	2.31670514316803
O	-3.75024798947290	4.97847540339856	0.56998589006631
O	-2.53737516538921	4.66270097050151	2.93366163749198
O	-1.15668126175801	3.43010475383261	4.82696768161809
Si	2.04602243658639	0.84005126148808	3.68176514896257
Si	0.32575384448070	1.91681528487801	1.52809817343409
Si	-3.13537435260880	5.74507595022993	1.86628070180962
Si	-1.09834045902778	4.02251128953726	3.32447478404908
O	8.29016496512117	-6.04402200771337	3.49679311204268
O	-3.20303611906530	-3.07840283349471	6.58962892241040
O	6.68148170963711	-7.84704067786276	2.44512613325741
O	-1.31396646951023	-1.56717111910537	7.68642546829944
O	8.25488387366462	-6.34173918354096	0.87240802950851
O	-5.41303174458657	-3.47171869028996	5.22775160233043
O	6.65241971971913	-3.92699815274287	3.57130789371938
O	-5.11983127917253	-1.21752878939166	6.59184696262483
O	-8.67479484616009	0.33318760292671	5.58314039628103
O	4.47774618669217	-2.51288942419037	3.25111490679874
O	-6.44764815369629	1.04612074421238	6.95122878998195
O	4.49052403749764	-5.06493976877852	2.60558814226701
O	-3.88718243413550	0.86298217132702	7.57862076957310
O	-2.25043218929158	2.84745107731277	7.13659311637336
O	0.40061853382749	2.65800918248876	6.86213113396558
O	4.25485152022340	-7.39999806253588	1.37522671635005
O	-1.51143721912906	0.86601237828837	8.78328380798528
O	0.71916087137056	0.08244372317860	7.53226616858949
Si	8.12648919389461	-7.12408267468301	2.28707633223468
Si	-2.12884469787540	-2.97502849666101	7.82215701655676

Si	8.20106769063907	-4.41772358253689	3.55217342753388
Si	-4.81395976426446	-2.81557312748470	6.58066204994564
Si	4.52777040331622	-0.89531801594741	3.37706666495023
Si	-8.08470488567552	1.12691722218718	6.87838700372011
Si	5.36154896623981	-3.70572334608949	2.61365750780729
Si	-5.30036658496772	0.07172654827673	7.56792913645932
Si	-0.93290943485344	3.48881154935073	6.43394643130368
Si	3.44331626474109	-6.09993886959024	1.92463837460584
Si	-2.74939338878375	1.79092882898975	8.26643426386610
Si	1.29667039841116	1.31705735622103	6.64844979396687
Si	5.41706278724796	-8.50903586436322	1.64892532036223
Si	-0.51811286225998	-0.38557208421419	8.47485128580126
O	1.79092986376752	4.26956217096987	-4.06998237603014
O	-0.06772179823678	5.26866518716325	-2.46135252468918
O	-0.61373333507255	7.68361782133859	-3.46143964124801
Si	0.69882953067638	3.91652356611938	-2.92045188476120
Si	-1.20180191018248	6.40757361663053	-2.65227172427213
O	1.25470336006454	-1.71950167762630	-4.51555479328268
O	-3.97303678768816	3.95819705640429	-4.56063986252131
O	3.34066069099671	-2.95597157845227	-3.48169121171979
O	-2.44789091445945	5.82221154460624	-3.50123627615772
O	-3.82375899529274	4.16193392704056	-1.92216038667090
O	1.33416276374512	-4.40509225201763	-4.41653733152383
O	-4.66199176478354	1.78635847659092	-5.93238539502290
O	4.72020232853511	-3.30698778652814	-1.25829848553160
O	-2.58056696109237	1.70658562118209	-4.35485880022777
O	-3.16871019920991	-0.36506844880046	-6.53383540172381
O	-1.11252680765014	-1.80426849546305	-5.66316125257991
O	-0.34528023678048	0.32853098512045	-4.22659092616686
O	-0.37674011343647	2.85219942101133	-3.48565824738457
O	1.78285862289751	6.92756693814395	-4.27177208138621
Si	-3.83914231912563	5.00276504993712	-3.31708091791362
Si	2.19385980179608	-3.03904894072199	-4.64160895302255
Si	-4.06469420766058	2.33470554635593	-4.53656081347743
Si	4.00809484785064	-4.07529163278088	-2.50881932506566
Si	-4.32380318547048	0.66700331888624	-7.07276569627340
Si	-0.30096394270516	-1.29318119816630	-4.35572079301244
Si	-1.23062182690593	1.47430097015233	-3.50422752583652
Si	2.55306855939371	5.56489543079421	-4.71155169509359
Si	0.62691992537869	8.05207608618616	-4.45454596729447
O	-2.79643746458103	-5.58938206401411	-7.10962453831832
O	-4.52108528705317	-4.32098865053275	-5.53697366876954
O	-2.91755205395934	-2.92641562319575	-7.22963780449866
O	-2.83434348714454	0.12475674636955	0.17491220692349
O	-2.48828126002452	2.75774664529245	-0.15296053105631
O	-1.64365572557418	0.99481582553321	-2.00487141106862
O	-5.12717082025960	2.79271728777545	-0.00105064720081
Si	-3.76740163067875	-4.29180363408608	-6.97691761129429
Si	-1.84400081316795	1.29639345445516	-0.43706964840134
Si	-3.81833674565178	3.67389066455797	-0.38328689390782
O	-5.06781280265639	1.94562387271802	-3.32358250024314
O	2.81634349514621	-5.05179528493331	-1.98321497716244
O	-5.61694450651815	-2.51561114354907	2.79590849204560
O	-7.37766503264798	-1.78949941751295	4.66713402454344
O	-0.25535830304399	-6.28800609281932	-3.40133841267814
O	-8.01587660573794	0.20188673073015	3.01537898541286
O	-0.38705437243982	-5.57325829552838	-5.96524637784496
O	-0.93575736469595	-1.93636523878346	-3.01936942274223

O	-3.48601492772851	-6.50668479027043	0.73540926246317
O	-0.98876722816002	-4.26470178549006	-1.83954503284925
O	-6.59255826667709	1.03623599209259	-1.39057041066975
O	0.62532935499259	-6.07722982726254	-0.87239005513183
O	-8.33242190728465	-0.64487746767018	0.49866951413027
O	-7.36013344047883	1.77385134376018	1.02078759804755
O	-1.87245527615712	-6.75183142337563	-1.40436030255944
O	-6.54179103978614	-0.21107149971875	-3.70648974856895
O	2.70677941519743	-5.41979292084196	0.64048523781532
O	-9.07690716738307	-2.65062370703758	-1.04479307594869
O	-4.41532998021283	-0.31807773607345	-2.12828885540354
O	-7.05588643055570	-2.99358842683606	0.63083893495182
O	-8.04171414179923	-2.38444047726682	-3.48650948917688
O	-5.38269180344091	-2.60362622750708	-3.73182131527936
O	-7.26553821258990	-4.36671277871700	-1.89225283129052
O	-5.00677460418652	-5.15005600299282	-3.07594869640788
O	-2.89942731135313	-3.61661591759691	-3.59740190191730
O	-5.69281573857617	-6.15768059514490	-0.72053576424475
O	-2.76283451784092	-2.46922951903182	-1.17956211081463
O	-4.75855384337299	-4.21405164267453	0.90860432872459
O	-4.78843651472694	-1.54869728478975	0.55166381371356
Si	-6.47231308327599	-3.01068277910163	4.08132313832446
Si	0.60117003476166	-5.82768972259449	-4.70630825224675
Si	-8.49400899469106	-0.65684142043943	4.31493871681876
Si	-1.24415944957257	-6.06886622442015	-7.25930661203450
Si	-6.64359079192395	2.28368334339824	-0.35428198004984
Si	-2.44099467711274	-7.48799833037634	-0.05676099072917
Si	-0.62560614325532	-5.83342612811759	-1.88655964324107
Si	-8.42755520053237	0.63769911413627	1.49731145876695
Si	-5.63020903888257	0.58552695978192	-2.62469338349901
Si	2.24846208977824	-6.01987898645340	-0.81167265574474
Si	-8.49033774514242	-2.25957706292649	0.41896210810516
Si	-6.70787741099070	-1.75460711012060	-4.17413225792117
Si	-8.52234780017827	-3.44390200734326	-2.35604206097393
Si	-4.44736650953076	-3.91295506116633	-3.97118960880044
Si	-6.26052944705325	-5.61833537527833	-2.14566985355873
Si	-1.92752051392444	-3.06686103708444	-2.39890842433547
Si	-4.96036288011833	-5.82178415979489	0.69870374240674
Al	-3.74978111682297	-1.13255456832155	-0.78834549522939
Si	-5.54314760770895	-2.81706281874555	1.19634502148170
Si	-2.11388090857472	-1.54974329299338	-6.91308951173056
H	11.67374699702643	-5.53703597610685	-0.03807401112871
H	9.19348301645010	-8.10585799886946	2.36346301173795
H	-3.88155000876224	1.39981100096613	-8.25725800032553
H	-5.54661899552567	-0.07985099306462	-7.37196300328683
H	-6.93206199615409	-1.73335099582839	-5.60666697012843
H	-9.61083499191274	-4.23371599191576	-2.91527898453730
H	-4.80806199817700	-4.38616399178304	-7.98466800545035
H	-1.25558101356750	-7.52827097626603	-7.35483799461042
H	-8.46603499538241	2.53218803953132	6.77163500448767
H	-4.20950401730688	6.50518801601299	2.49497301246520
H	-1.81143200431461	9.00591900712576	0.20164700281778
H	0.67612791782801	11.32783060943679	-2.29765489794259
H	-4.95337002459453	5.93157202116194	-3.34598500665219
H	-7.43212797417071	3.36467296567383	-0.94325498696037
H	-9.81433503859816	1.14100201698778	1.45030699959740
H	-3.26082300280968	2.50672900140285	9.43327000198400
H	-0.74504100243452	4.85495000035889	6.90563299732231

H	1.17176499886339	6.84606700194875	4.60638299946241
H	0.01383700256209	-0.85268900556777	9.75320600152032
H	2.65630400656086	1.61378699971665	7.09828799925854
H	4.53076900364462	3.47379800060641	4.86924399711491
H	1.53720800157647	-6.93798299802077	-4.97365699160735
H	4.63541500408179	9.21535600915818	-0.64625199714907
H	-8.67488798719781	0.53574601364187	8.08886496856476
H	-5.67875099944474	-0.35844199707288	8.92691800033467
H	-5.40516100698229	-3.45826600551534	7.74596999809606
H	-2.86007299881265	-3.03986699359807	9.10083199614305
H	-1.22700999684074	-4.11162699920521	7.70400899356491
H	2.57413299983466	5.42807900365095	-6.16363200619141
H	0.22482900188843	8.10275900842199	-5.85603199562510
H	2.79507604655028	11.25689303138659	-3.42192602971915
H	6.06067099549267	7.26917500556270	-3.76691099853318
H	10.01064999835349	4.23779400009210	-2.76113100557632
H	9.87926499580485	0.42371400059616	-3.59460900830240
H	11.16639500616525	0.78744100420218	-1.60472499560864
H	9.89847199791722	3.90341200051782	-0.38688399669644
H	-1.32399899543014	-1.19594398820320	-8.10330100994197
H	-0.61662898370186	-5.49814398612351	-8.46011601444716
H	2.84818299541005	-3.04332199748771	-5.95038399694903
H	-7.31517899021228	-4.15812798314550	3.75060700730075
H	-9.78249996885127	-1.30915796825860	4.07996999514264
H	-9.40319696375238	-2.75383198643504	1.45013695992140
H	-5.76898899362872	-6.41021299354177	1.77986099597266
H	-3.08084499804139	-8.75082999053652	-0.46797100499831
H	-7.02180600474957	-6.69287800627016	-2.78419800450434
H	2.78359002943122	-7.36886303802640	-0.99247302594236
H	4.89691600350257	-9.57737995663664	2.49409400774463
H	5.82084504600461	-9.04713703128151	0.34374493363283
H	-1.34437699668876	-7.77605499639724	0.87467401091495
H	10.66950900917743	-2.02029697749005	2.56380700010184
H	8.82314098851337	-3.95574099047620	4.78387697041884
H	11.59079999885577	-3.97453401164113	-1.85887999044226
H	5.33815600352509	-0.46451099703970	4.51995499068574
H	7.15219501355616	1.34004701512035	2.37234700558164
H	7.32251200543142	6.57841100917731	-0.51330699789538
H	5.00684300592095	-4.86484799481434	-3.24385800766155
H	7.90214799823966	-7.00114298944191	-1.50659200126049
H	2.44258286476886	-6.49974697957157	2.91143208801661
Ga	-3.31649988582108	-0.23183648481709	2.02530009691178
H	-4.51166556003617	0.38586397834458	2.74754742221242
C	-2.28340381697679	-2.02983411715245	2.35935195892476
H	-1.75767111934652	-0.73074646588016	2.54770343563048
H	-1.81881503903738	0.40099792162614	2.67756880981844
H	-2.06912379916095	-2.63035620429877	1.47347204974456
C	-2.18158596333038	-2.57126232886825	3.58025741517900
H	-2.33263455078805	-1.97475360482637	4.47952707338085
H	-1.93074427894316	-3.61775721641333	3.74737837521207

TS5-GaH 2

Electronic energy (r^2SCAN-3c): -30694.652358910393 E_h
Electronic energy (ωB97X-D4rev/def2-mTZVPP): -30702.909157630278 E_h
G_{corr} (673.15 K, 1 atm): 0.44257 E_h
Number of atoms: 252

Cartesian coordinates (Å)

O	1.58952345756679	2.85069426832259	0.99146694652031
O	-1.94939664278828	6.78067598372609	1.42937652191650
O	5.26710046272316	2.08064170191643	0.80383142977825
O	3.64926317620203	3.92050954654103	-0.17807473136496
O	6.12629061013138	4.57138923935383	0.42385573795312
O	1.48792449571968	3.30601116484904	-1.62914825865219
O	1.37741204590703	5.30945907305417	0.06570775925089
O	-1.70683355502690	6.89195807816130	-1.18637103439395
O	0.32870788560292	7.69320198398556	0.41352014958117
O	2.93484766585378	7.46349635732634	-0.07159500400420
Si	4.86735549870326	3.64805123462573	0.86434869060317
Si	2.03248562271988	3.85255739497565	-0.20612157424007
Si	-1.29100996859147	7.63101088711506	0.20602375451097
Si	1.65879854857531	6.79202975168815	0.66024044644584
O	8.95270019860155	-3.83463614865789	2.22543852393384
O	2.03276926153089	6.67844388090093	2.24243494099712
O	7.14259447606897	-2.27861719370167	-0.92334288479541
O	2.49387246461871	4.74234048230812	4.06175014703393
O	6.74890666613821	-4.90417797953981	-0.77390279749775
O	0.03362901136151	5.21121704516314	3.14148644021439
O	5.85297486733084	-3.26983803025079	1.13493422666194
O	4.45051790178024	4.07821618207115	2.38150371147375
O	9.40130096112846	-5.01096428884240	-1.12750047764136
O	5.15919396378021	-0.24856974865685	2.01589624553246
O	7.73720026739753	4.38203019067238	-1.70083119831025
O	10.70336294385095	-3.18460544181736	0.34549886184517
O	5.43138777362560	5.63831623476414	-1.90421347167871
O	4.07767424078677	5.65561609212119	-4.15589707852650
O	8.76032242820614	-1.46156835261495	1.04426181742865
O	3.92955400616359	7.74615702234620	-2.54202623695363
O	1.17420007061743	9.52087928785865	-4.04876711294337
O	8.98058836330579	2.07102764597361	-1.88493509319393
O	6.95029193973453	0.19280160046317	0.10049037409952
O	2.27344982113741	9.55427664347847	-1.56565458624160
O	9.00121415620419	-0.54916464366937	-1.44341587621465
Si	1.45142788672685	5.89013464026598	3.54436715704506
Si	6.11431159842912	-3.44086340154476	-0.45197137064559
Si	3.63923169866582	3.63180162779711	3.72044321090522
Si	8.07907654234984	-5.83619337900012	-0.65717893838006
Si	10.87674846503547	-4.45890354622724	-0.67211764233967
Si	6.67917789682775	5.32800434641244	-0.91194396745145
Si	9.79509989252878	-2.60831380502334	1.56587572662184
Si	4.92209193132543	6.59068709760102	-3.12028329821219
Si	9.20640246399154	3.67287041047470	-1.66893535722902
Si	6.17437163048560	0.85543263564698	1.36990296515159
Si	1.72926407600548	10.45327287383996	-2.82702413200872
Si	9.80077436739146	0.68288247356238	-2.15374087195751
Si	7.95577507869050	-1.01664111890579	-0.30393531333763
Si	3.47170446744071	8.51854609964241	-1.18763370226012
O	2.97791312511627	-0.47501118987012	3.59844514061457

O	0.95698413355700	0.79419738799262	2.49722005458330
O	1.25772574133305	0.86796565386654	5.08609712635479
O	2.99038322840927	2.16007592756839	3.49130245566953
O	-0.39398051063970	1.19364553471209	0.31181540716604
O	-0.72982484866992	2.84834942539037	2.29947555314684
O	-3.74457998615435	4.98229899331138	0.56461372238912
O	-2.53301554527824	4.66973191404190	2.92908457425087
O	-1.13419513589650	3.45358445590492	4.81328944270800
Si	2.06325147662669	0.84853201544219	3.68347499125425
Si	0.36114915196131	1.94299678613783	1.51906927326209
Si	-3.13720222559234	5.75027589351655	1.85979670525714
Si	-1.08354201445657	4.05801627732185	3.31306877508921
O	8.29052865083118	-6.04414865490677	3.49660890351499
O	-3.19978032607521	-3.06133990457202	6.57442586832699
O	6.68131174488092	-7.84646201889489	2.44499364377077
O	-1.31348751185833	-1.56651744551051	7.68352420628511
O	8.25503412888975	-6.34122764431111	0.87238304925576
O	-5.39724004474309	-3.45307616057382	5.20330608432346
O	6.65288178830540	-3.92719253542914	3.57109637596099
O	-5.12576776350689	-1.21353587393450	6.59197752638443
O	-8.66693063046927	0.30944491075772	5.59836134506018
O	4.47855348928632	-2.51449705801067	3.25096676599088
O	-6.44543365848202	1.05619435835322	6.95836800129593
O	4.48976590373241	-5.06614407623881	2.60741047594179
O	-3.88426439834886	0.86143984592315	7.57367626735048
O	-2.24969748368021	2.84093095347034	7.11851035394104
O	0.40048566081023	2.65579951027948	6.85175643296266
O	4.25493620634077	-7.40054894984206	1.37531920418427
O	-1.50899648649741	0.87008295914326	8.77842076292041
O	0.71838405936655	0.08009172560128	7.52898323040290
Si	8.12654798601663	-7.12423025304142	2.28665354451658
Si	-2.13192350249877	-2.97421986551818	7.81689865547328
Si	8.20185650026582	-4.41812385355734	3.55197008150340
Si	-4.81718126320699	-2.81130880046620	6.57117907526006
Si	4.53334703730881	-0.89773549380469	3.37284240400046
Si	-8.08448675662781	1.12585199149272	6.87999232287899
Si	5.36247081851611	-3.70861171310970	2.61271141818281
Si	-5.30263523473983	0.07511434136674	7.56902882523021
Si	-0.93037006842546	3.49152747265480	6.42515974509658
Si	3.44309855091315	-6.10091616270625	1.92383030087359
Si	-2.74844871355661	1.79327172286001	8.26049131227189
Si	1.29839868205157	1.31539700832569	6.64944130182526
Si	5.41723673748746	-8.50947926813716	1.64885164866927
Si	-0.51904784899792	-0.38524836140442	8.47393916686208
O	1.76352296072005	4.26443956082485	-4.08465108304914
O	-0.06790739506156	5.26890691806657	-2.45284296838968
O	-0.61074055354393	7.68416327000894	-3.45739001229420
Si	0.68983128602084	3.91235858619233	-2.91514410077348
Si	-1.20156662408953	6.40825692033549	-2.64923735523762
O	1.26459542683890	-1.70464321184353	-4.53558014798215
O	-3.95937031975636	3.95779134783769	-4.55892146841152
O	3.33880967193290	-2.95239654401214	-3.48035621864112
O	-2.44882640024218	5.82879695796052	-3.49775815413267
O	-3.81004193031906	4.15710229594122	-1.92010838882497
O	1.33519980247317	-4.39786694951397	-4.42059261343485
O	-4.64533870317138	1.78883828729894	-5.92941415002484
O	4.71873961263971	-3.30780368385421	-1.25882232758821
O	-2.59910344406027	1.68990596846076	-4.29727589500602

O	-3.16840175296531	-0.36624627074223	-6.53499873265974
O	-1.11518993134554	-1.79710552183375	-5.65543136580328
O	-0.35816845151653	0.32280140885679	-4.19089526907020
O	-0.39445758050842	2.83654602229413	-3.43575807385084
O	1.78224777488655	6.92576241547452	-4.27091036697064
Si	-3.83670338224542	5.00098331941136	-3.31382540653435
Si	2.19459994787329	-3.03160346247158	-4.64479738836269
Si	-4.07078968458160	2.33442501992845	-4.52221709038429
Si	4.00556101673028	-4.07454937508325	-2.51095741905836
Si	-4.32217288816368	0.66795179391334	-7.07099482901237
Si	-0.29473454772037	-1.29549968066075	-4.34919477838769
Si	-1.24818618723416	1.45412333648298	-3.44919782722636
Si	2.54399065501117	5.55814859643917	-4.71052471355279
Si	0.62687182193411	8.05104701737929	-4.45417445231618
O	-2.79654635546060	-5.59182747047308	-7.12036262493111
O	-4.48962366314706	-4.32666543578217	-5.52069053457978
O	-2.91188624191649	-2.92802803071467	-7.22988314757306
O	-2.80804065712770	0.11788441519077	0.31403637125976
O	-2.49252259786359	2.74930383253830	-0.13344501791264
O	-1.64096686752529	0.96229826923394	-1.94717532001019
O	-5.13314531665597	2.80394311501940	0.00350809738274
Si	-3.76354366241930	-4.29220906586028	-6.97420630569690
Si	-1.85749716265007	1.27449043725629	-0.37943715457827
Si	-3.81712213788970	3.66877132057187	-0.38015372904543
O	-5.10633339160340	1.96774945738653	-3.33198770284775
O	2.81443740630881	-5.05118655438268	-1.98354748979553
O	-5.59527279036412	-2.56560481926749	2.74734682813616
O	-7.35378315335900	-1.77840600281242	4.62292235798188
O	-0.25811061039583	-6.26284448420343	-3.39564481738982
O	-8.03083028424296	0.23734813259267	3.02958333201064
O	-0.38840856101828	-5.57419299937258	-5.96449661336467
O	-0.88731306778950	-1.97272126627739	-3.01454819489425
O	-3.48726786609213	-6.51899188781833	0.73798604160424
O	-1.00488539025218	-4.26485325881394	-1.78359771266832
O	-6.59334044780133	1.03540835600248	-1.38210397605478
O	0.62609047222871	-6.08242533058131	-0.86744062907348
O	-8.29475757722357	-0.66666975694954	0.52601931621708
O	-7.36900386835731	1.78127444970510	1.02193680387524
O	-1.86889805119174	-6.76209685555056	-1.40598164414329
O	-6.50055408605134	-0.23013942399243	-3.67595880543640
O	2.70858846591386	-5.41874212879179	0.64009821674570
O	-9.06997675156838	-2.63223961767571	-1.05775859969649
O	-4.34130246365811	-0.22368692963846	-2.08066867312886
O	-7.13118429397622	-3.10775320339560	0.68700392579519
O	-8.02683377453207	-2.39944896992619	-3.49585782538260
O	-5.36453808496459	-2.60538541109982	-3.72960056469713
O	-7.26349694395493	-4.37367056371156	-1.88318790025935
O	-5.00164501762720	-5.15374401707865	-3.06593550569860
O	-2.89237026437794	-3.61204203255156	-3.55079154130899
O	-5.69929210735811	-6.20847905985359	-0.73287967671233
O	-2.71317811417212	-2.39435792763898	-1.15093035852625
O	-4.77529674566996	-4.24345753089099	0.85090323609100
O	-4.96045350218469	-1.61202909515632	0.37777286176837
Si	-6.46018189871051	-3.01089027494012	4.04318576472351
Si	0.60124736266197	-5.82129913608089	-4.70503285504066
Si	-8.49082517231554	-0.65512984546696	4.30849067866012
Si	-1.24367721184400	-6.06938267780979	-7.26039203299725
Si	-6.64620777566148	2.28456484945330	-0.35091027544893

Si	-2.43954897328242	-7.49324874867630	-0.05846700863297
Si	-0.62929917983922	-5.82680168846553	-1.87559490711926
Si	-8.42475541003829	0.63530423714940	1.49719953620720
Si	-5.60523979102644	0.59609308418076	-2.60026584882777
Si	2.24802719102667	-6.02042445360632	-0.81209472130840
Si	-8.49856040482592	-2.27698653737585	0.42200935331840
Si	-6.69084668933503	-1.75953602019948	-4.17055392252083
Si	-8.51584633712649	-3.44615908991233	-2.35692032425232
Si	-4.43023001700977	-3.91580342434311	-3.95329693146864
Si	-6.26059030334813	-5.62613559647051	-2.14511293831554
Si	-1.91596901321010	-3.04900612867958	-2.36020837541635
Si	-4.97029299924494	-5.84904357379041	0.68076500236221
Al	-3.74528362190659	-1.09443858874262	-0.72572184907536
Si	-5.58862468604891	-2.85947251946693	1.13361906698921
Si	-2.11176653811232	-1.54928272627649	-6.91062129143030
H	11.67374700221659	-5.53703601356104	-0.03807398447369
H	9.19348299273600	-8.10585800081278	2.36346298869711
H	-3.88155001877687	1.39981099140960	-8.25725798788431
H	-5.54661898608803	-0.07985098971903	-7.37196300742497
H	-6.93206199166813	-1.73335099178172	-5.60666696687247
H	-9.61083499866083	-4.23371600216931	-2.91527899996622
H	-4.80806201428008	-4.38616400187181	-7.98466801586965
H	-1.25558100102304	-7.52827100322349	-7.35483800003721
H	-8.46603500311199	2.53218800567984	6.77163499898537
H	-4.20950399258314	6.50518799102888	2.49497299400651
H	-1.81143200269988	9.00591900317830	0.20164700178566
H	0.67612796000911	11.32783101554427	-2.29765498448611
H	-4.95336995970752	5.93157195530691	-3.34598498842653
H	-7.43212804170110	3.36467305276267	-0.94325502154161
H	-9.81433496982151	1.14100198856871	1.45030700064654
H	-3.26082300504222	2.50672900127416	9.43327000149393
H	-0.74504100594514	4.85495000566497	6.90563299689955
H	1.17176499903439	6.84606699975254	4.60638300795247
H	0.01383700482831	-0.85268900971823	9.75320601268632
H	2.65630402863479	1.61378700183691	7.09828800665667
H	4.53076898552508	3.47379799967935	4.86924398141455
H	1.53720799916334	-6.93798299774779	-4.97365699680789
H	4.63541500151335	9.21535600224067	-0.64625199760367
H	-8.67488799897128	0.53574599667496	8.08886500171164
H	-5.67875099900247	-0.35844199904119	8.92691800014751
H	-5.40516100496479	-3.45826600582198	7.74597000627616
H	-2.86007300049971	-3.03986699824716	9.10083199584087
H	-1.22700999854426	-4.11162699469859	7.70400899664635
H	2.57413299622693	5.42807899523731	-6.16363200827043
H	0.22482899879961	8.10275900040078	-5.85603199784622
H	2.79507604541339	11.25689302902249	-3.42192602632197
H	6.06067099495860	7.26917499923034	-3.76691099451859
H	10.01064999778431	4.23779399881345	-2.76113100225312
H	9.87926500494681	0.42371400132996	-3.59460899994596
H	11.16639498725395	0.78744100049654	-1.60472500505326
H	9.89847200277985	3.90341200313587	-0.38688399546937
H	-1.32399899912783	-1.19594400046994	-8.10330099403534
H	-0.61662900200419	-5.49814399582755	-8.46011600118050
H	2.84818301166744	-3.04332200187882	-5.95038402307719
H	-7.31517900481947	-4.15812800641519	3.75060699768572
H	-9.78250001203607	-1.30915801008614	4.07997000050664
H	-9.40319700258656	-2.75383200616066	1.45013699871763
H	-5.76898900097272	-6.41021299969493	1.77986100285842

H	-3.08084500641491	-8.75083000186864	-0.46797100394758
H	-7.02180600507390	-6.69287800583577	-2.78419800156486
H	2.78359000138112	-7.36886300781573	-0.99247300555126
H	4.89691601091216	-9.57738000933439	2.49409397254285
H	5.82084498519757	-9.04713699718660	0.34374503692772
H	-1.34437699317695	-7.77605499685744	0.87467400612224
H	10.66950900443715	-2.02029700007892	2.56380701032123
H	8.82314101767488	-3.95574099086785	4.78387703569051
H	11.59080000759849	-3.97453398796740	-1.85888002610882
H	5.33815598903356	-0.46451101029560	4.51995499029477
H	7.15219500553421	1.34004700263753	2.37234699891024
H	7.32251199124547	6.57841099060936	-0.51330699832466
H	5.00684298414292	-4.86484798818471	-3.24385799302280
H	7.90214800260849	-7.00114300106418	-1.50659200258865
H	2.44258300564738	-6.49974699780289	2.91143199788153
Ga	-3.22336772050351	-0.30039270160197	2.25544962777061
H	-4.70398385190648	0.02098930828546	2.50906673484499
C	-2.20620923200802	-2.10720972464799	2.20100625471722
H	-1.44805663852601	-2.02863365735292	1.41444384835611
C	-2.54518571478391	-3.35365270781009	2.57322294876386
H	-3.23140742343501	-3.54238194322104	3.39702947699917
H	-2.13769975393184	-4.23207310866645	2.07495599022828
C	-1.60044577752385	-0.99914356303789	3.85070553618109
C	-2.05816153142391	0.35966793002500	3.83631007161024
H	-0.57787691837676	-1.20654874237151	3.54775699366009
H	-2.01367805448037	-1.69292634914015	4.57718946480487
H	-1.31544007753054	1.10861161285306	3.60769651640561
H	-2.76889775838983	0.64323572361713	4.61157433865652

TS6-GaH 2

Electronic energy (r^2SCAN-3c): -30773.244418057093 E_h
Electronic energy (ωB97X-D4rev/def2-mTZVPP): -30781.600982451240 E_h
G_{corr} (673.15 K, 1 atm): 0.49027 E_h
Number of atoms: 258

Cartesian coordinates (Å)

O	1.56312182673272	2.76616109798863	0.92742105441429
O	-1.94172270131058	6.79711693300784	1.43338996240078
O	5.27431938964424	2.07807812383538	0.79419098313839
O	3.63865640026324	3.90778442529513	-0.16774022586904
O	6.11852775654279	4.57397902191175	0.42082851302231
O	1.49919395161399	3.31588001594716	-1.65082280642864
O	1.36813000873344	5.27554382441467	0.11404223582799
O	-1.71584556630458	6.89543961026598	-1.18640637286286
O	0.33645246351152	7.68085896978670	0.39472853797689
O	2.93348440329359	7.42959550511712	-0.10950602104129
Si	4.86711680728849	3.64166684443497	0.86322250374531
Si	2.02342720256288	3.82705365117366	-0.20829310781084
Si	-1.28485198101751	7.63477664500371	0.20153416863102
Si	1.66479938934597	6.77839281587265	0.65370034053811
O	8.95203925032217	-3.83500769088492	2.22580318503974
O	2.05542776653928	6.71294232539684	2.23629387524629
O	7.14500777945080	-2.28383779798626	-0.92374596470639
O	2.52496774179094	4.77696874193730	4.06268360175443
O	6.74687022384345	-4.90667729110492	-0.76844383748350
O	0.07494598080946	5.22491204147011	3.10341654363522

O	5.84892726023155	-3.26507706100766	1.13552627959268
O	4.45700059035965	4.06511043258942	2.38323481902979
O	9.40004708740290	-5.00993707896448	-1.12749706739602
O	5.14964449612882	-0.24051579991897	2.01924242599019
O	7.73499177408698	4.38263685669121	-1.70077432556280
O	10.70469981168330	-3.18589751565758	0.34647010515349
O	5.42726524579074	5.64406336086285	-1.90291859135949
O	4.06479570160876	5.64708838571846	-4.14508843895036
O	8.76267960981548	-1.46250893407142	1.04348900172871
O	3.93869285352601	7.76234787032183	-2.55928440043762
O	1.17052725985481	9.52057496123303	-4.04539729633169
O	8.98000347247359	2.07145708749546	-1.88514252735891
O	6.94805285628881	0.18495889349203	0.10283802338875
O	2.27452877571229	9.55345043574848	-1.56411364454434
O	8.99958449068056	-0.54902275124936	-1.44407399126105
Si	1.47946654712635	5.91372739812862	3.53473694104923
Si	6.11256988733220	-3.44234387185782	-0.45012993562449
Si	3.63879654172644	3.63489495012115	3.72249552629150
Si	8.07873083834890	-5.83679955379858	-0.65592390347436
Si	10.87617880869579	-4.45947051483287	-0.67230105908795
Si	6.67655201307704	5.32949006724850	-0.91290935616716
Si	9.79578172455253	-2.60991366676811	1.56648034395073
Si	4.91980142021041	6.59067215474481	-3.12425423924429
Si	9.20512447845606	3.67329824245538	-1.66892938819866
Si	6.17424779234774	0.85226139933130	1.37039731309132
Si	1.72975866019244	10.45305236718344	-2.82530980537825
Si	9.80014125695255	0.68265009025770	-2.15345589183062
Si	7.95617586346309	-1.02101953340417	-0.30416049316136
Si	3.47392770005836	8.51455958040533	-1.19632972021999
O	2.98211749112675	-0.45632072245223	3.62516725045375
O	0.94533444478179	0.77047195972529	2.50707348726856
O	1.23366062255435	0.87991129057542	5.09389068575648
O	2.94476686552738	2.18481608104291	3.49172767736008
O	-0.50026154212691	1.12586905985093	0.37935423928959
O	-0.69930291721899	2.85284247563993	2.31555610186859
O	-3.74919877296273	5.00283327178542	0.58268190231299
O	-2.49060619627932	4.69392706119582	2.93813874963337
O	-1.06585339523525	3.48249798694515	4.81918009437092
Si	2.04669822625479	0.85146996486961	3.69477101899878
Si	0.33618141275780	1.90068048940796	1.52134452751573
Si	-3.12097648234350	5.76510367488474	1.87274720058661
Si	-1.03553816508637	4.07715772043138	3.31721334912057
O	8.28338256665750	-6.04168130561446	3.49625561423016
O	-3.20349513662955	-3.05407773797061	6.57767299286838
O	6.68277188126767	-7.84929637991042	2.44443115426049
O	-1.31764467540965	-1.56359976020598	7.68795328882419
O	8.25911612963844	-6.34313534383591	0.87237062753134
O	-5.39662307047381	-3.43980116166419	5.19246423880128
O	6.65090658129536	-3.91704618906604	3.57095616698928
O	-5.13237857811514	-1.21056270252236	6.58835290548883
O	-8.66312329758761	0.29319715982380	5.61161261808975
O	4.47089676246194	-2.50879554813091	3.24943539125543
O	-6.44209385847330	1.06261578116242	6.95944520702353
O	4.49021784471050	-5.06358187593720	2.61107833712832
O	-3.88362530917560	0.86278254416695	7.57093010130813
O	-2.24750451417560	2.83517070141593	7.10237696642158
O	0.40377026379815	2.65498343013011	6.88511658411750
O	4.25667231154625	-7.39755997264970	1.37895851360236

O	-1.50621550694624	0.87506947341766	8.77486816044031
O	0.72233019579567	0.07541983630141	7.53622167213330
Si	8.12605458675032	-7.12422781834798	2.28735940556913
Si	-2.13372140757801	-2.97476191516113	7.81447235415962
Si	8.19774480337495	-4.41537074105189	3.55225842389473
Si	-4.81777265260409	-2.81113695447343	6.56623916985967
Si	4.52907049322547	-0.89313589375816	3.37745430891585
Si	-8.08208637399627	1.12530619256983	6.88314125831633
Si	5.35735103679519	-3.70310385095695	2.61394269347786
Si	-5.30191807583598	0.07463227078912	7.56914614434262
Si	-0.91653788489903	3.49317055513776	6.43538895133570
Si	3.44264861532903	-6.09694793423780	1.92537769996176
Si	-2.74646067162462	1.79565563450463	8.25586377051700
Si	1.29674833421220	1.31442826925534	6.66049671615670
Si	5.41636545151086	-8.50889488475693	1.64900295027403
Si	-0.52031925059492	-0.38679776256183	8.47657729426334
O	1.73227098820034	4.26439694541437	-4.10850482383538
O	-0.06431129822197	5.27803181843716	-2.44286929697541
O	-0.61902140764877	7.68455826601499	-3.46115619279232
Si	0.67785019298579	3.91711554739631	-2.92019572868164
Si	-1.20371356375712	6.41053809307216	-2.64650504987409
O	1.27735014450162	-1.68736540747877	-4.55590001768107
O	-3.95955308089432	3.95351168945188	-4.55162936984377
O	3.33744258030699	-2.94969245734832	-3.47884967242087
O	-2.44706960517065	5.81615495578498	-3.49040129711813
O	-3.84109015095832	4.16641880747268	-1.90873486540743
O	1.33070862288787	-4.38721037612082	-4.42499357843843
O	-4.64698405024707	1.79064488153545	-5.93240283268635
O	4.71885455454633	-3.30860158258211	-1.25990748592902
O	-2.61285944028412	1.67843320913379	-4.28484522736064
O	-3.17006223695769	-0.36468374831282	-6.53528436793676
O	-1.11657644962492	-1.77974943867112	-5.64834892372051
O	-0.36417188655582	0.32348518361065	-4.15258457759763
O	-0.42348814430737	2.84132430810465	-3.40884944330195
O	1.77763477098024	6.92594625567677	-4.26772879352459
Si	-3.84091552011481	5.00007387570378	-3.31012043511016
Si	2.19738250971079	-3.02487889460358	-4.64669766111588
Si	-4.07881687644212	2.33117486186647	-4.52177523693475
Si	4.00404786615583	-4.07483040942113	-2.51102688638440
Si	-4.32289276480903	0.66799896313570	-7.07276017746688
Si	-0.28215314714686	-1.29162556343851	-4.34579391883259
Si	-1.26757757114282	1.45208259274351	-3.42457224147073
Si	2.53463696008855	5.55679446596582	-4.71171114991698
Si	0.62334251225814	8.05184644432480	-4.45333920508484
O	-2.79704915850126	-5.58927585599735	-7.11745038565636
O	-4.47026055957887	-4.30596838124989	-5.50749874988723
O	-2.90468403742652	-2.92609239202393	-7.23122730661814
O	-3.00771338420727	0.20567808886229	0.28753937999680
O	-2.49333771685803	2.79203782211253	-0.11794415145248
O	-1.68254379572973	0.96885685335623	-1.92158708022350
O	-5.13540147385126	2.81623904542306	0.03228340139931
Si	-3.76085981395332	-4.28711754985154	-6.97035423989102
Si	-1.93183598995650	1.28778568182690	-0.35990998534882
Si	-3.82949509910554	3.69596307654216	-0.36543246612189
O	-5.12790835182426	1.96924723849446	-3.34033576843861
O	2.81171805310526	-5.05184203724278	-1.98744024788688
O	-5.60159217604614	-2.58704791860812	2.70707497591720
O	-7.33765203343678	-1.76615764696552	4.58436955169959

O	-0.25806247833810	-6.24840406937845	-3.39406961554609
O	-8.04935362902006	0.27485113993220	3.04635100264939
O	-0.38806218606824	-5.57398095670005	-5.96428748040403
O	-0.83984799194191	-2.00252348508212	-3.01636882143361
O	-3.48923332400968	-6.54214231973944	0.73392936212410
O	-1.02346660779959	-4.27473591709623	-1.75638960205976
O	-6.56312524094515	1.02862105475810	-1.36132008104714
O	0.62827849509544	-6.08685148155184	-0.86526821701364
O	-8.26164907833246	-0.68018746691772	0.55416808840363
O	-7.38114467288458	1.79149489417202	1.02046135021944
O	-1.86407154325775	-6.77614842838727	-1.41099804107380
O	-6.55619936531545	-0.21949149555332	-3.67683404533541
O	2.71767281489135	-5.41642990489079	0.63648599219517
O	-9.06818545670575	-2.61496479804991	-1.06381802716952
O	-4.36248233027083	-0.26465598391218	-2.16086406840722
O	-7.15617639067578	-3.16203937955483	0.67911221206307
O	-8.04771464143056	-2.40455828929156	-3.50848468093526
O	-5.38616218655218	-2.59546320557219	-3.72358183355080
O	-7.26632789374896	-4.37050433535840	-1.88702163932854
O	-5.00384100509094	-5.14258883163510	-3.06140473062959
O	-2.91138301708048	-3.56723524567330	-3.50386154166462
O	-5.70576394374541	-6.21544829413184	-0.73409452054079
O	-2.63011523972454	-2.32212882963241	-1.11638917737238
O	-4.75536135240666	-4.26556344635240	0.83744516561075
O	-4.99665769129616	-1.69279156521181	0.27843186031566
Si	-6.45033571530800	-3.01394059835270	4.02262736437356
Si	0.60337677326833	-5.81569271843021	-4.70427954054906
Si	-8.49046833262520	-0.65258840109324	4.30784736024225
Si	-1.24395781499505	-6.06892623143222	-7.25954934427446
Si	-6.64411179417697	2.28858532736465	-0.34537407326076
Si	-2.43433735014354	-7.50183873340064	-0.06052617197047
Si	-0.62896273299702	-5.83091389485677	-1.86859926537789
Si	-8.42568667176083	0.63686558876739	1.50280255217688
Si	-5.62562695152443	0.59153626350134	-2.62129160252569
Si	2.25011927853942	-6.01943745131553	-0.81072859943573
Si	-8.49841938291388	-2.28656072598185	0.42254110726440
Si	-6.71258644121214	-1.75529851993152	-4.17139527777996
Si	-8.52043885396334	-3.44484193757050	-2.35766417657718
Si	-4.43500200278470	-3.89592195226809	-3.93842761703225
Si	-6.26262139411453	-5.62544940733739	-2.14372382714798
Si	-1.89253792333507	-3.03437642815360	-2.33541304907850
Si	-4.96409093774060	-5.86969279348139	0.67878866973473
Al	-3.78030052826020	-1.10245790032000	-0.78327717306437
Si	-5.60926136408617	-2.91046732863172	1.09928610371179
Si	-2.10970341447100	-1.54525899006109	-6.90867703941661
H	11.67374700182747	-5.53703599815734	-0.03807399901677
H	9.19348300157205	-8.10585799994226	2.36346300095278
H	-3.88155000229345	1.39981100053892	-8.25725800058301
H	-5.54661900021758	-0.07985099913599	-7.37196299997229
H	-6.93206199841587	-1.73335099815256	-5.60666699746392
H	-9.61083500068397	-4.23371600052322	-2.91527899808237
H	-4.80806199774505	-4.38616400069158	-7.98466799989170
H	-1.25558100036459	-7.52827099902338	-7.35483800038286
H	-8.46603500082352	2.53218800046047	6.77163500108337
H	-4.20950399940829	6.50518799905617	2.49497299888879
H	-1.81143199981773	9.00591899805708	0.20164700203629
H	0.67612799923806	11.32783099951328	-2.29765499974696
H	-4.95337000040467	5.93157199985445	-3.34598500237551

H	-7.43212799859655	3.36467299934769	-0.94325499770468
H	-9.81433500081359	1.14100200039934	1.45030699983253
H	-3.26082299933679	2.50672899903335	9.43327000104385
H	-0.74504100053928	4.85494999851456	6.90563299962808
H	1.17176499849782	6.84606699767707	4.60638300102422
H	0.01383699978297	-0.85268900107073	9.75320600111798
H	2.65630400067203	1.61378700067623	7.09828800016443
H	4.53076900176480	3.47379799781559	4.86924400027427
H	1.53720799978132	-6.93798299937203	-4.97365699816814
H	4.63541500107873	9.21535600149258	-0.64625199840589
H	-8.67488799901696	0.53574600096762	8.08886499843087
H	-5.67875099883603	-0.35844199767783	8.92691800004917
H	-5.40516100004805	-3.45826600069055	7.74596999976539
H	-2.86007299930374	-3.03986699772627	9.10083200031332
H	-1.22700999755112	-4.11162699813971	7.70400899671924
H	2.57413299888191	5.42807899839832	-6.16363199912918
H	0.22482900133017	8.10275900029407	-5.85603199922588
H	2.79507599845961	11.25689300064472	-3.42192599987406
H	6.06067100078574	7.26917500017016	-3.76691099946286
H	10.01064999858272	4.23779399924157	-2.76113100047234
H	9.87926499788986	0.42371400053867	-3.59460900064363
H	11.16639499825807	0.78744100169909	-1.60472500009586
H	9.89847199806869	3.90341200100247	-0.38688400172419
H	-1.32399899929805	-1.19594399941248	-8.10330099964663
H	-0.61662900168277	-5.49814399762803	-8.46011599936270
H	2.84818300011327	-3.04332200110841	-5.95038399911012
H	-7.31517899983477	-4.15812800038838	3.75060700182729
H	-9.78250000192246	-1.30915800166087	4.07996999796795
H	-9.40319699884986	-2.75383200062802	1.45013700171555
H	-5.76898900025593	-6.41021300007316	1.77986100189171
H	-3.08084499944755	-8.75082999796682	-0.46797100060256
H	-7.02180600203164	-6.69287800102014	-2.78419799861074
H	2.78359000058848	-7.36886300070994	-0.99247300203636
H	4.89691599980028	-9.57738000011209	2.49409399908936
H	5.82084500123691	-9.04713700183953	0.34374499999041
H	-1.34437699981725	-7.77605499858333	0.87467400166954
H	10.66950899875226	-2.02029700005696	2.56380700016752
H	8.82314099954661	-3.95574100151653	4.78387700041062
H	11.59079999834757	-3.97453400008153	-1.85888000156382
H	5.33815600006747	-0.46451100174949	4.51995500117916
H	7.15219500173989	1.34004700160803	2.37234699780200
H	7.32251199878605	6.57841100150284	-0.51330699885087
H	5.00684300203968	-4.86484799847934	-3.24385800110580
H	7.90214800021602	-7.00114299794957	-1.50659200248076
H	2.44258299925123	-6.49974699982734	2.91143200058857
Ga	-3.87701129374415	0.16855226680762	2.08398741885817
H	-5.38719727319478	-0.02542697813012	2.09756444488128
C	-1.03073712257281	-3.31685780816419	2.09468794170064
H	-1.50977865593449	-3.56507568137413	1.14626239061321
C	-0.37253179299071	-4.25614185033478	2.76700738431764
H	0.11817558376187	-4.03957756585993	3.71421216924528
H	-0.29792805433267	-5.27123267864624	2.38790125848455
C	-1.18606288548861	-1.89202907231939	2.54514996396080
C	-2.65779286216044	-1.56183918967045	2.78870793976768
H	-0.78218911041242	-1.22888779984808	1.76761728111556
H	-0.59558144746379	-1.72302933465202	3.45476936040522
H	-3.20890778254601	-1.95533217466898	1.91780261244634
H	-3.07632521326386	-2.08865619876544	3.64712811620446

C	-3.23366440467334	1.46678138090766	3.45793561739280
C	-2.38364346806635	0.34100497241187	3.77275117383870
H	-2.77699742409219	2.32077289766847	2.98845479635033
H	-4.05153751872772	1.69046246870994	4.13848450305628
H	-1.35143019766997	0.38025018241931	3.43947315797239
H	-2.53179454372931	-0.19876889269568	4.70412372448950

TS7-GaH 2

Electronic energy (r^{2}SCAN-3c): -30773.255584996619 E_h
Electronic energy (ωB97X-D4rev/def2-mTZVPP): -30781.588071218892 E_h
G_corr (673.15 K, 1 atm): 0.48790 E_h
Number of atoms: 258

Cartesian coordinates (Å)

O	1.49205515522301	2.89838796120751	1.03373793047774
O	-1.94085080295915	6.77691349311629	1.43100164055117
O	5.26519835841534	2.07910186780040	0.80455795926159
O	3.60359267802295	3.88015153815669	-0.15820977756504
O	6.09191126809409	4.57544009277816	0.41667273645493
O	1.44128880731294	3.28572807789981	-1.61093191478782
O	1.36986986944374	5.32804168770215	0.03007911514481
O	-1.70717776265225	6.88689112916916	-1.19154162526704
O	0.32545336499652	7.70416908636341	0.40663721957933
O	2.93446354663901	7.47848522410348	-0.04667831262168
Si	4.84307127248535	3.64115409196825	0.86590072755891
Si	1.98352594848089	3.84497443165207	-0.18872123708299
Si	-1.29387549390022	7.62932499749830	0.20195057052699
Si	1.64862953243026	6.79481866338678	0.65932409027754
O	8.94692524647873	-3.83101392297475	2.22531814693287
O	1.99896136929411	6.65200341364648	2.24311170113056
O	7.14593867134316	-2.28012046126451	-0.92104623632016
O	2.50573958364801	4.75186635296111	4.08539770358933
O	6.74835574739456	-4.90469870811925	-0.76958507531629
O	0.04739283252589	5.11546077496648	3.14447975413434
O	5.85018202404638	-3.26713182680924	1.13662913479387
O	4.44094222701582	4.07475602236352	2.38502085083958
O	9.40114241932950	-5.01108087457100	-1.12868839200157
O	5.15482277135766	-0.24729686549485	2.01869242146802
O	7.73385131770350	4.37803479038054	-1.68857674519382
O	10.70147969018257	-3.18594710624231	0.34661462455908
O	5.42954719040969	5.63114035949659	-1.91293765356309
O	4.10243106564368	5.68287074030640	-4.18635750746981
O	8.76218987851073	-1.45799910510552	1.04478082271687
O	3.92268567539745	7.73614535330950	-2.52899152821312
O	1.17418640618967	9.52383169082152	-4.04994247939705
O	8.98090221943942	2.07193349702442	-1.88591586918746
O	6.94629947554170	0.19035648367911	0.10250924722362
O	2.27237993347835	9.55336329674642	-1.56502898924647
O	8.99960649096211	-0.54723668831161	-1.44338449365706
Si	1.43168368240105	5.86299869064674	3.55189689928697
Si	6.11606885410985	-3.43984321501889	-0.44936013286086
Si	3.63233695309533	3.63010030173211	3.72638118587148
Si	8.07962683725704	-5.83619824690027	-0.65702255651624
Si	10.87649270111372	-4.45891124702630	-0.67211597601567
Si	6.67022739405146	5.32723075728481	-0.90999035772744
Si	9.79390499531512	-2.60704888129302	1.56632915606843

Si	4.92579472944689	6.59705440315970	-3.11981657309794
Si	9.20504610584894	3.67389496977432	-1.66751012355929
Si	6.17159955262014	0.85583951777355	1.37195297472387
Si	1.72957160733546	10.45327158342083	-2.82613697734221
Si	9.80065914982151	0.68400115294527	-2.15396600513238
Si	7.95572291625848	-1.01467636078106	-0.30277993214871
Si	3.46860421255662	8.51825879632369	-1.17822173245002
O	2.97273716361770	-0.47131609261285	3.60666524797341
O	0.95826661061877	0.76752497499627	2.47577296386756
O	1.24315452020697	0.87698388789314	5.07966201784646
O	2.97286627009690	2.16388583557859	3.48611002650426
O	-0.40157322562578	1.19389772543036	0.27249501924449
O	-0.82044499177371	2.73071166714659	2.36156563919858
O	-3.74290117885775	4.99361579597471	0.56278377587371
O	-2.53406030444486	4.66365622955925	2.92869048875726
O	-1.17706860979229	3.43126743108556	4.83860565726548
Si	2.04706538415596	0.84978309855389	3.67448765397067
Si	0.30300788467340	1.91255856259320	1.52886760186947
Si	-3.13276838861719	5.74914781656434	1.86356840249426
Si	-1.11157685427249	3.99797729659972	3.32716117560310
O	8.28728018085451	-6.04181594733731	3.49576673661542
O	-3.19578191490934	-3.07259893701904	6.57630377641103
O	6.68131433843569	-7.84556463504670	2.44440190725092
O	-1.31792054391402	-1.56640792154376	7.69631802690599
O	8.25796832628009	-6.34252650153687	0.87178791312857
O	-5.42834704122417	-3.48804089471638	5.24855681673337
O	6.64794966299644	-3.92568653026427	3.57290644745027
O	-5.12372408166276	-1.21981615122008	6.59266274729084
O	-8.67444814309837	0.33541781750052	5.58177987470450
O	4.46934823323208	-2.51186406507623	3.25001994141730
O	-6.44688020898668	1.04657061365193	6.95248196160645
O	4.48259691233225	-5.06061810572466	2.60580920564844
O	-3.88429001658625	0.85645291735505	7.58231450454652
O	-2.25247181070085	2.84977117316992	7.14706895709551
O	0.39847782038626	2.65658240816211	6.85982401250675
O	4.25684498632209	-7.39804869810782	1.37277798472761
O	-1.50818716497812	0.86900631862595	8.78725370039861
O	0.71335002857608	0.07810957168933	7.52174857139211
Si	8.12711169340754	-7.12330101349436	2.28681658470570
Si	-2.13246215825716	-2.97624657213466	7.81962664977825
Si	8.19809254380038	-4.41559638504041	3.55241902439807
Si	-4.80918164875290	-2.81519325352178	6.58354166923050
Si	4.52723515994348	-0.89371187965727	3.37601877384714
Si	-8.08423648128235	1.12746421407461	6.87796715685174
Si	5.35807979259882	-3.70392801310028	2.61443408641590
Si	-5.30100277677280	0.07043673661771	7.56764209264453
Si	-0.93808553193524	3.48936593143272	6.44041825612937
Si	3.44242774789016	-6.10184934948674	1.92451960267284
Si	-2.75125343716746	1.78843327511727	8.27039622275280
Si	1.29544112834580	1.31749085491568	6.64613260087772
Si	5.41791583570732	-8.50912609693975	1.64867245819820
Si	-0.51831361903847	-0.38311246986942	8.47733278087964
O	1.82502649813449	4.27686719257386	-4.04571507806038
O	-0.06808270578045	5.26964306276271	-2.47189831237750
O	-0.61066618486564	7.68737650530082	-3.45939953487508
Si	0.70790743853878	3.92055674830775	-2.92230528982628
Si	-1.20364596038360	6.40784281583959	-2.65776062196617
O	1.24704356301054	-1.73149222026970	-4.50345154914424

O	-3.98369711018311	3.94938873943866	-4.55595508868418
O	3.34105424961685	-2.95798105557771	-3.48272444425782
O	-2.44807019521383	5.82997242600876	-3.51509727909901
O	-3.80485630028592	4.17545490691952	-1.92635364024744
O	1.33903342428252	-4.41423980384416	-4.41442360150634
O	-4.66835096197034	1.77589603924007	-5.92069657948735
O	4.72081341795526	-3.30656348461741	-1.25788459891198
O	-2.54740786951451	1.72144830201183	-4.39940312268972
O	-3.17111261665017	-0.36889940060904	-6.53601033487274
O	-1.11325195614128	-1.80529887621556	-5.66175978708221
O	-0.32794370894325	0.33106186550161	-4.23664610797833
O	-0.34671970683345	2.86268168294495	-3.52979687636978
O	1.78642086985114	6.93141347097353	-4.27446013447326
Si	-3.83742127385078	5.00482541540604	-3.32261426698816
Si	2.19138693757387	-3.04360481878718	-4.64106039625237
Si	-4.04721447782152	2.32345671633894	-4.53308990888222
Si	4.00909420298973	-4.07457181119295	-2.50930219698532
Si	-4.32543022619888	0.66717630173823	-7.07018431555361
Si	-0.30606242417713	-1.29000966801886	-4.35280785247440
Si	-1.20936174220132	1.49059608530592	-3.52830791952426
Si	2.56204793842994	5.57145413249089	-4.71206782538953
Si	0.62779541325408	8.05366722698883	-4.45501980469568
O	-2.79790990255977	-5.59322424648472	-7.11564546292573
O	-4.51868136046372	-4.33071423684430	-5.53907229239305
O	-2.91352768073629	-2.93052936886503	-7.22906646424052
O	-2.82413028954683	0.14874705616457	0.14393470019459
O	-2.47030326546758	2.78112992389975	-0.15895933778109
O	-1.62817504405269	1.03951968848896	-2.02855330302771
O	-5.12963842416640	2.82532976028094	-0.02181577166745
Si	-3.76659378454333	-4.29390689799593	-6.97865670715187
Si	-1.85091188858864	1.30462311322686	-0.45139122223022
Si	-3.80696752784467	3.68460092646514	-0.38786669359004
O	-5.00018756643940	1.90291928635861	-3.29185882926314
O	2.81800299416409	-5.05013626784961	-1.97894267724482
O	-5.59379951368902	-2.52391517973749	2.82964400671019
O	-7.38162408756058	-1.78881270485197	4.67041139236100
O	-0.25250560984158	-6.30259628308474	-3.40269297465152
O	-8.01446084072547	0.19945643739818	3.01125828246951
O	-0.39100476873752	-5.57324981763180	-5.96374541555387
O	-0.95762572051059	-1.91654570043555	-3.01477790130480
O	-3.48558142032786	-6.51024178037562	0.74257733037127
O	-0.97562667094897	-4.25986717761149	-1.86834462624782
O	-6.58325544014557	1.03788727544821	-1.38542930121993
O	0.62190111510594	-6.07116685849032	-0.87321081278100
O	-8.33738485299511	-0.63518007729166	0.49539496871918
O	-7.37424198672280	1.79236067401665	1.01385910629493
O	-1.87593425762691	-6.74071944930522	-1.40046928441940
O	-6.53735636876382	-0.20631679741572	-3.70785247160329
O	2.69675428858578	-5.42239580846323	0.64379353645811
O	-9.06528235617846	-2.65852064147995	-1.03410713258041
O	-4.45672911875484	-0.40337568958605	-2.09715994050065
O	-7.02446161432931	-2.95069598659248	0.63651349482925
O	-8.04385614710371	-2.37872606952613	-3.48118608675497
O	-5.39245588866793	-2.61266563187855	-3.74278540044417
O	-7.26283811834665	-4.36704890447673	-1.89061019674717
O	-5.00474145791513	-5.15404654520841	-3.07821235610036
O	-2.90358358661930	-3.61466806764501	-3.60058552892838
O	-5.68741543993839	-6.15338535982630	-0.72064593592609

O	-2.76973922152687	-2.49604834964279	-1.16554083290555
O	-4.75295499758081	-4.21791389477186	0.92431020030769
O	-4.70340544157836	-1.54692077546962	0.65138487667370
Si	-6.47719261977342	-3.01481664253217	4.10124679258672
Si	0.59957961674536	-5.83195889067217	-4.70838848304688
Si	-8.49532526150215	-0.65276082945541	4.31196918482063
Si	-1.24524760040360	-6.06931859350456	-7.26058328921146
Si	-6.63703744333793	2.29157252340265	-0.35512354599210
Si	-2.44130665030452	-7.48601622831715	-0.05685540757052
Si	-0.62707648130670	-5.83159898410869	-1.89508487974059
Si	-8.42550242881923	0.64883616823158	1.49518795798947
Si	-5.61752377915361	0.55888165853192	-2.60574813489090
Si	2.24594258273366	-6.02032250244696	-0.81312383398029
Si	-8.48035437120798	-2.24935176312678	0.42492733727447
Si	-6.71053565492805	-1.74954783959561	-4.17418800928150
Si	-8.52012226610271	-3.44316120059578	-2.35431337589138
Si	-4.45058982052592	-3.91455804859755	-3.97529687237732
Si	-6.25848848082844	-5.61770847658137	-2.14768030962249
Si	-1.93676923030593	-3.06259138811862	-2.39992715062049
Si	-4.95954695193091	-5.82380189464758	0.70047000697398
Al	-3.70709183988094	-1.12451956634462	-0.74581514918242
Si	-5.51951835792840	-2.81735387474227	1.22878140544850
Si	-2.11382477040331	-1.55082530747441	-6.91282684410971
H	11.67374700357141	-5.53703599846776	-0.03807399934400
H	9.19348301432711	-8.10585800905693	2.36346300340767
H	-3.88154999655809	1.39981100410981	-8.25725800266080
H	-5.54661900621081	-0.07985100505905	-7.37196299911307
H	-6.93206199782706	-1.73335099931553	-5.60666699927623
H	-9.61083499566021	-4.23371599677813	-2.91527899534716
H	-4.80806199412435	-4.38616400242986	-7.98466799667127
H	-1.25558099748556	-7.52827099862432	-7.35483799897269
H	-8.46603500075459	2.53218799540561	6.77163500049619
H	-4.20950399608990	6.50518799692674	2.49497299742384
H	-1.81143199878100	9.00591899606495	0.20164700207372
H	0.67612800524930	11.32783104180234	-2.29765500826361
H	-4.95337000857099	5.93157200663643	-3.34598500359742
H	-7.43212798091397	3.36467297448719	-0.94325498981718
H	-9.81433500746434	1.14100200184390	1.45030699867994
H	-3.26082299998968	2.50672899954241	9.43327000171862
H	-0.74504100057590	4.85494999337126	6.90563299854679
H	1.17176499826893	6.84606699509964	4.60638299885689
H	0.01383699005300	-0.85268899663049	9.75320598566939
H	2.65630399860906	1.61378699938050	7.09828800125834
H	4.53076900466927	3.47379799659845	4.86924400381948
H	1.53720800774224	-6.93798300715453	-4.97365700199001
H	4.63541500285172	9.21535600138892	-0.64625199817181
H	-8.67488799973708	0.53574600072461	8.08886499927684
H	-5.67875100236211	-0.35844199608855	8.92691800869708
H	-5.40516097982390	-3.45826597975529	7.74596996528570
H	-2.86007294157689	-3.03986699643161	9.10083190118803
H	-1.22701000259339	-4.11162706851063	7.70400915148531
H	2.57413299990167	5.42807899731863	-6.16363199846628
H	0.22482900254412	8.10275899789732	-5.85603199820408
H	2.79507599477177	11.25689299761983	-3.42192599794425
H	6.06067100225374	7.26917500068667	-3.76691100023274
H	10.01064999896344	4.23779399937728	-2.76113100073048
H	9.87926499899350	0.42371400128583	-3.59460900167019
H	11.16639499865901	0.78744100221105	-1.60472500044026

H	9.89847199843270	3.90341200133150	-0.38688400143670
H	-1.32399900042953	-1.19594400207167	-8.10330099654381
H	-0.61662900405193	-5.49814399904571	-8.46011599708359
H	2.84818299870987	-3.04332200186070	-5.95038399759346
H	-7.31517901174431	-4.15812801721304	3.75060699925595
H	-9.78250000029185	-1.30915800197890	4.07996999690251
H	-9.40319699727614	-2.75383200122301	1.45013700454530
H	-5.76898899556314	-6.41021299842228	1.77986100079622
H	-3.08084500445297	-8.75082999767894	-0.46797100451718
H	-7.02180599963805	-6.69287799906178	-2.78419799602380
H	2.78359001737930	-7.36886301856437	-0.99247301394532
H	4.89691601053944	-9.57737999126828	2.49409398864937
H	5.82084502147473	-9.04713702212437	0.34374498504989
H	-1.34437698714750	-7.77605500558267	0.87467401150408
H	10.66950899936039	-2.02029700109556	2.56380699999579
H	8.82314100359159	-3.95574100216114	4.78387699807161
H	11.59079999918274	-3.97453400104720	-1.85888000209808
H	5.33815600610316	-0.46451100450110	4.51995500304996
H	7.15219500174253	1.34004700095396	2.37234699655203
H	7.32251199752034	6.57841099992317	-0.51330699919382
H	5.00684300537291	-4.86484799755769	-3.24385800472553
H	7.90214800081328	-7.00114300020565	-1.50659200436848
H	2.44258282564361	-6.49974687761233	2.91143201601857
Ga	-3.63982722222614	-0.18501972671241	2.11160673496074
H	-4.78692211095298	0.94047609197201	2.08700785264723
C	-0.33611358212691	-3.81414178740020	4.10501415303763
H	-1.02871782837342	-4.44836815285618	4.65815073646420
C	-0.29054226458000	-2.45671581500722	4.39320754346330
H	0.58006935170374	-1.90796906861120	4.03116343772599
H	-0.66450328465809	-2.10798457898264	5.35200344768858
C	0.16738694896390	-4.29122434944881	2.79527184412385
C	-0.77073166229246	-3.71971309568586	1.69859713661376
H	0.19705833217179	-5.38315452089112	2.73089043710755
H	1.17858961157152	-3.90322534934746	2.60717303169601
H	-0.36114192161474	-3.96620022269497	0.71326245387815
H	-1.75877152789183	-4.19537819461942	1.76443050610952
C	-1.52563497111554	-1.77973968869867	3.16089463827549
C	-0.91424874574212	-2.20171906298088	1.83301489825893
H	-1.44935500453003	-0.73650551648548	3.47280358040357
H	-2.44039796429793	-2.26149653289861	3.49036732392696
H	-1.49148334701801	-1.84533072640236	0.97965775051034
H	0.07159272047978	-1.72573811225651	1.74354583925571

TS8-GaH 2

Electronic energy (r^2SCAN-3c): -30773.270462651242 E_h
Electronic energy (ωB97X-D4rev/def2-mTZVPP): -30781.601234137910 E_h
G_{corr} (673.15 K, 1 atm): 0.49404 E_h
Number of atoms: 258

Cartesian coordinates (Å)

O	1.49208009832638	2.91374565346951	1.03183816682430
O	-1.94166683891976	6.77646640609215	1.43184232181708
O	5.26290435308370	2.08040825653105	0.80833199407423
O	3.60808617458661	3.88650146406258	-0.15824172281391
O	6.09710961693838	4.57367529106168	0.41684109150840
O	1.44961860200354	3.28975707602229	-1.61427230721575
O	1.37386723754077	5.33705615659445	0.01934661185776
O	-1.70427454854114	6.88683631822212	-1.18939164828939
O	0.32547935547801	7.70769477758060	0.41239796557216
O	2.93595293599129	7.48615597743045	-0.04032192011956
Si	4.84526785639743	3.64409834926263	0.86773119990263
Si	1.98812490997410	3.85339644513090	-0.19270779879975
Si	-1.29318415317842	7.62987738662852	0.20429382741726
Si	1.64975743497999	6.79865694304834	0.66078218709625
O	8.94623777729755	-3.83020073123545	2.22495370156691
O	1.99966202736445	6.64734414151054	2.24355076519233
O	7.14471969431358	-2.27940092795004	-0.91811484047399
O	2.50183589492679	4.74595057708049	4.08449883619150
O	6.74845073007957	-4.90443106883627	-0.77187774121139
O	0.04306875422551	5.11833172360078	3.14690270680252
O	5.84787058494505	-3.27322904950503	1.13694116868955
O	4.44430013307923	4.08232494307099	2.38571918416749
O	9.40138585559231	-5.01143847293569	-1.12855144272708
O	5.15470628500331	-0.24928677099781	2.01847718329337
O	7.73492107039590	4.37909623673804	-1.69079743795962
O	10.70080243463375	-3.18556562660614	0.34639102038947
O	5.43038375265976	5.63089305740349	-1.91236415032509
O	4.09808507828836	5.67862058894887	-4.18145027197558
O	8.76205274307409	-1.45655710902243	1.04538197580405
O	3.92137766600245	7.73428523615040	-2.52712956204540
O	1.17448441456223	9.52346268576060	-4.05037002982468
O	8.98094275436047	2.07201002030799	-1.88590513518107
O	6.94524376114940	0.19207233203606	0.10301963793735
O	2.27229534967282	9.55383917294660	-1.56558478014214
O	8.99843044488344	-0.54681847651324	-1.44310218113164
Si	1.43040718213634	5.86067303270458	3.55291876297725
Si	6.11524587701697	-3.44040224262388	-0.44940798277725
Si	3.63526819936740	3.63058817917879	3.72499960265862
Si	8.07941585046361	-5.83596253113955	-0.65754416310515
Si	10.87651666701927	-4.45869974804975	-0.67200874576178
Si	6.67161288388982	5.32704530981829	-0.91029108690900
Si	9.79351451084569	-2.60611269160143	1.56611918004515
Si	4.92463094879038	6.59576020753473	-3.11919026759592
Si	9.20536258566779	3.67399024748826	-1.66772501234224
Si	6.16990504686798	0.85648530382193	1.37317713879323
Si	1.72937891118312	10.45331806692585	-2.82663951442030
Si	9.80037439755892	0.68398392305598	-2.15382604144681
Si	7.95463441163607	-1.01300995829953	-0.30175600353898
Si	3.46875589805812	8.51955949458301	-1.17759378502434
O	2.97785396005619	-0.47192685472707	3.61975081717155

O	0.98631018469646	0.77147661883389	2.45713871576295
O	1.24412158997314	0.89775567883893	5.07217646013070
O	2.98914551486764	2.16038625138677	3.47970695346864
O	-0.39816988373345	1.20073833988650	0.27292947094153
O	-0.81044238828126	2.72607333478865	2.36988672719089
O	-3.74556921239480	4.99523765854472	0.56079403777491
O	-2.53448405700720	4.65700268308423	2.92251937763679
O	-1.18191218266386	3.43407906735453	4.84122755939582
Si	2.05580052504367	0.85121466249350	3.67284724355623
Si	0.31216975445383	1.91777001050760	1.52703893240504
Si	-3.13321977356255	5.74770953656385	1.86289280066884
Si	-1.11137542346422	3.99574380032431	3.32856722620428
O	8.28888140579724	-6.04194707075754	3.49570779552344
O	-3.19782887241928	-3.06808019751450	6.57973685849031
O	6.68211724998799	-7.84605645141566	2.44535746601817
O	-1.30379211909366	-1.57415333650814	7.68906816898927
O	8.25614039909525	-6.34190026833407	0.87177208555004
O	-5.42299396345627	-3.48210578953666	5.24123792277966
O	6.64823752085548	-3.92766819626528	3.57379125719653
O	-5.12808847541829	-1.21857823151607	6.59208710835881
O	-8.67526771039853	0.33856007461257	5.58015909819878
O	4.47054767587323	-2.51243016281703	3.25161252427556
O	-6.44762336333817	1.04791563046874	6.95271095769365
O	4.47948943615840	-5.06091140390347	2.61476443014179
O	-3.88506023429435	0.85615140599027	7.58165346647990
O	-2.25355626105129	2.85063549873671	7.14959319759522
O	0.39661571704721	2.65800751461135	6.86421453057113
O	4.25753553467417	-7.39830149660607	1.37630393562120
O	-1.50984759175928	0.86219444391642	8.78283973756461
O	0.71640938356289	0.07972869325657	7.51714369072717
Si	8.12759329517915	-7.12308427300011	2.28667951199424
Si	-2.13088298128736	-2.97503243381042	7.81888734804668
Si	8.19890582905157	-4.41572794959622	3.55227475839613
Si	-4.81064528961635	-2.81304619727532	6.58258987328463
Si	4.52867882858772	-0.89376575233093	3.37787147759341
Si	-8.08465827127746	1.12811320300180	6.87810011938548
Si	5.35796373068943	-3.70493844749974	2.61640234865187
Si	-5.30193609080203	0.07146002504709	7.56781560992184
Si	-0.93926959029671	3.48978384423755	6.44201881157599
Si	3.44388474304402	-6.10095807926482	1.92425763403878
Si	-2.75089419809700	1.78686155073807	8.27047902947873
Si	1.29589251693282	1.32157939510489	6.64226598686494
Si	5.41904777994453	-8.50963613694218	1.64940740208801
Si	-0.51360093629334	-0.38538068334142	8.47247092553480
O	1.81444768810642	4.27370920996365	-4.05374273790407
O	-0.06996445678775	5.26809278973699	-2.47262068081431
O	-0.61062123944937	7.68760519853693	-3.45860541421725
Si	0.70486611064371	3.91809022144430	-2.92188543410197
Si	-1.20342608093360	6.40824470634263	-2.65645925382639
O	1.24547356501335	-1.73103072315912	-4.50195675728147
O	-3.98089786335222	3.94941139756318	-4.55475311681120
O	3.34191103761548	-2.95631735659736	-3.48344048991564
O	-2.44984418508773	5.83278096281212	-3.51247128902863
O	-3.80013156840884	4.17082582279685	-1.92548669511410
O	1.34068426641590	-4.41314183762692	-4.41567649495894
O	-4.66659664502186	1.77674442179985	-5.92015611077682
O	4.72056337040181	-3.30603621921513	-1.25842771585036
O	-2.55416329271331	1.71501890168925	-4.38618206358896

O	-3.17092485290140	-0.36819123562715	-6.53610803312261
O	-1.11445677310459	-1.80331109766135	-5.65924920426780
O	-0.33434312286905	0.32623023375534	-4.22062544005002
O	-0.35440931262516	2.85577662038350	-3.51360222258250
O	1.78532393870654	6.93015539401975	-4.27393718098571
Si	-3.83686097728286	5.00410228208877	-3.32033739118788
Si	2.19150690678605	-3.04176412968329	-4.64119249940695
Si	-4.05015277537105	2.32369087645305	-4.53002217297553
Si	4.00932460204152	-4.07344928662293	-2.51044903293298
Si	-4.32533472144341	0.66776692663331	-7.06979050496625
Si	-0.30874698920304	-1.29373161205497	-4.34699819371856
Si	-1.21796754028057	1.48387031768991	-3.51174491248759
Si	2.55873914360364	5.56895203205450	-4.71187060414903
Si	0.62755629293072	8.05322312171568	-4.45479693024349
O	-2.79734608681012	-5.59339828939298	-7.11672415753949
O	-4.51971950723126	-4.33517690892775	-5.53953114346685
O	-2.91404128999181	-2.93035070023921	-7.22623534592307
O	-2.81850461199354	0.13371125418692	0.17055525538297
O	-2.47925529667595	2.77292605407220	-0.14527925041923
O	-1.64227420895206	1.03032665390313	-2.01364752317333
O	-5.13350436282795	2.82595599030743	-0.02040424354233
Si	-3.76647897936492	-4.29470187390672	-6.97862535792222
Si	-1.85605762763853	1.29701418261281	-0.43401383921540
Si	-3.80875383230287	3.68307268962954	-0.38559612376207
O	-5.01249006627735	1.90924861174614	-3.29362719741135
O	2.81815541012820	-5.04969116689396	-1.98120713907194
O	-5.61653303816759	-2.51568921650994	2.81778184605346
O	-7.38482804525545	-1.78950770054787	4.67624581336066
O	-0.25303589150079	-6.29320197488852	-3.40031257538308
O	-8.01220914023281	0.19492234899590	3.00977262843968
O	-0.39032419800303	-5.57370782823129	-5.96363758321318
O	-0.95775962943078	-1.92896831580030	-3.01187668460553
O	-3.48229282294913	-6.50227362578276	0.73770031512721
O	-0.97856364450948	-4.26176863974336	-1.84359355311560
O	-6.58699296210130	1.03653039491465	-1.38321178419141
O	0.62690091764744	-6.07691684798243	-0.87183461333527
O	-8.33902762064791	-0.63520322140731	0.49377127303023
O	-7.37688237167912	1.79286881632518	1.01493348146535
O	-1.87150276376865	-6.74749963777346	-1.40202147857044
O	-6.53061846120196	-0.21138166438134	-3.70266299080659
O	2.70742514714732	-5.41958159399217	0.64042393895076
O	-9.07386982020151	-2.65465838251799	-1.04075442965982
O	-4.44662445657684	-0.38558156640574	-2.08705438416685
O	-7.03545703570593	-2.95694361818818	0.62104823678684
O	-8.03552875975857	-2.38412381727162	-3.48259235725493
O	-5.38036867042555	-2.61149467500100	-3.74412712868871
O	-7.26645195871472	-4.36954753427910	-1.88635916420815
O	-5.00714173790488	-5.15418581454014	-3.07669297791396
O	-2.89456105925079	-3.63431129671359	-3.60415458849289
O	-5.68990672471178	-6.15690852219060	-0.72054802378696
O	-2.79080837650188	-2.50739550954472	-1.17754506636370
O	-4.76286746013807	-4.21700951694388	0.92068014280570
O	-4.72230736569671	-1.54467360074101	0.64231232867379
Si	-6.48081623759672	-3.01257412254249	4.10081887760929
Si	0.60006119746886	-5.83040905758197	-4.70756136532846
Si	-8.49615754470389	-0.65243936311543	4.31297905906725
Si	-1.24459566962077	-6.06942298992572	-7.26046110044730
Si	-6.63985939630129	2.29145672438047	-0.35469158125374

Si	-2.44019357544660	-7.48655531672127	-0.05700943473382
Si	-0.62433797181587	-5.83147728682384	-1.88873547344297
Si	-8.42646183531824	0.64782450217726	1.49531176368435
Si	-5.61632995055297	0.56200166520806	-2.60186839853837
Si	2.24989949645918	-6.02023277930081	-0.81419075700039
Si	-8.48901936054684	-2.24823010775816	0.41854313997514
Si	-6.70238803411884	-1.75263699363830	-4.17431986693658
Si	-8.52028630148362	-3.44383105517394	-2.35571048657485
Si	-4.44540991062004	-3.91970450663628	-3.97583612105670
Si	-6.26045915859331	-5.61826443356913	-2.14687650809305
Si	-1.93857235817616	-3.07359205180218	-2.39743211842792
Si	-4.96044582552261	-5.82576224484004	0.69867353975839
Al	-3.69819628984066	-1.12517675117686	-0.74385880737871
Si	-5.53643657335543	-2.82056311305295	1.21981417688788
Si	-2.11364416789518	-1.55040573367277	-6.91167819126244
H	11.67374700163264	-5.53703599828466	-0.03807399829122
H	9.19348300119891	-8.10585799970070	2.36346300096816
H	-3.88155000284984	1.39981100028375	-8.25725800164973
H	-5.54661900027315	-0.07985099891684	-7.37196300059718
H	-6.93206199768435	-1.73335099705178	-5.60666699241235
H	-9.61083500026432	-4.23371599945371	-2.91527899760164
H	-4.80806199726635	-4.38616400096029	-7.98466800000951
H	-1.25558100073346	-7.52827099914588	-7.35483799994677
H	-8.46603499972682	2.53218799685643	6.77163500167724
H	-4.20950399980320	6.50518799953169	2.49497299980766
H	-1.81143199982633	9.00591899690888	0.20164700252341
H	0.67612800185829	11.32783100330101	-2.29765500153444
H	-4.95336999965396	5.93157199766839	-3.34598500088122
H	-7.43212799840141	3.36467299858895	-0.94325499777719
H	-9.81433500355925	1.14100200126591	1.45030699974440
H	-3.26082299717418	2.50672899826654	9.43327000275023
H	-0.74504099834367	4.85494999699797	6.90563300085819
H	1.17176499822778	6.84606699790544	4.60638300164192
H	0.01383699481213	-0.85268899616625	9.75320598689970
H	2.65630398182173	1.61378699587795	7.09828799492101
H	4.53076900356213	3.47379799812806	4.86924400112842
H	1.53720799984822	-6.93798299914092	-4.97365699821897
H	4.63541500131961	9.21535600165356	-0.64625199813043
H	-8.67488799768997	0.53574600251129	8.08886499603046
H	-5.67875099730934	-0.35844199797570	8.92691799826833
H	-5.40516100157014	-3.45826600382855	7.74597000630542
H	-2.86007300182227	-3.03986699652329	9.10083200009531
H	-1.22700999282781	-4.11162700101369	7.70400899726211
H	2.57413299860858	5.42807899811119	-6.16363199983833
H	0.22482900164355	8.10275900035076	-5.85603199892276
H	2.79507599645269	11.25689300010149	-3.42192599883697
H	6.06067100100282	7.26917500010599	-3.76691099950552
H	10.01064999870621	4.23779399893785	-2.76113100014849
H	9.87926499758001	0.42371400043550	-3.59460900111852
H	11.16639499838743	0.78744100172610	-1.60472499963877
H	9.89847199758846	3.90341200108726	-0.38688400227759
H	-1.32399899944233	-1.19594399931947	-8.10330099957430
H	-0.61662900216157	-5.49814399736495	-8.46011599949264
H	2.84818300018148	-3.04332200121205	-5.95038399923634
H	-7.31517899247374	-4.15812799184290	3.75060700394275
H	-9.78250000294109	-1.30915800215423	4.07996999761230
H	-9.40319699824965	-2.75383200072836	1.45013699961842
H	-5.76898899951400	-6.41021299996419	1.77986100180108

H	-3.08084503467500	-8.75083001464955	-0.46797103053276
H	-7.02180600330140	-6.69287800192359	-2.78419799924229
H	2.78358999925680	-7.36886299820331	-0.99247300119699
H	4.89691599854841	-9.57737999970490	2.49409399982990
H	5.82084499943157	-9.04713700039244	0.34374500138754
H	-1.34437696050930	-7.77605497549181	0.87467403880600
H	10.66950899831076	-2.02029700031247	2.56380700021006
H	8.82314099935666	-3.95574100180081	4.78387700068534
H	11.59079999845566	-3.97453399981163	-1.85888000256563
H	5.33815600278843	-0.46451100218868	4.51995500132570
H	7.15219500283785	1.34004700233879	2.37234699811438
H	7.32251199879287	6.57841100200713	-0.51330699853121
H	5.00684300262260	-4.86484799874971	-3.24385800208774
H	7.90214800003260	-7.00114299756732	-1.50659200233637
H	2.44258300478168	-6.49974701199431	2.91143199775023
Ga	-3.74060917636608	-0.21082029028154	2.09350436027209
H	-4.84411283847079	0.94076463575232	2.06952470243492
C	0.10340222742574	-2.74881240674193	4.22023827885218
H	0.13159622228616	-3.34107077472337	5.13312381146023
C	-0.88379955963092	-1.70364662937836	4.08718692867838
H	-0.59649883959682	-0.85041885528431	3.45510548608858
H	-1.31750305076498	-1.35315987690605	5.02684696375245
C	0.84176494262404	-3.21291189708144	3.02522001521794
C	-0.17092303168631	-3.93612888385248	2.09102764654013
H	1.66950215445680	-3.87714827852728	3.29488541091551
H	1.25415976335522	-2.35521015757889	2.47530419459679
H	0.28688239241134	-4.16910453053331	1.12260266273002
H	-0.46910339888331	-4.88442437105687	2.55675115110550
C	-1.90801829951494	-2.54396026555437	3.28501914801743
C	-1.38485270731799	-3.02165282990484	1.94176745055794
H	-2.72268168219120	-1.69113193669988	3.05630903411127
H	-2.38794716018109	-3.32832129607940	3.88081521696192
H	-2.17916577506899	-3.53654204326502	1.38931264459527
H	-1.10679079006941	-2.14069199077044	1.34168191003563

TS9-GaH 2

Electronic energy (r^{2}SCAN-3c): -30773.268863623554 E_h
Electronic energy (ωB97X-D4rev/def2-mTZVPP): -30781.626542935832 E_h
G_corr (673.15 K, 1 atm): 0.49391 E_h
Number of atoms: 258

Cartesian coordinates (Å)

O	1.51456700139130	3.09672511492095	1.08110307141225
O	-1.95092639521935	6.76240690568565	1.42530747921815
O	5.25890065068269	2.08153864103387	0.81766189580787
O	3.64354684107333	3.90341557841016	-0.18079614583166
O	6.12491345683078	4.56514502426821	0.41332197310448
O	1.45033994798498	3.31172573845624	-1.59781463873500
O	1.45150064468633	5.44121366473502	-0.09098013615672
O	-1.67198937528076	6.88937519593139	-1.18808201819842
O	0.31308387535932	7.73920421114299	0.45689528354325
O	2.91472275881349	7.63191247032824	-0.00478502771681
Si	4.86376106263401	3.65082331379102	0.86520516543640
Si	2.02624244991085	3.93429992820725	-0.21426091156125
Si	-1.29665013692275	7.63256398225135	0.21389143550042
Si	1.66361869634347	6.86131588140438	0.65852439620150

O	8.94248497903838	-3.82750369638687	2.22480980136596
O	2.01545243144863	6.62756606348888	2.22985637924806
O	7.14588785424494	-2.27768901241426	-0.91662604242305
O	2.49877654712117	4.73774712304643	4.07716751726442
O	6.74973824781438	-4.90316935009879	-0.76736652607115
O	0.03810752779300	5.13873822309623	3.15296924374671
O	5.84934918697809	-3.26737249460718	1.13968838944790
O	4.45565553467051	4.10691676559676	2.37689696159862
O	9.40160741609015	-5.01222997544189	-1.13073068948956
O	5.15978909391138	-0.25426877480817	2.01701068493841
O	7.73753506917648	4.38640233387627	-1.70729579808050
O	10.69820038138780	-3.18593614459975	0.34610902189017
O	5.42570691366192	5.64485290946437	-1.90456897134143
O	4.12388206947690	5.70286797164760	-4.20169840865963
O	8.76317224464570	-1.45255198462542	1.04590793999335
O	3.87939063811210	7.72350979074819	-2.51932479937733
O	1.23476549687046	9.55246590501132	-4.03778921274001
O	8.98016614064999	2.07270495378815	-1.88598982010785
O	6.94579091265925	0.19632697097764	0.10134838883529
O	2.36083378983392	9.69947453187451	-1.59099056382981
O	8.99810717149922	-0.54563051384536	-1.44358850849644
Si	1.43712362119574	5.86266069060349	3.54801745574097
Si	6.11710785307058	-3.43866672109880	-0.44587062033042
Si	3.65422139227677	3.64422931577817	3.71752763192063
Si	8.08011550699341	-5.83628353539860	-0.65720946274280
Si	10.87588499649615	-4.45909031752666	-0.67215195087566
Si	6.67707366526616	5.32955999052848	-0.91728119264788
Si	9.79209042775371	-2.60512749664274	1.56625842749277
Si	4.91830686797793	6.61211160010112	-3.11022196950432
Si	9.20569607685550	3.67481487634345	-1.66968821704599
Si	6.17041503453934	0.85557511490149	1.37427759540321
Si	1.52533450431997	10.52305706718856	-2.74164441412790
Si	9.80029297168474	0.68462422624197	-2.15422307630024
Si	7.95497915143187	-1.01060486832968	-0.30123412587812
Si	3.47644024589486	8.58344060829586	-1.19701114823107
O	2.96774553471977	-0.46707663234748	3.59171797549822
O	1.02544049614950	0.89102592023835	2.43166609322898
O	1.25790709586295	0.92805247301288	5.05752813320871
O	3.05425945865645	2.15574474468315	3.48086010296173
O	-0.26321117342456	1.34912417253578	0.19962048870208
O	-0.82905913443896	2.78033602413605	2.33275312364591
O	-3.69792248800211	4.90922425145010	0.59924895229170
O	-2.54103916077734	4.68989244040003	2.99047574280602
O	-1.14416793368868	3.41245014951750	4.84217707262328
Si	2.08014632844049	0.87773954174078	3.66227266174095
Si	0.36093909278367	2.05022296011266	1.51571418587889
Si	-3.13188877810526	5.73216208910043	1.87939531856872
Si	-1.11271726187753	4.01569204055593	3.34618455951992
O	8.28815796613518	-6.04106526197312	3.49464800320240
O	-3.20082214105112	-3.08233019844487	6.57671944682664
O	6.68073149870411	-7.84390377098036	2.44297877090253
O	-1.30858878350739	-1.57523670318404	7.68348738836722
O	8.26090916277137	-6.34288549630412	0.87113899753887
O	-5.42251489054748	-3.45822814030493	5.21882312120713
O	6.64636125980578	-3.92709897383201	3.57595240436847
O	-5.11500976314913	-1.21959131143154	6.59689143805656
O	-8.69469960434935	0.37668826900954	5.56173664536934
O	4.46898220130820	-2.51203026478833	3.25473582095085

O	-6.45364475090670	1.03693731377917	6.94407167402084
O	4.48218285416615	-5.06116283164462	2.60785036669597
O	-3.89317507530547	0.86759173642128	7.58164108294893
O	-2.25207711265046	2.85154588889388	7.14638794731027
O	0.40185084972132	2.65714477471612	6.88139974724269
O	4.25539506463292	-7.39711276050135	1.37327194514289
O	-1.50803263057936	0.86243863556076	8.77526464140393
O	0.70965125672868	0.07201681880256	7.49582153638003
Si	8.12734674285366	-7.12306069440546	2.28624477837855
Si	-2.13530698509103	-2.97825751311632	7.81391615218166
Si	8.19688059151079	-4.41497030072610	3.55261860366682
Si	-4.81061549101674	-2.81643616815269	6.57454431439074
Si	4.52413907302280	-0.89345429451239	3.37513995015488
Si	-8.08966766587744	1.12915149827101	6.87521967384978
Si	5.35651674117174	-3.70352362449699	2.61758097972036
Si	-5.30339152334977	0.06951990602246	7.56633348952559
Si	-0.93171842388591	3.48619869596263	6.44927260589904
Si	3.44142138922232	-6.10007630310680	1.92432185567851
Si	-2.75194691596163	1.78733379188384	8.26880008138755
Si	1.29848618567749	1.32254242690994	6.63921537675213
Si	5.41650813444762	-8.50857753707960	1.64911876927913
Si	-0.51551497416771	-0.38954015915021	8.46551207949293
O	1.87031036412245	4.29396299487590	-4.02978257888016
O	-0.07251303816286	5.26379769806275	-2.50471839737182
O	-0.56635069118358	7.71324234381310	-3.44379075267272
Si	0.73777870816197	3.92938181361456	-2.93116171007438
Si	-1.18302032692693	6.42808690542293	-2.66451341912514
O	1.23393334795132	-1.75889300434002	-4.47842381853546
O	-3.95772614151346	3.95891132221191	-4.53887924588579
O	3.34661741635184	-2.96132246835228	-3.48444411551709
O	-2.44843745890709	5.87400947809701	-3.50908866438176
O	-3.75168016995946	4.18147594138298	-1.90463482831992
O	1.34937597436367	-4.43057427619110	-4.41135032000678
O	-4.64814836395272	1.78958416547307	-5.91985968578784
O	4.72316042017363	-3.30463897721831	-1.25659561408433
O	-2.50497613764949	1.74580175702846	-4.43353147592783
O	-3.14878955794529	-0.34772762959668	-6.54699549625882
O	-1.11062339175657	-1.82584019770138	-5.67519370574893
O	-0.30623721231305	0.32023430480077	-4.28823708085416
O	-0.27848685768932	2.85554268311865	-3.57407698461029
O	1.80615248900816	6.94688889802014	-4.31768732470683
Si	-3.81828270537704	5.01515419718010	-3.30494079446432
Si	2.19345328733953	-3.05664110766020	-4.63758359138080
Si	-4.01424802846214	2.33204555823797	-4.53502006311935
Si	4.01257875717970	-4.07529875610570	-2.50660438214057
Si	-4.34315967626972	0.65521038059510	-7.05861220051066
Si	-0.31603354360231	-1.30408970378813	-4.36244891007735
Si	-1.15927180756246	1.49867559015160	-3.58323068183120
Si	2.58651927707236	5.58108862031255	-4.72697664111294
Si	0.66056946616559	8.08725447578712	-4.45794577419302
O	-2.79295311890221	-5.58500569991235	-7.12014915779954
O	-4.54989985998998	-4.36204108154730	-5.55515952452997
O	-2.94304212631296	-2.91784976353983	-7.21909353094832
O	-2.43805003712789	-0.08724117318828	0.11222280492576
O	-2.53864299185912	2.62535745250564	-0.15440702218935
O	-1.55805120294146	1.05120148966854	-2.07804857307592
O	-5.14830943045548	2.80214121236983	-0.02858186058367
Si	-3.77400052957006	-4.29718442265715	-6.98099904770955

Si	-1.72414487259213	1.27044116542590	-0.49880401964337
Si	-3.80261068629251	3.62683307956116	-0.38501548948896
O	-4.93606799177855	1.89931708635516	-3.27517748891253
O	2.82175258906297	-5.04934951199626	-1.97591612269113
O	-5.80242907045023	-2.43430801165519	2.79643867213644
O	-7.43373734158319	-1.80032924557077	4.76189322618649
O	-0.25240419613202	-6.31970873881305	-3.40465386081519
O	-8.00588048953502	0.15549082903996	3.00047838345300
O	-0.39011427562865	-5.57145782962325	-5.96339713061192
O	-1.01589492973471	-1.89359661798084	-3.02729191814769
O	-3.48773721919188	-6.49382118975432	0.73541846173002
O	-0.95340935146158	-4.25444847793025	-1.90811819618400
O	-6.62228082607003	1.05842904696508	-1.43039090866705
O	0.62328646948856	-6.06984914543525	-0.87752407033559
O	-8.39661713238890	-0.62521092815888	0.46543493498014
O	-7.35652446537556	1.75729430086896	1.01657654312319
O	-1.87785618969146	-6.72791575984711	-1.39654062393598
O	-6.41485332063442	-0.22889069339009	-3.70325487117533
O	2.69383527309285	-5.42056157808271	0.64570396918887
O	-9.10746982322178	-2.67696931483940	-1.04229126799165
O	-4.39871497767399	-0.30029918898705	-1.92686482024397
O	-6.99576656494246	-2.87159853594561	0.50328818145625
O	-8.01209747985228	-2.35065144692152	-3.45436179125998
O	-5.35490265052375	-2.62879652618859	-3.74484401267379
O	-7.26523028575384	-4.36833640925119	-1.90804309614298
O	-4.99911640339960	-5.17283763367974	-3.07892695438726
O	-2.87729393638791	-3.67575617408900	-3.67125594329027
O	-5.69165897720775	-6.14319937000636	-0.71635953057669
O	-2.85821927235318	-2.59593144520861	-1.23512918229493
O	-4.81529248306726	-4.22567053667320	0.98868090699008
O	-4.57107497093970	-1.60258243435822	0.75191498166460
Si	-6.53008315503182	-2.99510695139061	4.12278638052261
Si	0.59752284202848	-5.84035637109177	-4.70767911009307
Si	-8.50616131489547	-0.65200946092117	4.32623285004123
Si	-1.24364643512801	-6.07084173234399	-7.25905629538769
Si	-6.65909238947312	2.27965722976634	-0.36329419304389
Si	-2.44497461254182	-7.47901237981148	-0.05434855520212
Si	-0.62404660977910	-5.83248118167097	-1.90033074956383
Si	-8.43352658264183	0.63202482838591	1.49659662394991
Si	-5.56706693109865	0.56974108219222	-2.56991052523164
Si	2.24627309095926	-6.01949017563140	-0.81218379028501
Si	-8.49448154067103	-2.24290972672940	0.39920982192051
Si	-6.67194412774915	-1.75939401994463	-4.17026979521749
Si	-8.52066090995899	-3.43806630398183	-2.35887907751413
Si	-4.44253819319145	-3.95078934325042	-3.99307089815376
Si	-6.25934189661585	-5.61971117275664	-2.14667202750742
Si	-1.95683966358582	-3.09583899551353	-2.44824346088174
Si	-4.96963870529153	-5.82112783913320	0.71047321072729
Al	-3.65741849552133	-1.18717506864606	-0.67216131232423
Si	-5.54568105367497	-2.79368651207251	1.23128679126311
Si	-2.11467086293695	-1.55237277170690	-6.91943344213661
H	11.67354000542654	-5.53749700658839	-0.03812999883775
H	9.19325800785835	-8.10610400127470	2.36365699164997
H	-3.96156899572222	1.36419900717944	-8.28506100792220
H	-5.56398600287855	-0.12270100581419	-7.29597599927632
H	-6.93632900080769	-1.73777200028510	-5.60525601332512
H	-9.61070200235782	-4.23261600689859	-2.91602400362700
H	-4.80937899286698	-4.38478700328085	-7.98416802664936

H	-1.26201399370256	-7.53013899665437	-7.34713302628881
H	-8.46716135082970	2.53175414100927	6.77435929809959
H	-4.20618400421495	6.50892100992489	2.49273000250352
H	-1.81213400567532	9.00646801251058	0.20177799977211
H	0.23145505173229	10.95012003469765	-2.10809602411479
H	-4.94421500297366	5.93630901537854	-3.34769399954156
H	-7.43440299871108	3.36541400704333	-0.94274900316159
H	-9.81224999662228	1.14342200307830	1.44886400014132
H	-3.26051299790122	2.50761899510447	9.43266799005775
H	-0.74472599726435	4.85510199806791	6.90777200210405
H	1.17261700628368	6.84873800811668	4.60176299100533
H	0.01371300692745	-0.85239399348684	9.75320305154206
H	2.65644499487792	1.61373199572658	7.09816899309436
H	4.53016699780804	3.47572500807208	4.86954699827276
H	1.53638200595453	-6.93834200352665	-4.97336599793707
H	4.68077902674672	9.17388300869582	-0.65420601394822
H	-8.67443696541928	0.53296804424597	8.08822292894988
H	-5.67876699472913	-0.35798100538454	8.92693201083493
H	-5.40538800156161	-3.45794000566727	7.74582399633147
H	-2.86030600709494	-3.03941598254743	9.10082100224688
H	-1.22727298360156	-4.11134501501064	7.70409198016925
H	2.59107100396820	5.38865100434752	-6.17172401710436
H	0.22684599771358	8.11439300353931	-5.85590800871639
H	2.35648383194826	11.66568558073940	-3.24752285597681
H	6.05893606058723	7.26486301665985	-3.76938203746344
H	10.01162899783907	4.23690500206674	-2.76123799923550
H	9.87912999907179	0.42295200088988	-3.59486201049077
H	11.16666300496081	0.78647299874719	-1.60521600234840
H	9.89955900187037	3.90245199948788	-0.38701600071269
H	-1.31531298923855	-1.20646999024861	-8.10616501161129
H	-0.61432195086067	-5.50729798611648	-8.46106605060642
H	2.84834100279858	-3.04402000318612	-5.95043800600926
H	-7.31528199494430	-4.15742498451907	3.75037499914787
H	-9.78237296907020	-1.30797897255169	4.07942699679101
H	-9.40254698318981	-2.75213298930528	1.45267098024734
H	-5.76896399239853	-6.40939898822656	1.78048399258459
H	-3.08137200609347	-8.75069599879511	-0.46757200477512
H	-7.02203599234332	-6.69280399751455	-2.78388199844396
H	2.78306899137276	-7.36900598840886	-0.99232699002010
H	4.89569300339843	-9.57645900264801	2.49579400975624
H	5.81992198664007	-9.04892398985833	0.34487200563385
H	-1.34472299376417	-7.77598298772860	0.87489000907895
H	10.66941403685565	-2.02071797769892	2.56381701158774
H	8.82291801069104	-3.95596099961638	4.78390497438987
H	11.59057501550584	-3.97495100377164	-1.85889602368401
H	5.33835299741699	-0.46465099843611	4.51988999423893
H	7.15208500292055	1.34023100719816	2.37194499757734
H	7.32230800659593	6.57802299858416	-0.51391500228438
H	5.00675700165857	-4.86530400370767	-3.24361800294021
H	7.90182200188486	-7.00149500086619	-1.50647500142262
H	2.44258807300831	-6.50026703292798	2.91141095908497
Ga	-2.97875800186964	-1.56511351927324	2.72877976792154
H	-3.47723438664947	-0.21858875599651	3.29673568059366
C	-0.73877774899922	-1.46163435096571	3.40985001388801
H	-0.74020989859588	-0.51320434127395	3.94288143132017
C	-0.49568790049708	-2.59826013173206	4.08469705304902
H	-3.25778470910953	-3.04696509459026	3.00241703366711
H	-0.30392136081152	-2.53236079368437	5.15510157394291

C	-0.98231653947808	-1.44623977769025	1.92494298982526
C	-0.37957049867375	-2.68255762616777	1.24550871262692
H	-0.45017127491318	-0.56271699628947	1.56715731552348
H	-1.94776233175820	-0.69421136187456	1.01993838441040
H	-0.68135441529099	-2.73936704925080	0.19698922355931
H	0.71390763222015	-2.55526052125528	1.25959933189581
C	-0.37657114013502	-3.94929456644760	3.45002182350464
C	-0.73305325950862	-3.98899734518737	1.95511783963046
H	-0.99148045147029	-4.66815901804153	4.00919730318249
H	0.66360279680053	-4.27910726497713	3.60489429906570
H	-0.19879951191704	-4.81628159390024	1.47460966430394
H	-1.80032091030479	-4.19499833381805	1.83007957655637

TS10-GaH 2

Electronic energy (r^{2}SCAN-3c): -30773.284389211702 E_h
Electronic energy (ωB97X-D4rev/def2-mTZVPP): -30781.644484152202 E_h
G_{corr} (673.15 K, 1 atm): 0.49551 E_h
Number of atoms: 258

Cartesian coordinates (Å)

O	1.50238319060985	2.97886316929754	1.05395343124203
O	-1.95013552717086	6.75691441598064	1.40337312195979
O	5.26604714161834	2.07553942709557	0.80471990306575
O	3.62414535549481	3.88588138730571	-0.16983310873988
O	6.10630402688580	4.56961703125892	0.41306114629747
O	1.44041354428399	3.29745550514729	-1.60066882300138
O	1.41380909380231	5.37875871659820	-0.02218267037302
O	-1.70630108854167	6.89509463297305	-1.21263684008920
O	0.31054425962048	7.71882811855409	0.40447766022648
O	2.90601303322880	7.57389596335236	-0.04971365155092
Si	4.85570904557001	3.63941898227947	0.86474235100221
Si	2.00621768471119	3.88252920625097	-0.19580717805870
Si	-1.30347371751001	7.62904263393043	0.18919044588088
Si	1.65173987897594	6.83419870056503	0.64757014569118
O	8.95003089904133	-3.83351488807449	2.22519369196702
O	1.99535754243393	6.66048751348065	2.22942310303205
O	7.14548602994407	-2.28061562053969	-0.92077802132227
O	2.50774220889358	4.75659883547839	4.06857861168926
O	6.74898183196850	-4.90504079419395	-0.77015202955545
O	0.04435225129550	5.12465527821800	3.13530469715556
O	5.85329369608899	-3.26887718222469	1.13886326893374
O	4.44977958251019	4.07907065247658	2.38075247314971
O	9.40127891385239	-5.01172323601444	-1.12885186630764
O	5.16060817566362	-0.25329605011887	2.01743798070207
O	7.73294296580629	4.38407416518500	-1.70053057243301
O	10.70272036563401	-3.18590259233837	0.34607986579446
O	5.42071639247355	5.64945701396894	-1.90369697666949
O	4.12425573368507	5.70452379920211	-4.20404863657065
O	8.76332597860442	-1.45924502803368	1.04417519329464
O	3.88987953149952	7.74043754895371	-2.53721573558355
O	1.23017735511020	9.54769068202357	-4.02954471448566
O	8.97985301369284	2.07252766118404	-1.88632785222572
O	6.95003597481362	0.19171039200918	0.10115971851722
O	2.35997413341179	9.69515056729285	-1.58361178309515
O	9.00003466948119	-0.54808052783810	-1.44435976336761
Si	1.43319733734816	5.86864458673273	3.54110530832306

Si	6.11636670651979	-3.44089685348676	-0.44667850364412
Si	3.63667392826775	3.63102765695981	3.72014440155939
Si	8.07955860091898	-5.83684897453205	-0.65653083711413
Si	10.87626604829408	-4.45941766659207	-0.67209157984669
Si	6.67088099848851	5.32998721270854	-0.91450441370615
Si	9.79462483826711	-2.60846555089457	1.56637926779022
Si	4.91988641770629	6.61430941650077	-3.11382541101542
Si	9.20351335142898	3.67481544209998	-1.66890497592136
Si	6.17353444320678	0.85153742776092	1.37251276362536
Si	1.52694680609238	10.52070729234404	-2.73632347333089
Si	9.80012831640057	0.68386252770564	-2.15394741967434
Si	7.95649342307169	-1.01616134996082	-0.30303843180350
Si	3.47593760335984	8.57325714737651	-1.20129049436169
O	2.97663797463396	-0.46620683309777	3.60021340675152
O	0.97905432257314	0.79712021153571	2.43945811713229
O	1.24372219665350	0.89711833020162	5.06324864045293
O	2.98154394179473	2.16582221329439	3.47626016288827
O	-0.34466569773163	1.27805290856046	0.21246864575259
O	-0.82012448224694	2.73944449715107	2.34680479525290
O	-3.79064732157357	4.97944814678055	0.58243035132229
O	-2.53645213563742	4.65980282722122	2.92353053228772
O	-1.17326133995943	3.43600454336000	4.83371260221108
Si	2.05109953901286	0.85013794594521	3.65966503879801
Si	0.33186879755666	1.95895436904346	1.51699215845624
Si	-3.14110983148325	5.74281293441959	1.86226485293389
Si	-1.10907472807375	4.00183677053612	3.32357330781486
O	8.28910349131455	-6.04317083984578	3.49602292576072
O	-3.20557117984324	-3.05728448037080	6.58600500440347
O	6.68161128477243	-7.84674125120470	2.44398233669407
O	-1.31879717245211	-1.55947533591112	7.70566628783822
O	8.25889854506695	-6.34318564758922	0.87186504833052
O	-5.44630915719359	-3.50815242392797	5.28381403710461
O	6.65154670105012	-3.92541128710234	3.57415826261591
O	-5.16757176144516	-1.22301680943136	6.60992802815529
O	-8.63453334625232	0.31748360701565	5.57859023026220
O	4.47528325939267	-2.51327009913511	3.25448036972147
O	-6.43699406822211	1.07169550903501	6.97080834767503
O	4.49169759907092	-5.06630708519762	2.60910803469686
O	-3.87261582517487	0.84365991149376	7.58371735996357
O	-2.25531433634914	2.84961157134446	7.14036449839502
O	0.39929548916817	2.65781260342561	6.85486958974015
O	4.25497549023926	-7.39907097202984	1.37540356317032
O	-1.49365276204282	0.88217487173739	8.78612847156356
O	0.71611598411757	0.07903964584353	7.51238319041709
Si	8.12672621960064	-7.12407972123796	2.28697501621136
Si	-2.12961209787576	-2.96936356718361	7.81613059440860
Si	8.19969137867897	-4.41691447476095	3.55255588520113
Si	-4.81279383009544	-2.80902650423434	6.59864430495070
Si	4.52764606827301	-0.89589468524927	3.37602744850929
Si	-8.07359918737990	1.12720772653230	6.88053891137158
Si	5.35970201765377	-3.70546849228252	2.61725063221296
Si	-5.29942231350363	0.07574208728160	7.57482932076500
Si	-0.93768713246287	3.48902927289778	6.43918942546556
Si	3.44369125246211	-6.09919470522815	1.92688724719307
Si	-2.74577314701156	1.78713907794987	8.26955786927407
Si	1.29701426841218	1.31795454766568	6.63655034308005
Si	5.41620422048193	-8.50890794158084	1.64949194566114
Si	-0.51360688016720	-0.38026258164887	8.47491713506135

O	1.87237547992618	4.29610283800617	-4.02605707726928
O	-0.05786722962787	5.27864959769024	-2.48974218478230
O	-0.58319465231159	7.70532390324022	-3.46195044472663
Si	0.74000099917792	3.93840406922939	-2.92624924376035
Si	-1.18947700791331	6.41997714527734	-2.67606811008041
O	1.25528444193025	-1.73794231447479	-4.51388436755142
O	-3.99471318271280	3.93757696838469	-4.54467302774206
O	3.34437041539365	-2.96064660725190	-3.48165200292514
O	-2.43422673164478	5.82559196192919	-3.52786628939232
O	-3.84486055243511	4.20686052266673	-1.92449607920501
O	1.34121578597424	-4.41709297990500	-4.41818195097296
O	-4.66589408360878	1.77140785687992	-5.91508798994610
O	4.72151687951746	-3.30765973213775	-1.25608843599019
O	-2.47742708621265	1.76706647828250	-4.49190733318060
O	-3.15215447910468	-0.35149944776235	-6.54997849386549
O	-1.11788207283384	-1.81232106320573	-5.66133172207979
O	-0.29084163772545	0.33274551295853	-4.27772862630731
O	-0.28784400721057	2.87649412887841	-3.57196834359042
O	1.80246770761678	6.94540163304087	-4.31477483463602
Si	-3.83265488525724	5.01321197616014	-3.33270517027916
Si	2.19902399584824	-3.05122816106652	-4.64113449513693
Si	-4.00606860520890	2.30969477748788	-4.54293394314721
Si	4.01010444416818	-4.07775556000904	-2.50534826898902
Si	-4.34534674761923	0.65010190293976	-7.06230845092075
Si	-0.29315736366046	-1.29297181557954	-4.36507644089653
Si	-1.15770234239596	1.51525572053128	-3.60324012448673
Si	2.58693631904529	5.58253435038044	-4.72688688154133
Si	0.65690923898473	8.08527349387167	-4.45848005422007
O	-2.79445275760073	-5.58815715310458	-7.13827073212388
O	-4.47595552446671	-4.35264270210172	-5.51550721805745
O	-2.93060743091818	-2.91942675064278	-7.22777414221455
O	-2.76891225151588	0.25177927967160	0.08066516914727
O	-2.42701653011108	2.85258508500673	-0.21292278104716
O	-1.61135850105909	1.08402113459496	-2.09977573843018
O	-5.09517241111594	2.76434730091030	-0.03702226648249
Si	-3.76463132238899	-4.29503340099619	-6.97581237668044
Si	-1.76880123696068	1.40852313651055	-0.53871662797493
Si	-3.81425020154195	3.69595969357785	-0.39867644065370
O	-4.85774085419870	1.82189220951764	-3.25421573309078
O	2.81700517901266	-5.05234494765488	-1.98025334610926
O	-5.50813831955922	-2.72155196957753	2.82684158327103
O	-7.33689427455970	-1.77889385465323	4.58759438662973
O	-0.25574945435997	-6.29077298381612	-3.39470950251962
O	-8.01737382119220	0.20816452725676	2.99382098124450
O	-0.39836572386088	-5.57487069405292	-5.95789824153950
O	-0.94656593352945	-1.89007958955563	-3.01571240512303
O	-3.49779848906327	-6.53137485683803	0.73981738966999
O	-0.94848186760701	-4.24095281350069	-1.85504430500956
O	-6.61326836501978	1.07079790224596	-1.44440041264228
O	0.62613137766610	-6.07729532053930	-0.86682545389091
O	-8.38181588701774	-0.61689851686537	0.46613543568941
O	-7.33757786070306	1.76757791011018	1.00246916619155
O	-1.87869551173227	-6.71852174640020	-1.39331219964894
O	-6.48225360898128	-0.21011649067945	-3.73320973956455
O	2.70811248326850	-5.41809189526592	0.64290090742238
O	-9.06950184054993	-2.67924137200338	-1.02536564109613
O	-4.52731375540393	-0.48210178503783	-1.97225570126886
O	-7.00574560586240	-2.86872966553340	0.63743072321921

O	-8.02262639798352	-2.37103532161120	-3.45304694105780
O	-5.38357681357463	-2.62444365782646	-3.75331426470411
O	-7.25572427790240	-4.37935530674091	-1.87000273272796
O	-4.99248430924588	-5.15579909565645	-3.05234052620175
O	-2.90079992886150	-3.59277507781705	-3.54372999959106
O	-5.71087960689666	-6.24430028529713	-0.73602201317547
O	-2.69138240151417	-2.44381291931377	-1.11190840964325
O	-4.79270235680763	-4.24347196388969	0.75743241607569
O	-4.59431689683717	-1.56756546244577	0.80064334453181
Si	-6.45429411592021	-3.05312414113020	4.10428202342110
Si	0.59659127355533	-5.83346296300181	-4.70357935645190
Si	-8.48019973338125	-0.65714318309391	4.29493482070020
Si	-1.24295003566840	-6.07220958977957	-7.26022910206060
Si	-6.62897809250992	2.28761330760692	-0.37447055442940
Si	-2.43686373221214	-7.48824019546251	-0.06010451005263
Si	-0.61979263441051	-5.81781967420059	-1.88272844302190
Si	-8.42805073770332	0.65502231570029	1.48057348398724
Si	-5.59208344750897	0.52523178975982	-2.58898589432626
Si	2.24933386357659	-6.01968741316842	-0.80786996642128
Si	-8.48886777146095	-2.23402263865957	0.42278706770193
Si	-6.69723891595376	-1.75709727619863	-4.17547636700426
Si	-8.51007437681961	-3.45378413348662	-2.34567764919441
Si	-4.43087530896128	-3.92186836582206	-3.95371055593573
Si	-6.25638205466427	-5.63528606195201	-2.14318966969124
Si	-1.90693460577370	-3.03593732811475	-2.36640323017468
Si	-4.97450142252067	-5.86346536607228	0.66764721227128
Al	-3.68359691512017	-1.13578656527615	-0.64476836851342
Si	-5.49195551762404	-2.85334175097445	1.20756509620484
Si	-2.11085759956336	-1.55108670253071	-6.91742271198711
H	11.67353999802181	-5.53749699537876	-0.03813000151476
H	9.19325800600167	-8.10610399693060	2.36365699766376
H	-3.96156900445649	1.36419900173347	-8.28506097656531
H	-5.56398599859751	-0.12270100290041	-7.29597599957204
H	-6.93632899820305	-1.73777199853700	-5.60525599707239
H	-9.61070198734079	-4.23261599172594	-2.91602399698619
H	-4.80937899309678	-4.38478699664533	-7.98416800188775
H	-1.26201400730841	-7.53013898879179	-7.34713299473540
H	-8.46716099962910	2.53175401634004	6.77435899779753
H	-4.20618399737597	6.50892099461827	2.49273000010045
H	-1.81213399956174	9.00646799682894	0.20177799943572
H	0.23145498499467	10.95011988415168	-2.10809596634155
H	-4.94421498242315	5.93630897785673	-3.34769397590362
H	-7.43440302477755	3.36541402966413	-0.94274901283626
H	-9.81224997879609	1.14342199687885	1.44886400339497
H	-3.26051300248447	2.50761900017243	9.43266799248815
H	-0.74472599787930	4.85510200261042	6.90777200251869
H	1.17261700612435	6.84873799248547	4.60176299270669
H	0.01371299192547	-0.85239400160714	9.75320299513676
H	2.65644500313161	1.61373200307677	7.09816899825019
H	4.53016699556286	3.47572500951429	4.86954699776740
H	1.53638200134098	-6.93834200265082	-4.97336599786080
H	4.68077899628375	9.17388299683224	-0.65420600226264
H	-8.67443699134065	0.53296801336652	8.08822298350047
H	-5.67876700659811	-0.35798099933378	8.92693200743144
H	-5.40538800147288	-3.45793999430147	7.74582397607054
H	-2.86030600081387	-3.03941600222560	9.10082100326909
H	-1.22727300064382	-4.11134500360905	7.70409199946136
H	2.59107099726060	5.38865100113099	-6.17172398640752

H	0.22684599670885	8.11439300189097	-5.85590801551140
H	2.35648400372556	11.66568599009305	-3.24752299968690
H	6.05893599607191	7.26486299851158	-3.76938199626445
H	10.01162899748572	4.23690500242417	-2.76123799882304
H	9.87912999943610	0.42295200138032	-3.59486200013902
H	11.16666299980508	0.78647299924450	-1.60521600254148
H	9.89955900227186	3.90245199976205	-0.38701600083361
H	-1.31531299958039	-1.20646999827872	-8.10616499933999
H	-0.61432199559493	-5.50729800068979	-8.46106600364375
H	2.84834100674104	-3.04402000132707	-5.95043801230733
H	-7.31528194753408	-4.15742495350748	3.75037500696130
H	-9.78237300501794	-1.30797899524910	4.07942700722333
H	-9.40254700373141	-2.75213299919456	1.45267099894375
H	-5.76896401151910	-6.40939900328618	1.78048400481963
H	-3.08137197386728	-8.75069597880634	-0.46757197348222
H	-7.02203600648888	-6.69280401029805	-2.78388200822455
H	2.78306900416499	-7.36900599932698	-0.99232701923082
H	4.89569300865632	-9.57645897706450	2.49579398499159
H	5.81992202228471	-9.04892402355203	0.34487197762605
H	-1.34472300965187	-7.77598304659054	0.87488995240082
H	10.66941400475172	-2.02071799249109	2.56381700030544
H	8.82291799798832	-3.95596099658651	4.78390499792396
H	11.59057499743675	-3.97495100223228	-1.85889599889689
H	5.33835300088255	-0.46465100103707	4.51988899611558
H	7.15208500063827	1.34023100225179	2.37194499639806
H	7.32230800008119	6.57802299706513	-0.51391500194370
H	5.00675699944670	-4.86530399965970	-3.24361800354980
H	7.90182200077218	-7.00149500042883	-1.50647500119415
H	2.44258788714782	-6.50026697178864	2.91141107681613
Ga	-3.41752812145206	-0.95963331707790	2.89036911102160
H	-3.37443106133194	0.57087834041124	2.19279503795720
C	-0.90385689958771	-1.75024446270141	3.78517642100022
H	-0.59749772251271	-0.74217841999267	4.06225523078367
C	-0.54297935122595	-2.77085888282310	4.57144286236271
H	-4.29823720187854	-0.74988821615947	4.14487728313311
H	0.06988080976339	-2.56564416409327	5.44825028238815
C	-1.69104500606131	-1.91172380404093	2.52238034211754
C	-1.74227032927841	-3.37622942140188	2.07597835444315
H	-1.22587573676488	-1.29972889431538	1.73578834879462
H	-3.06547133984395	0.41876106209212	1.16522744534285
H	-2.45751344583070	-3.52547511230083	1.26395254653356
H	-0.75464402470453	-3.64281713383085	1.66759978129505
C	-0.93008324183220	-4.19716800697068	4.30307381734270
C	-2.04474483906345	-4.29346214468014	3.25944494012691
H	-1.24253365857012	-4.68083975260728	5.23812018945435
H	-0.04401021873018	-4.75634266965906	3.96154453628652
H	-2.16458879834037	-5.32892923123682	2.92116185931895
H	-2.99651935117499	-3.99382118171151	3.72225293020850

TS11-GaH 2

Electronic energy (r^2SCAN-3c): -30772.078564391526 E_h
Electronic energy (ωB97X-D4rev/def2-mTZVPP): -30780.422954697802 E_h
G_{corr} (673.15 K, 1 atm): 0.46326 E_h
Number of atoms: 256

Cartesian coordinates (Å)

O	1.48292807288880	2.97392176168951	1.03372707575250
O	-1.94449987440790	6.77280615499123	1.43481820768914
O	5.25725963954468	2.08092247505156	0.81562494868038
O	3.61652012981519	3.89369776402423	-0.15977805409113
O	6.11229074750244	4.56388410453886	0.40356921361388
O	1.44935947689649	3.30610607844723	-1.61457243602622
O	1.40251419634216	5.38293023046048	-0.01907803767969
O	-1.68461202090876	6.89373580665353	-1.18301184852322
O	0.31919650701543	7.73076008029718	0.43679275233146
O	2.90890428544443	7.56680444106502	-0.05996213640422
Si	4.85577974766839	3.64874576247190	0.86477264639572
Si	1.99776528482439	3.88848945761235	-0.20388384918297
Si	-1.29441751040483	7.63472971143316	0.21518518478413
Si	1.65775693229141	6.83625762154530	0.65174558088076
O	8.94215041536426	-3.82721400232118	2.22456176350921
O	2.01958203286105	6.65703260967803	2.22992642213279
O	7.14405967961101	-2.27673700580684	-0.91667536515718
O	2.52186255087097	4.76671480201074	4.07772821115716
O	6.74971473010620	-4.90268811237239	-0.76841967113874
O	0.06399528148865	5.12539323176167	3.13614053871530
O	5.84761864691794	-3.26828949099773	1.13916311263500
O	4.46820182761076	4.10908344436845	2.38040860576829
O	9.40158495916782	-5.01218633908818	-1.13052099953357
O	5.16085290677334	-0.25325212644473	2.01574058708787
O	7.73514031744442	4.38862701310022	-1.70885318752120
O	10.69825976322366	-3.18589269278112	0.34615180866149
O	5.41990808171761	5.65451413131765	-1.90265775128025
O	4.09209660994806	5.67252717709009	-4.17200770738707
O	8.76294400272178	-1.45261049139713	1.04507135934421
O	3.89214860557713	7.74771377691468	-2.54606222417876
O	1.24370036644372	9.54807244606139	-4.03530374743767
O	8.97936371244122	2.07372457001417	-1.88614480338460
O	6.94679113879741	0.19788659919559	0.10024430037287
O	2.35944000502427	9.69568828699650	-1.58280380272046
O	8.99776432848069	-0.54535485142705	-1.44443204570020
Si	1.44708424250807	5.87516639088756	3.54204805490761
Si	6.11614916664290	-3.43867882031922	-0.44609921160471
Si	3.65662148058555	3.65463660468320	3.71639225768909
Si	8.07991024020395	-5.83607271088555	-0.65735773101527
Si	10.87595076871790	-4.45902013003116	-0.67207433079941
Si	6.67278155513149	5.33222745734227	-0.92032319393332
Si	9.79192097094358	-2.60489662407013	1.56608026511650
Si	4.91042375247736	6.60869266545059	-3.11836763790456
Si	9.20373566130026	3.67635063404444	-1.66982724575964
Si	6.17084319360358	0.85697531291865	1.37279648683511
Si	1.52782659380329	10.52028984340203	-2.73805137275160
Si	9.79991510368066	0.68560759723475	-2.15428839746631
Si	7.95473843660334	-1.01017575945639	-0.30191406019367
Si	3.47704839401746	8.57389103611237	-1.20617770283576
O	2.98260601452844	-0.44535159294249	3.61062673295567

O	0.98537854461929	0.83774962268970	2.48566250360050
O	1.27149362716040	0.94692755682064	5.07704566022333
O	3.02089632567056	2.18444507947989	3.45367362166957
O	-0.36289574775099	1.19130291266341	0.30546462783487
O	-0.83860317632807	2.77294954852608	2.33717256230662
O	-3.67083020801851	4.90790762328601	0.59763889609674
O	-2.52530427526046	4.70053106689306	2.99859561264180
O	-1.12340175706462	3.41861009193562	4.84445270774991
Si	2.08118039020900	0.88912724885125	3.67730368389003
Si	0.32018114975707	1.97043378388140	1.54081993465237
Si	-3.12217657137865	5.73604502714845	1.88340448840533
Si	-1.09827592974002	4.01641910577501	3.34588249892392
O	8.28386680863907	-6.03916304531581	3.49404879936001
O	-3.20355044923795	-3.07961371074948	6.57659875561169
O	6.68188797931552	-7.84609177821826	2.44347784206365
O	-1.31196735405433	-1.57212473069790	7.68984992405706
O	8.25990491457502	-6.34313346317560	0.87090081598145
O	-5.39923857467149	-3.45673741066474	5.19890738980060
O	6.64616951307889	-3.91969721762821	3.57667758570414
O	-5.13139162848294	-1.22007992551722	6.59479596555567
O	-8.66763845692656	0.31778199522055	5.59328461548591
O	4.46511679783596	-2.50834773280432	3.25101132025117
O	-6.44432467776957	1.05404958499837	6.95840271380808
O	4.48583482580211	-5.06187435486546	2.61150750577437
O	-3.88354460528888	0.85421578096309	7.58310689112305
O	-2.25714799247512	2.84987027070702	7.13982275140357
O	0.39784589081666	2.65924774767774	6.90402682823174
O	4.25624053187439	-7.39719667591564	1.37543723911092
O	-1.50160911572020	0.87002968116280	8.77680776482426
O	0.71374961248167	0.06982698800274	7.50391165660358
Si	8.12711874004434	-7.12261436848883	2.28646831046897
Si	-2.13578683285757	-2.97571137087155	7.81214564569380
Si	8.19521077380283	-4.41275997867141	3.55258779781840
Si	-4.81592453315006	-2.81610010283784	6.56737598334363
Si	4.52870587974319	-0.89078460520617	3.37600726430171
Si	-8.08214269485896	1.12547763212275	6.88033643743110
Si	5.35499140760497	-3.70075551155971	2.61846741617030
Si	-5.30183183796681	0.06941950664509	7.56832523988235
Si	-0.93104235000815	3.48647628704549	6.45415783936194
Si	3.44437846678717	-6.09887064873873	1.92685294923944
Si	-2.75050780879698	1.78659415534183	8.26792667940457
Si	1.29754916917614	1.32828648652677	6.65843904296175
Si	5.41721370396660	-8.50900961769002	1.64955977109318
Si	-0.51664124762935	-0.38861873795544	8.46871843619471
O	1.80017200552900	4.27297827192019	-4.06458002394907
O	-0.07949784734816	5.26902194013287	-2.49036444383374
O	-0.56393073879212	7.72271414229221	-3.43273488272576
Si	0.69705133832297	3.91701574896353	-2.92760646585643
Si	-1.18562626785496	6.43780703416960	-2.65697444150777
O	1.24674121164473	-1.73117292403763	-4.50630055125387
O	-3.91837271073658	3.97768124045420	-4.55629397528646
O	3.34293257841199	-2.95492244797759	-3.48085768085915
O	-2.44925398367877	5.89389977531473	-3.50776773154092
O	-3.74709687572608	4.17812549801196	-1.91276566627598
O	1.34478407782720	-4.41189825873589	-4.41473375504473
O	-4.62325198564366	1.81373587038626	-5.94339002121154
O	4.72201215674578	-3.30562753530709	-1.25724096831147
O	-2.59156516840085	1.69232631478458	-4.30074489711602

O	-3.14610419243542	-0.34439043628375	-6.54501338182327
O	-1.11522466405580	-1.82908678293586	-5.67122424869934
O	-0.34926985890006	0.31915245954095	-4.26952666719023
O	-0.36107581918475	2.83845056967713	-3.49818003603343
O	1.79429748904376	6.93477158122178	-4.31231869017217
Si	-3.81072207775053	5.02273728471557	-3.30906728892580
Si	2.19534569133081	-3.04126420983209	-4.64072095450622
Si	-4.05256234410473	2.35631322073616	-4.53232762382620
Si	4.01048521575455	-4.07373136912871	-2.50811459119354
Si	-4.33959247237899	0.65719683466995	-7.06158070230838
Si	-0.31111995036185	-1.30400483207350	-4.36518742184313
Si	-1.21160240692976	1.45676376490878	-3.50449540445613
Si	2.56017229465019	5.56096045913226	-4.72306674454745
Si	0.65974756697309	8.08615702842992	-4.45491874707838
O	-2.79164334990701	-5.58061677535912	-7.11412719796285
O	-4.53354619470143	-4.34296224619550	-5.54314606753225
O	-2.94280726577217	-2.91360837594043	-7.22748885607307
O	-2.63810092205689	-0.07764733235765	0.21086197659395
O	-2.53321093482210	2.61789514924692	-0.17339074913199
O	-1.53636354133445	0.93458990146531	-2.00126642915204
O	-5.14502229106508	2.81515228715978	-0.02509904383608
Si	-3.77258105730612	-4.29134411540386	-6.97827942693447
Si	-1.80144727270433	1.19716086307541	-0.43974225294383
Si	-3.79451256770266	3.62902289565613	-0.38964514110984
O	-5.10145962944294	1.99778321849599	-3.34946128052204
O	2.81903500592948	-5.04920446709249	-1.97810944895571
O	-5.60372957954754	-2.51756835055239	2.74710425621546
O	-7.36230238801986	-1.78295280943253	4.63219594054652
O	-0.26065116948313	-6.26966288753924	-3.39656714759427
O	-8.02808448351127	0.22958023661852	3.02505939283453
O	-0.38431313862956	-5.57297886523926	-5.96583859003542
O	-0.95805169682282	-1.93655347165118	-3.02592689638054
O	-3.48799229944138	-6.50521551456777	0.73816834048614
O	-0.99947491146984	-4.24903461170372	-1.81450238756128
O	-6.60027580457718	1.04461840979004	-1.41163775182916
O	0.62348064757214	-6.06306438509682	-0.86756031568923
O	-8.29509549584521	-0.67426293640324	0.52160428591520
O	-7.36274285095780	1.76690194719920	1.01059256960623
O	-1.87184693654424	-6.74333056127443	-1.39824408381826
O	-6.45583233887758	-0.22645981503593	-3.69748011969481
O	2.70094187013169	-5.41915053709412	0.64581781020638
O	-9.07315097872520	-2.64016240490556	-1.05449681059635
O	-4.30763820405932	-0.16037739414534	-2.07649405663642
O	-7.14332551223989	-3.13227751841786	0.70365820573928
O	-8.01242825126476	-2.37955559606570	-3.48043900655357
O	-5.34716586320854	-2.60947858915294	-3.73301451707992
O	-7.26039847728038	-4.36792845579530	-1.89424391515022
O	-5.00160308150180	-5.16281427856506	-3.07398379592604
O	-2.87760597844834	-3.66377250694742	-3.63131322901038
O	-5.69380908418643	-6.17784345852768	-0.72527433906319
O	-2.84011884415030	-2.49915629832134	-1.22581967665290
O	-4.76114237369744	-4.21417977385259	0.88023506809105
O	-5.04907170020069	-1.57900938057952	0.32467906480294
Si	-6.45733557326756	-3.00382311094221	4.04196731067232
Si	0.60077441584578	-5.82706795712199	-4.70559794958870
Si	-8.49091164197919	-0.65553221630049	4.31027828970179
Si	-1.24355362725743	-6.07008981937824	-7.25861558805407
Si	-6.65358315224724	2.28135978174237	-0.36362051552890

Si	-2.44414507984420	-7.48661843844795	-0.05643124397286
Si	-0.62959856762474	-5.81883081761986	-1.88127199235891
Si	-8.42377999011318	0.62767511905543	1.49426632209188
Si	-5.59001202502410	0.62307134372100	-2.61608243312061
Si	2.24802262385640	-6.01789130265492	-0.80973875058499
Si	-8.49951643895720	-2.28508293249392	0.42508611930593
Si	-6.67532203597751	-1.75858875381835	-4.17132584194560
Si	-8.51518785165209	-3.44011730781191	-2.35850153106391
Si	-4.43831300088443	-3.93606771100010	-3.97854706706263
Si	-6.26056651691276	-5.62251345144329	-2.14538766212024
Si	-1.95237647635494	-3.07324472639169	-2.41702659398587
Si	-4.96947478926625	-5.82048456938917	0.69193506797226
Al	-3.79166788001676	-1.14107218405585	-0.76623426256600
Si	-5.60382062560102	-2.83263193192985	1.13870384908585
Si	-2.11412586103908	-1.55006071035242	-6.91889523977154
H	11.67353999564651	-5.53749700541573	-0.03812999158603
H	9.19325799672828	-8.10610399352039	2.36365699561470
H	-3.96156900051312	1.36419899809751	-8.28506099615421
H	-5.56398599870682	-0.12270100128279	-7.29597600125935
H	-6.93632899787489	-1.73777199798247	-5.60525599680165
H	-9.61070199674673	-4.23261599862521	-2.91602399768323
H	-4.80937899877218	-4.38478699994882	-7.98416800181652
H	-1.26201400450263	-7.53013899246911	-7.34713299885286
H	-8.46716099728382	2.53175399144809	6.77435900298738
H	-4.20618400700113	6.50892100727100	2.49273000612106
H	-1.81213400204969	9.00646799966528	0.20177800023830
H	0.23145500806242	10.95011894196466	-2.10809599029032
H	-4.94421500042109	5.93630900163425	-3.34769400026792
H	-7.43440299751212	3.36541399600596	-0.94274899645226
H	-9.81225000837825	1.14342200484315	1.44886400350800
H	-3.26051300187271	2.50761900090689	9.43266799886301
H	-0.74472599736825	4.85510199731576	6.90777200206398
H	1.17261700040065	6.84873799842319	4.60176299768482
H	0.01371299667045	-0.85239399994879	9.75320299768798
H	2.65644500044792	1.61373199825840	7.09816899858534
H	4.53016700007275	3.47572500202946	4.86954700231769
H	1.53638200253084	-6.93834199854224	-4.97336599760116
H	4.68077899615335	9.17388299867359	-0.65420600317089
H	-8.67443699474505	0.53296801208438	8.08822298454852
H	-5.67876700103957	-0.35798099905676	8.92693200298744
H	-5.40538800153943	-3.45794000131581	7.74582399954815
H	-2.86030600158872	-3.03941600078407	9.10082099902673
H	-1.22727299844004	-4.11134499986458	7.70409199869308
H	2.59107099741822	5.38865100101097	-6.17172399595201
H	0.22684599660052	8.11439300010328	-5.85590800339162
H	2.35648399858064	11.66568599940528	-3.24752299769952
H	6.05893599790274	7.26486300049107	-3.76938199760242
H	10.01162899752926	4.23690500275284	-2.76123800016117
H	9.87912999931355	0.42295200142011	-3.59486200230256
H	11.16666300163915	0.78647299994428	-1.60521600141124
H	9.89955900140167	3.90245199977798	-0.38701600028383
H	-1.31531299840805	-1.20646999879805	-8.10616500394555
H	-0.61432200054128	-5.50729800060689	-8.46106599939467
H	2.84834100217181	-3.04402000078646	-5.95043800486428
H	-7.31528199768094	-4.15742499700515	3.75037500148791
H	-9.78237299797811	-1.30797899852104	4.07942699921622
H	-9.40254698845117	-2.75213299501107	1.45267099024353
H	-5.76896400281268	-6.40939899868273	1.78048400350086

H	-3.08137200306785	-8.75069599822582	-0.46757200348481
H	-7.02203599933236	-6.69280400160128	-2.78388200114586
H	2.78306900464731	-7.36900600683156	-0.99232701006330
H	4.89569300622958	-9.57645898778389	2.49579399297354
H	5.81992200577391	-9.04892401048264	0.34487199480199
H	-1.34472299893643	-7.77598299845602	0.87489000441374
H	10.66941399831084	-2.02071799589944	2.56381699896377
H	8.82291800448375	-3.95596099333492	4.78390501121707
H	11.59057500213880	-3.97495099481701	-1.85889601235729
H	5.33835300211303	-0.46465100255808	4.51988999914430
H	7.15208500322901	1.34023100434803	2.37194499821993
H	7.32230800151571	6.57802300033940	-0.51391500194348
H	5.00675699885612	-4.86530399877702	-3.24361800057659
H	7.90182200057865	-7.00149500247350	-1.50647500303769
H	2.44258796836098	-6.50026699980698	2.91141102356298
Ga	-2.40140045522382	-0.81481220530351	2.01905268418989
C	-0.17673652185923	-1.92217121723238	3.68624765172831
H	0.18600122141525	-1.01867646914689	4.15912583873164
C	-0.20830475658714	-3.06888284546159	4.37284436422155
H	-2.94086478423220	0.07427329309392	3.14149246035001
H	0.15511138105178	-3.10008437218563	5.39689605993795
C	-0.61922790239723	-1.86731922206759	2.27567901321085
C	-1.51398576145122	-2.90696888659898	1.84579510379579
H	0.07625619615788	-1.47563790563065	1.53296365950263
H	-3.08202141137504	-2.27459164255310	1.74216524339771
H	-1.54895578223647	-3.15048604920357	0.78396013174063
C	-0.72673478610511	-4.32324716621749	3.73036787722911
C	-1.92358455981212	-3.98642200599272	2.83114316361908
H	-1.03666361465805	-5.05766460226578	4.48083032514773
H	0.07219286610153	-4.79651904714744	3.13653360538841
H	-2.29623240857088	-4.85631959407952	2.28402431667358
H	-2.73555150829726	-3.63286591825216	3.47723477140090

TS12-GaH 2

Electronic energy (r^2SCAN-3c): -30772.031996794492 E_h
Electronic energy (ωB97X-D4rev/def2-mTZVPP): -30780.376101319125 E_h
G_{corr} (673.15 K, 1 atm): 0.46821 E_h
Number of atoms: 256

Cartesian coordinates (Å)

O	1.52998354380550	3.03098784984198	1.07868425356053
O	-1.95664919730660	6.75381389955503	1.40841392212958
O	5.26057562301310	2.07952729582503	0.81213821458194
O	3.63815998960414	3.90226285869471	-0.17755438623914
O	6.11979058858490	4.56570907066884	0.41273264717752
O	1.44664881369027	3.30372505865948	-1.59482943514160
O	1.42723141250882	5.40379704783139	-0.04778234648913
O	-1.69157163150285	6.89400758073651	-1.20267758352681
O	0.30938360205928	7.72689359016334	0.42907095633328
O	2.90759922879449	7.59683523252889	-0.03538763834337
Si	4.86094771337766	3.64730110830334	0.86535576779907
Si	2.01953370202938	3.90470030348322	-0.19825545744215
Si	-1.30257280684346	7.62924883275923	0.20116303638909
Si	1.65595960789819	6.84612633733764	0.65259437859391
O	8.94643041238860	-3.83065994637595	2.22336030633666
O	2.00785953693707	6.65423893828995	2.22947718608233

O	7.13972235984402	-2.28023714575434	-0.91290458067909
O	2.51636423372909	4.76622380408085	4.07699428697182
O	6.74569999225119	-4.90369695213532	-0.75478613883428
O	0.04965757470571	5.13470749456564	3.14219003968955
O	5.83347627822123	-3.25826969899367	1.14603298725523
O	4.45675654499247	4.09597855418449	2.37923646595218
O	9.39844136763919	-5.00968581716331	-1.12783609604720
O	5.15090419622550	-0.25321710912296	2.01716125908616
O	7.73586639913060	4.38652118803527	-1.70626761994000
O	10.70135990581181	-3.18517233343986	0.34563281371192
O	5.42330712174472	5.64796075783904	-1.90394542760784
O	4.12807494076824	5.70596099263454	-4.20621020723041
O	8.76322587401342	-1.45587118535575	1.04453280899191
O	3.88653424459471	7.73439463102522	-2.53143998087374
O	1.23179911013192	9.55084857018745	-4.03402239067311
O	8.97984100019402	2.07268222513241	-1.88589041740234
O	6.94306595442657	0.19216623530543	0.10412125542603
O	2.36101313013889	9.69773295758988	-1.58792566767333
O	8.99377548694228	-0.54645641830814	-1.44463237537931
Si	1.43997168916538	5.87266156128099	3.54394335499742
Si	6.11064977646566	-3.43935810025671	-0.43727365927454
Si	3.64644314396072	3.64646481768655	3.71998037110825
Si	8.07800368273615	-5.83645620465670	-0.65405690131090
Si	10.87511690652467	-4.45921655653446	-0.67213135411336
Si	6.67460550271590	5.32983018760203	-0.91630404418781
Si	9.79385011299783	-2.60634728675487	1.56566248975952
Si	4.92053985789136	6.61395821147668	-3.11221517207785
Si	9.20453274825171	3.67513556273862	-1.66945100115088
Si	6.16687455090772	0.85326305716274	1.37613165062894
Si	1.52618469575981	10.52213366586212	-2.73895189991157
Si	9.79894023928566	0.68368766829661	-2.15349783629873
Si	7.95232104746205	-1.01306388928981	-0.30051606854218
Si	3.47635564943208	8.57778506524383	-1.20082234060322
O	2.98448006972227	-0.44093149094678	3.63704758213084
O	0.99367427001957	0.84223338151602	2.44963944979758
O	1.23720949106639	0.91890448599516	5.06766372990784
O	2.99974638125178	2.17975187904931	3.48005767736813
O	-0.27960761240879	1.34921419436402	0.19097046171748
O	-0.81884072145161	2.77450108409985	2.32841606546324
O	-3.73293135374150	4.90908094767183	0.60470494021888
O	-2.53185491937212	4.68321474281266	2.97555057810773
O	-1.13939822052093	3.42199870650020	4.83915727823669
Si	2.05918612423340	0.87661437535264	3.67318252490892
Si	0.35608159998970	2.00482687356631	1.52316258329507
Si	-3.13561653922643	5.72822281743547	1.87656830094432
Si	-1.10153167908646	4.01071748444935	3.33768211326672
O	8.29771283449416	-6.04465549422942	3.49646900238716
O	-3.19886865062453	-3.07770971170599	6.57918063092613
O	6.68665211767926	-7.85001836809579	2.44837325625324
O	-1.31172655181048	-1.57045049403973	7.68929818060363
O	8.26251275819239	-6.34689213316092	0.87205026386714
O	-5.42762934400954	-3.46920386777124	5.23286062379722
O	6.65216881215103	-3.93816116087876	3.57432761499803
O	-5.12066536463708	-1.21975480510256	6.59473310438035
O	-8.68951690561960	0.38126640849857	5.55586303271028
O	4.48691853842704	-2.50702002519072	3.28431240440260
O	-6.45288462395068	1.03728115934857	6.94451063722841
O	4.47249913055709	-5.05115045576766	2.61537634268899

O	-3.89293184343123	0.86809228223948	7.58134669584417
O	-2.25270842122510	2.85144687251498	7.14193748512747
O	0.40217631000942	2.66044564176684	6.88519656483440
O	4.26611273982889	-7.39551124967541	1.38520728286848
O	-1.50864212338715	0.86576724182573	8.77848386374585
O	0.71615777482098	0.07769787639017	7.51420096385994
Si	8.12969152295075	-7.12529879133373	2.28825873033669
Si	-2.13070263397419	-2.97614591030725	7.81600280637184
Si	8.20327258404831	-4.41927220209630	3.55218524304173
Si	-4.80603376946782	-2.81541838031924	6.58066348894651
Si	4.52460460364234	-0.88628742029461	3.38390638545616
Si	-8.08758569085666	1.12980902151717	6.87549140486238
Si	5.35705345552531	-3.70118730435788	2.62955851901125
Si	-5.30133185826710	0.06946585796751	7.56683687728511
Si	-0.93020978863680	3.48706809806905	6.44705498188897
Si	3.44258881989251	-6.10085192469067	1.92776988026670
Si	-2.75020195418608	1.78919903821297	8.26749799279689
Si	1.29552424979871	1.32211629899167	6.64478870598676
Si	5.42128718934545	-8.51050766962255	1.65242102366913
Si	-0.51604580977349	-0.38754556412433	8.46963219040857
O	1.87851862068229	4.29858106768121	-4.02151675171190
O	-0.06787063146593	5.26683397031644	-2.49504220950261
O	-0.57232127914112	7.70658387055165	-3.45346355974904
Si	0.74440284142709	3.93388955624141	-2.92553582418850
Si	-1.18553360005846	6.42306050817845	-2.67002819108900
O	1.23631349058015	-1.75760671034081	-4.46453632351006
O	-3.97897640851529	3.95225209068724	-4.53673906491955
O	3.34846347984040	-2.96103793994667	-3.48196857660536
O	-2.44231908619084	5.85091704555870	-3.51786575211228
O	-3.77076004781579	4.18443947004868	-1.90477570690022
O	1.34680681279211	-4.42733304888470	-4.41413898605619
O	-4.65808978152805	1.78152891398247	-5.91676749184355
O	4.71515532566817	-3.30923268387660	-1.24951291553517
O	-2.49208770737618	1.76194236914779	-4.46537836012458
O	-3.14907228334712	-0.34871384158129	-6.54612064040331
O	-1.09360115447375	-1.81810067483084	-5.69218028777703
O	-0.30167858417776	0.32358169884556	-4.28627314003018
O	-0.27098706427288	2.86607933052154	-3.58423265360672
O	1.80706828636767	6.94784500103939	-4.31754018117084
Si	-3.82298320031397	5.01234333224415	-3.30810303879297
Si	2.19547464702966	-3.05644882310754	-4.63487523751283
Si	-4.01198066926837	2.32591122089730	-4.53926122133034
Si	4.01041083241646	-4.07968579923290	-2.50419393454638
Si	-4.34393390202614	0.65320664414673	-7.05947485079339
Si	-0.31355724745128	-1.30051246853677	-4.37136076439526
Si	-1.15455548136713	1.51361867937855	-3.60152559939791
Si	2.59005389993550	5.58359637688043	-4.72684900472487
Si	0.66012024286866	8.08645286249678	-4.45872810322021
O	-2.79306513830881	-5.58545178060677	-7.11468799624197
O	-4.56999134220469	-4.36653193743934	-5.56309998801168
O	-2.94469110590029	-2.91938385668333	-7.20911999062410
O	-2.46206456948513	-0.06534215054551	0.06366847311760
O	-2.54445769360315	2.64466656164468	-0.15519224203443
O	-1.58186849737824	1.07862049756298	-2.09870574644766
O	-5.14896322530104	2.77548164155358	-0.04300175357383
Si	-3.77669961729278	-4.29992898799861	-6.98041223814877
Si	-1.72481044511168	1.30299758817298	-0.51733416913563
Si	-3.81833315254567	3.63222810797569	-0.38571963098108

O	-4.90550688801059	1.87150616120614	-3.26520042480094
O	2.81605719664849	-5.05313516835363	-1.98544223894370
O	-5.78229956784344	-2.43920733611238	2.81601001209762
O	-7.43503451571575	-1.80359711414092	4.76218831936721
O	-0.25070768251542	-6.31763672469654	-3.40409866441340
O	-8.00145883880883	0.13974330904887	2.99167375239330
O	-0.38905418082914	-5.57166386041433	-5.96338728361889
O	-1.04183313170126	-1.89355028963444	-3.05274860840058
O	-3.48374463303573	-6.49441872504154	0.73125355680687
O	-0.96935929689958	-4.25678994658263	-1.91320020200621
O	-6.67348532165328	1.08078358671126	-1.46106512289670
O	0.62215929690678	-6.06361988651465	-0.87580232523202
O	-8.42845468737715	-0.61122715186608	0.45180289876476
O	-7.35186585787525	1.74969176831930	1.01621432195120
O	-1.87636151508015	-6.73156545849291	-1.40032972037852
O	-6.43786154287691	-0.22036868681316	-3.72717989422870
O	2.72071729032609	-5.43750576672198	0.63152942294675
O	-9.09708890022597	-2.69047369387248	-1.03115707130596
O	-4.49842646492397	-0.33912355271177	-1.89356087441924
O	-6.94755075893303	-2.77920415369415	0.48570945274884
O	-8.00720500914424	-2.35689563597121	-3.44670045211042
O	-5.35248018522652	-2.62295837102874	-3.75073299468552
O	-7.26379600052771	-4.37430703577720	-1.89244105442295
O	-5.00377238439045	-5.16856984908240	-3.07959930359045
O	-2.87954853210132	-3.68999908189936	-3.69846651950125
O	-5.68878645231850	-6.15558926591573	-0.71941231491165
O	-2.89367049961846	-2.60145202673399	-1.27722431834410
O	-4.82913168326972	-4.23662060685198	0.98306517598489
O	-4.44558128523659	-1.64852251918921	0.82970663700431
Si	-6.52709296997830	-3.00081250070142	4.13749127104165
Si	0.59860971234143	-5.84001866866592	-4.70690101187139
Si	-8.50537766800027	-0.65379542507147	4.32642322178492
Si	-1.24305621477220	-6.07095603583060	-7.25797981933477
Si	-6.66921278115387	2.27951029926569	-0.36748271174822
Si	-2.43997676081149	-7.48225225320321	-0.05745030212274
Si	-0.62204305333135	-5.83153632107620	-1.89856497882675
Si	-8.43814590646258	0.63425909861007	1.49676703550859
Si	-5.60869605545749	0.57157984838007	-2.57896319136962
Si	2.24575569345588	-6.02172097277166	-0.81499379548911
Si	-8.48595969789123	-2.22893352867037	0.40068528901587
Si	-6.67542930028570	-1.76113513682370	-4.17403056057172
Si	-8.51671413218555	-3.44558423015319	-2.35365839271696
Si	-4.44991937965117	-3.95272131760957	-4.00335709826329
Si	-6.25811039781437	-5.62389843200950	-2.14560808418276
Si	-1.96634755797163	-3.10866135464050	-2.47148734524050
Si	-4.96751213858491	-5.83585162589590	0.70720781087508
Al	-3.67386566839267	-1.19813391304681	-0.69435638322445
Si	-5.52287416478593	-2.80141199862860	1.25960967464061
Si	-2.11501832485331	-1.55248304519122	-6.92288975935398
H	11.67353996966578	-5.53749698673506	-0.03813000186098
H	9.19325799414693	-8.10610399191223	2.36365697321288
H	-3.96156901040245	1.36419899425334	-8.28506100160320
H	-5.56398597914823	-0.12270099533114	-7.29597599513178
H	-6.93632899789622	-1.73777198794660	-5.60525597649064
H	-9.61070198617790	-4.23261599035638	-2.91602401478953
H	-4.80937902611946	-4.38478701080896	-7.98416802000667
H	-1.26201400189274	-7.53013899456889	-7.34713298492833
H	-8.46716099628699	2.53175398758348	6.77435899238792

H	-4.20618400849263	6.50892102072103	2.49273000446514
H	-1.81213399048200	9.00646795678553	0.20177799537489
H	0.23145499972410	10.95011899980674	-2.10809599485393
H	-4.94421500466796	5.93630898828879	-3.34769395249891
H	-7.43440305211532	3.36541405141185	-0.94274904995029
H	-9.81224996501897	1.14342199196358	1.44886399889878
H	-3.26051300628176	2.50761900412140	9.43266800330153
H	-0.74472599675911	4.85510200904363	6.90777200485244
H	1.17261698376485	6.84873802531684	4.60176301806957
H	0.01371299671538	-0.85239400242064	9.75320300015199
H	2.65644502715632	1.61373200067678	7.09816900846847
H	4.53016699746301	3.47572497343338	4.86954699509698
H	1.53638201067426	-6.93834201047811	-4.97336600102575
H	4.68077899592181	9.17388299267304	-0.65420599991559
H	-8.67443699898821	0.53296800203326	8.08822299777710
H	-5.67876700112393	-0.35798099930278	8.92693200000891
H	-5.40538800405115	-3.45794000701006	7.74582400788367
H	-2.86030600255558	-3.03941600228525	9.10082099782146
H	-1.22727299674199	-4.11134500200591	7.70409199968939
H	2.59107099786078	5.38865100082782	-6.17172401029376
H	0.22684599965318	8.11439300636787	-5.85590803713584
H	2.35648400129152	11.66568600080373	-3.24752299721817
H	6.05893599916206	7.26486299529573	-3.76938199240255
H	10.01162899911584	4.23690500289254	-2.76123800434994
H	9.87913000009522	0.42295200324409	-3.59486200641997
H	11.16666300787395	0.78647300046448	-1.60521600023013
H	9.89955900194446	3.90245199937865	-0.38701599711985
H	-1.31531299351927	-1.20647000415134	-8.10616500378760
H	-0.61432198663518	-5.50729799282364	-8.46106602282610
H	2.84834101460205	-3.04402000407888	-5.95043803534885
H	-7.31528200581543	-4.15742500393858	3.75037499410265
H	-9.78237298545372	-1.30797899639102	4.07942700225383
H	-9.40254701265454	-2.75213300390939	1.45267101827093
H	-5.76896400769450	-6.40939900191523	1.78048401531455
H	-3.08137201534376	-8.75069600611914	-0.46757200225526
H	-7.02203600350108	-6.69280401519696	-2.78388201007474
H	2.78306901311782	-7.36900600231785	-0.99232700529649
H	4.89569299930886	-9.57645898734254	2.49579399331495
H	5.81992200642897	-9.04892401581178	0.34487198380632
H	-1.34472298498555	-7.77598300005955	0.87489000847284
H	10.66941398835092	-2.02071800991440	2.56381699324839
H	8.82291801958078	-3.95596099249867	4.78390504555221
H	11.59057502081586	-3.97495099137160	-1.85889601822496
H	5.33835300692542	-0.46465099694408	4.51989000891730
H	7.15208500325587	1.34023100298663	2.37194499645349
H	7.32230800638889	6.57802300456341	-0.51391500186642
H	5.00675697896573	-4.86530398784950	-3.24361799784941
H	7.90182200094955	-7.00149502024571	-1.50647497406293
H	2.44258797182878	-6.50026701189721	2.91141104931779
Ga	-2.89355314048851	-1.61869245086919	2.39477578856614
C	1.80086595948288	-2.55187950156134	1.37425938813636
H	2.82802826332439	-2.88392431353472	1.28145215404019
C	1.12085289368792	-2.71939451296497	2.51285045750647
H	-2.99818406378503	-0.18222399699074	2.97639726936404
H	1.58972996310863	-3.16943406604557	3.38477644840391
C	1.17727203982428	-1.93758367003489	0.22212621948084
C	-0.14313104921780	-1.71629884403375	0.17985728834848
H	1.78483271260945	-1.71280586727469	-0.65177531455754

H	-3.16989994797877	-2.98556178756748	3.06429519362329
H	-0.56344115718593	-1.36129578947909	-0.75277488807237
C	-0.27381273889176	-2.17539232660052	2.66325326772768
C	-1.06005434504422	-2.08439735838255	1.32530774690522
H	-0.19786582163376	-1.17643177771401	3.12397709465777
H	-0.82245714765139	-2.79781774718587	3.38284251530832
H	-1.46295934055715	-3.07749950632470	1.04966540070183
H	-1.88461245211166	-0.77624031303006	0.74812509395501

TS13-GaH 2

Electronic energy (r^{2}SCAN-3c): -30772.063545150762 E_h
Electronic energy (ωB97X-D4rev/def2-mTZVPP): -30780.406646163938 E_h
G_corr (673.15 K, 1 atm): 0.47464 E_h
Number of atoms: 256

Cartesian coordinates (Å)

O	1.51831418626681	2.99610301659013	1.06926000145912
O	-1.95426964760912	6.75313122469501	1.40090484125403
O	5.26715143808933	2.07801846436175	0.80287167339434
O	3.62960161709557	3.89262085096289	-0.17324095114855
O	6.11021592147962	4.57100042959176	0.41442969807512
O	1.44196500791386	3.29786711012141	-1.59472153062957
O	1.41858501297615	5.38601880456906	-0.02833535658810
O	-1.70324061773046	6.89385503337384	-1.21349376412047
O	0.30830528315453	7.71992634254875	0.41056086421962
O	2.90527508074511	7.58220275743000	-0.04448252704387
Si	4.85744376754953	3.64264952133588	0.86381352236358
Si	2.01082725485344	3.88859047013726	-0.19335740406516
Si	-1.30514473173284	7.62742391376508	0.18975913222063
Si	1.65187060844712	6.83806617154008	0.64919858960474
O	8.94508106446218	-3.82997429389953	2.22456326706679
O	1.99902183912494	6.66049223921244	2.22985655809584
O	7.14585267017094	-2.28138373421325	-0.91812253546618
O	2.50876642326970	4.75754964301669	4.06943980039264
O	6.74830336668195	-4.90510962200461	-0.76685244767339
O	0.04561427444759	5.12936391704443	3.13640538498071
O	5.84900653974913	-3.26688661364772	1.14006185125590
O	4.45119702749253	4.07961707749004	2.38048966500107
O	9.40075038607589	-5.01147908942931	-1.12935186563770
O	5.15953977597981	-0.25002767807486	2.01576793711492
O	7.73341989614481	4.38396241164300	-1.70117636923690
O	10.70166227282373	-3.18675390085555	0.34686929094616
O	5.42164164605388	5.64789917615213	-1.90500303639751
O	4.13280996878512	5.71279872094562	-4.21316901632263
O	8.76531183318238	-1.45573617824756	1.04372216295652
O	3.88877290969515	7.73756415039557	-2.53406785571985
O	1.22674694356003	9.54784181869674	-4.02922067357190
O	8.98006136563446	2.07244727924908	-1.88589479094016
O	6.94950191223052	0.19208602234607	0.10024465366486
O	2.36087624526481	9.69653810398788	-1.58524884086255
O	8.99893162739693	-0.54831252301905	-1.44608227033970
Si	1.43494415310803	5.87022407874806	3.54119545356455
Si	6.11533528452030	-3.44083402745882	-0.44502489122509
Si	3.63872428736510	3.63398633685792	3.72074987273505
Si	8.07935701266307	-5.83685093391864	-0.65622043786035
Si	10.87580348405774	-4.45956632527513	-0.67216375550971

Si	6.67159779524000	5.33024318724040	-0.91516219765566
Si	9.79380067011116	-2.60728580957959	1.56632784731044
Si	4.92164812252105	6.61561654674829	-3.11337151335870
Si	9.20369170847072	3.67490222774371	-1.66893794206400
Si	6.17409493411536	0.85445762174046	1.37063010485224
Si	1.52621709636123	10.52098485606283	-2.73709188036530
Si	9.80009700854418	0.68399006508692	-2.15417569969423
Si	7.95671608207953	-1.01545753164167	-0.30334139191667
Si	3.47576638727386	8.57471168330038	-1.20060673703943
O	2.97234135277267	-0.46306757574263	3.59188934079375
O	0.97603382451521	0.81419765002756	2.44457073067354
O	1.24580599282451	0.88853717275780	5.06761325685159
O	2.98944464148635	2.16487140883307	3.48683168513669
O	-0.30676518369792	1.30818502215324	0.18815264942295
O	-0.82256254706388	2.75569976677374	2.32996597477931
O	-3.77743439591657	4.95027536836765	0.59513761044591
O	-2.53566864672064	4.66973474756806	2.94433292908177
O	-1.15873640741877	3.42977716821504	4.83169851193538
Si	2.04715842897048	0.85831870731274	3.66158858743884
Si	0.33829590767890	1.97430177795832	1.51227933077046
Si	-3.14103145201474	5.73640960188789	1.86668586566218
Si	-1.10630672148690	4.00484220365419	3.32379295435898
O	8.28339411758406	-6.03994247535884	3.49466420650820
O	-3.20118102044881	-3.05817373478322	6.58425554827091
O	6.68308103478387	-7.84907789777546	2.44409045494412
O	-1.30496721932308	-1.56833332474970	7.69222762369892
O	8.26071706170739	-6.34395332198326	0.87158539385439
O	-5.44626596641910	-3.51467362589818	5.29032328537251
O	6.64883665629808	-3.91671799311055	3.57665640960073
O	-5.15486263985397	-1.21861696146580	6.59816650685401
O	-8.63418714216755	0.32029540921215	5.57263699222275
O	4.46687524613682	-2.50907376891238	3.25125290907739
O	-6.43938738391984	1.06675311234528	6.96781509124919
O	4.49032623447352	-5.06399596376134	2.61439725090521
O	-3.87641314739513	0.84883797294875	7.58359804107363
O	-2.25176200785261	2.84919972821760	7.14020779074360
O	0.40127226022919	2.65629644542051	6.85976879910971
O	4.25755388719951	-7.39588477460556	1.37803867291790
O	-1.49939625380664	0.87157434285794	8.78163568368420
O	0.72306473250657	0.07901537700002	7.51858115152358
Si	8.12643464998759	-7.12372205486033	2.28706291476467
Si	-2.12762925419366	-2.96940834976115	7.81884233236624
Si	8.19612373172362	-4.41360449073617	3.55265861251035
Si	-4.81037438520918	-2.80717031808707	6.59914889898509
Si	4.52661895962321	-0.89101342309691	3.37382444637967
Si	-8.07568801985264	1.12650404464337	6.87826932153771
Si	5.35609748857624	-3.70285721655518	2.61892186054088
Si	-5.29882574737450	0.07559992448446	7.57226220469803
Si	-0.93351281090051	3.48816682541953	6.43773270567410
Si	3.44291898534174	-6.09675328515177	1.92892546785033
Si	-2.74502183494391	1.78788767156054	8.26825355074412
Si	1.29847890325230	1.31715954879493	6.63760801696670
Si	5.41600875128250	-8.50842389119795	1.64967943142818
Si	-0.51026609110213	-0.38384692752073	8.47141962905147
O	1.89181635723533	4.30217461927249	-4.01365670221457
O	-0.05737564666427	5.27366647912689	-2.49152566233302
O	-0.58366243290339	7.69947807260440	-3.46474384607664
Si	0.75056263470036	3.93844697815276	-2.92552743163721

Si	-1.18936985743051	6.41459072340653	-2.67680067497023
O	1.24593702164818	-1.74949000097850	-4.49480092001339
O	-4.00685607874231	3.93170591999793	-4.53353395272896
O	3.34590725025766	-2.96238497414776	-3.48323562689243
O	-2.43567831621359	5.81922368761001	-3.52579154213954
O	-3.83710376419183	4.20559236124538	-1.91367047151312
O	1.34418646484492	-4.42396475947448	-4.41838872589678
O	-4.66725119022230	1.76549202104014	-5.90754737013618
O	4.72146187847328	-3.30690761877353	-1.25615804262714
O	-2.46025302636357	1.78341554013284	-4.51347210447848
O	-3.15121835892294	-0.35218787407357	-6.54990288727959
O	-1.11430985772087	-1.81726115884318	-5.66550960683428
O	-0.28663515744771	0.33154447991043	-4.29099072851558
O	-0.26113591116118	2.87651717993350	-3.59431376507362
O	1.80609943742175	6.94845826297694	-4.31647593472766
Si	-3.83502082733074	5.00844735539964	-3.32364855968020
Si	2.19719730279510	-3.05503307312999	-4.63935330576092
Si	-3.99715737845302	2.30400174829770	-4.53935028632692
Si	4.01046869898094	-4.07832451721941	-2.50525426394666
Si	-4.34523514759093	0.65021941016005	-7.06027469620512
Si	-0.30094128880437	-1.29334007574753	-4.36392241280819
Si	-1.14431793487747	1.52415278090848	-3.61934841984352
Si	2.59325511761671	5.58686585727200	-4.72738600450267
Si	0.65647467934961	8.08422193649552	-4.45881398281345
O	-2.79411369734282	-5.59024561787100	-7.14100304655455
O	-4.49469161144601	-4.37332437618773	-5.52595754925816
O	-2.93594499986273	-2.92096469935159	-7.21870127976702
O	-2.63624454214286	0.14821132931496	0.07700363135947
O	-2.44730779675778	2.79864682110743	-0.21137688826584
O	-1.59965757976450	1.10099681051611	-2.11972809720696
O	-5.10444560320011	2.75557304423871	-0.03965931038110
Si	-3.76700657608420	-4.29999405865338	-6.97781938180507
Si	-1.74806010570008	1.37411991935840	-0.54532374596780
Si	-3.81076177096879	3.67097107436008	-0.39407296755296
O	-4.82361557600111	1.79800685192930	-3.24097097373834
O	2.81664652448198	-5.05187869250799	-1.98074706611088
O	-5.50662220802380	-2.74521929447232	2.83862807838479
O	-7.33342948059440	-1.77865158685610	4.59249471762813
O	-0.25302106759264	-6.30210574342693	-3.39377788737507
O	-8.01546732690983	0.19334316702476	2.98402045063341
O	-0.40091845162508	-5.57633340039338	-5.95562900802410
O	-0.98108069386170	-1.87720253928104	-3.01836731462480
O	-3.49432732021462	-6.52762903031532	0.74746908937660
O	-0.93132622771681	-4.23717879859476	-1.87799861890588
O	-6.63616398229184	1.08307067083722	-1.46240605503432
O	0.62771449033186	-6.07649286040285	-0.86536715488699
O	-8.41665159134426	-0.60455179740113	0.44922693304438
O	-7.34037950694691	1.76396586846072	1.00054347985134
O	-1.88034925876425	-6.70684046756960	-1.38828023530083
O	-6.45627186140277	-0.22066732210328	-3.72906535316560
O	2.71623152553489	-5.41505201362842	0.64070068248848
O	-9.06471386889723	-2.69156629125440	-1.02067075313125
O	-4.54789114045356	-0.47889054010006	-1.90319937769281
O	-6.97342487145833	-2.79289620346872	0.62170084536072
O	-8.00213046506023	-2.38155372243194	-3.44596476319118
O	-5.36212074907075	-2.62811356902044	-3.76123596898952
O	-7.25332868102687	-4.39000986587569	-1.85610075544949
O	-4.99113720126894	-5.16106662163243	-3.05199257386681

O	-2.88550083418389	-3.62951745363741	-3.58542008483210
O	-5.70313935857190	-6.26329639551401	-0.74108216021571
O	-2.74314147985908	-2.51141003803216	-1.14334664957875
O	-4.81493655739390	-4.25431653457539	0.75525527055080
O	-4.47840618722100	-1.61576132174084	0.86586302266579
Si	-6.45709490950935	-3.05794808186991	4.11256128876625
Si	0.59560459694158	-5.83770702063084	-4.70311544139524
Si	-8.47932044039444	-0.65920246757913	4.29228372070802
Si	-1.24233408823806	-6.07276643706035	-7.26020974525500
Si	-6.63632233569940	2.28674783606741	-0.37774654698376
Si	-2.43612924009982	-7.48578361612868	-0.05900138413720
Si	-0.61437684618087	-5.81800223110303	-1.88453732368576
Si	-8.43339424035142	0.65619456448689	1.47676816701659
Si	-5.58733180407250	0.52056564293525	-2.57208867695829
Si	2.25131097394516	-6.01835008122009	-0.80615177158449
Si	-8.48673635605685	-2.22212460779303	0.42023611459021
Si	-6.68136614471306	-1.76381445965585	-4.17566534046772
Si	-8.50288945550097	-3.46177468479231	-2.34230506731095
Si	-4.42791650980042	-3.93838930853640	-3.96695543247411
Si	-6.25266927804004	-5.64199927673613	-2.14208623427473
Si	-1.91968279392944	-3.05648095784354	-2.39373970170585
Si	-4.97765890550858	-5.87403709807650	0.66451978626950
Al	-3.64874816678810	-1.15190977640683	-0.62571478015020
Si	-5.47342168827025	-2.84840452876307	1.21934520151123
Si	-2.11156410217263	-1.55296696449541	-6.91734667583087
H	11.67353999590003	-5.53749699981858	-0.03813000156586
H	9.19325799204019	-8.10610398017920	2.36365700245943
H	-3.96156901004179	1.36419899391922	-8.28506098618957
H	-5.56398598326550	-0.12270099056939	-7.29597600365722
H	-6.93632899118293	-1.73777199604362	-5.60525593376824
H	-9.61070197914585	-4.23261598059131	-2.91602398605307
H	-4.80937898708973	-4.38478700785493	-7.98416798536532
H	-1.26201399778752	-7.53013900654968	-7.34713299670674
H	-8.46716099487964	2.53175398749063	6.77435899221741
H	-4.20618400268236	6.50892099677788	2.49273000159218
H	-1.81213399817137	9.00646799193790	0.20177800029599
H	0.23145499509259	10.95011898886433	-2.10809599240109
H	-4.94421499434440	5.93630897117520	-3.34769399897535
H	-7.43440298126373	3.36541396363181	-0.94274899881607
H	-9.81224998394564	1.14342198848462	1.44886400510267
H	-3.26051299265023	2.50761898486630	9.43266798817375
H	-0.74472599324135	4.85510198951597	6.90777200215536
H	1.17261700277611	6.84873799039273	4.60176299279198
H	0.01371299341845	-0.85239398402299	9.75320296442064
H	2.65644495675786	1.61373198558846	7.09816900103055
H	4.53016700361941	3.47572500968032	4.86954700726527
H	1.53638199422992	-6.93834199351657	-4.97336599634690
H	4.68077899502860	9.17388299444919	-0.65420600281474
H	-8.67443700879185	0.53296799112588	8.08822301355450
H	-5.67876700113957	-0.35798100653753	8.92693200970030
H	-5.40538800712880	-3.45794000785734	7.74582400564427
H	-2.86030597406616	-3.03941602350663	9.10082096618680
H	-1.22727303539955	-4.11134497670550	7.70409203456835
H	2.59107099743888	5.38865099693879	-6.17172399393102
H	0.22684599790183	8.11439299684757	-5.85590800261190
H	2.35648403334391	11.66568610934330	-3.24752302754964
H	6.05893599295144	7.26486300076481	-3.76938199927695
H	10.01162899894117	4.23690499878970	-2.76123799549207

H	9.87912999779171	0.42295200073243	-3.59486199559376
H	11.16666299879457	0.78647300042357	-1.60521600354857
H	9.89955900217628	3.90245199970313	-0.38701600528925
H	-1.31531300442878	-1.20647000698741	-8.10616499569390
H	-0.61432200517121	-5.50729800430138	-8.46106598859502
H	2.84834099813744	-3.04402000168683	-5.95043800158532
H	-7.31528194252053	-4.15742493150844	3.75037500831727
H	-9.78237302126406	-1.30797900434751	4.07942700178098
H	-9.40254698429045	-2.75213299533079	1.45267094786554
H	-5.76896398925698	-6.40939898271513	1.78048397138886
H	-3.08137199301733	-8.75069600131249	-0.46757199443829
H	-7.02203599890196	-6.69280399699442	-2.78388199106447
H	2.78306900446557	-7.36900600200766	-0.99232700161654
H	4.89569300724202	-9.57645899124140	2.49579400312187
H	5.81992199991392	-9.04892399686590	0.34487200198965
H	-1.34472301877270	-7.77598300710325	0.87488998969805
H	10.66941399163466	-2.02071800706861	2.56381699407113
H	8.82291797871644	-3.95596102078535	4.78390496031386
H	11.59057499670637	-3.97495100194281	-1.85889599640586
H	5.33835295151235	-0.46465103242911	4.51988993299029
H	7.15208502150873	1.34023101362897	2.37194501071899
H	7.32230799527097	6.57802299372815	-0.51391499863078
H	5.00675698999039	-4.86530398998261	-3.24361798273973
H	7.90182200117119	-7.00149499435056	-1.50647499373476
H	2.44258799718762	-6.50026700062850	2.91141100595918
Ga	-2.99307950083320	-1.62555107345251	2.79107408670097
C	0.32599295194391	-2.73220033737792	3.78144707677343
H	1.09304647447936	-2.30156679333502	4.42020993914992
C	-0.66309565981921	-3.47709583731229	4.30694701401574
H	-2.20415942364915	-0.22580731664779	2.21109311030992
H	-0.69278108112361	-3.68538863841176	5.37205964452809
C	0.40567277295207	-2.52184900428845	2.34405136140307
C	-0.67581660218443	-2.75446111557685	1.57633437412317
H	1.32301447389145	-2.13291087686503	1.91309918119210
H	-3.75901617191155	-1.04719819437614	4.00946240735672
H	-0.66214545574243	-2.54102672269263	0.51089166014237
C	-1.66226320113965	-4.14033826683496	3.39567313064062
C	-1.94512117876615	-3.23793495776428	2.19045833710469
H	-2.58198611191826	-4.39328180520029	3.93520197718586
H	-1.23374251888515	-5.09585349088250	3.03654220367375
H	-2.56245047655646	-3.74776452567516	1.44706003293712
H	-2.40666750200346	-0.07712830310251	1.27318308046313

TS14-GaH 2

Electronic energy (r^2SCAN-3c): -30770.886468614353 E_h
Electronic energy (ωB97X-D4rev/def2-mTZVPP): -30779.211442735388 E_h
G_{corr} (673.15 K, 1 atm): 0.43953 E_h
Number of atoms: 254

Cartesian coordinates (Å)

O	1.50613729139848	2.97277762998568	1.04298860020102
O	-1.94959938645064	6.76531483435327	1.42523221501240
O	5.26241601275336	2.08242076624457	0.80711131460215
O	3.62386961920999	3.90089280855240	-0.16653528535038
O	6.11228570986768	4.56921364893333	0.41106426858374
O	1.45414117129684	3.30742234226459	-1.61149780984174
O	1.40415547368929	5.38022986481784	-0.01627928208528
O	-1.69202985412388	6.89780522771419	-1.18985118242258
O	0.31579129894104	7.72600676290365	0.43329494656081
O	2.90652352485873	7.56810424279030	-0.05825705346978
Si	4.85574117422525	3.64873843153240	0.86401259208311
Si	2.00447675000813	3.88715364705708	-0.20025522248821
Si	-1.29772762078819	7.63281517161691	0.21072316280313
Si	1.65609933812595	6.83432358681412	0.65218531722270
O	8.94189134168059	-3.82752550064403	2.22492382001581
O	2.01740329376811	6.65677983170198	2.23087017013475
O	7.14704601287553	-2.27893223253565	-0.91936689505754
O	2.51016951686205	4.75571124275067	4.07320653481716
O	6.74941228786115	-4.90390965266985	-0.76682655929048
O	0.04982716142859	5.14133569481040	3.14235840195761
O	5.84990040988958	-3.26452552330928	1.13777457723460
O	4.45803197047709	4.09285037906557	2.38137996477308
O	9.40152560608702	-5.01226545490148	-1.13061470127907
O	5.15114007217428	-0.24425360383408	2.01782659865612
O	7.73373376551936	4.38665314361576	-1.70448558671490
O	10.69871284073285	-3.18662935503795	0.34671532290849
O	5.42107243925096	5.65402791879377	-1.90162250218236
O	4.09060032548444	5.67086236971974	-4.16989505216409
O	8.76311755661673	-1.45376917563543	1.04523948025019
O	3.89184838528125	7.74619297046210	-2.54447034693442
O	1.24202227703516	9.54728288093506	-4.03423840510243
O	8.97945850854645	2.07391287927927	-1.88656843564294
O	6.94358341713437	0.19192405595478	0.10257574069420
O	2.36001586349883	9.69591358009033	-1.58286614695100
O	8.99809369845987	-0.54434511191499	-1.44305843715265
Si	1.44316900229805	5.87433800650292	3.54214546568289
Si	6.11742719466187	-3.43879983594677	-0.44783846244998
Si	3.64767398473147	3.64243140274141	3.71995317153823
Si	8.08022738048887	-5.83658981954020	-0.65698927717210
Si	10.87593061802352	-4.45927573300190	-0.67218371807885
Si	6.67206235686884	5.33220040320977	-0.91679082597075
Si	9.79221223637086	-2.60554080326134	1.56632180191269
Si	4.91039180600407	6.60750961124423	-3.11734853357465
Si	9.20324424381566	3.67652282320643	-1.66931015361331
Si	6.16943285980570	0.85827020086792	1.37213079378038
Si	1.52786595298421	10.52000106278022	-2.73778003518991
Si	9.79998428376202	0.68595622901752	-2.15428137040015
Si	7.95503919835136	-1.01192621939186	-0.30193248579644
Si	3.47659294667871	8.57320462333403	-1.20508571828111
O	2.97826887795224	-0.45869490773052	3.62655873470672

O	0.99584563679032	0.82589902662305	2.46658726715578
O	1.24935169244292	0.92400703185496	5.07573262237234
O	3.01478791681675	2.16717097393664	3.48187881145216
O	-0.33773633627664	1.22919731547855	0.26468691170680
O	-0.81714537884233	2.77961912368563	2.32933618898387
O	-3.70692538164989	4.92052270623674	0.59216264940713
O	-2.53132115660726	4.68528050142227	2.97761189202574
O	-1.13378387547340	3.42071870702215	4.83763622965958
Si	2.06493875644127	0.87190303141430	3.67702443288463
Si	0.33320047235675	1.96533423212534	1.53129477973586
Si	-3.13025042211558	5.73356020951567	1.87608488927962
Si	-1.09911137569507	4.01591292672283	3.33606181954004
O	8.28147821740220	-6.03858279380442	3.49408784923832
O	-3.20000229642734	-3.06036247922631	6.57513362685000
O	6.68151305618911	-7.84585646493893	2.44300574272932
O	-1.31135439672432	-1.56811715626525	7.68261730797383
O	8.26128387267192	-6.34324579341534	0.87121439767675
O	-5.40991014170786	-3.46273637406675	5.21739402459499
O	6.64494145968605	-3.91705996430761	3.57608872963627
O	-5.12879484462218	-1.21620733968908	6.59186521872262
O	-8.67494985017642	0.33951449167155	5.58090838673904
O	4.46031797249023	-2.50723790728861	3.24464784189634
O	-6.44721862508630	1.05130300068091	6.95514894815534
O	4.48167240699323	-5.05708875235772	2.60824911924142
O	-3.88425605365290	0.85709189571770	7.57972577750684
O	-2.25440551731876	2.84753722561252	7.13683163083566
O	0.39854795516060	2.66047451524076	6.89193404974380
O	4.25833178591190	-7.39427961994260	1.37314584390842
O	-1.50547935613346	0.86771156704878	8.77821275577836
O	0.71413230812457	0.07439075490781	7.50849419759190
Si	8.12667909644811	-7.12285982649910	2.28660549527571
Si	-2.13195335579253	-2.97551075822618	7.81453334291822
Si	8.19424878631517	-4.41228364628560	3.55268080483970
Si	-4.81369198333145	-2.81278115897406	6.57557014520927
Si	4.52696644575634	-0.88835945706277	3.37738326574770
Si	-8.08480739886357	1.12766995912738	6.87895333545191
Si	5.35569321577190	-3.69987037488790	2.61523613634140
Si	-5.30242507484751	0.07215342034754	7.56819298002108
Si	-0.93152815902236	3.48839293742801	6.44559655855340
Si	3.44163113866290	-6.09911765250934	1.92557329531478
Si	-2.74979842067015	1.78882784964536	8.26683871721845
Si	1.29646751176495	1.32731173119242	6.65145976596664
Si	5.41644395783411	-8.50859653925442	1.64908709138634
Si	-0.51587527160927	-0.38703179005897	8.46975645047319
O	1.79552207269354	4.27275712740364	-4.06429089816697
O	-0.07734093823075	5.27200133433671	-2.48467940049471
O	-0.56786006656307	7.72027089048846	-3.43823468600149
Si	0.69766207446861	3.91830634250719	-2.92088931868641
Si	-1.18702432329727	6.43587578199045	-2.65986219939561
O	1.25266152164457	-1.72670075716966	-4.50729511445991
O	-3.93009836889300	3.97293921445578	-4.55639335437239
O	3.34438776919239	-2.95516400532214	-3.48182359575586
O	-2.44643346487631	5.88332662290205	-3.51268908079137
O	-3.75598743318032	4.17770939832265	-1.91626687898300
O	1.34131019748317	-4.40952051969138	-4.41285791726135
O	-4.63372463489703	1.80795008175496	-5.93931556754822
O	4.72285754061096	-3.30516209061644	-1.25754772699801
O	-2.58525318547194	1.69614736196648	-4.31968360247305

O	-3.14652250535204	-0.34292549004956	-6.54515295030662
O	-1.10469674266035	-1.81517027677917	-5.67736654416847
O	-0.34393442065993	0.32288352878357	-4.25364267852423
O	-0.36623360195250	2.84057529098240	-3.48331282305047
O	1.79410956115205	6.93453410574403	-4.31197435483495
Si	-3.81303957148039	5.02089060140792	-3.31259814588326
Si	2.19577802828932	-3.04097990795681	-4.64054944973602
Si	-4.05327056395024	2.35077054818324	-4.53245882840698
Si	4.01108216154453	-4.07339960552072	-2.50855218991589
Si	-4.34102892038517	0.65689018367439	-7.06144101937121
Si	-0.30460077583031	-1.29908051053828	-4.36590098314050
Si	-1.21732982378137	1.45987628968821	-3.50127115088761
Si	2.55877079552840	5.55956641296553	-4.72207910894755
Si	0.65926637569025	8.08533056557934	-4.45543351653614
O	-2.79171889674171	-5.58035721622114	-7.10944517040387
O	-4.55482098608331	-4.34756883052623	-5.55184802280024
O	-2.94309727563195	-2.91378042665967	-7.21529740133045
O	-2.65747023146363	-0.02287717587801	0.21308411338168
O	-2.51441511363672	2.65713618684601	-0.16327430072737
O	-1.58212166987524	0.94260972976820	-2.00433044284467
O	-5.13566813456272	2.79372488693492	-0.03587820947355
Si	-3.77511123129458	-4.29254351885580	-6.97715968089326
Si	-1.79241835208784	1.22988954685264	-0.43666529774536
Si	-3.79754031150919	3.63624866379798	-0.39132455152553
O	-5.08613220799859	1.98300182744486	-3.33717506969861
O	2.81984998096475	-5.04842338540652	-1.97902789612754
O	-5.64316791763557	-2.50127223131324	2.78695761061070
O	-7.38618163603040	-1.78988489456716	4.67736662770855
O	-0.25693431393185	-6.28226401823949	-3.40107947276331
O	-8.01532327253137	0.19922747934872	3.01340672395370
O	-0.38093082423528	-5.57137585111693	-5.96833828267111
O	-0.95755301604462	-1.94247404875238	-3.03623066040948
O	-3.48374031055327	-6.49583719076356	0.73055119782257
O	-1.00542019128787	-4.25922003919132	-1.83710315906234
O	-6.62456244707026	1.04682181264317	-1.41742249339081
O	0.62375787117912	-6.05865064030022	-0.87276849565068
O	-8.33346872884612	-0.64908624056026	0.49633012928653
O	-7.35615101228552	1.76733792507517	1.01580515494561
O	-1.86996062203036	-6.75338705173994	-1.40365633190756
O	-6.47048362534770	-0.22122473089612	-3.70927111911010
O	2.70210233935204	-5.41619557706949	0.64275323052831
O	-9.08627081224346	-2.65228286812899	-1.04898123062709
O	-4.36147595485175	-0.20289170003896	-2.05904291696878
O	-7.07476699468647	-3.01477722825310	0.62789466337037
O	-8.01300278464027	-2.37447262828078	-3.47242597395247
O	-5.34758988897309	-2.60838718684434	-3.73830197966002
O	-7.26569997035000	-4.36866277211805	-1.89467623983075
O	-5.00212239529744	-5.15740950364375	-3.07395135108757
O	-2.87088339479401	-3.67646646672626	-3.67159749209753
O	-5.69344497341327	-6.15374080470463	-0.71799652157666
O	-2.87484924192373	-2.51802750584460	-1.28457190403147
O	-4.76889439911547	-4.21445753023935	0.93218870318438
O	-4.81506536577631	-1.56288631094958	0.52099378199478
Si	-6.48160799519136	-3.00475863899449	4.07919924647489
Si	0.60201502561728	-5.82749282784708	-4.70670212080887
Si	-8.49585636686253	-0.65400517434546	4.31487871401376
Si	-1.24350538912483	-6.06967948697728	-7.25798518390416
Si	-6.65335798337157	2.28101000180056	-0.36436910756761

Si	-2.44226336959977	-7.48622875200858	-0.05492633714038
Si	-0.63088842652120	-5.82750373844191	-1.88784307041596
Si	-8.42562937227976	0.63448387521533	1.49452290181489
Si	-5.60984486300656	0.61614565980098	-2.61578444968662
Si	2.24742611969158	-6.01772326468000	-0.81177764134711
Si	-8.49848356999204	-2.26286097636986	0.41527186766874
Si	-6.67750051224487	-1.75900770265736	-4.17318612755321
Si	-8.51863747350606	-3.44054866774338	-2.35787588501821
Si	-4.44049498980461	-3.93715188790300	-3.98926983379848
Si	-6.25987014353637	-5.61884291659789	-2.14507377034747
Si	-1.95318349234016	-3.08735063406611	-2.44820738496266
Si	-4.96348463135121	-5.81926752340297	0.70228370071822
Al	-3.74146999069979	-1.13779310843735	-0.77024308159585
Si	-5.56324555093546	-2.81059412179897	1.18555968150490
Si	-2.11345799903052	-1.54808475491552	-6.91836689202411
H	11.67354001758137	-5.53749702797856	-0.03813000583662
H	9.19325800519585	-8.10610401296593	2.36365699928141
H	-3.96156899966641	1.36419899386504	-8.28506099448768
H	-5.56398600181004	-0.12270100097189	-7.29597600031242
H	-6.93632900434749	-1.73777200414861	-5.60525600428146
H	-9.61070198579544	-4.23261598525649	-2.91602398619653
H	-4.80937898924432	-4.38478702634243	-7.98416798320440
H	-1.26201397830561	-7.53013904662510	-7.34713299099746
H	-8.46716099437486	2.53175390796239	6.77435899758271
H	-4.20618399734439	6.50892099485251	2.49272999966108
H	-1.81213398469563	9.00646796201324	0.20177799932959
H	0.23145504731023	10.95011877223132	-2.10809619241913
H	-4.94421499664234	5.93630900063770	-3.34769400129320
H	-7.43440300233355	3.36541400204122	-0.94274899044413
H	-9.81225000782015	1.14342200269177	1.44886400114507
H	-3.26051299521109	2.50761900074273	9.43266798738639
H	-0.74472599711814	4.85510198711069	6.90777200493753
H	1.17261700529757	6.84873798283437	4.60176298991162
H	0.01371300318419	-0.85239399222145	9.75320300190108
H	2.65644498201797	1.61373200263315	7.09816901158624
H	4.53016700028179	3.47572500420661	4.86954700915991
H	1.53638200799062	-6.93834200329964	-4.97336599581331
H	4.68077900878186	9.17388298598967	-0.65420600185600
H	-8.67443703170878	0.53296795597594	8.08822303972547
H	-5.67876700367463	-0.35798101429179	8.92693201348730
H	-5.40538801472372	-3.45794001577360	7.74582402197823
H	-2.86030600168689	-3.03941599351462	9.10082099075129
H	-1.22727299776315	-4.11134500418949	7.70409199004042
H	2.59107099983375	5.38865098289933	-6.17172400653200
H	0.22684599894863	8.11439298542145	-5.85590799661578
H	2.35648395181975	11.66568595168167	-3.24752296420833
H	6.05893600275937	7.26486299334499	-3.76938199658188
H	10.01162899555082	4.23690500277652	-2.76123799664980
H	9.87912999279455	0.42295200016611	-3.59486198969991
H	11.16666300953131	0.78647300051469	-1.60521599862956
H	9.89955900440293	3.90245199615490	-0.38701600218392
H	-1.31531299766141	-1.20647002110991	-8.10616499950993
H	-0.61432202159870	-5.50729801082327	-8.46106597389390
H	2.84834100789940	-3.04401999638022	-5.95043802665939
H	-7.31528198845309	-4.15742499595474	3.75037501040559
H	-9.78237301464790	-1.30797901448682	4.07942699182729
H	-9.40254702441847	-2.75213301685244	1.45267100632421
H	-5.76896401334757	-6.40939902143082	1.78048401383672

H	-3.08137201251825	-8.75069599627546	-0.46757201755735
H	-7.02203601247018	-6.69280402570605	-2.78388201091632
H	2.78306905400030	-7.36900610998245	-0.99232703383668
H	4.89569298347435	-9.57645901010944	2.49579403605975
H	5.81992200898472	-9.04892400030922	0.34487195962097
H	-1.34472298633703	-7.77598299364630	0.87489002556852
H	10.66941399814118	-2.02071803848826	2.56381700599336
H	8.82291801329721	-3.95596104203697	4.78390498556070
H	11.59057498856785	-3.97495100922534	-1.85889598397279
H	5.33835295585668	-0.46465104217578	4.51988993792594
H	7.15208504014972	1.34023101701377	2.37194502526205
H	7.32230799032563	6.57802297408328	-0.51391500102613
H	5.00675698803476	-4.86530399200977	-3.24361798336243
H	7.90182200487825	-7.00149498966131	-1.50647498705813
H	2.44258799135346	-6.50026700616376	2.91141105116214
Ga	-2.95309736965634	-0.42455107186551	2.10875322380975
C	0.45476159646409	-2.48473364373921	2.57036659173252
H	1.51974598792449	-2.70492984862130	2.55695139357604
C	-0.07956990241611	-1.83180845104648	3.64357864703401
H	0.52859333079512	-1.53860207198738	4.49195812748894
C	-0.35484177962462	-2.93031964127210	1.48739198247575
C	-1.71526186977822	-2.94340686129648	1.63726516908670
H	0.10316858889629	-3.40125243027318	0.62387895420924
H	-4.05331783775881	0.41568531405677	2.77636832431024
H	-2.33146348233014	-3.48140568632710	0.92410222617801
C	-1.46992421273733	-1.47774090606207	3.62311402339122
C	-2.36626019572860	-2.28454416464850	2.74414388745744
H	-1.47887914684398	-0.09219837144978	2.97719602242675
H	-1.90904051531718	-1.13563646175690	4.56135287315954
H	-3.20723942719727	-2.79062478807023	3.21697469036884

S2.1.3 Ga^+ site

Ga^+-zeolite

Electronic energy (r^{2}SCAN-3c): -30537.572831120258 E_h
Electronic energy (ωB97X-D4rev/def2-mTZVPP): -30545.62892 E_h
G_{corr} (673.15 K, 1 atm): 0.37104 E_h
Number of atoms: 232

Cartesian coordinates (Å)

O	1.48999900000000	2.89497100000000	1.02816400000000
O	-1.94571200000000	6.76992300000000	1.42422500000000
O	5.26884000000000	2.07946200000000	0.80353300000000
O	3.60785100000000	3.88316800000000	-0.15429600000000
O	6.09379600000000	4.57758500000000	0.42020300000000
O	1.45435800000000	3.28529600000000	-1.61244700000000
O	1.37096000000000	5.32610300000000	0.03161400000000
O	-1.70744400000000	6.88691500000000	-1.19151500000000
O	0.32478900000000	7.70123400000000	0.40885700000000
O	2.93345500000000	7.47845200000000	-0.04666900000000
Si	4.84698800000000	3.64020100000000	0.87037900000000
Si	1.98935300000000	3.84417700000000	-0.18698000000000
Si	-1.29293700000000	7.62883200000000	0.20198000000000
Si	1.65008600000000	6.79247900000000	0.66103700000000
O	8.95206900000000	-3.83459400000000	2.22547500000000
O	1.99927200000000	6.65209700000000	2.24441900000000

O	7.14508600000000	-2.28023900000000	-0.92317400000000
O	2.50347000000000	4.74885200000000	4.08459400000000
O	6.74870900000000	-4.90465400000000	-0.77152800000000
O	0.04377200000000	5.12088900000000	3.14376500000000
O	5.85423700000000	-3.26707400000000	1.13668200000000
O	4.44125100000000	4.07290900000000	2.38816900000000
O	9.40128700000000	-5.01127200000000	-1.12820300000000
O	5.15475900000000	-0.24733400000000	2.01879400000000
O	7.73363900000000	4.37769700000000	-1.68822300000000
O	5.42977900000000	5.63167800000000	-1.91100600000000
O	4.09841500000000	5.67994700000000	-4.18148700000000
O	8.76190500000000	-1.46071700000000	1.04435500000000
O	3.92199900000000	7.73616900000000	-2.52923700000000
O	1.16528900000000	9.51827200000000	-4.04140700000000
O	8.98103100000000	2.07167000000000	-1.88677300000000
O	6.94781700000000	0.19012800000000	0.10282100000000
O	2.27310000000000	9.55408800000000	-1.56326200000000
O	8.99984300000000	-0.54788300000000	-1.44291400000000
Si	1.43267400000000	5.86173000000000	3.55338600000000
Si	6.11564900000000	-3.44040700000000	-0.44910800000000
Si	3.62942900000000	3.62329000000000	3.72719300000000
Si	8.07916700000000	-5.83638100000000	-0.65660600000000
Si	6.67115900000000	5.32648500000000	-0.90799200000000
Si	9.79483400000000	-2.60852600000000	1.56635300000000
Si	4.92576000000000	6.59594200000000	-3.11832600000000
Si	9.20515200000000	3.67304600000000	-1.66749200000000
Si	6.17202200000000	0.85343200000000	1.37304400000000
Si	9.80043400000000	0.68280600000000	-2.15353200000000
Si	7.95512700000000	-1.01658400000000	-0.30263200000000
Si	3.46910700000000	8.51771600000000	-1.17776400000000
O	2.97664500000000	-0.46948900000000	3.61191400000000
O	0.95560500000000	0.76685900000000	2.47394500000000
O	1.24084500000000	0.88448100000000	5.08046800000000
O	2.96549000000000	2.16225200000000	3.48224000000000
O	-0.41267900000000	1.19627100000000	0.27242100000000
O	-0.82169800000000	2.73504500000000	2.35479400000000
O	-3.76723400000000	4.98550900000000	0.57568600000000
O	-2.53634100000000	4.65749700000000	2.92661300000000
O	-1.17845600000000	3.43294300000000	4.83858100000000
Si	2.04213200000000	0.84196100000000	3.67551900000000
Si	0.30464200000000	1.90897300000000	1.52988900000000
Si	-3.13472500000000	5.74647600000000	1.86701000000000
Si	-1.10838400000000	3.99741500000000	3.32790400000000
O	8.28905800000000	-6.04328900000000	3.49632900000000
O	-3.19958700000000	-3.06500100000000	6.58243400000000
O	6.68188900000000	-7.84728300000000	2.44443200000000
O	-1.31121700000000	-1.56713900000000	7.69274600000000
O	8.25756000000000	-6.34262400000000	0.87200100000000
O	-5.42641400000000	-3.48469600000000	5.24633100000000
O	6.65199200000000	-3.92489400000000	3.57201700000000
O	-5.13645700000000	-1.21950600000000	6.59050300000000
O	-8.67001700000000	0.34220000000000	5.57557700000000
O	4.47613900000000	-2.51215800000000	3.25174600000000
O	-6.44618000000000	1.04908400000000	6.95251000000000
O	4.49182500000000	-5.06517300000000	2.60561700000000
O	-3.88616800000000	0.85885100000000	7.57986900000000
O	-2.25347000000000	2.84988300000000	7.14622900000000
O	0.39969600000000	2.65936800000000	6.86157100000000

O	4.25518200000000	-7.39951300000000	1.37539800000000
O	-1.50898900000000	0.86657400000000	8.78622700000000
O	0.71984100000000	0.08492400000000	7.53138500000000
Si	8.12655700000000	-7.12399300000000	2.28701500000000
Si	-2.12804000000000	-2.97387500000000	7.81778100000000
Si	8.20002700000000	-4.41697100000000	3.55230400000000
Si	-4.80729400000000	-2.81442400000000	6.58597200000000
Si	4.52708800000000	-0.89473800000000	3.37734600000000
Si	-8.08184100000000	1.12851800000000	6.87942100000000
Si	5.36038600000000	-3.70480400000000	2.61456800000000
Si	-5.29985600000000	0.06966000000000	7.56815100000000
Si	-0.93664400000000	3.48829800000000	6.44206200000000
Si	3.44306900000000	-6.09950300000000	1.92546100000000
Si	-2.74920400000000	1.78678600000000	8.27067000000000
Si	1.29660200000000	1.31811900000000	6.64660900000000
Si	5.41669300000000	-8.50877900000000	1.64882400000000
Si	-0.51608200000000	-0.38615700000000	8.47528100000000
O	1.81701600000000	4.27206800000000	-4.05369000000000
O	-0.05860200000000	5.26892700000000	-2.46305000000000
O	-0.62421100000000	7.67317400000000	-3.47194700000000
Si	0.71234800000000	3.91755300000000	-2.91779800000000
Si	-1.20113900000000	6.39801900000000	-2.65308100000000
O	1.24673200000000	-1.73373900000000	-4.50099600000000
O	-3.99335100000000	3.94050700000000	-4.54680300000000
O	3.34240100000000	-2.95941500000000	-3.48316100000000
O	-2.44671400000000	5.79416900000000	-3.49099000000000
O	-3.86982700000000	4.16648300000000	-1.91219200000000
O	1.33697600000000	-4.41385300000000	-4.41704700000000
O	-4.66932200000000	1.77065400000000	-5.91872500000000
O	4.72061000000000	-3.30733500000000	-1.25790800000000
O	-2.54079800000000	1.72115000000000	-4.40347500000000
O	-3.17106100000000	-0.37000500000000	-6.53635500000000
O	-1.11507200000000	-1.80088800000000	-5.65633800000000
O	-0.32505500000000	0.32792900000000	-4.22105100000000
O	-0.35307800000000	2.85919700000000	-3.50719200000000
O	1.78205800000000	6.92737400000000	-4.27164600000000
Si	-3.84949300000000	4.99404200000000	-3.31314400000000
Si	2.19317200000000	-3.04577000000000	-4.63988700000000
Si	-4.04335600000000	2.31458100000000	-4.53330600000000
Si	4.00853000000000	-4.07685600000000	-2.50753100000000
Si	-4.32446600000000	0.66459200000000	-7.07165600000000
Si	-0.30480500000000	-1.29408300000000	-4.34530000000000
Si	-1.21110400000000	1.48511700000000	-3.51928800000000
Si	2.55993100000000	5.56876700000000	-4.71165100000000
Si	0.62338100000000	8.04885600000000	-4.45446900000000
O	-2.79770800000000	-5.59428700000000	-7.11958200000000
O	-4.50973500000000	-4.33152200000000	-5.53479000000000
O	-2.91195800000000	-2.93077300000000	-7.22793400000000
O	-2.85974800000000	0.16894900000000	0.16618900000000
O	-2.47726400000000	2.78739200000000	-0.15941100000000
O	-1.65552500000000	1.03229000000000	-2.02373800000000
O	-5.12062600000000	2.77974100000000	0.02129900000000
Si	-3.76445300000000	-4.29517000000000	-6.97863700000000
Si	-1.85930600000000	1.30691900000000	-0.44623500000000
Si	-3.82009700000000	3.68039800000000	-0.37669000000000
O	-4.98676200000000	1.88699000000000	-3.28615000000000
O	2.81604200000000	-5.05228800000000	-1.98250800000000
O	-5.60334200000000	-2.51606400000000	2.81981800000000

O	-7.38102900000000	-1.79061100000000	4.66889500000000
O	-0.25236900000000	-6.30448200000000	-3.40253400000000
O	-8.00587900000000	0.18795900000000	3.00612800000000
O	-0.39300600000000	-5.57370900000000	-5.96216500000000
O	-0.96066000000000	-1.91683200000000	-3.00805300000000
O	-3.48843000000000	-6.50655500000000	0.73156900000000
O	-0.96456400000000	-4.26601600000000	-1.86475000000000
O	-6.57734000000000	1.03363900000000	-1.38816200000000
O	0.62672700000000	-6.08491600000000	-0.87289300000000
O	-8.34600300000000	-0.63832000000000	0.48732200000000
O	-7.34641400000000	1.76219000000000	1.02139800000000
O	-1.87463500000000	-6.74480700000000	-1.40402200000000
O	-6.53254200000000	-0.21355600000000	-3.70539000000000
O	2.70841800000000	-5.41939300000000	0.64027600000000
O	-9.07576400000000	-2.65887000000000	-1.04132100000000
O	-4.45362100000000	-0.41867400000000	-2.08508600000000
O	-7.03982200000000	-2.95590000000000	0.63197200000000
O	-8.04665700000000	-2.37681300000000	-3.48298200000000
O	-5.39365200000000	-2.61844300000000	-3.74174900000000
O	-7.26292500000000	-4.36378800000000	-1.89304200000000
O	-5.00487200000000	-5.15683500000000	-3.07522900000000
O	-2.90448500000000	-3.61265100000000	-3.58343400000000
O	-5.69977800000000	-6.15329800000000	-0.71696000000000
O	-2.75141300000000	-2.50624500000000	-1.14295900000000
O	-4.76194600000000	-4.20808700000000	0.90453100000000
O	-4.74838300000000	-1.52112100000000	0.63435600000000
Si	-6.47410200000000	-3.01736500000000	4.10170900000000
Si	0.59922100000000	-5.83324000000000	-4.70655000000000
Si	-8.49409000000000	-0.65672700000000	4.31572000000000
Si	-1.24374900000000	-6.06997000000000	-7.26015000000000
Si	-6.63733900000000	2.28318400000000	-0.35848800000000
Si	-2.43904500000000	-7.48696100000000	-0.05807500000000
Si	-0.61987800000000	-5.83836600000000	-1.89024400000000
Si	-8.42983300000000	0.63699400000000	1.49443500000000
Si	-5.60716300000000	0.54385500000000	-2.60641200000000
Si	2.24945100000000	-6.02072000000000	-0.81040800000000
Si	-8.49256600000000	-2.25457500000000	0.41688900000000
Si	-6.70934200000000	-1.75630000000000	-4.17557600000000
Si	-8.52092000000000	-3.44447000000000	-2.35875600000000
Si	-4.44564500000000	-3.91845100000000	-3.97022600000000
Si	-6.26015900000000	-5.61867200000000	-2.14668500000000
Si	-1.92574600000000	-3.07125300000000	-2.38600100000000
Si	-4.95938900000000	-5.82291900000000	0.69804100000000
Al	-3.68614100000000	-1.14001500000000	-0.74203600000000
Si	-5.53636400000000	-2.82056100000000	1.22116200000000
Si	-2.11161100000000	-1.55180500000000	-6.91108600000000
H	9.19348300000000	-8.10585800000000	2.36346300000000
H	-3.88155000000000	1.39981100000000	-8.25725800000000
H	-5.54661900000000	-0.07985100000000	-7.37196300000000
H	-6.93206200000000	-1.73335100000000	-5.60666700000000
H	-9.61083500000000	-4.23371600000000	-2.91527900000000
H	-4.80806200000000	-4.38616400000000	-7.98466800000000
H	-1.25558100000000	-7.52827100000000	-7.35483800000000
H	-8.46603500000000	2.53218800000000	6.77163500000000
H	-4.20950400000000	6.50518800000000	2.49497300000000
H	-1.81143200000000	9.00591900000000	0.20164700000000
H	-4.95337000000000	5.93157200000000	-3.34598500000000
H	-7.43212800000000	3.36467300000000	-0.94325500000000

H	-9.81433500000000	1.14100200000000	1.45030700000000
H	-3.26082300000000	2.50672900000000	9.43326900000000
H	-0.74504100000000	4.85495100000000	6.90563300000000
H	1.17176500000000	6.84606700000000	4.60638300000000
H	0.01383700000000	-0.85268900000000	9.75320600000000
H	2.65630400000000	1.61378700000000	7.09828800000000
H	4.53076900000000	3.47379800000000	4.86924400000000
H	1.53720800000000	-6.93798300000000	-4.97365700000000
H	4.63541500000000	9.21535600000000	-0.64625200000000
H	-8.67488800000000	0.53574600000000	8.08886500000000
H	-5.67875100000000	-0.35844200000000	8.92691800000000
H	-5.40516100000000	-3.45826600000000	7.74597000000000
H	-2.86007300000000	-3.03986700000000	9.10083200000000
H	-1.22701000000000	-4.11162700000000	7.70400900000000
H	2.57413300000000	5.42807900000000	-6.16363200000000
H	0.22482900000000	8.10275900000000	-5.85603200000000
H	6.06067100000000	7.26917500000000	-3.76691100000000
H	10.01065000000000	4.23779400000000	-2.76113100000000
H	9.87926500000000	0.42371400000000	-3.59460900000000
H	11.16639500000000	0.78744100000000	-1.60472500000000
H	9.89847200000000	3.90341200000000	-0.38688400000000
H	-1.32399900000000	-1.19594400000000	-8.10330100000000
H	-0.61662900000000	-5.49814400000000	-8.46011600000000
H	2.84818300000000	-3.04332200000000	-5.95038400000000
H	-7.31517900000000	-4.15812800000000	3.75060700000000
H	-9.78250000000000	-1.30915800000000	4.07997000000000
H	-9.40319700000000	-2.75383200000000	1.45013700000000
H	-5.76898900000000	-6.41021300000000	1.77986100000000
H	-3.08084500000000	-8.75083000000000	-0.46797100000000
H	-7.02180600000000	-6.69287800000000	-2.78419800000000
H	2.78359000000000	-7.36886300000000	-0.99247300000000
H	4.89691600000000	-9.57738000000000	2.49409400000000
H	5.82084500000000	-9.04713700000000	0.34374500000000
H	-1.34437700000000	-7.77605500000000	0.87467400000000
H	8.82314100000000	-3.95574100000000	4.78387700000000
H	5.33815600000000	-0.46451100000000	4.51995500000000
H	7.15219500000000	1.34004700000000	2.37234700000000
H	7.32251200000000	6.57841100000000	-0.51330700000000
H	5.00684300000000	-4.86484800000000	-3.24385800000000
H	7.90214800000000	-7.00114300000000	-1.50659200000000
H	2.44258300000000	-6.49974700000000	2.91143200000000
Ga	-4.07775400000000	0.13280900000000	1.97859600000000

Complex (Ga-modified-zeolite + C_2H_6)

Electronic energy (r^2SCAN-3c): -30617.377992892725 E_h
Electronic energy (ωB97X-D4rev/def2-mTZVPP): -30625.54306 E_h
G_{corr} (673.15 K, 1 atm): 0.42707 E_h
Number of atoms: 240

Cartesian coordinates (Å)

```
O        1.48436500000000     2.88883700000000     1.02502500000000
O       -1.94435900000000     6.77204100000000     1.42564500000000
O        5.26761300000000     2.07881900000000     0.80550700000000
O        3.60508900000000     3.88098800000000    -0.15158300000000
O        6.09347100000000     4.57611100000000     0.41814000000000
O        1.45381800000000     3.28471700000000    -1.61360800000000
O        1.36893800000000     5.32318300000000     0.03520300000000
O       -1.70700100000000     6.88681400000000    -1.19086600000000
O        0.32581000000000     7.70094000000000     0.40822900000000
O        2.93346700000000     7.47550500000000    -0.04891500000000
Si       4.84668200000000     3.64001800000000     0.87033800000000
Si       1.98647600000000     3.84114800000000    -0.18666800000000
Si      -1.29195700000000     7.62941100000000     0.20187100000000
Si       1.65024500000000     6.79093100000000     0.66048400000000
O        8.95264600000000    -3.83487800000000     2.22550600000000
O        1.99953700000000     6.65255000000000     2.24434900000000
O        7.14406700000000    -2.27932200000000    -0.92304600000000
O        2.50286300000000     4.74740400000000     4.08319800000000
O        6.74888700000000    -4.90411000000000    -0.77292600000000
O        0.04315900000000     5.12155700000000     3.14420900000000
O        5.85411000000000    -3.26826000000000     1.13632200000000
O        4.44443500000000     4.07608100000000     2.38838000000000
O        9.40139800000000    -5.01147900000000    -1.12776400000000
O        5.15836400000000    -0.25016800000000     2.01770300000000
O        7.73392200000000     4.37824200000000    -1.68926900000000
O        5.42927900000000     5.63209100000000    -1.91122900000000
O        4.09775400000000     5.67966100000000    -4.18134200000000
O        8.76145800000000    -1.46100200000000     1.04433200000000
O        3.92250300000000     7.73725500000000    -2.53027000000000
O        1.16534900000000     9.51799000000000    -4.04103200000000
O        8.98100700000000     2.07154900000000    -1.88650400000000
O        6.94947500000000     0.19197200000000     0.10180300000000
O        2.27316200000000     9.55399800000000    -1.56280100000000
O        9.00053600000000    -0.54848000000000    -1.44312100000000
Si       1.43289100000000     5.86188500000000     3.55292300000000
Si       6.11511000000000    -3.44035600000000    -0.44984600000000
Si       3.63113000000000     3.62409100000000     3.72536900000000
Si       8.07888400000000    -5.83625000000000    -0.65675700000000
Si       6.67109700000000     5.32657200000000    -0.90900800000000
Si       9.79486600000000    -2.60850500000000     1.56623900000000
Si       4.92541700000000     6.59607000000000    -3.11896700000000
Si       9.20518500000000     3.67300800000000    -1.66762300000000
Si       6.17309400000000     0.85336000000000     1.37247500000000
Si       9.80053000000000     0.68274900000000    -2.15358200000000
Si       7.95534500000000    -1.01635900000000    -0.30297200000000
Si       3.46916200000000     8.51723100000000    -1.17811500000000
O        2.97822600000000    -0.47047900000000     3.60675000000000
O        0.95435900000000     0.76378100000000     2.47524600000000
O        1.25271700000000     0.88792600000000     5.07944400000000
O        2.96946700000000     2.16271700000000     3.47328400000000
```

O	-0.42436800000000	1.18785100000000	0.27972500000000
O	-0.82216000000000	2.73336700000000	2.35830900000000
O	-3.76917300000000	4.99383800000000	0.57183900000000
O	-2.53731200000000	4.65642000000000	2.92225100000000
O	-1.18272100000000	3.43481000000000	4.83781600000000
Si	2.04685600000000	0.84295600000000	3.67088900000000
Si	0.29945100000000	1.90361700000000	1.52961800000000
Si	-3.13463200000000	5.74907200000000	1.86527000000000
Si	-1.10924000000000	3.99861700000000	3.32683700000000
O	8.29008300000000	-6.04385000000000	3.49657000000000
O	-3.19925300000000	-3.06672800000000	6.58200100000000
O	6.68178700000000	-7.84749100000000	2.44487400000000
O	-1.31022100000000	-1.56995700000000	7.69574500000000
O	8.25581400000000	-6.34195600000000	0.87228300000000
O	-5.42167900000000	-3.48023100000000	5.23940200000000
O	6.65246900000000	-3.92628800000000	3.57166800000000
O	-5.13327700000000	-1.21913700000000	6.59024300000000
O	-8.67122900000000	0.34024400000000	5.57729500000000
O	4.47732900000000	-2.51352800000000	3.25242100000000
O	-6.44646600000000	1.04805400000000	6.95111900000000
O	4.49158400000000	-5.06602200000000	2.60591600000000
O	-3.88667200000000	0.85887200000000	7.57877500000000
O	-2.25430200000000	2.84899800000000	7.14490100000000
O	0.39831100000000	2.65893300000000	6.85866500000000
O	4.25508900000000	-7.39986400000000	1.37528000000000
O	-1.50989800000000	0.86582000000000	8.78549200000000
O	0.71449600000000	0.08317100000000	7.52286500000000
Si	8.12637900000000	-7.12418000000000	2.28698400000000
Si	-2.12874100000000	-2.97541200000000	7.81844000000000
Si	8.20074300000000	-4.41759900000000	3.55219000000000
Si	-4.80799500000000	-2.81529700000000	6.58308100000000
Si	4.52939500000000	-0.89622300000000	3.37596700000000
Si	-8.08272300000000	1.12818500000000	6.87924800000000
Si	5.36097700000000	-3.70637400000000	2.61417400000000
Si	-5.30049000000000	0.06978900000000	7.56820900000000
Si	-0.93821700000000	3.48849200000000	6.44058000000000
Si	3.44274900000000	-6.09993700000000	1.92490400000000
Si	-2.74968700000000	1.78680200000000	8.26968200000000
Si	1.29756400000000	1.31963800000000	6.64530800000000
Si	5.41669100000000	-8.50889800000000	1.64882800000000
Si	-0.51649000000000	-0.38597400000000	8.47503400000000
O	1.81597500000000	4.27174100000000	-4.05421200000000
O	-0.05732100000000	5.26989100000000	-2.46237000000000
O	-0.62464300000000	7.67332200000000	-3.47184200000000
Si	0.71087000000000	3.91710500000000	-2.91852800000000
Si	-1.20075600000000	6.39790400000000	-2.65263200000000
O	1.24715200000000	-1.73161800000000	-4.50393800000000
O	-3.99200600000000	3.94147200000000	-4.54885300000000
O	3.34172500000000	-2.95887400000000	-3.48321700000000
O	-2.44639200000000	5.79350500000000	-3.48963300000000
O	-3.87464700000000	4.16590800000000	-1.91392000000000
O	1.33696100000000	-4.41288300000000	-4.41774500000000
O	-4.66846500000000	1.77202500000000	-5.92057400000000
O	4.72000200000000	-3.30729100000000	-1.25818600000000
O	-2.54323100000000	1.71983200000000	-4.39997600000000
O	-3.17149600000000	-0.37021100000000	-6.53663000000000
O	-1.11685300000000	-1.79953200000000	-5.65383400000000
O	-0.32683300000000	0.32848900000000	-4.21634800000000

O	-0.35593400000000	2.86054700000000	-3.50762400000000
O	1.78184200000000	6.92726300000000	-4.27147100000000
Si	-3.84979500000000	4.99402600000000	-3.31470000000000
Si	2.19256000000000	-3.04448300000000	-4.64049300000000
Si	-4.04435300000000	2.31524400000000	-4.53410300000000
Si	4.00780800000000	-4.07671000000000	-2.50818900000000
Si	-4.32438300000000	0.66453000000000	-7.07206400000000
Si	-0.30483400000000	-1.29339900000000	-4.34324500000000
Si	-1.21380100000000	1.48530500000000	-3.51447000000000
Si	2.55918800000000	5.56832800000000	-4.71168200000000
Si	0.62315200000000	8.04867400000000	-4.45435300000000
O	-2.79791700000000	-5.59425800000000	-7.12112500000000
O	-4.50268500000000	-4.32817500000000	-5.53169000000000
O	-2.91003500000000	-2.93070400000000	-7.23017100000000
O	-2.88656700000000	0.18913400000000	0.17131000000000
O	-2.47479500000000	2.79772600000000	-0.15667100000000
O	-1.65631700000000	1.03778300000000	-2.01790000000000
O	-5.12121400000000	2.78660100000000	0.02642000000000
Si	-3.76339300000000	-4.29418000000000	-6.97834600000000
Si	-1.87139800000000	1.30785300000000	-0.44007400000000
Si	-3.81916400000000	3.68605400000000	-0.37722600000000
O	-4.99287400000000	1.89062200000000	-3.29002300000000
O	2.81575700000000	-5.05237000000000	-1.98273700000000
O	-5.60457700000000	-2.50552100000000	2.81318200000000
O	-7.37911800000000	-1.78929700000000	4.66705100000000
O	-0.25292500000000	-6.30091900000000	-3.40152100000000
O	-8.00727100000000	0.19454100000000	3.00941000000000
O	-0.39360300000000	-5.57404100000000	-5.96180300000000
O	-0.95397200000000	-1.91919600000000	-3.00505400000000
O	-3.48867800000000	-6.50695400000000	0.73216200000000
O	-0.96683600000000	-4.26529400000000	-1.85747200000000
O	-6.56310900000000	1.02942500000000	-1.38233100000000
O	0.62653500000000	-6.08542700000000	-0.87243900000000
O	-8.33178500000000	-0.64564600000000	0.49436300000000
O	-7.34606700000000	1.76156600000000	1.01976500000000
O	-1.87456000000000	-6.74601900000000	-1.40409000000000
O	-6.53366700000000	-0.21442300000000	-3.70101000000000
O	2.70853300000000	-5.41934300000000	0.64002100000000
O	-9.07422400000000	-2.65494400000000	-1.04306900000000
O	-4.43698300000000	-0.41669600000000	-2.09932700000000
O	-7.05450600000000	-2.98762900000000	0.64635000000000
O	-8.05043200000000	-2.37613000000000	-3.48618400000000
O	-5.39746700000000	-2.61965700000000	-3.74040500000000
O	-7.26287800000000	-4.36215300000000	-1.89615400000000
O	-5.00473400000000	-5.15755200000000	-3.07591700000000
O	-2.90702200000000	-3.60563800000000	-3.57072100000000
O	-5.69975400000000	-6.14926800000000	-0.71589900000000
O	-2.73915400000000	-2.49256900000000	-1.12977400000000
O	-4.75828600000000	-4.20728900000000	0.91063900000000
O	-4.79542200000000	-1.52085100000000	0.59656800000000
Si	-6.47155300000000	-3.01436200000000	4.09379700000000
Si	0.59897300000000	-5.83242700000000	-4.70615100000000
Si	-8.49394500000000	-0.65671800000000	4.31569200000000
Si	-1.24390000000000	-6.06994100000000	-7.26022900000000
Si	-6.63535000000000	2.28336600000000	-0.36028200000000
Si	-2.43961800000000	-7.48684900000000	-0.05772900000000
Si	-0.62119800000000	-5.83683200000000	-1.88859900000000
Si	-8.42875200000000	0.63429400000000	1.49390000000000

Si	-5.59892000000000	0.54126200000000	-2.60823500000000
Si	2.24884500000000	-6.02084800000000	-0.81081400000000
Si	-8.49151300000000	-2.26200900000000	0.41856800000000
Si	-6.71110200000000	-1.75585500000000	-4.17510300000000
Si	-8.52143100000000	-3.44311000000000	-2.36010100000000
Si	-4.44455600000000	-3.91611700000000	-3.96625900000000
Si	-6.26015000000000	-5.61778700000000	-2.14671000000000
Si	-1.92423700000000	-3.06528000000000	-2.37548900000000
Si	-4.95881900000000	-5.82037600000000	0.69962200000000
Al	-3.69046000000000	-1.13366300000000	-0.73612900000000
Si	-5.54352700000000	-2.82117000000000	1.21325000000000
Si	-2.11118100000000	-1.55144500000000	-6.91053400000000
H	9.19348300000000	-8.10585800000000	2.36346300000000
H	-3.88155000000000	1.39981100000000	-8.25725800000000
H	-5.54661900000000	-0.07985100000000	-7.37196300000000
H	-6.93206200000000	-1.73335100000000	-5.60666700000000
H	-9.61083500000000	-4.23371600000000	-2.91527900000000
H	-4.80806200000000	-4.38616400000000	-7.98466800000000
H	-1.25558100000000	-7.52827100000000	-7.35483800000000
H	-8.46603500000000	2.53218800000000	6.77163500000000
H	-4.20950400000000	6.50518800000000	2.49497300000000
H	-1.81143200000000	9.00591900000000	0.20164700000000
H	-4.95337000000000	5.93157200000000	-3.34598500000000
H	-7.43212800000000	3.36467300000000	-0.94325500000000
H	-9.81433500000000	1.14100200000000	1.45030700000000
H	-3.26082300000000	2.50672900000000	9.43326900000000
H	-0.74504100000000	4.85495100000000	6.90563300000000
H	1.17176500000000	6.84606700000000	4.60638300000000
H	0.01383700000000	-0.85268900000000	9.75320600000000
H	2.65630400000000	1.61378700000000	7.09828800000000
H	4.53076900000000	3.47379800000000	4.86924400000000
H	1.53720800000000	-6.93798300000000	-4.97365700000000
H	4.63541500000000	9.21535600000000	-0.64625200000000
H	-8.67488800000000	0.53574600000000	8.08886500000000
H	-5.67875100000000	-0.35844200000000	8.92691800000000
H	-5.40516100000000	-3.45826600000000	7.74597000000000
H	-2.86007300000000	-3.03986700000000	9.10083200000000
H	-1.22701000000000	-4.11162700000000	7.70400900000000
H	2.57413300000000	5.42807900000000	-6.16363200000000
H	0.22482900000000	8.10275900000000	-5.85603200000000
H	6.06067100000000	7.26917500000000	-3.76691100000000
H	10.01065000000000	4.23779400000000	-2.76113100000000
H	9.87926500000000	0.42371400000000	-3.59460900000000
H	11.16639500000000	0.78744100000000	-1.60472500000000
H	9.89847200000000	3.90341200000000	-0.38688400000000
H	-1.32399900000000	-1.19594400000000	-8.10330100000000
H	-0.61662900000000	-5.49814400000000	-8.46011600000000
H	2.84818300000000	-3.04332200000000	-5.95038400000000
H	-7.31517900000000	-4.15812800000000	3.75060700000000
H	-9.78250000000000	-1.30915800000000	4.07997000000000
H	-9.40319700000000	-2.75383200000000	1.45013700000000
H	-5.76898900000000	-6.41021300000000	1.77986100000000
H	-3.08084500000000	-8.75083000000000	-0.46797100000000
H	-7.02180600000000	-6.69287800000000	-2.78419800000000
H	2.78359000000000	-7.36886300000000	-0.99247300000000
H	4.89691600000000	-9.57738000000000	2.49409400000000
H	5.82084500000000	-9.04713700000000	0.34374500000000
H	-1.34437700000000	-7.77605500000000	0.87467400000000

H	8.82314100000000	-3.95574100000000	4.78387700000000
H	5.33815600000000	-0.46451100000000	4.51995500000000
H	7.15219500000000	1.34004700000000	2.37234700000000
H	7.32251200000000	6.57841100000000	-0.51330700000000
H	5.00684300000000	-4.86484800000000	-3.24385800000000
H	7.90214800000000	-7.00114300000000	-1.50659200000000
H	2.44258300000000	-6.49974700000000	2.91143200000000
Ga	-4.25428600000000	0.28465400000000	1.95227800000000
C	-1.92697100000000	-0.49835600000000	4.32331700000000
H	-2.89104300000000	-0.47656200000000	4.84434500000000
H	-1.81090000000000	0.44774700000000	3.78007300000000
H	-1.14333700000000	-0.52462300000000	5.08618700000000
C	-1.80902500000000	-1.70556100000000	3.39663800000000
H	-1.91255600000000	-2.64151000000000	3.95404900000000
H	-0.84708000000000	-1.71608800000000	2.87486900000000
H	-2.59056400000000	-1.72062400000000	2.62244200000000

TS1-Ga

Electronic energy (r^2SCAN-3c): -30617.265703001296 E_h
Electronic energy (ωB97X-D4rev/def2-mTZVPP): -30625.47624 E_h
G_corr (673.15 K, 1 atm): 0.42982 E_h
Number of atoms: 240

Cartesian coordinates (Å)

O	1.51435300000000	2.90701400000000	0.82828500000000
O	-1.98810400000000	6.85127700000000	1.26948000000000
O	5.23448400000000	2.15888900000000	0.65249700000000
O	3.59642200000000	3.98116100000000	-0.31923600000000
O	6.07452200000000	4.65527100000000	0.27521500000000
O	1.44233300000000	3.37484900000000	-1.78289300000000
O	1.33408100000000	5.37675300000000	-0.08221800000000
O	-1.75123300000000	6.96978400000000	-1.34279400000000
O	0.28963900000000	7.76434100000000	0.25204000000000
O	2.89448000000000	7.53529400000000	-0.22150600000000
Si	4.82282500000000	3.72192000000000	0.71760800000000
Si	1.97989900000000	3.91425600000000	-0.35285800000000
Si	-1.32939300000000	7.70796300000000	0.04982300000000
Si	1.61786000000000	6.86050600000000	0.50821200000000
O	8.91746800000000	-3.75602400000000	2.07777300000000
O	1.98096400000000	6.75117800000000	2.09231200000000
O	7.10838500000000	-2.20128000000000	-1.07236100000000
O	2.46300200000000	4.82760200000000	3.92075000000000
O	6.71216600000000	-4.82548400000000	-0.92032200000000
O	-0.00177800000000	5.26454600000000	3.00046300000000
O	5.81775000000000	-3.18743400000000	0.98725500000000
O	4.40718500000000	4.15074200000000	2.23478300000000
O	9.36477600000000	-4.93117700000000	-1.27558300000000
O	5.11791100000000	-0.16574600000000	1.87031600000000
O	7.69876800000000	4.45951500000000	-1.84282400000000
O	5.39365100000000	5.71706300000000	-2.05363300000000
O	4.04757800000000	5.74319000000000	-4.31116900000000
O	8.72530400000000	-1.38263500000000	0.89620300000000
O	3.89285000000000	7.82601900000000	-2.68900400000000
O	1.13053400000000	9.59820500000000	-4.18971800000000
O	8.94448200000000	2.15088800000000	-2.03393600000000
O	6.91272200000000	0.26862000000000	-0.04569000000000

O	2.23738700000000	9.63304000000000	-1.71097400000000
O	8.96429800000000	-0.46903000000000	-1.59099200000000
Si	1.40980700000000	5.96182700000000	3.39936900000000
Si	6.07819000000000	-3.36165400000000	-0.59902100000000
Si	3.59582400000000	3.70454700000000	3.57472800000000
Si	8.04269400000000	-5.75691400000000	-0.80451300000000
Si	6.63942700000000	5.40705800000000	-1.05754200000000
Si	9.75932800000000	-2.52974200000000	1.41831800000000
Si	4.88773800000000	6.67256100000000	-3.26763400000000
Si	9.16954300000000	3.75225900000000	-1.81627900000000
Si	6.13741000000000	0.93252100000000	1.22351000000000
Si	9.76435100000000	0.76200200000000	-2.30147200000000
Si	7.91951100000000	-0.93907500000000	-0.45141800000000
Si	3.43450100000000	8.59505500000000	-1.33275300000000
O	2.94352200000000	-0.39057800000000	3.46554600000000
O	0.91648600000000	0.84503700000000	2.33631100000000
O	1.20478400000000	0.96025500000000	4.93422800000000
O	2.92834900000000	2.24354000000000	3.33601200000000
O	-0.47964400000000	1.25436000000000	0.16281700000000
O	-0.77557400000000	2.88777200000000	2.17887000000000
O	-3.81973100000000	5.06927500000000	0.42677900000000
O	-2.56597600000000	4.73852000000000	2.76864600000000
O	-1.19290900000000	3.52734800000000	4.67654700000000
Si	2.01167700000000	0.92045300000000	3.53251700000000
Si	0.29818800000000	1.98910400000000	1.37128700000000
Si	-3.17415800000000	5.82825300000000	1.71496500000000
Si	-1.12295500000000	4.11317700000000	3.17083600000000
O	8.25291800000000	-5.96424600000000	3.34870400000000
O	-3.24136400000000	-2.97915700000000	6.43554700000000
O	6.64556600000000	-7.76789100000000	2.29655900000000
O	-1.35144600000000	-1.48535500000000	7.54329000000000
O	8.22070600000000	-6.26232700000000	0.72445400000000
O	-5.44977200000000	-3.38412800000000	5.07477400000000
O	6.61667900000000	-3.84498100000000	3.42261800000000
O	-5.18418500000000	-1.14056600000000	6.45088300000000
O	-8.69763800000000	0.38821700000000	5.44965600000000
O	4.44133700000000	-2.43336900000000	3.10241800000000
O	-6.47920800000000	1.13953300000000	6.81068500000000
O	4.45583200000000	-4.98652000000000	2.45759100000000
O	-3.91971900000000	0.93709700000000	7.42215400000000
O	-2.28888800000000	2.91853300000000	6.97533000000000
O	0.36440500000000	2.73854700000000	6.70903800000000
O	4.21903000000000	-7.32035000000000	1.22752000000000
O	-1.54414400000000	0.94989800000000	8.63446400000000
O	0.68305200000000	0.16417600000000	7.38471900000000
Si	8.09002300000000	-7.04481800000000	2.13899300000000
Si	-2.16710700000000	-2.89420600000000	7.66819900000000
Si	8.16454300000000	-4.33813100000000	3.40419500000000
Si	-4.85239200000000	-2.73895100000000	6.43129300000000
Si	4.49429400000000	-0.81714800000000	3.22831100000000
Si	-8.11818800000000	1.20570000000000	6.73338900000000
Si	5.32503600000000	-3.62730500000000	2.46473000000000
Si	-5.33943000000000	0.15313000000000	7.42365200000000
Si	-0.96774000000000	3.57027800000000	6.28536800000000
Si	3.40624100000000	-6.02031900000000	1.77599300000000
Si	-2.78460500000000	1.86738300000000	8.11651500000000
Si	1.26081700000000	1.39552400000000	6.50027500000000
Si	5.38024300000000	-8.42951400000000	1.50072900000000

Si	-0.55430300000000	-0.30758400000000	8.32688200000000
O	1.74219000000000	4.34711000000000	-4.23078300000000
O	-0.09583000000000	5.34983200000000	-2.60192700000000
O	-0.65856900000000	7.75453200000000	-3.61840300000000
Si	0.66144200000000	3.99424700000000	-3.07080700000000
Si	-1.23691400000000	6.48002400000000	-2.80092800000000
O	1.23042500000000	-1.62667700000000	-4.69082100000000
O	-4.01403700000000	4.03080000000000	-4.71068100000000
O	3.30285800000000	-2.87308000000000	-3.62807500000000
O	-2.47986400000000	5.87505200000000	-3.64015900000000
O	-3.92638300000000	4.25162400000000	-2.06972900000000
O	1.29819500000000	-4.31868400000000	-4.56911300000000
O	-4.69610100000000	1.86175300000000	-6.08040000000000
O	4.68349800000000	-3.22764100000000	-1.40722700000000
O	-2.61724800000000	1.78347700000000	-4.49266200000000
O	-3.20497100000000	-0.28755700000000	-6.68316800000000
O	-1.15694500000000	-1.72352500000000	-5.80136800000000
O	-0.38287500000000	0.40621800000000	-4.36279200000000
O	-0.42203400000000	2.92913200000000	-3.61603300000000
O	1.74510800000000	7.00622700000000	-4.41756900000000
Si	-3.88484100000000	5.07857300000000	-3.47304000000000
Si	2.16040300000000	-2.95488800000000	-4.79288100000000
Si	-4.10214800000000	2.40582300000000	-4.68290800000000
Si	3.96974800000000	-3.99563500000000	-2.65786600000000
Si	-4.35885800000000	0.74358900000000	-7.22240700000000
Si	-0.32670500000000	-1.21612200000000	-4.50294800000000
Si	-1.27094900000000	1.54584600000000	-3.63764400000000
Si	2.51372100000000	5.64300800000000	-4.86026300000000
Si	0.58822400000000	8.12920100000000	-4.60265100000000
O	-2.83270800000000	-5.51051900000000	-7.26816500000000
O	-4.50536300000000	-4.22826000000000	-5.65929200000000
O	-2.94714900000000	-2.84644200000000	-7.39160000000000
O	-2.98641600000000	0.33639500000000	0.08793200000000
O	-2.49666200000000	2.90161900000000	-0.33134600000000
O	-1.68424200000000	1.07047200000000	-2.13487500000000
O	-5.14103800000000	2.83868300000000	-0.12706200000000
Si	-3.79710200000000	-4.20931400000000	-7.12173100000000
Si	-1.90973200000000	1.40056500000000	-0.57647000000000
Si	-3.85862900000000	3.77379900000000	-0.53656000000000
O	-5.10447800000000	2.01851100000000	-3.47034700000000
O	2.77890500000000	-4.97267900000000	-2.13312100000000
O	-5.60721700000000	-2.49757400000000	2.61627100000000
O	-7.38144200000000	-1.69477400000000	4.45964500000000
O	-0.29751800000000	-6.18308900000000	-3.54420200000000
O	-8.06542600000000	0.32571600000000	2.88325400000000
O	-0.42507300000000	-5.49385500000000	-6.11213000000000
O	-0.91737100000000	-1.87515500000000	-3.15975900000000
O	-3.52942200000000	-6.44819400000000	0.58753400000000
O	-1.03452200000000	-4.17975200000000	-1.94142900000000
O	-6.61135400000000	1.10874300000000	-1.53559500000000
O	0.59068800000000	-6.00318300000000	-1.01928500000000
O	-8.32883000000000	-0.58672400000000	0.37884800000000
O	-7.38695800000000	1.84963700000000	0.87089300000000
O	-1.90797000000000	-6.67582400000000	-1.55302800000000
O	-6.56907400000000	-0.13973300000000	-3.84218500000000
O	2.67537700000000	-5.33864400000000	0.49012900000000
O	-9.10038600000000	-2.55584800000000	-1.19873700000000
O	-4.41508900000000	-0.23238000000000	-2.26740300000000

O	-7.14830500000000	-3.01238900000000	0.54635300000000
O	-8.08668200000000	-2.30822700000000	-3.64421000000000
O	-5.42980600000000	-2.52809600000000	-3.87410200000000
O	-7.29709800000000	-4.28610900000000	-2.04100900000000
O	-5.03745800000000	-5.07153400000000	-3.21598100000000
O	-2.94874000000000	-3.48773200000000	-3.65689400000000
O	-5.74065000000000	-6.11099300000000	-0.87428700000000
O	-2.68989000000000	-2.27482300000000	-1.25128900000000
O	-4.78982700000000	-4.15246500000000	0.70188000000000
O	-4.97407400000000	-1.50792200000000	0.27318500000000
Si	-6.48874800000000	-2.94178900000000	3.90233300000000
Si	0.56400300000000	-5.74270000000000	-4.85139600000000
Si	-8.52416500000000	-0.57720200000000	4.16069700000000
Si	-1.27950500000000	-5.98960800000000	-7.40780400000000
Si	-6.67714700000000	2.35569600000000	-0.50955200000000
Si	-2.47507800000000	-7.41341000000000	-0.20566300000000
Si	-0.66640700000000	-5.74432800000000	-2.02283700000000
Si	-8.46113000000000	0.71160600000000	1.35002700000000
Si	-5.64179500000000	0.64749400000000	-2.76758800000000
Si	2.21185400000000	-5.94076700000000	-0.95996900000000
Si	-8.52547800000000	-2.20129500000000	0.27862300000000
Si	-6.74791900000000	-1.67857500000000	-4.32106000000000
Si	-8.55513800000000	-3.36500200000000	-2.50535900000000
Si	-4.47040600000000	-3.82161800000000	-4.08956800000000
Si	-6.29559600000000	-5.54383700000000	-2.29266700000000
Si	-1.93819900000000	-2.94697800000000	-2.48382500000000
Si	-4.99963600000000	-5.76083400000000	0.53856800000000
Al	-3.78411200000000	-1.01821200000000	-0.87375900000000
Si	-5.60375400000000	-2.77624500000000	1.00283000000000
Si	-2.14760800000000	-1.47116900000000	-7.06025200000000
H	9.15731700000000	-8.02622600000000	2.21548900000000
H	-3.91771600000000	1.47944300000000	-8.40523200000000
H	-5.58278500000000	-0.00021900000000	-7.51993700000000
H	-6.96822800000000	-1.65371900000000	-5.75464100000000
H	-9.64700100000000	-4.15408400000000	-3.06325300000000
H	-4.84422800000000	-4.30653200000000	-8.13264200000000
H	-1.29174700000000	-7.44863900000000	-7.50281200000000
H	-8.50220100000000	2.61182000000000	6.62366100000000
H	-4.24567000000000	6.58482000000000	2.34699900000000
H	-1.84759800000000	9.08555100000000	0.05367300000000
H	-4.98953600000000	6.01120400000000	-3.49395900000000
H	-7.46829400000000	3.44430500000000	-1.09122900000000
H	-9.85050100000000	1.22063400000000	1.30233300000000
H	-3.29698900000000	2.58636100000000	9.28529500000000
H	-0.78120700000000	4.93458300000000	6.75765900000000
H	1.13559900000000	6.92569900000000	4.45840900000000
H	-0.02232900000000	-0.77305700000000	9.60523200000000
H	2.62013800000000	1.69341900000000	6.95031400000000
H	4.49460300000000	3.55343000000000	4.72127000000000
H	1.50104200000000	-6.85835100000000	-5.12163100000000
H	4.59924900000000	9.29498800000000	-0.79422600000000
H	-8.71105400000000	0.61537800000000	7.94089100000000
H	-5.71491700000000	-0.27881000000000	8.77894400000000
H	-5.44132700000000	-3.37863400000000	7.59799600000000
H	-2.89623900000000	-2.96023500000000	8.95285800000000
H	-1.26317600000000	-4.03199500000000	7.55603500000000
H	2.53796700000000	5.50771100000000	-6.31160600000000
H	0.18866300000000	8.18239100000000	-6.00400600000000

H	6.02450500000000	7.34880700000000	-3.91488500000000
H	9.97448400000000	4.31742600000000	-2.90910500000000
H	9.84309900000000	0.50334600000000	-3.74258300000000
H	11.13022900000000	0.86707300000000	-1.75269900000000
H	9.86230600000000	3.98304400000000	-0.53485800000000
H	-1.36016500000000	-1.11631200000000	-8.25127500000000
H	-0.65279500000000	-5.41851200000000	-8.60809000000000
H	2.81201700000000	-2.96369000000000	-6.09835800000000
H	-7.35134500000000	-4.07849600000000	3.60263300000000
H	-9.81866600000000	-1.22952600000000	3.93199600000000
H	-9.43936300000000	-2.67420000000000	1.30216300000000
H	-5.80515500000000	-6.33058100000000	1.63188700000000
H	-3.11701100000000	-8.67119800000000	-0.61594500000000
H	-7.05797200000000	-6.61324600000000	-2.93217200000000
H	2.74742400000000	-7.28923100000000	-1.14044700000000
H	4.86075000000000	-9.49774800000000	2.34612000000000
H	5.78467900000000	-8.96750500000000	0.19577100000000
H	-1.38054300000000	-7.69642300000000	0.72670000000000
H	8.78697500000000	-3.87610900000000	4.63590300000000
H	5.30199000000000	-0.38487900000000	4.37198100000000
H	7.11602900000000	1.41967900000000	2.22437300000000
H	7.28634600000000	6.65804300000000	-0.66128100000000
H	4.97067700000000	-4.78521600000000	-3.39183200000000
H	7.86598200000000	-6.92151100000000	-1.65456600000000
H	2.40641700000000	-6.42011500000000	2.76345800000000
Ga	-4.03638200000000	0.88320800000000	1.72133200000000
C	-2.33179800000000	0.41665500000000	3.79959200000000
H	-2.50110200000000	1.14149500000000	4.58973200000000
H	-3.06200500000000	1.77513500000000	2.62613800000000
H	-1.36396800000000	0.44080200000000	3.31235300000000
C	-3.16909100000000	-0.67793400000000	3.66534300000000
H	-3.91905100000000	-0.85909900000000	4.42883000000000
H	-2.84130400000000	-1.55235500000000	3.10735100000000
H	-4.41756200000000	-0.35103300000000	2.69153600000000

I2-Ga

Electronic energy (r^2SCAN-3c): -30617.294901821107 E_h
Electronic energy (ωB97X-D4rev/def2-mTZVPP): -30625.47612 E_h
G_{corr} (673.15 K, 1 atm): 0.41960 E_h
Number of atoms: 240

Cartesian coordinates (Å)

O	1.49120800000000	2.98848900000000	0.90070100000000
O	-1.98064700000000	6.85555700000000	1.28508300000000
O	5.23007400000000	2.15903100000000	0.65299300000000
O	3.57740000000000	3.96403600000000	-0.32097700000000
O	6.05903600000000	4.65710700000000	0.27096600000000
O	1.39211200000000	3.35951400000000	-1.75031000000000
O	1.33794100000000	5.41004900000000	-0.12027700000000
O	-1.73104000000000	6.96153100000000	-1.33199200000000
O	0.29103000000000	7.78423200000000	0.27246400000000
O	2.89953000000000	7.55994000000000	-0.19447800000000
Si	4.81022200000000	3.72102500000000	0.71354800000000
Si	1.95850000000000	3.93068700000000	-0.34038300000000
Si	-1.32721800000000	7.70964900000000	0.06039100000000
Si	1.61679600000000	6.87633300000000	0.51459300000000

O	8.91222000000000	-3.75227600000000	2.07714800000000
O	1.97317600000000	6.72835300000000	2.09599400000000
O	7.10978500000000	-2.20137600000000	-1.06919600000000
O	2.47236800000000	4.83418600000000	3.94195700000000
O	6.71167200000000	-4.82560400000000	-0.91668300000000
O	0.01483400000000	5.20222000000000	2.99530200000000
O	5.81168400000000	-3.18568600000000	0.98765900000000
O	4.39703900000000	4.15072300000000	2.23141500000000
O	9.36427900000000	-4.93059100000000	-1.27647300000000
O	5.11538900000000	-0.16270900000000	1.86993000000000
O	7.69789500000000	4.45639600000000	-1.83658300000000
O	5.39406000000000	5.71029300000000	-2.06012700000000
O	4.07482100000000	5.76735000000000	-4.34060800000000
O	8.72600600000000	-1.37875300000000	0.89735500000000
O	3.88585000000000	7.81356600000000	-2.67643600000000
O	1.14117100000000	9.60421900000000	-4.20027800000000
O	8.94468200000000	2.15084900000000	-2.03355500000000
O	6.91058100000000	0.26876600000000	-0.04603700000000
O	2.23522000000000	9.63178500000000	-1.71415500000000
O	8.96359900000000	-0.46813300000000	-1.59135600000000
Si	1.40075700000000	5.94545400000000	3.40581100000000
Si	6.07881800000000	-3.36118800000000	-0.59804400000000
Si	3.59425800000000	3.71016500000000	3.57758200000000
Si	8.04334800000000	-5.75703500000000	-0.80449800000000
Si	6.63628900000000	5.40678200000000	-1.05827100000000
Si	9.75851000000000	-2.52826800000000	1.41834600000000
Si	4.89270600000000	6.67744400000000	-3.26686000000000
Si	9.16986700000000	3.75237400000000	-1.81565200000000
Si	6.13707600000000	0.93547400000000	1.22205300000000
Si	9.76483700000000	0.76268300000000	-2.30195400000000
Si	7.92044800000000	-0.93739300000000	-0.45093800000000
Si	3.43303900000000	8.59890500000000	-1.32737600000000
O	2.94144500000000	-0.38673000000000	3.46260000000000
O	0.91548900000000	0.83534900000000	2.32702000000000
O	1.19941100000000	0.95898200000000	4.93407700000000
O	2.91765000000000	2.24998400000000	3.33442500000000
O	-0.38519300000000	1.31249500000000	0.10349100000000
O	-0.84223200000000	2.82679100000000	2.20379600000000
O	-3.75502400000000	5.04340100000000	0.41946800000000
O	-2.56649900000000	4.74556400000000	2.79152600000000
O	-1.19795100000000	3.50632700000000	4.69085500000000
Si	2.01196700000000	0.92609900000000	3.53787500000000
Si	0.29262200000000	2.01758200000000	1.38837200000000
Si	-3.16719900000000	5.82300400000000	1.71894300000000
Si	-1.14149900000000	4.08440600000000	3.18615200000000
O	8.24734900000000	-5.96128900000000	3.34739900000000
O	-3.23801000000000	-2.98606400000000	6.43068100000000
O	6.64538900000000	-7.76718600000000	2.29566300000000
O	-1.34830600000000	-1.48958100000000	7.54201500000000
O	8.22291700000000	-6.26253100000000	0.72416600000000
O	-5.46011000000000	-3.40048800000000	5.08609400000000
O	6.61382000000000	-3.83795000000000	3.42445100000000
O	-5.17095100000000	-1.14010600000000	6.44560600000000
O	-8.70361600000000	0.39819700000000	5.44420700000000
O	4.43239400000000	-2.43072900000000	3.09892900000000
O	-6.48080500000000	1.13554300000000	6.81196000000000
O	4.45158900000000	-4.98313000000000	2.46239400000000
O	-3.92045900000000	0.93378100000000	7.43425000000000

O	-2.28958600000000	2.92818300000000	6.99752500000000
O	0.36503800000000	2.73805100000000	6.71703300000000
O	4.22053800000000	-7.31691700000000	1.22762600000000
O	-1.54009500000000	0.94966600000000	8.63391800000000
O	0.68261600000000	0.15663800000000	7.37929800000000
Si	8.09005000000000	-7.04430700000000	2.13884000000000
Si	-2.16993000000000	-2.89676100000000	7.66664400000000
Si	8.16190700000000	-4.33534000000000	3.40437200000000
Si	-4.85232600000000	-2.73677100000000	6.42792200000000
Si	4.49522000000000	-0.81481800000000	3.22750800000000
Si	-8.11871900000000	1.20542300000000	6.73142400000000
Si	5.32247900000000	-3.62534800000000	2.46586400000000
Si	-5.33970700000000	0.14800400000000	7.41962000000000
Si	-0.97372400000000	3.56689900000000	6.29770800000000
Si	3.40612000000000	-6.01920500000000	1.77792100000000
Si	-2.78886500000000	1.86395800000000	8.12249600000000
Si	1.25737500000000	1.39621800000000	6.50606500000000
Si	5.38022000000000	-8.42933200000000	1.50057600000000
Si	-0.55663200000000	-0.31013300000000	8.32750900000000
O	1.80974200000000	4.35805000000000	-4.18058100000000
O	-0.10778800000000	5.33864900000000	-2.62452900000000
O	-0.63757700000000	7.76701300000000	-3.60064700000000
Si	0.67897900000000	3.99658200000000	-3.07532400000000
Si	-1.23369000000000	6.48853500000000	-2.80152000000000
O	1.20901000000000	-1.65199600000000	-4.65714900000000
O	-4.00038500000000	4.05320300000000	-4.72086700000000
O	3.30377200000000	-2.87581900000000	-3.62977000000000
O	-2.48556300000000	5.92173900000000	-3.65282700000000
O	-3.83920700000000	4.24844200000000	-2.07645600000000
O	1.30501500000000	-4.33375500000000	-4.56506500000000
O	-4.69590700000000	1.87417400000000	-6.08874500000000
O	4.68496100000000	-3.22498100000000	-1.40796900000000
O	-2.60015400000000	1.81250700000000	-4.53437800000000
O	-3.20233800000000	-0.28443400000000	-6.68057200000000
O	-1.15673100000000	-1.75136900000000	-5.81582700000000
O	-0.37662400000000	0.40643100000000	-4.43807900000000
O	-0.36253700000000	2.93978300000000	-3.71117900000000
O	1.75271200000000	7.00998800000000	-4.42339600000000
Si	-3.87091400000000	5.08990100000000	-3.47084100000000
Si	2.15602900000000	-2.96434700000000	-4.78993000000000
Si	-4.09311100000000	2.42902700000000	-4.69841100000000
Si	3.97183100000000	-3.99530100000000	-2.65798600000000
Si	-4.35913900000000	0.74648300000000	-7.22158600000000
Si	-0.34721700000000	-1.22033900000000	-4.51555200000000
Si	-1.23359200000000	1.57318700000000	-3.71910200000000
Si	2.53385600000000	5.65341100000000	-4.86048200000000
Si	0.59541500000000	8.13385100000000	-4.60361800000000
O	-2.83101000000000	-5.50918900000000	-7.26469600000000
O	-4.54575000000000	-4.24825300000000	-5.68241800000000
O	-2.96400900000000	-2.84443200000000	-7.39139600000000
O	-2.69442300000000	0.12580800000000	-0.00200400000000
O	-2.53617500000000	2.80177200000000	-0.32809900000000
O	-1.59852500000000	1.11469800000000	-2.20362100000000
O	-5.17014900000000	2.88724800000000	-0.15793600000000
Si	-3.80393700000000	-4.21402300000000	-7.12689700000000
Si	-1.81643300000000	1.39402900000000	-0.64619300000000
Si	-3.85423300000000	3.74539400000000	-0.54218500000000
O	-5.08508800000000	2.04137000000000	-3.47942600000000

O	2.78217700000000	-4.97078600000000	-2.12805900000000
O	-5.61087200000000	-2.46099200000000	2.64439300000000
O	-7.40131400000000	-1.70724800000000	4.49073100000000
O	-0.29082600000000	-6.20517200000000	-3.54194900000000
O	-8.06305800000000	0.30459000000000	2.87669300000000
O	-0.42634800000000	-5.49558100000000	-6.10962600000000
O	-0.99340800000000	-1.82745100000000	-3.16626800000000
O	-3.52587200000000	-6.43869600000000	0.59725200000000
O	-0.99869400000000	-4.16311300000000	-1.99122000000000
O	-6.62055000000000	1.12482900000000	-1.55303600000000
O	0.58960000000000	-5.98972500000000	-1.01436100000000
O	-8.33967900000000	-0.58395800000000	0.36741700000000
O	-7.40147100000000	1.85181700000000	0.86505500000000
O	-1.91214200000000	-6.64965400000000	-1.54237500000000
O	-6.50220500000000	-0.14612900000000	-3.84031800000000
O	2.67215500000000	-5.33582400000000	0.49381400000000
O	-9.09511100000000	-2.56666500000000	-1.19339800000000
O	-4.36159300000000	-0.14134000000000	-2.21381800000000
O	-7.13616800000000	-2.99859500000000	0.55560000000000
O	-8.05774800000000	-2.30050600000000	-3.63089100000000
O	-5.39510700000000	-2.53040800000000	-3.87666000000000
O	-7.29173500000000	-4.28802300000000	-2.04481400000000
O	-5.03542200000000	-5.08319700000000	-3.22464000000000
O	-2.92136100000000	-3.55245600000000	-3.74017300000000
O	-5.72417300000000	-6.09175200000000	-0.87041400000000
O	-2.80765400000000	-2.39945700000000	-1.31829200000000
O	-4.78707900000000	-4.14176700000000	0.74840400000000
O	-4.95672600000000	-1.49684400000000	0.30365500000000
Si	-6.49423400000000	-2.93584100000000	3.91714700000000
Si	0.56209700000000	-5.75185000000000	-4.85136800000000
Si	-8.52615100000000	-0.57942100000000	4.16369300000000
Si	-1.27956400000000	-5.98969200000000	-7.40747200000000
Si	-6.68373700000000	2.36114700000000	-0.50561600000000
Si	-2.47948900000000	-7.40606300000000	-0.20197800000000
Si	-0.66197800000000	-5.74000800000000	-2.02927100000000
Si	-8.46167800000000	0.71022000000000	1.34896800000000
Si	-5.61845800000000	0.67901700000000	-2.75570900000000
Si	2.21185000000000	-5.93945900000000	-0.95805800000000
Si	-8.52014700000000	-2.19879800000000	0.28356800000000
Si	-6.71965800000000	-1.67895700000000	-4.31852500000000
Si	-8.54942100000000	-3.36341900000000	-2.50407100000000
Si	-4.47030000000000	-3.84693600000000	-4.11430600000000
Si	-6.29160200000000	-5.54474300000000	-2.29106100000000
Si	-1.96630800000000	-2.97814900000000	-2.53972600000000
Si	-4.99730400000000	-5.74420000000000	0.55179700000000
Al	-3.80807600000000	-1.07500600000000	-0.88766300000000
Si	-5.59765600000000	-2.75322700000000	1.03777000000000
Si	-2.15448500000000	-1.47472200000000	-7.06531800000000
H	9.15731700000000	-8.02622600000000	2.21548900000000
H	-3.91771600000000	1.47944300000000	-8.40523200000000
H	-5.58278500000000	-0.00021900000000	-7.51993700000000
H	-6.96822800000000	-1.65371900000000	-5.75464100000000
H	-9.64700100000000	-4.15408400000000	-3.06325300000000
H	-4.84422800000000	-4.30653200000000	-8.13264200000000
H	-1.29174700000000	-7.44863900000000	-7.50281200000000
H	-8.50220100000000	2.61182000000000	6.62366100000000
H	-4.24567000000000	6.58482000000000	2.34699900000000
H	-1.84759800000000	9.08555100000000	0.05367300000000

H	-4.98953600000000	6.01120400000000	-3.49395900000000
H	-7.46829400000000	3.44430500000000	-1.09122900000000
H	-9.85050100000000	1.22063400000000	1.30233300000000
H	-3.29698900000000	2.58636100000000	9.28529500000000
H	-0.78120700000000	4.93458300000000	6.75765900000000
H	1.13559900000000	6.92569900000000	4.45840900000000
H	-0.02232900000000	-0.77305700000000	9.60523200000000
H	2.62013800000000	1.69341900000000	6.95031400000000
H	4.49460300000000	3.55343000000000	4.72127000000000
H	1.50104200000000	-6.85835100000000	-5.12163100000000
H	4.59924900000000	9.29498800000000	-0.79422600000000
H	-8.71105400000000	0.61537800000000	7.94089100000000
H	-5.71491700000000	-0.27881000000000	8.77894400000000
H	-5.44132700000000	-3.37863400000000	7.59799600000000
H	-2.89623900000000	-2.96023500000000	8.95285800000000
H	-1.26317600000000	-4.03199500000000	7.55603500000000
H	2.53796700000000	5.50771100000000	-6.31160600000000
H	0.18866300000000	8.18239100000000	-6.00400600000000
H	6.02450500000000	7.34880700000000	-3.91488500000000
H	9.97448400000000	4.31742600000000	-2.90910500000000
H	9.84309900000000	0.50334600000000	-3.74258300000000
H	11.13022900000000	0.86707300000000	-1.75269900000000
H	9.86230600000000	3.98304400000000	-0.53485800000000
H	-1.36016500000000	-1.11631200000000	-8.25127500000000
H	-0.65279500000000	-5.41851200000000	-8.60809000000000
H	2.81201700000000	-2.96369000000000	-6.09835800000000
H	-7.35134500000000	-4.07849600000000	3.60263300000000
H	-9.81866600000000	-1.22952600000000	3.93199600000000
H	-9.43936300000000	-2.67420000000000	1.30216300000000
H	-5.80515500000000	-6.33058100000000	1.63188700000000
H	-3.11701100000000	-8.67119800000000	-0.61594500000000
H	-7.05797200000000	-6.61324600000000	-2.93217200000000
H	2.74742400000000	-7.28923100000000	-1.14044700000000
H	4.86075000000000	-9.49774800000000	2.34612000000000
H	5.78467900000000	-8.96750500000000	0.19577100000000
H	-1.38054300000000	-7.69642300000000	0.72670000000000
H	8.78697500000000	-3.87610900000000	4.63590300000000
H	5.30199000000000	-0.38487900000000	4.37198100000000
H	7.11602900000000	1.41967900000000	2.22437300000000
H	7.28634600000000	6.65804300000000	-0.66128100000000
H	4.97067700000000	-4.78521600000000	-3.39183200000000
H	7.86598200000000	-6.92151100000000	-1.65456600000000
H	2.40641700000000	-6.42011500000000	2.76345800000000
Ga	-0.90115400000000	-1.51007600000000	2.08205800000000
C	0.39197500000000	-2.75666200000000	2.97659500000000
H	1.41688900000000	-2.50203100000000	2.67564200000000
H	-2.20752700000000	-0.34914000000000	0.79235500000000
H	0.18582100000000	-3.75761700000000	2.56691500000000
C	0.25595000000000	-2.76287500000000	4.50859700000000
H	0.93533200000000	-3.48972600000000	4.97010100000000
H	-0.76133900000000	-3.02635000000000	4.82164700000000
H	0.48681700000000	-1.78489400000000	4.94520800000000

TS2-Ga

Electronic energy (r^2SCAN-3c): -30617.288884504778 E_h
Electronic energy (ωB97X-D4rev/def2-mTZVPP): -30625.46717 E_h
G_{corr} (673.15 K, 1 atm): 0.41304 E_h
Number of atoms: 240

Cartesian coordinates (Å)

O	1.49871800000000	2.97783600000000	0.90168200000000
O	-1.97944800000000	6.85630100000000	1.28412300000000
O	5.23065100000000	2.15819500000000	0.65102300000000
O	3.57803500000000	3.96447900000000	-0.32020100000000
O	6.06020900000000	4.65669700000000	0.27074100000000
O	1.39392900000000	3.35912800000000	-1.74961800000000
O	1.33597500000000	5.40365200000000	-0.11016900000000
O	-1.73413500000000	6.96186100000000	-1.33471600000000
O	0.29061900000000	7.78241300000000	0.26612800000000
O	2.89880800000000	7.55459900000000	-0.19942500000000
Si	4.81136300000000	3.72080700000000	0.71211600000000
Si	1.95883100000000	3.92691600000000	-0.33925600000000
Si	-1.32862700000000	7.70910500000000	0.05716000000000
Si	1.61531700000000	6.87510700000000	0.51256100000000
O	8.91066600000000	-3.75118600000000	2.07714900000000
O	1.97150500000000	6.73498400000000	2.09535500000000
O	7.10989100000000	-2.20141800000000	-1.06881300000000
O	2.47807000000000	4.84305200000000	3.94155800000000
O	6.71140000000000	-4.82569000000000	-0.91577700000000
O	0.01862800000000	5.19987100000000	2.99444800000000
O	5.80946200000000	-3.18543900000000	0.98687100000000
O	4.39989200000000	4.14599000000000	2.23203800000000
O	9.36404600000000	-4.93022500000000	-1.27629300000000
O	5.11966800000000	-0.16326900000000	1.86738400000000
O	7.69774000000000	4.45614000000000	-1.83679800000000
O	5.39358600000000	5.71072300000000	-2.06015200000000
O	4.07676400000000	5.76909700000000	-4.34288600000000
O	8.72619600000000	-1.37846900000000	0.89710400000000
O	3.88743500000000	7.81537000000000	-2.67791300000000
O	1.13936000000000	9.60380900000000	-4.19902900000000
O	8.94465800000000	2.15044700000000	-2.03256900000000
O	6.91347500000000	0.26997800000000	-0.04841400000000
O	2.23565600000000	9.63200700000000	-1.71373400000000
O	8.96538300000000	-0.46962200000000	-1.59255600000000
Si	1.40128400000000	5.94935400000000	3.40384400000000
Si	6.07829600000000	-3.36111500000000	-0.59926700000000
Si	3.59292800000000	3.71375800000000	3.57734500000000
Si	8.04336500000000	-5.75698800000000	-0.80463500000000
Si	6.63613500000000	5.40727000000000	-1.05922000000000
Si	9.75857600000000	-2.52807200000000	1.41823300000000
Si	4.89289500000000	6.67804500000000	-3.26777700000000
Si	9.16976000000000	3.75240900000000	-1.81568300000000
Si	6.14022600000000	0.93682800000000	1.21888200000000
Si	9.76530300000000	0.76293200000000	-2.30234600000000
Si	7.92217100000000	-0.93772500000000	-0.45231700000000
Si	3.43298700000000	8.59836000000000	-1.32824000000000
O	2.94447700000000	-0.38024500000000	3.45491200000000
O	0.90577500000000	0.81952400000000	2.32226200000000
O	1.19703400000000	0.94892400000000	4.93218100000000
O	2.89941600000000	2.25921300000000	3.33323900000000

O	-0.38711300000000	1.31706100000000	0.09525500000000
O	-0.83360400000000	2.82021700000000	2.21288200000000
O	-3.76331600000000	5.05278200000000	0.41583100000000
O	-2.56409000000000	4.74316000000000	2.78246200000000
O	-1.20343300000000	3.51043000000000	4.68995400000000
Si	2.00854100000000	0.92792000000000	3.53492700000000
Si	0.28903600000000	2.01585000000000	1.37836900000000
Si	-3.16868800000000	5.82467000000000	1.71458500000000
Si	-1.14077600000000	4.08501400000000	3.18341900000000
O	8.24518900000000	-5.96039300000000	3.34720000000000
O	-3.23776000000000	-2.98544000000000	6.42993100000000
O	6.64564400000000	-7.76742900000000	2.29594500000000
O	-1.34554600000000	-1.49151900000000	7.54035400000000
O	8.22288200000000	-6.26233900000000	0.72425400000000
O	-5.46118600000000	-3.40126300000000	5.08694600000000
O	6.61242500000000	-3.83598300000000	3.42454800000000
O	-5.16958000000000	-1.13983000000000	6.44582900000000
O	-8.70235100000000	0.39635900000000	5.44440400000000
O	4.42957600000000	-2.42894700000000	3.09696800000000
O	-6.48082900000000	1.13619100000000	6.81258900000000
O	4.44763500000000	-4.98056100000000	2.46413900000000
O	-3.92014100000000	0.93301500000000	7.43420400000000
O	-2.28802700000000	2.92690600000000	6.99800200000000
O	0.36561400000000	2.73787900000000	6.70874900000000
O	4.22195500000000	-7.31599000000000	1.22807500000000
O	-1.54063600000000	0.94849200000000	8.63377900000000
O	0.68202100000000	0.15655900000000	7.37823500000000
Si	8.09013000000000	-7.04414900000000	2.13876300000000
Si	-2.17106600000000	-2.89706000000000	7.66663500000000
Si	8.16103800000000	-4.33452500000000	3.40431100000000
Si	-4.85366200000000	-2.73684500000000	6.42729000000000
Si	4.49903700000000	-0.81279500000000	3.22434200000000
Si	-8.11934800000000	1.20509200000000	6.73076600000000
Si	5.32181700000000	-3.62476300000000	2.46500800000000
Si	-5.34059300000000	0.14822900000000	7.41951000000000
Si	-0.97524100000000	3.56823800000000	6.29542200000000
Si	3.40610600000000	-6.01969600000000	1.77714500000000
Si	-2.78960200000000	1.86365200000000	8.12255400000000
Si	1.25679700000000	1.39553000000000	6.50407700000000
Si	5.38089600000000	-8.42977600000000	1.50065800000000
Si	-0.55714900000000	-0.31045300000000	8.32765200000000
O	1.81442100000000	4.36016400000000	-4.17665600000000
O	-0.10429700000000	5.34011700000000	-2.62158600000000
O	-0.63896400000000	7.76422400000000	-3.60258100000000
Si	0.68022800000000	3.99701600000000	-3.07494700000000
Si	-1.23473700000000	6.48533500000000	-2.80291100000000
O	1.20897000000000	-1.65053100000000	-4.66082900000000
O	-4.00663700000000	4.05045100000000	-4.72074400000000
O	3.30257600000000	-2.87489400000000	-3.62979400000000
O	-2.48398800000000	5.91469700000000	-3.65464700000000
O	-3.84772400000000	4.25089400000000	-2.07694000000000
O	1.30669500000000	-4.33418200000000	-4.56539200000000
O	-4.69760500000000	1.87164700000000	-6.08713200000000
O	4.68469900000000	-3.22442800000000	-1.40880200000000
O	-2.59235900000000	1.81826300000000	-4.54530000000000
O	-3.20381800000000	-0.28618700000000	-6.68177700000000
O	-1.15999500000000	-1.74999600000000	-5.81154000000000
O	-0.37501300000000	0.40764900000000	-4.43340000000000

O	-0.35710400000000	2.94243500000000	-3.71716100000000
O	1.75442700000000	7.01166900000000	-4.42382200000000
Si	-3.87338900000000	5.08772700000000	-3.47217100000000
Si	2.15451300000000	-2.96297400000000	-4.79115900000000
Si	-4.09023500000000	2.42519800000000	-4.69770100000000
Si	3.97094100000000	-3.99455900000000	-2.65958700000000
Si	-4.35973700000000	0.74632500000000	-7.22131900000000
Si	-0.34775500000000	-1.21828500000000	-4.51257600000000
Si	-1.23284400000000	1.57726100000000	-3.71740500000000
Si	2.53472400000000	5.65446700000000	-4.86052600000000
Si	0.59459000000000	8.13309900000000	-4.60357800000000
O	-2.83178100000000	-5.51034400000000	-7.26810800000000
O	-4.53214800000000	-4.24506600000000	-5.67670600000000
O	-2.95943400000000	-2.84586900000000	-7.39560800000000
O	-2.71623000000000	0.15927300000000	0.00469900000000
O	-2.51950000000000	2.82736800000000	-0.33022200000000
O	-1.60264600000000	1.13032400000000	-2.20364500000000
O	-5.16492600000000	2.88780800000000	-0.15569600000000
Si	-3.80204100000000	-4.21288100000000	-7.12647300000000
Si	-1.83523200000000	1.39358600000000	-0.64067800000000
Si	-3.85078300000000	3.74919500000000	-0.54203200000000
O	-5.07179700000000	2.02989400000000	-3.47382600000000
O	2.78273600000000	-4.97017500000000	-2.12658800000000
O	-5.60509500000000	-2.46668200000000	2.64518900000000
O	-7.39929300000000	-1.70630900000000	4.48757600000000
O	-0.29050600000000	-6.20188300000000	-3.54042800000000
O	-8.06353000000000	0.30728200000000	2.87636300000000
O	-0.42755900000000	-5.49582300000000	-6.10912500000000
O	-0.98395500000000	-1.82894100000000	-3.16104300000000
O	-3.52650700000000	-6.43999100000000	0.59813600000000
O	-0.99928800000000	-4.16128000000000	-1.98311000000000
O	-6.61260100000000	1.12351200000000	-1.55078100000000
O	0.58893200000000	-5.98948300000000	-1.01318900000000
O	-8.33959200000000	-0.58373000000000	0.36806400000000
O	-7.40014900000000	1.85331800000000	0.86360200000000
O	-1.91184000000000	-6.65023500000000	-1.54209000000000
O	-6.50975000000000	-0.14355900000000	-3.84098700000000
O	2.67017000000000	-5.33583300000000	0.49473400000000
O	-9.09441600000000	-2.56740500000000	-1.19192300000000
O	-4.36889300000000	-0.17229100000000	-2.22155000000000
O	-7.13486700000000	-2.99775500000000	0.55911900000000
O	-8.06341800000000	-2.29957500000000	-3.63205600000000
O	-5.40383500000000	-2.53215600000000	-3.87830900000000
O	-7.29212900000000	-4.28770400000000	-2.04498200000000
O	-5.03540100000000	-5.08312200000000	-3.22457100000000
O	-2.92734500000000	-3.53882500000000	-3.71744700000000
O	-5.72475300000000	-6.09339400000000	-0.87106600000000
O	-2.78218500000000	-2.38005100000000	-1.29024600000000
O	-4.78623700000000	-4.14315900000000	0.74674600000000
O	-4.95142100000000	-1.49664300000000	0.30779000000000
Si	-6.49342600000000	-2.93641300000000	3.91479000000000
Si	0.56155100000000	-5.75118500000000	-4.85143700000000
Si	-8.52550700000000	-0.57907800000000	4.16135800000000
Si	-1.28023200000000	-5.98969600000000	-7.40796000000000
Si	-6.67808800000000	2.36175900000000	-0.50664800000000
Si	-2.48021700000000	-7.40602100000000	-0.20201100000000
Si	-0.66392900000000	-5.73709600000000	-2.02821100000000
Si	-8.46036100000000	0.71222300000000	1.34704000000000

Si	-5.61385700000000	0.66779600000000	-2.75384200000000
Si	2.21041300000000	-5.93979800000000	-0.95892300000000
Si	-8.51861000000000	-2.19870300000000	0.28448600000000
Si	-6.72484700000000	-1.67657300000000	-4.31840100000000
Si	-8.55004000000000	-3.36283200000000	-2.50397500000000
Si	-4.47009300000000	-3.84155900000000	-4.10789100000000
Si	-6.29184300000000	-5.54407900000000	-2.29163800000000
Si	-1.96438600000000	-2.96692200000000	-2.52195200000000
Si	-4.99830600000000	-5.74441200000000	0.55074900000000
Al	-3.78625000000000	-1.04917700000000	-0.86335000000000
Si	-5.59400600000000	-2.75198100000000	1.03555400000000
Si	-2.15392400000000	-1.47455800000000	-7.06462500000000
H	9.15731700000000	-8.02622600000000	2.21548900000000
H	-3.91771600000000	1.47944300000000	-8.40523200000000
H	-5.58278500000000	-0.00021900000000	-7.51993700000000
H	-6.96822800000000	-1.65371900000000	-5.75464100000000
H	-9.64700100000000	-4.15408400000000	-3.06325300000000
H	-4.84422800000000	-4.30653200000000	-8.13264200000000
H	-1.29174700000000	-7.44863900000000	-7.50281200000000
H	-8.50220100000000	2.61182000000000	6.62366100000000
H	-4.24567000000000	6.58482000000000	2.34699900000000
H	-1.84759800000000	9.08555100000000	0.05367300000000
H	-4.98953600000000	6.01120400000000	-3.49395900000000
H	-7.46829400000000	3.44430500000000	-1.09122900000000
H	-9.85050100000000	1.22063400000000	1.30233300000000
H	-3.29698900000000	2.58636100000000	9.28529500000000
H	-0.78120700000000	4.93458300000000	6.75765900000000
H	1.13559900000000	6.92569900000000	4.45840900000000
H	-0.02232900000000	-0.77305700000000	9.60523200000000
H	2.62013800000000	1.69341900000000	6.95031400000000
H	4.49460300000000	3.55343000000000	4.72127000000000
H	1.50104200000000	-6.85835100000000	-5.12163100000000
H	4.59924900000000	9.29498800000000	-0.79422600000000
H	-8.71105400000000	0.61537800000000	7.94089100000000
H	-5.71491700000000	-0.27881000000000	8.77894400000000
H	-5.44132700000000	-3.37863400000000	7.59799600000000
H	-2.89623900000000	-2.96023500000000	8.95285800000000
H	-1.26317600000000	-4.03199500000000	7.55603500000000
H	2.53796700000000	5.50771100000000	-6.31160600000000
H	0.18866300000000	8.18239100000000	-6.00400600000000
H	6.02450500000000	7.34880700000000	-3.91488500000000
H	9.97448400000000	4.31742600000000	-2.90910500000000
H	9.84309900000000	0.50334600000000	-3.74258300000000
H	11.13022900000000	0.86707300000000	-1.75269900000000
H	9.86230600000000	3.98304400000000	-0.53485800000000
H	-1.36016500000000	-1.11631200000000	-8.25127500000000
H	-0.65279500000000	-5.41851200000000	-8.60809000000000
H	2.81201700000000	-2.96369000000000	-6.09835800000000
H	-7.35134500000000	-4.07849600000000	3.60263300000000
H	-9.81866600000000	-1.22952600000000	3.93199600000000
H	-9.43936300000000	-2.67420000000000	1.30216300000000
H	-5.80515500000000	-6.33058100000000	1.63188700000000
H	-3.11701100000000	-8.67119800000000	-0.61594500000000
H	-7.05797200000000	-6.61324600000000	-2.93217200000000
H	2.74742400000000	-7.28923100000000	-1.14044700000000
H	4.86075000000000	-9.49774800000000	2.34612000000000
H	5.78467900000000	-8.96750500000000	0.19577100000000
H	-1.38054300000000	-7.69642300000000	0.72670000000000

H	8.78697500000000	-3.87610900000000	4.63590300000000
H	5.30199000000000	-0.38487900000000	4.37198100000000
H	7.11602900000000	1.41967900000000	2.22437300000000
H	7.28634600000000	6.65804300000000	-0.66128100000000
H	4.97067700000000	-4.78521600000000	-3.39183200000000
H	7.86598200000000	-6.92151100000000	-1.65456600000000
H	2.40641700000000	-6.42011500000000	2.76345800000000
Ga	-0.75711800000000	-1.42510000000000	2.07793700000000
C	0.47158100000000	-2.69525200000000	2.99547900000000
H	1.50794500000000	-2.46060300000000	2.72524500000000
H	-2.08453400000000	-0.41622400000000	0.90848900000000
H	0.24232500000000	-3.67890900000000	2.55843100000000
C	0.28727100000000	-2.72255600000000	4.52154900000000
H	0.92980700000000	-3.48378100000000	4.97909700000000
H	-0.74585600000000	-2.95837700000000	4.80122700000000
H	0.54164700000000	-1.76313300000000	4.98332400000000

I3-Ga

Electronic energy (r^{2}SCAN-3c): -30617.371052221115 E_h
Electronic energy (ωB97X-D4rev/def2-mTZVPP): -30625.55890 E_h
G_corr (673.15 K, 1 atm): 0.42468 E_h
Number of atoms: 240

Cartesian coordinates (Å)

O	1.44169700000000	2.97988100000000	0.87490800000000
O	-1.97954300000000	6.85277900000000	1.27879100000000
O	5.23078000000000	2.15851300000000	0.65656400000000
O	3.56645000000000	3.95638100000000	-0.30493400000000
O	6.05332900000000	4.65654000000000	0.26976400000000
O	1.40818200000000	3.36451800000000	-1.76425500000000
O	1.33552900000000	5.40955300000000	-0.12497200000000
O	-1.74386000000000	6.96662200000000	-1.33990500000000
O	0.28880800000000	7.78265600000000	0.25919600000000
O	2.89827300000000	7.56052100000000	-0.19231700000000
Si	4.80684900000000	3.71897600000000	0.72014300000000
Si	1.94762000000000	3.92523100000000	-0.34077900000000
Si	-1.32951000000000	7.70895900000000	0.05369700000000
Si	1.61319400000000	6.87351500000000	0.51131400000000
O	8.91554800000000	-3.75466100000000	2.07745000000000
O	1.96052500000000	6.72767400000000	2.09464000000000
O	7.10872400000000	-2.20007200000000	-1.07096700000000
O	2.46799400000000	4.82935000000000	3.93829700000000
O	6.71270800000000	-4.82475400000000	-0.91999700000000
O	0.00890700000000	5.19343200000000	2.99614100000000
O	5.81812100000000	-3.18777500000000	0.98868700000000
O	4.40192200000000	4.15466100000000	2.23746200000000
O	9.36521200000000	-4.93171600000000	-1.27618900000000
O	5.11893700000000	-0.16848100000000	1.87078300000000
O	7.69742200000000	4.45710300000000	-1.83538600000000
O	5.39366000000000	5.71064600000000	-2.06045000000000
O	4.06294000000000	5.76026600000000	-4.33074400000000
O	8.72551500000000	-1.38080900000000	0.89651100000000
O	3.88547600000000	7.81500500000000	-2.67643200000000
O	1.13483000000000	9.60151300000000	-4.19477500000000
O	8.94487200000000	2.15137200000000	-2.03474100000000
O	6.91147500000000	0.27033600000000	-0.04519100000000

O	2.23624500000000	9.63308000000000	-1.71246000000000
O	8.96363400000000	-0.46803000000000	-1.59082000000000
Si	1.39481700000000	5.93972000000000	3.40536800000000
Si	6.07978100000000	-3.36060000000000	-0.59714400000000
Si	3.59413700000000	3.70521000000000	3.57890300000000
Si	8.04308700000000	-5.75669900000000	-0.80469800000000
Si	6.63418400000000	5.40614500000000	-1.05651200000000
Si	9.75854300000000	-2.52866300000000	1.41835000000000
Si	4.88945700000000	6.67591900000000	-3.26680400000000
Si	9.16900800000000	3.75278700000000	-1.81538700000000
Si	6.13553100000000	0.93330400000000	1.22514800000000
Si	9.76433100000000	0.76262300000000	-2.30156700000000
Si	7.91893600000000	-0.93638000000000	-0.45051100000000
Si	3.43274000000000	8.59793500000000	-1.32563000000000
O	2.94170600000000	-0.39035900000000	3.46600900000000
O	0.92953300000000	0.84833700000000	2.32042500000000
O	1.20418400000000	0.96552900000000	4.92974600000000
O	2.93288100000000	2.24277000000000	3.33351300000000
O	-0.46088200000000	1.26438600000000	0.13722200000000
O	-0.86137100000000	2.80525600000000	2.21683000000000
O	-3.79506000000000	5.07388300000000	0.41803200000000
O	-2.57218600000000	4.73787900000000	2.77481100000000
O	-1.22018200000000	3.51348700000000	4.69289000000000
Si	2.01018900000000	0.92308200000000	3.52751000000000
Si	0.26485700000000	1.98952900000000	1.38355400000000
Si	-3.17068700000000	5.82790000000000	1.71597100000000
Si	-1.14863600000000	4.07522600000000	3.18146800000000
O	8.25331800000000	-5.96375500000000	3.34835400000000
O	-3.24586800000000	-3.02304100000000	6.45787600000000
O	6.64541800000000	-7.76717400000000	2.29643900000000
O	-1.35681000000000	-1.48647700000000	7.54085600000000
O	8.22087500000000	-6.26271100000000	0.72406500000000
O	-5.46508200000000	-3.40739700000000	5.09997600000000
O	6.61570400000000	-3.84606300000000	3.42409200000000
O	-5.15578600000000	-1.14181700000000	6.44183900000000
O	-8.71040900000000	0.41835000000000	5.43071000000000
O	4.43995500000000	-2.43320400000000	3.10381100000000
O	-6.48295700000000	1.12129100000000	6.80103700000000
O	4.45530500000000	-4.98576000000000	2.45717100000000
O	-3.92215400000000	0.93968300000000	7.43092600000000
O	-2.28909800000000	2.93005700000000	7.00107100000000
O	0.36307800000000	2.73819100000000	6.71223400000000
O	4.21863000000000	-7.32017400000000	1.22693100000000
O	-1.54573800000000	0.94498000000000	8.63876400000000
O	0.67933400000000	0.16154300000000	7.37829300000000
Si	8.09040100000000	-7.04433200000000	2.13899600000000
Si	-2.16407200000000	-2.89915600000000	7.67596100000000
Si	8.16405000000000	-4.33747300000000	3.40433100000000
Si	-4.84538500000000	-2.73992900000000	6.44127500000000
Si	4.49193800000000	-0.81586400000000	3.22946100000000
Si	-8.11890600000000	1.20701600000000	6.73060900000000
Si	5.32443400000000	-3.62572000000000	2.46647800000000
Si	-5.33429300000000	0.14731100000000	7.41884400000000
Si	-0.97380600000000	3.56822900000000	6.29509300000000
Si	3.40673300000000	-6.02021500000000	1.77716300000000
Si	-2.78569700000000	1.86620000000000	8.12359400000000
Si	1.25929400000000	1.39672600000000	6.49751500000000
Si	5.38053500000000	-8.42917400000000	1.50081700000000

Si	-0.55536800000000	-0.30884800000000	8.32526400000000
O	1.78159200000000	4.35353600000000	-4.20181500000000
O	-0.10183500000000	5.34940000000000	-2.61879000000000
O	-0.65320300000000	7.76285800000000	-3.61299500000000
Si	0.67108100000000	3.99952300000000	-3.07192700000000
Si	-1.23906800000000	6.48516900000000	-2.80444600000000
O	1.21360400000000	-1.65001100000000	-4.65219700000000
O	-4.02242600000000	4.02715300000000	-4.70280800000000
O	3.30608500000000	-2.87900000000000	-3.63069300000000
O	-2.48422700000000	5.89589900000000	-3.65344200000000
O	-3.87223200000000	4.24982300000000	-2.07130900000000
O	1.29938100000000	-4.33151800000000	-4.56415500000000
O	-4.70689000000000	1.85519600000000	-6.07135700000000
O	4.68448000000000	-3.22753200000000	-1.40573300000000
O	-2.58846400000000	1.79700600000000	-4.54577300000000
O	-3.20669600000000	-0.28833400000000	-6.68330700000000
O	-1.14776600000000	-1.72268100000000	-5.81035100000000
O	-0.36519600000000	0.40982100000000	-4.37911000000000
O	-0.39191700000000	2.94209700000000	-3.66906000000000
O	1.74714000000000	7.00874600000000	-4.42068500000000
Si	-3.87866700000000	5.08123800000000	-3.46821600000000
Si	2.15755900000000	-2.96449800000000	-4.78811400000000
Si	-4.08623100000000	2.40206200000000	-4.68459800000000
Si	3.97275700000000	-3.99674200000000	-2.65563800000000
Si	-4.36110200000000	0.74543300000000	-7.21994000000000
Si	-0.33882400000000	-1.21215800000000	-4.50077600000000
Si	-1.24925300000000	1.56794900000000	-3.67578700000000
Si	2.52426400000000	5.64998800000000	-4.86032000000000
Si	0.58990800000000	8.13198500000000	-4.60292400000000
O	-2.83349200000000	-5.51268500000000	-7.26155800000000
O	-4.55898300000000	-4.24949700000000	-5.68772300000000
O	-2.95145100000000	-2.84963300000000	-7.37426400000000
O	-2.90718100000000	0.25235000000000	-0.02678300000000
O	-2.50669100000000	2.87393500000000	-0.31122200000000
O	-1.67929400000000	1.11453900000000	-2.17608700000000
O	-5.15934300000000	2.88096800000000	-0.15271700000000
Si	-3.80291300000000	-4.21472600000000	-7.12634000000000
Si	-1.89453200000000	1.39694400000000	-0.60330700000000
Si	-3.85269300000000	3.76651800000000	-0.53211600000000
O	-5.04350000000000	1.98175300000000	-3.44496300000000
O	2.78047400000000	-4.97269200000000	-2.13093900000000
O	-5.62433300000000	-2.43351600000000	2.67859000000000
O	-7.41645500000000	-1.70823800000000	4.51746200000000
O	-0.28897100000000	-6.22249300000000	-3.55125600000000
O	-8.04893200000000	0.27299300000000	2.85940900000000
O	-0.42660200000000	-5.49368300000000	-6.11156400000000
O	-0.99397400000000	-1.83903600000000	-3.16478200000000
O	-3.52307400000000	-6.42974300000000	0.58552100000000
O	-1.01096200000000	-4.18570800000000	-2.01447300000000
O	-6.62549500000000	1.11918300000000	-1.53773300000000
O	0.58943800000000	-5.99921500000000	-1.02192100000000
O	-8.38862700000000	-0.54911400000000	0.33869100000000
O	-7.39398200000000	1.85854600000000	0.87146800000000
O	-1.91042000000000	-6.66592400000000	-1.55223500000000
O	-6.59258100000000	-0.12404400000000	-3.86410500000000
O	2.67125200000000	-5.34005300000000	0.49231200000000
O	-9.10639300000000	-2.58109200000000	-1.18323800000000
O	-4.51689400000000	-0.33445100000000	-2.26450500000000

O	-7.05184500000000	-2.83982600000000	0.47323000000000
O	-8.08267200000000	-2.30043400000000	-3.62878600000000
O	-5.43068100000000	-2.53101700000000	-3.89062300000000
O	-7.29982500000000	-4.28632800000000	-2.03561000000000
O	-5.04242900000000	-5.07050500000000	-3.22370600000000
O	-2.94018000000000	-3.53440000000000	-3.75172400000000
O	-5.73109700000000	-6.07487600000000	-0.86742400000000
O	-2.80396900000000	-2.42079800000000	-1.32092000000000
O	-4.79755900000000	-4.13578000000000	0.76401300000000
O	-4.71142500000000	-1.46361000000000	0.51192000000000
Si	-6.50980500000000	-2.93451200000000	3.95450200000000
Si	0.56404000000000	-5.75219100000000	-4.85499500000000
Si	-8.53197800000000	-0.57345200000000	4.16532400000000
Si	-1.28024200000000	-5.98981700000000	-7.40767900000000
Si	-6.67274200000000	2.36909800000000	-0.50269500000000
Si	-2.47415800000000	-7.40861300000000	-0.20648000000000
Si	-0.65790900000000	-5.75688400000000	-2.03946600000000
Si	-8.46407300000000	0.72775600000000	1.34763200000000
Si	-5.67066600000000	0.64097700000000	-2.76713900000000
Si	2.21277800000000	-5.94040900000000	-0.95894100000000
Si	-8.52297700000000	-2.16354900000000	0.27194500000000
Si	-6.75072400000000	-1.67235700000000	-4.32420000000000
Si	-8.55726000000000	-3.36581500000000	-2.50282300000000
Si	-4.48724500000000	-3.83409900000000	-4.12440200000000
Si	-6.29581300000000	-5.53841700000000	-2.29531700000000
Si	-1.96504800000000	-2.99255000000000	-2.55209600000000
Si	-4.99471000000000	-5.74871000000000	0.54998400000000
Al	-3.74241900000000	-1.05345200000000	-0.93628300000000
Si	-5.55527500000000	-2.74077800000000	1.08100600000000
Si	-2.14931100000000	-1.47117100000000	-7.06057900000000
H	9.15731700000000	-8.02622600000000	2.21548900000000
H	-3.91771600000000	1.47944300000000	-8.40523200000000
H	-5.58278500000000	-0.00021900000000	-7.51993700000000
H	-6.96822800000000	-1.65371900000000	-5.75464100000000
H	-9.64700100000000	-4.15408400000000	-3.06325300000000
H	-4.84422800000000	-4.30653200000000	-8.13264200000000
H	-1.29174700000000	-7.44863900000000	-7.50281200000000
H	-8.50220100000000	2.61182000000000	6.62366100000000
H	-4.24567000000000	6.58482000000000	2.34699900000000
H	-1.84759800000000	9.08555100000000	0.05367300000000
H	-4.98953600000000	6.01120400000000	-3.49395900000000
H	-7.46829400000000	3.44430500000000	-1.09122900000000
H	-9.85050100000000	1.22063400000000	1.30233300000000
H	-3.29698900000000	2.58636100000000	9.28529500000000
H	-0.78120700000000	4.93458300000000	6.75765900000000
H	1.13559900000000	6.92569900000000	4.45840900000000
H	-0.02232900000000	-0.77305700000000	9.60523200000000
H	2.62013800000000	1.69341900000000	6.95031400000000
H	4.49460300000000	3.55343000000000	4.72127000000000
H	1.50104200000000	-6.85835100000000	-5.12163100000000
H	4.59924900000000	9.29498800000000	-0.79422600000000
H	-8.71105400000000	0.61537800000000	7.94089100000000
H	-5.71491700000000	-0.27881000000000	8.77894400000000
H	-5.44132700000000	-3.37863400000000	7.59799600000000
H	-2.89623900000000	-2.96023500000000	8.95285800000000
H	-1.26317600000000	-4.03199500000000	7.55603500000000
H	2.53796700000000	5.50771100000000	-6.31160600000000
H	0.18866300000000	8.18239100000000	-6.00400600000000

H	6.02450500000000	7.34880700000000	-3.91488500000000
H	9.97448400000000	4.31742600000000	-2.90910500000000
H	9.84309900000000	0.50334600000000	-3.74258300000000
H	11.13022900000000	0.86707300000000	-1.75269900000000
H	9.86230600000000	3.98304400000000	-0.53485800000000
H	-1.36016500000000	-1.11631200000000	-8.25127500000000
H	-0.65279500000000	-5.41851200000000	-8.60809000000000
H	2.81201700000000	-2.96369000000000	-6.09835800000000
H	-7.35134500000000	-4.07849600000000	3.60263300000000
H	-9.81866600000000	-1.22952600000000	3.93199600000000
H	-9.43936300000000	-2.67420000000000	1.30216300000000
H	-5.80515500000000	-6.33058100000000	1.63188700000000
H	-3.11701100000000	-8.67119800000000	-0.61594500000000
H	-7.05797200000000	-6.61324600000000	-2.93217200000000
H	2.74742400000000	-7.28923100000000	-1.14044700000000
H	4.86075000000000	-9.49774800000000	2.34612000000000
H	5.78467900000000	-8.96750500000000	0.19577100000000
H	-1.38054300000000	-7.69642300000000	0.72670000000000
H	8.78697500000000	-3.87610900000000	4.63590300000000
H	5.30199000000000	-0.38487900000000	4.37198100000000
H	7.11602900000000	1.41967900000000	2.22437300000000
H	7.28634600000000	6.65804300000000	-0.66128100000000
H	4.97067700000000	-4.78521600000000	-3.39183200000000
H	7.86598200000000	-6.92151100000000	-1.65456600000000
H	2.40641700000000	-6.42011500000000	2.76345800000000
Ga	-3.61554200000000	-0.25284900000000	1.81781800000000
C	-2.07861500000000	-1.28680200000000	2.45579500000000
H	-1.23033400000000	-0.58974600000000	2.44557600000000
H	-4.61624300000000	0.83290500000000	2.27193800000000
H	-1.85972000000000	-2.03250600000000	1.67967900000000
C	-2.21826100000000	-1.93680600000000	3.82870600000000
H	-1.28636900000000	-2.42402600000000	4.14056600000000
H	-2.99973800000000	-2.70089000000000	3.83208000000000
H	-2.47140800000000	-1.20400700000000	4.60198900000000

TS3-Ga

Electronic energy (r^2SCAN-3c): -30617.289206961243 E_h
Electronic energy (ωB97X-D4rev/def2-mTZVPP): -30625.45226 E_h
G_{corr} (673.15 K, 1 atm): 0.42070 E_h
Number of atoms: 240

Cartesian coordinates (Å)

O	1.45251700000000	2.97689500000000	0.88128700000000
O	-1.98098800000000	6.85124800000000	1.27736400000000
O	5.23159600000000	2.15813100000000	0.65436200000000
O	3.56741700000000	3.95624100000000	-0.30856900000000
O	6.05225200000000	4.65755300000000	0.27068300000000
O	1.40345700000000	3.36038100000000	-1.76056300000000
O	1.33518500000000	5.40639000000000	-0.12144600000000
O	-1.74131800000000	6.96492000000000	-1.34037900000000
O	0.28864700000000	7.78107900000000	0.26059900000000
O	2.89760800000000	7.55848700000000	-0.19398300000000
Si	4.80611100000000	3.71820500000000	0.71822600000000
Si	1.94821200000000	3.92261700000000	-0.33969100000000
Si	-1.32966400000000	7.70803100000000	0.05326100000000
Si	1.61318500000000	6.87196400000000	0.51132300000000

O	8.91532500000000	-3.75452700000000	2.07749200000000
O	1.96249500000000	6.72835900000000	2.09470500000000
O	7.10955400000000	-2.20089900000000	-1.07082600000000
O	2.46886500000000	4.83138400000000	3.93964900000000
O	6.71238800000000	-4.82516000000000	-0.91877600000000
O	0.00915700000000	5.19649200000000	2.99737300000000
O	5.81723400000000	-3.18689700000000	0.98855300000000
O	4.39850900000000	4.14958000000000	2.23636600000000
O	9.36500900000000	-4.93149400000000	-1.27615700000000
O	5.11868900000000	-0.16715500000000	1.87071800000000
O	7.69692700000000	4.45646100000000	-1.83419200000000
O	5.39337000000000	5.71053700000000	-2.06069000000000
O	4.07005500000000	5.76674300000000	-4.33790500000000
O	8.72590000000000	-1.38074000000000	0.89659000000000
O	3.88586700000000	7.81518100000000	-2.67634400000000
O	1.13219300000000	9.60086400000000	-4.19295400000000
O	8.94488500000000	2.15125800000000	-2.03448100000000
O	6.91184700000000	0.26950800000000	-0.04540300000000
O	2.23643100000000	9.63322100000000	-1.71206500000000
O	8.96408600000000	-0.46833500000000	-1.59097500000000
Si	1.39582300000000	5.94139100000000	3.40524100000000
Si	6.07955400000000	-3.36069300000000	-0.59734400000000
Si	3.59191900000000	3.70481200000000	3.57966500000000
Si	8.04306200000000	-5.75676400000000	-0.80459700000000
Si	6.63361500000000	5.40627000000000	-1.05629800000000
Si	9.75872600000000	-2.52878300000000	1.41834400000000
Si	4.89116400000000	6.67744400000000	-3.26680200000000
Si	9.16884400000000	3.75276300000000	-1.81527400000000
Si	6.13623400000000	0.93355400000000	1.22417600000000
Si	9.76444200000000	0.76264900000000	-2.30165900000000
Si	7.91957300000000	-0.93702800000000	-0.45080800000000
Si	3.43260200000000	8.59764600000000	-1.32560900000000
O	2.93960400000000	-0.38982400000000	3.46086000000000
O	0.91546400000000	0.84623200000000	2.32748000000000
O	1.20079900000000	0.95339000000000	4.93303100000000
O	2.92298400000000	2.24402900000000	3.34038800000000
O	-0.44894700000000	1.27067700000000	0.13027300000000
O	-0.85966700000000	2.81269900000000	2.21075900000000
O	-3.79501500000000	5.06547400000000	0.42288000000000
O	-2.57248400000000	4.74082100000000	2.78034400000000
O	-1.21590600000000	3.51096300000000	4.69114700000000
Si	2.00502800000000	0.92123500000000	3.52804900000000
Si	0.26632500000000	1.99290800000000	1.38186500000000
Si	-3.17144800000000	5.82635500000000	1.71643500000000
Si	-1.14788700000000	4.07767400000000	3.18069400000000
O	8.25250400000000	-5.96350200000000	3.34833500000000
O	-3.24327000000000	-2.99199100000000	6.44867400000000
O	6.64555600000000	-7.76734300000000	2.29644000000000
O	-1.34711200000000	-1.48784400000000	7.53828900000000
O	8.22157200000000	-6.26293900000000	0.72408000000000
O	-5.45797800000000	-3.40719300000000	5.10138800000000
O	6.61538700000000	-3.84516900000000	3.42403400000000
O	-5.18356800000000	-1.14107700000000	6.45092200000000
O	-8.69723300000000	0.41047500000000	5.43222900000000
O	4.43942600000000	-2.43247100000000	3.10379800000000
O	-6.48033800000000	1.13697800000000	6.81088800000000
O	4.45458200000000	-4.98506900000000	2.45766000000000
O	-3.91754000000000	0.93143400000000	7.43096100000000

O	-2.28861300000000	2.92812900000000	6.99957800000000
O	0.36316800000000	2.73710000000000	6.71100700000000
O	4.21896700000000	-7.31984100000000	1.22740500000000
O	-1.54520300000000	0.94485400000000	8.63893300000000
O	0.68181400000000	0.16151500000000	7.38033100000000
Si	8.09042600000000	-7.04434600000000	2.13897900000000
Si	-2.16383200000000	-2.89480500000000	7.67508300000000
Si	8.16372200000000	-4.33723300000000	3.40426900000000
Si	-4.85141800000000	-2.73204000000000	6.44259300000000
Si	4.49149500000000	-0.81520700000000	3.22851900000000
Si	-8.11714700000000	1.20789600000000	6.73149400000000
Si	5.32409200000000	-3.62534200000000	2.46622400000000
Si	-5.33794200000000	0.15317900000000	7.42246500000000
Si	-0.97383100000000	3.56806900000000	6.29347700000000
Si	3.40657600000000	-6.02000600000000	1.77692400000000
Si	-2.78632500000000	1.86531800000000	8.12250500000000
Si	1.25993300000000	1.39664300000000	6.49778700000000
Si	5.38058700000000	-8.42919200000000	1.50080500000000
Si	-0.55303700000000	-0.30650000000000	8.32705900000000
O	1.79900100000000	4.35682400000000	-4.19198100000000
O	-0.09749700000000	5.34751400000000	-2.61982200000000
O	-0.65450300000000	7.75805700000000	-3.61583900000000
Si	0.67838700000000	3.99932900000000	-3.07403200000000
Si	-1.23754200000000	6.48059400000000	-2.80451600000000
O	1.21164300000000	-1.65276100000000	-4.65272600000000
O	-4.02657900000000	4.02673900000000	-4.70229300000000
O	3.30613700000000	-2.87848600000000	-3.63087100000000
O	-2.48325700000000	5.88670100000000	-3.64863700000000
O	-3.89043500000000	4.25163500000000	-2.06790800000000
O	1.30242500000000	-4.33373700000000	-4.56442300000000
O	-4.70643900000000	1.85431400000000	-6.07173300000000
O	4.68477600000000	-3.22706400000000	-1.40621500000000
O	-2.57861900000000	1.80697900000000	-4.55845600000000
O	-3.20635300000000	-0.28955800000000	-6.68348300000000
O	-1.15115600000000	-1.73094100000000	-5.80971400000000
O	-0.36198100000000	0.41009100000000	-4.39811400000000
O	-0.37452200000000	2.94621400000000	-3.69384100000000
O	1.74948700000000	7.01034300000000	-4.42154200000000
Si	-3.88224800000000	5.07879200000000	-3.46670100000000
Si	2.15729300000000	-2.96511400000000	-4.78851600000000
Si	-4.08145400000000	2.40106900000000	-4.68686200000000
Si	3.97264300000000	-3.99633100000000	-2.65629000000000
Si	-4.36062400000000	0.74454900000000	-7.22010200000000
Si	-0.34084700000000	-1.21304300000000	-4.50328900000000
Si	-1.23676900000000	1.57458600000000	-3.69328900000000
Si	2.52975300000000	5.65323800000000	-4.86079600000000
Si	0.58955500000000	8.13089500000000	-4.60310600000000
O	-2.83343100000000	-5.51313600000000	-7.26431000000000
O	-4.54912700000000	-4.24922600000000	-5.68348700000000
O	-2.95243400000000	-2.84970200000000	-7.38054100000000
O	-2.88190500000000	0.25331300000000	-0.03798700000000
O	-2.49710100000000	2.87735300000000	-0.32640800000000
O	-1.65320200000000	1.12982500000000	-2.19056800000000
O	-5.15327300000000	2.86882600000000	-0.14457400000000
Si	-3.80209700000000	-4.21467600000000	-7.12637800000000
Si	-1.88322800000000	1.39780700000000	-0.61723800000000
Si	-3.85021200000000	3.76044900000000	-0.53208200000000
O	-5.02947100000000	1.97452700000000	-3.44325800000000

O	2.78128700000000	-4.97255000000000	-2.13047000000000
O	-5.59986100000000	-2.47388200000000	2.67613400000000
O	-7.40560700000000	-1.70954400000000	4.49991900000000
O	-0.29122600000000	-6.21726900000000	-3.54953900000000
O	-8.04853500000000	0.27644900000000	2.85799400000000
O	-0.42700000000000	-5.49395600000000	-6.11129600000000
O	-0.99471700000000	-1.83111100000000	-3.16253100000000
O	-3.52469700000000	-6.43498700000000	0.58965200000000
O	-1.00891400000000	-4.17798400000000	-2.00916200000000
O	-6.62536500000000	1.12219900000000	-1.54508400000000
O	0.58903700000000	-5.99688600000000	-1.02165200000000
O	-8.38834100000000	-0.55211300000000	0.33788400000000
O	-7.38763800000000	1.85238900000000	0.86980300000000
O	-1.91172600000000	-6.65934300000000	-1.54932200000000
O	-6.58729500000000	-0.12406100000000	-3.86647800000000
O	2.67121700000000	-5.33995600000000	0.49233600000000
O	-9.10558100000000	-2.58299600000000	-1.18290800000000
O	-4.51584000000000	-0.34152100000000	-2.25789400000000
O	-7.05994300000000	-2.85157200000000	0.48788300000000
O	-8.08180900000000	-2.30001200000000	-3.62724400000000
O	-5.43225300000000	-2.53295800000000	-3.89146400000000
O	-7.29843700000000	-4.28794100000000	-2.03383100000000
O	-5.04097200000000	-5.07154400000000	-3.22232600000000
O	-2.94144400000000	-3.52752300000000	-3.73756500000000
O	-5.73201100000000	-6.08729600000000	-0.87033300000000
O	-2.79527600000000	-2.40874300000000	-1.30400000000000
O	-4.79365700000000	-4.13893900000000	0.74050700000000
O	-4.72440600000000	-1.46269000000000	0.51075100000000
Si	-6.50050600000000	-2.94237500000000	3.94767100000000
Si	0.56318900000000	-5.75224800000000	-4.85433800000000
Si	-8.52671000000000	-0.57701900000000	4.16139100000000
Si	-1.28019400000000	-5.98987400000000	-7.40794200000000
Si	-6.66958800000000	2.36699500000000	-0.50579100000000
Si	-2.47428000000000	-7.40831900000000	-0.20656400000000
Si	-0.65946300000000	-5.74981900000000	-2.03779200000000
Si	-8.46394100000000	0.72564800000000	1.34436600000000
Si	-5.66327800000000	0.63565700000000	-2.76680500000000
Si	2.21215200000000	-5.94014800000000	-0.95928900000000
Si	-8.52318900000000	-2.16742500000000	0.27322400000000
Si	-6.75018900000000	-1.67235400000000	-4.32423700000000
Si	-8.55563900000000	-3.36704000000000	-2.50235900000000
Si	-4.48449400000000	-3.83278400000000	-4.11961200000000
Si	-6.29492800000000	-5.54043100000000	-2.29541500000000
Si	-1.96565300000000	-2.98114800000000	-2.53992800000000
Si	-4.99620700000000	-5.75205300000000	0.54536200000000
Al	-3.74472800000000	-1.04275900000000	-0.91338000000000
Si	-5.55249300000000	-2.74368400000000	1.07052200000000
Si	-2.14963300000000	-1.47295100000000	-7.06129700000000
H	9.15731700000000	-8.02622600000000	2.21548900000000
H	-3.91771600000000	1.47944300000000	-8.40523200000000
H	-5.58278500000000	-0.00021900000000	-7.51993700000000
H	-6.96822800000000	-1.65371900000000	-5.75464100000000
H	-9.64700100000000	-4.15408400000000	-3.06325300000000
H	-4.84422800000000	-4.30653200000000	-8.13264200000000
H	-1.29174700000000	-7.44863900000000	-7.50281200000000
H	-8.50220100000000	2.61182000000000	6.62366100000000
H	-4.24567000000000	6.58482000000000	2.34699900000000
H	-1.84759800000000	9.08555100000000	0.05367300000000

H	-4.98953600000000	6.01120400000000	-3.49395900000000
H	-7.46829400000000	3.44430500000000	-1.09122900000000
H	-9.85050100000000	1.22063400000000	1.30233300000000
H	-3.29698900000000	2.58636100000000	9.28529500000000
H	-0.78120700000000	4.93458300000000	6.75765900000000
H	1.13559900000000	6.92569900000000	4.45840900000000
H	-0.02232900000000	-0.77305700000000	9.60523200000000
H	2.62013800000000	1.69341900000000	6.95031400000000
H	4.49460300000000	3.55343000000000	4.72127000000000
H	1.50104200000000	-6.85835100000000	-5.12163100000000
H	4.59924900000000	9.29498800000000	-0.79422600000000
H	-8.71105400000000	0.61537800000000	7.94089100000000
H	-5.71491700000000	-0.27881000000000	8.77894400000000
H	-5.44132700000000	-3.37863400000000	7.59799600000000
H	-2.89623900000000	-2.96023500000000	8.95285800000000
H	-1.26317600000000	-4.03199500000000	7.55603500000000
H	2.53796700000000	5.50771100000000	-6.31160600000000
H	0.18866300000000	8.18239100000000	-6.00400600000000
H	6.02450500000000	7.34880700000000	-3.91488500000000
H	9.97448400000000	4.31742600000000	-2.90910500000000
H	9.84309900000000	0.50334600000000	-3.74258300000000
H	11.13022900000000	0.86707300000000	-1.75269900000000
H	9.86230600000000	3.98304400000000	-0.53485800000000
H	-1.36016500000000	-1.11631200000000	-8.25127500000000
H	-0.65279500000000	-5.41851200000000	-8.60809000000000
H	2.81201700000000	-2.96369000000000	-6.09835800000000
H	-7.35134500000000	-4.07849600000000	3.60263300000000
H	-9.81866600000000	-1.22952600000000	3.93199600000000
H	-9.43936300000000	-2.67420000000000	1.30216300000000
H	-5.80515500000000	-6.33058100000000	1.63188700000000
H	-3.11701100000000	-8.67119800000000	-0.61594500000000
H	-7.05797200000000	-6.61324600000000	-2.93217200000000
H	2.74742400000000	-7.28923100000000	-1.14044700000000
H	4.86075000000000	-9.49774800000000	2.34612000000000
H	5.78467900000000	-8.96750500000000	0.19577100000000
H	-1.38054300000000	-7.69642300000000	0.72670000000000
H	8.78697500000000	-3.87610900000000	4.63590300000000
H	5.30199000000000	-0.38487900000000	4.37198100000000
H	7.11602900000000	1.41967900000000	2.22437300000000
H	7.28634600000000	6.65804300000000	-0.66128100000000
H	4.97067700000000	-4.78521600000000	-3.39183200000000
H	7.86598200000000	-6.92151100000000	-1.65456600000000
H	2.40641700000000	-6.42011500000000	2.76345800000000
Ga	-3.49253300000000	-0.27841500000000	1.95206200000000
C	-1.86482900000000	-1.65027600000000	2.37365600000000
H	-1.01034800000000	-1.07172800000000	2.02209700000000
H	-3.72574700000000	-0.34600100000000	3.90828200000000
H	-2.15817900000000	-2.45209000000000	1.69136400000000
C	-1.98311600000000	-1.88927800000000	3.75546500000000
H	-1.27508700000000	-1.43493400000000	4.44741600000000
H	-2.46732200000000	-2.79724300000000	4.10389300000000
H	-3.13956700000000	-0.96614800000000	4.21043900000000

I4-Ga

Electronic energy (r^2SCAN-3c): -30617.320189667553 E_h
Electronic energy (ωB97X-D4rev/def2-mTZVPP): -30625.50379 E_h
G_{corr} (673.15 K, 1 atm): 0.39720 E_h
Number of atoms: 240

Cartesian coordinates (Å)

O	1.45270900000000	2.97722500000000	0.88488400000000
O	-1.98035800000000	6.85162600000000	1.27873800000000
O	5.23241600000000	2.15849900000000	0.65468000000000
O	3.56865000000000	3.95927300000000	-0.30385100000000
O	6.05446300000000	4.65760600000000	0.27187100000000
O	1.41068700000000	3.36245100000000	-1.75842200000000
O	1.33422600000000	5.40623000000000	-0.11741700000000
O	-1.74102700000000	6.96551300000000	-1.33851900000000
O	0.28932400000000	7.78202700000000	0.26239100000000
O	2.89772100000000	7.55802200000000	-0.19402600000000
Si	4.80834800000000	3.71885600000000	0.72061300000000
Si	1.94970100000000	3.92320200000000	-0.33538900000000
Si	-1.32865800000000	7.70866900000000	0.05455100000000
Si	1.61374700000000	6.87233800000000	0.51281500000000
O	8.91558400000000	-3.75480200000000	2.07750600000000
O	1.96318600000000	6.72954200000000	2.09628200000000
O	7.10956600000000	-2.20106900000000	-1.07112400000000
O	2.46847600000000	4.82992100000000	3.93920600000000
O	6.71241400000000	-4.82523500000000	-0.91881500000000
O	0.00899300000000	5.19646600000000	2.99769100000000
O	5.81748000000000	-3.18671200000000	0.98850600000000
O	4.40185700000000	4.14936600000000	2.23912400000000
O	9.36502800000000	-4.93158300000000	-1.27617200000000
O	5.11780900000000	-0.16683300000000	1.87102900000000
O	7.69702600000000	4.45659800000000	-1.83460000000000
O	5.39342000000000	5.71086800000000	-2.05962700000000
O	4.06554500000000	5.76234300000000	-4.33299700000000
O	8.72584000000000	-1.38098700000000	0.89657400000000
O	3.88584100000000	7.81535500000000	-2.67666600000000
O	1.13131900000000	9.59896400000000	-4.19111200000000
O	8.94487300000000	2.15123900000000	-2.03456800000000
O	6.91137500000000	0.26894000000000	-0.04506200000000
O	2.23675400000000	9.63347000000000	-1.71135500000000
O	8.96384000000000	-0.46820700000000	-1.59083700000000
Si	1.39635900000000	5.94095700000000	3.40575800000000
Si	6.07961700000000	-3.36079400000000	-0.59732700000000
Si	3.59189200000000	3.70315900000000	3.57990700000000
Si	8.04308100000000	-5.75684200000000	-0.80452200000000
Si	6.63409100000000	5.40622700000000	-1.05591700000000
Si	9.75875000000000	-2.52891900000000	1.41838900000000
Si	4.89011100000000	6.67621900000000	-3.26665000000000
Si	9.16886600000000	3.75271400000000	-1.81531300000000
Si	6.13606000000000	0.93322500000000	1.22461700000000
Si	9.76438500000000	0.76256000000000	-2.30160000000000
Si	7.91938800000000	-0.93723300000000	-0.45072300000000
Si	3.43277300000000	8.59737300000000	-1.32559200000000
O	2.93939500000000	-0.39052600000000	3.46214900000000
O	0.91551200000000	0.84248900000000	2.32646200000000
O	1.20006900000000	0.95673300000000	4.93254000000000
O	2.92291400000000	2.24287900000000	3.33818100000000

O	-0.44531000000000	1.27406600000000	0.12628500000000
O	-0.85954500000000	2.80967800000000	2.21321400000000
O	-3.79798700000000	5.06761600000000	0.42454800000000
O	-2.57238500000000	4.73875800000000	2.77907800000000
O	-1.21812900000000	3.51153900000000	4.69187000000000
Si	2.00432500000000	0.92019000000000	3.52797600000000
Si	0.26675600000000	1.99034000000000	1.38225700000000
Si	-3.17055300000000	5.82695200000000	1.71732100000000
Si	-1.14685000000000	4.07670100000000	3.18086600000000
O	8.25237800000000	-5.96355800000000	3.34839300000000
O	-3.23527600000000	-2.98283300000000	6.43208400000000
O	6.64548900000000	-7.76742400000000	2.29638000000000
O	-1.34768700000000	-1.48667500000000	7.54049500000000
O	8.22154900000000	-6.26299600000000	0.72413100000000
O	-5.45592300000000	-3.40058900000000	5.09402700000000
O	6.61562700000000	-3.84485600000000	3.42397600000000
O	-5.17075200000000	-1.13900200000000	6.44328600000000
O	-8.70410400000000	0.42038800000000	5.42886200000000
O	4.43941000000000	-2.43252200000000	3.10338400000000
O	-6.48212100000000	1.12867500000000	6.80627700000000
O	4.45528900000000	-4.98525300000000	2.45740100000000
O	-3.92067200000000	0.93689900000000	7.43077600000000
O	-2.28827200000000	2.92842400000000	7.00000200000000
O	0.36394700000000	2.73822200000000	6.71084200000000
O	4.21888300000000	-7.31972800000000	1.22720900000000
O	-1.54598600000000	0.94456200000000	8.63948200000000
O	0.68270900000000	0.16406700000000	7.38320700000000
Si	8.09033000000000	-7.04441400000000	2.13904800000000
Si	-2.16408800000000	-2.89390900000000	7.66925300000000
Si	8.16378000000000	-4.33728000000000	3.40431500000000
Si	-4.84498500000000	-2.73380000000000	6.43853600000000
Si	4.49108300000000	-0.81533300000000	3.22902900000000
Si	-8.11832700000000	1.20880600000000	6.73138800000000
Si	5.32430900000000	-3.62529700000000	2.46621900000000
Si	-5.33628900000000	0.15059600000000	7.42125400000000
Si	-0.97321300000000	3.56826400000000	6.29422500000000
Si	3.40667200000000	-6.01992000000000	1.77727200000000
Si	-2.78566100000000	1.86586600000000	8.12348700000000
Si	1.26025100000000	1.39681400000000	6.49801900000000
Si	5.38041300000000	-8.42914100000000	1.50076300000000
Si	-0.55279200000000	-0.30694800000000	8.32745200000000
O	1.78904900000000	4.35322300000000	-4.19585600000000
O	-0.09577100000000	5.34787800000000	-2.61461500000000
O	-0.65680400000000	7.75558300000000	-3.61680600000000
Si	0.67637500000000	3.99700100000000	-3.06835200000000
Si	-1.23702700000000	6.47901100000000	-2.80192000000000
O	1.21041700000000	-1.65328300000000	-4.65131900000000
O	-4.02568700000000	4.02396000000000	-4.69849100000000
O	3.30621800000000	-2.87837400000000	-3.63068600000000
O	-2.48318400000000	5.88170300000000	-3.64268400000000
O	-3.89583300000000	4.24856400000000	-2.06321700000000
O	1.30326900000000	-4.33383500000000	-4.56492200000000
O	-4.70415100000000	1.85323300000000	-6.06944300000000
O	4.68463300000000	-3.22725500000000	-1.40600700000000
O	-2.57842000000000	1.80241400000000	-4.55153800000000
O	-3.20717800000000	-0.29046300000000	-6.68429700000000
O	-1.15330300000000	-1.72576100000000	-5.80420300000000
O	-0.36317000000000	0.40734300000000	-4.37728800000000

O	-0.38297500000000	2.94069600000000	-3.67086900000000
O	1.74749600000000	7.00799900000000	-4.42056700000000
Si	-3.88346700000000	5.07600300000000	-3.46318500000000
Si	2.15723200000000	-2.96465600000000	-4.78835300000000
Si	-4.08027700000000	2.39793800000000	-4.68300300000000
Si	3.97249000000000	-3.99644100000000	-2.65615400000000
Si	-4.36059000000000	0.74470400000000	-7.21966000000000
Si	-0.34207700000000	-1.21463200000000	-4.49538500000000
Si	-1.24459400000000	1.56795600000000	-3.67376600000000
Si	2.52596900000000	5.64962300000000	-4.85973900000000
Si	0.58840600000000	8.12930800000000	-4.60256500000000
O	-2.83346300000000	-5.51418800000000	-7.26702200000000
O	-4.54309100000000	-4.24925300000000	-5.68201500000000
O	-2.94848100000000	-2.85081500000000	-7.37978400000000
O	-2.88326000000000	0.25114900000000	0.00975300000000
O	-2.50465800000000	2.87251000000000	-0.31198400000000
O	-1.67473000000000	1.12048200000000	-2.17499200000000
O	-5.15594100000000	2.86836800000000	-0.13231100000000
Si	-3.80038100000000	-4.21469500000000	-7.12685900000000
Si	-1.89339500000000	1.38802200000000	-0.59802300000000
Si	-3.85206000000000	3.76027200000000	-0.52710600000000
O	-5.02858300000000	1.97366800000000	-3.43938700000000
O	2.78059200000000	-4.97209800000000	-2.13007500000000
O	-5.63552400000000	-2.44011500000000	2.67203800000000
O	-7.41592300000000	-1.71079500000000	4.52093700000000
O	-0.29169700000000	-6.21437700000000	-3.54931600000000
O	-8.04266700000000	0.27114600000000	2.86060600000000
O	-0.42751200000000	-5.49375000000000	-6.11113600000000
O	-0.99204500000000	-1.83868500000000	-3.15559700000000
O	-3.52244600000000	-6.42672900000000	0.58592800000000
O	-1.01009800000000	-4.18110400000000	-1.99762600000000
O	-6.61154600000000	1.11543000000000	-1.53705900000000
O	0.58991800000000	-6.00025500000000	-1.02063900000000
O	-8.37353400000000	-0.56288400000000	0.34486500000000
O	-7.38684700000000	1.84624700000000	0.87111500000000
O	-1.91025000000000	-6.66470700000000	-1.55103500000000
O	-6.56894500000000	-0.13177200000000	-3.85389600000000
O	2.67151200000000	-5.33939100000000	0.49255600000000
O	-9.10841400000000	-2.57819200000000	-1.18836300000000
O	-4.48239600000000	-0.33035100000000	-2.24253800000000
O	-7.08118800000000	-2.89481200000000	0.49501500000000
O	-8.08336500000000	-2.29656600000000	-3.63176400000000
O	-5.43088100000000	-2.53778200000000	-3.88891400000000
O	-7.29874400000000	-4.28403500000000	-2.04257500000000
O	-5.04088100000000	-5.07728700000000	-3.22497300000000
O	-2.94134200000000	-3.53054000000000	-3.72719300000000
O	-5.73258200000000	-6.07004000000000	-0.86506000000000
O	-2.79009400000000	-2.41179900000000	-1.28947000000000
O	-4.79060100000000	-4.13165700000000	0.76841100000000
O	-4.79453200000000	-1.45123900000000	0.47545300000000
Si	-6.51061700000000	-2.93680700000000	3.95036300000000
Si	0.56322900000000	-5.75179300000000	-4.85446300000000
Si	-8.53010000000000	-0.57649000000000	4.16653400000000
Si	-1.27989900000000	-5.99013100000000	-7.40823900000000
Si	-6.67042300000000	2.36417300000000	-0.50546400000000
Si	-2.47519700000000	-7.40756800000000	-0.20585900000000
Si	-0.65903400000000	-5.75241100000000	-2.03590100000000
Si	-8.46305100000000	0.71778800000000	1.34563600000000

Si	-5.64243200000000	0.62828700000000	-2.75591500000000
Si	2.21282400000000	-5.94051500000000	-0.95875700000000
Si	-8.52550700000000	-2.17821500000000	0.27184000000000
Si	-6.74591800000000	-1.67413000000000	-4.32302000000000
Si	-8.55688600000000	-3.36360500000000	-2.50679700000000
Si	-4.48211700000000	-3.83636400000000	-4.11711600000000
Si	-6.29576200000000	-5.53809400000000	-2.29533600000000
Si	-1.96627600000000	-2.98366600000000	-2.52971500000000
Si	-4.99478900000000	-5.74318100000000	0.55183300000000
Al	-3.73401800000000	-1.04503000000000	-0.88246200000000
Si	-5.57206200000000	-2.73978900000000	1.06859200000000
Si	-2.14845700000000	-1.47239400000000	-7.05962600000000
H	9.15728300000000	-8.02626000000000	2.21556100000000
H	-3.91760400000000	1.47911400000000	-8.40540300000000
H	-5.58260200000000	-0.00041100000000	-7.52016900000000
H	-6.96820900000000	-1.65437900000000	-5.75461600000000
H	-9.64681400000000	-4.15433000000000	-3.06318500000000
H	-4.84412500000000	-4.30676300000000	-8.13272300000000
H	-1.29157000000000	-7.44864800000000	-7.50270600000000
H	-8.50166900000000	2.61200100000000	6.62419000000000
H	-4.24517600000000	6.58516500000000	2.34732400000000
H	-1.84726300000000	9.08567700000000	0.05369800000000
H	-4.98917000000000	6.01155900000000	-3.49447900000000
H	-7.46908700000000	3.44332500000000	-1.09110200000000
H	-9.85074200000000	1.21932600000000	1.30359000000000
H	-3.29731900000000	2.58565900000000	9.28565700000000
H	-0.78111300000000	4.93427900000000	6.75887700000000
H	1.13613400000000	6.92554800000000	4.45871000000000
H	-0.02292700000000	-0.77450400000000	9.60498400000000
H	2.62031500000000	1.69275600000000	6.95041000000000
H	4.49435400000000	3.55265200000000	4.72130200000000
H	1.50105900000000	-6.85839900000000	-5.12145500000000
H	4.59927900000000	9.29491400000000	-0.79421400000000
H	-8.71003600000000	0.61530400000000	7.94133700000000
H	-5.71437100000000	-0.27837500000000	8.77989900000000
H	-5.43873400000000	-3.38051000000000	7.59916500000000
H	-2.89776900000000	-2.96029200000000	8.95206500000000
H	-1.26360300000000	-4.03217500000000	7.55536100000000
H	2.53821200000000	5.50781000000000	-6.31160700000000
H	0.18886300000000	8.18252000000000	-6.00405300000000
H	6.02467100000000	7.34874800000000	-3.91464800000000
H	9.97454700000000	4.31734100000000	-2.90910000000000
H	9.84310300000000	0.50323900000000	-3.74256500000000
H	11.13020900000000	0.86696600000000	-1.75263100000000
H	9.86234200000000	3.98293000000000	-0.53485200000000
H	-1.36005700000000	-1.11656800000000	-8.25128200000000
H	-0.65257100000000	-5.41863000000000	-8.60803100000000
H	2.81236600000000	-2.96399500000000	-6.09819400000000
H	-7.35117300000000	-4.07953300000000	3.60440300000000
H	-9.81843400000000	-1.23073500000000	3.93360300000000
H	-9.44046600000000	-2.67386600000000	1.30175100000000
H	-5.80490500000000	-6.33288000000000	1.63153000000000
H	-3.11672000000000	-8.67149700000000	-0.61573600000000
H	-7.05773600000000	-6.61351200000000	-2.93197000000000
H	2.74756500000000	-7.28926500000000	-1.14008100000000
H	4.86065100000000	-9.49767100000000	2.34615700000000
H	5.78460300000000	-8.96747400000000	0.19574000000000
H	-1.38046000000000	-7.69627000000000	0.72672400000000

H	8.78683600000000	-3.87609300000000	4.63596800000000
H	5.30148800000000	-0.38479300000000	4.37230300000000
H	7.11594500000000	1.41935800000000	2.22462300000000
H	7.28647800000000	6.65792800000000	-0.66112400000000
H	4.97079100000000	-4.78539600000000	-3.39152200000000
H	7.86594400000000	-6.92155300000000	-1.65450400000000
H	2.40640200000000	-6.41991900000000	2.76351300000000
Ga	-3.96588700000000	0.15924100000000	1.97515000000000
C	-1.62284200000000	-1.99988000000000	1.95209500000000
H	-0.78588800000000	-1.43919700000000	1.54297100000000
H	-6.07734200000000	3.25025500000000	3.93226200000000
H	-2.05326000000000	-2.76989700000000	1.31571400000000
C	-2.09947100000000	-1.74802600000000	3.17314200000000
H	-1.64620600000000	-0.99741900000000	3.81833000000000
H	-2.91748300000000	-2.32745300000000	3.59568600000000
H	-6.18295800000000	2.51742700000000	3.99870300000000

TS-Ga-concerted

Electronic energy (r^2SCAN-3c): -30617.265698079598 E_h
Electronic energy (ωB97X-D4rev/def2-mTZVPP): -30625.43212 E_h
G_{corr} (673.15 K, 1 atm): 0.41664 E_h
Number of atoms: 240

Cartesian coordinates (Å)

O	1.51434500000000	2.90726800000000	0.82832800000000
O	-1.98810100000000	6.85132000000000	1.26951600000000
O	5.23446000000000	2.15890000000000	0.65252200000000
O	3.59645200000000	3.98119800000000	-0.31928300000000
O	6.07454200000000	4.65526500000000	0.27521800000000
O	1.44231600000000	3.37485400000000	-1.78290200000000
O	1.33413300000000	5.37688800000000	-0.08241600000000
O	-1.75116800000000	6.96975800000000	-1.34276900000000
O	0.28964700000000	7.76440100000000	0.25212200000000
O	2.89451100000000	7.53541300000000	-0.22139700000000
Si	4.82282200000000	3.72193900000000	0.71760000000000
Si	1.97992500000000	3.91435400000000	-0.35291800000000
Si	-1.32937500000000	7.70797600000000	0.04984200000000
Si	1.61787600000000	6.86056000000000	0.50822100000000
O	8.91747800000000	-3.75602900000000	2.07777300000000
O	1.98096500000000	6.75108000000000	2.09231100000000
O	7.10838000000000	-2.20127100000000	-1.07236500000000
O	2.46293100000000	4.82750800000000	3.92074700000000
O	6.71217100000000	-4.82548100000000	-0.92035100000000
O	-0.00186300000000	5.26460600000000	3.00055300000000
O	5.81777400000000	-3.18745700000000	0.98725000000000
O	4.40716500000000	4.15079600000000	2.23476600000000
O	9.36478100000000	-4.93118300000000	-1.27558000000000
O	5.11787500000000	-0.16573800000000	1.87032800000000
O	7.69877500000000	4.45951700000000	-1.84283000000000
O	5.39366000000000	5.71704800000000	-2.05363900000000
O	4.04761800000000	5.74322400000000	-4.31121000000000
O	8.72529400000000	-1.38263700000000	0.89620900000000
O	3.89282700000000	7.82597700000000	-2.68896600000000
O	1.13056000000000	9.59822800000000	-4.18975400000000
O	8.94448200000000	2.15088900000000	-2.03393800000000
O	6.91269800000000	0.26861700000000	-0.04567400000000

O	2.23738000000000	9.63303900000000	-1.71099000000000
O	8.96429000000000	-0.46901800000000	-1.59097900000000
Si	1.40978600000000	5.96179700000000	3.39939200000000
Si	6.07819600000000	-3.36165600000000	-0.59903100000000
Si	3.59584400000000	3.70454100000000	3.57471600000000
Si	8.04269300000000	-5.75691400000000	-0.80451600000000
Si	6.63943500000000	5.40705500000000	-1.05754300000000
Si	9.75932700000000	-2.52974000000000	1.41831700000000
Si	4.88774700000000	6.67256200000000	-3.26762500000000
Si	9.16954700000000	3.75225900000000	-1.81628000000000
Si	6.13738700000000	0.93252600000000	1.22352500000000
Si	9.76434900000000	0.76200300000000	-2.30147000000000
Si	7.91949900000000	-0.93906800000000	-0.45140900000000
Si	3.43450300000000	8.59507600000000	-1.33274200000000
O	2.94356800000000	-0.39066800000000	3.46571700000000
O	0.91695600000000	0.84493200000000	2.33594900000000
O	1.20474100000000	0.96034200000000	4.93408900000000
O	2.92852800000000	2.24346500000000	3.33601100000000
O	-0.47957500000000	1.25447800000000	0.16282300000000
O	-0.77528500000000	2.88761800000000	2.17924700000000
O	-3.81965100000000	5.06938200000000	0.42659600000000
O	-2.56598800000000	4.73835300000000	2.76840700000000
O	-1.19324100000000	3.52747100000000	4.67662400000000
Si	2.01179400000000	0.92041800000000	3.53246100000000
Si	0.29833000000000	1.98914800000000	1.37129600000000
Si	-3.17416300000000	5.82825200000000	1.71488500000000
Si	-1.12297000000000	4.11317400000000	3.17087600000000
O	8.25294700000000	-5.96425900000000	3.34870800000000
O	-3.24135700000000	-2.97908000000000	6.43554100000000
O	6.64556200000000	-7.76788700000000	2.29656300000000
O	-1.35141500000000	-1.48536100000000	7.54328000000000
O	8.22068300000000	-6.26231600000000	0.72445800000000
O	-5.44974700000000	-3.38413900000000	5.07475200000000
O	6.61669300000000	-3.84502000000000	3.42261400000000
O	-5.18432900000000	-1.14060900000000	6.45097100000000
O	-8.69766000000000	0.38830900000000	5.44960700000000
O	4.44137300000000	-2.43340100000000	3.10241600000000
O	-6.47921600000000	1.13954600000000	6.81069500000000
O	4.45584300000000	-4.98654200000000	2.45758300000000
O	-3.91969500000000	0.93697800000000	7.42216000000000
O	-2.28887500000000	2.91845400000000	6.97536700000000
O	0.36440900000000	2.73858900000000	6.70890500000000
O	4.21902200000000	-7.32036300000000	1.22751200000000
O	-1.54415800000000	0.94986500000000	8.63451600000000
O	0.68299200000000	0.16422500000000	7.38461600000000
Si	8.09002200000000	-7.04482100000000	2.13899200000000
Si	-2.16710100000000	-2.89419600000000	7.66819900000000
Si	8.16456100000000	-4.33814600000000	3.40419400000000
Si	-4.85239500000000	-2.73896600000000	6.43128100000000
Si	4.49432300000000	-0.81718700000000	3.22833300000000
Si	-8.11820000000000	1.20571100000000	6.73338400000000
Si	5.32505900000000	-3.62733700000000	2.46472100000000
Si	-5.33946900000000	0.15313100000000	7.42368900000000
Si	-0.96780200000000	3.57030000000000	6.28540500000000
Si	3.40624000000000	-6.02033000000000	1.77599000000000
Si	-2.78462900000000	1.86732200000000	8.11653200000000
Si	1.26081700000000	1.39555400000000	6.50016700000000
Si	5.38024300000000	-8.42951700000000	1.50072900000000

Si	-0.55428600000000	-0.30757600000000	8.32687100000000
O	1.74227100000000	4.34712700000000	-4.23076000000000
O	-0.09586700000000	5.34981100000000	-2.60200700000000
O	-0.65852100000000	7.75458000000000	-3.61836000000000
Si	0.66146900000000	3.99425400000000	-3.07084500000000
Si	-1.23690700000000	6.48005300000000	-2.80094400000000
O	1.23036800000000	-1.62675500000000	-4.69067700000000
O	-4.01402000000000	4.03082700000000	-4.71073500000000
O	3.30287400000000	-2.87310000000000	-3.62808800000000
O	-2.47988700000000	5.87519300000000	-3.64022000000000
O	-3.92612300000000	4.25155200000000	-2.06983200000000
O	1.29821000000000	-4.31873400000000	-4.56909900000000
O	-4.69609100000000	1.86175100000000	-6.08039800000000
O	4.68349600000000	-3.22764100000000	-1.40722400000000
O	-2.61720800000000	1.78351900000000	-4.49269300000000
O	-3.20496600000000	-0.28756400000000	-6.68316300000000
O	-1.15689800000000	-1.72354800000000	-5.80141300000000
O	-0.38285300000000	0.40622900000000	-4.36288200000000
O	-0.42194100000000	2.92912500000000	-3.61615400000000
O	1.74512500000000	7.00624200000000	-4.41757900000000
Si	-3.88480200000000	5.07860000000000	-3.47309100000000
Si	2.16039100000000	-2.95492200000000	-4.79286600000000
Si	-4.10211700000000	2.40584600000000	-4.68292300000000
Si	3.96975200000000	-3.99564200000000	-2.65786200000000
Si	-4.35885300000000	0.74358500000000	-7.22240300000000
Si	-0.32675500000000	-1.21611900000000	-4.50294400000000
Si	-1.27089300000000	1.54586800000000	-3.63770900000000
Si	2.51375300000000	5.64303200000000	-4.86027100000000
Si	0.58824300000000	8.12921800000000	-4.60265600000000
O	-2.83271200000000	-5.51052800000000	-7.26815900000000
O	-4.50548900000000	-4.22830900000000	-5.65936100000000
O	-2.94715900000000	-2.84645400000000	-7.39156900000000
O	-2.98623300000000	0.33611100000000	0.08776000000000
O	-2.49673500000000	2.90148700000000	-0.33125400000000
O	-1.68413200000000	1.07052500000000	-2.13491800000000
O	-5.14109000000000	2.83885600000000	-0.12710400000000
Si	-3.79711700000000	-4.20933100000000	-7.12174300000000
Si	-1.90966900000000	1.40049800000000	-0.57649900000000
Si	-3.85859900000000	3.77380700000000	-0.53662300000000
O	-5.10440900000000	2.01850800000000	-3.47033800000000
O	2.77890700000000	-4.97268000000000	-2.13311400000000
O	-5.60747500000000	-2.49740900000000	2.61630800000000
O	-7.38153400000000	-1.69480300000000	4.45984400000000
O	-0.29748300000000	-6.18321100000000	-3.54420300000000
O	-8.06539400000000	0.32559600000000	2.88320500000000
O	-0.42508900000000	-5.49385900000000	-6.11211600000000
O	-0.91764700000000	-1.87497400000000	-3.15975800000000
O	-3.52942300000000	-6.44815000000000	0.58753800000000
O	-1.03437200000000	-4.17971800000000	-1.94166000000000
O	-6.61130200000000	1.10876200000000	-1.53559300000000
O	0.59068100000000	-6.00317600000000	-1.01927800000000
O	-8.32890800000000	-0.58669300000000	0.37877100000000
O	-7.38700300000000	1.84965700000000	0.87087000000000
O	-1.90799400000000	-6.67574200000000	-1.55300500000000
O	-6.56904200000000	-0.13971700000000	-3.84220500000000
O	2.67537100000000	-5.33864900000000	0.49013400000000
O	-9.10047300000000	-2.55587200000000	-1.19875100000000
O	-4.41510500000000	-0.23244800000000	-2.26741600000000

O	-7.14825600000000	-3.01227100000000	0.54614800000000
O	-8.08666100000000	-2.30818800000000	-3.64418600000000
O	-5.42977600000000	-2.52810100000000	-3.87412800000000
O	-7.29712200000000	-4.28609900000000	-2.04103600000000
O	-5.03748400000000	-5.07154600000000	-3.21601000000000
O	-2.94869900000000	-3.48790100000000	-3.65710900000000
O	-5.74063500000000	-6.11087900000000	-0.87426600000000
O	-2.69022700000000	-2.27521500000000	-1.25142100000000
O	-4.78985800000000	-4.15241600000000	0.70209400000000
O	-4.97387700000000	-1.50781400000000	0.27348200000000
Si	-6.48882900000000	-2.94175800000000	3.90242500000000
Si	0.56399600000000	-5.74273500000000	-4.85139600000000
Si	-8.52418300000000	-0.57719800000000	4.16071200000000
Si	-1.27950800000000	-5.98960900000000	-7.40780100000000
Si	-6.67714500000000	2.35571600000000	-0.50954500000000
Si	-2.47508800000000	-7.41338300000000	-0.20565800000000
Si	-0.66638400000000	-5.74433600000000	-2.02287200000000
Si	-8.46113200000000	0.71160500000000	1.35000900000000
Si	-5.64176500000000	0.64749800000000	-2.76759200000000
Si	2.21185000000000	-5.94076900000000	-0.95996600000000
Si	-8.52551500000000	-2.20126400000000	0.27857700000000
Si	-6.74789500000000	-1.67856400000000	-4.32106200000000
Si	-8.55515800000000	-3.36498400000000	-2.50537100000000
Si	-4.47043600000000	-3.82166600000000	-4.08964300000000
Si	-6.29561000000000	-5.54381600000000	-2.29267000000000
Si	-1.93828500000000	-2.94707400000000	-2.48396400000000
Si	-4.99963600000000	-5.76077700000000	0.53860900000000
Al	-3.78413900000000	-1.01835100000000	-0.87381000000000
Si	-5.60378800000000	-2.77615800000000	1.00289600000000
Si	-2.14760800000000	-1.47118000000000	-7.06025700000000
H	9.15731700000000	-8.02622600000000	2.21548900000000
H	-3.91771600000000	1.47944300000000	-8.40523200000000
H	-5.58278500000000	-0.00021900000000	-7.51993700000000
H	-6.96822800000000	-1.65371900000000	-5.75464100000000
H	-9.64700100000000	-4.15408400000000	-3.06325300000000
H	-4.84422800000000	-4.30653200000000	-8.13264200000000
H	-1.29174700000000	-7.44863900000000	-7.50281200000000
H	-8.50220100000000	2.61182000000000	6.62366100000000
H	-4.24567000000000	6.58482000000000	2.34699900000000
H	-1.84759800000000	9.08555100000000	0.05367300000000
H	-4.98953600000000	6.01120400000000	-3.49395900000000
H	-7.46829400000000	3.44430500000000	-1.09122900000000
H	-9.85050100000000	1.22063400000000	1.30233300000000
H	-3.29698900000000	2.58636100000000	9.28529500000000
H	-0.78120700000000	4.93458300000000	6.75765900000000
H	1.13559900000000	6.92569900000000	4.45840900000000
H	-0.02232900000000	-0.77305700000000	9.60523200000000
H	2.62013800000000	1.69341900000000	6.95031400000000
H	4.49460300000000	3.55343000000000	4.72127000000000
H	1.50104200000000	-6.85835100000000	-5.12163100000000
H	4.59924900000000	9.29498800000000	-0.79422600000000
H	-8.71105400000000	0.61537800000000	7.94089100000000
H	-5.71491700000000	-0.27881000000000	8.77894400000000
H	-5.44132700000000	-3.37863400000000	7.59799600000000
H	-2.89623900000000	-2.96023500000000	8.95285800000000
H	-1.26317600000000	-4.03199500000000	7.55603500000000
H	2.53796700000000	5.50771100000000	-6.31160600000000
H	0.18866300000000	8.18239100000000	-6.00400600000000

H	6.02450500000000	7.34880700000000	-3.91488500000000
H	9.97448400000000	4.31742600000000	-2.90910500000000
H	9.84309900000000	0.50334600000000	-3.74258300000000
H	11.13022900000000	0.86707300000000	-1.75269900000000
H	9.86230600000000	3.98304400000000	-0.53485800000000
H	-1.36016500000000	-1.11631200000000	-8.25127500000000
H	-0.65279500000000	-5.41851200000000	-8.60809000000000
H	2.81201700000000	-2.96369000000000	-6.09835800000000
H	-7.35134500000000	-4.07849600000000	3.60263300000000
H	-9.81866600000000	-1.22952600000000	3.93199600000000
H	-9.43936300000000	-2.67420000000000	1.30216300000000
H	-5.80515500000000	-6.33058100000000	1.63188700000000
H	-3.11701100000000	-8.67119800000000	-0.61594500000000
H	-7.05797200000000	-6.61324600000000	-2.93217200000000
H	2.74742400000000	-7.28923100000000	-1.14044700000000
H	4.86075000000000	-9.49774800000000	2.34612000000000
H	5.78467900000000	-8.96750500000000	0.19577100000000
H	-1.38054300000000	-7.69642300000000	0.72670000000000
H	8.78697500000000	-3.87610900000000	4.63590300000000
H	5.30199000000000	-0.38487900000000	4.37198100000000
H	7.11602900000000	1.41967900000000	2.22437300000000
H	7.28634600000000	6.65804300000000	-0.66128100000000
H	4.97067700000000	-4.78521600000000	-3.39183200000000
H	7.86598200000000	-6.92151100000000	-1.65456600000000
H	2.40641700000000	-6.42011500000000	2.76345800000000
Ga	-4.03564300000000	0.88333800000000	1.72137900000000
C	-2.32897100000000	0.41300300000000	3.79505600000000
H	-2.49201900000000	1.14015900000000	4.58436100000000
H	-3.05883100000000	1.77335600000000	2.62514300000000
H	-1.36264900000000	0.43186400000000	3.30453400000000
C	-3.17114600000000	-0.67843300000000	3.66577600000000
H	-3.92020500000000	-0.85425100000000	4.43142200000000
H	-2.84815200000000	-1.55569400000000	3.10950300000000
H	-4.41956000000000	-0.34977700000000	2.69188800000000

S2.1.4 [GaO]$^+$ site

GaO-zeolite

Electronic energy (r^{2}SCAN-3c): -30612.746717467358 E_h
Electronic energy (ωB97X-D4rev/def2-mTZVPP): -30620.855185594275 E_h
G_{corr} (673.15 K, 1 atm): 0.36434494 E_h
Number of atoms: 241

Cartesian coordinates (Å)

O	1.49482300000000	2.85384500000000	1.00177700000000
O	-1.95263000000000	6.76390300000000	1.41712700000000
O	5.27078100000000	2.07813900000000	0.80158100000000
O	3.61052000000000	3.88129800000000	-0.15381100000000
O	6.09549300000000	4.57651500000000	0.42059300000000
O	1.46334400000000	3.28836100000000	-1.62137600000000
O	1.36723600000000	5.30921100000000	0.04718300000000
O	-1.72234600000000	6.89348300000000	-1.19736500000000
O	0.32016400000000	7.68916000000000	0.39552600000000
O	2.92761200000000	7.46620300000000	-0.05671800000000
Si	4.84909200000000	3.63882300000000	0.87224600000000
Si	1.99295200000000	3.83350700000000	-0.18717400000000

Si	-1.29968100000000	7.62597100000000	0.20052800000000
Si	1.64561700000000	6.78407200000000	0.66062800000000
O	8.95228200000000	-3.83524700000000	2.22518000000000
O	1.99472500000000	6.65779900000000	2.24412000000000
O	7.14532800000000	-2.28046700000000	-0.92384500000000
O	2.50450000000000	4.75178000000000	4.07936900000000
O	6.74848200000000	-4.90500900000000	-0.77348000000000
O	0.04197900000000	5.12196400000000	3.13962600000000
O	5.85522300000000	-3.26873800000000	1.13697200000000
O	4.44358000000000	4.07008800000000	2.38918000000000
O	9.40133400000000	-5.01126900000000	-1.12794700000000
O	5.15544300000000	-0.24900500000000	2.01865100000000
O	7.73360700000000	4.37825500000000	-1.68915600000000
O	10.70380500000000	-3.18532900000000	0.34606800000000
O	5.42985700000000	5.63268200000000	-1.90983800000000
O	4.08528500000000	5.66807400000000	-4.16784700000000
O	8.76208200000000	-1.46171800000000	1.04385400000000
O	3.92375500000000	7.73944200000000	-2.53237800000000
O	1.16696700000000	9.51758500000000	-4.04059300000000
O	8.98086800000000	2.07179200000000	-1.88700400000000
O	6.94795000000000	0.18911800000000	0.10285500000000
O	2.27364400000000	9.55375000000000	-1.56202600000000
O	8.99987500000000	-0.54814800000000	-1.44353000000000
Si	1.42734400000000	5.86197200000000	3.55248100000000
Si	6.11732200000000	-3.44058100000000	-0.44811400000000
Si	3.62751400000000	3.62316100000000	3.72812500000000
Si	8.07943300000000	-5.83647900000000	-0.65660400000000
Si	10.87670800000000	-4.45884500000000	-0.67183300000000
Si	6.67139700000000	5.32643100000000	-0.90720300000000
Si	9.79513200000000	-2.60888700000000	1.56656300000000
Si	4.92292800000000	6.59382800000000	-3.11820500000000
Si	9.20493500000000	3.67339200000000	-1.66746200000000
Si	6.17207200000000	0.85245900000000	1.37371900000000
Si	1.73009400000000	10.45219400000000	-2.82305100000000
Si	9.80044800000000	0.68312300000000	-2.15352000000000
Si	7.95559200000000	-1.01647400000000	-0.30269100000000
Si	3.46808200000000	8.51466400000000	-1.17731600000000
O	2.97786800000000	-0.46862100000000	3.61513100000000
O	0.95239700000000	0.75853800000000	2.48671500000000
O	1.24532100000000	0.88683600000000	5.08954500000000
O	2.96209200000000	2.16325400000000	3.47781700000000
O	-0.43441700000000	1.15634800000000	0.30326900000000
O	-0.81291500000000	2.75222300000000	2.33734000000000
O	-3.77356100000000	4.97480700000000	0.58431200000000
O	-2.53340000000000	4.66342300000000	2.93706600000000
O	-1.15062300000000	3.42899800000000	4.84030400000000
Si	2.04133900000000	0.84197000000000	3.68635100000000
Si	0.30319500000000	1.89774500000000	1.54352100000000
Si	-3.13720400000000	5.73990300000000	1.87609400000000
Si	-1.11067500000000	3.99359500000000	3.33344500000000
O	8.28886700000000	-6.04325500000000	3.49640800000000
O	-3.19934500000000	-3.06692900000000	6.58373300000000
O	6.68178000000000	-7.84732500000000	2.44463700000000
O	-1.30795400000000	-1.56834400000000	7.69588800000000
O	8.25602200000000	-6.34258800000000	0.87203000000000
O	-5.42489100000000	-3.49280200000000	5.25102800000000
O	6.65248600000000	-3.92449600000000	3.57257400000000
O	-5.15264800000000	-1.22528500000000	6.59736400000000

O	-8.66836300000000	0.34637200000000	5.57549100000000
O	4.47556500000000	-2.51184300000000	3.25079500000000
O	-6.44490900000000	1.05613500000000	6.96375000000000
O	4.49319400000000	-5.06520200000000	2.60594300000000
O	-3.87898000000000	0.84543900000000	7.59169300000000
O	-2.25432000000000	2.85501100000000	7.15090600000000
O	0.39963300000000	2.65892600000000	6.87567700000000
O	4.25513300000000	-7.39942700000000	1.37470900000000
O	-1.50184400000000	0.87293000000000	8.78606400000000
O	0.72602800000000	0.08046300000000	7.53728400000000
Si	8.12659400000000	-7.12384300000000	2.28740900000000
Si	-2.12777500000000	-2.97225600000000	7.81779400000000
Si	8.20033600000000	-4.41679000000000	3.55256700000000
Si	-4.80574000000000	-2.81014200000000	6.58960600000000
Si	4.52635600000000	-0.89376700000000	3.37858700000000
Si	-8.07621900000000	1.13123000000000	6.88169300000000
Si	5.36087300000000	-3.70361800000000	2.61590000000000
Si	-5.29832200000000	0.07072600000000	7.56625200000000
Si	-0.93716000000000	3.48602700000000	6.44658600000000
Si	3.44472400000000	-6.09945000000000	1.92747800000000
Si	-2.74988700000000	1.78602200000000	8.26960900000000
Si	1.29441100000000	1.31901400000000	6.65683600000000
Si	5.41705400000000	-8.50884500000000	1.64904100000000
Si	-0.51688200000000	-0.38450900000000	8.47687900000000
O	1.78436600000000	4.26747300000000	-4.07118400000000
O	-0.06367300000000	5.27343600000000	-2.45436000000000
O	-0.62710000000000	7.67546000000000	-3.47394600000000
Si	0.70046700000000	3.92022100000000	-2.91224700000000
Si	-1.20583400000000	6.40330800000000	-2.65295300000000
O	1.24815600000000	-1.73242800000000	-4.49483600000000
O	-3.98951700000000	3.93644200000000	-4.54064600000000
O	3.34257800000000	-2.96108600000000	-3.48252600000000
O	-2.44566000000000	5.79358900000000	-3.49684300000000
O	-3.85576600000000	4.16408100000000	-1.90821300000000
O	1.33118800000000	-4.41088300000000	-4.41599500000000
O	-4.66797600000000	1.76944000000000	-5.91531300000000
O	4.72074200000000	-3.30783600000000	-1.25658300000000
O	-2.56182900000000	1.69698200000000	-4.36893400000000
O	-3.17076300000000	-0.36817700000000	-6.53553200000000
O	-1.10955300000000	-1.79001300000000	-5.65783400000000
O	-0.32818800000000	0.32829000000000	-4.19743000000000
O	-0.38455600000000	2.85427600000000	-3.46218600000000
O	1.77746900000000	6.92462900000000	-4.26964200000000
Si	-3.84672700000000	4.99464100000000	-3.30810400000000
Si	2.19438600000000	-3.04616700000000	-4.63845100000000
Si	-4.05361800000000	2.31332400000000	-4.52502000000000
Si	4.01008200000000	-4.07748700000000	-2.50534700000000
Si	-4.32498400000000	0.66585500000000	-7.07122200000000
Si	-0.30192500000000	-1.29182500000000	-4.34345000000000
Si	-1.22669700000000	1.47316600000000	-3.49159400000000
Si	2.55014200000000	5.56385400000000	-4.71058200000000
Si	0.62245300000000	8.04990500000000	-4.45417100000000
O	-2.79784500000000	-5.59418800000000	-7.11511900000000
O	-4.52704900000000	-4.33661700000000	-5.54151000000000
O	-2.91207500000000	-2.93106800000000	-7.21488500000000
O	-2.88764900000000	0.14254900000000	0.16362300000000
O	-2.48739200000000	2.77294600000000	-0.15345600000000
O	-1.67728500000000	0.98875800000000	-1.99843300000000

O	-5.11946900000000	2.75553200000000	-0.00386300000000
Si	-3.76627700000000	-4.29672900000000	-6.97887000000000
Si	-1.85980500000000	1.30878500000000	-0.42850000000000
Si	-3.82893000000000	3.67698300000000	-0.37003500000000
O	-5.01482900000000	1.89401200000000	-3.28660300000000
O	2.81438900000000	-5.05146700000000	-1.98238600000000
O	-5.61100800000000	-2.52389500000000	2.82594600000000
O	-7.39459600000000	-1.80221100000000	4.68006000000000
O	-0.24894600000000	-6.31382100000000	-3.40549500000000
O	-8.01077000000000	0.16345100000000	2.99735700000000
O	-0.39373600000000	-5.57318600000000	-5.96183800000000
O	-0.96847200000000	-1.92341700000000	-3.01268800000000
O	-3.48469300000000	-6.49711200000000	0.72633800000000
O	-0.96179900000000	-4.27722200000000	-1.87490200000000
O	-6.60689700000000	1.02947900000000	-1.38733200000000
O	0.62857200000000	-6.08863300000000	-0.87195800000000
O	-8.37054400000000	-0.62070900000000	0.47077300000000
O	-7.35484500000000	1.76497900000000	1.02257600000000
O	-1.87289600000000	-6.75057700000000	-1.40669400000000
O	-6.55178400000000	-0.21327700000000	-3.71357000000000
O	2.70874600000000	-5.41914700000000	0.64151400000000
O	-9.08172700000000	-2.66320300000000	-1.03937300000000
O	-4.49050200000000	-0.41428100000000	-2.09038900000000
O	-7.01100600000000	-2.88782700000000	0.58781300000000
O	-8.04230100000000	-2.38604900000000	-3.48072200000000
O	-5.38697600000000	-2.61257100000000	-3.74264500000000
O	-7.26533200000000	-4.36637600000000	-1.88374800000000
O	-5.00851700000000	-5.15252200000000	-3.07287900000000
O	-2.90296100000000	-3.62805100000000	-3.60753000000000
O	-5.69951500000000	-6.15578000000000	-0.71741000000000
O	-2.76647400000000	-2.53697400000000	-1.17069000000000
O	-4.76680300000000	-4.19875900000000	0.88542200000000
O	-4.67564600000000	-1.51768400000000	0.67855200000000
Si	-6.47819400000000	-3.01716300000000	4.12103700000000
Si	0.60064600000000	-5.83374900000000	-4.70837100000000
Si	-8.49409200000000	-0.65658600000000	4.32200800000000
Si	-1.24374600000000	-6.07029300000000	-7.26014100000000
Si	-6.64841800000000	2.28231900000000	-0.35222700000000
Si	-2.43808200000000	-7.48776000000000	-0.05814600000000
Si	-0.61390400000000	-5.85107100000000	-1.89294300000000
Si	-8.43425700000000	0.64184800000000	1.50238700000000
Si	-5.64513400000000	0.55670600000000	-2.61180400000000
Si	2.25281400000000	-6.02077800000000	-0.80838500000000
Si	-8.49919500000000	-2.23388000000000	0.41220000000000
Si	-6.71185000000000	-1.75995900000000	-4.17734200000000
Si	-8.52348600000000	-3.44796000000000	-2.35419400000000
Si	-4.45210100000000	-3.92260200000000	-3.97946300000000
Si	-6.26321700000000	-5.61933700000000	-2.14534100000000
Si	-1.92192500000000	-3.09500800000000	-2.41216400000000
Si	-4.95876300000000	-5.82319900000000	0.69541200000000
Al	-3.68968200000000	-1.16826500000000	-0.80726600000000
Si	-5.53945000000000	-2.82528200000000	1.24488900000000
Si	-2.11149000000000	-1.54948500000000	-6.91047700000000
H	11.67374700000000	-5.53703600000000	-0.03807400000000
H	9.19348300000000	-8.10585800000000	2.36346300000000
H	-3.88155000000000	1.39981100000000	-8.25725800000000
H	-5.54661900000000	-0.07985100000000	-7.37196300000000
H	-6.93206200000000	-1.73335100000000	-5.60666700000000

H	-9.61083500000000	-4.23371600000000	-2.91527900000000
H	-4.80806200000000	-4.38616400000000	-7.98466800000000
H	-1.25558100000000	-7.52827100000000	-7.35483800000000
H	-8.46603500000000	2.53218800000000	6.77163500000000
H	-4.20950400000000	6.50518800000000	2.49497300000000
H	-1.81143200000000	9.00591900000000	0.20164700000000
H	0.67612800000000	11.32783200000000	-2.29765500000000
H	-4.95337000000000	5.93157200000000	-3.34598500000000
H	-7.43212800000000	3.36467300000000	-0.94325500000000
H	-9.81433500000000	1.14100200000000	1.45030700000000
H	-3.26082300000000	2.50672900000000	9.43326900000000
H	-0.74504100000000	4.85495100000000	6.90563300000000
H	1.17176500000000	6.84606700000000	4.60638300000000
H	0.01383700000000	-0.85268900000000	9.75320600000000
H	2.65630400000000	1.61378700000000	7.09828800000000
H	4.53076900000000	3.47379800000000	4.86924400000000
H	1.53720800000000	-6.93798300000000	-4.97365700000000
H	4.63541500000000	9.21535600000000	-0.64625200000000
H	-8.67488800000000	0.53574600000000	8.08886500000000
H	-5.67875100000000	-0.35844200000000	8.92691800000000
H	-5.40516100000000	-3.45826600000000	7.74597000000000
H	-2.86007300000000	-3.03986700000000	9.10083200000000
H	-1.22701000000000	-4.11162700000000	7.70400900000000
H	2.57413300000000	5.42807900000000	-6.16363200000000
H	0.22482900000000	8.10275900000000	-5.85603200000000
H	2.79507600000000	11.25689300000000	-3.42192600000000
H	6.06067100000000	7.26917500000000	-3.76691100000000
H	10.01065000000000	4.23779400000000	-2.76113100000000
H	9.87926500000000	0.42371400000000	-3.59460900000000
H	11.16639500000000	0.78744100000000	-1.60472500000000
H	9.89847200000000	3.90341200000000	-0.38688400000000
H	-1.32399900000000	-1.19594400000000	-8.10330100000000
H	-0.61662900000000	-5.49814400000000	-8.46011600000000
H	2.84818300000000	-3.04332200000000	-5.95038400000000
H	-7.31517900000000	-4.15812800000000	3.75060700000000
H	-9.78250000000000	-1.30915800000000	4.07997000000000
H	-9.40319700000000	-2.75383200000000	1.45013700000000
H	-5.76898900000000	-6.41021300000000	1.77986100000000
H	-3.08084500000000	-8.75083000000000	-0.46797100000000
H	-7.02180600000000	-6.69287800000000	-2.78419800000000
H	2.78359000000000	-7.36886300000000	-0.99247300000000
H	4.89691600000000	-9.57738000000000	2.49409400000000
H	5.82084500000000	-9.04713700000000	0.34374500000000
H	-1.34437700000000	-7.77605500000000	0.87467400000000
H	10.66950900000000	-2.02029700000000	2.56380700000000
H	8.82314100000000	-3.95574100000000	4.78387700000000
H	11.59080000000000	-3.97453400000000	-1.85888000000000
H	5.33815600000000	-0.46451100000000	4.51995500000000
H	7.15219500000000	1.34004700000000	2.37234700000000
H	7.32251200000000	6.57841100000000	-0.51330700000000
H	5.00684300000000	-4.86484800000000	-3.24385800000000
H	7.90214800000000	-7.00114300000000	-1.50659200000000
H	2.44258300000000	-6.49974700000000	2.91143200000000
Ga	-3.95759100000000	-0.05330300000000	1.78145800000000
O	-4.08227200000000	0.69050200000000	3.26784100000000

Complex (GaO-zeolite + C_2H_6)

Electronic energy (r^2SCAN-3c): -30692.552787355024 E_h
Electronic energy (ωB97X-D4rev/def2-mTZVPP): -30700.773210613967 E_h
G_{corr} (673.15 K, 1 atm): 0.41606152 E_h
Number of atoms: 249

Cartesian coordinates (Å)

O	1.48271500000000	2.85466700000000	1.00005100000000
O	-1.95151200000000	6.76413900000000	1.41796200000000
O	5.26848900000000	2.07774200000000	0.80488000000000
O	3.60597800000000	3.87857400000000	-0.14879800000000
O	6.09594700000000	4.57384200000000	0.41638200000000
O	1.46153000000000	3.28977100000000	-1.62207600000000
O	1.36522400000000	5.31016300000000	0.04769500000000
O	-1.72263500000000	6.89384200000000	-1.19774000000000
O	0.31979900000000	7.69066900000000	0.39547900000000
O	2.92752200000000	7.46596500000000	-0.05738800000000
Si	4.84917900000000	3.63946900000000	0.87201800000000
Si	1.98817900000000	3.83362800000000	-0.18698500000000
Si	-1.30054300000000	7.62592900000000	0.20021500000000
Si	1.64466900000000	6.78539200000000	0.65976400000000
O	8.95212900000000	-3.83488100000000	2.22517400000000
O	1.99433200000000	6.65847300000000	2.24366800000000
O	7.14402700000000	-2.27923700000000	-0.92345800000000
O	2.50781500000000	4.75258600000000	4.07888500000000
O	6.74879100000000	-4.90439300000000	-0.77376500000000
O	0.04714900000000	5.11496000000000	3.13919800000000
O	5.85458800000000	-3.26939300000000	1.13682600000000
O	4.45111500000000	4.07760200000000	2.38936400000000
O	9.40141400000000	-5.01127100000000	-1.12788400000000
O	5.15900100000000	-0.25199200000000	2.01737300000000
O	7.73450000000000	4.37946600000000	-1.69165600000000
O	10.70355300000000	-3.18509000000000	0.34590400000000
O	5.42934000000000	5.63303400000000	-1.91057800000000
O	4.08410700000000	5.66701700000000	-4.16730500000000
O	8.76158000000000	-1.46150100000000	1.04379300000000
O	3.92388700000000	7.74006200000000	-2.53292800000000
O	1.17010800000000	9.51885300000000	-4.04306500000000
O	8.98076600000000	2.07172800000000	-1.88662800000000
O	6.94930700000000	0.19137000000000	0.10183700000000
O	2.27339800000000	9.55383100000000	-1.56228300000000
O	9.00031000000000	-0.54848400000000	-1.44377800000000
Si	1.42917600000000	5.86189600000000	3.55186000000000
Si	6.11672900000000	-3.44035200000000	-0.44862800000000
Si	3.63253600000000	3.62681500000000	3.72470900000000
Si	8.07937700000000	-5.83631500000000	-0.65679800000000
Si	10.87676200000000	-4.45879700000000	-0.67185900000000
Si	6.67175800000000	5.32675600000000	-0.90955300000000
Si	9.79499300000000	-2.60853600000000	1.56634500000000
Si	4.92207500000000	6.59395500000000	-3.11922800000000
Si	9.20519000000000	3.67344800000000	-1.66777100000000
Si	6.17294800000000	0.85277900000000	1.37316100000000
Si	1.73019700000000	10.45247500000000	-2.82355400000000
Si	9.80054400000000	0.68321900000000	-2.15362400000000
Si	7.95563300000000	-1.01581800000000	-0.30306700000000
Si	3.46792800000000	8.51498300000000	-1.17783300000000
O	2.98067300000000	-0.46755200000000	3.61216800000000

O	0.95434500000000	0.75682000000000	2.48725900000000
O	1.26259300000000	0.90204400000000	5.08647200000000
O	2.97142700000000	2.16629300000000	3.46120500000000
O	-0.44451000000000	1.14517000000000	0.31275700000000
O	-0.81841700000000	2.74155400000000	2.34781700000000
O	-3.77205100000000	4.97784300000000	0.58165000000000
O	-2.53075000000000	4.66591000000000	2.93663200000000
O	-1.15395400000000	3.42705400000000	4.84068500000000
Si	2.05095900000000	0.84762600000000	3.67939700000000
Si	0.29725500000000	1.89349800000000	1.54411400000000
Si	-3.13829100000000	5.74026800000000	1.87464600000000
Si	-1.11137000000000	3.99398100000000	3.33312200000000
O	8.28956800000000	-6.04345000000000	3.49638300000000
O	-3.19828300000000	-3.07060400000000	6.58186900000000
O	6.68151100000000	-7.84685400000000	2.44475800000000
O	-1.30854900000000	-1.57138300000000	7.69972200000000
O	8.25554200000000	-6.34231600000000	0.87202000000000
O	-5.41951200000000	-3.48638400000000	5.24322700000000
O	6.65250300000000	-3.92555900000000	3.57241700000000
O	-5.13956600000000	-1.22320800000000	6.59439900000000
O	-8.67134800000000	0.34172200000000	5.57942800000000
O	4.47586500000000	-2.51303400000000	3.25152400000000
O	-6.44562100000000	1.05308300000000	6.96320500000000
O	4.49239900000000	-5.06573200000000	2.60633900000000
O	-3.87754900000000	0.84392200000000	7.58979300000000
O	-2.25510700000000	2.85225900000000	7.14836300000000
O	0.39790000000000	2.65916600000000	6.87729400000000
O	4.25487600000000	-7.39981000000000	1.37461400000000
O	-1.50155400000000	0.87226700000000	8.78466500000000
O	0.71907400000000	0.07735500000000	7.52317600000000
Si	8.12659800000000	-7.12388200000000	2.28722000000000
Si	-2.12868300000000	-2.97459300000000	7.81890700000000
Si	8.20075600000000	-4.41708100000000	3.55243300000000
Si	-4.80652500000000	-2.81196000000000	6.58703700000000
Si	4.52934500000000	-0.89514100000000	3.37717000000000
Si	-8.07820000000000	1.13069500000000	6.88047100000000
Si	5.36112800000000	-3.70496100000000	2.61555300000000
Si	-5.29934000000000	0.07123800000000	7.56628100000000
Si	-0.93820700000000	3.48664600000000	6.44625700000000
Si	3.44434000000000	-6.09995600000000	1.92682200000000
Si	-2.74991500000000	1.78601300000000	8.26930400000000
Si	1.29606600000000	1.32275300000000	6.65588100000000
Si	5.41712300000000	-8.50895500000000	1.64901200000000
Si	-0.51647600000000	-0.38446600000000	8.47539000000000
O	1.78163300000000	4.26734900000000	-4.07183000000000
O	-0.06710700000000	5.27366900000000	-2.45665700000000
O	-0.62328200000000	7.68007500000000	-3.47013500000000
Si	0.69685100000000	3.92009100000000	-2.91325300000000
Si	-1.20691900000000	6.40639900000000	-2.65470000000000
O	1.24769600000000	-1.73155900000000	-4.49621200000000
O	-3.98555700000000	3.93815300000000	-4.54219100000000
O	3.34188100000000	-2.96073800000000	-3.48257900000000
O	-2.44640800000000	5.80482300000000	-3.50446100000000
O	-3.83555000000000	4.16792100000000	-1.91096100000000
O	1.33136700000000	-4.41081200000000	-4.41605500000000
O	-4.66665900000000	1.77086700000000	-5.91504600000000
O	4.72018100000000	-3.30802600000000	-1.25678400000000
O	-2.56588200000000	1.69402700000000	-4.36188400000000

O	-3.17082300000000	-0.36774700000000	-6.53564300000000
O	-1.11105400000000	-1.79010800000000	-5.65650700000000
O	-0.33107400000000	0.32716900000000	-4.19322400000000
O	-0.38724100000000	2.85249300000000	-3.46060700000000
O	1.77795900000000	6.92498400000000	-4.26996900000000
Si	-3.84302500000000	4.99767900000000	-3.31048200000000
Si	2.19347500000000	-3.04546800000000	-4.63891900000000
Si	-4.05540700000000	2.31505900000000	-4.52303500000000
Si	4.00933800000000	-4.07739000000000	-2.50604900000000
Si	-4.32520300000000	0.66669500000000	-7.07053900000000
Si	-0.30308600000000	-1.29247500000000	-4.34204900000000
Si	-1.23017100000000	1.47106200000000	-3.48579400000000
Si	2.54878800000000	5.56319700000000	-4.71060300000000
Si	0.62368000000000	8.05113700000000	-4.45428900000000
O	-2.79790100000000	-5.59401400000000	-7.11590100000000
O	-4.52394500000000	-4.33568600000000	-5.54021300000000
O	-2.91150900000000	-2.93084500000000	-7.21620000000000
O	-2.90206200000000	0.14656000000000	0.17256300000000
O	-2.48486700000000	2.77496400000000	-0.14784300000000
O	-1.67594900000000	0.98707700000000	-1.99124100000000
O	-5.11922300000000	2.76071300000000	-0.02173900000000
Si	-3.76593400000000	-4.29606300000000	-6.97878300000000
Si	-1.86965400000000	1.30557500000000	-0.42197300000000
Si	-3.82641400000000	3.67906100000000	-0.37203600000000
O	-5.02124500000000	1.89972900000000	-3.28727400000000
O	2.81407100000000	-5.05145400000000	-1.98225700000000
O	-5.61109300000000	-2.51710100000000	2.82086100000000
O	-7.39332100000000	-1.80025600000000	4.67997300000000
O	-0.24885500000000	-6.31327500000000	-3.40523300000000
O	-8.01770600000000	0.16826800000000	2.99958000000000
O	-0.39379300000000	-5.57335400000000	-5.96188500000000
O	-0.96513600000000	-1.92722800000000	-3.01097100000000
O	-3.48487500000000	-6.49512700000000	0.72629300000000
O	-0.96158300000000	-4.27840300000000	-1.87092300000000
O	-6.59899800000000	1.02230200000000	-1.38094700000000
O	0.62847700000000	-6.09059100000000	-0.87162500000000
O	-8.35753400000000	-0.62380700000000	0.47649900000000
O	-7.36876100000000	1.77549800000000	1.01499600000000
O	-1.87282600000000	-6.75241200000000	-1.40699400000000
O	-6.54755000000000	-0.21394500000000	-3.71053700000000
O	2.70817800000000	-5.41924500000000	0.64150300000000
O	-9.08217400000000	-2.65836700000000	-1.04124900000000
O	-4.47438000000000	-0.40829300000000	-2.09815100000000
O	-7.02446500000000	-2.91231700000000	0.59709200000000
O	-8.04323000000000	-2.38499900000000	-3.48274700000000
O	-5.38751100000000	-2.61336900000000	-3.74146800000000
O	-7.26627500000000	-4.36473300000000	-1.88617200000000
O	-5.00907100000000	-5.15354500000000	-3.07330300000000
O	-2.90361300000000	-3.62781900000000	-3.60173900000000
O	-5.70018200000000	-6.15158800000000	-0.71623300000000
O	-2.76086700000000	-2.53514200000000	-1.16385800000000
O	-4.76429500000000	-4.19457200000000	0.88805000000000
O	-4.71352000000000	-1.50838500000000	0.65213300000000
Si	-6.47904600000000	-3.01297900000000	4.11434100000000
Si	0.60047600000000	-5.83356900000000	-4.70842200000000
Si	-8.49503800000000	-0.65443800000000	4.32005500000000
Si	-1.24398700000000	-6.07021800000000	-7.26029100000000
Si	-6.64913100000000	2.28313400000000	-0.35322500000000

Si	-2.43916000000000	-7.48714500000000	-0.05766400000000
Si	-0.61446100000000	-5.85187600000000	-1.89247700000000
Si	-8.43253000000000	0.64498500000000	1.50273900000000
Si	-5.63920400000000	0.55697300000000	-2.60957300000000
Si	2.25225200000000	-6.02114500000000	-0.80883000000000
Si	-8.50095600000000	-2.23496800000000	0.41329800000000
Si	-6.71133300000000	-1.75895900000000	-4.17677500000000
Si	-8.52461300000000	-3.44542500000000	-2.35483500000000
Si	-4.45186400000000	-3.92241700000000	-3.97767000000000
Si	-6.26438400000000	-5.61796600000000	-2.14525600000000
Si	-1.92127900000000	-3.09493400000000	-2.40739900000000
Si	-4.95879100000000	-5.81867700000000	0.69628900000000
Al	-3.69048100000000	-1.16926000000000	-0.80492800000000
Si	-5.54706800000000	-2.82235100000000	1.23730900000000
Si	-2.11172800000000	-1.54897800000000	-6.91023800000000
H	11.67374700000000	-5.53703600000000	-0.03807400000000
H	9.19348300000000	-8.10585800000000	2.36346300000000
H	-3.88155000000000	1.39981100000000	-8.25725800000000
H	-5.54661900000000	-0.07985100000000	-7.37196300000000
H	-6.93206200000000	-1.73335100000000	-5.60666700000000
H	-9.61083500000000	-4.23371600000000	-2.91527900000000
H	-4.80806200000000	-4.38616400000000	-7.98466800000000
H	-1.25558100000000	-7.52827100000000	-7.35483800000000
H	-8.46603500000000	2.53218800000000	6.77163500000000
H	-4.20950400000000	6.50518800000000	2.49497300000000
H	-1.81143200000000	9.00591900000000	0.20164700000000
H	0.67612800000000	11.32783200000000	-2.29765500000000
H	-4.95337000000000	5.93157200000000	-3.34598500000000
H	-7.43212800000000	3.36467300000000	-0.94325500000000
H	-9.81433500000000	1.14100200000000	1.45030700000000
H	-3.26082300000000	2.50672900000000	9.43326900000000
H	-0.74504100000000	4.85495100000000	6.90563300000000
H	1.17176500000000	6.84606700000000	4.60638300000000
H	0.01383700000000	-0.85268900000000	9.75320600000000
H	2.65630400000000	1.61378700000000	7.09828800000000
H	4.53076900000000	3.47379800000000	4.86924400000000
H	1.53720800000000	-6.93798300000000	-4.97365700000000
H	4.63541500000000	9.21535600000000	-0.64625200000000
H	-8.67488800000000	0.53574600000000	8.08886500000000
H	-5.67875100000000	-0.35844200000000	8.92691800000000
H	-5.40516100000000	-3.45826600000000	7.74597000000000
H	-2.86007300000000	-3.03986700000000	9.10083200000000
H	-1.22701000000000	-4.11162700000000	7.70400900000000
H	2.57413300000000	5.42807900000000	-6.16363200000000
H	0.22482900000000	8.10275900000000	-5.85603200000000
H	2.79507600000000	11.25689300000000	-3.42192600000000
H	6.06067100000000	7.26917500000000	-3.76691100000000
H	10.01065000000000	4.23779400000000	-2.76113100000000
H	9.87926500000000	0.42371400000000	-3.59460900000000
H	11.16639500000000	0.78744100000000	-1.60472500000000
H	9.89847200000000	3.90341200000000	-0.38688400000000
H	-1.32399900000000	-1.19594400000000	-8.10330100000000
H	-0.61662900000000	-5.49814400000000	-8.46011600000000
H	2.84818300000000	-3.04332200000000	-5.95038400000000
H	-7.31517900000000	-4.15812800000000	3.75060700000000
H	-9.78250000000000	-1.30915800000000	4.07997000000000
H	-9.40319700000000	-2.75383200000000	1.45013700000000
H	-5.76898900000000	-6.41021300000000	1.77986100000000

H	-3.08084500000000	-8.75083000000000	-0.46797100000000
H	-7.02180600000000	-6.69287800000000	-2.78419800000000
H	2.78359000000000	-7.36886300000000	-0.99247300000000
H	4.89691600000000	-9.57738000000000	2.49409400000000
H	5.82084500000000	-9.04713700000000	0.34374500000000
H	-1.34437700000000	-7.77605500000000	0.87467400000000
H	10.66950900000000	-2.02029700000000	2.56380700000000
H	8.82314100000000	-3.95574100000000	4.78387700000000
H	11.59080000000000	-3.97453400000000	-1.85888000000000
H	5.33815600000000	-0.46451100000000	4.51995500000000
H	7.15219500000000	1.34004700000000	2.37234700000000
H	7.32251200000000	6.57841100000000	-0.51330700000000
H	5.00684300000000	-4.86484800000000	-3.24385800000000
H	7.90214800000000	-7.00114300000000	-1.50659200000000
H	2.44258300000000	-6.49974700000000	2.91143200000000
Ga	-4.08960900000000	0.04029300000000	1.72295200000000
C	-1.80020000000000	-0.56271200000000	4.36142700000000
H	-2.62559400000000	-0.62924200000000	5.07525200000000
H	-1.83127300000000	0.44397600000000	3.93441900000000
H	-0.86047500000000	-0.67743600000000	4.90895300000000
C	-1.91051000000000	-1.63826600000000	3.28783100000000
H	-1.82308900000000	-2.64576600000000	3.70826000000000
H	-1.15219900000000	-1.52728900000000	2.50504900000000
H	-2.90745000000000	-1.63019600000000	2.80754900000000
O	-4.50714800000000	0.97971500000000	3.03772900000000

TS1-GaO-I

Electronic energy (r[2]SCAN-3c): -30692.511211655354 E_h
Electronic energy (ωB97X-D4rev/def2-mTZVPP): -30700.728790613146 E_h
G_corr (673.15 K, 1 atm): 0.42022806 E_h
Number of atoms: 249

Cartesian coordinates (Å)

O	1.54334700000000	2.85940700000000	1.02830100000000
O	-1.92703100000000	6.75408200000000	1.43965200000000
O	5.29354000000000	2.07020600000000	0.82160200000000
O	3.64626400000000	3.88590600000000	-0.13948800000000
O	6.13084600000000	4.56447500000000	0.44021600000000
O	1.49680100000000	3.28131000000000	-1.60055300000000
O	1.39637700000000	5.30728000000000	0.06142300000000
O	-1.69060000000000	6.88307500000000	-1.17528700000000
O	0.34572300000000	7.68438800000000	0.42089900000000
O	2.95442200000000	7.46248100000000	-0.03396500000000
Si	4.87911000000000	3.63344100000000	0.89028900000000
Si	2.02839600000000	3.83428900000000	-0.17062700000000
Si	-1.27444400000000	7.61641800000000	0.22352100000000
Si	1.67173400000000	6.77987300000000	0.68144000000000
O	8.97084200000000	-3.83887300000000	2.24367600000000
O	2.02272100000000	6.64895300000000	2.26415000000000
O	7.17184300000000	-2.28977800000000	-0.90224500000000
O	2.53429400000000	4.74645200000000	4.09981000000000
O	6.77455000000000	-4.91392500000000	-0.75145100000000
O	0.06991100000000	5.11359300000000	3.16480700000000
O	5.88062700000000	-3.27694600000000	1.15814400000000
O	4.47307300000000	4.06353900000000	2.40766700000000
O	9.42686400000000	-5.01990300000000	-1.11075700000000

O	5.17926500000000	-0.25622800000000	2.03822600000000
O	7.76073600000000	4.37199600000000	-1.67439400000000
O	10.72775400000000	-3.19569100000000	0.36647300000000
O	5.45552600000000	5.62452000000000	-1.88927200000000
O	4.11416100000000	5.66182000000000	-4.15128600000000
O	8.79014900000000	-1.46555300000000	1.06221800000000
O	3.94909200000000	7.72998000000000	-2.51194400000000
O	1.18857200000000	9.50770600000000	-4.01967800000000
O	9.00628200000000	2.06328900000000	-1.86708100000000
O	6.97134800000000	0.18061500000000	0.12189300000000
O	2.29916100000000	9.54559600000000	-1.54334600000000
O	9.02318100000000	-0.55622900000000	-1.42643300000000
Si	1.45415200000000	5.85440300000000	3.57232500000000
Si	6.14314900000000	-3.44934600000000	-0.42722900000000
Si	3.65684200000000	3.61944000000000	3.74687900000000
Si	8.10573100000000	-5.84532400000000	-0.63783600000000
Si	10.90187700000000	-4.46785000000000	-0.65296700000000
Si	6.69877100000000	5.31785100000000	-0.88932500000000
Si	9.81933000000000	-2.61587000000000	1.58545400000000
Si	4.94899700000000	6.58551400000000	-3.09841200000000
Si	9.23065700000000	3.66509000000000	-1.64883100000000
Si	6.19624000000000	0.84523300000000	1.39258600000000
Si	1.75472200000000	10.44309400000000	-2.80456000000000
Si	9.82564300000000	0.67489200000000	-2.13475800000000
Si	7.98070800000000	-1.02353400000000	-0.28376600000000
Si	3.49357900000000	8.50718600000000	-1.15799500000000
O	2.99953300000000	-0.47527400000000	3.63284100000000
O	0.97920400000000	0.76928700000000	2.50727300000000
O	1.26777700000000	0.87724600000000	5.10884900000000
O	2.99449900000000	2.15649600000000	3.50434100000000
O	-0.37751200000000	1.16640200000000	0.30715200000000
O	-0.78223500000000	2.76132000000000	2.34006800000000
O	-3.72713200000000	4.94231800000000	0.61611400000000
O	-2.50514900000000	4.66876400000000	2.98232900000000
O	-1.10757200000000	3.40531000000000	4.85757400000000
Si	2.06885000000000	0.84033700000000	3.70620400000000
Si	0.33943400000000	1.91036400000000	1.55453200000000
Si	-3.10907100000000	5.72649900000000	1.90267600000000
Si	-1.08493200000000	3.98510200000000	3.35416200000000
O	8.31413800000000	-6.05020500000000	3.51395900000000
O	-3.17154100000000	-3.07561100000000	6.60006000000000
O	6.70751600000000	-7.85472100000000	2.46245000000000
O	-1.28288600000000	-1.57636800000000	7.70911700000000
O	8.28624600000000	-6.35260200000000	0.88998000000000
O	-5.39221000000000	-3.49100100000000	5.26076900000000
O	6.67419300000000	-3.93420800000000	3.59504400000000
O	-5.11365500000000	-1.23031900000000	6.61227600000000
O	-8.64900300000000	0.34731800000000	5.58991600000000
O	4.49783900000000	-2.51897000000000	3.27036000000000
O	-6.42158300000000	1.04119900000000	6.97825900000000
O	4.51448600000000	-5.07155500000000	2.62581200000000
O	-3.85361100000000	0.83900000000000	7.60965200000000
O	-2.22687300000000	2.84498700000000	7.16705100000000
O	0.42515700000000	2.64783900000000	6.90473700000000
O	4.28130700000000	-7.40694100000000	1.39351700000000
O	-1.47841900000000	0.86181100000000	8.80381400000000
O	0.75072800000000	0.06846800000000	7.55311900000000
Si	8.15288900000000	-7.13192800000000	2.30583300000000

Si	-2.10128800000000	-2.98108700000000	7.83764200000000
Si	8.22331300000000	-4.42387900000000	3.57183300000000
Si	-4.77935900000000	-2.81830700000000	6.60750700000000
Si	4.54962700000000	-0.90059200000000	3.39744300000000
Si	-8.05373000000000	1.12304100000000	6.89950600000000
Si	5.38418800000000	-3.71117200000000	2.63632600000000
Si	-5.27225900000000	0.06392400000000	7.58563300000000
Si	-0.90790600000000	3.47650500000000	6.46349200000000
Si	3.46926500000000	-6.10798600000000	1.94584300000000
Si	-2.72334200000000	1.78026300000000	8.28742900000000
Si	1.32030000000000	1.31080600000000	6.67615600000000
Si	5.44267800000000	-8.51726600000000	1.66791800000000
Si	-0.49073900000000	-0.39270000000000	8.49514600000000
O	1.81808100000000	4.25895900000000	-4.04982700000000
O	-0.03334400000000	5.26172200000000	-2.43544000000000
O	-0.60283400000000	7.66038600000000	-3.45677000000000
Si	0.73245200000000	3.90956700000000	-2.89312900000000
Si	-1.17855000000000	6.38923600000000	-2.63089600000000
O	1.27019000000000	-1.74763500000000	-4.46980500000000
O	-3.96478200000000	3.92495500000000	-4.51454300000000
O	3.37025600000000	-2.97000400000000	-3.46404900000000
O	-2.41942600000000	5.77548000000000	-3.46858000000000
O	-3.84949600000000	4.16055400000000	-1.87743100000000
O	1.36140000000000	-4.42388900000000	-4.39578400000000
O	-4.63886800000000	1.75904000000000	-5.89315600000000
O	4.74709000000000	-3.31711500000000	-1.23630300000000
O	-2.52857800000000	1.69155700000000	-4.35240500000000
O	-3.14606200000000	-0.38064000000000	-6.51697000000000
O	-1.08295400000000	-1.80672300000000	-5.64222700000000
O	-0.29877600000000	0.31658000000000	-4.19439000000000
O	-0.34631600000000	2.84173400000000	-3.44887700000000
O	1.80528200000000	6.91705200000000	-4.25166500000000
Si	-3.82442800000000	4.98219500000000	-3.28020500000000
Si	2.22020100000000	-3.05672500000000	-4.61871900000000
Si	-4.02351800000000	2.30194100000000	-4.50192300000000
Si	4.03655400000000	-4.08579400000000	-2.48612300000000
Si	-4.29890800000000	0.65670300000000	-7.05075000000000
Si	-0.28012100000000	-1.30396700000000	-4.32656800000000
Si	-1.19119400000000	1.46187300000000	-3.47974200000000
Si	2.57820800000000	5.55620000000000	-4.69186200000000
Si	0.64666600000000	8.03905400000000	-4.43476900000000
O	-2.77305900000000	-5.60523800000000	-7.09536600000000
O	-4.51094000000000	-4.34865800000000	-5.52926000000000
O	-2.88534600000000	-2.94342200000000	-7.19787900000000
O	-2.79088200000000	0.08248800000000	0.17583300000000
O	-2.46093700000000	2.73760000000000	-0.15887000000000
O	-1.62781400000000	0.96648500000000	-1.98983600000000
O	-5.09767100000000	2.74200700000000	0.01776600000000
Si	-3.74162800000000	-4.30725600000000	-6.96158500000000
Si	-1.81392300000000	1.27828000000000	-0.41727200000000
Si	-3.79956800000000	3.64904500000000	-0.34559400000000
O	-4.97932900000000	1.87854400000000	-3.26187900000000
O	2.84110100000000	-5.05938600000000	-1.96195200000000
O	-5.62484400000000	-2.49789100000000	2.84939500000000
O	-7.38291300000000	-1.81305200000000	4.72651900000000
O	-0.22603400000000	-6.32036100000000	-3.38727400000000
O	-7.97906600000000	0.14336500000000	3.01465900000000
O	-0.36706300000000	-5.58151400000000	-5.94395600000000

O	-0.95568600000000	-1.93054500000000	-2.99656400000000
O	-3.45672500000000	-6.50181100000000	0.74663700000000
O	-0.94240400000000	-4.28269400000000	-1.85625400000000
O	-6.59994400000000	1.02852200000000	-1.37613600000000
O	0.65257700000000	-6.09304400000000	-0.85459400000000
O	-8.35173900000000	-0.62979200000000	0.48834600000000
O	-7.33513400000000	1.75796300000000	1.04370200000000
O	-1.84730400000000	-6.75886700000000	-1.38743600000000
O	-6.52076300000000	-0.22103400000000	-3.69681400000000
O	2.73064400000000	-5.42852700000000	0.66140100000000
O	-9.05878600000000	-2.67635200000000	-1.01963000000000
O	-4.48029200000000	-0.42329800000000	-2.04778000000000
O	-6.97598400000000	-2.88671400000000	0.59196700000000
O	-8.01447700000000	-2.39101400000000	-3.45994300000000
O	-5.35800100000000	-2.62205100000000	-3.72664800000000
O	-7.24112200000000	-4.37498000000000	-1.86879600000000
O	-4.98397500000000	-5.16346900000000	-3.05952300000000
O	-2.87314000000000	-3.64983300000000	-3.60954600000000
O	-5.66885100000000	-6.14683000000000	-0.69552300000000
O	-2.77849700000000	-2.56654000000000	-1.17433800000000
O	-4.74383500000000	-4.20967400000000	0.95182600000000
O	-4.61709100000000	-1.53732800000000	0.72985200000000
Si	-6.46798000000000	-3.01947300000000	4.14688000000000
Si	0.62584700000000	-5.84318000000000	-4.68997600000000
Si	-8.47344600000000	-0.66321300000000	4.34271000000000
Si	-1.21862500000000	-6.07911700000000	-7.24127200000000
Si	-6.62405400000000	2.27400000000000	-0.33096100000000
Si	-2.41287300000000	-7.49526300000000	-0.03927100000000
Si	-0.59101300000000	-5.85586800000000	-1.87541200000000
Si	-8.40867500000000	0.63218100000000	1.52016800000000
Si	-5.62432900000000	0.54938800000000	-2.58554700000000
Si	2.27684400000000	-6.02911300000000	-0.79029400000000
Si	-8.47194500000000	-2.24257800000000	0.42849200000000
Si	-6.68288300000000	-1.76704100000000	-4.15858600000000
Si	-8.49828400000000	-3.45509200000000	-2.33740700000000
Si	-4.42773700000000	-3.93437300000000	-3.96781800000000
Si	-6.23678600000000	-5.62538300000000	-2.12802100000000
Si	-1.90887300000000	-3.10777200000000	-2.40429100000000
Si	-4.93170900000000	-5.82826800000000	0.72164900000000
Al	-3.66403700000000	-1.17700000000000	-0.77580200000000
Si	-5.51373500000000	-2.82389500000000	1.27029700000000
Si	-2.08629600000000	-1.56095000000000	-6.89261000000000
H	11.69917500000000	-5.54591300000000	-0.01904600000000
H	9.21891100000000	-8.11473500000000	2.38249100000000
H	-3.85612200000000	1.39093400000000	-8.23823000000000
H	-5.52119100000000	-0.08872800000000	-7.35293500000000
H	-6.90663400000000	-1.74222800000000	-5.58763900000000
H	-9.58540700000000	-4.24259300000000	-2.89625100000000
H	-4.78263400000000	-4.39504100000000	-7.96564000000000
H	-1.23015300000000	-7.53714800000000	-7.33581000000000
H	-8.44060700000000	2.52331100000000	6.79066300000000
H	-4.18407600000000	6.49631100000000	2.51400100000000
H	-1.78600400000000	8.99704200000000	0.22067500000000
H	0.70155600000000	11.31895500000000	-2.27862700000000
H	-4.92794200000000	5.92269500000000	-3.32695700000000
H	-7.40670000000000	3.35579600000000	-0.92422700000000
H	-9.78890700000000	1.13212500000000	1.46933500000000
H	-3.23539500000000	2.49785200000000	9.45229700000000

H	-0.71961300000000	4.84607400000000	6.92466100000000
H	1.19719300000000	6.83719000000000	4.62541100000000
H	0.03926500000000	-0.86156600000000	9.77223400000000
H	2.68173200000000	1.60491000000000	7.11731600000000
H	4.55619700000000	3.46492100000000	4.88827200000000
H	1.56263600000000	-6.94686000000000	-4.95462900000000
H	4.66084300000000	9.20647900000000	-0.62722400000000
H	-8.64946000000000	0.52686900000000	8.10789300000000
H	-5.65332300000000	-0.36731900000000	8.94594600000000
H	-5.37973300000000	-3.46714300000000	7.76499800000000
H	-2.83464500000000	-3.04874400000000	9.11986000000000
H	-1.20158200000000	-4.12050400000000	7.72303700000000
H	2.59956100000000	5.41920200000000	-6.14460400000000
H	0.25025700000000	8.09388200000000	-5.83700400000000
H	2.82050400000000	11.24801600000000	-3.40289800000000
H	6.08609900000000	7.26029800000000	-3.74788300000000
H	10.03607800000000	4.22891700000000	-2.74210300000000
H	9.90469300000000	0.41483700000000	-3.57558100000000
H	11.19182300000000	0.77856400000000	-1.58569700000000
H	9.92390000000000	3.89453500000000	-0.36785600000000
H	-1.29857100000000	-1.20482100000000	-8.08427300000000
H	-0.59120100000000	-5.50702100000000	-8.44108800000000
H	2.87361100000000	-3.05219900000000	-5.93135600000000
H	-7.28975100000000	-4.16700500000000	3.76963500000000
H	-9.75707200000000	-1.31803500000000	4.09899800000000
H	-9.37776900000000	-2.76270900000000	1.46916500000000
H	-5.74356100000000	-6.41909000000000	1.79888900000000
H	-3.05541700000000	-8.75970700000000	-0.44894300000000
H	-6.99637800000000	-6.70175500000000	-2.76517000000000
H	2.80901800000000	-7.37774000000000	-0.97344500000000
H	4.92234400000000	-9.58625700000000	2.51312200000000
H	5.84627300000000	-9.05601400000000	0.36277300000000
H	-1.31894900000000	-7.78493200000000	0.89370200000000
H	10.69493700000000	-2.02917400000000	2.58283500000000
H	8.84856900000000	-3.96461800000000	4.80290500000000
H	11.61622800000000	-3.98341100000000	-1.83985200000000
H	5.36358400000000	-0.47338800000000	4.53898300000000
H	7.17762300000000	1.33117000000000	2.39137500000000
H	7.34794000000000	6.56953400000000	-0.49427900000000
H	5.03227100000000	-4.87372500000000	-3.22483000000000
H	7.92757600000000	-7.01002000000000	-1.48756400000000
H	2.46801100000000	-6.50862400000000	2.93046000000000
Ga	-3.67706200000000	-0.24507200000000	1.90731400000000
C	-2.35791400000000	-1.53672100000000	3.23703000000000
H	-3.09316700000000	-2.32876800000000	3.42258200000000
H	-2.83742900000000	-0.29699000000000	3.44166700000000
H	-1.91430000000000	-1.35494500000000	4.22924700000000
C	-1.24970500000000	-1.98698600000000	2.27002100000000
H	-0.64987400000000	-2.78949100000000	2.71357300000000
H	-0.56662100000000	-1.17007900000000	2.02467400000000
H	-1.65248000000000	-2.37949300000000	1.32903800000000
O	-3.70835100000000	0.87805600000000	3.22205700000000

I2-GaO-I

Electronic energy (r^{2}SCAN-3c): -30692.640708051567 E_h
Electronic energy (ωB97X-D4rev/def2-mTZVPP): -30700.867941572182 E_h
G_{corr} (673.15 K, 1 atm): 0.45696331 E_h
Number of atoms: 249

Cartesian coordinates (Å)

O	1.51793500000000	2.88232600000000	1.04628400000000
O	-1.91918400000000	6.76292400000000	1.44573100000000
O	5.29377500000000	2.06956200000000	0.82029500000000
O	3.63084100000000	3.87051800000000	-0.13985100000000
O	6.11653100000000	4.56823800000000	0.43800300000000
O	1.47017300000000	3.27930200000000	-1.59464500000000
O	1.39495200000000	5.31579400000000	0.05141800000000
O	-1.68998700000000	6.88157500000000	-1.17543100000000
O	0.34778200000000	7.69097400000000	0.42120500000000
O	2.95754300000000	7.46816000000000	-0.02944700000000
Si	4.86961500000000	3.63004600000000	0.88601800000000
Si	2.01173600000000	3.83458800000000	-0.17025900000000
Si	-1.27182800000000	7.61899300000000	0.21964600000000
Si	1.67232100000000	6.78421300000000	0.67789500000000
O	8.97747200000000	-3.84357700000000	2.24445900000000
O	2.02078400000000	6.64475600000000	2.26191400000000
O	7.17126100000000	-2.28976200000000	-0.90442700000000
O	2.53320500000000	4.74480800000000	4.10419700000000
O	6.77383000000000	-4.91402700000000	-0.75262400000000
O	0.07332700000000	5.10443600000000	3.16545300000000
O	5.87955700000000	-3.27620400000000	1.15534100000000
O	4.46351300000000	4.06040200000000	2.40439800000000
O	9.42664100000000	-5.02000000000000	-1.10896900000000
O	5.17801600000000	-0.25489500000000	2.03842400000000
O	7.75892200000000	4.36860300000000	-1.66832600000000
O	10.72899900000000	-3.19414600000000	0.36507100000000
O	5.45553800000000	5.62277400000000	-1.89267600000000
O	4.11691000000000	5.66342600000000	-4.15571000000000
O	8.78729500000000	-1.46998700000000	1.06343400000000
O	3.94823000000000	7.72776900000000	-2.51107400000000
O	1.20273800000000	9.51441300000000	-4.03194500000000
O	9.00642800000000	2.06289200000000	-1.86756600000000
O	6.97225100000000	0.17958800000000	0.12251400000000
O	2.29804000000000	9.54458300000000	-1.54585300000000
O	9.02524300000000	-0.55650600000000	-1.42373100000000
Si	1.45745700000000	5.85344400000000	3.57172100000000
Si	6.14158100000000	-3.44943300000000	-0.43047900000000
Si	3.65477400000000	3.61622400000000	3.74678200000000
Si	8.10471900000000	-5.84531600000000	-0.63764100000000
Si	10.90196300000000	-4.46780900000000	-0.65298600000000
Si	6.69584600000000	5.31792100000000	-0.88917800000000
Si	9.82047400000000	-2.61746400000000	1.58536000000000
Si	4.94899500000000	6.58541500000000	-3.10049600000000
Si	9.23042300000000	3.66449000000000	-1.64833900000000
Si	6.19679800000000	0.84484600000000	1.39187900000000
Si	1.75552700000000	10.44431000000000	-2.80692400000000
Si	9.82589100000000	0.67431200000000	-2.13455600000000
Si	7.98079300000000	-1.02565000000000	-0.28364800000000
Si	3.49395600000000	8.50862300000000	-1.15942000000000
O	3.00315800000000	-0.47978800000000	3.63792500000000

O	0.98341700000000	0.75390700000000	2.50070500000000
O	1.26295800000000	0.87089000000000	5.10432700000000
O	2.98813500000000	2.15439900000000	3.50879900000000
O	-0.38026000000000	1.16998500000000	0.30966100000000
O	-0.79325500000000	2.72247300000000	2.37949800000000
O	-3.71947800000000	4.97043400000000	0.58936000000000
O	-2.50768100000000	4.65639700000000	2.95492200000000
O	-1.15088800000000	3.41823900000000	4.85949600000000
Si	2.06946000000000	0.83204700000000	3.70202800000000
Si	0.33505300000000	1.89994300000000	1.55817700000000
Si	-3.10849500000000	5.73488300000000	1.88551700000000
Si	-1.08573800000000	3.98683000000000	3.34892500000000
O	8.31428200000000	-6.05218900000000	3.51545800000000
O	-3.17335400000000	-3.07548000000000	6.59918900000000
O	6.70699200000000	-7.85574900000000	2.46348700000000
O	-1.28619800000000	-1.57599800000000	7.70967000000000
O	8.28260700000000	-6.35151400000000	0.89106600000000
O	-5.40400700000000	-3.49366500000000	5.26935100000000
O	6.67730400000000	-3.93395800000000	3.59092200000000
O	-5.10353100000000	-1.22619300000000	6.60690300000000
O	-8.65144500000000	0.32688300000000	5.60154800000000
O	4.50108100000000	-2.52132000000000	3.27003100000000
O	-6.42182200000000	1.03619900000000	6.96395700000000
O	4.51666400000000	-5.07380700000000	2.62455600000000
O	-3.86343800000000	0.85414600000000	7.59592500000000
O	-2.22808500000000	2.84136600000000	7.16608900000000
O	0.42411500000000	2.64948900000000	6.88561200000000
O	4.28028700000000	-7.40847400000000	1.39407900000000
O	-1.48738800000000	0.85480800000000	8.80527700000000
O	0.74559000000000	0.07541800000000	7.55385400000000
Si	8.15201100000000	-7.13282100000000	2.30608700000000
Si	-2.10205500000000	-2.98271900000000	7.83644200000000
Si	8.22563400000000	-4.42584500000000	3.57127900000000
Si	-4.78148200000000	-2.82292900000000	6.60642900000000
Si	4.55314200000000	-0.90394900000000	3.39709200000000
Si	-8.05976500000000	1.11872800000000	6.89790600000000
Si	5.38611500000000	-3.71375500000000	2.63320600000000
Si	-5.27525000000000	0.06189200000000	7.58690600000000
Si	-0.91073200000000	3.47959200000000	6.46138300000000
Si	3.46859900000000	-6.10854000000000	1.94434300000000
Si	-2.72439500000000	1.77907800000000	8.28951800000000
Si	1.32124100000000	1.30940900000000	6.66922400000000
Si	5.44221900000000	-8.51768900000000	1.66782300000000
Si	-0.49160700000000	-0.39536700000000	8.49507400000000
O	1.82419300000000	4.26207000000000	-4.03861300000000
O	-0.05053400000000	5.26085300000000	-2.44928800000000
O	-0.58429000000000	7.68233800000000	-3.43817900000000
Si	0.72131300000000	3.90922600000000	-2.89861100000000
Si	-1.18159300000000	6.40326100000000	-2.63984200000000
O	1.27419600000000	-1.73782000000000	-4.48118800000000
O	-3.95378200000000	3.93878600000000	-4.53270700000000
O	3.36789300000000	-2.96734800000000	-3.46383100000000
O	-2.42419500000000	5.82990200000000	-3.50369200000000
O	-3.75401100000000	4.16822700000000	-1.90462000000000
O	1.36100500000000	-4.41939100000000	-4.39682700000000
O	-4.64216400000000	1.76655900000000	-5.89840400000000
O	4.74612800000000	-3.31616700000000	-1.23878200000000
O	-2.54531300000000	1.69234300000000	-4.34375900000000

O	-3.14363000000000	-0.37459900000000	-6.51576900000000
O	-1.08284700000000	-1.80889500000000	-5.64563100000000
O	-0.31117600000000	0.31886500000000	-4.20083300000000
O	-0.34723900000000	2.84410400000000	-3.47391900000000
O	1.80793500000000	6.91871800000000	-4.25391300000000
Si	-3.80770200000000	4.99777900000000	-3.30112000000000
Si	2.21856200000000	-3.05178800000000	-4.62076800000000
Si	-4.03419200000000	2.31459200000000	-4.50519400000000
Si	4.03425900000000	-4.08510700000000	-2.48882500000000
Si	-4.29966100000000	0.65922400000000	-7.05004400000000
Si	-0.27939100000000	-1.30173800000000	-4.33180400000000
Si	-1.19987000000000	1.46625300000000	-3.48347000000000
Si	2.57906900000000	5.55616300000000	-4.69146200000000
Si	0.65362500000000	8.04509400000000	-4.43575300000000
O	-2.77146500000000	-5.60078700000000	-7.09320300000000
O	-4.50986800000000	-4.34567900000000	-5.52616000000000
O	-2.89253000000000	-2.93754400000000	-7.20017100000000
O	-2.78759500000000	0.10017300000000	0.19018000000000
O	-2.45532100000000	2.74870900000000	-0.13367900000000
O	-1.61898600000000	0.98945100000000	-1.98607800000000
O	-5.10707200000000	2.80678500000000	-0.03155100000000
Si	-3.74312000000000	-4.30433900000000	-6.95931300000000
Si	-1.82358900000000	1.28264200000000	-0.41265200000000
Si	-3.78269700000000	3.66738300000000	-0.36763200000000
O	-5.01077000000000	1.90940100000000	-3.27488000000000
O	2.84162800000000	-5.06061700000000	-1.96338400000000
O	-5.56536200000000	-2.52760800000000	2.85063500000000
O	-7.35463000000000	-1.79305400000000	4.68413400000000
O	-0.22655700000000	-6.30984900000000	-3.38356800000000
O	-7.98417400000000	0.19852900000000	3.03500700000000
O	-0.36400800000000	-5.58248000000000	-5.94481900000000
O	-0.93776400000000	-1.93718400000000	-3.00072400000000
O	-3.45850700000000	-6.51805800000000	0.75482100000000
O	-0.95306500000000	-4.27635200000000	-1.84166200000000
O	-6.58837800000000	1.02532100000000	-1.36304800000000
O	0.65105100000000	-6.08807800000000	-0.85338900000000
O	-8.32228400000000	-0.63394600000000	0.51761300000000
O	-7.35786100000000	1.79623900000000	1.03740400000000
O	-1.84798300000000	-6.75753100000000	-1.38576200000000
O	-6.51770800000000	-0.22019600000000	-3.68630800000000
O	2.73226800000000	-5.42819400000000	0.66001300000000
O	-9.03821800000000	-2.66280100000000	-1.01631000000000
O	-4.44427500000000	-0.37976700000000	-2.06386700000000
O	-6.98121400000000	-2.91717800000000	0.63539800000000
O	-8.00644200000000	-2.40068800000000	-3.46293900000000
O	-5.34946900000000	-2.61465700000000	-3.72468100000000
O	-7.24110800000000	-4.38128800000000	-1.85822400000000
O	-4.98363100000000	-5.15819100000000	-3.05663600000000
O	-2.86684300000000	-3.64980200000000	-3.60913300000000
O	-5.66437100000000	-6.16906900000000	-0.70181100000000
O	-2.77944300000000	-2.53103900000000	-1.18803900000000
O	-4.73736000000000	-4.22954500000000	0.93471400000000
O	-4.61544800000000	-1.56772500000000	0.70180400000000
Si	-6.45160200000000	-3.02371700000000	4.12466700000000
Si	0.62610200000000	-5.84019800000000	-4.68798500000000
Si	-8.47205900000000	-0.65877600000000	4.33065000000000
Si	-1.21853700000000	-6.07821000000000	-7.24051200000000
Si	-6.61907100000000	2.28370900000000	-0.33331100000000

Si	-2.41186500000000	-7.49807400000000	-0.03942500000000
Si	-0.59608700000000	-5.84673600000000	-1.87138000000000
Si	-8.40039000000000	0.64974200000000	1.51860000000000
Si	-5.61788700000000	0.56449200000000	-2.58409100000000
Si	2.27447900000000	-6.02873600000000	-0.79155300000000
Si	-8.45739100000000	-2.24613300000000	0.44116500000000
Si	-6.67721700000000	-1.76369600000000	-4.15621400000000
Si	-8.49296400000000	-3.45598500000000	-2.33301900000000
Si	-4.42274500000000	-3.92973000000000	-3.96398200000000
Si	-6.23432900000000	-5.62877000000000	-2.12665000000000
Si	-1.91046100000000	-3.09500900000000	-2.40052900000000
Si	-4.93228000000000	-5.84147200000000	0.71680000000000
Al	-3.67444800000000	-1.14817300000000	-0.76283200000000
Si	-5.48960800000000	-2.83228200000000	1.25456700000000
Si	-2.08817500000000	-1.55853400000000	-6.89307200000000
H	11.69917500000000	-5.54591300000000	-0.01904600000000
H	9.21891100000000	-8.11473500000000	2.38249100000000
H	-3.85612200000000	1.39093400000000	-8.23823000000000
H	-5.52119100000000	-0.08872800000000	-7.35293500000000
H	-6.90663400000000	-1.74222800000000	-5.58763900000000
H	-9.58540700000000	-4.24259300000000	-2.89625100000000
H	-4.78263400000000	-4.39504100000000	-7.96564000000000
H	-1.23015300000000	-7.53714800000000	-7.33581000000000
H	-8.44060700000000	2.52331100000000	6.79066300000000
H	-4.18407600000000	6.49631100000000	2.51400100000000
H	-1.78600400000000	8.99704200000000	0.22067500000000
H	0.70155600000000	11.31895500000000	-2.27862700000000
H	-4.92794200000000	5.92269500000000	-3.32695700000000
H	-7.40670000000000	3.35579600000000	-0.92422700000000
H	-9.78890700000000	1.13212500000000	1.46933500000000
H	-3.23539500000000	2.49785200000000	9.45229700000000
H	-0.71961300000000	4.84607400000000	6.92466100000000
H	1.19719300000000	6.83719000000000	4.62541100000000
H	0.03926500000000	-0.86156600000000	9.77223400000000
H	2.68173200000000	1.60491000000000	7.11731600000000
H	4.55619700000000	3.46492100000000	4.88827200000000
H	1.56263600000000	-6.94686000000000	-4.95462900000000
H	4.66084300000000	9.20647900000000	-0.62722400000000
H	-8.64946000000000	0.52686900000000	8.10789300000000
H	-5.65332300000000	-0.36731900000000	8.94594600000000
H	-5.37973300000000	-3.46714300000000	7.76499800000000
H	-2.83464500000000	-3.04874400000000	9.11986000000000
H	-1.20158200000000	-4.12050400000000	7.72303700000000
H	2.59956100000000	5.41920200000000	-6.14460400000000
H	0.25025700000000	8.09388200000000	-5.83700400000000
H	2.82050400000000	11.24801600000000	-3.40289800000000
H	6.08609900000000	7.26029800000000	-3.74788300000000
H	10.03607800000000	4.22891700000000	-2.74210300000000
H	9.90469300000000	0.41483700000000	-3.57558100000000
H	11.19182300000000	0.77856400000000	-1.58569700000000
H	9.92390000000000	3.89453500000000	-0.36785600000000
H	-1.29857100000000	-1.20482100000000	-8.08427300000000
H	-0.59120100000000	-5.50702100000000	-8.44108800000000
H	2.87361100000000	-3.05219900000000	-5.93135600000000
H	-7.28975100000000	-4.16700500000000	3.76963500000000
H	-9.75707200000000	-1.31803500000000	4.09899800000000
H	-9.37776900000000	-2.76270900000000	1.46916500000000
H	-5.74356100000000	-6.41909000000000	1.79888900000000

H	-3.05541700000000	-8.75970700000000	-0.44894300000000
H	-6.99637800000000	-6.70175500000000	-2.76517000000000
H	2.80901800000000	-7.37774000000000	-0.97344500000000
H	4.92234400000000	-9.58625700000000	2.51312200000000
H	5.84627300000000	-9.05601400000000	0.36277300000000
H	-1.31894900000000	-7.78493200000000	0.89370200000000
H	10.69493700000000	-2.02917400000000	2.58283500000000
H	8.84856900000000	-3.96461800000000	4.80290500000000
H	11.61622800000000	-3.98341100000000	-1.83985200000000
H	5.36358400000000	-0.47338800000000	4.53898300000000
H	7.17762300000000	1.33117000000000	2.39137500000000
H	7.34794000000000	6.56953400000000	-0.49427900000000
H	5.03227100000000	-4.87372500000000	-3.22483000000000
H	7.92757600000000	-7.01002000000000	-1.48756400000000
H	2.46801100000000	-6.50862400000000	2.93046000000000
Ga	-3.50721600000000	-0.39109000000000	2.00097100000000
C	-2.17682300000000	-1.44027000000000	2.98668900000000
H	-2.69659100000000	-1.99262700000000	3.77824700000000
H	-4.88798400000000	1.06008700000000	3.25922300000000
H	-1.51335200000000	-0.72415600000000	3.48837100000000
C	-1.37196000000000	-2.38699800000000	2.08724800000000
H	-0.62861800000000	-2.93693300000000	2.67559100000000
H	-0.83163100000000	-1.84254600000000	1.30618500000000
H	-2.01148800000000	-3.12344500000000	1.58927400000000
O	-4.57494800000000	0.99994500000000	2.35092300000000

TS2-GaO-I

Electronic energy (r²SCAN-3c): -30692.535055220942 E_h
Electronic energy (ωB97X-D4rev/def2-mTZVPP): -30700.753645586494 E_h
G_corr (673.15 K, 1 atm): 0.42384214 E_h
Number of atoms: 249

Cartesian coordinates (Å)

O	1.57704300000000	2.85584100000000	1.01776700000000
O	-1.91329100000000	6.77793600000000	1.46079500000000
O	5.29468800000000	2.07253800000000	0.81937900000000
O	3.65894200000000	3.89810700000000	-0.15173700000000
O	6.13914000000000	4.56683400000000	0.44120200000000
O	1.49967900000000	3.29041900000000	-1.60763700000000
O	1.39908600000000	5.30697300000000	0.07161600000000
O	-1.67961600000000	6.87840700000000	-1.16148500000000
O	0.35649500000000	7.69070500000000	0.43037800000000
O	2.96231500000000	7.45793800000000	-0.04553400000000
Si	4.88583200000000	3.63688700000000	0.88379200000000
Si	2.04197100000000	3.84195800000000	-0.18471500000000
Si	-1.26303900000000	7.62367100000000	0.22794200000000
Si	1.68275200000000	6.78412800000000	0.67806800000000
O	8.97947500000000	-3.84502500000000	2.24549400000000
O	2.04630400000000	6.65972200000000	2.26105400000000
O	7.16747900000000	-2.28660400000000	-0.90622400000000
O	2.53398900000000	4.74632600000000	4.09423100000000
O	6.77411300000000	-4.91125600000000	-0.76229400000000
O	0.07083100000000	5.16059400000000	3.16745300000000
O	5.88089300000000	-3.28000600000000	1.15249900000000
O	4.47337400000000	4.06583700000000	2.40194000000000
O	9.42704700000000	-5.02233900000000	-1.10555500000000

O	5.17885600000000	-0.25146500000000	2.03607300000000
O	7.76110100000000	4.37115100000000	-1.67721200000000
O	10.72871400000000	-3.19341600000000	0.36450700000000
O	5.45613500000000	5.62770600000000	-1.88776100000000
O	4.10274700000000	5.64850100000000	-4.13884600000000
O	8.78467600000000	-1.47223100000000	1.06374200000000
O	3.95248800000000	7.73482000000000	-2.52058100000000
O	1.19943400000000	9.51027300000000	-4.02811100000000
O	9.00606300000000	2.06225600000000	-1.86649800000000
O	6.97469100000000	0.18222200000000	0.12097300000000
O	2.29873300000000	9.54500300000000	-1.54563000000000
O	9.02686800000000	-0.55737600000000	-1.42298000000000
Si	1.47465800000000	5.87264900000000	3.56801800000000
Si	6.13942200000000	-3.44935100000000	-0.43503400000000
Si	3.66246800000000	3.62240300000000	3.74216900000000
Si	8.10234300000000	-5.84512700000000	-0.63778200000000
Si	10.90220500000000	-4.46801000000000	-0.65268500000000
Si	6.70203300000000	5.31922400000000	-0.89236200000000
Si	9.82043800000000	-2.61817400000000	1.58547600000000
Si	4.94682200000000	6.58217600000000	-3.10171800000000
Si	9.23151500000000	3.66384900000000	-1.64944400000000
Si	6.19915100000000	0.84699500000000	1.38917100000000
Si	1.75489100000000	10.44398400000000	-2.80730600000000
Si	9.82623700000000	0.67385200000000	-2.13454900000000
Si	7.98090400000000	-1.02629500000000	-0.28444900000000
Si	3.49694100000000	8.50949200000000	-1.16653400000000
O	3.00489200000000	-0.47615200000000	3.63055600000000
O	0.97959200000000	0.77337100000000	2.51231900000000
O	1.27130300000000	0.87108800000000	5.10539000000000
O	2.99314600000000	2.16161900000000	3.50393600000000
O	-0.38086900000000	1.17108500000000	0.33343500000000
O	-0.73888900000000	2.79723400000000	2.34012700000000
O	-3.68956000000000	4.97851000000000	0.58190200000000
O	-2.50493500000000	4.66145400000000	2.95817600000000
O	-1.12460800000000	3.42287500000000	4.84357700000000
Si	2.07959800000000	0.83988300000000	3.70495400000000
Si	0.36870700000000	1.92233700000000	1.54510600000000
Si	-3.10114100000000	5.74528800000000	1.88605300000000
Si	-1.06432500000000	4.02483400000000	3.34348900000000
O	8.31285700000000	-6.05309800000000	3.51715900000000
O	-3.17718800000000	-3.05974800000000	6.59443700000000
O	6.70824200000000	-7.86167200000000	2.46784200000000
O	-1.28640300000000	-1.57554600000000	7.70491300000000
O	8.26709800000000	-6.34520000000000	0.89469400000000
O	-5.39604300000000	-3.47494700000000	5.24993800000000
O	6.67779200000000	-3.93195100000000	3.58969300000000
O	-5.10941900000000	-1.22010800000000	6.60835900000000
O	-8.64149600000000	0.27120900000000	5.64467700000000
O	4.50157500000000	-2.51996400000000	3.26502000000000
O	-6.41668600000000	1.05667800000000	6.98016600000000
O	4.51433600000000	-5.07255100000000	2.62675000000000
O	-3.85682000000000	0.84876400000000	7.59173100000000
O	-2.22908900000000	2.83548000000000	7.14713000000000
O	0.42359900000000	2.64724600000000	6.88362500000000
O	4.28392000000000	-7.40676400000000	1.39504000000000
O	-1.48131500000000	0.86133000000000	8.79813700000000
O	0.74675100000000	0.07093100000000	7.55079900000000
Si	8.15004900000000	-7.13373300000000	2.30722500000000

Si	-2.10702900000000	-2.98210700000000	7.83478600000000
Si	8.22586600000000	-4.42677800000000	3.57146700000000
Si	-4.79505000000000	-2.81850700000000	6.59693600000000
Si	4.55697900000000	-0.90388100000000	3.39342600000000
Si	-8.05780400000000	1.11583000000000	6.90394800000000
Si	5.38716600000000	-3.71538500000000	2.63050700000000
Si	-5.27856400000000	0.06675600000000	7.58797500000000
Si	-0.90753000000000	3.47952400000000	6.45394600000000
Si	3.46859000000000	-6.10895700000000	1.94329700000000
Si	-2.72393200000000	1.78051800000000	8.28381700000000
Si	1.32224500000000	1.30764900000000	6.67151500000000
Si	5.44301500000000	-8.51881000000000	1.66846200000000
Si	-0.49222100000000	-0.39559700000000	8.49360900000000
O	1.79157000000000	4.25351200000000	-4.06228300000000
O	-0.04693700000000	5.25701100000000	-2.43719200000000
O	-0.58736500000000	7.67530300000000	-3.43579000000000
Si	0.71224400000000	3.90146300000000	-2.89832200000000
Si	-1.17837800000000	6.39918200000000	-2.62798300000000
O	1.28614100000000	-1.71860400000000	-4.50912800000000
O	-3.93925200000000	3.94398300000000	-4.53391400000000
O	3.36364500000000	-2.96342300000000	-3.46233200000000
O	-2.42718600000000	5.82157600000000	-3.47618700000000
O	-3.77708800000000	4.14308000000000	-1.89902700000000
O	1.35676800000000	-4.40943200000000	-4.39870300000000
O	-4.62697900000000	1.77802400000000	-5.90921300000000
O	4.74307800000000	-3.31510400000000	-1.24036900000000
O	-2.58102400000000	1.67579400000000	-4.27948400000000
O	-3.13995400000000	-0.37045200000000	-6.51511900000000
O	-1.08675000000000	-1.80877600000000	-5.64287100000000
O	-0.33282100000000	0.31467300000000	-4.18329700000000
O	-0.37246900000000	2.82824800000000	-3.42639500000000
O	1.80366900000000	6.91386500000000	-4.25056700000000
Si	-3.81265400000000	4.99012800000000	-3.29166400000000
Si	2.21814000000000	-3.04462500000000	-4.62465300000000
Si	-4.05308800000000	2.32087200000000	-4.50101200000000
Si	4.03080100000000	-4.08437500000000	-2.49147900000000
Si	-4.29726200000000	0.65932200000000	-7.05205000000000
Si	-0.27252300000000	-1.30522300000000	-4.33448400000000
Si	-1.22323200000000	1.44501900000000	-3.44261500000000
Si	2.56876900000000	5.54850200000000	-4.69113300000000
Si	0.65030900000000	8.04130100000000	-4.43390700000000
O	-2.77006600000000	-5.59750100000000	-7.09553900000000
O	-4.48792000000000	-4.34099700000000	-5.51062900000000
O	-2.89664000000000	-2.93304400000000	-7.20492400000000
O	-2.78349500000000	0.07174100000000	0.28432600000000
O	-2.48587300000000	2.71690500000000	-0.10684300000000
O	-1.61413800000000	0.95022900000000	-1.93926300000000
O	-5.12068700000000	2.82886600000000	0.04053800000000
Si	-3.74268000000000	-4.30201800000000	-6.95474200000000
Si	-1.83270700000000	1.25721200000000	-0.37409100000000
Si	-3.79369000000000	3.66317900000000	-0.35648000000000
O	-5.08850200000000	1.94933600000000	-3.31082300000000
O	2.84020000000000	-5.06100700000000	-1.96451200000000
O	-5.50759700000000	-2.61947300000000	2.79415000000000
O	-7.31665100000000	-1.77542700000000	4.60074000000000
O	-0.22829000000000	-6.29420200000000	-3.38036300000000
O	-8.04408200000000	0.29045300000000	3.08204400000000
O	-0.36170600000000	-5.58275500000000	-5.94584300000000

O	-0.88494600000000	-1.97033500000000	-3.00165000000000
O	-3.46451300000000	-6.53928200000000	0.75987700000000
O	-0.96265900000000	-4.28065100000000	-1.80313900000000
O	-6.54987100000000	1.02386600000000	-1.33280800000000
O	0.65221300000000	-6.09524300000000	-0.85111100000000
O	-8.24909000000000	-0.66138000000000	0.57763200000000
O	-7.38466600000000	1.82298900000000	1.04557100000000
O	-1.84599200000000	-6.76916300000000	-1.38702600000000
O	-6.51005800000000	-0.24199000000000	-3.63868100000000
O	2.73476000000000	-5.42774500000000	0.65864000000000
O	-9.01628400000000	-2.62176900000000	-1.03069700000000
O	-4.33751100000000	-0.26243900000000	-2.08540100000000
O	-7.06573500000000	-3.06991800000000	0.71279000000000
O	-7.99854600000000	-2.43282400000000	-3.48621500000000
O	-5.33172100000000	-2.60507200000000	-3.71594300000000
O	-7.24314200000000	-4.38950800000000	-1.84607000000000
O	-4.98356700000000	-5.15422400000000	-3.04508100000000
O	-2.86261000000000	-3.63870300000000	-3.57018700000000
O	-5.67001200000000	-6.21942000000000	-0.71139100000000
O	-2.72060700000000	-2.46016400000000	-1.16381300000000
O	-4.72705700000000	-4.24553500000000	0.84415300000000
O	-4.85183500000000	-1.59050000000000	0.48084500000000
Si	-6.42503300000000	-3.02744500000000	4.06726100000000
Si	0.62639400000000	-5.83487200000000	-4.68613300000000
Si	-8.46795400000000	-0.65750000000000	4.32795100000000
Si	-1.21821200000000	-6.07785900000000	-7.24047700000000
Si	-6.62513900000000	2.28598100000000	-0.31545800000000
Si	-2.41341700000000	-7.50369700000000	-0.03798200000000
Si	-0.60043600000000	-5.84731200000000	-1.86311500000000
Si	-8.39950300000000	0.65009700000000	1.53499200000000
Si	-5.59785800000000	0.58334800000000	-2.57685700000000
Si	2.27350400000000	-6.03013300000000	-0.79272900000000
Si	-8.45440000000000	-2.26817300000000	0.45581800000000
Si	-6.66960800000000	-1.77122800000000	-4.15094500000000
Si	-8.48900400000000	-3.45785000000000	-2.32822100000000
Si	-4.41167500000000	-3.92719400000000	-3.94514300000000
Si	-6.23612100000000	-5.63711700000000	-2.11981300000000
Si	-1.89286300000000	-3.08112400000000	-2.37225500000000
Si	-4.93688700000000	-5.85549300000000	0.70148800000000
Al	-3.72172000000000	-1.14309600000000	-0.75623300000000
Si	-5.52379500000000	-2.86839400000000	1.18017300000000
Si	-2.08841000000000	-1.55800600000000	-6.89295800000000
H	11.69917500000000	-5.54591300000000	-0.01904600000000
H	9.21891100000000	-8.11473500000000	2.38249100000000
H	-3.85612200000000	1.39093400000000	-8.23823000000000
H	-5.52119100000000	-0.08872800000000	-7.35293500000000
H	-6.90663400000000	-1.74222800000000	-5.58763900000000
H	-9.58540700000000	-4.24259300000000	-2.89625100000000
H	-4.78263400000000	-4.39504100000000	-7.96564000000000
H	-1.23015300000000	-7.53714800000000	-7.33581000000000
H	-8.44060700000000	2.52331100000000	6.79066300000000
H	-4.18407600000000	6.49631100000000	2.51400100000000
H	-1.78600400000000	8.99704200000000	0.22067500000000
H	0.70155600000000	11.31895500000000	-2.27862700000000
H	-4.92794200000000	5.92269500000000	-3.32695700000000
H	-7.40670000000000	3.35579600000000	-0.92422700000000
H	-9.78890700000000	1.13212500000000	1.46933500000000
H	-3.23539500000000	2.49785200000000	9.45229700000000

H	-0.71961300000000	4.84607400000000	6.92466100000000
H	1.19719300000000	6.83719000000000	4.62541100000000
H	0.03926500000000	-0.86156600000000	9.77223400000000
H	2.68173200000000	1.60491000000000	7.11731600000000
H	4.55619700000000	3.46492100000000	4.88827200000000
H	1.56263600000000	-6.94686000000000	-4.95462900000000
H	4.66084300000000	9.20647900000000	-0.62722400000000
H	-8.64946000000000	0.52686900000000	8.10789300000000
H	-5.65332300000000	-0.36731900000000	8.94594600000000
H	-5.37973300000000	-3.46714300000000	7.76499800000000
H	-2.83464500000000	-3.04874400000000	9.11986000000000
H	-1.20158200000000	-4.12050400000000	7.72303700000000
H	2.59956100000000	5.41920200000000	-6.14460400000000
H	0.25025700000000	8.09388200000000	-5.83700400000000
H	2.82050400000000	11.24801600000000	-3.40289800000000
H	6.08609900000000	7.26029800000000	-3.74788300000000
H	10.03607800000000	4.22891700000000	-2.74210300000000
H	9.90469300000000	0.41483700000000	-3.57558100000000
H	11.19182300000000	0.77856400000000	-1.58569700000000
H	9.92390000000000	3.89453500000000	-0.36785600000000
H	-1.29857100000000	-1.20482100000000	-8.08427300000000
H	-0.59120100000000	-5.50702100000000	-8.44108800000000
H	2.87361100000000	-3.05219900000000	-5.93135600000000
H	-7.28975100000000	-4.16700500000000	3.76963500000000
H	-9.75707200000000	-1.31803500000000	4.09899800000000
H	-9.37776900000000	-2.76270900000000	1.46916500000000
H	-5.74356100000000	-6.41909000000000	1.79888900000000
H	-3.05541700000000	-8.75970700000000	-0.44894300000000
H	-6.99637800000000	-6.70175500000000	-2.76517000000000
H	2.80901800000000	-7.37774000000000	-0.97344500000000
H	4.92234400000000	-9.58625700000000	2.51312200000000
H	5.84627300000000	-9.05601400000000	0.36277300000000
H	-1.31894900000000	-7.78493200000000	0.89370200000000
H	10.69493700000000	-2.02917400000000	2.58283500000000
H	8.84856900000000	-3.96461800000000	4.80290500000000
H	11.61622800000000	-3.98341100000000	-1.83985200000000
H	5.36358400000000	-0.47338800000000	4.53898300000000
H	7.17762300000000	1.33117000000000	2.39137500000000
H	7.34794000000000	6.56953400000000	-0.49427900000000
H	5.03227100000000	-4.87372500000000	-3.22483000000000
H	7.92757600000000	-7.01002000000000	-1.48756400000000
H	2.46801100000000	-6.50862400000000	2.93046000000000
Ga	-3.44672000000000	-0.27456600000000	2.08823800000000
C	-2.17422200000000	0.18804100000000	3.72321000000000
H	-2.81726400000000	0.51682200000000	4.53666500000000
H	-5.01080100000000	1.21779500000000	2.97566000000000
H	-1.34248800000000	0.83069200000000	3.47448800000000
C	-1.99590700000000	-1.21000300000000	3.54987700000000
H	-2.48554000000000	-1.92837900000000	4.20437100000000
H	-1.10588400000000	-1.57566900000000	3.04087100000000
H	-2.96046200000000	-1.84307100000000	2.29438800000000
O	-5.05800100000000	0.43917900000000	2.41031600000000

I3-GaO-I

Electronic energy (r^2SCAN-3c): -30692.587127227613 E_h
Electronic energy (ωB97X-D4rev/def2-mTZVPP): -30700.813916420731 E_h
G_{corr} (673.15 K, 1 atm): 0.41539689 E_h
Number of atoms: 249

Cartesian coordinates (Å)

O	1.52003900000000	2.88325100000000	1.04488800000000
O	-1.91969000000000	6.76252400000000	1.44630700000000
O	5.29289100000000	2.07016300000000	0.82206800000000
O	3.63333100000000	3.87387000000000	-0.13858400000000
O	6.12002400000000	4.56709000000000	0.43755100000000
O	1.47489700000000	3.28074500000000	-1.59514100000000
O	1.39548600000000	5.31686300000000	0.05115800000000
O	-1.68893800000000	6.88158600000000	-1.17390500000000
O	0.34808500000000	7.69140100000000	0.42369000000000
O	2.95770000000000	7.46893700000000	-0.02888600000000
Si	4.87186800000000	3.63162000000000	0.88730700000000
Si	2.01455700000000	3.83650300000000	-0.17010800000000
Si	-1.27125400000000	7.61923000000000	0.22127800000000
Si	1.67311500000000	6.78466400000000	0.67912700000000
O	8.97645000000000	-3.84277900000000	2.24425800000000
O	2.02253300000000	6.64415400000000	2.26265400000000
O	7.17032700000000	-2.28934400000000	-0.90257100000000
O	2.53079700000000	4.74031400000000	4.10197900000000
O	6.77367600000000	-4.91350800000000	-0.75065900000000
O	0.07150200000000	5.10823400000000	3.16549000000000
O	5.87575700000000	-3.27534700000000	1.15609900000000
O	4.46731900000000	4.06520600000000	2.40505400000000
O	9.42633600000000	-5.01996700000000	-1.10897700000000
O	5.17968700000000	-0.25636500000000	2.03748800000000
O	7.75964900000000	4.36936700000000	-1.67025500000000
O	10.72846100000000	-3.19408200000000	0.36500000000000
O	5.45573400000000	5.62297000000000	-1.89220300000000
O	4.11454200000000	5.66113800000000	-4.15305600000000
O	8.78759100000000	-1.46882900000000	1.06362900000000
O	3.94793700000000	7.72762500000000	-2.51109800000000
O	1.20223800000000	9.51358100000000	-4.03114800000000
O	9.00643000000000	2.06279000000000	-1.86745100000000
O	6.97278300000000	0.18118400000000	0.12193300000000
O	2.29806200000000	9.54467900000000	-1.54568100000000
O	9.02487600000000	-0.55676900000000	-1.42410100000000
Si	1.45778600000000	5.85283000000000	3.57212000000000
Si	6.14033300000000	-3.44920300000000	-0.42944100000000
Si	3.65707500000000	3.61585100000000	3.74516800000000
Si	8.10442200000000	-5.84530700000000	-0.63723500000000
Si	10.90167600000000	-4.46784100000000	-0.65300200000000
Si	6.69695200000000	5.31786200000000	-0.88980800000000
Si	9.82017800000000	-2.61709600000000	1.58535400000000
Si	4.94849700000000	6.58484000000000	-3.10026500000000
Si	9.23071700000000	3.66436400000000	-1.64854500000000
Si	6.19707700000000	0.84540500000000	1.39161500000000
Si	1.75553900000000	10.44422100000000	-2.80679100000000
Si	9.82582000000000	0.67410400000000	-2.13452100000000
Si	7.98064500000000	-1.02510100000000	-0.28337400000000
Si	3.49406700000000	8.50872200000000	-1.15937500000000
O	3.00296000000000	-0.48027800000000	3.63728700000000

O	0.98752000000000	0.75670100000000	2.50058100000000
O	1.26737500000000	0.88664600000000	5.10251800000000
O	2.99927600000000	2.15134600000000	3.49964900000000
O	-0.38008400000000	1.17144000000000	0.31188300000000
O	-0.79109600000000	2.72676100000000	2.37724800000000
O	-3.71842100000000	4.96782300000000	0.59088400000000
O	-2.50808800000000	4.65590400000000	2.95679300000000
O	-1.14836300000000	3.41837800000000	4.86019200000000
Si	2.07298500000000	0.83518800000000	3.70305800000000
Si	0.33784200000000	1.90199900000000	1.55956500000000
Si	-3.10802800000000	5.73416000000000	1.88714400000000
Si	-1.08467000000000	3.98794500000000	3.35068200000000
O	8.31292200000000	-6.05153100000000	3.51518700000000
O	-3.17205400000000	-3.07947700000000	6.59928000000000
O	6.70783100000000	-7.85724600000000	2.46390100000000
O	-1.29550800000000	-1.57035600000000	7.71457900000000
O	8.28311400000000	-6.35184600000000	0.89118300000000
O	-5.40381000000000	-3.49346600000000	5.26812500000000
O	6.67685000000000	-3.93189600000000	3.59099700000000
O	-5.10267000000000	-1.22746400000000	6.60766800000000
O	-8.65087500000000	0.32809600000000	5.60074700000000
O	4.49953000000000	-2.52043000000000	3.27302600000000
O	-6.42084600000000	1.03490500000000	6.96336400000000
O	4.51328900000000	-5.07148100000000	2.62968000000000
O	-3.86267500000000	0.85422900000000	7.59683100000000
O	-2.22990400000000	2.84241500000000	7.16505500000000
O	0.42360600000000	2.64998600000000	6.89054100000000
O	4.28272900000000	-7.40488600000000	1.39546500000000
O	-1.48371000000000	0.85888200000000	8.80579000000000
O	0.74588000000000	0.07562100000000	7.55306300000000
Si	8.15184300000000	-7.13307500000000	2.30625500000000
Si	-2.10046200000000	-2.98135900000000	7.83678200000000
Si	8.22494500000000	-4.42525700000000	3.57125400000000
Si	-4.77919900000000	-2.82425600000000	6.60587000000000
Si	4.55366800000000	-0.90195100000000	3.39674800000000
Si	-8.05847700000000	1.11871700000000	6.89819700000000
Si	5.38452100000000	-3.71351700000000	2.63434200000000
Si	-5.27353700000000	0.06085100000000	7.58738700000000
Si	-0.91012500000000	3.47891500000000	6.46319400000000
Si	3.46677900000000	-6.10645000000000	1.94565700000000
Si	-2.72267500000000	1.77922800000000	8.28998200000000
Si	1.32132600000000	1.30970100000000	6.67082600000000
Si	5.44174000000000	-8.51689500000000	1.66794300000000
Si	-0.49067300000000	-0.39476900000000	8.49428300000000
O	1.81872700000000	4.26027700000000	-4.04202100000000
O	-0.05001200000000	5.26029500000000	-2.44722000000000
O	-0.58535400000000	7.68101700000000	-3.43873200000000
Si	0.72080700000000	3.90819000000000	-2.89678100000000
Si	-1.18083400000000	6.40275600000000	-2.63799100000000
O	1.27366600000000	-1.73814000000000	-4.48039300000000
O	-3.95263900000000	3.93882400000000	-4.53161100000000
O	3.36778500000000	-2.96777100000000	-3.46423800000000
O	-2.42421100000000	5.82746900000000	-3.49952800000000
O	-3.75866100000000	4.16482400000000	-1.90280900000000
O	1.35930200000000	-4.41911000000000	-4.39674100000000
O	-4.64070500000000	1.76703700000000	-5.89872500000000
O	4.74601100000000	-3.31589600000000	-1.23942800000000
O	-2.54856600000000	1.68960400000000	-4.33659300000000

O	-3.14343300000000	-0.37439700000000	-6.51548300000000
O	-1.08312900000000	-1.80839100000000	-5.64507200000000
O	-0.31273700000000	0.31819900000000	-4.19712400000000
O	-0.35148900000000	2.84109900000000	-3.46290600000000
O	1.80686800000000	6.91753100000000	-4.25324500000000
Si	-3.80826400000000	4.99686400000000	-3.29880300000000
Si	2.21831500000000	-3.05240700000000	-4.62057100000000
Si	-4.03523500000000	2.31501900000000	-4.50470600000000
Si	4.03402600000000	-4.08559000000000	-2.48885100000000
Si	-4.29930500000000	0.65908000000000	-7.05027400000000
Si	-0.27966100000000	-1.30254300000000	-4.33072200000000
Si	-1.20266200000000	1.46258800000000	-3.47725500000000
Si	2.57739000000000	5.55470300000000	-4.69122800000000
Si	0.65318200000000	8.04447600000000	-4.43555800000000
O	-2.77147200000000	-5.60029400000000	-7.09174400000000
O	-4.51061300000000	-4.34313200000000	-5.52589300000000
O	-2.89276100000000	-2.93707400000000	-7.20043500000000
O	-2.78532700000000	0.08795600000000	0.19687300000000
O	-2.46179800000000	2.74057300000000	-0.13112100000000
O	-1.62487200000000	0.98000000000000	-1.98084000000000
O	-5.10856200000000	2.80324300000000	-0.02628400000000
Si	-3.74314800000000	-4.30397700000000	-6.95913800000000
Si	-1.82267000000000	1.27858800000000	-0.40794400000000
Si	-3.78510100000000	3.66514100000000	-0.36547300000000
O	-5.01690100000000	1.91213000000000	-3.27718000000000
O	2.84147500000000	-5.06142000000000	-1.96480700000000
O	-5.56881800000000	-2.52395200000000	2.84614800000000
O	-7.35451900000000	-1.79331400000000	4.68389100000000
O	-0.22590200000000	-6.31435600000000	-3.38487300000000
O	-7.98463800000000	0.19519500000000	3.03351000000000
O	-0.36397800000000	-5.58230000000000	-5.94488200000000
O	-0.94005600000000	-1.93673500000000	-3.00007800000000
O	-3.46060600000000	-6.51920400000000	0.75166900000000
O	-0.94955500000000	-4.27987300000000	-1.84822600000000
O	-6.58796700000000	1.02418700000000	-1.36225000000000
O	0.65242000000000	-6.09095600000000	-0.85463900000000
O	-8.32540900000000	-0.63310400000000	0.51486300000000
O	-7.35583400000000	1.79272300000000	1.03840300000000
O	-1.84771400000000	-6.75890600000000	-1.38680200000000
O	-6.51961100000000	-0.22085000000000	-3.68581500000000
O	2.73617100000000	-5.42656900000000	0.65808400000000
O	-9.04226300000000	-2.66241900000000	-1.01823100000000
O	-4.44189800000000	-0.37482400000000	-2.06768100000000
O	-6.98472500000000	-2.91559000000000	0.62942100000000
O	-8.00873400000000	-2.39982100000000	-3.46363400000000
O	-5.35136900000000	-2.61448900000000	-3.72194600000000
O	-7.24144600000000	-4.37976600000000	-1.85919800000000
O	-4.98450600000000	-5.15812900000000	-3.05625600000000
O	-2.86941200000000	-3.64732500000000	-3.60662300000000
O	-5.66822700000000	-6.16977200000000	-0.70156700000000
O	-2.77354400000000	-2.53520800000000	-1.18229500000000
O	-4.74152400000000	-4.22815900000000	0.92830900000000
O	-4.62545800000000	-1.56263200000000	0.69456400000000
Si	-6.45088400000000	-3.02339400000000	4.12463500000000
Si	0.62617600000000	-5.84094500000000	-4.68800700000000
Si	-8.47207400000000	-0.65925000000000	4.33152800000000
Si	-1.21846400000000	-6.07819100000000	-7.24026800000000
Si	-6.62071200000000	2.28294300000000	-0.33280400000000

Si	-2.41133700000000	-7.49858700000000	-0.03965800000000
Si	-0.59410800000000	-5.85075000000000	-1.87236600000000
Si	-8.40169600000000	0.64846700000000	1.51905100000000
Si	-5.62009300000000	0.56641300000000	-2.58630200000000
Si	2.27570100000000	-6.02875900000000	-0.79112600000000
Si	-8.46172000000000	-2.24500400000000	0.43866800000000
Si	-6.67872900000000	-1.76441200000000	-4.15621400000000
Si	-8.49408100000000	-3.45581900000000	-2.33381700000000
Si	-4.42407600000000	-3.92947200000000	-3.96327100000000
Si	-6.23555100000000	-5.62871000000000	-2.12654500000000
Si	-1.90782500000000	-3.09829000000000	-2.39980700000000
Si	-4.93287000000000	-5.84235900000000	0.71558000000000
Al	-3.67396500000000	-1.15377900000000	-0.77450100000000
Si	-5.49599000000000	-2.83583800000000	1.25211400000000
Si	-2.08793500000000	-1.55848500000000	-6.89294200000000
H	11.69917500000000	-5.54591300000000	-0.01904600000000
H	9.21891100000000	-8.11473500000000	2.38249100000000
H	-3.85612200000000	1.39093400000000	-8.23823000000000
H	-5.52119100000000	-0.08872800000000	-7.35293500000000
H	-6.90663400000000	-1.74222800000000	-5.58763900000000
H	-9.58540700000000	-4.24259300000000	-2.89625100000000
H	-4.78263400000000	-4.39504100000000	-7.96564000000000
H	-1.23015300000000	-7.53714800000000	-7.33581000000000
H	-8.44060700000000	2.52331100000000	6.79066300000000
H	-4.18407600000000	6.49631100000000	2.51400100000000
H	-1.78600400000000	8.99704200000000	0.22067500000000
H	0.70155600000000	11.31895500000000	-2.27862700000000
H	-4.92794200000000	5.92269500000000	-3.32695700000000
H	-7.40670000000000	3.35579600000000	-0.92422700000000
H	-9.78890700000000	1.13212500000000	1.46933500000000
H	-3.23539500000000	2.49785200000000	9.45229700000000
H	-0.71961300000000	4.84607400000000	6.92466100000000
H	1.19719300000000	6.83719000000000	4.62541100000000
H	0.03926500000000	-0.86156600000000	9.77223400000000
H	2.68173200000000	1.60491000000000	7.11731600000000
H	4.55619700000000	3.46492100000000	4.88827200000000
H	1.56263600000000	-6.94686000000000	-4.95462900000000
H	4.66084300000000	9.20647900000000	-0.62722400000000
H	-8.64946000000000	0.52686900000000	8.10789300000000
H	-5.65332300000000	-0.36731900000000	8.94594600000000
H	-5.37973300000000	-3.46714300000000	7.76499800000000
H	-2.83464500000000	-3.04874400000000	9.11986000000000
H	-1.20158200000000	-4.12050400000000	7.72303700000000
H	2.59956100000000	5.41920200000000	-6.14460400000000
H	0.25025700000000	8.09388200000000	-5.83700400000000
H	2.82050400000000	11.24801600000000	-3.40289800000000
H	6.08609900000000	7.26029800000000	-3.74788300000000
H	10.03607800000000	4.22891700000000	-2.74210300000000
H	9.90469300000000	0.41483700000000	-3.57558100000000
H	11.19182300000000	0.77856400000000	-1.58569700000000
H	9.92390000000000	3.89453500000000	-0.36785600000000
H	-1.29857100000000	-1.20482100000000	-8.08427300000000
H	-0.59120100000000	-5.50702100000000	-8.44108800000000
H	2.87361100000000	-3.05219900000000	-5.93135600000000
H	-7.28975100000000	-4.16700500000000	3.76963500000000
H	-9.75707200000000	-1.31803500000000	4.09899800000000
H	-9.37776900000000	-2.76270900000000	1.46916500000000
H	-5.74356100000000	-6.41909000000000	1.79888900000000

H	-3.05541700000000	-8.75970700000000	-0.44894300000000
H	-6.99637800000000	-6.70175500000000	-2.76517000000000
H	2.80901800000000	-7.37774000000000	-0.97344500000000
H	4.92234400000000	-9.58625700000000	2.51312200000000
H	5.84627300000000	-9.05601400000000	0.36277300000000
H	-1.31894900000000	-7.78493200000000	0.89370200000000
H	10.69493700000000	-2.02917400000000	2.58283500000000
H	8.84856900000000	-3.96461800000000	4.80290500000000
H	11.61622800000000	-3.98341100000000	-1.83985200000000
H	5.36358400000000	-0.47338800000000	4.53898300000000
H	7.17762300000000	1.33117000000000	2.39137500000000
H	7.34794000000000	6.56953400000000	-0.49427900000000
H	5.03227100000000	-4.87372500000000	-3.22483000000000
H	7.92757600000000	-7.01002000000000	-1.48756400000000
H	2.46801100000000	-6.50862400000000	2.93046000000000
Ga	-3.53178000000000	-0.40895400000000	1.96728700000000
C	0.04242200000000	-2.66248900000000	4.33276200000000
H	-0.98095200000000	-2.89467400000000	4.61841000000000
H	-4.79947200000000	1.06590000000000	3.30259600000000
H	0.17911500000000	-1.84150100000000	3.63311000000000
C	1.07567800000000	-3.34938000000000	4.80448700000000
H	0.93956900000000	-4.17478000000000	5.49908700000000
H	2.09492200000000	-3.11331100000000	4.51033000000000
H	-2.56425600000000	-1.28035300000000	2.78118000000000
O	-4.53900000000000	1.00253600000000	2.37797500000000

I5-GaO-II

Electronic energy (r^2SCAN-3c): -30614.021983381655 E_h
Electronic energy (ωB97X-D4rev/def2-mTZVPP): -30622.149948525679 E_h
G_{corr} (673.15 K, 1 atm): 0.37962920 E_h
Number of atoms: 243

Cartesian coordinates (Å)

O	1.52096700000000	2.88629300000000	1.04559200000000
O	-1.91961600000000	6.76296800000000	1.44655500000000
O	5.29361400000000	2.07020400000000	0.82113200000000
O	3.63405900000000	3.87442100000000	-0.13968600000000
O	6.11960900000000	4.56785100000000	0.43853600000000
O	1.47523200000000	3.28095300000000	-1.59532800000000
O	1.39639600000000	5.31833400000000	0.04902900000000
O	-1.68825900000000	6.88140700000000	-1.17369300000000
O	0.34821300000000	7.69197000000000	0.42422200000000
O	2.95811800000000	7.47022600000000	-0.02806200000000
Si	4.87171500000000	3.63120400000000	0.88714700000000
Si	2.01528700000000	3.83762400000000	-0.17075600000000
Si	-1.27099700000000	7.61938500000000	0.22136900000000
Si	1.67342700000000	6.78529100000000	0.67892700000000
O	8.97773100000000	-3.84373700000000	2.24449100000000
O	2.02265000000000	6.64385500000000	2.26236800000000
O	7.17078200000000	-2.28922800000000	-0.90462200000000
O	2.53181500000000	4.74242900000000	4.10345000000000
O	6.77404200000000	-4.91366600000000	-0.75326900000000
O	0.07175100000000	5.10857500000000	3.16631600000000
O	5.88023800000000	-3.27628800000000	1.15544400000000
O	4.46538900000000	4.06321400000000	2.40488800000000
O	9.42674300000000	-5.02015400000000	-1.10903600000000

O	5.17823300000000	-0.25519000000000	2.03840800000000
O	7.75939400000000	4.36909100000000	-1.66960200000000
O	10.72889500000000	-3.19400500000000	0.36496700000000
O	5.45590000000000	5.62290000000000	-1.89210000000000
O	4.11467900000000	5.66123800000000	-4.15308200000000
O	8.78707600000000	-1.47001900000000	1.06341500000000
O	3.94782700000000	7.72736300000000	-2.51090400000000
O	1.20219400000000	9.51363800000000	-4.03126200000000
O	9.00643200000000	2.06289900000000	-1.86772100000000
O	6.97220900000000	0.18023700000000	0.12255800000000
O	2.29804700000000	9.54473600000000	-1.54588000000000
O	9.02505600000000	-0.55638600000000	-1.42359000000000
Si	1.45813400000000	5.85334500000000	3.57225400000000
Si	6.14161100000000	-3.44927400000000	-0.43029200000000
Si	3.65612200000000	3.61602900000000	3.74626500000000
Si	8.10465100000000	-5.84525500000000	-0.63765800000000
Si	10.90192300000000	-4.46775900000000	-0.65292800000000
Si	6.69678800000000	5.31780200000000	-0.88922900000000
Si	9.82033000000000	-2.61744900000000	1.58539500000000
Si	4.94861800000000	6.58474200000000	-3.10010700000000
Si	9.23062100000000	3.66438700000000	-1.64847300000000
Si	6.19646400000000	0.84487300000000	1.39229800000000
Si	1.75549100000000	10.44426100000000	-2.80691500000000
Si	9.82582600000000	0.67418200000000	-2.13447300000000
Si	7.98039300000000	-1.02534500000000	-0.28345300000000
Si	3.49418900000000	8.50899300000000	-1.15945100000000
O	3.00342100000000	-0.47960500000000	3.63831100000000
O	0.98677100000000	0.75995800000000	2.50043700000000
O	1.26631200000000	0.87659800000000	5.10444000000000
O	2.99455000000000	2.15293800000000	3.50623000000000
O	-0.37773300000000	1.17521400000000	0.30910300000000
O	-0.79197300000000	2.72891600000000	2.37486100000000
O	-3.71760100000000	4.96777400000000	0.59099500000000
O	-2.50839900000000	4.65718700000000	2.95794700000000
O	-1.14863900000000	3.41780500000000	4.85933500000000
Si	2.07140000000000	0.83340200000000	3.70228600000000
Si	0.33860000000000	1.90416600000000	1.55880700000000
Si	-3.10795000000000	5.73456800000000	1.88719900000000
Si	-1.08482000000000	3.98858900000000	3.34989100000000
O	8.31472500000000	-6.05234600000000	3.51547100000000
O	-3.17396600000000	-3.07632000000000	6.60133100000000
O	6.70706000000000	-7.85589300000000	2.46351600000000
O	-1.28668300000000	-1.57600400000000	7.71111700000000
O	8.28235100000000	-6.35138000000000	0.89106000000000
O	-5.40517700000000	-3.49386800000000	5.26888900000000
O	6.67753200000000	-3.93429300000000	3.59090500000000
O	-5.10601200000000	-1.22697700000000	6.60707100000000
O	-8.65100400000000	0.32857300000000	5.60022300000000
O	4.50180100000000	-2.52129200000000	3.27017300000000
O	-6.42160200000000	1.03617200000000	6.96399000000000
O	4.51747600000000	-5.07416600000000	2.62393400000000
O	-3.86405900000000	0.85463100000000	7.59566500000000
O	-2.22877200000000	2.84152300000000	7.16533400000000
O	0.42414300000000	2.65018300000000	6.88818900000000
O	4.28021600000000	-7.40864800000000	1.39401000000000
O	-1.48720500000000	0.85525900000000	8.80510400000000
O	0.74550900000000	0.07588500000000	7.55370700000000
Si	8.15196200000000	-7.13284000000000	2.30608500000000

Si	-2.10211100000000	-2.98309000000000	7.83680700000000
Si	8.22571600000000	-4.42599000000000	3.57132900000000
Si	-4.78054900000000	-2.82358500000000	6.60601500000000
Si	4.55288800000000	-0.90391800000000	3.39736900000000
Si	-8.05917900000000	1.11885600000000	6.89800500000000
Si	5.38621600000000	-3.71374700000000	2.63333800000000
Si	-5.27507600000000	0.06097800000000	7.58698700000000
Si	-0.91029400000000	3.47920600000000	6.46197200000000
Si	3.46860800000000	-6.10850400000000	1.94450000000000
Si	-2.72428400000000	1.77894200000000	8.28931800000000
Si	1.32154200000000	1.31016700000000	6.66993800000000
Si	5.44213600000000	-8.51764400000000	1.66784800000000
Si	-0.49154100000000	-0.39555500000000	8.49490300000000
O	1.81899800000000	4.26023000000000	-4.04223300000000
O	-0.05031900000000	5.25991800000000	-2.44797500000000
O	-0.58530200000000	7.68104700000000	-3.43872100000000
Si	0.72110100000000	3.90803400000000	-2.89700800000000
Si	-1.18076400000000	6.40272500000000	-2.63803000000000
O	1.27367900000000	-1.73842300000000	-4.48043600000000
O	-3.95248300000000	3.93871400000000	-4.53146600000000
O	3.36780400000000	-2.96778100000000	-3.46390100000000
O	-2.42449500000000	5.82788400000000	-3.49935100000000
O	-3.75888500000000	4.16516100000000	-1.90264900000000
O	1.35978900000000	-4.41941900000000	-4.39666900000000
O	-4.64072900000000	1.76701700000000	-5.89865000000000
O	4.74604000000000	-3.31625300000000	-1.23869200000000
O	-2.54788000000000	1.68971300000000	-4.33771900000000
O	-3.14355000000000	-0.37456500000000	-6.51555600000000
O	-1.08316000000000	-1.80856200000000	-5.64511600000000
O	-0.31252900000000	0.31791200000000	-4.19732900000000
O	-0.35086600000000	2.84077600000000	-3.46337300000000
O	1.80686600000000	6.91749400000000	-4.25329900000000
Si	-3.80831700000000	4.99688200000000	-3.29875300000000
Si	2.21855400000000	-3.05243300000000	-4.62044500000000
Si	-4.03474400000000	2.31491000000000	-4.50472200000000
Si	4.03435500000000	-4.08537600000000	-2.48846100000000
Si	-4.29936300000000	0.65913300000000	-7.05022400000000
Si	-0.27961300000000	-1.30265500000000	-4.33084000000000
Si	-1.20239300000000	1.46257800000000	-3.47771200000000
Si	2.57755000000000	5.55472900000000	-4.69124300000000
Si	0.65322200000000	8.04445800000000	-4.43557200000000
O	-2.77155700000000	-5.60044600000000	-7.09171300000000
O	-4.51095700000000	-4.34352900000000	-5.52605300000000
O	-2.89269100000000	-2.93726100000000	-7.20028900000000
O	-2.78204900000000	0.08686400000000	0.19723100000000
O	-2.46247800000000	2.73998100000000	-0.13161000000000
O	-1.62536300000000	0.98015000000000	-1.98162700000000
O	-5.10905600000000	2.80415600000000	-0.02579400000000
Si	-3.74321900000000	-4.30405400000000	-6.95913800000000
Si	-1.82149100000000	1.27882400000000	-0.40841900000000
Si	-3.78520600000000	3.66513700000000	-0.36540600000000
O	-5.01573800000000	1.91157600000000	-3.27683800000000
O	2.84136400000000	-5.06092800000000	-1.96401600000000
O	-5.56950500000000	-2.52368700000000	2.84661000000000
O	-7.35530300000000	-1.79356800000000	4.68429900000000
O	-0.22602300000000	-6.31445800000000	-3.38530500000000
O	-7.98461700000000	0.19463200000000	3.03306600000000
O	-0.36392900000000	-5.58222300000000	-5.94506800000000

O	-0.93997000000000	-1.93717600000000	-3.00024400000000
O	-3.46023300000000	-6.51858500000000	0.75191500000000
O	-0.94982600000000	-4.28012200000000	-1.84807100000000
O	-6.58768900000000	1.02440800000000	-1.36216000000000
O	0.65178800000000	-6.09109000000000	-0.85476400000000
O	-8.32619200000000	-0.63276300000000	0.51412200000000
O	-7.35591500000000	1.79274900000000	1.03849700000000
O	-1.84790400000000	-6.75926900000000	-1.38703100000000
O	-6.51876300000000	-0.22105400000000	-3.68534000000000
O	2.73323700000000	-5.42848700000000	0.65930500000000
O	-9.04235700000000	-2.66288400000000	-1.01803400000000
O	-4.44146000000000	-0.37499000000000	-2.06625300000000
O	-6.98389100000000	-2.91419600000000	0.62882100000000
O	-8.00842300000000	-2.39974600000000	-3.46336900000000
O	-5.35110000000000	-2.61471200000000	-3.72207700000000
O	-7.24125900000000	-4.37986800000000	-1.85934000000000
O	-4.98416900000000	-5.15842900000000	-3.05622900000000
O	-2.86914300000000	-3.64797400000000	-3.60729200000000
O	-5.66769500000000	-6.16967700000000	-0.70161900000000
O	-2.77389400000000	-2.53607600000000	-1.18266700000000
O	-4.74159200000000	-4.22794900000000	0.92871800000000
O	-4.62304100000000	-1.56292400000000	0.69603500000000
Si	-6.45133300000000	-3.02341100000000	4.12504100000000
Si	0.62630100000000	-5.84087100000000	-4.68839300000000
Si	-8.47217300000000	-0.65929400000000	4.33132700000000
Si	-1.21857100000000	-6.07819600000000	-7.24039500000000
Si	-6.62073000000000	2.28309200000000	-0.33260300000000
Si	-2.41171400000000	-7.49849800000000	-0.03975100000000
Si	-0.59457100000000	-5.85096400000000	-1.87308700000000
Si	-8.40176400000000	0.64847600000000	1.51885200000000
Si	-5.61935200000000	0.56621600000000	-2.58565400000000
Si	2.27523000000000	-6.02914700000000	-0.79160900000000
Si	-8.46156700000000	-2.24477000000000	0.43862100000000
Si	-6.67848000000000	-1.76439300000000	-4.15610200000000
Si	-8.49400200000000	-3.45585700000000	-2.33373100000000
Si	-4.42411000000000	-3.92975200000000	-3.96346300000000
Si	-6.23540900000000	-5.62876000000000	-2.12658800000000
Si	-1.90810100000000	-3.09848400000000	-2.40032300000000
Si	-4.93303000000000	-5.84187900000000	0.71574800000000
Al	-3.67267500000000	-1.15379500000000	-0.77338300000000
Si	-5.49551900000000	-2.83498700000000	1.25269600000000
Si	-2.08797000000000	-1.55853800000000	-6.89296200000000
H	11.69917500000000	-5.54591300000000	-0.01904600000000
H	9.21891100000000	-8.11473500000000	2.38249100000000
H	-3.85612200000000	1.39093400000000	-8.23823000000000
H	-5.52119100000000	-0.08872800000000	-7.35293500000000
H	-6.90663400000000	-1.74222800000000	-5.58763900000000
H	-9.58540700000000	-4.24259300000000	-2.89625100000000
H	-4.78263400000000	-4.39504100000000	-7.96564000000000
H	-1.23015300000000	-7.53714800000000	-7.33581000000000
H	-8.44060700000000	2.52331100000000	6.79066300000000
H	-4.18407600000000	6.49631100000000	2.51400100000000
H	-1.78600400000000	8.99704200000000	0.22067500000000
H	0.70155600000000	11.31895500000000	-2.27862700000000
H	-4.92794200000000	5.92269500000000	-3.32695700000000
H	-7.40670000000000	3.35579600000000	-0.92422700000000
H	-9.78890700000000	1.13212500000000	1.46933500000000
H	-3.23539500000000	2.49785200000000	9.45229700000000

H	-0.71961300000000	4.84607400000000	6.92466100000000
H	1.19719300000000	6.83719000000000	4.62541100000000
H	0.03926500000000	-0.86156600000000	9.77223400000000
H	2.68173200000000	1.60491000000000	7.11731600000000
H	4.55619700000000	3.46492100000000	4.88827200000000
H	1.56263600000000	-6.94686000000000	-4.95462900000000
H	4.66084300000000	9.20647900000000	-0.62722400000000
H	-8.64946000000000	0.52686900000000	8.10789300000000
H	-5.65332300000000	-0.36731900000000	8.94594600000000
H	-5.37973300000000	-3.46714300000000	7.76499800000000
H	-2.83464500000000	-3.04874400000000	9.11986000000000
H	-1.20158200000000	-4.12050400000000	7.72303700000000
H	2.59956100000000	5.41920200000000	-6.14460400000000
H	0.25025700000000	8.09388200000000	-5.83700400000000
H	2.82050400000000	11.24801600000000	-3.40289800000000
H	6.08609900000000	7.26029800000000	-3.74788300000000
H	10.03607800000000	4.22891700000000	-2.74210300000000
H	9.90469300000000	0.41483700000000	-3.57558100000000
H	11.19182300000000	0.77856400000000	-1.58569700000000
H	9.92390000000000	3.89453500000000	-0.36785600000000
H	-1.29857100000000	-1.20482100000000	-8.08427300000000
H	-0.59120100000000	-5.50702100000000	-8.44108800000000
H	2.87361100000000	-3.05219900000000	-5.93135600000000
H	-7.28975100000000	-4.16700500000000	3.76963500000000
H	-9.75707200000000	-1.31803500000000	4.09899800000000
H	-9.37776900000000	-2.76270900000000	1.46916500000000
H	-5.74356100000000	-6.41909000000000	1.79888900000000
H	-3.05541700000000	-8.75970700000000	-0.44894300000000
H	-6.99637800000000	-6.70175500000000	-2.76517000000000
H	2.80901800000000	-7.37774000000000	-0.97344500000000
H	4.92234400000000	-9.58625700000000	2.51312200000000
H	5.84627300000000	-9.05601400000000	0.36277300000000
H	-1.31894900000000	-7.78493200000000	0.89370200000000
H	10.69493700000000	-2.02917400000000	2.58283500000000
H	8.84856900000000	-3.96461800000000	4.80290500000000
H	11.61622800000000	-3.98341100000000	-1.83985200000000
H	5.36358400000000	-0.47338800000000	4.53898300000000
H	7.17762300000000	1.33117000000000	2.39137500000000
H	7.34794000000000	6.56953400000000	-0.49427900000000
H	5.03227100000000	-4.87372500000000	-3.22483000000000
H	7.92757600000000	-7.01002000000000	-1.48756400000000
H	2.46801100000000	-6.50862400000000	2.93046000000000
Ga	-3.52769700000000	-0.40914500000000	1.96898500000000
H	-4.79593400000000	1.06765900000000	3.30113400000000
H	-2.56022300000000	-1.27797600000000	2.78589500000000
O	-4.53792500000000	1.00143100000000	2.37602800000000

TS1-GaO-I-concerted

Electronic energy (r^2SCAN-3c): -30692.492911659308 E_h
Electronic energy (ωB97X-D4rev/def2-mTZVPP): -30700.702692828632 E_h
G_{corr} (673.15 K, 1 atm): 0.41664317 E_h
Number of atoms: 249

Cartesian coordinates (Å)

O	1.51913700000000	2.85681500000000	1.03269800000000
O	-1.91956000000000	6.76132400000000	1.44442200000000
O	5.29463900000000	2.06833500000000	0.81798100000000
O	3.63060800000000	3.86932800000000	-0.13702400000000
O	6.11799700000000	4.56702500000000	0.43630500000000
O	1.47406000000000	3.27933400000000	-1.59753300000000
O	1.39127100000000	5.30493700000000	0.06635800000000
O	-1.69516000000000	6.88281300000000	-1.17777500000000
O	0.34646900000000	7.68636600000000	0.41183300000000
O	2.95509900000000	7.45799700000000	-0.03939100000000
Si	4.87178100000000	3.62925000000000	0.88539000000000
Si	2.01186900000000	3.82833800000000	-0.16943100000000
Si	-1.27506100000000	7.61748800000000	0.21888600000000
Si	1.66991500000000	6.78121200000000	0.67626000000000
O	8.97482100000000	-3.84191400000000	2.24427700000000
O	2.02009000000000	6.65542400000000	2.26096700000000
O	7.17168400000000	-2.29017900000000	-0.90372100000000
O	2.54625300000000	4.76153500000000	4.10436600000000
O	6.77345600000000	-4.91417300000000	-0.75111000000000
O	0.08591900000000	5.09551500000000	3.15625000000000
O	5.87745800000000	-3.27503400000000	1.15572900000000
O	4.46969600000000	4.05734100000000	2.40515500000000
O	9.42638200000000	-5.01983100000000	-1.10908900000000
O	5.18068100000000	-0.25498900000000	2.03695100000000
O	7.75890800000000	4.36915800000000	-1.67006600000000
O	10.72857200000000	-3.19486700000000	0.36559400000000
O	5.45451500000000	5.62406900000000	-1.89236600000000
O	4.11726200000000	5.66433800000000	-4.15604300000000
O	8.78828300000000	-1.46896200000000	1.06297100000000
O	3.95048900000000	7.73178300000000	-2.51438300000000
O	1.19924600000000	9.51318600000000	-4.02872000000000
O	9.00623500000000	2.06275300000000	-1.86691200000000
O	6.97408400000000	0.17999200000000	0.12085200000000
O	2.29869600000000	9.54508500000000	-1.54473400000000
O	9.02595600000000	-0.55745300000000	-1.42525200000000
Si	1.45885600000000	5.85836400000000	3.56897000000000
Si	6.14167800000000	-3.44931200000000	-0.42977000000000
Si	3.65578600000000	3.62402500000000	3.74734100000000
Si	8.10483300000000	-5.84537400000000	-0.63748900000000
Si	10.90198300000000	-4.46797100000000	-0.65307100000000
Si	6.69596300000000	5.31848800000000	-0.89058700000000
Si	9.82032900000000	-2.61723400000000	1.58546200000000
Si	4.94909500000000	6.58672400000000	-3.10108200000000
Si	9.23031500000000	3.66470200000000	-1.64850100000000
Si	6.19901800000000	0.84531100000000	1.38990300000000
Si	1.75537500000000	10.44422300000000	-2.80601300000000
Si	9.82616000000000	0.67463900000000	-2.13486200000000
Si	7.98222500000000	-1.02580500000000	-0.28459000000000
Si	3.49368800000000	8.50745100000000	-1.16037400000000
O	3.00510000000000	-0.46846600000000	3.63295900000000

O	0.96696500000000	0.75062000000000	2.51114100000000
O	1.25955100000000	0.86581300000000	5.10638300000000
O	2.97010600000000	2.16962600000000	3.50682200000000
O	-0.39840100000000	1.14865700000000	0.32015400000000
O	-0.79655400000000	2.72765000000000	2.36982300000000
O	-3.71913200000000	4.96726800000000	0.59776500000000
O	-2.49877100000000	4.66817400000000	2.96769500000000
O	-1.12118000000000	3.41374400000000	4.85866300000000
Si	2.06722300000000	0.83987700000000	3.70235900000000
Si	0.32561400000000	1.89583800000000	1.55628800000000
Si	-3.10824700000000	5.73367500000000	1.89360000000000
Si	-1.08305600000000	3.98656500000000	3.35031400000000
O	8.31156500000000	-6.05076400000000	3.51507900000000
O	-3.17287700000000	-3.06825700000000	6.59789100000000
O	6.70746900000000	-7.85633200000000	2.46368900000000
O	-1.28725600000000	-1.57368100000000	7.70367900000000
O	8.28289000000000	-6.35150200000000	0.89118100000000
O	-5.39884500000000	-3.49673200000000	5.26983200000000
O	6.67557900000000	-3.93066800000000	3.59197500000000
O	-5.10602700000000	-1.22741900000000	6.61140400000000
O	-8.64487800000000	0.32239900000000	5.60477200000000
O	4.49729500000000	-2.51848900000000	3.26892800000000
O	-6.41993300000000	1.04538900000000	6.98102600000000
O	4.51471500000000	-5.07150200000000	2.62621900000000
O	-3.85172000000000	0.83523900000000	7.61268300000000
O	-2.22663500000000	2.84234700000000	7.16745300000000
O	0.42369400000000	2.64602900000000	6.89446900000000
O	4.28171500000000	-7.40720400000000	1.39468800000000
O	-1.47730500000000	0.86217900000000	8.80394100000000
O	0.74628400000000	0.06671400000000	7.54781600000000
Si	8.15208300000000	-7.13253700000000	2.30628100000000
Si	-2.10306700000000	-2.98207100000000	7.83874100000000
Si	8.22392500000000	-4.42439500000000	3.57146800000000
Si	-4.78721000000000	-2.81902800000000	6.60843500000000
Si	4.55417300000000	-0.90111500000000	3.39541400000000
Si	-8.05556100000000	1.12085400000000	6.89846700000000
Si	5.38443900000000	-3.71150700000000	2.63396700000000
Si	-5.27576600000000	0.06587500000000	7.58692600000000
Si	-0.91067400000000	3.47886400000000	6.46309800000000
Si	3.46918900000000	-6.10823000000000	1.94513700000000
Si	-2.72442900000000	1.77961000000000	8.28940900000000
Si	1.32009000000000	1.30939300000000	6.67349700000000
Si	5.44276500000000	-8.51786500000000	1.66800800000000
Si	-0.49184600000000	-0.39275200000000	8.49544400000000
O	1.82513000000000	4.26355700000000	-4.04036400000000
O	-0.04488800000000	5.26425200000000	-2.44422800000000
O	-0.58975700000000	7.67742800000000	-3.44361900000000
Si	0.72422800000000	3.91313700000000	-2.89858600000000
Si	-1.18282500000000	6.40048700000000	-2.63859300000000
O	1.27304900000000	-1.74162300000000	-4.47866500000000
O	-3.95825600000000	3.93248900000000	-4.52569400000000
O	3.36754300000000	-2.96885900000000	-3.46397800000000
O	-2.42159300000000	5.81377200000000	-3.49862800000000
O	-3.77772800000000	4.17015800000000	-1.89623800000000
O	1.35987300000000	-4.42171300000000	-4.39620500000000
O	-4.64230500000000	1.76317300000000	-5.89572500000000
O	4.74633500000000	-3.31597500000000	-1.23870500000000
O	-2.53438000000000	1.69288700000000	-4.35623100000000

O	-3.14519900000000	-0.37700500000000	-6.51662100000000
O	-1.08435900000000	-1.80769400000000	-5.64327700000000
O	-0.30211500000000	0.32046400000000	-4.20151200000000
O	-0.34524000000000	2.84841900000000	-3.47378000000000
O	1.80777100000000	6.91976000000000	-4.25323300000000
Si	-3.81226200000000	4.99378800000000	-3.29534200000000
Si	2.21823200000000	-3.05449300000000	-4.62065100000000
Si	-4.02791500000000	2.30867200000000	-4.50438000000000
Si	4.03469100000000	-4.08544600000000	-2.48839900000000
Si	-4.30014600000000	0.65875500000000	-7.05015200000000
Si	-0.27864300000000	-1.30030600000000	-4.33118200000000
Si	-1.19336300000000	1.46911600000000	-3.48964000000000
Si	2.57962500000000	5.55833700000000	-4.69212200000000
Si	0.65136500000000	8.04437800000000	-4.43547500000000
O	-2.77236300000000	-5.60269700000000	-7.09576100000000
O	-4.50078800000000	-4.34533200000000	-5.52258400000000
O	-2.88855600000000	-2.93978500000000	-7.20141800000000
O	-2.81518100000000	0.11322600000000	0.16658600000000
O	-2.44763700000000	2.75948900000000	-0.14486500000000
O	-1.61681100000000	0.99008300000000	-1.99244600000000
O	-5.09901100000000	2.78579300000000	-0.02518000000000
Si	-3.74158400000000	-4.30481200000000	-6.95981100000000
Si	-1.82940200000000	1.29172700000000	-0.42241800000000
Si	-3.78833500000000	3.66763000000000	-0.35893200000000
O	-4.98629900000000	1.88952000000000	-3.26562800000000
O	2.84151100000000	-5.06031500000000	-1.96236900000000
O	-5.55188300000000	-2.54761800000000	2.85413900000000
O	-7.35930000000000	-1.80289900000000	4.68636200000000
O	-0.22420800000000	-6.31984800000000	-3.38500300000000
O	-7.99674000000000	0.17626900000000	3.02416500000000
O	-0.36697500000000	-5.58253100000000	-5.94349800000000
O	-0.94127500000000	-1.92919400000000	-2.99774300000000
O	-3.45994700000000	-6.51431500000000	0.75307600000000
O	-0.93726600000000	-4.27903900000000	-1.85494700000000
O	-6.57933500000000	1.02171600000000	-1.36221500000000
O	0.65223800000000	-6.09292200000000	-0.85309400000000
O	-8.32967700000000	-0.62821100000000	0.50313200000000
O	-7.35846800000000	1.78949600000000	1.03220200000000
O	-1.84880700000000	-6.75400400000000	-1.38489100000000
O	-6.52173400000000	-0.21874900000000	-3.69073200000000
O	2.73222000000000	-5.42832800000000	0.66060100000000
O	-9.04300100000000	-2.66773300000000	-1.01520300000000
O	-4.45886300000000	-0.42001800000000	-2.07138500000000
O	-6.98361200000000	-2.90608600000000	0.63381800000000
O	-8.01318900000000	-2.39698400000000	-3.46205700000000
O	-5.35971300000000	-2.61970900000000	-3.72513600000000
O	-7.23933000000000	-4.37757200000000	-1.86101200000000
O	-4.98233700000000	-5.16047500000000	-3.05505400000000
O	-2.87501400000000	-3.63774200000000	-3.59108300000000
O	-5.66854400000000	-6.16884300000000	-0.70128100000000
O	-2.74487900000000	-2.53894100000000	-1.15514700000000
O	-4.73616200000000	-4.21905900000000	0.91606200000000
O	-4.62088300000000	-1.55174000000000	0.70835500000000
Si	-6.45228700000000	-3.02419900000000	4.12958000000000
Si	0.62549000000000	-5.84248700000000	-4.68910500000000
Si	-8.46998900000000	-0.65971400000000	4.33214000000000
Si	-1.21901100000000	-6.07871100000000	-7.24118900000000
Si	-6.61887600000000	2.28162400000000	-0.33030000000000

Si	-2.41291900000000	-7.49672700000000	-0.03927800000000
Si	-0.59195600000000	-5.85240900000000	-1.87433200000000
Si	-8.40246900000000	0.64875800000000	1.52019100000000
Si	-5.61647700000000	0.55235400000000	-2.58346400000000
Si	2.27579200000000	-6.02951600000000	-0.79101800000000
Si	-8.46492600000000	-2.23812400000000	0.43975300000000
Si	-6.68394500000000	-1.76325200000000	-4.15670500000000
Si	-8.49551100000000	-3.45505900000000	-2.33261000000000
Si	-4.42572400000000	-3.92874300000000	-3.96046700000000
Si	-6.23625000000000	-5.62792200000000	-2.12725300000000
Si	-1.90084700000000	-3.09535500000000	-2.39338900000000
Si	-4.93378000000000	-5.83682500000000	0.71415300000000
Al	-3.66836000000000	-1.16543800000000	-0.77318500000000
Si	-5.49835200000000	-2.83154000000000	1.25903600000000
Si	-2.08790300000000	-1.55887400000000	-6.89320300000000
H	11.69917500000000	-5.54591300000000	-0.01904600000000
H	9.21891100000000	-8.11473500000000	2.38249100000000
H	-3.85612200000000	1.39093400000000	-8.23823000000000
H	-5.52119100000000	-0.08872800000000	-7.35293500000000
H	-6.90663400000000	-1.74222800000000	-5.58763900000000
H	-9.58540700000000	-4.24259300000000	-2.89625100000000
H	-4.78263400000000	-4.39504100000000	-7.96564000000000
H	-1.23015300000000	-7.53714800000000	-7.33581000000000
H	-8.44060700000000	2.52331100000000	6.79066300000000
H	-4.18407600000000	6.49631100000000	2.51400100000000
H	-1.78600400000000	8.99704200000000	0.22067500000000
H	0.70155600000000	11.31895500000000	-2.27862700000000
H	-4.92794200000000	5.92269500000000	-3.32695700000000
H	-7.40670000000000	3.35579600000000	-0.92422700000000
H	-9.78890700000000	1.13212500000000	1.46933500000000
H	-3.23539500000000	2.49785200000000	9.45229700000000
H	-0.71961300000000	4.84607400000000	6.92466100000000
H	1.19719300000000	6.83719000000000	4.62541100000000
H	0.03926500000000	-0.86156600000000	9.77223400000000
H	2.68173200000000	1.60491000000000	7.11731600000000
H	4.55619700000000	3.46492100000000	4.88827200000000
H	1.56263600000000	-6.94686000000000	-4.95462900000000
H	4.66084300000000	9.20647900000000	-0.62722400000000
H	-8.64946000000000	0.52686900000000	8.10789300000000
H	-5.65332300000000	-0.36731900000000	8.94594600000000
H	-5.37973300000000	-3.46714300000000	7.76499800000000
H	-2.83464500000000	-3.04874400000000	9.11986000000000
H	-1.20158200000000	-4.12050400000000	7.72303700000000
H	2.59956100000000	5.41920200000000	-6.14460400000000
H	0.25025700000000	8.09388200000000	-5.83700400000000
H	2.82050400000000	11.24801600000000	-3.40289800000000
H	6.08609900000000	7.26029800000000	-3.74788300000000
H	10.03607800000000	4.22891700000000	-2.74210300000000
H	9.90469300000000	0.41483700000000	-3.57558100000000
H	11.19182300000000	0.77856400000000	-1.58569700000000
H	9.92390000000000	3.89453500000000	-0.36785600000000
H	-1.29857100000000	-1.20482100000000	-8.08427300000000
H	-0.59120100000000	-5.50702100000000	-8.44108800000000
H	2.87361100000000	-3.05219900000000	-5.93135600000000
H	-7.28975100000000	-4.16700500000000	3.76963500000000
H	-9.75707200000000	-1.31803500000000	4.09899800000000
H	-9.37776900000000	-2.76270900000000	1.46916500000000
H	-5.74356100000000	-6.41909000000000	1.79888900000000

H	-3.05541700000000	-8.75970700000000	-0.44894300000000
H	-6.99637800000000	-6.70175500000000	-2.76517000000000
H	2.80901800000000	-7.37774000000000	-0.97344500000000
H	4.92234400000000	-9.58625700000000	2.51312200000000
H	5.84627300000000	-9.05601400000000	0.36277300000000
H	-1.31894900000000	-7.78493200000000	0.89370200000000
H	10.69493700000000	-2.02917400000000	2.58283500000000
H	8.84856900000000	-3.96461800000000	4.80290500000000
H	11.61622800000000	-3.98341100000000	-1.83985200000000
H	5.36358400000000	-0.47338800000000	4.53898300000000
H	7.17762300000000	1.33117000000000	2.39137500000000
H	7.34794000000000	6.56953400000000	-0.49427900000000
H	5.03227100000000	-4.87372500000000	-3.22483000000000
H	7.92757600000000	-7.01002000000000	-1.48756400000000
H	2.46801100000000	-6.50862400000000	2.93046000000000
Ga	-3.76639300000000	-0.15964900000000	1.90711100000000
C	-2.19967500000000	-1.39423300000000	2.84018100000000
H	-1.48480300000000	-1.24737800000000	2.02796600000000
H	-3.56547700000000	0.98648900000000	4.13105900000000
H	-2.77800200000000	-2.32047000000000	2.81665700000000
C	-1.87940100000000	-0.87914300000000	4.07308400000000
H	-2.35324700000000	-1.24400700000000	4.97802900000000
H	-3.01857900000000	0.80798200000000	4.67879800000000
H	-1.07344000000000	-0.16093600000000	4.19085200000000
O	-4.58826300000000	1.09691700000000	2.70850400000000

I2-GaO-II

Electronic energy (r^2SCAN-3c): -30692.606676910651 E_h
Electronic energy (ωB97X-D4rev/def2-mTZVPP): -30700.83399 E_h
G_{corr} (673.15 K, 1 atm): 0.41962608 E_h
Number of atoms: 249

Cartesian coordinates (Å)

O	1.50965900000000	2.89901300000000	1.04650100000000
O	-1.93845600000000	6.77058700000000	1.43596400000000
O	5.27558200000000	2.07967900000000	0.81225900000000
O	3.61590100000000	3.88333300000000	-0.15037200000000
O	6.10105600000000	4.57760000000000	0.42953800000000
O	1.45372400000000	3.28406800000000	-1.59990300000000
O	1.37925800000000	5.32726800000000	0.04074900000000
O	-1.69664300000000	6.88730700000000	-1.18083700000000
O	0.33239800000000	7.70208800000000	0.42271600000000
O	2.94133800000000	7.47936400000000	-0.03660800000000
Si	4.85272600000000	3.64105700000000	0.87651600000000
Si	1.99627400000000	3.84528000000000	-0.17847700000000
Si	-1.28604000000000	7.62853700000000	0.21347200000000
Si	1.65761100000000	6.79368300000000	0.67101900000000
O	8.95839100000000	-3.83281000000000	2.23508100000000
O	2.01027200000000	6.65060200000000	2.25405700000000
O	7.15473900000000	-2.28032900000000	-0.91231500000000
O	2.51217500000000	4.74984700000000	4.09644000000000
O	6.75660700000000	-4.90468300000000	-0.76077300000000
O	0.05344600000000	5.12107500000000	3.15584700000000
O	5.86049400000000	-3.26707200000000	1.14616700000000
O	4.44593100000000	4.07055600000000	2.39542000000000
O	9.40945100000000	-5.01060000000000	-1.11834800000000

O	5.16152700000000	-0.24548800000000	2.02886100000000
O	7.74167700000000	4.37778300000000	-1.67758600000000
O	10.71098300000000	-3.18499200000000	0.35599000000000
O	5.43813100000000	5.63179500000000	-1.90198200000000
O	4.10925200000000	5.68164400000000	-4.17434000000000
O	8.77027800000000	-1.45924000000000	1.05470200000000
O	3.93056300000000	7.73623000000000	-2.51884900000000
O	1.17853800000000	9.52097300000000	-4.03592700000000
O	8.98922100000000	2.07223400000000	-1.87634700000000
O	6.95474500000000	0.18950500000000	0.11295300000000
O	2.28103100000000	9.55439000000000	-1.55422700000000
O	9.00822300000000	-0.54694100000000	-1.43295300000000
Si	1.44112500000000	5.86302700000000	3.56299400000000
Si	6.12464800000000	-3.43979500000000	-0.43985700000000
Si	3.63764000000000	3.62596800000000	3.73744700000000
Si	8.08779100000000	-5.83592900000000	-0.64705800000000
Si	10.88487100000000	-4.45848100000000	-0.66223300000000
Si	6.67868400000000	5.32739900000000	-0.89866800000000
Si	9.80298400000000	-2.60741200000000	1.57607500000000
Si	4.93397300000000	6.59681100000000	-3.10927100000000
Si	9.21322200000000	3.67403200000000	-1.65753300000000
Si	6.17980700000000	0.85549000000000	1.38206300000000
Si	1.73756600000000	10.45309900000000	-2.81574300000000
Si	9.80889900000000	0.68406300000000	-2.14394800000000
Si	7.96403900000000	-1.01535800000000	-0.29278300000000
Si	3.47686500000000	8.51847100000000	-1.16802300000000
O	2.98166700000000	-0.47219400000000	3.61988400000000
O	0.96104100000000	0.76673500000000	2.48844900000000
O	1.24665500000000	0.87352100000000	5.09288900000000
O	2.97420600000000	2.16157000000000	3.50100700000000
O	-0.38395600000000	1.19889800000000	0.28072700000000
O	-0.80858700000000	2.73783900000000	2.36604900000000
O	-3.74450000000000	4.97416100000000	0.58446000000000
O	-2.52677800000000	4.66309500000000	2.94449200000000
O	-1.16481800000000	3.42882100000000	4.84821500000000
Si	2.05098200000000	0.84342200000000	3.68786700000000
Si	0.31744200000000	1.91629100000000	1.53935400000000
Si	-3.12741200000000	5.74343100000000	1.87565300000000
Si	-1.10117500000000	3.99944500000000	3.33814300000000
O	8.29646800000000	-6.04230300000000	3.50600700000000
O	-3.18805400000000	-3.06031200000000	6.58459700000000
O	6.68950200000000	-7.84549000000000	2.45428700000000
O	-1.29896200000000	-1.56896900000000	7.69524300000000
O	8.26576300000000	-6.34214900000000	0.88177700000000
O	-5.41793400000000	-3.48287900000000	5.25347100000000
O	6.65905600000000	-3.92459300000000	3.58237800000000
O	-5.11986600000000	-1.21807600000000	6.60099500000000
O	-8.66681100000000	0.33994900000000	5.58924000000000
O	4.48199800000000	-2.51153900000000	3.26082300000000
O	-6.43955400000000	1.04837700000000	6.96251500000000
O	4.49655800000000	-5.06273200000000	2.61670800000000
O	-3.87769700000000	0.85845200000000	7.58913900000000
O	-2.24289700000000	2.84840100000000	7.15629200000000
O	0.40838500000000	2.65824200000000	6.87170100000000
O	4.26356600000000	-7.39854000000000	1.38440300000000
O	-1.50348300000000	0.86347000000000	8.79523000000000
O	0.72747800000000	0.08282300000000	7.54041000000000
Si	8.13514000000000	-7.12323400000000	2.29671400000000

Si	-2.12161600000000	-2.97341800000000	7.82656700000000
Si	8.20810700000000	-4.41601800000000	3.56206900000000
Si	-4.80211600000000	-2.81413700000000	6.59214000000000
Si	4.53505100000000	-0.89351500000000	3.38653600000000
Si	-8.07648200000000	1.12843800000000	6.88808500000000
Si	5.36866000000000	-3.70376300000000	2.62427500000000
Si	-5.29386000000000	0.07132600000000	7.57736400000000
Si	-0.92800300000000	3.48918900000000	6.45083400000000
Si	3.45204300000000	-6.09978900000000	1.93526000000000
Si	-2.74288100000000	1.78751400000000	8.28001300000000
Si	1.30464800000000	1.31822500000000	6.65757900000000
Si	5.42562800000000	-8.50862400000000	1.65863400000000
Si	-0.50864900000000	-0.38599000000000	8.48531900000000
O	1.83115600000000	4.27401700000000	-4.03740000000000
O	-0.05548100000000	5.26741900000000	-2.45799100000000
O	-0.60788800000000	7.67972600000000	-3.45516800000000
Si	0.71744200000000	3.91676800000000	-2.91049300000000
Si	-1.19339500000000	6.40248500000000	-2.64476400000000
O	1.25379500000000	-1.73205500000000	-4.49027100000000
O	-3.97559900000000	3.94912700000000	-4.54260100000000
O	3.35050300000000	-2.95683600000000	-3.47268700000000
O	-2.44016200000000	5.81401400000000	-3.49022300000000
O	-3.82863800000000	4.16921600000000	-1.90640000000000
O	1.34944400000000	-4.41307700000000	-4.40636400000000
O	-4.65714300000000	1.77752600000000	-5.91282900000000
O	4.72941400000000	-3.30602700000000	-1.24813000000000
O	-2.54435000000000	1.71856300000000	-4.37810900000000
O	-3.16177500000000	-0.36846000000000	-6.52577600000000
O	-1.10526200000000	-1.80810100000000	-5.65194200000000
O	-0.32366000000000	0.32823400000000	-4.22728800000000
O	-0.34036400000000	2.85913800000000	-3.51369300000000
O	1.79310100000000	6.92928800000000	-4.26346700000000
Si	-3.83518100000000	4.99920700000000	-3.30474900000000
Si	2.20086400000000	-3.04230200000000	-4.63062500000000
Si	-4.04138700000000	2.32362000000000	-4.52275700000000
Si	4.01750200000000	-4.07440600000000	-2.49909500000000
Si	-4.31614000000000	0.66679500000000	-7.06091900000000
Si	-0.30005500000000	-1.29308800000000	-4.34151700000000
Si	-1.20238900000000	1.48588000000000	-3.51265800000000
Si	2.56938400000000	5.56954400000000	-4.70156200000000
Si	0.63423200000000	8.05102200000000	-4.44488900000000
O	-2.78887000000000	-5.59273500000000	-7.10568700000000
O	-4.51392300000000	-4.33282600000000	-5.53141100000000
O	-2.90715100000000	-2.92986700000000	-7.21888200000000
O	-2.79604800000000	0.12883100000000	0.17460500000000
O	-2.46486200000000	2.76784100000000	-0.15422100000000
O	-1.61453100000000	1.02828200000000	-2.01394700000000
O	-5.11442300000000	2.78710400000000	0.00856900000000
Si	-3.75853600000000	-4.29450300000000	-6.96937000000000
Si	-1.83905700000000	1.28862600000000	-0.43558000000000
Si	-3.80251300000000	3.66885100000000	-0.37177600000000
O	-5.00618700000000	1.91034600000000	-3.28815800000000
O	2.82652200000000	-5.05027200000000	-1.96971100000000
O	-5.60604200000000	-2.51103800000000	2.83038200000000
O	-7.37694900000000	-1.79100200000000	4.68802300000000
O	-0.24450300000000	-6.28971000000000	-3.38774700000000
O	-8.00243700000000	0.19371900000000	3.02092600000000
O	-0.38257900000000	-5.57400800000000	-5.95245300000000

O	-0.95439600000000	-1.92082400000000	-3.00459400000000
O	-3.47523100000000	-6.50455200000000	0.74985100000000
O	-0.97561300000000	-4.25598800000000	-1.83626200000000
O	-6.58668500000000	1.03874300000000	-1.38506400000000
O	0.63173100000000	-6.06765200000000	-0.85776300000000
O	-8.32782600000000	-0.64227400000000	0.50506300000000
O	-7.34771000000000	1.77164700000000	1.02904100000000
O	-1.86512600000000	-6.74301300000000	-1.39078700000000
O	-6.52121900000000	-0.21162700000000	-3.69860700000000
O	2.71037600000000	-5.41866800000000	0.65380200000000
O	-9.06191400000000	-2.65766300000000	-1.02919700000000
O	-4.43581900000000	-0.37736900000000	-2.07768700000000
O	-7.02889900000000	-2.97204600000000	0.64495500000000
O	-8.03190500000000	-2.37978500000000	-3.47289000000000
O	-5.37674700000000	-2.61347400000000	-3.73220700000000
O	-7.25494900000000	-4.36668700000000	-1.88299700000000
O	-4.99699400000000	-5.15665400000000	-3.06934800000000
O	-2.88975200000000	-3.62787200000000	-3.59799600000000
O	-5.67982100000000	-6.14757300000000	-0.70795100000000
O	-2.78664600000000	-2.50228700000000	-1.16965600000000
O	-4.74173800000000	-4.21059000000000	0.93583700000000
O	-4.73919600000000	-1.53313800000000	0.63657400000000
Si	-6.47090600000000	-3.01313000000000	4.10889900000000
Si	0.60820700000000	-5.83028200000000	-4.69622200000000
Si	-8.48709700000000	-0.65501900000000	4.32524700000000
Si	-1.23601600000000	-6.06904200000000	-7.24995700000000
Si	-6.63143600000000	2.28472300000000	-0.34809300000000
Si	-2.43266200000000	-7.48579500000000	-0.04640100000000
Si	-0.61797500000000	-5.82642500000000	-1.87668900000000
Si	-8.41844100000000	0.63935300000000	1.50529400000000
Si	-5.60804900000000	0.56176900000000	-2.59866800000000
Si	2.25555100000000	-6.01869000000000	-0.80108600000000
Si	-8.47585600000000	-2.25764200000000	0.43124500000000
Si	-6.69676800000000	-1.75395900000000	-4.16520800000000
Si	-8.51152300000000	-3.44359700000000	-2.34701700000000
Si	-4.43996500000000	-3.91982500000000	-3.96724100000000
Si	-6.25009000000000	-5.61777400000000	-2.13671600000000
Si	-1.93420200000000	-3.06777900000000	-2.39202800000000
Si	-4.94891700000000	-5.81833100000000	0.71251500000000
Al	-3.69632700000000	-1.11870900000000	-0.72762500000000
Si	-5.52274100000000	-2.81589000000000	1.23198800000000
Si	-2.10532900000000	-1.55150800000000	-6.90283800000000
H	11.68201100000000	-5.53663200000000	-0.02821500000000
H	9.20174700000000	-8.10545400000000	2.37332200000000
H	-3.87328600000000	1.40021500000000	-8.24739900000000
H	-5.53835500000000	-0.07944700000000	-7.36210400000000
H	-6.92379800000000	-1.73294700000000	-5.59680800000000
H	-9.60257100000000	-4.23331200000000	-2.90542000000000
H	-4.79979800000000	-4.38576000000000	-7.97480900000000
H	-1.24731700000000	-7.52786700000000	-7.34497900000000
H	-8.45777100000000	2.53259200000000	6.78149400000000
H	-4.20124000000000	6.50559200000000	2.50483200000000
H	-1.80316800000000	9.00632300000000	0.21150600000000
H	0.68439200000000	11.32823500000000	-2.28779600000000
H	-4.94510600000000	5.93197600000000	-3.33612600000000
H	-7.42386400000000	3.36507600000000	-0.93339600000000
H	-9.80607100000000	1.14140600000000	1.46016600000000
H	-3.25255900000000	2.50713300000000	9.44312800000000

H	-0.73677700000000	4.85535500000000	6.91549200000000
H	1.18002900000000	6.84647100000000	4.61624200000000
H	0.02210100000000	-0.85228500000000	9.76306500000000
H	2.66456800000000	1.61419100000000	7.10814700000000
H	4.53903300000000	3.47420200000000	4.87910300000000
H	1.54547200000000	-6.93757900000000	-4.96379800000000
H	4.64367900000000	9.21576000000000	-0.63639300000000
H	-8.66662400000000	0.53615000000000	8.09872400000000
H	-5.67048700000000	-0.35803800000000	8.93677700000000
H	-5.39689700000000	-3.45786300000000	7.75582900000000
H	-2.85180900000000	-3.03946300000000	9.11069100000000
H	-1.21874600000000	-4.11122300000000	7.71386800000000
H	2.58239700000000	5.42848300000000	-6.15377300000000
H	0.23309300000000	8.10316300000000	-5.84617300000000
H	2.80334000000000	11.25729700000000	-3.41206700000000
H	6.06893500000000	7.26957900000000	-3.75705200000000
H	10.01891400000000	4.23819800000000	-2.75127200000000
H	9.88752900000000	0.42411800000000	-3.58475000000000
H	11.17465900000000	0.78784500000000	-1.59486600000000
H	9.90673600000000	3.90381600000000	-0.37702500000000
H	-1.31573500000000	-1.19554000000000	-8.09344200000000
H	-0.60836500000000	-5.49774000000000	-8.45025700000000
H	2.85644700000000	-3.04291800000000	-5.94052500000000
H	-7.30691500000000	-4.15772400000000	3.76046600000000
H	-9.77423600000000	-1.30875400000000	4.08982900000000
H	-9.39493300000000	-2.75342800000000	1.45999600000000
H	-5.76072500000000	-6.40980900000000	1.78972000000000
H	-3.07258100000000	-8.75042600000000	-0.45811200000000
H	-7.01354200000000	-6.69247400000000	-2.77433900000000
H	2.79185400000000	-7.36845900000000	-0.98261400000000
H	4.90518000000000	-9.57697600000000	2.50395300000000
H	5.82910900000000	-9.04673300000000	0.35360400000000
H	-1.33611300000000	-7.77565100000000	0.88453300000000
H	10.67777300000000	-2.01989300000000	2.57366600000000
H	8.83140500000000	-3.95533700000000	4.79373600000000
H	11.59906400000000	-3.97413000000000	-1.84902100000000
H	5.34642000000000	-0.46410700000000	4.52981400000000
H	7.16045900000000	1.34045100000000	2.38220600000000
H	7.33077600000000	6.57881500000000	-0.50344800000000
H	5.01510700000000	-4.86444400000000	-3.23399900000000
H	7.91041200000000	-7.00073900000000	-1.49673300000000
H	2.45084700000000	-6.49934300000000	2.92129100000000
Ga	-3.91831500000000	0.03474500000000	2.13443200000000
C	-0.78323300000000	-3.43370500000000	2.68627400000000
H	-1.56288700000000	-4.16338700000000	2.92653800000000
H	-0.29143500000000	-3.11615400000000	3.61408600000000
H	-0.02842300000000	-3.92520500000000	2.06245100000000
C	-1.36174100000000	-2.25713500000000	1.93362600000000
H	-1.84526100000000	-2.58368500000000	1.01059400000000
H	-0.59561900000000	-1.51188000000000	1.68682000000000
H	-2.10610000000000	-1.47623700000000	3.61516300000000
O	-2.41958000000000	-1.60805400000000	2.70957800000000

TS2-GaO-II

Electronic energy (r^{2}SCAN-3c): -30692.527417600726 E_h
Electronic energy (ωB97X-D4rev/def2-mTZVPP): -30700.74172 E_h
G_{corr} (673.15 K, 1 atm): 0.41802992 E_h
Number of atoms: 249

Cartesian coordinates (Å)

O	1.50129700000000	2.89446200000000	1.03356300000000
O	-1.93773200000000	6.77112700000000	1.43761000000000
O	5.27604700000000	2.08058800000000	0.81430900000000
O	3.61787800000000	3.88551000000000	-0.14586200000000
O	6.10448400000000	4.57700700000000	0.42918800000000
O	1.46396400000000	3.28789400000000	-1.60498300000000
O	1.37954300000000	5.32756100000000	0.04050700000000
O	-1.69871000000000	6.88835000000000	-1.17890600000000
O	0.33296000000000	7.70188700000000	0.42187200000000
O	2.94156900000000	7.47907300000000	-0.03701700000000
Si	4.85627200000000	3.64219700000000	0.87987600000000
Si	1.99949500000000	3.84708200000000	-0.17967100000000
Si	-1.28533200000000	7.62926700000000	0.21549900000000
Si	1.65850800000000	6.79362900000000	0.67204000000000
O	8.95891000000000	-3.83325700000000	2.23518400000000
O	2.01014700000000	6.65077100000000	2.25458900000000
O	7.15397700000000	-2.28002700000000	-0.91271500000000
O	2.51080700000000	4.74625700000000	4.09348000000000
O	6.75677400000000	-4.90438100000000	-0.76128600000000
O	0.05223700000000	5.12309000000000	3.15485100000000
O	5.86134800000000	-3.26643900000000	1.14661600000000
O	4.45143700000000	4.07713700000000	2.39719100000000
O	9.40948300000000	-5.01086200000000	-1.11849300000000
O	5.15940600000000	-0.24540600000000	2.02987900000000
O	7.74242600000000	4.37887500000000	-1.67994600000000
O	10.71082700000000	-3.18474400000000	0.35575900000000
O	5.43829300000000	5.63242300000000	-1.90094700000000
O	4.10064000000000	5.67420600000000	-4.16533000000000
O	8.77012000000000	-1.45936300000000	1.05466100000000
O	3.93018200000000	7.73681000000000	-2.51986600000000
O	1.17760400000000	9.51927400000000	-4.03403000000000
O	8.98914000000000	2.07238600000000	-1.87693400000000
O	6.95341900000000	0.18924200000000	0.11391600000000
O	2.28116800000000	9.55445300000000	-1.55371000000000
O	9.00703500000000	-0.54631800000000	-1.43257200000000
Si	1.44110700000000	5.86185900000000	3.56375200000000
Si	6.12449900000000	-3.43988700000000	-0.43896700000000
Si	3.64057700000000	3.62532400000000	3.73612000000000
Si	8.08763000000000	-5.83602300000000	-0.64677100000000
Si	10.88465700000000	-4.45847500000000	-0.66213400000000
Si	6.68014600000000	5.32723300000000	-0.89894600000000
Si	9.80278000000000	-2.60765300000000	1.57627300000000
Si	4.93231200000000	6.59515600000000	-3.10879200000000
Si	9.21358400000000	3.67382300000000	-1.65778900000000
Si	6.17855600000000	0.85434900000000	1.38392600000000
Si	1.73777400000000	10.45297400000000	-2.81509200000000
Si	9.80859100000000	0.68367700000000	-2.14366200000000
Si	7.96293900000000	-1.01543500000000	-0.29210500000000
Si	3.47722100000000	8.51829300000000	-1.16810800000000
O	2.98283300000000	-0.47132000000000	3.62823700000000

O	0.96980200000000	0.77423500000000	2.49001400000000
O	1.25212400000000	0.89921400000000	5.09292000000000
O	2.98631100000000	2.15979000000000	3.48840800000000
O	-0.40191600000000	1.18405300000000	0.29922300000000
O	-0.81067900000000	2.74366000000000	2.36276900000000
O	-3.74150400000000	4.97415600000000	0.58787700000000
O	-2.52652600000000	4.66439800000000	2.94961500000000
O	-1.15799500000000	3.42836800000000	4.85132400000000
Si	2.05625500000000	0.84653400000000	3.69141900000000
Si	0.31635300000000	1.91577100000000	1.54688000000000
Si	-3.12476800000000	5.74414500000000	1.88127800000000
Si	-1.10028300000000	3.99958000000000	3.34360500000000
O	8.29591800000000	-6.04212800000000	3.50592400000000
O	-3.19120200000000	-3.07561400000000	6.59282400000000
O	6.68995400000000	-7.84655400000000	2.45416900000000
O	-1.30443000000000	-1.56773600000000	7.70112600000000
O	8.26569100000000	-6.34209500000000	0.88188000000000
O	-5.41894600000000	-3.48364800000000	5.25107100000000
O	6.65934500000000	-3.92297100000000	3.58237200000000
O	-5.12361100000000	-1.22146700000000	6.60141600000000
O	-8.67039200000000	0.34991300000000	5.58457700000000
O	4.48115900000000	-2.51174800000000	3.26043000000000
O	-6.43960200000000	1.04512200000000	6.96053200000000
O	4.49886700000000	-5.06392700000000	2.61594700000000
O	-3.87947200000000	0.85934600000000	7.59152700000000
O	-2.24680500000000	2.85114000000000	7.15680700000000
O	0.40710500000000	2.66059000000000	6.88399700000000
O	4.26365700000000	-7.39867100000000	1.38469500000000
O	-1.49977700000000	0.86752700000000	8.79399700000000
O	0.72632800000000	0.08126100000000	7.53695300000000
Si	8.13482400000000	-7.12338400000000	2.29694400000000
Si	-2.12121000000000	-2.97501000000000	7.82618800000000
Si	8.20759200000000	-4.41580800000000	3.56226800000000
Si	-4.79711200000000	-2.81595900000000	6.59146600000000
Si	4.53392700000000	-0.89414000000000	3.38869200000000
Si	-8.07451600000000	1.12927000000000	6.88892900000000
Si	5.36805500000000	-3.70372900000000	2.62468600000000
Si	-5.29203300000000	0.06746300000000	7.57632600000000
Si	-0.92851500000000	3.48783400000000	6.45631500000000
Si	3.45159700000000	-6.09939600000000	1.93565800000000
Si	-2.74279300000000	1.78634600000000	8.28024200000000
Si	1.30348000000000	1.32116900000000	6.66295400000000
Si	5.42514900000000	-8.50849400000000	1.65867400000000
Si	-0.50996100000000	-0.38752300000000	8.48401600000000
O	1.81098400000000	4.26905200000000	-4.05073900000000
O	-0.05628900000000	5.26832800000000	-2.45323500000000
O	-0.61181700000000	7.67861300000000	-3.45666900000000
Si	0.71268300000000	3.91631600000000	-2.90717300000000
Si	-1.19393900000000	6.40336700000000	-2.64164600000000
O	1.25187500000000	-1.73515700000000	-4.48379700000000
O	-3.97415800000000	3.94457300000000	-4.53663100000000
O	3.35104000000000	-2.95898400000000	-3.47336200000000
O	-2.44040400000000	5.80892300000000	-3.48441100000000
O	-3.83425000000000	4.16409400000000	-1.90173500000000
O	1.34522900000000	-4.41361700000000	-4.40608700000000
O	-4.65635500000000	1.77548400000000	-5.91009800000000
O	4.72925600000000	-3.30644800000000	-1.24792400000000
O	-2.55232200000000	1.70584500000000	-4.36151400000000

O	-3.16136600000000	-0.36753800000000	-6.52535000000000
O	-1.10415700000000	-1.80134900000000	-5.64977700000000
O	-0.32422300000000	0.32582800000000	-4.20755800000000
O	-0.35867900000000	2.85294100000000	-3.48040300000000
O	1.78872500000000	6.92596600000000	-4.26138300000000
Si	-3.83596100000000	4.99723000000000	-3.29999700000000
Si	2.20093900000000	-3.04517800000000	-4.62917200000000
Si	-4.04433100000000	2.32015600000000	-4.51854300000000
Si	4.01758800000000	-4.07603900000000	-2.49752800000000
Si	-4.31586800000000	0.66648700000000	-7.06107900000000
Si	-0.30044000000000	-1.29568600000000	-4.33512900000000
Si	-1.21158900000000	1.47561300000000	-3.49412600000000
Si	2.56333800000000	5.56573800000000	-4.70113700000000
Si	0.63258600000000	8.05029900000000	-4.44427500000000
O	-2.78915000000000	-5.59239000000000	-7.10335900000000
O	-4.52092600000000	-4.33178300000000	-5.53351200000000
O	-2.90687300000000	-2.92954200000000	-7.21345200000000
O	-2.81955600000000	0.10911100000000	0.17727600000000
O	-2.48190800000000	2.75407800000000	-0.14374200000000
O	-1.63889700000000	1.00273200000000	-1.99774500000000
O	-5.12035200000000	2.78670900000000	0.01669700000000
Si	-3.75906100000000	-4.29491200000000	-6.96909800000000
Si	-1.84528600000000	1.28661400000000	-0.42331100000000
Si	-3.81105500000000	3.67031900000000	-0.36520500000000
O	-5.01441300000000	1.90893900000000	-3.28569300000000
O	2.82476200000000	-5.05103400000000	-1.97170700000000
O	-5.64141900000000	-2.48748900000000	2.82470900000000
O	-7.38751000000000	-1.79452000000000	4.70304100000000
O	-0.24304400000000	-6.30522400000000	-3.39323500000000
O	-7.99892700000000	0.17931700000000	3.01682700000000
O	-0.38290400000000	-5.57307400000000	-5.95308700000000
O	-0.96530800000000	-1.92112800000000	-3.00169500000000
O	-3.47707000000000	-6.49801800000000	0.73949500000000
O	-0.96291600000000	-4.26854400000000	-1.85671200000000
O	-6.58638700000000	1.03430000000000	-1.37635000000000
O	0.63460000000000	-6.08047700000000	-0.86244000000000
O	-8.34612600000000	-0.63243900000000	0.49471600000000
O	-7.34935300000000	1.77032800000000	1.03420300000000
O	-1.86496400000000	-6.74835200000000	-1.39506800000000
O	-6.53243200000000	-0.21215500000000	-3.69729400000000
O	2.71497800000000	-5.41864200000000	0.65134600000000
O	-9.07455900000000	-2.65740300000000	-1.03295800000000
O	-4.44819500000000	-0.38512200000000	-2.08559000000000
O	-7.01748300000000	-2.93320400000000	0.60245200000000
O	-8.03477600000000	-2.38002300000000	-3.47336200000000
O	-5.37750800000000	-2.61165900000000	-3.73067100000000
O	-7.25871200000000	-4.36450700000000	-1.88288600000000
O	-5.00096500000000	-5.15431600000000	-3.06832800000000
O	-2.89185700000000	-3.63174700000000	-3.60324400000000
O	-5.68746400000000	-6.14244200000000	-0.70566700000000
O	-2.78538000000000	-2.53299500000000	-1.16828800000000
O	-4.75628700000000	-4.20186300000000	0.93116000000000
O	-4.71625500000000	-1.51629700000000	0.65704700000000
Si	-6.47714300000000	-3.01041600000000	4.12039000000000
Si	0.60828900000000	-5.83303800000000	-4.69737700000000
Si	-8.48940800000000	-0.65512400000000	4.33064600000000
Si	-1.23585700000000	-6.06921300000000	-7.24980200000000
Si	-6.63601600000000	2.28452800000000	-0.34230500000000

Si	-2.43182100000000	-7.48584300000000	-0.04726400000000
Si	-0.61190600000000	-5.84072300000000	-1.88139400000000
Si	-8.42240300000000	0.63946000000000	1.50943900000000
Si	-5.62063100000000	0.56278900000000	-2.60035800000000
Si	2.25793100000000	-6.01979300000000	-0.80024200000000
Si	-8.48559300000000	-2.24699000000000	0.42245600000000
Si	-6.69991500000000	-1.75601000000000	-4.16574900000000
Si	-8.51562000000000	-3.44332400000000	-2.34778300000000
Si	-4.44287900000000	-3.92114900000000	-3.96962700000000
Si	-6.25403300000000	-5.61641500000000	-2.13620700000000
Si	-1.92806400000000	-3.08620900000000	-2.39710300000000
Si	-4.95007400000000	-5.81685800000000	0.71197300000000
Al	-3.69245200000000	-1.15103300000000	-0.77056800000000
Si	-5.53460400000000	-2.81614700000000	1.24131100000000
Si	-2.10434700000000	-1.55083200000000	-6.90163500000000
H	11.68201100000000	-5.53663200000000	-0.02821500000000
H	9.20174700000000	-8.10545400000000	2.37332200000000
H	-3.87328600000000	1.40021500000000	-8.24739900000000
H	-5.53835500000000	-0.07944700000000	-7.36210400000000
H	-6.92379800000000	-1.73294700000000	-5.59680800000000
H	-9.60257100000000	-4.23331200000000	-2.90542000000000
H	-4.79979800000000	-4.38576000000000	-7.97480900000000
H	-1.24731700000000	-7.52786700000000	-7.34497900000000
H	-8.45777100000000	2.53259200000000	6.78149400000000
H	-4.20124000000000	6.50559200000000	2.50483200000000
H	-1.80316800000000	9.00632300000000	0.21150600000000
H	0.68439200000000	11.32823500000000	-2.28779600000000
H	-4.94510600000000	5.93197600000000	-3.33612600000000
H	-7.42386400000000	3.36507600000000	-0.93339600000000
H	-9.80607100000000	1.14140600000000	1.46016600000000
H	-3.25255900000000	2.50713300000000	9.44312800000000
H	-0.73677700000000	4.85535500000000	6.91549200000000
H	1.18002900000000	6.84647100000000	4.61624200000000
H	0.02210100000000	-0.85228500000000	9.76306500000000
H	2.66456800000000	1.61419100000000	7.10814700000000
H	4.53903300000000	3.47420200000000	4.87910300000000
H	1.54547200000000	-6.93757900000000	-4.96379800000000
H	4.64367900000000	9.21576000000000	-0.63639300000000
H	-8.66662400000000	0.53615000000000	8.09872400000000
H	-5.67048700000000	-0.35803800000000	8.93677700000000
H	-5.39689700000000	-3.45786300000000	7.75582900000000
H	-2.85180900000000	-3.03946300000000	9.11069100000000
H	-1.21874600000000	-4.11122300000000	7.71386800000000
H	2.58239700000000	5.42848300000000	-6.15377300000000
H	0.23309300000000	8.10316300000000	-5.84617300000000
H	2.80334000000000	11.25729700000000	-3.41206700000000
H	6.06893500000000	7.26957900000000	-3.75705200000000
H	10.01891400000000	4.23819800000000	-2.75127200000000
H	9.88752900000000	0.42411800000000	-3.58475000000000
H	11.17465900000000	0.78784500000000	-1.59486600000000
H	9.90673600000000	3.90381600000000	-0.37702500000000
H	-1.31573500000000	-1.19554000000000	-8.09344200000000
H	-0.60836500000000	-5.49774000000000	-8.45025700000000
H	2.85644700000000	-3.04291800000000	-5.94052500000000
H	-7.30691500000000	-4.15772400000000	3.76046600000000
H	-9.77423600000000	-1.30875400000000	4.08982900000000
H	-9.39493300000000	-2.75342800000000	1.45999600000000
H	-5.76072500000000	-6.40980900000000	1.78972000000000

H	-3.07258100000000	-8.75042600000000	-0.45811200000000
H	-7.01354200000000	-6.69247400000000	-2.77433900000000
H	2.79185400000000	-7.36845900000000	-0.98261400000000
H	4.90518000000000	-9.57697600000000	2.50395300000000
H	5.82910900000000	-9.04673300000000	0.35360400000000
H	-1.33611300000000	-7.77565100000000	0.88453300000000
H	10.67777300000000	-2.01989300000000	2.57366600000000
H	8.83140500000000	-3.95533700000000	4.79373600000000
H	11.59906400000000	-3.97413000000000	-1.84902100000000
H	5.34642000000000	-0.46410700000000	4.52981400000000
H	7.16045900000000	1.34045100000000	2.38220600000000
H	7.33077600000000	6.57881500000000	-0.50344800000000
H	5.01510700000000	-4.86444400000000	-3.23399900000000
H	7.91041200000000	-7.00073900000000	-1.49673300000000
H	2.45084700000000	-6.49934300000000	2.92129100000000
Ga	-3.77001700000000	-0.21885900000000	1.96105200000000
C	-0.49622700000000	-2.18260300000000	3.85637500000000
H	-1.00553000000000	-2.94603800000000	4.45295600000000
H	-0.41468000000000	-1.27335800000000	4.46151300000000
H	0.51628500000000	-2.53057400000000	3.62129300000000
C	-1.26875500000000	-1.90358700000000	2.58271400000000
H	-1.38027800000000	-2.82043900000000	1.98391000000000
H	-0.73687500000000	-1.16942800000000	1.96078600000000
H	-2.99550500000000	-0.05084800000000	3.39074300000000
O	-2.59176800000000	-1.44351400000000	2.89278400000000

I3-GaO-II

Electronic energy (r^2SCAN-3c): -30692.612440389221 E_h
Electronic energy (ωB97X-D4rev/def2-mTZVPP): -30700.84144 E_h
G_{corr} (673.15 K, 1 atm): 0.42404048 E_h
Number of atoms: 249

Cartesian coordinates (Å)

O	1.49176500000000	2.88279700000000	1.03209300000000
O	-1.93652800000000	6.77247900000000	1.43489000000000
O	5.27741800000000	2.07854400000000	0.81239800000000
O	3.61259300000000	3.87907100000000	-0.14231400000000
O	6.09948700000000	4.57743000000000	0.42923400000000
O	1.46077600000000	3.28732700000000	-1.60390600000000
O	1.37641400000000	5.32133400000000	0.04736400000000
O	-1.70595200000000	6.89017300000000	-1.18347600000000
O	0.33244300000000	7.69906900000000	0.41190900000000
O	2.94044500000000	7.47392800000000	-0.04100000000000
Si	4.85385100000000	3.63885100000000	0.88061200000000
Si	1.99413900000000	3.83996400000000	-0.17487200000000
Si	-1.28628900000000	7.62931400000000	0.21067100000000
Si	1.65680200000000	6.79076400000000	0.67007100000000
O	8.96067100000000	-3.83464100000000	2.23525400000000
O	2.00543500000000	6.65546000000000	2.25391000000000
O	7.15365900000000	-2.28014700000000	-0.91378500000000
O	2.51148100000000	4.74914600000000	4.09177200000000
O	6.75683200000000	-4.90462500000000	-0.76269700000000
O	0.05234900000000	5.12037700000000	3.15063300000000
O	5.86316000000000	-3.26769700000000	1.14646800000000
O	4.44959100000000	4.07076700000000	2.39852700000000
O	9.40957200000000	-5.01075400000000	-1.11822900000000

O	5.16277900000000	-0.24802100000000	2.02906800000000
O	7.74151300000000	4.37803900000000	-1.67774400000000
O	10.71195100000000	-3.18479800000000	0.35583000000000
O	5.43779500000000	5.63254400000000	-1.90111900000000
O	4.09975600000000	5.67391100000000	-4.16499700000000
O	8.77015100000000	-1.46102100000000	1.05404500000000
O	3.93108000000000	7.73820900000000	-2.52129600000000
O	1.17840400000000	9.51990800000000	-4.03437800000000
O	8.98919800000000	2.07220300000000	-1.87703100000000
O	6.95573200000000	0.18948100000000	0.11306600000000
O	2.28132000000000	9.55404200000000	-1.55336500000000
O	9.00808100000000	-0.54742600000000	-1.43313800000000
Si	1.43954600000000	5.86224600000000	3.56238300000000
Si	6.12478700000000	-3.44022600000000	-0.43913700000000
Si	3.63657200000000	3.62204100000000	3.73758000000000
Si	8.08764700000000	-5.83603800000000	-0.64682200000000
Si	10.88485200000000	-4.45848900000000	-0.66208700000000
Si	6.67864600000000	5.32690400000000	-0.89774100000000
Si	9.80334900000000	-2.60836000000000	1.57628600000000
Si	4.93223800000000	6.59514400000000	-3.10907800000000
Si	9.21315500000000	3.67371000000000	-1.65751200000000
Si	6.17995100000000	0.85303500000000	1.38346100000000
Si	1.73816000000000	10.45292200000000	-2.81458600000000
Si	9.80869400000000	0.68345200000000	-2.14367200000000
Si	7.96359200000000	-1.01626800000000	-0.29277400000000
Si	3.47713300000000	8.51707600000000	-1.16831400000000
O	2.98509800000000	-0.47248400000000	3.62435200000000
O	0.96666200000000	0.75992400000000	2.48666400000000
O	1.25118600000000	0.88372700000000	5.09329700000000
O	2.97337700000000	2.16052900000000	3.49172200000000
O	-0.41492400000000	1.17918300000000	0.29807000000000
O	-0.81444700000000	2.73541500000000	2.36636000000000
O	-3.75359900000000	4.99138900000000	0.58062200000000
O	-2.52750400000000	4.65895400000000	2.93520000000000
O	-1.16833700000000	3.43748300000000	4.85013600000000
Si	2.05088800000000	0.83938300000000	3.68953300000000
Si	0.30881300000000	1.90164600000000	1.54992600000000
Si	-3.12567100000000	5.74803400000000	1.87688000000000
Si	-1.10118000000000	3.99777400000000	3.33959500000000
O	8.29804200000000	-6.04326500000000	3.50634300000000
O	-3.19147400000000	-3.07487300000000	6.59400900000000
O	6.68962400000000	-7.84608300000000	2.45413800000000
O	-1.30281900000000	-1.56790500000000	7.70213000000000
O	8.26524400000000	-6.34206900000000	0.88187500000000
O	-5.42180700000000	-3.48865600000000	5.25876800000000
O	6.66062100000000	-3.92568900000000	3.58180000000000
O	-5.12809100000000	-1.22140300000000	6.60156400000000
O	-8.66819200000000	0.34760000000000	5.58515000000000
O	4.48447700000000	-2.51292800000000	3.26127600000000
O	-6.43909300000000	1.04620800000000	6.96008900000000
O	4.50030500000000	-5.06517100000000	2.61486800000000
O	-3.88008800000000	0.86047900000000	7.59178200000000
O	-2.24600400000000	2.85250800000000	7.15837500000000
O	0.40768100000000	2.65937000000000	6.87261000000000
O	4.26276200000000	-7.39944600000000	1.38429000000000
O	-1.50056400000000	0.86694100000000	8.79508600000000
O	0.72971400000000	0.08379500000000	7.54356900000000
Si	8.13487400000000	-7.12355300000000	2.29694300000000

Si	-2.11940500000000	-2.97399300000000	7.82689700000000
Si	8.20889400000000	-4.41693500000000	3.56225800000000
Si	-4.79643400000000	-2.81446600000000	6.59517300000000
Si	4.53523600000000	-0.89527500000000	3.38810500000000
Si	-8.07378400000000	1.12928400000000	6.88939000000000
Si	5.36933000000000	-3.70479100000000	2.62461300000000
Si	-5.29135300000000	0.06841100000000	7.57694800000000
Si	-0.92924700000000	3.48825900000000	6.45377400000000
Si	3.45172100000000	-6.09951500000000	1.93574900000000
Si	-2.74185900000000	1.78658100000000	8.28023400000000
Si	1.30378100000000	1.31770200000000	6.65953200000000
Si	5.42496100000000	-8.50837200000000	1.65864400000000
Si	-0.50914400000000	-0.38696700000000	8.48451400000000
O	1.80843500000000	4.27010200000000	-4.04964000000000
O	-0.05758200000000	5.27167700000000	-2.45156700000000
O	-0.61241200000000	7.68098400000000	-3.45741000000000
Si	0.71112100000000	3.91900900000000	-2.90450200000000
Si	-1.19614700000000	6.40514100000000	-2.64480300000000
O	1.25775600000000	-1.72942000000000	-4.49157900000000
O	-3.98024700000000	3.94096100000000	-4.53773300000000
O	3.35041400000000	-2.95909600000000	-3.47260800000000
O	-2.43892300000000	5.80957500000000	-3.49381000000000
O	-3.82704000000000	4.16760400000000	-1.90811800000000
O	1.34232400000000	-4.41032200000000	-4.40647100000000
O	-4.66127100000000	1.77101200000000	-5.90654800000000
O	4.72889200000000	-3.30722900000000	-1.24741400000000
O	-2.54677600000000	1.70783700000000	-4.37288300000000
O	-3.16262700000000	-0.36785900000000	-6.52595100000000
O	-1.10333900000000	-1.79355100000000	-5.64785300000000
O	-0.32098700000000	0.32844900000000	-4.19534800000000
O	-0.36294800000000	2.85648800000000	-3.47512100000000
O	1.78814300000000	6.92624900000000	-4.26131000000000
Si	-3.83482400000000	4.99888200000000	-3.30647200000000
Si	2.20206200000000	-3.04396700000000	-4.62976700000000
Si	-4.04265400000000	2.31599200000000	-4.51799000000000
Si	4.01731600000000	-4.07645400000000	-2.49697400000000
Si	-4.31688800000000	0.66640800000000	-7.06092000000000
Si	-0.29398700000000	-1.29200400000000	-4.33510100000000
Si	-1.21451400000000	1.47917000000000	-3.49186400000000
Si	2.56249800000000	5.56576100000000	-4.70078100000000
Si	0.63283800000000	8.05126700000000	-4.44450800000000
O	-2.78920100000000	-5.59301600000000	-7.10602600000000
O	-4.51222000000000	-4.33178800000000	-5.52934200000000
O	-2.90364000000000	-2.92989800000000	-7.21169200000000
O	-2.85882800000000	0.15588400000000	0.18004200000000
O	-2.47679400000000	2.78044700000000	-0.13729300000000
O	-1.66217200000000	1.01335900000000	-1.99707900000000
O	-5.12058500000000	2.79869300000000	0.00793100000000
Si	-3.75733500000000	-4.29470400000000	-6.96876100000000
Si	-1.85527300000000	1.30804200000000	-0.42230800000000
Si	-3.81305300000000	3.68546800000000	-0.36906400000000
O	-4.99759100000000	1.89576700000000	-3.27602200000000
O	2.82364700000000	-5.05127100000000	-1.97214600000000
O	-5.61460700000000	-2.49785000000000	2.83697100000000
O	-7.38288300000000	-1.79227800000000	4.69307000000000
O	-0.24316100000000	-6.30476600000000	-3.39391000000000
O	-7.99903000000000	0.18320000000000	3.01621100000000
O	-0.38354700000000	-5.57298700000000	-5.95303400000000

O	-0.94933300000000	-1.92564400000000	-3.00198800000000
O	-3.47647500000000	-6.50218900000000	0.74030500000000
O	-0.96172200000000	-4.27128100000000	-1.85300800000000
O	-6.58508000000000	1.03480400000000	-1.37259300000000
O	0.63510000000000	-6.08488200000000	-0.86259700000000
O	-8.34744500000000	-0.62726700000000	0.49442100000000
O	-7.35319900000000	1.77887600000000	1.03315800000000
O	-1.86501700000000	-6.75004100000000	-1.39618300000000
O	-6.53580700000000	-0.21080100000000	-3.69679200000000
O	2.71480400000000	-5.41887500000000	0.65132400000000
O	-9.06629400000000	-2.65950600000000	-1.02784400000000
O	-4.46737900000000	-0.41159200000000	-2.07495600000000
O	-7.00288800000000	-2.91116000000000	0.61086700000000
O	-8.03537200000000	-2.38131800000000	-3.47196200000000
O	-5.38053700000000	-2.61214800000000	-3.73239900000000
O	-7.25686800000000	-4.36576500000000	-1.87870700000000
O	-4.99890800000000	-5.15256300000000	-3.06573400000000
O	-2.89375800000000	-3.62107600000000	-3.58955500000000
O	-5.68706400000000	-6.14852900000000	-0.70698400000000
O	-2.75503100000000	-2.51275400000000	-1.15588000000000
O	-4.75564900000000	-4.20974100000000	0.92938700000000
O	-4.66403200000000	-1.53730400000000	0.68879800000000
Si	-6.47277200000000	-3.01352100000000	4.12365200000000
Si	0.60857700000000	-5.83179800000000	-4.69755100000000
Si	-8.48894200000000	-0.65392500000000	4.32859600000000
Si	-1.23554700000000	-6.06947600000000	-7.25018700000000
Si	-6.63462200000000	2.28853500000000	-0.34257200000000
Si	-2.43009200000000	-7.48767700000000	-0.04821100000000
Si	-0.61070000000000	-5.84290800000000	-1.88103500000000
Si	-8.42205600000000	0.64506600000000	1.50895500000000
Si	-5.62171000000000	0.55541600000000	-2.59519900000000
Si	2.25841400000000	-6.02028600000000	-0.79989000000000
Si	-8.48009700000000	-2.24211800000000	0.42639100000000
Si	-6.70195000000000	-1.75521900000000	-4.16567900000000
Si	-8.51376300000000	-3.44492300000000	-2.34575300000000
Si	-4.44060900000000	-3.91804100000000	-3.96598000000000
Si	-6.25264100000000	-5.61760500000000	-2.13640600000000
Si	-1.91733500000000	-3.08132900000000	-2.39096500000000
Si	-4.94971900000000	-5.82432200000000	0.71023500000000
Al	-3.68219800000000	-1.14562800000000	-0.76112000000000
Si	-5.51948200000000	-2.81997200000000	1.25080400000000
Si	-2.10349800000000	-1.54938000000000	-6.90092500000000
H	11.68201100000000	-5.53663200000000	-0.02821500000000
H	9.20174700000000	-8.10545400000000	2.37332200000000
H	-3.87328600000000	1.40021500000000	-8.24739900000000
H	-5.53835500000000	-0.07944700000000	-7.36210400000000
H	-6.92379800000000	-1.73294700000000	-5.59680800000000
H	-9.60257100000000	-4.23331200000000	-2.90542000000000
H	-4.79979800000000	-4.38576000000000	-7.97480900000000
H	-1.24731700000000	-7.52786700000000	-7.34497900000000
H	-8.45777100000000	2.53259200000000	6.78149400000000
H	-4.20124000000000	6.50559200000000	2.50483200000000
H	-1.80316800000000	9.00632300000000	0.21150600000000
H	0.68439200000000	11.32823500000000	-2.28779600000000
H	-4.94510600000000	5.93197600000000	-3.33612600000000
H	-7.42386400000000	3.36507600000000	-0.93339600000000
H	-9.80607100000000	1.14140600000000	1.46016600000000
H	-3.25255900000000	2.50713300000000	9.44312800000000

H	-0.73677700000000	4.85535500000000	6.91549200000000
H	1.18002900000000	6.84647100000000	4.61624200000000
H	0.02210100000000	-0.85228500000000	9.76306500000000
H	2.66456800000000	1.61419100000000	7.10814700000000
H	4.53903300000000	3.47420200000000	4.87910300000000
H	1.54547200000000	-6.93757900000000	-4.96379800000000
H	4.64367900000000	9.21576000000000	-0.63639300000000
H	-8.66662400000000	0.53615000000000	8.09872400000000
H	-5.67048700000000	-0.35803800000000	8.93677700000000
H	-5.39689700000000	-3.45786300000000	7.75582900000000
H	-2.85180900000000	-3.03946300000000	9.11069100000000
H	-1.21874600000000	-4.11122300000000	7.71386800000000
H	2.58239700000000	5.42848300000000	-6.15377300000000
H	0.23309300000000	8.10316300000000	-5.84617300000000
H	2.80334000000000	11.25729700000000	-3.41206700000000
H	6.06893500000000	7.26957900000000	-3.75705200000000
H	10.01891400000000	4.23819800000000	-2.75127200000000
H	9.88752900000000	0.42411800000000	-3.58475000000000
H	11.17465900000000	0.78784500000000	-1.59486600000000
H	9.90673600000000	3.90381600000000	-0.37702500000000
H	-1.31573500000000	-1.19554000000000	-8.09344200000000
H	-0.60836500000000	-5.49774000000000	-8.45025700000000
H	2.85644700000000	-3.04291800000000	-5.94052500000000
H	-7.30691500000000	-4.15772400000000	3.76046600000000
H	-9.77423600000000	-1.30875400000000	4.08982900000000
H	-9.39493300000000	-2.75342800000000	1.45999600000000
H	-5.76072500000000	-6.40980900000000	1.78972000000000
H	-3.07258100000000	-8.75042600000000	-0.45811200000000
H	-7.01354200000000	-6.69247400000000	-2.77433900000000
H	2.79185400000000	-7.36845900000000	-0.98261400000000
H	4.90518000000000	-9.57697600000000	2.50395300000000
H	5.82910900000000	-9.04673300000000	0.35360400000000
H	-1.33611300000000	-7.77565100000000	0.88453300000000
H	10.67777300000000	-2.01989300000000	2.57366600000000
H	8.83140500000000	-3.95533700000000	4.79373600000000
H	11.59906400000000	-3.97413000000000	-1.84902100000000
H	5.34642000000000	-0.46410700000000	4.52981400000000
H	7.16045900000000	1.34045100000000	2.38220600000000
H	7.33077600000000	6.57881500000000	-0.50344800000000
H	5.01510700000000	-4.86444400000000	-3.23399900000000
H	7.91041200000000	-7.00073900000000	-1.49673300000000
H	2.45084700000000	-6.49934300000000	2.92129100000000
Ga	-3.77744300000000	-0.18579500000000	1.94410000000000
C	-0.77140600000000	-2.42550200000000	3.61649900000000
H	-1.39822100000000	-2.94220400000000	4.35009100000000
H	-0.28169200000000	-1.58246700000000	4.11469600000000
H	0.00024400000000	-3.11570600000000	3.25724700000000
C	-1.61621400000000	-1.92955100000000	2.45970100000000
H	-2.12052700000000	-2.77817500000000	1.96760800000000
H	-0.97468600000000	-1.44650700000000	1.70602900000000
H	-4.76375100000000	0.90611600000000	2.35853600000000
O	-2.57767300000000	-0.99819800000000	2.96879600000000

TS3-GaO-II

Electronic energy (r^{2}SCAN-3c): -30692.514164557713 E_h
Electronic energy (ωB97X-D4rev/def2-mTZVPP): -30700.73131 E_h
G_{corr} (673.15 K, 1 atm): 0.41050833 E_h
Number of atoms: 249

Cartesian coordinates (Å)

O	1.48886100000000	2.88178500000000	1.03241300000000
O	-1.93532300000000	6.77384200000000	1.43634100000000
O	5.27658300000000	2.07871800000000	0.81308500000000
O	3.61016700000000	3.87764500000000	-0.14140400000000
O	6.09860900000000	4.57709700000000	0.42781800000000
O	1.45817400000000	3.28650200000000	-1.60376800000000
O	1.37515500000000	5.32084200000000	0.04826800000000
O	-1.70486000000000	6.88949200000000	-1.18308300000000
O	0.33282500000000	7.69986700000000	0.41182900000000
O	2.94058500000000	7.47297400000000	-0.04167000000000
Si	4.85291100000000	3.63929300000000	0.87963000000000
Si	1.99133600000000	3.83907500000000	-0.17502300000000
Si	-1.28617700000000	7.62944100000000	0.21063000000000
Si	1.65639000000000	6.79080900000000	0.66938400000000
O	8.96033000000000	-3.83432400000000	2.23520500000000
O	2.00526500000000	6.65546200000000	2.25351800000000
O	7.15360900000000	-2.28002900000000	-0.91366800000000
O	2.51291300000000	4.75121300000000	4.09261000000000
O	6.75672100000000	-4.90456000000000	-0.76222300000000
O	0.05370900000000	5.11791300000000	3.15146000000000
O	5.86218200000000	-3.26703700000000	1.14618500000000
O	4.45082600000000	4.07232500000000	2.39816500000000
O	9.40946300000000	-5.01061100000000	-1.11819300000000
O	5.16252400000000	-0.24745500000000	2.02887900000000
O	7.74157000000000	4.37813500000000	-1.67795100000000
O	10.71172100000000	-3.18473400000000	0.35576400000000
O	5.43751100000000	5.63252200000000	-1.90177700000000
O	4.10165700000000	5.67575700000000	-4.16735200000000
O	8.76992600000000	-1.46078800000000	1.05418200000000
O	3.93132100000000	7.73849500000000	-2.52141500000000
O	1.17875300000000	9.52034900000000	-4.03486300000000
O	8.98915100000000	2.07222800000000	-1.87677100000000
O	6.95547500000000	0.18952900000000	0.11292300000000
O	2.28128100000000	9.55402100000000	-1.55337900000000
O	9.00813700000000	-0.54728000000000	-1.43307400000000
Si	1.43947700000000	5.86285800000000	3.56194900000000
Si	6.12457500000000	-3.44011500000000	-0.43949200000000
Si	3.63791900000000	3.62528700000000	3.73713400000000
Si	8.08764200000000	-5.83600900000000	-0.64685100000000
Si	10.88485100000000	-4.45849300000000	-0.66214700000000
Si	6.67839800000000	5.32720800000000	-0.89867100000000
Si	9.80330100000000	-2.60818400000000	1.57619600000000
Si	4.93239800000000	6.59577200000000	-3.10965300000000
Si	9.21316700000000	3.67384000000000	-1.65755900000000
Si	6.18015000000000	0.85369100000000	1.38297600000000
Si	1.73808100000000	10.45296200000000	-2.81475100000000
Si	9.80876000000000	0.68365600000000	-2.14375800000000
Si	7.96370100000000	-1.01616800000000	-0.29290800000000
Si	3.47703200000000	8.51709000000000	-1.16847000000000
O	2.98513900000000	-0.47182600000000	3.62330000000000

O	0.96965900000000	0.75681800000000	2.48394500000000
O	1.25244000000000	0.88211300000000	5.09154800000000
O	2.97353700000000	2.16356700000000	3.49052100000000
O	-0.41724800000000	1.17488900000000	0.29924800000000
O	-0.81347000000000	2.73068200000000	2.37228500000000
O	-3.75145000000000	4.99442900000000	0.57799600000000
O	-2.52673400000000	4.65897400000000	2.93330900000000
O	-1.17131000000000	3.43772700000000	4.85054500000000
Si	2.05374500000000	0.84263700000000	3.68774400000000
Si	0.30546500000000	1.89981600000000	1.54810700000000
Si	-3.12559400000000	5.74887500000000	1.87532800000000
Si	-1.10193900000000	3.99738800000000	3.33916500000000
O	8.29739300000000	-6.04303300000000	3.50623500000000
O	-3.19183200000000	-3.07422700000000	6.59238000000000
O	6.68941000000000	-7.84589300000000	2.45403200000000
O	-1.30226100000000	-1.56870800000000	7.70087100000000
O	8.26526100000000	-6.34196100000000	0.88190300000000
O	-5.42258700000000	-3.48744600000000	5.25775200000000
O	6.66043800000000	-3.92486700000000	3.58172300000000
O	-5.12375700000000	-1.22064400000000	6.60083100000000
O	-8.66942300000000	0.34570500000000	5.58686300000000
O	4.48383200000000	-2.51284200000000	3.26090500000000
O	-6.43936300000000	1.04570100000000	6.96004400000000
O	4.50021100000000	-5.06515700000000	2.61480400000000
O	-3.87952700000000	0.85992800000000	7.59113400000000
O	-2.24565200000000	2.85179800000000	7.15867700000000
O	0.40729000000000	2.65878000000000	6.87039700000000
O	4.26258100000000	-7.39924200000000	1.38414100000000
O	-1.50042900000000	0.86630800000000	8.79435700000000
O	0.72818800000000	0.08202000000000	7.53938300000000
Si	8.13476600000000	-7.12356700000000	2.29685300000000
Si	-2.12081000000000	-2.97409500000000	7.82604800000000
Si	8.20877300000000	-4.41674500000000	3.56217100000000
Si	-4.79829400000000	-2.81537100000000	6.59420300000000
Si	4.53627200000000	-0.89554600000000	3.38752500000000
Si	-8.07483400000000	1.12891700000000	6.88891700000000
Si	5.36926800000000	-3.70501100000000	2.62423800000000
Si	-5.29203800000000	0.06859000000000	7.57678900000000
Si	-0.93003100000000	3.48888500000000	6.45323500000000
Si	3.45138800000000	-6.09942100000000	1.93560000000000
Si	-2.74222100000000	1.78646900000000	8.28047600000000
Si	1.30380700000000	1.31790600000000	6.65830700000000
Si	5.42479700000000	-8.50832400000000	1.65852900000000
Si	-0.50912700000000	-0.38728100000000	8.48400300000000
O	1.81314200000000	4.27129100000000	-4.04710900000000
O	-0.05725600000000	5.27166000000000	-2.45313600000000
O	-0.61129700000000	7.68159500000000	-3.45637500000000
Si	0.71166000000000	3.91920100000000	-2.90637800000000
Si	-1.19621600000000	6.40509200000000	-2.64521200000000
O	1.25775700000000	-1.72867300000000	-4.49386400000000
O	-3.97953000000000	3.94243600000000	-4.53938600000000
O	3.34998800000000	-2.95821000000000	-3.47243600000000
O	-2.43914200000000	5.81162000000000	-3.49483500000000
O	-3.82500900000000	4.16825900000000	-1.90937700000000
O	1.34371300000000	-4.41031300000000	-4.40655600000000
O	-4.66072100000000	1.77241800000000	-5.90817000000000
O	4.72875400000000	-3.30693100000000	-1.24771200000000
O	-2.54509000000000	1.71053700000000	-4.37646600000000

O	-3.16296100000000	-0.36829700000000	-6.52626800000000
O	-1.10518200000000	-1.79568900000000	-5.64699500000000
O	-0.32141700000000	0.32841300000000	-4.19890700000000
O	-0.35864400000000	2.85785900000000	-3.48418900000000
O	1.78916300000000	6.92720700000000	-4.26177300000000
Si	-3.83464400000000	4.99922800000000	-3.30721300000000
Si	2.20172200000000	-3.04304100000000	-4.63038900000000
Si	-4.04182000000000	2.31723700000000	-4.51951300000000
Si	4.01708200000000	-4.07594300000000	-2.49774400000000
Si	-4.31697800000000	0.66652500000000	-7.06106700000000
Si	-0.29463800000000	-1.29199200000000	-4.33567500000000
Si	-1.21280300000000	1.48152400000000	-3.49561100000000
Si	2.56370300000000	5.56685500000000	-4.70109500000000
Si	0.63300900000000	8.05151400000000	-4.44451400000000
O	-2.78936900000000	-5.59305500000000	-7.10659100000000
O	-4.50757200000000	-4.32923700000000	-5.52760200000000
O	-2.90266900000000	-2.93010300000000	-7.21494400000000
O	-2.86787600000000	0.16534000000000	0.17029600000000
O	-2.46962800000000	2.78692100000000	-0.13969900000000
O	-1.65490700000000	1.02046400000000	-1.99963200000000
O	-5.11884100000000	2.80269000000000	0.00819700000000
Si	-3.75686500000000	-4.29391700000000	-6.96877400000000
Si	-1.85869500000000	1.30828400000000	-0.42419800000000
Si	-3.81016400000000	3.68606600000000	-0.36984400000000
O	-4.99650000000000	1.89642400000000	-3.27828400000000
O	2.82396100000000	-5.05091900000000	-1.97186400000000
O	-5.61400000000000	-2.49664900000000	2.83685100000000
O	-7.38279500000000	-1.79160900000000	4.69345500000000
O	-0.24374100000000	-6.30038700000000	-3.39294600000000
O	-8.00120800000000	0.18777200000000	3.01854900000000
O	-0.38342100000000	-5.57291500000000	-5.95318500000000
O	-0.94444100000000	-1.92688000000000	-3.00073000000000
O	-3.47606200000000	-6.50157800000000	0.74147300000000
O	-0.96609000000000	-4.26883100000000	-1.84597600000000
O	-6.57946900000000	1.03527200000000	-1.37293200000000
O	0.63440600000000	-6.08137400000000	-0.86167700000000
O	-8.34162700000000	-0.63052500000000	0.49816700000000
O	-7.35337300000000	1.77983000000000	1.03174900000000
O	-1.86445300000000	-6.75019300000000	-1.39548600000000
O	-6.53877200000000	-0.20841900000000	-3.69832100000000
O	2.71412200000000	-5.41844700000000	0.65144100000000
O	-9.06440000000000	-2.65894500000000	-1.02683200000000
O	-4.46471200000000	-0.41606200000000	-2.08707400000000
O	-7.00673000000000	-2.92529700000000	0.61904100000000
O	-8.03893100000000	-2.37919100000000	-3.47262800000000
O	-5.38543800000000	-2.61293400000000	-3.73222900000000
O	-7.25637600000000	-4.36471000000000	-1.88169000000000
O	-4.99852300000000	-5.15322200000000	-3.06684700000000
O	-2.89692700000000	-3.61451800000000	-3.58154300000000
O	-5.68582500000000	-6.14625400000000	-0.70692200000000
O	-2.74819400000000	-2.50106100000000	-1.14667600000000
O	-4.75167700000000	-4.20921300000000	0.93350100000000
O	-4.68268800000000	-1.53256400000000	0.67704400000000
Si	-6.47350600000000	-3.01223800000000	4.12015500000000
Si	0.60855200000000	-5.83096000000000	-4.69767800000000
Si	-8.48919100000000	-0.65343100000000	4.32775400000000
Si	-1.23588600000000	-6.06935500000000	-7.25036000000000
Si	-6.63169000000000	2.28872700000000	-0.34273900000000

Si	-2.43112800000000	-7.48739400000000	-0.04791900000000
Si	-0.61253200000000	-5.83974300000000	-1.88020300000000
Si	-8.42038600000000	0.64499600000000	1.50834600000000
Si	-5.61949800000000	0.55474000000000	-2.59781300000000
Si	2.25788400000000	-6.01998900000000	-0.80026000000000
Si	-8.47718700000000	-2.24545900000000	0.42877900000000
Si	-6.70492500000000	-1.75326300000000	-4.16544100000000
Si	-8.51430700000000	-3.44360900000000	-2.34571600000000
Si	-4.44163900000000	-3.91547800000000	-3.96374100000000
Si	-6.25246200000000	-5.61683600000000	-2.13678000000000
Si	-1.91915400000000	-3.07455300000000	-2.38432600000000
Si	-4.94995900000000	-5.82127000000000	0.71119500000000
Al	-3.69143100000000	-1.13871100000000	-0.75725600000000
Si	-5.51768300000000	-2.81672800000000	1.24830500000000
Si	-2.10374800000000	-1.54952900000000	-6.90116400000000
H	11.68201100000000	-5.53663200000000	-0.02821500000000
H	9.20174700000000	-8.10545400000000	2.37332200000000
H	-3.87328600000000	1.40021500000000	-8.24739900000000
H	-5.53835500000000	-0.07944700000000	-7.36210400000000
H	-6.92379800000000	-1.73294700000000	-5.59680800000000
H	-9.60257100000000	-4.23331200000000	-2.90542000000000
H	-4.79979800000000	-4.38576000000000	-7.97480900000000
H	-1.24731700000000	-7.52786700000000	-7.34497900000000
H	-8.45777100000000	2.53259200000000	6.78149400000000
H	-4.20124000000000	6.50559200000000	2.50483200000000
H	-1.80316800000000	9.00632300000000	0.21150600000000
H	0.68439200000000	11.32823500000000	-2.28779600000000
H	-4.94510600000000	5.93197600000000	-3.33612600000000
H	-7.42386400000000	3.36507600000000	-0.93339600000000
H	-9.80607100000000	1.14140600000000	1.46016600000000
H	-3.25255900000000	2.50713300000000	9.44312800000000
H	-0.73677700000000	4.85535500000000	6.91549200000000
H	1.18002900000000	6.84647100000000	4.61624200000000
H	0.02210100000000	-0.85228500000000	9.76306500000000
H	2.66456800000000	1.61419100000000	7.10814700000000
H	4.53903300000000	3.47420200000000	4.87910300000000
H	1.54547200000000	-6.93757900000000	-4.96379800000000
H	4.64367900000000	9.21576000000000	-0.63639300000000
H	-8.66662400000000	0.53615000000000	8.09872400000000
H	-5.67048700000000	-0.35803800000000	8.93677700000000
H	-5.39689700000000	-3.45786200000000	7.75582900000000
H	-2.85180900000000	-3.03946300000000	9.11069100000000
H	-1.21874600000000	-4.11122300000000	7.71386800000000
H	2.58239700000000	5.42848300000000	-6.15377300000000
H	0.23309300000000	8.10316300000000	-5.84617300000000
H	2.80334000000000	11.25729700000000	-3.41206700000000
H	6.06893500000000	7.26957900000000	-3.75705200000000
H	10.01891400000000	4.23819800000000	-2.75127200000000
H	9.88752900000000	0.42411800000000	-3.58475000000000
H	11.17465900000000	0.78784500000000	-1.59486600000000
H	9.90673600000000	3.90381600000000	-0.37702500000000
H	-1.31573500000000	-1.19554000000000	-8.09344200000000
H	-0.60836500000000	-5.49774000000000	-8.45025700000000
H	2.85644700000000	-3.04291800000000	-5.94052500000000
H	-7.30691500000000	-4.15772400000000	3.76046600000000
H	-9.77423600000000	-1.30875400000000	4.08982900000000
H	-9.39493300000000	-2.75342800000000	1.45999600000000
H	-5.76072500000000	-6.40980900000000	1.78972000000000

H	-3.07258100000000	-8.75042600000000	-0.45811200000000
H	-7.01354200000000	-6.69247400000000	-2.77433900000000
H	2.79185400000000	-7.36845900000000	-0.98261400000000
H	4.90518000000000	-9.57697600000000	2.50395300000000
H	5.82910900000000	-9.04673300000000	0.35360400000000
H	-1.33611300000000	-7.77565100000000	0.88453300000000
H	10.67777300000000	-2.01989300000000	2.57366600000000
H	8.83140500000000	-3.95533700000000	4.79373600000000
H	11.59906400000000	-3.97413000000000	-1.84902100000000
H	5.34642000000000	-0.46410700000000	4.52981400000000
H	7.16045900000000	1.34045100000000	2.38220600000000
H	7.33077600000000	6.57881500000000	-0.50344800000000
H	5.01510700000000	-4.86444400000000	-3.23399900000000
H	7.91041200000000	-7.00073900000000	-1.49673300000000
H	2.45084700000000	-6.49934300000000	2.92129100000000
Ga	-3.71892000000000	-0.22592700000000	1.97613700000000
C	-0.70324900000000	-2.36320100000000	3.95275200000000
H	-0.99656900000000	-3.28073900000000	4.45728800000000
H	-1.79230500000000	-1.57181900000000	3.85484700000000
H	0.10199300000000	-1.79292100000000	4.41082800000000
C	-0.86129000000000	-2.29474900000000	2.55587600000000
H	-1.37657600000000	-3.07585900000000	2.00506100000000
H	-0.29246900000000	-1.58590600000000	1.96282300000000
H	-4.72661100000000	0.86504200000000	2.36658400000000
O	-2.44744900000000	-1.13869500000000	2.80904400000000

I4-GaO-II

Electronic energy (r^2SCAN-3c): -30692.588876951984 E_h
Electronic energy (ωB97X-D4rev/def2-mTZVPP): -30700.81544 E_h
G_{corr} (673.15 K, 1 atm): 0.41121510 E_h
Number of atoms: 249

Cartesian coordinates (Å)

O	1.49059500000000	2.89257300000000	1.03605400000000
O	-1.93708300000000	6.77082700000000	1.43494700000000
O	5.27763400000000	2.07904700000000	0.81118200000000
O	3.61237200000000	3.88012900000000	-0.14317100000000
O	6.09852400000000	4.57855200000000	0.43022200000000
O	1.46010200000000	3.28761800000000	-1.60371800000000
O	1.37724600000000	5.32551100000000	0.04229100000000
O	-1.70463200000000	6.88984400000000	-1.18239600000000
O	0.33193400000000	7.70103800000000	0.41472300000000
O	2.94085200000000	7.47731600000000	-0.03863100000000
Si	4.85303200000000	3.63900900000000	0.88023900000000
Si	1.99383700000000	3.84292500000000	-0.17602500000000
Si	-1.28636100000000	7.62929600000000	0.21149400000000
Si	1.65686600000000	6.79269500000000	0.66972600000000
O	8.96069600000000	-3.83452100000000	2.23547900000000
O	2.00465000000000	6.65426200000000	2.25352200000000
O	7.15145900000000	-2.27964700000000	-0.91309600000000
O	2.51617900000000	4.75469600000000	4.09637100000000
O	6.75664000000000	-4.90383200000000	-0.76069300000000
O	0.05653300000000	5.11364700000000	3.15268900000000
O	5.85866100000000	-3.26505700000000	1.14629800000000
O	4.44730000000000	4.06882700000000	2.39840900000000
O	9.40908300000000	-5.01067600000000	-1.11809100000000

O	5.16430100000000	-0.24746800000000	2.02656200000000
O	7.74136000000000	4.37724200000000	-1.67659900000000
O	10.71205700000000	-3.18482100000000	0.35582900000000
O	5.43821900000000	5.63228700000000	-1.90107200000000
O	4.09865800000000	5.67261100000000	-4.16374200000000
O	8.77005900000000	-1.46111700000000	1.05367300000000
O	3.93064500000000	7.73729900000000	-2.52063000000000
O	1.17893900000000	9.51982200000000	-4.03494100000000
O	8.98939100000000	2.07172000000000	-1.87683300000000
O	6.95796200000000	0.19136600000000	0.11159400000000
O	2.28127300000000	9.55424100000000	-1.55378400000000
O	9.00837300000000	-0.54872500000000	-1.43392100000000
Si	1.44138700000000	5.86205500000000	3.56309700000000
Si	6.12210700000000	-3.44025000000000	-0.43883700000000
Si	3.63572300000000	3.62265400000000	3.73910900000000
Si	8.08690900000000	-5.83607100000000	-0.64616800000000
Si	10.88435000000000	-4.45872200000000	-0.66218000000000
Si	6.67869800000000	5.32670500000000	-0.89722300000000
Si	9.80325400000000	-2.60859400000000	1.57623500000000
Si	4.93202700000000	6.59449500000000	-3.10898900000000
Si	9.21326400000000	3.67312700000000	-1.65741600000000
Si	6.18155400000000	0.85406400000000	1.38144600000000
Si	1.73804900000000	10.45295300000000	-2.81503100000000
Si	9.80869700000000	0.68269200000000	-2.14363100000000
Si	7.96356500000000	-1.01708400000000	-0.29337400000000
Si	3.47732300000000	8.51772400000000	-1.16847200000000
O	2.99389800000000	-0.46398200000000	3.63265300000000
O	0.97244500000000	0.76100100000000	2.48269900000000
O	1.24260600000000	0.88309000000000	5.09327200000000
O	2.96453200000000	2.16501200000000	3.49979100000000
O	-0.40544600000000	1.18521500000000	0.29096600000000
O	-0.81546200000000	2.72638200000000	2.37014400000000
O	-3.74948800000000	4.98156400000000	0.58310800000000
O	-2.52481000000000	4.65832900000000	2.93744700000000
O	-1.17017600000000	3.43169800000000	4.85013700000000
Si	2.04789100000000	0.83778400000000	3.69107900000000
Si	0.31337700000000	1.90112100000000	1.54731200000000
Si	-3.12531700000000	5.74519100000000	1.87682800000000
Si	-1.09919300000000	3.99388200000000	3.33842600000000
O	8.29347800000000	-6.04188200000000	3.50595700000000
O	-3.18889300000000	-3.06141100000000	6.58832800000000
O	6.69067200000000	-7.84933900000000	2.45407900000000
O	-1.30312400000000	-1.56557700000000	7.69954700000000
O	8.26587400000000	-6.34217000000000	0.88221400000000
O	-5.41860800000000	-3.48201000000000	5.25529800000000
O	6.66026400000000	-3.91821700000000	3.58124900000000
O	-5.12230800000000	-1.21795700000000	6.59895300000000
O	-8.66841300000000	0.34665900000000	5.58574500000000
O	4.48096700000000	-2.50972200000000	3.26020000000000
O	-6.43963300000000	1.04623500000000	6.95988000000000
O	4.49996400000000	-5.06353500000000	2.62053900000000
O	-3.87887800000000	0.86080700000000	7.58824800000000
O	-2.24516400000000	2.85006400000000	7.15655200000000
O	0.40746200000000	2.65924900000000	6.87526700000000
O	4.26457400000000	-7.39362200000000	1.38787600000000
O	-1.50306800000000	0.86490000000000	8.79647900000000
O	0.72936700000000	0.08583600000000	7.54510200000000
Si	8.13365800000000	-7.12414300000000	2.29711700000000

Si	-2.11866400000000	-2.97266100000000	7.82715800000000
Si	8.20687300000000	-4.41551500000000	3.56210300000000
Si	-4.79820100000000	-2.81386900000000	6.59541800000000
Si	4.53934500000000	-0.89218300000000	3.38726900000000
Si	-8.07559200000000	1.12930600000000	6.88895800000000
Si	5.36657100000000	-3.70395700000000	2.62462700000000
Si	-5.29216200000000	0.07081100000000	7.57777200000000
Si	-0.92757000000000	3.48853500000000	6.45302800000000
Si	3.44702900000000	-6.09323900000000	1.93729100000000
Si	-2.74122800000000	1.78762000000000	8.28069800000000
Si	1.30455100000000	1.31815200000000	6.65766700000000
Si	5.42232400000000	-8.50626600000000	1.65840600000000
Si	-0.50748400000000	-0.38558300000000	8.48553600000000
O	1.80604400000000	4.26897000000000	-4.05052500000000
O	-0.05975300000000	5.26978300000000	-2.45268800000000
O	-0.61131300000000	7.68142000000000	-3.45602800000000
Si	0.70907000000000	3.91682400000000	-2.90500900000000
Si	-1.19610200000000	6.40534700000000	-2.64449200000000
O	1.25502100000000	-1.73100400000000	-4.48652000000000
O	-3.97794200000000	3.94423600000000	-4.53887600000000
O	3.35108600000000	-2.95890400000000	-3.47404300000000
O	-2.44069300000000	5.81330600000000	-3.49271600000000
O	-3.82088900000000	4.16452500000000	-1.90697000000000
O	1.34167000000000	-4.41049900000000	-4.40773100000000
O	-4.66085600000000	1.77422300000000	-5.90972500000000
O	4.72736800000000	-3.30740500000000	-1.24851900000000
O	-2.55319400000000	1.70701600000000	-4.36732800000000
O	-3.16140900000000	-0.36631100000000	-6.52532000000000
O	-1.10134800000000	-1.79563100000000	-5.65113600000000
O	-0.32493700000000	0.32753400000000	-4.19999800000000
O	-0.36523400000000	2.85357700000000	-3.47353600000000
O	1.78794400000000	6.92558100000000	-4.26115200000000
Si	-3.83425500000000	4.99875100000000	-3.30465000000000
Si	2.20114700000000	-3.04422300000000	-4.62942100000000
Si	-4.04638600000000	2.31950800000000	-4.51963000000000
Si	4.01605000000000	-4.07752500000000	-2.49813900000000
Si	-4.31642900000000	0.66650900000000	-7.06123800000000
Si	-0.29728500000000	-1.29375200000000	-4.33534200000000
Si	-1.21740200000000	1.47607600000000	-3.49217800000000
Si	2.56155700000000	5.56449900000000	-4.70050900000000
Si	0.63311600000000	8.05098000000000	-4.44445700000000
O	-2.78893000000000	-5.59202900000000	-7.10375200000000
O	-4.52435200000000	-4.33414700000000	-5.53464600000000
O	-2.90756700000000	-2.92875500000000	-7.20947200000000
O	-2.83916300000000	0.12886500000000	0.17834500000000
O	-2.48166900000000	2.76277400000000	-0.13512600000000
O	-1.66139200000000	1.00516100000000	-1.99838800000000
O	-5.12135000000000	2.79318900000000	0.00729400000000
Si	-3.75928200000000	-4.29529700000000	-6.96877300000000
Si	-1.84908200000000	1.29462400000000	-0.42276900000000
Si	-3.81099100000000	3.67619200000000	-0.36900300000000
O	-5.01256400000000	1.90650600000000	-3.28361900000000
O	2.82218500000000	-5.05314400000000	-1.97786200000000
O	-5.61416300000000	-2.48929500000000	2.83651300000000
O	-7.38281500000000	-1.79180400000000	4.69508600000000
O	-0.24221000000000	-6.30652000000000	-3.39285300000000
O	-7.99933700000000	0.18410900000000	3.01727800000000
O	-0.38322800000000	-5.57402700000000	-5.95252400000000

O	-0.96170500000000	-1.92340700000000	-3.00474300000000
O	-3.47816100000000	-6.50530900000000	0.74117700000000
O	-0.96306900000000	-4.27033300000000	-1.86038400000000
O	-6.59368600000000	1.03673900000000	-1.37784500000000
O	0.63622800000000	-6.08210800000000	-0.86262500000000
O	-8.34881900000000	-0.62845600000000	0.49549100000000
O	-7.34912400000000	1.77511200000000	1.03468500000000
O	-1.86496900000000	-6.74822900000000	-1.39527800000000
O	-6.53670000000000	-0.21178200000000	-3.69885300000000
O	2.72683200000000	-5.41444700000000	0.64361400000000
O	-9.06435000000000	-2.65964000000000	-1.02808000000000
O	-4.46302600000000	-0.39088100000000	-2.07942300000000
O	-7.00308000000000	-2.91225900000000	0.61623000000000
O	-8.03159500000000	-2.38422700000000	-3.47217600000000
O	-5.37469100000000	-2.61046800000000	-3.73245900000000
O	-7.25680900000000	-4.36733400000000	-1.87660900000000
O	-4.99999900000000	-5.15250300000000	-3.06720200000000
O	-2.88881700000000	-3.63371900000000	-3.61086300000000
O	-5.68616700000000	-6.14888900000000	-0.70687600000000
O	-2.78801000000000	-2.53051000000000	-1.18134800000000
O	-4.75810600000000	-4.20971900000000	0.93014300000000
O	-4.67654000000000	-1.52702500000000	0.67895300000000
Si	-6.47387300000000	-3.01121200000000	4.11915000000000
Si	0.60845300000000	-5.83271800000000	-4.69630000000000
Si	-8.48958100000000	-0.65381000000000	4.32769800000000
Si	-1.23538600000000	-6.06929200000000	-7.24937200000000
Si	-6.63612900000000	2.28687500000000	-0.34368300000000
Si	-2.42941000000000	-7.48744800000000	-0.04792400000000
Si	-0.60973400000000	-5.84188900000000	-1.88003100000000
Si	-8.42171100000000	0.64423600000000	1.50823400000000
Si	-5.62709400000000	0.56305700000000	-2.60028500000000
Si	2.25981600000000	-6.01909200000000	-0.80038200000000
Si	-8.47904000000000	-2.24369200000000	0.42664000000000
Si	-6.69862800000000	-1.75707100000000	-4.16624300000000
Si	-8.51223900000000	-3.44639500000000	-2.34575700000000
Si	-4.44106400000000	-3.92149200000000	-3.97152500000000
Si	-6.25163500000000	-5.61852100000000	-2.13589600000000
Si	-1.92535500000000	-3.08878000000000	-2.40308200000000
Si	-4.94864400000000	-5.82422200000000	0.71075100000000
Al	-3.68806100000000	-1.15064500000000	-0.77517000000000
Si	-5.51560100000000	-2.81873600000000	1.24859400000000
Si	-2.10406000000000	-1.54978100000000	-6.90120800000000
H	11.68201100000000	-5.53663200000000	-0.02821500000000
H	9.20174700000000	-8.10545400000000	2.37332200000000
H	-3.87328600000000	1.40021500000000	-8.24739900000000
H	-5.53835500000000	-0.07944700000000	-7.36210400000000
H	-6.92379800000000	-1.73294700000000	-5.59680800000000
H	-9.60257100000000	-4.23331200000000	-2.90542000000000
H	-4.79979800000000	-4.38576000000000	-7.97480900000000
H	-1.24731700000000	-7.52786700000000	-7.34497900000000
H	-8.45777100000000	2.53259200000000	6.78149400000000
H	-4.20124000000000	6.50559200000000	2.50483200000000
H	-1.80316800000000	9.00632300000000	0.21150600000000
H	0.68439200000000	11.32823500000000	-2.28779600000000
H	-4.94510600000000	5.93197600000000	-3.33612600000000
H	-7.42386400000000	3.36507600000000	-0.93339600000000
H	-9.80607100000000	1.14140600000000	1.46016600000000
H	-3.25255900000000	2.50713300000000	9.44312800000000

H	-0.73677700000000	4.85535500000000	6.91549200000000
H	1.18002900000000	6.84647100000000	4.61624200000000
H	0.02210100000000	-0.85228500000000	9.76306500000000
H	2.66456800000000	1.61419100000000	7.10814700000000
H	4.53903300000000	3.47420200000000	4.87910300000000
H	1.54547200000000	-6.93757900000000	-4.96379800000000
H	4.64367900000000	9.21576000000000	-0.63639300000000
H	-8.66662400000000	0.53615000000000	8.09872400000000
H	-5.67048700000000	-0.35803800000000	8.93677700000000
H	-5.39689700000000	-3.45786200000000	7.75582900000000
H	-2.85180900000000	-3.03946300000000	9.11069100000000
H	-1.21874600000000	-4.11122300000000	7.71386800000000
H	2.58239700000000	5.42848300000000	-6.15377300000000
H	0.23309300000000	8.10316300000000	-5.84617300000000
H	2.80334000000000	11.25729700000000	-3.41206700000000
H	6.06893500000000	7.26957900000000	-3.75705200000000
H	10.01891400000000	4.23819800000000	-2.75127200000000
H	9.88752900000000	0.42411800000000	-3.58475000000000
H	11.17465900000000	0.78784500000000	-1.59486600000000
H	9.90673600000000	3.90381600000000	-0.37702500000000
H	-1.31573500000000	-1.19554000000000	-8.09344200000000
H	-0.60836500000000	-5.49774000000000	-8.45025700000000
H	2.85644700000000	-3.04291800000000	-5.94052500000000
H	-7.30691500000000	-4.15772400000000	3.76046600000000
H	-9.77423600000000	-1.30875400000000	4.08982900000000
H	-9.39493300000000	-2.75342800000000	1.45999600000000
H	-5.76072500000000	-6.40980900000000	1.78972000000000
H	-3.07258100000000	-8.75042600000000	-0.45811200000000
H	-7.01354200000000	-6.69247400000000	-2.77433900000000
H	2.79185400000000	-7.36845900000000	-0.98261400000000
H	4.90518000000000	-9.57697600000000	2.50395300000000
H	5.82910900000000	-9.04673300000000	0.35360400000000
H	-1.33611300000000	-7.77565100000000	0.88453300000000
H	10.67777300000000	-2.01989300000000	2.57366600000000
H	8.83140500000000	-3.95533700000000	4.79373600000000
H	11.59906400000000	-3.97413000000000	-1.84902100000000
H	5.34642000000000	-0.46410700000000	4.52981400000000
H	7.16045900000000	1.34045100000000	2.38220600000000
H	7.33077600000000	6.57881500000000	-0.50344800000000
H	5.01510700000000	-4.86444400000000	-3.23399900000000
H	7.91041200000000	-7.00073900000000	-1.49673300000000
H	2.45084700000000	-6.49934300000000	2.92129100000000
Ga	-3.67450900000000	-0.29533700000000	1.94108600000000
C	0.93532400000000	-3.37891300000000	3.70018200000000
H	0.12319400000000	-3.88648400000000	4.21556300000000
H	-2.33545200000000	-1.34601600000000	3.60119600000000
H	1.88640800000000	-3.30759900000000	4.22231900000000
C	0.77072000000000	-2.86624600000000	2.48638500000000
H	-0.18256600000000	-2.91745300000000	1.96775100000000
H	1.58403200000000	-2.35821800000000	1.97492200000000
H	-4.54143800000000	0.84704900000000	2.48407800000000
O	-2.44945100000000	-1.38264400000000	2.64630100000000

TS1-GaO-II-concerted

Electronic energy (r^{2}SCAN-3c): -30692.472139338181 E_h
Electronic energy (ωB97X-D4rev/def2-mTZVPP): -30700.6840742368 E_h
G_{corr} (673.15 K, 1 atm): 0.41856759 E_h
Number of atoms: 249

Cartesian coordinates (Å)

O	1.49997200000000	2.88445000000000	1.03295000000000
O	-1.93588600000000	6.77436400000000	1.43643100000000
O	5.27797500000000	2.07963100000000	0.81157100000000
O	3.61579100000000	3.88348200000000	-0.14556400000000
O	6.10100400000000	4.57803900000000	0.43009500000000
O	1.46100600000000	3.28882500000000	-1.60393600000000
O	1.37742500000000	5.32242400000000	0.04664900000000
O	-1.70358600000000	6.88899500000000	-1.18182000000000
O	0.33430800000000	7.69937700000000	0.41506100000000
O	2.94156900000000	7.47441500000000	-0.04252900000000
Si	4.85436100000000	3.63961000000000	0.88061700000000
Si	1.99722500000000	3.84049100000000	-0.17439800000000
Si	-1.28326000000000	7.63011300000000	0.21136100000000
Si	1.65995200000000	6.79052300000000	0.67065500000000
O	8.96163900000000	-3.83534800000000	2.23530900000000
O	2.01225200000000	6.65777600000000	2.25378500000000
O	7.15359400000000	-2.27993500000000	-0.91426900000000
O	2.50833600000000	4.74493800000000	4.08934500000000
O	6.75705900000000	-4.90434700000000	-0.76333000000000
O	0.04989300000000	5.13761000000000	3.14840300000000
O	5.86479300000000	-3.26740000000000	1.14681900000000
O	4.44873700000000	4.07244500000000	2.39778600000000
O	9.40973200000000	-5.01108800000000	-1.11824800000000
O	5.16078900000000	-0.24675300000000	2.02987400000000
O	7.74169500000000	4.37859800000000	-1.67847900000000
O	10.71215000000000	-3.18459800000000	0.35571000000000
O	5.43793500000000	5.63299800000000	-1.90068300000000
O	4.10329600000000	5.67672200000000	-4.16787800000000
O	8.77013300000000	-1.46127600000000	1.05403800000000
O	3.93161300000000	7.73891400000000	-2.52249200000000
O	1.17893200000000	9.52209700000000	-4.03635400000000
O	8.98921300000000	2.07242200000000	-1.87761300000000
O	6.95483800000000	0.18928700000000	0.11400700000000
O	2.28090900000000	9.55354700000000	-1.55439600000000
O	9.00742700000000	-0.54685100000000	-1.43259200000000
Si	1.44430500000000	5.86512900000000	3.56186800000000
Si	6.12503600000000	-3.44004100000000	-0.43851300000000
Si	3.63687200000000	3.61865200000000	3.73665400000000
Si	8.08747500000000	-5.83603700000000	-0.64668500000000
Si	10.88472600000000	-4.45842800000000	-0.66196600000000
Si	6.67911200000000	5.32670200000000	-0.89721100000000
Si	9.80326900000000	-2.60853800000000	1.57638400000000
Si	4.93380600000000	6.59569500000000	-3.10853900000000
Si	9.21314600000000	3.67367100000000	-1.65758000000000
Si	6.17848200000000	0.85299400000000	1.38453500000000
Si	1.73831500000000	10.45338200000000	-2.81491000000000
Si	9.80842400000000	0.68324800000000	-2.14343300000000
Si	7.96272700000000	-1.01609100000000	-0.29212400000000
Si	3.47779300000000	8.51742400000000	-1.16947700000000
O	2.98453100000000	-0.47259700000000	3.62913700000000

O	0.97105800000000	0.76650900000000	2.48535100000000
O	1.25232200000000	0.89528600000000	5.09434900000000
O	2.98006800000000	2.15627000000000	3.49126800000000
O	-0.39876400000000	1.19025800000000	0.28527600000000
O	-0.80771600000000	2.75584900000000	2.35022800000000
O	-3.74092100000000	4.97761400000000	0.58955900000000
O	-2.52690900000000	4.66256300000000	2.94842800000000
O	-1.15045300000000	3.44114900000000	4.84831800000000
Si	2.04896700000000	0.83856200000000	3.69092700000000
Si	0.31333600000000	1.90351500000000	1.55016400000000
Si	-3.11973900000000	5.74812400000000	1.88156600000000
Si	-1.09058600000000	4.00373900000000	3.33674400000000
O	8.29845100000000	-6.04345600000000	3.50640100000000
O	-3.19211900000000	-3.07058700000000	6.59661400000000
O	6.69000100000000	-7.84690200000000	2.45425800000000
O	-1.30662800000000	-1.56410400000000	7.71019300000000
O	8.26504000000000	-6.34226100000000	0.88185000000000
O	-5.42442400000000	-3.48740400000000	5.26121800000000
O	6.66125400000000	-3.92549000000000	3.58180200000000
O	-5.13864600000000	-1.21989800000000	6.60701900000000
O	-8.65352800000000	0.35845200000000	5.57573300000000
O	4.48586200000000	-2.51216800000000	3.26145300000000
O	-6.43642800000000	1.05078100000000	6.97245200000000
O	4.50257500000000	-5.06568100000000	2.61407100000000
O	-3.87523200000000	0.84991400000000	7.61374200000000
O	-2.24836800000000	2.84601100000000	7.14721100000000
O	0.40826500000000	2.65848000000000	6.88368200000000
O	4.26282500000000	-7.39936800000000	1.38456900000000
O	-1.48870600000000	0.87607200000000	8.79701000000000
O	0.73497300000000	0.08216400000000	7.54609100000000
Si	8.13473600000000	-7.12363300000000	2.29709600000000
Si	-2.11885200000000	-2.97276200000000	7.82699300000000
Si	8.20891700000000	-4.41701800000000	3.56231000000000
Si	-4.79365100000000	-2.81310700000000	6.59577500000000
Si	4.53315200000000	-0.89444700000000	3.38960300000000
Si	-8.07002900000000	1.13097600000000	6.89220100000000
Si	5.36940200000000	-3.70399600000000	2.62505700000000
Si	-5.29045900000000	0.06696100000000	7.58692600000000
Si	-0.92368500000000	3.48645300000000	6.45601600000000
Si	3.45188700000000	-6.09881900000000	1.93653000000000
Si	-2.73434600000000	1.78700500000000	8.28342500000000
Si	1.30515400000000	1.31648800000000	6.66098600000000
Si	5.42468800000000	-8.50812400000000	1.65870800000000
Si	-0.50557000000000	-0.38623900000000	8.48496000000000
O	1.81478700000000	4.27316600000000	-4.04807700000000
O	-0.05843700000000	5.27085800000000	-2.45365600000000
O	-0.60920000000000	7.68350000000000	-3.45500100000000
Si	0.71601600000000	3.92090100000000	-2.90586200000000
Si	-1.19384400000000	6.40706100000000	-2.64472900000000
O	1.25520100000000	-1.73495000000000	-4.48186300000000
O	-3.98042300000000	3.94284800000000	-4.53581000000000
O	3.35150300000000	-2.96152900000000	-3.47364900000000
O	-2.43885900000000	5.81646100000000	-3.49532700000000
O	-3.80943900000000	4.16862800000000	-1.90615600000000
O	1.34036300000000	-4.41332200000000	-4.40565700000000
O	-4.66270300000000	1.77137700000000	-5.90621800000000
O	4.72907700000000	-3.30739500000000	-1.24711600000000
O	-2.54184300000000	1.71333500000000	-4.38260900000000

O	-3.16157800000000	-0.36666100000000	-6.52503000000000
O	-1.09637600000000	-1.79405200000000	-5.65609500000000
O	-0.31634200000000	0.33050500000000	-4.20860400000000
O	-0.35516300000000	2.86029400000000	-3.48669700000000
O	1.78999600000000	6.92817900000000	-4.26211200000000
Si	-3.83118700000000	5.00133200000000	-3.30556400000000
Si	2.20171200000000	-3.04765200000000	-4.62824800000000
Si	-4.03936200000000	2.31782100000000	-4.52050600000000
Si	4.01802200000000	-4.07752600000000	-2.49582200000000
Si	-4.31657800000000	0.66635400000000	-7.06080900000000
Si	-0.29504000000000	-1.29099100000000	-4.33961100000000
Si	-1.20621800000000	1.48391700000000	-3.50595500000000
Si	2.56602700000000	5.56849200000000	-4.70156500000000
Si	0.63459600000000	8.05280900000000	-4.44492700000000
O	-2.78922700000000	-5.59301200000000	-7.10396500000000
O	-4.53332300000000	-4.34207900000000	-5.53874600000000
O	-2.90946400000000	-2.92957900000000	-7.20351100000000
O	-2.81229000000000	0.09671600000000	0.14083300000000
O	-2.49954300000000	2.74166500000000	-0.12824400000000
O	-1.65336000000000	1.01845100000000	-2.01212600000000
O	-5.13469300000000	2.81724500000000	0.00403600000000
Si	-3.76062600000000	-4.29755000000000	-6.96911800000000
Si	-1.83634300000000	1.29149300000000	-0.43404500000000
Si	-3.80912800000000	3.67881800000000	-0.36992000000000
O	-4.99151600000000	1.89538700000000	-3.27525200000000
O	2.82364800000000	-5.05227600000000	-1.97315500000000
O	-5.62552900000000	-2.50279700000000	2.83006900000000
O	-7.38114300000000	-1.79051100000000	4.68975100000000
O	-0.23846100000000	-6.32298700000000	-3.39637400000000
O	-7.99461400000000	0.18369200000000	3.01098400000000
O	-0.38481600000000	-5.57324100000000	-5.95195900000000
O	-0.97337200000000	-1.91462000000000	-3.01175300000000
O	-3.47793700000000	-6.50998900000000	0.74123000000000
O	-0.94559600000000	-4.27398700000000	-1.88781600000000
O	-6.58560200000000	1.03496700000000	-1.37501600000000
O	0.63573300000000	-6.08568200000000	-0.86384700000000
O	-8.35693800000000	-0.62467300000000	0.48773400000000
O	-7.36013800000000	1.78247300000000	1.02991100000000
O	-1.86646300000000	-6.74354800000000	-1.39540400000000
O	-6.53713500000000	-0.21027300000000	-3.70161800000000
O	2.71722800000000	-5.41896300000000	0.65032000000000
O	-9.06901100000000	-2.66368200000000	-1.02815700000000
O	-4.47578500000000	-0.41504200000000	-2.07903600000000
O	-6.98881400000000	-2.88665700000000	0.58882400000000
O	-8.02747700000000	-2.38411800000000	-3.46799900000000
O	-5.37137400000000	-2.61139700000000	-3.73773200000000
O	-7.25687400000000	-4.36993300000000	-1.87436200000000
O	-4.99843600000000	-5.15124800000000	-3.06450000000000
O	-2.88497500000000	-3.64228700000000	-3.63042700000000
O	-5.68661100000000	-6.15827300000000	-0.70949500000000
O	-2.78817600000000	-2.56421700000000	-1.19511300000000
O	-4.76145600000000	-4.21994500000000	0.92373500000000
O	-4.61866200000000	-1.56295300000000	0.69328400000000
Si	-6.47072000000000	-3.01552400000000	4.12429900000000
Si	0.60850200000000	-5.83558300000000	-4.69791700000000
Si	-8.48635800000000	-0.65492500000000	4.32665000000000
Si	-1.23544300000000	-6.06955200000000	-7.24961800000000
Si	-6.64015800000000	2.28974600000000	-0.34858800000000

Si	-2.42788400000000	-7.48866300000000	-0.04936300000000
Si	-0.60569800000000	-5.84892900000000	-1.88704900000000
Si	-8.42641500000000	0.64358200000000	1.50369800000000
Si	-5.62431300000000	0.55669400000000	-2.60104500000000
Si	2.25955800000000	-6.02027800000000	-0.79920800000000
Si	-8.47873800000000	-2.24162600000000	0.42230000000000
Si	-6.69698300000000	-1.75778300000000	-4.16700900000000
Si	-8.51156600000000	-3.44864400000000	-2.34514100000000
Si	-4.44195500000000	-3.92582700000000	-3.97753800000000
Si	-6.25115700000000	-5.62054300000000	-2.13598600000000
Si	-1.91957300000000	-3.10068700000000	-2.42342600000000
Si	-4.94974300000000	-5.83428600000000	0.70817000000000
Al	-3.67753200000000	-1.17807300000000	-0.79568400000000
Si	-5.51332100000000	-2.82959800000000	1.25092000000000
Si	-2.10436300000000	-1.55013900000000	-6.90196900000000
H	11.68201100000000	-5.53663200000000	-0.02821500000000
H	9.20174700000000	-8.10545400000000	2.37332200000000
H	-3.87328600000000	1.40021500000000	-8.24739900000000
H	-5.53835500000000	-0.07944700000000	-7.36210400000000
H	-6.92379800000000	-1.73294700000000	-5.59680800000000
H	-9.60257100000000	-4.23331200000000	-2.90542000000000
H	-4.79979800000000	-4.38576000000000	-7.97480900000000
H	-1.24731700000000	-7.52786700000000	-7.34497900000000
H	-8.45777100000000	2.53259200000000	6.78149400000000
H	-4.20124000000000	6.50559200000000	2.50483200000000
H	-1.80316800000000	9.00632300000000	0.21150600000000
H	0.68439200000000	11.32823500000000	-2.28779600000000
H	-4.94510600000000	5.93197600000000	-3.33612600000000
H	-7.42386400000000	3.36507600000000	-0.93339600000000
H	-9.80607100000000	1.14140600000000	1.46016600000000
H	-3.25255900000000	2.50713300000000	9.44312800000000
H	-0.73677700000000	4.85535500000000	6.91549200000000
H	1.18002900000000	6.84647100000000	4.61624200000000
H	0.02210100000000	-0.85228500000000	9.76306500000000
H	2.66456800000000	1.61419100000000	7.10814700000000
H	4.53903300000000	3.47420200000000	4.87910300000000
H	1.54547200000000	-6.93757900000000	-4.96379800000000
H	4.64367900000000	9.21576000000000	-0.63639300000000
H	-8.66662400000000	0.53615000000000	8.09872400000000
H	-5.67048700000000	-0.35803800000000	8.93677700000000
H	-5.39689700000000	-3.45786300000000	7.75582900000000
H	-2.85180900000000	-3.03946300000000	9.11069100000000
H	-1.21874600000000	-4.11122300000000	7.71386800000000
H	2.58239700000000	5.42848300000000	-6.15377300000000
H	0.23309300000000	8.10316300000000	-5.84617300000000
H	2.80334000000000	11.25729700000000	-3.41206700000000
H	6.06893500000000	7.26957900000000	-3.75705200000000
H	10.01891400000000	4.23819800000000	-2.75127200000000
H	9.88752900000000	0.42411800000000	-3.58475000000000
H	11.17465900000000	0.78784500000000	-1.59486600000000
H	9.90673600000000	3.90381600000000	-0.37702500000000
H	-1.31573500000000	-1.19554000000000	-8.09344200000000
H	-0.60836500000000	-5.49774000000000	-8.45025700000000
H	2.85644700000000	-3.04291800000000	-5.94052500000000
H	-7.30691500000000	-4.15772400000000	3.76046600000000
H	-9.77423600000000	-1.30875400000000	4.08982900000000
H	-9.39493300000000	-2.75342800000000	1.45999600000000
H	-5.76072500000000	-6.40980900000000	1.78972000000000

H	-3.07258100000000	-8.75042600000000	-0.45811200000000
H	-7.01354200000000	-6.69247400000000	-2.77433900000000
H	2.79185400000000	-7.36845900000000	-0.98261400000000
H	4.90518000000000	-9.57697600000000	2.50395300000000
H	5.82910900000000	-9.04673300000000	0.35360400000000
H	-1.33611300000000	-7.77565100000000	0.88453300000000
H	10.67777300000000	-2.01989300000000	2.57366600000000
H	8.83140500000000	-3.95533700000000	4.79373600000000
H	11.59906400000000	-3.97413000000000	-1.84902100000000
H	5.34642000000000	-0.46410700000000	4.52981400000000
H	7.16045900000000	1.34045100000000	2.38220600000000
H	7.33077600000000	6.57881500000000	-0.50344800000000
H	5.01510700000000	-4.86444400000000	-3.23399900000000
H	7.91041200000000	-7.00073900000000	-1.49673300000000
H	2.45084700000000	-6.49934300000000	2.92129100000000
Ga	-3.64451200000000	-0.28093200000000	1.90118400000000
C	-5.12848600000000	2.25144700000000	3.85324700000000
H	-6.17476100000000	1.97689700000000	3.97523100000000
H	-4.95533800000000	1.38541700000000	2.57570700000000
H	-4.92490800000000	3.23112700000000	3.42477900000000
C	-4.15444300000000	1.57207600000000	4.51346600000000
H	-4.35424500000000	0.64387100000000	5.02786800000000
H	-3.12797200000000	1.91198800000000	4.54140600000000
H	-4.93538300000000	0.90636100000000	1.79908200000000
O	-2.85842300000000	-0.32677300000000	3.40046000000000

S2.2 Large cluster (112T)

Geometries optimized at the r^{2}SCAN-D4/def2-SVP level.
Single-point energies at the ωB97X-D4rev/def2-mTZVPP level.

S2.2.1 Gas-phase molecules

Ethane

Electronic energy (r^{2}SCAN-D4/def2-SVP): -79.688218328050 E_h
Electronic energy (ωB97X-D4rev/def2-mTZVPP): -79.901813201150 E_h
G_corr (673.15 K, 1 atm): 0.01571282 E_h
Number of atoms: 8

Cartesian coordinates (Å)

C	-0.29918144227652	0.00000095657818	-1.21661695298404
H	-0.29917895891905	1.02792276709989	-1.62416276691178
H	0.59101656525055	-0.51398771963948	-1.62421718835251
H	-1.18938199678830	-0.51398339054996	-1.62421707448706
C	-0.29918140580474	-0.00000097784181	0.31211486163823
H	-1.18938199007104	0.51398335597961	0.71971498754909
H	-0.29917902749061	-1.02792276138943	0.71966071750833
H	0.59101656809971	0.51398776976299	0.71971505603973

Ethene

Electronic energy (r^2SCAN-D4/def2-SVP): -78.456685385020 E_h
Electronic energy (ωB97X-D4rev/def2-mTZVPP): -78.653140557158 E_h
G_{corr} (673.15 K, 1 atm): -0.00538137 E_h
Number of atoms: 6

Cartesian coordinates (Å)

```
C        -0.09562676162712        0.11753418033476       -1.07770928221429
H        -0.38667069820613        1.02845134776577       -1.61892390105899
H         0.54774473218489       -0.58998618960313       -1.61890867393230
C        -0.50273615964892       -0.11753418113965        0.17320719189188
H        -1.14610091274324        0.58999008326578        0.71440950754602
H        -0.21169896595946       -1.02845524062352        0.71441888776768
```

H_2

Electronic energy (r^2SCAN-D4/def2-SVP): -1.165360038568 E_h
Electronic energy (ωB97X-D4rev/def2-mTZVPP): -1.179320400888 E_h
G_{corr} (673.15 K, 1 atm): -0.02358138 E_h
Number of atoms: 2

Cartesian coordinates (Å)

```
H         0.20177354300000        0.00000000000000       -0.62292934555560
H         0.20177354300000        0.00000000000000        0.13588975955560
```

Benzene

Electronic energy (r^2SCAN-D4/def2-SVP): -231.904252402887 E_h
Electronic energy (ωB97X-D4rev/def2-mTZVPP): -232.426830121033 E_h
G_{corr} (673.15 K, 1 atm): 0.02452818 E_h
Number of atoms: 12

Cartesian coordinates (Å)

```
C         0.25743520876810        0.00000000325338       -1.00538394096342
C        -0.90324400964789       -0.35616701831329       -0.30438402311759
C         1.41811442930078        0.35616701865058       -0.30438402163405
H        -1.81250227993799       -0.63503697669962       -0.85345442574064
H         2.32737270077618        0.63503697049803       -0.85345442373677
C        -0.90324400902743       -0.35616701737708        1.09756277782781
C         1.41811443037180        0.35616701710214        1.09756277900877
H        -1.81250227893429       -0.63503697287172        1.64663317922083
H         2.32737270114970        0.63503696724087        1.64663318124592
C         0.25743520964086        0.00000000297700        1.79856269792590
H         0.25743521123046        0.00000000349405        2.89674069315217
H         0.25743520830971        0.00000000204567       -2.10356193718892
```

S2.2.2 $[GaH_2]^+$ site

GaH_2-zeolite

Electronic energy (r^2SCAN-D4/def2-SVP): -48178.783639863686 E_h
Electronic energy (ωB97X-D4rev/def2-mTZVPP): -48222.308496129200 E_h
G_{corr} (673.15 K, 1 atm): 1.93574908 E_h
Number of atoms: 379

Cartesian coordinates (Å)

Si	48.560682871	36.032605386	35.302779992
Si	46.528217072	36.977646965	37.341062067
Si	44.101107387	36.092529808	35.632205750
Si	44.623651362	38.501657266	33.728379195
Si	47.772348102	38.541516211	33.543438386
Si	48.745627136	38.765412542	30.513428272
Si	51.806021651	38.124663608	30.329435664
Si	54.677999754	38.462665452	38.536699910
Si	57.777265792	38.489876713	38.803332806
Si	56.558351033	37.023184946	34.996489649
Si	52.598203938	36.083820761	32.661476850
Si	51.487109620	37.161241456	35.482929519
Si	54.136519604	36.093707973	36.717408191
Si	45.623258838	36.072393295	40.024568052
Si	41.741515142	38.013476172	41.949877256
Si	43.610591291	38.839594426	44.071724272
Si	47.726526154	38.457997718	46.613004466
Si	48.756107823	38.670851902	43.584704198
Si	46.063452523	38.179104912	42.322693571
Si	51.721975497	37.997895448	43.463687996
Si	53.664633657	38.785384736	41.344796408
Si	56.184630095	38.106067727	43.084638648
Si	44.616812629	50.314664268	33.735395275
Si	47.765133657	50.278356380	33.557807665
Si	48.743513531	50.061135176	30.524023261
Si	43.867088770	46.934649320	30.715823495
Si	48.874949680	46.962775144	30.428334186
Si	46.325092370	45.965302636	29.027158053
Si	48.558265032	52.788823238	35.311192015
Si	46.531206674	51.840631584	37.354901193
Si	44.103090967	52.728225328	35.635790399
Si	43.868267007	41.887005146	30.713265372
Si	48.874452799	41.862870852	30.421534455
Si	46.325347798	42.858140809	29.024904436
Si	51.886846848	42.864864491	30.525941593
Si	57.969776702	42.906674002	39.190078315
Si	58.733806843	47.857377377	36.908924706
Si	56.877074187	48.676177596	34.757495173
Si	55.868730098	48.297798354	31.973554842
Si	52.764672190	48.325738480	32.243638582
Si	56.557817691	51.798759226	34.996202875
Si	52.599607357	52.738089165	32.658919820
Si	51.489387210	51.660057715	35.477711441
Si	54.134982356	52.729040017	36.714944510
Si	51.805685869	50.699128830	30.331473327
Si	54.679009228	50.358952067	38.531419817
Si	57.778477239	50.330943692	38.801909230
Si	51.886597029	45.955136451	30.525928594
Si	57.970262642	45.925272825	39.184084268
Si	58.735010357	40.965112338	36.914291701

Si	56.877953418	40.147098012	34.760867854
Si	55.872321139	40.523746344	31.974296028
Si	52.768765202	40.493504649	32.238961650
Si	48.666537291	40.940516974	48.516360677
Si	41.741611097	50.785899842	41.946070323
Si	43.709212501	50.016076355	44.069534110
Si	47.726913102	50.362564621	46.609387139
Si	48.767951991	50.155268659	43.576357353
Si	46.084895630	50.774245710	42.365799889
Si	44.752837515	45.948995183	46.551386560
Si	47.929054481	45.948974804	46.148902846
Si	48.675641026	47.890684784	48.513753093
Si	45.619939202	52.769622348	40.023170298
Si	44.750750963	42.860413233	46.547365298
Si	47.931443393	42.853385956	46.173840813
Si	58.955990956	41.841304201	41.953168272
Si	56.320004090	42.832274056	43.279764585
Si	55.796981378	48.296046673	45.169750022
Si	52.646085287	48.352445105	45.336285771
Si	51.671921900	48.628027959	48.353964649
Si	51.738450213	50.826614790	43.459718007
Si	53.667743500	50.032028759	41.339336557
Si	56.183593329	50.709731719	43.088267782
Si	58.958635334	46.981029618	41.934736446
Si	56.317376188	45.963924399	43.267506128
Si	55.796152674	40.496678121	45.170636346
Si	52.681700897	40.440505181	45.348848144
Si	51.669943360	40.194638817	48.351634101
O	49.982781502	36.640048289	35.859318361
O	47.515702467	35.917964640	36.574991586
O	46.663439568	36.834027963	38.965346918
O	44.969642693	36.655480982	36.930145279
O	44.495241909	36.946391846	34.261326449
O	46.175158070	38.852959405	33.273205473
O	47.933578823	37.018527040	34.133661493
O	48.601996450	38.668562000	32.145848364
O	50.187865652	38.131942563	30.081999863
O	52.219325329	36.835595111	31.247674568
O	52.298593983	39.554574901	30.983115168
O	54.602415173	36.886984447	38.081408725
O	56.173858538	38.733995607	39.135847977
O	56.916825880	38.602077054	35.309933623
O	54.993895492	36.663336619	35.421658745
O	51.812769117	36.858719526	33.901741685
O	52.535376857	36.328255726	36.431932478
O	42.424860844	37.880490107	43.449300387
O	45.107193987	38.315680113	43.633735216
O	48.547574384	38.684698861	45.202330467
O	47.576411813	37.839273440	42.812669225
O	45.555565571	36.910888434	41.436071364
O	50.115272886	37.845097418	43.228553755
O	52.412543470	37.943687256	41.968126957
O	52.079260536	39.420014443	44.217870574
O	53.574298884	38.747834774	39.710076863
O	55.074309023	38.118231694	41.859122168
O	44.495384199	51.871510482	34.266875033
O	46.169658843	49.957053731	33.289294441
O	47.917145537	51.802998101	34.149066920

O	48.591226684	50.157509131	32.156709679
O	48.597896587	48.512766329	29.988646046
O	45.411364439	46.592975701	30.240044346
O	47.900050397	45.997935206	29.522710402
O	45.877518642	44.411814096	28.740200911
O	49.984109915	52.178067303	35.853721675
O	47.528282947	52.899616077	36.598293566
O	46.652241975	51.972793222	38.985106546
O	44.976559275	52.173854012	36.935573664
O	48.592193055	40.314028063	29.980847786
O	45.412898257	42.227541545	30.237372651
O	47.901671100	42.829671597	29.516076630
O	50.432459677	42.226697003	30.099383713
O	51.674346291	44.410040153	31.031192458
O	58.591547692	44.416551910	39.362059204
O	58.126346479	47.832143770	35.381218670
O	58.289287802	46.473263455	37.676877153
O	58.217583055	49.189959979	37.703419006
O	56.998350466	48.629037146	33.133458202
O	56.913358451	50.219722072	35.310758906
O	54.347399432	48.581907253	32.576062968
O	52.555881401	46.761004760	31.798797198
O	54.993540297	52.159558950	35.420545274
O	51.812107610	51.958106358	33.895020253
O	52.534113297	52.500052628	36.424722094
O	50.185469963	50.699536733	30.097175797
O	52.228264279	51.991240217	31.241125123
O	52.291543404	49.270210887	30.992653232
O	54.597143272	51.935186092	38.080177394
O	56.174142600	50.089164384	39.132432892
O	50.431352701	46.593839092	30.103528132
O	58.122541075	40.995597413	35.388400542
O	58.303265372	42.348400146	37.690035977
O	58.216658223	39.631711181	37.705330268
O	57.000684461	40.196512132	33.136478327
O	54.350694417	40.240653622	32.575098056
O	52.553481005	42.059432898	31.800660430
O	50.278850989	40.942979387	48.805252785
O	48.269909212	42.303618045	47.678779832
O	48.220120079	39.581663212	47.711656775
O	42.463706756	50.881997661	43.431195663
O	45.170406209	50.684037871	43.714362509
O	48.530213287	50.097793790	45.189925883
O	47.624318698	51.037387291	42.809233408
O	45.567683987	52.040113314	41.492343900
O	44.302061457	44.409297879	46.858742641
O	46.299980726	46.005577128	46.004580645
O	48.462190853	44.404186939	46.018542519
O	50.281349561	47.893293327	48.830125747
O	48.324917990	46.553247498	47.616339103
O	48.228422096	49.257826769	47.722218122
O	46.300150382	42.817486438	45.985991243
O	58.650376169	41.948718229	40.323425313
O	57.915520136	42.726424236	42.899154057
O	55.900371639	44.399266584	43.470348622
O	55.994173099	46.730113672	44.686414673
O	54.245721948	48.595522154	45.650521747
O	56.177443702	49.268935956	43.898099967

O	51.797839034	48.585226750	46.706508066
O	50.131336082	50.971380328	43.237187623
O	52.422285723	50.885885461	41.963544546
O	52.087278223	49.407996451	44.216922027
O	53.576296841	50.069291709	39.705682814
O	55.079622633	50.690748774	41.856788074
O	58.670460267	46.892500916	40.300049600
O	57.900229451	46.077510470	42.841675848
O	55.944831294	42.069520893	44.687517050
O	54.268145394	40.118892179	45.667531533
O	56.200068227	39.551289757	43.886160720
O	51.810530170	40.230430779	46.706512873
H	48.702871362	34.676568244	34.772565866
H	44.419875251	34.676322627	35.346829339
H	42.683628407	36.258975103	36.022158957
H	43.726523133	38.680216478	32.577941211
H	47.730594397	38.010996401	29.830569038
H	52.421136636	38.002379606	29.030379787
H	58.465353268	38.743362535	40.066966856
H	58.088382014	37.108508758	38.286194614
H	56.651060555	36.758840975	33.558678099
H	57.541582073	36.246994821	35.782467407
H	52.141233711	34.673921991	32.562044671
H	54.071037026	36.146097417	32.821438136
H	54.418081919	34.675478269	36.917894802
H	44.265382390	36.148734272	39.494230475
H	46.085044778	34.677315684	40.196440171
H	42.177304178	36.886191594	41.136491098
H	40.290194813	38.107003297	42.090376678
H	43.614691599	38.689573153	45.487246793
H	46.266760189	38.594605241	46.380864381
H	48.065373583	37.113072076	47.131272477
H	52.195251260	36.881483483	44.271734144
H	55.858295687	36.988841751	44.011824321
H	57.529558019	37.965437314	42.519250265
H	41.636484485	38.780153701	36.634494801
H	40.338248639	40.845234588	36.817795235
H	43.726295711	50.140518159	32.578070824
H	47.730502479	50.809921518	29.830551197
H	42.910974065	46.071594304	29.969678286
H	43.636013018	48.346500263	30.474316401
H	46.055517840	46.690812611	27.779371156
H	40.338195451	47.975591870	36.817798680
H	41.636403449	50.040687140	36.634466843
H	48.702714157	54.144376547	34.772532306
H	44.419584799	54.144606094	35.346948050
H	42.683574792	52.561616139	36.022275759
H	43.636069423	40.474360869	30.474326854
H	42.910997640	42.749252508	29.969681372
H	46.055550549	42.130076025	27.779376809
H	52.768240412	42.805897822	29.339146537
H	60.152182769	47.950749095	36.769108003
H	56.115707931	49.276599344	30.928771527
H	56.049418290	46.904456635	31.507081424
H	57.541464680	52.574079099	35.782445963
H	56.650952298	52.062214123	33.558655554
H	52.141089608	54.147065236	32.562019146
H	54.070917209	52.674924865	32.821418302

H	54.418140421	54.145525073	36.917811094
H	52.421044390	50.818602390	29.030362565
H	58.465268666	50.077735795	40.066952197
H	58.088278420	51.712581938	38.286178871
H	52.768217283	46.015091534	29.339142101
H	60.152233951	40.870367687	36.769119252
H	56.049458218	41.916584845	31.507089825
H	56.115777280	39.544444038	30.928782723
H	48.027948168	40.958435310	49.819978604
H	40.290114763	50.713683390	42.090582461
H	42.176849933	51.934815245	41.136521049
H	43.615015124	50.131729410	45.487214710
H	48.065228639	51.707917767	47.131231656
H	46.266684593	50.226321085	46.380825032
H	40.476352170	46.423809081	42.229529801
H	44.578029760	46.685732001	47.785515427
H	48.027870710	47.862536808	49.819961519
H	46.084738834	54.143667565	40.196331471
H	44.265252478	52.672179111	39.494179472
H	40.476382218	42.397038343	42.229542218
H	44.578054574	42.135174328	47.785513307
H	58.818795591	40.408566696	42.221796398
H	60.309668468	42.345550427	42.255160442
H	56.725235480	48.548903522	46.274186856
H	51.671680742	50.045758235	48.769744695
H	52.721672684	47.848744600	49.007593385
H	52.195144448	51.939533512	44.271718210
H	55.858180477	51.832229498	44.011805810
H	57.529465448	50.855664233	42.519235697
H	60.309638962	46.475584454	42.255156083
H	58.818735571	48.412544373	42.221785997
H	56.725176589	40.272281027	46.274313225
H	52.722235356	40.971966505	49.006965758
H	51.671814816	38.775268647	48.769820882
Si	48.770800624	40.999168624	35.409338088
Si	46.870629766	40.037622417	37.433760973
Si	41.577166492	40.155518031	37.179876591
Si	44.268005618	40.878412497	35.854672869
Si	44.637123848	45.941975040	33.464739890
Si	47.815755121	45.939037445	33.192673085
Si	48.763467533	47.827503982	35.432870539
Si	46.857465944	48.781354616	37.465629115
Si	41.573133402	48.670581940	37.175047703
Si	44.262470266	47.945508045	35.866782729
Si	44.635689231	42.889576764	33.458802137
Si	47.813457065	42.892825009	33.184068745
Si	54.814589118	42.896648117	38.826096984
Si	51.687850617	48.538573954	35.171101958
Si	54.399131743	47.960378030	36.442875650
Si	54.818121226	45.933321001	38.830099816
Si	51.689270288	40.279745677	35.161142282
Si	54.396045030	40.860999781	36.444570652
Si	45.730393712	40.480649370	40.159740622
Si	42.647401791	40.422630139	40.050520443
Si	41.766327942	45.945057489	41.708171354
Si	43.907341403	46.863378596	43.784240602
Si	48.903993720	47.032767751	43.337292780
Si	46.304532438	45.988186908	41.946898218

Si	45.705510411	48.444111857	40.217910179
Si	42.625673618	48.404383681	40.037373824
Si	41.775263558	42.892891773	41.758918640
Si	43.819096548	41.937601883	43.835188271
Si	48.987648063	41.778876702	43.432376563
Al	46.382258376	42.796876013	42.109451923
Si	51.928551673	42.856830530	43.599349631
Si	53.954549535	41.878164666	41.531441096
Si	51.837704663	45.917149925	43.549905486
Si	53.917542751	46.937265469	41.529082312
O	46.917089593	38.500012753	36.850374476
O	44.177311508	39.500712736	34.965521526
O	54.386592176	39.425283416	37.242883059
O	51.577473943	38.770857979	35.802308702
O	42.315526717	39.388461875	41.253668685
O	50.380682399	41.196144036	35.490622432
O	48.191120729	40.862653685	36.937681038
O	48.092168000	42.297461011	34.682022477
O	48.408307350	39.632807213	34.579715623
O	46.847816418	40.008440748	39.063666165
O	45.520672204	40.806749455	36.906905497
O	41.631062416	40.057698483	38.826600286
O	42.855410465	41.069247014	36.650776795
O	44.510802936	42.192737444	34.921811085
O	44.159665003	49.320794826	34.972673588
O	43.731859266	46.748417508	32.359575035
O	44.063022823	44.415710063	33.571277040
O	46.196428047	45.930817796	32.961571293
O	48.405017607	44.415686295	33.074030766
O	48.563788439	46.841687494	32.045747454
O	50.374702890	47.636837032	35.521645365
O	48.174974142	47.954706020	36.960396827
O	48.096926942	46.529385730	34.692590925
O	48.406555101	49.193330451	34.598255218
O	46.832907863	48.827295513	39.098147483
O	46.919522539	50.317695255	36.874112213
O	45.504052248	48.034062304	36.926519951
O	41.623237544	48.810768978	38.818349420
O	42.848813455	47.747003332	36.661110726
O	44.514042311	46.635557600	34.929816349
O	43.730468268	42.081428876	32.355391715
O	46.194172057	42.903350386	32.952812180
O	48.561915825	41.989739660	32.038073093
O	54.243354764	44.416090159	38.613120328
O	56.348278659	42.956701891	39.391959759
O	55.464485289	47.983696212	35.208289919
O	51.850172091	48.693606216	33.545425193
O	52.919438213	47.691731086	35.820507690
O	51.588983650	50.052647247	35.802757547
O	54.816015808	46.722885271	37.410594938
O	54.388651616	49.399421043	37.235926010
O	56.349591787	45.883620248	39.407848089
O	55.464188653	40.835905192	35.212486464
O	51.856627586	40.111618121	33.537642509
O	52.919283292	41.124865245	35.813879207
O	54.803111878	42.105846960	37.407470629
O	45.943918777	42.094390129	40.487946844
O	44.215990762	40.227626214	39.566491513

O	45.984485716	39.618597246	41.520305878
O	42.429079995	41.964981778	40.558365849
O	42.236115403	49.361163122	41.290393210
O	43.675885678	48.461123707	43.518379345
O	48.781354284	48.627054532	42.968919807
O	42.790797667	45.979546446	42.995002698
O	41.611479879	44.411285722	41.143947796
O	43.783657203	46.601595529	45.391750605
O	45.393661877	46.402460590	43.242534887
O	48.590199906	46.843870827	44.941751667
O	47.868788342	46.180774249	42.381867718
O	46.019515242	44.437812328	41.438443087
O	45.884636671	46.860175765	40.617806292
O	44.195949812	48.676769852	39.604008903
O	45.941089427	49.355602918	41.549642496
O	42.403223790	46.838048645	40.470986904
O	43.330067943	40.387794314	43.593805022
O	48.769840702	40.202738395	43.012145770
O	42.772102240	42.942682323	43.062669471
O	43.719286992	42.216672751	45.449580095
O	45.319794634	42.183684854	43.302431724
O	48.687841683	41.922284055	45.058861896
O	48.037809080	42.744611912	42.555180384
O	53.550627892	40.334023894	41.882470927
O	50.551120095	42.142258272	43.118394292
O	52.947387627	42.902386304	42.309258065
O	51.667776928	44.390800971	44.113127397
O	53.874098835	42.099148994	39.907990351
O	55.493993188	42.148834235	42.034764955
O	52.431968283	46.823453762	44.783930297
O	53.530654665	48.486665688	41.880522647
O	50.411625230	46.508071858	43.004531072
O	52.872273354	45.927792709	42.274639009
O	53.866326946	46.730134810	39.903150971
O	55.441984320	46.647309306	42.058882298
O	52.596337236	41.998408677	44.832368592
Ga	45.665179324	43.871128079	39.496956066
H	46.985334907	44.117897127	38.687898900
H	44.137845783	44.044343740	39.216163961

Complex (GaH_2-zeolite + C_2H_6)

Electronic energy (r^2SCAN-D4/def2-SVP): -48258.48375721 E_h
Electronic energy (ωB97X-D4rev/def2-mTZVPP): -48302.222363564500 E_h
G_{corr} (673.15 K, 1 atm): 1.99478225 E_h
Number of atoms: 387

Cartesian coordinates (Å)

Si	48.559703000	36.031737000	35.302533000
Si	46.527522000	36.971144000	37.337248000
Si	44.100157000	36.091689000	35.631674000
Si	44.622446000	38.502128000	33.728607000
Si	47.771432000	38.540778000	33.542343000
Si	48.746288000	38.764179000	30.512605000
Si	51.806065000	38.123693000	30.329176000
Si	54.674776000	38.465688000	38.542282000
Si	57.775822000	38.489766000	38.804278000

Si	56.558564000	37.023719000	34.996825000
Si	52.597890000	36.083906000	32.661536000
Si	51.485376000	37.161311000	35.481981000
Si	54.134720000	36.094489000	36.718018000
Si	45.625203000	36.075851000	40.026064000
Si	41.744843000	38.024642000	41.949485000
Si	43.601164000	38.886068000	44.080643000
Si	47.725645000	38.467457000	46.622856000
Si	48.755863000	38.685639000	43.600788000
Si	46.061796000	38.198775000	42.319999000
Si	51.718473000	38.004027000	43.472301000
Si	53.663600000	38.789186000	41.351879000
Si	56.183254000	38.108208000	43.087905000
Si	44.614850000	50.313011000	33.737180000
Si	47.763622000	50.278105000	33.559323000
Si	48.743609000	50.062174000	30.524772000
Si	43.866768000	46.934664000	30.715971000
Si	48.874249000	46.963541000	30.430200000
Si	46.325159000	45.965647000	29.027561000
Si	48.557026000	52.788910000	35.311674000
Si	46.531462000	51.841991000	37.355536000
Si	44.103120000	52.728303000	35.636397000
Si	43.868073000	41.886871000	30.713214000
Si	48.874480000	41.863125000	30.422495000
Si	46.325335000	42.858092000	29.024958000
Si	51.888209000	42.865036000	30.527109000
Si	57.976020000	42.910330000	39.190661000
Si	58.734522000	47.857360000	36.909388000
Si	56.878418000	48.676142000	34.757468000
Si	55.869522000	48.297979000	31.973856000
Si	52.766121000	48.327396000	32.242751000
Si	56.557960000	51.798635000	34.996168000
Si	52.599755000	52.738266000	32.658163000
Si	51.488548000	51.660235000	35.475344000
Si	54.133402000	52.728735000	36.713656000
Si	51.806252000	50.700194000	30.331340000
Si	54.676729000	50.355069000	38.534117000
Si	57.777394000	50.331028000	38.802575000
Si	51.888040000	45.955088000	30.527412000
Si	57.974739000	45.923546000	39.183518000
Si	58.735242000	40.966150000	36.915817000
Si	56.876573000	40.147673000	34.762093000
Si	55.873535000	40.523741000	31.974409000
Si	52.770103000	40.492101000	32.238591000
Si	48.690498000	40.951040000	48.527466000
Si	41.746588000	50.774434000	41.944554000
Si	43.712562000	49.975439000	44.077649000
Si	47.725506000	50.354029000	46.615281000
Si	48.763792000	50.143856000	43.582803000
Si	46.081103000	50.766468000	42.363562000
Si	44.909957000	46.013254000	46.539922000
Si	47.953418000	46.027140000	46.134892000
Si	48.698237000	47.879902000	48.521878000
Si	45.620121000	52.768662000	40.025966000
Si	44.912624000	42.797923000	46.537107000
Si	47.966244000	42.782224000	46.161581000
Si	58.958243000	41.841795000	41.956200000
Si	56.333992000	42.832744000	43.274099000

Si	55.789841000	48.286282000	45.173782000
Si	52.631588000	48.330702000	45.342275000
Si	51.675153000	48.630680000	48.362840000
Si	51.737672000	50.819658000	43.467909000
Si	53.669377000	50.032017000	41.343716000
Si	56.182213000	50.708656000	43.090717000
Si	58.961132000	46.980873000	41.936856000
Si	56.331598000	45.964835000	43.259838000
Si	55.788362000	40.508146000	45.175333000
Si	52.661467000	40.463985000	45.354530000
Si	51.674134000	40.191077000	48.361371000
O	49.980631000	36.641668000	35.859413000
O	47.514093000	35.909061000	36.573719000
O	46.664257000	36.832336000	38.961481000
O	44.967473000	36.651029000	36.931588000
O	44.495384000	36.947091000	34.262300000
O	46.174100000	38.854834000	33.275312000
O	47.931058000	37.018776000	34.135324000
O	48.600377000	38.663242000	32.144198000
O	50.187989000	38.129797000	30.081085000
O	52.220343000	36.834643000	31.246817000
O	52.297195000	39.553806000	30.983375000
O	54.593303000	36.891508000	38.082133000
O	56.172632000	38.728990000	39.140046000
O	56.918986000	38.602291000	35.309981000
O	54.994255000	36.665485000	35.424064000
O	51.810510000	36.859638000	33.900313000
O	52.533710000	36.325647000	36.428345000
O	42.413274000	37.928267000	43.456730000
O	45.086133000	38.360253000	43.611439000
O	48.557107000	38.696411000	45.218542000
O	47.567314000	37.861337000	42.834383000
O	45.566454000	36.923576000	41.433774000
O	50.110910000	37.856158000	43.235018000
O	52.408612000	37.953546000	41.976252000
O	52.075942000	39.421884000	44.233849000
O	53.572812000	38.751455000	39.717205000
O	55.073079000	38.118217000	41.861887000
O	44.494899000	51.869994000	34.268410000
O	46.168182000	49.953416000	33.294387000
O	47.913601000	51.803004000	34.150715000
O	48.587570000	50.159075000	32.156890000
O	48.601239000	48.513722000	29.988739000
O	45.411432000	46.591955000	30.241464000
O	47.900099000	45.999295000	29.522906000
O	45.878244000	44.411975000	28.740361000
O	49.982657000	52.175735000	35.852181000
O	47.528701000	52.901989000	36.600186000
O	46.651137000	51.971435000	38.986168000
O	44.976606000	52.175064000	36.936597000
O	48.595043000	40.313667000	29.982196000
O	45.412899000	42.228200000	30.238044000
O	47.901737000	42.828556000	29.515481000
O	50.432246000	42.229253000	30.102266000
O	51.679001000	44.410026000	31.034012000
O	58.602193000	44.417951000	39.367093000
O	58.127064000	47.831665000	35.381720000
O	58.297694000	46.471124000	37.677205000

O	58.213314000	49.187472000	37.704919000
O	56.999692000	48.628856000	33.133365000
O	56.914388000	50.219749000	35.310594000
O	54.348488000	48.583593000	32.576728000
O	52.558793000	46.762342000	31.798505000
O	54.993489000	52.158799000	35.420373000
O	51.810060000	51.956846000	33.892077000
O	52.532534000	52.504034000	36.419728000
O	50.185599000	50.701970000	30.100208000
O	52.230743000	51.992850000	31.239128000
O	52.293153000	49.271124000	30.991248000
O	54.590178000	51.929431000	38.077159000
O	56.173220000	50.092619000	39.135015000
O	50.430637000	46.591528000	30.109170000
O	58.118173000	40.998992000	35.391799000
O	58.314385000	42.352111000	37.692020000
O	58.211000000	39.635542000	37.708059000
O	57.001187000	40.198071000	33.137692000
O	54.351873000	40.238743000	32.573687000
O	52.554880000	42.058101000	31.800773000
O	50.287815000	40.902322000	48.873892000
O	48.411319000	42.339805000	47.676790000
O	48.213976000	39.611148000	47.704514000
O	42.463948000	50.834578000	43.432661000
O	45.163269000	50.656037000	43.706435000
O	48.531095000	50.092082000	45.196211000
O	47.620549000	51.023271000	42.811352000
O	45.571425000	52.041360000	41.497088000
O	44.937860000	44.409748000	46.849442000
O	46.384557000	46.507415000	45.988040000
O	48.107287000	44.407568000	46.001249000
O	50.288814000	47.932724000	48.894671000
O	48.466034000	46.518338000	47.611818000
O	48.224930000	49.228302000	47.709635000
O	46.392623000	42.327312000	45.971460000
O	58.644851000	41.949593000	40.328103000
O	57.934184000	42.733537000	42.913849000
O	55.899273000	44.399967000	43.435491000
O	55.997073000	46.723240000	44.680532000
O	54.234537000	48.556057000	45.661143000
O	56.158498000	49.271720000	43.907419000
O	51.781700000	48.557408000	46.714250000
O	50.129983000	50.954596000	43.240488000
O	52.421315000	50.878112000	41.971337000
O	52.092180000	49.407159000	44.232861000
O	53.574982000	50.068584000	39.710178000
O	55.081014000	50.694871000	41.856314000
O	58.662895000	46.893773000	40.303921000
O	57.920386000	46.069673000	42.856144000
O	55.948228000	42.078922000	44.684189000
O	54.253091000	40.162689000	45.674959000
O	56.182969000	39.549305000	43.897197000
O	51.791693000	40.256645000	46.715014000
H	48.702865000	34.676576000	34.772526000
H	44.419871000	34.676335000	35.346775000
H	42.683621000	36.258985000	36.022101000
H	43.726520000	38.680255000	32.577916000
H	47.730608000	38.011013000	29.830535000

H	52.421155000	38.002393000	29.030359000
H	58.465339000	38.743348000	40.066963000
H	58.088368000	37.108498000	38.286188000
H	56.651061000	36.758839000	33.558667000
H	57.541575000	36.246988000	35.782458000
H	52.141232000	34.673929000	32.562019000
H	54.071039000	36.146098000	32.821418000
H	54.418070000	34.675475000	36.917874000
H	44.265361000	36.148745000	39.494181000
H	46.085013000	34.677316000	40.196397000
H	42.177283000	36.886199000	41.136444000
H	40.290172000	38.107018000	42.090328000
H	43.614669000	38.689547000	45.487210000
H	46.266721000	38.594606000	46.380819000
H	48.065324000	37.113065000	47.131240000
H	52.195223000	36.881478000	44.271710000
H	55.858264000	36.988828000	44.011812000
H	57.529531000	37.965423000	42.519243000
H	41.636484000	38.780156000	36.634489000
H	40.338250000	40.845237000	36.817790000
H	43.726310000	50.140516000	32.578056000
H	47.730535000	50.809932000	29.830531000
H	42.910996000	46.071616000	29.969655000
H	43.636036000	48.346514000	30.474309000
H	46.055546000	46.690835000	27.779345000
H	40.338196000	47.975594000	36.817794000
H	41.636406000	50.040687000	36.634464000
H	48.702733000	54.144382000	34.772514000
H	44.419611000	54.144617000	35.346911000
H	42.683591000	52.561638000	36.022238000
H	43.636082000	40.474383000	30.474295000
H	42.911011000	42.749275000	29.969668000
H	46.055574000	42.130090000	27.779347000
H	52.768260000	42.805899000	29.339130000
H	60.152188000	47.950738000	36.769118000
H	56.115732000	49.276601000	30.928775000
H	56.049437000	46.904458000	31.507080000
H	57.541482000	52.574071000	35.782456000
H	56.650976000	52.062211000	33.558662000
H	52.141118000	54.147069000	32.562015000
H	54.070944000	52.674927000	32.821417000
H	54.418155000	54.145522000	36.917812000
H	52.421081000	50.818607000	29.030355000
H	58.465267000	50.077722000	40.066962000
H	58.088286000	51.712571000	38.286190000
H	52.768240000	46.015103000	29.339128000
H	60.152230000	40.870355000	36.769124000
H	56.049470000	41.916585000	31.507084000
H	56.115788000	39.544445000	30.928775000
H	48.027903000	40.958423000	49.819947000
H	40.290109000	50.713696000	42.090541000
H	42.176856000	51.934814000	41.136478000
H	43.614997000	50.131750000	45.487185000
H	48.065202000	51.707912000	47.131214000
H	46.266662000	50.226317000	46.380794000
H	40.476351000	46.423810000	42.229525000
H	44.578025000	46.685730000	47.785501000
H	48.027835000	47.862532000	49.819938000

H	46.084739000	54.143674000	40.196307000
H	44.265256000	52.672185000	39.494148000
H	40.476380000	42.397039000	42.229537000
H	44.578049000	42.135174000	47.785498000
H	58.818771000	40.408551000	42.221797000
H	60.309652000	42.345528000	42.255168000
H	56.725215000	48.548888000	46.274188000
H	51.671650000	50.045745000	48.769736000
H	52.721638000	47.848730000	49.007583000
H	52.195134000	51.939527000	44.271710000
H	55.858171000	51.832216000	44.011810000
H	57.529458000	50.855650000	42.519244000
H	60.309627000	46.475568000	42.255169000
H	58.818726000	48.412529000	42.221796000
H	56.725143000	40.272261000	46.274306000
H	52.722191000	40.971950000	49.006949000
H	51.671767000	38.775255000	48.769795000
Si	48.772348000	41.000969000	35.403358000
Si	46.871379000	40.025530000	37.419461000
Si	41.576676000	40.153535000	37.183771000
Si	44.264622000	40.877957000	35.854115000
Si	44.630475000	45.942152000	33.466765000
Si	47.811570000	45.941072000	33.194114000
Si	48.766742000	47.827399000	35.431721000
Si	46.861679000	48.783827000	37.459610000
Si	41.571629000	48.673766000	37.178207000
Si	44.260207000	47.945195000	35.869086000
Si	44.631506000	42.889444000	33.459320000
Si	47.810209000	42.894689000	33.183338000
Si	54.818302000	42.897880000	38.823563000
Si	51.689440000	48.539655000	35.168480000
Si	54.399588000	47.953521000	36.441226000
Si	54.819420000	45.930095000	38.825610000
Si	51.690083000	40.279560000	35.161357000
Si	54.395111000	40.865888000	36.446265000
Si	45.742506000	40.481876000	40.142510000
Si	42.653642000	40.428413000	40.048995000
Si	41.754829000	45.940502000	41.688205000
Si	43.917699000	46.784734000	43.794228000
Si	48.963083000	47.016815000	43.306168000
Si	46.332262000	45.974126000	41.932376000
Si	45.708569000	48.439322000	40.208897000
Si	42.627124000	48.399348000	40.028877000
Si	41.762812000	42.902161000	41.741948000
Si	43.818406000	42.018771000	43.844489000
Si	49.049960000	41.801043000	43.405604000
Al	46.407397000	42.795450000	42.081350000
Si	51.935894000	42.862374000	43.552601000
Si	53.992470000	41.870395000	41.531886000
Si	51.847999000	45.920946000	43.503787000
Si	53.956608000	46.946780000	41.526174000
O	46.916729000	38.489625000	36.832352000
O	44.170333000	39.501870000	34.963185000
O	54.387778000	39.435311000	37.252778000
O	51.576984000	38.770871000	35.802850000
O	42.337230000	39.402218000	41.266375000
O	50.381737000	41.199029000	35.483618000
O	48.190773000	40.854556000	36.929504000

O	48.091623000	42.300873000	34.681449000
O	48.411166000	39.635573000	34.572116000
O	46.849108000	39.983060000	39.048360000
O	45.522507000	40.801521000	36.899982000
O	41.634625000	40.046674000	38.831216000
O	42.856218000	41.067689000	36.658165000
O	44.503654000	42.194400000	34.923228000
O	44.153032000	49.318866000	34.972759000
O	43.728889000	46.749563000	32.359432000
O	44.057516000	44.415146000	33.568204000
O	46.191502000	45.933653000	32.969112000
O	48.400276000	44.417826000	33.070516000
O	48.557125000	46.845039000	32.046647000
O	50.377612000	47.635848000	35.518466000
O	48.181853000	47.957600000	36.960461000
O	48.097720000	46.529116000	34.694055000
O	48.409192000	49.193156000	34.597334000
O	46.833868000	48.833182000	39.091502000
O	46.920702000	50.320664000	36.869192000
O	45.512504000	48.032161000	36.916030000
O	41.619877000	48.828814000	38.821094000
O	42.852527000	47.751111000	36.675707000
O	44.498358000	46.632284000	34.932572000
O	43.728845000	42.079223000	32.355494000
O	46.190557000	42.903746000	32.955320000
O	48.558404000	41.990185000	32.038296000
O	54.230809000	44.414455000	38.631927000
O	56.354008000	42.968134000	39.380109000
O	55.466223000	47.983402000	35.208124000
O	51.849690000	48.696549000	33.542793000
O	52.920926000	47.691251000	35.814730000
O	51.591742000	50.053148000	35.802034000
O	54.810916000	46.705077000	37.398143000
O	54.390587000	49.386466000	37.244535000
O	56.353163000	45.873968000	39.393883000
O	55.459990000	40.832219000	35.211501000
O	51.860490000	40.109805000	33.538623000
O	52.916661000	41.126584000	35.817973000
O	54.803113000	42.119945000	37.397672000
O	45.981112000	42.093231000	40.456836000
O	44.221488000	40.239509000	39.561557000
O	45.998700000	39.634162000	41.510144000
O	42.424808000	41.971345000	40.547943000
O	42.260421000	49.350949000	41.294483000
O	43.695030000	48.396289000	43.599966000
O	48.765330000	48.612777000	42.984206000
O	42.787723000	45.960393000	42.965410000
O	41.580795000	44.412544000	41.112458000
O	43.799914000	46.396906000	45.381957000
O	45.398648000	46.336085000	43.226576000
O	48.790184000	46.750305000	44.921384000
O	47.888480000	46.168077000	42.393619000
O	46.070428000	44.432509000	41.390684000
O	45.910072000	46.861468000	40.615517000
O	44.198258000	48.653675000	39.589765000
O	45.926981000	49.355220000	41.538434000
O	42.386038000	46.833104000	40.448578000
O	43.322291000	40.457304000	43.684479000

O	48.771540000	40.221546000	43.041103000
O	42.753096000	42.979178000	43.046234000
O	43.751849000	42.427737000	45.437927000
O	45.304287000	42.232279000	43.260207000
O	48.898259000	42.025928000	45.044726000
O	48.050177000	42.752614000	42.570883000
O	53.555422000	40.337881000	41.893987000
O	50.599962000	42.125449000	42.980793000
O	53.025552000	42.922317000	42.323475000
O	51.617105000	44.393549000	44.048122000
O	53.900015000	42.078746000	39.907751000
O	55.540163000	42.118939000	42.022879000
O	52.395859000	46.811169000	44.771498000
O	53.540923000	48.485927000	41.887641000
O	50.468786000	46.550685000	42.875801000
O	52.950742000	45.911295000	42.288545000
O	53.885482000	46.746512000	39.899885000
O	55.490842000	46.677044000	42.040757000
O	52.545892000	42.014693000	44.822656000
Ga	45.682778000	43.868915000	39.464841000
H	46.977686000	44.109157000	38.615275000
H	44.148316000	44.038628000	39.217585000
C	50.325047000	42.861401000	39.263726000
H	50.779266000	42.191029000	40.016130000
H	50.897022000	42.742766000	38.324223000
H	49.303547000	42.485555000	39.069518000
C	50.314829000	44.312946000	39.742544000
H	51.338319000	44.686573000	39.931950000
H	49.740956000	44.424850000	40.681605000
H	49.856939000	44.987686000	38.994300000

TS1-GaH$_2$

Electronic energy (r^{2}SCAN-D4/def2-SVP): -48258.39825344 E_h
Electronic energy (ωB97X-D4rev/def2-mTZVPP): -48302.140288268500 E_h
G_{corr} (673.15 K, 1 atm): 1.99338756 E_h
Number of atoms: 387

Cartesian coordinates (Å)

Si	48.559938000	36.019000000	35.319319000
Si	46.525977000	36.926369000	37.337870000
Si	44.097008000	36.085203000	35.629551000
Si	44.632099000	38.488921000	33.714004000
Si	47.773353000	38.509625000	33.530608000
Si	48.751067000	38.751429000	30.513643000
Si	51.804839000	38.125422000	30.327494000
Si	54.678820000	38.461716000	38.535880000
Si	57.777509000	38.490926000	38.802967000
Si	56.556767000	37.021752000	34.996500000
Si	52.596232000	36.084209000	32.664307000
Si	51.483182000	37.159089000	35.488799000
Si	54.136542000	36.093027000	36.716294000
Si	45.624768000	36.061129000	40.031068000
Si	41.747669000	38.036369000	41.936297000
Si	43.620762000	38.874819000	44.077347000
Si	47.727394000	38.474881000	46.632346000
Si	48.750698000	38.732589000	43.625016000

Si	46.086958000	38.103071000	42.375139000
Si	51.742425000	38.023920000	43.477456000
Si	53.671091000	38.783443000	41.342555000
Si	56.182150000	38.110635000	43.088663000
Si	44.600059000	50.307164000	33.748219000
Si	47.749443000	50.285833000	33.587143000
Si	48.736514000	50.072173000	30.546726000
Si	43.866240000	46.933052000	30.717280000
Si	48.871676000	46.969349000	30.446812000
Si	46.328713000	45.968640000	29.030281000
Si	48.548649000	52.794050000	35.340077000
Si	46.538632000	51.834901000	37.404854000
Si	44.110480000	52.726588000	35.650421000
Si	43.868689000	41.885906000	30.713965000
Si	48.881976000	41.863323000	30.429356000
Si	46.328431000	42.857204000	29.025099000
Si	51.884875000	42.865285000	30.522512000
Si	57.979132000	42.907328000	39.185549000
Si	58.734589000	47.856940000	36.908187000
Si	56.881345000	48.677747000	34.755308000
Si	55.871693000	48.297915000	31.973988000
Si	52.770649000	48.323710000	32.242828000
Si	56.558655000	51.799067000	34.995907000
Si	52.600830000	52.738379000	32.657986000
Si	51.488375000	51.660433000	35.474214000
Si	54.134372000	52.729039000	36.713304000
Si	51.808814000	50.697539000	30.334562000
Si	54.677972000	50.358720000	38.531882000
Si	57.777907000	50.330597000	38.801969000
Si	51.885900000	45.957382000	30.527490000
Si	57.978284000	45.923650000	39.181346000
Si	58.734521000	40.966201000	36.912109000
Si	56.871563000	40.144965000	34.761103000
Si	55.870800000	40.523735000	31.973089000
Si	52.764627000	40.496244000	32.239069000
Si	48.687225000	40.960739000	48.532902000
Si	41.752216000	50.760741000	41.945654000
Si	43.770364000	49.960648000	44.080673000
Si	47.724385000	50.357808000	46.615301000
Si	48.754778000	50.177780000	43.580530000
Si	46.078432000	50.906210000	42.392112000
Si	44.897556000	46.011282000	46.543869000
Si	47.943813000	46.042909000	46.133026000
Si	48.699623000	47.885096000	48.522271000
Si	45.604152000	52.791280000	40.058154000
Si	44.930779000	42.786827000	46.529161000
Si	47.980007000	42.799570000	46.182685000
Si	58.960877000	41.840866000	41.943747000
Si	56.330612000	42.838680000	43.260300000
Si	55.790015000	48.281623000	45.174354000
Si	52.625976000	48.320872000	45.339412000
Si	51.675027000	48.630403000	48.362860000
Si	51.742346000	50.818996000	43.468348000
Si	53.668767000	50.032128000	41.340201000
Si	56.181702000	50.707209000	43.090962000
Si	58.962505000	46.981688000	41.930862000
Si	56.326863000	45.967224000	43.253738000
Si	55.794234000	40.523504000	45.179219000

Si	52.672494000	40.490838000	45.364777000
Si	51.673213000	40.192517000	48.359412000
O	49.977390000	36.642095000	35.871128000
O	47.522420000	35.859637000	36.595887000
O	46.656475000	36.830654000	38.966654000
O	44.963866000	36.613837000	36.942497000
O	44.502706000	36.939867000	34.263753000
O	46.181661000	38.829973000	33.243198000
O	47.914198000	37.002130000	34.159632000
O	48.622676000	38.600215000	32.143059000
O	50.190691000	38.133292000	30.055497000
O	52.211375000	36.839971000	31.253877000
O	52.286742000	39.556125000	30.987219000
O	54.608900000	36.887013000	38.076970000
O	56.173938000	38.736441000	39.133890000
O	56.912449000	38.600887000	35.312848000
O	54.991130000	36.660258000	35.417653000
O	51.808659000	36.852239000	33.908586000
O	52.533875000	36.329411000	36.437781000
O	42.413939000	37.948204000	43.445414000
O	45.094664000	38.280294000	43.648170000
O	48.586643000	38.727086000	45.246739000
O	47.596449000	37.817608000	42.904215000
O	45.603866000	36.831694000	41.481462000
O	50.133000000	37.969513000	43.224615000
O	52.431083000	37.937727000	41.981898000
O	52.151560000	39.425230000	44.237474000
O	53.571855000	38.737661000	39.708190000
O	55.087823000	38.125629000	41.848789000
O	44.495338000	51.863234000	34.283282000
O	46.158542000	49.934754000	33.330353000
O	47.881064000	51.808094000	34.190506000
O	48.565570000	50.188229000	32.176650000
O	48.591924000	48.521008000	30.019090000
O	45.411611000	46.589636000	30.244202000
O	47.902280000	46.007293000	29.531222000
O	45.889391000	44.412920000	28.740854000
O	49.984976000	52.179412000	35.850706000
O	47.549885000	52.893105000	36.655756000
O	46.626853000	51.947456000	39.043094000
O	44.993487000	52.179058000	36.948902000
O	48.585189000	40.309969000	30.019112000
O	45.410456000	42.225715000	30.231768000
O	47.902908000	42.821901000	29.523107000
O	50.437094000	42.218686000	30.084371000
O	51.665256000	44.410113000	31.025529000
O	58.602115000	44.415913000	39.363252000
O	58.128723000	47.831850000	35.380064000
O	58.294646000	46.469428000	37.673094000
O	58.216309000	49.187241000	37.704937000
O	57.003942000	48.629416000	33.131341000
O	56.918229000	50.220622000	35.309599000
O	54.351937000	48.581305000	32.580410000
O	52.566245000	46.757428000	31.798393000
O	54.994350000	52.158114000	35.420300000
O	51.811618000	51.955994000	33.891056000
O	52.534292000	52.500788000	36.420136000
O	50.185375000	50.698753000	30.124179000

O	52.238513000	51.991296000	31.238219000
O	52.302315000	49.267828000	30.989672000
O	54.595360000	51.933969000	38.078210000
O	56.173656000	50.089724000	39.132029000
O	50.429737000	46.604791000	30.121573000
O	58.114273000	40.995543000	35.389224000
O	58.308095000	42.353919000	37.682917000
O	58.215197000	39.635350000	37.706760000
O	56.996393000	40.193188000	33.136946000
O	54.347098000	40.241526000	32.568776000
O	52.546745000	42.061912000	31.801602000
O	50.286821000	40.906895000	48.866093000
O	48.395422000	42.362517000	47.707830000
O	48.196014000	39.621349000	47.718345000
O	42.503697000	50.783894000	43.418024000
O	45.197818000	50.711641000	43.750479000
O	48.530277000	50.110342000	45.194368000
O	47.633668000	51.094416000	42.820784000
O	45.566270000	52.231404000	41.604390000
O	44.933672000	44.401628000	46.826608000
O	46.373369000	46.521596000	46.008684000
O	48.109219000	44.422716000	46.007318000
O	50.289144000	47.937117000	48.901019000
O	48.473218000	46.527749000	47.606407000
O	48.229755000	49.233449000	47.708417000
O	46.414297000	42.327916000	45.956985000
O	58.660338000	41.941264000	40.312269000
O	57.925321000	42.742765000	42.877490000
O	55.891853000	44.403178000	43.427340000
O	56.011794000	46.720896000	44.680492000
O	54.230975000	48.533313000	45.658235000
O	56.153970000	49.271996000	43.910524000
O	51.780183000	48.550133000	46.713717000
O	50.137290000	50.958276000	43.237800000
O	52.427382000	50.883166000	41.972462000
O	52.097562000	49.405575000	44.232466000
O	53.575161000	50.071662000	39.706522000
O	55.084438000	50.686271000	41.853310000
O	58.674927000	46.894989000	40.295744000
O	57.912114000	46.068851000	42.836438000
O	55.977788000	42.087259000	44.678683000
O	54.262685000	40.226320000	45.712381000
O	56.156478000	39.548265000	43.903567000
O	51.788410000	40.261733000	46.712195000
H	48.702871000	34.676568000	34.772566000
H	44.419875000	34.676323000	35.346829000
H	42.683628000	36.258975000	36.022159000
H	43.726523000	38.680216000	32.577941000
H	47.730594000	38.010996000	29.830569000
H	52.421136000	38.002380000	29.030381000
H	58.465353000	38.743363000	40.066967000
H	58.088382000	37.108509000	38.286195000
H	56.651061000	36.758841000	33.558678000
H	57.541582000	36.246995000	35.782467000
H	52.141234000	34.673922000	32.562045000
H	54.071037000	36.146097000	32.821438000
H	54.418082000	34.675478000	36.917895000
H	44.265382000	36.148734000	39.494230000

H	46.085045000	34.677316000	40.196440000
H	42.177304000	36.886192000	41.136491000
H	40.290195000	38.107003000	42.090377000
H	43.614692000	38.689573000	45.487247000
H	46.266760000	38.594605000	46.380864000
H	48.065374000	37.113072000	47.131272000
H	52.195251000	36.881484000	44.271734000
H	55.858296000	36.988842000	44.011824000
H	57.529558000	37.965437000	42.519250000
H	41.636484000	38.780154000	36.634495000
H	40.338249000	40.845235000	36.817795000
H	43.726296000	50.140518000	32.578071000
H	47.730503000	50.809921000	29.830551000
H	42.910974000	46.071594000	29.969678000
H	43.636013000	48.346500000	30.474316000
H	46.055518000	46.690812000	27.779371000
H	40.338195000	47.975592000	36.817799000
H	41.636403000	50.040687000	36.634467000
H	48.702714000	54.144377000	34.772532000
H	44.419585000	54.144606000	35.346948000
H	42.683575000	52.561616000	36.022276000
H	43.636069000	40.474361000	30.474327000
H	42.910998000	42.749253000	29.969681000
H	46.055551000	42.130076000	27.779377000
H	52.768240000	42.805898000	29.339147000
H	60.152183000	47.950749000	36.769108000
H	56.115708000	49.276599000	30.928772000
H	56.049418000	46.904457000	31.507081000
H	57.541465000	52.574079000	35.782446000
H	56.650952000	52.062214000	33.558656000
H	52.141090000	54.147065000	32.562019000
H	54.070917000	52.674925000	32.821418000
H	54.418140000	54.145525000	36.917811000
H	52.421044000	50.818602000	29.030363000
H	58.465269000	50.077736000	40.066952000
H	58.088278000	51.712582000	38.286179000
H	52.768217000	46.015092000	29.339142000
H	60.152234000	40.870368000	36.769119000
H	56.049458000	41.916585000	31.507090000
H	56.115777000	39.544444000	30.928783000
H	48.027948000	40.958435000	49.819978000
H	40.290115000	50.713683000	42.090582000
H	42.176850000	51.934815000	41.136521000
H	43.615015000	50.131729000	45.487215000
H	48.065229000	51.707918000	47.131232000
H	46.266685000	50.226321000	46.380825000
H	40.476352000	46.423809000	42.229530000
H	44.578030000	46.685732000	47.785515000
H	48.027871000	47.862537000	49.819961000
H	46.084739000	54.143668000	40.196331000
H	44.265252000	52.672179000	39.494179000
H	40.476382000	42.397038000	42.229542000
H	44.578054000	42.135174000	47.785513000
H	58.818796000	40.408567000	42.221796000
H	60.309668000	42.345550000	42.255160000
H	56.725235000	48.548903000	46.274187000
H	51.671681000	50.045758000	48.769745000
H	52.721673000	47.848745000	49.007593000

H	52.195144000	51.939533000	44.271718000
H	55.858180000	51.832229000	44.011806000
H	57.529465000	50.855664000	42.519236000
H	60.309639000	46.475584000	42.255156000
H	58.818736000	48.412544000	42.221786000
H	56.725177000	40.272281000	46.274313000
H	52.722235000	40.971966000	49.006966000
H	51.671815000	38.775269000	48.769821000
Si	48.764478000	40.977135000	35.384597000
Si	46.854381000	39.950125000	37.387316000
Si	41.597968000	40.170741000	37.158386000
Si	44.279557000	40.872222000	35.828283000
Si	44.621210000	45.938689000	33.477052000
Si	47.807577000	45.951609000	33.218380000
Si	48.769613000	47.838804000	35.451845000
Si	46.863826000	48.756732000	37.490579000
Si	41.562189000	48.679806000	37.186785000
Si	44.242379000	47.946654000	35.872364000
Si	44.663676000	42.886921000	33.448096000
Si	47.826313000	42.905501000	33.187628000
Si	54.824663000	42.898649000	38.819936000
Si	51.691157000	48.538632000	35.167751000
Si	54.402855000	47.960057000	36.439528000
Si	54.826748000	45.933399000	38.823209000
Si	51.686945000	40.279652000	35.166047000
Si	54.393354000	40.862347000	36.445227000
Si	45.856215000	40.384153000	40.181291000
Si	42.711082000	40.470212000	40.064582000
Si	41.758986000	45.964820000	41.681303000
Si	43.950871000	46.754156000	43.820780000
Si	48.937939000	47.052623000	43.300306000
Si	46.292871000	45.982023000	41.980131000
Si	45.637676000	48.529361000	40.241450000
Si	42.598975000	48.406186000	40.012486000
Si	41.756601000	42.920440000	41.763512000
Si	43.833636000	42.025895000	43.806199000
Si	49.014626000	41.841009000	43.453500000
Al	46.442589000	42.925212000	42.045930000
Si	51.896941000	42.859226000	43.536524000
Si	53.970112000	41.873532000	41.522016000
Si	51.815338000	45.929340000	43.488935000
Si	53.938843000	46.944276000	41.521683000
O	46.916575000	38.427248000	36.779010000
O	44.188740000	39.495493000	34.940757000
O	54.383442000	39.428258000	37.245636000
O	51.573391000	38.769916000	35.804486000
O	42.349516000	39.397746000	41.230625000
O	50.370752000	41.190021000	35.493099000
O	48.168904000	40.793388000	36.903110000
O	48.079839000	42.288383000	34.685206000
O	48.415752000	39.623706000	34.537212000
O	46.872119000	39.858206000	39.017089000
O	45.493549000	40.728446000	36.919939000
O	41.751594000	40.094230000	38.798751000
O	42.838395000	41.092871000	36.557782000
O	44.605613000	42.179061000	34.912116000
O	44.097798000	49.309144000	34.962515000
O	43.724682000	46.735745000	32.358182000

O	44.069445000	44.402246000	33.575077000
O	46.187824000	45.941043000	32.992390000
O	48.410314000	44.431209000	33.114142000
O	48.555195000	46.842394000	32.062571000
O	50.380757000	47.633618000	35.519738000
O	48.201887000	47.959539000	36.985903000
O	48.087972000	46.545112000	34.716214000
O	48.417438000	49.206289000	34.617686000
O	46.811740000	48.723995000	39.120671000
O	46.940077000	50.311710000	36.944653000
O	45.528709000	48.057444000	36.863968000
O	41.567069000	48.846773000	38.829146000
O	42.858215000	47.758554000	36.720651000
O	44.454976000	46.624456000	34.939790000
O	43.746074000	42.071722000	32.359857000
O	46.213962000	42.919810000	32.915722000
O	48.599327000	42.014236000	32.049752000
O	54.246458000	44.416379000	38.612000000
O	56.357158000	42.959008000	39.387873000
O	55.468045000	47.986937000	35.205300000
O	51.851461000	48.691331000	33.541328000
O	52.923899000	47.691534000	35.815823000
O	51.591768000	50.052634000	35.798914000
O	54.822471000	46.717606000	37.401093000
O	54.389640000	49.395180000	37.238631000
O	56.357255000	45.876796000	39.401601000
O	55.452578000	40.829416000	35.205593000
O	51.858984000	40.116566000	33.543496000
O	52.909819000	41.128374000	35.828009000
O	54.812149000	42.110449000	37.399177000
O	46.395672000	41.939032000	40.507223000
O	44.307497000	40.340499000	39.656023000
O	46.034120000	39.510397000	41.527631000
O	42.441977000	41.995562000	40.581588000
O	42.232630000	49.320249000	41.306423000
O	43.880677000	48.382123000	43.592662000
O	48.730049000	48.648237000	42.978112000
O	42.783384000	46.022202000	42.961933000
O	41.594618000	44.427253000	41.130664000
O	43.762520000	46.416577000	45.415340000
O	45.397477000	46.104399000	43.352380000
O	48.767593000	46.773708000	44.914223000
O	47.860448000	46.221745000	42.382274000
O	46.125232000	44.506531000	41.251763000
O	45.702488000	47.041791000	40.895917000
O	44.153626000	48.716384000	39.547711000
O	45.869923000	49.606159000	41.431474000
O	42.374326000	46.832555000	40.415880000
O	43.411215000	40.442418000	43.632804000
O	48.658127000	40.274018000	43.066811000
O	42.694408000	42.975653000	43.106658000
O	43.804083000	42.381391000	45.411572000
O	45.259815000	42.306698000	43.113288000
O	48.945665000	42.027510000	45.102877000
O	48.031311000	42.887499000	42.715846000
O	53.545383000	40.335140000	41.868568000
O	50.564818000	42.101383000	42.971474000
O	52.982649000	42.913789000	42.303365000

O	51.563860000	44.395457000	44.009684000
O	53.885538000	42.095140000	39.898382000
O	55.512312000	42.124323000	42.026557000
O	52.375937000	46.803685000	44.763127000
O	53.526746000	48.485170000	41.877240000
O	50.443973000	46.583043000	42.865807000
O	52.919133000	45.913682000	42.273173000
O	53.880980000	46.737473000	39.895571000
O	55.469561000	46.678818000	42.047379000
O	52.520884000	42.032945000	44.814886000
Ga	45.936173000	44.423414000	39.065245000
H	46.207692000	45.877516000	38.542538000
H	44.579471000	43.631989000	38.914219000
C	47.790838000	43.477568000	38.732842000
H	48.136989000	42.420811000	38.737164000
H	47.713342000	43.652612000	37.635225000
H	46.894290000	42.558061000	39.684310000
C	48.916517000	44.338608000	39.342056000
H	49.886111000	44.135056000	38.849504000
H	49.053540000	44.137959000	40.420392000
H	48.719221000	45.421176000	39.230287000

I2-GaH$_2$

Electronic energy (r^{2}SCAN-D4/def2-SVP): -48258.41279055 E_h
Electronic energy (ωB97X-D4rev/def2-mTZVPP): -48302.155155995900 E_h
G_{corr} (673.15 K, 1 atm): 1.99090993 E_h
Number of atoms: 387

Cartesian coordinates (Å)

Si	48.560268000	36.037862000	35.302953000
Si	46.531176000	36.996985000	37.352948000
Si	44.103909000	36.095353000	35.635342000
Si	44.615714000	38.502726000	33.732289000
Si	47.769188000	38.551693000	33.539584000
Si	48.750634000	38.761431000	30.505565000
Si	51.809277000	38.119084000	30.330052000
Si	54.677263000	38.466734000	38.545168000
Si	57.775519000	38.489348000	38.805296000
Si	56.558837000	37.023271000	34.996121000
Si	52.600458000	36.082606000	32.661845000
Si	51.488715000	37.161299000	35.480118000
Si	54.137025000	36.094540000	36.716342000
Si	45.621743000	36.074109000	40.038594000
Si	41.737203000	38.001992000	41.955331000
Si	43.506668000	38.854529000	44.085065000
Si	47.727540000	38.461777000	46.629025000
Si	48.753253000	38.669133000	43.626278000
Si	46.044067000	38.185674000	42.338589000
Si	51.694412000	37.981257000	43.459059000
Si	53.647568000	38.778092000	41.352957000
Si	56.183342000	38.106246000	43.085561000
Si	44.590191000	50.336790000	33.732254000
Si	47.752189000	50.264396000	33.566348000
Si	48.741539000	50.063478000	30.522322000
Si	43.868755000	46.935391000	30.713512000
Si	48.883930000	46.967020000	30.405477000

Si	46.325073000	45.965378000	29.024275000
Si	48.560456000	52.792360000	35.316808000
Si	46.566812000	51.849657000	37.358914000
Si	44.109985000	52.723526000	35.636006000
Si	43.867491000	41.890131000	30.711807000
Si	48.874235000	41.849483000	30.407884000
Si	46.324670000	42.856144000	29.024541000
Si	51.890525000	42.867180000	30.529947000
Si	57.968922000	42.906240000	39.187525000
Si	58.733593000	47.858743000	36.908829000
Si	56.882307000	48.676118000	34.757720000
Si	55.870175000	48.297469000	31.973637000
Si	52.766174000	48.330286000	32.241871000
Si	56.558290000	51.798914000	34.995771000
Si	52.600285000	52.739088000	32.662051000
Si	51.487693000	51.662392000	35.480880000
Si	54.137007000	52.727669000	36.715665000
Si	51.809050000	50.702863000	30.333720000
Si	54.679749000	50.355858000	38.541092000
Si	57.777017000	50.331651000	38.803885000
Si	51.889501000	45.955439000	30.525301000
Si	57.968207000	45.927289000	39.181760000
Si	58.734707000	40.964579000	36.914625000
Si	56.882146000	40.147532000	34.761207000
Si	55.874461000	40.523489000	31.974037000
Si	52.772045000	40.488945000	32.234367000
Si	48.660556000	40.942777000	48.517592000
Si	41.741882000	50.803915000	41.940745000
Si	43.553323000	49.981536000	44.077599000
Si	47.727324000	50.359441000	46.621751000
Si	48.762452000	50.160067000	43.607390000
Si	46.056430000	50.634932000	42.322851000
Si	44.786036000	45.958857000	46.546908000
Si	47.932722000	45.962080000	46.169355000
Si	48.665760000	47.878126000	48.517500000
Si	45.618281000	52.751978000	40.039749000
Si	44.771249000	42.854711000	46.543775000
Si	47.928505000	42.838006000	46.158774000
Si	58.955898000	41.841406000	41.948833000
Si	56.315688000	42.833268000	43.274682000
Si	55.800764000	48.299347000	45.159903000
Si	52.634073000	48.368108000	45.310305000
Si	51.673684000	48.627287000	48.345313000
Si	51.703538000	50.845843000	43.452115000
Si	53.649582000	50.040598000	41.348152000
Si	56.183700000	50.710448000	43.088388000
Si	58.957981000	46.981575000	41.931043000
Si	56.310736000	45.965972000	43.263601000
Si	55.796821000	40.498691000	45.164125000
Si	52.662182000	40.431514000	45.327753000
Si	51.670812000	40.194770000	48.347184000
O	49.985496000	36.638915000	35.857301000
O	47.519040000	35.940370000	36.581198000
O	46.653655000	36.851809000	38.979652000
O	44.975930000	36.673874000	36.926367000
O	44.494480000	36.945754000	34.261293000
O	46.168888000	38.861269000	33.288914000
O	47.939137000	37.030535000	34.134688000

O	48.587866000	38.683079000	32.137016000
O	50.189375000	38.107580000	30.095835000
O	52.234367000	36.832648000	31.244709000
O	52.296455000	39.554065000	30.977399000
O	54.598374000	36.894735000	38.077077000
O	56.173351000	38.725703000	39.149632000
O	56.916122000	38.602184000	35.309270000
O	54.994180000	36.663430000	35.420381000
O	51.811800000	36.861584000	33.897739000
O	52.536083000	36.325788000	36.427010000
O	42.368763000	37.834935000	43.473467000
O	45.001132000	38.432356000	43.555144000
O	48.591685000	38.720700000	45.248950000
O	47.515819000	37.844435000	42.939471000
O	45.571051000	36.891085000	41.465131000
O	50.084784000	37.804313000	43.240189000
O	52.384006000	37.939962000	41.961121000
O	52.040208000	39.406880000	44.211088000
O	53.569919000	38.742498000	39.717068000
O	55.055179000	38.112343000	41.875348000
O	44.488009000	51.900094000	34.242412000
O	46.150048000	49.941131000	33.348662000
O	47.921243000	51.780094000	34.175016000
O	48.549036000	50.159513000	32.148723000
O	48.615918000	48.519110000	29.969804000
O	45.413902000	46.596604000	30.236304000
O	47.903308000	46.002390000	29.507111000
O	45.877296000	44.410557000	28.745484000
O	49.986972000	52.187431000	35.863698000
O	47.527272000	52.939514000	36.597767000
O	46.661209000	51.992833000	38.986915000
O	45.005750000	52.116030000	36.901960000
O	48.621455000	40.302248000	29.944083000
O	45.415800000	42.224024000	30.239554000
O	47.903313000	42.821277000	29.506585000
O	50.430568000	42.237088000	30.113310000
O	51.686068000	44.411904000	31.038506000
O	58.587227000	44.417427000	39.358635000
O	58.136989000	47.837073000	35.376763000
O	58.284180000	46.473777000	37.672940000
O	58.208173000	49.188115000	37.703774000
O	56.999227000	48.628757000	33.133402000
O	56.913638000	50.219995000	35.309603000
O	54.349100000	48.578704000	32.577764000
O	52.546519000	46.766126000	31.801029000
O	54.993997000	52.159214000	35.419820000
O	51.808994000	51.960041000	33.897003000
O	52.536606000	52.498455000	36.425928000
O	50.186213000	50.710619000	30.120514000
O	52.238690000	51.992661000	31.241819000
O	52.298559000	49.271594000	30.986872000
O	54.598817000	51.928793000	38.077615000
O	56.174550000	50.095223000	39.147594000
O	50.436912000	46.585736000	30.082965000
O	58.131298000	40.991450000	35.385017000
O	58.299795000	42.349537000	37.685886000
O	58.206025000	39.634983000	37.706613000
O	57.001921000	40.197070000	33.136637000

O	54.353019000	40.236098000	32.573252000
O	52.557454000	42.056246000	31.801213000
O	50.277110000	40.950234000	48.783131000
O	48.245898000	42.295771000	47.671417000
O	48.196413000	39.570443000	47.749634000
O	42.396426000	50.966431000	43.452954000
O	45.056384000	50.419389000	43.587808000
O	48.570195000	50.103666000	45.225846000
O	47.544560000	50.976459000	42.884736000
O	45.557502000	51.925331000	41.461256000
O	44.427874000	44.412192000	46.919764000
O	46.310606000	46.080577000	45.926563000
O	48.417364000	44.403970000	46.022435000
O	50.279709000	47.877104000	48.790253000
O	48.266615000	46.520238000	47.670216000
O	48.204575000	49.242405000	47.730902000
O	46.300858000	42.752401000	45.924454000
O	58.655295000	41.947706000	40.317395000
O	57.909348000	42.729242000	42.885052000
O	55.894594000	44.400236000	43.462013000
O	55.999534000	46.729605000	44.686910000
O	54.239692000	48.614825000	45.596677000
O	56.205731000	49.265346000	43.890278000
O	51.822773000	48.596726000	46.701673000
O	50.094183000	51.022032000	43.232113000
O	52.393140000	50.891547000	41.956415000
O	52.035073000	49.420784000	44.207487000
O	53.572577000	50.075851000	39.713197000
O	55.059549000	50.696472000	41.874532000
O	58.674563000	46.893829000	40.294742000
O	57.891366000	46.078248000	42.828270000
O	55.953617000	42.073097000	44.687321000
O	54.256796000	40.125517000	45.626097000
O	56.214629000	39.553405000	43.883374000
O	51.824068000	40.220501000	46.704553000
H	48.702872000	34.676574000	34.772695000
H	44.419837000	34.676337000	35.346997000
H	42.683622000	36.259035000	36.022304000
H	43.726523000	38.680140000	32.577986000
H	47.730566000	38.010963000	29.830644000
H	52.421097000	38.002332000	29.030449000
H	58.465363000	38.743461000	40.067008000
H	58.088407000	37.108576000	38.286260000
H	56.651050000	36.758847000	33.558744000
H	57.541580000	36.247026000	35.782535000
H	52.141238000	34.673908000	32.562133000
H	54.071025000	36.146108000	32.821519000
H	54.418092000	34.675512000	36.917976000
H	44.265409000	36.148773000	39.494358000
H	46.085094000	34.677378000	40.196556000
H	42.177287000	36.886195000	41.136614000
H	40.290210000	38.107052000	42.090488000
H	43.614706000	38.689677000	45.487338000
H	46.266824000	38.594739000	46.380986000
H	48.065446000	37.113219000	47.131387000
H	52.195292000	36.881547000	44.271808000
H	55.858356000	36.988960000	44.011896000
H	57.529603000	37.965547000	42.519304000

H	41.636483000	38.780153000	36.634499000
H	40.338248000	40.845237000	36.817801000
H	43.726280000	50.140516000	32.578074000
H	47.730453000	50.809900000	29.830539000
H	42.910941000	46.071572000	29.969701000
H	43.635982000	48.346486000	30.474316000
H	46.055479000	46.690763000	27.779386000
H	40.338194000	47.975594000	36.817801000
H	41.636404000	50.040688000	36.634469000
H	48.702699000	54.144389000	34.772484000
H	44.419581000	54.144625000	35.346923000
H	42.683570000	52.561646000	36.022274000
H	43.636041000	40.474333000	30.474367000
H	42.910980000	42.749228000	29.969696000
H	46.055512000	42.130045000	27.779417000
H	52.768211000	42.805890000	29.339169000
H	60.152177000	47.950787000	36.769057000
H	56.115676000	49.276586000	30.928723000
H	56.049384000	46.904449000	31.507055000
H	57.541452000	52.574116000	35.782345000
H	56.650921000	52.062220000	33.558569000
H	52.141038000	54.147058000	32.561958000
H	54.070881000	52.674937000	32.821346000
H	54.418129000	54.145558000	36.917733000
H	52.420994000	50.818569000	29.030320000
H	58.465296000	50.077834000	40.066871000
H	58.088268000	51.712649000	38.286076000
H	52.768181000	46.015065000	29.339143000
H	60.152240000	40.870417000	36.769122000
H	56.049448000	41.916581000	31.507103000
H	56.115758000	39.544437000	30.928811000
H	48.028025000	40.958621000	49.820049000
H	40.290146000	50.713752000	42.090580000
H	42.176904000	51.934830000	41.136499000
H	43.615044000	50.131763000	45.487201000
H	48.065315000	51.708061000	47.131181000
H	46.266740000	50.226474000	46.380825000
H	40.476354000	46.423812000	42.229533000
H	44.578011000	46.685825000	47.785528000
H	48.027950000	47.862710000	49.819959000
H	46.084765000	54.143716000	40.196310000
H	44.265268000	52.672236000	39.494164000
H	40.476383000	42.397041000	42.229547000
H	44.578082000	42.135259000	47.785528000
H	58.818858000	40.408668000	42.221812000
H	60.309695000	42.345680000	42.255152000
H	56.725264000	48.549041000	46.274147000
H	51.671746000	50.045934000	48.769665000
H	52.721746000	47.848930000	49.007575000
H	52.195166000	51.939599000	44.271676000
H	55.858205000	51.832343000	44.011722000
H	57.529486000	50.855767000	42.519150000
H	60.309663000	46.475702000	42.255101000
H	58.818737000	48.412645000	42.221722000
H	56.725244000	40.272425000	46.274338000
H	52.722315000	40.972162000	49.006999000
H	51.671901000	38.775451000	48.769946000
Si	48.786393000	41.021524000	35.379853000

Si	46.881520000	40.082431000	37.410748000
Si	41.572878000	40.146146000	37.193178000
Si	44.247745000	40.879953000	35.848386000
Si	44.657481000	45.961684000	33.464314000
Si	47.832245000	45.918669000	33.147538000
Si	48.779422000	47.784039000	35.388434000
Si	46.893499000	48.787202000	37.424885000
Si	41.580571000	48.666593000	37.176335000
Si	44.230602000	48.000535000	35.828321000
Si	44.618404000	42.906029000	33.463805000
Si	47.803953000	42.881738000	33.150066000
Si	54.811091000	42.895485000	38.833951000
Si	51.694418000	48.543300000	35.166590000
Si	54.405827000	47.950836000	36.447143000
Si	54.813021000	45.935641000	38.838462000
Si	51.699502000	40.276418000	35.154870000
Si	54.402801000	40.872854000	36.448649000
Si	45.744257000	40.477878000	40.162845000
Si	42.655683000	40.402630000	40.056213000
Si	41.757536000	45.912707000	41.721527000
Si	43.753681000	46.912885000	43.818765000
Si	48.914016000	47.043214000	43.417636000
Si	46.205576000	46.014077000	42.160356000
Si	45.782330000	48.278472000	40.187766000
Si	42.670321000	48.390187000	40.049050000
Si	41.773918000	42.884713000	41.740740000
Si	43.733716000	41.885671000	43.863394000
Si	48.964495000	41.777083000	43.420325000
Al	46.244126000	42.825195000	42.282544000
Si	51.898111000	42.861903000	43.567934000
Si	53.944222000	41.867680000	41.529719000
Si	51.823250000	45.914496000	43.533924000
Si	53.903543000	46.948593000	41.527673000
O	46.931697000	38.524540000	36.889122000
O	44.147510000	39.499661000	34.962768000
O	54.396783000	39.445292000	37.260779000
O	51.578728000	38.770958000	35.802573000
O	42.374232000	39.364420000	41.273496000
O	50.395916000	41.204063000	35.471428000
O	48.203589000	40.897334000	36.907021000
O	48.122194000	42.329546000	34.657482000
O	48.407322000	39.650441000	34.567669000
O	46.875923000	40.156757000	39.039039000
O	45.528083000	40.815281000	36.861436000
O	41.640020000	40.008242000	38.839537000
O	42.856051000	41.061268000	36.681589000
O	44.459072000	42.196155000	34.914708000
O	44.021616000	49.357384000	34.928235000
O	43.739640000	46.749104000	32.356518000
O	44.075327000	44.440412000	33.600570000
O	46.210641000	45.928156000	32.942792000
O	48.405363000	44.394278000	32.972050000
O	48.572991000	46.851892000	32.022561000
O	50.391588000	47.624097000	35.508955000
O	48.160770000	47.900928000	36.899549000
O	48.156205000	46.460772000	34.656163000
O	48.410404000	49.147350000	34.559927000
O	46.883790000	48.674118000	39.052763000

O	47.073217000	50.347081000	36.928890000
O	45.472707000	48.244001000	36.846739000
O	41.683658000	48.808480000	38.819408000
O	42.838956000	47.728680000	36.639103000
O	44.568227000	46.695733000	34.911559000
O	43.714526000	42.114667000	32.347445000
O	46.180158000	42.895510000	32.967558000
O	48.524565000	41.937767000	32.019883000
O	54.228822000	44.416228000	38.657645000
O	56.347385000	42.953052000	39.393125000
O	55.476231000	47.977187000	35.217182000
O	51.853566000	48.709561000	33.541462000
O	52.929038000	47.699846000	35.812107000
O	51.582473000	50.053677000	35.804476000
O	54.804561000	46.696250000	37.402235000
O	54.399418000	49.381727000	37.254127000
O	56.347473000	45.888550000	39.409236000
O	55.473480000	40.842111000	35.218999000
O	51.862878000	40.099186000	33.532446000
O	52.927967000	41.121449000	35.807597000
O	54.794704000	42.133860000	37.398196000
O	45.897906000	42.104735000	40.596684000
O	44.233335000	40.231021000	39.572923000
O	46.079665000	39.576226000	41.458820000
O	42.410966000	41.947724000	40.534935000
O	42.366275000	49.437711000	41.257659000
O	43.164862000	48.431611000	43.678645000
O	48.842056000	48.630903000	43.014151000
O	42.798101000	45.878851000	42.997542000
O	41.587214000	44.396754000	41.114985000
O	43.752549000	46.521784000	45.402560000
O	45.271524000	46.901520000	43.181091000
O	48.703246000	46.880647000	45.045763000
O	47.771964000	46.210059000	42.594392000
O	45.758138000	44.479222000	42.061444000
O	46.020849000	46.668839000	40.600424000
O	44.259992000	48.504287000	39.616186000
O	46.066863000	49.229252000	41.475216000
O	42.377163000	46.849415000	40.514779000
O	43.051755000	40.393462000	43.727200000
O	48.812541000	40.185728000	43.021581000
O	42.797565000	42.942242000	43.021289000
O	43.684471000	42.302382000	45.451345000
O	45.247371000	41.856756000	43.305741000
O	48.731948000	41.926406000	45.060926000
O	47.934803000	42.693460000	42.589542000
O	53.535547000	40.326918000	41.892155000
O	50.500715000	42.213307000	43.048314000
O	52.929771000	42.899478000	42.285880000
O	51.688183000	44.392097000	44.114427000
O	53.878964000	42.069761000	39.901828000
O	55.480664000	42.143117000	42.039299000
O	52.413820000	46.841568000	44.754198000
O	53.513500000	48.494910000	41.890830000
O	50.378004000	46.460094000	42.989077000
O	52.847880000	45.932771000	42.248897000
O	53.869015000	46.759311000	39.897979000
O	55.423980000	46.652471000	42.065079000

O	52.540687000	41.979647000	44.796508000
Ga	46.690314000	45.139294000	38.927654000
H	46.076448000	45.960618000	37.738807000
H	45.655385000	43.855861000	39.131283000
C	48.582747000	44.927422000	39.487096000
H	48.977207000	45.940372000	39.706304000
H	48.657222000	44.358161000	40.434885000
H	45.803539000	42.880956000	39.866632000
C	49.436699000	44.250771000	38.397004000
H	50.499969000	44.197754000	38.701824000
H	49.408827000	44.799941000	37.436366000
H	49.112562000	43.212960000	38.193621000

TS2-GaH$_2$

Electronic energy (r^{2}SCAN-D4/def2-SVP): -48258.41133956 E_h
Electronic energy (ωB97X-D4rev/def2-mTZVPP): -48302.153431844600 E_h
G_{corr} (673.15 K, 1 atm): 1.99207415 E_h
Number of atoms: 387

Cartesian coordinates (Å)

Si	48.558624000	36.037889000	35.301329000
Si	46.529832000	36.996036000	37.352754000
Si	44.103743000	36.095038000	35.636022000
Si	44.616456000	38.502252000	33.732028000
Si	47.769408000	38.550592000	33.537562000
Si	48.751574000	38.760456000	30.504188000
Si	51.808993000	38.118113000	30.330009000
Si	54.676422000	38.466771000	38.544768000
Si	57.775231000	38.489001000	38.805331000
Si	56.558179000	37.022898000	34.996207000
Si	52.600468000	36.082505000	32.661948000
Si	51.487531000	37.160678000	35.479575000
Si	54.135712000	36.094017000	36.715726000
Si	45.621891000	36.074326000	40.038726000
Si	41.738764000	38.005348000	41.953511000
Si	43.517061000	38.853235000	44.083023000
Si	47.727560000	38.461640000	46.625386000
Si	48.749678000	38.674905000	43.619101000
Si	46.043362000	38.191636000	42.329133000
Si	51.694414000	37.982480000	43.459742000
Si	53.647955000	38.778207000	41.353003000
Si	56.183031000	38.106294000	43.085468000
Si	44.595740000	50.339448000	33.727822000
Si	47.755970000	50.262167000	33.561067000
Si	48.743471000	50.062291000	30.519403000
Si	43.869667000	46.935352000	30.712083000
Si	48.885478000	46.965623000	30.401901000
Si	46.325183000	45.964161000	29.023081000
Si	48.563390000	52.790517000	35.315049000
Si	46.563594000	51.845290000	37.357130000
Si	44.109232000	52.723733000	35.634208000
Si	43.866932000	41.889709000	30.712444000
Si	48.874372000	41.846563000	30.406685000
Si	46.324524000	42.855058000	29.025110000
Si	51.890205000	42.866476000	30.529799000
Si	57.969172000	42.905534000	39.187405000

Si	58.733401000	47.858054000	36.909137000
Si	56.880534000	48.675875000	34.758176000
Si	55.869216000	48.297433000	31.973373000
Si	52.764011000	48.329644000	32.242697000
Si	56.558773000	51.798600000	34.995792000
Si	52.600447000	52.739304000	32.661635000
Si	51.490331000	51.663108000	35.481413000
Si	54.138833000	52.727698000	36.716280000
Si	51.807945000	50.703635000	30.333106000
Si	54.680492000	50.356408000	38.541555000
Si	57.777512000	50.331344000	38.803832000
Si	51.889326000	45.954396000	30.524272000
Si	57.969551000	45.926540000	39.182655000
Si	58.734603000	40.964001000	36.914415000
Si	56.880755000	40.147389000	34.761563000
Si	55.873393000	40.523328000	31.974047000
Si	52.770172000	40.488600000	32.235404000
Si	48.661550000	40.941991000	48.517090000
Si	41.741196000	50.804686000	41.940941000
Si	43.551265000	49.979535000	44.077771000
Si	47.727204000	50.359246000	46.624111000
Si	48.765425000	50.163967000	43.611796000
Si	46.060338000	50.636509000	42.326798000
Si	44.786037000	45.957029000	46.548252000
Si	47.931243000	45.960877000	46.171746000
Si	48.664946000	47.877207000	48.518117000
Si	45.618708000	52.751104000	40.038910000
Si	44.769600000	42.854062000	46.543338000
Si	47.930199000	42.838693000	46.158983000
Si	58.955841000	41.841243000	41.948962000
Si	56.315794000	42.832739000	43.274713000
Si	55.801233000	48.299753000	45.159545000
Si	52.635078000	48.369501000	45.309932000
Si	51.673728000	48.627052000	48.344924000
Si	51.704451000	50.846447000	43.451754000
Si	53.650002000	50.041004000	41.348055000
Si	56.184054000	50.710430000	43.088599000
Si	58.958120000	46.981543000	41.931461000
Si	56.310312000	45.965451000	43.264239000
Si	55.795944000	40.498774000	45.165293000
Si	52.662026000	40.432493000	45.329842000
Si	51.670220000	40.194523000	48.348302000
O	49.983909000	36.640469000	35.855760000
O	47.515886000	35.939467000	36.576675000
O	46.656716000	36.845611000	38.977371000
O	44.974169000	36.672224000	36.927301000
O	44.494775000	36.945896000	34.261637000
O	46.169429000	38.860068000	33.285560000
O	47.941254000	37.028728000	34.129174000
O	48.588930000	38.685565000	32.135116000
O	50.189412000	38.104631000	30.094181000
O	52.235701000	36.832056000	31.244052000
O	52.293241000	39.553730000	30.978897000
O	54.596749000	36.895172000	38.076100000
O	56.173064000	38.725645000	39.148256000
O	56.914658000	38.602173000	35.309659000
O	54.993679000	36.662830000	35.420036000
O	51.811651000	36.862448000	33.896844000

O	52.534946000	36.323565000	36.425353000
O	42.372826000	37.840142000	43.471036000
O	45.011217000	38.422107000	43.561150000
O	48.586870000	38.721033000	45.242790000
O	47.519597000	37.849479000	42.924279000
O	45.571252000	36.888711000	41.465275000
O	50.085716000	37.812871000	43.238529000
O	52.386450000	37.939331000	41.962419000
O	52.046481000	39.406874000	44.211666000
O	53.569959000	38.743511000	39.716936000
O	55.057079000	38.113729000	41.873470000
O	44.489544000	51.901689000	34.240457000
O	46.153466000	49.949072000	33.332981000
O	47.928272000	51.776193000	34.172968000
O	48.560155000	50.153966000	32.148280000
O	48.616001000	48.518126000	29.967681000
O	45.414748000	46.597056000	30.235177000
O	47.903665000	46.001431000	29.505173000
O	45.877098000	44.409808000	28.745389000
O	49.989468000	52.188576000	35.864819000
O	47.527969000	52.930096000	36.595556000
O	46.659151000	51.988026000	38.985308000
O	45.003674000	52.114858000	36.900910000
O	48.624387000	40.300550000	29.938600000
O	45.415104000	42.224597000	30.240139000
O	47.902798000	42.820453000	29.507827000
O	50.429871000	42.238258000	30.112880000
O	51.685537000	44.411729000	31.038344000
O	58.588042000	44.416762000	39.358961000
O	58.133725000	47.835483000	35.378076000
O	58.284735000	46.473371000	37.673731000
O	58.208451000	49.188110000	37.703508000
O	56.997401000	48.628936000	33.133905000
O	56.913574000	50.219771000	35.310127000
O	54.347240000	48.579045000	32.575857000
O	52.543782000	46.766247000	31.800788000
O	54.994262000	52.158990000	35.419773000
O	51.809935000	51.960081000	33.897346000
O	52.538074000	52.498700000	36.427233000
O	50.185679000	50.712339000	30.114148000
O	52.236271000	51.993553000	31.241794000
O	52.292926000	49.272974000	30.990519000
O	54.600789000	51.929599000	38.078400000
O	56.174910000	50.094358000	39.147708000
O	50.438190000	46.585829000	30.078106000
O	58.130337000	40.991124000	35.385198000
O	58.299926000	42.349296000	37.685725000
O	58.206938000	39.634609000	37.706914000
O	57.000588000	40.197123000	33.136965000
O	54.351678000	40.235863000	32.572519000
O	52.556980000	42.056163000	31.801041000
O	50.277425000	40.948011000	48.788867000
O	48.254699000	42.297289000	47.670785000
O	48.199078000	39.571477000	47.745337000
O	42.397002000	50.966818000	43.452443000
O	45.054836000	50.413486000	43.584671000
O	48.573117000	50.105454000	45.229347000
O	47.545346000	50.981367000	42.891968000

O	45.559051000	51.921350000	41.458979000
O	44.423751000	44.412497000	46.919902000
O	46.309511000	46.080040000	45.923524000
O	48.420268000	44.406001000	46.022927000
O	50.279474000	47.875720000	48.786982000
O	48.260846000	46.520037000	47.673086000
O	48.203504000	49.242421000	47.732716000
O	46.302550000	42.757261000	45.935371000
O	58.655180000	41.947233000	40.317437000
O	57.909570000	42.729880000	42.884713000
O	55.894819000	44.400039000	43.462700000
O	55.998407000	46.729876000	44.687136000
O	54.240311000	48.618620000	45.595045000
O	56.206998000	49.264987000	43.889488000
O	51.823946000	48.598183000	46.701184000
O	50.094988000	51.026524000	43.232407000
O	52.392692000	50.891682000	41.955587000
O	52.032926000	49.420681000	44.206656000
O	53.572496000	50.077092000	39.713058000
O	55.059365000	50.697190000	41.874925000
O	58.675837000	46.893777000	40.294967000
O	57.890644000	46.079154000	42.828423000
O	55.954164000	42.072953000	44.687208000
O	54.256848000	40.128851000	45.631254000
O	56.212070000	39.553113000	43.884237000
O	51.821710000	40.222296000	46.705333000
H	48.702871000	34.676568000	34.772566000
H	44.419875000	34.676323000	35.346829000
H	42.683628000	36.258975000	36.022159000
H	43.726523000	38.680216000	32.577941000
H	47.730595000	38.010997000	29.830569000
H	52.421136000	38.002380000	29.030380000
H	58.465353000	38.743363000	40.066967000
H	58.088382000	37.108509000	38.286195000
H	56.651061000	36.758841000	33.558678000
H	57.541582000	36.246995000	35.782467000
H	52.141234000	34.673922000	32.562045000
H	54.071037000	36.146097000	32.821438000
H	54.418082000	34.675478000	36.917895000
H	44.265382000	36.148734000	39.494230000
H	46.085045000	34.677316000	40.196440000
H	42.177304000	36.886192000	41.136491000
H	40.290195000	38.107003000	42.090377000
H	43.614692000	38.689573000	45.487247000
H	46.266760000	38.594605000	46.380864000
H	48.065374000	37.113072000	47.131273000
H	52.195251000	36.881484000	44.271734000
H	55.858296000	36.988842000	44.011824000
H	57.529558000	37.965437000	42.519250000
H	41.636484000	38.780154000	36.634495000
H	40.338249000	40.845235000	36.817795000
H	43.726296000	50.140518000	32.578071000
H	47.730503000	50.809921000	29.830551000
H	42.910974000	46.071594000	29.969678000
H	43.636013000	48.346500000	30.474316000
H	46.055518000	46.690813000	27.779371000
H	40.338195000	47.975592000	36.817799000
H	41.636403000	50.040687000	36.634467000

H	48.702714000	54.144377000	34.772532000
H	44.419585000	54.144606000	35.346948000
H	42.683575000	52.561616000	36.022276000
H	43.636069000	40.474361000	30.474327000
H	42.910998000	42.749253000	29.969681000
H	46.055551000	42.130076000	27.779377000
H	52.768240000	42.805898000	29.339147000
H	60.152183000	47.950749000	36.769108000
H	56.115708000	49.276599000	30.928772000
H	56.049418000	46.904457000	31.507081000
H	57.541465000	52.574079000	35.782446000
H	56.650952000	52.062214000	33.558656000
H	52.141090000	54.147065000	32.562019000
H	54.070917000	52.674925000	32.821418000
H	54.418140000	54.145525000	36.917811000
H	52.421044000	50.818602000	29.030363000
H	58.465269000	50.077736000	40.066952000
H	58.088278000	51.712582000	38.286179000
H	52.768217000	46.015092000	29.339142000
H	60.152234000	40.870368000	36.769119000
H	56.049458000	41.916585000	31.507090000
H	56.115777000	39.544444000	30.928783000
H	48.027948000	40.958435000	49.819979000
H	40.290115000	50.713683000	42.090582000
H	42.176850000	51.934815000	41.136521000
H	43.615015000	50.131729000	45.487215000
H	48.065229000	51.707918000	47.131232000
H	46.266685000	50.226321000	46.380825000
H	40.476352000	46.423809000	42.229530000
H	44.578030000	46.685732000	47.785515000
H	48.027871000	47.862537000	49.819962000
H	46.084739000	54.143668000	40.196331000
H	44.265252000	52.672179000	39.494179000
H	40.476382000	42.397038000	42.229542000
H	44.578055000	42.135174000	47.785513000
H	58.818796000	40.408567000	42.221796000
H	60.309668000	42.345550000	42.255160000
H	56.725235000	48.548904000	46.274187000
H	51.671681000	50.045758000	48.769745000
H	52.721673000	47.848745000	49.007593000
H	52.195144000	51.939533000	44.271718000
H	55.858180000	51.832229000	44.011806000
H	57.529465000	50.855664000	42.519236000
H	60.309639000	46.475584000	42.255156000
H	58.818736000	48.412544000	42.221786000
H	56.725177000	40.272281000	46.274313000
H	52.722235000	40.971966000	49.006966000
H	51.671815000	38.775269000	48.769821000
Si	48.783448000	41.016493000	35.384140000
Si	46.877140000	40.075894000	37.420459000
Si	41.573102000	40.145700000	37.193907000
Si	44.249044000	40.878925000	35.851014000
Si	44.661733000	45.957910000	33.459124000
Si	47.836978000	45.910822000	33.141052000
Si	48.776284000	47.776763000	35.384491000
Si	46.886761000	48.778282000	37.421450000
Si	41.581437000	48.664880000	37.172491000
Si	44.232171000	47.995565000	35.823408000

Si	44.619795000	42.901850000	33.465157000
Si	47.805358000	42.874017000	33.149781000
Si	54.811270000	42.896003000	38.833825000
Si	51.694311000	48.543144000	35.170053000
Si	54.405474000	47.951645000	36.448752000
Si	54.815089000	45.935986000	38.839126000
Si	51.697820000	40.276356000	35.157241000
Si	54.401568000	40.873309000	36.448633000
Si	45.747893000	40.513578000	40.167635000
Si	42.664269000	40.402433000	40.051801000
Si	41.756082000	45.906676000	41.724334000
Si	43.747825000	46.911375000	43.821318000
Si	48.917476000	47.048614000	43.426146000
Si	46.208681000	46.007672000	42.174998000
Si	45.786541000	48.283846000	40.192905000
Si	42.670945000	48.386424000	40.053004000
Si	41.775754000	42.878423000	41.738450000
Si	43.748354000	41.889364000	43.858302000
Si	48.959402000	41.785678000	43.415223000
Al	46.246809000	42.807308000	42.235002000
Si	51.897160000	42.861436000	43.568895000
Si	53.943939000	41.868607000	41.530352000
Si	51.824768000	45.914273000	43.535418000
Si	53.903180000	46.948710000	41.527401000
O	46.928375000	38.522041000	36.882431000
O	44.150060000	39.501970000	34.960306000
O	54.396475000	39.446130000	37.260738000
O	51.581871000	38.770016000	35.803013000
O	42.370877000	39.369005000	41.271668000
O	50.393543000	41.201413000	35.477419000
O	48.198896000	40.891654000	36.908804000
O	48.125080000	42.327634000	34.658482000
O	48.408900000	39.648976000	34.564954000
O	46.873644000	40.137417000	39.044155000
O	45.529352000	40.816764000	36.862371000
O	41.642379000	40.006705000	38.838172000
O	42.853812000	41.062781000	36.676915000
O	44.460605000	42.197979000	34.917840000
O	44.043873000	49.360161000	34.932352000
O	43.742854000	46.749796000	32.355764000
O	44.078699000	44.438524000	33.597743000
O	46.215156000	45.926498000	32.938944000
O	48.407694000	44.386724000	32.965370000
O	48.577438000	46.848506000	32.019810000
O	50.388044000	47.629192000	35.516367000
O	48.146615000	47.881753000	36.894662000
O	48.163902000	46.449782000	34.651461000
O	48.397853000	49.140568000	34.561497000
O	46.888994000	48.652405000	39.050089000
O	47.060842000	50.337453000	36.929098000
O	45.464960000	48.219569000	36.860814000
O	41.693938000	48.800702000	38.815309000
O	42.834062000	47.726587000	36.622808000
O	44.576101000	46.692880000	34.907547000
O	43.714583000	42.113248000	32.348208000
O	46.181506000	42.892437000	32.968034000
O	48.523894000	41.929833000	32.018831000
O	54.229992000	44.417559000	38.656097000

O	56.347612000	42.954059000	39.392659000
O	55.472068000	47.979690000	35.215622000
O	51.852736000	48.705418000	33.544881000
O	52.926298000	47.698479000	35.818491000
O	51.582828000	50.054488000	35.805443000
O	54.808099000	46.698517000	37.403496000
O	54.398193000	49.383558000	37.253988000
O	56.348773000	45.889177000	39.411131000
O	55.472414000	40.843091000	35.218944000
O	51.861147000	40.102164000	33.534212000
O	52.926538000	41.123031000	35.808604000
O	54.793928000	42.134609000	37.398202000
O	45.907374000	42.119531000	40.573820000
O	44.236820000	40.235817000	39.568878000
O	46.064539000	39.579052000	41.454868000
O	42.404161000	41.948543000	40.526310000
O	42.364502000	49.438931000	41.255481000
O	43.160979000	48.430815000	43.676770000
O	48.844740000	48.635200000	43.017305000
O	42.793665000	45.875379000	43.003249000
O	41.587105000	44.394258000	41.115060000
O	43.753490000	46.526529000	45.404955000
O	45.264511000	46.902102000	43.175459000
O	48.702660000	46.886240000	45.052329000
O	47.772983000	46.219453000	42.600091000
O	45.768404000	44.478576000	42.038020000
O	46.032587000	46.665684000	40.600449000
O	44.264426000	48.502579000	39.626353000
O	46.084091000	49.229083000	41.477510000
O	42.383857000	46.846337000	40.520975000
O	43.073416000	40.394433000	43.720224000
O	48.810286000	40.193629000	43.019047000
O	42.803523000	42.939626000	43.014930000
O	43.685427000	42.304867000	45.448122000
O	45.264414000	41.878335000	43.313655000
O	48.731282000	41.931293000	45.057491000
O	47.938211000	42.710317000	42.585157000
O	53.536215000	40.327712000	41.891004000
O	50.499930000	42.217539000	43.047114000
O	52.930624000	42.900740000	42.287530000
O	51.690248000	44.393011000	44.115751000
O	53.877672000	42.073438000	39.902444000
O	55.481113000	42.144183000	42.038387000
O	52.416931000	46.842898000	44.753631000
O	53.513292000	48.495410000	41.890137000
O	50.378157000	46.462176000	42.993089000
O	52.844502000	45.934488000	42.246404000
O	53.869505000	46.759960000	39.897468000
O	55.422576000	46.651943000	42.066119000
O	52.540719000	41.980558000	44.797580000
Ga	46.673819000	45.210671000	39.004969000
H	45.942183000	45.893126000	37.798930000
H	45.589677000	43.852064000	39.171587000
C	48.550070000	44.873659000	39.526661000
H	48.998028000	45.866336000	39.737234000
H	48.598675000	44.308040000	40.477873000
H	45.751878000	43.080352000	39.765952000
C	49.346649000	44.145295000	38.428237000

H	50.408938000	44.041059000	38.721397000
H	49.333846000	44.687206000	37.463396000
H	48.965714000	43.124573000	38.242153000

I3-GaH_2

Electronic energy (r^2SCAN-D4/def2-SVP): -48258.46846130 E_h
Electronic energy (ωB97X-D4rev/def2-mTZVPP): -48302.209637828900 E_h
G_{corr} (673.15 K, 1 atm): 1.98064694 E_h
Number of atoms: 387

Cartesian coordinates (Å)

Si	48.556869000	36.033621000	35.296951000
Si	46.526350000	36.980989000	37.346295000
Si	44.101505000	36.092390000	35.633652000
Si	44.620891000	38.504094000	33.732766000
Si	47.771513000	38.531326000	33.544720000
Si	48.742269000	38.770661000	30.511585000
Si	51.806292000	38.125117000	30.329428000
Si	54.674725000	38.464332000	38.536631000
Si	57.776173000	38.488983000	38.803717000
Si	56.557148000	37.022571000	34.996621000
Si	52.598474000	36.083796000	32.657672000
Si	51.487318000	37.161152000	35.475505000
Si	54.131158000	36.091788000	36.713557000
Si	45.621804000	36.070524000	40.027906000
Si	41.740789000	38.011188000	41.950670000
Si	43.603162000	38.834258000	44.072753000
Si	47.726540000	38.457791000	46.613396000
Si	48.754214000	38.669374000	43.586335000
Si	46.059693000	38.171565000	42.325983000
Si	51.719385000	37.996347000	43.463136000
Si	53.663184000	38.784660000	41.346250000
Si	56.184581000	38.105823000	43.084490000
Si	44.615865000	50.314440000	33.737511000
Si	47.765413000	50.289536000	33.557018000
Si	48.740956000	50.056228000	30.521275000
Si	43.867867000	46.935064000	30.714313000
Si	48.875503000	46.957849000	30.408743000
Si	46.321018000	45.963665000	29.024036000
Si	48.555413000	52.788362000	35.305345000
Si	46.529687000	51.839835000	37.358184000
Si	44.103200000	52.728597000	35.636505000
Si	43.869320000	41.886799000	30.711399000
Si	48.874773000	41.867692000	30.401509000
Si	46.321415000	42.859889000	29.021667000
Si	51.881093000	42.864920000	30.519641000
Si	57.970641000	42.906143000	39.189073000
Si	58.733674000	47.858098000	36.908892000
Si	56.877288000	48.676514000	34.757859000
Si	55.867952000	48.297074000	31.973360000
Si	52.761492000	48.322702000	32.243530000
Si	56.556457000	51.799354000	34.996529000
Si	52.599658000	52.738011000	32.655884000
Si	51.489331000	51.659894000	35.471804000
Si	54.130147000	52.730540000	36.711827000
Si	51.805593000	50.698572000	30.331216000

Si	54.676092000	50.356898000	38.531323000
Si	57.777668000	50.331646000	38.802309000
Si	51.880713000	45.954933000	30.519517000
Si	57.970871000	45.925523000	39.183433000
Si	58.734817000	40.964476000	36.914131000
Si	56.879408000	40.146569000	34.760677000
Si	55.871696000	40.524450000	31.974126000
Si	52.765960000	40.496561000	32.239257000
Si	48.664414000	40.941118000	48.515386000
Si	41.740734000	50.788707000	41.946134000
Si	43.699772000	50.021506000	44.070027000
Si	47.726855000	50.362398000	46.609947000
Si	48.767082000	50.155174000	43.577461000
Si	46.082261000	50.772145000	42.366123000
Si	44.742623000	45.947475000	46.552674000
Si	47.920336000	45.946232000	46.151156000
Si	48.673584000	47.889131000	48.513020000
Si	45.618996000	52.769814000	40.025694000
Si	44.738930000	42.862890000	46.548252000
Si	47.921443000	42.854513000	46.174828000
Si	58.956147000	41.841041000	41.951565000
Si	56.318702000	42.832457000	43.279935000
Si	55.797519000	48.296904000	45.169290000
Si	52.646582000	48.353784000	45.334826000
Si	51.671280000	48.627225000	48.353269000
Si	51.736407000	50.827605000	43.459370000
Si	53.666761000	50.032271000	41.340833000
Si	56.183717000	50.709794000	43.088261000
Si	58.958662000	46.981028000	41.933960000
Si	56.316204000	45.963959000	43.268140000
Si	55.796791000	40.495968000	45.169933000
Si	52.681496000	40.439103000	45.346684000
Si	51.669252000	40.195139000	48.350780000
O	49.976938000	36.653672000	35.845520000
O	47.517051000	35.928497000	36.573933000
O	46.662461000	36.835001000	38.970260000
O	44.969618000	36.653557000	36.932484000
O	44.495974000	36.948000000	34.263437000
O	46.175552000	38.864003000	33.291789000
O	47.922175000	36.999029000	34.113498000
O	48.590511000	38.681388000	32.143510000
O	50.187135000	38.137814000	30.088288000
O	52.220854000	36.831987000	31.241521000
O	52.301928000	39.552914000	30.985004000
O	54.587645000	36.890774000	38.077017000
O	56.172094000	38.725933000	39.136350000
O	56.914476000	38.601740000	35.310345000
O	54.992245000	36.663034000	35.420755000
O	51.810638000	36.864193000	33.892847000
O	52.529324000	36.313970000	36.419376000
O	42.420857000	37.870900000	43.450950000
O	45.100842000	38.315424000	43.634053000
O	48.550963000	38.684331000	45.204662000
O	47.572390000	37.835685000	42.820347000
O	45.554915000	36.897221000	41.445641000
O	50.112991000	37.843104000	43.227078000
O	52.411152000	37.941797000	41.967755000
O	52.076538000	39.418510000	44.217281000

O	53.573000000	38.751012000	39.711356000
O	55.073385000	38.117888000	41.859670000
O	44.496272000	51.871581000	34.267609000
O	46.171114000	49.950268000	33.302443000
O	47.907471000	51.822158000	34.129612000
O	48.583718000	50.147287000	32.153783000
O	48.591045000	48.509838000	29.980939000
O	45.413464000	46.596786000	30.238635000
O	47.899873000	45.991846000	29.506107000
O	45.864441000	44.411864000	28.742723000
O	49.978511000	52.165784000	35.842159000
O	47.528661000	52.892283000	36.595390000
O	46.652713000	51.973933000	38.987782000
O	44.976166000	52.175324000	36.937036000
O	48.585750000	40.316878000	29.972046000
O	45.415639000	42.224125000	30.236466000
O	47.901812000	42.835733000	29.498457000
O	50.433757000	42.222671000	30.074975000
O	51.658786000	44.410008000	31.022823000
O	58.591724000	44.416462000	39.359795000
O	58.129108000	47.835118000	35.379880000
O	58.287423000	46.473723000	37.675582000
O	58.215252000	49.189415000	37.704247000
O	56.996264000	48.630940000	33.133621000
O	56.910030000	50.219916000	35.311712000
O	54.345109000	48.572888000	32.575576000
O	52.541415000	46.759552000	31.797655000
O	54.991718000	52.160284000	35.419594000
O	51.810384000	51.953899000	33.888188000
O	52.528534000	52.511750000	36.414802000
O	50.184865000	50.693570000	30.099739000
O	52.227778000	51.993891000	31.236684000
O	52.293743000	49.271559000	30.994218000
O	54.584505000	51.931270000	38.076180000
O	56.172696000	50.095256000	39.132297000
O	50.432688000	46.597894000	30.078458000
O	58.126842000	40.992237000	35.386261000
O	58.301247000	42.348539000	37.687893000
O	58.212204000	39.633285000	37.706448000
O	56.999397000	40.194583000	33.136057000
O	54.348792000	40.249833000	32.575429000
O	52.539588000	42.060816000	31.799357000
O	50.277917000	40.945908000	48.800333000
O	48.261010000	42.303037000	47.679181000
O	48.219715000	39.580512000	47.713075000
O	42.459228000	50.894299000	43.432415000
O	45.165373000	50.677941000	43.712277000
O	48.532229000	50.099097000	45.191348000
O	47.620960000	51.035684000	42.811984000
O	45.566560000	52.040626000	41.494768000
O	44.281567000	44.410176000	46.856831000
O	46.290993000	45.993862000	46.008130000
O	48.459572000	44.403386000	46.017969000
O	50.280431000	47.890012000	48.825268000
O	48.315997000	46.551846000	47.618360000
O	48.227976000	49.257525000	47.722614000
O	46.289820000	42.830470000	45.990016000
O	58.653950000	41.948053000	40.320852000

O	57.913071000	42.726485000	42.894184000
O	55.900634000	44.399406000	43.473331000
O	55.995001000	46.730775000	44.686982000
O	54.246207000	48.598877000	45.647861000
O	56.179776000	49.268595000	43.897218000
O	51.799633000	48.584940000	46.705901000
O	50.129417000	50.972548000	43.235428000
O	52.421325000	50.887218000	41.963457000
O	52.084485000	49.408957000	44.216734000
O	53.575343000	50.066348000	39.707085000
O	55.079007000	50.690786000	41.857367000
O	58.672904000	46.892401000	40.298629000
O	57.898168000	46.078050000	42.838856000
O	55.947323000	42.068984000	44.688038000
O	54.268296000	40.117282000	45.664038000
O	56.202100000	39.551463000	43.885168000
O	51.812575000	40.230645000	46.705833000
H	48.702871000	34.676563000	34.772437000
H	44.419913000	34.676308000	35.346662000
H	42.683635000	36.258916000	36.022014000
H	43.726524000	38.680293000	32.577897000
H	47.730623000	38.011030000	29.830495000
H	52.421176000	38.002428000	29.030311000
H	58.465343000	38.743264000	40.066926000
H	58.088357000	37.108441000	38.286129000
H	56.651071000	36.758835000	33.558612000
H	57.541584000	36.246963000	35.782399000
H	52.141230000	34.673936000	32.561956000
H	54.071049000	36.146087000	32.821357000
H	54.418072000	34.675444000	36.917813000
H	44.265356000	36.148695000	39.494103000
H	46.084996000	34.677253000	40.196324000
H	42.177321000	36.886188000	41.136368000
H	40.290180000	38.106955000	42.090266000
H	43.614678000	38.689470000	45.487155000
H	46.266697000	38.594471000	46.380743000
H	48.065301000	37.112925000	47.131158000
H	52.195211000	36.881420000	44.271661000
H	55.858236000	36.988724000	44.011753000
H	57.529513000	37.965327000	42.519197000
H	41.636486000	38.780154000	36.634491000
H	40.338249000	40.845232000	36.817789000
H	43.726311000	50.140520000	32.578067000
H	47.730552000	50.809943000	29.830563000
H	42.911007000	46.071617000	29.969655000
H	43.636044000	48.346515000	30.474317000
H	46.055556000	46.690862000	27.779356000
H	40.338197000	47.975590000	36.817797000
H	41.636403000	50.040686000	36.634465000
H	48.702729000	54.144364000	34.772581000
H	44.419588000	54.144587000	35.346973000
H	42.683579000	52.561586000	36.022278000
H	43.636098000	40.474388000	30.474287000
H	42.911015000	42.749277000	29.969667000
H	46.055589000	42.130107000	27.779337000
H	52.768270000	42.805906000	29.339124000
H	60.152189000	47.950711000	36.769159000
H	56.115740000	49.276612000	30.928820000

H	56.049452000	46.904465000	31.507108000
H	57.541477000	52.574043000	35.782547000
H	56.650984000	52.062208000	33.558742000
H	52.141141000	54.147072000	32.562080000
H	54.070953000	52.674912000	32.821490000
H	54.418152000	54.145492000	36.917889000
H	52.421094000	50.818636000	29.030405000
H	58.465241000	50.077637000	40.067034000
H	58.088289000	51.712515000	38.286282000
H	52.768254000	46.015118000	29.339141000
H	60.152228000	40.870318000	36.769116000
H	56.049469000	41.916589000	31.507076000
H	56.115796000	39.544452000	30.928754000
H	48.027871000	40.958250000	49.819908000
H	40.290083000	50.713615000	42.090585000
H	42.176795000	51.934800000	41.136544000
H	43.614986000	50.131696000	45.487229000
H	48.065142000	51.707774000	47.131282000
H	46.266629000	50.226168000	46.380825000
H	40.476350000	46.423806000	42.229527000
H	44.578049000	46.685639000	47.785502000
H	48.027791000	47.862364000	49.819964000
H	46.084713000	54.143620000	40.196353000
H	44.265236000	52.672122000	39.494195000
H	40.476381000	42.397035000	42.229537000
H	44.578028000	42.135090000	47.785498000
H	58.818734000	40.408466000	42.221781000
H	60.309642000	42.345421000	42.255169000
H	56.725207000	48.548767000	46.274227000
H	51.671615000	50.045583000	48.769825000
H	52.721599000	47.848559000	49.007612000
H	52.195123000	51.939468000	44.271761000
H	55.858156000	51.832116000	44.011890000
H	57.529445000	50.855561000	42.519321000
H	60.309614000	46.475467000	42.255211000
H	58.818734000	48.412444000	42.221850000
H	56.725109000	40.272137000	46.274288000
H	52.722156000	40.971771000	49.006932000
H	51.671729000	38.775086000	48.769696000
Si	48.778169000	40.971405000	35.408323000
Si	46.864550000	40.046315000	37.441333000
Si	41.575089000	40.154160000	37.181563000
Si	44.262559000	40.879929000	35.854034000
Si	44.640038000	45.941818000	33.460022000
Si	47.815940000	45.942574000	33.183812000
Si	48.768843000	47.854451000	35.429959000
Si	46.853366000	48.776108000	37.471193000
Si	41.572331000	48.670500000	37.175026000
Si	44.259554000	47.945911000	35.863884000
Si	44.637026000	42.890481000	33.455022000
Si	47.814148000	42.888541000	33.176219000
Si	54.815340000	42.896273000	38.830413000
Si	51.693944000	48.540139000	35.173214000
Si	54.401770000	47.953989000	36.443962000
Si	54.818354000	45.933147000	38.834259000
Si	51.697120000	40.277820000	35.164079000
Si	54.400441000	40.867362000	36.445538000
Si	45.721884000	40.488670000	40.170830000

Si	42.645819000	40.418205000	40.050005000
Si	41.768106000	45.942838000	41.710809000
Si	43.897554000	46.874864000	43.794210000
Si	48.898933000	47.032544000	43.343140000
Si	46.291351000	45.989911000	41.979745000
Si	45.702276000	48.431023000	40.225455000
Si	42.625637000	48.404009000	40.038740000
Si	41.777273000	42.889633000	41.757819000
Si	43.813435000	41.925087000	43.844304000
Si	48.978414000	41.777890000	43.434794000
Al	46.363962000	42.790926000	42.137224000
Si	51.923906000	42.856260000	43.598883000
Si	53.949670000	41.876556000	41.532175000
Si	51.835933000	45.917091000	43.550766000
Si	53.913993000	46.938489000	41.530056000
O	46.908151000	38.506968000	36.863803000
O	44.164480000	39.500254000	34.968735000
O	54.390239000	39.433653000	37.246452000
O	51.597329000	38.767799000	35.804356000
O	42.318596000	39.385033000	41.255628000
O	50.384994000	41.186517000	35.502790000
O	48.188396000	40.862215000	36.935108000
O	48.092344000	42.245154000	34.651043000
O	48.436304000	39.588359000	34.599282000
O	46.847028000	40.038811000	39.069927000
O	45.516630000	40.811133000	36.903807000
O	41.629533000	40.048136000	38.826823000
O	42.850238000	41.074867000	36.651122000
O	44.511065000	42.189311000	34.915624000
O	44.152040000	49.323441000	34.974688000
O	43.732212000	46.749007000	32.357783000
O	44.065030000	44.416556000	33.568459000
O	46.197233000	45.927813000	32.949128000
O	48.404147000	44.415344000	33.113855000
O	48.571560000	46.819855000	32.024266000
O	50.377083000	47.645032000	35.529764000
O	48.172521000	47.957142000	36.956482000
O	48.092898000	46.580978000	34.661696000
O	48.431165000	49.236049000	34.615098000
O	46.834235000	48.800034000	39.103699000
O	46.912868000	50.313654000	36.885689000
O	45.498516000	48.034509000	36.926911000
O	41.626546000	48.812167000	38.817227000
O	42.844292000	47.741696000	36.655390000
O	44.521545000	46.641013000	34.923087000
O	43.730638000	42.082607000	32.352868000
O	46.194844000	42.905454000	32.945686000
O	48.568000000	42.011318000	32.015767000
O	54.240955000	44.415744000	38.626707000
O	56.349479000	42.955287000	39.395197000
O	55.467468000	47.979339000	35.209865000
O	51.849281000	48.692084000	33.546655000
O	52.922587000	47.686709000	35.818955000
O	51.605253000	50.054951000	35.804502000
O	54.815756000	46.713764000	37.409478000
O	54.390831000	49.391259000	37.239397000
O	56.350464000	45.884736000	39.410255000
O	55.468891000	40.840527000	35.214012000

O	51.855604000	40.114117000	33.539194000
O	52.924521000	41.130086000	35.811809000
O	54.804580000	42.114903000	37.406294000
O	45.915125000	42.094129000	40.522811000
O	44.213394000	40.223711000	39.563883000
O	45.980947000	39.602793000	41.514668000
O	42.418108000	41.960032000	40.552596000
O	42.240327000	49.367110000	41.288074000
O	43.642957000	48.467367000	43.522032000
O	48.781863000	48.626685000	42.971188000
O	42.802604000	45.975764000	42.991097000
O	41.610434000	44.409715000	41.148095000
O	43.769785000	46.609801000	45.400866000
O	45.400832000	46.448270000	43.274531000
O	48.579984000	46.846653000	44.946823000
O	47.864553000	46.180460000	42.387281000
O	45.977105000	44.438282000	41.511492000
O	45.869987000	46.850522000	40.640681000
O	44.196831000	48.668879000	39.603500000
O	45.940293000	49.355934000	41.546859000
O	42.396834000	46.839675000	40.472445000
O	43.306631000	40.381345000	43.601813000
O	48.769367000	40.200733000	43.012699000
O	42.786133000	42.936777000	43.053126000
O	43.703107000	42.214669000	45.456699000
O	45.326598000	42.144146000	43.336761000
O	48.673511000	41.918609000	45.060825000
O	48.028674000	42.741905000	42.556615000
O	53.548616000	40.332506000	41.886169000
O	50.542081000	42.146562000	43.125207000
O	52.936379000	42.900043000	42.302861000
O	51.671537000	44.390820000	44.115047000
O	53.874950000	42.091941000	39.907219000
O	55.487512000	42.151725000	42.037228000
O	52.435713000	46.824797000	44.781088000
O	53.529038000	48.487702000	41.884257000
O	50.406406000	46.505419000	43.012665000
O	52.864226000	45.929247000	42.269656000
O	53.867249000	46.736606000	39.902920000
O	55.437142000	46.644933000	42.060961000
O	52.597357000	41.996356000	44.827805000
Ga	45.644977000	43.903177000	39.533896000
H	44.109336000	44.050252000	39.264153000
H	42.093794000	44.785234000	36.676984000
C	47.333595000	44.233566000	38.571557000
H	47.820794000	45.125751000	39.016219000
H	48.015955000	43.384878000	38.782405000
H	42.096008000	44.025676000	36.662178000
C	47.150508000	44.422501000	37.058934000
H	48.123192000	44.556920000	36.551190000
H	46.534580000	45.310419000	36.825978000
H	46.658738000	43.550755000	36.589999000

I4-GaH_2

Electronic energy (r^2SCAN-D4/def2-SVP): -48257.30158930 E_h
Electronic energy (ωB97X-D4rev/def2-mTZVPP): -48301.027492053500 E_h
G_{corr} (673.15 K, 1 atm): 1.98173127 E_h
Number of atoms: 385

Cartesian coordinates (Å)

Si	48.557548000	36.033086000	35.298413000
Si	46.526415000	36.981162000	37.346581000
Si	44.101548000	36.092580000	35.633786000
Si	44.621799000	38.504473000	33.732361000
Si	47.772435000	38.530816000	33.544656000
Si	48.742241000	38.770585000	30.511795000
Si	51.806095000	38.125932000	30.329491000
Si	54.675834000	38.462072000	38.536361000
Si	57.776614000	38.489345000	38.803491000
Si	56.557577000	37.022643000	34.996698000
Si	52.598281000	36.083993000	32.658799000
Si	51.487446000	37.161777000	35.477991000
Si	54.132692000	36.092266000	36.715212000
Si	45.621815000	36.070659000	40.028123000
Si	41.740863000	38.011221000	41.950771000
Si	43.602489000	38.834615000	44.072911000
Si	47.726572000	38.457984000	46.613466000
Si	48.754104000	38.670411000	43.586446000
Si	46.059369000	38.172269000	42.325605000
Si	51.719757000	37.996566000	43.462829000
Si	53.662910000	38.785349000	41.345172000
Si	56.184510000	38.106231000	43.084510000
Si	44.616379000	50.313845000	33.737447000
Si	47.766139000	50.289897000	33.557176000
Si	48.740809000	50.056111000	30.521336000
Si	43.867711000	46.935083000	30.714350000
Si	48.874546000	46.957606000	30.410122000
Si	46.320944000	45.963579000	29.024212000
Si	48.555811000	52.788850000	35.306279000
Si	46.529581000	51.839846000	37.358414000
Si	44.103200000	52.728495000	35.636587000
Si	43.869170000	41.886770000	30.711594000
Si	48.873751000	41.867963000	30.403270000
Si	46.321365000	42.859845000	29.021923000
Si	51.881172000	42.864730000	30.520091000
Si	57.970996000	42.906282000	39.189685000
Si	58.733615000	47.857679000	36.908970000
Si	56.877612000	48.676796000	34.757525000
Si	55.868556000	48.297050000	31.973255000
Si	52.762464000	48.322435000	32.243238000
Si	56.556915000	51.799260000	34.996466000
Si	52.599475000	52.737825000	32.656624000
Si	51.489240000	51.659269000	35.473512000
Si	54.131316000	52.730232000	36.713063000
Si	51.805569000	50.697845000	30.331247000
Si	54.676829000	50.359272000	38.530962000
Si	57.777936000	50.331429000	38.801998000
Si	51.880834000	45.955017000	30.519919000
Si	57.971281000	45.925367000	39.183930000
Si	58.734803000	40.964823000	36.914224000

Si	56.879435000	40.146262000	34.760432000
Si	55.872329000	40.524467000	31.974034000
Si	52.766885000	40.496793000	32.238960000
Si	48.664121000	40.941315000	48.515262000
Si	41.740870000	50.788695000	41.946085000
Si	43.699696000	50.021215000	44.069992000
Si	47.726970000	50.362532000	46.609665000
Si	48.767169000	50.154472000	43.576965000
Si	46.081976000	50.771575000	42.365470000
Si	44.741837000	45.947256000	46.552989000
Si	47.919357000	45.946225000	46.151060000
Si	48.673424000	47.889335000	48.512774000
Si	45.618968000	52.769818000	40.025835000
Si	44.738083000	42.863580000	46.548600000
Si	47.920401000	42.855021000	46.174778000
Si	58.956221000	41.841054000	41.951725000
Si	56.318421000	42.832427000	43.279883000
Si	55.797722000	48.296650000	45.169308000
Si	52.646397000	48.354265000	45.335117000
Si	51.671243000	48.627551000	48.353324000
Si	51.736752000	50.827547000	43.458962000
Si	53.666558000	50.031449000	41.339757000
Si	56.183658000	50.709549000	43.088198000
Si	58.958754000	46.981190000	41.933948000
Si	56.316177000	45.963986000	43.268074000
Si	55.796962000	40.496267000	45.170080000
Si	52.681787000	40.438565000	45.347154000
Si	51.669250000	40.195127000	48.350784000
O	49.978162000	36.650937000	35.847930000
O	47.517315000	35.928164000	36.575094000
O	46.662238000	36.835648000	38.970654000
O	44.969788000	36.653449000	36.932682000
O	44.496147000	36.948674000	34.263949000
O	46.176599000	38.862328000	33.289752000
O	47.923649000	36.999466000	34.115484000
O	48.592768000	38.679089000	32.143869000
O	50.187264000	38.139636000	30.086151000
O	52.218776000	36.833839000	31.243950000
O	52.302144000	39.553990000	30.984301000
O	54.593115000	36.887254000	38.079938000
O	56.172568000	38.728992000	39.135351000
O	56.916536000	38.601502000	35.309998000
O	54.992751000	36.663666000	35.421713000
O	51.812388000	36.862728000	33.896106000
O	52.530967000	36.319162000	36.424290000
O	42.420438000	37.870799000	43.451317000
O	45.100188000	38.316888000	43.633312000
O	48.551199000	38.685535000	45.204904000
O	47.572003000	37.836863000	42.820703000
O	45.555230000	36.897127000	41.446048000
O	50.113143000	37.844443000	43.227320000
O	52.411304000	37.942169000	41.967303000
O	52.078266000	39.418770000	44.216304000
O	53.573426000	38.749638000	39.710180000
O	55.073212000	38.119606000	41.859798000
O	44.496455000	51.870874000	34.268124000
O	46.171917000	49.950852000	33.302048000
O	47.908572000	51.822091000	34.130674000

O	48.584469000	50.148505000	32.153830000
O	48.589689000	48.509465000	29.982051000
O	45.413419000	46.596487000	30.239089000
O	47.899793000	45.991496000	29.506549000
O	45.864347000	44.411801000	28.742649000
O	49.979399000	52.168110000	35.843853000
O	47.528673000	52.892552000	36.596044000
O	46.652595000	51.973582000	38.988082000
O	44.976102000	52.175596000	36.937383000
O	48.583992000	40.317244000	29.974117000
O	45.415587000	42.224467000	30.237063000
O	47.901743000	42.835920000	29.498962000
O	50.433230000	42.222010000	30.077855000
O	51.658825000	44.409973000	31.022655000
O	58.592504000	44.416448000	39.360343000
O	58.127886000	47.833743000	35.380392000
O	58.287538000	46.473440000	37.676010000
O	58.216194000	49.189540000	37.703961000
O	56.997201000	48.630506000	33.133329000
O	56.912382000	50.220174000	35.311146000
O	54.345870000	48.573243000	32.575402000
O	52.542791000	46.759094000	31.797798000
O	54.992264000	52.159456000	35.420564000
O	51.811521000	51.954748000	33.890368000
O	52.529860000	52.507520000	36.418222000
O	50.185060000	50.691908000	30.098784000
O	52.226489000	51.992578000	31.238226000
O	52.294465000	49.270653000	30.993479000
O	54.588550000	51.934803000	38.078914000
O	56.172925000	50.093304000	39.131314000
O	50.432210000	46.598376000	30.081254000
O	58.125144000	40.993631000	35.387040000
O	58.301083000	42.348526000	37.688514000
O	58.214379000	39.632582000	37.706007000
O	57.000259000	40.194975000	33.135869000
O	54.349523000	40.249336000	32.574960000
O	52.540961000	42.061319000	31.799634000
O	50.277831000	40.946431000	48.799134000
O	48.259979000	42.302962000	47.678911000
O	48.218771000	39.580464000	47.713812000
O	42.459184000	50.894139000	43.432532000
O	45.165487000	50.677118000	43.711933000
O	48.531895000	50.098884000	45.190844000
O	47.620955000	51.034257000	42.810891000
O	45.566630000	52.040838000	41.495037000
O	44.280240000	44.410402000	46.858700000
O	46.290000000	45.992824000	46.007982000
O	48.459335000	44.403630000	46.018153000
O	50.280478000	47.889455000	48.824290000
O	48.314800000	46.552326000	47.618108000
O	48.228380000	49.258012000	47.722595000
O	46.288786000	42.831960000	45.989855000
O	58.654420000	41.947821000	40.320984000
O	57.912743000	42.726330000	42.893998000
O	55.900602000	44.399438000	43.473081000
O	55.995636000	46.730514000	44.687248000
O	54.246261000	48.598116000	45.647578000
O	56.180071000	49.268471000	43.897383000

O	51.799856000	48.587627000	46.705960000
O	50.129596000	50.972031000	43.235277000
O	52.421654000	50.886801000	41.963016000
O	52.085606000	49.408675000	44.215640000
O	53.575624000	50.067296000	39.705896000
O	55.078916000	50.689066000	41.857320000
O	58.673386000	46.892718000	40.298592000
O	57.898013000	46.078110000	42.838392000
O	55.947266000	42.069304000	44.688279000
O	54.268650000	40.117433000	45.664592000
O	56.202309000	39.551824000	43.885301000
O	51.812654000	40.228367000	46.705821000
H	48.702868000	34.676567000	34.772567000
H	44.419872000	34.676322000	35.346831000
H	42.683625000	36.258974000	36.022161000
H	43.726520000	38.680216000	32.577942000
H	47.730591000	38.010996000	29.830570000
H	52.421132000	38.002379000	29.030381000
H	58.465350000	38.743362000	40.066967000
H	58.088379000	37.108508000	38.286195000
H	56.651057000	36.758841000	33.558678000
H	57.541579000	36.246994000	35.782467000
H	52.141230000	34.673921000	32.562045000
H	54.071034000	36.146097000	32.821438000
H	54.418079000	34.675478000	36.917895000
H	44.265380000	36.148733000	39.494232000
H	46.085042000	34.677315000	40.196441000
H	42.177302000	36.886191000	41.136492000
H	40.290192000	38.107003000	42.090377000
H	43.614689000	38.689572000	45.487248000
H	46.266758000	38.594604000	46.380866000
H	48.065371000	37.113070000	47.131273000
H	52.195249000	36.881483000	44.271735000
H	55.858293000	36.988841000	44.011825000
H	57.529555000	37.965437000	42.519250000
H	41.636484000	38.780154000	36.634495000
H	40.338248000	40.845234000	36.817796000
H	43.726292000	50.140517000	32.578072000
H	47.730498000	50.809921000	29.830553000
H	42.910970000	46.071593000	29.969681000
H	43.636009000	48.346499000	30.474317000
H	46.055514000	46.690812000	27.779372000
H	40.338195000	47.975591000	36.817799000
H	41.636402000	50.040687000	36.634467000
H	48.702710000	54.144376000	34.772534000
H	44.419580000	54.144605000	35.346950000
H	42.683571000	52.561614000	36.022278000
H	43.636066000	40.474360000	30.474328000
H	42.910994000	42.749252000	29.969683000
H	46.055547000	42.130075000	27.779378000
H	52.768236000	42.805898000	29.339147000
H	60.152179000	47.950749000	36.769109000
H	56.115704000	49.276599000	30.928772000
H	56.049414000	46.904456000	31.507082000
H	57.541460000	52.574079000	35.782447000
H	56.650948000	52.062214000	33.558656000
H	52.141085000	54.147065000	32.562020000
H	54.070913000	52.674924000	32.821419000

H	54.418136000	54.145525000	36.917812000
H	52.421040000	50.818602000	29.030364000
H	58.465265000	50.077735000	40.066953000
H	58.088275000	51.712582000	38.286180000
H	52.768213000	46.015091000	29.339143000
H	60.152231000	40.870367000	36.769120000
H	56.049455000	41.916585000	31.507090000
H	56.115774000	39.544444000	30.928783000
H	48.027946000	40.958434000	49.819980000
H	40.290111000	50.713679000	42.090584000
H	42.176845000	51.934815000	41.136524000
H	43.615012000	50.131727000	45.487217000
H	48.065225000	51.707916000	47.131233000
H	46.266681000	50.226320000	46.380827000
H	40.476352000	46.423809000	42.229530000
H	44.578027000	46.685731000	47.785517000
H	48.027868000	47.862535000	49.819963000
H	46.084735000	54.143666000	40.196333000
H	44.265249000	52.672178000	39.494181000
H	40.476382000	42.397038000	42.229542000
H	44.578052000	42.135172000	47.785514000
H	58.818793000	40.408566000	42.221797000
H	60.309666000	42.345550000	42.255161000
H	56.725233000	48.548903000	46.274187000
H	51.671678000	50.045757000	48.769746000
H	52.721670000	47.848743000	49.007594000
H	52.195141000	51.939533000	44.271719000
H	55.858177000	51.832229000	44.011807000
H	57.529462000	50.855664000	42.519236000
H	60.309636000	46.475584000	42.255157000
H	58.818732000	48.412544000	42.221787000
H	56.725174000	40.272280000	46.274314000
H	52.722233000	40.971965000	49.006967000
H	51.671813000	38.775267000	48.769821000
Si	48.778222000	40.972043000	35.407828000
Si	46.866165000	40.046770000	37.440513000
Si	41.575382000	40.153984000	37.182091000
Si	44.262886000	40.880792000	35.855048000
Si	44.636934000	45.941554000	33.460828000
Si	47.813083000	45.942731000	33.185221000
Si	48.769073000	47.854314000	35.429706000
Si	46.854675000	48.775908000	37.470489000
Si	41.572651000	48.670438000	37.175390000
Si	44.259515000	47.945084000	35.864818000
Si	44.633761000	42.890905000	33.456137000
Si	47.811273000	42.888977000	33.177829000
Si	54.816973000	42.896330000	38.828418000
Si	51.693219000	48.539180000	35.172735000
Si	54.401555000	47.959162000	36.443947000
Si	54.819913000	45.933007000	38.832303000
Si	51.696273000	40.278788000	35.163677000
Si	54.400057000	40.862145000	36.445592000
Si	45.720480000	40.488511000	40.169330000
Si	42.644722000	40.417566000	40.049255000
Si	41.767992000	45.942788000	41.710669000
Si	43.896938000	46.874332000	43.794346000
Si	48.897761000	47.031627000	43.342661000
Si	46.289855000	45.989635000	41.978871000

Si	45.701109000	48.430878000	40.224192000
Si	42.624803000	48.404329000	40.038038000
Si	41.777213000	42.889527000	41.757256000
Si	43.812885000	41.925285000	43.844338000
Si	48.977156000	41.778870000	43.434298000
Al	46.362590000	42.790563000	42.136300000
Si	51.923060000	42.856466000	43.601146000
Si	53.948570000	41.878966000	41.532045000
Si	51.834687000	45.916501000	43.553118000
Si	53.912959000	46.935998000	41.530166000
O	46.908225000	38.507032000	36.863726000
O	44.167530000	39.502281000	34.967464000
O	54.388105000	39.426111000	37.242690000
O	51.592247000	38.769260000	35.804120000
O	42.320060000	39.384879000	41.256227000
O	50.384910000	41.190060000	35.498517000
O	48.192130000	40.860507000	36.935912000
O	48.088652000	42.245185000	34.652731000
O	48.436276000	39.589317000	34.598347000
O	46.846584000	40.039696000	39.069132000
O	45.520962000	40.814238000	36.900519000
O	41.626602000	40.046958000	38.827852000
O	42.853598000	41.071231000	36.656340000
O	44.505496000	42.192500000	34.917733000
O	44.153470000	49.321698000	34.973757000
O	43.730726000	46.749273000	32.357731000
O	44.060108000	44.416678000	33.569214000
O	46.194315000	45.926901000	32.950783000
O	48.401768000	44.415635000	33.115943000
O	48.568797000	46.819554000	32.025376000
O	50.377147000	47.641543000	35.526149000
O	48.175992000	47.959094000	36.957396000
O	48.088952000	46.581991000	34.663011000
O	48.432349000	49.236147000	34.614802000
O	46.833691000	48.799318000	39.103009000
O	46.912595000	50.313749000	36.885547000
O	45.502475000	48.031781000	36.923611000
O	41.623822000	48.812340000	38.818107000
O	42.847027000	47.744378000	36.659952000
O	44.516442000	46.638292000	34.924715000
O	43.729239000	42.082340000	32.353007000
O	46.191860000	42.906509000	32.947741000
O	48.565257000	42.012134000	32.017197000
O	54.246560000	44.415696000	38.612204000
O	56.349874000	42.956005000	39.396719000
O	55.466099000	47.982487000	35.208825000
O	51.850718000	48.692155000	33.546506000
O	52.922187000	47.687858000	35.820972000
O	51.600665000	50.053622000	35.804178000
O	54.819352000	46.723598000	37.413309000
O	54.388911000	49.398708000	37.235597000
O	56.350895000	45.884131000	39.411482000
O	55.466994000	40.837158000	35.212694000
O	51.857385000	40.113932000	33.539221000
O	52.923761000	41.129008000	35.814325000
O	54.808634000	42.105056000	37.410014000
O	45.912730000	42.094287000	40.521834000
O	44.212212000	40.223277000	39.562013000

O	45.980106000	39.603085000	41.513415000
O	42.417342000	41.959718000	40.551753000
O	42.241409000	49.367405000	41.288090000
O	43.642508000	48.466851000	43.522425000
O	48.782547000	48.625920000	42.970741000
O	42.801917000	45.975152000	42.991335000
O	41.610521000	44.409703000	41.146970000
O	43.768995000	46.609051000	45.400926000
O	45.399955000	46.447372000	43.274250000
O	48.577977000	46.846865000	44.946350000
O	47.863143000	46.180242000	42.386247000
O	45.974526000	44.437945000	41.510157000
O	45.868205000	46.850381000	40.640092000
O	44.195899000	48.668729000	39.601558000
O	45.938881000	49.355794000	41.545680000
O	42.396855000	46.839907000	40.472656000
O	43.304627000	40.381867000	43.603203000
O	48.768833000	40.201596000	43.012128000
O	42.786527000	42.937148000	43.052069000
O	43.702366000	42.216272000	45.456447000
O	45.326167000	42.143088000	43.336377000
O	48.671370000	41.919050000	45.060169000
O	48.027636000	42.742534000	42.555180000
O	53.547155000	40.334065000	41.881953000
O	50.541049000	42.147817000	43.126495000
O	52.935951000	42.900772000	42.305677000
O	51.670925000	44.390608000	44.118549000
O	53.872640000	42.100708000	39.908149000
O	55.486697000	42.151441000	42.037727000
O	52.433148000	46.825016000	44.783354000
O	53.527703000	48.486089000	41.880190000
O	50.404953000	46.502928000	43.013412000
O	52.863926000	45.928569000	42.273009000
O	53.864883000	46.727494000	39.904067000
O	55.436493000	46.645308000	42.061594000
O	52.596302000	41.996101000	44.829754000
Ga	45.646087000	43.902709000	39.532202000
H	44.111359000	44.050505000	39.251973000
C	47.337204000	44.233122000	38.574253000
H	48.018267000	43.383611000	38.786007000
H	47.824038000	45.124509000	39.020889000
C	47.157819000	44.423538000	37.061404000
H	46.665089000	43.553250000	36.590740000
H	46.544741000	45.313226000	36.827757000
H	48.131838000	44.555943000	36.555624000

TS3-GaH$_2$

Electronic energy (r^{2}SCAN-D4/def2-SVP): -48257.21198461 E_h
Electronic energy (ωB97X-D4rev/def2-mTZVPP): -48300.932722333200 E_h
G_{corr} (673.15 K, 1 atm): 1.97701341 E_h
Number of atoms: 385

Cartesian coordinates (Å)

Si	48.559265000	36.024591000	35.314958000
Si	46.544167000	36.956392000	37.348539000
Si	44.103160000	36.091830000	35.632828000
Si	44.609980000	38.485962000	33.723048000
Si	47.762290000	38.529272000	33.539726000
Si	48.747428000	38.756511000	30.511103000
Si	51.808900000	38.123213000	30.329902000
Si	54.677580000	38.460900000	38.545803000
Si	57.776440000	38.489659000	38.804717000
Si	56.558437000	37.022988000	34.996714000
Si	52.598177000	36.083174000	32.664343000
Si	51.484825000	37.158429000	35.486521000
Si	54.137315000	36.094274000	36.719562000
Si	45.621296000	36.078499000	40.040474000
Si	41.742179000	38.009347000	41.946793000
Si	43.520428000	38.860105000	44.078437000
Si	47.727530000	38.461010000	46.625177000
Si	48.752971000	38.676048000	43.616719000
Si	46.052239000	38.227274000	42.318844000
Si	51.697433000	37.984378000	43.459995000
Si	53.649254000	38.781223000	41.352420000
Si	56.183730000	38.107012000	43.085941000
Si	44.613576000	50.313212000	33.737051000
Si	47.761683000	50.273738000	33.556038000
Si	48.745027000	50.063531000	30.523078000
Si	43.866391000	46.933390000	30.714291000
Si	48.873228000	46.969575000	30.426938000
Si	46.325871000	45.966455000	29.027652000
Si	48.557053000	52.785693000	35.316566000
Si	46.532602000	51.819994000	37.373718000
Si	44.106448000	52.725242000	35.641045000
Si	43.868353000	41.885639000	30.711087000
Si	48.880286000	41.858203000	30.414588000
Si	46.326013000	42.857360000	29.024067000
Si	51.887938000	42.864827000	30.525147000
Si	57.972187000	42.906317000	39.189936000
Si	58.733692000	47.857653000	36.909279000
Si	56.878805000	48.676176000	34.758521000
Si	55.871015000	48.297907000	31.973641000
Si	52.768088000	48.326947000	32.240644000
Si	56.558126000	51.799102000	34.995914000
Si	52.600734000	52.738487000	32.659743000
Si	51.491308000	51.660862000	35.478444000
Si	54.137607000	52.728413000	36.715350000
Si	51.807446000	50.699738000	30.331662000
Si	54.679542000	50.358377000	38.536326000
Si	57.777949000	50.331078000	38.802884000
Si	51.887962000	45.955142000	30.528129000
Si	57.970391000	45.927392000	39.183620000
Si	58.734356000	40.966098000	36.915399000

Si	56.879306000	40.146873000	34.762162000
Si	55.875056000	40.524123000	31.974000000
Si	52.771503000	40.492557000	32.237012000
Si	48.657443000	40.943939000	48.514939000
Si	41.738956000	50.804174000	41.947143000
Si	43.619326000	50.027088000	44.076216000
Si	47.727365000	50.362024000	46.613526000
Si	48.757168000	50.155450000	43.587719000
Si	46.056949000	50.748435000	42.355108000
Si	44.734908000	45.943280000	46.554385000
Si	47.904483000	45.952063000	46.157303000
Si	48.666294000	47.886184000	48.512425000
Si	45.614902000	52.766998000	40.040502000
Si	44.751998000	42.859530000	46.543437000
Si	47.919882000	42.854695000	46.164679000
Si	58.956568000	41.840834000	41.949107000
Si	56.315817000	42.833712000	43.277128000
Si	55.798736000	48.298675000	45.165039000
Si	52.640012000	48.364741000	45.322955000
Si	51.670169000	48.626416000	48.349588000
Si	51.717322000	50.838076000	43.454454000
Si	53.656926000	50.040027000	41.343369000
Si	56.183367000	50.709955000	43.088082000
Si	58.957819000	46.981701000	41.931815000
Si	56.308530000	45.965777000	43.264886000
Si	55.797165000	40.498670000	45.165619000
Si	52.664018000	40.433747000	45.330751000
Si	51.668463000	40.195778000	48.347705000
O	49.980709000	36.638497000	35.866168000
O	47.519143000	35.880047000	36.588660000
O	46.663690000	36.828031000	38.973059000
O	44.982974000	36.670389000	36.918243000
O	44.495031000	36.929918000	34.251158000
O	46.165562000	38.856358000	33.295399000
O	47.920487000	37.019560000	34.158843000
O	48.586290000	38.632225000	32.138859000
O	50.190995000	38.127463000	30.082686000
O	52.222093000	36.837178000	31.251034000
O	52.298815000	39.555193000	30.981453000
O	54.601595000	36.886934000	38.083973000
O	56.173623000	38.728373000	39.145507000
O	56.918451000	38.601543000	35.309380000
O	54.993547000	36.665491000	35.423532000
O	51.810053000	36.855905000	33.905080000
O	52.535571000	36.329196000	36.434803000
O	42.383811000	37.841556000	43.461674000
O	45.021437000	38.451017000	43.553241000
O	48.583031000	38.715180000	45.238861000
O	47.524014000	37.858403000	42.908845000
O	45.567545000	36.937591000	41.440823000
O	50.088629000	37.814537000	43.236172000
O	52.389040000	37.940530000	41.962457000
O	52.047798000	39.410057000	44.210023000
O	53.570515000	38.743602000	39.715905000
O	55.058663000	38.118016000	41.872720000
O	44.494826000	51.868994000	34.269544000
O	46.166523000	49.956758000	33.287419000
O	47.921180000	51.794163000	34.154594000

O	48.590096000	50.152473000	32.156064000
O	48.604207000	48.517516000	29.977964000
O	45.412763000	46.591590000	30.242585000
O	47.901023000	46.000357000	29.523224000
O	45.881571000	44.411900000	28.741565000
O	49.987917000	52.181683000	35.853396000
O	47.532268000	52.873922000	36.608819000
O	46.645405000	51.960820000	39.003241000
O	44.983336000	52.156837000	36.933257000
O	48.597935000	40.307336000	29.985368000
O	45.413346000	42.227229000	30.236234000
O	47.903047000	42.823508000	29.513804000
O	50.436081000	42.225874000	30.090139000
O	51.675223000	44.409171000	31.032119000
O	58.589544000	44.417581000	39.361445000
O	58.129702000	47.833436000	35.379981000
O	58.285881000	46.472755000	37.674438000
O	58.214082000	49.189204000	37.703330000
O	56.999284000	48.628099000	33.134265000
O	56.913084000	50.219927000	35.310404000
O	54.349613000	48.583202000	32.575523000
O	52.559771000	46.760328000	31.799966000
O	54.993581000	52.159975000	35.418728000
O	51.814807000	51.957188000	33.895669000
O	52.536826000	52.496899000	36.426874000
O	50.186701000	50.704638000	30.101190000
O	52.232342000	51.989532000	31.242314000
O	52.292767000	49.267681000	30.987811000
O	54.602901000	51.932949000	38.077894000
O	56.174520000	50.089113000	39.137886000
O	50.429815000	46.592922000	30.115060000
O	58.123701000	40.995698000	35.388381000
O	58.301045000	42.351091000	37.687312000
O	58.208478000	39.635047000	37.706558000
O	57.001137000	40.197609000	33.137628000
O	54.352725000	40.242247000	32.573552000
O	52.548680000	42.058494000	31.802645000
O	50.275317000	40.955401000	48.778564000
O	48.232199000	42.295585000	47.672837000
O	48.202987000	39.570188000	47.743084000
O	42.421702000	50.960472000	43.446288000
O	45.111100000	50.582641000	43.670765000
O	48.550628000	50.100268000	45.205761000
O	47.582144000	51.018117000	42.848171000
O	45.561314000	52.052424000	41.517434000
O	44.314176000	44.401382000	46.892498000
O	46.276543000	45.993755000	45.983499000
O	48.445983000	44.407982000	46.025836000
O	50.278572000	47.877867000	48.802786000
O	48.277046000	46.546293000	47.635795000
O	48.222574000	49.258925000	47.730346000
O	46.290034000	42.812995000	45.938595000
O	58.659748000	41.945792000	40.316829000
O	57.908199000	42.728744000	42.882528000
O	55.896681000	44.399831000	43.468805000
O	55.998644000	46.730873000	44.686805000
O	54.243495000	48.606950000	45.623838000
O	56.192472000	49.266401000	43.892896000

O	51.811233000	48.594701000	46.703790000
O	50.109979000	50.989460000	43.228816000
O	52.408439000	50.894653000	41.959458000
O	52.065320000	49.418074000	44.209941000
O	53.573576000	50.076888000	39.708286000
O	55.070588000	50.692856000	41.864069000
O	58.676680000	46.895106000	40.294993000
O	57.888495000	46.079574000	42.826383000
O	55.956206000	42.072541000	44.689228000
O	54.258177000	40.127005000	45.630193000
O	56.213687000	39.553643000	43.884157000
O	51.822904000	40.218513000	46.704575000
H	48.702871000	34.676568000	34.772566000
H	44.419875000	34.676323000	35.346829000
H	42.683628000	36.258975000	36.022159000
H	43.726523000	38.680217000	32.577941000
H	47.730594000	38.010996000	29.830569000
H	52.421136000	38.002380000	29.030380000
H	58.465353000	38.743363000	40.066967000
H	58.088382000	37.108509000	38.286195000
H	56.651061000	36.758841000	33.558678000
H	57.541582000	36.246995000	35.782467000
H	52.141234000	34.673922000	32.562045000
H	54.071037000	36.146097000	32.821438000
H	54.418082000	34.675478000	36.917895000
H	44.265382000	36.148734000	39.494231000
H	46.085045000	34.677316000	40.196440000
H	42.177304000	36.886192000	41.136491000
H	40.290195000	38.107003000	42.090377000
H	43.614692000	38.689573000	45.487247000
H	46.266760000	38.594605000	46.380864000
H	48.065374000	37.113072000	47.131273000
H	52.195251000	36.881484000	44.271734000
H	55.858296000	36.988842000	44.011824000
H	57.529558000	37.965437000	42.519250000
H	41.636485000	38.780154000	36.634495000
H	40.338249000	40.845235000	36.817795000
H	43.726296000	50.140518000	32.578071000
H	47.730503000	50.809921000	29.830551000
H	42.910974000	46.071594000	29.969678000
H	43.636013000	48.346500000	30.474316000
H	46.055518000	46.690813000	27.779371000
H	40.338195000	47.975592000	36.817799000
H	41.636403000	50.040687000	36.634467000
H	48.702714000	54.144377000	34.772532000
H	44.419585000	54.144606000	35.346948000
H	42.683575000	52.561616000	36.022276000
H	43.636069000	40.474361000	30.474327000
H	42.910998000	42.749253000	29.969681000
H	46.055551000	42.130076000	27.779377000
H	52.768240000	42.805898000	29.339147000
H	60.152183000	47.950749000	36.769108000
H	56.115708000	49.276599000	30.928772000
H	56.049418000	46.904457000	31.507081000
H	57.541465000	52.574079000	35.782446000
H	56.650952000	52.062214000	33.558656000
H	52.141090000	54.147065000	32.562019000
H	54.070917000	52.674925000	32.821418000

H	54.418140000	54.145525000	36.917811000
H	52.421044000	50.818602000	29.030363000
H	58.465269000	50.077736000	40.066952000
H	58.088278000	51.712582000	38.286179000
H	52.768217000	46.015092000	29.339142000
H	60.152234000	40.870368000	36.769119000
H	56.049458000	41.916585000	31.507090000
H	56.115777000	39.544444000	30.928783000
H	48.027948000	40.958435000	49.819979000
H	40.290115000	50.713683000	42.090582000
H	42.176850000	51.934815000	41.136521000
H	43.615015000	50.131729000	45.487215000
H	48.065229000	51.707918000	47.131232000
H	46.266685000	50.226321000	46.380825000
H	40.476352000	46.423809000	42.229530000
H	44.578030000	46.685732000	47.785515000
H	48.027871000	47.862537000	49.819961000
H	46.084739000	54.143668000	40.196331000
H	44.265252000	52.672179000	39.494179000
H	40.476382000	42.397038000	42.229542000
H	44.578055000	42.135174000	47.785513000
H	58.818796000	40.408567000	42.221796000
H	60.309668000	42.345550000	42.255160000
H	56.725236000	48.548904000	46.274187000
H	51.671681000	50.045758000	48.769745000
H	52.721673000	47.848745000	49.007593000
H	52.195144000	51.939534000	44.271718000
H	55.858180000	51.832229000	44.011806000
H	57.529465000	50.855664000	42.519236000
H	60.309639000	46.475584000	42.255156000
H	58.818736000	48.412544000	42.221786000
H	56.725177000	40.272281000	46.274313000
H	52.722235000	40.971966000	49.006966000
H	51.671815000	38.775269000	48.769821000
Si	48.782690000	41.001697000	35.367714000
Si	46.883860000	40.012128000	37.402274000
Si	41.582460000	40.157591000	37.182524000
Si	44.246822000	40.849372000	35.827302000
Si	44.622401000	45.932969000	33.467772000
Si	47.809321000	45.944182000	33.182925000
Si	48.769880000	47.816347000	35.422153000
Si	46.863332000	48.738771000	37.470754000
Si	41.568737000	48.677101000	37.187033000
Si	44.252215000	47.938979000	35.865614000
Si	44.645155000	42.876874000	33.454861000
Si	47.821178000	42.900521000	33.163417000
Si	54.817161000	42.894305000	38.833868000
Si	51.693507000	48.540792000	35.166645000
Si	54.401844000	47.956658000	36.446424000
Si	54.817276000	45.934618000	38.835844000
Si	51.698149000	40.275614000	35.160683000
Si	54.401325000	40.861539000	36.452045000
Si	45.794813000	40.538780000	40.153179000
Si	42.685999000	40.431908000	40.062624000
Si	41.775178000	45.945381000	41.713899000
Si	43.828216000	46.920693000	43.830572000
Si	48.873936000	47.032965000	43.365605000
Si	46.202018000	46.016916000	42.109728000

Si	45.700063000	48.369810000	40.224331000
Si	42.634459000	48.416052000	40.036093000
Si	41.773118000	42.906693000	41.762345000
Si	43.740407000	41.889190000	43.840631000
Si	48.955139000	41.786764000	43.427443000
Al	46.262608000	42.828205000	42.202399000
Si	51.895650000	42.867820000	43.579607000
Si	53.939419000	41.874898000	41.533287000
Si	51.814516000	45.923713000	43.545284000
Si	53.896933000	46.942376000	41.528145000
O	46.991646000	38.464373000	36.863200000
O	44.102427000	39.481245000	34.934329000
O	54.390313000	39.428236000	37.253485000
O	51.572364000	38.769742000	35.804220000
O	42.384215000	39.359045000	41.245150000
O	50.390244000	41.200836000	35.480357000
O	48.190517000	40.855856000	36.889374000
O	48.120927000	42.327170000	34.670134000
O	48.418240000	39.645403000	34.536756000
O	46.890356000	40.080244000	39.028097000
O	45.502317000	40.684567000	36.854488000
O	41.684187000	40.055097000	38.827093000
O	42.846167000	41.082225000	36.630184000
O	44.545125000	42.165939000	34.912960000
O	44.156008000	49.312824000	34.968225000
O	43.723429000	46.733468000	32.354720000
O	44.067621000	44.399521000	33.573590000
O	46.187937000	45.936146000	32.979386000
O	48.404008000	44.424410000	33.036098000
O	48.545804000	46.863529000	32.043584000
O	50.381687000	47.631941000	35.507560000
O	48.187427000	47.922562000	36.952009000
O	48.116459000	46.501212000	34.692922000
O	48.394105000	49.177013000	34.592227000
O	46.847722000	48.690421000	39.097679000
O	46.925612000	50.289160000	36.919000000
O	45.523747000	48.002309000	36.887844000
O	41.619005000	48.834855000	38.827632000
O	42.850976000	47.756110000	36.681316000
O	44.470845000	46.620654000	34.930014000
O	43.735323000	42.072519000	32.354281000
O	46.202591000	42.900316000	32.942482000
O	48.573890000	41.984443000	32.033255000
O	54.243138000	44.414305000	38.629593000
O	56.350738000	42.951398000	39.399366000
O	55.467834000	47.981997000	35.212335000
O	51.854112000	48.698677000	33.541456000
O	52.922671000	47.692928000	35.818314000
O	51.586008000	50.051921000	35.801661000
O	54.811298000	46.711481000	37.408241000
O	54.390440000	49.392371000	37.244328000
O	56.349627000	45.888310000	39.411720000
O	55.464546000	40.833436000	35.215996000
O	51.864836000	40.105486000	33.538389000
O	52.921725000	41.121615000	35.822485000
O	54.804624000	42.112373000	37.408751000
O	46.038310000	42.166616000	40.479679000
O	44.267278000	40.301800000	39.591530000

O	46.098523000	39.634176000	41.465499000
O	42.430705000	41.966810000	40.569989000
O	42.302593000	49.410835000	41.278033000
O	43.367766000	48.472375000	43.606233000
O	48.794980000	48.628452000	42.983463000
O	42.842255000	45.947226000	42.967981000
O	41.604017000	44.417136000	41.132660000
O	43.717204000	46.568871000	45.423815000
O	45.383333000	46.755062000	43.335083000
O	48.595418000	46.866124000	44.982147000
O	47.807300000	46.187567000	42.453999000
O	45.778433000	44.474647000	41.909788000
O	45.845188000	46.816171000	40.704985000
O	44.205319000	48.624523000	39.571702000
O	45.945148000	49.371133000	41.483657000
O	42.368986000	46.860142000	40.474410000
O	43.069342000	40.393094000	43.690143000
O	48.813226000	40.196722000	43.020762000
O	42.768696000	42.965621000	43.065863000
O	43.714753000	42.241226000	45.442365000
O	45.233091000	41.896805000	43.227921000
O	48.699757000	41.924976000	45.063504000
O	47.944415000	42.712936000	42.582801000
O	53.532694000	40.331389000	41.885808000
O	50.496286000	42.218908000	43.067434000
O	52.921561000	42.901403000	42.291662000
O	51.682736000	44.398426000	44.120757000
O	53.873182000	42.087936000	39.906203000
O	55.475836000	42.147107000	42.043060000
O	52.422816000	46.838552000	44.765557000
O	53.517952000	48.493312000	41.879832000
O	50.372275000	46.480609000	43.012229000
O	52.833394000	45.938851000	42.253876000
O	53.862321000	46.738776000	39.899579000
O	55.416812000	46.643211000	42.065569000
O	52.544603000	41.984001000	44.803745000
Ga	46.062089000	43.670358000	39.204693000
H	44.751937000	44.457380000	38.950932000
C	47.958327000	44.557900000	39.417504000
H	48.416544000	44.218350000	40.359021000
H	47.876582000	45.644747000	39.279137000
C	48.130968000	43.742247000	38.264700000
H	48.627630000	42.764342000	38.331729000
H	46.639418000	42.920651000	37.815482000
H	48.116855000	44.178852000	37.254648000

I5-GaH$_2$

Electronic energy (r^{2}SCAN-D4/def2-SVP): -48257.25182240 E_h
Electronic energy (ωB97X-D4rev/def2-mTZVPP): -48300.973025172700 E_h
G_{corr} (673.15 K, 1 atm): 1.96913062 E_h
Number of atoms: 385

Cartesian coordinates (Å)

Si	48.562545000	36.032382000	35.303786000
Si	46.528792000	36.978054000	37.340462000
Si	44.101202000	36.092713000	35.631994000
Si	44.624171000	38.502001000	33.728731000
Si	47.774735000	38.544238000	33.545107000
Si	48.746142000	38.764540000	30.513408000
Si	51.804773000	38.123246000	30.330241000
Si	54.679136000	38.451681000	38.537244000
Si	57.777891000	38.490791000	38.802794000
Si	56.560634000	37.024271000	34.996773000
Si	52.598311000	36.083878000	32.662714000
Si	51.487981000	37.162820000	35.485332000
Si	54.139391000	36.093940000	36.721005000
Si	45.623416000	36.072164000	40.023838000
Si	41.741719000	38.013953000	41.950217000
Si	43.615120000	38.839584000	44.071433000
Si	47.726352000	38.457791000	46.612970000
Si	48.756660000	38.669135000	43.584042000
Si	46.064608000	38.177397000	42.322562000
Si	51.724481000	37.999689000	43.463883000
Si	53.662685000	38.793149000	41.341836000
Si	56.184182000	38.108540000	43.085425000
Si	44.617845000	50.314632000	33.735253000
Si	47.767915000	50.276069000	33.559431000
Si	48.744158000	50.062438000	30.524196000
Si	43.866987000	46.934609000	30.716029000
Si	48.875346000	46.963509000	30.429762000
Si	46.325451000	45.965699000	29.027474000
Si	48.560450000	52.789514000	35.313531000
Si	46.531900000	51.840572000	37.354779000
Si	44.103270000	52.728086000	35.635657000
Si	43.868125000	41.887039000	30.713530000
Si	48.874773000	41.861942000	30.422430000
Si	46.325591000	42.857860000	29.025191000
Si	51.887728000	42.865205000	30.526413000
Si	57.974299000	42.908075000	39.194051000
Si	58.733096000	47.853881000	36.910195000
Si	56.875451000	48.676919000	34.757106000
Si	55.869606000	48.297779000	31.973005000
Si	52.764802000	48.325106000	32.245730000
Si	56.560073000	51.797910000	34.996382000
Si	52.599615000	52.737927000	32.660701000
Si	51.490329000	51.658082000	35.481471000
Si	54.138774000	52.728601000	36.718708000
Si	51.804218000	50.700237000	30.332184000
Si	54.680924000	50.370237000	38.531236000
Si	57.779463000	50.329855000	38.801136000
Si	51.887486000	45.954915000	30.526378000
Si	57.974798000	45.924743000	39.187848000
Si	58.734217000	40.968947000	36.916066000

Si	56.876683000	40.146719000	34.760591000
Si	55.873209000	40.523788000	31.973862000
Si	52.768895000	40.494106000	32.241226000
Si	48.668090000	40.939595000	48.516742000
Si	41.741856000	50.785397000	41.946185000
Si	43.711599000	50.015237000	44.069459000
Si	47.726796000	50.362691000	46.609850000
Si	48.767502000	50.157103000	43.576895000
Si	46.086332000	50.777106000	42.366751000
Si	44.756686000	45.950515000	46.550462000
Si	47.932114000	45.950595000	46.148074000
Si	48.676697000	47.891314000	48.514260000
Si	45.619914000	52.769815000	40.023062000
Si	44.755151000	42.858597000	46.546526000
Si	47.935726000	42.852265000	46.171481000
Si	58.957032000	41.840771000	41.953298000
Si	56.320201000	42.832141000	43.276318000
Si	55.797871000	48.291589000	45.170374000
Si	52.644626000	48.349757000	45.338862000
Si	51.672082000	48.629042000	48.354969000
Si	51.741901000	50.824434000	43.459708000
Si	53.666469000	50.026457000	41.335530000
Si	56.183081000	50.707531000	43.088822000
Si	58.959981000	46.981728000	41.934061000
Si	56.317965000	45.963934000	43.263573000
Si	55.796163000	40.500737000	45.171552000
Si	52.678511000	40.442995000	45.351683000
Si	51.669977000	40.193540000	48.353297000
O	49.986157000	36.634575000	35.861932000
O	47.516213000	35.917978000	36.574851000
O	46.663613000	36.833876000	38.964748000
O	44.970218000	36.656145000	36.929400000
O	44.495439000	36.946510000	34.261045000
O	46.176734000	38.853671000	33.276521000
O	47.938678000	37.021757000	34.135868000
O	48.602390000	38.670632000	32.146078000
O	50.186666000	38.127052000	30.081507000
O	52.219587000	36.834435000	31.248131000
O	52.290234000	39.552912000	30.990166000
O	54.608119000	36.872632000	38.092907000
O	56.173735000	38.734152000	39.133536000
O	56.927702000	38.601606000	35.308030000
O	54.996815000	36.668531000	35.427363000
O	51.815473000	36.859942000	33.904197000
O	52.539299000	36.336773000	36.436721000
O	42.426959000	37.883159000	43.448956000
O	45.110939000	38.310457000	43.636078000
O	48.545361000	38.684634000	45.201177000
O	47.578840000	37.837561000	42.809455000
O	45.555859000	36.909941000	41.435634000
O	50.117530000	37.845576000	43.230726000
O	52.413364000	37.948619000	41.967602000
O	52.083727000	39.422366000	44.216537000
O	53.574403000	38.746889000	39.706815000
O	55.072961000	38.130379000	41.860720000
O	44.495770000	51.871478000	34.266677000
O	46.171647000	49.957262000	33.291370000
O	47.922474000	51.799200000	34.153575000

O	48.592904000	50.158482000	32.157119000
O	48.600696000	48.513691000	29.989473000
O	45.411352000	46.593374000	30.240090000
O	47.900205000	45.999344000	29.523578000
O	45.878669000	44.411882000	28.740881000
O	49.988124000	52.184894000	35.857790000
O	47.529182000	52.900498000	36.599631000
O	46.651873000	51.972075000	38.985242000
O	44.977309000	52.173534000	36.935012000
O	48.595796000	40.313071000	29.979655000
O	45.412877000	42.227354000	30.237571000
O	47.901758000	42.828367000	29.516719000
O	50.432481000	42.228161000	30.101701000
O	51.676754000	44.410094000	31.032840000
O	58.597496000	44.417004000	39.369728000
O	58.115824000	47.823816000	35.386130000
O	58.293375000	46.468845000	37.679135000
O	58.219828000	49.187863000	37.704400000
O	56.999608000	48.626624000	33.133299000
O	56.924081000	50.220448000	35.309145000
O	54.347756000	48.584989000	32.571908000
O	52.558485000	46.760988000	31.797980000
O	54.996302000	52.155055000	35.425372000
O	51.815810000	51.957673000	33.899286000
O	52.538797000	52.488885000	36.432195000
O	50.184313000	50.703771000	30.094903000
O	52.227197000	51.991572000	31.242810000
O	52.282902000	49.271580000	30.999489000
O	54.606927000	51.950065000	38.091210000
O	56.174463000	50.086316000	39.129000000
O	50.431493000	46.592784000	30.105833000
O	58.112283000	41.004111000	35.393679000
O	58.308247000	42.353480000	37.692665000
O	58.216562000	39.635040000	37.707064000
O	57.002171000	40.199305000	33.136368000
O	54.351099000	40.237655000	32.571298000
O	52.555995000	42.059384000	31.799813000
O	50.279599000	40.941369000	48.809383000
O	48.277289000	42.301930000	47.675366000
O	48.221572000	39.581186000	47.711262000
O	42.465434000	50.880156000	43.430697000
O	45.172243000	50.685507000	43.715546000
O	48.530078000	50.097909000	45.190346000
O	47.625807000	51.042261000	42.810337000
O	45.567831000	52.042479000	41.493375000
O	44.313053000	44.409100000	46.858790000
O	46.303471000	46.014256000	46.002761000
O	48.460796000	44.404744000	46.018897000
O	50.282010000	47.893948000	48.831990000
O	48.329102000	46.554216000	47.615207000
O	48.229181000	49.258299000	47.722633000
O	46.304614000	42.808970000	45.985709000
O	58.655400000	41.945568000	40.322972000
O	57.916133000	42.727282000	42.897253000
O	55.898335000	44.399267000	43.461361000
O	55.999582000	46.726696000	44.685738000
O	54.245517000	48.583434000	45.652148000
O	56.175910000	49.268357000	43.901176000

O	51.796833000	48.593057000	46.707414000
O	50.134262000	50.967093000	43.238504000
O	52.423988000	50.882898000	41.962852000
O	52.095859000	49.405823000	44.214817000
O	53.576658000	50.072955000	39.701653000
O	55.079255000	50.680395000	41.857168000
O	58.676331000	46.896559000	40.298731000
O	57.900703000	46.075794000	42.837144000
O	55.946886000	42.072854000	44.686834000
O	54.266643000	40.128905000	45.668324000
O	56.199810000	39.552014000	43.889699000
O	51.807963000	40.222940000	46.708019000
H	48.702870000	34.676569000	34.772565000
H	44.419874000	34.676324000	35.346828000
H	42.683627000	36.258977000	36.022157000
H	43.726522000	38.680219000	32.577940000
H	47.730594000	38.010998000	29.830568000
H	52.421136000	38.002381000	29.030379000
H	58.465352000	38.743362000	40.066966000
H	58.088381000	37.108509000	38.286194000
H	56.651059000	36.758841000	33.558677000
H	57.541581000	36.246995000	35.782467000
H	52.141232000	34.673923000	32.562043000
H	54.071036000	36.146098000	32.821437000
H	54.418080000	34.675479000	36.917894000
H	44.265381000	36.148736000	39.494229000
H	46.085043000	34.677317000	40.196439000
H	42.177302000	36.886193000	41.136490000
H	40.290193000	38.107005000	42.090375000
H	43.614690000	38.689574000	45.487246000
H	46.266759000	38.594606000	46.380863000
H	48.065372000	37.113072000	47.131271000
H	52.195250000	36.881484000	44.271733000
H	55.858294000	36.988841000	44.011823000
H	57.529557000	37.965437000	42.519250000
H	41.636484000	38.780154000	36.634494000
H	40.338249000	40.845235000	36.817795000
H	43.726296000	50.140520000	32.578070000
H	47.730503000	50.809923000	29.830551000
H	42.910974000	46.071597000	29.969677000
H	43.636014000	48.346502000	30.474317000
H	46.055518000	46.690815000	27.779370000
H	40.338195000	47.975593000	36.817798000
H	41.636404000	50.040688000	36.634467000
H	48.702715000	54.144378000	34.772532000
H	44.419586000	54.144607000	35.346948000
H	42.683576000	52.561618000	36.022275000
H	43.636069000	40.474363000	30.474325000
H	42.910997000	42.749255000	29.969681000
H	46.055550000	42.130078000	27.779376000
H	52.768240000	42.805899000	29.339146000
H	60.152183000	47.950749000	36.769108000
H	56.115709000	49.276600000	30.928772000
H	56.049419000	46.904457000	31.507081000
H	57.541466000	52.574079000	35.782446000
H	56.650953000	52.062214000	33.558656000
H	52.141091000	54.147066000	32.562019000
H	54.070918000	52.674925000	32.821419000

H	54.418141000	54.145525000	36.917811000
H	52.421045000	50.818603000	29.030363000
H	58.465269000	50.077735000	40.066952000
H	58.088279000	51.712582000	38.286179000
H	52.768218000	46.015093000	29.339142000
H	60.152233000	40.870367000	36.769119000
H	56.049458000	41.916585000	31.507089000
H	56.115777000	39.544445000	30.928782000
H	48.027947000	40.958435000	49.819978000
H	40.290115000	50.713685000	42.090582000
H	42.176851000	51.934816000	41.136520000
H	43.615015000	50.131731000	45.487214000
H	48.065229000	51.707918000	47.131231000
H	46.266685000	50.226322000	46.380825000
H	40.476352000	46.423810000	42.229529000
H	44.578029000	46.685733000	47.785515000
H	48.027870000	47.862537000	49.819960000
H	46.084740000	54.143669000	40.196331000
H	44.265253000	52.672181000	39.494179000
H	40.476382000	42.397039000	42.229542000
H	44.578054000	42.135175000	47.785513000
H	58.818794000	40.408566000	42.221796000
H	60.309668000	42.345549000	42.255160000
H	56.725235000	48.548903000	46.274187000
H	51.671681000	50.045758000	48.769745000
H	52.721672000	47.848744000	49.007593000
H	52.195145000	51.939534000	44.271718000
H	55.858181000	51.832229000	44.011806000
H	57.529466000	50.855664000	42.519236000
H	60.309639000	46.475584000	42.255156000
H	58.818736000	48.412544000	42.221786000
H	56.725175000	40.272280000	46.274313000
H	52.722234000	40.971966000	49.006965000
H	51.671813000	38.775268000	48.769820000
Si	48.770279000	41.003236000	35.411777000
Si	46.870738000	40.037179000	37.435033000
Si	41.576809000	40.155173000	37.180137000
Si	44.267866000	40.878000000	35.855564000
Si	44.636767000	45.941558000	33.464993000
Si	47.815000000	45.937677000	33.192068000
Si	48.762746000	47.822221000	35.434077000
Si	46.858125000	48.782312000	37.465521000
Si	41.573371000	48.670472000	37.174864000
Si	44.262974000	47.945782000	35.866769000
Si	44.634844000	42.889515000	33.459642000
Si	47.812593000	42.892650000	33.183551000
Si	54.824799000	42.898606000	38.818205000
Si	51.685984000	48.533374000	35.178808000
Si	54.399214000	47.981431000	36.446676000
Si	54.828610000	45.932879000	38.822160000
Si	51.688086000	40.284267000	35.169313000
Si	54.396812000	40.840691000	36.448595000
Si	45.728767000	40.478525000	40.159707000
Si	42.645995000	40.422023000	40.050079000
Si	41.766442000	45.945142000	41.707644000
Si	43.911335000	46.861535000	43.781708000
Si	48.904342000	47.035343000	43.334965000
Si	46.307715000	45.987302000	41.940608000

Si	45.706645000	48.447803000	40.217885000
Si	42.626107000	48.404404000	40.037477000
Si	41.775284000	42.892345000	41.757911000
Si	43.825812000	41.940340000	43.832282000
Si	48.990397000	41.777511000	43.425952000
Al	46.385736000	42.799037000	42.100332000
Si	51.923437000	42.859249000	43.603055000
Si	53.952909000	41.891033000	41.529774000
Si	51.831784000	45.916441000	43.554134000
Si	53.915198000	46.926011000	41.527013000
O	46.918788000	38.500148000	36.849514000
O	44.176666000	39.500830000	34.965592000
O	54.381921000	39.395781000	37.230496000
O	51.568163000	38.773288000	35.803190000
O	42.313163000	39.390099000	41.254764000
O	50.380409000	41.203695000	35.497359000
O	48.191271000	40.863180000	36.939921000
O	48.091800000	42.300233000	34.682653000
O	48.411805000	39.636944000	34.579894000
O	46.846602000	40.003781000	39.064731000
O	45.520772000	40.805684000	36.907404000
O	41.629444000	40.055717000	38.826675000
O	42.855656000	41.068769000	36.652218000
O	44.510320000	42.192417000	34.922513000
O	44.159895000	49.320663000	34.972059000
O	43.731707000	46.748218000	32.359720000
O	44.061780000	44.415597000	33.572020000
O	46.195809000	45.929156000	32.960797000
O	48.405381000	44.414912000	33.069710000
O	48.562885000	46.843104000	32.047090000
O	50.374173000	47.626829000	35.526102000
O	48.175498000	47.954506000	36.961491000
O	48.095499000	46.524913000	34.693494000
O	48.409802000	49.187431000	34.596427000
O	46.833416000	48.832186000	39.097784000
O	46.921680000	50.318262000	36.872440000
O	45.504300000	48.035706000	36.926696000
O	41.624199000	48.811065000	38.818063000
O	42.849136000	47.747049000	36.660683000
O	44.515320000	46.635882000	34.929838000
O	43.730195000	42.081381000	32.355669000
O	46.193305000	42.903689000	32.953348000
O	48.560077000	41.986915000	32.038768000
O	54.279239000	44.416921000	38.544804000
O	56.353321000	42.958441000	39.400008000
O	55.452185000	48.000812000	35.201291000
O	51.856325000	48.683632000	33.554117000
O	52.914591000	47.692093000	35.843379000
O	51.576895000	50.049221000	35.803450000
O	54.840050000	46.764241000	37.426935000
O	54.382371000	49.429916000	37.222819000
O	56.354666000	45.883030000	39.416172000
O	55.452421000	40.819541000	35.205527000
O	51.862494000	40.121908000	33.546579000
O	52.914823000	41.124333000	35.836927000
O	54.826855000	42.066026000	37.423857000
O	45.941121000	42.092275000	40.484305000
O	44.214345000	40.224287000	39.566487000

O	45.982825000	39.617537000	41.521554000
O	42.426830000	41.964759000	40.555924000
O	42.234931000	49.359986000	41.290935000
O	43.682522000	48.460295000	43.517752000
O	48.774882000	48.629107000	42.968264000
O	42.791633000	45.980411000	42.993893000
O	41.610564000	44.411238000	41.144346000
O	43.786702000	46.598910000	45.389292000
O	45.396184000	46.397778000	43.237824000
O	48.597266000	46.840989000	44.939442000
O	47.871995000	46.181082000	42.378453000
O	46.025608000	44.438751000	41.428625000
O	45.886974000	46.863915000	40.615522000
O	44.196345000	48.679540000	39.605412000
O	45.943747000	49.358978000	41.549619000
O	42.403661000	46.837874000	40.470184000
O	43.343605000	40.388298000	43.590497000
O	48.770021000	40.200879000	43.010990000
O	42.774025000	42.940704000	43.060225000
O	43.723223000	42.218642000	45.446979000
O	45.325767000	42.194098000	43.300536000
O	48.695319000	41.928415000	45.052138000
O	48.042819000	42.741001000	42.543576000
O	53.540055000	40.345286000	41.865828000
O	50.554090000	42.138441000	43.103199000
O	52.958805000	42.910843000	42.328570000
O	51.646140000	44.391049000	44.115669000
O	53.867991000	42.136618000	39.910848000
O	55.495867000	42.142055000	42.033924000
O	52.416197000	46.820011000	44.794768000
O	53.521121000	48.477663000	41.862515000
O	50.413639000	46.517198000	42.994469000
O	52.880155000	45.922249000	42.292512000
O	53.859858000	46.693764000	39.905412000
O	55.442617000	46.654439000	42.059144000
O	52.579094000	42.002486000	44.843468000
Ga	45.654427000	43.869728000	39.486446000
H	44.120925000	44.041261000	39.236937000
C	50.420589000	43.690601000	39.274156000
H	50.712962000	43.066592000	38.417722000
H	46.960746000	44.110268000	38.657802000
H	50.118581000	43.163217000	40.190140000
C	50.426260000	45.026939000	39.206084000
H	50.724219000	45.560250000	38.292254000
H	50.128998000	45.645932000	40.064163000

TS4-GaH$_2$

Electronic energy (r^{2}SCAN-D4/def2-SVP): -48257.16774939 E_h
Electronic energy (ωB97X-D4rev/def2-mTZVPP): -48300.884890247000 E_h
G_{corr} (673.15 K, 1 atm): 1.97142162 E_h
Number of atoms: 385

Cartesian coordinates (Å)

Si	48.557321000	36.020630000	35.325351000
Si	46.562003000	36.948750000	37.356083000
Si	44.106928000	36.093662000	35.636453000
Si	44.589155000	38.490679000	33.736737000
Si	47.748961000	38.542230000	33.578174000
Si	48.733777000	38.758408000	30.535650000
Si	51.809853000	38.127196000	30.334108000
Si	54.677696000	38.460781000	38.542313000
Si	57.776522000	38.489573000	38.804208000
Si	56.559365000	37.023452000	34.996520000
Si	52.598182000	36.083411000	32.662846000
Si	51.483875000	37.158956000	35.484090000
Si	54.136792000	36.094327000	36.718950000
Si	45.617551000	36.071811000	40.040089000
Si	41.741269000	38.009254000	41.949300000
Si	43.562991000	38.846378000	44.076392000
Si	47.726401000	38.457576000	46.616391000
Si	48.755640000	38.663488000	43.593365000
Si	46.047703000	38.201527000	42.318684000
Si	51.705786000	37.987039000	43.460132000
Si	53.655067000	38.784919000	41.349514000
Si	56.184256000	38.106594000	43.085339000
Si	44.616230000	50.312330000	33.737895000
Si	47.761731000	50.296112000	33.550973000
Si	48.746431000	50.067189000	30.521624000
Si	43.864949000	46.931913000	30.717483000
Si	48.872252000	46.968672000	30.435214000
Si	46.325352000	45.966756000	29.029111000
Si	48.553852000	52.791078000	35.314011000
Si	46.527027000	51.845141000	37.366599000
Si	44.103020000	52.729708000	35.639176000
Si	43.869381000	41.886916000	30.713964000
Si	48.877326000	41.865171000	30.430804000
Si	46.327942000	42.856798000	29.025780000
Si	51.887272000	42.861818000	30.525382000
Si	57.969945000	42.904909000	39.189847000
Si	58.733544000	47.856921000	36.910212000
Si	56.875491000	48.675973000	34.758865000
Si	55.870439000	48.297903000	31.973425000
Si	52.767337000	48.324393000	32.240792000
Si	56.556882000	51.799251000	34.996296000
Si	52.600277000	52.738228000	32.657997000
Si	51.487919000	51.658852000	35.474026000
Si	54.132179000	52.729690000	36.712778000
Si	51.807101000	50.699950000	30.330934000
Si	54.677333000	50.358605000	38.532545000
Si	57.777948000	50.331402000	38.802425000
Si	51.886836000	45.953097000	30.527548000
Si	57.971073000	45.924539000	39.185008000
Si	58.735052000	40.965274000	36.914709000

Si	56.884049000	40.146941000	34.760195000
Si	55.874925000	40.524183000	31.975440000
Si	52.774324000	40.495080000	32.241522000
Si	48.660999000	40.940889000	48.513303000
Si	41.741194000	50.792115000	41.946459000
Si	43.684510000	50.029638000	44.071527000
Si	47.727129000	50.361803000	46.609614000
Si	48.761821000	50.150490000	43.577124000
Si	46.073916000	50.777067000	42.366489000
Si	44.741063000	45.950378000	46.551436000
Si	47.918932000	45.946214000	46.153606000
Si	48.672359000	47.886939000	48.512593000
Si	45.616449000	52.771432000	40.032986000
Si	44.740091000	42.865539000	46.547594000
Si	47.915850000	42.852045000	46.164360000
Si	58.956051000	41.840839000	41.950920000
Si	56.317275000	42.833213000	43.279553000
Si	55.796977000	48.298910000	45.169451000
Si	52.648216000	48.359504000	45.335213000
Si	51.670553000	48.627220000	48.353267000
Si	51.732224000	50.829390000	43.458208000
Si	53.665403000	50.035270000	41.340816000
Si	56.183648000	50.710081000	43.088047000
Si	58.957989000	46.981102000	41.935302000
Si	56.313226000	45.965267000	43.267951000
Si	55.796103000	40.496575000	45.167148000
Si	52.668519000	40.433836000	45.336318000
Si	51.668612000	40.195498000	48.349578000
O	49.982577000	36.634407000	35.866822000
O	47.528256000	35.855816000	36.607007000
O	46.663997000	36.824386000	38.983292000
O	44.999332000	36.680029000	36.911584000
O	44.487620000	36.929035000	34.250742000
O	46.151103000	38.886623000	33.358030000
O	47.902174000	37.026689000	34.187833000
O	48.548045000	38.629212000	32.160153000
O	50.187488000	38.142540000	30.116913000
O	52.226252000	36.835739000	31.247431000
O	52.315942000	39.554880000	30.982619000
O	54.599614000	36.885736000	38.085236000
O	56.173629000	38.729014000	39.142661000
O	56.921909000	38.601712000	35.307719000
O	54.995235000	36.666690000	35.424931000
O	51.808524000	36.856917000	33.901977000
O	52.536366000	36.329574000	36.431307000
O	42.407833000	37.851883000	43.454235000
O	45.069286000	38.388309000	43.604185000
O	48.556251000	38.690742000	45.211816000
O	47.548810000	37.853998000	42.840101000
O	45.545945000	36.915675000	41.449038000
O	50.099106000	37.809476000	43.232690000
O	52.397484000	37.943878000	41.963725000
O	52.051020000	39.413691000	44.212507000
O	53.572909000	38.748969000	39.713777000
O	55.063891000	38.119425000	41.868421000
O	44.497415000	51.867235000	34.273870000
O	46.170005000	49.956346000	33.288284000
O	47.901710000	51.819605000	34.145056000

O	48.591440000	50.174357000	32.153097000
O	48.603162000	48.517872000	29.989774000
O	45.410603000	46.592911000	30.242427000
O	47.899529000	46.002783000	29.528934000
O	45.883892000	44.411891000	28.741632000
O	49.979414000	52.169448000	35.845872000
O	47.532049000	52.895958000	36.607513000
O	46.649811000	51.970438000	38.996318000
O	44.975687000	52.185921000	36.943225000
O	48.581304000	40.312183000	30.018853000
O	45.414370000	42.228552000	30.238174000
O	47.904444000	42.824842000	29.517781000
O	50.435893000	42.217602000	30.095643000
O	51.671234000	44.407395000	31.029592000
O	58.590493000	44.415200000	39.362486000
O	58.124154000	47.831777000	35.383091000
O	58.289423000	46.472166000	37.677427000
O	58.215102000	49.188611000	37.704694000
O	56.998260000	48.628488000	33.134850000
O	56.910279000	50.219616000	35.311678000
O	54.348712000	48.584195000	32.574081000
O	52.562487000	46.758488000	31.797830000
O	54.992084000	52.160383000	35.419047000
O	51.811583000	51.954737000	33.891317000
O	52.530839000	52.502561000	36.419830000
O	50.187425000	50.705481000	30.093921000
O	52.232006000	51.991484000	31.239534000
O	52.288067000	49.269104000	30.992492000
O	54.591968000	51.933141000	38.076816000
O	56.173231000	50.092354000	39.132722000
O	50.428942000	46.594751000	30.119335000
O	58.129810000	40.994555000	35.385960000
O	58.300248000	42.349404000	37.687874000
O	58.210308000	39.634148000	37.706052000
O	57.005159000	40.197919000	33.135687000
O	54.355320000	40.245737000	32.584071000
O	52.555463000	42.060417000	31.801769000
O	50.276592000	40.951882000	48.789449000
O	48.244810000	42.294033000	47.669134000
O	48.218026000	39.573327000	47.722578000
O	42.451046000	50.913708000	43.435770000
O	45.155980000	50.670708000	43.710552000
O	48.535701000	50.100545000	45.192705000
O	47.613813000	51.031665000	42.816166000
O	45.566805000	52.061106000	41.510422000
O	44.290481000	44.411449000	46.867033000
O	46.290133000	45.994940000	46.007687000
O	48.454478000	44.400994000	46.017907000
O	50.280165000	47.888259000	48.822888000
O	48.312798000	46.545902000	47.624456000
O	48.227468000	49.254720000	47.720992000
O	46.284130000	42.827542000	45.964743000
O	58.654548000	41.945776000	40.319723000
O	57.910737000	42.727325000	42.889875000
O	55.899415000	44.400093000	43.472309000
O	55.990743000	46.732275000	44.686135000
O	54.247711000	48.606184000	45.649570000
O	56.181040000	49.268242000	43.895819000

O	51.800860000	48.587463000	46.705911000
O	50.125538000	50.966935000	43.230597000
O	52.420270000	50.889984000	41.962873000
O	52.086899000	49.412675000	44.215680000
O	53.574496000	50.070636000	39.706440000
O	55.078775000	50.691545000	41.857077000
O	58.671840000	46.892018000	40.299920000
O	57.895644000	46.079959000	42.839574000
O	55.949990000	42.070686000	44.689223000
O	54.260250000	40.119356000	45.639672000
O	56.211126000	39.552836000	43.884733000
O	51.817667000	40.222258000	46.705529000
H	48.702871000	34.676568000	34.772566000
H	44.419875000	34.676323000	35.346829000
H	42.683628000	36.258975000	36.022159000
H	43.726523000	38.680217000	32.577941000
H	47.730594000	38.010996000	29.830569000
H	52.421136000	38.002380000	29.030380000
H	58.465353000	38.743363000	40.066967000
H	58.088382000	37.108509000	38.286195000
H	56.651061000	36.758841000	33.558678000
H	57.541582000	36.246995000	35.782467000
H	52.141234000	34.673922000	32.562045000
H	54.071037000	36.146097000	32.821438000
H	54.418082000	34.675478000	36.917895000
H	44.265382000	36.148734000	39.494230000
H	46.085045000	34.677316000	40.196440000
H	42.177304000	36.886192000	41.136491000
H	40.290195000	38.107003000	42.090377000
H	43.614692000	38.689573000	45.487247000
H	46.266760000	38.594605000	46.380864000
H	48.065374000	37.113072000	47.131273000
H	52.195251000	36.881483000	44.271734000
H	55.858296000	36.988842000	44.011824000
H	57.529558000	37.965437000	42.519250000
H	41.636484000	38.780154000	36.634495000
H	40.338249000	40.845235000	36.817795000
H	43.726296000	50.140518000	32.578071000
H	47.730503000	50.809921000	29.830551000
H	42.910974000	46.071594000	29.969678000
H	43.636013000	48.346500000	30.474316000
H	46.055518000	46.690813000	27.779371000
H	40.338195000	47.975592000	36.817799000
H	41.636403000	50.040687000	36.634467000
H	48.702714000	54.144377000	34.772532000
H	44.419585000	54.144606000	35.346948000
H	42.683575000	52.561616000	36.022276000
H	43.636069000	40.474361000	30.474327000
H	42.910998000	42.749253000	29.969681000
H	46.055551000	42.130076000	27.779377000
H	52.768240000	42.805898000	29.339147000
H	60.152183000	47.950749000	36.769108000
H	56.115708000	49.276599000	30.928772000
H	56.049418000	46.904457000	31.507081000
H	57.541465000	52.574079000	35.782446000
H	56.650952000	52.062214000	33.558656000
H	52.141090000	54.147065000	32.562019000
H	54.070917000	52.674925000	32.821418000

H	54.418140000	54.145525000	36.917811000
H	52.421044000	50.818602000	29.030363000
H	58.465269000	50.077736000	40.066952000
H	58.088278000	51.712582000	38.286179000
H	52.768217000	46.015092000	29.339142000
H	60.152234000	40.870368000	36.769119000
H	56.049458000	41.916585000	31.507090000
H	56.115777000	39.544444000	30.928783000
H	48.027948000	40.958435000	49.819979000
H	40.290115000	50.713683000	42.090582000
H	42.176850000	51.934815000	41.136521000
H	43.615015000	50.131729000	45.487215000
H	48.065229000	51.707918000	47.131232000
H	46.266685000	50.226321000	46.380825000
H	40.476352000	46.423809000	42.229530000
H	44.578030000	46.685732000	47.785515000
H	48.027871000	47.862537000	49.819962000
H	46.084739000	54.143668000	40.196331000
H	44.265252000	52.672179000	39.494179000
H	40.476382000	42.397038000	42.229542000
H	44.578055000	42.135174000	47.785513000
H	58.818796000	40.408567000	42.221796000
H	60.309668000	42.345550000	42.255160000
H	56.725235000	48.548904000	46.274187000
H	51.671681000	50.045758000	48.769745000
H	52.721673000	47.848745000	49.007593000
H	52.195144000	51.939533000	44.271718000
H	55.858180000	51.832230000	44.011806000
H	57.529465000	50.855664000	42.519236000
H	60.309639000	46.475584000	42.255156000
H	58.818736000	48.412544000	42.221786000
H	56.725177000	40.272281000	46.274313000
H	52.722235000	40.971966000	49.006966000
H	51.671815000	38.775269000	48.769821000
Si	48.781994000	41.012108000	35.405967000
Si	46.896161000	39.995214000	37.414416000
Si	41.574159000	40.154497000	37.189632000
Si	44.232057000	40.827890000	35.834761000
Si	44.622040000	45.927962000	33.472701000
Si	47.801580000	45.957824000	33.199725000
Si	48.767448000	47.857515000	35.424783000
Si	46.855066000	48.782119000	37.472048000
Si	41.571304000	48.674645000	37.182631000
Si	44.261358000	47.940575000	35.870322000
Si	44.644923000	42.871000000	33.470135000
Si	47.822516000	42.910569000	33.193357000
Si	54.814707000	42.893552000	38.830840000
Si	51.692871000	48.538131000	35.169258000
Si	54.399867000	47.956416000	36.445332000
Si	54.818784000	45.931571000	38.832875000
Si	51.692925000	40.278467000	35.161391000
Si	54.402332000	40.858695000	36.447343000
Si	45.729919000	40.495520000	40.139965000
Si	42.648665000	40.422914000	40.052420000
Si	41.772524000	45.947378000	41.711477000
Si	43.893570000	46.891530000	43.799014000
Si	48.890336000	47.026544000	43.348089000
Si	46.275058000	45.978729000	42.008387000

Si	45.703833000	48.417261000	40.229374000
Si	42.628541000	48.407865000	40.036700000
Si	41.777261000	42.898262000	41.761373000
Si	43.771973000	41.907005000	43.850967000
Si	48.967121000	41.775133000	43.421733000
Al	46.305427000	42.799592000	42.163716000
Si	51.908865000	42.866449000	43.591372000
Si	53.943994000	41.877907000	41.534599000
Si	51.834186000	45.924414000	43.552594000
Si	53.910858000	46.938607000	41.528369000
O	47.051088000	38.445504000	36.882682000
O	44.019177000	39.475858000	34.928438000
O	54.391669000	39.424441000	37.247569000
O	51.573295000	38.769755000	35.800528000
O	42.347732000	39.374547000	41.253835000
O	50.392335000	41.204627000	35.498988000
O	48.188739000	40.871340000	36.927476000
O	48.116172000	42.324726000	34.692248000
O	48.428035000	39.652282000	34.566146000
O	46.850495000	40.040722000	39.043448000
O	45.506933000	40.603322000	36.818890000
O	41.620539000	40.053238000	38.838466000
O	42.856690000	41.078439000	36.680077000
O	44.529735000	42.145880000	34.920562000
O	44.162834000	49.314162000	34.972469000
O	43.718936000	46.723410000	32.357638000
O	44.069104000	44.395728000	33.598543000
O	46.183499000	45.934908000	32.972190000
O	48.402071000	44.439191000	33.091295000
O	48.548468000	46.859878000	32.052309000
O	50.375379000	47.636008000	35.514146000
O	48.194426000	47.988792000	36.957869000
O	48.078262000	46.559049000	34.700396000
O	48.422585000	49.222582000	34.592869000
O	46.833042000	48.791402000	39.101308000
O	46.901748000	50.319038000	36.885330000
O	45.526977000	48.011204000	36.903795000
O	41.620352000	48.826459000	38.823693000
O	42.855985000	47.755861000	36.679863000
O	44.486075000	46.626031000	34.933197000
O	43.738235000	42.079763000	32.356156000
O	46.203799000	42.903139000	32.961931000
O	48.581318000	42.017655000	32.047806000
O	54.241993000	44.413822000	38.620492000
O	56.348421000	42.953398000	39.396454000
O	55.461932000	47.982874000	35.208109000
O	51.854322000	48.690043000	33.544434000
O	52.917510000	47.687127000	35.826105000
O	51.592779000	50.051238000	35.801646000
O	54.815112000	46.715386000	37.409388000
O	54.388582000	49.393824000	37.239983000
O	56.350028000	45.883786000	39.409521000
O	55.472903000	40.836821000	35.217803000
O	51.856290000	40.112877000	33.536625000
O	52.927194000	41.118928000	35.811732000
O	54.802013000	42.106618000	37.409987000
O	45.928246000	42.116640000	40.496242000
O	44.215208000	40.248383000	39.549929000

O	46.003513000	39.621698000	41.484121000
O	42.434581000	41.966307000	40.561855000
O	42.258655000	49.377743000	41.286379000
O	43.588543000	48.476286000	43.536134000
O	48.774842000	48.621658000	42.973330000
O	42.821115000	45.976210000	42.979323000
O	41.612865000	44.413873000	41.142871000
O	43.759244000	46.604212000	45.403590000
O	45.414060000	46.531530000	43.295861000
O	48.589629000	46.849059000	44.958070000
O	47.861412000	46.162110000	42.406251000
O	45.916614000	44.435570000	41.630439000
O	45.888741000	46.853754000	40.666379000
O	44.200106000	48.648735000	39.591864000
O	45.923085000	49.378159000	41.527768000
O	42.381188000	46.844948000	40.465211000
O	43.180673000	40.386781000	43.646835000
O	48.816278000	40.188843000	43.011471000
O	42.778809000	42.953687000	43.061607000
O	43.698224000	42.227647000	45.457971000
O	45.282638000	42.020787000	43.301278000
O	48.672648000	41.919164000	45.049957000
O	47.982028000	42.698727000	42.542595000
O	53.539540000	40.333969000	41.887024000
O	50.514888000	42.198692000	43.092682000
O	52.930302000	42.903449000	42.301134000
O	51.680644000	44.399154000	44.122436000
O	53.871150000	42.095223000	39.909303000
O	55.481873000	42.150337000	42.040573000
O	52.443101000	46.831453000	44.778127000
O	53.528161000	48.488887000	41.878800000
O	50.403664000	46.507648000	43.015064000
O	52.856765000	45.932542000	42.265043000
O	53.863488000	46.731464000	39.900747000
O	55.433662000	46.643526000	42.059265000
O	52.564062000	41.988559000	44.816490000
Ga	45.833207000	43.714864000	39.337131000
H	44.544299000	44.525974000	39.044257000
C	47.886996000	44.232552000	39.201525000
H	47.237663000	43.323469000	38.355532000
H	46.292707000	42.820328000	37.868445000
H	48.519838000	43.921548000	40.051053000
C	48.379863000	45.114251000	38.302876000
H	47.815844000	45.381703000	37.395255000
H	49.371606000	45.582121000	38.400439000

I6-GaH$_2$

Electronic energy (r^2SCAN-D4/def2-SVP): -48257.23967596 E_h
Electronic energy (ωB97X-D4rev/def2-mTZVPP): -48300.960078937000 E_h
G_{corr} (673.15 K, 1 atm): 1.96260999 E_h
Number of atoms: 385

Cartesian coordinates (Å)

Si	-1.271844000	-8.379974000	-3.281142000
Si	-3.303069000	-7.433245000	-1.248423000
Si	-5.731321000	-8.320420000	-2.960394000
Si	-5.213145000	-5.920591000	-4.863332000
Si	-2.064170000	-5.872071000	-5.046122000
Si	-1.090981000	-5.648795000	-8.076689000
Si	1.972342000	-6.284667000	-8.264285000
Si	4.846710000	-5.943042000	-0.056906000
Si	7.944657000	-5.911315000	0.208220000
Si	6.725956000	-7.382480000	-3.597356000
Si	2.764865000	-8.324892000	-5.928632000
Si	1.652684000	-7.244709000	-3.105769000
Si	4.306924000	-8.310276000	-1.872896000
Si	-4.209554000	-8.335183000	1.437665000
Si	-8.090550000	-6.394558000	3.355256000
Si	-6.245071000	-5.564265000	5.480677000
Si	-2.104859000	-5.941975000	8.022656000
Si	-1.078851000	-5.733964000	4.998892000
Si	-3.774407000	-6.227680000	3.732883000
Si	1.885242000	-6.406279000	4.868009000
Si	3.829518000	-5.618867000	2.750094000
Si	6.352622000	-6.292738000	4.490190000
Si	-5.223819000	5.903265000	-4.865453000
Si	-2.075851000	5.875328000	-5.047753000
Si	-1.096525000	5.648796000	-8.080557000
Si	-5.972892000	2.518581000	-7.886949000
Si	-0.966268000	2.550138000	-8.169473000
Si	-3.513423000	1.549650000	-9.572160000
Si	-1.284848000	8.380016000	-3.294286000
Si	-3.312093000	7.431611000	-1.246575000
Si	-5.738598000	8.317674000	-2.968343000
Si	-5.967880000	-2.529228000	-7.889339000
Si	-0.962455000	-2.547371000	-8.169557000
Si	-3.509689000	-1.557615000	-9.572728000
Si	2.051096000	-1.547820000	-8.070741000
Si	8.134049000	-1.496338000	0.591079000
Si	8.895571000	3.454267000	-1.692810000
Si	7.038645000	4.270464000	-3.845347000
Si	6.031180000	3.889800000	-6.629150000
Si	2.928290000	3.916145000	-6.360548000
Si	6.716801000	7.393385000	-3.609245000
Si	2.758072000	8.328682000	-5.947180000
Si	1.647988000	7.251015000	-3.128523000
Si	4.292559000	8.323746000	-1.891476000
Si	1.965478000	6.287398000	-8.272857000
Si	4.838724000	5.955354000	-0.072614000
Si	7.938900000	5.929465000	0.197312000
Si	2.049056000	1.542900000	-8.072992000
Si	8.133058000	1.522443000	0.583897000
Si	8.900346000	-3.437494000	-1.683335000

Si	7.043670000	-4.258893000	-3.835494000
Si	6.039230000	-3.884259000	-6.622141000
Si	2.937383000	-3.916346000	-6.358597000
Si	-1.169451000	-3.455348000	9.921169000
Si	-8.096648000	6.378666000	3.343784000
Si	-6.133083000	5.613112000	5.467803000
Si	-2.111224000	5.961693000	8.006140000
Si	-1.071366000	5.751789000	4.972587000
Si	-3.757010000	6.367284000	3.760881000
Si	-5.081642000	1.549573000	7.951779000
Si	-1.906328000	1.548496000	7.551532000
Si	-1.161147000	3.490388000	9.912554000
Si	-4.221337000	8.362436000	1.420656000
Si	-5.084909000	-1.542421000	7.951357000
Si	-1.908728000	-1.550198000	7.582033000
Si	9.121622000	-2.557388000	3.353528000
Si	6.482942000	-1.565311000	4.680118000
Si	5.958955000	3.899892000	6.567472000
Si	2.809324000	3.954976000	6.734527000
Si	1.834539000	4.230887000	9.751815000
Si	1.898134000	6.425570000	4.855415000
Si	3.828313000	5.628909000	2.735868000
Si	6.344632000	6.310569000	4.483483000
Si	9.121000000	2.582398000	3.334155000
Si	6.480438000	1.566412000	4.667355000
Si	5.964373000	-3.900279000	6.572455000
Si	2.846286000	-3.961261000	6.748615000
Si	1.837237000	-4.201810000	9.754640000
O	0.152434000	-7.775070000	-2.727152000
O	-2.313711000	-8.498055000	-2.006605000
O	-3.173049000	-7.569487000	0.377127000
O	-4.860328000	-7.753116000	-1.665897000
O	-5.338475000	-7.479122000	-4.339908000
O	-3.661735000	-5.560808000	-5.314365000
O	-1.901817000	-7.390341000	-4.445476000
O	-1.235604000	-5.757443000	-6.445669000
O	0.354295000	-6.272619000	-8.511894000
O	2.382305000	-7.571194000	-7.340842000
O	2.469750000	-4.853740000	-7.616216000
O	4.779781000	-7.520105000	-0.509179000
O	6.341567000	-5.663746000	0.541031000
O	7.084925000	-5.803342000	-3.285493000
O	5.162020000	-7.742510000	-3.171012000
O	1.982262000	-7.552034000	-4.685333000
O	2.706314000	-8.065983000	-2.152389000
O	-7.418441000	-6.537817000	4.859348000
O	-4.745142000	-6.063339000	5.029012000
O	-1.272852000	-5.716486000	6.618210000
O	-2.267874000	-6.567775000	4.244771000
O	-4.274641000	-7.506054000	2.854990000
O	0.278417000	-6.560269000	4.632179000
O	2.577726000	-6.461342000	3.372908000
O	2.241421000	-4.982246000	5.619432000
O	3.741332000	-5.659105000	1.115070000
O	5.240371000	-6.282092000	3.266279000
O	-5.344965000	7.460910000	-4.337318000
O	-3.670152000	5.543107000	-5.309248000
O	-1.930037000	7.401651000	-4.460810000

O	-1.251150000	5.749858000	-6.448649000
O	-1.240774000	4.099439000	-8.612826000
O	-4.427022000	2.177270000	-8.358635000
O	-1.938382000	1.584004000	-9.076582000
O	-3.959318000	-0.004370000	-9.857715000
O	0.140816000	7.765234000	-2.755374000
O	-2.312184000	8.486798000	-2.005186000
O	-3.188844000	7.564172000	0.383018000
O	-4.865539000	7.767723000	-1.666874000
O	-1.250306000	-4.097594000	-8.600205000
O	-4.421870000	-2.189655000	-8.361269000
O	-1.933483000	-1.583574000	-9.080561000
O	0.596562000	-2.188623000	-8.493792000
O	1.837851000	-0.002010000	-7.567006000
O	8.754691000	0.014086000	0.762615000
O	8.287573000	3.427134000	-3.220352000
O	8.452156000	2.070668000	-0.923270000
O	8.378348000	4.787381000	-0.899979000
O	7.160557000	4.221647000	-5.469361000
O	7.073232000	5.814595000	-3.293791000
O	4.509995000	4.174711000	-6.026676000
O	2.721696000	2.350342000	-6.802680000
O	5.152210000	7.753287000	-3.184874000
O	1.971259000	7.547638000	-4.711271000
O	2.691598000	8.093623000	-2.182470000
O	0.345326000	6.286790000	-8.507308000
O	2.387324000	7.580311000	-7.364302000
O	2.452742000	4.858752000	-7.611821000
O	4.754061000	7.530904000	-0.525501000
O	6.334536000	5.687705000	0.527433000
O	0.591219000	2.180786000	-8.487793000
O	8.286806000	-3.407909000	-3.208714000
O	8.466452000	-2.053720000	-0.909435000
O	8.384239000	-4.770991000	-0.891237000
O	7.167796000	-4.209412000	-5.459836000
O	4.518608000	-4.167733000	-6.019867000
O	2.720464000	-2.350010000	-6.795401000
O	0.445245000	-3.448888000	10.198723000
O	-1.580875000	-2.090779000	9.092775000
O	-1.617006000	-4.816118000	9.121096000
O	-7.377495000	6.481227000	4.830002000
O	-4.670274000	6.276269000	5.110047000
O	-1.308092000	5.699891000	6.586319000
O	-2.216522000	6.628844000	4.202234000
O	-4.272636000	7.637231000	2.891483000
O	-5.522200000	0.008458000	8.264861000
O	-3.534962000	1.614070000	7.403242000
O	-1.381079000	0.000732000	7.420904000
O	0.444817000	3.495322000	10.228559000
O	-1.511282000	2.148863000	9.021147000
O	-1.608577000	4.855377000	9.117105000
O	-3.538880000	-1.592348000	7.377179000
O	8.817683000	-2.453760000	1.723008000
O	8.077114000	-1.671004000	4.293629000
O	6.063847000	0.001756000	4.870711000
O	6.153827000	2.333339000	6.084888000
O	4.408573000	4.202527000	7.048405000
O	6.340800000	4.870512000	5.294457000

O	1.960685000	4.187257000	8.104284000
O	0.291239000	6.569509000	4.631604000
O	2.582953000	6.482985000	3.359281000
O	2.248665000	5.007758000	5.613494000
O	3.736887000	5.664679000	1.101888000
O	5.240338000	6.288253000	3.252209000
O	8.830975000	2.490900000	1.699850000
O	8.064103000	1.680299000	4.244154000
O	6.114317000	-2.327336000	6.089823000
O	4.434607000	-4.278133000	7.062856000
O	6.371116000	-4.845995000	5.289007000
O	1.982661000	-4.173249000	8.110065000
H	-1.128714000	-9.733375000	-3.818005000
H	-5.411872000	-9.735429000	-3.242934000
H	-7.148697000	-8.152941000	-2.568699000
H	-6.107570000	-5.734281000	-6.015160000
H	-2.103554000	-6.403359000	-8.762663000
H	2.586862000	-6.410025000	-9.563509000
H	8.632355000	-5.656757000	1.471616000
H	8.256119000	-7.293341000	-0.307714000
H	6.818105000	-7.647680000	-5.034896000
H	7.709305000	-8.157273000	-2.810863000
H	2.309370000	-9.735831000	-6.029206000
H	4.238345000	-8.262332000	-5.771216000
H	4.586921000	-9.729563000	-1.673653000
H	-5.566252000	-8.259754000	0.903207000
H	-3.745679000	-9.729638000	1.606264000
H	-7.654545000	-7.522217000	2.545176000
H	-9.542116000	-6.301557000	3.498329000
H	-6.217417000	-5.714452000	6.894219000
H	-3.565022000	-5.806990000	7.787696000
H	-1.765470000	-7.286899000	8.539011000
H	2.363995000	-7.518856000	5.678875000
H	6.026971000	-7.409572000	5.418312000
H	7.697437000	-6.433268000	3.924666000
H	-8.197001000	-5.632207000	-1.958371000
H	-9.496338000	-3.567690000	-1.776539000
H	-6.114010000	5.726204000	-6.024256000
H	-2.110606000	6.395609000	-8.772888000
H	-6.927461000	1.654743000	-8.629362000
H	-6.203713000	3.930441000	-8.126447000
H	-3.783707000	2.273984000	-10.820589000
H	-9.500303000	3.562664000	-1.782313000
H	-8.203254000	5.628321000	-1.967518000
H	-1.139402000	9.734454000	-3.833770000
H	-5.422331000	9.732860000	-3.258707000
H	-7.157447000	8.149578000	-2.581767000
H	-6.199268000	-3.941726000	-8.120351000
H	-6.925771000	-1.667623000	-8.626526000
H	-3.781147000	-2.286719000	-10.816845000
H	2.931427000	-1.606116000	-9.258577000
H	10.313701000	3.548734000	-1.834064000
H	6.275614000	4.867990000	-7.674738000
H	6.210767000	2.496224000	-7.094716000
H	7.700351000	8.169769000	-2.823761000
H	6.809925000	7.655773000	-5.047100000
H	2.298527000	9.737379000	-6.044729000
H	4.229266000	8.266567000	-5.784524000

H	4.576236000	9.740488000	-1.689315000
H	2.579792000	6.406304000	-9.573838000
H	8.626160000	5.677509000	1.462330000
H	8.247945000	7.310692000	-0.319720000
H	2.929606000	1.603040000	-9.261388000
H	10.317627000	-3.531650000	-1.828284000
H	6.213442000	-2.491760000	-7.090441000
H	6.280951000	-4.864297000	-7.666976000
H	-1.804591000	-3.439284000	11.224481000
H	-9.548993000	6.305121000	3.488239000
H	-7.663016000	7.526399000	2.532873000
H	-6.223281000	5.727644000	6.884837000
H	-1.773268000	7.307896000	8.526813000
H	-3.571369000	5.824922000	7.778050000
H	-9.360450000	2.015341000	3.630653000
H	-5.258122000	2.284084000	9.185808000
H	-1.808441000	3.464682000	11.218714000
H	-3.756304000	9.736657000	1.590438000
H	-5.575240000	8.263822000	0.889680000
H	-9.358212000	-2.011427000	3.633927000
H	-5.255483000	-2.266479000	9.189459000
H	8.985437000	-3.989663000	3.625009000
H	10.475119000	-2.051730000	3.656525000
H	6.887906000	4.152733000	7.671086000
H	1.833915000	5.649116000	10.165966000
H	2.885247000	3.452940000	10.405684000
H	2.355656000	7.539192000	5.666524000
H	6.018646000	7.433771000	5.406127000
H	7.690312000	6.456958000	3.914140000
H	10.472802000	2.078257000	3.653149000
H	8.980728000	4.014295000	3.618425000
H	6.892416000	-4.123794000	7.677932000
H	2.889572000	-3.423443000	10.410567000
H	1.840541000	-5.621069000	10.175912000
Si	-1.062328000	-3.406262000	-3.191323000
Si	-2.954497000	-4.374493000	-1.168044000
Si	-8.255990000	-4.256990000	-1.408926000
Si	-5.567832000	-3.549846000	-2.742778000
Si	-5.216780000	1.526150000	-5.135871000
Si	-2.034930000	1.536713000	-5.402001000
Si	-1.069647000	3.435117000	-3.171219000
Si	-2.979649000	4.370134000	-1.135431000
Si	-8.268174000	4.262462000	-1.418821000
Si	-5.578753000	3.538047000	-2.731367000
Si	-5.208876000	-1.527659000	-5.144687000
Si	-2.030751000	-1.511504000	-5.408940000
Si	4.979058000	-1.509912000	0.227659000
Si	1.852676000	4.130400000	-3.433688000
Si	4.561829000	3.554563000	-2.158836000
Si	4.981042000	1.526957000	0.228953000
Si	1.852068000	-4.126306000	-3.439120000
Si	4.559951000	-3.549574000	-2.151995000
Si	-4.085056000	-3.921201000	1.565451000
Si	-7.171294000	-3.982311000	1.459311000
Si	-8.070219000	1.536607000	3.108781000
Si	-5.933369000	2.461657000	5.189131000
Si	-0.928987000	2.628464000	4.742894000
Si	-3.532743000	1.575914000	3.368953000

Si	-4.132332000	4.023368000	1.620253000
Si	-7.212041000	3.994768000	1.436970000
Si	-8.059043000	-1.512829000	3.165113000
Si	-6.049636000	-2.477944000	5.254864000
Si	-0.856604000	-2.626051000	4.854222000
Al	-3.483963000	-1.620343000	3.548986000
Si	2.083652000	-1.543363000	4.999840000
Si	4.112610000	-2.523824000	2.933994000
Si	2.004473000	1.516766000	4.952097000
Si	4.081388000	2.533750000	2.927899000
O	-2.907644000	-5.914671000	-1.745292000
O	-5.665196000	-4.938831000	-3.614658000
O	4.550139000	-4.985444000	-1.353871000
O	1.729065000	-5.632352000	-2.793708000
O	-7.498136000	-5.026585000	2.657799000
O	0.547324000	-3.198533000	-3.123499000
O	-1.629550000	-3.555062000	-1.661489000
O	-1.754968000	-2.108972000	-3.910332000
O	-1.423566000	-4.770619000	-4.023954000
O	-2.977285000	-4.394602000	0.459907000
O	-4.299664000	-3.606206000	-1.708386000
O	-8.194057000	-4.346528000	0.238351000
O	-6.973957000	-3.349158000	-1.939119000
O	-5.339981000	-2.252569000	-3.699341000
O	-5.679639000	4.914960000	-3.622739000
O	-6.118158000	2.328373000	-6.245546000
O	-5.783589000	-0.002183000	-5.024696000
O	-3.654126000	1.525606000	-5.629921000
O	-1.442502000	0.014010000	-5.507950000
O	-1.286245000	2.432876000	-6.553219000
O	0.539553000	3.224741000	-3.087456000
O	-1.647942000	3.564550000	-1.640644000
O	-1.751792000	2.140791000	-3.906200000
O	-1.420251000	4.801478000	-4.003902000
O	-3.008914000	4.407598000	0.495364000
O	-2.931797000	5.906600000	-1.726704000
O	-4.316815000	3.604632000	-1.692964000
O	-8.219597000	4.410016000	0.223477000
O	-6.982828000	3.345450000	-1.920739000
O	-5.357861000	2.228441000	-3.676657000
O	-6.113388000	-2.328580000	-6.251331000
O	-3.648647000	-1.504767000	-5.648009000
O	-1.272656000	-2.407703000	-6.553521000
O	4.407284000	0.009269000	0.008981000
O	6.512251000	-1.448452000	0.794741000
O	5.626237000	3.577362000	-3.394070000
O	2.014052000	4.285129000	-5.058897000
O	3.081954000	3.283074000	-2.780905000
O	1.750256000	5.643645000	-2.801366000
O	4.977650000	2.317713000	-1.190027000
O	4.549264000	4.994223000	-1.367543000
O	6.512176000	1.478187000	0.806288000
O	5.628153000	-3.573172000	-3.384057000
O	2.025855000	-4.301894000	-5.060974000
O	3.082728000	-3.284959000	-2.781813000
O	4.967241000	-2.304852000	-1.188616000
O	-3.847863000	-2.319068000	1.911544000
O	-5.602708000	-4.167545000	0.973846000

O	-3.833289000	-4.800963000	2.913816000
O	-7.391442000	-2.441192000	1.972518000
O	-7.590830000	4.956973000	2.689618000
O	-6.175664000	4.057365000	4.922739000
O	-1.054394000	4.221929000	4.370886000
O	-7.040998000	1.574109000	4.391557000
O	-8.222208000	0.002789000	2.544097000
O	-6.060443000	2.194980000	6.796127000
O	-4.439679000	2.015887000	4.659670000
O	-1.233075000	2.442342000	6.350310000
O	-1.965892000	1.771087000	3.798523000
O	-3.830505000	0.023604000	2.889694000
O	-3.942600000	2.446771000	2.034754000
O	-5.640565000	4.251074000	0.998097000
O	-3.904814000	4.952239000	2.941343000
O	-7.443742000	2.429824000	1.867377000
O	-6.573075000	-4.018007000	5.025882000
O	-1.063585000	-4.201822000	4.425796000
O	-7.074530000	-1.454239000	4.478554000
O	-6.132051000	-2.181474000	6.866366000
O	-4.545225000	-2.279292000	4.714992000
O	-1.135721000	-2.491035000	6.485462000
O	-1.826647000	-1.659722000	4.002293000
O	3.712830000	-4.069115000	3.284160000
O	0.699737000	-2.245724000	4.518449000
O	3.097946000	-1.502346000	3.705343000
O	1.834623000	-0.008755000	5.517401000
O	4.035369000	-2.302801000	1.309994000
O	5.650159000	-2.248656000	3.439778000
O	2.600935000	2.424673000	6.183722000
O	3.692096000	4.083139000	3.276183000
O	0.579858000	2.108043000	4.404742000
O	3.039431000	1.523304000	3.676393000
O	4.027443000	2.321174000	1.302257000
O	5.607554000	2.248051000	3.455507000
O	2.753025000	-2.404778000	6.229540000
Ga	-3.803772000	-0.533016000	0.879830000
H	-5.227368000	-0.269254000	0.296064000
C	-1.904134000	-0.354152000	0.470135000
H	-3.730917000	-0.384439000	-2.394052000
H	-4.126473000	0.262699000	-2.357376000
H	-1.217362000	-0.774018000	1.228060000
C	-1.350089000	0.248974000	-0.596758000
H	-1.941580000	0.694825000	-1.406465000
H	-0.256925000	0.323333000	-0.712483000

I7-GaH_2

Electronic energy (r^2SCAN-D4/def2-SVP): -48256.07813137 E_h
Electronic energy (ωB97X-D4rev/def2-mTZVPP): -48299.779483254500 E_h
G_{corr} (673.15 K, 1 atm): 1.96067751 E_h
Number of atoms: 383

Cartesian coordinates (Å)

Si	48.559516000	36.032151000	35.304868000
Si	46.526907000	36.979380000	37.343672000
Si	44.101403000	36.092756000	35.633777000
Si	44.623753000	38.505142000	33.729663000
Si	47.772629000	38.539742000	33.543189000
Si	48.745624000	38.764506000	30.513765000
Si	51.806363000	38.125329000	30.329147000
Si	54.678253000	38.461544000	38.538203000
Si	57.777121000	38.489932000	38.803541000
Si	56.558633000	37.023254000	34.996536000
Si	52.597967000	36.084186000	32.662224000
Si	51.486040000	37.162628000	35.483959000
Si	54.136731000	36.094348000	36.718534000
Si	45.622662000	36.072856000	40.026082000
Si	41.742033000	38.012643000	41.950383000
Si	43.605744000	38.841270000	44.072217000
Si	47.726346000	38.457580000	46.612415000
Si	48.755629000	38.669456000	43.583368000
Si	46.058739000	38.184565000	42.318835000
Si	51.717915000	37.995192000	43.463016000
Si	53.662287000	38.785741000	41.345828000
Si	56.184472000	38.106384000	43.085022000
Si	44.617111000	50.312429000	33.735742000
Si	47.764572000	50.279445000	33.558092000
Si	48.743293000	50.061891000	30.525684000
Si	43.866567000	46.935172000	30.716151000
Si	48.873688000	46.961338000	30.434785000
Si	46.325282000	45.965251000	29.027929000
Si	48.557983000	52.789254000	35.314132000
Si	46.530664000	51.838207000	37.358077000
Si	44.103434000	52.727912000	35.637075000
Si	43.867462000	41.886643000	30.713529000
Si	48.872308000	41.863134000	30.427779000
Si	46.325491000	42.857868000	29.025818000
Si	51.887958000	42.864530000	30.527714000
Si	57.970138000	42.906626000	39.190343000
Si	58.733868000	47.857197000	36.909170000
Si	56.876781000	48.676135000	34.757717000
Si	55.869528000	48.297889000	31.973650000
Si	52.766355000	48.326127000	32.243267000
Si	56.557939000	51.798804000	34.996117000
Si	52.599480000	52.737949000	32.659253000
Si	51.489505000	51.659566000	35.478741000
Si	54.136013000	52.728697000	36.715302000
Si	51.805432000	50.698770000	30.331237000
Si	54.679718000	50.359309000	38.531578000
Si	57.778688000	50.330821000	38.801930000
Si	51.887655000	45.955119000	30.527222000
Si	57.970322000	45.925219000	39.184395000
Si	58.734993000	40.965502000	36.914660000

Si	56.878957000	40.147094000	34.760786000
Si	55.874051000	40.523731000	31.974290000
Si	52.771900000	40.492709000	32.237597000
Si	48.666279000	40.939412000	48.515265000
Si	41.741776000	50.786200000	41.946603000
Si	43.708779000	50.017072000	44.069905000
Si	47.726931000	50.362569000	46.608876000
Si	48.765929000	50.153117000	43.575023000
Si	46.082786000	50.778040000	42.366543000
Si	44.749781000	45.949054000	46.551391000
Si	47.926699000	45.949191000	46.147179000
Si	48.675409000	47.890983000	48.513188000
Si	45.619171000	52.770180000	40.025049000
Si	44.747443000	42.862023000	46.547957000
Si	47.928134000	42.853956000	46.169101000
Si	58.956085000	41.841201000	41.952982000
Si	56.319808000	42.832548000	43.279482000
Si	55.796585000	48.296106000	45.170101000
Si	52.646162000	48.353529000	45.336791000
Si	51.671469000	48.627912000	48.354280000
Si	51.737756000	50.826965000	43.459506000
Si	53.667346000	50.033352000	41.339010000
Si	56.183438000	50.709725000	43.088266000
Si	58.958444000	46.981088000	41.934893000
Si	56.316227000	45.964341000	43.266962000
Si	55.795445000	40.497284000	45.170209000
Si	52.676615000	40.439547000	45.346238000
Si	51.669580000	40.194637000	48.351987000
O	49.982934000	36.638628000	35.859802000
O	47.515995000	35.919911000	36.578039000
O	46.663395000	36.835392000	38.967905000
O	44.969103000	36.652197000	36.933353000
O	44.495924000	36.950836000	34.265574000
O	46.176038000	38.854580000	33.273860000
O	47.931789000	37.017681000	34.135834000
O	48.602748000	38.661519000	32.145345000
O	50.188604000	38.134465000	30.079913000
O	52.218155000	36.837024000	31.249096000
O	52.301160000	39.555570000	30.980839000
O	54.603836000	36.885677000	38.083511000
O	56.173890000	38.733866000	39.137423000
O	56.918520000	38.602033000	35.309337000
O	54.994426000	36.664360000	35.423070000
O	51.814198000	36.859054000	33.903457000
O	52.536050000	36.333161000	36.434362000
O	42.422511000	37.878064000	43.451090000
O	45.103529000	38.324483000	43.630318000
O	48.545360000	38.686935000	45.200837000
O	47.572035000	37.845826000	42.810149000
O	45.553494000	36.910827000	41.437955000
O	50.111484000	37.835594000	43.230243000
O	52.408835000	37.944623000	41.967191000
O	52.072056000	39.418585000	44.216750000
O	53.574057000	38.748196000	39.710811000
O	55.071824000	38.119389000	41.861445000
O	44.495360000	51.868216000	34.269856000
O	46.170017000	49.957889000	33.286421000
O	47.915860000	51.802102000	34.153682000

O	48.593912000	50.163660000	32.158421000
O	48.595550000	48.511561000	29.996998000
O	45.410745000	46.590826000	30.241567000
O	47.899517000	45.998036000	29.526651000
O	45.879367000	44.411738000	28.738923000
O	49.985162000	52.179730000	35.854603000
O	47.529802000	52.896839000	36.602729000
O	46.650786000	51.969763000	38.988401000
O	44.977136000	52.174664000	36.937038000
O	48.590909000	40.314587000	29.985840000
O	45.412302000	42.229755000	30.239244000
O	47.901216000	42.829350000	29.519543000
O	50.431058000	42.227233000	30.108421000
O	51.677499000	44.410030000	31.033116000
O	58.591487000	44.416549000	39.362961000
O	58.125302000	47.831460000	35.381921000
O	58.289303000	46.473073000	37.677188000
O	58.218370000	49.190092000	37.703417000
O	56.999031000	48.628548000	33.133754000
O	56.913284000	50.219675000	35.310874000
O	54.348572000	48.583341000	32.576554000
O	52.559481000	46.761131000	31.798717000
O	54.993613000	52.159793000	35.420036000
O	51.813652000	51.958021000	33.896486000
O	52.535220000	52.496507000	36.427198000
O	50.185506000	50.697027000	30.094855000
O	52.226060000	51.990734000	31.242069000
O	52.292329000	49.270225000	30.992542000
O	54.600880000	51.935568000	38.079996000
O	56.174516000	50.087278000	39.132264000
O	50.430572000	46.593557000	30.110189000
O	58.122649000	40.996445000	35.388800000
O	58.303498000	42.349212000	37.690013000
O	58.215076000	39.632725000	37.705804000
O	57.002607000	40.197636000	33.136496000
O	54.353132000	40.239122000	32.575588000
O	52.558053000	42.059375000	31.800915000
O	50.278646000	40.943399000	48.805407000
O	48.270555000	42.298706000	47.671277000
O	48.222089000	39.578606000	47.712809000
O	42.463753000	50.884013000	43.431601000
O	45.170215000	50.685656000	43.716109000
O	48.529415000	50.096589000	45.188991000
O	47.623535000	51.037189000	42.808382000
O	45.567243000	52.048105000	41.497674000
O	44.298683000	44.410104000	46.862351000
O	46.297678000	46.003869000	46.007008000
O	48.460770000	44.404373000	46.018070000
O	50.281103000	47.893194000	48.830797000
O	48.325976000	46.555067000	47.613259000
O	48.229431000	49.259098000	47.722640000
O	46.296641000	42.820305000	45.985498000
O	58.650791000	41.948125000	40.323057000
O	57.915051000	42.726793000	42.897836000
O	55.899723000	44.399418000	43.469160000
O	55.993448000	46.730246000	44.685960000
O	54.245859000	48.595173000	45.652299000
O	56.176512000	49.268903000	43.898096000

O	51.797201000	48.585283000	46.706582000
O	50.130654000	50.967886000	43.236277000
O	52.422288000	50.887232000	41.963447000
O	52.089961000	49.409520000	44.216955000
O	53.576145000	50.071301000	39.705199000
O	55.079839000	50.690841000	41.856372000
O	58.670044000	46.892803000	40.300142000
O	57.899127000	46.077922000	42.840997000
O	55.945676000	42.070276000	44.687680000
O	54.264863000	40.121858000	45.661084000
O	56.201618000	39.551557000	43.886627000
O	51.810503000	40.228952000	46.706813000
H	48.702871000	34.676568000	34.772566000
H	44.419875000	34.676323000	35.346829000
H	42.683628000	36.258975000	36.022159000
H	43.726523000	38.680216000	32.577941000
H	47.730594000	38.010996000	29.830569000
H	52.421136000	38.002380000	29.030380000
H	58.465353000	38.743363000	40.066967000
H	58.088382000	37.108509000	38.286195000
H	56.651061000	36.758841000	33.558678000
H	57.541582000	36.246995000	35.782467000
H	52.141234000	34.673922000	32.562045000
H	54.071037000	36.146097000	32.821438000
H	54.418082000	34.675478000	36.917895000
H	44.265382000	36.148734000	39.494230000
H	46.085045000	34.677316000	40.196440000
H	42.177304000	36.886192000	41.136491000
H	40.290195000	38.107003000	42.090377000
H	43.614692000	38.689573000	45.487247000
H	46.266760000	38.594605000	46.380864000
H	48.065374000	37.113072000	47.131273000
H	52.195251000	36.881484000	44.271734000
H	55.858296000	36.988842000	44.011824000
H	57.529558000	37.965437000	42.519250000
H	41.636485000	38.780154000	36.634495000
H	40.338249000	40.845235000	36.817795000
H	43.726296000	50.140518000	32.578071000
H	47.730503000	50.809921000	29.830551000
H	42.910974000	46.071594000	29.969678000
H	43.636013000	48.346500000	30.474316000
H	46.055518000	46.690813000	27.779370000
H	40.338195000	47.975592000	36.817799000
H	41.636403000	50.040687000	36.634467000
H	48.702714000	54.144377000	34.772532000
H	44.419585000	54.144606000	35.346948000
H	42.683575000	52.561616000	36.022276000
H	43.636069000	40.474361000	30.474327000
H	42.910998000	42.749253000	29.969681000
H	46.055551000	42.130076000	27.779377000
H	52.768240000	42.805898000	29.339147000
H	60.152183000	47.950749000	36.769108000
H	56.115708000	49.276599000	30.928772000
H	56.049418000	46.904457000	31.507081000
H	57.541465000	52.574079000	35.782446000
H	56.650952000	52.062214000	33.558656000
H	52.141090000	54.147065000	32.562019000
H	54.070917000	52.674925000	32.821418000

H	54.418140000	54.145525000	36.917811000
H	52.421044000	50.818602000	29.030363000
H	58.465269000	50.077736000	40.066952000
H	58.088278000	51.712582000	38.286179000
H	52.768217000	46.015092000	29.339142000
H	60.152234000	40.870368000	36.769119000
H	56.049458000	41.916585000	31.507090000
H	56.115777000	39.544444000	30.928783000
H	48.027948000	40.958435000	49.819979000
H	40.290115000	50.713683000	42.090582000
H	42.176850000	51.934815000	41.136521000
H	43.615015000	50.131729000	45.487215000
H	48.065229000	51.707918000	47.131232000
H	46.266685000	50.226321000	46.380825000
H	40.476352000	46.423809000	42.229530000
H	44.578030000	46.685732000	47.785515000
H	48.027871000	47.862537000	49.819962000
H	46.084739000	54.143668000	40.196331000
H	44.265252000	52.672179000	39.494179000
H	40.476382000	42.397038000	42.229542000
H	44.578055000	42.135174000	47.785513000
H	58.818796000	40.408567000	42.221796000
H	60.309668000	42.345550000	42.255160000
H	56.725235000	48.548904000	46.274187000
H	51.671681000	50.045758000	48.769745000
H	52.721673000	47.848745000	49.007593000
H	52.195144000	51.939534000	44.271718000
H	55.858180000	51.832229000	44.011806000
H	57.529466000	50.855664000	42.519236000
H	60.309639000	46.475584000	42.255156000
H	58.818736000	48.412544000	42.221786000
H	56.725177000	40.272281000	46.274313000
H	52.722235000	40.971967000	49.006966000
H	51.671815000	38.775269000	48.769821000
Si	48.774698000	40.995633000	35.409042000
Si	46.876029000	40.041961000	37.434026000
Si	41.576235000	40.153618000	37.184226000
Si	44.269090000	40.883035000	35.858096000
Si	44.630775000	45.942437000	33.465079000
Si	47.811457000	45.936755000	33.199244000
Si	48.763491000	47.828112000	35.433850000
Si	46.858555000	48.777489000	37.468671000
Si	41.571974000	48.672091000	37.177493000
Si	44.262909000	47.941298000	35.869155000
Si	44.626095000	42.889508000	33.460431000
Si	47.806312000	42.889275000	33.191995000
Si	54.815107000	42.896058000	38.826282000
Si	51.687530000	48.537853000	35.170175000
Si	54.398700000	47.960939000	36.443092000
Si	54.818231000	45.932843000	38.829584000
Si	51.689924000	40.280654000	35.158391000
Si	54.396648000	40.858779000	36.445053000
Si	45.724818000	40.488142000	40.154146000
Si	42.645519000	40.420110000	40.049125000
Si	41.766614000	45.945539000	41.706139000
Si	43.910228000	46.864857000	43.783676000
Si	48.898740000	47.030239000	43.332358000
Si	46.299374000	45.985276000	41.946858000

Si	45.699134000	48.444517000	40.218816000
Si	42.622657000	48.406095000	40.036232000
Si	41.774739000	42.893126000	41.757801000
Si	43.814895000	41.936415000	43.836950000
Si	48.982541000	41.780148000	43.421179000
Al	46.365196000	42.797363000	42.105850000
Si	51.923070000	42.860484000	43.595663000
Si	53.953412000	41.878751000	41.532157000
Si	51.835349000	45.919720000	43.549117000
Si	53.915925000	46.937624000	41.528247000
O	46.910558000	38.502026000	36.852348000
O	44.177524000	39.509065000	34.962719000
O	54.386840000	39.423198000	37.243367000
O	51.571932000	38.773278000	35.801777000
O	42.324418000	39.384390000	41.254351000
O	50.384325000	41.204679000	35.479852000
O	48.211799000	40.842730000	36.940171000
O	48.085429000	42.294162000	34.690397000
O	48.414529000	39.632130000	34.574341000
O	46.846923000	40.014488000	39.063116000
O	45.535042000	40.817140000	36.893810000
O	41.622089000	40.051854000	38.831181000
O	42.862288000	41.063286000	36.666964000
O	44.490298000	42.203449000	34.927010000
O	44.163941000	49.314284000	34.971089000
O	43.730924000	46.755451000	32.360685000
O	44.054242000	44.416857000	33.558999000
O	46.192502000	45.932582000	32.968263000
O	48.398416000	44.412565000	33.084345000
O	48.562270000	46.837267000	32.052510000
O	50.374710000	47.633291000	35.517654000
O	48.180912000	47.957141000	36.963162000
O	48.093501000	46.525543000	34.701416000
O	48.404405000	49.190301000	34.595850000
O	46.830198000	48.822465000	39.100084000
O	46.916134000	50.314351000	36.876962000
O	45.511949000	48.023049000	36.921048000
O	41.617367000	48.816621000	38.820274000
O	42.851900000	47.750573000	36.669490000
O	44.503559000	46.626661000	34.934463000
O	43.726986000	42.076818000	32.355775000
O	46.187285000	42.900538000	32.961752000
O	48.555789000	41.989540000	32.043836000
O	54.243081000	44.415396000	38.611212000
O	56.348396000	42.956481000	39.392467000
O	55.463436000	47.984421000	35.208042000
O	51.851298000	48.693149000	33.544963000
O	52.918306000	47.691449000	35.821961000
O	51.585086000	50.051266000	35.802223000
O	54.815466000	46.722768000	37.410198000
O	54.387810000	49.399844000	37.236090000
O	56.349392000	45.882809000	39.407435000
O	55.464591000	40.834684000	35.212925000
O	51.860545000	40.108968000	33.535939000
O	52.919672000	41.123672000	35.815116000
O	54.802793000	42.103766000	37.408563000
O	45.930351000	42.100030000	40.485361000
O	44.212030000	40.229377000	39.556079000

O	45.978162000	39.620985000	41.512842000
O	42.423595000	41.961369000	40.557960000
O	42.237538000	49.361476000	41.291777000
O	43.674040000	48.462480000	43.519748000
O	48.777175000	48.625476000	42.966310000
O	42.797476000	45.981433000	42.987844000
O	41.608855000	44.411455000	41.143218000
O	43.780497000	46.599881000	45.390909000
O	45.400928000	46.411113000	43.250342000
O	48.585466000	46.841792000	44.937226000
O	47.867319000	46.176798000	42.375076000
O	45.997957000	44.437545000	41.450617000
O	45.868827000	46.863628000	40.625816000
O	44.191783000	48.678976000	39.598114000
O	45.933764000	49.364076000	41.544909000
O	42.396851000	46.839444000	40.465960000
O	43.314857000	40.389325000	43.600378000
O	48.784930000	40.199951000	43.008468000
O	42.774945000	42.944905000	43.059150000
O	43.714338000	42.220787000	45.450583000
O	45.318762000	42.170233000	43.307239000
O	48.680340000	41.928273000	45.046260000
O	48.027811000	42.731195000	42.532831000
O	53.547488000	40.334971000	41.882416000
O	50.542193000	42.160483000	43.102711000
O	52.946290000	42.904033000	42.308438000
O	51.669230000	44.393697000	44.114195000
O	53.872676000	42.100914000	39.908470000
O	55.492952000	42.148035000	42.035366000
O	52.431687000	46.825618000	44.782058000
O	53.529893000	48.487356000	41.878211000
O	50.408788000	46.507852000	43.003067000
O	52.868914000	45.929185000	42.272446000
O	53.865128000	46.728472000	39.902196000
O	55.440166000	46.647068000	42.058357000
O	52.582977000	41.996224000	44.828248000
Ga	45.619528000	43.882978000	39.484660000
H	44.083114000	44.042829000	39.249436000
C	47.230454000	44.230787000	38.442945000
H	47.134470000	44.769006000	37.483709000
C	48.464024000	43.849853000	38.824171000
H	48.663413000	43.307400000	39.761667000
H	49.352711000	44.064380000	38.209374000

I8-GaH$_2$

Electronic energy (r^{2}SCAN-D4/def2-SVP): -48334.51672219 E_h
Electronic energy (ωB97X-D4rev/def2-mTZVPP): -48378.424336373800 E_h
G_{corr} (673.15 K, 1 atm): 1.99426916 E_h
Number of atoms: 389

Cartesian coordinates (Å)

Si	48.555441000	36.029823000	35.299716000
Si	46.555804000	36.959049000	37.347607000
Si	44.104583000	36.092094000	35.629735000
Si	44.588548000	38.472343000	33.735988000
Si	47.750870000	38.540043000	33.557412000
Si	48.742196000	38.762134000	30.511153000
Si	51.811832000	38.115468000	30.334367000
Si	54.669088000	38.466874000	38.550528000
Si	57.772520000	38.487108000	38.806765000
Si	56.557603000	37.023461000	34.996903000
Si	52.601069000	36.080515000	32.656000000
Si	51.486992000	37.152392000	35.467207000
Si	54.125671000	36.089921000	36.710742000
Si	45.621391000	36.080882000	40.042988000
Si	41.741127000	38.007588000	41.950469000
Si	43.498364000	38.869956000	44.082093000
Si	47.726786000	38.461772000	46.626342000
Si	48.756913000	38.672634000	43.618943000
Si	46.038074000	38.262928000	42.299743000
Si	51.682962000	37.975332000	43.460111000
Si	53.638911000	38.787111000	41.360640000
Si	56.182282000	38.107726000	43.086932000
Si	44.621435000	50.322916000	33.727504000
Si	47.767356000	50.278599000	33.539795000
Si	48.750329000	50.062305000	30.511416000
Si	43.867894000	46.930407000	30.713771000
Si	48.877715000	46.968134000	30.407858000
Si	46.324219000	45.966601000	29.024738000
Si	48.558893000	52.784372000	35.313897000
Si	46.532103000	51.813379000	37.373223000
Si	44.107055000	52.724753000	35.639669000
Si	43.871643000	41.887031000	30.705194000
Si	48.885609000	41.852854000	30.378609000
Si	46.324856000	42.858802000	29.019086000
Si	51.887919000	42.865768000	30.522355000
Si	57.972599000	42.907595000	39.187874000
Si	58.733528000	47.859095000	36.908283000
Si	56.876277000	48.676447000	34.758956000
Si	55.870353000	48.297701000	31.972504000
Si	52.765336000	48.329493000	32.236949000
Si	56.557307000	51.799607000	34.995700000
Si	52.600562000	52.738608000	32.660881000
Si	51.491897000	51.662254000	35.481198000
Si	54.138764000	52.728057000	36.715070000
Si	51.806389000	50.701964000	30.329451000
Si	54.681056000	50.354838000	38.537927000
Si	57.777954000	50.331184000	38.803405000
Si	51.887485000	45.953549000	30.526077000
Si	57.970689000	45.929332000	39.182535000
Si	58.733985000	40.964679000	36.916278000

Si	56.887242000	40.147654000	34.760914000
Si	55.871766000	40.524178000	31.975125000
Si	52.766099000	40.491215000	32.244670000
Si	48.660863000	40.943406000	48.516316000
Si	41.739296000	50.805664000	41.943083000
Si	43.567000000	49.998603000	44.079614000
Si	47.727854000	50.358822000	46.624246000
Si	48.754771000	50.170514000	43.612286000
Si	46.053420000	50.719929000	42.351367000
Si	44.770205000	45.953876000	46.554528000
Si	47.919396000	45.964267000	46.189611000
Si	48.663154000	47.873355000	48.519725000
Si	45.614959000	52.761954000	40.044434000
Si	44.778494000	42.849309000	46.542570000
Si	47.936390000	42.841080000	46.155590000
Si	58.957165000	41.841117000	41.947588000
Si	56.319359000	42.833850000	43.277580000
Si	55.802571000	48.304935000	45.159565000
Si	52.646599000	48.378029000	45.314173000
Si	51.673129000	48.626694000	48.344437000
Si	51.707381000	50.845084000	43.449976000
Si	53.654171000	50.048194000	41.345074000
Si	56.184765000	50.712095000	43.087114000
Si	58.957305000	46.982185000	41.931403000
Si	56.303481000	45.966714000	43.264821000
Si	55.794667000	40.500229000	45.164281000
Si	52.648405000	40.434216000	45.320865000
Si	51.670065000	40.195049000	48.347984000
O	49.972704000	36.661206000	35.843565000
O	47.519276000	35.880960000	36.576118000
O	46.667863000	36.813184000	38.969824000
O	44.994162000	36.697030000	36.897893000
O	44.486933000	36.904803000	34.228952000
O	46.148555000	38.888571000	33.372012000
O	47.908753000	37.011821000	34.134880000
O	48.528757000	38.681522000	32.134573000
O	50.187814000	38.106116000	30.130023000
O	52.248314000	36.819920000	31.230205000
O	52.300106000	39.544420000	30.993232000
O	54.557590000	36.899755000	38.075049000
O	56.169138000	38.700521000	39.156007000
O	56.917868000	38.602110000	35.307947000
O	54.992693000	36.668282000	35.424840000
O	51.798157000	36.866556000	33.879032000
O	52.524106000	36.285367000	36.397282000
O	42.371307000	37.838091000	43.470137000
O	44.993516000	38.493906000	43.523193000
O	48.578832000	38.722667000	45.240287000
O	47.504624000	37.897250000	42.908985000
O	45.569611000	36.956997000	41.433022000
O	50.074375000	37.775420000	43.249939000
O	52.372014000	37.948125000	41.960721000
O	52.021561000	39.403221000	44.212806000
O	53.567385000	38.757019000	39.724111000
O	55.045788000	38.120322000	41.884324000
O	44.496156000	51.876837000	34.263333000
O	46.170780000	49.974415000	33.263563000
O	47.930796000	51.795822000	34.145668000

O	48.602333000	50.150290000	32.145494000
O	48.613499000	48.518235000	29.961267000
O	45.415369000	46.601140000	30.237621000
O	47.902335000	45.999582000	29.507718000
O	45.872558000	44.412928000	28.746939000
O	49.988759000	52.184326000	35.856881000
O	47.529494000	52.865348000	36.603852000
O	46.643694000	51.958742000	39.003213000
O	44.982523000	52.146992000	36.929917000
O	48.624088000	40.300292000	29.939587000
O	45.420025000	42.225002000	30.235722000
O	47.907647000	42.821962000	29.483274000
O	50.439817000	42.235105000	30.067579000
O	51.678753000	44.409101000	31.035010000
O	58.589550000	44.419444000	39.358697000
O	58.129369000	47.836041000	35.379159000
O	58.285122000	46.474525000	37.673125000
O	58.213698000	49.190627000	37.702602000
O	56.996283000	48.629841000	33.134824000
O	56.907996000	50.220000000	35.312057000
O	54.347607000	48.582479000	32.569737000
O	52.548930000	46.763349000	31.800846000
O	54.992352000	52.162861000	35.415757000
O	51.815242000	51.959951000	33.898938000
O	52.537220000	52.497129000	36.430790000
O	50.187346000	50.712044000	30.088281000
O	52.229942000	51.988813000	31.244693000
O	52.286985000	49.268006000	30.983826000
O	54.607427000	51.927911000	38.073548000
O	56.175227000	50.088196000	39.142905000
O	50.432722000	46.587312000	30.096233000
O	58.139776000	40.988167000	35.382766000
O	58.299436000	42.352144000	37.684514000
O	58.191905000	39.641223000	37.711067000
O	57.000912000	40.196682000	33.135937000
O	54.349875000	40.247067000	32.579967000
O	52.539283000	42.053587000	31.799913000
O	50.276848000	40.949001000	48.787579000
O	48.250104000	42.297816000	47.669356000
O	48.199982000	39.571193000	47.745500000
O	42.395394000	50.965030000	43.454336000
O	45.064465000	50.484519000	43.620886000
O	48.582401000	50.117340000	45.232466000
O	47.551497000	51.023168000	42.907022000
O	45.559506000	52.026625000	41.514352000
O	44.422968000	44.403859000	46.918639000
O	46.297902000	46.075191000	45.943537000
O	48.414794000	44.411288000	46.018827000
O	50.279153000	47.872922000	48.782254000
O	48.250187000	46.504638000	47.697702000
O	48.201442000	49.234310000	47.727145000
O	46.307420000	42.748124000	45.917682000
O	58.661382000	41.947536000	40.315012000
O	57.911564000	42.732063000	42.881766000
O	55.897046000	44.399650000	43.469353000
O	55.991062000	46.734329000	44.685762000
O	54.248404000	48.638128000	45.606779000
O	56.207087000	49.264900000	43.885107000

O	51.826141000	48.598887000	46.700637000
O	50.098771000	51.004135000	43.219056000
O	52.400661000	50.900337000	41.956353000
O	52.043133000	49.421900000	44.205896000
O	53.572634000	50.082635000	39.710399000
O	55.065633000	50.702339000	41.868647000
O	58.678929000	46.895612000	40.294198000
O	57.882537000	46.084568000	42.824447000
O	55.961076000	42.074659000	44.690960000
O	54.247487000	40.137146000	45.609989000
O	56.219006000	39.553509000	43.886801000
O	51.823648000	40.223878000	46.705603000
H	48.702871000	34.676564000	34.772559000
H	44.419877000	34.676318000	35.346822000
H	42.683629000	36.258968000	36.022154000
H	43.726523000	38.680216000	32.577940000
H	47.730594000	38.010993000	29.830565000
H	52.421136000	38.002376000	29.030376000
H	58.465354000	38.743351000	40.066961000
H	58.088381000	37.108499000	38.286188000
H	56.651061000	36.758835000	33.558672000
H	57.541582000	36.246988000	35.782461000
H	52.141231000	34.673917000	32.562040000
H	54.071036000	36.146090000	32.821432000
H	54.418080000	34.675471000	36.917890000
H	44.265382000	36.148728000	39.494225000
H	46.085044000	34.677309000	40.196435000
H	42.177308000	36.886190000	41.136484000
H	40.290196000	38.106997000	42.090371000
H	43.614692000	38.689566000	45.487242000
H	46.266761000	38.594594000	46.380859000
H	48.065374000	37.113061000	47.131266000
H	52.195251000	36.881476000	44.271729000
H	55.858295000	36.988830000	44.011818000
H	57.529558000	37.965426000	42.519244000
H	41.636485000	38.780154000	36.634495000
H	40.338249000	40.845234000	36.817795000
H	43.726297000	50.140516000	32.578070000
H	47.730505000	50.809918000	29.830551000
H	42.910975000	46.071591000	29.969678000
H	43.636015000	48.346497000	30.474314000
H	46.055520000	46.690810000	27.779370000
H	40.338196000	47.975591000	36.817799000
H	41.636403000	50.040687000	36.634467000
H	48.702717000	54.144372000	34.772533000
H	44.419587000	54.144601000	35.346949000
H	42.683577000	52.561611000	36.022276000
H	43.636070000	40.474358000	30.474327000
H	42.910998000	42.749249000	29.969679000
H	46.055551000	42.130073000	27.779374000
H	52.768241000	42.805893000	29.339143000
H	60.152185000	47.950740000	36.769106000
H	56.115709000	49.276593000	30.928771000
H	56.049419000	46.904450000	31.507080000
H	57.541468000	52.574072000	35.782446000
H	56.650955000	52.062207000	33.558656000
H	52.141094000	54.147060000	32.562019000
H	54.070920000	52.674918000	32.821419000

H	54.418145000	54.145518000	36.917811000
H	52.421047000	50.818597000	29.030362000
H	58.465271000	50.077725000	40.066952000
H	58.088282000	51.712572000	38.286179000
H	52.768219000	46.015088000	29.339141000
H	60.152234000	40.870359000	36.769115000
H	56.049458000	41.916578000	31.507086000
H	56.115778000	39.544437000	30.928779000
H	48.027950000	40.958422000	49.819975000
H	40.290116000	50.713680000	42.090585000
H	42.176850000	51.934813000	41.136523000
H	43.615017000	50.131725000	45.487216000
H	48.065230000	51.707907000	47.131234000
H	46.266687000	50.226310000	46.380825000
H	40.476352000	46.423809000	42.229530000
H	44.578034000	46.685723000	47.785515000
H	48.027873000	47.862524000	49.819961000
H	46.084741000	54.143662000	40.196333000
H	44.265255000	52.672173000	39.494181000
H	40.476382000	42.397038000	42.229542000
H	44.578055000	42.135166000	47.785512000
H	58.818795000	40.408556000	42.221792000
H	60.309670000	42.345538000	42.255156000
H	56.725239000	48.548891000	46.274185000
H	51.671683000	50.045745000	48.769748000
H	52.721675000	47.848731000	49.007592000
H	52.195148000	51.939526000	44.271718000
H	55.858185000	51.832218000	44.011807000
H	57.529469000	50.855653000	42.519236000
H	60.309641000	46.475572000	42.255155000
H	58.818739000	48.412533000	42.221785000
H	56.725176000	40.272268000	46.274309000
H	52.722236000	40.971953000	49.006962000
H	51.671815000	38.775256000	48.769814000
Si	48.799961000	40.990534000	35.378656000
Si	46.884000000	40.019121000	37.410077000
Si	41.567885000	40.146536000	37.199516000
Si	44.208385000	40.796976000	35.829211000
Si	44.639545000	45.913131000	33.461862000
Si	47.817053000	45.925981000	33.152517000
Si	48.772610000	47.810280000	35.388883000
Si	46.869240000	48.723248000	37.455568000
Si	41.578742000	48.670755000	37.177106000
Si	44.260257000	47.942237000	35.848499000
Si	44.649629000	42.854798000	33.462422000
Si	47.834335000	42.882298000	33.136193000
Si	54.816301000	42.899700000	38.841230000
Si	51.695919000	48.545132000	35.162954000
Si	54.400858000	47.948818000	36.446837000
Si	54.815794000	45.940261000	38.840082000
Si	51.715286000	40.267214000	35.175310000
Si	54.415666000	40.880555000	36.455121000
Si	45.759642000	40.594986000	40.147883000
Si	42.672473000	40.419595000	40.060131000
Si	41.761166000	45.927463000	41.714978000
Si	43.750259000	46.906719000	43.852948000
Si	48.897361000	47.056990000	43.467235000
Si	46.211334000	46.028282000	42.194555000

Si	45.761090000	48.327489000	40.228473000
Si	42.668331000	48.404002000	40.045217000
Si	41.764949000	42.899686000	41.746592000
Si	43.695025000	41.891895000	43.866435000
Si	48.975134000	41.782578000	43.426511000
Al	46.225703000	42.774485000	42.244764000
Si	51.892619000	42.883532000	43.560922000
Si	53.944498000	41.873631000	41.535695000
Si	51.823095000	45.935253000	43.544015000
Si	53.891929000	46.953552000	41.523190000
O	47.037281000	38.466801000	36.897828000
O	44.015874000	39.423064000	34.956749000
O	54.410761000	39.456444000	37.270701000
O	51.627553000	38.753847000	35.808374000
O	42.389780000	39.363936000	41.265641000
O	50.405287000	41.166002000	35.541453000
O	48.149068000	40.907158000	36.879396000
O	48.189743000	42.293236000	34.613096000
O	48.452834000	39.611908000	34.570844000
O	46.870009000	40.155107000	39.027686000
O	45.479092000	40.627157000	36.836370000
O	41.649788000	40.025976000	38.844561000
O	42.837240000	41.075253000	36.666410000
O	44.508940000	42.093182000	34.889678000
O	44.177249000	49.323296000	34.964038000
O	43.724302000	46.700017000	32.350770000
O	44.089383000	44.382520000	33.620665000
O	46.195045000	45.913772000	32.948126000
O	48.410130000	44.408756000	32.989739000
O	48.548798000	46.860984000	32.021976000
O	50.383171000	47.634820000	35.490811000
O	48.182549000	47.908809000	36.916525000
O	48.127243000	46.501446000	34.651537000
O	48.386741000	49.179231000	34.578841000
O	46.884812000	48.641401000	39.081865000
O	46.928415000	50.280747000	36.928655000
O	45.508544000	48.005140000	36.900127000
O	41.675459000	48.819481000	38.818133000
O	42.843779000	47.747163000	36.635429000
O	44.508128000	46.632081000	34.912628000
O	43.731226000	42.088949000	32.342855000
O	46.208759000	42.873768000	32.961582000
O	48.560219000	41.966401000	31.989523000
O	54.233125000	44.420098000	38.666521000
O	56.352163000	42.956429000	39.401564000
O	55.466726000	47.979654000	35.212756000
O	51.859053000	48.712087000	33.539566000
O	52.921167000	47.694449000	35.817060000
O	51.585026000	50.053471000	35.804872000
O	54.806259000	46.694963000	37.399987000
O	54.393098000	49.380324000	37.252790000
O	56.349826000	45.896471000	39.411761000
O	55.483989000	40.848811000	35.224039000
O	51.855290000	40.115161000	33.546899000
O	52.941365000	41.126632000	35.812658000
O	54.804172000	42.144348000	37.402252000
O	45.935144000	42.202101000	40.523588000
O	44.244298000	40.287676000	39.572654000

O	46.088423000	39.662592000	41.443566000
O	42.408227000	41.958260000	40.554030000
O	42.350051000	49.432857000	41.265455000
O	43.223475000	48.442061000	43.665380000
O	48.784150000	48.634551000	43.024508000
O	42.804457000	45.900488000	42.989068000
O	41.589211000	44.410097000	41.113574000
O	43.704041000	46.523445000	45.438445000
O	45.294500000	46.807758000	43.306945000
O	48.700894000	46.915849000	45.098832000
O	47.760674000	46.173500000	42.693364000
O	45.785397000	44.472633000	41.913442000
O	45.964059000	46.773485000	40.729170000
O	44.252718000	48.553862000	39.605483000
O	46.026530000	49.349607000	41.462081000
O	42.378449000	46.859327000	40.502281000
O	43.003531000	40.404492000	43.742961000
O	48.887582000	40.188992000	43.025825000
O	42.762886000	42.970147000	43.048528000
O	43.688499000	42.296659000	45.455919000
O	45.193909000	41.866263000	43.274544000
O	48.758326000	41.931300000	45.069240000
O	47.895817000	42.663770000	42.629518000
O	53.525650000	40.336245000	41.901751000
O	50.485649000	42.276863000	43.017278000
O	52.937509000	42.915813000	42.288446000
O	51.699379000	44.411435000	44.120799000
O	53.882915000	42.071996000	39.906595000
O	55.482363000	42.141661000	42.044576000
O	52.452758000	46.849721000	44.753364000
O	53.518270000	48.503186000	41.887843000
O	50.371951000	46.501062000	43.037739000
O	52.811847000	45.951878000	42.229193000
O	53.866947000	46.768073000	39.892442000
O	55.405247000	46.638928000	42.067393000
O	52.509687000	41.979542000	44.785855000
Ga	46.128902000	44.097101000	39.673032000
H	44.708563000	44.486974000	39.168571000
C	48.069736000	44.253383000	39.906949000
H	48.585477000	43.293612000	40.082768000
C	48.806507000	45.374590000	39.939956000
H	48.383393000	46.373755000	39.764663000
H	49.885668000	45.343973000	40.152241000
C	47.608661000	44.258879000	37.104316000
C	46.358259000	43.848859000	36.838282000
H	48.425163000	43.547485000	37.287093000
H	47.871268000	45.319310000	37.188465000
H	46.122719000	42.784218000	36.771178000
H	45.533417000	44.551193000	36.666783000

TS5-GaH$_2$

Electronic energy (r^{2}SCAN-D4/def2-SVP): -48334.47956703 E_h
Electronic energy (ωB97X-D4rev/def2-mTZVPP): -48378.377821299200 E_h
G_{corr} (673.15 K, 1 atm): 1.99870993 E_h
Number of atoms: 389

Cartesian coordinates (Å)

Si	48.555943000	36.028693000	35.301087000
Si	46.558804000	36.953438000	37.345926000
Si	44.104416000	36.092199000	35.630143000
Si	44.585130000	38.473984000	33.736747000
Si	47.747566000	38.541703000	33.556950000
Si	48.742023000	38.761077000	30.511875000
Si	51.812193000	38.115301000	30.334397000
Si	54.668965000	38.466738000	38.551461000
Si	57.772747000	38.487083000	38.806829000
Si	56.557692000	37.023369000	34.996926000
Si	52.601109000	36.080517000	32.656460000
Si	51.486659000	37.152858000	35.467566000
Si	54.126361000	36.090174000	36.711160000
Si	45.621787000	36.082765000	40.043479000
Si	41.742122000	38.008348000	41.951646000
Si	43.501038000	38.876602000	44.081432000
Si	47.726820000	38.461769000	46.625698000
Si	48.757626000	38.676346000	43.617851000
Si	46.040646000	38.272864000	42.297193000
Si	51.681999000	37.975082000	43.460623000
Si	53.638550000	38.786622000	41.361490000
Si	56.182181000	38.107882000	43.087124000
Si	44.620197000	50.323650000	33.727729000
Si	47.765327000	50.277681000	33.539680000
Si	48.750254000	50.063079000	30.512402000
Si	43.867346000	46.930706000	30.713590000
Si	48.877650000	46.969152000	30.410598000
Si	46.324321000	45.966857000	29.025410000
Si	48.558308000	52.784639000	35.315672000
Si	46.532117000	51.813026000	37.376691000
Si	44.107109000	52.724785000	35.640543000
Si	43.870645000	41.886797000	30.706950000
Si	48.885308000	41.852637000	30.386183000
Si	46.324616000	42.858278000	29.020726000
Si	51.888411000	42.866107000	30.523079000
Si	57.973175000	42.907629000	39.188207000
Si	58.733555000	47.859205000	36.908496000
Si	56.875644000	48.676349000	34.759530000
Si	55.870498000	48.297789000	31.972614000
Si	52.765360000	48.329657000	32.237177000
Si	56.557229000	51.799787000	34.995765000
Si	52.600655000	52.738747000	32.661249000
Si	51.491641000	51.662687000	35.481485000
Si	54.139173000	52.728006000	36.715506000
Si	51.806469000	50.702179000	30.329574000
Si	54.680770000	50.354982000	38.538666000
Si	57.777985000	50.331262000	38.803488000
Si	51.888009000	45.953831000	30.526524000
Si	57.971179000	45.929480000	39.182789000
Si	58.734049000	40.964806000	36.916644000

Si	56.886881000	40.147791000	34.761476000
Si	55.872558000	40.524178000	31.975157000
Si	52.767137000	40.491148000	32.243994000
Si	48.661663000	40.942320000	48.514417000
Si	41.739767000	50.806266000	41.944118000
Si	43.563240000	49.999779000	44.080173000
Si	47.727592000	50.359238000	46.624532000
Si	48.754327000	50.175921000	43.614489000
Si	46.051747000	50.730442000	42.354888000
Si	44.765845000	45.952233000	46.552836000
Si	47.917425000	45.964307000	46.183908000
Si	48.663170000	47.874360000	48.518760000
Si	45.613628000	52.763469000	40.048400000
Si	44.779858000	42.849773000	46.538977000
Si	47.936940000	42.843919000	46.144647000
Si	58.957147000	41.841136000	41.947886000
Si	56.319166000	42.833993000	43.277777000
Si	55.802059000	48.305483000	45.159957000
Si	52.646137000	48.379847000	45.315105000
Si	51.672433000	48.626785000	48.345478000
Si	51.705798000	50.846201000	43.450509000
Si	53.653640000	50.048643000	41.346008000
Si	56.184673000	50.712039000	43.087234000
Si	58.957303000	46.982213000	41.931873000
Si	56.303672000	45.966904000	43.265293000
Si	55.793985000	40.500228000	45.164727000
Si	52.647152000	40.434257000	45.321241000
Si	51.669801000	40.194892000	48.348921000
O	49.972962000	36.660605000	35.844649000
O	47.519355000	35.871841000	36.575825000
O	46.668891000	36.812189000	38.967539000
O	44.996271000	36.699084000	36.895819000
O	44.486184000	36.906796000	34.230189000
O	46.145902000	38.890404000	33.374368000
O	47.910163000	37.017315000	34.140754000
O	48.526551000	38.678197000	32.134759000
O	50.188002000	38.107001000	30.130531000
O	52.248040000	36.820090000	31.230640000
O	52.300165000	39.544678000	30.992929000
O	54.558219000	36.899638000	38.075373000
O	56.169249000	38.701246000	39.155322000
O	56.917263000	38.602209000	35.308048000
O	54.992618000	36.668330000	35.424664000
O	51.798313000	36.867745000	33.879285000
O	52.524750000	36.287976000	36.397671000
O	42.373822000	37.842254000	43.470381000
O	44.995857000	38.497940000	43.519557000
O	48.578437000	38.726147000	45.239421000
O	47.505594000	37.901075000	42.905990000
O	45.571463000	36.967763000	41.427171000
O	50.073979000	37.775164000	43.251079000
O	52.371813000	37.947615000	41.960803000
O	52.023514000	39.402556000	44.212312000
O	53.567052000	38.756996000	39.724271000
O	55.046257000	38.120332000	41.883647000
O	44.496116000	51.877198000	34.263244000
O	46.169546000	49.975056000	33.261751000
O	47.932736000	51.792612000	34.148191000

O	48.602727000	50.149286000	32.146599000
O	48.613067000	48.518335000	29.962897000
O	45.415029000	46.600595000	30.238299000
O	47.901636000	45.999635000	29.512306000
O	45.874309000	44.412665000	28.747224000
O	49.988932000	52.184720000	35.857364000
O	47.529260000	52.864747000	36.604993000
O	46.642377000	51.958683000	39.005727000
O	44.983576000	52.144801000	36.928705000
O	48.623014000	40.301135000	29.945477000
O	45.418747000	42.225359000	30.237050000
O	47.905743000	42.821643000	29.492767000
O	50.438456000	42.236028000	30.072372000
O	51.679534000	44.409305000	31.035775000
O	58.589832000	44.419496000	39.358478000
O	58.128406000	47.835636000	35.379589000
O	58.284879000	46.474646000	37.673166000
O	58.214066000	49.191021000	37.702285000
O	56.995803000	48.629487000	33.135305000
O	56.907253000	50.219907000	35.312115000
O	54.347378000	48.582679000	32.569755000
O	52.549363000	46.763158000	31.801290000
O	54.992245000	52.162858000	35.415750000
O	51.815543000	51.959318000	33.899072000
O	52.537707000	52.495843000	36.431121000
O	50.187283000	50.711914000	30.088604000
O	52.230073000	51.988461000	31.245120000
O	52.285983000	49.267659000	30.984221000
O	54.607946000	51.928018000	38.073883000
O	56.175238000	50.087206000	39.142016000
O	50.431975000	46.586480000	30.098947000
O	58.138914000	40.988614000	35.383266000
O	58.299142000	42.352197000	37.684529000
O	58.191787000	39.641092000	37.710742000
O	57.000864000	40.196878000	33.136408000
O	54.350306000	40.247018000	32.579812000
O	52.539750000	42.053832000	31.800480000
O	50.277080000	40.946676000	48.791456000
O	48.263302000	42.290167000	47.652915000
O	48.201826000	39.567189000	47.747948000
O	42.394825000	50.971239000	43.454791000
O	45.061269000	50.485711000	43.620532000
O	48.585695000	50.117306000	45.235002000
O	47.549878000	51.030438000	42.915267000
O	45.561227000	52.044534000	41.524994000
O	44.416842000	44.401758000	46.919207000
O	46.296520000	46.069788000	45.949030000
O	48.420137000	44.413055000	46.019537000
O	50.279228000	47.873985000	48.784877000
O	48.257184000	46.509348000	47.689047000
O	48.202787000	49.237350000	47.729552000
O	46.309633000	42.749065000	45.920296000
O	58.661711000	41.947266000	40.314886000
O	57.911332000	42.732277000	42.881131000
O	55.897247000	44.399821000	43.469291000
O	55.990894000	46.734747000	44.685664000
O	54.248514000	48.639385000	45.607033000
O	56.207283000	49.264658000	43.884807000

O	51.825807000	48.599487000	46.701255000
O	50.098618000	51.010948000	43.220809000
O	52.400027000	50.900108000	41.956346000
O	52.044102000	49.422496000	44.205085000
O	53.572430000	50.082213000	39.710618000
O	55.065992000	50.702071000	41.868110000
O	58.678990000	46.895751000	40.294322000
O	57.882811000	46.084241000	42.824501000
O	55.961464000	42.074549000	44.690844000
O	54.246886000	40.138315000	45.610291000
O	56.218426000	39.553739000	43.886706000
O	51.822728000	40.224188000	46.706006000
H	48.702871000	34.676568000	34.772566000
H	44.419875000	34.676323000	35.346829000
H	42.683628000	36.258975000	36.022159000
H	43.726523000	38.680216000	32.577941000
H	47.730594000	38.010996000	29.830569000
H	52.421136000	38.002380000	29.030380000
H	58.465353000	38.743363000	40.066967000
H	58.088382000	37.108509000	38.286195000
H	56.651061000	36.758841000	33.558678000
H	57.541582000	36.246995000	35.782467000
H	52.141234000	34.673922000	32.562045000
H	54.071037000	36.146097000	32.821438000
H	54.418082000	34.675478000	36.917895000
H	44.265382000	36.148734000	39.494231000
H	46.085045000	34.677316000	40.196440000
H	42.177304000	36.886192000	41.136491000
H	40.290195000	38.107003000	42.090377000
H	43.614692000	38.689573000	45.487247000
H	46.266760000	38.594605000	46.380864000
H	48.065374000	37.113072000	47.131273000
H	52.195251000	36.881484000	44.271734000
H	55.858296000	36.988842000	44.011824000
H	57.529558000	37.965437000	42.519250000
H	41.636484000	38.780154000	36.634495000
H	40.338249000	40.845235000	36.817795000
H	43.726296000	50.140518000	32.578071000
H	47.730502000	50.809921000	29.830551000
H	42.910974000	46.071594000	29.969678000
H	43.636013000	48.346500000	30.474316000
H	46.055518000	46.690813000	27.779371000
H	40.338196000	47.975592000	36.817799000
H	41.636403000	50.040687000	36.634467000
H	48.702714000	54.144377000	34.772532000
H	44.419585000	54.144606000	35.346948000
H	42.683575000	52.561616000	36.022276000
H	43.636069000	40.474361000	30.474327000
H	42.910998000	42.749252000	29.969681000
H	46.055551000	42.130076000	27.779377000
H	52.768240000	42.805898000	29.339147000
H	60.152183000	47.950749000	36.769108000
H	56.115708000	49.276599000	30.928772000
H	56.049418000	46.904457000	31.507081000
H	57.541465000	52.574079000	35.782446000
H	56.650952000	52.062214000	33.558656000
H	52.141090000	54.147065000	32.562019000
H	54.070917000	52.674925000	32.821418000

H	54.418140000	54.145525000	36.917811000
H	52.421044000	50.818602000	29.030363000
H	58.465269000	50.077736000	40.066952000
H	58.088278000	51.712582000	38.286179000
H	52.768217000	46.015092000	29.339142000
H	60.152234000	40.870368000	36.769119000
H	56.049458000	41.916585000	31.507090000
H	56.115777000	39.544444000	30.928783000
H	48.027948000	40.958435000	49.819979000
H	40.290115000	50.713683000	42.090582000
H	42.176850000	51.934815000	41.136521000
H	43.615015000	50.131729000	45.487215000
H	48.065229000	51.707918000	47.131232000
H	46.266685000	50.226321000	46.380825000
H	40.476352000	46.423809000	42.229530000
H	44.578030000	46.685732000	47.785515000
H	48.027871000	47.862537000	49.819962000
H	46.084739000	54.143668000	40.196331000
H	44.265252000	52.672179000	39.494179000
H	40.476382000	42.397038000	42.229542000
H	44.578055000	42.135174000	47.785513000
H	58.818796000	40.408567000	42.221796000
H	60.309668000	42.345550000	42.255160000
H	56.725235000	48.548904000	46.274187000
H	51.671681000	50.045758000	48.769745000
H	52.721673000	47.848745000	49.007593000
H	52.195144000	51.939534000	44.271718000
H	55.858180000	51.832229000	44.011806000
H	57.529465000	50.855664000	42.519236000
H	60.309639000	46.475584000	42.255156000
H	58.818736000	48.412544000	42.221786000
H	56.725177000	40.272281000	46.274313000
H	52.722235000	40.971966000	49.006966000
H	51.671815000	38.775269000	48.769821000
Si	48.799150000	40.997478000	35.375812000
Si	46.888573000	40.005112000	37.401230000
Si	41.568827000	40.147450000	37.199737000
Si	44.209182000	40.798130000	35.829733000
Si	44.638728000	45.914839000	33.462702000
Si	47.817621000	45.927771000	33.154247000
Si	48.768995000	47.807045000	35.390397000
Si	46.865957000	48.724269000	37.460375000
Si	41.577845000	48.671522000	37.179292000
Si	44.258157000	47.942263000	35.849337000
Si	44.650624000	42.854231000	33.465097000
Si	47.835282000	42.884196000	33.140965000
Si	54.816637000	42.899771000	38.842171000
Si	51.694649000	48.544946000	35.163413000
Si	54.400136000	47.949520000	36.447433000
Si	54.816084000	45.940237000	38.840660000
Si	51.715262000	40.268258000	35.174153000
Si	54.415642000	40.879964000	36.456055000
Si	45.764071000	40.589606000	40.133703000
Si	42.671348000	40.423110000	40.062117000
Si	41.769084000	45.932370000	41.719121000
Si	43.769990000	46.921162000	43.849136000
Si	48.896306000	47.061417000	43.451664000
Si	46.201354000	46.036948000	42.210416000

Si	45.759568000	48.327996000	40.237177000
Si	42.671914000	48.405796000	40.045349000
Si	41.765566000	42.904745000	41.748634000
Si	43.724803000	41.894989000	43.835454000
Si	48.963198000	41.789652000	43.389873000
Al	46.224714000	42.823799000	42.184435000
Si	51.889294000	42.883393000	43.560535000
Si	53.944019000	41.874194000	41.536944000
Si	51.824949000	45.936466000	43.546580000
Si	53.892662000	46.953153000	41.524326000
O	47.052952000	38.455793000	36.886991000
O	44.006230000	39.430107000	34.949559000
O	54.409369000	39.455491000	37.270452000
O	51.621677000	38.755890000	35.807647000
O	42.390416000	39.363085000	41.262634000
O	50.404706000	41.172262000	35.535144000
O	48.152332000	40.906236000	36.879236000
O	48.185042000	42.319525000	34.635301000
O	48.445897000	39.628164000	34.559684000
O	46.873947000	40.133239000	39.018320000
O	45.484590000	40.606996000	36.825509000
O	41.652804000	40.026413000	38.843506000
O	42.839782000	41.078044000	36.668023000
O	44.522298000	42.098759000	34.897395000
O	44.177397000	49.321649000	34.962073000
O	43.724632000	46.700212000	32.350800000
O	44.092563000	44.382533000	33.620367000
O	46.196245000	45.914748000	32.950944000
O	48.410747000	44.410056000	32.990375000
O	48.550633000	46.864187000	32.027056000
O	50.380452000	47.634227000	35.492007000
O	48.177868000	47.900293000	36.918537000
O	48.128716000	46.485110000	34.663532000
O	48.379488000	49.170015000	34.575273000
O	46.886625000	48.632257000	39.082990000
O	46.932459000	50.280321000	36.930238000
O	45.510168000	48.004328000	36.895987000
O	41.677540000	48.822725000	38.817792000
O	42.841467000	47.746484000	36.632409000
O	44.512236000	46.630417000	34.914555000
O	43.733085000	42.087045000	32.346084000
O	46.210981000	42.872561000	32.963945000
O	48.564678000	41.963104000	32.001247000
O	54.232884000	44.420021000	38.665006000
O	56.352462000	42.956593000	39.401363000
O	55.465306000	47.979768000	35.212695000
O	51.857837000	48.709678000	33.539978000
O	52.919117000	47.693923000	35.818826000
O	51.582445000	50.053002000	35.804389000
O	54.804460000	46.695304000	37.400471000
O	54.391663000	49.381199000	37.252476000
O	56.350050000	45.896060000	39.411210000
O	55.482573000	40.848429000	35.223767000
O	51.855648000	40.115823000	33.546316000
O	52.939789000	41.127047000	35.814669000
O	54.802955000	42.144076000	37.402840000
O	45.954151000	42.222714000	40.436014000
O	44.244411000	40.291707000	39.570612000

O	46.097249000	39.673142000	41.436311000
O	42.408596000	41.962048000	40.551759000
O	42.352090000	49.435475000	41.264677000
O	43.205856000	48.447264000	43.671531000
O	48.796718000	48.643611000	43.022325000
O	42.827281000	45.912494000	42.980062000
O	41.587465000	44.414587000	41.117162000
O	43.707296000	46.523640000	45.433185000
O	45.321696000	46.885532000	43.319563000
O	48.688421000	46.910171000	45.080532000
O	47.787255000	46.190567000	42.631255000
O	45.759024000	44.497634000	42.106959000
O	45.957913000	46.791383000	40.751327000
O	44.254271000	48.560730000	39.601649000
O	46.030554000	49.379430000	41.444252000
O	42.373531000	46.862318000	40.497406000
O	43.016414000	40.409393000	43.734515000
O	48.893523000	40.187522000	43.017649000
O	42.763335000	42.970628000	43.047557000
O	43.720352000	42.290831000	45.429025000
O	45.203531000	41.852249000	43.193032000
O	48.735537000	41.948890000	45.030219000
O	47.908641000	42.646064000	42.535020000
O	53.525869000	40.336271000	41.900709000
O	50.484115000	42.283620000	43.008415000
O	52.937153000	42.916159000	42.288814000
O	51.700987000	44.412003000	44.121322000
O	53.881930000	42.073474000	39.907079000
O	55.482389000	42.142144000	42.044180000
O	52.455167000	46.850648000	44.754864000
O	53.518690000	48.502830000	41.886809000
O	50.377319000	46.502898000	43.032706000
O	52.813348000	45.950940000	42.229936000
O	53.866063000	46.766322000	39.892981000
O	55.406649000	46.638862000	42.066517000
O	52.507169000	41.978893000	44.784630000
Ga	45.996676000	43.868108000	39.225878000
H	44.716352000	44.706690000	39.519699000
C	48.017740000	44.238084000	39.568460000
H	48.496733000	43.281104000	39.852667000
C	48.717359000	45.356579000	39.889371000
H	48.391605000	46.361941000	39.583597000
H	49.669009000	45.308584000	40.441033000
C	47.654271000	44.223317000	37.512882000
C	46.335312000	43.744092000	37.182432000
H	48.511830000	43.544059000	37.412603000
H	47.898413000	45.283153000	37.363634000
H	46.282226000	42.746447000	36.741039000
H	45.645228000	44.474759000	36.731419000

I9-GaH_2

Electronic energy (r^2SCAN-D4/def2-SVP): -48334.58440021 E_h
Electronic energy (ωB97X-D4rev/def2-mTZVPP): -48378.487333118000 E_h
G_{corr} (673.15 K, 1 atm): 2.00326240 E_h
Number of atoms: 389

Cartesian coordinates (Å)

Si	48.560339000	36.029837000	35.305385000
Si	46.527599000	36.966775000	37.340566000
Si	44.100165000	36.090995000	35.632112000
Si	44.624761000	38.499499000	33.725959000
Si	47.772246000	38.535277000	33.540058000
Si	48.747114000	38.762121000	30.512597000
Si	51.806051000	38.124609000	30.329184000
Si	54.677531000	38.461609000	38.537683000
Si	57.777268000	38.490036000	38.803317000
Si	56.558462000	37.023366000	34.996728000
Si	52.597861000	36.083929000	32.662525000
Si	51.485853000	37.161209000	35.484465000
Si	54.136619000	36.094036000	36.718707000
Si	45.623034000	36.072071000	40.025644000
Si	41.742646000	38.014777000	41.949042000
Si	43.612698000	38.843337000	44.071222000
Si	47.726507000	38.458875000	46.613571000
Si	48.757013000	38.670993000	43.585673000
Si	46.065211000	38.180062000	42.322534000
Si	51.722503000	37.999037000	43.464832000
Si	53.664612000	38.788456000	41.345578000
Si	56.184562000	38.106692000	43.085126000
Si	44.618890000	50.316031000	33.733700000
Si	47.766209000	50.280506000	33.556409000
Si	48.744336000	50.063351000	30.524351000
Si	43.867296000	46.934514000	30.716573000
Si	48.876087000	46.963023000	30.430709000
Si	46.325863000	45.965608000	29.027542000
Si	48.558755000	52.790369000	35.314594000
Si	46.531417000	51.843705000	37.356268000
Si	44.103072000	52.728589000	35.635871000
Si	43.867919000	41.887066000	30.714235000
Si	48.875469000	41.861171000	30.424722000
Si	46.325925000	42.857411000	29.025733000
Si	51.886802000	42.864790000	30.525868000
Si	57.969603000	42.907415000	39.191312000
Si	58.733441000	47.856554000	36.909682000
Si	56.874369000	48.676124000	34.758291000
Si	55.868430000	48.297791000	31.973394000
Si	52.763499000	48.324799000	32.244650000
Si	56.558098000	51.798655000	34.996282000
Si	52.599362000	52.737999000	32.659827000
Si	51.489446000	51.660076000	35.479894000
Si	54.136551000	52.728730000	36.716184000
Si	51.804676000	50.699404000	30.331346000
Si	54.679985000	50.360856000	38.531796000
Si	57.779045000	50.330731000	38.801784000
Si	51.886286000	45.955099000	30.525305000
Si	57.969831000	45.925129000	39.185835000
Si	58.734868000	40.966058000	36.915191000

Si	56.876620000	40.147309000	34.761693000
Si	55.872554000	40.523755000	31.974270000
Si	52.768468000	40.493901000	32.238969000
Si	48.668549000	40.941957000	48.517105000
Si	41.741991000	50.784769000	41.945628000
Si	43.711303000	50.013207000	44.069683000
Si	47.726828000	50.362478000	46.610488000
Si	48.768297000	50.165864000	43.578515000
Si	46.085663000	50.782498000	42.370562000
Si	44.762154000	45.953920000	46.549191000
Si	47.935786000	45.954792000	46.155386000
Si	48.676495000	47.888321000	48.515797000
Si	45.619066000	52.770548000	40.024590000
Si	44.763052000	42.856136000	46.544957000
Si	47.942135000	42.852406000	46.171715000
Si	58.956046000	41.841421000	41.954181000
Si	56.321611000	42.832043000	43.279330000
Si	55.796991000	48.296391000	45.169444000
Si	52.646307000	48.353256000	45.336002000
Si	51.672461000	48.628447000	48.353986000
Si	51.738924000	50.827138000	43.459313000
Si	53.668491000	50.032351000	41.338834000
Si	56.184095000	50.709536000	43.088437000
Si	58.958436000	46.981148000	41.935653000
Si	56.316524000	45.963917000	43.267358000
Si	55.795206000	40.497884000	45.171439000
Si	52.679277000	40.443226000	45.350189000
Si	51.670147000	40.194164000	48.352988000
O	49.981533000	36.639849000	35.861550000
O	47.515449000	35.906098000	36.576722000
O	46.664589000	36.830126000	38.964552000
O	44.968220000	36.647743000	36.932498000
O	44.496703000	36.945823000	34.262473000
O	46.176113000	38.849453000	33.268231000
O	47.930472000	37.015630000	34.137756000
O	48.605531000	38.656127000	32.144401000
O	50.188614000	38.130856000	30.076719000
O	52.218464000	36.836730000	31.249253000
O	52.295225000	39.555279000	30.984007000
O	54.602237000	36.885325000	38.083941000
O	56.173710000	38.734254000	39.135072000
O	56.918069000	38.602093000	35.309929000
O	54.994080000	36.664331000	35.423173000
O	51.812242000	36.857905000	33.903460000
O	52.535426000	36.330443000	36.433666000
O	42.425274000	37.885493000	43.449167000
O	45.108616000	38.316314000	43.633328000
O	48.547863000	38.685503000	45.203089000
O	47.577885000	37.839152000	42.813742000
O	45.557650000	36.910991000	41.436362000
O	50.116090000	37.844752000	43.229913000
O	52.412914000	37.946499000	41.969049000
O	52.079544000	39.421266000	44.218950000
O	53.574275000	38.749332000	39.710642000
O	55.074463000	38.121101000	41.859366000
O	44.496142000	51.872000000	34.266867000
O	46.171414000	49.960927000	33.283110000
O	47.917702000	51.801834000	34.154817000

O	48.596576000	50.165944000	32.157148000
O	48.596372000	48.513262000	29.994879000
O	45.411176000	46.593037000	30.239640000
O	47.900188000	45.998862000	29.525484000
O	45.880089000	44.411570000	28.740698000
O	49.985358000	52.180746000	35.856535000
O	47.529234000	52.903780000	36.601624000
O	46.651493000	51.973205000	38.986541000
O	44.977000000	52.175924000	36.935853000
O	48.591973000	40.312403000	29.985854000
O	45.412178000	42.227206000	30.237284000
O	47.901297000	42.827712000	29.520546000
O	50.432710000	42.225381000	30.099857000
O	51.672555000	44.410058000	31.030371000
O	58.592026000	44.416915000	39.364593000
O	58.120798000	47.829344000	35.383842000
O	58.289920000	46.472901000	37.678743000
O	58.219576000	49.190495000	37.703292000
O	56.997099000	48.628416000	33.134293000
O	56.914119000	50.219716000	35.311134000
O	54.346357000	48.582508000	32.573881000
O	52.555464000	46.760321000	31.798522000
O	54.993844000	52.159256000	35.420952000
O	51.813551000	51.958394000	33.897462000
O	52.535746000	52.495946000	36.428408000
O	50.185218000	50.699938000	30.091451000
O	52.225780000	51.990865000	31.242577000
O	52.286107000	49.270667000	30.996427000
O	54.601693000	51.937740000	38.082079000
O	56.174707000	50.086824000	39.131423000
O	50.431952000	46.595294000	30.101904000
O	58.119107000	40.997876000	35.390453000
O	58.305360000	42.349554000	37.691547000
O	58.216468000	39.632412000	37.705787000
O	57.000177000	40.197380000	33.137238000
O	54.350327000	40.239924000	32.573359000
O	52.553615000	42.060120000	31.800954000
O	50.279660000	40.939640000	48.811692000
O	48.282794000	42.307795000	47.678464000
O	48.219589000	39.585109000	47.710018000
O	42.465045000	50.877803000	43.430381000
O	45.170831000	50.687903000	43.718246000
O	48.534288000	50.108458000	45.191983000
O	47.624697000	51.049797000	42.814019000
O	45.565890000	52.047023000	41.496411000
O	44.330322000	44.409080000	46.856691000
O	46.308324000	46.029576000	45.999084000
O	48.458270000	44.407692000	46.014035000
O	50.282203000	47.894527000	48.831212000
O	48.326968000	46.542401000	47.630868000
O	48.227398000	49.250746000	47.716910000
O	46.311743000	42.797202000	45.982269000
O	58.648574000	41.948668000	40.324817000
O	57.918044000	42.727350000	42.902074000
O	55.900250000	44.398889000	43.468265000
O	55.991979000	46.730049000	44.686110000
O	54.245911000	48.599308000	45.648598000
O	56.179279000	49.268466000	43.897640000

O	51.799043000	48.586218000	46.706560000
O	50.132859000	50.977607000	43.234413000
O	52.423812000	50.886373000	41.963629000
O	52.084637000	49.406881000	44.215589000
O	53.576588000	50.071053000	39.705132000
O	55.080864000	50.689709000	41.856218000
O	58.669545000	46.893231000	40.301155000
O	57.899705000	46.078403000	42.842885000
O	55.944549000	42.070323000	44.687100000
O	54.266427000	40.123623000	45.668319000
O	56.199005000	39.551043000	43.887993000
O	51.808604000	40.230850000	46.707770000
H	48.702871000	34.676568000	34.772566000
H	44.419875000	34.676323000	35.346829000
H	42.683628000	36.258975000	36.022159000
H	43.726523000	38.680216000	32.577941000
H	47.730594000	38.010996000	29.830569000
H	52.421136000	38.002380000	29.030381000
H	58.465353000	38.743363000	40.066967000
H	58.088382000	37.108509000	38.286195000
H	56.651061000	36.758841000	33.558678000
H	57.541582000	36.246995000	35.782467000
H	52.141234000	34.673922000	32.562045000
H	54.071037000	36.146097000	32.821438000
H	54.418082000	34.675478000	36.917895000
H	44.265382000	36.148734000	39.494230000
H	46.085045000	34.677316000	40.196440000
H	42.177304000	36.886192000	41.136491000
H	40.290195000	38.107003000	42.090377000
H	43.614692000	38.689573000	45.487247000
H	46.266760000	38.594605000	46.380864000
H	48.065374000	37.113072000	47.131273000
H	52.195251000	36.881483000	44.271734000
H	55.858296000	36.988842000	44.011824000
H	57.529558000	37.965437000	42.519250000
H	41.636484000	38.780154000	36.634495000
H	40.338249000	40.845235000	36.817795000
H	43.726296000	50.140518000	32.578071000
H	47.730503000	50.809921000	29.830551000
H	42.910974000	46.071594000	29.969678000
H	43.636013000	48.346500000	30.474316000
H	46.055518000	46.690812000	27.779371000
H	40.338195000	47.975592000	36.817799000
H	41.636403000	50.040687000	36.634467000
H	48.702714000	54.144377000	34.772532000
H	44.419585000	54.144606000	35.346948000
H	42.683575000	52.561616000	36.022276000
H	43.636069000	40.474361000	30.474327000
H	42.910998000	42.749253000	29.969681000
H	46.055551000	42.130076000	27.779377000
H	52.768240000	42.805898000	29.339147000
H	60.152183000	47.950749000	36.769108000
H	56.115708000	49.276599000	30.928772000
H	56.049418000	46.904457000	31.507081000
H	57.541465000	52.574079000	35.782446000
H	56.650952000	52.062214000	33.558656000
H	52.141090000	54.147065000	32.562019000
H	54.070917000	52.674925000	32.821418000

H	54.418140000	54.145525000	36.917811000
H	52.421044000	50.818602000	29.030363000
H	58.465269000	50.077736000	40.066952000
H	58.088278000	51.712582000	38.286179000
H	52.768217000	46.015092000	29.339142000
H	60.152234000	40.870368000	36.769119000
H	56.049458000	41.916585000	31.507090000
H	56.115777000	39.544444000	30.928783000
H	48.027948000	40.958435000	49.819978000
H	40.290115000	50.713683000	42.090582000
H	42.176850000	51.934815000	41.136521000
H	43.615015000	50.131729000	45.487215000
H	48.065229000	51.707918000	47.131232000
H	46.266685000	50.226321000	46.380825000
H	40.476352000	46.423809000	42.229530000
H	44.578030000	46.685732000	47.785515000
H	48.027871000	47.862537000	49.819962000
H	46.084739000	54.143668000	40.196331000
H	44.265252000	52.672179000	39.494179000
H	40.476382000	42.397038000	42.229542000
H	44.578055000	42.135174000	47.785513000
H	58.818796000	40.408567000	42.221796000
H	60.309668000	42.345550000	42.255160000
H	56.725235000	48.548904000	46.274187000
H	51.671681000	50.045758000	48.769745000
H	52.721673000	47.848745000	49.007593000
H	52.195144000	51.939534000	44.271718000
H	55.858180000	51.832229000	44.011806000
H	57.529465000	50.855664000	42.519236000
H	60.309639000	46.475584000	42.255156000
H	58.818736000	48.412544000	42.221786000
H	56.725177000	40.272281000	46.274313000
H	52.722235000	40.971966000	49.006966000
H	51.671815000	38.775269000	48.769821000
Si	48.770326000	40.992742000	35.405387000
Si	46.866902000	40.019474000	37.429254000
Si	41.578823000	40.156930000	37.178120000
Si	44.268724000	40.876738000	35.851196000
Si	44.642994000	45.941985000	33.465257000
Si	47.820067000	45.935939000	33.194607000
Si	48.760927000	47.827307000	35.432324000
Si	46.857163000	48.788525000	37.468931000
Si	41.575261000	48.668799000	37.173207000
Si	44.266662000	47.946166000	35.866628000
Si	44.640485000	42.889967000	33.458926000
Si	47.816044000	42.891392000	33.186139000
Si	54.814401000	42.898034000	38.824600000
Si	51.686433000	48.536860000	35.174978000
Si	54.397815000	47.963777000	36.444955000
Si	54.818460000	45.933413000	38.828924000
Si	51.688703000	40.280308000	35.162295000
Si	54.394869000	40.858213000	36.445864000
Si	45.743289000	40.481676000	40.157140000
Si	42.654743000	40.425607000	40.051102000
Si	41.764471000	45.944388000	41.709113000
Si	43.904907000	46.857804000	43.783141000
Si	48.907645000	47.045239000	43.356516000
Si	46.313626000	45.986049000	41.945619000

Si	45.714649000	48.450759000	40.223369000
Si	42.632270000	48.401868000	40.039217000
Si	41.773722000	42.894817000	41.760075000
Si	43.824324000	41.943885000	43.827550000
Si	48.996437000	41.779403000	43.430496000
Al	46.391991000	42.800612000	42.090574000
Si	51.930074000	42.859166000	43.596856000
Si	53.958636000	41.881141000	41.531889000
Si	51.837598000	45.919503000	43.551357000
Si	53.917347000	46.936808000	41.528079000
O	46.916892000	38.485775000	36.838431000
O	44.177405000	39.501659000	34.959250000
O	54.384414000	39.421181000	37.241654000
O	51.573146000	38.771528000	35.802400000
O	42.321800000	39.385222000	41.248945000
O	50.378970000	41.197708000	35.489304000
O	48.188604000	40.843824000	36.931467000
O	48.088463000	42.296032000	34.687045000
O	48.410619000	39.631463000	34.569712000
O	46.849790000	39.984038000	39.057951000
O	45.518751000	40.793198000	36.905790000
O	41.641847000	40.060606000	38.823601000
O	42.853370000	41.071860000	36.639635000
O	44.525941000	42.192641000	34.923059000
O	44.167118000	49.320601000	34.971143000
O	43.735838000	46.747545000	32.360855000
O	44.068112000	44.415950000	33.574661000
O	46.201680000	45.927615000	32.958683000
O	48.411269000	44.412911000	33.080078000
O	48.570439000	46.838295000	32.049338000
O	50.372500000	47.634303000	35.526267000
O	48.170773000	47.958602000	36.957767000
O	48.098537000	46.523460000	34.696993000
O	48.405735000	49.188526000	34.591395000
O	46.835603000	48.842729000	39.099643000
O	46.921180000	50.322171000	36.870508000
O	45.503350000	48.038396000	36.932382000
O	41.633104000	48.804968000	38.815910000
O	42.848281000	47.745267000	36.650889000
O	44.528114000	46.636928000	34.930360000
O	43.732884000	42.082325000	32.357087000
O	46.198061000	42.904861000	32.948813000
O	48.566974000	41.987583000	32.042732000
O	54.246247000	44.417103000	38.602868000
O	56.348065000	42.958803000	39.390760000
O	55.457798000	47.988543000	35.205657000
O	51.851140000	48.687517000	33.549607000
O	52.914911000	47.690049000	35.830774000
O	51.584160000	50.051678000	35.803047000
O	54.821507000	46.729210000	37.412599000
O	54.386216000	49.404733000	37.234295000
O	56.349118000	45.882880000	39.409294000
O	55.460296000	40.832625000	35.211311000
O	51.857684000	40.113438000	33.539065000
O	52.916837000	41.125070000	35.818357000
O	54.804992000	42.101069000	37.409249000
O	45.985407000	42.090891000	40.469257000
O	44.224488000	40.238054000	39.569509000

O	45.991019000	39.618136000	41.518898000
O	42.426876000	41.963873000	40.562766000
O	42.235416000	49.360795000	41.288353000
O	43.684800000	48.458427000	43.517703000
O	48.779942000	48.635788000	42.976084000
O	42.776637000	45.980191000	43.004767000
O	41.606099000	44.411853000	41.143890000
O	43.785873000	46.599452000	45.392184000
O	45.381058000	46.382410000	43.232483000
O	48.616402000	46.860017000	44.965900000
O	47.864076000	46.184265000	42.424955000
O	46.056700000	44.441044000	41.417458000
O	45.902974000	46.870874000	40.623633000
O	44.203014000	48.676828000	39.610484000
O	45.947249000	49.365477000	41.552928000
O	42.409671000	46.836809000	40.475674000
O	43.338077000	40.391645000	43.591134000
O	48.773608000	40.203174000	43.013446000
O	42.766970000	42.946214000	43.066375000
O	43.731837000	42.220053000	45.443329000
O	45.318243000	42.195707000	43.279352000
O	48.709817000	41.927256000	45.058973000
O	48.043541000	42.746005000	42.558490000
O	53.550228000	40.337642000	41.881520000
O	50.558312000	42.141505000	43.102995000
O	52.960622000	42.908185000	42.316958000
O	51.661100000	44.392458000	44.110131000
O	53.874288000	42.104419000	39.909164000
O	55.500530000	42.145696000	42.032393000
O	52.435995000	46.822677000	44.785801000
O	53.529861000	48.486551000	41.877971000
O	50.413950000	46.521130000	43.008945000
O	52.871022000	45.930084000	42.275358000
O	53.865513000	46.728542000	39.902247000
O	55.441529000	46.647301000	42.058225000
O	52.589278000	42.001227000	44.835043000
Ga	45.665281000	43.884401000	39.459060000
H	44.110399000	44.034621000	39.303881000
C	49.197319000	44.756360000	39.569390000
H	48.935143000	44.503905000	40.610167000
C	50.019739000	45.792800000	39.347190000
H	50.329771000	46.075701000	38.330250000
H	50.419689000	46.393076000	40.174320000
C	48.605467000	43.887197000	38.490740000
C	47.125042000	44.195537000	38.163500000
H	48.712604000	42.822104000	38.778401000
H	49.197831000	44.014316000	37.560976000
H	46.814953000	43.599670000	37.276427000
H	47.028899000	45.254278000	37.842503000

I10-GaH_2

Electronic energy (r^2SCAN-D4/def2-SVP): -48413.03644146 E_h
Electronic energy (ωB97X-D4rev/def2-mTZVPP): -48457.146419081600 E_h
G_{corr} (673.15 K, 1 atm): 2.04288789 E_h
Number of atoms: 395

Cartesian coordinates (Å)

Si	48.550539000	36.033444000	35.301484000
Si	46.523199000	36.978508000	37.342934000
Si	44.093966000	36.094829000	35.623778000
Si	44.612851000	38.471366000	33.723190000
Si	47.761789000	38.528644000	33.530698000
Si	48.746902000	38.766731000	30.502172000
Si	51.808620000	38.124030000	30.329460000
Si	54.669504000	38.469635000	38.540802000
Si	57.770401000	38.484031000	38.808283000
Si	56.551029000	37.018105000	34.999203000
Si	52.594624000	36.083315000	32.663250000
Si	51.479230000	37.159630000	35.481344000
Si	54.125562000	36.091495000	36.716247000
Si	45.613036000	36.081193000	40.034040000
Si	41.735738000	38.021655000	41.941674000
Si	43.532029000	38.858653000	44.074378000
Si	47.717714000	38.467034000	46.622840000
Si	48.742337000	38.677013000	43.612810000
Si	46.046542000	38.209923000	42.318920000
Si	51.699062000	37.991985000	43.461364000
Si	53.652482000	38.778116000	41.351915000
Si	56.177670000	38.101917000	43.087076000
Si	44.613230000	50.362224000	33.732667000
Si	47.763722000	50.275358000	33.558203000
Si	48.749021000	50.056997000	30.520348000
Si	43.876952000	46.937940000	30.692967000
Si	48.884649000	46.964042000	30.406341000
Si	46.334406000	45.968988000	29.018890000
Si	48.568084000	52.788066000	35.305081000
Si	46.544936000	51.828215000	37.355378000
Si	44.113732000	52.737293000	35.622157000
Si	43.871487000	41.897210000	30.696231000
Si	48.880466000	41.861152000	30.396406000
Si	46.329463000	42.862527000	29.018491000
Si	51.889263000	42.864682000	30.525634000
Si	57.959184000	42.899879000	39.190416000
Si	58.738683000	47.853986000	36.911625000
Si	56.886687000	48.670344000	34.760901000
Si	55.876414000	48.293815000	31.975816000
Si	52.773263000	48.325918000	32.239954000
Si	56.565370000	51.794689000	34.997639000
Si	52.611157000	52.738079000	32.656182000
Si	51.499814000	51.663261000	35.472264000
Si	54.141766000	52.727307000	36.713063000
Si	51.818200000	50.698674000	30.331501000
Si	54.682034000	50.347535000	38.534611000
Si	57.782945000	50.327403000	38.804643000
Si	51.892296000	45.954253000	30.524249000
Si	57.965143000	45.922520000	39.184423000
Si	58.733154000	40.956621000	36.918163000

Si	56.878552000	40.143225000	34.765531000
Si	55.874129000	40.521530000	31.977253000
Si	52.770362000	40.489502000	32.233893000
Si	48.649083000	40.955564000	48.517486000
Si	41.744672000	50.807251000	41.938860000
Si	43.653768000	50.031627000	44.068781000
Si	47.727934000	50.368424000	46.609562000
Si	48.772335000	50.169062000	43.582262000
Si	46.074155000	50.744583000	42.348151000
Si	44.741439000	45.956857000	46.554342000
Si	47.911346000	45.957875000	46.167403000
Si	48.667861000	47.889601000	48.513302000
Si	45.624264000	52.772405000	40.026039000
Si	44.747446000	42.865830000	46.543727000
Si	47.917236000	42.856831000	46.191105000
Si	58.950611000	41.836664000	41.955876000
Si	56.312422000	42.829326000	43.284349000
Si	55.798746000	48.300758000	45.167052000
Si	52.645215000	48.365699000	45.325330000
Si	51.670598000	48.630922000	48.349100000
Si	51.728287000	50.837533000	43.454204000
Si	53.666922000	50.034233000	41.345196000
Si	56.188426000	50.708632000	43.089056000
Si	58.957514000	46.975722000	41.937783000
Si	56.311560000	45.961608000	43.273058000
Si	55.792968000	40.489469000	45.168844000
Si	52.677387000	40.428854000	45.340492000
Si	51.660695000	40.198700000	48.347551000
O	49.969923000	36.651088000	35.854449000
O	47.507480000	35.916475000	36.574778000
O	46.651189000	36.836054000	38.966920000
O	44.966227000	36.673521000	36.912567000
O	44.489272000	36.907539000	34.226037000
O	46.162584000	38.851044000	33.284707000
O	47.920069000	37.006961000	34.122450000
O	48.581994000	38.666455000	32.130561000
O	50.190136000	38.128353000	30.086221000
O	52.223050000	36.837221000	31.248825000
O	52.306400000	39.557630000	30.970844000
O	54.585216000	36.897586000	38.074203000
O	56.167310000	38.726012000	39.142741000
O	56.904745000	38.597806000	35.314148000
O	54.985477000	36.657565000	35.420401000
O	51.803638000	36.857994000	33.899764000
O	52.523401000	36.316671000	36.426349000
O	42.380196000	37.859049000	43.456373000
O	45.032027000	38.420420000	43.572525000
O	48.576807000	38.694538000	45.235345000
O	47.528473000	37.850912000	42.891910000
O	45.559291000	36.919048000	41.446865000
O	50.090834000	37.842917000	43.223473000
O	52.394827000	37.938219000	41.967125000
O	52.055858000	39.415384000	44.214009000
O	53.566804000	38.747636000	39.716801000
O	55.060824000	38.108862000	41.867341000
O	44.498959000	51.933653000	34.216670000
O	46.162523000	49.965452000	33.309475000
O	47.930636000	51.802309000	34.139759000

O	48.576223000	50.134183000	32.151924000
O	48.609290000	48.514254000	29.966330000
O	45.428229000	46.614965000	30.226379000
O	47.912810000	45.995297000	29.502262000
O	45.874607000	44.416413000	28.747108000
O	49.993321000	52.176257000	35.846971000
O	47.539198000	52.886556000	36.593474000
O	46.653302000	51.977383000	38.984383000
O	44.995509000	52.144045000	36.901477000
O	48.603454000	40.311839000	29.956289000
O	45.422495000	42.218947000	30.227161000
O	47.909458000	42.833092000	29.495774000
O	50.438025000	42.229911000	30.084235000
O	51.675959000	44.410036000	31.030999000
O	58.579093000	44.410991000	39.359573000
O	58.143398000	47.833949000	35.379140000
O	58.285425000	46.471528000	37.677412000
O	58.219654000	49.186130000	37.705209000
O	57.004403000	48.625228000	33.136609000
O	56.912791000	50.214225000	35.313664000
O	54.355326000	48.573861000	32.581573000
O	52.555087000	46.760906000	31.800155000
O	55.001071000	52.158871000	35.418881000
O	51.819701000	51.956253000	33.888350000
O	52.540493000	52.510469000	36.416366000
O	50.195493000	50.698865000	30.115296000
O	52.245196000	51.991958000	31.236911000
O	52.313031000	49.268745000	30.983453000
O	54.594130000	51.920707000	38.073532000
O	56.178675000	50.089133000	39.137270000
O	50.441894000	46.595215000	30.089454000
O	58.132059000	40.982117000	35.387801000
O	58.295001000	42.339576000	37.691247000
O	58.209370000	39.625206000	37.709020000
O	56.999299000	40.190970000	33.140735000
O	54.351431000	40.243632000	32.576589000
O	52.543845000	42.057076000	31.805312000
O	50.267637000	40.961277000	48.773939000
O	48.215967000	42.328876000	47.712572000
O	48.190171000	39.595219000	47.724475000
O	42.439946000	50.940762000	43.434528000
O	45.138442000	50.617545000	43.677035000
O	48.544773000	50.120305000	45.196210000
O	47.603949000	51.019616000	42.818283000
O	45.566747000	52.025732000	41.487112000
O	44.326331000	44.414236000	46.884525000
O	46.283905000	46.026513000	45.988148000
O	48.435745000	44.410695000	46.021349000
O	50.278097000	47.888017000	48.808192000
O	48.286295000	46.538436000	47.649700000
O	48.222115000	49.252763000	47.714143000
O	46.289152000	42.807598000	45.949360000
O	58.642520000	41.946018000	40.326189000
O	57.906871000	42.721045000	42.899105000
O	55.898513000	44.396986000	43.482225000
O	55.987705000	46.731869000	44.689681000
O	54.245586000	48.621490000	45.628186000
O	56.195495000	49.265201000	43.893924000

O	51.809079000	48.591360000	46.703165000
O	50.122224000	51.004905000	43.228236000
O	52.417740000	50.889794000	41.959805000
O	52.059662000	49.414089000	44.211839000
O	53.580352000	50.063343000	39.711079000
O	55.078275000	50.693457000	41.862906000
O	58.669613000	46.884391000	40.302622000
O	57.893512000	46.077017000	42.843278000
O	55.939095000	42.064175000	44.690974000
O	54.263877000	40.099206000	45.651124000
O	56.206621000	39.550326000	43.882823000
O	51.818204000	40.223755000	46.704862000
H	48.695747000	34.679249000	34.772421000
H	44.412792000	34.683097000	35.344379000
H	42.677520000	36.267230000	36.018695000
H	43.724204000	38.687834000	32.575415000
H	47.729277000	38.014429000	29.829684000
H	52.420228000	38.002341000	29.031641000
H	58.459950000	38.738213000	40.071104000
H	58.082353000	37.103569000	38.290328000
H	56.645862000	36.755040000	33.561295000
H	57.535839000	36.241702000	35.784986000
H	52.136656000	34.674112000	32.563224000
H	54.066960000	36.145388000	32.823726000
H	54.411023000	34.673579000	36.919916000
H	44.257013000	36.156713000	39.492058000
H	46.074363000	34.682945000	40.195291000
H	42.168975000	36.895703000	41.133695000
H	40.282690000	38.118632000	42.086545000
H	43.606327000	38.698291000	45.484765000
H	46.257361000	38.602471000	46.379197000
H	48.053417000	37.118702000	47.131262000
H	52.185719000	36.882272000	44.273696000
H	55.850274000	36.986709000	44.015265000
H	57.522260000	37.962277000	42.522827000
H	41.632406000	38.789357000	36.631235000
H	40.336010000	40.855677000	36.813565000
H	43.734771000	50.146633000	32.573749000
H	47.740645000	50.812971000	29.828351000
H	42.917096000	46.079054000	29.965627000
H	43.643595000	48.353332000	30.470847000
H	46.063047000	46.695724000	27.776681000
H	40.342569000	47.986032000	36.812373000
H	41.642793000	50.049881000	36.629338000
H	48.713812000	54.146873000	34.769657000
H	44.431612000	54.151336000	35.341227000
H	42.692939000	52.571167000	36.016274000
H	43.636743000	40.481516000	30.471035000
H	42.913559000	42.757064000	29.966639000
H	46.059110000	42.134724000	27.777607000
H	52.771631000	42.804102000	29.340921000
H	60.156401000	47.943471000	36.773081000
H	56.124219000	49.272923000	30.930707000
H	56.055304000	46.900788000	31.508793000
H	57.550363000	52.568948000	35.784417000
H	56.660555000	52.057466000	33.560233000
H	52.152246000	54.146553000	32.561528000
H	54.081220000	52.673388000	32.821584000

H	54.427665000	54.143334000	36.918124000
H	52.431656000	50.817528000	29.030107000
H	58.470225000	50.073465000	40.069255000
H	58.094819000	51.707819000	38.287666000
H	52.774271000	46.013620000	29.339226000
H	60.150097000	40.863485000	36.773961000
H	56.049978000	41.914085000	31.510768000
H	56.114991000	39.542142000	30.931531000
H	48.019447000	40.964400000	49.819204000
H	40.294504000	50.725275000	42.084408000
H	42.182955000	51.944267000	41.130948000
H	43.617310000	50.140908000	45.482708000
H	48.066943000	51.714044000	47.127888000
H	46.267846000	50.233562000	46.376507000
H	40.476649000	46.435022000	42.224432000
H	44.575783000	46.694228000	47.782348000
H	48.025333000	47.869557000	49.817664000
H	46.092511000	54.149649000	40.192075000
H	44.272355000	52.679307000	39.489229000
H	40.472938000	42.408253000	42.225117000
H	44.571932000	42.143704000	47.783005000
H	58.814175000	40.403609000	42.226252000
H	60.306341000	42.339580000	42.259999000
H	56.725290000	48.546775000	46.276215000
H	51.670764000	50.050164000	48.767484000
H	52.719430000	47.852824000	49.008132000
H	52.199464000	51.940363000	44.270800000
H	55.862409000	51.830266000	44.012585000
H	57.533541000	50.851882000	42.520990000
H	60.309883000	46.470031000	42.259383000
H	58.820023000	48.407776000	42.224730000
H	56.718010000	40.269413000	46.277658000
H	52.714007000	40.974027000	49.008250000
H	51.661914000	38.778074000	48.771701000
Si	48.781475000	40.984624000	35.371287000
Si	46.875155000	40.044353000	37.417210000
Si	41.579380000	40.163368000	37.176924000
Si	44.257688000	40.824209000	35.828013000
Si	44.643290000	45.950391000	33.448314000
Si	47.822535000	45.933584000	33.164666000
Si	48.773914000	47.818677000	35.417926000
Si	46.869910000	48.749153000	37.470184000
Si	41.576240000	48.683556000	37.171476000
Si	44.255559000	48.025825000	35.841146000
Si	44.637592000	42.900750000	33.444576000
Si	47.813860000	42.892533000	33.150889000
Si	54.798483000	42.893139000	38.835037000
Si	51.699294000	48.546655000	35.160400000
Si	54.404401000	47.941972000	36.442544000
Si	54.807715000	45.933906000	38.841927000
Si	51.697055000	40.270536000	35.148765000
Si	54.394234000	40.876555000	36.444863000
Si	45.791958000	40.557198000	40.173111000
Si	42.685983000	40.437161000	40.053578000
Si	41.766926000	45.946802000	41.709137000
Si	43.832890000	46.902084000	43.831231000
Si	48.910144000	47.046657000	43.391373000
Si	46.252442000	46.013209000	42.110631000

Si	45.713610000	48.367538000	40.230153000
Si	42.638537000	48.411516000	40.034309000
Si	41.771257000	42.905962000	41.754404000
Si	43.746461000	41.905750000	43.856036000
Si	48.981627000	41.781239000	43.495884000
Al	46.331918000	42.773334000	42.245847000
Si	51.925373000	42.855895000	43.600516000
Si	53.942784000	41.867878000	41.533874000
Si	51.839560000	45.915045000	43.554513000
Si	53.909254000	46.943322000	41.533371000
O	46.931814000	38.497746000	36.864849000
O	44.142049000	39.416423000	34.994045000
O	54.386801000	39.449559000	37.258228000
O	51.584040000	38.768135000	35.804784000
O	42.377796000	39.370502000	41.241283000
O	50.387561000	41.191860000	35.456714000
O	48.191655000	40.854406000	36.891000000
O	48.101284000	42.283291000	34.642563000
O	48.420305000	39.613680000	34.556487000
O	46.880209000	40.093863000	39.041333000
O	45.515694000	40.780896000	36.871378000
O	41.684331000	40.054231000	38.819347000
O	42.849711000	41.075667000	36.617496000
O	44.516243000	42.088412000	34.841410000
O	44.134641000	49.445488000	35.023436000
O	43.720114000	46.695137000	32.319007000
O	44.079623000	44.425910000	33.641090000
O	46.202842000	45.919308000	32.942150000
O	48.418349000	44.411293000	33.047140000
O	48.566323000	46.842161000	32.021468000
O	50.385402000	47.647141000	35.509176000
O	48.172267000	47.911921000	36.941814000
O	48.122906000	46.522828000	34.660905000
O	48.401764000	49.192074000	34.602577000
O	46.850537000	48.698009000	39.101532000
O	46.960450000	50.298584000	36.923609000
O	45.503378000	48.053651000	36.896333000
O	41.640187000	48.825635000	38.813442000
O	42.853866000	47.767538000	36.642391000
O	44.510144000	46.789430000	34.824913000
O	43.713768000	42.143581000	32.324036000
O	46.194287000	42.922608000	32.928556000
O	48.556842000	41.981767000	32.010426000
O	54.220533000	44.416074000	38.666484000
O	56.337308000	42.949100000	39.389123000
O	55.484036000	47.964445000	35.220774000
O	51.853482000	48.707548000	33.534324000
O	52.931238000	47.691937000	35.796972000
O	51.608190000	50.056938000	35.801254000
O	54.788571000	46.686338000	37.401686000
O	54.397757000	49.373102000	37.248784000
O	56.343736000	45.889722000	39.405696000
O	55.475148000	40.847190000	35.223821000
O	51.860036000	40.086166000	33.526427000
O	52.925073000	41.123050000	35.791325000
O	54.778263000	42.137914000	37.396957000
O	46.053440000	42.145519000	40.559745000
O	44.266406000	40.319782000	39.591948000

O	46.062096000	39.616970000	41.474565000
O	42.403308000	41.969311000	40.554152000
O	42.288470000	49.405507000	41.270742000
O	43.474901000	48.473130000	43.569165000
O	48.820840000	48.635373000	42.993800000
O	42.817769000	45.955747000	42.978406000
O	41.596495000	44.420544000	41.133198000
O	43.715678000	46.586996000	45.429106000
O	45.378704000	46.615249000	43.351651000
O	48.616539000	46.877772000	45.002818000
O	47.833591000	46.203159000	42.483495000
O	45.898306000	44.446861000	41.772520000
O	45.856175000	46.799922000	40.708618000
O	44.216035000	48.615191000	39.590429000
O	45.957439000	49.335708000	41.517623000
O	42.378995000	46.855064000	40.473938000
O	43.114324000	40.399851000	43.673811000
O	48.766492000	40.212197000	43.045598000
O	42.786609000	42.966631000	43.045022000
O	43.684176000	42.260995000	45.456669000
O	45.264436000	41.961014000	43.315815000
O	48.728263000	41.900860000	45.134305000
O	47.990336000	42.769258000	42.698001000
O	53.544819000	40.325188000	41.897420000
O	50.530431000	42.166422000	43.133450000
O	52.923187000	42.894951000	42.292475000
O	51.697951000	44.390358000	44.128139000
O	53.871489000	42.067993000	39.906890000
O	55.478334000	42.154386000	42.040177000
O	52.445070000	46.834836000	44.772468000
O	53.530785000	48.492041000	41.897542000
O	50.396772000	46.481775000	43.029586000
O	52.850623000	45.932233000	42.258649000
O	53.866977000	46.757999000	39.903812000
O	55.429673000	46.636408000	42.064134000
O	52.601275000	41.986088000	44.820237000
Ga	45.893379000	44.039721000	39.675276000
H	44.352984000	44.134757000	39.445658000
C	50.073919000	44.187049000	38.360542000
H	50.634911000	44.404256000	39.286436000
C	50.641774000	44.457893000	37.175818000
H	50.117149000	44.259071000	36.229354000
H	51.650901000	44.882386000	37.101510000
C	48.690375000	43.617386000	38.539368000
C	47.760148000	44.596141000	39.283354000
H	48.755368000	42.662778000	39.102126000
H	48.277899000	43.366129000	37.546522000
H	48.213882000	44.831696000	40.268620000
H	47.718425000	45.559515000	38.736838000
C	44.766759000	44.529911000	36.882366000
C	46.035791000	44.393270000	36.475742000
H	44.042875000	43.705281000	36.855036000
H	44.377153000	45.479882000	37.269106000
H	46.421544000	43.432032000	36.114705000
H	46.744961000	45.230816000	36.502642000

TS6-GaH$_2$

Electronic energy (r^{2}SCAN-D4/def2-SVP): -48412.96676132 E_h
Electronic energy (ωB97X-D4rev/def2-mTZVPP): -48457.069172788000 E_h
G_{corr} (673.15 K, 1 atm): 2.04320116 E_h
Number of atoms: 395

Cartesian coordinates (Å)

Si	48.567069000	36.031433000	35.304030000
Si	46.550296000	36.986048000	37.346679000
Si	44.104556000	36.091216000	35.618891000
Si	44.613101000	38.422025000	33.705807000
Si	47.765689000	38.544834000	33.531893000
Si	48.750036000	38.764863000	30.500705000
Si	51.809288000	38.120798000	30.329704000
Si	54.675426000	38.468925000	38.551487000
Si	57.774939000	38.489723000	38.805707000
Si	56.558632000	37.023680000	34.996850000
Si	52.599347000	36.082734000	32.665627000
Si	51.487766000	37.157944000	35.488082000
Si	54.140528000	36.096551000	36.722670000
Si	45.620682000	36.084284000	40.043909000
Si	41.739971000	38.009386000	41.948062000
Si	43.509420000	38.874995000	44.080353000
Si	47.726106000	38.459345000	46.622027000
Si	48.773785000	38.674946000	43.609513000
Si	46.037288000	38.291316000	42.285983000
Si	51.680816000	37.968862000	43.458516000
Si	53.637799000	38.787412000	41.360249000
Si	56.181659000	38.107755000	43.087072000
Si	44.619664000	50.326724000	33.729300000
Si	47.766609000	50.267179000	33.544361000
Si	48.750702000	50.063969000	30.513490000
Si	43.866738000	46.927732000	30.716340000
Si	48.877114000	46.974121000	30.411459000
Si	46.324608000	45.968589000	29.027185000
Si	48.559974000	52.781927000	35.315134000
Si	46.532863000	51.803550000	37.379940000
Si	44.108293000	52.724222000	35.641402000
Si	43.869993000	41.887525000	30.688939000
Si	48.886861000	41.854211000	30.390638000
Si	46.329275000	42.858994000	29.018659000
Si	51.889976000	42.865784000	30.524877000
Si	57.966263000	42.903121000	39.189953000
Si	58.733585000	47.855122000	36.912336000
Si	56.872064000	48.674677000	34.761442000
Si	55.871259000	48.297867000	31.973057000
Si	52.765601000	48.329584000	32.239123000
Si	56.559445000	51.798067000	34.995877000
Si	52.601188000	52.738581000	32.662187000
Si	51.492511000	51.659347000	35.482111000
Si	54.141898000	52.726631000	36.717486000
Si	51.806201000	50.703733000	30.330904000
Si	54.680973000	50.358517000	38.539843000
Si	57.777995000	50.330441000	38.803528000
Si	51.889221000	45.953875000	30.527880000
Si	57.975382000	45.922845000	39.188985000
Si	58.734418000	40.964950000	36.915684000

Si	56.875567000	40.149165000	34.766064000
Si	55.873545000	40.524209000	31.974177000
Si	52.768106000	40.488620000	32.233336000
Si	48.659000000	40.938211000	48.507016000
Si	41.739329000	50.806867000	41.944752000
Si	43.571532000	50.013134000	44.080105000
Si	47.727224000	50.361245000	46.623758000
Si	48.762278000	50.190963000	43.613686000
Si	46.052887000	50.739306000	42.357116000
Si	44.743753000	45.945261000	46.555797000
Si	47.907127000	45.960718000	46.179340000
Si	48.661139000	47.874699000	48.515809000
Si	45.613306000	52.765131000	40.048149000
Si	44.747872000	42.865756000	46.548793000
Si	47.912214000	42.853557000	46.122818000
Si	58.954908000	41.841369000	41.951127000
Si	56.318575000	42.836803000	43.279774000
Si	55.800293000	48.316349000	45.160526000
Si	52.656742000	48.396387000	45.319004000
Si	51.671776000	48.626839000	48.345213000
Si	51.705700000	50.849896000	43.449631000
Si	53.656430000	50.051543000	41.346474000
Si	56.185327000	50.714505000	43.085866000
Si	58.956414000	46.981172000	41.942317000
Si	56.307782000	45.971339000	43.275953000
Si	55.791220000	40.499878000	45.164399000
Si	52.641079000	40.428464000	45.319223000
Si	51.667988000	40.195244000	48.348812000
O	49.988443000	36.627297000	35.870174000
O	47.512604000	35.914021000	36.566788000
O	46.660578000	36.815100000	38.965730000
O	44.995024000	36.730004000	36.871277000
O	44.487314000	36.853815000	34.187129000
O	46.159046000	38.822549000	33.281544000
O	47.956018000	37.033502000	34.138301000
O	48.584938000	38.686479000	32.132057000
O	50.189512000	38.115026000	30.090984000
O	52.227623000	36.837989000	31.251944000
O	52.297247000	39.557266000	30.972169000
O	54.602027000	36.895479000	38.083932000
O	56.173200000	38.729821000	39.151067000
O	56.913575000	38.602786000	35.310886000
O	54.994624000	36.663615000	35.423696000
O	51.811403000	36.855361000	33.906089000
O	52.539592000	36.333186000	36.437179000
O	42.383134000	37.845131000	43.461105000
O	45.006847000	38.496292000	43.525322000
O	48.561007000	38.730361000	45.226810000
O	47.514952000	37.938332000	42.868308000
O	45.569109000	36.982717000	41.420302000
O	50.073067000	37.741608000	43.264501000
O	52.365751000	37.952431000	41.957031000
O	52.014158000	39.398120000	44.210556000
O	53.569388000	38.748076000	39.723116000
O	55.042882000	38.117434000	41.886000000
O	44.494681000	51.881519000	34.259775000
O	46.167968000	49.972849000	33.266713000
O	47.940059000	51.783257000	34.149902000

O	48.599668000	50.138833000	32.148005000
O	48.618956000	48.521636000	29.954819000
O	45.415175000	46.603772000	30.239493000
O	47.901704000	46.002812000	29.513782000
O	45.874078000	44.413294000	28.752669000
O	49.993685000	52.191921000	35.858020000
O	47.529347000	52.852901000	36.603708000
O	46.641956000	51.959207000	39.008260000
O	44.985269000	52.137212000	36.926010000
O	48.613711000	40.306473000	29.942342000
O	45.419903000	42.209695000	30.222371000
O	47.907286000	42.827932000	29.501451000
O	50.439991000	42.233819000	30.075054000
O	51.680541000	44.409084000	31.035730000
O	58.589417000	44.411740000	39.367309000
O	58.115542000	47.824886000	35.388396000
O	58.299706000	46.469340000	37.682370000
O	58.214358000	49.187770000	37.704930000
O	56.996631000	48.625625000	33.137162000
O	56.914934000	50.219112000	35.311506000
O	54.347758000	48.586507000	32.566795000
O	52.554417000	46.763116000	31.800064000
O	54.995088000	52.159701000	35.418543000
O	51.818147000	51.956432000	33.899540000
O	52.541796000	52.486695000	36.433129000
O	50.186225000	50.718985000	30.093568000
O	52.233177000	51.990329000	31.244241000
O	52.280180000	49.268789000	30.988793000
O	54.612759000	51.933273000	38.079226000
O	56.175241000	50.085439000	39.141393000
O	50.431051000	46.585839000	30.106752000
O	58.124646000	40.994261000	35.388221000
O	58.303053000	42.349778000	37.688176000
O	58.206229000	39.634269000	37.706125000
O	56.995730000	40.198512000	33.141014000
O	54.349710000	40.242675000	32.569984000
O	52.539535000	42.055508000	31.804856000
O	50.274625000	40.951066000	48.786773000
O	48.257084000	42.265305000	47.614542000
O	48.208822000	39.553280000	47.751873000
O	42.397263000	50.976333000	43.453562000
O	45.068616000	50.509493000	43.631540000
O	48.583206000	50.126973000	45.232783000
O	47.555055000	51.034383000	42.906001000
O	45.558791000	52.051222000	41.526782000
O	44.346826000	44.407304000	46.930970000
O	46.280663000	46.022095000	45.965006000
O	48.430765000	44.414155000	46.013207000
O	50.278097000	47.870558000	48.779301000
O	48.248544000	46.514081000	47.681679000
O	48.204102000	49.240209000	47.729131000
O	46.278851000	42.806944000	45.933618000
O	58.648376000	41.942504000	40.320315000
O	57.912389000	42.731896000	42.889307000
O	55.900331000	44.403615000	43.475413000
O	55.969485000	46.741822000	44.689632000
O	54.254723000	48.681659000	45.613180000
O	56.213131000	49.264984000	43.879959000

O	51.826793000	48.605168000	46.701307000
O	50.100314000	51.038505000	43.227451000
O	52.397701000	50.896498000	41.954850000
O	52.035285000	49.423444000	44.204408000
O	53.573407000	50.083457000	39.711571000
O	55.065665000	50.712694000	41.867690000
O	58.672828000	46.887810000	40.306865000
O	57.892457000	46.089239000	42.854308000
O	55.954325000	42.074530000	44.690459000
O	54.242208000	40.136077000	45.605337000
O	56.218191000	39.553295000	43.887232000
O	51.818705000	40.216482000	46.705590000
H	48.702871000	34.676568000	34.772566000
H	44.419875000	34.676323000	35.346829000
H	42.683628000	36.258975000	36.022159000
H	43.726523000	38.680216000	32.577941000
H	47.730594000	38.010996000	29.830569000
H	52.421136000	38.002380000	29.030380000
H	58.465353000	38.743363000	40.066967000
H	58.088382000	37.108509000	38.286195000
H	56.651061000	36.758841000	33.558678000
H	57.541582000	36.246995000	35.782467000
H	52.141234000	34.673922000	32.562045000
H	54.071037000	36.146097000	32.821438000
H	54.418082000	34.675478000	36.917895000
H	44.265382000	36.148734000	39.494230000
H	46.085045000	34.677316000	40.196440000
H	42.177304000	36.886192000	41.136491000
H	40.290195000	38.107003000	42.090377000
H	43.614691000	38.689573000	45.487247000
H	46.266760000	38.594605000	46.380864000
H	48.065374000	37.113072000	47.131273000
H	52.195251000	36.881484000	44.271734000
H	55.858296000	36.988842000	44.011824000
H	57.529558000	37.965437000	42.519250000
H	41.636484000	38.780154000	36.634495000
H	40.338249000	40.845235000	36.817795000
H	43.726296000	50.140518000	32.578071000
H	47.730502000	50.809922000	29.830551000
H	42.910974000	46.071594000	29.969678000
H	43.636013000	48.346500000	30.474316000
H	46.055518000	46.690813000	27.779371000
H	40.338196000	47.975592000	36.817799000
H	41.636403000	50.040687000	36.634467000
H	48.702714000	54.144377000	34.772532000
H	44.419585000	54.144606000	35.346948000
H	42.683575000	52.561616000	36.022276000
H	43.636069000	40.474361000	30.474327000
H	42.910998000	42.749253000	29.969681000
H	46.055551000	42.130076000	27.779377000
H	52.768240000	42.805898000	29.339147000
H	60.152183000	47.950749000	36.769108000
H	56.115708000	49.276599000	30.928772000
H	56.049418000	46.904457000	31.507081000
H	57.541465000	52.574079000	35.782446000
H	56.650952000	52.062214000	33.558656000
H	52.141090000	54.147065000	32.562019000
H	54.070917000	52.674925000	32.821418000

H	54.418140000	54.145525000	36.917811000
H	52.421044000	50.818602000	29.030363000
H	58.465269000	50.077736000	40.066952000
H	58.088278000	51.712582000	38.286179000
H	52.768217000	46.015092000	29.339142000
H	60.152234000	40.870368000	36.769119000
H	56.049458000	41.916585000	31.507090000
H	56.115777000	39.544444000	30.928783000
H	48.027948000	40.958435000	49.819979000
H	40.290115000	50.713683000	42.090582000
H	42.176850000	51.934815000	41.136521000
H	43.615015000	50.131729000	45.487215000
H	48.065229000	51.707918000	47.131232000
H	46.266685000	50.226321000	46.380825000
H	40.476352000	46.423809000	42.229530000
H	44.578030000	46.685732000	47.785515000
H	48.027871000	47.862537000	49.819962000
H	46.084739000	54.143668000	40.196331000
H	44.265252000	52.672179000	39.494179000
H	40.476382000	42.397038000	42.229542000
H	44.578055000	42.135174000	47.785513000
H	58.818796000	40.408567000	42.221796000
H	60.309668000	42.345550000	42.255160000
H	56.725235000	48.548904000	46.274187000
H	51.671681000	50.045758000	48.769745000
H	52.721673000	47.848745000	49.007593000
H	52.195144000	51.939533000	44.271718000
H	55.858180000	51.832229000	44.011806000
H	57.529465000	50.855664000	42.519236000
H	60.309639000	46.475584000	42.255156000
H	58.818736000	48.412544000	42.221786000
H	56.725177000	40.272281000	46.274313000
H	52.722235000	40.971966000	49.006966000
H	51.671815000	38.775269000	48.769821000
Si	48.771471000	41.031930000	35.359602000
Si	46.897468000	40.068967000	37.428787000
Si	41.573370000	40.149488000	37.172796000
Si	44.254272000	40.750760000	35.793211000
Si	44.638069000	45.908092000	33.473602000
Si	47.815830000	45.933552000	33.150770000
Si	48.761479000	47.793188000	35.401542000
Si	46.866499000	48.707952000	37.480240000
Si	41.576555000	48.674922000	37.178196000
Si	44.258546000	47.951295000	35.854444000
Si	44.658017000	42.853791000	33.432693000
Si	47.827067000	42.900143000	33.124004000
Si	54.804405000	42.900789000	38.834686000
Si	51.687752000	48.538492000	35.167847000
Si	54.395563000	47.957416000	36.449151000
Si	54.822314000	45.942533000	38.836657000
Si	51.690435000	40.272264000	35.151199000
Si	54.391752000	40.871201000	36.452840000
Si	45.707692000	40.629244000	40.140294000
Si	42.652068000	40.426338000	40.047270000
Si	41.771817000	45.941729000	41.715621000
Si	43.793856000	46.938260000	43.851709000
Si	48.926525000	47.079228000	43.449534000
Si	46.234525000	46.062173000	42.270790000

Si	45.749645000	48.316039000	40.259209000
Si	42.659656000	48.409474000	40.042050000
Si	41.769858000	42.904134000	41.754211000
Si	43.726868000	41.891134000	43.860057000
Si	48.965889000	41.787482000	43.339672000
Al	46.213727000	42.816040000	42.231431000
Si	51.896055000	42.900100000	43.570068000
Si	53.945476000	41.878357000	41.537481000
Si	51.862574000	45.946252000	43.565782000
Si	53.915153000	46.958774000	41.528869000
O	47.033638000	38.507274000	36.948185000
O	44.112851000	39.336123000	34.986769000
O	54.388137000	39.442852000	37.263986000
O	51.560656000	38.769985000	35.802740000
O	42.363712000	39.370564000	41.246678000
O	50.381829000	41.200586000	35.451947000
O	48.175470000	40.928803000	36.881710000
O	48.120258000	42.351258000	34.639320000
O	48.373323000	39.667201000	34.550890000
O	46.868635000	40.272354000	39.042904000
O	45.504733000	40.687430000	36.837886000
O	41.639962000	40.062113000	38.818813000
O	42.851519000	41.034446000	36.584997000
O	44.570873000	42.004682000	34.808792000
O	44.173721000	49.331711000	34.970536000
O	43.718070000	46.671858000	32.350283000
O	44.105851000	44.373373000	33.675386000
O	46.192573000	45.912642000	32.954201000
O	48.416570000	44.419805000	32.969056000
O	48.539891000	46.880617000	32.027117000
O	50.374644000	47.624300000	35.489803000
O	48.170773000	47.888147000	36.925659000
O	48.121338000	46.473871000	34.666471000
O	48.372182000	49.155731000	34.579863000
O	46.873934000	48.604447000	39.101771000
O	46.930016000	50.266138000	36.951993000
O	45.504551000	47.998704000	36.907289000
O	41.676826000	48.848527000	38.813593000
O	42.840680000	47.744489000	36.637849000
O	44.510780000	46.642187000	34.915756000
O	43.710331000	42.127635000	32.316566000
O	46.207332000	42.904520000	32.900119000
O	48.573460000	41.960733000	32.009554000
O	54.236643000	44.425708000	38.651159000
O	56.344355000	42.951278000	39.386220000
O	55.452503000	47.991642000	35.206978000
O	51.860221000	48.701740000	33.544997000
O	52.911363000	47.692022000	35.832158000
O	51.570813000	50.048845000	35.802845000
O	54.815910000	46.709180000	37.402602000
O	54.387055000	49.391964000	37.249327000
O	56.354394000	45.888524000	39.407850000
O	55.464881000	40.840070000	35.224287000
O	51.861656000	40.088545000	33.530087000
O	52.916715000	41.116099000	35.807494000
O	54.777111000	42.134116000	37.401083000
O	45.741378000	42.269795000	40.496948000
O	44.217145000	40.272388000	39.533569000

O	46.050905000	39.700080000	41.430381000
O	42.416873000	41.970610000	40.545450000
O	42.344353000	49.430146000	41.269541000
O	43.225123000	48.459767000	43.663607000
O	48.830234000	48.661175000	43.024149000
O	42.878724000	45.939386000	42.936500000
O	41.578624000	44.415442000	41.133995000
O	43.692791000	46.524936000	45.430020000
O	45.367947000	46.916123000	43.385371000
O	48.652741000	46.918639000	45.070114000
O	47.837431000	46.219471000	42.592403000
O	45.829294000	44.508141000	42.249636000
O	45.914253000	46.787810000	40.811330000
O	44.245989000	48.540404000	39.611712000
O	46.012893000	49.384905000	41.452825000
O	42.323748000	46.864465000	40.465683000
O	43.023699000	40.406206000	43.723803000
O	48.977491000	40.176419000	43.009454000
O	42.788682000	42.964528000	43.037154000
O	43.682096000	42.285275000	45.451912000
O	45.231023000	41.851323000	43.280031000
O	48.653235000	41.968980000	44.962884000
O	47.912032000	42.556685000	42.398874000
O	53.530514000	40.337646000	41.898033000
O	50.473061000	42.347789000	43.014765000
O	52.945014000	42.916591000	42.301371000
O	51.742416000	44.424751000	44.148232000
O	53.877204000	42.081121000	39.910055000
O	55.485791000	42.147812000	42.041757000
O	52.504283000	46.860552000	44.768165000
O	53.536411000	48.506698000	41.893414000
O	50.413959000	46.519926000	43.076555000
O	52.845526000	45.953662000	42.244823000
O	53.882908000	46.771401000	39.897576000
O	55.433005000	46.644745000	42.060979000
O	52.489375000	41.972257000	44.787954000
Ga	45.188858000	43.881432000	39.425813000
H	44.302249000	44.825545000	40.259663000
C	49.755033000	44.398663000	39.399065000
H	49.762754000	44.076340000	40.454075000
C	50.729905000	45.198891000	38.947252000
H	50.757730000	45.549126000	37.904496000
H	51.537184000	45.531762000	39.608997000
C	48.585909000	43.910818000	38.581226000
C	47.292512000	44.585201000	39.055885000
H	48.499311000	42.810893000	38.687254000
H	48.764170000	44.123151000	37.507539000
H	47.275880000	44.463716000	40.163286000
H	47.308508000	45.673755000	38.885694000
C	44.647540000	43.770698000	37.497973000
C	46.063708000	44.033322000	37.330153000
H	44.262424000	42.785960000	37.238069000
H	43.948158000	44.594633000	37.290223000
H	46.721740000	43.190462000	37.078780000
H	46.399581000	44.995520000	36.918281000

I11-GaH$_2$

Electronic energy (r^{2}SCAN-D4/def2-SVP): -48413.07724251 E_h
Electronic energy (ωB97X-D4rev/def2-mTZVPP): -48457.184845394600 E_h
G_{corr} (673.15 K, 1 atm): 2.05282910 E_h
Number of atoms: 395

Cartesian coordinates (Å)

Si	48.560277000	36.026329000	35.315690000
Si	46.526431000	36.965773000	37.353349000
Si	44.101104000	36.090152000	35.635777000
Si	44.624023000	38.505126000	33.729385000
Si	47.768715000	38.521091000	33.543958000
Si	48.744511000	38.759134000	30.520106000
Si	51.803363000	38.126187000	30.330419000
Si	54.682478000	38.436313000	38.531927000
Si	57.778864000	38.492128000	38.801873000
Si	56.560776000	37.023870000	34.996843000
Si	52.597533000	36.084605000	32.662818000
Si	51.489955000	37.163874000	35.488097000
Si	54.141032000	36.091497000	36.720628000
Si	45.619101000	36.067120000	40.031673000
Si	41.741398000	38.014788000	41.949410000
Si	43.620321000	38.829380000	44.072067000
Si	47.726264000	38.457828000	46.613435000
Si	48.751526000	38.663028000	43.584404000
Si	46.064776000	38.148111000	42.335967000
Si	51.732354000	38.004744000	43.463161000
Si	53.660956000	38.795867000	41.330631000
Si	56.183706000	38.111386000	43.084768000
Si	44.617308000	50.311698000	33.736472000
Si	47.761861000	50.302640000	33.555558000
Si	48.743657000	50.065210000	30.526329000
Si	43.865297000	46.935243000	30.715677000
Si	48.873573000	46.961394000	30.431709000
Si	46.324285000	45.965360000	29.027475000
Si	48.556306000	52.793937000	35.314497000
Si	46.527616000	51.848825000	37.361105000
Si	44.102370000	52.730351000	35.638500000
Si	43.867855000	41.886275000	30.714240000
Si	48.873238000	41.869098000	30.437117000
Si	46.326096000	42.857260000	29.026478000
Si	51.885981000	42.864089000	30.525407000
Si	57.999828000	42.906351000	39.196488000
Si	58.732632000	47.849169000	36.910220000
Si	56.873089000	48.679061000	34.755637000
Si	55.874815000	48.297261000	31.971544000
Si	52.770092000	48.323618000	32.244505000
Si	56.560785000	51.797740000	34.997027000
Si	52.599556000	52.737934000	32.659645000
Si	51.490378000	51.659726000	35.480280000
Si	54.136583000	52.731490000	36.719186000
Si	51.804153000	50.698870000	30.332016000
Si	54.679912000	50.386066000	38.528111000
Si	57.778980000	50.328722000	38.800474000
Si	51.885618000	45.955476000	30.525423000
Si	58.002696000	45.924535000	39.190229000
Si	58.733578000	40.972518000	36.915269000

Si	56.871124000	40.145120000	34.760310000
Si	55.876973000	40.524173000	31.972766000
Si	52.773218000	40.497770000	32.244276000
Si	48.662567000	40.945074000	48.516464000
Si	41.741546000	50.786346000	41.946956000
Si	43.712202000	50.018961000	44.069311000
Si	47.726211000	50.363024000	46.611452000
Si	48.767971000	50.163874000	43.580019000
Si	46.085681000	50.784870000	42.371153000
Si	44.741964000	45.946028000	46.550766000
Si	47.922131000	45.940070000	46.149344000
Si	48.673276000	47.887335000	48.511564000
Si	45.618069000	52.771528000	40.027267000
Si	44.729998000	42.863804000	46.548480000
Si	47.913150000	42.854869000	46.192592000
Si	58.961828000	41.837857000	41.946380000
Si	56.316192000	42.833932000	43.274749000
Si	55.803232000	48.290077000	45.170047000
Si	52.649555000	48.347820000	45.337130000
Si	51.671523000	48.628116000	48.352883000
Si	51.744142000	50.823283000	43.459347000
Si	53.661990000	50.019011000	41.328605000
Si	56.182387000	50.705099000	43.088370000
Si	58.965766000	46.984319000	41.929166000
Si	56.320651000	45.964640000	43.263964000
Si	55.803031000	40.503273000	45.171689000
Si	52.691030000	40.444404000	45.354642000
Si	51.668564000	40.194856000	48.350562000
O	49.985534000	36.638395000	35.858719000
O	47.527772000	35.910352000	36.598303000
O	46.659620000	36.839076000	38.979390000
O	44.971532000	36.632092000	36.940530000
O	44.497153000	36.953136000	34.271461000
O	46.176532000	38.849580000	33.268107000
O	47.913868000	37.004486000	34.150862000
O	48.608699000	38.626777000	32.150798000
O	50.187129000	38.140493000	30.068908000
O	52.210137000	36.835375000	31.250020000
O	52.285770000	39.552319000	30.999889000
O	54.621370000	36.854810000	38.097416000
O	56.173470000	38.735355000	39.128694000
O	56.931600000	38.600625000	35.308726000
O	54.996525000	36.668692000	35.426834000
O	51.820052000	36.860217000	33.907872000
O	52.540338000	36.339878000	36.442891000
O	42.432516000	37.876043000	43.445005000
O	45.118190000	38.289846000	43.653716000
O	48.550085000	38.668965000	45.202198000
O	47.584403000	37.813635000	42.812123000
O	45.548929000	36.877269000	41.458280000
O	50.125561000	37.865653000	43.224149000
O	52.420761000	37.946590000	41.966667000
O	52.108368000	39.426201000	44.210921000
O	53.573372000	38.733569000	39.695938000
O	55.074855000	38.147638000	41.858259000
O	44.495980000	51.865726000	34.275230000
O	46.171210000	49.961408000	33.285231000
O	47.900626000	51.829194000	34.142510000

O	48.594394000	50.175066000	32.158710000
O	48.593366000	48.513382000	30.003189000
O	45.409655000	46.591508000	30.240653000
O	47.899764000	45.999458000	29.522137000
O	45.879987000	44.411129000	28.739797000
O	49.982398000	52.176472000	35.848453000
O	47.533520000	52.901456000	36.607523000
O	46.651602000	51.972319000	38.991579000
O	44.974956000	52.191617000	36.944522000
O	48.577953000	40.316620000	30.020450000
O	45.412340000	42.228903000	30.239102000
O	47.901585000	42.828227000	29.521983000
O	50.432560000	42.219038000	30.103424000
O	51.667605000	44.409713000	31.027285000
O	58.620362000	44.415494000	39.378076000
O	58.102296000	47.814873000	35.391814000
O	58.309956000	46.456182000	37.675583000
O	58.215725000	49.178720000	37.709986000
O	57.004693000	48.625356000	33.132311000
O	56.935469000	50.222019000	35.307826000
O	54.352674000	48.584796000	32.564590000
O	52.558020000	46.759769000	31.797365000
O	54.996923000	52.149828000	35.430761000
O	51.816578000	51.958055000	33.898290000
O	52.535895000	52.495148000	36.431151000
O	50.184732000	50.699153000	30.090434000
O	52.224950000	51.991291000	31.242269000
O	52.280726000	49.270713000	31.001543000
O	54.604741000	51.968833000	38.101157000
O	56.172012000	50.092714000	39.125018000
O	50.430817000	46.597610000	30.105425000
O	58.098619000	41.010436000	35.398559000
O	58.317250000	42.362910000	37.688138000
O	58.217843000	39.640187000	37.710119000
O	57.004460000	40.199833000	33.136723000
O	54.355143000	40.237515000	32.566810000
O	52.558436000	42.061649000	31.798849000
O	50.277917000	40.950836000	48.793055000
O	48.246967000	42.317248000	47.703248000
O	48.220512000	39.588474000	47.705065000
O	42.466058000	50.884275000	43.430860000
O	45.173192000	50.692103000	43.720789000
O	48.533386000	50.104071000	45.193474000
O	47.626146000	51.048843000	42.813579000
O	45.566813000	52.052978000	41.501498000
O	44.263454000	44.411316000	46.845996000
O	46.292730000	45.981068000	46.012140000
O	48.464815000	44.397380000	46.016297000
O	50.280748000	47.889102000	48.821905000
O	48.318553000	46.550296000	47.615005000
O	48.226600000	49.256241000	47.722532000
O	46.283704000	42.839002000	45.998357000
O	58.690740000	41.934013000	40.310149000
O	57.904913000	42.728067000	42.867767000
O	55.899941000	44.401388000	43.466579000
O	56.017892000	46.725833000	44.689602000
O	54.250483000	48.579848000	45.649430000
O	56.181126000	49.266396000	43.901098000

O	51.801866000	48.593127000	46.705022000
O	50.136787000	50.970348000	43.240857000
O	52.424203000	50.878014000	41.961146000
O	52.103735000	49.403467000	44.210829000
O	53.575427000	50.076134000	39.694796000
O	55.076239000	50.663221000	41.858955000
O	58.711129000	46.903878000	40.289065000
O	57.897142000	46.074037000	42.816134000
O	55.964978000	42.075096000	44.690520000
O	54.277231000	40.130691000	45.676055000
O	56.201700000	39.555199000	43.888202000
O	51.813468000	40.219095000	46.704889000
H	48.702883000	34.676571000	34.772567000
H	44.419887000	34.676325000	35.346831000
H	42.683641000	36.258978000	36.022160000
H	43.726534000	38.680215000	32.577941000
H	47.730608000	38.010999000	29.830569000
H	52.421149000	38.002382000	29.030381000
H	58.465366000	38.743366000	40.066966000
H	58.088395000	37.108512000	38.286194000
H	56.651074000	36.758843000	33.558678000
H	57.541596000	36.246998000	35.782468000
H	52.141247000	34.673924000	32.562045000
H	54.071050000	36.146100000	32.821439000
H	54.418095000	34.675481000	36.917896000
H	44.265396000	36.148737000	39.494233000
H	46.085059000	34.677319000	40.196442000
H	42.177317000	36.886193000	41.136493000
H	40.290205000	38.106997000	42.090382000
H	43.614704000	38.689578000	45.487248000
H	46.266775000	38.594611000	46.380866000
H	48.065388000	37.113078000	47.131274000
H	52.195264000	36.881486000	44.271735000
H	55.858310000	36.988846000	44.011824000
H	57.529572000	37.965441000	42.519250000
H	41.636486000	38.780154000	36.634495000
H	40.338249000	40.845235000	36.817795000
H	43.726308000	50.140524000	32.578070000
H	47.730517000	50.809923000	29.830550000
H	42.910987000	46.071600000	29.969673000
H	43.636027000	48.346504000	30.474318000
H	46.055529000	46.690815000	27.779372000
H	40.338196000	47.975593000	36.817798000
H	41.636406000	50.040687000	36.634467000
H	48.702728000	54.144379000	34.772530000
H	44.419601000	54.144610000	35.346944000
H	42.683590000	52.561622000	36.022274000
H	43.636082000	40.474362000	30.474326000
H	42.911010000	42.749253000	29.969678000
H	46.055562000	42.130077000	27.779378000
H	52.768253000	42.805898000	29.339146000
H	60.152195000	47.950749000	36.769104000
H	56.115720000	49.276602000	30.928771000
H	56.049430000	46.904459000	31.507079000
H	57.541481000	52.574079000	35.782443000
H	56.650968000	52.062215000	33.558652000
H	52.141104000	54.147067000	32.562017000
H	54.070932000	52.674927000	32.821416000

H	54.418155000	54.145527000	36.917808000
H	52.421059000	50.818603000	29.030360000
H	58.465283000	50.077739000	40.066949000
H	58.088293000	51.712584000	38.286175000
H	52.768229000	46.015094000	29.339141000
H	60.152246000	40.870372000	36.769116000
H	56.049469000	41.916586000	31.507089000
H	56.115789000	39.544444000	30.928784000
H	48.027964000	40.958441000	49.819979000
H	40.290128000	50.713699000	42.090584000
H	42.176869000	51.934816000	41.136516000
H	43.615028000	50.131734000	45.487213000
H	48.065246000	51.707924000	47.131229000
H	46.266701000	50.226328000	46.380824000
H	40.476353000	46.423810000	42.229530000
H	44.578040000	46.685737000	47.785514000
H	48.027888000	47.862544000	49.819960000
H	46.084756000	54.143672000	40.196331000
H	44.265268000	52.672185000	39.494178000
H	40.476383000	42.397039000	42.229543000
H	44.578065000	42.135179000	47.785512000
H	58.818811000	40.408570000	42.221794000
H	60.309681000	42.345557000	42.255157000
H	56.725250000	48.548907000	46.274185000
H	51.671698000	50.045764000	48.769743000
H	52.721690000	47.848750000	49.007591000
H	52.195158000	51.939538000	44.271716000
H	55.858196000	51.832234000	44.011802000
H	57.529481000	50.855667000	42.519232000
H	60.309652000	46.475585000	42.255152000
H	58.818751000	48.412548000	42.221782000
H	56.725191000	40.272286000	46.274313000
H	52.722251000	40.971973000	49.006964000
H	51.671831000	38.775275000	48.769823000
Si	48.764485000	40.974856000	35.408243000
Si	46.859321000	40.023616000	37.442115000
Si	41.576502000	40.155006000	37.181495000
Si	44.267960000	40.885652000	35.857160000
Si	44.623217000	45.940469000	33.462980000
Si	47.797633000	45.953483000	33.205363000
Si	48.755126000	47.867058000	35.434382000
Si	46.851153000	48.791085000	37.469247000
Si	41.572769000	48.671431000	37.177679000
Si	44.263932000	47.938094000	35.868782000
Si	44.630021000	42.890763000	33.459817000
Si	47.807214000	42.901834000	33.208145000
Si	54.865260000	42.900240000	38.810275000
Si	51.693953000	48.537676000	35.182973000
Si	54.400663000	48.007722000	36.448868000
Si	54.871072000	45.939347000	38.809785000
Si	51.694379000	40.287003000	35.181453000
Si	54.399392000	40.820000000	36.455329000
Si	45.724449000	40.464492000	40.179797000
Si	42.645597000	40.424147000	40.051349000
Si	41.767577000	45.945858000	41.709492000
Si	43.912100000	46.868537000	43.780968000
Si	48.896809000	47.042003000	43.341646000
Si	46.305590000	45.985912000	41.943107000

Si	45.702029000	48.451093000	40.223162000
Si	42.623074000	48.404916000	40.038834000
Si	41.775466000	42.890937000	41.756469000
Si	43.811235000	41.931565000	43.844972000
Si	48.960706000	41.763994000	43.477911000
Al	46.368354000	42.805396000	42.135351000
Si	51.907507000	42.853031000	43.625522000
Si	53.928553000	41.909045000	41.523127000
Si	51.824561000	45.913565000	43.560455000
Si	53.907322000	46.909250000	41.522990000
O	46.898218000	38.488731000	36.852665000
O	44.181944000	39.512186000	34.960503000
O	54.377608000	39.362264000	37.213504000
O	51.571893000	38.773674000	35.807824000
O	42.302328000	39.396485000	41.257460000
O	50.372506000	41.191970000	35.492609000
O	48.193242000	40.822772000	36.936761000
O	48.069886000	42.273475000	34.696594000
O	48.418402000	39.612964000	34.570725000
O	46.838745000	40.003252000	39.070885000
O	45.524028000	40.810248000	36.905569000
O	41.629812000	40.055182000	38.827204000
O	42.855554000	41.068348000	36.653419000
O	44.507218000	42.203811000	34.927655000
O	44.165673000	49.310343000	34.969158000
O	43.722895000	46.753890000	32.359478000
O	44.048001000	44.414216000	33.558443000
O	46.182079000	45.927875000	32.955516000
O	48.394940000	44.429204000	33.146966000
O	48.562027000	46.826909000	32.047852000
O	50.363002000	47.652898000	35.503117000
O	48.186079000	47.986406000	36.969866000
O	48.050051000	46.591553000	34.690488000
O	48.416426000	49.241158000	34.610718000
O	46.826694000	48.836773000	39.100162000
O	46.898325000	50.324715000	36.872048000
O	45.513875000	48.022109000	36.920205000
O	41.619785000	48.813859000	38.820572000
O	42.852915000	47.750599000	36.669434000
O	44.501476000	46.624284000	34.932441000
O	43.732257000	42.072911000	32.357686000
O	46.190880000	42.910540000	32.957837000
O	48.572798000	42.024930000	32.053470000
O	54.373217000	44.420902000	38.456602000
O	56.380598000	42.947240000	39.426751000
O	55.437249000	48.021496000	35.189053000
O	51.876212000	48.684743000	33.560405000
O	52.911054000	47.694986000	35.869005000
O	51.587118000	50.053556000	35.807411000
O	54.887542000	46.826462000	37.451110000
O	54.377824000	49.467178000	37.205187000
O	56.384089000	45.895768000	39.436743000
O	55.436254000	40.804280000	35.195850000
O	51.880662000	40.133109000	33.560352000
O	52.908678000	41.129237000	35.873351000
O	54.872704000	42.011682000	37.451556000
O	45.939786000	42.066199000	40.532531000
O	44.213591000	40.214466000	39.573033000

O	45.974518000	39.578358000	41.524819000
O	42.429921000	41.969679000	40.550911000
O	42.232357000	49.360208000	41.292594000
O	43.685421000	48.466832000	43.510408000
O	48.776289000	48.636038000	42.974256000
O	42.791529000	45.985064000	42.996874000
O	41.615107000	44.411710000	41.147728000
O	43.787833000	46.618711000	45.390570000
O	45.396236000	46.400823000	43.241447000
O	48.575528000	46.842934000	44.943141000
O	47.869795000	46.198004000	42.375688000
O	46.040553000	44.436713000	41.442182000
O	45.888671000	46.869477000	40.620454000
O	44.193066000	48.682131000	39.606193000
O	45.939657000	49.370449000	41.549170000
O	42.401464000	46.838593000	40.470344000
O	43.352586000	40.376058000	43.584253000
O	48.732741000	40.199050000	43.020006000
O	42.763111000	42.930165000	43.067429000
O	43.699413000	42.199144000	45.461322000
O	45.314045000	42.200163000	43.336714000
O	48.664700000	41.886290000	45.105446000
O	48.015499000	42.754598000	42.628275000
O	53.520762000	40.355744000	41.823006000
O	50.534035000	42.107453000	43.180163000
O	52.907753000	42.903844000	42.320890000
O	51.635490000	44.389548000	44.126100000
O	53.865257000	42.206548000	39.910987000
O	55.463162000	42.149821000	42.050759000
O	52.427298000	46.816826000	44.792595000
O	53.503469000	48.463293000	41.831361000
O	50.408627000	46.523823000	43.012309000
O	52.864002000	45.912119000	42.288492000
O	53.866185000	46.634635000	39.906398000
O	55.429743000	46.656504000	42.072754000
O	52.605930000	42.007364000	44.850078000
Ga	45.886068000	43.837963000	39.443117000
H	44.387230000	44.070592000	39.041123000
C	50.658909000	45.164521000	37.910098000
H	50.587208000	46.258480000	38.034289000
C	50.270898000	44.600823000	36.756741000
H	50.316734000	43.511093000	36.627277000
H	49.864411000	45.176608000	35.920480000
C	51.095064000	44.358498000	39.101852000
C	49.933986000	43.971075000	40.042902000
H	51.858171000	44.901185000	39.689421000
H	51.571851000	43.423071000	38.747358000
H	50.363835000	43.349743000	40.850657000
H	49.520026000	44.868905000	40.544856000
C	47.691460000	43.989040000	38.660837000
C	48.814555000	43.164359000	39.356861000
H	47.598455000	43.708485000	37.591654000
H	47.948284000	45.067316000	38.625583000
H	48.394755000	42.475813000	40.110280000
H	49.278051000	42.488998000	38.612547000

TS7-GaH$_2$

Electronic energy (r^{2}SCAN-D4/def2-SVP): -48412.98841272 E_h
Electronic energy (ωB97X-D4rev/def2-mTZVPP): -48457.062642721800 E_h
G_{corr} (673.15 K, 1 atm): 2.05072350 E_h
Number of atoms: 395

Cartesian coordinates (Å)

Si	48.558057000	36.025129000	35.318294000
Si	46.527645000	36.968400000	37.357094000
Si	44.101662000	36.090786000	35.636988000
Si	44.621148000	38.505327000	33.731283000
Si	47.766798000	38.518903000	33.545367000
Si	48.743382000	38.758741000	30.521368000
Si	51.802528000	38.125614000	30.330713000
Si	54.682162000	38.434927000	38.532959000
Si	57.779043000	38.491765000	38.801963000
Si	56.560176000	37.023057000	34.996912000
Si	52.597582000	36.084650000	32.663171000
Si	51.488561000	37.164668000	35.488160000
Si	54.140709000	36.091348000	36.720738000
Si	45.617832000	36.064771000	40.034804000
Si	41.741678000	38.012219000	41.952997000
Si	43.624545000	38.828694000	44.071596000
Si	47.726287000	38.457329000	46.611998000
Si	48.748428000	38.662665000	43.582480000
Si	46.060478000	38.136777000	42.337497000
Si	51.728271000	38.002419000	43.463740000
Si	53.659296000	38.794194000	41.331634000
Si	56.183254000	38.111630000	43.085216000
Si	44.616363000	50.310637000	33.737971000
Si	47.762762000	50.302803000	33.556522000
Si	48.742974000	50.065127000	30.526642000
Si	43.864925000	46.935319000	30.715891000
Si	48.872866000	46.960145000	30.432029000
Si	46.323135000	45.965066000	29.027534000
Si	48.555610000	52.794614000	35.317093000
Si	46.528587000	51.845822000	37.364357000
Si	44.102845000	52.729434000	35.638716000
Si	43.867055000	41.886042000	30.714780000
Si	48.871875000	41.868860000	30.437688000
Si	46.324961000	42.856857000	29.026953000
Si	51.885079000	42.863666000	30.524608000
Si	58.006843000	42.905447000	39.196657000
Si	58.732661000	47.849552000	36.909830000
Si	56.870329000	48.679686000	34.756218000
Si	55.873327000	48.297229000	31.971134000
Si	52.768216000	48.321135000	32.245962000
Si	56.560620000	51.798434000	34.997020000
Si	52.599561000	52.737660000	32.659960000
Si	51.489465000	51.656559000	35.480243000
Si	54.136958000	52.731254000	36.719549000
Si	51.802960000	50.699619000	30.332318000
Si	54.679996000	50.388544000	38.530082000
Si	57.779307000	50.328834000	38.800553000
Si	51.884678000	45.954469000	30.524092000
Si	58.011129000	45.924805000	39.190780000
Si	58.733825000	40.971309000	36.914643000

Si	56.868738000	40.144650000	34.760956000
Si	55.875595000	40.524118000	31.972564000
Si	52.771625000	40.498899000	32.245417000
Si	48.663197000	40.942681000	48.513561000
Si	41.741103000	50.789059000	41.947952000
Si	43.704113000	50.023997000	44.069737000
Si	47.726182000	50.362772000	46.611992000
Si	48.764597000	50.161787000	43.581143000
Si	46.081103000	50.794329000	42.375350000
Si	44.734356000	45.945758000	46.549264000
Si	47.916837000	45.940467000	46.146884000
Si	48.672134000	47.887259000	48.510756000
Si	45.616874000	52.772879000	40.030743000
Si	44.722736000	42.865391000	46.546194000
Si	47.908883000	42.858220000	46.177089000
Si	58.962672000	41.837321000	41.946962000
Si	56.316068000	42.833930000	43.276131000
Si	55.802487000	48.290773000	45.170994000
Si	52.648598000	48.348674000	45.337086000
Si	51.670080000	48.627483000	48.353750000
Si	51.740988000	50.824169000	43.460147000
Si	53.661135000	50.022065000	41.329885000
Si	56.182415000	50.705067000	43.088854000
Si	58.966913000	46.984921000	41.929569000
Si	56.320699000	45.964393000	43.265739000
Si	55.800740000	40.503231000	45.173140000
Si	52.685199000	40.443881000	45.354170000
Si	51.666991000	40.194972000	48.352630000
O	49.985430000	36.638507000	35.857643000
O	47.528110000	35.910095000	36.601063000
O	46.658594000	36.838909000	38.982198000
O	44.973794000	36.636013000	36.938667000
O	44.497987000	36.952612000	34.270930000
O	46.176172000	38.852517000	33.273758000
O	47.914894000	37.002940000	34.150629000
O	48.606671000	38.627677000	32.151691000
O	50.186535000	38.140928000	30.069863000
O	52.210337000	36.835222000	31.250095000
O	52.283233000	39.551589000	31.003593000
O	54.623956000	36.854212000	38.097164000
O	56.173601000	38.739011000	39.125988000
O	56.928607000	38.600761000	35.309810000
O	54.996111000	36.668159000	35.426579000
O	51.822539000	36.863365000	33.908166000
O	52.540758000	36.344224000	36.444215000
O	42.431566000	37.876653000	43.449433000
O	45.119629000	38.274168000	43.660031000
O	48.550210000	38.676177000	45.201496000
O	47.583142000	37.809181000	42.814982000
O	45.551888000	36.853642000	41.471241000
O	50.123382000	37.860335000	43.227103000
O	52.419250000	37.946339000	41.966689000
O	52.110708000	39.423852000	44.208808000
O	53.573005000	38.732877000	39.695979000
O	55.075343000	38.149224000	41.857354000
O	44.497397000	51.865548000	34.274356000
O	46.173121000	49.957740000	33.291512000
O	47.903277000	51.827943000	34.144329000

O	48.594489000	50.174491000	32.159186000
O	48.593047000	48.512556000	30.005862000
O	45.410056000	46.591304000	30.241493000
O	47.899067000	45.998754000	29.523051000
O	45.879834000	44.410936000	28.739541000
O	49.983570000	52.176783000	35.848159000
O	47.534687000	52.899976000	36.610488000
O	46.649478000	51.970019000	38.994443000
O	44.977273000	52.186784000	36.942018000
O	48.577658000	40.316594000	30.021502000
O	45.412361000	42.229350000	30.240268000
O	47.900835000	42.828748000	29.523337000
O	50.431614000	42.219175000	30.104154000
O	51.667383000	44.409336000	31.028002000
O	58.626325000	44.415050000	39.377090000
O	58.097520000	47.813588000	35.393371000
O	58.311070000	46.455610000	37.674824000
O	58.216824000	49.178816000	37.710092000
O	57.001887000	48.624964000	33.133328000
O	56.933064000	50.221933000	35.309076000
O	54.350823000	48.583729000	32.564952000
O	52.557838000	46.757998000	31.796623000
O	54.996892000	52.150153000	35.430661000
O	51.819456000	51.953443000	33.898657000
O	52.537249000	52.486792000	36.433046000
O	50.183953000	50.699072000	30.089766000
O	52.224301000	51.992042000	31.242169000
O	52.275857000	49.272504000	31.008341000
O	54.608281000	51.970612000	38.101854000
O	56.172140000	50.089750000	39.123336000
O	50.430523000	46.597457000	30.104876000
O	58.095463000	41.010842000	35.399522000
O	58.317202000	42.362344000	37.687299000
O	58.221725000	39.638576000	37.710111000
O	57.002045000	40.200460000	33.137719000
O	54.353719000	40.238515000	32.567808000
O	52.558480000	42.062826000	31.798777000
O	50.277920000	40.948627000	48.800428000
O	48.263101000	42.305161000	47.678823000
O	48.226311000	39.580566000	47.709302000
O	42.462697000	50.897413000	43.432654000
O	45.168088000	50.694227000	43.722988000
O	48.536539000	50.102334000	45.195531000
O	47.623165000	51.050752000	42.819808000
O	45.570345000	52.071246000	41.512184000
O	44.250407000	44.412221000	46.843875000
O	46.288280000	45.976995000	46.017486000
O	48.467100000	44.400668000	46.014312000
O	50.280279000	47.888550000	48.822826000
O	48.319570000	46.553378000	47.610814000
O	48.229023000	49.259361000	47.725251000
O	46.279822000	42.850967000	46.008847000
O	58.696056000	41.932480000	40.309340000
O	57.903706000	42.728619000	42.864230000
O	55.900900000	44.401436000	43.468395000
O	56.020216000	46.726625000	44.690116000
O	54.250335000	48.578712000	45.650101000
O	56.180011000	49.265622000	43.900126000

O	51.801828000	48.591649000	46.704933000
O	50.134846000	50.968336000	43.239982000
O	52.423560000	50.879738000	41.961587000
O	52.107985000	49.405221000	44.209435000
O	53.574678000	50.077837000	39.695042000
O	55.077346000	50.663213000	41.857774000
O	58.718387000	46.905326000	40.287873000
O	57.895484000	46.074100000	42.811796000
O	55.966895000	42.074916000	44.691003000
O	54.273728000	40.137367000	45.675805000
O	56.199031000	39.555593000	43.888611000
O	51.810472000	40.220348000	46.705703000
H	48.702871000	34.676568000	34.772566000
H	44.419875000	34.676323000	35.346829000
H	42.683628000	36.258975000	36.022159000
H	43.726523000	38.680216000	32.577941000
H	47.730594000	38.010996000	29.830569000
H	52.421136000	38.002380000	29.030380000
H	58.465353000	38.743363000	40.066967000
H	58.088382000	37.108509000	38.286195000
H	56.651061000	36.758841000	33.558678000
H	57.541582000	36.246995000	35.782467000
H	52.141234000	34.673922000	32.562045000
H	54.071037000	36.146097000	32.821438000
H	54.418082000	34.675478000	36.917895000
H	44.265382000	36.148734000	39.494230000
H	46.085045000	34.677316000	40.196440000
H	42.177304000	36.886192000	41.136491000
H	40.290195000	38.107003000	42.090377000
H	43.614692000	38.689573000	45.487247000
H	46.266760000	38.594605000	46.380864000
H	48.065374000	37.113072000	47.131272000
H	52.195251000	36.881484000	44.271734000
H	55.858296000	36.988842000	44.011824000
H	57.529558000	37.965437000	42.519250000
H	41.636484000	38.780154000	36.634495000
H	40.338249000	40.845235000	36.817795000
H	43.726296000	50.140518000	32.578071000
H	47.730503000	50.809921000	29.830551000
H	42.910974000	46.071594000	29.969678000
H	43.636013000	48.346500000	30.474316000
H	46.055518000	46.690813000	27.779371000
H	40.338195000	47.975592000	36.817799000
H	41.636403000	50.040687000	36.634467000
H	48.702714000	54.144377000	34.772532000
H	44.419585000	54.144606000	35.346948000
H	42.683575000	52.561616000	36.022276000
H	43.636069000	40.474361000	30.474327000
H	42.910998000	42.749253000	29.969681000
H	46.055551000	42.130076000	27.779377000
H	52.768240000	42.805898000	29.339147000
H	60.152182000	47.950749000	36.769108000
H	56.115708000	49.276599000	30.928772000
H	56.049418000	46.904457000	31.507081000
H	57.541465000	52.574079000	35.782446000
H	56.650952000	52.062214000	33.558656000
H	52.141090000	54.147065000	32.562019000
H	54.070917000	52.674925000	32.821418000

H	54.418140000	54.145525000	36.917811000
H	52.421044000	50.818602000	29.030363000
H	58.465269000	50.077736000	40.066952000
H	58.088278000	51.712582000	38.286179000
H	52.768217000	46.015092000	29.339142000
H	60.152234000	40.870368000	36.769119000
H	56.049458000	41.916585000	31.507090000
H	56.115777000	39.544444000	30.928783000
H	48.027948000	40.958435000	49.819979000
H	40.290115000	50.713683000	42.090582000
H	42.176850000	51.934815000	41.136521000
H	43.615015000	50.131729000	45.487215000
H	48.065229000	51.707918000	47.131232000
H	46.266685000	50.226321000	46.380825000
H	40.476352000	46.423809000	42.229530000
H	44.578030000	46.685732000	47.785515000
H	48.027871000	47.862537000	49.819961000
H	46.084739000	54.143668000	40.196331000
H	44.265252000	52.672179000	39.494179000
H	40.476382000	42.397038000	42.229542000
H	44.578055000	42.135174000	47.785513000
H	58.818796000	40.408567000	42.221796000
H	60.309668000	42.345550000	42.255160000
H	56.725235000	48.548904000	46.274187000
H	51.671681000	50.045758000	48.769745000
H	52.721673000	47.848745000	49.007593000
H	52.195144000	51.939533000	44.271718000
H	55.858180000	51.832229000	44.011806000
H	57.529465000	50.855664000	42.519236000
H	60.309639000	46.475584000	42.255156000
H	58.818736000	48.412544000	42.221786000
H	56.725177000	40.272281000	46.274313000
H	52.722235000	40.971967000	49.006966000
H	51.671815000	38.775269000	48.769821000
Si	48.762594000	40.968386000	35.412020000
Si	46.856735000	40.021019000	37.449451000
Si	41.573177000	40.151740000	37.186426000
Si	44.262812000	40.883401000	35.860552000
Si	44.621743000	45.940896000	33.464749000
Si	47.798116000	45.951503000	33.206391000
Si	48.757585000	47.869157000	35.434996000
Si	46.850557000	48.790042000	37.472107000
Si	41.570638000	48.672838000	37.181666000
Si	44.260787000	47.937684000	35.870574000
Si	44.626703000	42.887990000	33.463442000
Si	47.805512000	42.898325000	33.210794000
Si	54.874508000	42.900234000	38.812341000
Si	51.691637000	48.530564000	35.189091000
Si	54.401143000	48.010699000	36.452379000
Si	54.882620000	45.941357000	38.812562000
Si	51.691998000	40.289900000	35.185462000
Si	54.398257000	40.818458000	36.457351000
Si	45.713399000	40.461511000	40.177860000
Si	42.642227000	40.413127000	40.048903000
Si	41.769139000	45.941797000	41.710540000
Si	43.913749000	46.878715000	43.780012000
Si	48.884557000	47.039117000	43.335711000
Si	46.291422000	45.985003000	41.946631000

Si	45.694870000	48.457115000	40.224268000
Si	42.620988000	48.406101000	40.039826000
Si	41.776631000	42.886251000	41.753372000
Si	43.836747000	41.932160000	43.829470000
Si	48.938667000	41.771263000	43.433965000
Al	46.344438000	42.823098000	42.075722000
Si	51.894448000	42.853123000	43.621149000
Si	53.926119000	41.910845000	41.525431000
Si	51.817200000	45.915874000	43.558808000
Si	53.905246000	46.909491000	41.525220000
O	46.908560000	38.487799000	36.850527000
O	44.173217000	39.513816000	34.956931000
O	54.374073000	39.361666000	37.214609000
O	51.569508000	38.776072000	35.807932000
O	42.305452000	39.388955000	41.259813000
O	50.372038000	41.199361000	35.504448000
O	48.193823000	40.825187000	36.941813000
O	48.077874000	42.275021000	34.701528000
O	48.425862000	39.610684000	34.569403000
O	46.836457000	39.988185000	39.073249000
O	45.528956000	40.803074000	36.893213000
O	41.620572000	40.037875000	38.828928000
O	42.854604000	41.065678000	36.660673000
O	44.500354000	42.203252000	34.929937000
O	44.163182000	49.309169000	34.968415000
O	43.724692000	46.755126000	32.359515000
O	44.050203000	44.413898000	33.554141000
O	46.183572000	45.931530000	32.960728000
O	48.394561000	44.426511000	33.157134000
O	48.565485000	46.822337000	32.049407000
O	50.366093000	47.634903000	35.520304000
O	48.188266000	47.980733000	36.971564000
O	48.061419000	46.586155000	34.693780000
O	48.428537000	49.240292000	34.608006000
O	46.826207000	48.835920000	39.100164000
O	46.907754000	50.323320000	36.872559000
O	45.519309000	48.022658000	36.911254000
O	41.615807000	48.819274000	38.822636000
O	42.852866000	47.752923000	36.673414000
O	44.498421000	46.623995000	34.933152000
O	43.732187000	42.072208000	32.357605000
O	46.190369000	42.906085000	32.963991000
O	48.573795000	42.025930000	32.054993000
O	54.384622000	44.422681000	38.451127000
O	56.387116000	42.947437000	39.429678000
O	55.432072000	48.023450000	35.188836000
O	51.875312000	48.669787000	33.567191000
O	52.906862000	47.687877000	35.883975000
O	51.580001000	50.047908000	35.806942000
O	54.890253000	46.828478000	37.453627000
O	54.373854000	49.469682000	37.206566000
O	56.392079000	45.897466000	39.441509000
O	55.433441000	40.804921000	35.197079000
O	51.878854000	40.142815000	33.564394000
O	52.905257000	41.134285000	35.881022000
O	54.870645000	42.011758000	37.453896000
O	45.919327000	42.053171000	40.508368000
O	44.205101000	40.193365000	39.560581000

O	45.961124000	39.552550000	41.512165000
O	42.405444000	41.952466000	40.546769000
O	42.237742000	49.367369000	41.291541000
O	43.659790000	48.474729000	43.512157000
O	48.777430000	48.636234000	42.971669000
O	42.807195000	45.983589000	42.988336000
O	41.595777000	44.409475000	41.156044000
O	43.784187000	46.625225000	45.390435000
O	45.413348000	46.454518000	43.254709000
O	48.568714000	46.843447000	44.938698000
O	47.869253000	46.197288000	42.361192000
O	45.998593000	44.456969000	41.462439000
O	45.882196000	46.885614000	40.629641000
O	44.189000000	48.687888000	39.597719000
O	45.931444000	49.392916000	41.538087000
O	42.392766000	46.843169000	40.470750000
O	43.374353000	40.374227000	43.576428000
O	48.745017000	40.192915000	43.007018000
O	42.779363000	42.923360000	43.053327000
O	43.708431000	42.197335000	45.448514000
O	45.341877000	42.201604000	43.329319000
O	48.636613000	41.908029000	45.059479000
O	48.012515000	42.734596000	42.537038000
O	53.519732000	40.356851000	41.817051000
O	50.519488000	42.125633000	43.158018000
O	52.898961000	42.904443000	42.315361000
O	51.638077000	44.391507000	44.124910000
O	53.865503000	42.217265000	39.911920000
O	55.460128000	42.153241000	42.051755000
O	52.429499000	46.819571000	44.785232000
O	53.503225000	48.464088000	41.826656000
O	50.401311000	46.520898000	43.008657000
O	52.851822000	45.914099000	42.278313000
O	53.868170000	46.629199000	39.906216000
O	55.425799000	46.653149000	42.074915000
O	52.592867000	42.004230000	44.842371000
Ga	45.623488000	43.910449000	39.268503000
H	43.993443000	44.015644000	39.395581000
C	50.682044000	45.153844000	38.004608000
H	50.660595000	46.253630000	38.073112000
C	49.856470000	44.520114000	37.076552000
H	50.102209000	43.479351000	36.813809000
H	49.438084000	45.099162000	36.244465000
C	51.245592000	44.373953000	39.136360000
C	50.067773000	43.933747000	40.051089000
H	51.978929000	44.947910000	39.723371000
H	51.737596000	43.455626000	38.760919000
H	50.459319000	43.263534000	40.835933000
H	49.633603000	44.811199000	40.570903000
C	48.340674000	44.111930000	38.193958000
C	48.988365000	43.213808000	39.239719000
H	47.716062000	43.668121000	37.402850000
H	48.028104000	45.123423000	38.477256000
H	48.253024000	42.784162000	39.935681000
H	49.426123000	42.336372000	38.722770000

I12-GaH$_2$

Electronic energy (r^{2}SCAN-D4/def2-SVP): -48413.11422163 E_h
Electronic energy (ωB97X-D4rev/def2-mTZVPP): -48457.223410609900 E_h
G_{corr} (673.15 K, 1 atm): 2.06650502 E_h
Number of atoms: 395

Cartesian coordinates (Å)

Si	48.555492000	36.020675000	35.319626000
Si	46.522250000	36.943225000	37.350890000
Si	44.099057000	36.087192000	35.635008000
Si	44.629233000	38.501679000	33.724101000
Si	47.770080000	38.505565000	33.535985000
Si	48.744744000	38.757403000	30.518933000
Si	51.806427000	38.132294000	30.327188000
Si	54.677918000	38.462717000	38.535181000
Si	57.777735000	38.489644000	38.802992000
Si	56.556232000	37.022064000	34.996953000
Si	52.595578000	36.085450000	32.662883000
Si	51.482496000	37.160989000	35.487547000
Si	54.134718000	36.093827000	36.718783000
Si	45.619114000	36.068885000	40.034380000
Si	41.742974000	38.018148000	41.944699000
Si	43.600883000	38.847076000	44.073289000
Si	47.726673000	38.459870000	46.618301000
Si	48.754636000	38.655340000	43.596406000
Si	46.064457000	38.161337000	42.336304000
Si	51.724113000	37.999066000	43.463050000
Si	53.668406000	38.782745000	41.344331000
Si	56.185617000	38.105399000	43.084310000
Si	44.621120000	50.307572000	33.736651000
Si	47.763476000	50.321619000	33.543837000
Si	48.744993000	50.067922000	30.520135000
Si	43.865734000	46.935241000	30.716133000
Si	48.872878000	46.958857000	30.434683000
Si	46.324486000	45.965092000	29.027332000
Si	48.549461000	52.797637000	35.315346000
Si	46.516442000	51.875754000	37.363115000
Si	44.099461000	52.735277000	35.640491000
Si	43.868289000	41.885626000	30.713931000
Si	48.872950000	41.871828000	30.441809000
Si	46.326827000	42.857310000	29.026448000
Si	51.882224000	42.862306000	30.522947000
Si	57.964317000	42.902717000	39.190476000
Si	58.733926000	47.857365000	36.911527000
Si	56.872998000	48.675053000	34.760799000
Si	55.869987000	48.297181000	31.973798000
Si	52.765898000	48.321623000	32.239940000
Si	56.555048000	51.799227000	34.997340000
Si	52.598011000	52.736932000	32.657708000
Si	51.482840000	51.658504000	35.474154000
Si	54.126534000	52.729623000	36.714346000
Si	51.807023000	50.694704000	30.328911000
Si	54.673040000	50.355770000	38.531401000
Si	57.777323000	50.331933000	38.802136000
Si	51.881832000	45.955365000	30.522566000
Si	57.966492000	45.921393000	39.186528000
Si	58.734975000	40.963784000	36.914013000

Si	56.875679000	40.146776000	34.762296000
Si	55.874250000	40.524345000	31.974330000
Si	52.772010000	40.497756000	32.236107000
Si	48.665820000	40.949967000	48.520668000
Si	41.743976000	50.781115000	41.944608000
Si	43.719478000	50.006965000	44.069951000
Si	47.726330000	50.361338000	46.611892000
Si	48.774294000	50.172479000	43.580925000
Si	46.087924000	50.782598000	42.371553000
Si	44.786684000	45.964892000	46.544945000
Si	47.954373000	45.958588000	46.151699000
Si	48.680469000	47.884801000	48.517047000
Si	45.616646000	52.772292000	40.030427000
Si	44.776567000	42.846034000	46.543236000
Si	47.942469000	42.838186000	46.194019000
Si	58.955385000	41.841054000	41.953102000
Si	56.319391000	42.834704000	43.281585000
Si	55.792648000	48.300723000	45.171934000
Si	52.642996000	48.352853000	45.339975000
Si	51.672502000	48.629231000	48.357299000
Si	51.738639000	50.825967000	43.462119000
Si	53.670875000	50.025643000	41.343479000
Si	56.184355000	50.710250000	43.088672000
Si	58.957656000	46.979814000	41.942374000
Si	56.324135000	45.966080000	43.275689000
Si	55.797593000	40.495176000	45.169650000
Si	52.685591000	40.438632000	45.350489000
Si	51.670424000	40.194017000	48.351129000
O	49.976211000	36.643824000	35.863034000
O	47.523519000	35.884808000	36.600848000
O	46.661579000	36.831720000	38.976756000
O	44.964859000	36.613312000	36.948439000
O	44.500948000	36.952541000	34.274101000
O	46.180529000	38.841965000	33.254529000
O	47.901437000	36.993816000	34.155942000
O	48.616531000	38.594472000	32.146333000
O	50.192044000	38.158950000	30.057997000
O	52.204236000	36.844740000	31.255444000
O	52.307994000	39.560692000	30.977185000
O	54.605314000	36.886379000	38.081848000
O	56.174045000	38.738513000	39.131403000
O	56.911233000	38.601762000	35.311343000
O	54.991494000	36.661143000	35.421249000
O	51.812596000	36.853008000	33.908968000
O	52.532572000	36.332941000	36.439542000
O	42.421326000	37.883820000	43.446655000
O	45.099460000	38.327469000	43.636351000
O	48.565729000	38.663667000	45.215255000
O	47.572568000	37.814094000	42.839445000
O	45.552832000	36.889633000	41.455800000
O	50.118598000	37.847152000	43.219647000
O	52.417575000	37.941017000	41.968639000
O	52.081951000	39.422363000	44.215588000
O	53.575467000	38.748738000	39.709552000
O	55.078887000	38.115440000	41.855451000
O	44.503293000	51.857479000	34.288751000
O	46.175956000	49.957660000	33.283757000
O	47.879608000	51.844875000	34.142440000

O	48.595804000	50.214208000	32.147213000
O	48.581979000	48.511551000	30.017189000
O	45.409469000	46.591854000	30.239539000
O	47.899790000	45.998172000	29.523261000
O	45.880333000	44.410990000	28.739028000
O	49.970647000	52.162365000	35.843199000
O	47.532035000	52.919377000	36.611275000
O	46.654671000	51.980091000	38.993015000
O	44.963950000	52.231518000	36.963835000
O	48.562399000	40.318980000	30.039322000
O	45.411789000	42.227766000	30.236839000
O	47.901634000	42.829977000	29.525211000
O	50.432899000	42.210124000	30.096242000
O	51.656106000	44.408942000	31.020065000
O	58.586595000	44.412188000	39.362877000
O	58.121681000	47.830817000	35.385670000
O	58.293193000	46.474097000	37.682807000
O	58.218842000	49.190502000	37.705287000
O	56.996488000	48.627993000	33.136646000
O	56.907716000	50.219205000	35.312668000
O	54.347749000	48.576665000	32.575193000
O	52.552717000	46.755920000	31.798601000
O	54.990675000	52.159602000	35.423170000
O	51.809040000	51.957583000	33.892892000
O	52.524668000	52.506351000	36.418956000
O	50.189967000	50.684884000	30.076347000
O	52.220964000	51.986937000	31.242622000
O	52.295827000	49.265913000	30.988101000
O	54.578994000	51.931362000	38.080627000
O	56.171682000	50.093123000	39.127170000
O	50.430920000	46.604583000	30.099409000
O	58.121878000	40.993682000	35.388454000
O	58.298047000	42.345244000	37.690169000
O	58.220717000	39.628591000	37.703932000
O	57.000352000	40.196053000	33.137961000
O	54.352725000	40.246432000	32.576640000
O	52.551720000	42.064677000	31.801953000
O	50.279675000	40.944941000	48.799644000
O	48.263972000	42.334273000	47.719255000
O	48.210612000	39.601078000	47.703317000
O	42.470550000	50.866627000	43.428226000
O	45.177230000	50.687892000	43.722003000
O	48.533254000	50.112697000	45.192693000
O	47.628710000	51.047085000	42.809476000
O	45.564814000	52.049209000	41.502161000
O	44.395990000	44.410995000	46.855530000
O	46.329401000	46.087710000	45.990357000
O	48.433195000	44.397782000	46.009165000
O	50.283369000	47.901264000	48.844318000
O	48.353450000	46.532748000	47.631221000
O	48.226126000	49.242429000	47.711865000
O	46.318312000	42.740495000	45.965115000
O	58.646683000	41.945802000	40.323577000
O	57.913968000	42.726537000	42.897736000
O	55.903480000	44.402239000	43.475937000
O	55.980372000	46.733360000	44.688789000
O	54.242193000	48.608742000	45.650429000
O	56.178836000	49.269953000	43.899199000

O	51.794446000	48.584294000	46.709811000
O	50.133969000	50.993790000	43.240262000
O	52.422912000	50.876196000	41.965807000
O	52.074678000	49.402926000	44.218788000
O	53.577103000	50.056814000	39.709885000
O	55.081297000	50.690889000	41.856159000
O	58.657747000	46.884826000	40.310445000
O	57.911362000	46.079266000	42.866701000
O	55.944568000	42.068588000	44.687242000
O	54.271540000	40.112381000	45.666980000
O	56.201838000	39.552047000	43.883379000
O	51.815057000	40.223625000	46.706714000
H	48.702860000	34.676566000	34.772565000
H	44.419864000	34.676320000	35.346828000
H	42.683616000	36.258972000	36.022158000
H	43.726512000	38.680218000	32.577942000
H	47.730581000	38.010994000	29.830569000
H	52.421124000	38.002378000	29.030380000
H	58.465341000	38.743359000	40.066967000
H	58.088369000	37.108506000	38.286195000
H	56.651047000	36.758839000	33.558678000
H	57.541568000	36.246991000	35.782467000
H	52.141221000	34.673920000	32.562044000
H	54.071024000	36.146095000	32.821437000
H	54.418069000	34.675476000	36.917894000
H	44.265369000	36.148732000	39.494228000
H	46.085031000	34.677312000	40.196438000
H	42.177291000	36.886190000	41.136489000
H	40.290185000	38.107009000	42.090371000
H	43.614681000	38.689570000	45.487245000
H	46.266745000	38.594600000	46.380862000
H	48.065359000	37.113066000	47.131271000
H	52.195239000	36.881481000	44.271733000
H	55.858281000	36.988837000	44.011824000
H	57.529544000	37.965433000	42.519251000
H	41.636483000	38.780154000	36.634495000
H	40.338248000	40.845234000	36.817796000
H	43.726283000	50.140513000	32.578072000
H	47.730488000	50.809920000	29.830553000
H	42.910961000	46.071589000	29.969683000
H	43.635999000	48.346497000	30.474315000
H	46.055506000	46.690810000	27.779370000
H	40.338195000	47.975591000	36.817800000
H	41.636401000	50.040687000	36.634466000
H	48.702701000	54.144374000	34.772535000
H	44.419569000	54.144602000	35.346952000
H	42.683560000	52.561610000	36.022277000
H	43.636057000	40.474359000	30.474328000
H	42.910986000	42.749252000	29.969685000
H	46.055539000	42.130075000	27.779375000
H	52.768228000	42.805898000	29.339147000
H	60.152170000	47.950749000	36.769113000
H	56.115695000	49.276597000	30.928772000
H	56.049407000	46.904455000	31.507084000
H	57.541449000	52.574079000	35.782449000
H	56.650937000	52.062213000	33.558659000
H	52.141075000	54.147064000	32.562022000
H	54.070902000	52.674923000	32.821421000

H	54.418126000	54.145523000	36.917814000
H	52.421030000	50.818602000	29.030365000
H	58.465254000	50.077733000	40.066955000
H	58.088264000	51.712580000	38.286183000
H	52.768205000	46.015089000	29.339144000
H	60.152222000	40.870364000	36.769122000
H	56.049447000	41.916584000	31.507091000
H	56.115766000	39.544444000	30.928782000
H	48.027932000	40.958429000	49.819978000
H	40.290102000	50.713668000	42.090581000
H	42.176831000	51.934814000	41.136526000
H	43.615002000	50.131725000	45.487217000
H	48.065211000	51.707912000	47.131234000
H	46.266668000	50.226315000	46.380826000
H	40.476351000	46.423808000	42.229529000
H	44.578020000	46.685727000	47.785517000
H	48.027853000	47.862529000	49.819963000
H	46.084721000	54.143664000	40.196332000
H	44.265237000	52.672173000	39.494181000
H	40.476382000	42.397038000	42.229542000
H	44.578044000	42.135170000	47.785514000
H	58.818780000	40.408563000	42.221798000
H	60.309656000	42.345544000	42.255163000
H	56.725221000	48.548900000	46.274189000
H	51.671663000	50.045752000	48.769747000
H	52.721656000	47.848739000	49.007596000
H	52.195131000	51.939529000	44.271720000
H	55.858165000	51.832225000	44.011810000
H	57.529450000	50.855661000	42.519240000
H	60.309626000	46.475584000	42.255160000
H	58.818720000	48.412541000	42.221790000
H	56.725162000	40.272276000	46.274314000
H	52.722219000	40.971960000	49.006967000
H	51.671799000	38.775263000	48.769818000
Si	48.772203000	40.958657000	35.394736000
Si	46.864598000	39.987942000	37.421898000
Si	41.585684000	40.161738000	37.176867000
Si	44.280353000	40.885293000	35.849009000
Si	44.629899000	45.936824000	33.459886000
Si	47.801640000	45.954563000	33.215255000
Si	48.764210000	47.893350000	35.413914000
Si	46.835824000	48.825624000	37.455111000
Si	41.576407000	48.668797000	37.173296000
Si	44.265981000	47.929042000	35.864683000
Si	44.640461000	42.890030000	33.454479000
Si	47.811931000	42.903942000	33.217343000
Si	54.807347000	42.893310000	38.829584000
Si	51.687515000	48.539624000	35.164745000
Si	54.392135000	47.956823000	36.442762000
Si	54.811410000	45.929791000	38.830852000
Si	51.688670000	40.278254000	35.154544000
Si	54.391507000	40.859455000	36.444285000
Si	45.771806000	40.467871000	40.168808000
Si	42.674204000	40.438045000	40.057182000
Si	41.762676000	45.945334000	41.705346000
Si	43.917273000	46.846837000	43.775472000
Si	48.942308000	47.050321000	43.356027000
Si	46.344500000	45.979637000	41.942244000

Si	45.722830000	48.449798000	40.221564000
Si	42.633480000	48.398045000	40.037515000
Si	41.770619000	42.900086000	41.764763000
Si	43.786059000	41.949487000	43.842719000
Si	48.992778000	41.755913000	43.498346000
Al	46.390375000	42.793409000	42.130705000
Si	51.924274000	42.849783000	43.611918000
Si	53.950759000	41.877070000	41.536678000
Si	51.854984000	45.914862000	43.556667000
Si	53.938189000	46.935079000	41.538075000
O	46.889780000	38.458118000	36.823199000
O	44.192693000	39.512841000	34.952139000
O	54.380814000	39.422370000	37.239929000
O	51.571450000	38.772478000	35.801034000
O	42.327210000	39.386993000	41.244011000
O	50.377329000	41.198509000	35.471265000
O	48.208531000	40.770294000	36.920336000
O	48.057714000	42.257242000	34.701523000
O	48.432841000	39.601911000	34.547877000
O	46.851752000	39.953246000	39.049914000
O	45.535090000	40.794881000	36.899623000
O	41.664102000	40.082882000	38.823168000
O	42.859834000	41.071595000	36.629697000
O	44.533730000	42.203833000	34.923955000
O	44.168070000	49.298460000	34.961274000
O	43.727760000	46.754426000	32.360632000
O	44.052472000	44.411203000	33.551668000
O	46.188222000	45.924423000	32.950275000
O	48.401207000	44.430997000	33.186041000
O	48.572682000	46.813411000	32.051238000
O	50.367835000	47.639237000	35.504098000
O	48.186646000	48.072539000	36.934922000
O	48.040844000	46.616998000	34.691574000
O	48.458753000	49.264329000	34.575451000
O	46.825954000	48.866193000	39.086691000
O	46.853242000	50.353843000	36.847476000
O	45.512693000	48.022108000	36.920698000
O	41.637454000	48.812448000	38.815312000
O	42.850157000	47.743518000	36.656827000
O	44.512893000	46.614187000	34.932963000
O	43.738452000	42.068624000	32.358465000
O	46.199140000	42.916247000	32.944974000
O	48.588689000	42.041325000	32.059432000
O	54.235541000	44.412638000	38.615477000
O	56.342229000	42.951011000	39.392051000
O	55.460603000	47.980724000	35.210840000
O	51.852272000	48.695937000	33.540191000
O	52.913149000	47.687092000	35.817725000
O	51.594319000	50.051713000	35.801916000
O	54.802207000	46.717780000	37.411002000
O	54.382295000	49.394741000	37.236957000
O	56.345078000	45.876037000	39.399301000
O	55.463086000	40.838242000	35.215125000
O	51.860279000	40.106961000	33.532384000
O	52.915059000	41.124918000	35.811976000
O	54.792774000	42.102794000	37.411157000
O	46.066013000	42.061037000	40.502361000
O	44.246463000	40.252476000	39.587273000

O	46.010395000	39.589381000	41.520439000
O	42.455328000	41.975244000	40.580017000
O	42.239593000	49.356959000	41.288201000
O	43.711253000	48.451304000	43.518390000
O	48.805487000	48.641633000	42.983965000
O	42.777247000	45.985355000	42.998085000
O	41.608107000	44.413071000	41.139309000
O	43.797512000	46.583016000	45.383869000
O	45.384838000	46.351929000	43.215324000
O	48.665577000	46.845756000	44.966176000
O	47.890014000	46.202031000	42.426814000
O	46.144010000	44.430398000	41.409294000
O	45.951537000	46.874764000	40.620306000
O	44.207365000	48.659844000	39.614097000
O	45.948066000	49.367592000	41.550687000
O	42.401818000	46.833461000	40.467061000
O	43.303067000	40.395914000	43.608038000
O	48.749882000	40.193938000	43.038873000
O	42.726626000	42.959107000	43.097819000
O	43.716506000	42.229232000	45.458507000
O	45.268776000	42.198606000	43.271651000
O	48.754095000	41.883194000	45.136860000
O	48.012178000	42.746898000	42.693457000
O	53.556804000	40.330405000	41.885428000
O	50.552742000	42.110440000	43.150419000
O	52.937381000	42.893568000	42.316455000
O	51.656876000	44.388129000	44.112012000
O	53.864926000	42.099789000	39.913157000
O	55.489108000	42.158404000	42.035429000
O	52.440807000	46.818035000	44.797365000
O	53.542293000	48.481488000	41.892774000
O	50.446689000	46.530911000	42.991452000
O	52.913036000	45.916351000	42.299956000
O	53.870912000	46.729273000	39.912249000
O	55.469582000	46.650932000	42.051365000
O	52.608801000	42.001081000	44.842985000
Ga	46.195408000	43.829541000	39.413693000
H	44.749519000	44.159387000	38.882029000
C	47.951683000	43.694875000	38.481000000
H	47.579540000	43.082190000	37.627286000
C	49.100151000	42.906644000	39.152003000
H	49.763322000	42.531531000	38.341941000
H	48.729997000	41.999180000	39.666322000
C	48.471126000	45.015813000	37.870087000
C	49.315104000	45.843057000	38.852391000
H	47.633564000	45.616619000	37.465894000
H	49.107796000	44.765168000	36.991314000
H	49.699621000	46.748180000	38.351178000
H	48.677171000	46.202496000	39.688263000
C	49.952727000	43.752968000	40.107009000
C	50.473025000	45.018891000	39.421634000
H	50.796897000	43.141383000	40.477837000
H	49.360424000	44.038225000	40.999562000
H	51.067227000	45.625257000	40.130591000
H	51.165334000	44.740476000	38.597890000

TS8-GaH$_2$

Electronic energy (r^{2}SCAN-D4/def2-SVP): -48413.03033650 E_h
Electronic energy (ωB97X-D4rev/def2-mTZVPP): -48457.127134928000 E_h
G_{corr} (673.15 K, 1 atm): 2.05387368 E_h
Number of atoms: 395

Cartesian coordinates (Å)

Si	48.553238000	36.012355000	35.318575000
Si	46.527513000	36.932667000	37.354036000
Si	44.104129000	36.080734000	35.632637000
Si	44.626647000	38.498494000	33.723761000
Si	47.765912000	38.492858000	33.539201000
Si	48.747390000	38.745993000	30.521234000
Si	51.810778000	38.124054000	30.322239000
Si	54.678630000	38.463093000	38.526997000
Si	57.781007000	38.483349000	38.797061000
Si	56.558325000	37.015503000	34.990827000
Si	52.600744000	36.078970000	32.654532000
Si	51.484353000	37.153513000	35.474933000
Si	54.133901000	36.086384000	36.707323000
Si	45.622024000	36.050191000	40.032768000
Si	41.754934000	38.043932000	41.936544000
Si	43.703632000	38.879321000	44.070095000
Si	47.730071000	38.463034000	46.612546000
Si	48.751440000	38.686529000	43.585151000
Si	46.080747000	38.080505000	42.351025000
Si	51.745613000	38.013439000	43.471172000
Si	53.682648000	38.776231000	41.339060000
Si	56.187463000	38.101551000	43.080902000
Si	44.610904000	50.295919000	33.740116000
Si	47.751520000	50.313889000	33.561584000
Si	48.743010000	50.069183000	30.535476000
Si	43.870391000	46.928528000	30.709769000
Si	48.874953000	46.952742000	30.447483000
Si	46.331605000	45.960377000	29.022991000
Si	48.548142000	52.792828000	35.333020000
Si	46.528343000	51.846219000	37.396465000
Si	44.110257000	52.725934000	35.644176000
Si	43.870456000	41.879154000	30.709757000
Si	48.877181000	41.861563000	30.443431000
Si	46.332019000	42.849853000	29.022335000
Si	51.887510000	42.856542000	30.517911000
Si	57.975389000	42.898749000	39.181883000
Si	58.738467000	47.850606000	36.904730000
Si	56.880755000	48.670300000	34.752451000
Si	55.875775000	48.291823000	31.967988000
Si	52.774579000	48.315162000	32.236506000
Si	56.561087000	51.793160000	34.990738000
Si	52.604037000	52.732436000	32.647683000
Si	51.490822000	51.656239000	35.461396000
Si	54.131248000	52.724535000	36.706202000
Si	51.812381000	50.690170000	30.325298000
Si	54.676188000	50.348721000	38.529800000
Si	57.781034000	50.326155000	38.796568000
Si	51.888266000	45.950286000	30.518947000
Si	57.982483000	45.914859000	39.178100000
Si	58.739975000	40.956553000	36.906985000

Si	56.875361000	40.141603000	34.757888000
Si	55.875386000	40.518080000	31.968886000
Si	52.772932000	40.492419000	32.233307000
Si	48.693643000	40.951963000	48.520202000
Si	41.757850000	50.751238000	41.938699000
Si	43.781207000	49.939066000	44.075233000
Si	47.727640000	50.352204000	46.611109000
Si	48.770560000	50.189804000	43.577003000
Si	46.080614000	50.882897000	42.385198000
Si	44.912338000	46.001563000	46.537025000
Si	47.948217000	46.025754000	46.130191000
Si	48.701360000	47.871653000	48.514193000
Si	45.608290000	52.783190000	40.051726000
Si	44.921513000	42.780559000	46.528728000
Si	47.968637000	42.780253000	46.166482000
Si	58.962846000	41.835692000	41.946939000
Si	56.338806000	42.829192000	43.267831000
Si	55.793005000	48.283427000	45.167192000
Si	52.628764000	48.322052000	45.329002000
Si	51.678624000	48.623561000	48.356260000
Si	51.740985000	50.818472000	43.462436000
Si	53.674341000	50.021984000	41.342000000
Si	56.188130000	50.702893000	43.085536000
Si	58.966183000	46.975280000	41.931484000
Si	56.336607000	45.958468000	43.260867000
Si	55.791971000	40.505117000	45.175439000
Si	52.676537000	40.470605000	45.366526000
Si	51.674374000	40.186359000	48.359024000
O	49.975478000	36.646726000	35.848792000
O	47.531879000	35.871668000	36.606427000
O	46.662255000	36.824073000	38.980197000
O	44.971475000	36.598854000	36.946844000
O	44.504825000	36.948869000	34.272319000
O	46.180140000	38.845041000	33.260879000
O	47.890125000	36.978739000	34.153261000
O	48.610896000	38.582562000	32.147269000
O	50.195053000	38.145849000	30.062334000
O	52.216824000	36.834423000	31.243206000
O	52.307979000	39.552599000	30.976911000
O	54.599065000	36.890164000	38.065465000
O	56.177037000	38.731745000	39.120932000
O	56.906225000	38.596958000	35.308078000
O	54.993374000	36.651801000	35.410608000
O	51.813954000	36.852181000	33.894657000
O	52.531714000	36.315273000	36.421722000
O	42.464125000	37.997161000	43.427598000
O	45.158586000	38.205270000	43.689924000
O	48.559023000	38.679287000	45.204462000
O	47.618394000	37.798843000	42.809413000
O	45.577649000	36.788806000	41.495462000
O	50.141853000	37.921043000	43.217172000
O	52.440305000	37.937051000	41.977080000
O	52.140301000	39.421610000	44.230734000
O	53.578742000	38.744964000	39.704634000
O	55.098687000	38.110061000	41.836241000
O	44.504277000	51.846560000	34.290334000
O	46.168489000	49.937182000	33.301842000
O	47.859284000	51.831446000	34.176058000

O	48.583997000	50.226256000	32.162930000
O	48.581441000	48.508855000	30.046336000
O	45.415553000	46.581046000	30.238630000
O	47.905860000	45.999610000	29.524388000
O	45.894349000	44.405726000	28.730865000
O	49.979294000	52.157002000	35.832243000
O	47.557250000	52.890179000	36.654255000
O	46.632101000	51.940786000	39.033823000
O	44.985963000	52.210885000	36.958715000
O	48.572015000	40.308674000	30.040567000
O	45.413220000	42.227225000	30.232875000
O	47.905352000	42.818889000	29.526289000
O	50.435957000	42.206532000	30.097653000
O	51.663255000	44.404066000	31.015605000
O	58.602296000	44.406469000	39.358013000
O	58.126854000	47.823967000	35.378688000
O	58.303425000	46.463270000	37.671902000
O	58.214354000	49.179187000	37.701214000
O	57.004703000	48.622630000	33.128628000
O	56.916666000	50.213774000	35.305521000
O	54.355213000	48.573812000	32.574432000
O	52.568150000	46.750157000	31.791226000
O	54.996422000	52.150896000	35.417666000
O	51.813046000	51.946767000	33.878147000
O	52.529879000	52.509521000	36.403058000
O	50.191948000	50.679898000	30.094282000
O	52.233287000	51.987236000	31.229024000
O	52.304506000	49.264300000	30.988696000
O	54.574464000	51.921582000	38.072355000
O	56.175358000	50.094547000	39.126665000
O	50.434733000	46.604960000	30.111729000
O	58.124432000	40.985805000	35.382754000
O	58.308743000	42.340710000	37.682401000
O	58.225715000	39.623607000	37.699523000
O	57.000515000	40.189682000	33.133598000
O	54.354088000	40.239074000	32.573062000
O	52.560795000	42.059610000	31.794670000
O	50.290681000	40.898459000	48.874078000
O	48.421700000	42.353136000	47.685728000
O	48.223811000	39.617454000	47.684052000
O	42.515649000	50.761763000	43.408034000
O	45.207051000	50.699121000	43.748511000
O	48.532066000	50.122258000	45.186597000
O	47.639372000	51.073860000	42.795728000
O	45.567429000	52.202586000	41.589053000
O	44.956715000	44.396779000	46.831289000
O	46.383308000	46.520005000	45.987808000
O	48.101350000	44.407423000	45.989932000
O	50.292453000	47.926002000	48.887274000
O	48.467504000	46.508095000	47.608937000
O	48.235709000	49.218043000	47.692334000
O	46.404903000	42.296920000	45.981800000
O	58.647563000	41.939578000	40.318434000
O	57.936837000	42.731307000	42.898369000
O	55.905433000	44.395344000	43.440683000
O	56.002640000	46.719324000	44.679294000
O	54.232996000	48.556822000	45.637351000
O	56.169997000	49.265028000	43.900117000

O	51.788274000	48.542474000	46.707006000
O	50.138686000	50.996043000	43.235634000
O	52.425082000	50.867733000	41.965402000
O	52.071478000	49.397192000	44.224480000
O	53.579282000	50.051107000	39.708073000
O	55.086029000	50.685361000	41.851348000
O	58.672105000	46.884012000	40.297881000
O	57.922710000	46.066491000	42.849661000
O	55.960522000	42.072072000	44.675617000
O	54.265661000	40.184986000	45.710085000
O	56.162361000	39.541051000	43.893421000
O	51.786749000	40.250997000	46.710857000
H	48.707157000	34.670921000	34.766404000
H	44.424161000	34.670675000	35.340667000
H	42.687913000	36.253327000	36.015997000
H	43.730809000	38.674573000	32.571781000
H	47.734878000	38.005349000	29.824408000
H	52.425421000	37.996733000	29.024219000
H	58.469638000	38.737714000	40.060806000
H	58.092666000	37.102861000	38.280034000
H	56.655344000	36.753194000	33.552517000
H	57.545865000	36.241346000	35.776306000
H	52.145518000	34.668275000	32.555883000
H	54.075321000	36.140450000	32.815276000
H	54.422366000	34.669831000	36.911733000
H	44.269666000	36.143087000	39.488067000
H	46.089328000	34.671667000	40.190277000
H	42.181588000	36.880545000	41.130328000
H	40.294482000	38.101364000	42.084210000
H	43.618979000	38.683926000	45.481084000
H	46.271042000	38.588955000	46.374701000
H	48.069656000	37.107421000	47.125110000
H	52.199536000	36.875836000	44.265572000
H	55.862578000	36.983192000	44.005663000
H	57.533841000	37.959788000	42.513090000
H	41.640780000	38.774509000	36.628334000
H	40.342545000	40.839589000	36.811635000
H	43.730580000	50.134868000	32.571911000
H	47.734785000	50.804275000	29.824392000
H	42.915258000	46.065944000	29.963522000
H	43.640296000	48.340852000	30.468154000
H	46.059803000	46.685165000	27.773209000
H	40.342492000	47.969946000	36.811639000
H	41.640698000	50.035042000	36.628305000
H	48.706998000	54.138729000	34.766374000
H	44.423866000	54.138957000	35.340791000
H	42.687857000	52.555965000	36.016116000
H	43.640354000	40.468714000	30.468167000
H	42.915283000	42.743607000	29.963524000
H	46.059836000	42.124430000	27.773214000
H	52.772525000	42.800253000	29.332986000
H	60.156468000	47.945105000	36.762952000
H	56.119992000	49.270952000	30.922611000
H	56.053704000	46.898810000	31.500923000
H	57.545746000	52.568434000	35.776288000
H	56.655234000	52.056568000	33.552498000
H	52.145372000	54.141419000	32.555861000
H	54.075199000	52.669278000	32.815260000

H	54.422423000	54.139878000	36.911653000
H	52.425327000	50.812957000	29.024204000
H	58.469551000	50.072088000	40.060794000
H	58.092561000	51.706935000	38.280022000
H	52.772502000	46.009444000	29.332983000
H	60.156519000	40.864719000	36.762961000
H	56.053744000	41.910939000	31.500930000
H	56.120063000	39.538799000	30.922621000
H	48.032229000	40.952784000	49.813817000
H	40.294399000	50.708023000	42.084420000
H	42.181128000	51.929169000	41.130365000
H	43.619299000	50.126080000	45.481056000
H	48.069508000	51.702267000	47.125073000
H	46.270965000	50.220670000	46.374665000
H	40.480648000	46.418163000	42.223368000
H	44.582317000	46.680082000	47.779356000
H	48.032150000	47.856884000	49.813802000
H	46.089018000	54.138019000	40.190171000
H	44.269534000	52.666528000	39.488020000
H	40.480679000	42.391393000	42.223381000
H	44.582341000	42.129525000	47.779353000
H	58.823077000	40.402918000	42.215637000
H	60.313953000	42.339899000	42.249002000
H	56.729518000	48.543255000	46.268028000
H	51.675960000	50.040107000	48.763586000
H	52.725953000	47.843094000	49.001435000
H	52.199428000	51.933884000	44.265559000
H	55.862462000	51.826580000	44.005649000
H	57.533747000	50.850016000	42.513079000
H	60.313923000	46.469939000	42.248999000
H	58.823017000	48.406896000	42.215629000
H	56.729459000	40.266631000	46.268153000
H	52.726516000	40.966315000	49.000806000
H	51.676096000	38.769618000	48.763657000
Si	48.767209000	40.932767000	35.409317000
Si	46.855546000	39.962845000	37.441799000
Si	41.586237000	40.153798000	37.172841000
Si	44.280006000	40.875458000	35.853461000
Si	44.616684000	45.926564000	33.462423000
Si	47.802266000	45.935719000	33.228432000
Si	48.770161000	47.879864000	35.423117000
Si	46.838608000	48.774624000	37.472193000
Si	41.565435000	48.672787000	37.178417000
Si	44.247613000	47.920196000	35.864507000
Si	44.638786000	42.873196000	33.454558000
Si	47.810285000	42.879832000	33.223649000
Si	54.815237000	42.895193000	38.822384000
Si	51.698651000	48.535723000	35.163639000
Si	54.403742000	47.946316000	36.437108000
Si	54.828567000	45.929252000	38.824286000
Si	51.689186000	40.272518000	35.154031000
Si	54.392015000	40.867855000	36.437140000
Si	45.757616000	40.418592000	40.188940000
Si	42.674802000	40.442494000	40.037396000
Si	41.758368000	45.941330000	41.676175000
Si	43.967246000	46.727541000	43.796508000
Si	48.972999000	47.061282000	43.319647000
Si	46.339334000	45.979049000	41.944188000

Si	45.642154000	48.519293000	40.226875000
Si	42.599163000	48.388719000	40.008360000
Si	41.769469000	42.883033000	41.736025000
Si	43.900397000	42.071768000	43.818321000
Si	49.025703000	41.800118000	43.420627000
Al	46.448670000	42.856201000	41.988010000
Si	51.919931000	42.839167000	43.558138000
Si	53.989850000	41.864191000	41.530784000
Si	51.839596000	45.911824000	43.491144000
Si	53.957395000	46.939556000	41.525751000
O	46.897877000	38.441537000	36.812206000
O	44.177478000	39.511108000	34.943945000
O	54.384653000	39.437214000	37.241954000
O	51.591423000	38.763357000	35.794515000
O	42.279201000	39.447221000	41.259048000
O	50.373286000	41.179632000	35.491925000
O	48.201089000	40.754071000	36.935119000
O	48.058228000	42.234427000	34.708791000
O	48.443412000	39.580717000	34.552204000
O	46.835886000	39.883518000	39.062071000
O	45.539072000	40.770175000	36.890853000
O	41.652859000	40.067346000	38.816101000
O	42.860205000	41.069409000	36.632072000
O	44.533994000	42.197006000	34.926987000
O	44.136735000	49.286402000	34.956345000
O	43.729962000	46.746446000	32.353199000
O	44.052805000	44.396780000	33.537736000
O	46.186512000	45.930358000	32.984524000
O	48.393637000	44.410661000	33.198943000
O	48.572194000	46.793656000	32.063544000
O	50.376274000	47.643660000	35.520813000
O	48.194027000	48.013611000	36.954175000
O	48.069536000	46.583129000	34.711525000
O	48.443890000	49.245994000	34.589596000
O	46.809395000	48.760381000	39.103161000
O	46.882556000	50.319264000	36.904580000
O	45.526932000	48.005743000	36.876502000
O	41.574164000	48.832167000	38.820295000
O	42.857337000	47.750413000	36.702875000
O	44.458722000	46.597462000	34.934758000
O	43.739551000	42.054380000	32.354829000
O	46.199303000	42.898630000	32.946837000
O	48.592489000	42.028652000	32.061743000
O	54.232420000	44.415832000	38.635227000
O	56.352566000	42.957295000	39.373295000
O	55.465887000	47.979083000	35.200998000
O	51.855485000	48.678621000	33.537744000
O	52.920525000	47.678982000	35.818571000
O	51.609389000	50.050192000	35.794325000
O	54.817709000	46.697837000	37.393194000
O	54.392109000	49.377866000	37.241072000
O	56.360479000	45.870736000	39.392692000
O	55.466367000	40.841365000	35.210585000
O	51.855387000	40.114488000	33.529689000
O	52.914549000	41.127980000	35.804642000
O	54.785951000	42.123266000	37.394274000
O	46.064651000	41.966688000	40.533144000
O	44.234094000	40.198272000	39.574662000

O	45.938731000	39.479419000	41.516871000
O	42.457671000	41.994161000	40.526246000
O	42.235290000	49.311534000	41.295481000
O	43.908443000	48.361252000	43.584437000
O	48.794070000	48.656224000	42.987695000
O	42.779600000	46.008406000	42.958949000
O	41.608925000	44.405878000	41.125967000
O	43.790417000	46.390205000	45.390639000
O	45.397932000	46.068949000	43.280917000
O	48.791272000	46.768242000	44.926472000
O	47.885644000	46.260857000	42.387437000
O	46.305190000	44.502223000	41.177623000
O	45.744615000	47.011704000	40.832842000
O	44.155645000	48.698487000	39.541286000
O	45.864800000	49.566361000	41.444515000
O	42.378492000	46.817391000	40.414559000
O	43.647815000	40.455071000	43.596450000
O	48.693725000	40.231247000	43.041015000
O	42.699481000	42.910197000	43.081246000
O	43.768781000	42.383460000	45.433885000
O	45.318646000	42.584606000	43.256097000
O	48.910567000	42.016063000	45.063881000
O	48.066438000	42.802943000	42.604949000
O	53.574727000	40.325365000	41.879432000
O	50.597667000	42.063122000	43.008091000
O	53.007430000	42.900347000	42.323886000
O	51.588088000	44.382028000	44.018092000
O	53.886110000	42.087200000	39.906752000
O	55.536037000	42.129841000	42.014546000
O	52.401124000	46.799885000	44.754927000
O	53.543124000	48.477540000	41.892714000
O	50.465562000	46.563920000	42.867990000
O	52.935232000	45.901352000	42.265535000
O	53.894930000	46.752704000	39.895541000
O	55.487147000	46.662571000	42.042555000
O	52.556942000	42.023676000	44.835665000
Ga	46.346084000	44.288111000	39.189536000
H	44.974661000	43.961395000	38.529297000
C	48.174372000	43.590542000	38.462724000
H	47.844757000	43.067821000	37.550119000
C	49.219738000	42.803788000	39.272695000
H	49.786223000	42.204005000	38.531971000
H	48.748604000	42.062596000	39.943165000
C	48.344855000	45.009362000	38.246603000
C	49.379333000	45.803234000	39.019843000
H	46.867607000	45.842792000	38.758421000
H	48.075076000	45.400734000	37.254211000
H	49.614087000	46.732128000	38.475127000
H	48.994825000	46.109541000	40.009589000
C	50.210396000	43.683376000	40.050987000
C	50.618409000	44.920197000	39.251804000
H	51.098407000	43.077930000	40.305252000
H	49.764907000	44.007869000	41.012725000
H	51.381684000	45.508242000	39.792245000
H	51.073440000	44.630408000	38.281455000

I13-GaH$_2$

Electronic energy (r^{2}SCAN-D4/def2-SVP): -48413.07886951 E_h
Electronic energy (ωB97X-D4rev/def2-mTZVPP): -48457.178544420000 E_h
G_{corr} (673.15 K, 1 atm): 2.05667430 E_h
Number of atoms: 395

Cartesian coordinates (Å)

Si	48.561604000	36.033400000	35.302958000
Si	46.526679000	36.974058000	37.345613000
Si	44.101249000	36.091976000	35.635381000
Si	44.615990000	38.513933000	33.740329000
Si	47.773720000	38.547492000	33.560769000
Si	48.741095000	38.767085000	30.519067000
Si	51.805835000	38.124281000	30.332723000
Si	54.679416000	38.432345000	38.538166000
Si	57.776788000	38.492849000	38.802603000
Si	56.563801000	37.025140000	34.996832000
Si	52.598489000	36.084575000	32.662648000
Si	51.489242000	37.168691000	35.484292000
Si	54.139263000	36.092479000	36.721720000
Si	45.622784000	36.074617000	40.028477000
Si	41.745134000	38.019517000	41.957357000
Si	43.617166000	38.884101000	44.080725000
Si	47.724547000	38.465424000	46.617698000
Si	48.752474000	38.685476000	43.586388000
Si	46.054931000	38.203210000	42.308851000
Si	51.724673000	38.009162000	43.471626000
Si	53.653350000	38.811771000	41.335198000
Si	56.180899000	38.116163000	43.088643000
Si	44.615655000	50.309485000	33.739884000
Si	47.769689000	50.274146000	33.571493000
Si	48.739917000	50.060841000	30.531054000
Si	43.866067000	46.935685000	30.715205000
Si	48.871058000	46.956122000	30.437191000
Si	46.324170000	45.964961000	29.027572000
Si	48.561689000	52.791820000	35.321053000
Si	46.532192000	51.842278000	37.359810000
Si	44.103938000	52.728104000	35.637588000
Si	43.866513000	41.886804000	30.712592000
Si	48.868051000	41.865053000	30.426934000
Si	46.323739000	42.858473000	29.025535000
Si	51.889624000	42.864815000	30.529854000
Si	58.023997000	42.908870000	39.195366000
Si	58.734416000	47.847078000	36.906673000
Si	56.882099000	48.682721000	34.750179000
Si	55.876141000	48.297488000	31.971210000
Si	52.773381000	48.324601000	32.246677000
Si	56.564602000	51.797648000	34.996249000
Si	52.598786000	52.736997000	32.663597000
Si	51.491302000	51.655423000	35.488543000
Si	54.144373000	52.728972000	36.723320000
Si	51.803291000	50.697633000	30.333633000
Si	54.684529000	50.395270000	38.532250000
Si	57.779570000	50.326268000	38.799959000
Si	51.888820000	45.955527000	30.528524000
Si	58.032319000	45.926568000	39.186607000
Si	58.735291000	40.974670000	36.914954000

Si	56.883398000	40.143971000	34.756131000
Si	55.880611000	40.524157000	31.972884000
Si	52.779550000	40.494235000	32.243108000
Si	48.692429000	40.947497000	48.523707000
Si	41.746021000	50.775046000	41.944546000
Si	43.709788000	49.977290000	44.077557000
Si	47.724901000	50.353640000	46.615673000
Si	48.759935000	50.130498000	43.582989000
Si	46.081306000	50.770287000	42.364477000
Si	44.902054000	46.015074000	46.537997000
Si	47.941698000	46.027126000	46.130322000
Si	48.697068000	47.880364000	48.519745000
Si	45.619358000	52.769238000	40.029152000
Si	44.899552000	42.799366000	46.535020000
Si	47.951777000	42.779787000	46.151984000
Si	58.968328000	41.836850000	41.946431000
Si	56.332446000	42.831564000	43.258772000
Si	55.803109000	48.267880000	45.172604000
Si	52.636657000	48.321304000	45.343876000
Si	51.676289000	48.632199000	48.361423000
Si	51.748366000	50.813834000	43.466788000
Si	53.660521000	50.020169000	41.327437000
Si	56.179695000	50.701133000	43.091679000
Si	58.975130000	46.986540000	41.920625000
Si	56.338326000	45.963008000	43.243948000
Si	55.794576000	40.519534000	45.175820000
Si	52.662317000	40.468708000	45.360112000
Si	51.673903000	40.189447000	48.363788000
O	49.986382000	36.639097000	35.855256000
O	47.521294000	35.921119000	36.579875000
O	46.665066000	36.834628000	38.970166000
O	44.969360000	36.641408000	36.938821000
O	44.496116000	36.957136000	34.272080000
O	46.174351000	38.879235000	33.317938000
O	47.928040000	37.016096000	34.134251000
O	48.580489000	38.675356000	32.149284000
O	50.185943000	38.132121000	30.096116000
O	52.221912000	36.832240000	31.245903000
O	52.296313000	39.551278000	30.995053000
O	54.609383000	36.851020000	38.105104000
O	56.171410000	38.724393000	39.136264000
O	56.948239000	38.599594000	35.303350000
O	55.000411000	36.677229000	35.434635000
O	51.818417000	36.865135000	33.903062000
O	52.540388000	36.340704000	36.435727000
O	42.424807000	37.927993000	43.459387000
O	45.103655000	38.351106000	43.621427000
O	48.537703000	38.692516000	45.202277000
O	47.572398000	37.872656000	42.794558000
O	45.553868000	36.920650000	41.436645000
O	50.115928000	37.861463000	43.240266000
O	52.409758000	37.968343000	41.973015000
O	52.097387000	39.427051000	44.228115000
O	53.571189000	38.739778000	39.700276000
O	55.065632000	38.162639000	41.867678000
O	44.496003000	51.866735000	34.271633000
O	46.172863000	49.948387000	33.309519000
O	47.920982000	51.797935000	34.165871000

O	48.586869000	50.166687000	32.162881000
O	48.591616000	48.508486000	30.007749000
O	45.411103000	46.591042000	30.242347000
O	47.899864000	45.996444000	29.521603000
O	45.875627000	44.411858000	28.739278000
O	49.993224000	52.194336000	35.863735000
O	47.533701000	52.903092000	36.610433000
O	46.648742000	51.966982000	38.991675000
O	44.978583000	52.179883000	36.939138000
O	48.594210000	40.315450000	29.982924000
O	45.413158000	42.231256000	30.242521000
O	47.901557000	42.829946000	29.512180000
O	50.429121000	42.229882000	30.119734000
O	51.681778000	44.410451000	31.035821000
O	58.645606000	44.417033000	39.382054000
O	58.105192000	47.811721000	35.388001000
O	58.326517000	46.446320000	37.665743000
O	58.210036000	49.171589000	37.710571000
O	57.011315000	48.625803000	33.127251000
O	56.951581000	50.224377000	35.303755000
O	54.355824000	48.586532000	32.568376000
O	52.564183000	46.761050000	31.798405000
O	55.001219000	52.145308000	35.433472000
O	51.821661000	51.958993000	33.907758000
O	52.545911000	52.472682000	36.444833000
O	50.183302000	50.692954000	30.096992000
O	52.221034000	51.988920000	31.247535000
O	52.285980000	49.271100000	31.002842000
O	54.625176000	51.979494000	38.108433000
O	56.173557000	50.087865000	39.128870000
O	50.429949000	46.592660000	30.115147000
O	58.107231000	41.013371000	35.395685000
O	58.335412000	42.372138000	37.683741000
O	58.203884000	39.650213000	37.714025000
O	57.013491000	40.201961000	33.132594000
O	54.361053000	40.235815000	32.570914000
O	52.565998000	42.058813000	31.798886000
O	50.288490000	40.900449000	48.878558000
O	48.426719000	42.328281000	47.655835000
O	48.223826000	39.605324000	47.699219000
O	42.465921000	50.841793000	43.431221000
O	45.164961000	50.649919000	43.707931000
O	48.529194000	50.081424000	45.197153000
O	47.623006000	51.020004000	42.812764000
O	45.574198000	52.052099000	41.506150000
O	44.931361000	44.411411000	46.845882000
O	46.375162000	46.514856000	45.987411000
O	48.082651000	44.406680000	46.006238000
O	50.289618000	47.929181000	48.885924000
O	48.462864000	46.524092000	47.602372000
O	48.223760000	49.233119000	47.715292000
O	46.380155000	42.315622000	45.986911000
O	58.707322000	41.931821000	40.308424000
O	57.927314000	42.736665000	42.876064000
O	55.896281000	44.399742000	43.410792000
O	56.052691000	46.710382000	44.681181000
O	54.243729000	48.501333000	45.662177000
O	56.155714000	49.267960000	43.914069000

O	51.789293000	48.574884000	46.712240000
O	50.137900000	50.922431000	43.245554000
O	52.425268000	50.876809000	41.967248000
O	52.138043000	49.407495000	44.225773000
O	53.574316000	50.089235000	39.693529000
O	55.077486000	50.658617000	41.858305000
O	58.742864000	46.913814000	40.276428000
O	57.915303000	46.057341000	42.796721000
O	55.969926000	42.087780000	44.681082000
O	54.257208000	40.186109000	45.676626000
O	56.187882000	39.552982000	43.904357000
O	51.790998000	40.242732000	46.716353000
H	48.702873000	34.676576000	34.772542000
H	44.419876000	34.676332000	35.346799000
H	42.683629000	36.258984000	36.022128000
H	43.726525000	38.680237000	32.577925000
H	47.730604000	38.011010000	29.830549000
H	52.421148000	38.002393000	29.030365000
H	58.465351000	38.743357000	40.066960000
H	58.088380000	37.108506000	38.286186000
H	56.651065000	36.758844000	33.558667000
H	57.541583000	36.246995000	35.782457000
H	52.141237000	34.673930000	32.562026000
H	54.071042000	36.146103000	32.821423000
H	54.418081000	34.675479000	36.917878000
H	44.265378000	36.148739000	39.494203000
H	46.085037000	34.677317000	40.196414000
H	42.177300000	36.886196000	41.136462000
H	40.290188000	38.107007000	42.090349000
H	43.614685000	38.689560000	45.487224000
H	46.266748000	38.594602000	46.380841000
H	48.065359000	37.113066000	47.131252000
H	52.195242000	36.881479000	44.271718000
H	55.858286000	36.988834000	44.011813000
H	57.529551000	37.965430000	42.519241000
H	41.636485000	38.780156000	36.634489000
H	40.338251000	40.845237000	36.817790000
H	43.726308000	50.140527000	32.578064000
H	47.730521000	50.809934000	29.830545000
H	42.910989000	46.071613000	29.969662000
H	43.636028000	48.346514000	30.474314000
H	46.055535000	46.690830000	27.779359000
H	40.338196000	47.975594000	36.817795000
H	41.636407000	50.040687000	36.634465000
H	48.702728000	54.144384000	34.772530000
H	44.419600000	54.144615000	35.346939000
H	42.683587000	52.561628000	36.022264000
H	43.636079000	40.474377000	30.474304000
H	42.911008000	42.749270000	29.969672000
H	46.055566000	42.130092000	27.779360000
H	52.768253000	42.805907000	29.339137000
H	60.152191000	47.950746000	36.769113000
H	56.115724000	49.276607000	30.928775000
H	56.049432000	46.904463000	31.507081000
H	57.541477000	52.574078000	35.782454000
H	56.650967000	52.062217000	33.558662000
H	52.141107000	54.147073000	32.562022000
H	54.070933000	52.674931000	32.821422000

H	54.418152000	54.145526000	36.917817000
H	52.421065000	50.818613000	29.030362000
H	58.465273000	50.077730000	40.066959000
H	58.088286000	51.712579000	38.286187000
H	52.768232000	46.015103000	29.339136000
H	60.152236000	40.870365000	36.769117000
H	56.049468000	41.916591000	31.507084000
H	56.115786000	39.544450000	30.928775000
H	48.027934000	40.958425000	49.819961000
H	40.290117000	50.713692000	42.090567000
H	42.176858000	51.934815000	41.136505000
H	43.615012000	50.131735000	45.487203000
H	48.065224000	51.707912000	47.131227000
H	46.266681000	50.226317000	46.380815000
H	40.476351000	46.423810000	42.229526000
H	44.578030000	46.685726000	47.785507000
H	48.027861000	47.862528000	49.819952000
H	46.084746000	54.143671000	40.196326000
H	44.265260000	52.672184000	39.494170000
H	40.476381000	42.397038000	42.229538000
H	44.578054000	42.135172000	47.785502000
H	58.818790000	40.408559000	42.221792000
H	60.309665000	42.345541000	42.255160000
H	56.725232000	48.548892000	46.274189000
H	51.671674000	50.045748000	48.769743000
H	52.721664000	47.848733000	49.007590000
H	52.195145000	51.939528000	44.271718000
H	55.858181000	51.832221000	44.011811000
H	57.529468000	50.855657000	42.519242000
H	60.309638000	46.475575000	42.255161000
H	58.818736000	48.412536000	42.221791000
H	56.725166000	40.272269000	46.274306000
H	52.722222000	40.971955000	49.006954000
H	51.671800000	38.775258000	48.769806000
Si	48.786506000	40.995235000	35.423118000
Si	46.868281000	40.041206000	37.426374000
Si	41.565334000	40.142087000	37.195353000
Si	44.248606000	40.884611000	35.863995000
Si	44.615329000	45.942336000	33.466147000
Si	47.795456000	45.931438000	33.204506000
Si	48.770323000	47.818321000	35.440019000
Si	46.864869000	48.782306000	37.458912000
Si	41.570107000	48.674857000	37.181375000
Si	44.259113000	47.942894000	35.870972000
Si	44.598859000	42.891220000	33.465413000
Si	47.784043000	42.879514000	33.197198000
Si	54.894968000	42.904540000	38.801113000
Si	51.691321000	48.529593000	35.185274000
Si	54.411871000	48.025371000	36.448692000
Si	54.908754000	45.951300000	38.803619000
Si	51.700195000	40.290375000	35.177166000
Si	54.410574000	40.812394000	36.454140000
Si	45.675932000	40.493270000	40.134166000
Si	42.607489000	40.406425000	40.039270000
Si	41.751072000	45.937909000	41.687307000
Si	43.910429000	46.788607000	43.795649000
Si	48.945726000	47.004051000	43.291833000
Si	46.310820000	45.980855000	41.931838000

Si	45.694731000	48.446367000	40.203957000
Si	42.617873000	48.399620000	40.028171000
Si	41.755275000	42.894354000	41.727494000
Si	43.845633000	42.021873000	43.834737000
Si	49.036721000	41.802762000	43.370678000
Al	46.396670000	42.802980000	42.054061000
Si	51.919154000	42.866397000	43.570092000
Si	53.971877000	41.917203000	41.517434000
Si	51.823046000	45.916499000	43.507903000
Si	53.939365000	46.915990000	41.515703000
O	46.898312000	38.499321000	36.854569000
O	44.136264000	39.512774000	34.967066000
O	54.384487000	39.357512000	37.216526000
O	51.572211000	38.777965000	35.807341000
O	42.330575000	39.403615000	41.282783000
O	50.393606000	41.215133000	35.482652000
O	48.216129000	40.830092000	36.951505000
O	48.058558000	42.257714000	34.682744000
O	48.446869000	39.615573000	34.596777000
O	46.815391000	40.033393000	39.056299000
O	45.542137000	40.825051000	36.864398000
O	41.577634000	39.995046000	38.841500000
O	42.866909000	41.054328000	36.721189000
O	44.432446000	42.208828000	34.931123000
O	44.145091000	49.314571000	34.971947000
O	43.723297000	46.759678000	32.358895000
O	44.029817000	44.419760000	33.555823000
O	46.176966000	45.924380000	32.969462000
O	48.379825000	44.403593000	33.109618000
O	48.554289000	46.817837000	32.051815000
O	50.382011000	47.612338000	35.508761000
O	48.194816000	47.969097000	36.968866000
O	48.071433000	46.539532000	34.697242000
O	48.426019000	49.188756000	34.601769000
O	46.826871000	48.828539000	39.090883000
O	46.918030000	50.321250000	36.871357000
O	45.521536000	48.028222000	36.904921000
O	41.604940000	48.829650000	38.825228000
O	42.857821000	47.754572000	36.691600000
O	44.481871000	46.627163000	34.934125000
O	43.712301000	42.075962000	32.353020000
O	46.163308000	42.897774000	32.978011000
O	48.532984000	41.993743000	32.038157000
O	54.437430000	44.430438000	38.425856000
O	56.405601000	42.945528000	39.425082000
O	55.441533000	48.036319000	35.184242000
O	51.877448000	48.680955000	33.563109000
O	52.922449000	47.702588000	35.871301000
O	51.562843000	50.044937000	35.806516000
O	54.911268000	46.848617000	37.451036000
O	54.378554000	49.485224000	37.202572000
O	56.415492000	45.908552000	39.441215000
O	55.448811000	40.798323000	35.196534000
O	51.885767000	40.125511000	33.556236000
O	52.925303000	41.123990000	35.862932000
O	54.884536000	42.005051000	37.450575000
O	45.834859000	42.112539000	40.464447000
O	44.169823000	40.219643000	39.527731000

O	45.948529000	39.637094000	41.496446000
O	42.357966000	41.952940000	40.511242000
O	42.255951000	49.348579000	41.297295000
O	43.675740000	48.398029000	43.601335000
O	48.742207000	48.601077000	42.979527000
O	42.795590000	45.951941000	42.956565000
O	41.548330000	44.410771000	41.119926000
O	43.788431000	46.398022000	45.382680000
O	45.401252000	46.359459000	43.238777000
O	48.777994000	46.733812000	44.906635000
O	47.876048000	46.153706000	42.372774000
O	46.019072000	44.442903000	41.397507000
O	45.877340000	46.869328000	40.620856000
O	44.185939000	48.661905000	39.580873000
O	45.917450000	49.366667000	41.529460000
O	42.382269000	46.830767000	40.446288000
O	43.359961000	40.456866000	43.677433000
O	48.769491000	40.219129000	43.024995000
O	42.792483000	42.962693000	42.995094000
O	43.742240000	42.439965000	45.426205000
O	45.345894000	42.252634000	43.292259000
O	48.861177000	42.046903000	45.003225000
O	48.056075000	42.731622000	42.486098000
O	53.512372000	40.377106000	41.813826000
O	50.597419000	42.119861000	42.979605000
O	53.020352000	42.945839000	42.354432000
O	51.579002000	44.394167000	44.061805000
O	53.885656000	42.237945000	39.910263000
O	55.521165000	42.102851000	42.029090000
O	52.356978000	46.807811000	44.779111000
O	53.496592000	48.460653000	41.816118000
O	50.452261000	46.542811000	42.856347000
O	52.937868000	45.897469000	42.305176000
O	53.897488000	46.630388000	39.901810000
O	55.468984000	46.701996000	42.064264000
O	52.526124000	42.022365000	44.842624000
Ga	45.261918000	43.899583000	39.577484000
H	43.717892000	44.053174000	39.676557000
C	48.905062000	44.014329000	37.524002000
H	47.954861000	43.937945000	36.974717000
C	50.011330000	44.409024000	36.866611000
H	46.262901000	44.208690000	38.426081000
H	49.954170000	44.611746000	35.786658000
C	48.900079000	43.647163000	38.987356000
C	50.313465000	43.470298000	39.547093000
H	48.306941000	42.722454000	39.135336000
H	48.376084000	44.437832000	39.568748000
H	50.278450000	43.424199000	40.650223000
H	50.734072000	42.502444000	39.200329000
C	51.346763000	44.587261000	37.538474000
C	51.211068000	44.612600000	39.068076000
H	51.824154000	45.515401000	37.165299000
H	52.025638000	43.765996000	37.218604000
H	52.197228000	44.554992000	39.554954000
H	50.763804000	45.580737000	39.379912000

TS9-GaH$_2$

Electronic energy (r^{2}SCAN-D4/def2-SVP): -48412.99984355 E_h
Electronic energy (ωB97X-D4rev/def2-mTZVPP): -48457.101216689900 E_h
G_{corr} (673.15 K, 1 atm): 2.04755947 E_h
Number of atoms: 395

Cartesian coordinates (Å)

Si	48.555722000	36.015224000	35.318853000
Si	46.517986000	36.911862000	37.341428000
Si	44.095616000	36.082279000	35.631349000
Si	44.635974000	38.491748000	33.714645000
Si	47.777804000	38.488619000	33.528221000
Si	48.747738000	38.755748000	30.511487000
Si	51.804656000	38.130676000	30.325768000
Si	54.678093000	38.466896000	38.533009000
Si	57.777707000	38.490333000	38.802809000
Si	56.553628000	37.019715000	34.996751000
Si	52.594553000	36.085833000	32.664701000
Si	51.479286000	37.163008000	35.489811000
Si	54.132732000	36.093270000	36.714883000
Si	45.623074000	36.064469000	40.035785000
Si	41.750662000	38.040348000	41.931147000
Si	43.600925000	38.890709000	44.078115000
Si	47.727812000	38.474359000	46.634998000
Si	48.750231000	38.709555000	43.632358000
Si	46.082854000	38.129108000	42.364106000
Si	51.735057000	38.015531000	43.471585000
Si	53.670830000	38.777595000	41.342598000
Si	56.183707000	38.108451000	43.086971000
Si	44.606968000	50.296599000	33.752822000
Si	47.752341000	50.299997000	33.580057000
Si	48.733800000	50.064901000	30.543943000
Si	43.864553000	46.934078000	30.716764000
Si	48.864111000	46.959043000	30.447086000
Si	46.323493000	45.967061000	29.030114000
Si	48.543819000	52.792152000	35.329843000
Si	46.523718000	51.828768000	37.401043000
Si	44.107569000	52.727900000	35.650003000
Si	43.868969000	41.884158000	30.710177000
Si	48.880017000	41.874566000	30.413301000
Si	46.323670000	42.859787000	29.020845000
Si	51.878166000	42.863258000	30.516032000
Si	57.975896000	42.901170000	39.185252000
Si	58.733962000	47.855333000	36.910965000
Si	56.877261000	48.675461000	34.759205000
Si	55.872143000	48.297014000	31.974271000
Si	52.771104000	48.319112000	32.239545000
Si	56.557270000	51.798545000	34.996777000
Si	52.599852000	52.737097000	32.653994000
Si	51.485610000	51.659095000	35.467243000
Si	54.127436000	52.729325000	36.712327000
Si	51.807785000	50.693072000	30.332350000
Si	54.671725000	50.355035000	38.536676000
Si	57.775419000	50.331396000	38.803242000
Si	51.879596000	45.956553000	30.523535000
Si	57.977323000	45.920266000	39.183226000
Si	58.734970000	40.962937000	36.910634000

Si	56.868654000	40.143772000	34.762125000
Si	55.871484000	40.524583000	31.972697000
Si	52.764626000	40.499008000	32.234876000
Si	48.682076000	40.969886000	48.532585000
Si	41.750984000	50.763441000	41.948880000
Si	43.768729000	49.963203000	44.082405000
Si	47.723155000	50.360223000	46.615702000
Si	48.774301000	50.210351000	43.583706000
Si	46.075568000	50.880658000	42.391072000
Si	44.896857000	46.008834000	46.545028000
Si	47.950599000	46.045713000	46.125408000
Si	48.699457000	47.885462000	48.520666000
Si	45.605948000	52.786928000	40.052889000
Si	44.933144000	42.793674000	46.530774000
Si	47.984579000	42.807805000	46.201531000
Si	58.959674000	41.840222000	41.943903000
Si	56.324591000	42.838698000	43.263597000
Si	55.785928000	48.290455000	45.172310000
Si	52.615684000	48.333909000	45.330086000
Si	51.675254000	48.630767000	48.362732000
Si	51.730521000	50.832528000	43.466481000
Si	53.662043000	50.025803000	41.347637000
Si	56.182233000	50.708210000	43.091078000
Si	58.960714000	46.980679000	41.937288000
Si	56.326369000	45.968701000	43.262692000
Si	55.797181000	40.515227000	45.174820000
Si	52.678715000	40.474882000	45.357681000
Si	51.673765000	40.192936000	48.355448000
O	49.970481000	36.650187000	35.865833000
O	47.523018000	35.854196000	36.598687000
O	46.659335000	36.826055000	38.969053000
O	44.956775000	36.587569000	36.955878000
O	44.507881000	36.945245000	34.272558000
O	46.188797000	38.834880000	33.251193000
O	47.898482000	36.975350000	34.146707000
O	48.621307000	38.583600000	32.137469000
O	50.192213000	38.154922000	30.045992000
O	52.200821000	36.845807000	31.258178000
O	52.298776000	39.560385000	30.978266000
O	54.610835000	36.893215000	38.070587000
O	56.174073000	38.743011000	39.129265000
O	56.902292000	38.600292000	35.315857000
O	54.987556000	36.654820000	35.413578000
O	51.810472000	36.851799000	33.911954000
O	52.529339000	36.333041000	36.441311000
O	42.406059000	37.949572000	43.445366000
O	45.080943000	38.332999000	43.625422000
O	48.602492000	38.696214000	45.254667000
O	47.580605000	37.814357000	42.914819000
O	45.594522000	36.853158000	41.476931000
O	50.126652000	37.944062000	43.213339000
O	52.429122000	37.931936000	41.978113000
O	52.131392000	39.421808000	44.230613000
O	53.572617000	38.737207000	39.708265000
O	55.087488000	38.119986000	41.848653000
O	44.497098000	51.851634000	34.292263000
O	46.165338000	49.934202000	33.320492000
O	47.871393000	51.831156000	34.162627000

O	48.573656000	50.185205000	32.174203000
O	48.573248000	48.511379000	30.028258000
O	45.411354000	46.591516000	30.246134000
O	47.898931000	45.997145000	29.527134000
O	45.875158000	44.413723000	28.741624000
O	49.977901000	52.168555000	35.836647000
O	47.546338000	52.870931000	36.646054000
O	46.631208000	51.948081000	39.036799000
O	44.982092000	52.197999000	36.959344000
O	48.566326000	40.317491000	30.032225000
O	45.411825000	42.221358000	30.228134000
O	47.903227000	42.829719000	29.502087000
O	50.437976000	42.210728000	30.060023000
O	51.648654000	44.408545000	31.015667000
O	58.595618000	44.410596000	39.364614000
O	58.124556000	47.829165000	35.384139000
O	58.302555000	46.465624000	37.676817000
O	58.203769000	49.180348000	37.709378000
O	57.000869000	48.627403000	33.135176000
O	56.914649000	50.219318000	35.311028000
O	54.351690000	48.574355000	32.580850000
O	52.560673000	46.752825000	31.797236000
O	54.993286000	52.156540000	35.423661000
O	51.811219000	51.950531000	33.884429000
O	52.527482000	52.508468000	36.410162000
O	50.185520000	50.680417000	30.114555000
O	52.231455000	51.990019000	31.235390000
O	52.308484000	49.265554000	30.986787000
O	54.574177000	51.927867000	38.078332000
O	56.170399000	50.101616000	39.136488000
O	50.425783000	46.610643000	30.118379000
O	58.115534000	40.990161000	35.387773000
O	58.302365000	42.348892000	37.681860000
O	58.224216000	39.629298000	37.705057000
O	56.994327000	40.190252000	33.138021000
O	54.347166000	40.252810000	32.569232000
O	52.532168000	42.064455000	31.802262000
O	50.285823000	40.915407000	48.846640000
O	48.369204000	42.386243000	47.739570000
O	48.188667000	39.637441000	47.707024000
O	42.504022000	50.789919000	43.420065000
O	45.197087000	50.714690000	43.754711000
O	48.525327000	50.124683000	45.191980000
O	47.631395000	51.081799000	42.806206000
O	45.560102000	52.192523000	41.584432000
O	44.929193000	44.403561000	46.851657000
O	46.375182000	46.507233000	46.006373000
O	48.140178000	44.426151000	45.995963000
O	50.289122000	47.937699000	48.900096000
O	48.475588000	46.530352000	47.601096000
O	48.231170000	49.233848000	47.705543000
O	46.418663000	42.349747000	45.950892000
O	58.660507000	41.937621000	40.311665000
O	57.917733000	42.739203000	42.873719000
O	55.894102000	44.404465000	43.440532000
O	55.994945000	46.725822000	44.683945000
O	54.222745000	48.565867000	45.632140000
O	56.171119000	49.270947000	43.907129000

O	51.783558000	48.552227000	46.713964000
O	50.130253000	51.043733000	43.256255000
O	52.410524000	50.871250000	41.967403000
O	52.052324000	49.408134000	44.227986000
O	53.573950000	50.058813000	39.713623000
O	55.073202000	50.687328000	41.863464000
O	58.668687000	46.887833000	40.303330000
O	57.913770000	46.073636000	42.853440000
O	55.971395000	42.081870000	44.679121000
O	54.267431000	40.191386000	45.697444000
O	56.171065000	39.550073000	43.895009000
O	51.799971000	40.255774000	46.709156000
H	48.702871000	34.676568000	34.772566000
H	44.419875000	34.676323000	35.346829000
H	42.683628000	36.258975000	36.022159000
H	43.726523000	38.680216000	32.577941000
H	47.730594000	38.010996000	29.830569000
H	52.421136000	38.002380000	29.030380000
H	58.465353000	38.743363000	40.066967000
H	58.088382000	37.108509000	38.286195000
H	56.651061000	36.758841000	33.558678000
H	57.541582000	36.246995000	35.782467000
H	52.141234000	34.673922000	32.562045000
H	54.071037000	36.146097000	32.821438000
H	54.418082000	34.675478000	36.917895000
H	44.265382000	36.148734000	39.494230000
H	46.085045000	34.677316000	40.196440000
H	42.177304000	36.886192000	41.136491000
H	40.290195000	38.107003000	42.090377000
H	43.614692000	38.689573000	45.487247000
H	46.266760000	38.594605000	46.380864000
H	48.065374000	37.113072000	47.131273000
H	52.195251000	36.881484000	44.271734000
H	55.858296000	36.988842000	44.011824000
H	57.529558000	37.965437000	42.519250000
H	41.636484000	38.780154000	36.634495000
H	40.338249000	40.845235000	36.817795000
H	43.726296000	50.140518000	32.578071000
H	47.730503000	50.809921000	29.830551000
H	42.910974000	46.071594000	29.969678000
H	43.636013000	48.346500000	30.474316000
H	46.055518000	46.690813000	27.779371000
H	40.338195000	47.975592000	36.817799000
H	41.636403000	50.040687000	36.634467000
H	48.702714000	54.144377000	34.772532000
H	44.419585000	54.144606000	35.346948000
H	42.683575000	52.561616000	36.022276000
H	43.636069000	40.474361000	30.474327000
H	42.910998000	42.749253000	29.969681000
H	46.055551000	42.130076000	27.779377000
H	52.768240000	42.805898000	29.339147000
H	60.152183000	47.950749000	36.769108000
H	56.115708000	49.276599000	30.928772000
H	56.049418000	46.904457000	31.507081000
H	57.541465000	52.574079000	35.782446000
H	56.650952000	52.062214000	33.558656000
H	52.141090000	54.147065000	32.562019000
H	54.070917000	52.674925000	32.821418000

H	54.418140000	54.145525000	36.917811000
H	52.421044000	50.818602000	29.030363000
H	58.465269000	50.077736000	40.066952000
H	58.088278000	51.712582000	38.286179000
H	52.768217000	46.015092000	29.339142000
H	60.152234000	40.870368000	36.769119000
H	56.049458000	41.916585000	31.507090000
H	56.115777000	39.544444000	30.928783000
H	48.027948000	40.958435000	49.819979000
H	40.290115000	50.713683000	42.090582000
H	42.176850000	51.934815000	41.136521000
H	43.615015000	50.131729000	45.487215000
H	48.065229000	51.707918000	47.131232000
H	46.266685000	50.226321000	46.380825000
H	40.476352000	46.423809000	42.229530000
H	44.578030000	46.685732000	47.785515000
H	48.027871000	47.862537000	49.819962000
H	46.084739000	54.143668000	40.196331000
H	44.265252000	52.672179000	39.494179000
H	40.476382000	42.397038000	42.229542000
H	44.578055000	42.135174000	47.785513000
H	58.818796000	40.408567000	42.221796000
H	60.309668000	42.345550000	42.255160000
H	56.725235000	48.548904000	46.274187000
H	51.671681000	50.045758000	48.769745000
H	52.721673000	47.848745000	49.007593000
H	52.195144000	51.939533000	44.271718000
H	55.858180000	51.832229000	44.011806000
H	57.529465000	50.855664000	42.519236000
H	60.309639000	46.475584000	42.255156000
H	58.818736000	48.412544000	42.221786000
H	56.725177000	40.272281000	46.274313000
H	52.722235000	40.971967000	49.006966000
H	51.671815000	38.775269000	48.769821000
Si	48.768835000	40.944493000	35.372594000
Si	46.841840000	39.935516000	37.376626000
Si	41.604836000	40.175308000	37.154214000
Si	44.281409000	40.881381000	35.818409000
Si	44.601328000	45.934732000	33.475466000
Si	47.786333000	45.959176000	33.236104000
Si	48.764089000	47.874554000	35.459323000
Si	46.850152000	48.745576000	37.503986000
Si	41.559778000	48.683812000	37.191262000
Si	44.250953000	47.925605000	35.887412000
Si	44.657157000	42.890177000	33.435649000
Si	47.809987000	42.906045000	33.184406000
Si	54.820029000	42.892291000	38.824654000
Si	51.688815000	48.539481000	35.162668000
Si	54.396651000	47.952459000	36.442237000
Si	54.821786000	45.932389000	38.828843000
Si	51.685184000	40.280250000	35.157199000
Si	54.388002000	40.870176000	36.441922000
Si	45.891205000	40.429102000	40.178065000
Si	42.732239000	40.484129000	40.067832000
Si	41.755894000	45.970368000	41.673669000
Si	43.949838000	46.754603000	43.817706000
Si	48.961271000	47.083028000	43.299268000
Si	46.296509000	46.002423000	41.977247000

Si	45.613377000	48.510644000	40.249725000
Si	42.584116000	48.414916000	40.012685000
Si	41.749649000	42.931884000	41.764742000
Si	43.811224000	42.032963000	43.797796000
Si	49.013390000	41.816638000	43.514930000
Al	46.453945000	42.911928000	42.061805000
Si	51.902225000	42.851892000	43.549758000
Si	53.958822000	41.868023000	41.521546000
Si	51.825271000	45.918731000	43.496422000
Si	53.943738000	46.941666000	41.529004000
O	46.880721000	38.413283000	36.768658000
O	44.187917000	39.502694000	34.934925000
O	54.378127000	39.439732000	37.248410000
O	51.574042000	38.774282000	35.805625000
O	42.377573000	39.389974000	41.218697000
O	50.370120000	41.197516000	35.469089000
O	48.174452000	40.742278000	36.886539000
O	48.029745000	42.208479000	34.646719000
O	48.462354000	39.569920000	34.540204000
O	46.877623000	39.861910000	39.005705000
O	45.492445000	40.736192000	36.913693000
O	41.785526000	40.104142000	38.792246000
O	42.835050000	41.102821000	36.538350000
O	44.616744000	42.184386000	34.900240000
O	44.139772000	49.294792000	34.979917000
O	43.714379000	46.742547000	32.357089000
O	44.046157000	44.399103000	33.556931000
O	46.169711000	45.938547000	32.993281000
O	48.393946000	44.435586000	33.207611000
O	48.550764000	46.809877000	32.061028000
O	50.373458000	47.636091000	35.507313000
O	48.217469000	48.000384000	36.998807000
O	48.043238000	46.610375000	34.712721000
O	48.438661000	49.254017000	34.633215000
O	46.788423000	48.733675000	39.133825000
O	46.876838000	50.294914000	36.938057000
O	45.544037000	47.978395000	36.883683000
O	41.552084000	48.864200000	38.832245000
O	42.864781000	47.768728000	36.738862000
O	44.423822000	46.608026000	34.941438000
O	43.741951000	42.062706000	32.355863000
O	46.202437000	42.942079000	32.886816000
O	48.608059000	42.064399000	32.028934000
O	54.241045000	44.412877000	38.637978000
O	56.353452000	42.947917000	39.388378000
O	55.464131000	47.983226000	35.210167000
O	51.852512000	48.690128000	33.537532000
O	52.916329000	47.687725000	35.816896000
O	51.597796000	50.052140000	35.797766000
O	54.805136000	46.701379000	37.398381000
O	54.389659000	49.382981000	37.248173000
O	56.355304000	45.878233000	39.395793000
O	55.454315000	40.836558000	35.208549000
O	51.861778000	40.105810000	33.536390000
O	52.906966000	41.133534000	35.817630000
O	54.796941000	42.125057000	37.392914000
O	46.466719000	41.972325000	40.491249000
O	44.334112000	40.403315000	39.672420000

O	46.069379000	39.532565000	41.512477000
O	42.437549000	42.003611000	40.588345000
O	42.224358000	49.318287000	41.315566000
O	43.874460000	48.383659000	43.600916000
O	48.844739000	48.686147000	42.983883000
O	42.786820000	46.028270000	42.948718000
O	41.585328000	44.433618000	41.121828000
O	43.763972000	46.404616000	45.410713000
O	45.398731000	46.112947000	43.348380000
O	48.763039000	46.793396000	44.908740000
O	47.849323000	46.333830000	42.362428000
O	46.230820000	44.506023000	41.294824000
O	45.632171000	46.999869000	40.861918000
O	44.137536000	48.737646000	39.548927000
O	45.865796000	49.546993000	41.474009000
O	42.355311000	46.839928000	40.402851000
O	43.344682000	40.458275000	43.657869000
O	48.668423000	40.257943000	43.094427000
O	42.679021000	43.002298000	43.113352000
O	43.816754000	42.405157000	45.399363000
O	45.220996000	42.266976000	43.056593000
O	48.963133000	41.988758000	45.166311000
O	48.004204000	42.863902000	42.819505000
O	53.547508000	40.327825000	41.874698000
O	50.555457000	42.097871000	43.019413000
O	52.953474000	42.903938000	42.285587000
O	51.594109000	44.388368000	44.035677000
O	53.880427000	42.078423000	39.895072000
O	55.496112000	42.137657000	42.029262000
O	52.379377000	46.812727000	44.759242000
O	53.529034000	48.480038000	41.893380000
O	50.445046000	46.542804000	42.867908000
O	52.930705000	45.903813000	42.280190000
O	53.881056000	46.743724000	39.900798000
O	55.477235000	46.673048000	42.045023000
O	52.555203000	42.020455000	44.809492000
Ga	46.817031000	44.639030000	39.159918000
H	45.473524000	44.237246000	38.441633000
C	48.497458000	43.680295000	37.517850000
H	47.680869000	43.416707000	36.829504000
C	49.547225000	44.383064000	37.039297000
H	47.540408000	46.022560000	39.239946000
H	49.557133000	44.649121000	35.972420000
C	48.388047000	43.194195000	38.948314000
C	49.667174000	43.469407000	39.785413000
H	48.314415000	42.089859000	38.830532000
H	47.180552000	42.551334000	39.748768000
H	49.434317000	43.508185000	40.864152000
H	50.356845000	42.608350000	39.656956000
C	50.734108000	44.770878000	37.874533000
C	50.412989000	44.748927000	39.374804000
H	51.102177000	45.768511000	37.563967000
H	51.569226000	44.073009000	37.643136000
H	51.344890000	44.829259000	39.965062000
H	49.807195000	45.639391000	39.630440000

I15-GaH_2

Electronic energy (r^2SCAN-D4/def2-SVP): -48413.06623454 E_h
Electronic energy (ωB97X-D4rev/def2-mTZVPP): -48457.164122484000 E_h
G_{corr} (673.15 K, 1 atm): 2.04172112 E_h
Number of atoms: 395

Cartesian coordinates (Å)

Si	48.555331000	36.025022000	35.307095000
Si	46.524099000	36.951614000	37.342656000
Si	44.099160000	36.088958000	35.632469000
Si	44.626255000	38.501779000	33.727397000
Si	47.773586000	38.515586000	33.541446000
Si	48.743201000	38.765170000	30.513752000
Si	51.806906000	38.129819000	30.327859000
Si	54.673851000	38.466236000	38.540568000
Si	57.775719000	38.489420000	38.804020000
Si	56.556690000	37.022163000	34.997138000
Si	52.596237000	36.085305000	32.661698000
Si	51.481245000	37.163388000	35.482568000
Si	54.130845000	36.094231000	36.717727000
Si	45.623069000	36.074924000	40.033598000
Si	41.744451000	38.026664000	41.944427000
Si	43.572638000	38.894107000	44.082861000
Si	47.726264000	38.467986000	46.628371000
Si	48.758226000	38.668752000	43.616703000
Si	46.058675000	38.194614000	42.329417000
Si	51.711739000	37.995193000	43.465740000
Si	53.660399000	38.788275000	41.351673000
Si	56.184299000	38.107946000	43.087206000
Si	44.619710000	50.315596000	33.733256000
Si	47.760937000	50.304984000	33.552042000
Si	48.740441000	50.059577000	30.526098000
Si	43.865277000	46.935441000	30.715339000
Si	48.870281000	46.955050000	30.427620000
Si	46.321218000	45.964692000	29.026621000
Si	48.549769000	52.795667000	35.314359000
Si	46.525315000	51.852718000	37.359910000
Si	44.101743000	52.730471000	35.637627000
Si	43.868080000	41.886126000	30.711824000
Si	48.873222000	41.870920000	30.414064000
Si	46.321942000	42.859452000	29.022920000
Si	51.880619000	42.863742000	30.520357000
Si	57.969978000	42.904183000	39.188677000
Si	58.735069000	47.858077000	36.910461000
Si	56.870671000	48.675275000	34.761531000
Si	55.872607000	48.296728000	31.973271000
Si	52.769647000	48.324147000	32.229555000
Si	56.554766000	51.799660000	34.996942000
Si	52.597774000	52.736894000	32.659277000
Si	51.480894000	51.664765000	35.478982000
Si	54.129757000	52.728573000	36.714456000
Si	51.806755000	50.691488000	30.327934000
Si	54.673755000	50.349897000	38.530516000
Si	57.777119000	50.331074000	38.802112000
Si	51.879650000	45.956008000	30.521689000
Si	57.968301000	45.921889000	39.184271000
Si	58.734942000	40.964474000	36.913932000

Si	56.878560000	40.145697000	34.760881000
Si	55.874368000	40.524667000	31.973796000
Si	52.770453000	40.496876000	32.234272000
Si	48.681737000	40.959488000	48.527857000
Si	41.748351000	50.770336000	41.941128000
Si	43.720458000	49.973869000	44.077351000
Si	47.725652000	50.354453000	46.616707000
Si	48.769741000	50.165204000	43.586674000
Si	46.083958000	50.776847000	42.369679000
Si	44.910916000	46.012568000	46.540413000
Si	47.960465000	46.021666000	46.149626000
Si	48.695371000	47.873482000	48.523228000
Si	45.618161000	52.770617000	40.029131000
Si	44.907768000	42.800767000	46.538386000
Si	47.961893000	42.783970000	46.177890000
Si	58.957425000	41.841214000	41.951419000
Si	56.326156000	42.836360000	43.273104000
Si	55.788637000	48.298318000	45.173851000
Si	52.641585000	48.346910000	45.345669000
Si	51.674954000	48.630870000	48.362472000
Si	51.737947000	50.822793000	43.465582000
Si	53.672922000	50.029885000	41.343733000
Si	56.183851000	50.710720000	43.089174000
Si	58.957895000	46.979724000	41.944410000
Si	56.327079000	45.968980000	43.268023000
Si	55.792861000	40.505686000	45.169914000
Si	52.661145000	40.453672000	45.342015000
Si	51.674474000	40.191922000	48.355430000
O	49.973304000	36.649655000	35.856580000
O	47.517117000	35.892351000	36.583793000
O	46.663319000	36.830183000	38.967808000
O	44.964471000	36.629237000	36.941137000
O	44.499703000	36.948701000	34.267354000
O	46.180594000	38.855027000	33.278385000
O	47.910880000	36.990750000	34.130799000
O	48.601827000	38.639519000	32.143233000
O	50.190193000	38.150289000	30.072945000
O	52.212202000	36.841169000	31.250853000
O	52.309049000	39.559330000	30.974763000
O	54.590435000	36.892020000	38.081501000
O	56.172098000	38.729160000	39.138048000
O	56.916078000	38.601025000	35.310488000
O	54.992067000	36.664060000	35.423938000
O	51.809672000	36.857656000	33.902748000
O	52.529191000	36.326615000	36.429978000
O	42.398754000	37.921808000	43.457970000
O	45.060292000	38.394787000	43.596582000
O	48.580631000	38.683947000	45.236198000
O	47.551565000	37.848178000	42.877048000
O	45.568799000	36.912822000	41.447669000
O	50.104927000	37.833543000	43.228844000
O	52.404967000	37.948657000	41.970623000
O	52.059728000	39.417713000	44.223865000
O	53.573291000	38.754235000	39.716605000
O	55.070074000	38.120479000	41.864780000
O	44.496668000	51.869516000	34.272490000
O	46.171170000	49.968140000	33.272668000
O	47.897631000	51.831271000	34.140994000

O	48.598273000	50.179189000	32.157784000
O	48.576224000	48.507230000	30.008639000
O	45.410115000	46.597978000	30.237928000
O	47.898046000	45.991676000	29.517904000
O	45.867840000	44.412095000	28.742902000
O	49.973157000	52.174913000	35.853578000
O	47.524285000	52.911032000	36.604285000
O	46.651315000	51.975235000	38.990399000
O	44.970780000	52.189407000	36.945313000
O	48.573613000	40.318416000	30.000696000
O	45.413486000	42.222945000	30.234947000
O	47.901376000	42.834277000	29.504814000
O	50.433272000	42.216879000	30.080636000
O	51.655351000	44.409663000	31.020001000
O	58.589893000	44.413989000	39.366107000
O	58.119065000	47.830664000	35.386443000
O	58.302111000	46.473023000	37.682020000
O	58.220526000	49.190598000	37.705068000
O	56.997115000	48.629182000	33.137473000
O	56.904225000	50.219209000	35.314453000
O	54.350077000	48.572519000	32.573457000
O	52.544927000	46.756406000	31.801679000
O	54.989934000	52.162377000	35.419140000
O	51.808263000	51.966211000	33.899016000
O	52.527466000	52.502239000	36.426944000
O	50.187844000	50.677552000	30.088596000
O	52.220364000	51.979130000	31.248496000
O	52.310443000	49.258941000	30.967498000
O	54.589160000	51.924725000	38.074847000
O	56.172083000	50.085334000	39.126500000
O	50.429427000	46.603643000	30.093341000
O	58.123894000	40.993753000	35.387586000
O	58.305016000	42.349688000	37.687774000
O	58.212070000	39.634001000	37.707083000
O	57.001377000	40.195045000	33.136461000
O	54.352018000	40.250280000	32.574498000
O	52.542294000	42.063011000	31.801192000
O	50.286196000	40.918018000	48.842942000
O	48.361580000	42.359733000	47.710990000
O	48.201461000	39.621211000	47.705019000
O	42.468274000	50.824869000	43.428643000
O	45.168481000	50.665732000	43.714217000
O	48.538481000	50.115140000	45.199100000
O	47.623994000	51.038257000	42.813465000
O	45.567955000	52.048556000	41.502579000
O	44.926716000	44.410883000	46.856907000
O	46.389839000	46.489632000	45.983200000
O	48.137083000	44.404879000	45.988809000
O	50.288548000	47.926978000	48.885169000
O	48.446956000	46.497120000	47.640494000
O	48.222952000	49.215065000	47.698526000
O	46.386231000	42.351087000	45.947487000
O	58.649219000	41.944831000	40.321570000
O	57.922536000	42.734126000	42.896221000
O	55.899254000	44.403908000	43.449965000
O	55.971534000	46.732138000	44.681165000
O	54.240188000	48.601755000	45.663654000
O	56.168978000	49.271778000	43.902168000

O	51.785252000	48.564163000	46.713990000
O	50.132779000	50.977462000	43.236359000
O	52.424185000	50.875124000	41.970254000
O	52.079659000	49.405075000	44.229045000
O	53.576084000	50.062886000	39.710483000
O	55.083661000	50.696164000	41.853888000
O	58.650120000	46.884898000	40.313912000
O	57.918946000	46.078857000	42.876864000
O	55.953154000	42.077671000	44.683740000
O	54.254459000	40.150845000	45.651736000
O	56.197812000	39.551652000	43.891618000
O	51.806799000	40.245492000	46.710512000
H	48.702871000	34.676568000	34.772566000
H	44.419875000	34.676323000	35.346829000
H	42.683628000	36.258975000	36.022159000
H	43.726523000	38.680216000	32.577941000
H	47.730594000	38.010996000	29.830569000
H	52.421136000	38.002381000	29.030381000
H	58.465353000	38.743363000	40.066967000
H	58.088382000	37.108509000	38.286195000
H	56.651061000	36.758841000	33.558678000
H	57.541582000	36.246995000	35.782467000
H	52.141234000	34.673922000	32.562045000
H	54.071037000	36.146097000	32.821438000
H	54.418082000	34.675478000	36.917895000
H	44.265382000	36.148734000	39.494230000
H	46.085045000	34.677316000	40.196440000
H	42.177304000	36.886192000	41.136491000
H	40.290195000	38.107003000	42.090377000
H	43.614692000	38.689573000	45.487247000
H	46.266760000	38.594605000	46.380864000
H	48.065374000	37.113072000	47.131272000
H	52.195251000	36.881484000	44.271734000
H	55.858296000	36.988842000	44.011824000
H	57.529558000	37.965437000	42.519250000
H	41.636485000	38.780154000	36.634495000
H	40.338249000	40.845235000	36.817795000
H	43.726296000	50.140518000	32.578071000
H	47.730503000	50.809921000	29.830551000
H	42.910974000	46.071594000	29.969678000
H	43.636013000	48.346500000	30.474316000
H	46.055518000	46.690812000	27.779371000
H	40.338195000	47.975592000	36.817799000
H	41.636404000	50.040687000	36.634467000
H	48.702714000	54.144377000	34.772532000
H	44.419585000	54.144606000	35.346948000
H	42.683575000	52.561616000	36.022276000
H	43.636069000	40.474361000	30.474327000
H	42.910998000	42.749253000	29.969681000
H	46.055551000	42.130076000	27.779377000
H	52.768240000	42.805898000	29.339147000
H	60.152183000	47.950749000	36.769108000
H	56.115708000	49.276599000	30.928772000
H	56.049418000	46.904457000	31.507081000
H	57.541465000	52.574079000	35.782446000
H	56.650952000	52.062214000	33.558655000
H	52.141090000	54.147065000	32.562019000
H	54.070917000	52.674925000	32.821418000

H	54.418140000	54.145525000	36.917811000
H	52.421044000	50.818602000	29.030363000
H	58.465269000	50.077736000	40.066952000
H	58.088278000	51.712582000	38.286179000
H	52.768217000	46.015092000	29.339142000
H	60.152234000	40.870368000	36.769119000
H	56.049458000	41.916585000	31.507090000
H	56.115777000	39.544444000	30.928783000
H	48.027948000	40.958435000	49.819979000
H	40.290115000	50.713683000	42.090582000
H	42.176850000	51.934815000	41.136521000
H	43.615015000	50.131729000	45.487215000
H	48.065229000	51.707917000	47.131231000
H	46.266685000	50.226321000	46.380825000
H	40.476352000	46.423809000	42.229530000
H	44.578030000	46.685732000	47.785516000
H	48.027871000	47.862537000	49.819962000
H	46.084739000	54.143668000	40.196331000
H	44.265252000	52.672179000	39.494179000
H	40.476382000	42.397038000	42.229542000
H	44.578055000	42.135174000	47.785513000
H	58.818796000	40.408567000	42.221796000
H	60.309668000	42.345550000	42.255160000
H	56.725236000	48.548904000	46.274187000
H	51.671681000	50.045758000	48.769745000
H	52.721673000	47.848745000	49.007593000
H	52.195144000	51.939534000	44.271718000
H	55.858180000	51.832229000	44.011806000
H	57.529465000	50.855664000	42.519236000
H	60.309639000	46.475584000	42.255156000
H	58.818736000	48.412544000	42.221786000
H	56.725177000	40.272281000	46.274313000
H	52.722235000	40.971966000	49.006966000
H	51.671815000	38.775269000	48.769821000
Si	48.779230000	40.963400000	35.394100000
Si	46.870540000	39.998289000	37.408747000
Si	41.585341000	40.161425000	37.181547000
Si	44.273740000	40.881036000	35.846146000
Si	44.629132000	45.937444000	33.459468000
Si	47.795612000	45.942402000	33.201790000
Si	48.751091000	47.861028000	35.422065000
Si	46.864354000	48.798089000	37.462577000
Si	41.579979000	48.668803000	37.169418000
Si	44.277001000	47.940768000	35.865031000
Si	44.633709000	42.892965000	33.452018000
Si	47.802386000	42.893033000	33.188093000
Si	54.813040000	42.890442000	38.826189000
Si	51.681167000	48.552397000	35.142726000
Si	54.381774000	47.950768000	36.434546000
Si	54.808684000	45.929133000	38.821543000
Si	51.691558000	40.278832000	35.152593000
Si	54.394673000	40.865144000	36.442391000
Si	45.784681000	40.485731000	40.150437000
Si	42.684852000	40.443008000	40.058495000
Si	41.753615000	45.942389000	41.689626000
Si	43.921640000	46.780669000	43.791828000
Si	48.984178000	47.040574000	43.353359000
Si	46.369879000	45.968665000	41.952035000

Si	45.736343000	48.437759000	40.221990000
Si	42.642738000	48.391607000	40.028323000
Si	41.758619000	42.909511000	41.753590000
Si	43.753868000	42.020232000	43.866516000
Si	49.032341000	41.783233000	43.471021000
Al	46.369664000	42.787060000	42.117283000
Si	51.918740000	42.867555000	43.557229000
Si	53.968349000	41.873172000	41.530401000
Si	51.873212000	45.927063000	43.530892000
Si	53.958602000	46.944142000	41.527538000
O	46.904449000	38.465514000	36.818912000
O	44.173197000	39.504162000	34.957069000
O	54.385927000	39.435440000	37.250705000
O	51.577890000	38.773317000	35.802389000
O	42.351737000	39.396570000	41.257440000
O	50.382845000	41.202344000	35.464776000
O	48.204478000	40.808108000	36.921449000
O	48.045210000	42.226385000	34.660523000
O	48.448911000	39.582991000	34.576281000
O	46.864921000	39.966679000	39.036033000
O	45.532046000	40.790925000	36.891386000
O	41.669447000	40.080249000	38.829881000
O	42.857606000	41.073698000	36.633567000
O	44.531301000	42.193731000	34.915141000
O	44.176829000	49.315817000	34.969402000
O	43.723304000	46.748556000	32.358397000
O	44.047754000	44.413801000	33.570715000
O	46.182480000	45.911853000	32.934855000
O	48.398245000	44.418669000	33.166320000
O	48.569658000	46.806789000	32.043697000
O	50.362120000	47.644801000	35.455252000
O	48.201943000	48.000466000	36.961822000
O	48.028284000	46.596701000	34.680726000
O	48.411910000	49.241412000	34.605411000
O	46.845171000	48.850154000	39.093960000
O	46.904525000	50.332853000	36.866640000
O	45.523460000	48.029784000	36.920553000
O	41.648630000	48.823033000	38.811271000
O	42.858128000	47.749986000	36.652515000
O	44.524509000	46.632168000	34.924592000
O	43.728020000	42.078776000	32.353951000
O	46.187718000	42.920872000	32.928926000
O	48.574810000	42.031138000	32.028792000
O	54.229047000	44.408959000	38.636338000
O	56.347770000	42.954519000	39.386489000
O	55.458317000	47.980217000	35.209848000
O	51.856554000	48.725083000	33.521954000
O	52.904386000	47.696349000	35.798264000
O	51.581513000	50.055322000	35.798626000
O	54.779135000	46.696653000	37.390573000
O	54.377125000	49.380654000	37.243398000
O	56.345053000	45.878299000	39.379639000
O	55.465800000	40.835536000	35.213338000
O	51.861920000	40.101292000	33.530742000
O	52.919602000	41.125207000	35.806877000
O	54.797157000	42.118442000	37.397740000
O	46.079581000	42.076995000	40.477748000
O	44.254950000	40.258814000	39.583163000

O	46.040302000	39.616606000	41.504206000
O	42.463458000	41.980113000	40.583920000
O	42.264355000	49.349783000	41.286461000
O	43.722692000	48.395623000	43.594086000
O	48.784594000	48.629330000	43.002190000
O	42.776175000	45.969376000	42.973484000
O	41.584348000	44.414780000	41.109397000
O	43.804660000	46.403010000	45.381976000
O	45.388802000	46.300607000	43.217297000
O	48.812302000	46.785267000	44.970740000
O	47.908238000	46.181417000	42.461911000
O	46.172939000	44.429861000	41.388793000
O	45.990463000	46.871143000	40.633706000
O	44.220809000	48.627828000	39.608662000
O	45.937585000	49.365194000	41.546423000
O	42.387951000	46.828256000	40.448766000
O	43.244495000	40.462667000	43.710517000
O	48.790767000	40.207790000	43.064542000
O	42.690533000	43.004722000	43.099095000
O	43.731317000	42.422291000	45.461038000
O	45.220177000	42.217978000	43.238551000
O	48.920698000	41.974811000	45.118774000
O	47.977549000	42.733247000	42.714985000
O	53.547769000	40.336567000	41.895086000
O	50.556785000	42.168313000	43.001750000
O	52.979711000	42.919397000	42.302023000
O	51.643102000	44.400468000	44.074908000
O	53.881830000	42.076984000	39.904249000
O	55.510648000	42.136916000	42.028680000
O	52.446839000	46.817187000	44.785534000
O	53.549706000	48.484518000	41.892098000
O	50.489483000	46.568591000	42.928127000
O	52.949226000	45.911262000	42.290369000
O	53.879314000	46.744499000	39.901158000
O	55.493938000	46.667137000	42.034738000
O	52.544136000	42.003397000	44.807713000
Ga	46.385559000	43.862504000	39.408435000
H	41.916189000	44.368513000	37.322725000
C	48.611409000	44.063168000	37.523066000
H	47.969758000	43.647149000	36.732824000
C	49.637845000	44.861661000	37.166039000
H	45.026231000	44.203647000	38.697454000
H	49.829192000	45.032374000	36.097202000
C	48.323921000	43.641035000	38.937007000
C	49.400588000	44.146456000	39.928791000
H	48.350290000	42.527255000	38.947050000
H	41.863643000	44.444098000	38.077040000
H	49.050701000	44.120069000	40.975002000
H	50.259206000	43.441862000	39.888048000
C	50.546405000	45.528731000	38.163724000
C	49.913177000	45.542867000	39.558365000
H	50.783288000	46.557209000	37.829936000
H	51.523754000	44.998171000	38.192509000
H	50.644011000	45.890508000	40.310778000
H	49.074649000	46.272513000	39.568720000

I16-GaH_2

Electronic energy (r^2SCAN-D4/def2-SVP): -48411.88451726 E_h
Electronic energy (ωB97X-D4rev/def2-mTZVPP): -48455.970561856500 E_h
G_{corr} (673.15 K, 1 atm): 2.03245981 E_h
Number of atoms: 393

Cartesian coordinates (Å)

Si	48.576849000	36.033957000	35.330123000
Si	46.532370000	37.007751000	37.378296000
Si	44.106043000	36.094284000	35.629810000
Si	44.660449000	38.427483000	33.659632000
Si	47.777895000	38.541402000	33.476955000
Si	48.768434000	38.756204000	30.486633000
Si	51.797587000	38.115037000	30.320273000
Si	54.684459000	38.470453000	38.544753000
Si	57.778360000	38.492336000	38.803881000
Si	56.556500000	37.022111000	34.996102000
Si	52.597951000	36.080298000	32.671809000
Si	51.497498000	37.126874000	35.513973000
Si	54.159418000	36.094578000	36.726666000
Si	45.612977000	36.066167000	40.068141000
Si	41.738168000	38.021558000	41.930584000
Si	43.483189000	38.900394000	44.074837000
Si	47.731029000	38.470433000	46.652662000
Si	48.732860000	38.675908000	43.690531000
Si	46.085484000	38.139213000	42.357642000
Si	51.694093000	37.992998000	43.461422000
Si	53.649867000	38.761628000	41.350966000
Si	56.184359000	38.105199000	43.085284000
Si	44.613033000	50.318708000	33.735189000
Si	47.764411000	50.268920000	33.534000000
Si	48.760540000	50.073581000	30.500485000
Si	43.864425000	46.931704000	30.715166000
Si	48.879160000	47.003058000	30.392880000
Si	46.323787000	45.973271000	29.028330000
Si	48.554160000	52.778617000	35.296481000
Si	46.532522000	51.810967000	37.362360000
Si	44.106203000	52.723955000	35.639010000
Si	43.875244000	41.885080000	30.705968000
Si	48.901446000	41.860842000	30.359000000
Si	46.324955000	42.861298000	29.013865000
Si	51.883896000	42.866498000	30.515897000
Si	57.960928000	42.897291000	39.184978000
Si	58.732961000	47.855714000	36.911277000
Si	56.879622000	48.674735000	34.759731000
Si	55.870049000	48.298292000	31.972120000
Si	52.763593000	48.335061000	32.234858000
Si	56.557452000	51.798296000	34.996653000
Si	52.604013000	52.741150000	32.658302000
Si	51.489290000	51.666046000	35.468581000
Si	54.129162000	52.730522000	36.712477000
Si	51.810069000	50.714176000	30.331058000
Si	54.671470000	50.355035000	38.544381000
Si	57.774498000	50.332226000	38.804684000
Si	51.889543000	45.952352000	30.530091000
Si	57.974325000	45.924410000	39.183335000
Si	58.732852000	40.963619000	36.910552000

Si	56.847671000	40.148560000	34.771924000
Si	55.862848000	40.524468000	31.969706000
Si	52.745162000	40.481213000	32.224437000
Si	48.652206000	40.963914000	48.529012000
Si	41.739877000	50.807292000	41.947522000
Si	43.572592000	50.004070000	44.079119000
Si	47.726617000	50.360560000	46.620651000
Si	48.753955000	50.164647000	43.604587000
Si	46.050200000	50.699269000	42.337218000
Si	44.781350000	45.957206000	46.546976000
Si	47.933403000	45.967475000	46.153423000
Si	48.670066000	47.885840000	48.517378000
Si	45.618588000	52.759534000	40.037104000
Si	44.803207000	42.841544000	46.538814000
Si	47.946638000	42.842870000	46.219830000
Si	58.954344000	41.840400000	41.943693000
Si	56.306443000	42.837229000	43.271464000
Si	55.797438000	48.296453000	45.164136000
Si	52.625770000	48.350607000	45.312376000
Si	51.672429000	48.626288000	48.349096000
Si	51.702775000	50.839816000	43.459844000
Si	53.646333000	50.033175000	41.354361000
Si	56.182610000	50.708779000	43.089786000
Si	58.960132000	46.981506000	41.930059000
Si	56.314232000	45.967729000	43.265867000
Si	55.803604000	40.500358000	45.162206000
Si	52.676694000	40.439227000	45.330476000
Si	51.670853000	40.195335000	48.342209000
O	50.009834000	36.580048000	35.914902000
O	47.518219000	35.956303000	36.596864000
O	46.632524000	36.860846000	39.005983000
O	44.985752000	36.714940000	36.901579000
O	44.494746000	36.889407000	34.223200000
O	46.185896000	38.689560000	33.085974000
O	48.011391000	37.079465000	34.182138000
O	48.688857000	38.659421000	32.129591000
O	50.187707000	38.096974000	30.023584000
O	52.204164000	36.849763000	31.271852000
O	52.259538000	39.559438000	30.963240000
O	54.652841000	36.896694000	38.073676000
O	56.177559000	38.761053000	39.141128000
O	56.893292000	38.603318000	35.321183000
O	54.990906000	36.652070000	35.410479000
O	51.817883000	36.824100000	33.933818000
O	52.554890000	36.330219000	36.475377000
O	42.376970000	37.857012000	43.448808000
O	44.965543000	38.593943000	43.436382000
O	48.672430000	38.719026000	45.319349000
O	47.479421000	37.778481000	43.122588000
O	45.590990000	36.813004000	41.535965000
O	50.079736000	37.878076000	43.217456000
O	52.388614000	37.924998000	41.965415000
O	52.067425000	39.413615000	44.207325000
O	53.568429000	38.714150000	39.715004000
O	55.061480000	38.102979000	41.869736000
O	44.494756000	51.876635000	34.261364000
O	46.164796000	49.959574000	33.283984000
O	47.937848000	51.795383000	34.116339000

O	48.583091000	50.118830000	32.131405000
O	48.669580000	48.545085000	29.894410000
O	45.413645000	46.601011000	30.242915000
O	47.901560000	46.021122000	29.509295000
O	45.876731000	44.417158000	28.753180000
O	49.980906000	52.169712000	35.842644000
O	47.519102000	52.864285000	36.578887000
O	46.652609000	51.972113000	38.988748000
O	44.979556000	52.140907000	36.926824000
O	48.621980000	40.303521000	29.949995000
O	45.417767000	42.210735000	30.216898000
O	47.907875000	42.822316000	29.476508000
O	50.447640000	42.242831000	30.013813000
O	51.687153000	44.406410000	31.039075000
O	58.576161000	44.408684000	39.363925000
O	58.132168000	47.833411000	35.380621000
O	58.291608000	46.465821000	37.672981000
O	58.200190000	49.180284000	37.709716000
O	56.996354000	48.625876000	33.135396000
O	56.913875000	50.218855000	35.310123000
O	54.346667000	48.589949000	32.564028000
O	52.555116000	46.766869000	31.799227000
O	54.993521000	52.155342000	35.423985000
O	51.806817000	51.952169000	33.882026000
O	52.528939000	52.522918000	36.405243000
O	50.188908000	50.763086000	30.112510000
O	52.263453000	51.998497000	31.232317000
O	52.271362000	49.272366000	30.985541000
O	54.570321000	51.925143000	38.077230000
O	56.170611000	50.108627000	39.146497000
O	50.426951000	46.573340000	30.117888000
O	58.094929000	40.996597000	35.394386000
O	58.302503000	42.348025000	37.683438000
O	58.226358000	39.625896000	37.701632000
O	56.971806000	40.193227000	33.147587000
O	54.331138000	40.244594000	32.543561000
O	52.493997000	42.047530000	31.811707000
O	50.274094000	40.956260000	48.754919000
O	48.207368000	42.353828000	47.761703000
O	48.149135000	39.602074000	47.766113000
O	42.396509000	50.970000000	43.457936000
O	45.067829000	50.496451000	43.619075000
O	48.565915000	50.094859000	45.224344000
O	47.551793000	51.005402000	42.887460000
O	45.565539000	52.008531000	41.497038000
O	44.459338000	44.398423000	46.909882000
O	46.310662000	46.088366000	45.947894000
O	48.418136000	44.404405000	46.035057000
O	50.280446000	47.887443000	48.813400000
O	48.299006000	46.539607000	47.642447000
O	48.212180000	49.255384000	47.739033000
O	46.331587000	42.707633000	45.912071000
O	58.652657000	41.938574000	40.311827000
O	57.895322000	42.728429000	42.864395000
O	55.893605000	44.404098000	43.468000000
O	56.011409000	46.728987000	44.690278000
O	54.233382000	48.591235000	45.600779000
O	56.199024000	49.266307000	43.896186000

O	51.814335000	48.577294000	46.704230000
O	50.096088000	51.017264000	43.240200000
O	52.393337000	50.885801000	41.963077000
O	52.039725000	49.414760000	44.214119000
O	53.570376000	50.063761000	39.718754000
O	55.059819000	50.686899000	41.874866000
O	58.686751000	46.890515000	40.292016000
O	57.892935000	46.074995000	42.822495000
O	55.963698000	42.073622000	44.686068000
O	54.268158000	40.129521000	45.634209000
O	56.211959000	39.555331000	43.877409000
O	51.835789000	40.216234000	46.701305000
H	48.702872000	34.676567000	34.772567000
H	44.419876000	34.676322000	35.346831000
H	42.683629000	36.258974000	36.022161000
H	43.726523000	38.680215000	32.577942000
H	47.730594000	38.010995000	29.830570000
H	52.421136000	38.002379000	29.030380000
H	58.465354000	38.743362000	40.066966000
H	58.088383000	37.108508000	38.286194000
H	56.651060000	36.758840000	33.558677000
H	57.541582000	36.246994000	35.782466000
H	52.141233000	34.673921000	32.562045000
H	54.071037000	36.146097000	32.821438000
H	54.418082000	34.675478000	36.917894000
H	44.265383000	36.148733000	39.494232000
H	46.085046000	34.677315000	40.196441000
H	42.177306000	36.886191000	41.136493000
H	40.290196000	38.107002000	42.090379000
H	43.614693000	38.689573000	45.487248000
H	46.266762000	38.594604000	46.380865000
H	48.065376000	37.113071000	47.131273000
H	52.195253000	36.881483000	44.271734000
H	55.858297000	36.988841000	44.011824000
H	57.529559000	37.965437000	42.519249000
H	41.636485000	38.780153000	36.634495000
H	40.338248000	40.845234000	36.817796000
H	43.726295000	50.140518000	32.578071000
H	47.730501000	50.809920000	29.830552000
H	42.910973000	46.071593000	29.969680000
H	43.636012000	48.346499000	30.474317000
H	46.055517000	46.690811000	27.779372000
H	40.338196000	47.975591000	36.817799000
H	41.636403000	50.040687000	36.634467000
H	48.702714000	54.144376000	34.772533000
H	44.419584000	54.144605000	35.346949000
H	42.683575000	52.561615000	36.022277000
H	43.636069000	40.474360000	30.474328000
H	42.910997000	42.749251000	29.969682000
H	46.055549000	42.130075000	27.779378000
H	52.768239000	42.805897000	29.339146000
H	60.152182000	47.950749000	36.769107000
H	56.115707000	49.276599000	30.928771000
H	56.049417000	46.904456000	31.507081000
H	57.541464000	52.574079000	35.782445000
H	56.650952000	52.062213000	33.558655000
H	52.141089000	54.147064000	32.562019000
H	54.070916000	52.674924000	32.821418000

H	54.418140000	54.145524000	36.917810000
H	52.421043000	50.818602000	29.030362000
H	58.465269000	50.077735000	40.066951000
H	58.088279000	51.712581000	38.286178000
H	52.768216000	46.015091000	29.339142000
H	60.152234000	40.870367000	36.769118000
H	56.049458000	41.916584000	31.507089000
H	56.115777000	39.544443000	30.928782000
H	48.027951000	40.958434000	49.819979000
H	40.290116000	50.713683000	42.090584000
H	42.176850000	51.934815000	41.136523000
H	43.615016000	50.131728000	45.487216000
H	48.065230000	51.707917000	47.131232000
H	46.266686000	50.226320000	46.380826000
H	40.476352000	46.423809000	42.229530000
H	44.578031000	46.685731000	47.785516000
H	48.027873000	47.862536000	49.819962000
H	46.084739000	54.143667000	40.196332000
H	44.265253000	52.672178000	39.494181000
H	40.476382000	42.397038000	42.229543000
H	44.578056000	42.135173000	47.785514000
H	58.818797000	40.408566000	42.221795000
H	60.309670000	42.345550000	42.255159000
H	56.725237000	48.548903000	46.274186000
H	51.671683000	50.045758000	48.769745000
H	52.721675000	47.848744000	49.007593000
H	52.195146000	51.939533000	44.271718000
H	55.858182000	51.832229000	44.011805000
H	57.529466000	50.855664000	42.519235000
H	60.309640000	46.475584000	42.255155000
H	58.818737000	48.412544000	42.221785000
H	56.725178000	40.272281000	46.274312000
H	52.722238000	40.971966000	49.006966000
H	51.671817000	38.775268000	48.769821000
Si	48.710707000	41.093460000	35.284677000
Si	46.816350000	40.117899000	37.386593000
Si	41.621526000	40.185968000	37.104661000
Si	44.279152000	40.842816000	35.751157000
Si	44.629961000	45.934826000	33.470732000
Si	47.817152000	45.999222000	33.122280000
Si	48.777518000	47.814524000	35.395180000
Si	46.873113000	48.725830000	37.453677000
Si	41.569717000	48.679450000	37.191207000
Si	44.247269000	47.947775000	35.862468000
Si	44.724352000	42.886592000	33.419474000
Si	47.876068000	42.980946000	33.060184000
Si	54.793830000	42.889076000	38.830035000
Si	51.705649000	48.552105000	35.166011000
Si	54.405592000	47.946791000	36.450413000
Si	54.821355000	45.938947000	38.845071000
Si	51.666323000	40.234747000	35.146565000
Si	54.357134000	40.870683000	36.449278000
Si	45.940324000	40.542543000	40.293623000
Si	42.771672000	40.474015000	40.079237000
Si	41.770418000	45.948444000	41.709893000
Si	43.794606000	46.921642000	43.830432000
Si	48.894719000	47.045382000	43.368934000
Si	46.185101000	46.043469000	42.135265000

Si	45.734718000	48.320627000	40.205131000
Si	42.656665000	48.421054000	40.037714000
Si	41.762890000	42.926207000	41.763513000
Si	43.695403000	41.899575000	43.835809000
Si	48.940243000	41.770167000	43.580933000
Al	46.272390000	42.872315000	42.335305000
Si	51.869104000	42.841400000	43.576777000
Si	53.917853000	41.860812000	41.525611000
Si	51.804170000	45.910894000	43.521509000
Si	53.902604000	46.943592000	41.534878000
O	46.980286000	38.545125000	36.978639000
O	44.316289000	39.443238000	34.905876000
O	54.364859000	39.445068000	37.265435000
O	51.554947000	38.740967000	35.814242000
O	42.341526000	39.372374000	41.197369000
O	50.323545000	41.110377000	35.431977000
O	48.040507000	41.015622000	36.781293000
O	48.207496000	42.475181000	34.580388000
O	48.194090000	39.768494000	34.473642000
O	46.999962000	40.432580000	38.995757000
O	45.345402000	40.691405000	36.986112000
O	41.935230000	40.140828000	38.719956000
O	42.774777000	41.091150000	36.324420000
O	44.744461000	42.121167000	34.857701000
O	44.148405000	49.323370000	34.966734000
O	43.712072000	46.703822000	32.349915000
O	44.112888000	44.387746000	33.603352000
O	46.191038000	45.966629000	32.972865000
O	48.438984000	44.502950000	32.861570000
O	48.505413000	46.977965000	32.002361000
O	50.390847000	47.649166000	35.497035000
O	48.188542000	47.910119000	36.918866000
O	48.160547000	46.498128000	34.640927000
O	48.398608000	49.181271000	34.576918000
O	46.870862000	48.667227000	39.077266000
O	46.937201000	50.282569000	36.914425000
O	45.520900000	48.012101000	36.876464000
O	41.639635000	48.847039000	38.830920000
O	42.847485000	47.758096000	36.678124000
O	44.459777000	46.633361000	34.921768000
O	43.775568000	42.080387000	32.351919000
O	46.256171000	42.958853000	32.841519000
O	48.624002000	42.004010000	31.979670000
O	54.238618000	44.419578000	38.668815000
O	56.337025000	42.926185000	39.376062000
O	55.471425000	47.976587000	35.216250000
O	51.863395000	48.713109000	33.541781000
O	52.927235000	47.692076000	35.817602000
O	51.612683000	50.061665000	35.806447000
O	54.806767000	46.691024000	37.404150000
O	54.400560000	49.374740000	37.259741000
O	56.354095000	45.895253000	39.415993000
O	55.425767000	40.830815000	35.215705000
O	51.861980000	40.055511000	33.530477000
O	52.869903000	41.105857000	35.817828000
O	54.748311000	42.141670000	37.384932000
O	46.369908000	42.087517000	40.692854000
O	44.397429000	40.348075000	39.801637000

O	46.376078000	39.380412000	41.323286000
O	42.442630000	41.990275000	40.582086000
O	42.346102000	49.430218000	41.276880000
O	43.230681000	48.448479000	43.671334000
O	48.823172000	48.641870000	42.997689000
O	42.840852000	45.924010000	42.959611000
O	41.577951000	44.426133000	41.119304000
O	43.729556000	46.510391000	45.413403000
O	45.342028000	46.877509000	43.288003000
O	48.683911000	46.855085000	44.995002000
O	47.786354000	46.221759000	42.498621000
O	45.753034000	44.499193000	42.066323000
O	45.884698000	46.761407000	40.672770000
O	44.230887000	48.594362000	39.576230000
O	46.005761000	49.324928000	41.459477000
O	42.363182000	46.871987000	40.479772000
O	42.958238000	40.424444000	43.747131000
O	48.671449000	40.208003000	43.105317000
O	42.738082000	42.998987000	43.082092000
O	43.742859000	42.284059000	45.428826000
O	45.154391000	41.812346000	43.143449000
O	48.830116000	41.865153000	45.237230000
O	47.902400000	42.809197000	42.927678000
O	53.534969000	40.312249000	41.880262000
O	50.478312000	42.124085000	43.119986000
O	52.866795000	42.868535000	42.266017000
O	51.635889000	44.381512000	44.083857000
O	53.866680000	42.059799000	39.897565000
O	55.444129000	42.162735000	42.047084000
O	52.400890000	46.824394000	44.750797000
O	53.506425000	48.487219000	41.898022000
O	50.381831000	46.491553000	42.959658000
O	52.854580000	45.916537000	42.253112000
O	53.872864000	46.759777000	39.902754000
O	55.425226000	46.653949000	42.068847000
O	52.566135000	41.991372000	44.800669000
Ga	47.631905000	42.686662000	39.206266000
C	50.368058000	42.617526000	38.474612000
H	50.557066000	41.587281000	38.139922000
C	50.884790000	43.647302000	37.770127000
H	46.656483000	43.350144000	38.167013000
H	51.499762000	43.428898000	36.884374000
C	49.553307000	42.810073000	39.725417000
C	49.844848000	44.192876000	40.348697000
H	49.759365000	41.997764000	40.450514000
H	49.203020000	44.390567000	41.225649000
H	50.892309000	44.172388000	40.718368000
C	50.698380000	45.095933000	38.135357000
C	49.719163000	45.310686000	39.303041000
H	50.374084000	45.667069000	37.244079000
H	51.691571000	45.522912000	38.388598000
H	49.893286000	46.297219000	39.772613000
H	48.677643000	45.354852000	38.914217000

TS11-GaH$_2$

Electronic energy (r^{2}SCAN-D4/def2-SVP): -48411.82174494 E_h
Electronic energy (ωB97X-D4rev/def2-mTZVPP): -48455.902733343200 E_h
G_{corr} (673.15 K, 1 atm): 2.03213129 E_h
Number of atoms: 393

Cartesian coordinates (Å)

Si	48.565482000	36.027621000	35.319279000
Si	46.529781000	36.976796000	37.347433000
Si	44.101482000	36.091362000	35.629359000
Si	44.642956000	38.463323000	33.690455000
Si	47.776353000	38.530016000	33.497964000
Si	48.760788000	38.755563000	30.494156000
Si	51.801217000	38.119897000	30.322470000
Si	54.682722000	38.469204000	38.544949000
Si	57.776692000	38.491243000	38.805042000
Si	56.555541000	37.021135000	34.996162000
Si	52.596416000	36.082832000	32.669951000
Si	51.486006000	37.144785000	35.503405000
Si	54.146518000	36.094927000	36.720978000
Si	45.623962000	36.077331000	40.044138000
Si	41.739466000	38.015541000	41.938092000
Si	43.482040000	38.873488000	44.080148000
Si	47.729309000	38.466920000	46.642062000
Si	48.742852000	38.676959000	43.664634000
Si	46.068851000	38.190161000	42.352413000
Si	51.692794000	37.985247000	43.459661000
Si	53.645250000	38.769560000	41.349741000
Si	56.183508000	38.106257000	43.085466000
Si	44.610103000	50.307273000	33.742277000
Si	47.765410000	50.266929000	33.548362000
Si	48.751803000	50.065532000	30.509786000
Si	43.865742000	46.932868000	30.713110000
Si	48.873352000	46.984673000	30.408855000
Si	46.324041000	45.968256000	29.027089000
Si	48.555268000	52.778080000	35.301341000
Si	46.531374000	51.807388000	37.372521000
Si	44.107380000	52.723057000	35.641820000
Si	43.870624000	41.883792000	30.709059000
Si	48.889664000	41.862299000	30.392354000
Si	46.324928000	42.858593000	29.019528000
Si	51.886772000	42.864472000	30.521795000
Si	57.977500000	42.900321000	39.183533000
Si	58.732654000	47.854423000	36.909565000
Si	56.882101000	48.677202000	34.755773000
Si	55.872228000	48.298156000	31.971959000
Si	52.769120000	48.330270000	32.240651000
Si	56.559163000	51.798549000	34.995953000
Si	52.603201000	52.739972000	32.656120000
Si	51.492669000	51.659745000	35.467715000
Si	54.131730000	52.730624000	36.711430000
Si	51.807976000	50.709916000	30.332368000
Si	54.676342000	50.363172000	38.545130000
Si	57.775380000	50.331543000	38.804709000
Si	51.890514000	45.951801000	30.530443000
Si	57.992224000	45.926187000	39.182571000
Si	58.733815000	40.964098000	36.910664000

Si	56.860179000	40.146559000	34.766493000
Si	55.869645000	40.523990000	31.971022000
Si	52.759816000	40.484229000	32.227543000
Si	48.655097000	40.952869000	48.524153000
Si	41.739019000	50.810066000	41.948999000
Si	43.565855000	50.006787000	44.080233000
Si	47.726450000	50.361119000	46.618727000
Si	48.752497000	50.156249000	43.600437000
Si	46.041514000	50.697051000	42.334085000
Si	44.755626000	45.946134000	46.550922000
Si	47.912505000	45.962274000	46.154457000
Si	48.666343000	47.887373000	48.514779000
Si	45.616220000	52.760407000	40.042462000
Si	44.785462000	42.842171000	46.535098000
Si	47.938962000	42.848427000	46.193599000
Si	58.957378000	41.839279000	41.941979000
Si	56.309546000	42.834137000	43.268499000
Si	55.802597000	48.291898000	45.160662000
Si	52.624068000	48.357434000	45.305411000
Si	51.672276000	48.625823000	48.346493000
Si	51.699521000	50.847558000	43.454583000
Si	53.643595000	50.046030000	41.350392000
Si	56.182352000	50.709331000	43.089536000
Si	58.964263000	46.983633000	41.922613000
Si	56.314169000	45.964280000	43.262278000
Si	55.802170000	40.501424000	45.162175000
Si	52.667764000	40.437768000	45.323889000
Si	51.670996000	40.195713000	48.343174000
O	49.991279000	36.609827000	35.894021000
O	47.509765000	35.907576000	36.582059000
O	46.653150000	36.843712000	38.973244000
O	44.972055000	36.673707000	36.919245000
O	44.498838000	36.917784000	34.242922000
O	46.180979000	38.769212000	33.166971000
O	47.969492000	37.043394000	34.161276000
O	48.655808000	38.630453000	32.128626000
O	50.190559000	38.116670000	30.033691000
O	52.205799000	36.847529000	31.266089000
O	52.278684000	39.559048000	30.967383000
O	54.633498000	36.897901000	38.070964000
O	56.175907000	38.744944000	39.148930000
O	56.899650000	38.602153000	35.317464000
O	54.989992000	36.654619000	35.412381000
O	51.815017000	36.838183000	33.924730000
O	52.543533000	36.336120000	36.460039000
O	42.358725000	37.842971000	43.463058000
O	44.970129000	38.496124000	43.503329000
O	48.641034000	38.733002000	45.292329000
O	47.494053000	37.813120000	43.051192000
O	45.600893000	36.896336000	41.470412000
O	50.081135000	37.843165000	43.231113000
O	52.384749000	37.931252000	41.962402000
O	52.055523000	39.408749000	44.206957000
O	53.567732000	38.723080000	39.714112000
O	55.056222000	38.113793000	41.874448000
O	44.496545000	51.865337000	34.271091000
O	46.166964000	49.942594000	33.309112000
O	47.929287000	51.796834000	34.124866000

O	48.576363000	50.122827000	32.140967000
O	48.644325000	48.530095000	29.927416000
O	45.414783000	46.593436000	30.245085000
O	47.902011000	46.009925000	29.510993000
O	45.878311000	44.413736000	28.745170000
O	49.985213000	52.168394000	35.838474000
O	47.528752000	52.852818000	36.591745000
O	46.651178000	51.966925000	38.999505000
O	44.982319000	52.148854000	36.933092000
O	48.608320000	40.308061000	29.974100000
O	45.413334000	42.218953000	30.225287000
O	47.904210000	42.824088000	29.498319000
O	50.440671000	42.237811000	30.054001000
O	51.688916000	44.406829000	31.040113000
O	58.594427000	44.411168000	39.364760000
O	58.129427000	47.831157000	35.380223000
O	58.295410000	46.460902000	37.667501000
O	58.194964000	49.175883000	37.710879000
O	57.002036000	48.626811000	33.132211000
O	56.920707000	50.220167000	35.308740000
O	54.351401000	48.589724000	32.570114000
O	52.565061000	46.764993000	31.796162000
O	54.994807000	52.153808000	35.423119000
O	51.813045000	51.946152000	33.882000000
O	52.532068000	52.513536000	36.408281000
O	50.186389000	50.738960000	30.113590000
O	52.251831000	52.001333000	31.230287000
O	52.277716000	49.276517000	30.998433000
O	54.581272000	51.933105000	38.078371000
O	56.171901000	50.110532000	39.152643000
O	50.427334000	46.578656000	30.126000000
O	58.107356000	40.993885000	35.390621000
O	58.309418000	42.353893000	37.679331000
O	58.213897000	39.634091000	37.706532000
O	56.986449000	40.193860000	33.142553000
O	54.343382000	40.241219000	32.556265000
O	52.523252000	42.051504000	31.808167000
O	50.275301000	40.953088000	48.766121000
O	48.224997000	42.325929000	47.719003000
O	48.168276000	39.584963000	47.763848000
O	42.396135000	50.979482000	43.458749000
O	45.064858000	50.487680000	43.618711000
O	48.561584000	50.088046000	45.219936000
O	47.548299000	50.988318000	42.878436000
O	45.561666000	52.016331000	41.506585000
O	44.403784000	44.392122000	46.905683000
O	46.288570000	46.048883000	45.954117000
O	48.427632000	44.409288000	46.032982000
O	50.279348000	47.880573000	48.799457000
O	48.279157000	46.547284000	47.637495000
O	48.214723000	49.261366000	47.741038000
O	46.318999000	42.744797000	45.915379000
O	58.666172000	41.937605000	40.308147000
O	57.898115000	42.729533000	42.860521000
O	55.893462000	44.401060000	43.463246000
O	56.032748000	46.725226000	44.691361000
O	54.234391000	48.575535000	45.590266000
O	56.197884000	49.264772000	43.892776000

O	51.820182000	48.582803000	46.701193000
O	50.091522000	51.014987000	43.236942000
O	52.389262000	50.898263000	41.957507000
O	52.044003000	49.425881000	44.209821000
O	53.568941000	50.076282000	39.714950000
O	55.057498000	50.693268000	41.875887000
O	58.711472000	46.899124000	40.280875000
O	57.885367000	46.072378000	42.795633000
O	55.966559000	42.074440000	44.684949000
O	54.261816000	40.137550000	45.625478000
O	56.214812000	39.554860000	43.880696000
O	51.832763000	40.226627000	46.700925000
H	48.702871000	34.676568000	34.772566000
H	44.419875000	34.676323000	35.346829000
H	42.683628000	36.258975000	36.022159000
H	43.726523000	38.680216000	32.577941000
H	47.730594000	38.010996000	29.830569000
H	52.421136000	38.002380000	29.030380000
H	58.465353000	38.743363000	40.066967000
H	58.088382000	37.108509000	38.286195000
H	56.651061000	36.758841000	33.558678000
H	57.541582000	36.246995000	35.782467000
H	52.141234000	34.673922000	32.562045000
H	54.071037000	36.146097000	32.821438000
H	54.418082000	34.675478000	36.917895000
H	44.265382000	36.148734000	39.494230000
H	46.085045000	34.677316000	40.196440000
H	42.177304000	36.886192000	41.136491000
H	40.290195000	38.107003000	42.090377000
H	43.614692000	38.689573000	45.487247000
H	46.266760000	38.594605000	46.380864000
H	48.065374000	37.113072000	47.131273000
H	52.195251000	36.881484000	44.271734000
H	55.858296000	36.988842000	44.011824000
H	57.529558000	37.965437000	42.519250000
H	41.636484000	38.780154000	36.634495000
H	40.338249000	40.845235000	36.817795000
H	43.726296000	50.140518000	32.578071000
H	47.730502000	50.809921000	29.830551000
H	42.910974000	46.071594000	29.969678000
H	43.636013000	48.346500000	30.474316000
H	46.055518000	46.690812000	27.779372000
H	40.338195000	47.975592000	36.817799000
H	41.636403000	50.040687000	36.634467000
H	48.702714000	54.144377000	34.772532000
H	44.419585000	54.144606000	35.346948000
H	42.683575000	52.561616000	36.022276000
H	43.636069000	40.474361000	30.474327000
H	42.910998000	42.749253000	29.969681000
H	46.055551000	42.130076000	27.779377000
H	52.768240000	42.805898000	29.339147000
H	60.152182000	47.950749000	36.769108000
H	56.115708000	49.276599000	30.928772000
H	56.049418000	46.904457000	31.507081000
H	57.541465000	52.574079000	35.782446000
H	56.650952000	52.062214000	33.558656000
H	52.141090000	54.147065000	32.562019000
H	54.070917000	52.674925000	32.821418000

H	54.418140000	54.145525000	36.917811000
H	52.421044000	50.818602000	29.030363000
H	58.465269000	50.077736000	40.066952000
H	58.088278000	51.712582000	38.286179000
H	52.768217000	46.015092000	29.339142000
H	60.152234000	40.870368000	36.769119000
H	56.049458000	41.916585000	31.507090000
H	56.115777000	39.544444000	30.928783000
H	48.027948000	40.958435000	49.819979000
H	40.290115000	50.713683000	42.090582000
H	42.176850000	51.934815000	41.136521000
H	43.615015000	50.131729000	45.487215000
H	48.065229000	51.707918000	47.131232000
H	46.266685000	50.226321000	46.380825000
H	40.476352000	46.423809000	42.229530000
H	44.578030000	46.685732000	47.785515000
H	48.027871000	47.862537000	49.819961000
H	46.084739000	54.143668000	40.196331000
H	44.265252000	52.672179000	39.494179000
H	40.476382000	42.397038000	42.229542000
H	44.578055000	42.135174000	47.785513000
H	58.818796000	40.408567000	42.221796000
H	60.309668000	42.345550000	42.255160000
H	56.725235000	48.548904000	46.274187000
H	51.671681000	50.045758000	48.769745000
H	52.721673000	47.848745000	49.007593000
H	52.195144000	51.939533000	44.271718000
H	55.858180000	51.832229000	44.011806000
H	57.529465000	50.855664000	42.519236000
H	60.309639000	46.475584000	42.255156000
H	58.818736000	48.412544000	42.221786000
H	56.725177000	40.272281000	46.274313000
H	52.722235000	40.971966000	49.006966000
H	51.671815000	38.775269000	48.769821000
Si	48.741068000	41.045668000	35.326299000
Si	46.856224000	40.042012000	37.396464000
Si	41.608790000	40.173383000	37.144237000
Si	44.281155000	40.860886000	35.799005000
Si	44.608971000	45.932430000	33.470096000
Si	47.804357000	45.976242000	33.156104000
Si	48.786701000	47.819638000	35.409920000
Si	46.866610000	48.710747000	37.460247000
Si	41.564234000	48.683435000	37.199563000
Si	44.238104000	47.934143000	35.865910000
Si	44.683453000	42.881937000	33.431537000
Si	47.842944000	42.943501000	33.116896000
Si	54.816479000	42.895522000	38.825746000
Si	51.709621000	48.540476000	35.177714000
Si	54.416448000	47.954661000	36.453943000
Si	54.846654000	45.949379000	38.846639000
Si	51.678067000	40.256433000	35.147979000
Si	54.376956000	40.875576000	36.448058000
Si	45.913188000	40.536072000	40.223314000
Si	42.747849000	40.457314000	40.080272000
Si	41.770008000	45.946823000	41.710229000
Si	43.778533000	46.929884000	43.844381000
Si	48.867920000	47.035193000	43.364644000
Si	46.142076000	46.039844000	42.160130000

Si	45.700433000	48.311654000	40.206482000
Si	42.639236000	48.425035000	40.036280000
Si	41.762686000	42.919209000	41.761084000
Si	43.707297000	41.882231000	43.823366000
Si	48.939928000	41.781771000	43.503585000
Al	46.240825000	42.856274000	42.264462000
Si	51.875977000	42.852999000	43.555050000
Si	53.923863000	41.866476000	41.516421000
Si	51.788783000	45.914017000	43.508835000
Si	53.893200000	46.955228000	41.529032000
O	46.955186000	38.491988000	36.862295000
O	44.241147000	39.476194000	34.923814000
O	54.383122000	39.453651000	37.268839000
O	51.556209000	38.759101000	35.809959000
O	42.380945000	39.361637000	41.227500000
O	50.357562000	41.167442000	35.435352000
O	48.128329000	40.900905000	36.835962000
O	48.142563000	42.398011000	34.630417000
O	48.315493000	39.707336000	34.490369000
O	46.944948000	40.117894000	39.019804000
O	45.439758000	40.722281000	36.944174000
O	41.838088000	40.088044000	38.774491000
O	42.813643000	41.094414000	36.469959000
O	44.656337000	42.159249000	34.887955000
O	44.134986000	49.308369000	34.967477000
O	43.712823000	46.726273000	32.350002000
O	44.075687000	44.390735000	33.566071000
O	46.178755000	45.961150000	32.998156000
O	48.414834000	44.468423000	32.954754000
O	48.508771000	46.924638000	32.018896000
O	50.398613000	47.634113000	35.521701000
O	48.202872000	47.920279000	36.936734000
O	48.144666000	46.513817000	34.663213000
O	48.423946000	49.192190000	34.592306000
O	46.853512000	48.617294000	39.083056000
O	46.920612000	50.271908000	36.935748000
O	45.534992000	47.985625000	36.852818000
O	41.611495000	48.857045000	38.840530000
O	42.854263000	47.765131000	36.712738000
O	44.415673000	46.612358000	34.928736000
O	43.755626000	42.064571000	32.354836000
O	46.225855000	42.937847000	32.878921000
O	48.601847000	42.001037000	32.011835000
O	54.265864000	44.429709000	38.661742000
O	56.354423000	42.934752000	39.382820000
O	55.467584000	47.987488000	35.208260000
O	51.868946000	48.690669000	33.553519000
O	52.930255000	47.686519000	35.843483000
O	51.612520000	50.054852000	35.807232000
O	54.841057000	46.705670000	37.407656000
O	54.406842000	49.384840000	37.258787000
O	56.373276000	45.903362000	39.432594000
O	55.443002000	40.835256000	35.213378000
O	51.870246000	40.071516000	33.531958000
O	52.891327000	41.115220000	35.820354000
O	54.773065000	42.149960000	37.380761000
O	46.298657000	42.136122000	40.555123000
O	44.359015000	40.364143000	39.732179000

O	46.257817000	39.551755000	41.460040000
O	42.423294000	41.979978000	40.574891000
O	42.348380000	49.432622000	41.281563000
O	43.209424000	48.453488000	43.676675000
O	48.836402000	48.632427000	42.991825000
O	42.842473000	45.929035000	42.959322000
O	41.586325000	44.423846000	41.122004000
O	43.705431000	46.521933000	45.427043000
O	45.335755000	46.893726000	43.324320000
O	48.643674000	46.858083000	44.987662000
O	47.757396000	46.224298000	42.479293000
O	45.698916000	44.496380000	42.132994000
O	45.805818000	46.758251000	40.704708000
O	44.209503000	48.602716000	39.556897000
O	45.986226000	49.331051000	41.444658000
O	42.354093000	46.875065000	40.478371000
O	42.986252000	40.402500000	43.729567000
O	48.750777000	40.203011000	43.065391000
O	42.744156000	42.981722000	43.075552000
O	43.746930000	42.259904000	45.419455000
O	45.166360000	41.809734000	43.130494000
O	48.784460000	41.901975000	45.152314000
O	47.904584000	42.767571000	42.762029000
O	53.525324000	40.322822000	41.874303000
O	50.470111000	42.194777000	43.061703000
O	52.880389000	42.887836000	42.249832000
O	51.665868000	44.388461000	44.085780000
O	53.879813000	42.070873000	39.888480000
O	55.450471000	42.155114000	42.044629000
O	52.375153000	46.841020000	44.729017000
O	53.497964000	48.498975000	41.889414000
O	50.339075000	46.436135000	42.950070000
O	52.815434000	45.938603000	42.220175000
O	53.888312000	46.770624000	39.896502000
O	55.402780000	46.654056000	42.085216000
O	52.544040000	41.982880000	44.777963000
Ga	47.336519000	43.191137000	39.250540000
C	50.270111000	42.757685000	38.428473000
H	50.330435000	41.763525000	37.973106000
C	51.037081000	43.772709000	37.980110000
H	46.676749000	43.325157000	37.851477000
H	51.723018000	43.610746000	37.137258000
C	49.375586000	42.916999000	39.603472000
C	49.159264000	44.261718000	40.101529000
H	49.442162000	42.129166000	40.374049000
H	47.485377000	44.653443000	40.044088000
H	49.095285000	44.420140000	41.188280000
C	50.998639000	45.124364000	38.647664000
C	49.601482000	45.421025000	39.222963000
H	51.270432000	45.929605000	37.939214000
H	51.759208000	45.156019000	39.455934000
H	49.568095000	46.366116000	39.793391000
H	48.907109000	45.542614000	38.368686000

I17-GaH_2

Electronic energy (r^2SCAN-D4/def2-SVP): -48411.85781235 E_h
Electronic energy (ωB97X-D4rev/def2-mTZVPP): -48455.936685078800 E_h
G_{corr} (673.15 K, 1 atm): 2.03142448 E_h
Number of atoms: 393

Cartesian coordinates (Å)

Si	48.557416000	36.027355000	35.312252000
Si	46.528256000	36.978410000	37.338835000
Si	44.100566000	36.092577000	35.633473000
Si	44.622439000	38.495923000	33.722635000
Si	47.765546000	38.530954000	33.532751000
Si	48.743482000	38.770996000	30.512262000
Si	51.816390000	38.141097000	30.323344000
Si	54.675799000	38.486364000	38.526037000
Si	57.777289000	38.493440000	38.799908000
Si	56.556902000	37.020723000	34.995713000
Si	52.595934000	36.085030000	32.667037000
Si	51.477169000	37.154397000	35.495174000
Si	54.139096000	36.099687000	36.722644000
Si	45.625610000	36.080027000	40.029962000
Si	41.745538000	38.020655000	41.949476000
Si	43.567968000	38.897248000	44.082267000
Si	47.726966000	38.467187000	46.627893000
Si	48.755200000	38.690833000	43.616468000
Si	46.056807000	38.222434000	42.310709000
Si	51.718333000	38.002279000	43.461542000
Si	53.674059000	38.784472000	41.348035000
Si	56.184291000	38.105586000	43.084282000
Si	44.611065000	50.308636000	33.741482000
Si	47.761264000	50.277337000	33.560339000
Si	48.743570000	50.061458000	30.523820000
Si	43.865201000	46.935609000	30.715822000
Si	48.871088000	46.965258000	30.430267000
Si	46.323030000	45.965502000	29.027999000
Si	48.555156000	52.786628000	35.307359000
Si	46.530746000	51.838727000	37.357073000
Si	44.103307000	52.727866000	35.637388000
Si	43.867859000	41.886491000	30.710491000
Si	48.867145000	41.869517000	30.429138000
Si	46.325377000	42.859666000	29.024553000
Si	51.888515000	42.860055000	30.530843000
Si	57.964164000	42.900925000	39.174130000
Si	58.734535000	47.858892000	36.911148000
Si	56.878169000	48.673951000	34.759459000
Si	55.869065000	48.297978000	31.974468000
Si	52.766307000	48.326538000	32.241606000
Si	56.556620000	51.798456000	34.996228000
Si	52.600568000	52.738531000	32.655746000
Si	51.488429000	51.659690000	35.469328000
Si	54.129403000	52.728902000	36.710822000
Si	51.807407000	50.700987000	30.331607000
Si	54.674253000	50.346761000	38.535199000
Si	57.776614000	50.332368000	38.803342000
Si	51.888147000	45.951971000	30.528679000
Si	57.966798000	45.917059000	39.182832000
Si	58.743790000	40.958437000	36.906003000

Si	56.907725000	40.144141000	34.754415000
Si	55.881339000	40.523838000	31.975467000
Si	52.789427000	40.482215000	32.204845000
Si	48.680266000	40.962213000	48.529364000
Si	41.746169000	50.776876000	41.945535000
Si	43.705092000	49.981054000	44.078130000
Si	47.725687000	50.354756000	46.613254000
Si	48.768891000	50.139955000	43.579542000
Si	46.075622000	50.755953000	42.357783000
Si	44.898829000	46.014531000	46.539622000
Si	47.945841000	46.032303000	46.135407000
Si	48.698505000	47.884434000	48.522614000
Si	45.620253000	52.767939000	40.026531000
Si	44.898199000	42.803326000	46.536931000
Si	47.945939000	42.794858000	46.202522000
Si	58.954554000	41.843407000	41.953654000
Si	56.338591000	42.837044000	43.291232000
Si	55.785610000	48.296723000	45.175348000
Si	52.631284000	48.345878000	45.340884000
Si	51.675159000	48.630736000	48.363215000
Si	51.730780000	50.827076000	43.464674000
Si	53.667844000	50.029647000	41.347343000
Si	56.182307000	50.710715000	43.089833000
Si	58.958436000	46.979220000	41.942013000
Si	56.329321000	45.968325000	43.271414000
Si	55.793804000	40.500921000	45.172380000
Si	52.673176000	40.448517000	45.350244000
Si	51.674234000	40.191733000	48.355107000
O	49.978339000	36.626522000	35.883098000
O	47.505623000	35.904334000	36.578118000
O	46.661557000	36.839052000	38.963865000
O	44.967072000	36.664178000	36.929320000
O	44.490273000	36.943381000	34.260496000
O	46.169790000	38.829883000	33.247211000
O	47.936954000	37.018597000	34.145308000
O	48.607021000	38.644272000	32.141960000
O	50.199056000	38.173200000	30.078798000
O	52.208798000	36.867502000	31.272060000
O	52.368135000	39.579575000	30.907380000
O	54.614671000	36.906919000	38.075443000
O	56.173818000	38.762884000	39.117422000
O	56.913100000	38.600140000	35.306398000
O	54.991875000	36.659182000	35.419086000
O	51.808469000	36.827540000	33.922803000
O	52.536476000	36.351314000	36.457467000
O	42.398298000	37.914872000	43.463305000
O	45.057648000	38.422674000	43.578137000
O	48.586377000	38.659702000	45.235873000
O	47.551726000	37.882235000	42.857239000
O	45.571172000	36.932846000	41.436860000
O	50.109467000	37.878893000	43.204683000
O	52.418204000	37.943863000	41.968837000
O	52.075264000	39.418206000	44.225900000
O	53.579228000	38.758687000	39.711497000
O	55.082628000	38.103532000	41.849636000
O	44.494571000	51.866556000	34.271244000
O	46.165747000	49.944603000	33.306322000
O	47.908586000	51.806288000	34.143057000

O	48.579055000	50.151081000	32.155459000
O	48.608291000	48.515078000	29.980150000
O	45.410503000	46.589168000	30.244851000
O	47.898267000	46.000565000	29.522281000
O	45.875231000	44.412663000	28.738420000
O	49.979653000	52.167570000	35.844583000
O	47.529110000	52.894993000	36.598125000
O	46.652004000	51.970967000	38.987372000
O	44.976549000	52.174917000	36.938105000
O	48.568883000	40.322078000	29.993955000
O	45.413449000	42.229138000	30.237926000
O	47.902297000	42.837633000	29.515617000
O	50.430021000	42.221782000	30.114279000
O	51.681017000	44.406658000	31.034695000
O	58.589746000	44.408813000	39.354939000
O	58.131894000	47.835494000	35.381481000
O	58.292655000	46.475647000	37.680859000
O	58.212036000	49.189840000	37.704691000
O	56.997963000	48.628546000	33.135153000
O	56.906096000	50.218207000	35.311830000
O	54.348133000	48.583420000	32.578809000
O	52.561246000	46.761200000	31.797674000
O	54.992166000	52.161200000	35.418528000
O	51.807480000	51.953062000	33.885086000
O	52.528342000	52.512969000	36.409884000
O	50.185727000	50.706734000	30.108250000
O	52.236943000	51.995100000	31.234685000
O	52.294560000	49.271309000	30.990360000
O	54.576673000	51.918271000	38.070570000
O	56.172514000	50.098047000	39.137878000
O	50.427643000	46.586544000	30.118826000
O	58.171331000	40.974094000	35.365120000
O	58.313375000	42.342519000	37.677077000
O	58.224717000	39.625449000	37.695699000
O	57.015576000	40.190111000	33.129100000
O	54.363354000	40.247930000	32.589910000
O	52.555869000	42.057572000	31.809387000
O	50.285771000	40.922033000	48.837305000
O	48.346393000	42.371637000	47.734260000
O	48.201521000	39.630302000	47.694623000
O	42.460085000	50.845996000	43.434993000
O	45.160029000	50.649457000	43.702456000
O	48.525736000	50.087042000	45.191592000
O	47.617613000	51.000900000	42.800284000
O	45.571850000	52.036348000	41.495748000
O	44.918820000	44.413071000	46.855951000
O	46.375236000	46.504547000	45.991068000
O	48.108315000	44.413207000	46.009245000
O	50.289010000	47.933156000	48.895984000
O	48.462937000	46.527126000	47.608566000
O	48.228368000	49.234474000	47.711545000
O	46.377696000	42.348607000	45.956605000
O	58.625604000	41.951173000	40.327210000
O	57.936225000	42.734865000	42.919073000
O	55.906486000	44.403653000	43.463323000
O	55.977872000	46.731866000	44.684993000
O	54.232269000	48.586132000	45.659889000
O	56.163860000	49.273289000	43.905248000

O	51.779939000	48.556324000	46.714431000
O	50.123201000	50.974131000	43.242322000
O	52.414633000	50.874298000	41.967980000
O	52.075125000	49.415041000	44.233770000
O	53.574639000	50.060715000	39.713756000
O	55.076564000	50.698915000	41.859402000
O	58.653187000	46.879531000	40.311260000
O	57.919420000	46.078841000	42.874236000
O	55.954821000	42.076182000	44.697811000
O	54.262222000	40.137587000	45.669106000
O	56.188326000	39.550849000	43.887264000
O	51.805619000	40.238922000	46.710412000
H	48.702859000	34.676577000	34.772548000
H	44.419861000	34.676336000	35.346807000
H	42.683615000	36.258990000	36.022134000
H	43.726513000	38.680241000	32.577930000
H	47.730590000	38.011010000	29.830554000
H	52.421134000	38.002389000	29.030370000
H	58.465339000	38.743356000	40.066965000
H	58.088367000	37.108504000	38.286191000
H	56.651050000	36.758841000	33.558672000
H	57.541568000	36.246992000	35.782462000
H	52.141222000	34.673929000	32.562032000
H	54.071027000	36.146101000	32.821429000
H	54.418067000	34.675479000	36.917885000
H	44.265364000	36.148745000	39.494209000
H	46.085021000	34.677322000	40.196421000
H	42.177283000	36.886198000	41.136472000
H	40.290176000	38.107022000	42.090354000
H	43.614674000	38.689566000	45.487231000
H	46.266736000	38.594609000	46.380846000
H	48.065345000	37.113073000	47.131258000
H	52.195230000	36.881483000	44.271723000
H	55.858273000	36.988836000	44.011818000
H	57.529538000	37.965430000	42.519247000
H	41.636481000	38.780156000	36.634490000
H	40.338250000	40.845238000	36.817793000
H	43.726301000	50.140527000	32.578066000
H	47.730513000	50.809934000	29.830545000
H	42.910979000	46.071614000	29.969666000
H	43.636019000	48.346516000	30.474318000
H	46.055526000	46.690830000	27.779360000
H	40.338195000	47.975595000	36.817796000
H	41.636405000	50.040688000	36.634466000
H	48.702722000	54.144385000	34.772529000
H	44.419595000	54.144618000	35.346938000
H	42.683580000	52.561633000	36.022265000
H	43.636067000	40.474380000	30.474307000
H	42.910997000	42.749275000	29.969679000
H	46.055554000	42.130092000	27.779363000
H	52.768242000	42.805904000	29.339141000
H	60.152182000	47.950744000	36.769114000
H	56.115715000	49.276603000	30.928775000
H	56.049422000	46.904459000	31.507082000
H	57.541470000	52.574077000	35.782453000
H	56.650960000	52.062215000	33.558661000
H	52.141101000	54.147071000	32.562021000
H	54.070927000	52.674929000	32.821421000

H	54.418146000	54.145526000	36.917815000
H	52.421057000	50.818611000	29.030362000
H	58.465266000	50.077729000	40.066959000
H	58.088280000	51.712577000	38.286186000
H	52.768222000	46.015100000	29.339137000
H	60.152225000	40.870361000	36.769122000
H	56.049457000	41.916588000	31.507088000
H	56.115774000	39.544448000	30.928778000
H	48.027922000	40.958434000	49.819966000
H	40.290112000	50.713692000	42.090566000
H	42.176852000	51.934818000	41.136504000
H	43.615007000	50.131740000	45.487204000
H	48.065218000	51.707918000	47.131226000
H	46.266674000	50.226324000	46.380815000
H	40.476351000	46.423810000	42.229527000
H	44.578021000	46.685736000	47.785509000
H	48.027853000	47.862536000	49.819953000
H	46.084740000	54.143675000	40.196325000
H	44.265253000	52.672189000	39.494169000
H	40.476381000	42.397040000	42.229540000
H	44.578046000	42.135178000	47.785506000
H	58.818778000	40.408558000	42.221797000
H	60.309655000	42.345538000	42.255164000
H	56.725224000	48.548895000	46.274190000
H	51.671666000	50.045753000	48.769743000
H	52.721655000	47.848739000	49.007591000
H	52.195139000	51.939532000	44.271718000
H	55.858175000	51.832223000	44.011810000
H	57.529460000	50.855657000	42.519242000
H	60.309630000	46.475575000	42.255163000
H	58.818728000	48.412536000	42.221792000
H	56.725155000	40.272271000	46.274310000
H	52.722210000	40.971960000	49.006959000
H	51.671787000	38.775264000	48.769810000
Si	48.780112000	41.010159000	35.364742000
Si	46.902869000	40.038276000	37.394863000
Si	41.581242000	40.152840000	37.187327000
Si	44.275519000	40.879424000	35.846796000
Si	44.613135000	45.947355000	33.469023000
Si	47.799312000	45.953816000	33.195155000
Si	48.770248000	47.833832000	35.433868000
Si	46.860905000	48.774710000	37.461291000
Si	41.566602000	48.678902000	37.185167000
Si	44.252265000	47.943149000	35.874664000
Si	44.615042000	42.895833000	33.455308000
Si	47.794509000	42.908652000	33.184160000
Si	54.788625000	42.883275000	38.813569000
Si	51.693089000	48.540077000	35.166338000
Si	54.400759000	47.936889000	36.442636000
Si	54.809287000	45.921713000	38.831162000
Si	51.684735000	40.250073000	35.064968000
Si	54.388591000	40.870198000	36.402105000
Si	45.755666000	40.509543000	40.126233000
Si	42.660074000	40.428775000	40.052192000
Si	41.752926000	45.942880000	41.681486000
Si	43.911179000	46.794310000	43.798102000
Si	48.969459000	47.009237000	43.302780000
Si	46.321150000	45.983330000	41.954231000

Si	45.692720000	48.425440000	40.206086000
Si	42.616296000	48.404890000	40.025900000
Si	41.754376000	42.909511000	41.745525000
Si	43.768156000	42.009825000	43.855241000
Si	49.028740000	41.818727000	43.502209000
Al	46.380659000	42.789818000	42.125913000
Si	51.940756000	42.863857000	43.577509000
Si	53.991137000	41.860879000	41.548820000
Si	51.863845000	45.917643000	43.510104000
Si	53.958908000	46.946920000	41.526628000
O	46.933256000	38.491566000	36.832788000
O	44.183724000	39.504197000	34.954593000
O	54.363007000	39.447226000	37.230732000
O	51.544966000	38.774388000	35.775591000
O	42.370657000	39.383532000	41.261633000
O	50.390850000	41.207470000	35.326167000
O	48.234684000	40.848657000	36.902725000
O	48.069942000	42.318557000	34.684544000
O	48.375862000	39.646690000	34.552279000
O	46.849918000	40.015341000	39.021671000
O	45.560027000	40.817335000	36.859217000
O	41.637383000	40.046897000	38.836525000
O	42.876142000	41.051966000	36.673019000
O	44.474079000	42.205378000	34.919203000
O	44.142060000	49.315544000	34.975935000
O	43.720239000	46.760437000	32.359420000
O	44.038119000	44.420876000	33.554557000
O	46.177901000	45.947759000	32.983959000
O	48.386123000	44.430938000	33.066051000
O	48.540301000	46.858272000	32.044951000
O	50.380819000	47.638439000	35.522482000
O	48.188094000	47.958966000	36.964144000
O	48.097578000	46.539360000	34.693900000
O	48.417249000	49.203355000	34.603937000
O	46.826873000	48.807946000	39.093958000
O	46.915571000	50.314328000	36.878524000
O	45.518820000	48.021580000	36.904402000
O	41.603149000	48.848017000	38.827677000
O	42.855263000	47.757701000	36.701776000
O	44.465246000	46.626265000	34.938334000
O	43.720574000	42.078467000	32.351245000
O	46.175108000	42.916458000	32.954833000
O	48.546579000	42.002647000	32.043110000
O	54.206768000	44.409213000	38.665971000
O	56.340830000	42.949372000	39.325993000
O	55.472550000	47.970465000	35.214167000
O	51.846744000	48.695364000	33.540008000
O	52.923446000	47.686809000	35.807414000
O	51.602854000	50.053921000	35.800879000
O	54.795120000	46.676358000	37.391541000
O	54.395945000	49.364734000	37.254540000
O	56.345421000	45.866294000	39.391220000
O	55.527026000	40.869491000	35.237080000
O	51.842941000	40.009594000	33.448701000
O	52.962382000	41.081404000	35.642060000
O	54.707167000	42.137850000	37.371116000
O	46.001332000	42.117636000	40.469447000
O	44.229224000	40.276820000	39.555307000

O	46.032375000	39.643758000	41.477993000
O	42.415742000	41.963440000	40.566146000
O	42.267379000	49.353704000	41.298968000
O	43.668690000	48.401627000	43.605768000
O	48.806269000	48.609239000	42.980730000
O	42.807602000	45.961645000	42.941842000
O	41.569187000	44.415429000	41.107141000
O	43.785511000	46.397000000	45.383041000
O	45.409951000	46.373430000	43.254919000
O	48.775890000	46.751897000	44.915991000
O	47.887625000	46.181685000	42.375975000
O	46.034833000	44.433207000	41.448420000
O	45.877030000	46.847724000	40.627235000
O	44.187373000	48.647296000	39.577831000
O	45.911026000	49.345520000	41.532973000
O	42.366359000	46.837342000	40.434958000
O	43.218962000	40.463929000	43.719022000
O	48.750567000	40.244884000	43.106280000
O	42.731915000	43.003117000	43.058609000
O	43.731861000	42.436109000	45.444503000
O	45.248547000	42.160132000	43.242610000
O	48.912978000	41.995723000	45.147803000
O	47.986245000	42.785005000	42.734205000
O	53.578880000	40.322619000	41.917991000
O	50.561956000	42.175523000	43.058499000
O	52.993202000	42.903794000	42.315626000
O	51.670837000	44.399236000	44.087068000
O	53.933203000	42.050777000	39.928941000
O	55.533140000	42.136178000	42.044201000
O	52.411066000	46.829287000	44.760421000
O	53.546006000	48.485131000	41.899296000
O	50.466118000	46.507395000	42.881725000
O	52.946715000	45.907473000	42.276376000
O	53.887913000	46.762610000	39.898332000
O	55.490936000	46.666232000	42.041755000
O	52.573815000	42.002097000	44.825491000
Ga	45.529304000	43.899660000	39.543856000
C	51.316319000	41.251885000	38.443883000
H	51.886218000	40.493528000	37.895622000
C	51.361594000	42.562426000	38.110714000
H	43.975089000	43.980773000	39.413468000
H	51.955190000	42.912919000	37.256235000
C	50.501356000	40.782449000	39.559443000
C	49.560622000	41.593910000	40.088567000
H	50.603705000	39.743249000	39.902440000
H	46.707977000	44.265978000	38.582053000
H	48.870010000	41.196805000	40.838232000
C	50.649817000	43.576761000	38.971214000
C	49.361369000	42.991239000	39.563748000
H	50.430622000	44.500849000	38.403756000
H	51.337123000	43.885399000	39.793937000
H	48.967947000	43.659412000	40.353372000
H	48.578248000	42.950256000	38.772920000

TS12-GaH$_2$

Electronic energy (r^{2}SCAN-D4/def2-SVP): -48411.76368831 E_h
Electronic energy (ωB97X-D4rev/def2-mTZVPP): -48455.846446621200 E_h
G_{corr} (673.15 K, 1 atm): 2.02955322 E_h
Number of atoms: 393

Cartesian coordinates (Å)

Si	48.556301000	36.016912000	35.323122000
Si	46.528179000	36.943269000	37.333859000
Si	44.097489000	36.086734000	35.628628000
Si	44.634454000	38.470942000	33.700400000
Si	47.764193000	38.503314000	33.512945000
Si	48.750305000	38.758987000	30.510825000
Si	51.810646000	38.136067000	30.321775000
Si	54.674879000	38.492591000	38.521596000
Si	57.777059000	38.492780000	38.800424000
Si	56.551654000	37.019146000	34.996160000
Si	52.593892000	36.085410000	32.669897000
Si	51.474121000	37.150072000	35.501353000
Si	54.136446000	36.096795000	36.718081000
Si	45.626147000	36.064490000	40.029904000
Si	41.748663000	38.040608000	41.929991000
Si	43.596859000	38.890918000	44.078579000
Si	47.728294000	38.472969000	46.639230000
Si	48.758605000	38.697585000	43.645848000
Si	46.084252000	38.118710000	42.367003000
Si	51.737111000	38.014292000	43.466251000
Si	53.681793000	38.781791000	41.343603000
Si	56.184995000	38.106391000	43.085288000
Si	44.607184000	50.303323000	33.748659000
Si	47.755853000	50.282729000	33.569774000
Si	48.742692000	50.068045000	30.531807000
Si	43.864953000	46.932868000	30.717371000
Si	48.870051000	46.973526000	30.438104000
Si	46.325564000	45.968248000	29.030337000
Si	48.549866000	52.786867000	35.317609000
Si	46.528755000	51.829241000	37.381422000
Si	44.106179000	52.727373000	35.643957000
Si	43.869786000	41.885297000	30.710097000
Si	48.880044000	41.870207000	30.423048000
Si	46.327704000	42.858867000	29.022572000
Si	51.884606000	42.859716000	30.524393000
Si	57.962868000	42.901218000	39.173119000
Si	58.734047000	47.858153000	36.911244000
Si	56.880002000	48.674391000	34.758379000
Si	55.869429000	48.297929000	31.974260000
Si	52.767169000	48.324513000	32.242004000
Si	56.557335000	51.798000000	34.996642000
Si	52.601745000	52.738950000	32.653881000
Si	51.489044000	51.660606000	35.464702000
Si	54.127121000	52.729394000	36.711753000
Si	51.808725000	50.701664000	30.332942000
Si	54.671459000	50.349150000	38.538222000
Si	57.775791000	50.332773000	38.803615000
Si	51.886752000	45.952211000	30.528897000
Si	57.969010000	45.919175000	39.183415000
Si	58.741728000	40.957172000	36.905794000

Si	56.883749000	40.146054000	34.761523000
Si	55.872728000	40.524003000	31.974227000
Si	52.772640000	40.483729000	32.214672000
Si	48.678230000	40.974812000	48.532880000
Si	41.750792000	50.764529000	41.949223000
Si	43.762442000	49.957039000	44.081150000
Si	47.723519000	50.360284000	46.615043000
Si	48.783788000	50.208047000	43.584554000
Si	46.080894000	50.837548000	42.379128000
Si	44.904879000	46.009153000	46.543472000
Si	47.952652000	46.047609000	46.126553000
Si	48.698607000	47.885739000	48.521293000
Si	45.611944000	52.779889000	40.040165000
Si	44.932035000	42.795868000	46.534207000
Si	47.977334000	42.811294000	46.214352000
Si	58.954678000	41.843267000	41.950003000
Si	56.335327000	42.840808000	43.282918000
Si	55.784531000	48.297716000	45.172104000
Si	52.617269000	48.343954000	45.327219000
Si	51.676092000	48.630730000	48.361003000
Si	51.727809000	50.838107000	43.463743000
Si	53.663410000	50.027551000	41.351347000
Si	56.182781000	50.710657000	43.090192000
Si	58.959429000	46.979917000	41.940509000
Si	56.328061000	45.970557000	43.271609000
Si	55.796362000	40.507670000	45.174908000
Si	52.683280000	40.463741000	45.358937000
Si	51.673533000	40.192602000	48.354387000
O	49.973778000	36.626616000	35.891336000
O	47.507964000	35.860269000	36.589893000
O	46.654675000	36.832818000	38.963005000
O	44.963928000	36.641991000	36.931607000
O	44.495327000	36.923662000	34.250304000
O	46.174210000	38.785262000	33.188838000
O	47.929214000	37.006525000	34.159915000
O	48.638431000	38.598428000	32.140616000
O	50.197629000	38.162899000	30.049307000
O	52.200242000	36.864357000	31.274262000
O	52.339586000	39.574403000	30.925963000
O	54.616715000	36.916991000	38.061033000
O	56.173150000	38.765767000	39.114238000
O	56.886533000	38.602460000	35.316392000
O	54.986010000	36.648213000	35.409483000
O	51.806768000	36.828173000	33.927519000
O	52.532177000	36.339557000	36.458774000
O	42.401955000	37.951455000	43.445228000
O	45.074286000	38.333027000	43.620310000
O	48.621849000	38.670786000	45.267680000
O	47.579276000	37.814486000	42.930090000
O	45.603138000	36.841333000	41.479320000
O	50.128736000	37.935120000	43.201314000
O	52.433166000	37.938620000	41.972802000
O	52.119733000	39.422358000	44.228407000
O	53.575714000	38.754092000	39.707858000
O	55.093536000	38.107634000	41.841576000
O	44.495557000	51.861082000	34.280049000
O	46.163201000	49.937221000	33.316185000
O	47.892353000	51.812965000	34.152857000

O	48.572716000	50.159867000	32.163034000
O	48.611440000	48.522487000	29.984601000
O	45.411300000	46.591033000	30.245859000
O	47.899620000	46.006874000	29.529157000
O	45.882337000	44.413803000	28.741643000
O	49.979710000	52.165043000	35.837874000
O	47.539969000	52.877666000	36.623477000
O	46.638756000	51.955275000	39.015589000
O	44.981711000	52.182745000	36.947040000
O	48.569132000	40.317455000	30.020390000
O	45.411878000	42.220716000	30.226344000
O	47.903911000	42.830609000	29.515246000
O	50.437208000	42.217674000	30.076419000
O	51.672754000	44.405375000	31.029609000
O	58.587404000	44.408579000	39.355922000
O	58.131840000	47.833617000	35.381502000
O	58.293190000	46.473165000	37.679616000
O	58.208187000	49.186712000	37.706739000
O	56.999583000	48.627330000	33.134206000
O	56.911864000	50.218391000	35.310371000
O	54.348896000	48.583381000	32.578944000
O	52.566052000	46.758380000	31.796892000
O	54.993621000	52.156821000	35.423774000
O	51.807457000	51.949652000	33.879518000
O	52.527082000	52.518972000	36.402957000
O	50.185879000	50.713682000	30.119326000
O	52.245649000	51.996881000	31.230578000
O	52.293709000	49.270971000	30.992435000
O	54.565452000	51.920697000	38.076033000
O	56.171322000	50.104190000	39.138908000
O	50.426582000	46.592223000	30.128283000
O	58.149508000	40.973311000	35.372522000
O	58.311496000	42.341783000	37.676683000
O	58.230740000	39.622652000	37.697310000
O	56.997382000	40.187492000	33.136791000
O	54.351527000	40.251409000	32.581071000
O	52.533719000	42.056330000	31.811988000
O	50.284415000	40.922457000	48.833377000
O	48.347153000	42.397496000	47.757589000
O	48.180641000	39.646616000	47.702820000
O	42.497456000	50.787393000	43.423902000
O	45.192459000	50.697726000	43.739501000
O	48.524344000	50.123598000	45.191087000
O	47.631804000	51.059925000	42.799129000
O	45.561766000	52.132592000	41.548951000
O	44.942742000	44.405412000	46.856715000
O	46.377291000	46.509237000	45.988616000
O	48.135788000	44.427566000	45.996514000
O	50.289388000	47.934430000	48.894377000
O	48.462431000	46.528248000	47.607964000
O	48.231176000	49.233162000	47.704277000
O	46.413995000	42.350170000	45.945907000
O	58.624355000	41.948169000	40.323520000
O	57.931649000	42.737343000	42.906922000
O	55.903512000	44.405886000	43.460851000
O	55.984905000	46.731257000	44.688289000
O	54.222568000	48.587066000	45.629410000
O	56.175114000	49.272209000	43.904120000

O	51.785035000	48.553130000	46.712759000
O	50.127744000	51.061062000	43.258771000
O	52.406439000	50.871271000	41.964448000
O	52.039785000	49.413615000	44.228332000
O	53.575457000	50.052710000	39.717424000
O	55.071510000	50.697435000	41.864567000
O	58.661504000	46.880970000	40.308223000
O	57.916427000	46.078143000	42.866712000
O	55.961066000	42.078452000	44.690328000
O	54.269640000	40.164123000	45.694943000
O	56.173963000	39.550161000	43.889955000
O	51.803700000	40.244327000	46.709220000
H	48.702871000	34.676568000	34.772566000
H	44.419875000	34.676323000	35.346829000
H	42.683628000	36.258975000	36.022159000
H	43.726523000	38.680216000	32.577941000
H	47.730594000	38.010996000	29.830569000
H	52.421136000	38.002380000	29.030380000
H	58.465353000	38.743363000	40.066967000
H	58.088382000	37.108509000	38.286195000
H	56.651061000	36.758841000	33.558678000
H	57.541582000	36.246995000	35.782467000
H	52.141234000	34.673922000	32.562045000
H	54.071037000	36.146097000	32.821438000
H	54.418082000	34.675478000	36.917895000
H	44.265382000	36.148734000	39.494230000
H	46.085045000	34.677316000	40.196440000
H	42.177304000	36.886192000	41.136491000
H	40.290195000	38.107003000	42.090377000
H	43.614692000	38.689573000	45.487247000
H	46.266760000	38.594605000	46.380864000
H	48.065374000	37.113072000	47.131273000
H	52.195251000	36.881484000	44.271734000
H	55.858296000	36.988842000	44.011824000
H	57.529558000	37.965437000	42.519250000
H	41.636484000	38.780154000	36.634495000
H	40.338249000	40.845235000	36.817795000
H	43.726296000	50.140518000	32.578071000
H	47.730503000	50.809921000	29.830551000
H	42.910974000	46.071594000	29.969678000
H	43.636013000	48.346500000	30.474316000
H	46.055518000	46.690813000	27.779371000
H	40.338195000	47.975592000	36.817799000
H	41.636403000	50.040687000	36.634467000
H	48.702714000	54.144377000	34.772532000
H	44.419585000	54.144606000	35.346948000
H	42.683575000	52.561616000	36.022276000
H	43.636069000	40.474361000	30.474327000
H	42.910998000	42.749253000	29.969681000
H	46.055551000	42.130076000	27.779377000
H	52.768240000	42.805898000	29.339147000
H	60.152183000	47.950749000	36.769108000
H	56.115708000	49.276599000	30.928772000
H	56.049418000	46.904457000	31.507081000
H	57.541465000	52.574079000	35.782446000
H	56.650952000	52.062214000	33.558656000
H	52.141090000	54.147065000	32.562019000
H	54.070917000	52.674925000	32.821418000

H	54.418140000	54.145525000	36.917811000
H	52.421044000	50.818602000	29.030363000
H	58.465269000	50.077736000	40.066952000
H	58.088278000	51.712582000	38.286179000
H	52.768217000	46.015092000	29.339142000
H	60.152234000	40.870368000	36.769119000
H	56.049458000	41.916585000	31.507090000
H	56.115777000	39.544444000	30.928783000
H	48.027948000	40.958435000	49.819979000
H	40.290115000	50.713683000	42.090582000
H	42.176850000	51.934815000	41.136521000
H	43.615015000	50.131729000	45.487215000
H	48.065229000	51.707918000	47.131232000
H	46.266685000	50.226321000	46.380825000
H	40.476352000	46.423809000	42.229530000
H	44.578030000	46.685732000	47.785515000
H	48.027871000	47.862537000	49.819961000
H	46.084739000	54.143668000	40.196331000
H	44.265252000	52.672179000	39.494179000
H	40.476382000	42.397038000	42.229542000
H	44.578055000	42.135174000	47.785513000
H	58.818796000	40.408567000	42.221796000
H	60.309668000	42.345550000	42.255160000
H	56.725235000	48.548904000	46.274187000
H	51.671681000	50.045758000	48.769745000
H	52.721673000	47.848745000	49.007593000
H	52.195144000	51.939533000	44.271718000
H	55.858180000	51.832229000	44.011806000
H	57.529465000	50.855664000	42.519236000
H	60.309639000	46.475584000	42.255156000
H	58.818736000	48.412544000	42.221786000
H	56.725177000	40.272281000	46.274313000
H	52.722235000	40.971967000	49.006966000
H	51.671815000	38.775269000	48.769821000
Si	48.743618000	40.996879000	35.347701000
Si	46.871377000	39.969175000	37.376014000
Si	41.607495000	40.176263000	37.147899000
Si	44.293873000	40.862711000	35.811910000
Si	44.612032000	45.937921000	33.478344000
Si	47.801404000	45.971981000	33.208172000
Si	48.771972000	47.849171000	35.449147000
Si	46.855108000	48.753208000	37.485915000
Si	41.560334000	48.683128000	37.189901000
Si	44.247794000	47.937670000	35.883383000
Si	44.669681000	42.889891000	33.438260000
Si	47.824564000	42.931622000	33.169279000
Si	54.785383000	42.902362000	38.814606000
Si	51.696129000	48.540647000	35.169058000
Si	54.403212000	47.942395000	36.443647000
Si	54.812325000	45.937645000	38.838586000
Si	51.669238000	40.248263000	35.092672000
Si	54.370197000	40.890280000	36.408196000
Si	45.888060000	40.413548000	40.175010000
Si	42.728106000	40.487803000	40.068661000
Si	41.752598000	45.968305000	41.673776000
Si	43.944920000	46.746611000	43.806052000
Si	48.978697000	47.080292000	43.308810000
Si	46.319629000	45.996222000	41.946632000

Si	45.640469000	48.486217000	40.231662000
Si	42.596755000	48.414273000	40.015863000
Si	41.745622000	42.934015000	41.765605000
Si	43.788218000	42.037936000	43.814724000
Si	49.028381000	41.816598000	43.549449000
Al	46.450750000	42.896216000	42.100309000
Si	51.925780000	42.853045000	43.567420000
Si	53.982749000	41.865490000	41.538553000
Si	51.843159000	45.915737000	43.498303000
Si	53.950891000	46.950209000	41.532815000
O	46.946572000	38.437544000	36.780753000
O	44.218500000	39.479803000	34.932944000
O	54.355868000	39.473587000	37.245174000
O	51.548551000	38.767437000	35.794204000
O	42.368184000	39.393849000	41.218039000
O	50.356442000	41.178046000	35.356425000
O	48.165272000	40.832181000	36.873861000
O	48.070079000	42.326271000	34.670557000
O	48.341361000	39.648511000	34.518118000
O	46.870619000	39.860153000	39.002246000
O	45.489760000	40.718726000	36.918690000
O	41.787472000	40.117063000	38.787514000
O	42.839381000	41.089084000	36.517241000
O	44.630482000	42.163631000	34.891778000
O	44.136415000	49.310139000	34.982031000
O	43.716917000	46.733811000	32.357140000
O	44.065205000	44.400713000	33.578174000
O	46.179957000	45.956082000	32.999800000
O	48.411582000	44.456624000	33.083892000
O	48.534681000	46.876262000	32.052589000
O	50.382422000	47.643409000	35.529402000
O	48.199085000	47.967757000	36.981770000
O	48.094185000	46.557682000	34.707512000
O	48.423193000	49.219119000	34.617847000
O	46.805690000	48.755133000	39.116904000
O	46.901320000	50.299414000	36.915919000
O	45.530064000	47.999131000	36.892642000
O	41.567202000	48.858054000	38.831707000
O	42.859477000	47.766310000	36.727840000
O	44.434530000	46.622760000	34.939383000
O	43.746968000	42.075747000	32.354145000
O	46.213373000	42.946986000	32.888078000
O	48.602038000	42.030695000	32.042276000
O	54.204118000	44.426864000	38.677231000
O	56.338147000	42.949704000	39.323104000
O	55.472547000	47.974447000	35.213140000
O	51.848421000	48.689120000	33.541957000
O	52.925472000	47.685798000	35.811910000
O	51.611485000	50.056063000	35.799798000
O	54.804248000	46.685969000	37.396039000
O	54.397135000	49.370801000	37.253668000
O	56.348193000	45.872862000	39.399034000
O	55.499440000	40.876893000	35.234217000
O	51.843555000	40.022833000	33.475928000
O	52.923975000	41.101480000	35.688100000
O	54.682094000	42.167136000	37.367805000
O	46.461626000	41.953788000	40.522994000
O	44.330257000	40.405574000	39.681535000

O	46.075558000	39.517319000	41.509121000
O	42.441917000	42.007707000	40.594140000
O	42.231945000	49.323358000	41.312737000
O	43.858158000	48.375724000	43.600713000
O	48.884307000	48.683439000	42.990207000
O	42.769892000	46.015616000	42.959077000
O	41.583042000	44.433561000	41.116701000
O	43.778770000	46.388658000	45.398347000
O	45.381967000	46.114265000	43.287843000
O	48.776284000	46.796903000	44.919143000
O	47.853504000	46.342920000	42.378849000
O	46.298180000	44.480953000	41.284590000
O	45.700432000	46.944245000	40.765665000
O	44.154164000	48.728496000	39.560566000
O	45.894754000	49.469529000	41.501799000
O	42.369935000	46.840115000	40.413255000
O	43.335787000	40.461045000	43.666459000
O	48.682591000	40.254916000	43.134897000
O	42.660678000	43.006465000	43.124482000
O	43.800470000	42.411921000	45.414893000
O	45.198666000	42.284062000	43.078583000
O	48.969362000	41.984191000	45.199808000
O	48.000257000	42.846936000	42.853496000
O	53.576121000	40.324089000	41.899321000
O	50.563029000	42.117879000	43.055615000
O	52.974967000	42.898039000	42.303419000
O	51.632602000	44.390714000	44.057200000
O	53.920321000	42.057149000	39.915983000
O	55.522479000	42.145211000	42.036536000
O	52.392314000	46.823556000	44.752758000
O	53.539163000	48.486513000	41.913260000
O	50.449527000	46.514007000	42.872210000
O	52.935560000	45.906099000	42.271690000
O	53.890563000	46.781176000	39.902047000
O	55.482526000	46.668383000	42.047914000
O	52.574949000	42.014087000	44.824220000
Ga	47.195931000	44.448648000	39.329350000
C	51.178958000	41.628441000	38.528604000
H	52.103430000	41.110345000	38.257777000
C	50.861651000	42.824596000	37.993969000
H	46.061091000	43.977553000	38.338653000
H	51.515177000	43.305685000	37.252739000
C	50.313645000	40.996229000	39.510131000
C	49.253361000	41.645856000	40.035703000
H	50.560326000	39.989297000	39.874351000
H	47.922533000	45.842437000	39.363185000
H	48.711289000	41.140864000	40.840155000
C	49.551283000	43.508610000	38.305960000
C	48.875947000	43.066930000	39.649652000
H	48.873081000	43.330295000	37.437510000
H	49.717352000	44.603714000	38.314195000
H	49.266651000	43.694491000	40.488707000
H	47.360732000	42.414215000	39.950031000

I19-GaH_2

Electronic energy (r^2SCAN-D4/def2-SVP): -48411.82199736 E_h
Electronic energy (ωB97X-D4rev/def2-mTZVPP): -48455.900888330400 E_h
G_{corr} (673.15 K, 1 atm): 2.02781612 E_h
Number of atoms: 393

Cartesian coordinates (Å)

Si	48.550589000	36.024487000	35.312580000
Si	46.528235000	36.953399000	37.350684000
Si	44.100473000	36.088789000	35.635065000
Si	44.618415000	38.494799000	33.730907000
Si	47.763820000	38.526231000	33.546992000
Si	48.748174000	38.751175000	30.518314000
Si	51.807529000	38.121615000	30.330042000
Si	54.672341000	38.464519000	38.536977000
Si	57.776554000	38.488891000	38.803260000
Si	56.556390000	37.023315000	34.997676000
Si	52.599032000	36.082384000	32.661713000
Si	51.479624000	37.152232000	35.479828000
Si	54.130048000	36.092041000	36.717873000
Si	45.619438000	36.054771000	40.031026000
Si	41.750813000	38.052905000	41.947109000
Si	43.727718000	38.893462000	44.076471000
Si	47.725059000	38.464210000	46.617040000
Si	48.767808000	38.635887000	43.590820000
Si	46.082989000	38.073044000	42.357530000
Si	51.735046000	38.005262000	43.473234000
Si	53.672178000	38.801347000	41.348355000
Si	56.183845000	38.109580000	43.088221000
Si	44.637973000	50.352974000	33.700718000
Si	47.770467000	50.304919000	33.521468000
Si	48.755591000	50.072899000	30.511730000
Si	43.872013000	46.933467000	30.714396000
Si	48.892641000	46.959821000	30.406657000
Si	46.327598000	45.963436000	29.021928000
Si	48.563503000	52.798526000	35.324640000
Si	46.529019000	51.866484000	37.348451000
Si	44.099732000	52.734275000	35.627950000
Si	43.865907000	41.889771000	30.718818000
Si	48.876559000	41.842693000	30.426930000
Si	46.327358000	42.851076000	29.030054000
Si	51.883200000	42.864204000	30.524306000
Si	57.965244000	42.906369000	39.192273000
Si	58.732254000	47.854282000	36.910041000
Si	56.853933000	48.675995000	34.762682000
Si	55.860779000	48.297338000	31.970879000
Si	52.745737000	48.323030000	32.245745000
Si	56.557216000	51.798606000	34.996851000
Si	52.597746000	52.738623000	32.662606000
Si	51.489790000	51.669085000	35.489298000
Si	54.140897000	52.729168000	36.719192000
Si	51.798647000	50.703088000	30.327093000
Si	54.680604000	50.360683000	38.530375000
Si	57.780694000	50.329508000	38.800792000
Si	51.880806000	45.953639000	30.515032000
Si	57.974861000	45.921564000	39.188533000
Si	58.734967000	40.965065000	36.915862000

Si	56.872011000	40.148851000	34.764042000
Si	55.869636000	40.523726000	31.974192000
Si	52.764254000	40.495332000	32.236234000
Si	48.691798000	40.952670000	48.525133000
Si	41.747192000	50.760160000	41.923577000
Si	43.713623000	49.956780000	44.074502000
Si	47.726896000	50.350265000	46.636226000
Si	48.775615000	50.193512000	43.631462000
Si	46.110798000	50.831038000	42.409557000
Si	44.946538000	46.009014000	46.544229000
Si	47.986669000	46.006989000	46.205563000
Si	48.686672000	47.852896000	48.533699000
Si	45.619963000	52.773816000	40.027495000
Si	44.929014000	42.791502000	46.536354000
Si	47.990701000	42.773968000	46.155357000
Si	58.956618000	41.842102000	41.957732000
Si	56.334873000	42.835336000	43.278964000
Si	55.798733000	48.297338000	45.170746000
Si	52.662078000	48.339303000	45.344674000
Si	51.677325000	48.629610000	48.354303000
Si	51.753952000	50.814291000	43.465150000
Si	53.681524000	50.031958000	41.338220000
Si	56.187094000	50.710251000	43.089174000
Si	58.959941000	46.981205000	41.941107000
Si	56.324016000	45.966339000	43.265463000
Si	55.784240000	40.508501000	45.179150000
Si	52.653933000	40.468524000	45.361225000
Si	51.671591000	40.189958000	48.365762000
O	49.973695000	36.643323000	35.859391000
O	47.515559000	35.885806000	36.589283000
O	46.659165000	36.825064000	38.976185000
O	44.969223000	36.635611000	36.937486000
O	44.496899000	36.939064000	34.261551000
O	46.170526000	38.852370000	33.276575000
O	47.916594000	37.004974000	34.141769000
O	48.595331000	38.648698000	32.148196000
O	50.188995000	38.112100000	30.090130000
O	52.234533000	36.836618000	31.246004000
O	52.289865000	39.557612000	30.981256000
O	54.582538000	36.889224000	38.084793000
O	56.172032000	38.730121000	39.128787000
O	56.912271000	38.603534000	35.311380000
O	54.992865000	36.665331000	35.426701000
O	51.805572000	36.856035000	33.897269000
O	52.529167000	36.314525000	36.423392000
O	42.474873000	38.029231000	43.431339000
O	45.168879000	38.185875000	43.704090000
O	48.549699000	38.665157000	45.206128000
O	47.619171000	37.777323000	42.808950000
O	45.568794000	36.792381000	41.493579000
O	50.135699000	37.823777000	43.241690000
O	52.423937000	37.961616000	41.976105000
O	52.085879000	39.429376000	44.228968000
O	53.576519000	38.767348000	39.713182000
O	55.083777000	38.131168000	41.853492000
O	44.502683000	51.902346000	34.247237000
O	46.179255000	50.023084000	33.200771000
O	47.928198000	51.802645000	34.170046000

O	48.641105000	50.208412000	32.146733000
O	48.598975000	48.516708000	30.007847000
O	45.413241000	46.605310000	30.224667000
O	47.904296000	46.003322000	29.510711000
O	45.884781000	44.407687000	28.747643000
O	49.987497000	52.191781000	35.874515000
O	47.529561000	52.931962000	36.608260000
O	46.642158000	51.969240000	38.982465000
O	44.972875000	52.185278000	36.931094000
O	48.606229000	40.297184000	29.972971000
O	45.410878000	42.221031000	30.237568000
O	47.900701000	42.816695000	29.531570000
O	50.430755000	42.221841000	30.102831000
O	51.660891000	44.411354000	31.023716000
O	58.593656000	44.413353000	39.371393000
O	58.089943000	47.819169000	35.395887000
O	58.304379000	46.469454000	37.684280000
O	58.228425000	49.191396000	37.704084000
O	56.981791000	48.630024000	33.139127000
O	56.907490000	50.219040000	35.317172000
O	54.332145000	48.579488000	32.555673000
O	52.525466000	46.760809000	31.800297000
O	54.992392000	52.161343000	35.419872000
O	51.812596000	51.968043000	33.907405000
O	52.538522000	52.496552000	36.440232000
O	50.185320000	50.711063000	30.048485000
O	52.213670000	51.988516000	31.249647000
O	52.252906000	49.272841000	31.006894000
O	54.611110000	51.938910000	38.083598000
O	56.175531000	50.076484000	39.123024000
O	50.442584000	46.599352000	30.047051000
O	58.115796000	40.998354000	35.392579000
O	58.308083000	42.348468000	37.694405000
O	58.217872000	39.631863000	37.707017000
O	56.995224000	40.198888000	33.139597000
O	54.346341000	40.240965000	32.571467000
O	52.552878000	42.063566000	31.800743000
O	50.287500000	40.899780000	48.883848000
O	48.427863000	42.349587000	47.680194000
O	48.221992000	39.620261000	47.686040000
O	42.458209000	50.791307000	43.416159000
O	45.152005000	50.671116000	43.714120000
O	48.597239000	50.150903000	45.247141000
O	47.629986000	51.111714000	42.909455000
O	45.590024000	52.080649000	41.516309000
O	44.956766000	44.407263000	46.851882000
O	46.423350000	46.477677000	45.954993000
O	48.174221000	44.402005000	45.978584000
O	50.287945000	47.912277000	48.849914000
O	48.384073000	46.437439000	47.734974000
O	48.194450000	49.179239000	47.694393000
O	46.414447000	42.329631000	45.976338000
O	58.634101000	41.947598000	40.331147000
O	57.934738000	42.735119000	42.916877000
O	55.902478000	44.401704000	43.454026000
O	55.981050000	46.731563000	44.680691000
O	54.255149000	48.610259000	45.666396000
O	56.175940000	49.269294000	43.897348000

O	51.800710000	48.566059000	46.707156000
O	50.148963000	50.949590000	43.215315000
O	52.441643000	50.884290000	41.972348000
O	52.097054000	49.394126000	44.223443000
O	53.577920000	50.073799000	39.705495000
O	55.094586000	50.691648000	41.847343000
O	58.663597000	46.890624000	40.308642000
O	57.912019000	46.079464000	42.861356000
O	55.947691000	42.077824000	44.685002000
O	54.248179000	40.175696000	45.681152000
O	56.176991000	39.548395000	43.901451000
O	51.784928000	40.246851000	46.718673000
H	48.702884000	34.676560000	34.772583000
H	44.419890000	34.676310000	35.346851000
H	42.683642000	36.258960000	36.022184000
H	43.726533000	38.680192000	32.577953000
H	47.730598000	38.010983000	29.830584000
H	52.421139000	38.002370000	29.030390000
H	58.465368000	38.743369000	40.066969000
H	58.088397000	37.108513000	38.286198000
H	56.651071000	36.758841000	33.558684000
H	57.541596000	36.246998000	35.782472000
H	52.141245000	34.673915000	32.562057000
H	54.071047000	36.146094000	32.821448000
H	54.418097000	34.675478000	36.917905000
H	44.265401000	36.148723000	39.494252000
H	46.085068000	34.677309000	40.196460000
H	42.177326000	36.886185000	41.136510000
H	40.290213000	38.106984000	42.090400000
H	43.614709000	38.689580000	45.487262000
H	46.266785000	38.594601000	46.380883000
H	48.065403000	37.113071000	47.131287000
H	52.195272000	36.881484000	44.271745000
H	55.858318000	36.988848000	44.011830000
H	57.529578000	37.965444000	42.519254000
H	41.636488000	38.780152000	36.634500000
H	40.338247000	40.845231000	36.817797000
H	43.726291000	50.140509000	32.578076000
H	47.730492000	50.809909000	29.830557000
H	42.910969000	46.071575000	29.969690000
H	43.636007000	48.346485000	30.474315000
H	46.055510000	46.690795000	27.779382000
H	40.338196000	47.975589000	36.817801000
H	41.636402000	50.040687000	36.634468000
H	48.702707000	54.144368000	34.772536000
H	44.419575000	54.144594000	35.346959000
H	42.683569000	52.561599000	36.022287000
H	43.636072000	40.474342000	30.474347000
H	42.910999000	42.749230000	29.969684000
H	46.055547000	42.130060000	27.779390000
H	52.768238000	42.805891000	29.339152000
H	60.152184000	47.950754000	36.769102000
H	56.115701000	49.276596000	30.928769000
H	56.049414000	46.904454000	31.507080000
H	57.541459000	52.574081000	35.782439000
H	56.650944000	52.062214000	33.558650000
H	52.141079000	54.147059000	32.562017000
H	54.070908000	52.674921000	32.821415000

H	54.418135000	54.145524000	36.917807000
H	52.421032000	50.818594000	29.030363000
H	58.465272000	50.077742000	40.066946000
H	58.088277000	51.712587000	38.286172000
H	52.768212000	46.015083000	29.339147000
H	60.152243000	40.870374000	36.769117000
H	56.049460000	41.916582000	31.507092000
H	56.115781000	39.544440000	30.928787000
H	48.027974000	40.958437000	49.819992000
H	40.290118000	50.713674000	42.090599000
H	42.176848000	51.934813000	41.136538000
H	43.615024000	50.131719000	45.487226000
H	48.065239000	51.707917000	47.131237000
H	46.266696000	50.226318000	46.380835000
H	40.476353000	46.423808000	42.229533000
H	44.578038000	46.685728000	47.785522000
H	48.027889000	47.862537000	49.819970000
H	46.084737000	54.143660000	40.196338000
H	44.265252000	52.672169000	39.494190000
H	40.476384000	42.397037000	42.229545000
H	44.578063000	42.135170000	47.785520000
H	58.818814000	40.408575000	42.221796000
H	60.309682000	42.345562000	42.255157000
H	56.725247000	48.548912000	46.274184000
H	51.671695000	50.045763000	48.769747000
H	52.721690000	47.848751000	49.007596000
H	52.195150000	51.939535000	44.271719000
H	55.858186000	51.832236000	44.011801000
H	57.529470000	50.855671000	42.519230000
H	60.309648000	46.475594000	42.255149000
H	58.818743000	48.412553000	42.221780000
H	56.725198000	40.272291000	46.274316000
H	52.722261000	40.971973000	49.006973000
H	51.671842000	38.775274000	48.769832000
Si	48.759992000	40.969795000	35.426541000
Si	46.859934000	39.982447000	37.453689000
Si	41.579147000	40.157615000	37.178445000
Si	44.271996000	40.860290000	35.861921000
Si	44.709338000	45.936186000	33.444536000
Si	47.861355000	45.888101000	33.150720000
Si	48.721191000	47.815015000	35.379047000
Si	46.811132000	48.831411000	37.430288000
Si	41.607570000	48.640291000	37.118546000
Si	44.281377000	47.967068000	35.809330000
Si	44.655167000	42.886675000	33.473590000
Si	47.826642000	42.853249000	33.189318000
Si	54.805200000	42.906780000	38.821894000
Si	51.668109000	48.543111000	35.186941000
Si	54.377677000	47.968077000	36.444722000
Si	54.823597000	45.938150000	38.818770000
Si	51.684132000	40.269933000	35.159589000
Si	54.387198000	40.860866000	36.444418000
Si	45.735164000	40.401652000	40.195393000
Si	42.664689000	40.433838000	40.039830000
Si	41.745397000	45.905502000	41.730239000
Si	43.885965000	46.760033000	43.795092000
Si	49.016166000	47.090034000	43.525320000
Si	46.474212000	45.975384000	42.038998000

Si	45.878067000	48.474054000	40.267086000
Si	42.721410000	48.334335000	40.052008000
Si	41.766015000	42.861405000	41.736996000
Si	43.919205000	42.100892000	43.812368000
Si	49.033757000	41.755447000	43.408775000
Al	46.428219000	42.862559000	41.932585000
Si	51.915935000	42.864123000	43.569640000
Si	53.986686000	41.889857000	41.541495000
Si	51.874372000	45.935422000	43.537898000
Si	53.945119000	46.941412000	41.520517000
O	46.929128000	38.459909000	36.824879000
O	44.158722000	39.489288000	34.962606000
O	54.381958000	39.425621000	37.241285000
O	51.585991000	38.761575000	35.799934000
O	42.243274000	39.470314000	41.277149000
O	50.372429000	41.177934000	35.503093000
O	48.185116000	40.813949000	36.952003000
O	48.091640000	42.276570000	34.697635000
O	48.415991000	39.615686000	34.574659000
O	46.831633000	39.890078000	39.073407000
O	45.523293000	40.756165000	36.904947000
O	41.635745000	40.069819000	38.821740000
O	42.852907000	41.073290000	36.638223000
O	44.538015000	42.168649000	34.922690000
O	44.223287000	49.359926000	34.946565000
O	43.767313000	46.731457000	32.360513000
O	44.115732000	44.425899000	33.626299000
O	46.248371000	45.884141000	32.880154000
O	48.449360000	44.365224000	33.058115000
O	48.630144000	46.802921000	32.029557000
O	50.333450000	47.668128000	35.536923000
O	48.063509000	47.934127000	36.881034000
O	48.125495000	46.477714000	34.656102000
O	48.353836000	49.165215000	34.536862000
O	46.892327000	48.900966000	39.060643000
O	46.930149000	50.356137000	36.828781000
O	45.398129000	48.130164000	36.997727000
O	41.826345000	48.716010000	38.748691000
O	42.801256000	47.707203000	36.444941000
O	44.702850000	46.693139000	34.887920000
O	43.730539000	42.117261000	32.356827000
O	46.209798000	42.889914000	32.949739000
O	48.566045000	41.938588000	32.046753000
O	54.239946000	44.427044000	38.598507000
O	56.342653000	42.967362000	39.377010000
O	55.421378000	48.005650000	35.191533000
O	51.848418000	48.681281000	33.563600000
O	52.881674000	47.685809000	35.860167000
O	51.575574000	50.059902000	35.809118000
O	54.826194000	46.734806000	37.402322000
O	54.370383000	49.409407000	37.233170000
O	56.352427000	45.881523000	39.397667000
O	55.457610000	40.839503000	35.214147000
O	51.851955000	40.116282000	33.535832000
O	52.908492000	41.123706000	35.815659000
O	54.783922000	42.107237000	37.409163000
O	45.980389000	41.959520000	40.516063000
O	44.215368000	40.133379000	39.584390000

O	45.949628000	39.477962000	41.532454000
O	42.467591000	41.992375000	40.521337000
O	42.266639000	49.364069000	41.225125000
O	43.733209000	48.381239000	43.573155000
O	48.718370000	48.641367000	43.079929000
O	42.687329000	45.939889000	43.076866000
O	41.601906000	44.390292000	41.138003000
O	43.845036000	46.423281000	45.391418000
O	45.289272000	46.249166000	43.100711000
O	48.927869000	46.863052000	45.152228000
O	47.908356000	46.135446000	42.788101000
O	46.478085000	44.544330000	41.212397000
O	46.365753000	46.955790000	40.698558000
O	44.330006000	48.509769000	39.740218000
O	46.056534000	49.429345000	41.562498000
O	42.442575000	46.795168000	40.525271000
O	43.739674000	40.470497000	43.597252000
O	48.798999000	40.172373000	43.035850000
O	42.663573000	42.874626000	43.100543000
O	43.786236000	42.399435000	45.431769000
O	45.288132000	42.713636000	43.224550000
O	48.909149000	41.982284000	45.053539000
O	48.020532000	42.683235000	42.586270000
O	53.560001000	40.351087000	41.886563000
O	50.586435000	42.122757000	42.990165000
O	53.023118000	42.926477000	42.355525000
O	51.596717000	44.405827000	44.049175000
O	53.875163000	42.120143000	39.920311000
O	55.538449000	42.137156000	42.020644000
O	52.494395000	46.801530000	44.791170000
O	53.545871000	48.486285000	41.878522000
O	50.497455000	46.653369000	42.996781000
O	52.916429000	45.919557000	42.271423000
O	53.878158000	46.744151000	39.892986000
O	55.473306000	46.659106000	42.042008000
O	52.523759000	42.025227000	44.846294000
Ga	47.290712000	45.082574000	39.379777000
C	50.612446000	43.402071000	38.299949000
H	50.904230000	42.516342000	37.721238000
C	50.394090000	44.589366000	37.680182000
H	47.983351000	43.047763000	38.493406000
H	50.528286000	44.695610000	36.594389000
C	50.476281000	43.295529000	39.749903000
C	49.831207000	44.262226000	40.455227000
H	50.840945000	42.395490000	40.259843000
H	46.104515000	45.120616000	38.352310000
H	49.644206000	44.138680000	41.527209000
C	50.105776000	45.809709000	38.521967000
C	49.257103000	45.453552000	39.752516000
H	49.658212000	46.625568000	37.938034000
H	51.083334000	46.206070000	38.892589000
H	49.216739000	46.324702000	40.434420000
H	47.428432000	42.787308000	38.959990000

I20-GaH_2

Electronic energy (r^2SCAN-D4/def2-SVP): -48410.68505443 E_h
Electronic energy (ωB97X-D4rev/def2-mTZVPP): -48454.746127064600 E_h
G_{corr} (673.15 K, 1 atm): 2.01482255 E_h
Number of atoms: 391

Cartesian coordinates (Å)

Si	48.555504000	36.027693000	35.304318000
Si	46.524842000	36.969782000	37.342628000
Si	44.100646000	36.091677000	35.632760000
Si	44.629589000	38.500624000	33.724592000
Si	47.776138000	38.520502000	33.537512000
Si	48.742684000	38.770446000	30.510728000
Si	51.807358000	38.132115000	30.326363000
Si	54.676963000	38.468048000	38.536146000
Si	57.776536000	38.489645000	38.803515000
Si	56.555158000	37.020826000	34.996672000
Si	52.595854000	36.085569000	32.663516000
Si	51.480988000	37.160389000	35.487464000
Si	54.133109000	36.093970000	36.716867000
Si	45.623023000	36.076542000	40.031100000
Si	41.742977000	38.014104000	41.943394000
Si	43.556497000	38.855969000	44.075299000
Si	47.727259000	38.460904000	46.621171000
Si	48.748619000	38.672558000	43.604945000
Si	46.055095000	38.203527000	42.320566000
Si	51.711092000	37.992602000	43.459551000
Si	53.661107000	38.777284000	41.346648000
Si	56.185102000	38.105698000	43.084683000
Si	44.617403000	50.308366000	33.739184000
Si	47.765672000	50.293301000	33.557525000
Si	48.739893000	50.054840000	30.522738000
Si	43.865231000	46.935793000	30.714778000
Si	48.870813000	46.956162000	30.413258000
Si	46.318372000	45.963315000	29.025134000
Si	48.553590000	52.788334000	35.305007000
Si	46.526172000	51.836456000	37.362216000
Si	44.103334000	52.728128000	35.638470000
Si	43.867993000	41.885422000	30.710904000
Si	48.871842000	41.873455000	30.406639000
Si	46.320013000	42.860167000	29.021771000
Si	51.879675000	42.861954000	30.520250000
Si	57.963933000	42.900934000	39.184559000
Si	58.734207000	47.858900000	36.909533000
Si	56.874444000	48.675565000	34.760330000
Si	55.868992000	48.296995000	31.973779000
Si	52.764030000	48.322080000	32.237835000
Si	56.555240000	51.799822000	34.996387000
Si	52.599328000	52.737012000	32.656509000
Si	51.486595000	51.656184000	35.471955000
Si	54.129252000	52.729054000	36.710883000
Si	51.806578000	50.695371000	30.330275000
Si	54.674811000	50.349822000	38.529876000
Si	57.777193000	50.331656000	38.802289000
Si	51.879857000	45.953614000	30.520491000
Si	57.963144000	45.922371000	39.182165000
Si	58.735401000	40.962040000	36.911634000

Si	56.879182000	40.145092000	34.760868000
Si	55.876757000	40.524973000	31.973251000
Si	52.773872000	40.493239000	32.227776000
Si	48.662028000	40.949486000	48.520622000
Si	41.741618000	50.787003000	41.945879000
Si	43.701977000	50.019151000	44.071529000
Si	47.726762000	50.361673000	46.610389000
Si	48.767841000	50.156452000	43.577522000
Si	46.081544000	50.774453000	42.366709000
Si	44.769311000	45.960919000	46.549675000
Si	47.937437000	45.959856000	46.146895000
Si	48.679265000	47.889903000	48.515861000
Si	45.618309000	52.770277000	40.027333000
Si	44.774123000	42.846289000	46.542552000
Si	47.936981000	42.841063000	46.194812000
Si	58.955692000	41.840992000	41.946822000
Si	56.316013000	42.836554000	43.274040000
Si	55.793165000	48.297951000	45.170926000
Si	52.641315000	48.351790000	45.335451000
Si	51.672442000	48.628307000	48.355382000
Si	51.735293000	50.828130000	43.460302000
Si	53.667570000	50.033070000	41.341992000
Si	56.183461000	50.710479000	43.088375000
Si	58.957021000	46.979800000	41.937462000
Si	56.317724000	45.967788000	43.267702000
Si	55.799505000	40.499830000	45.165648000
Si	52.675044000	40.440209000	45.336620000
Si	51.670662000	40.195297000	48.347333000
O	49.974574000	36.643867000	35.860892000
O	47.511249000	35.908527000	36.576454000
O	46.663340000	36.832807000	38.966947000
O	44.965482000	36.647366000	36.935692000
O	44.497704000	36.947906000	34.264087000
O	46.181923000	38.846784000	33.265309000
O	47.923056000	36.994536000	34.122381000
O	48.607565000	38.654231000	32.141358000
O	50.190997000	38.158063000	30.070581000
O	52.207867000	36.847163000	31.256516000
O	52.320115000	39.563795000	30.960087000
O	54.604122000	36.893937000	38.075284000
O	56.173412000	38.737062000	39.135769000
O	56.908513000	38.600732000	35.311743000
O	54.990014000	36.658938000	35.418250000
O	51.811670000	36.851604000	33.909113000
O	52.530711000	36.329913000	36.438454000
O	42.399268000	37.864143000	43.454283000
O	45.058456000	38.398396000	43.591790000
O	48.576529000	38.681082000	45.226424000
O	47.545783000	37.843859000	42.868799000
O	45.561048000	36.922712000	41.440165000
O	50.103232000	37.849989000	43.216938000
O	52.408407000	37.934005000	41.966615000
O	52.066469000	39.415691000	44.212841000
O	53.573479000	38.745070000	39.711891000
O	55.072390000	38.114009000	41.861166000
O	44.496903000	51.864076000	34.274371000
O	46.172845000	49.950055000	33.299212000
O	47.904269000	51.828383000	34.124868000

O	48.585652000	50.146681000	32.155479000
O	48.585806000	48.507790000	29.984999000
O	45.411071000	46.597088000	30.239936000
O	47.897199000	45.989881000	29.508939000
O	45.860994000	44.411936000	28.743196000
O	49.977105000	52.166538000	35.840958000
O	47.527997000	52.884170000	36.596625000
O	46.652164000	51.971433000	38.991822000
O	44.974019000	52.179798000	36.942607000
O	48.569429000	40.321873000	29.991505000
O	45.413729000	42.221659000	30.234189000
O	47.900593000	42.839487000	29.499340000
O	50.432548000	42.218099000	30.074940000
O	51.656163000	44.408022000	31.020991000
O	58.581551000	44.412112000	39.357755000
O	58.128719000	47.836591000	35.381050000
O	58.288254000	46.475483000	37.678030000
O	58.218723000	49.191397000	37.704121000
O	56.995111000	48.631114000	33.135955000
O	56.902925000	50.219336000	35.314002000
O	54.346248000	48.571442000	32.576352000
O	52.543413000	46.756870000	31.798421000
O	54.990424000	52.164390000	35.415957000
O	51.811289000	51.953892000	33.889946000
O	52.527749000	52.503864000	36.417223000
O	50.185729000	50.686746000	30.100127000
O	52.226307000	51.988948000	31.239491000
O	52.302416000	49.266000000	30.982617000
O	54.587045000	51.923357000	38.070267000
O	56.172453000	50.089948000	39.129875000
O	50.429407000	46.597947000	30.087962000
O	58.130457000	40.987309000	35.383071000
O	58.296144000	42.345657000	37.683063000
O	58.217121000	39.629787000	37.704202000
O	57.001980000	40.193038000	33.136427000
O	54.354744000	40.253924000	32.574164000
O	52.535251000	42.060801000	31.804404000
O	50.278250000	40.950505000	48.786466000
O	48.241619000	42.329129000	47.720281000
O	48.204065000	39.595505000	47.715001000
O	42.460144000	50.888919000	43.432362000
O	45.166016000	50.678263000	43.713561000
O	48.530510000	50.098874000	45.190890000
O	47.621063000	51.034644000	42.810508000
O	45.565284000	52.044583000	41.498289000
O	44.383940000	44.406974000	46.862773000
O	46.311273000	46.076180000	45.995787000
O	48.426250000	44.401000000	46.020562000
O	50.282629000	47.899981000	48.841617000
O	48.345527000	46.548437000	47.617578000
O	48.228167000	49.253644000	47.720004000
O	46.312426000	42.746810000	45.947361000
O	58.651288000	41.944477000	40.315951000
O	57.908390000	42.730050000	42.881350000
O	55.896929000	44.403333000	43.465969000
O	55.985753000	46.731901000	44.685442000
O	54.241205000	48.597359000	45.650062000
O	56.175788000	49.270432000	43.899478000

O	51.794346000	48.578383000	46.707715000
O	50.128505000	50.977179000	43.236875000
O	52.420684000	50.885577000	41.964742000
O	52.079615000	49.409985000	44.219996000
O	53.575260000	50.066605000	39.708616000
O	55.079557000	50.693830000	41.856737000
O	58.659226000	46.884963000	40.304705000
O	57.903887000	46.079047000	42.853442000
O	55.955100000	42.072863000	44.684742000
O	54.265277000	40.129427000	45.646719000
O	56.206507000	39.552808000	43.883106000
O	51.821834000	40.226816000	46.703904000
H	48.702872000	34.676567000	34.772567000
H	44.419876000	34.676321000	35.346830000
H	42.683629000	36.258973000	36.022160000
H	43.726523000	38.680214000	32.577942000
H	47.730595000	38.010995000	29.830570000
H	52.421136000	38.002379000	29.030381000
H	58.465354000	38.743363000	40.066967000
H	58.088383000	37.108509000	38.286195000
H	56.651061000	36.758841000	33.558678000
H	57.541583000	36.246995000	35.782468000
H	52.141234000	34.673921000	32.562045000
H	54.071037000	36.146097000	32.821439000
H	54.418083000	34.675478000	36.917895000
H	44.265383000	36.148733000	39.494231000
H	46.085046000	34.677315000	40.196441000
H	42.177305000	36.886191000	41.136492000
H	40.290195000	38.107002000	42.090377000
H	43.614692000	38.689573000	45.487247000
H	46.266761000	38.594605000	46.380865000
H	48.065374000	37.113072000	47.131273000
H	52.195252000	36.881484000	44.271735000
H	55.858296000	36.988842000	44.011825000
H	57.529559000	37.965438000	42.519250000
H	41.636485000	38.780153000	36.634495000
H	40.338248000	40.845234000	36.817795000
H	43.726294000	50.140516000	32.578071000
H	47.730501000	50.809920000	29.830551000
H	42.910973000	46.071592000	29.969679000
H	43.636012000	48.346498000	30.474316000
H	46.055517000	46.690811000	27.779371000
H	40.338196000	47.975591000	36.817799000
H	41.636403000	50.040687000	36.634467000
H	48.702712000	54.144375000	34.772532000
H	44.419583000	54.144604000	35.346948000
H	42.683573000	52.561614000	36.022276000
H	43.636069000	40.474359000	30.474328000
H	42.910997000	42.749250000	29.969681000
H	46.055550000	42.130074000	27.779377000
H	52.768240000	42.805897000	29.339147000
H	60.152182000	47.950750000	36.769108000
H	56.115706000	49.276599000	30.928771000
H	56.049417000	46.904456000	31.507081000
H	57.541463000	52.574079000	35.782445000
H	56.650950000	52.062214000	33.558655000
H	52.141087000	54.147065000	32.562018000
H	54.070915000	52.674924000	32.821418000

H	54.418139000	54.145525000	36.917810000
H	52.421043000	50.818601000	29.030362000
H	58.465267000	50.077736000	40.066951000
H	58.088277000	51.712582000	38.286178000
H	52.768216000	46.015091000	29.339142000
H	60.152234000	40.870368000	36.769119000
H	56.049458000	41.916585000	31.507090000
H	56.115777000	39.544444000	30.928783000
H	48.027949000	40.958435000	49.819979000
H	40.290113000	50.713681000	42.090582000
H	42.176848000	51.934814000	41.136521000
H	43.615014000	50.131728000	45.487215000
H	48.065227000	51.707917000	47.131231000
H	46.266684000	50.226320000	46.380825000
H	40.476352000	46.423809000	42.229530000
H	44.578029000	46.685731000	47.785516000
H	48.027870000	47.862537000	49.819961000
H	46.084737000	54.143667000	40.196331000
H	44.265251000	52.672178000	39.494179000
H	40.476382000	42.397038000	42.229542000
H	44.578054000	42.135173000	47.785514000
H	58.818796000	40.408568000	42.221796000
H	60.309668000	42.345552000	42.255160000
H	56.725235000	48.548904000	46.274186000
H	51.671680000	50.045759000	48.769744000
H	52.721672000	47.848745000	49.007593000
H	52.195143000	51.939534000	44.271718000
H	55.858179000	51.832230000	44.011805000
H	57.529464000	50.855665000	42.519235000
H	60.309638000	46.475586000	42.255156000
H	58.818735000	48.412545000	42.221785000
H	56.725177000	40.272282000	46.274313000
H	52.722236000	40.971967000	49.006966000
H	51.671816000	38.775269000	48.769821000
Si	48.780937000	40.974286000	35.386949000
Si	46.888253000	40.026946000	37.416461000
Si	41.589357000	40.162714000	37.177958000
Si	44.284084000	40.883714000	35.845785000
Si	44.621505000	45.938534000	33.459132000
Si	47.793698000	45.951093000	33.188747000
Si	48.763765000	47.860388000	35.430774000
Si	46.853778000	48.766821000	37.475689000
Si	41.573174000	48.671572000	37.175528000
Si	44.262808000	47.934575000	35.868208000
Si	44.631639000	42.893727000	33.448338000
Si	47.798554000	42.897409000	33.178607000
Si	54.804436000	42.889541000	38.828301000
Si	51.686739000	48.538526000	35.159187000
Si	54.392582000	47.944199000	36.439568000
Si	54.805722000	45.928552000	38.831044000
Si	51.692438000	40.269449000	35.138071000
Si	54.391329000	40.868258000	36.438387000
Si	45.791050000	40.512507000	40.154056000
Si	42.688660000	40.436730000	40.058819000
Si	41.765383000	45.947768000	41.702714000
Si	43.893826000	46.865938000	43.801396000
Si	48.917991000	47.032798000	43.334045000
Si	46.290995000	45.987362000	41.998003000

Si	45.697856000	48.426093000	40.231297000
Si	42.623606000	48.406671000	40.034925000
Si	41.769790000	42.904635000	41.768553000
Si	43.741560000	41.927127000	43.853459000
Si	48.975942000	41.778199000	43.500472000
Al	46.338493000	42.777163000	42.164659000
Si	51.905091000	42.859562000	43.580487000
Si	53.941794000	41.868278000	41.529338000
Si	51.842251000	45.919723000	43.537286000
Si	53.926526000	46.943243000	41.528185000
O	46.911227000	38.487422000	36.838268000
O	44.190178000	39.506106000	34.957287000
O	54.384183000	39.439798000	37.249154000
O	51.573692000	38.771526000	35.802921000
O	42.364526000	39.367693000	41.238643000
O	50.386652000	41.201339000	35.425556000
O	48.221531000	40.829336000	36.919261000
O	48.040150000	42.231240000	34.649458000
O	48.435045000	39.589460000	34.579479000
O	46.877083000	40.008061000	39.043043000
O	45.543874000	40.808965000	36.890071000
O	41.675723000	40.083874000	38.825629000
O	42.864235000	41.071958000	36.629896000
O	44.531695000	42.198560000	34.912981000
O	44.162297000	49.310214000	34.973999000
O	43.719743000	46.753648000	32.358299000
O	44.042854000	44.414227000	33.561435000
O	46.177716000	45.923651000	32.943299000
O	48.392580000	44.425683000	33.149675000
O	48.560194000	46.817001000	32.028424000
O	50.370697000	47.634064000	35.495864000
O	48.185013000	47.965831000	36.963155000
O	48.044624000	46.606678000	34.665132000
O	48.433275000	49.249994000	34.624160000
O	46.833760000	48.778759000	39.108620000
O	46.897401000	50.306307000	36.894937000
O	45.510979000	48.009282000	36.922673000
O	41.623104000	48.823616000	38.817861000
O	42.850403000	47.746024000	36.667195000
O	44.506748000	46.624364000	34.928535000
O	43.726438000	42.075923000	32.353030000
O	46.183778000	42.928735000	32.920361000
O	48.572211000	42.035170000	32.020700000
O	54.222962000	44.409451000	38.646039000
O	56.341204000	42.942777000	39.385721000
O	55.467998000	47.973007000	35.213733000
O	51.847774000	48.702875000	33.534320000
O	52.917323000	47.686442000	35.802125000
O	51.593467000	50.049263000	35.800609000
O	54.792035000	46.692330000	37.397342000
O	54.385198000	49.376104000	37.244920000
O	56.341659000	45.877505000	39.395681000
O	55.472444000	40.844064000	35.217800000
O	51.869008000	40.075255000	33.518888000
O	52.920052000	41.120062000	35.789925000
O	54.776731000	42.126473000	37.394682000
O	46.066842000	42.112804000	40.494122000
O	44.262647000	40.283631000	39.582854000

O	46.046672000	39.627997000	41.498159000
O	42.454988000	41.967944000	40.594843000
O	42.244715000	49.364316000	41.291177000
O	43.651465000	48.460957000	43.534878000
O	48.786653000	48.627906000	42.972371000
O	42.802231000	45.981901000	42.979881000
O	41.607823000	44.415396000	41.135988000
O	43.759788000	46.581737000	45.404880000
O	45.398543000	46.429397000	43.293810000
O	48.632938000	46.832217000	44.942692000
O	47.865369000	46.186041000	42.393847000
O	45.989007000	44.432427000	41.524874000
O	45.855092000	46.848585000	40.663675000
O	44.196197000	48.666694000	39.600970000
O	45.936041000	49.359601000	41.546051000
O	42.389030000	46.840993000	40.460265000
O	43.169879000	40.399517000	43.653518000
O	48.754826000	40.212144000	43.046759000
O	42.728304000	42.979058000	43.100612000
O	43.706477000	42.236612000	45.463275000
O	45.229615000	42.074616000	43.258538000
O	48.770527000	41.894837000	45.144700000
O	47.956801000	42.769114000	42.739366000
O	53.545990000	40.324683000	41.890063000
O	50.512898000	42.172702000	43.093965000
O	52.916745000	42.892612000	42.283439000
O	51.666171000	44.394331000	44.101657000
O	53.870074000	42.072381000	39.901942000
O	55.475621000	42.155436000	42.037451000
O	52.427414000	46.825307000	44.776413000
O	53.534263000	48.489014000	41.889369000
O	50.422271000	46.512636000	42.976280000
O	52.891108000	45.923933000	42.273762000
O	53.870828000	46.748145000	39.900784000
O	55.454420000	46.654199000	42.051172000
O	52.569837000	41.993397000	44.809341000
Ga	45.931914000	43.938210000	39.514971000
C	50.263875000	43.910201000	37.578844000
H	51.286112000	43.873849000	37.178737000
C	49.241396000	44.327887000	36.799911000
H	49.424563000	44.621188000	35.758754000
C	50.037482000	43.515256000	38.967362000
C	48.862629000	43.783984000	39.583521000
H	50.862311000	43.061972000	39.533949000
H	44.452823000	44.010948000	38.993855000
H	48.774359000	43.588510000	40.660281000
C	47.817619000	44.305298000	37.310482000
C	47.732879000	44.471905000	38.849807000
H	47.355983000	43.345608000	36.985427000
H	47.215450000	45.092652000	36.816413000
H	47.800516000	45.561757000	39.093922000

TS14-GaH$_2$

Electronic energy (r^{2}SCAN-D4/def2-SVP): -48410.62853569 E_h
Electronic energy (ωB97X-D4rev/def2-mTZVPP): -48454.677493084700 E_h
G_{corr} (673.15 K, 1 atm): 2.01146442 E_h
Number of atoms: 391

Cartesian coordinates (Å)

Si	48.554310000	36.023652000	35.305047000
Si	46.527749000	36.956761000	37.337550000
Si	44.099636000	36.090059000	35.630689000
Si	44.627638000	38.488790000	33.717059000
Si	47.774544000	38.511737000	33.524620000
Si	48.749654000	38.761777000	30.501806000
Si	51.806190000	38.126387000	30.325909000
Si	54.676558000	38.465388000	38.541401000
Si	57.776575000	38.489356000	38.804270000
Si	56.554072000	37.020562000	34.997152000
Si	52.596427000	36.084832000	32.666285000
Si	51.477012000	37.159802000	35.489670000
Si	54.133054000	36.094218000	36.718115000
Si	45.625163000	36.080563000	40.033965000
Si	41.743319000	38.013346000	41.941527000
Si	43.516127000	38.864362000	44.076608000
Si	47.728419000	38.462858000	46.629950000
Si	48.752514000	38.667660000	43.629113000
Si	46.061301000	38.224245000	42.324430000
Si	51.699146000	37.984365000	43.458363000
Si	53.654058000	38.774533000	41.351058000
Si	56.184891000	38.105359000	43.085184000
Si	44.612712000	50.310141000	33.739556000
Si	47.758938000	50.280048000	33.563781000
Si	48.739113000	50.060808000	30.531526000
Si	43.866647000	46.934416000	30.715857000
Si	48.869737000	46.959692000	30.437346000
Si	46.324013000	45.965999000	29.028888000
Si	48.555076000	52.788783000	35.320962000
Si	46.531512000	51.829385000	37.375684000
Si	44.105720000	52.726588000	35.641405000
Si	43.866680000	41.885016000	30.710150000
Si	48.877996000	41.863565000	30.399520000
Si	46.321097000	42.859493000	29.021238000
Si	51.881629000	42.865817000	30.520276000
Si	57.965709000	42.902098000	39.187209000
Si	58.734120000	47.858501000	36.909719000
Si	56.878986000	48.675153000	34.759572000
Si	55.870635000	48.297469000	31.974523000
Si	52.768527000	48.323710000	32.241213000
Si	56.556918000	51.799238000	34.996032000
Si	52.599432000	52.737652000	32.657957000
Si	51.490193000	51.661091000	35.477006000
Si	54.135230000	52.728385000	36.714126000
Si	51.807074000	50.694736000	30.331331000
Si	54.678199000	50.352393000	38.534941000
Si	57.777606000	50.331489000	38.802977000
Si	51.883682000	45.956979000	30.525141000
Si	57.963486000	45.925514000	39.182783000
Si	58.734104000	40.963172000	36.913439000

Si	56.868058000	40.146157000	34.765368000
Si	55.874309000	40.524787000	31.972267000
Si	52.768553000	40.493377000	32.226928000
Si	48.657595000	40.952511000	48.519849000
Si	41.739005000	50.799624000	41.948547000
Si	43.655481000	50.033162000	44.074924000
Si	47.726877000	50.362340000	46.611854000
Si	48.764065000	50.163000000	43.582776000
Si	46.062981000	50.774451000	42.365390000
Si	44.745067000	45.948278000	46.551821000
Si	47.917854000	45.953650000	46.150514000
Si	48.672102000	47.887425000	48.512982000
Si	45.614198000	52.771115000	40.039064000
Si	44.774124000	42.847758000	46.536795000
Si	47.938962000	42.849081000	46.188591000
Si	58.955548000	41.840827000	41.948160000
Si	56.313532000	42.836268000	43.277142000
Si	55.794437000	48.300285000	45.167680000
Si	52.635676000	48.362347000	45.325990000
Si	51.670758000	48.627357000	48.352652000
Si	51.722098000	50.837873000	43.455632000
Si	53.660270000	50.038144000	41.344055000
Si	56.183245000	50.710758000	43.088076000
Si	58.956729000	46.980727000	41.934359000
Si	56.310797000	45.967642000	43.267830000
Si	55.798501000	40.496772000	45.164039000
Si	52.667827000	40.431291000	45.330062000
Si	51.669243000	40.195656000	48.346574000
O	49.972039000	36.641241000	35.864870000
O	47.506071000	35.888034000	36.571157000
O	46.666184000	36.824469000	38.960620000
O	44.965268000	36.650801000	36.930903000
O	44.500245000	36.936618000	34.256462000
O	46.180536000	38.839148000	33.260071000
O	47.926202000	36.992902000	34.122592000
O	48.609320000	38.642224000	32.131128000
O	50.191195000	38.134725000	30.062565000
O	52.213738000	36.846379000	31.258226000
O	52.298871000	39.562848000	30.966187000
O	54.606389000	36.892409000	38.076804000
O	56.173353000	38.737101000	39.138092000
O	56.902131000	38.601535000	35.315044000
O	54.988963000	36.657565000	35.418261000
O	51.811165000	36.852431000	33.911441000
O	52.530841000	36.336455000	36.441713000
O	42.380171000	37.846081000	43.459420000
O	45.015269000	38.457746000	43.544561000
O	48.602805000	38.698225000	45.252630000
O	47.521464000	37.841176000	42.937896000
O	45.579133000	36.943365000	41.433416000
O	50.091335000	37.821704000	43.226368000
O	52.395291000	37.934711000	41.963196000
O	52.049826000	39.410950000	44.207387000
O	53.571496000	38.741712000	39.715191000
O	55.064695000	38.111880000	41.867572000
O	44.495129000	51.865802000	34.273415000
O	46.166361000	49.953021000	33.291817000
O	47.907332000	51.801126000	34.162968000

O	48.589605000	50.168147000	32.164120000
O	48.580250000	48.509448000	30.007902000
O	45.412986000	46.593510000	30.243987000
O	47.898805000	45.993714000	29.528375000
O	45.872342000	44.413575000	28.742244000
O	49.985169000	52.177561000	35.851872000
O	47.535776000	52.883358000	36.617425000
O	46.644954000	51.961168000	39.006057000
O	44.982759000	52.169235000	36.938025000
O	48.591291000	40.312105000	29.975659000
O	45.411207000	42.221923000	30.230683000
O	47.900821000	42.830731000	29.501759000
O	50.434490000	42.226122000	30.071798000
O	51.662902000	44.410538000	31.025291000
O	58.579713000	44.414293000	39.359419000
O	58.133567000	47.836299000	35.379233000
O	58.284001000	46.474685000	37.676027000
O	58.214902000	49.190140000	37.703517000
O	56.998710000	48.628715000	33.135201000
O	56.907373000	50.219104000	35.311691000
O	54.349853000	48.576587000	32.581279000
O	52.556737000	46.757638000	31.799642000
O	54.992332000	52.161989000	35.417323000
O	51.813618000	51.957552000	33.894634000
O	52.534046000	52.500763000	36.424448000
O	50.186451000	50.683146000	30.099800000
O	52.224028000	51.987810000	31.242361000
O	52.304552000	49.266566000	30.986137000
O	54.597224000	51.925908000	38.073757000
O	56.174231000	50.089016000	39.136896000
O	50.428863000	46.602819000	30.109994000
O	58.115674000	40.991867000	35.389915000
O	58.298169000	42.347249000	37.685694000
O	58.217750000	39.629887000	37.705607000
O	56.993671000	40.194827000	33.141222000
O	54.349776000	40.249205000	32.565189000
O	52.532928000	42.061026000	31.804479000
O	50.276739000	40.957103000	48.774239000
O	48.226907000	42.325422000	47.714627000
O	48.195651000	39.588142000	47.735442000
O	42.437758000	50.939028000	43.441582000
O	45.136509000	50.640934000	43.699409000
O	48.541610000	50.103886000	45.198220000
O	47.599278000	51.026436000	42.828559000
O	45.562918000	52.070866000	41.521917000
O	44.343864000	44.396613000	46.867264000
O	46.290442000	46.019071000	45.997517000
O	48.446554000	44.405096000	46.021106000
O	50.280443000	47.888129000	48.821501000
O	48.311870000	46.547665000	47.623868000
O	48.226243000	49.256515000	47.724502000
O	46.313367000	42.778409000	45.932822000
O	58.654010000	41.944932000	40.316638000
O	57.904727000	42.728057000	42.879800000
O	55.897370000	44.402280000	43.473768000
O	55.991802000	46.732993000	44.687159000
O	54.238954000	48.604311000	45.629845000
O	56.186018000	49.268510000	43.895344000

O	51.803250000	48.588491000	46.705685000
O	50.116250000	50.999597000	43.233381000
O	52.411240000	50.891599000	41.960186000
O	52.064159000	49.417418000	44.212925000
O	53.574441000	50.070946000	39.709386000
O	55.073173000	50.694889000	41.861684000
O	58.666914000	46.889417000	40.299330000
O	57.893030000	46.080244000	42.837698000
O	55.953814000	42.070774000	44.686489000
O	54.261079000	40.121802000	45.629833000
O	56.213956000	39.553739000	43.880690000
O	51.827771000	40.217897000	46.703810000
H	48.702871000	34.676568000	34.772566000
H	44.419875000	34.676323000	35.346829000
H	42.683628000	36.258975000	36.022159000
H	43.726523000	38.680216000	32.577941000
H	47.730594000	38.010996000	29.830569000
H	52.421136000	38.002380000	29.030380000
H	58.465353000	38.743363000	40.066967000
H	58.088382000	37.108509000	38.286195000
H	56.651061000	36.758841000	33.558678000
H	57.541582000	36.246995000	35.782467000
H	52.141234000	34.673922000	32.562045000
H	54.071037000	36.146097000	32.821438000
H	54.418082000	34.675478000	36.917895000
H	44.265382000	36.148734000	39.494230000
H	46.085045000	34.677316000	40.196440000
H	42.177304000	36.886192000	41.136491000
H	40.290195000	38.107003000	42.090377000
H	43.614692000	38.689573000	45.487247000
H	46.266760000	38.594605000	46.380864000
H	48.065374000	37.113072000	47.131273000
H	52.195251000	36.881484000	44.271734000
H	55.858296000	36.988842000	44.011824000
H	57.529558000	37.965437000	42.519250000
H	41.636485000	38.780154000	36.634495000
H	40.338249000	40.845235000	36.817795000
H	43.726296000	50.140518000	32.578071000
H	47.730502000	50.809921000	29.830551000
H	42.910974000	46.071594000	29.969678000
H	43.636013000	48.346500000	30.474316000
H	46.055518000	46.690813000	27.779371000
H	40.338195000	47.975592000	36.817799000
H	41.636403000	50.040687000	36.634467000
H	48.702714000	54.144377000	34.772532000
H	44.419585000	54.144606000	35.346948000
H	42.683575000	52.561616000	36.022276000
H	43.636069000	40.474361000	30.474327000
H	42.910998000	42.749253000	29.969681000
H	46.055551000	42.130076000	27.779377000
H	52.768240000	42.805898000	29.339147000
H	60.152183000	47.950749000	36.769108000
H	56.115708000	49.276599000	30.928772000
H	56.049418000	46.904457000	31.507081000
H	57.541465000	52.574079000	35.782446000
H	56.650952000	52.062214000	33.558656000
H	52.141090000	54.147065000	32.562019000
H	54.070917000	52.674925000	32.821418000

H	54.418140000	54.145525000	36.917811000
H	52.421044000	50.818602000	29.030363000
H	58.465269000	50.077736000	40.066952000
H	58.088278000	51.712582000	38.286179000
H	52.768217000	46.015092000	29.339142000
H	60.152234000	40.870368000	36.769119000
H	56.049458000	41.916585000	31.507090000
H	56.115777000	39.544444000	30.928783000
H	48.027948000	40.958436000	49.819979000
H	40.290115000	50.713683000	42.090582000
H	42.176850000	51.934815000	41.136521000
H	43.615015000	50.131729000	45.487215000
H	48.065229000	51.707918000	47.131232000
H	46.266685000	50.226321000	46.380825000
H	40.476352000	46.423809000	42.229530000
H	44.578030000	46.685732000	47.785515000
H	48.027871000	47.862537000	49.819962000
H	46.084739000	54.143668000	40.196331000
H	44.265252000	52.672179000	39.494179000
H	40.476382000	42.397038000	42.229542000
H	44.578055000	42.135174000	47.785513000
H	58.818796000	40.408567000	42.221796000
H	60.309668000	42.345550000	42.255160000
H	56.725235000	48.548904000	46.274187000
H	51.671681000	50.045758000	48.769745000
H	52.721673000	47.848745000	49.007593000
H	52.195144000	51.939534000	44.271718000
H	55.858180000	51.832229000	44.011806000
H	57.529465000	50.855664000	42.519236000
H	60.309639000	46.475584000	42.255156000
H	58.818736000	48.412544000	42.221786000
H	56.725177000	40.272281000	46.274313000
H	52.722235000	40.971967000	49.006966000
H	51.671815000	38.775269000	48.769821000
Si	48.768972000	40.971682000	35.366714000
Si	46.880473000	40.005217000	37.397582000
Si	41.596113000	40.166665000	37.171176000
Si	44.274699000	40.869243000	35.827594000
Si	44.624246000	45.935950000	33.469989000
Si	47.810873000	45.933577000	33.207103000
Si	48.768825000	47.829766000	35.437000000
Si	46.859135000	48.754508000	37.480190000
Si	41.567661000	48.677337000	37.186313000
Si	44.255421000	47.937914000	35.871785000
Si	44.643543000	42.886756000	33.443127000
Si	47.802374000	42.886773000	33.157606000
Si	54.805847000	42.891439000	38.833957000
Si	51.691943000	48.541334000	35.163700000
Si	54.399609000	47.947386000	36.443923000
Si	54.807035000	45.932288000	38.837041000
Si	51.685117000	40.271532000	35.144205000
Si	54.384368000	40.867451000	36.447035000
Si	45.856854000	40.525212000	40.157573000
Si	42.719122000	40.446896000	40.071140000
Si	41.773079000	45.951432000	41.708384000
Si	43.858006000	46.904714000	43.822594000
Si	48.884719000	47.038003000	43.346348000
Si	46.222946000	46.000569000	42.062499000

Si	45.680182000	48.405691000	40.231746000
Si	42.621045000	48.418138000	40.034377000
Si	41.768776000	42.914370000	41.772066000
Si	43.725865000	41.895703000	43.827481000
Si	48.960304000	41.774450000	43.496973000
Al	46.293213000	42.815715000	42.177570000
Si	51.893201000	42.862278000	43.581696000
Si	53.935436000	41.868600000	41.532248000
Si	51.820186000	45.921183000	43.540738000
Si	53.905740000	46.944400000	41.529843000
O	46.936545000	38.465234000	36.822885000
O	44.169507000	39.493656000	34.939399000
O	54.379816000	39.438537000	37.255374000
O	51.560079000	38.773148000	35.803997000
O	42.390592000	39.355876000	41.231796000
O	50.376886000	41.207683000	35.428868000
O	48.202088000	40.821707000	36.892097000
O	48.046273000	42.248815000	34.643339000
O	48.436629000	39.594874000	34.550657000
O	46.903097000	39.995869000	39.022061000
O	45.507966000	40.739696000	36.891997000
O	41.738720000	40.084401000	38.813479000
O	42.845506000	41.088027000	36.583131000
O	44.576739000	42.178949000	34.903365000
O	44.156389000	49.310264000	34.971830000
O	43.729729000	46.743733000	32.358270000
O	44.057751000	44.406423000	33.568521000
O	46.191552000	45.932463000	32.984163000
O	48.406207000	44.409349000	33.111751000
O	48.562460000	46.825696000	32.055813000
O	50.379739000	47.635300000	35.511220000
O	48.189900000	47.944981000	36.968123000
O	48.099921000	46.521085000	34.709827000
O	48.400112000	49.189173000	34.600643000
O	46.832116000	48.728923000	39.109112000
O	46.917811000	50.299425000	36.913735000
O	45.527031000	48.004000000	36.895994000
O	41.606077000	48.837994000	38.826883000
O	42.854259000	47.757421000	36.688726000
O	44.477969000	46.620446000	34.935455000
O	43.727726000	42.074925000	32.352865000
O	46.190316000	42.927098000	32.897302000
O	48.575904000	42.003595000	32.016544000
O	54.226164000	44.411622000	38.651066000
O	56.342428000	42.939344000	39.389498000
O	55.473447000	47.972721000	35.216808000
O	51.848688000	48.698138000	33.537619000
O	52.923564000	47.691280000	35.806865000
O	51.590726000	50.052445000	35.800312000
O	54.795917000	46.694347000	37.401949000
O	54.391361000	49.379687000	37.247792000
O	56.342331000	45.882736000	39.404319000
O	55.454338000	40.839013000	35.216687000
O	51.871719000	40.085420000	33.527400000
O	52.902676000	41.123227000	35.816974000
O	54.772711000	42.127585000	37.399379000
O	46.195404000	42.139363000	40.453103000
O	44.309090000	40.334444000	39.634476000

O	46.147337000	39.634740000	41.483866000
O	42.447443000	41.973086000	40.596141000
O	42.272695000	49.392314000	41.287091000
O	43.491954000	48.477451000	43.570750000
O	48.812914000	48.636345000	42.978724000
O	42.831999000	45.968383000	42.968620000
O	41.603170000	44.421545000	41.134714000
O	43.728970000	46.581884000	45.421543000
O	45.401215000	46.628858000	43.341040000
O	48.600326000	46.853334000	44.958680000
O	47.823352000	46.214002000	42.407922000
O	45.866801000	44.456075000	41.760691000
O	45.816632000	46.849620000	40.703926000
O	44.189044000	48.659846000	39.573670000
O	45.919303000	49.389456000	41.507352000
O	42.370549000	46.857683000	40.463719000
O	43.060193000	40.396331000	43.686361000
O	48.793917000	40.198140000	43.052333000
O	42.731220000	42.984328000	43.101013000
O	43.744519000	42.224317000	45.433654000
O	45.189393000	41.921501000	43.152465000
O	48.767831000	41.890983000	45.144714000
O	47.919229000	42.740363000	42.745615000
O	53.541476000	40.323836000	41.889844000
O	50.489231000	42.210157000	43.082765000
O	52.900909000	42.889125000	42.277881000
O	51.683765000	44.395624000	44.115818000
O	53.864924000	42.074714000	39.903253000
O	55.467364000	42.160145000	42.040912000
O	52.416118000	46.837282000	44.765662000
O	53.524853000	48.493240000	41.888230000
O	50.380842000	46.475470000	42.993935000
O	52.850857000	45.934166000	42.259414000
O	53.864038000	46.749674000	39.900707000
O	55.428312000	46.643775000	42.060333000
O	52.555531000	41.982514000	44.800517000
Ga	46.268803000	43.675476000	39.196432000
C	50.196091000	43.340982000	38.065194000
H	51.123305000	42.921690000	37.651463000
C	49.266779000	43.890549000	37.211020000
H	49.439617000	43.933934000	36.130124000
C	50.014143000	43.352006000	39.485727000
C	49.021974000	44.138388000	40.028470000
H	50.754621000	42.870867000	40.134785000
H	44.864889000	44.234313000	38.806948000
H	49.013975000	44.333973000	41.107651000
C	48.032344000	44.404217000	37.750655000
C	48.007914000	44.778990000	39.202220000
H	47.037509000	43.200411000	37.673097000
H	47.420283000	45.028183000	37.079239000
H	47.779951000	45.833301000	39.432591000

I21-GaH_2

Electronic energy (r^2SCAN-D4/def2-SVP): -48410.68901830 E_h
Electronic energy (ωB97X-D4rev/def2-mTZVPP): -48454.744691548700 E_h
G_{corr} (673.15 K, 1 atm): 2.00986611 E_h
Number of atoms: 391

Cartesian coordinates (Å)

Si	48.554182000	36.020588000	35.318786000
Si	46.526112000	36.948992000	37.347896000
Si	44.099779000	36.088516000	35.632921000
Si	44.630010000	38.486871000	33.713364000
Si	47.772516000	38.506452000	33.516672000
Si	48.752297000	38.754281000	30.503157000
Si	51.807171000	38.127952000	30.324080000
Si	54.678107000	38.466585000	38.547131000
Si	57.776070000	38.489510000	38.805280000
Si	56.554807000	37.020758000	34.996929000
Si	52.595525000	36.084836000	32.667789000
Si	51.478439000	37.154783000	35.495093000
Si	54.136166000	36.095007000	36.720779000
Si	45.622703000	36.081682000	40.043727000
Si	41.742806000	38.011379000	41.944477000
Si	43.484783000	38.875103000	44.081133000
Si	47.728232000	38.464044000	46.634802000
Si	48.749108000	38.663556000	43.643190000
Si	46.053307000	38.234199000	42.327407000
Si	51.687760000	37.978130000	43.457427000
Si	53.644672000	38.773869000	41.354702000
Si	56.183959000	38.106313000	43.085786000
Si	44.612079000	50.308713000	33.739496000
Si	47.758568000	50.290987000	33.551357000
Si	48.744154000	50.062196000	30.521456000
Si	43.865747000	46.934709000	30.712671000
Si	48.870530000	46.963949000	30.425576000
Si	46.323497000	45.964908000	29.026445000
Si	48.550617000	52.787228000	35.315068000
Si	46.528732000	51.819178000	37.381595000
Si	44.106710000	52.725323000	35.643812000
Si	43.867216000	41.884182000	30.711196000
Si	48.876730000	41.864794000	30.423726000
Si	46.324578000	42.857137000	29.024596000
Si	51.885429000	42.862895000	30.525188000
Si	57.964370000	42.900778000	39.186348000
Si	58.733490000	47.858460000	36.910823000
Si	56.874192000	48.674934000	34.761928000
Si	55.871820000	48.297631000	31.973733000
Si	52.769513000	48.327019000	32.235323000
Si	56.555505000	51.799901000	34.996253000
Si	52.600582000	52.738108000	32.657745000
Si	51.487559000	51.660749000	35.473559000
Si	54.131914000	52.728918000	36.712156000
Si	51.807386000	50.699218000	30.329889000
Si	54.675305000	50.351413000	38.537878000
Si	57.776464000	50.332057000	38.803796000
Si	51.886188000	45.953761000	30.527009000
Si	57.963763000	45.925469000	39.183215000
Si	58.734038000	40.963645000	36.913972000

Si	56.874126000	40.146030000	34.763819000
Si	55.879197000	40.524593000	31.972287000
Si	52.777216000	40.488857000	32.224247000
Si	48.654853000	40.952974000	48.519815000
Si	41.739497000	50.806955000	41.947178000
Si	43.591619000	50.018264000	44.078923000
Si	47.726995000	50.361464000	46.616845000
Si	48.755644000	50.164354000	43.595594000
Si	46.047971000	50.736664000	42.350654000
Si	44.745534000	45.942944000	46.553500000
Si	47.910610000	45.955669000	46.158375000
Si	48.666107000	47.883725000	48.513735000
Si	45.612591000	52.766300000	40.047432000
Si	44.770249000	42.849929000	46.538280000
Si	47.928062000	42.852004000	46.188452000
Si	58.954940000	41.840550000	41.944378000
Si	56.307097000	42.837026000	43.273398000
Si	55.795985000	48.302752000	45.164471000
Si	52.634303000	48.367759000	45.317804000
Si	51.670456000	48.626296000	48.349141000
Si	51.708102000	50.842931000	43.454237000
Si	53.653404000	50.041470000	41.348212000
Si	56.183347000	50.711127000	43.088054000
Si	58.956272000	46.980817000	41.934240000
Si	56.307172000	45.968931000	43.266989000
Si	55.799115000	40.498710000	45.161477000
Si	52.660063000	40.431566000	45.319808000
Si	51.670122000	40.195720000	48.343593000
O	49.974902000	36.635954000	35.874536000
O	47.512292000	35.880232000	36.590040000
O	46.663519000	36.829528000	38.971757000
O	44.966126000	36.638681000	36.936967000
O	44.502012000	36.938026000	34.261295000
O	46.179897000	38.827855000	33.240240000
O	47.918981000	37.003698000	34.153304000
O	48.621364000	38.591660000	32.129009000
O	50.195043000	38.143145000	30.047158000
O	52.206585000	36.850565000	31.263686000
O	52.306399000	39.563903000	30.960598000
O	54.609911000	36.894655000	38.078200000
O	56.173852000	38.734685000	39.147382000
O	56.905519000	38.601306000	35.312685000
O	54.989386000	36.659877000	35.419749000
O	51.811914000	36.846407000	33.917675000
O	52.533182000	36.335896000	36.448818000
O	42.363871000	37.837520000	43.468085000
O	44.978639000	38.504609000	43.511980000
O	48.617533000	38.711943000	45.268230000
O	47.495317000	37.845538000	42.983595000
O	45.581835000	36.940143000	41.446078000
O	50.078670000	37.804098000	43.231688000
O	52.381400000	37.934027000	41.959787000
O	52.034585000	39.405988000	44.205257000
O	53.567915000	38.739551000	39.717390000
O	55.054883000	38.113754000	41.875495000
O	44.496366000	51.863407000	34.276221000
O	46.167008000	49.953867000	33.290983000
O	47.905946000	51.815616000	34.141072000

O	48.587483000	50.160410000	32.153264000
O	48.602398000	48.514359000	29.983521000
O	45.412564000	46.591065000	30.242443000
O	47.900355000	45.998151000	29.516943000
O	45.878063000	44.411011000	28.740244000
O	49.980554000	52.172073000	35.843631000
O	47.532235000	52.867298000	36.612121000
O	46.645285000	51.961216000	39.010707000
O	44.981450000	52.163560000	36.940259000
O	48.585590000	40.312320000	30.006693000
O	45.411439000	42.225685000	30.234781000
O	47.901061000	42.826493000	29.517144000
O	50.433643000	42.224794000	30.089688000
O	51.675814000	44.407937000	31.032682000
O	58.577214000	44.413332000	39.359866000
O	58.128134000	47.835638000	35.382097000
O	58.285101000	46.473649000	37.676246000
O	58.213084000	49.189458000	37.705038000
O	56.996420000	48.627828000	33.137692000
O	56.902316000	50.218973000	35.313534000
O	54.350150000	48.580264000	32.575379000
O	52.556489000	46.759844000	31.800050000
O	54.990636000	52.163518000	35.415898000
O	51.813579000	51.953870000	33.891168000
O	52.530525000	52.503359000	36.419845000
O	50.187136000	50.700344000	30.099377000
O	52.232145000	51.989199000	31.240390000
O	52.297813000	49.266621000	30.981368000
O	54.591351000	51.922630000	38.070205000
O	56.172701000	50.092411000	39.138548000
O	50.428331000	46.590463000	30.112801000
O	58.122103000	40.990904000	35.387715000
O	58.295243000	42.348499000	37.683100000
O	58.210501000	39.632094000	37.705389000
O	57.000191000	40.196331000	33.139772000
O	54.356809000	40.244579000	32.567932000
O	52.542534000	42.057813000	31.807410000
O	50.275262000	40.956837000	48.764978000
O	48.221196000	42.323148000	47.711098000
O	48.181651000	39.586009000	47.747962000
O	42.409224000	50.971796000	43.451572000
O	45.089070000	50.537058000	43.650551000
O	48.559567000	50.102677000	45.214269000
O	47.565357000	51.013468000	42.867142000
O	45.557151000	52.052103000	41.525392000
O	44.359932000	44.395043000	46.901995000
O	46.284256000	46.018616000	45.971783000
O	48.439336000	44.406830000	46.024141000
O	50.278649000	47.878784000	48.802215000
O	48.279071000	46.539564000	47.642203000
O	48.217076000	49.254678000	47.731878000
O	46.305465000	42.777184000	45.924626000
O	58.658248000	41.941836000	40.311262000
O	57.895948000	42.728481000	42.865373000
O	55.894467000	44.403589000	43.472721000
O	55.991508000	46.734109000	44.687079000
O	54.238703000	48.615884000	45.613280000
O	56.196766000	49.267136000	43.891882000

O	51.812871000	48.591819000	46.703611000
O	50.101355000	51.009381000	43.230046000
O	52.400852000	50.893553000	41.959219000
O	52.047673000	49.420294000	44.209629000
O	53.571653000	50.073665000	39.712908000
O	55.066478000	50.696625000	41.867422000
O	58.668775000	46.889188000	40.298608000
O	57.888860000	46.081483000	42.834012000
O	55.960939000	42.072757000	44.686842000
O	54.256265000	40.127210000	45.612159000
O	56.220063000	39.555016000	43.880002000
O	51.832945000	40.218572000	46.701604000
H	48.702871000	34.676569000	34.772565000
H	44.419874000	34.676324000	35.346828000
H	42.683628000	36.258977000	36.022158000
H	43.726523000	38.680219000	32.577941000
H	47.730594000	38.010998000	29.830568000
H	52.421136000	38.002381000	29.030380000
H	58.465353000	38.743362000	40.066967000
H	58.088382000	37.108508000	38.286194000
H	56.651060000	36.758841000	33.558678000
H	57.541582000	36.246994000	35.782467000
H	52.141233000	34.673923000	32.562044000
H	54.071037000	36.146098000	32.821438000
H	54.418081000	34.675478000	36.917894000
H	44.265382000	36.148736000	39.494230000
H	46.085044000	34.677317000	40.196439000
H	42.177303000	36.886193000	41.136491000
H	40.290194000	38.107005000	42.090376000
H	43.614691000	38.689574000	45.487246000
H	46.266760000	38.594606000	46.380864000
H	48.065373000	37.113072000	47.131272000
H	52.195250000	36.881484000	44.271734000
H	55.858295000	36.988841000	44.011824000
H	57.529557000	37.965437000	42.519250000
H	41.636484000	38.780154000	36.634494000
H	40.338249000	40.845235000	36.817795000
H	43.726297000	50.140520000	32.578071000
H	47.730504000	50.809923000	29.830551000
H	42.910975000	46.071597000	29.969677000
H	43.636014000	48.346502000	30.474317000
H	46.055519000	46.690814000	27.779371000
H	40.338195000	47.975593000	36.817798000
H	41.636404000	50.040687000	36.634467000
H	48.702716000	54.144378000	34.772533000
H	44.419587000	54.144608000	35.346948000
H	42.683577000	52.561618000	36.022276000
H	43.636070000	40.474363000	30.474325000
H	42.910998000	42.749255000	29.969681000
H	46.055551000	42.130078000	27.779376000
H	52.768241000	42.805899000	29.339146000
H	60.152184000	47.950748000	36.769108000
H	56.115709000	49.276600000	30.928772000
H	56.049419000	46.904457000	31.507082000
H	57.541466000	52.574079000	35.782447000
H	56.650954000	52.062214000	33.558656000
H	52.141092000	54.147066000	32.562020000
H	54.070919000	52.674925000	32.821419000

H	54.418142000	54.145525000	36.917812000
H	52.421046000	50.818603000	29.030363000
H	58.465270000	50.077735000	40.066953000
H	58.088280000	51.712581000	38.286180000
H	52.768218000	46.015092000	29.339142000
H	60.152234000	40.870367000	36.769119000
H	56.049459000	41.916585000	31.507090000
H	56.115777000	39.544444000	30.928783000
H	48.027948000	40.958436000	49.819978000
H	40.290116000	50.713686000	42.090582000
H	42.176852000	51.934816000	41.136521000
H	43.615016000	50.131730000	45.487215000
H	48.065230000	51.707918000	47.131232000
H	46.266686000	50.226322000	46.380825000
H	40.476352000	46.423809000	42.229530000
H	44.578030000	46.685733000	47.785515000
H	48.027871000	47.862537000	49.819962000
H	46.084741000	54.143669000	40.196332000
H	44.265254000	52.672181000	39.494180000
H	40.476382000	42.397039000	42.229542000
H	44.578055000	42.135175000	47.785513000
H	58.818795000	40.408566000	42.221796000
H	60.309669000	42.345549000	42.255161000
H	56.725236000	48.548903000	46.274187000
H	51.671682000	50.045758000	48.769745000
H	52.721673000	47.848744000	49.007594000
H	52.195146000	51.939533000	44.271719000
H	55.858182000	51.832229000	44.011806000
H	57.529467000	50.855663000	42.519236000
H	60.309640000	46.475583000	42.255157000
H	58.818737000	48.412543000	42.221787000
H	56.725176000	40.272280000	46.274313000
H	52.722235000	40.971966000	49.006966000
H	51.671814000	38.775268000	48.769820000
Si	48.783292000	40.978655000	35.348987000
Si	46.893556000	40.002933000	37.391379000
Si	41.597188000	40.162338000	37.179326000
Si	44.279844000	40.868818000	35.828376000
Si	44.610553000	45.933373000	33.462866000
Si	47.797035000	45.947716000	33.190376000
Si	48.767082000	47.843994000	35.411772000
Si	46.865889000	48.729670000	37.469815000
Si	41.569664000	48.679129000	37.190864000
Si	44.252170000	47.930424000	35.865036000
Si	44.642177000	42.883509000	33.446346000
Si	47.812097000	42.902951000	33.179279000
Si	54.803804000	42.889415000	38.836424000
Si	51.694589000	48.544780000	35.157996000
Si	54.395895000	47.942552000	36.446827000
Si	54.806018000	45.932183000	38.839991000
Si	51.696586000	40.263048000	35.138262000
Si	54.392253000	40.870091000	36.448641000
Si	45.867416000	40.545760000	40.166200000
Si	42.725337000	40.442849000	40.077494000
Si	41.773843000	45.946773000	41.710482000
Si	43.806158000	46.927404000	43.839952000
Si	48.884564000	47.043275000	43.376600000
Si	46.185426000	46.035617000	42.159900000

Si	45.705712000	48.346561000	40.223327000
Si	42.639728000	48.420083000	40.034728000
Si	41.763652000	42.916393000	41.763608000
Si	43.696512000	41.884967000	43.827061000
Si	48.933396000	41.771504000	43.491606000
Al	46.223993000	42.822815000	42.198108000
Si	51.871835000	42.866561000	43.569237000
Si	53.918164000	41.868527000	41.529354000
Si	51.815588000	45.923873000	43.537059000
Si	53.899607000	46.947467000	41.528845000
O	46.927804000	38.458088000	36.831660000
O	44.181634000	39.496855000	34.935573000
O	54.386108000	39.444833000	37.263402000
O	51.561276000	38.769595000	35.806841000
O	42.407079000	39.353583000	41.244524000
O	50.392550000	41.205696000	35.415854000
O	48.242432000	40.768069000	36.875154000
O	48.082575000	42.293924000	34.675247000
O	48.426286000	39.627871000	34.504206000
O	46.914824000	40.037876000	39.015662000
O	45.533313000	40.753653000	36.872691000
O	41.745320000	40.066827000	38.821102000
O	42.859097000	41.075037000	36.603357000
O	44.552021000	42.190570000	34.912886000
O	44.153561000	49.303526000	34.965293000
O	43.718619000	46.745633000	32.353150000
O	44.049777000	44.401440000	33.547411000
O	46.177251000	45.934841000	32.976980000
O	48.399740000	44.428084000	33.086113000
O	48.542382000	46.846941000	32.039824000
O	50.379546000	47.632978000	35.487768000
O	48.211893000	47.949583000	36.950418000
O	48.089017000	46.546222000	34.684725000
O	48.414372000	49.218186000	34.598145000
O	46.855697000	48.651400000	39.094190000
O	46.911473000	50.285728000	36.932201000
O	45.539610000	47.988231000	36.866993000
O	41.622125000	48.849745000	38.830833000
O	42.857527000	47.760417000	36.695085000
O	44.448351000	46.609037000	34.929011000
O	43.735651000	42.062064000	32.355424000
O	46.197608000	42.913917000	32.927544000
O	48.580618000	42.006791000	32.041812000
O	54.223911000	44.410932000	38.663408000
O	56.341376000	42.936883000	39.391900000
O	55.466595000	47.973312000	35.217003000
O	51.856747000	48.706940000	33.534805000
O	52.916212000	47.687558000	35.813149000
O	51.591591000	50.051964000	35.801758000
O	54.789455000	46.682966000	37.397815000
O	54.389006000	49.371190000	37.255781000
O	56.342171000	45.886673000	39.404002000
O	55.461375000	40.839228000	35.218057000
O	51.882955000	40.069911000	33.523383000
O	52.909887000	41.117997000	35.815207000
O	54.774882000	42.137053000	37.394276000
O	46.190957000	42.166716000	40.456130000
O	44.318685000	40.340439000	39.646209000

O	46.190055000	39.623653000	41.462492000
O	42.437726000	41.970963000	40.587164000
O	42.330907000	49.424167000	41.277078000
O	43.283304000	48.463694000	43.643746000
O	48.814981000	48.637819000	42.992424000
O	42.855450000	45.941201000	42.952794000
O	41.593766000	44.422018000	41.123496000
O	43.708390000	46.540541000	45.426301000
O	45.369757000	46.840179000	43.347438000
O	48.624420000	46.869243000	44.995367000
O	47.799657000	46.209381000	42.478547000
O	45.769872000	44.490014000	42.052887000
O	45.849056000	46.802847000	40.731441000
O	44.212779000	48.603130000	39.565935000
O	45.961546000	49.374329000	41.458089000
O	42.354566000	46.868826000	40.471753000
O	42.981092000	40.402573000	43.732234000
O	48.812817000	40.187893000	43.051063000
O	42.727407000	42.988054000	43.090038000
O	43.739795000	42.250687000	45.424989000
O	45.147237000	41.833348000	43.121648000
O	48.744224000	41.893741000	45.135841000
O	47.877299000	42.695046000	42.708144000
O	53.525255000	40.323331000	41.890142000
O	50.455984000	42.233816000	43.078807000
O	52.876112000	42.888374000	42.262876000
O	51.683666000	44.399044000	44.113813000
O	53.866930000	42.059673000	39.897556000
O	55.447344000	42.161913000	42.046842000
O	52.420304000	46.841920000	44.756899000
O	53.518846000	48.495671000	41.890386000
O	50.373171000	46.477305000	42.998717000
O	52.839250000	45.936747000	42.249034000
O	53.863891000	46.758267000	39.897825000
O	55.420707000	46.643986000	42.061564000
O	52.535122000	41.978444000	44.782858000
Ga	46.725850000	43.508681000	39.035272000
C	50.340718000	42.629425000	38.629767000
H	50.810301000	41.656540000	38.442353000
C	50.522728000	43.686768000	37.726721000
H	51.141056000	43.536531000	36.832605000
C	49.568814000	42.820955000	39.780127000
C	48.964945000	44.076853000	40.027120000
H	49.444975000	42.004224000	40.500379000
H	45.854857000	44.768038000	39.294658000
H	48.521385000	44.274176000	41.013599000
C	49.925180000	44.936003000	37.959552000
C	49.153878000	45.139716000	39.110149000
H	47.224176000	42.783995000	37.758443000
H	50.098304000	45.760454000	37.259016000
H	48.713918000	46.125087000	39.315179000
